# Investigation of the Importance of Protein 3D Structure for Assessing Conservation of Lysine Acetylation Sites in Protein Homologs

**DOI:** 10.3389/fmicb.2021.805181

**Published:** 2022-01-31

**Authors:** Kristen M. Jew, Van Thi Bich Le, Kiana Amaral, Allysa Ta, Nina M. Nguyen May, Melissa Law, Nicole Adelstein, Misty L. Kuhn

**Affiliations:** Department of Chemistry and Biochemistry, San Francisco State University, San Francisco, CA, United States

**Keywords:** lysine acetylation, N-epsilon lysine acetylation, protein acetylation, non-enzymatic acetylation, *Escherichia coli* acetylation, conservation of protein acetylation sites, Gcn5-related *N*-acetyltransferase (GNAT)

## Abstract

Acetylation is a protein post-translational modification (PTM) that can affect a variety of cellular processes. In bacteria, two PTM *N*ε-acetylation mechanisms have been identified: non-enzymatic/chemical acetylation via acetyl phosphate or acetyl coenzyme A and enzymatic acetylation via protein acetyltransferases. Prior studies have shown that extensive acetylation of *N*ε-lysine residues of numerous proteins from a variety of bacteria occurs via non-enzymatic acetylation. In *Escherichia coli*, new *N*ε-lysine acetyltransferases (KATs) that enzymatically acetylate other proteins have been identified, thus expanding the repertoire of protein substrates that are potentially regulated by acetylation. Therefore, we designed a study to leverage the wealth of structural data in the Protein Data Bank (PDB) to determine: (1) the 3D location of lysine residues on substrate proteins that are acetylated by *E. coli* KATs, and (2) investigate whether these residues are conserved on 3D structures of their homologs. Five *E. coli* KAT substrate proteins that were previously identified as being acetylated by YiaC and had 3D structures in the PDB were selected for further analysis: adenylate kinase (Adk), isocitrate dehydrogenase (Icd), catalase HPII (KatE), methionyl-tRNA formyltransferase (Fmt), and a peroxide stress resistance protein (YaaA). We methodically compared over 350 protein structures of these *E. coli* enzymes and their homologs; to accurately determine lysine residue conservation requires a strategy that incorporates both flexible structural alignments and visual inspection. Moreover, our results revealed discrepancies in conclusions about lysine residue conservation in homologs when examining linear amino acid sequences compared to 3D structures.

## Introduction

Post-translational modifications (PTMs) are chemical changes that occur on proteins and include, but are not limited to, the covalent attachment of various functional groups on proteins. Often, these modifications can affect protein function by altering DNA/RNA binding (Matsuzaki et al., 2005; Xu et al., 2019), protein-protein interactions (Su et al., 2017), and protein localization (Ishfaq et al., 2012). Numerous types of covalent PTMs have been identified, including glycosylation (Schäffer and Messner, 2017), phosphorylation (Gnad et al., 2010), succinylation (Weinert et al., 2013b), methylation (Lanouette et al., 2014), ubiquitination (Udeshi et al., 2013), and acetylation, among others (Macek et al., 2019). While the PTM field is vast, we are specifically interested in the chemical/non-enzymatic and enzymatic mechanisms of *N*ε-acetylation on bacterial proteins. Non-enzymatic acetylation mechanisms utilize the acetyl donors acetyl Coenzyme A (AcCoA) (Tanner et al., 1999) or acetyl phosphate (AcP) (Weinert et al., 2013a; Kuhn et al., 2014), whereas enzymatic acetylation uses AcCoA. AcP is produced via the phosphotransacetylase-acetate kinase (Pta-AckA) pathway, which converts AcCoA to AcP via Pta, and AcP is converted to acetate via the acetate kinase AckA enzyme (Kakuda et al., 1994). AcCoA can be produced from pyruvate via pyruvate dehydrogenase or from acetate via acetyl-CoA synthetase (Acs) (Wolfe, 2005; Enjalbert et al., 2017). Studies have shown that non-enzymatic *N*ε-acetylation of proteins by AcP can be enhanced by deleting *ackA* or reduced by deleting *pta* (Weinert et al., 2013a; Kuhn et al., 2014). However, background levels of *N*ε-acetylation have been detected even when *pta* and *ackA* are deleted (Kuhn et al., 2014) and may be a result of non-enzymatic acetylation by AcCoA or protein lysine acetyltransferases (KATs) (Christensen et al., 2018).

As the methodologies for detecting protein PTMs have advanced, many studies have reported hundreds of proteins within a single organism are acetylated. In *E. coli*, this has largely been attributed to non-enzymatic acetylation by AcP, but evidence for enzymatic acetylation of many proteins via KATs also exists (Christensen et al., 2018). In bacteria, protein acetylation occurs on *N*α-amines of N-terminal amino acids and *N*ε-amines of lysine residues (Carabetta and Cristea, 2017; Carabetta et al., 2019; Christensen et al., 2019a,b; VanDrisse and Escalante-Semerena, 2019). The most well-studied bacterial *N*ε-acetyltransferase is YfiQ (or PatZ), which has been shown to acetylate a variety of bacterial proteins and is important for many cellular processes, including bacterial pathogenesis, virulence and stress resistance (Starai and Escalante-Semerena, 2004; Ma and Wood, 2011; Castano-Cerezo et al., 2014; Liu et al., 2018; Luu and Carabetta, 2021). Four additional KATs in *E. coli* have also been identified (YiaC, YjaB, PhnO, and RimI) (Christensen et al., 2018), but RimI and YiaC are also able to acetylate *N*α-amines of proteins (Vetting et al., 2008; Parks and Escalante-Semerena, 2020). Recent studies have highlighted the diversity of proteins that are acetylated in prokaryotes, but deep knowledge of how acetylation affects their physiology remains relatively limited. While hundreds of bacterial proteins have been identified as *N*ε-acetylated in *E. coli*, these reports have led to significant debate in the field regarding the relevance of this modification in prokaryotes. The main debate is centered on: (1) whether *N*ε-acetylation has a regulatory role and targets lysine residues on specific proteins, and (2) whether *N*ε-acetylation is inherently random and is just the product of spontaneous acetyl donation via a high energy molecule in an optimal chemical environment. If *N*ε-acetylation is indeed a physiologically important mode of bacterial protein regulation, these sites of *N*ε-acetylation theoretically should be conserved across homologs.

**FIGURE 1:**
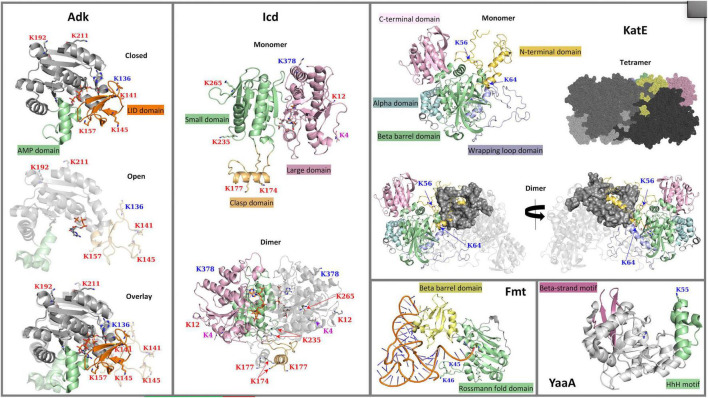
3D crystal structures of *Escherichia coli* K-12 lysine acetyltransferase (KAT) substrate proteins (Adk, Icd, KatE, Fmt, and YaaA). The crystal structures of Adk (PDB ID: 1ake and 6f7u) in complex with an AP5A inhibitor or phosphomethylphosphonic acid guanylate ester, respectively, Icd (PDB ID: 1ai2) in complex with NADP^+^, KatE (PDB ID: 1cf9) in complex with heme, Fmt (PDB ID: 2fmt) in complex with formyl-methionyl-tRNAfMet, and YaaA (PDB ID: 5caj) in complex with benzamidine are shown with ribbon representations. Ligands in each structure are shown with sticks. Lysine (K) acetylation sites are shown with sticks and their labels are colored based on type of acetylation that occurs: KAT sites are in blue, AcP sites are in red, and sites acetylated by both a KAT and AcP are in purple. **(Adk)** Two conformations of the Adk monomer are shown: the closed (PDB ID: 1ake) and open (PDB ID: 6f7u) conformations are at the top and middle of the panel, respectively; an overlay of the two conformations is shown at the bottom of the panel. The core domain is in gray, the AMP-binding domain is in green, and the ATP-binding LID domain is in orange. **(Icd)** A single monomer of the Icd structure is shown at the top of the panel with the small domain in light green, the large domain in pink and the clasp domain in orange. The dimeric form of the protein is shown at the bottom of the panel with one subunit colored by domains and the other subunit in gray. **(KatE)** The KatE protein exists as a tetramer, but a single monomer and dimer of the tetramer are shown to clarify domains and how the monomers of the tetramer intertwine. Each monomer is composed of the N-terminal arm domain in light orange, the C-terminal domain in pink, an alpha-helical domain in light blue, a beta-barrel domain in green, and a wrapping loop domain in violet. One monomer of the dimer is colored in gray, while the other monomer is colored by domains as described. Two views of the dimer are shown and are rotated by 180°. The tetramer is shown as a surface representation with each monomer colored in varying shades of gray, except for a single monomer that is colored by domains as in the single monomer view. **(Fmt)** A single monomer of the Fmt protein is shown with the Rossmann fold domain colored in green and the beta-barrel oligonucleotide binding fold domain in yellow; the formyl-methionyl-tRNAfMet is in orange and blue. **(YaaA)** The YaaA monomer is shown in gray ribbons with the beta-strand motif in pink and the helix-hairpin-helix (HhH) DNA-binding motif in green.

Most knowledge about the location of *N*ε-acetylated lysine residues on specific proteins has been limited to their positions in the linear amino acid sequence. Additionally, no obvious “signature sequence” has been determined for either non-enzymatic or enzymatic *N*ε-acetylation of prokaryotic proteins. Therefore, we previously suggested analysis of 3D protein structures is necessary for determining substrate specificity (Christensen et al., 2018). However, only a few studies have investigated sites of acetylation on 3D protein structures of KAT or AcP substrate proteins (Weinert et al., 2013a; Kuhn et al., 2014; Nakayasu et al., 2017; Christensen et al., 2018). Thus, in this study we focused on determining the conservation of these lysine residues across homologs using both their linear sequences and their 3D structures. Our main aim was to determine whether different analyses of the linear sequences and 3D structural comparisons would yield the same conclusion about conservation of lysine residues in homologs. Therefore, we selected five different *E. coli* proteins that were previously identified as being acetylated by KAT proteins (Christensen et al., 2018) and had structures in the Protein Data Bank (PDB) (Figure 1). We analyzed the location of the acetylated lysine residues on these structures and compared them to structures of homologs in the PDB with greater than or equal to 30% sequence identity to ensure coverage across domains of life. The KAT substrate proteins we selected for our study included: adenylate kinase (Adk), isocitrate dehydrogenase (Icd), methionyl-tRNA formyltransferase (Fmt), catalase HPII (KatE), and a peroxide stress resistance protein (YaaA). These proteins varied in size, oligomeric state, and type of reaction they catalyze. Most importantly, they exhibited a variety of characteristics regarding their acetylation patterns. For example, some proteins were acetylated by a single KAT or multiple KATs on the same residue, some were acetylated by both AcP and a KAT on the same residue, and some were acetylated by both AcP and a KAT on different residues (Kuhn et al., 2014; Christensen et al., 2018). We also selected these substrate proteins because they were identified as being acetylated by either YiaC or both YiaC and YfiQ, and less is known about the YiaC protein and its substrate specificity.

## Materials and Methods

### UniProt IDs for *Escherichia coli* Lysine Acetyltransferases and Substrate Proteins

**FIGURE 2:**
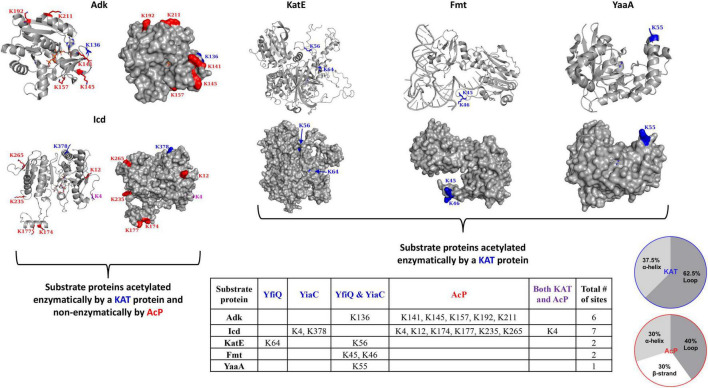
*Escherichia coli* K-12 lysine acetyltransferase (KAT) and acetyl phosphate (AcP) acetylation sites on substrate proteins Adk, Icd, KatE, Fmt, and YaaA. The table details sites of acetylation that occur via KATs (blue) YfiQ, YiaC, or both YfiQ and YiaC, AcP (red), or both KATs and AcP (purple). The crystal structures of the substrate proteins are in gray ribbons and the lysine residues that were previously identified as acetylated in *E. coli* K-12 are shown with sticks and colored by type of acetylation. AcP acetylation sites are in red, KAT acetylation sites are in blue, and both AcP and KAT acetylation sites are in purple. Monomers from the crystal structures are the same as those described in Figure 1 and Adk is shown in the closed conformation. The pie charts show the percentage of KAT and AcP sites on Adk, Icd, Fmt, KatE, and YaaA *E. coli* proteins that are located on alpha helices, beta strands, or loops.

Lysine acetyltransferases (KATs) from *E. coli* K-12 referred to in this study include YiaC (UniProt ID P37664) and YfiQ/PatZ (UniProt ID P76594). The *E. coli* K-12 KAT substrate proteins we analyzed in this work include Adk (UniProt ID P69441), Icd (UniProt ID P08200), KatE (UniProt ID P21179), Fmt (UniProt ID P23882), and YaaA (UniProt ID P0A8I3). A prior study identified K136 of Adk as a site of acetylation for both YfiQ and YiaC, K4 and K378 of Icd were sites of acetylation for YiaC, K56 of KatE was acetylated by both YfiQ and YiaC and K64 was acetylated by YfiQ, K45 and K46 of Fmt were also acetylated by both YfiQ and YiaC (Christensen et al., 2018). See Figure 2 for a full list of sites of acetylation via KATs and/or AcP on substrate proteins.

### Identification of Homologs of *Escherichia coli* Lysine Acetyltransferase Substrate Proteins in the Protein Data Bank

Amino acid sequences of substrate proteins (Adk, Icd, KatE, Fmt, and YaaA) were used to query the Protein Data Bank (PDB) via the MMseqs2 method (Steinegger and Söding, 2017) and identify structures of homologs with 30% or greater sequence identity that had been deposited into the PDB prior to January 5, 2021. To facilitate the acquisition and compilation of this data from the PDB, a Python class called protein3Dcompare and method (gethomologs) were written to generate a Microsoft Excel sheet containing PDB IDs, UniProt IDs, organism names, and protein sequences. The corresponding pdb files were then downloaded locally for further analysis.

### Comparison of 3D Structures of Substrate Proteins and Their Homologs

The following 3D crystal structures of the *E. coli* K-12 substrate proteins were selected as targets for comparing homologs identified in the PDB: Adk (PDB ID: 1ake), Icd (PDB ID: 1ai2), KatE (PDB ID: 1cf9), and Fmt (PDB ID: 2fmt). No homologs were identified in the PDB for YaaA (PDB ID: 5caj). Python-Pymol scripts to automate structural alignments of target and homolog proteins were written into protein3Dcompare and the method (ChainByChain) was used to identify individual chains within pdb files. Structural alignments were then performed between all possible chain combinations of the target and homolog proteins using the FATCAT (Flexible structure AlignmenT by Chaining AFPs (Aligned Fragment Pairs) with Twists) 1.0 algorithm (Ye and Godzik, 2003). FATCAT is a freely available code that performs flexible alignments using structures in a pdb file format. We downloaded the program from the website that hosted FATCAT v1.0 and used it for our analyses; however, this version is no longer available and has been replaced by v2.0 (Li et al., 2020). Since FATCAT saves the aligned target and homolog structures as a single pdb file, we used a method (ImportGlob) to decouple the structures and save them as a cif file for later viewing in Pymol. The sequence alignments and statistics generated from FATCAT were saved into an xml file using a similar method of the class (Supplementary Figure 1 in Data Sheet 1). Individual pairs of target and homolog protein structures were then manually compared in Pymol to identify whether acetylated lysine sites on target proteins were conserved in 3D space on homolog structures (Supplementary Figures 2A–D in Data Sheets 2–5). Identities of residues in each homolog structure were recorded (Supplementary Table 1).

### Multiple Sequence Alignments Based on Flexible Structure Alignment by Chaining Aligned Fragment Pairs With Twists 3D Structural Alignments

We wrote a script called MSA3D to generate multiple sequence alignments (MSAs) that were based on the structural comparisons from FATCAT for each substrate protein and their homologs. This script iterates through individual FATCAT target-homolog alignments (saved as xml files in a single directory) to produce a compiled MSA of all homologs and their target protein. The MSA is then saved as a fasta file and visualized with ESPript^1^ (Robert and Gouet, 2014) (Supplementary Figures 3A–D in Data Sheet 6). Note, only residues present in the FATCAT output are shown and are based on numbering from structures deposited into the PDB and are not necessarily full sequences found in UniProt.

### Identification of Homologs of *Escherichia coli* Lysine Acetyltransferase Substrate Proteins Not Constrained to the Protein Data Bank

Homologs of KAT substrate proteins that were not constrained to those with 3D structures in the PDB were identified using the BLAST tool within the UniProt database^2^ (UniProt Consortium, 2021). The sequence of each individual substrate protein was used as the query to search the UniProtKB reference proteome plus Swiss-Prot database with an e-threshold set to 10, no filtering, with gaps, matrix set to auto and hits set to 250. To increase organism coverage, the number of hits was expanded to 500 for all proteins except KatE. We manually selected sequences from these lists that covered the specific regions where lysine sites of acetylation were located (e.g., N-terminal extension of KatE) and were from unique organisms and taxonomic groups. When possible, we selected sequences that had been manually annotated and reviewed in Swiss-Prot. The EMBL-EBI multiple sequence alignment tool MUSCLE^3^ (Madeira et al., 2019) was used to align selected sequences and visualized with ESPript (Supplementary Figure 4 in Data Sheet 7).

## Results

### Structural Analysis of *Escherichia coli* Lysine Acetyltransferase and Acetyl Phosphate Substrate Proteins and Sites of Acetylation

#### Overview of *Escherichia. coli* Lysine Acetyltransferase Substrate Protein Functions, 3D Crystal Structures and Location of Acetylated Lysine Residues

##### Adenylate Kinase

Adenylate kinase (Adk) is a phosphotransferase that catalyzes the reversible conversion of ATP and AMP to two molecules of ADP in the presence of Mg^2+^. Adk proceeds through a ternary complex with both substrates bound during catalysis. Its activity is especially important for maintaining adenine nucleotide homeostasis, and is critical for cell growth via nucleotide, protein, and phospholipid synthesis (Glaser et al., 1975). Additionally, in some bacterial pathogens Adk is essential for survival (Thach et al., 2014) and can be a virulence factor (Markaryan et al., 2001). This enzyme is found in all domains of life, but variations to its structure and cellular localization exist across these domains. In bacteria, Adk is found in the cytoplasm, while its localization in eukaryotes varies significantly; different isoforms are located in the cytosol, mitochondria, and nucleus (Panayiotou et al., 2014). Structurally, Adk is a monomer in many organisms, but in the bacterium *Paracoccus denitrificans* it is a dimer (Perrier et al., 1998), and in the Archaea *Sulfolobus acidocaldarius* it is a trimer (Vonrhein et al., 1998). Interestingly, Wild *et al.* proposed the Adk protein from maize assembles into a supramolecular complex of proto-rods largely through its LID domain and this assembly is regulated via diurnal cycles of the plant and corresponding Adk activity (Wild et al., 1997). Additionally, different organisms and Adk isoforms within the same organism adopt structures with varying lengths of their LID domains (Mukhopadhyay et al., 2011).

In *E. coli*, Adk is a 214 amino acid monomeric protein with a molecular weight of ∼24 kDa, and its structure contains three defined domains, two of which are mobile. The CORE domain houses the active site where AMP (residues 57-59, 85-88) and ATP (residues 10-15, 132-133) bind. Two domains that close over the core include the AMP-binding domain (comprised of residues 30-59) and the ATP-binding LID (or INSERT) domain (comprised of residues 122-159), which is significantly more mobile (Figure 1) (Müller et al., 1996). A total of 20 crystal structures of this protein from *E. coli* have been deposited into the PDB and include a combination of different liganded and conformational states (Table 1). The flexibility of the LID domain of the *E. coli* Adk protein has been observed in several structures, including conformational extremes of the LID in the open (PDB ID: 6f7u) (Rogne et al., 2018) and closed (PDB ID: 1ake) (Müller and Schulz, 1992) forms (Figure 1). Adk was previously shown to be acetylated by YfiQ, YiaC, and/or AcP on six different lysine residues: 136, 141, 145, 157, 192, and 211 (Kuhn et al., 2014; Christensen et al., 2018). K136 was acetylated by both YfiQ and YiaC, whereas the remaining residues were acetylated by AcP (Figure 2). In the *E. coli* 1ake structure, these lysine residues are located on the flexible loop of the LID (K136, K141, K145, and K157) and the core (K192 and K211) domains (Figure 1). All of these residues are surface exposed on the monomer (Figure 2).

**TABLE 1:** Total number of adenylate kinase (Adk), isocitrate dehydrogenase (Icd), catalase HPII (KatE), methionyl-tRNA formyltransferase (Fmt), and a peroxide stress resistance protein (YaaA) *Escherichia coli* and homolog 3D structures analyzed in this study.

		Adk		Icd		KatE		Fmt		YaaA
*E. coli*		20		35		48		2		1
*Bacteria*	Gram negative	22	(7)	9	(5)	17	(6)	6	(6)	0
	Gram positive	29	(5)	1	(1)	17	(6)	16	(5)	0
	Gram variable	2	(1)	0		0		3	(1)	0
*Eukaryote*		32	(18)	15	(2)	57	(10)	9	(3)	0
*Archaea*		0		12	(4)	0		0		0
*Synthetic construct*		4	(3)	0		0		0		0
Total # of structures		109	(34)	72	(12)	139	(22)	36	(15)	1

*Structures are grouped by domain of life and numbers in parentheses indicate the number of unique organisms represented in each set. E. coli proteins are not included in the Gram-negative protein totals.*

##### Isocitrate Dehydrogenase

In *E. coli*, isocitrate dehydrogenase (Icd) is located in the cytosol and catalyzes the oxidative decarboxylation reaction that converts isocitrate and NADP^+^ to alpha-ketoglutarate, NADPH and carbon dioxide in the presence of Mg^2+^. In this reaction, NADP^+^ oxidizes isocitrate to an unstable intermediate, oxaloacetate, which is spontaneously decarboxylated to form alpha-ketoglutarate. This enzyme is found in the tricarboxylic acid cycle (TCA) and is at a critical branch point to the glyoxylate bypass. The flux between these two pathways is regulated by Icd, which shuttles carbon either through the TCA cycle to form acetyl-coenzyme A (AcCoA) for energy production or the glyoxylate bypass when generation of gluconeogenic precursors for cell growth is required (Hurley et al., 1989). Additionally, Icd is also a major producer of reducing power in the form of NADPH, which is used for a variety of cellular processes and defenses, including biosynthesis of lipids and nucleotides and protection from oxidative damage [reviewed in (Spaans et al., 2015)].

The *E. coli* Icd protein is 416 amino acids in length with a molecular weight of ∼46 kDa and is a dimer (Hurley et al., 1989); however, variability in size and oligomerization of Icd exists in different organisms (Stokke et al., 2007; Wang et al., 2018). Each monomer of the *E. coli* protein is comprised of three different domains: small, large, and clasp (Hurley et al., 1989). The approximate residues within these domains include, 1-124 and 321-416 for the large domain, 125-162 and 200-320 for the small domain, and 163-199 for the clasp domain (Figure 1). Thirty-five crystal structures of the Icd protein from *E. coli* in various liganded and mutant forms have been determined (Table 1). Two primary conformations of the *E. coli* Icd structure have been determined: open (e.g., PDB ID: 1sjs) and closed (e.g., PDB ID: 1ai2). Icd was previously found to be acetylated by YiaC on K4 and K378 and by AcP on K4, K12, K174, K177, K235, and K265 (Figure 2) (Kuhn et al., 2014; Christensen et al., 2018). The two residues acetylated by YiaC are located on the large domain; K4 is on the flexible N-terminus, whereas K378 is located on an alpha helix. Both residues are surface exposed, and neither residue formed any interaction with other residues in the protein or between monomers (Figure 2). The AcP acetylated residues are distributed on a combination of the large (K4 and K12), clasp (K174 and K177), and small (K235 and K265) domains. K4, K12, and K235 are located on loops, whereas K174 and K177 are on alpha helices; K265 is on a beta-strand (Figure 1). K174 and K235 are both located at the dimer interface, while all other AcP site lysine residues are surface exposed.

##### Catalase HPII

Catalase enzymes convert hydrogen peroxide (H_2_O_2_) to water and molecular oxygen and are important for maintaining intracellular concentrations of H_2_O_2_ and bacterial response to oxidative stress [reviewed in Zamocky et al. (2008), Yuan et al. (2021). Two types of heme-dependent catalase enzymes are present in *E. coli*, including the bifunctional catalase hydroxyperoxidase HPI (KatG) and the monofunctional catalase HPII (KatE). Catalases are present in all domains of life, but the KatE protein has only been found in bacteria, fungi, and archaea. *katG* expression is induced by H_2_O_2_ and is part of the OxyR regulon, while *katE* is not induced by H_2_O_2_ but rather by the sigma factor RpoS in stationary phase (Schellhorn and Hassan, 1988). These genes also appear to be induced by acetylation, albeit via an unknown mechanism (Ma and Wood, 2011). In addition to these two catalases, the alkyl hydroperoxide reductase AhpC protein can also help scavenge H_2_O_2_ and is regulated by OxyR (Seaver and Imlay, 2001). One study showed that in absence of polyamines, all three of these genes (*katE, katG*, and *ahpC*) are not expressed in *E. coli.* Furthermore, they suggested this was due to direct regulation of *oxyR* and *rpoS* by polyamines to induce expression of the corresponding catalases and reductase (Jung and Kim, 2003). Therefore, polyamines appear to be critical molecules for providing *E. coli* protection from H_2_O_2_-induced oxidative stress.

The *E. coli* KatE protein is very large and adopts a homotetrameric structure; a single monomer is ∼84 kDa and made of 753 amino acids (Figure 1). A total of 48 crystal structures of this enzyme from *E. coli* have been deposited into the PDB (Table 1). KatE adopts the catalase fold, which has an alpha-helical domain (residues 505-564) and a beta-barrel domain (residues 128-390) that are linked by a wrapping loop domain (residues 391-504) and a C-terminal domain (residues 600-753). The helical portion of the wrapping loop domain contains residues important for heme binding. Additionally, there is a very long N-terminal arm (residues 1-127) that is thread through the wrapping loop of another subunit of the tetramer to form substantial intersubunit interactions (Figure 1; Bravo et al., 1995; Díaz et al., 2012). Our prior studies showed KatE is acetylated by YiaC on K56 and by YfiQ on both K56 and K64 (Christensen et al., 2018); no residues were identified as acetylated by AcP. Both K56 and K64 are located on the N-terminal arm of the protein, with K64 on a small alpha helix and K56 on a mostly unstructured loop (Figure 2).

##### Methionyl-tRNA Formyltransferase

For protein synthesis to occur in bacteria, an initiator tRNA is aminoacylated by methionyl-tRNA synthetase and it is then formylated on the 3′ end by the enzyme methionyl-tRNA formyltransferase (Fmt), using substrates *N-*10 formyltetrahydrofolate (FTHF) and L-methioninyl-tRNA^Met^ [reviewed in Schmitt et al., 1998; Gualerzi and Pon, 2015]. Once formylated, the product *N-*formyl-methionyl-tRNA^fMet^ binds to the initiation factor 2 (IF2), which is then recognized by initiation factor (IF3) at the P-site of the 30S subunit of the ribosome. Formylation ensures the L-methionyl-tRNA^fMet^ interacts with IF2 instead of the elongation factor EF-Tu. It has also been suggested that *N-*formyl-methionyl-tRNA^fMet^ and IF2 may tune protein biosynthesis based on levels of specific cellular metabolites (Gualerzi and Pon, 2015), and another study has suggested N-terminal protein formylation could act as a signal (fMet/N-degron) for protein degradation in bacteria (Piatkov et al., 2015). Additional studies have shown that proper functioning of Fmt is required for cell growth (Guillon et al., 1992), but its essentiality has been called into question (Vanunu et al., 2017). While Fmt may not be essential, it has been reported that deleting *fmt* in *Bacillus subtilis* causes defects in biofilm formation, sporulation, and motility, as well as increased sensitivity to oxidative stress and antibiotics (Cai et al., 2017).

The Fmt protein from *E. coli* is an ∼34 kDa monomer of 315 amino acids. The structure consists of an N-terminal Rossmann fold domain (residues 1-189) and a C-terminal beta-barrel oligonucleotide binding fold domain (residues 209-314), which are linked by residues 190-208 (Figure 1). Within the N-terminal domain, there is a flexible insertion loop (Loop 1) comprised of residues 34-49, which lies above the active site and is critical for initiator tRNA recognition (Schmitt et al., 1996; Ramesh et al., 1999). The C-terminal domain is positively charged and points toward the active site. These two regions are important for binding tRNA. Two structures of this protein from *E. coli* have been deposited into the PDB: 1fmt and 2fmt (Table 1). The 1fmt structure shows Loop 1 as disordered, whereas the 2fmt is in complex with *N-*formyl-methionyl-tRNA^fMet^ and has this loop in an ordered conformation (Schmitt et al., 1996, 1998). Previously, we showed Fmt was acetylated in *E. coli* by both YiaC and YfiQ on K45 and K46 (Figure 2; Christensen et al., 2018). Both of these residues are located on Loop 1, which in absence of formyl-methionyl-tRNA^fMet^, undergoes trypsin proteolysis and completely inactivates the enzyme (Schmitt et al., 1996).

##### A Peroxide Stress Resistance Protein

A peroxide stress resistance protein (YaaA) belongs to the DUF328/UPF0246 domain of unknown function family and is the most understudied of the five *E. coli* KAT substrate proteins we investigated. Despite this, a handful of thorough studies with YaaA have provided steady advances in knowledge about this protein. For example, *yaaA* was shown to be induced by H_2_O_2_ and is dependent on the peroxide response regulator OxyR (Zheng et al., 2001). The YaaA protein is also overexpressed in *E. coli* upon exposure to mixtures of metals (Gómez-Sagasti et al., 2014) and it only becomes functional after the OxyR regulon is expressed during H_2_O_2_ stress (Liu et al., 2011). Recently, Paczosa et al. (2020) showed YaaA plays a role in *Klebsiella pneumoniae* virulence during mouse lung infection by protecting the bacterium from polymorphonuclear (PMN) effector functions, specifically H_2_O_2_ (Paczosa et al., 2020). Furthermore, Liu et al. showed YaaA protects cells from the Fenton reaction, and thereby DNA and protein damage via hydroxyl radicals in *E. coli*, by decreasing free iron in cells that are stressed by H_2_O_2_ (Liu et al., 2011). It has also been reported that YaaA does not scavenge iron (Paczosa et al., 2020) and it does not affect iron importer expression (Liu et al., 2011). Importantly, the YaaA protein does not decrease *E. coli* cellular H_2_O_2_ levels and it does not inhibit the reduction of ferric iron in *E. coli* (Liu et al., 2011).

While the 3D crystal structure of YaaA has been determined (PDB ID: 5caj) (Prahlad et al., 2020), it does not have any homologs with structures deposited into the PDB. YaaA is 258 amino acids in length, has a molecular weight of ∼30 kDa and was crystallized as a monomer. It is the first structural representative of this family of proteins and adopts a unique “cantaloupe” fold (Figure 1). This structural fold resembles a wedge-like entity that has a beta-strand motif (residues 187-202; 239-258) on one side of the wedge and a helix-hairpin-helix (HhH) DNA-binding motif (residues 35-66) on the opposite side of the wedge. The core is composed of atypical secondary structures (residues 6-22, 67-78, and 123-135), which are located in both the cleft (residues 209-213) and exterior of the protein (Prahlad et al., 2020). Our previous study showed this protein was acetylated by both YfiQ and YiaC on a single lysine residue, K55 (Christensen et al., 2018; Figure 2). This residue is located on the end of the α3 alpha helix, which is on the surface of the protein.

#### General Trends in the Location and Accessibility of Lysine Acetyltransferase Acetylation Sites on *Escherichia coli* Substrate Proteins

The five different *E. coli* KAT substrate proteins we selected for analysis exhibited different characteristics in terms of type of acetylation that occurred. For example, the KatE, Fmt, and YaaA proteins were identified only as acetylated enzymatically by a KAT protein, whereas Adk and Icd were acetylated both enzymatically via a KAT protein and non-enzymatically by AcP. Icd was the only protein in our dataset that had a single lysine that was acetylated at the same site enzymatically and non-enzymatically. In general, KAT lysine acetylation sites on these proteins were only one or two sites per protein, and if multiple sites existed, they were generally located close to each other in linear amino acid sequence. The exception to this observation was Icd, which had a KAT site near both its N- and C-termini. On the other hand, non-enzymatic AcP sites were distributed more broadly on the protein structure and were numerous. Next, we investigated the type of secondary structure that contained these acetylation sites on all five substrate proteins to determine if patterns for enzymatic or non-enzymatic acetylation would emerge. Our examination of the substrate protein monomers showed the KAT sites were located on either loops or alpha helices, with loops being more favorable (Figure 2). The AcP sites were located on loops, alpha helices, and beta strands, with relatively even distribution between all three types of secondary structure (Figure 2).

Next, we examined whether the acetylation sites were located on regions of the proteins that were surface accessible, at the interfaces of known oligomers, interacted with ligands, or were in active sites of the proteins. All acetylated lysine sites on the monomers of all substrate proteins were surface accessible, except the KatE protein (Figure 2). The *E. coli* substrate proteins that have been reported to adopt higher-ordered oligomers include Icd (dimer) and KatE (tetramer). Adk from *E. coli* is monomeric and the oligomeric state of YaaA is not currently known. Therefore, we investigated whether any of the acetylated lysine residues were located at oligomeric interfaces of the Icd and KatE proteins. We found that only AcP sites were located at the dimer interface of Icd, and KAT sites were on the exterior of the protein. Both KAT sites on the KatE protein did not interact with other monomers of the tetramer. The only KAT substrate protein with a lysine residue that was identified as being acetylated by either acetylation mechanism and was near a known active site was K235 of Icd. While this residue is not directly located within the active site, it is on the same loop that contains the catalytically important K230 residue (Bolduc et al., 1995; Lee et al., 1995). K235 also interacts with an adjacent loop (discussed below) that contains S113, which becomes phosphorylated and regulates Icd activity (Hurley et al., 1990). None of the lysine residues identified as acetylated in the five KAT substrate proteins interacted directly with any small molecules in any of the crystal structures we analyzed. However, one of the lysine residues identified as acetylated in Fmt did interact with tRNA.

#### Specific Interactions of Lysine Acetyltransferase and Acetyl Phosphate Acetylation Site Lysine Residues in Crystal Structures

##### Adenylate Kinase

**FIGURE 3:**
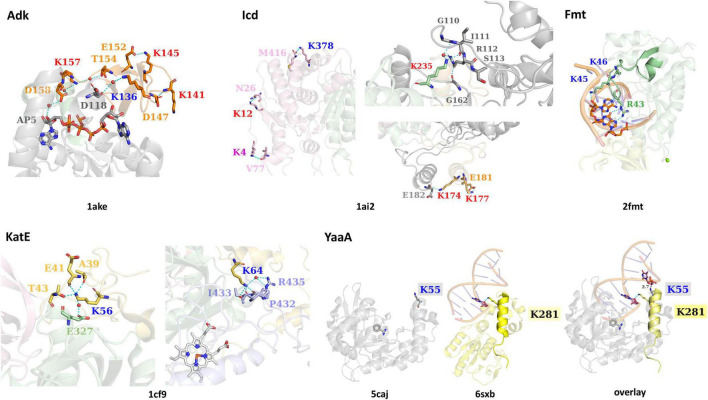
The interactions of lysine residues identified as acetylated in *E. coli* K-12 KAT protein substrates Adk, Icd, KatE, Fmt, and YaaA. Structures of each substrate protein are colored by domains as in Figure 1 and PDB IDs are shown beneath each substrate protein structure. Residues surrounding KAT or AcP lysine sites of acetylation and interact via H-bonding are shown as sticks and colored by domain. Cyan dashes are used for H-bonding interactions and black dashes (in the YaaA structure) show distance measurements. Ligands are shown in sticks and water molecules are red spheres. Specific lysine residue labels are colored red for AcP acetylation sites, dark blue for KAT acetylation sites, and purple for both AcP and KAT acetylation site (in Icd). An overlay of the YaaA protein helix-hairpin-helix (HhH) domain compared to another DNA-binding protein with an HhH domain in complex with DNA (PDB ID: 6sxb) is also shown.

Four of the six lysine residues identified as acetylated on Adk form H-bonds with other residues or ligands in the protein crystal structure (PDB ID: 1ake; Figure 3). Three of these residues (K141, K145, and K157) are acetylated by AcP, one residue (K136) is acetylated by a KAT, and all of these lysine residues are located on the LID domain. For K141, its *N*ε-amine does not directly interact with another residue, but its backbone nitrogen forms an H-bond with the side chain oxygen of D147, which is also located on the LID domain. The remaining three residues (K136, K145, and K157) all form H-bonds through their *N*ε-amines. K136 forms a water-mediated H-bond with the backbone oxygen of D118, which is located on the CORE domain. K145 forms an H-bond with the side chain oxygen of E152, which is located on the LID domain. K157 is the only lysine residue that interacts with the ligand through a network of water-mediated H-bonds, including the side chain oxygens of T154 and D158 on the LID domain, three water molecules, and the 3′-OH of one ribose of the AP5 inhibitor ligand.

##### Isocitrate Dehydrogenase

All Icd residues identified as acetylated by a KAT or AcP, except K265, form H-bonds with other residues of the protein (PDB ID: 1ai2; Figure 3). K4, K12, K177, and K378 form intrasubunit H-bonds, whereas K174 and K235 form intersubunit H-bonds in the PDB ID: 1ai2 structure. None of these lysine residues are located within the active site of the protein and do not interact with any of the ligands present in the crystal structure. The interactions that occur between the *N*ε-amine of these lysine residues and other residues of the protein include the following. In the large domain, K4 forms an H-bond with the backbone oxygen of V77, K12 forms an H-bond with the side chain oxygen of N26, and K378 forms an H-bond with the carboxy terminus of the protein at residue M416. The two lysine residues of the clasp domain form the following interactions via their *N*ε-amines. K177 forms an H-bond with the side chain oxygen of E181, which is located on the same alpha helix as K177. At the interface between clasp domains of the dimer, K174 forms an H-bond with the backbone oxygen of E182 of the adjacent monomer. Finally, the *N*ε-amine of K235 on the small domain forms a water-mediated H-bond with the side chain of R112 and the backbone oxygen of G110. A second H-bond between the delta amine of the side chain of R112 is formed with the backbone oxygen of G161. G110 and R112 are located on a loop that contains the residue S113, which interacts directly with isocitrate in the PDB ID: 1ai2 structure. Residues on this loop have been previously identified as being post-translationally modified, including S113 via phosphorylation (Borthwick et al., 1984), and K100 via succinylation (Zhang et al., 2011).

##### Catalase HPII

Both residues of the KatE protein are located on the N-terminal domain, are acetylated enzymatically by a KAT protein, and form intrasubunit H-bond interactions with other residues in the monomer (PDB ID: 1cf9; Figure 3). K56 forms H-bonds through its *N*ε-amine to the side chain of T43 and backbone oxygens of A39 and E41 of the N-terminal domain. Additionally, it has a water-mediated H-bond to the side chain oxygen of E327 on the beta-barrel domain. The *N*ε-amine of K64 forms H-bonds with residues in the wrapping loop domain, including the backbone oxygen atoms of P432, I433, and R435; it also forms a water-mediated H-bond to the side chain amine of R435. The K64 residue is not surface exposed, and instead is drawn inward toward the wrapping loop domain.

##### Methionyl-tRNA Formyltransferase

The two adjacent lysine residues of Fmt that are acetylated by a KAT protein (K45, K46) are located on a loop that interacts with tRNA (PDB ID: 2fmt; Figure 3). The *N*ε-amine of K45 forms an H-bond with the 2′-OH of adenosine 72 on the acceptor stem of tRNA. When both chains of the structure are compared, there are two slightly different conformations of cytosine 1 of the tRNA; one chain has the terminal phosphate 3.6 Å from K46 and the other is 11.2 Å away. R43, which is on the same loop as K45 and K46 and is critical for substrate binding and catalysis, forms bidentate H-bonding interactions between its side chain amines and the guanine base N7 amine and C6 carbonyl oxygen of guanosine 70 on the acceptor stem of tRNA (Figure 3).

##### A Peroxide Stress Resistance Protein

K55 is the only residue that was identified as acetylated for the YaaA protein and is located on the helix-hairpin-helix (HhH) DNA-binding motif. Prahlad *et al.* showed that the HhH motifs of the PDB ID: 6sxb DNA excision repair protein and YaaA protein exhibited structural similarity of this motif even though the remaining domains of the proteins are divergent (Jones et al., 2020; Prahlad et al., 2020). Since the K55 residue lies on the HhH motif and is thought to bind DNA and the 6sxb protein is in complex with DNA, we also aligned these two structures to assess whether K55 could theoretically interact with DNA at this same site (PDB ID: 5caj; Figure 3). In the 6sxb structure, an H-bond between the *N*ε-amine of K281 and the phosphate backbone (O3′) of DG6 of DNA is present. This lysine is located on the same helix of the HhH motif as K55 of YaaA, indicating a direct interaction with DNA may be possible.

### Identification and Analysis of *Escherichia coli* Lysine Acetyltransferase Substrate Protein Homolog Structures and Lysine Conservation

#### Identification of *Escherichia coli* KAT Substrate Protein Homolog Structures

**FIGURE 4:**
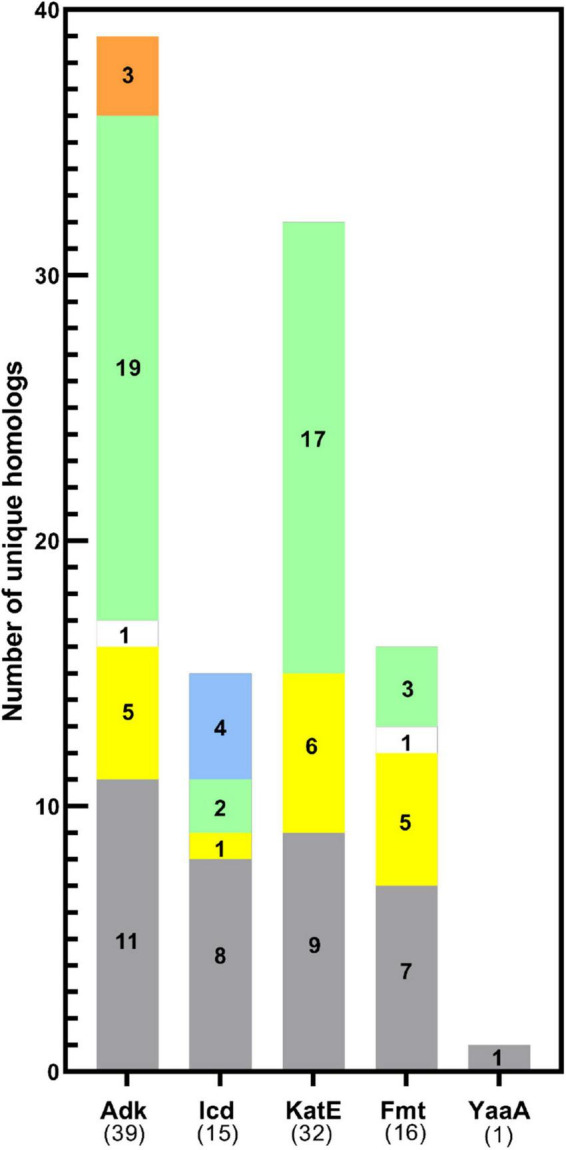
Distribution of KAT substrate protein homolog 3D structures across domains of life. The total number of homologs of each KAT substrate protein with unique UniProt IDs analyzed in this study are shown in parentheses on the x-axis. The target *E. coli* K-12 strain protein was also included in the total. Bars are colored by domain of life: Gram-negative (gray), Gram-positive (yellow), or Gram-variable (white) Bacteria, Eukaryotes (green), Archaea (blue), and synthetic constructs of ancestral proteins (orange).

While sites of protein acetylation have been determined for many organisms, most knowledge about protein acetylation in bacteria is based on studies with *E. coli.* Relatively few studies have investigated whether sites of acetylation on *E. coli* KAT substrate proteins are conserved on proteins from other organisms, and even less is known about their structural conservation. Therefore, we searched the PDB using the primary sequences of the five *E. coli* KAT substrate proteins and a 30% sequence identity threshold to identify the 3D structures of their homologs using the MMseq2 method (Steinegger and Söding, 2017) within the PDB. This approach yielded a total of 357 structures representing the five *E. coli* substrate proteins and their homologs (Table 1). A significant number (106; ∼30%) of these structures were of the *E. coli* proteins themselves that were crystallized in various liganded states, space groups, conformations, or variants (Table 1 and Supplementary Table 2 in Data Sheet 12). Only one structure of the *E. coli* YaaA protein has been determined, and no homologs for this protein were identified in the PDB. The remaining KAT substrate proteins had structures of homologs that included representatives from other Bacteria (subdivided into Gram-negative, Gram-positive, and Gram-variable), Eukaryotes, Archaea, and some ancestral synthetic constructs (Figure 4 and Supplementary Table 2). A total of 58 unique organisms were represented in this dataset. The KAT substrate proteins Adk, Icd, KatE, and Fmt had structures of Bacteria and Eukaryote homologs, but Icd was the only protein with structures of homologs from Archaea and Adk was the only one with synthetic ancestor construct structures. Adk and KatE homologs were nearly evenly split between Bacteria and Eukaryotes, whereas Icd and Fmt had more homologs from Bacteria (Figure 4). A summary of the organisms identified for each of the substrate protein homologs is shown in Supplementary Table 2.

#### Rigid vs. Flexible Structural Alignment Strategies for Determining Lysine Residue Conservation on 3D Structures of Homologs

**FIGURE 5:**
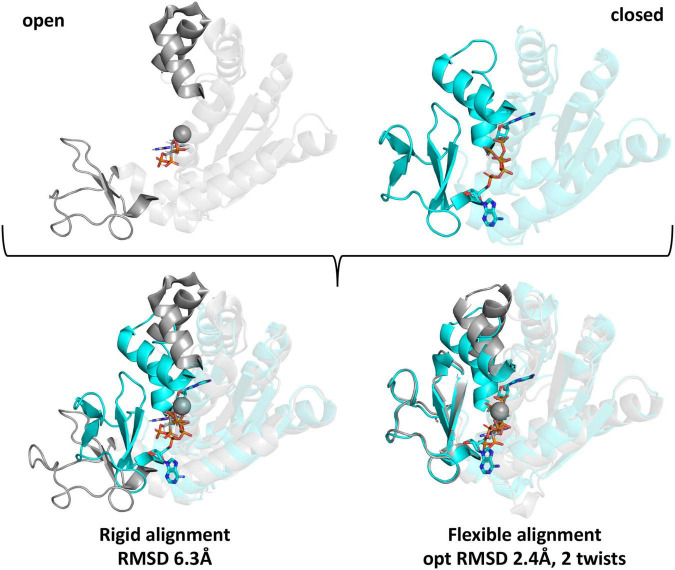
Comparison of rigid and flexible structural alignments of open and closed forms of the *E. coli* Adk protein using Pymol and FATCAT. The open form of the Adk protein crystal structure is shown in gray (PDB ID: 6f7u chain A) and the closed form is cyan (PDB ID: 1ake chain A). Ligands are shown as sticks and spheres are metal ions from the crystal structures. Regions of the protein that adopt significantly variable conformations are in bold colors and the remainder of the protein is transparent. Structural alignments between the open and closed forms using rigid or flexible alignment methods and their corresponding RMSD-values between alpha carbon atoms are indicated beneath the aligned structures.

To determine whether individual lysine residues previously identified as acetylated on the five *E. coli* KAT substrate proteins were conserved on homolog structures, we utilized two strategies. First, we used Pymol to perform a rigid structural alignment of the target and homolog proteins and manually recorded the identity of the residue of the homolog protein that corresponded in 3D space with the location of the acetylated lysine sites of the target protein (data not shown). Due to inherent flexibility of some of the substrate proteins and variability of their crystallized conformations, determining the corresponding residue in 3D on homologs was subjective when comparing mobile regions. Therefore, we switched our strategy and utilized a flexible alignment program called FATCAT. A comparison of these two methods is shown in Figure 5, where the RMSD between alpha carbon atoms for the rigid alignment of two Adk proteins in Pymol was 6.3 Å and the RMSD for the flexible alignment of these proteins with FATCAT was 2.4 Å. In addition, the FATCAT approach enabled structures with more drastic conformational changes to be compared more accurately.

#### Conservation of Acetylated Lysine Residues Across Homologs With 3D Structures in the Protein Data Bank for Each Lysine Acetyltransferase Substrate Protein

**FIGURE 6:**
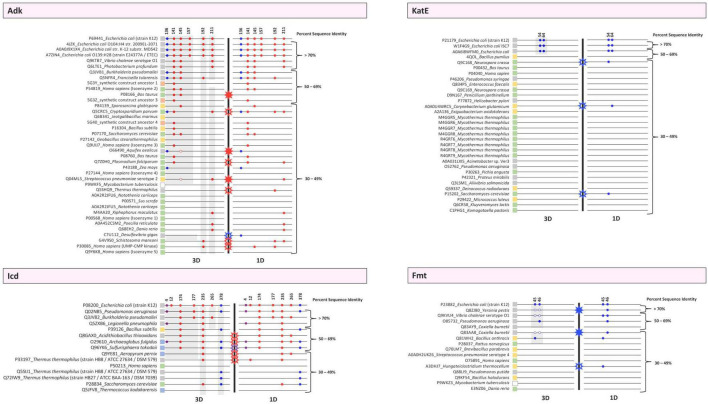
Conservation plots of selected lysine residues in both 3D structures and primary sequences of the target *E. coli* K-12 proteins (Adk, Icd, KatE, Fmt) and their homologs. Sequence and structural alignments between the target proteins and their homologs were analyzed to determine whether selected lysine residues were conserved in 1D and 3D. Alignments were performed using Cobalt and FATCAT and were then manually inspected. Each protein is indicated by its UniProt ID followed by the name of the organism. Strain or isozyme information is also included. PDB IDs were used when no UniProt ID existed for a given protein. Colored boxes adjacent to these labels correspond to whether they are Eukaryotes (green), Archaea (blue), Bacteria [Gram-negative (gray), Gram-positive (yellow), or Gram-variable (white)], or synthetic constructs (orange). Each protein is represented by a thin black line and organized based on percent sequence identity compared to the *E. coli* K-12 target protein. A thick black line separates the 3D and 1D conservation plots. Colored circles are used to indicate whether a lysine residue is conserved in both the target and homolog. The colors of the circles correspond to lysine acetyltransferase (KAT) acetylation sites (blue), AcP acetylation sites (red), or both KAT and AcP acetylation sites (purple). These sites were identified in previous studies with *E. coli* K-12 and are indicated by their residue number. The circles only indicate the presence of a lysine residue in 1D and 3D in homologs and not whether the residue has been identified as acetylated in the homolog *in vivo*. For Adk, open circles indicate a lysine residue was present during manual inspection of 3D structures but was not present in the FATCAT output. For Fmt, open circles mean that the residues were disordered in the structure but were present in the linear sequence for that region. Stars indicate a discrepancy between 3D and 1D lysine conservation within the same protein. Open stars represent a discrepancy in the 1D analysis, whereas filled stars represent a discrepancy in the 3D analysis. For example, if a residue is present in the 1D analysis but not the 3D analysis, an open star is shown. On the other hand, if a residue is present in the 3D analysis but not 1D analysis, a closed star is shown. The color of the stars corresponds to the type of acetylation site, i.e., KAT (blue), AcP (red), or both KAT and AcP (purple) that has the discrepancy. Gray highlighting on the 3D panels identifies proteins that contain similar structural domains as in the *E. coli* target proteins.

To explore the conservation of *E. coli* lysine acetylation site residues in homologs, we used FATCAT to generate pairwise sequence/structure comparisons between chain A of the target *E. coli* proteins and all chains of the homolog protein structures for all KAT substrate proteins. The rationale for comparing all chains of the homolog structures was because we thought some chains would reveal different conformational changes, have disordered regions, or adopt different liganded states. After compiling the xml files from the FATCAT output, we used the following selection criteria to choose a single chain from each unique structure for further analysis. First, we selected the chain alignment that exhibited the lowest opt-rmsd-value. If multiple chains of the single structure had the same value, we then chose the chain with the longest sequence length. Finally, if the sequence lengths were identical, we chose the alignment with the highest score. These results were compiled and figures of a single chain of each protein structure and the FATCAT alignment between target and homolog were generated (Supplementary Figure 2 and Supplementary Table 3 in Data Sheet 10). We also manually inspected the FATCAT structural alignments to ensure the sequence alignment output accurately reflected what was observed in 3D. There were two Adk homologs (UniProt IDs O66490 and Q04ML5) that did not show the correct residue in the 3D FATCAT alignment output but were indeed present after manual inspection of the structural overlays (Figure 6 and Supplementary Figure 2A).

Since many protein homologs had several structures in the PDB, we selected a single structural representative for each unique UniProt ID to assess residue conservation. The highest resolution WT structures of homologs were chosen as representatives and are listed in Supplementary Table 4. Furthermore, we generated a multiple sequence alignment that was based on structural alignments via FATCAT for these selected homolog sequences/structures (Supplementary Figure 3); FATCAT output parameters were recorded in Supplementary Table 5 in Data Sheet 11. Additionally, we performed multiple sequence alignments of primary sequences of the homologs and targets without taking into account their 3D structures (Supplementary Figures 5A–D in Data Sheet 8). We used all of these results to determine which method, either primary sequence (1D) comparisons or structural (3D) comparisons, provided equivalent accuracy in determining acetylation site lysine residue conservation in homologs. The analysis of this data is presented below.

##### Conservation of *Escherichia coli* Acetylated Lysine Residues Across Adenylate Kinase Homologs in 1D and 3D

Based on our analysis of 3D structures of Adk homologs with unique UniProt IDs, we found that only homologs of different strains of *E. coli* had all six lysine residues conserved (Figure 6). Five homologs retained the KAT K136 site, including proteins from *Burkholderia pseudomallei, Francisella tularensis, Cryptosporidium parvum, Aquifex aeolicus*, and *Zea mays*, which include three Gram-negative bacteria and two Eukaryotes. Interestingly, this site was not conserved in all Gram-negative bacteria, and no Gram-positive bacteria had a lysine residue at this position in 3D space. The most conserved AcP site was K141, followed by K145, which are both located on the LID domain of Adk. K157 was only conserved in *E. coli*, and the conservation of K192 and K211 was scattered and limited across the dataset. When we compared the conservation of these lysine residues based on primary sequence alignments (1D) with their conservation by 3D structural comparisons, we observed similar trends but there were some discrepancies in conservation. The sites that were most variable in their conservation between 1D and 3D analysis were AcP sites (Figure 6). Only one discrepancy in conservation was observed for the KAT site. Of the nine discrepancies observed, three had a site conserved by 3D analysis but not 1D analysis, whereas the remaining six were conserved by 1D analysis but not 3D analysis (Figure 6). Therefore, analyzing 3D structures of proteins for lysine conservation is more accurate than primary sequence comparisons of homologs alone.

##### Conservation of *Escherichia coli* Acetylated Lysine Residues Across Isocitrate Dehydrogenase Homologs in 1D and 3D

Our analysis of the Icd homolog proteins showed that nearly half of the homologs retained the KAT K378 lysine residue, including *Pseudomonas aeruginosa, Bacillus subtilis, Archaeoglobus fulgidus, Sulfurisphaera tokodaii, Thermus thermophilus, and Saccharomyces cerevisiae* (Figure 6). Similar to Adk, not all Gram-negative homologs retained the KAT site, but the distribution in conservation was broader and included representatives from Gram-negative and Gram-positive bacteria, Archaea, and Eukaryotes. The KAT K4 site, which is also acetylated by AcP, was less conserved than the K378 site. Only three organisms had this residue conserved, including *Pseudomonas aeruginosa, Legionella pneumophila*, and *Archaeoglobus fulgidus*. The most conserved lysine residue was the K235 AcP site, which is located at the interface between dimers, followed by the K174 AcP site, which is on the clasp domain and directly interacts with an opposite monomer of the dimer. The K12 AcP site was not found in any homologs, and K177 and K265 AcP sites were extremely limited in conservation. Similar to Adk, the sites that were most variable in conservation when comparing 3D structural analyses with 1D primary sequence analyses were AcP sites. Only one discrepancy was observed for a KAT K4 site. All five discrepancies in conservation were from 1D analyses, again indicating that analyzing conservation by 3D structure is more reliable (Figure 6).

##### Conservation of *Escherichia coli* Acetylated Lysine Residues Across Catalase HPII Homologs in 1D and 3D

The conservation of KAT sites (K54 and K64) in KatE homologs based on 3D structures was limited to proteins from different strains of *E. coli* (Figure 6). None of the other homologs had these two lysine residues conserved. It is likely that this lack of conservation is because the N-terminal arm is longer in the *E. coli* protein than other homologs that have been structurally determined. Alternatively, it is possible this portion of the N-terminal arm is so incredibly flexible that an accurate assessment of conservation in 3D space is not possible (see below for further discussion). Similar to Adk and Icd, analyzing lysine conservation by primary sequence alone showed discrepancies between 3D and 1D analyses. All three proteins that had the lysine residue conserved based on primary sequence analysis were not conserved in 3D space.

##### Conservation of *Escherichia coli* Acetylated Lysine Residues Across Methionyl-tRNA Formyltransferase Homologs in 1D and 3D

We faced new challenges when trying to assess lysine conservation based on 3D structures of the Fmt protein homologs. Three of the homologs have structures where the two KAT K45/K46 residues are located on a disordered region of the protein (Q8ZJ80, Q9KVU4, and Q83AA8). This is not unusual, and in fact the target *E. coli* Fmt protein that was crystallized in absence of tRNA also has this region disordered. Therefore, we examined all of the structures using FATCAT and then looked at whether the residues of the disordered loop primary sequence had lysine residues in a similar location as the target protein. We found five homolog proteins had at least one of these KAT site lysine residues conserved and three of the homologs had both lysine residues conserved (Figure 6). The overwhelming majority of these proteins that contained a conserved lysine were from Gram-negative bacteria *Yersinia pestis, Vibrio cholerae, Pseudomonas aeruginosa*, and *Coxiella burnetti* (Figure 6). The only exception was the protein from the Gram-positive bacterium *Bacillus anthracis*. However, not all homologs from bacteria and no Eukaryotic homolog had these residues conserved.

#### Percent Sequence Identity Was Not an Indicator of Lysine Conservation in Homologs

We were surprised at the variability of lysine conservation across homologs based on percent sequence identity. Initially, we expected protein homologs with high percent sequence identity would retain KAT site lysine residues and that there would be a threshold in percent sequence identity that could be used to exclude homologs. However, even in this limited dataset we observed KAT site lysine conservation in homologs at varying percent sequence identities. For example, not all protein homologs with high sequence identity retained comparable KAT site lysine residues, but some proteins with low sequence identity did. A similar trend was observed for AcP sites. Additionally, the Icd protein, which has two KAT sites, had homologs with significant variability in lysine residue conservation for each site with one site being more conserved across lower percent sequence identity homologs (K378). On the other hand, the KatE and Fmt proteins had KAT sites mainly conserved at high percent sequence identity when comparing 3D structures but exhibited much more variability when examining conservation by primary sequence alone.

#### Effect of Structural Domain Deviations Between Homologs and Targets on Interpreting Lysine Conservation in Homologs Across Taxonomic Groups

**FIGURE 7:**
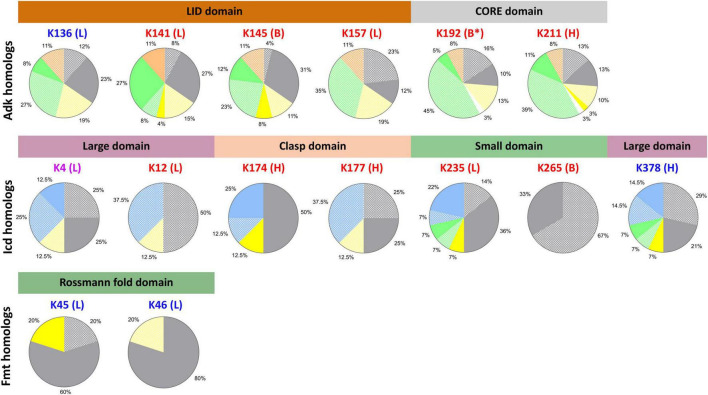
Residue conservation in KAT substrate protein homologs by domain of life based on presence of 3D structural domains. Each lysine residue previously identified as acetylated in *E. coli* K-12 by AcP and KATs on Adk, Icd, and Fmt proteins are shown above individual pie charts. The color of the lysine residue corresponds to the type of acetylation: AcP (red), KAT (blue), AcP and KAT (purple). The letters in parentheses indicate the type of secondary structure on which the residue was found in the *E. coli* substrate proteins: loop (L), alpha helix (H), beta strand (B). Stars indicate the lysine was located on the end of the type of secondary structure. The structural domains for each protein are shown with colored bars and are colored according to domains in Figure 1. Pie charts show the conservation of lysine residue by domain of life when structural domains of the protein homologs are taken into account. Protein homologs from Gram-negative, Gram-positive, and Gram-variable bacteria are colored gray, yellow, and white, respectively. Eukaryotes are green, Archaea are blue, and synthetic constructs are orange. Solid colors indicate the lysine residue was conserved and dot patterns indicate the lysine residue was not conserved. Only proteins that had the structural domain present were used to calculate lysine residue conservation.

During our analysis of structures of KAT substrate protein homologs, we observed that while many of the core domains of these proteins were conserved between target and homologs, many domains outside of the core were variable. In some cases, complete domains that were present in target proteins were missing from homolog structures and would artificially skew our conclusions about lysine conservation. Therefore, we reanalyzed our lysine conservation results based on whether entire domains compared to the target were present in 3D structures (Figure 6 gray shading, Figure 7 and Supplementary Figure 6 in Data Sheet 9). Our secondary analysis showed the following results for lysine conservation in Adk, Icd, and Fmt homologs. The only structures of KatE homologs with conserved N-terminal extension were from *E. coli*, therefore we excluded all other KatE homologs from this analysis.

##### Adenylate Kinase

A total of 31% of all homolog structures that contained the full LID domain had the K136 KAT site lysine conserved (Figure 7). Nearly 66% of the Gram-negative organisms retained this lysine residue as opposed to only 23% of Eukaryotes. No Gram-positive bacteria had this lysine conserved, which is consistent with our first observations. The three other residues of the LID domain that were identified as acetylated by AcP (K141, K145, and K157) exhibited variable conservation. K141 was the most conserved (69%) across all homologs, with equivalent conservation in Gram-negative bacteria and Eukaryotes (77%). All ancestral synthetic constructs retained this lysine as opposed to only 21% of Gram-positive bacteria. Similarly, 51% of homologs had the K145 AcP site lysine conserved; these were 89% of Gram-negative bacteria, 42% of Gram-positive bacteria, and 34% of Eukaryotes. In contrast, only 12% of all homologs had the K157 AcP site conserved, which were all *E. coli* proteins. The AcP site lysine residues in the core domain (K192, K211) were only conserved in 15% and 27% of homologs, respectively. A total of 38% of Gram-negative and 10% of Eukaryote homologs had K192 conserved and 50% of Gram-negative, 23% of Gram-positive, and 22% of Eukaryotes had K211 conserved. Thus, the majority of conserved lysine residues are in Gram-negative bacterial homologs.

##### Isocitrate Dehydrogenase

Nearly 50% of all Icd homolog structures had the K378 KAT site lysine residue and 37.5% had the K4 KAT site lysine residue conserved (Figure 7). These residues are both located on the large domain of the Icd protein, however, their conservation across various taxonomic groups was different. For example, after analyzing structures for conserved domains, we found the loop where K4 is located was not conserved in any Eukaryotic homolog, but the alpha helix where K378 is located is conserved in structures of all Eukaryotic homologs. The only Gram-positive bacterial homolog in our dataset has both of these secondary structures of the large domain conserved, but only K378 was conserved. We also found 42% of Gram-negative bacteria and 50% of all Archaea and Eukaryotic homologs had the K378 KAT site lysine residue conserved. On the other hand, K4 was conserved in one-third of Archaea and half of Gram-negative bacteria. Only two homologs from *Pseudomonas aeruginosa* and *Archaeoglobus fuigidus* had both KAT site lysine residues conserved. The AcP site lysine residue (K12, K174, K177, K235, and K265) conservation across homologs was also variable. K12 is located on the same loop as K4 on the large domain, but K12 was not conserved in any homolog. K174 and K177 are located on the clasp domain and K174 was the most conserved of the two residues across homologs. In fact, 87.5% of all homologs retained the comparable K174 residue, but only 25% of homologs had K177 conserved. Not all Eukaryotic homologs retained a similar clasp domain to the *E. coli* protein and therefore did not have these residues conserved. All Gram-negative and Gram-positive bacterial homologs and two-thirds of all Archaeal homologs had K174 conserved. Alternatively, K177 was only conserved in 50% of Gram-negative bacterial homologs. A similar trend was observed for K235 and K65, which are located on the small domain of Icd. Seventy-two percent of all homologs had the K235 site conserved, compared to only 33% that had the K265 site conserved. Additionally, the portion of the small domain where the K265 residue is located was only observed in structures of Icd homologs from Gram-negative bacteria. The K235 residue is located at the interface between monomers in the *E. coli* Icd structure and was conserved in 72% of Gram-negative bacteria, the only Gram-positive bacterial homolog, half of the Eukaryotic homologs, and 76% of Archaeal homologs.

##### Catalase HPII

Structures of the KatE homologs were grouped according to clades that were previously defined (Klotz et al., 1997; Yuan et al., 2021; Supplementary Figure 6). Since the *E. coli* KatE protein is the only representative of Clade II catalases that has been crystallized and exhibits an extended N-terminal domain and an extra C-terminal domain that is absent in Clade I and III members, we were unable to assess conservation of the K56 and K64 residues on structures of homologs.

##### Methionyl-tRNA Formyltransferase

We filtered Fmt homolog structures based on the presence or absence of the extended loop on the Rossmann fold domain where the two KAT site lysine residues (K45, K46) are located and found only Gram-negative and Gram-positive bacteria had this loop conserved (Figures 6, 7). Strikingly, most Fmt Gram-positive bacterial homologs did not have this loop conserved or exhibited other structural differences in domain structure compared to Gram-negative homologs. Furthermore, 80% of the homologs that had the loop conserved also had the K45 or K46 residue conserved, but the conservation was different across organisms. For example, the only Gram-positive bacterial homolog and 75% of Gram-negative bacterial homologs had the K45 residue conserved, while all Gram-negative bacterial homologs had K46 conserved. K46 was not conserved in the Gram-positive homolog.

#### Expansion of Lysine Acetyltransferase Protein Substrate Lysine Residue Conservation Analysis on Structurally Uncharacterized Homologs With High Percent Sequence Identity

**FIGURE 8:**
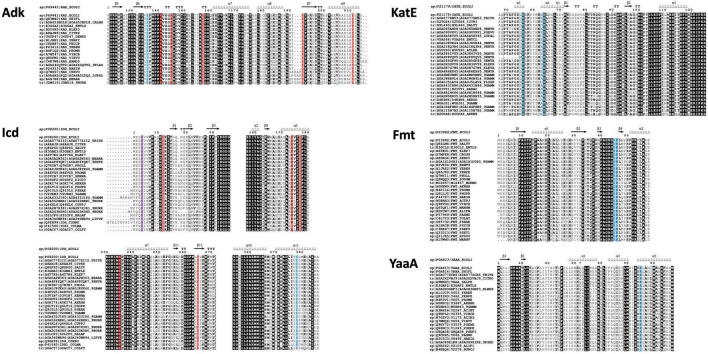
Conservation of *E. coli* substrate protein (Adk, Icd, KatE, Fmt, and YaaA) acetylated lysine sites across homologs identified by sequence from UniProt. Sequences were cropped to only show regions containing acetylated sites in the *E. coli* proteins (blue highlighting indicates KAT acetylation sites, red indicates AcP acetylation sites, and purple indicates both AcP and KAT acetylation sites). UniProt IDs for each protein are listed adjacent to each sequence and the *E. coli* substrate protein sequence and secondary structure of the target structure used in our comparative analysis is shown on top (PDB ID: 1ake for Adk, PDB ID: 1ai2 for Icd, PDB ID: 1cf9 for KatE, PDB ID: 2fmt for Fmt, and PDB ID: 5caj). Sequences are ordered from highest to lowest percent sequence identity compared to the target protein: approximately 99–66% (Adk), 99–73% (Icd), 99–62% (KatE), 91–56% (Fmt), and 100–58% (YaaA). Complete sequence alignments and details about selected proteins are shown in Supplementary Figure 4. See section “MATERIALS AND METHODS” for further details.

Since the structural coverage of KatE homologs with an extended N-terminal arm was limited to the *E. coli* target protein, we could not draw meaningful conclusions about KAT site lysine residue conservation in homologs. Therefore, we performed a search to identify KatE homologs by primary sequence in UniProt (see section “MATERIALS AND METHODS” for more details). Based on these results we manually selected 28 sequences that exhibited coverage of the comparable N-terminal region of KatE that contained the two lysine residues identified as acetylated in *E. coli* and were from unique organisms and taxonomic groups. Each of these proteins had both lysine residues conserved and their percent sequence identity compared to the *E. coli* target protein ranged between approximately 56–91% (Figure 8, Supplementary Figure 4). We were also limited in our ability to draw conclusions about KAT site lysine residue conservation in YaaA homologs because the target protein is the only structural representative. Thus, we performed a similar search to identify YaaA homologs and manually selected 23 sequences from unique organisms and taxonomic groups for further analysis. All but one of these homologs had the lysine residue conserved, which had the lowest percent sequence identity of ∼58% (Figure 8, Supplementary Figure 4).

To determine whether the trend of inconsistent KAT and/or AcP site lysine conservation across homologs with high percent sequence identity was also seen when the dataset was broadened to include more unstructurally characterized representatives, we gathered and analyzed homolog sequences for the Adk, Icd, and Fmt proteins (Figure 8, Supplementary Figure 4). These sequences had between 66.82–99.53%, 73.8–99.63%, and 56.59–91.75% sequence identity for Adk, Icd, and Fmt homologs compared to target, respectively. The KAT site K136 in the Adk homologs was not conserved in 5/19 sequences, which had between 68–75% sequence identity compared to the target protein. AcP site lysine residues varied in their conservation across this dataset. For example, K141 was conserved in all protein sequences, K145 was mostly conserved (17/19) but two proteins had a positively charged residue at the corresponding position, K157 was only conserved in 11/19 proteins and was replaced by either an aspartate or alanine residue, K192 also varied in its conservation with 11/19 proteins having the residue conserved, and K211 was the least conserved AcP site, with only 10/19 proteins having lysine at this position by primary sequence analysis.

For Icd, the two KAT sites (K4 and K378) both exhibited variability in lysine conservation. K378 was conserved in 19/26 of the sequences and some proteins ranging in percent identity from 73–81% did not have the residue conserved, but others in this identity range did. K4 was conserved in 20/26 proteins and deviations to the residue at this position were to the positively charged histidine residue. Like the Adk protein, the AcP site lysine residues on Icd were also variably conserved. For example, the K12 site was only conserved in 7/26 proteins, but the adjacent residue is also a lysine residue and is conserved in 25/26 proteins. The K174 residue was conserved in 25/26 sequences, K177 was conserved in 19/26 sequences, K235 was completely conserved, and K265 was only conserved in 18/26 sequences. The Fmt homologs that contained the same loop and structural domains of the target protein ranged in sequence identity from 56–91% and showed at least one of the two KAT site lysine residues were conserved in 26/28 proteins. Only 16/28 had both lysine residues conserved and were distributed across protein homologs with varying sequence identities.

## Discussion

While advances have been made to understand non-enzymatic acetylation of proteins in a variety of bacteria (Nakayasu et al., 2017), the depth of knowledge about KAT substrate specificity and how their cognate substrates are recognized is still limited. Part of the complexity of understanding KAT substrate specificity is that unlike AcP, which has been shown to bind in active sites and near positively charged residues on the surface of some proteins (Kuhn et al., 2014), KAT enzymes typically acetylate regions of substrate proteins that are flexible and surface exposed (present study and Christensen et al., 2018). Additionally, some of these KAT proteins acetylate proteins at the *N*α-amine of the N-terminus of proteins in addition to the *N*ε-amine of internal lysine residues (Vetting et al., 2008; Parks and Escalante-Semerena, 2020), making identification of a “signature sequence” for acetylation via analysis of primary sequence or 3D structure challenging. This substrate promiscuity of the KAT proteins is not unusual, especially because they belong to the Gcn5-related *N-*acetyltransferase (GNAT) superfamily. Numerous GNAT proteins exhibit substrate promiscuity, which has ultimately enabled them to evolve to acetylate a variety of substrates of all different shapes and sizes ranging from proteins to small molecules.

Outstanding questions remain regarding whether acetylation of specific lysine residues on proteins identified as acetylated by KATs alter their enzymatic activity or protein function, structure, stability, protein-protein interactions, or oligomerization. Additionally, it is not completely understood whether the role of protein acetylation of these substrate proteins is to regulate them in a similar fashion as other post-translational modifications like phosphorylation. We do not know if the comparable lysine residues in KAT substrate protein homologs will also be acetylated in other organisms even if they are conserved. However, Nakayasu et al. has shown that numerous proteins in a variety of bacteria are indeed acetylated by AcP (Nakayasu et al., 2017), so it is likely that KAT acetylation is also conserved in other bacterial species. Thus, progress has been made, but these questions will ultimately require additional collective efforts of cross-disciplinary investigators to study these enzymes via *in silico, in vitro*, and *in vivo* studies.

### Possible Implications of Adenylate Kinase, Isocitrate Dehydrogenase, Catalase HPII, Methionyl-tRNA Formyltransferase, and a Peroxide Stress Resistance Protein Acetylation

The five KAT substrate proteins that we investigated in this study have important functions in bacterial physiology, with many having defined roles in bacterial oxidative stress responses, pathogenesis, and virulence. While several of these proteins have been very well-studied and characterized for decades, roles of other protein substrates are comparatively in their infancy. Yet, acetylation of all of these proteins adds another possible layer of complexity to the tunability of their functions under specific cellular conditions and requires further dedicated study. Below is a description of the possible implications of acetylation on specific KAT site lysine residues of these substrate proteins based on the scientific literature. However, further experiments are required to determine their validity.

#### Adenylate Kinase

The K136 KAT site and several AcP sites are located on the LID domain of the Adk protein. Previous studies have shown that the conformational dynamics of the LID domain of this protein are linked to the identity of the nucleotide triphosphate that binds (ATP causes closed conformation, GTP causes open conformation) (Rogne et al., 2018) and whether salt bridges are formed or broken between different lysine residues of the LID (Song and Zhu, 2013). In the open conformation of this protein, K136 forms a salt bridge with D118. Thus, perhaps it is possible that acetylation of the LID of the Adk protein could affect its conformation and therefore nucleotide binding. In fact, it is known that the activity of this enzyme can be regulated via different protein-protein interactions, including binding to the ribosome through its core domain (possibly via its open conformation) which reduces activity (DeMott et al., 2017).

#### Isocitrate Dehydrogenase

The Icd *E. coli* protein was the first enzyme identified as being regulated by phosphorylation, and since then has been shown to be regulated through other post-translational modifications, including succinylation and acetylation. Additionally, Venkat et al. (2018) has performed a study of the effects of acetylation of several lysine residues on Icd activity using genetic code expansion and mutagenesis. They characterized two of the residues we analyzed in our study, including K177 and K235, which were both acetylated by AcP. Ultimately, they found acetylated K177 only slightly increased Icd activity, whereas acetylated K235 decreased activity by approximately 40%. Therefore, acetylation of Icd can directly affect enzyme activity depending upon the location of specific lysine acetylation sites. The K4 and K378 KAT site residues were not included in their study.

#### Catalase HPII

The two KAT site lysine residues on the KatE protein (K56 and K64) are located on its extended N-terminal domain. To our knowledge, there is no information in the literature about the importance of these two residues; however, Sevinc et al. (1998) has shown that deleting portions of the N-terminus (residues 3-74) caused the enzyme to lose nearly all activity. Additionally, there is conflicting information in the literature regarding acetylation of *E. coli* KatE and KatG proteins. Ma and Wood showed these two enzymes were not acetylated (Ma and Wood, 2011), but we previously showed KatE was acetylated by YiaC and YfiQ and KatG was acetylated by AcP (Kuhn et al., 2014; Christensen et al., 2018). It is currently unclear why there is a discrepancy.

#### Methionyl-tRNA Formyltransferase

Interestingly, if a functional complex between Fmt and L-methionyl-tRNA^fMet^ is not present, the loop that contains the two KAT site lysine residues (K45, K46) is susceptible to trypsinolysis (Schmitt et al., 1996; Ramesh et al., 1999). Previous studies have shown this loop is critical for activity, and removing residues 38-47 and replacing them with Leu-Gly-Gly caused the activity to decrease four orders of magnitude (Schmitt et al., 1998). Since trypsin cannot cleave acetylated peptides, perhaps acetylation of these residues on Fmt is a preventative measure to restrict trypsinolysis when the protein is not bound to substrate.

#### A Peroxide Stress Resistance Protein

It is clear that YaaA is important during H_2_O_2_ stress, but a defined function for the YaaA protein and the K55 KAT site remains elusive. Two additional studies have provided information about YaaA that may be relevant for determining its function. First, Suzuki et al. (2014) suggested a role for YaaA in ribosome assembly in *Bacillus subtilis* whereby YaaA enhanced a mutant L2 protein binding to 23S rRNA and also led to improved L16 binding to the 50S subunit (Suzuki et al., 2014). Additionally, they showed YaaA only improves binding of an L2 mutant, not WT L2. Thus, they concluded YaaA may only play a role in enhancing binding of the L2 protein and therefore ribosome assembly under harsh physiological conditions. Secondly, Prahlad et al. (2020) showed YaaA binds tightly to duplex DNA with positive cooperativity as the concentration of YaaA increases (Prahlad et al., 2020). However, it is not known whether multiple YaaA molecules bind to the DNA simultaneously or if additional molecules of YaaA bind to a YaaA protein already bound to DNA to form YaaA higher-ordered oligomers. They also reported that YaaA does not bind to a specific DNA sequence and likely binds the minor groove of DNA. Acetylation of K55 may therefore be important for regulating DNA binding to YaaA.

### Discrepancies in Lysine Conservation and Importance of 3D Structural Coverage of Proteins

The main aim of this current study was to elucidate whether the KAT site lysine residues identified as acetylated in *E. coli* K-12 substrate proteins were conserved in homologs by comparing lysine residue conservation based on their 3D structures and primary sequences. While this study was limited to five KAT substrate proteins and their homologs, we showed that discrepancies in lysine residue conservation conclusions between 1D and 3D analyses exist. Furthermore, we found the structural comparisons of homologs provided a more complete picture of specific locations of KAT and AcP site lysine residues between secondary structure boundaries than is possible by primary sequence alone. We also showed that not all AcP or KAT sites were conserved in homologs, even when considering structural domain variability. Moreover, there was no pattern to the type of acetylation that was conserved, e.g., AcP or KAT, across homologs based on either thresholds for sequence identity. In fact, we saw similar trends between different substrate proteins whereby not every protein homolog with high percent sequence identity had all KAT and AcP site lysine residues conserved, even when accounting for homolog structural domain differences.

While the structural comparisons provided insight about residue conservation and detected deviations in structural domains across homologs, we did encounter some challenges to this approach. For example, some proteins exhibit significant mobility of their N- and C-terminal regions of their proteins or other loops where acetylation sites occur, which can render them disordered in the structure and unable to be compared. Despite this, the approach we used enhanced our ability to compare structures with variable conformations, making it easier for the non-expert and expert alike to lessen subjective determination of lysine conservation. However, due to the inherent flexibility of loops and ends of alpha helices and beta strands, and different conformational states of proteins that can occur due to ligand binding or crystal packing, this method will not guarantee completely accurate determination of lysine residue conservation. Careful visual inspection of sites is still required. Further caveats to analyzing lysine residue conservation on KAT and AcP substrate proteins by 3D inspection include limited structural coverage of homologs across diverse taxonomic groups and variability in structural domains of homologs.

### New Questions About Defining and Identifying Conservation of Lysine Acetyltransferase Site Lysine Residues in Homologs

The fact that we observed variability in conservation of KAT sites across substrate protein homologs using either 1D or 3D comparisons disproved our hypothesis that all KAT sites would be conserved across substrate protein homologs with high sequence identity and structural similarity. However, this new insight allows us to ask and address additional questions from different perspectives about why this might occur. It is currently unclear whether lysine acetylation sites that occur on loops or unstructured regions must be in an identical location in protein sequence to be considered conserved, or if a lysine at a different position on the same loop could become acetylated instead due to the inherent flexibility of these secondary structures. For example, could the acetylation site be located within a range of residues on a loop due to its inherent flexibility? The lack of lysine residue conservation in protein substrate homologs may also be due to other factors. For instance, in organisms where these lysine residues are not conserved, do they have different metabolic requirements that would not necessitate these particular proteins be regulated in the same manner? Is it possible that this lack of lysine conservation is restricted to only certain substrate proteins, or does a different KAT protein with different substrate specificity exist in these organisms? Alternatively, is the lack of lysine conservation across substrate proteins due to the fact that not all organisms have the same KAT proteins? To correctly interpret the lysine conservation data in homologs across different taxonomic groups, we need to know more about KAT conservation in these organisms. While previously reported data show conservation of some KATs in different organisms, the sequence similarity between GNAT proteins of similar function can be quite low and make accurate bioinformatic predictions of conservation challenging. Additionally, an expanded analysis of acetylation sites on a larger dataset of KAT and AcP substrate proteins is required and is currently underway in our laboratory. The results and workflow developed in this current study provide insight as to how protein structures can be utilized to obtain more accurate information about conservation of acetylation sites on substrate proteins.

## Data Availability Statement

The original contributions presented in the study are included in the article/Supplementary Material, further inquiries can be directed to the corresponding author.

## Author Contributions

MK conceptualized the project. KA, AT, NN, and NA wrote code. KJ, VL, KA, AT, ML, and MK performed experiments and data analysis and wrote portions of the manuscript and prepared figures. KJ and MK wrote the first draft of the manuscript. KJ, VL, KA, AT, ML, NA, and MK edited the manuscript. All authors contributed to the article and approved the submitted version.

## Conflict of Interest

The authors declare that the research was conducted in the absence of any commercial or financial relationships that could be construed as a potential conflict of interest.

## Publisher’s Note

All claims expressed in this article are solely those of the authors and do not necessarily represent those of their affiliated organizations, or those of the publisher, the editors and the reviewers. Any product that may be evaluated in this article, or claim that may be made by its manufacturer, is not guaranteed or endorsed by the publisher.

## Footnotes

1
https://espript.ibcp.fr

2
https://www.uniprot.org/

3
https://www.ebi.ac.uk/Tools/msa/muscle/
