# Supplementary material for: Investigation of the Importance of Protein 3D Structure for Assessing Conservation of Lysine Acetylation Sites in Protein Homologs
Source: Front Microbiol. 2022 Jan 31;12:805181. doi: 10.3389/fmicb.2021.805181 (PMC8843374; doi:10.3389/fmicb.2021.805181)
Supplement: Supplementary file 1 [file Data_Sheet_1.PDF]

Supplemental Figure SF1. Flowchart and details of Python scripts used in this study. Individual Python scripts were created to obtain data, align and compare structures, and generate the multiple sequence alignment of homologs based on structural alignments to the target protein structures. Output were saved as excel, xml, and cif files. Abbreviations include: Protein Data Bank (PDB) and Flexible structure AlignmenT by Chaining Aligned fragment pairs allowing Twists (FATCAT).

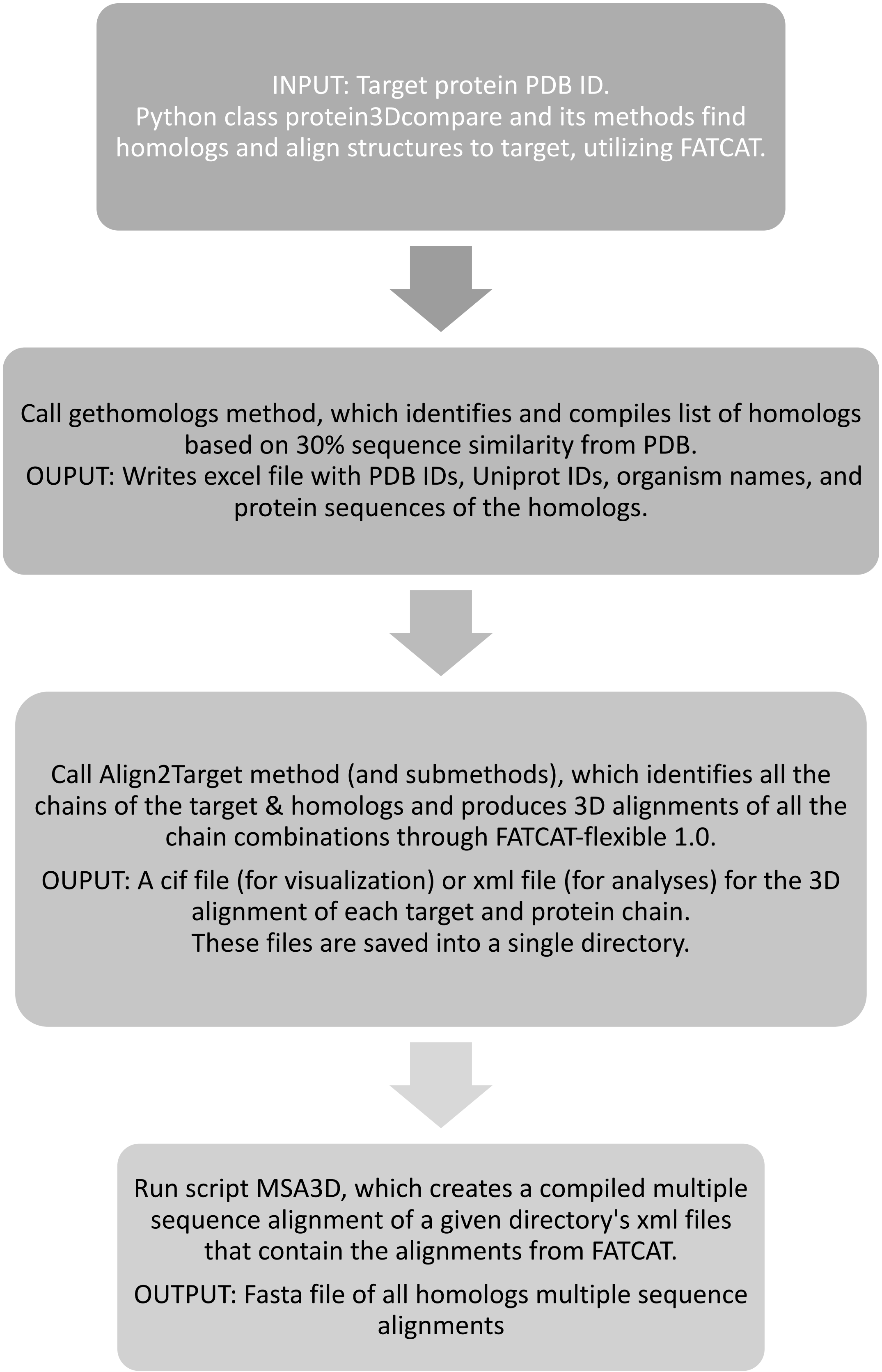
INPUT: Target protein PDB ID.
Python class protein3Dcompare and its methods find homologs and align structures to target, utilizing FATCAT.
Call gethomologs method, which identifies and compiles list of homologs based on 30% sequence similarity from PDB.
OUPUT: Writes excel file with PDB IDs, Uniprot IDs, organism names, and protein sequences of the homologs.
Call Align2Target method (and submethods), which identifies all the chains of the target & homologs and produces 3D alignments of all the chain combinations through FATCAT-flexible 1.0.
OUPUT: A cif file (for visualization) or xml file (for analyses) for the 3D alignment of each target and protein chain.
These files are saved into a single directory.
Run script MSA3D, which creates a compiled multiple sequence alignment of a given directory's xml files that contain the alignments from FATCAT.
OUTPUT: Fasta file of all homologs multiple sequence alignments
