# Supplementary material for: Investigation of the Importance of Protein 3D Structure for Assessing Conservation of Lysine Acetylation Sites in Protein Homologs
Source: Front Microbiol. 2022 Jan 31;12:805181. doi: 10.3389/fmicb.2021.805181 (PMC8843374; doi:10.3389/fmicb.2021.805181)

**Supplemental Figure SF2A. Compiled pairwise sequence (Cobalt) and structural (FATCAT) alignments between the *E. coli* substrate protein target (Adk-adenylate kinase; PDB ID: 1ake) and homologs sorted by UniProt ID.** Lysine residues previously identified as acetylated in the target protein are highlighted in yellow in the sequence alignments and FATCAT structural alignment xml files to examine conservation. 3D protein structures are shown as ribbon representations with the target protein in cyan and the homolog protein in gray. Blue lysine residues correspond to KAT (lysine acetyltransferase) acetylation sites, red lysine residues correspond to AcP (acetyl phosphate) acetylation sites, and purple lysine residues correspond to sites acetylated by both KAT and AcP. 1D sequence alignments are not shown between *E. coli* proteins because sequences were identical.

UniProt ID: A0A0J9X1X4

PDB ID: 3X2S_A

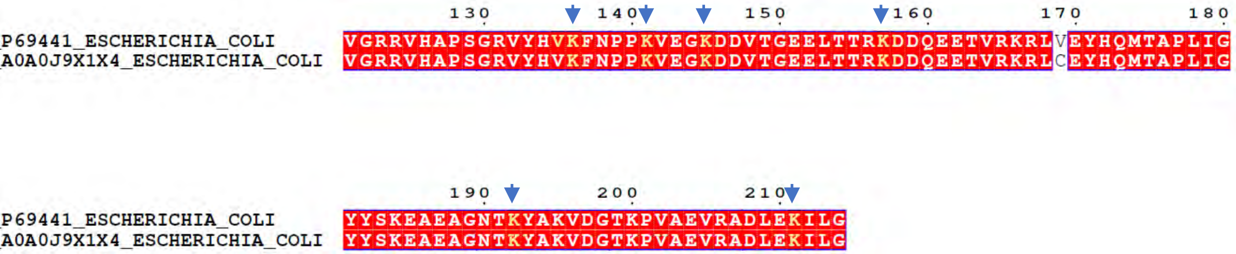


Full sequences in supplemental file.

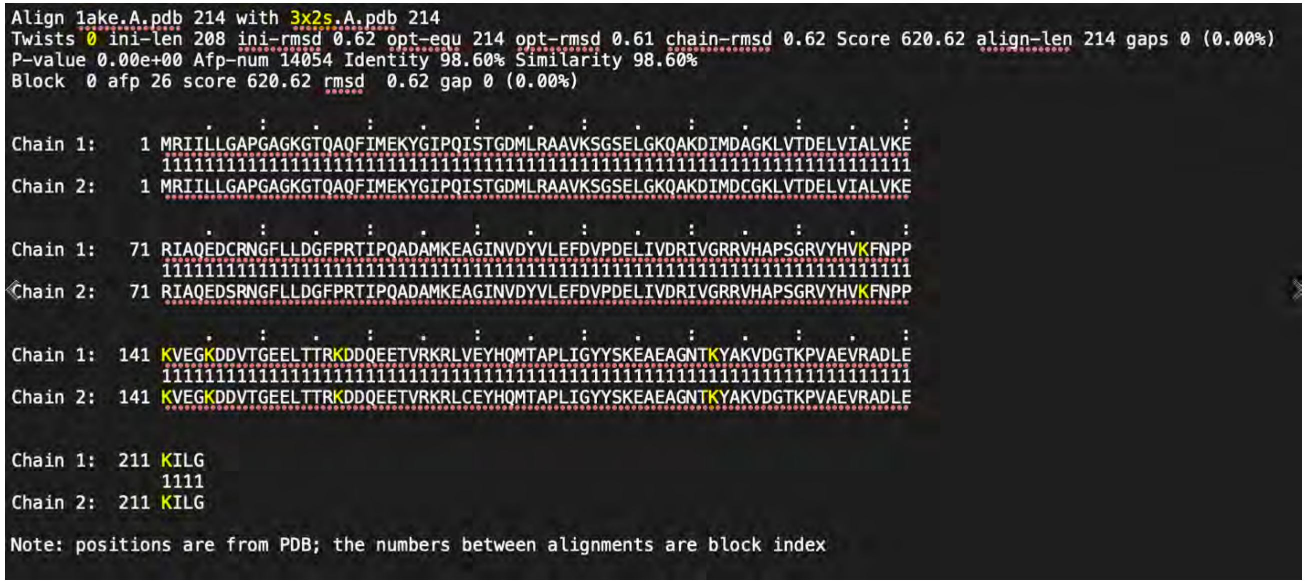

```
Align 1ake.A.pdb 214 with 3x2s.A.pdb 214
Twists 0 ini-len 208 ini-rmsd 0.62 opt-equ 214 opt-rmsd 0.61 chain-rmsd 0.62 Score 620.62 align-len 214 gaps 0 (0.00%)
P-value 0.00e+00 Afp-num 14054 Identity 98.60% Similarity 98.60%
Block  0 afp 26 score 620.62 rmsd  0.62 gap 0 (0.00%)

Chain 1:    1 MRIILLGAPGAGKGTQAQFIMEKYGIPQISTGDMLRAAVKSGSELGKQAKDIMDAGKLVTDELVIALVKE
              1111111111111111111111111111111111111111111111111111111111111111111111
Chain 2:    1 MRIILLGAPGAGKGTQAQFIMEKYGIPQISTGDMLRAAVKSGSELGKQAKDIMDCGKLVTDELVIALVKE

Chain 1:   71 RIAQEDCRNGFLLDGFPRTIPQADAMKEAGINVDYVLEFDVPDELIVDRIVGRRVHAPSGRVYHVKFNPP
              1111111111111111111111111111111111111111111111111111111111111111111111
Chain 2:   71 RIAQEDSRNGFLLDGFPRTIPQADAMKEAGINVDYVLEFDVPDELIVDRIVGRRVHAPSGRVYHVKFNPP

Chain 1:  141 KVEGKDDVTGEELTTRKDDQEETVRKRLVEYHQMTAPLIGYYSKEAEAGNTKYAKVDGTKPVAEVRADLE
              1111111111111111111111111111111111111111111111111111111111111111111111
Chain 2:  141 KVEGKDDVTGEELTTRKDDQEETVRKRLCEYHQMTAPLIGYYSKEAEAGNTKYAKVDGTKPVAEVRADLE

Chain 1:  211 KILG
              1111
Chain 2:  211 KILG

Note: positions are from PDB; the numbers between alignments are block index
```

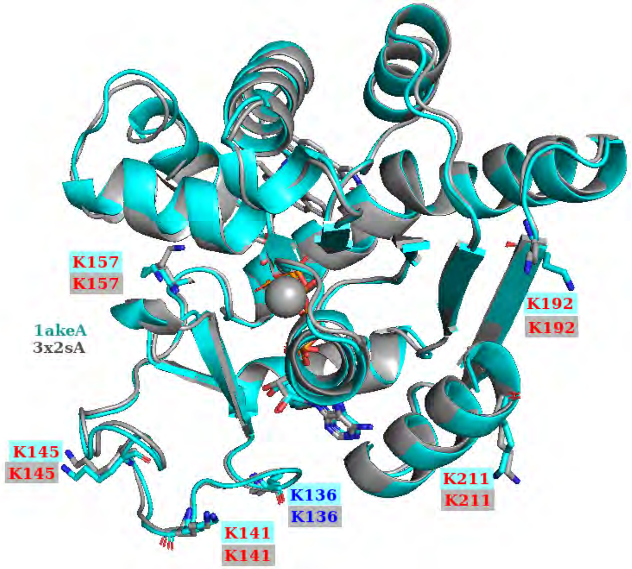


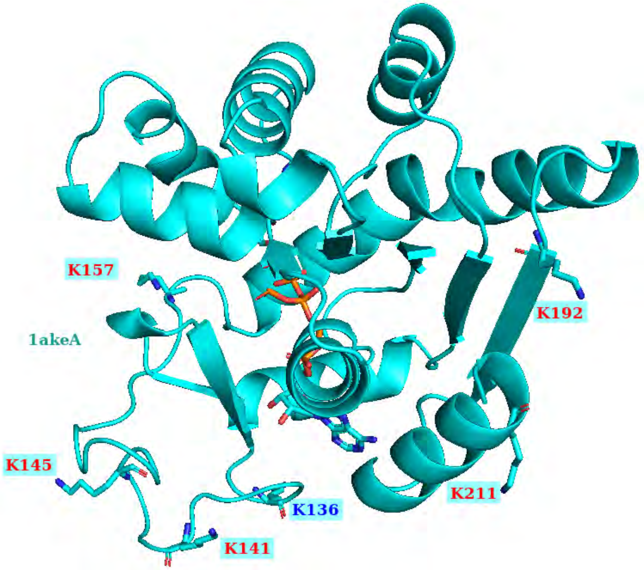


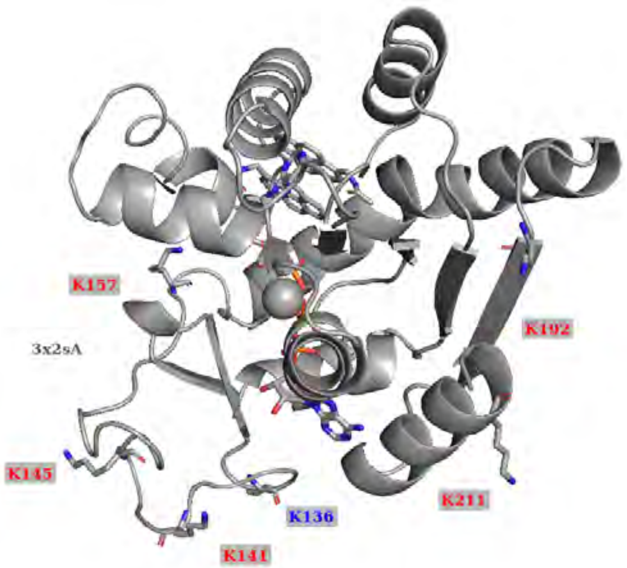

UniProt ID: A0A2R2JFU5

PDB ID: 5X6K_A

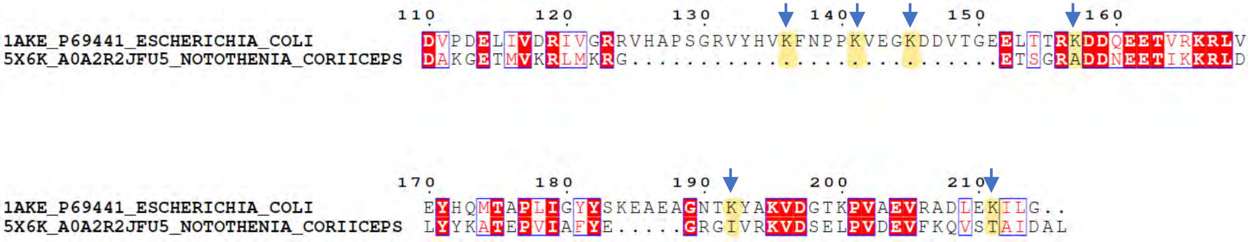


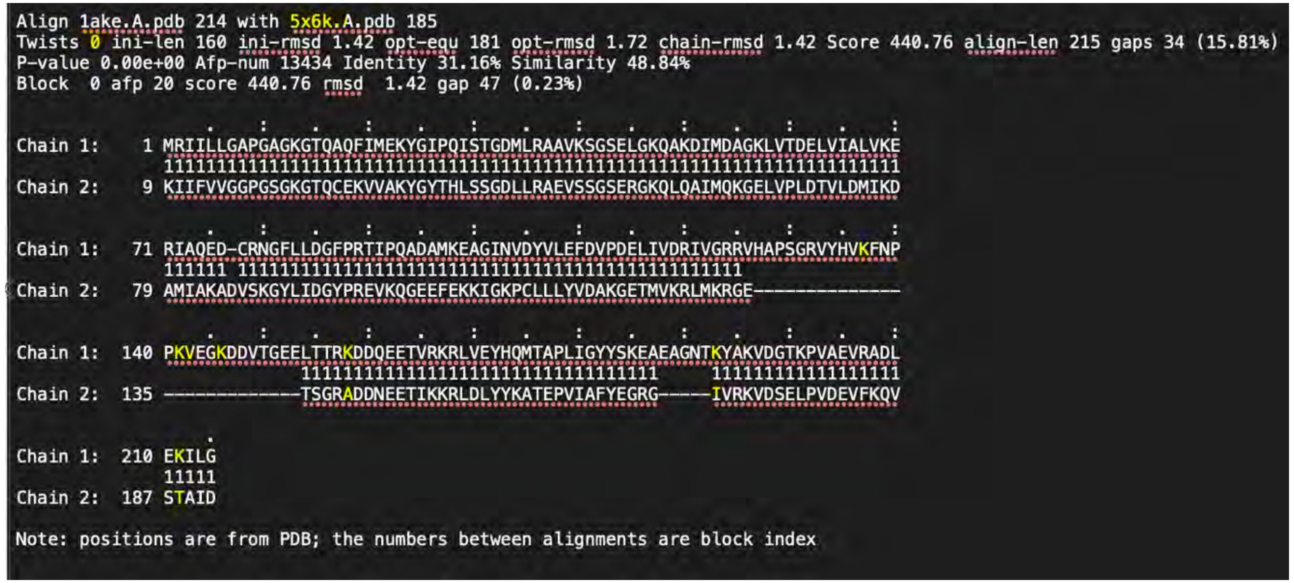

```
Align 1ake.A.pdb 214 with 5x6k.A.pdb 185
Twists 0 ini-len 160 ini-rmsd 1.42 opt-equ 181 opt-rmsd 1.72 chain-rmsd 1.42 Score 440.76 align-len 215 gaps 34 (15.81%)
P-value 0.00e+00 Afp-num 13434 Identity 31.16% Similarity 48.84%
Block  0 afp 20 score 440.76 rmsd  1.42 gap 47 (0.23%)

                .    :    .    :    .    :    .    :    .    :    .    :    .    :
Chain 1:    1 MRIILLGAPGAGKGTQAQFIMEKYGIPQISTGDMLRAAVKSGSELGKQAKDIMDAGKLVTDELVIALVKE
              1111111111111111111111111111111111111111111111111111111111111111111111
Chain 2:    9 KIIFVVGGPGSGKGTQCEKVVAKYGYTHLSSGDLLRAEVSSGSERGKQLQAIMQKGELVPLDTVLDMIKD

                .    :    .    :    .    :    .    :    .    :    .    :    .    :
Chain 1:   71 RIAQED-CRNGFLLDGFPRTIPQADAMKEAGINVDYVLEFDVPDELIVDRIVGRRVHAPSGRVYHVKFNP
              111111 1111111111111111111111111111111111111111111111111
Chain 2:   79 AMIAKADVSKGYLIDGYPREVKQGEEFEKKIGKPCLLLYVDAKGETMVKRLMKRGE--------------

                .    :    .    :    .    :    .    :    .    :    .    :    .    :
Chain 1:  140 PKVEGKDDVTGEELTTRKDDQEETVRKRLVEYHQMTAPLIGYYSKEAEAGNTKYAKVDGTKPVAEVRADL
                          111111111111111111111111111111111     11111111111111111
Chain 2:  135 -------------TSGRADDNEETIKKRLDLYYKATEPVIAFYEGRG-----IVRKVDSELPVDEVFKQV

                .
Chain 1:  210 EKILG
              11111
Chain 2:  187 STAID

Note: positions are from PDB; the numbers between alignments are block index
```

Full sequences in supplemental file.

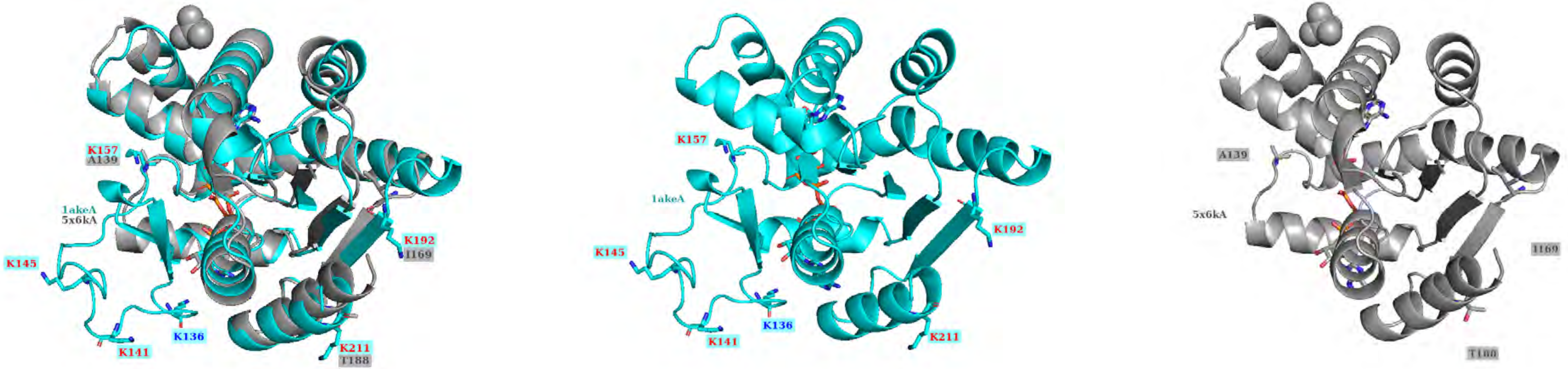

UniProt ID: A0A2R2JFU5

PDB ID: 5YCB_B

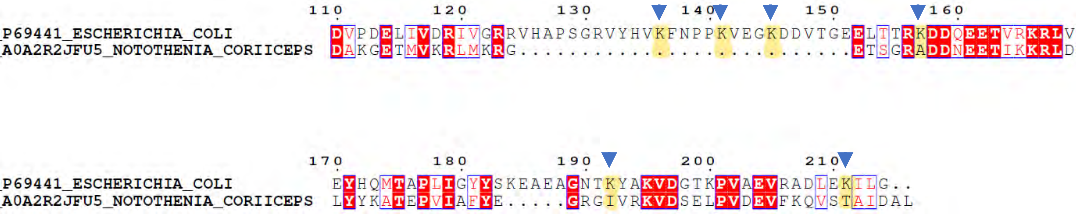


Full sequences in supplemental file.

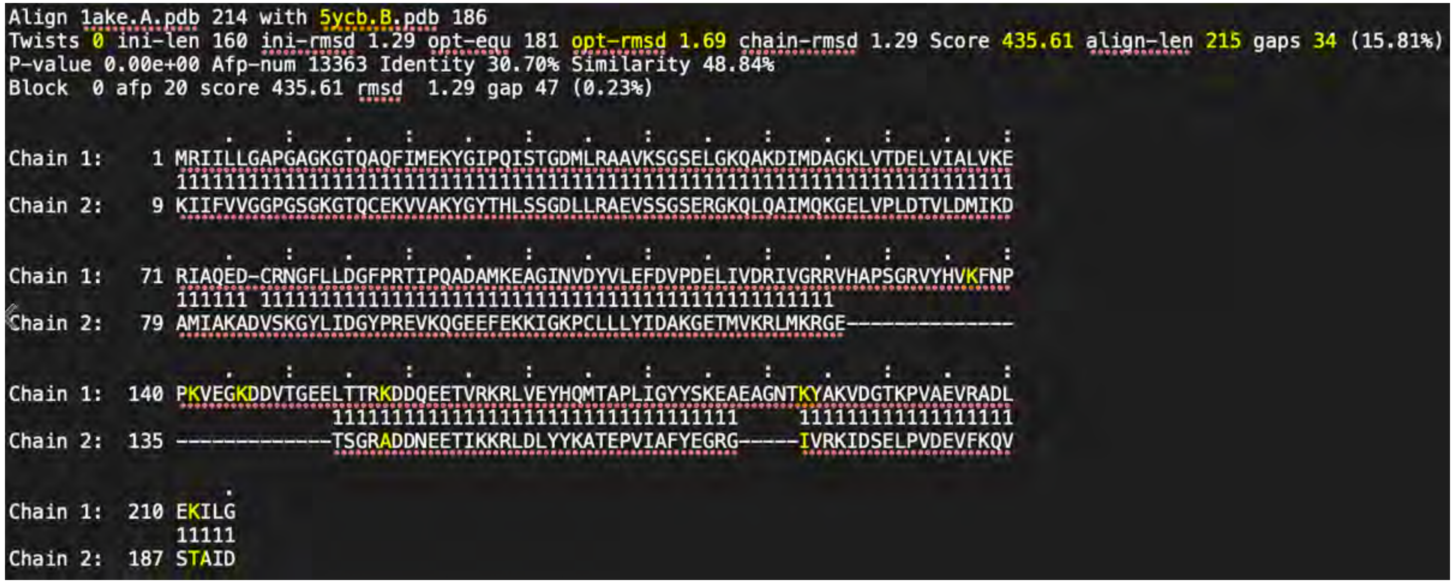

```
Align 1ake.A.pdb 214 with 5ycb.B.pdb 186
Twists 0 ini-len 160 ini-rmsd 1.29 opt-equ 181 opt-rmsd 1.69 chain-rmsd 1.29 Score 435.61 align-len 215 gaps 34 (15.81%)
P-value 0.00e+00 Afp-num 13363 Identity 30.70% Similarity 48.84%
Block  0 afp 20 score 435.61 rmsd  1.29 gap 47 (0.23%)

Chain 1:    1 MRIILLGAPGAGKGTQAQFIMEKYGIPQISTGDMLRAAVKSGSELGKQAKDIMDAGKLVTDELVIALVKE
                1111111111111111111111111111111111111111111111111111111111111111111111
Chain 2:    9 KIIFVVGGPGSGKGTQCEKVVAKYGYTHLSSGDLLRAEVSSGSERGKQLQAIMQKGELVPLDTVLDMIKD

Chain 1:   71 RIAQED-CRNGFLLDGFPRTIPQADAMKEAGINVDYVLEFDVPDELIVDRIVGRRVHAPSGRVYHVKFNP
                111111 1111111111111111111111111111111111111111111111111
Chain 2:   79 AMIAKADVSKGYLIDGYPREVKQGEEFEKKIGKPCLLLYIDAKGETMVKRLMKRGE--------------

Chain 1:  140 PKVEGKDDVTGEELTTRKDDQEETVRKRLVEYHQMTAPLIGYYSKEAEAGNTKYAKVDGTKPVAEVRADL
                             1111111111111111111111111111111111     111111111111111111
Chain 2:  135 -------------TSGRADDNEETIKKRLDLYYKATEPVIAFYEGRG-----IVRKIDSELPVDEVFKQV

Chain 1:  210 EKILG
              11111
Chain 2:  187 STAID
```

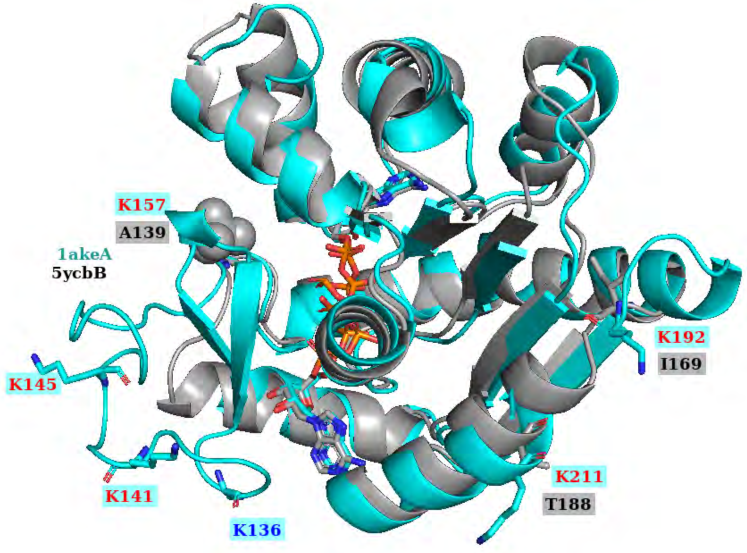


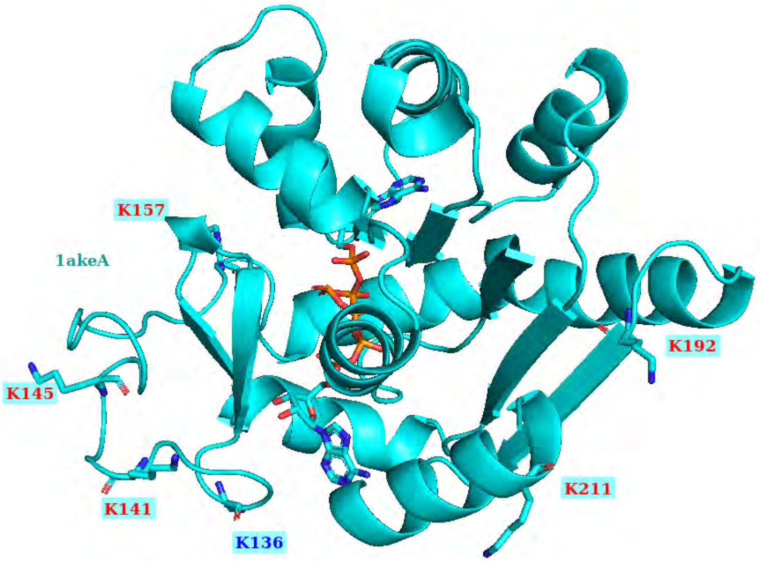


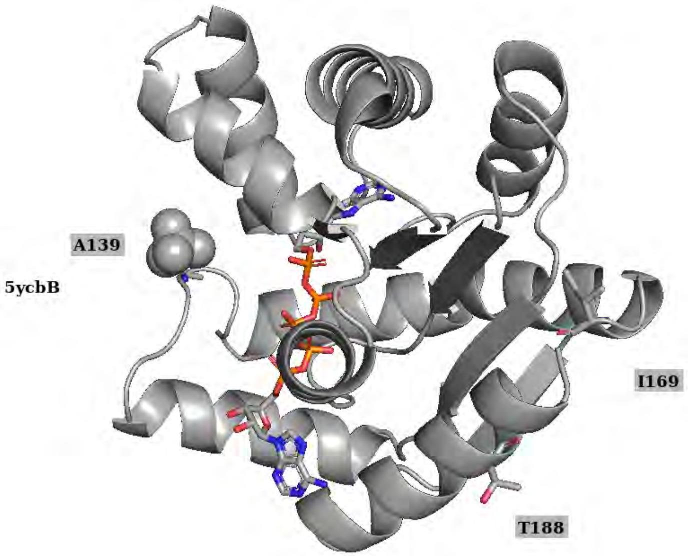

UniProt ID: A0A2R2JFU5

PDB ID: 5YCC_B

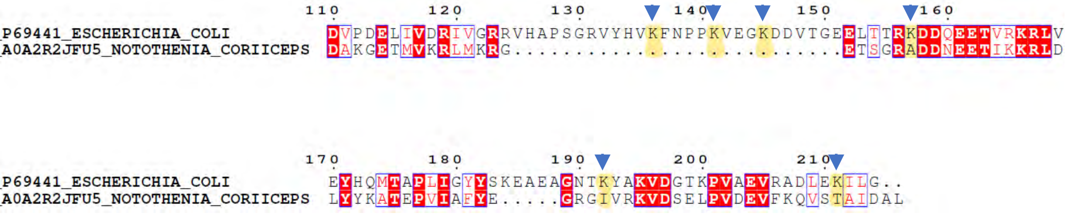


Full sequences in supplemental file.

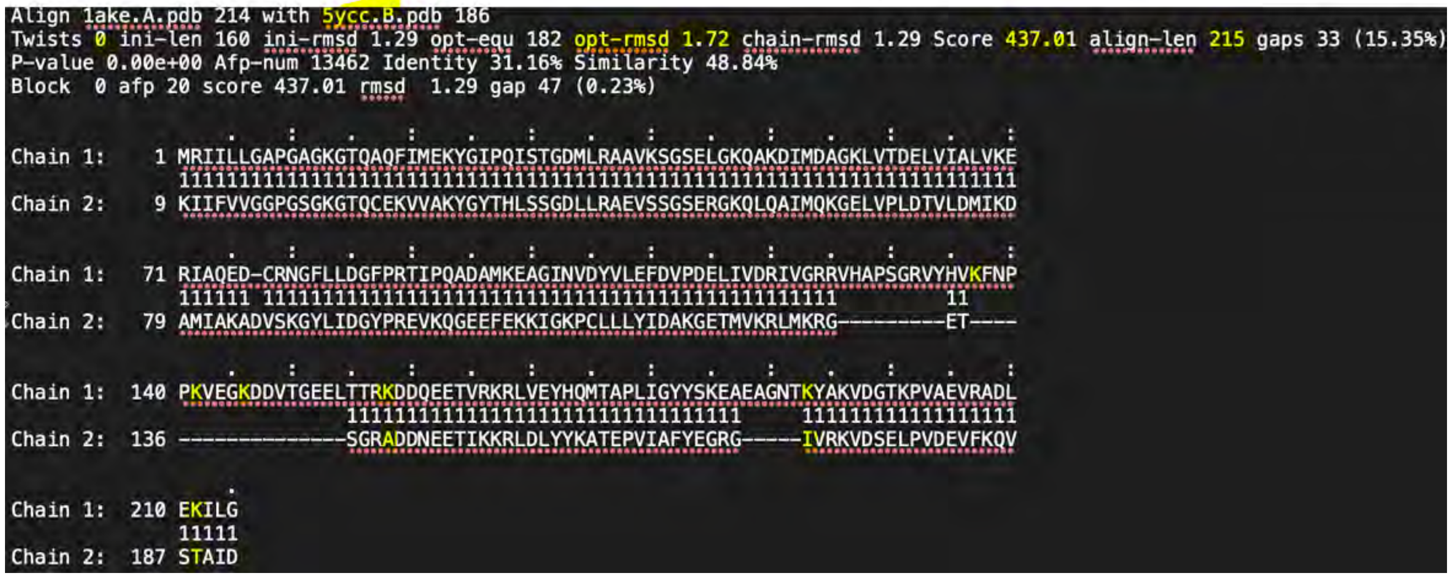

```
Align 1ake.A.pdb 214 with 5ycc.B.pdb 186
Twists 0 ini-len 160 ini-rmsd 1.29 opt-equ 182 opt-rmsd 1.72 chain-rmsd 1.29 Score 437.01 align-len 215 gaps 33 (15.35%)
P-value 0.00e+00 Afp-num 13462 Identity 31.16% Similarity 48.84%
Block  0 afp 20 score 437.01 rmsd  1.29 gap 47 (0.23%)

                .    :    .    :    .    :    .    :    .    :    .    :    .    :
Chain 1:    1 MRIILLGAPGAGKGTQAQFIMEKYGIPQISTGDMLRAAVKSGSELGKQAKDIMDAGKLVTDELVIALVKE
              1111111111111111111111111111111111111111111111111111111111111111111111
Chain 2:    9 KIIFVVGGPGSGKGTQCEKVVAKYGYTHLSSGDLLRAEVSSGSERGKQLQAIMQKGELVPLDTVLDMIKD

                .    :    .    :    .    :    .    :    .    :    .    :    .    :
Chain 1:   71 RIAQED-CRNGFLLDGFPRTIPQADAMKEAGINVDYVLEFDVPDELIVDRIVGRRVHAPSGRVYHVKFNP
              111111 11111111111111111111111111111111111111111111111111         11
Chain 2:   79 AMIAKADVSKGYLIDGYPREVKQGEEFEKKIGKPCLLLYIDAKGETMVKRLMKRG---------ET----

                .    :    .    :    .    :    .    :    .    :    .    :    .    :
Chain 1:  140 PKVEGKDDVTGEELTTRKDDQEETVRKRLVEYHQMTAPLIGYYSKEAEAGNTKYAKVDGTKPVAEVRADL
                            111111111111111111111111111111111     1111111111111111
Chain 2:  136 --------------SGRADDNEETIKKRLDLYYKATEPVIAFYEGRG-----IVRKVDSELPVDEVFKQV

                .
Chain 1:  210 EKILG
              11111
Chain 2:  187 STAID
```

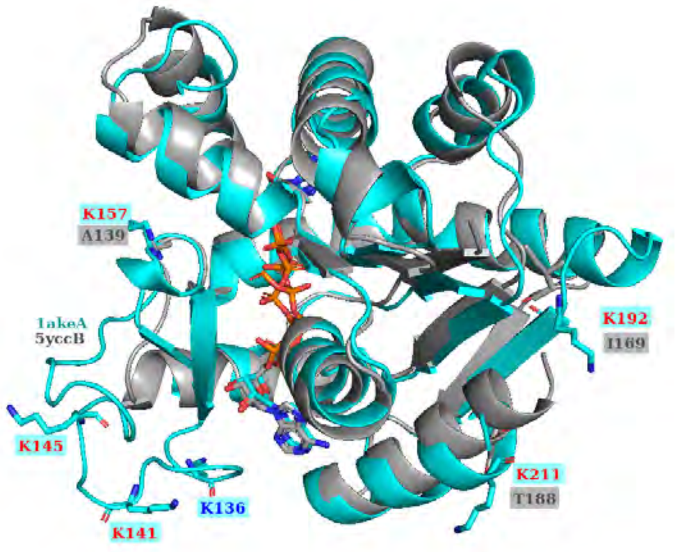


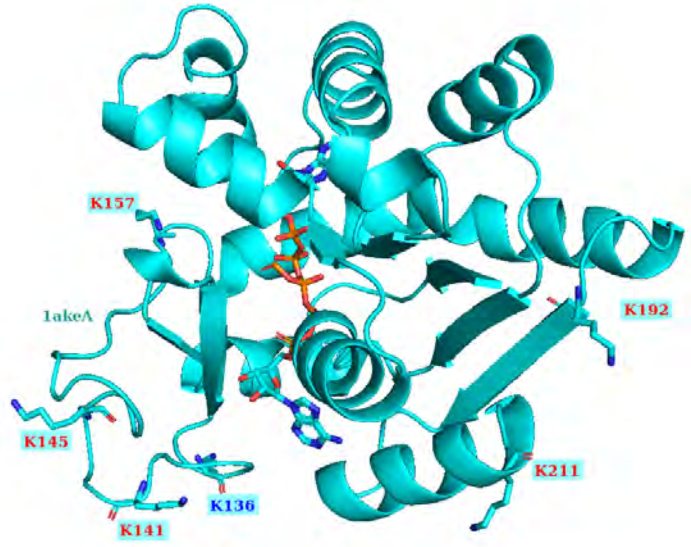


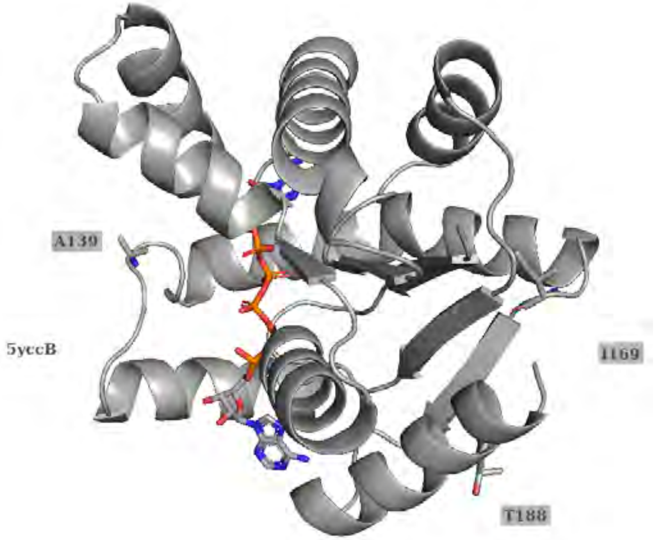

UniProt ID: A0A2R2JFU6

PDB ID: 5X6L_A

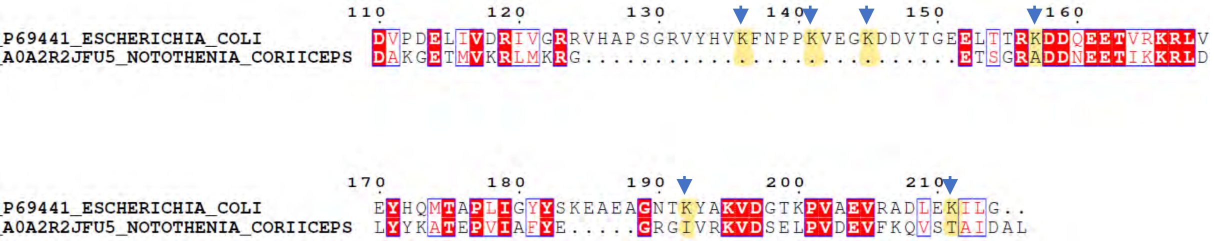


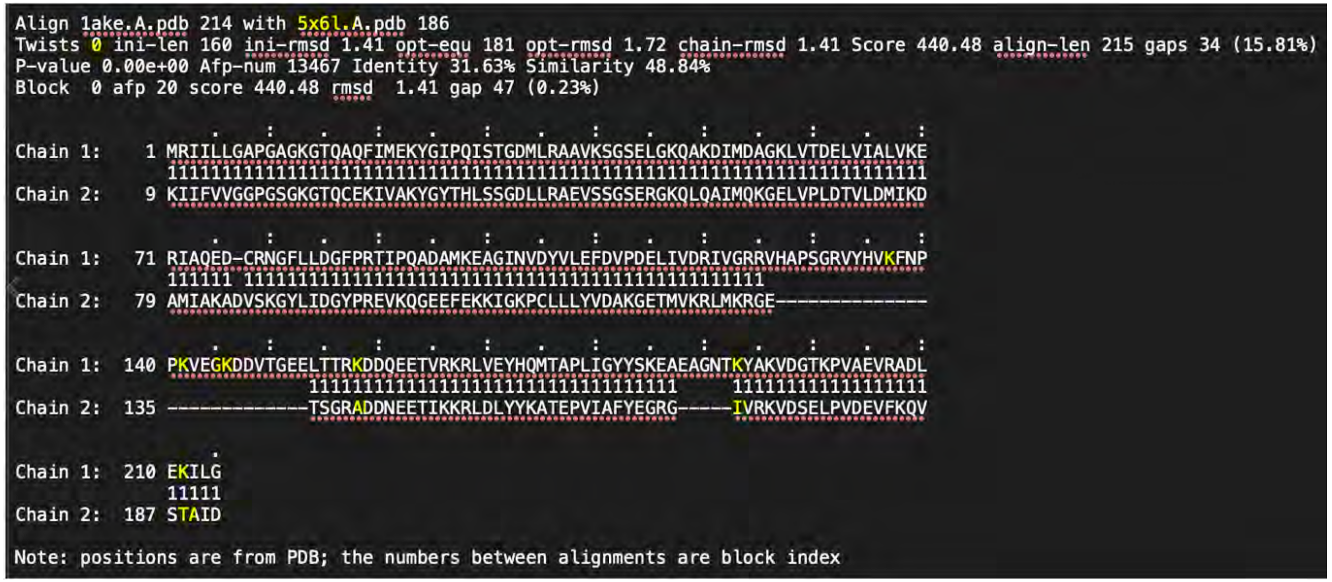

```
Align 1ake.A.pdb 214 with 5x6l.A.pdb 186
Twists 0 ini-len 160 ini-rmsd 1.41 opt-equ 181 opt-rmsd 1.72 chain-rmsd 1.41 Score 440.48 align-len 215 gaps 34 (15.81%)
P-value 0.00e+00 Afp-num 13467 Identity 31.63% Similarity 48.84%
Block  0 afp 20 score 440.48 rmsd  1.41 gap 47 (0.23%)

                .    :    .    :    .    :    .    :    .    :    .    :    .    :
Chain 1:    1 MRIILLGAPGAGKGTQAQFIMEKYGIPQISTGDMLRAAVKSGSELGKQAKDIMDAGKLVTDELVIALVKE
              1111111111111111111111111111111111111111111111111111111111111111111111
Chain 2:    9 KIIFVVGGPGSGKGTQCEKIVAKYGYTHLSSGDLLRAEVSSGSERGKQLQAIMQKGELVPLDTVLDMIKD

                .    :    .    :    .    :    .    :    .    :    .    :    .    :
Chain 1:   71 RIAQED-CRNGFLLDGFPRTIPQADAMKEAGINVDYVLEFDVPDELIVDRIVGRRVHAPSGRVYHVKFNP
              111111 111111111111111111111111111111111111111111111111111111
Chain 2:   79 AMIAKADVSKGYLIDGYPREVKQGEEFEKKIGKPCLLLYVDAKGETMVKRLMKRGE--------------

                .    :    .    :    .    :    .    :    .    :    .    :    .    :
Chain 1:  140 PKVEGKDDVTGEELTTRKDDQEETVRKRLVEYHQMTAPLIGYYSKEAEAGNTKYAKVDGTKPVAEVRADL
                           111111111111111111111111111111111111     111111111111111111
Chain 2:  135 -------------TSGRADDNEETIKKRLDLYYKATEPVIAFYEGRG-----IVRKVDSELPVDEVFKQV

                .
Chain 1:  210 EKILG
              11111
Chain 2:  187 STAID

Note: positions are from PDB; the numbers between alignments are block index
```

Full sequences in supplemental file.

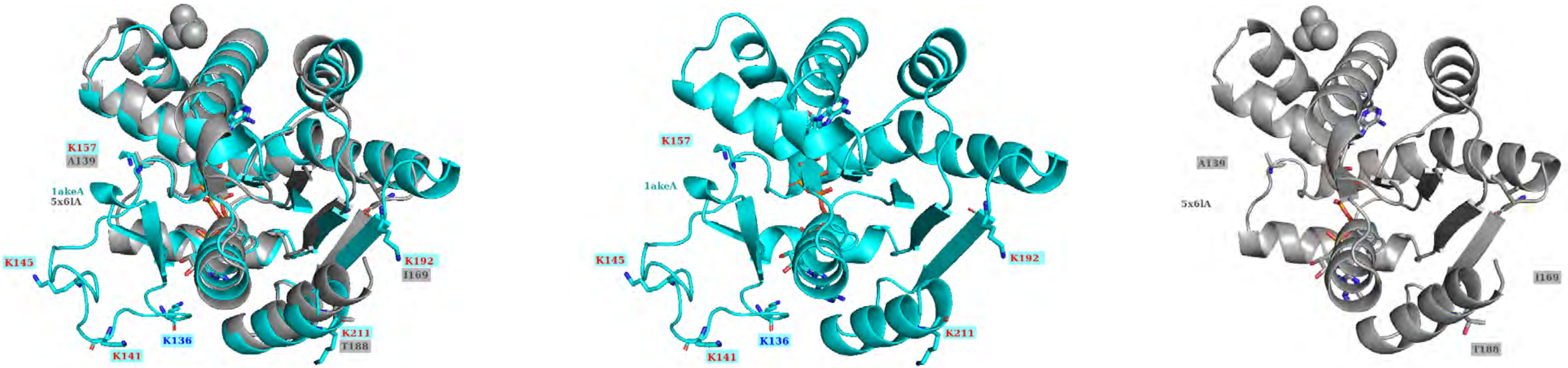

UniProt ID: A0A2R2JFU6

PDB ID: 5XRU_A

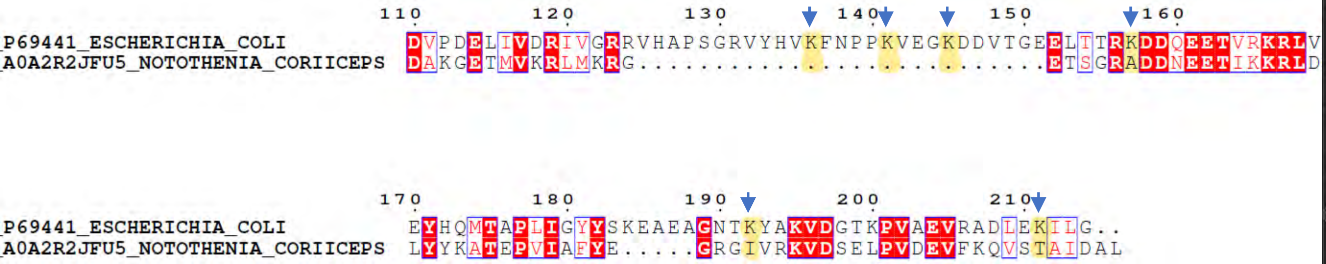


Full sequences in supplemental file.

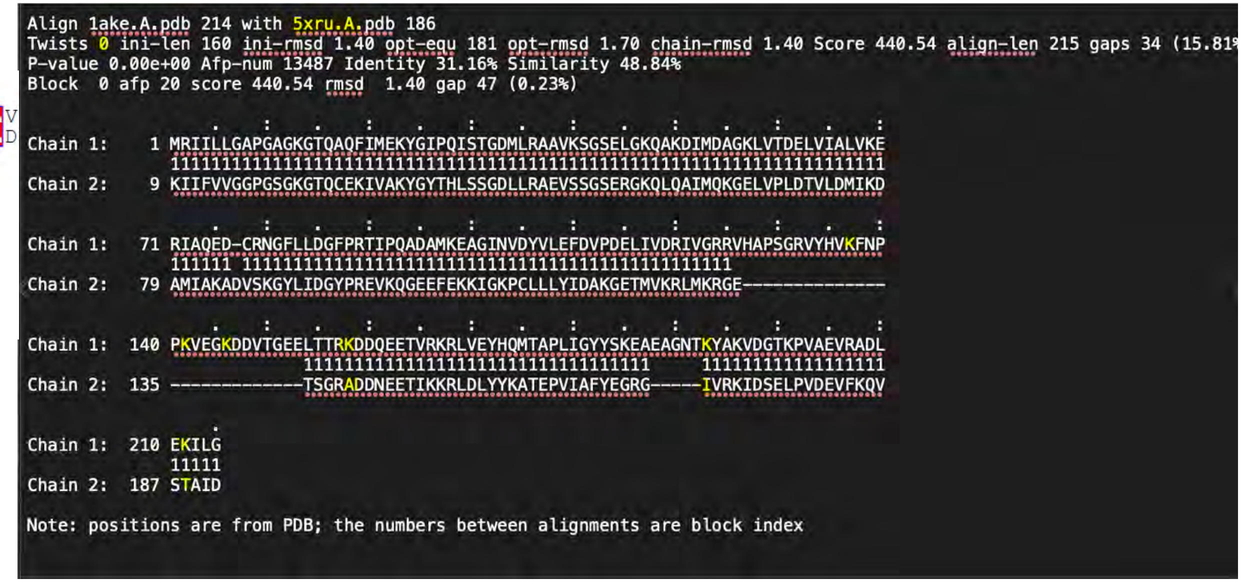


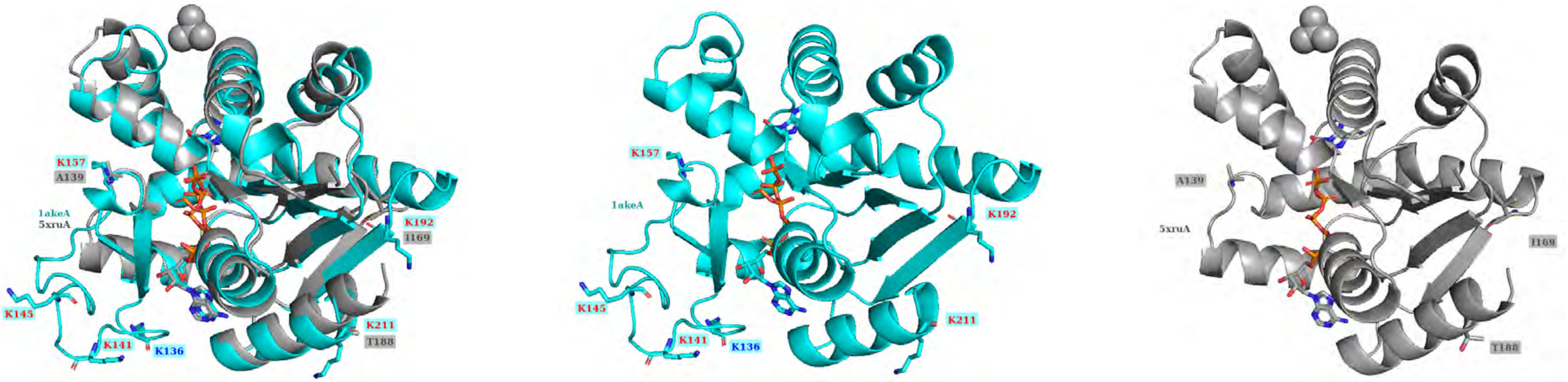

UniProt ID: A0A452CSM2

PDB ID: 5YCD_A

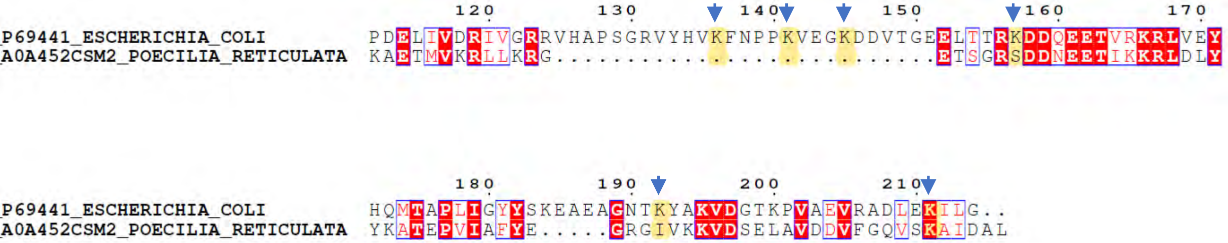


Full sequences in supplemental file.

```
Align 1ake.A.pdb 214 with 5ycd.A.pdb 191
Twists 0 ini-len 160 ini-rmsd 1.41 opt-equ 181 opt-rmsd 1.72 chain-rmsd 1.41 Score 440.29 align-len 215 gaps 34 (15.81%)
P-value 0.00e+00 Afp-num 13777 Identity 31.63% Similarity 48.84%
Block  0 afp 20 score 440.29 rmsd  1.41 gap 47 (0.23%)

                  .    :    .    :    .    :    .    :    .    :    .    :    .    :
Chain 1:    1 MRIILLGAPGAGKGTQAQFIMEKYGIPQISTGDMLRAAVKSGSELGKQAKDIMDAGKLVTDELVIALVKE
              1111111111111111111111111111111111111111111111111111111111111111111111
Chain 2:    9 KIIFVVGGPGSGKGTQCEKIVAKYGYTHLSSGDLLRAEVASGSERGKQLQAIMQKGELVPLDTVLDMIKD

                  .    :    .    :    .    :    .    :    .    :    .    :    .    :
Chain 1:   71 RIAQED-CRNGFLLDGFPRTIPQADAMKEAGINVDYVLEFDVPDELIVDRIVGRRVHAPSGRVYHVKFNP
              111111 11111111111111111111111111111111111111111111111
Chain 2:   79 AMIAKADVSKGFLIDGYPREVKQGEEFEKKIGKPCLLLYVDAKAETMVKRLLKRGE--------------

                  .    :    .    :    .    :    .    :    .    :    .    :    .    :
Chain 1:  140 PKVEGKDDVTGEELTTRKDDQEETVRKRLVEYHQMTAPLIGYYSKEAEAGNTKYAKVDGTKPVAEVRADL
                           111111111111111111111111111111111     111111111111111111
Chain 2:  135 -------------TSGRSDDNEETIKKRLDLYYKATEPVIAFYEGRG-----IVKKVDSELAVDDVFGQV

                  .
Chain 1:  210 EKILG
              11111
Chain 2:  187 SKAID

Note: positions are from PDB; the numbers between alignments are block index
```

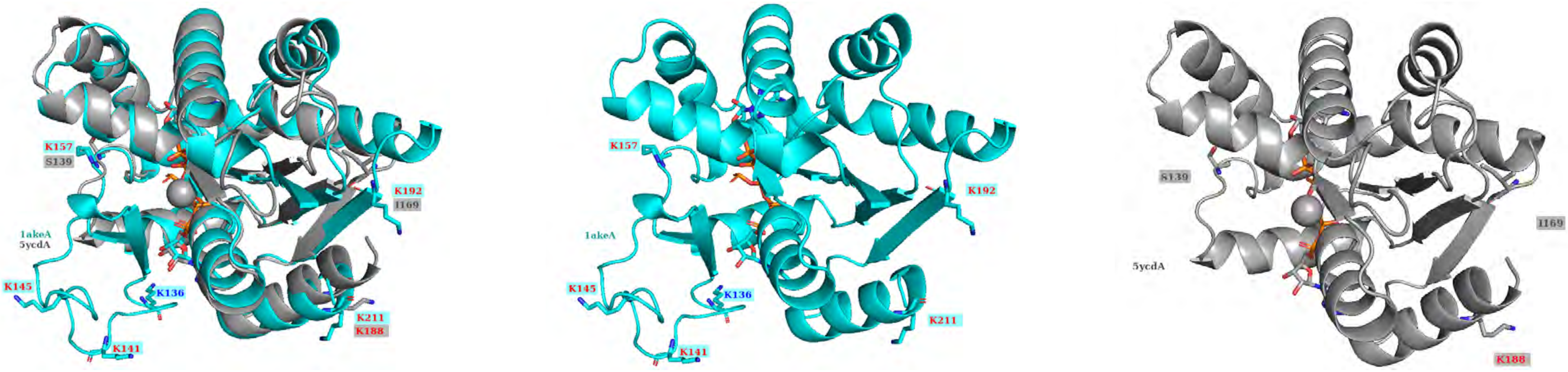

# UniProt ID: A7ZIN4
# PDB ID: 6HAP_A

```
                         130        140        150        160        170        180
P69441_ESCHERICHIA_COLI  VGRRVHAPSGRVYHVKFNPPKVEGKDDVTGEELTTRKDDQEETVRKRLVEYHQMTAPLIG
A7ZIN4_ESCHERICHIA_COLI  VGRRVHAPSGRVYHVKFNPPKVEGKDDVTGEELTTRKDDQEETVRKRLVEYHQMTAPLIG

                         190        200        210
P69441_ESCHERICHIA_COLI  YYSKEAEAGNTKYAKVDGTKPVAEVRADLEKILG
A7ZIN4_ESCHERICHIA_COLI  YYSKEAEAGNTKYAKVDGTKPVAEVRADLEKILG
```

Full sequences in supplemental file.

```
Align 1ake.A.pdb 214 with 6hap.A.pdb 214
Twists 0 ini-len 208 ini-rmsd 1.24 opt-equ 214 opt-rmsd 1.34 chain-rmsd 1.24 Score 612.53 align-len 214 gaps 0 (0.00%)
P-value 0.00e+00 Afp-num 14167 Identity 98.13% Similarity 98.60%
Block  0 afp 26 score 612.53 rmsd  1.24 gap 5 (0.02%)

                .    :    .    :    .    :    .    :    .    :    .    :    .    :
Chain 1:    1 MRIILLGAPGAGKGTQAQFIMEKYGIPQISTGDMLRAAVKSGSELGKQAKDIMDAGKLVTDELVIALVKE
              1111111111111111111111111111111111111111111111111111111111111111111111
Chain 2:    1 MRIILLGAPGAGKGTQAQFIMEKYGIPQISTGDMLRAAVKSGSELGKQAKDIMDAGKLVTDELVIALVRE

                .    :    .    :    .    :    .    :    .    :    .    :    .    :
Chain 1:   71 RIAQEDCRNGFLLDGFPRTIPQADAMKEAGINVDYVLEFDVPDELIVDRIVGRRVHAPSGRVYHVKFNPP
              1111111111111111111111111111111111111111111111111111111111111111111111
Chain 2:   71 RICQEDSRNGFLLDGFPRTIPQADAMKEAGINVDYVLEFDVPDELIVDRIVGRRVHAPSGRVYHVKFNPP

                .    :    .    :    .    :    .    :    .    :    .    :    .    :
Chain 1:  141 KVEGKDDVTGEELTTRKDDQEETVRKRLVEYHQMTAPLIGYYSKEAEAGNTKYAKVDGTKPVAEVRADLE
              1111111111111111111111111111111111111111111111111111111111111111111111
Chain 2:  141 KVEGKDDVTGEELTTRKDDQEETVRKRLVEYHQMTAPLIGYYSKEAEAGNTKYAKVDGTKPVCEVRADLE

Chain 1:  211 KILG
              1111
Chain 2:  211 KILG

Note: positions are from PDB; the numbers between alignments are block index
```

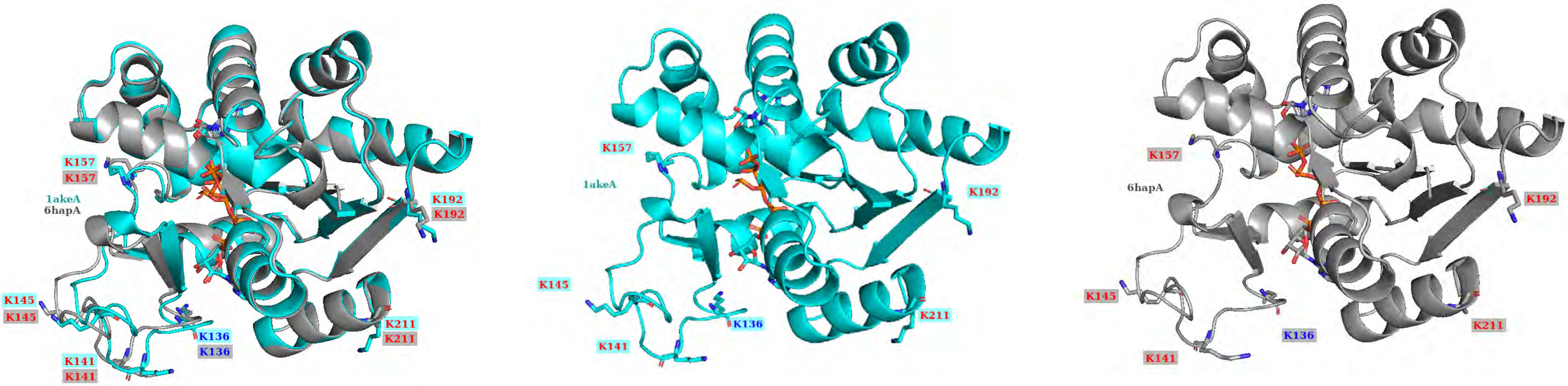

UniProt ID: C7U112
PDB ID: 2XB4_A

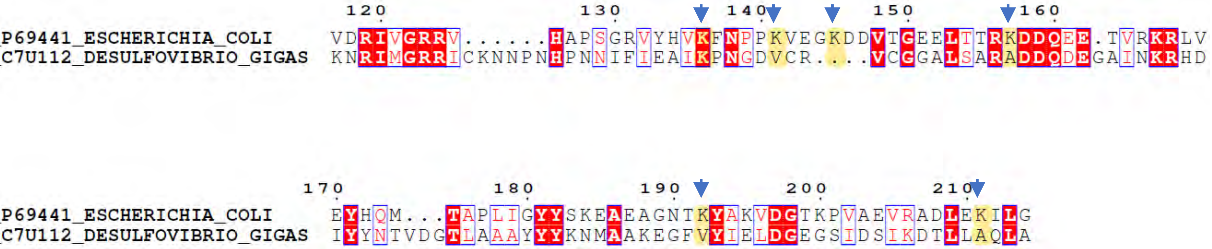


Full sequences in supplemental file.

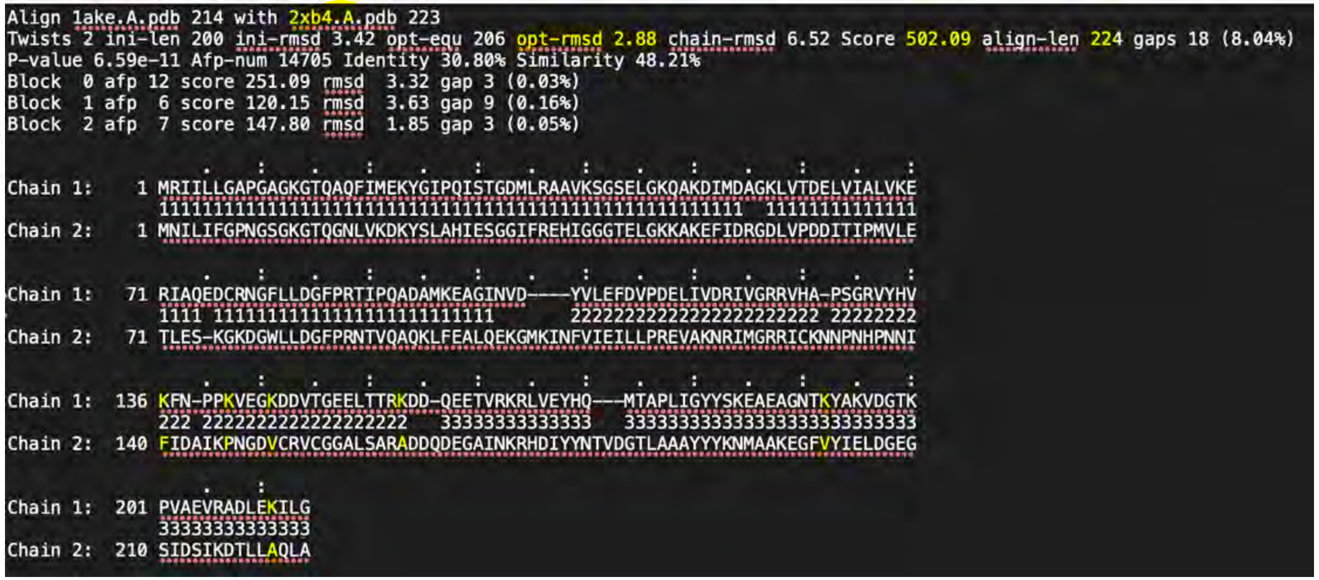

```
Align 1ake.A.pdb 214 with 2xb4.A.pdb 223
Twists 2 ini-len 200 ini-rmsd 3.42 opt-equ 206 opt-rmsd 2.88 chain-rmsd 6.52 Score 502.09 align-len 224 gaps 18 (8.04%)
P-value 6.59e-11 Afp-num 14705 Identity 30.80% Similarity 48.21%
Block  0 afp 12 score 251.09 rmsd  3.32 gap 3 (0.03%)
Block  1 afp  6 score 120.15 rmsd  3.63 gap 9 (0.16%)
Block  2 afp  7 score 147.80 rmsd  1.85 gap 3 (0.05%)

Chain 1:    1 MRIILLGAPGAGKGTQAQFIMEKYGIPQISTGDMLRAAVKSGSELGKQAKDIMDAGKLVTDELVIALVKE
              1111111111111111111111111111111111111111111111111111111  11111111111111
Chain 2:    1 MNILIFGPNGSGKGTQGNLVKDKYSLAHIESGGIFREHIGGGTELGKKAKEFIDRGDLVPDDITIPMVLE

Chain 1:   71 RIAQEDCRNGFLLDGFPRTIPQADAMKEAGINVD----YVLEFDVPDELIVDRIVGRRVHA-PSGRVYHV
              1111 11111111111111111111111111111      2222222222222222222222 22222222
Chain 2:   71 TLES-KGKDGWLLDGFPRNTVQAQKLFEALQEKGMKINFVIEILLPREVAKNRIMGRRICKNNPNHPNNI

Chain 1:  136 KFN-PPKVEGKDDVTGEELTTRKDD-QEETVRKRLVEYHQ---MTAPLIGYYSKEAEAGNTKYAKVDGTK
              222 22222222222222222222   33333333333333   3333333333333333333333333333
Chain 2:  140 FIDAIKPNGDVCRVCGGALSARADDQDEGAINKRHDIYYNTVDGTLAAAYYYKNMAAKEGFVYIELDGEG

Chain 1:  201 PVAEVRADLEKILG
              33333333333333
Chain 2:  210 SIDSIKDTLLAQLA
```

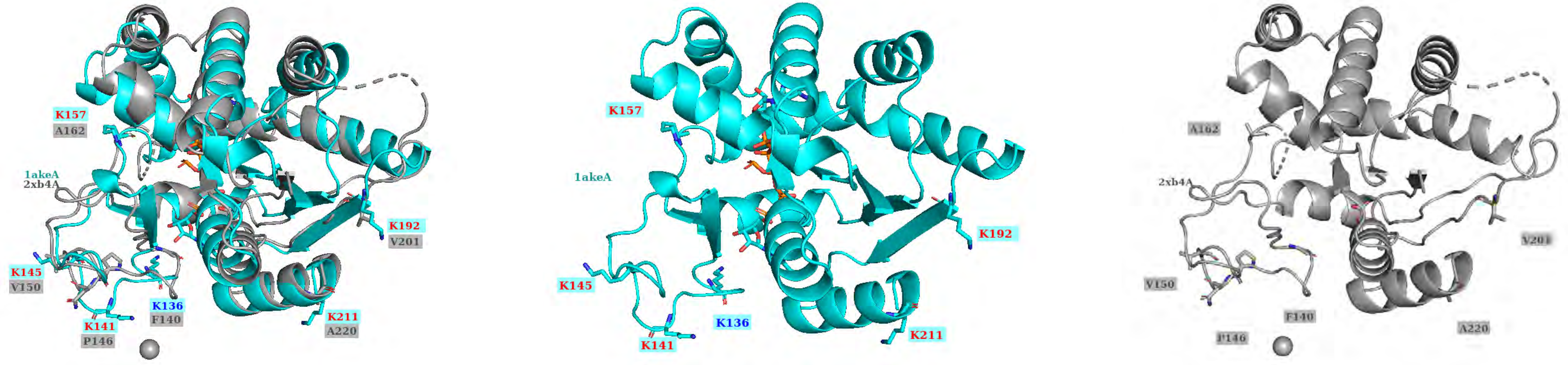

UniProt ID: C7U112

PDB ID: 3L0P_A

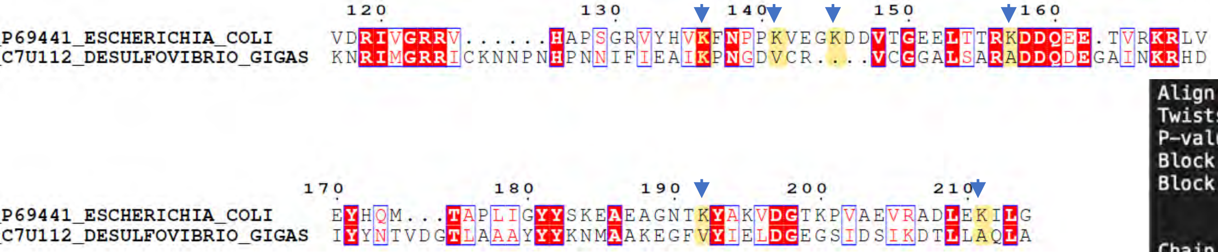


Full sequences in supplemental file.

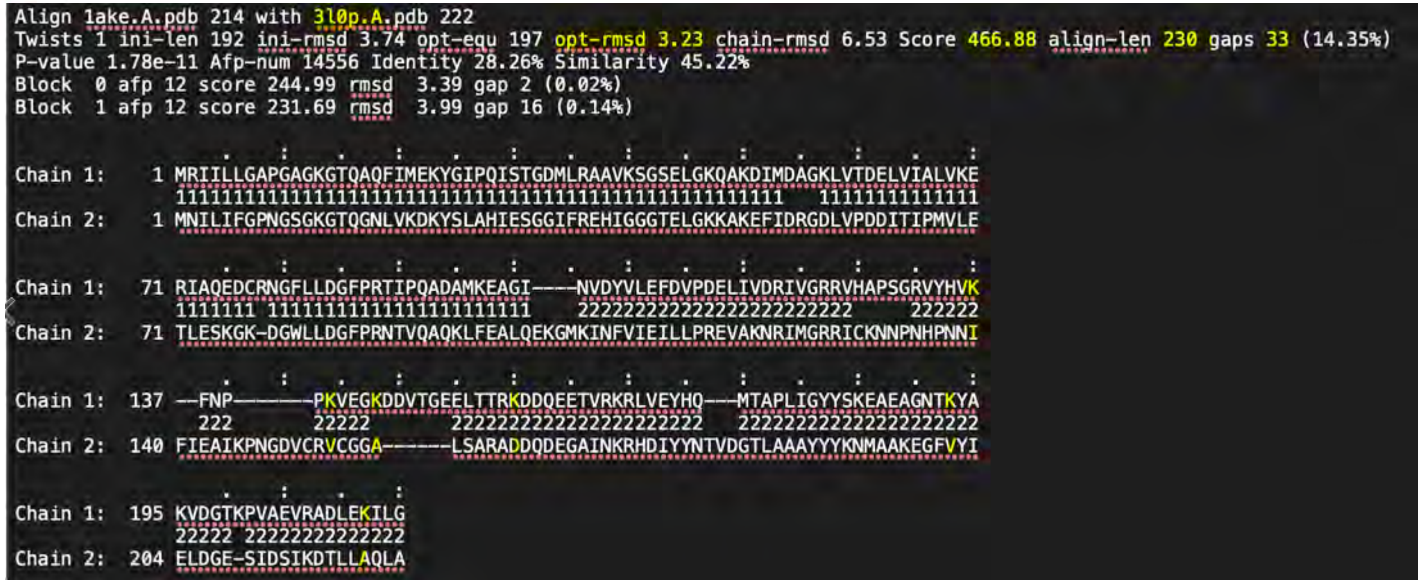

```
Align 1ake.A.pdb 214 with 3l0p.A.pdb 222
Twists 1 ini-len 192 ini-rmsd 3.74 opt-equ 197 opt-rmsd 3.23 chain-rmsd 6.53 Score 466.88 align-len 230 gaps 33 (14.35%)
P-value 1.78e-11 Afp-num 14556 Identity 28.26% Similarity 45.22%
Block  0 afp 12 score 244.99 rmsd  3.39 gap 2 (0.02%)
Block  1 afp 12 score 231.69 rmsd  3.99 gap 16 (0.14%)

                .    :    .    :    .    :    .    :    .    :    .    :    .    :
Chain 1:    1 MRIILLGAPGAGKGTQAQFIMEKYGIPQISTGDMLRAAVKSGSELGKQAKDIMDAGKLVTDELVIALVKE
              11111111111111111111111111111111111111111111111111111   11111111111111
Chain 2:    1 MNILIFGPNGSGKGTQGNLVKDKYSLAHIESGGIFREHIGGGTELGKKAKEFIDRGDLVPDDITIPMVLE

                .    :    .    :    .    :    .    :    .    :    .    :    .    :
Chain 1:   71 RIAQEDCRNGFLLDGFPRTIPQADAMKEAGI----NVDYVLEFDVPDELIVDRIVGRRVHAPSGRVYHVK
              1111111 111111111111111111111111    2222222222222222222222222     222222
Chain 2:   71 TLESKGK-DGWLLDGFPRNTVQAQKLFEALQEKGMKINFVIEILLPREVAKNRIMGRRICKNNPNHPNNI

                .    :    .    :    .    :    .    :    .    :    .    :    .    :
Chain 1:  137 --FNP-------PKVEGKDDVTGEELTTRKDDQEETVRKRLVEYHQ---MTAPLIGYYSKEAEAGNTKYA
                222       22222       2222222222222222222222   2222222222222222222222
Chain 2:  140 FIEAIKPNGDVCRVCGGA------LSARADDQDEGAINKRHDIYYNTVDGTLAAAYYYKNMAAKEGFVYI

                .    :    .    :
Chain 1:  195 KVDGTKPVAEVRADLEKILG
              22222 222222222222222
Chain 2:  204 ELDGE-SIDSIKDTLLAQLA
```

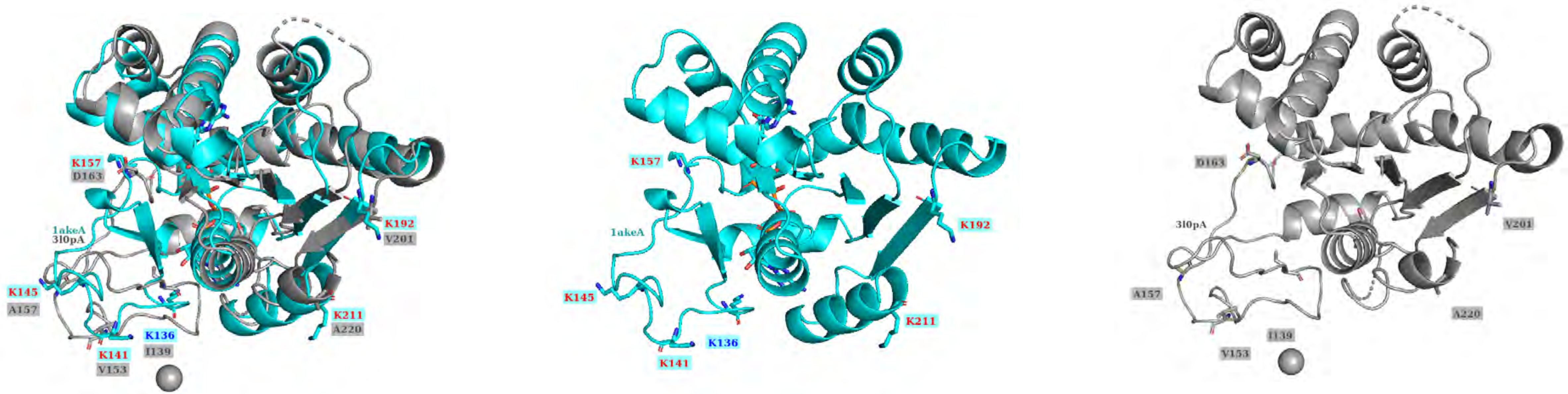

UniProt ID: C7U112

PDB ID: 3L0S_C

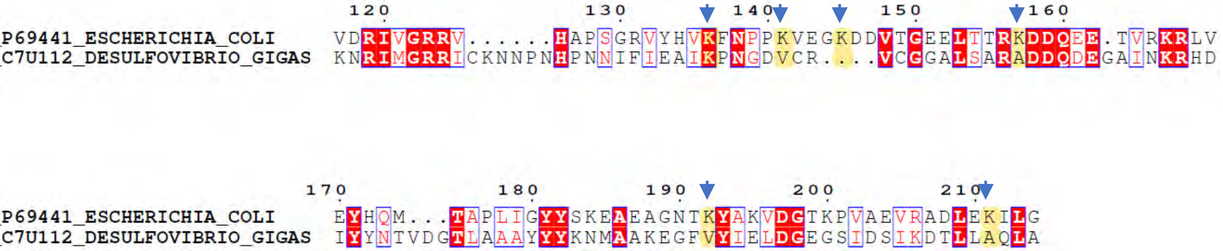


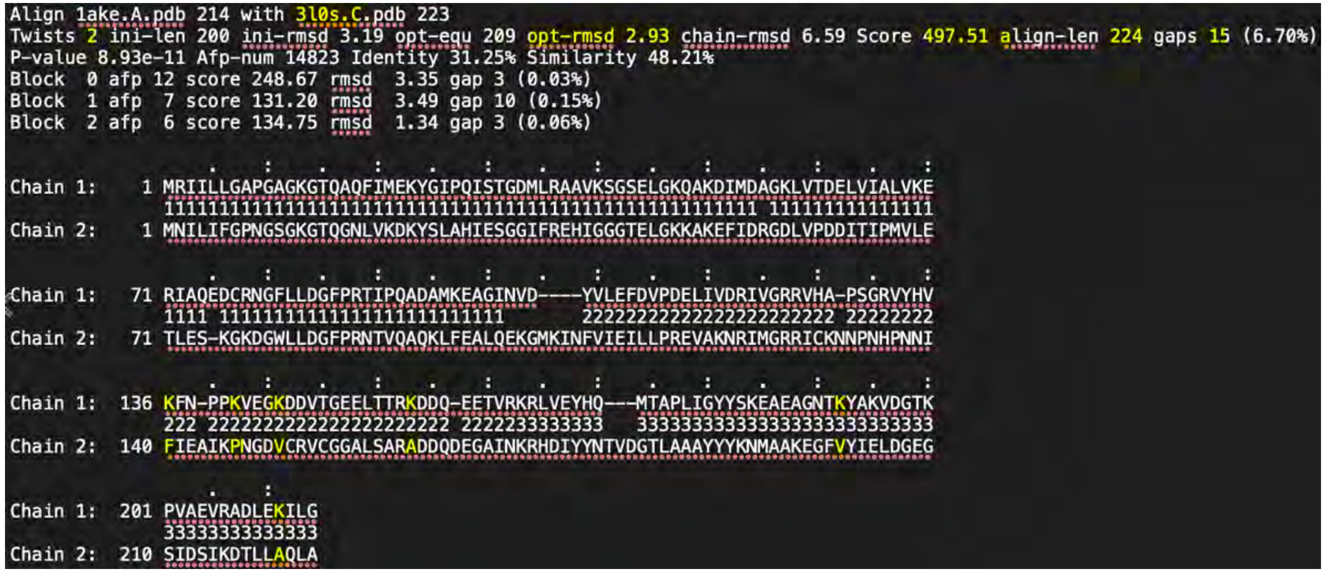

```
Align 1ake.A.pdb 214 with 3l0s.C.pdb 223
Twists 2 ini-len 200 ini-rmsd 3.19 opt-equ 209 opt-rmsd 2.93 chain-rmsd 6.59 Score 497.51 align-len 224 gaps 15 (6.70%)
P-value 8.93e-11 Afp-num 14823 Identity 31.25% Similarity 48.21%
Block  0 afp 12 score 248.67 rmsd  3.35 gap 3 (0.03%)
Block  1 afp  7 score 131.20 rmsd  3.49 gap 10 (0.15%)
Block  2 afp  6 score 134.75 rmsd  1.34 gap 3 (0.06%)

                  .    :    .    :    .    :    .    :    .    :    .    :    .    :
Chain 1:    1 MRIILLGAPGAGKGTQAQFIMEKYGIPQISTGDMLRAAVKSGSELGKQAKDIMDAGKLVTDELVIALVKE
              1111111111111111111111111111111111111111111111111111111 111111111111111
Chain 2:    1 MNILIFGPNGSGKGTQGNLVKDKYSLAHIESGGIFREHIGGGTELGKKAKEFIDRGDLVPDDITIPMVLE

                  .    :    .    :    .    :    .    :    .    :    .    :    .    :
Chain 1:   71 RIAQEDCRNGFLLDGFPRTIPQADAMKEAGINVD----YVLEFDVPDELIVDRIVGRRVHA-PSGRVYHV
              1111 1111111111111111111111111111      22222222222222222222222 22222222
Chain 2:   71 TLES-KGKDGWLLDGFPRNTVQAQKLFEALQEKGMKINFVIEILLPREVAKNRIMGRRICKNNPNHPNNI

                  .    :    .    :    .    :    .    :    .    :    .    :    .    :
Chain 1:  136 KFN-PPKVEGKDDVTGEELTTRKDDQ-EETVRKRLVEYHQ---MTAPLIGYYSKEAEAGNTKYAKVDGTK
              222 2222222222222222222222 2222233333333   33333333333333333333333333
Chain 2:  140 FIEAIKPNGDVCRVCGGALSARADDQDEGAINKRHDIYYNTVDGTLAAAYYYKNMAAKEGFVYIELDGEG

                  .    :
Chain 1:  201 PVAEVRADLEKILG
              33333333333333
Chain 2:  210 SIDSIKDTLLAQLA
```

Full sequences in supplemental file.

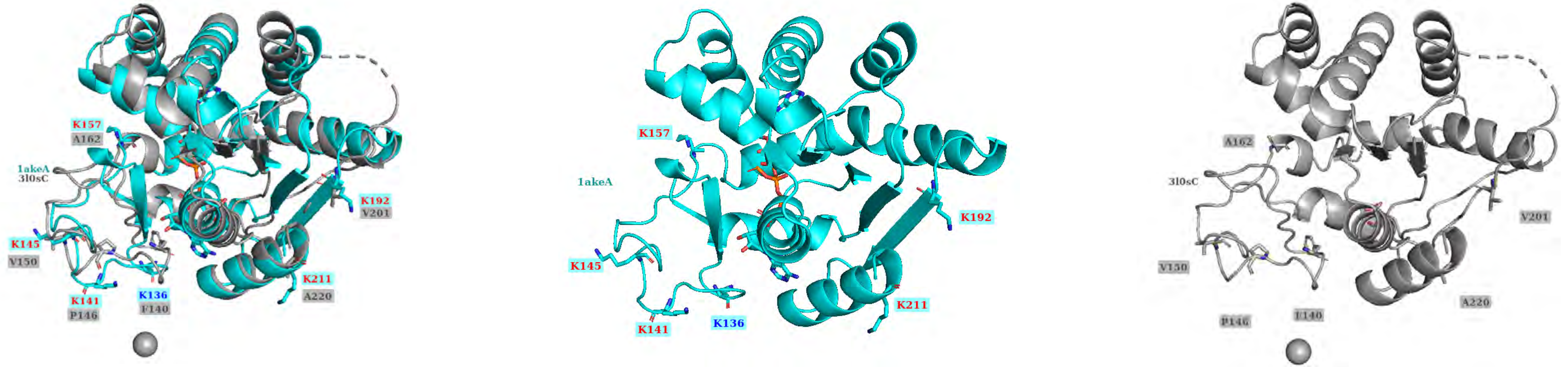

UniProt ID: G4V9S0

PDB ID: 3UMF_A

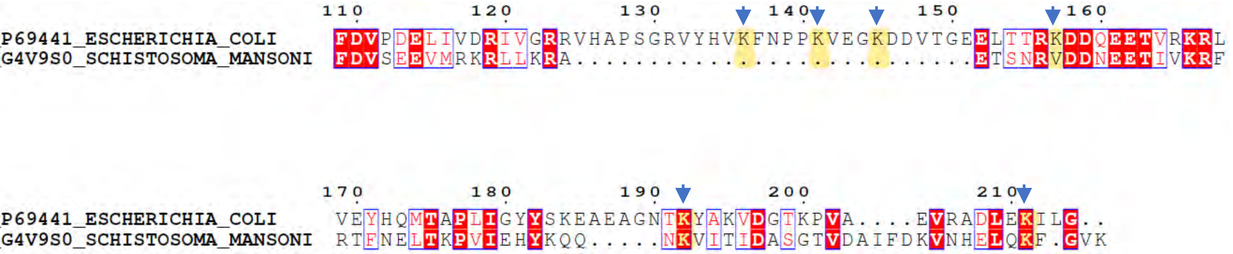


Full sequences in supplemental file.

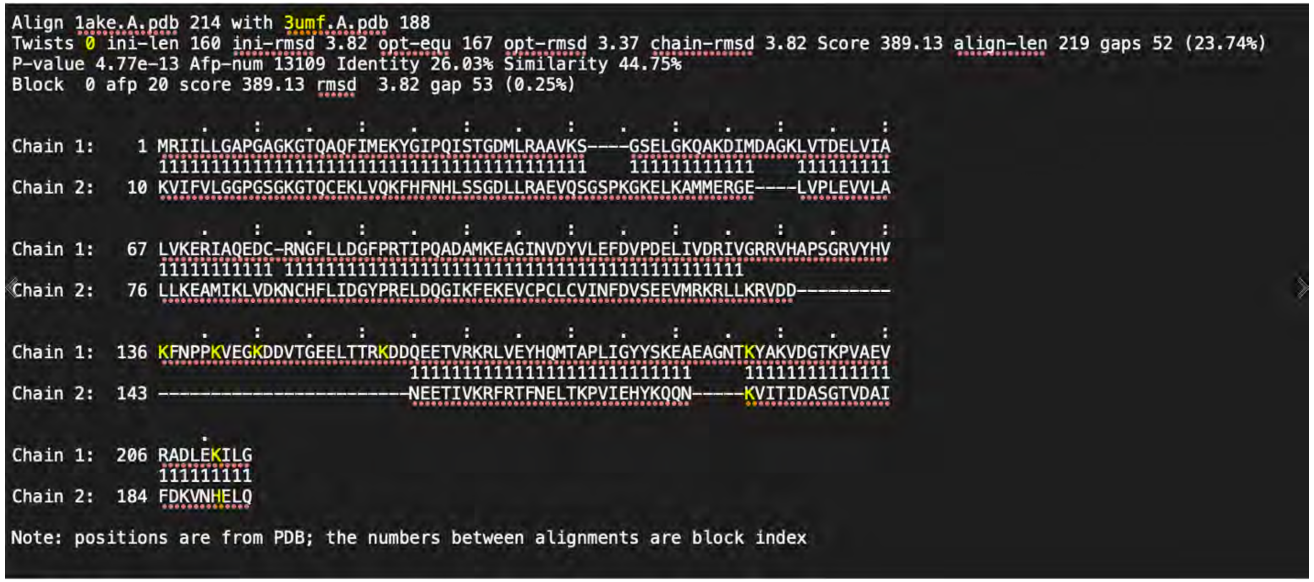

```
Align 1ake.A.pdb 214 with 3umf.A.pdb 188
Twists 0 ini-len 160 ini-rmsd 3.82 opt-equ 167 opt-rmsd 3.37 chain-rmsd 3.82 Score 389.13 align-len 219 gaps 52 (23.74%)
P-value 4.77e-13 Afp-num 13109 Identity 26.03% Similarity 44.75%
Block  0 afp 20 score 389.13 rmsd  3.82 gap 53 (0.25%)

                  .    :    .    :    .    :    .    :    .    :    .    :    .    :
Chain 1:    1 MRIILLGAPGAGKGTQAQFIMEKYGIPQISTGDMLRAAVKS----GSELGKQAKDIMDAGKLVTDELVIA
              11111111111111111111111111111111111111111    111111111111    111111111
Chain 2:   10 KVIFVLGGPGSGKGTQCEKLVQKFHFNHLSSGDLLRAEVQSGSPKGKELKAMMERGE----LVPLEVVLA

                  .    :    .    :    .    :    .    :    .    :    .    :    .    :
Chain 1:   67 LVKERIAQEDC-RNGFLLDGFPRTIPQADAMKEAGINVDYVLEFDVPDELIVDRIVGRRVHAPSGRVYHV
              11111111111 111111111111111111111111111111111111111111111111
Chain 2:   76 LLKEAMIKLVDKNCHFLIDGYPRELDQGIKFEKEVCPCLCVINFDVSEEVMRKRLLKRVDD----------

                  .    :    .    :    .    :    .    :    .    :    .    :    .    :
Chain 1:  136 KFNPPKVEGKDDVTGEELTTRKDDQEETVRKRLVEYHQMTAPLIGYYSKEAEAGNTKYAKVDGTKPVAEV
                                      111111111111111111111111111     11111111111111
Chain 2:  143 ------------------------NEETIVKRFRTFNELTKPVIEHYKQQN-----KVITIDASGTVDAI

                  .
Chain 1:  206 RADLEKILG
              111111111
Chain 2:  184 FDKVNHELQ

Note: positions are from PDB; the numbers between alignments are block index
```

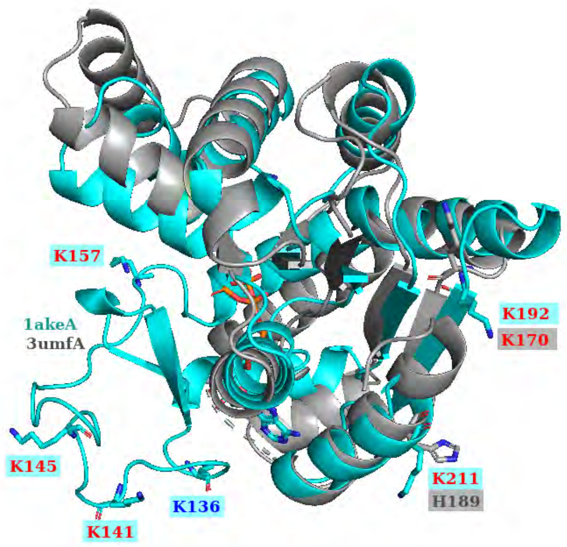


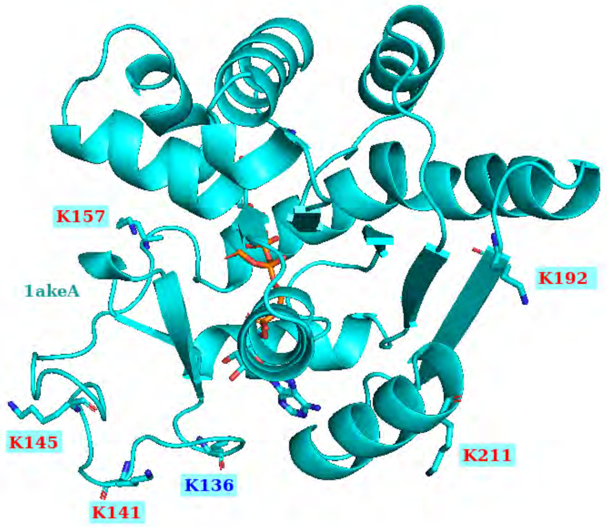


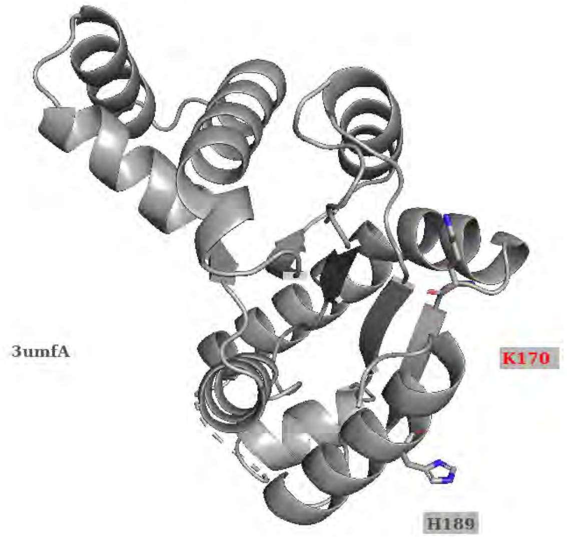

UniProt ID: M4AA20

PDB ID: 5YCF_B

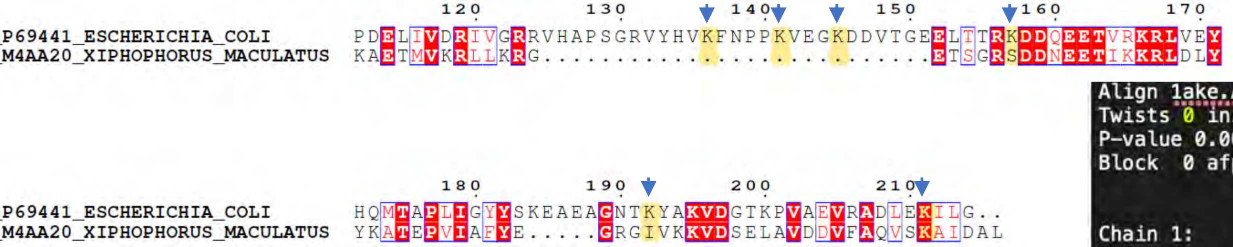


Full sequences in supplemental file.

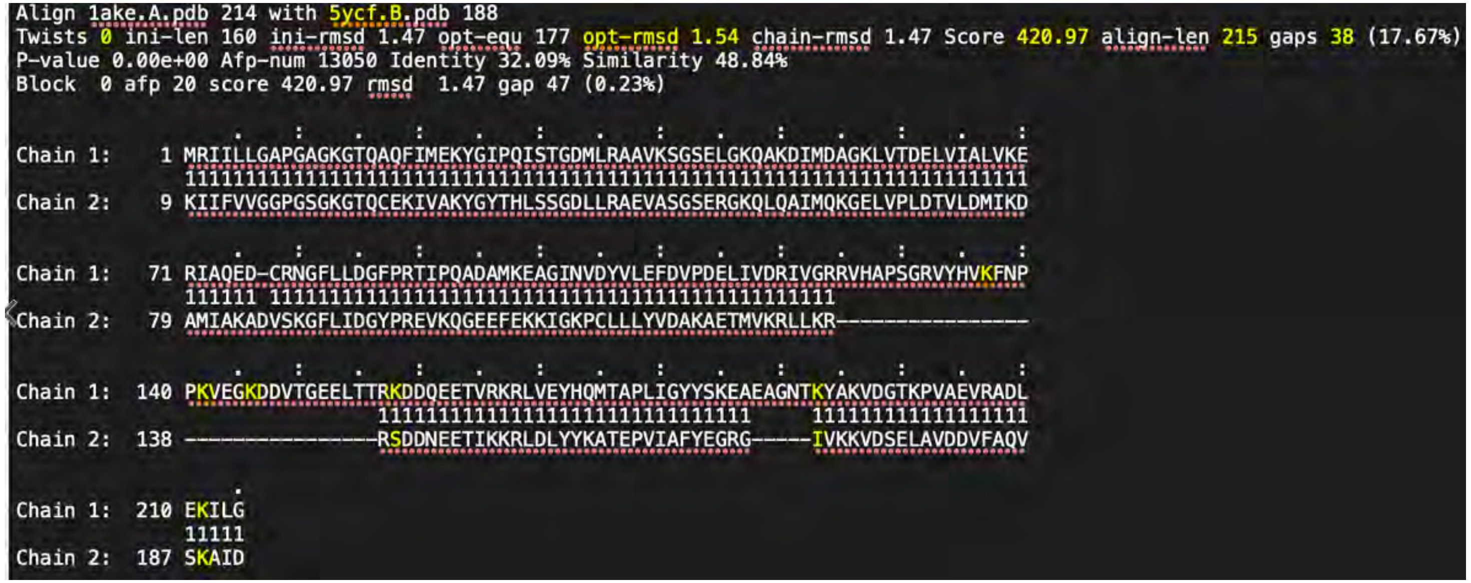

```
Align 1ake.A.pdb 214 with 5ycf.B.pdb 188
Twists 0 ini-len 160 ini-rmsd 1.47 opt-equ 177 opt-rmsd 1.54 chain-rmsd 1.47 Score 420.97 align-len 215 gaps 38 (17.67%)
P-value 0.00e+00 Afp-num 13050 Identity 32.09% Similarity 48.84%
Block  0 afp 20 score 420.97 rmsd  1.47 gap 47 (0.23%)

                  .    :    .    :    .    :    .    :    .    :    .    :    .    :
Chain 1:    1 MRIILLGAPGAGKGTQAQFIMEKYGIPQISTGDMLRAAVKSGSELGKQAKDIMDAGKLVTDELVIALVKE
              1111111111111111111111111111111111111111111111111111111111111111111111
Chain 2:    9 KIIFVVGGPGSGKGTQCEKIVAKYGYTHLSSGDLLRAEVASGSERGKQLQAIMQKGELVPLDTVLDMIKD

                  .    :    .    :    .    :    .    :    .    :    .    :    .    :
Chain 1:   71 RIAQED-CRNGFLLDGFPRTIPQADAMKEAGINVDYVLEFDVPDELIVDRIVGRRVHAPSGRVYHVKFNP
              111111 11111111111111111111111111111111111111111111111
Chain 2:   79 AMIAKADVSKGFLIDGYPREVKQGEEFEKKIGKPCLLLYVDAKAETMVKRLLKR---------------

                  .    :    .    :    .    :    .    :    .    :    .    :    .    :
Chain 1:  140 PKVEGKDDVTGEELTTRKDDQEETVRKRLVEYHQMTAPLIGYYSKEAEAGNTKYAKVDGTKPVAEVRADL
                              1111111111111111111111111111111     111111111111111111
Chain 2:  138 ----------------RSDDNEETIKKRLDLYYKATEPVIAFYEGRG-----IVKKVDSELAVDDVFAQV

                  .
Chain 1:  210 EKILG
              11111
Chain 2:  187 SKAID
```

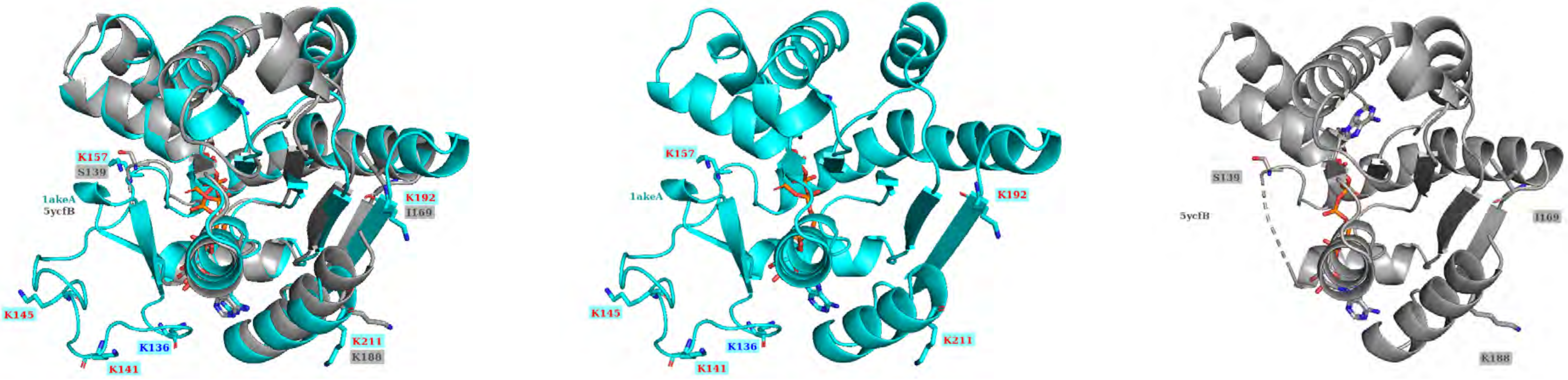

UniProt ID: none

PDB ID: 4JZK_A

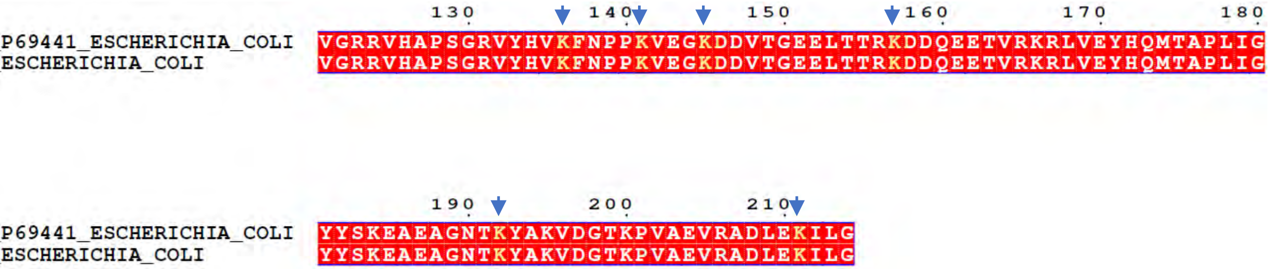


Full sequences in supplemental file.

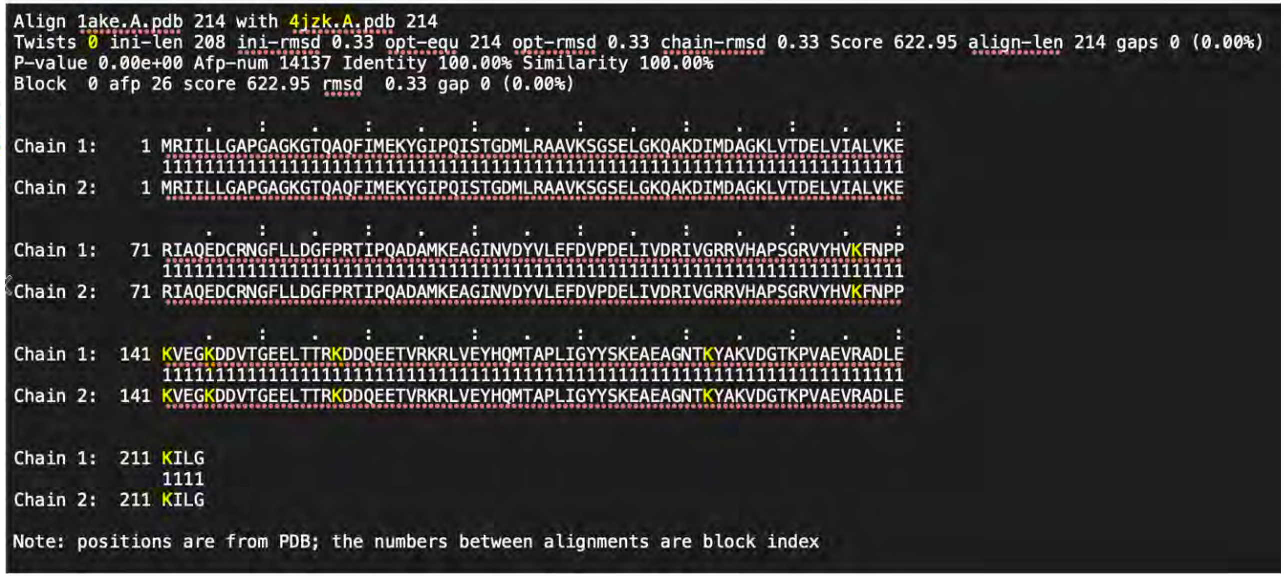

```
Align 1ake.A.pdb 214 with 4jzk.A.pdb 214
Twists 0 ini-len 208 ini-rmsd 0.33 opt-equ 214 opt-rmsd 0.33 chain-rmsd 0.33 Score 622.95 align-len 214 gaps 0 (0.00%)
P-value 0.00e+00 Afp-num 14137 Identity 100.00% Similarity 100.00%
Block  0 afp 26 score 622.95 rmsd  0.33 gap 0 (0.00%)

Chain 1:    1 MRIILLGAPGAGKGTQAQFIMEKYGIPQISTGDMLRAAVKSGSELGKQAKDIMDAGKLVTDELVIALVKE
              1111111111111111111111111111111111111111111111111111111111111111111111
Chain 2:    1 MRIILLGAPGAGKGTQAQFIMEKYGIPQISTGDMLRAAVKSGSELGKQAKDIMDAGKLVTDELVIALVKE

Chain 1:   71 RIAQEDCRNGFLLDGFPRTIPQADAMKEAGINVDYVLEFDVPDELIVDRIVGRRVHAPSGRVYHVKFNPP
              1111111111111111111111111111111111111111111111111111111111111111111111
Chain 2:   71 RIAQEDCRNGFLLDGFPRTIPQADAMKEAGINVDYVLEFDVPDELIVDRIVGRRVHAPSGRVYHVKFNPP

Chain 1:  141 KVEGKDDVTGEELTTRKDDQEETVRKRLVEYHQMTAPLIGYYSKEAEAGNTKYAKVDGTKPVAEVRADLE
              1111111111111111111111111111111111111111111111111111111111111111111111
Chain 2:  141 KVEGKDDVTGEELTTRKDDQEETVRKRLVEYHQMTAPLIGYYSKEAEAGNTKYAKVDGTKPVAEVRADLE

Chain 1:  211 KILG
              1111
Chain 2:  211 KILG

Note: positions are from PDB; the numbers between alignments are block index
```

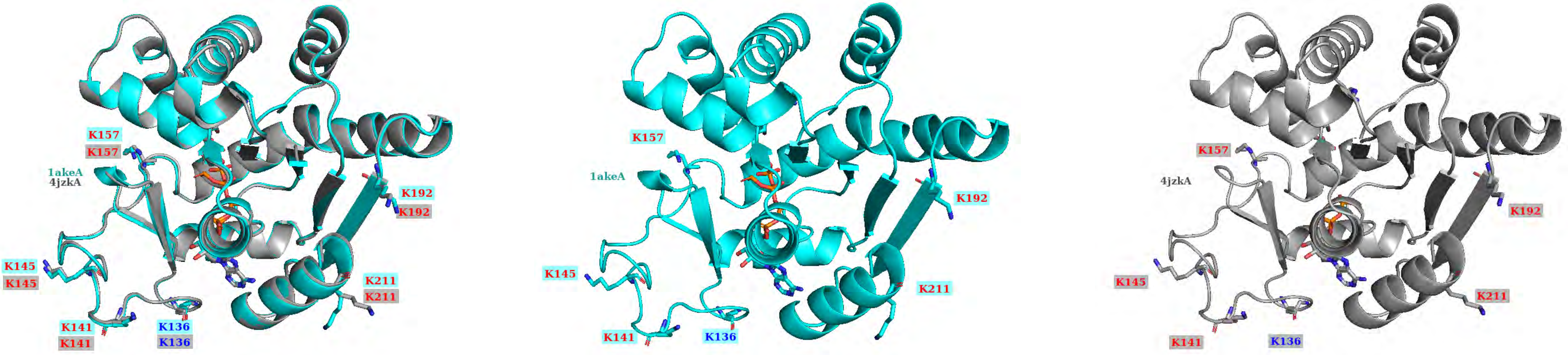

UniProt ID: O66490

PDB ID: 2RGX_A

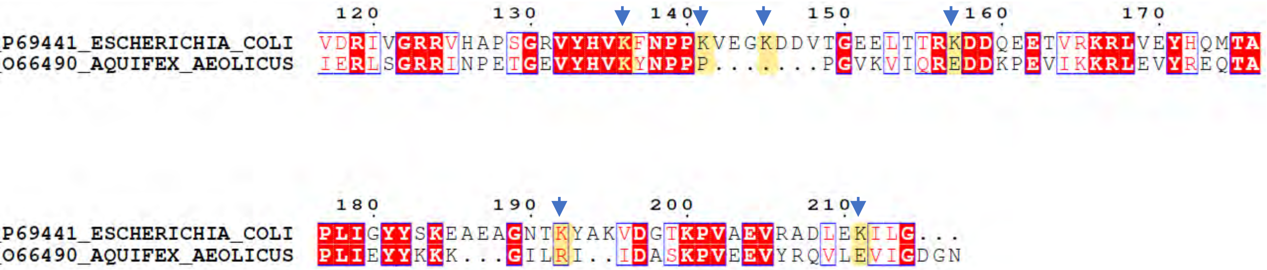


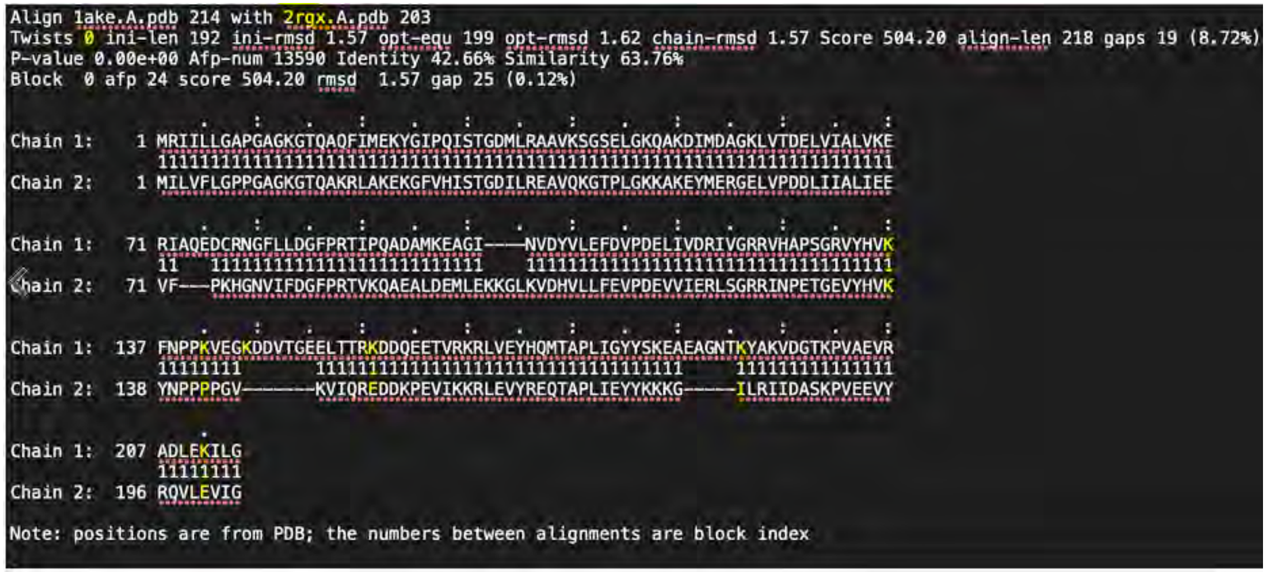

```
Align 1ake.A.pdb 214 with 2rgx.A.pdb 203
Twists 0 ini-len 192 ini-rmsd 1.57 opt-equ 199 opt-rmsd 1.62 chain-rmsd 1.57 Score 504.20 align-len 218 gaps 19 (8.72%)
P-value 0.00e+00 Afp-num 13590 Identity 42.66% Similarity 63.76%
Block  0 afp 24 score 504.20 rmsd  1.57 gap 25 (0.12%)

Chain 1:    1 MRIILLGAPGAGKGTQAQFIMEKYGIPQISTGDMLRAAVKSGSELGKQAKDIMDAGKLVTDELVIALVKE
              1111111111111111111111111111111111111111111111111111111111111111111111
Chain 2:    1 MILVFLGPPGAGKGTQAKRLAKEKGFVHISTGDILREAVQKGTPLGKKAKEYMERGELVPDDLIIALIEE

Chain 1:   71 RIAQEDCRNGFLLDGFPRTIPQADAMKEAGI----NVDYVLEFDVPDELIVDRIVGRRVHAPSGRVYHVK
              11   111111111111111111111111111    11111111111111111111111111111111111
Chain 2:   71 VF---PKHGNVIFDGFPRTVKQAEALDEMLEKKGLKVDHVLLFEVPDEVVIERLSGRRINPETGEVYHVK

Chain 1:  137 FNPPKVEGKDDVTGEELTTRKDDQEETVRKRLVEYHQMTAPLIGYYSKEAEAGNTKYAKVDGTKPVAEVR
              11111111       111111111111111111111111111111111111111     111111111111111
Chain 2:  138 YNPPPPGV-------KVIQREDDKPEVIKKRLEVYREQTAPLIEYYKKKG-----ILRIIDASKPVEEVY

Chain 1:  207 ADLEKILG
              11111111
Chain 2:  196 RQVLEVIG

Note: positions are from PDB; the numbers between alignments are block index
```

Full sequences in supplemental file.

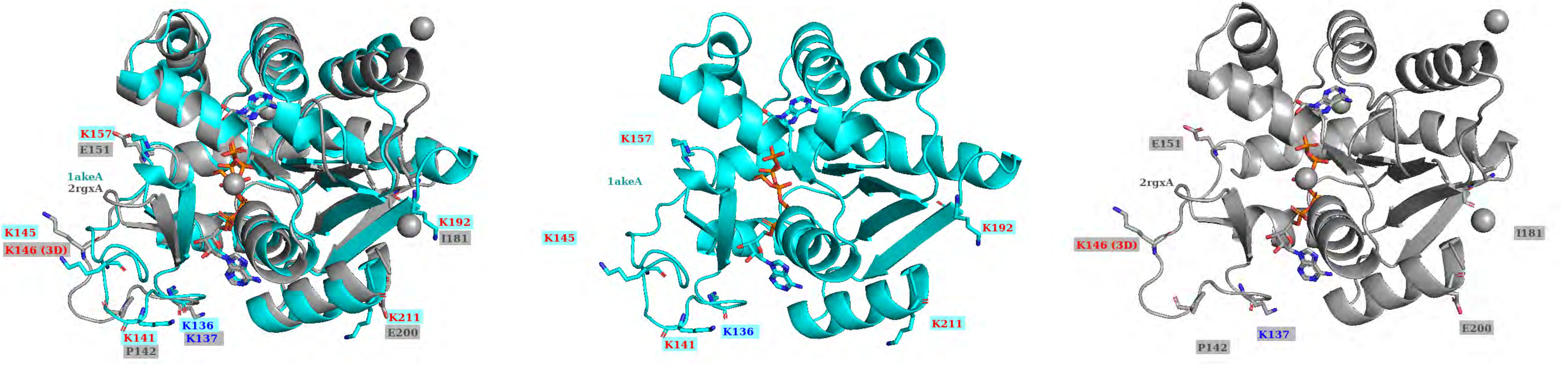

UniProt ID: O66490

PDB ID: 2RH5_B

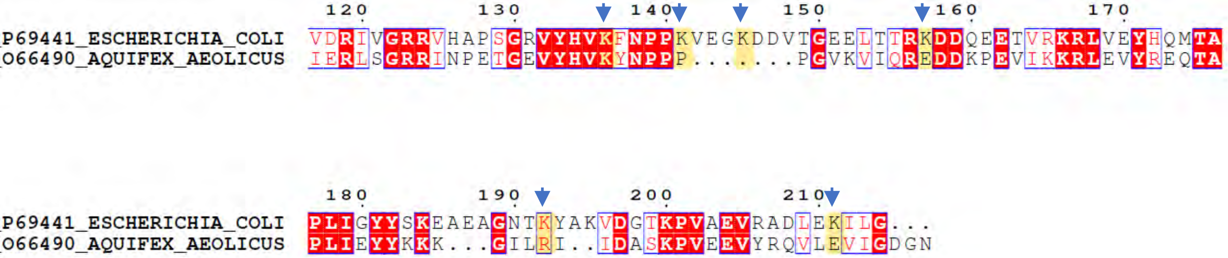


Full sequences in supplemental file.

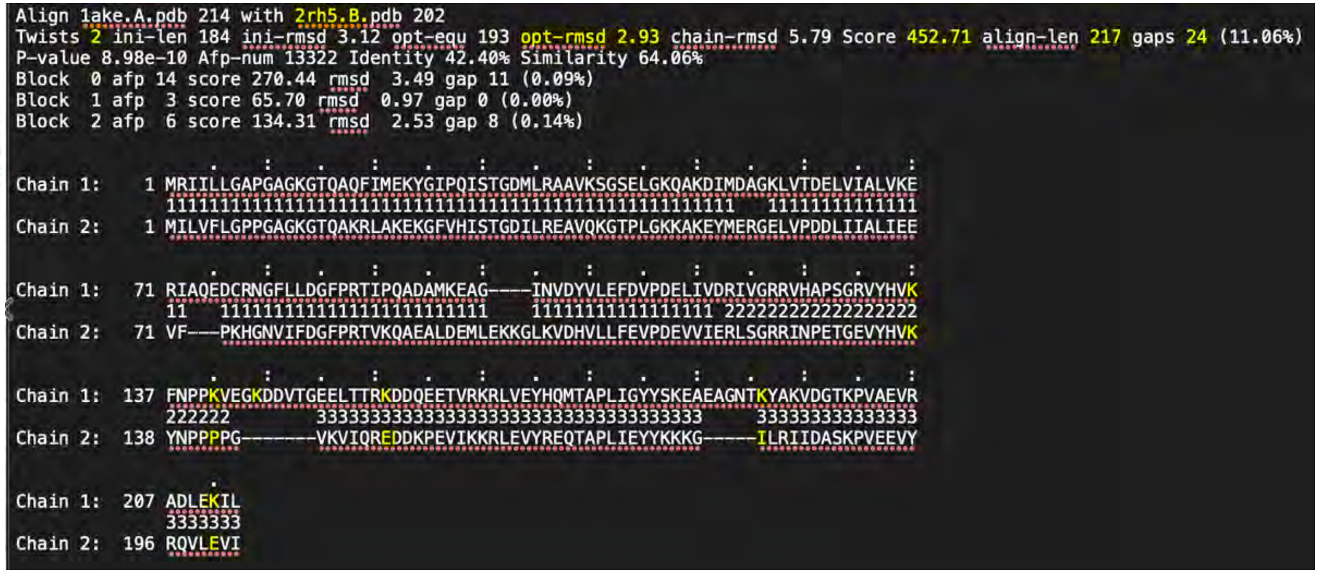


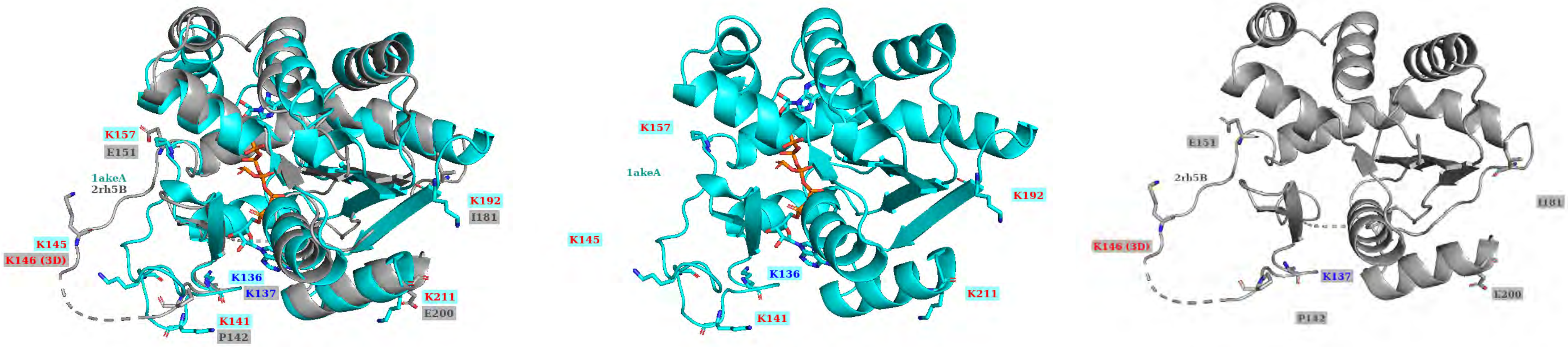

UniProt ID: O66490

PDB ID: 3SR0_A

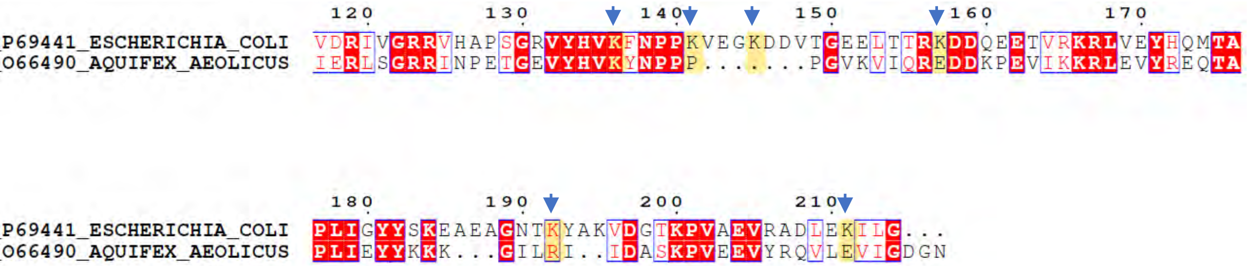


Full sequences in supplemental file.

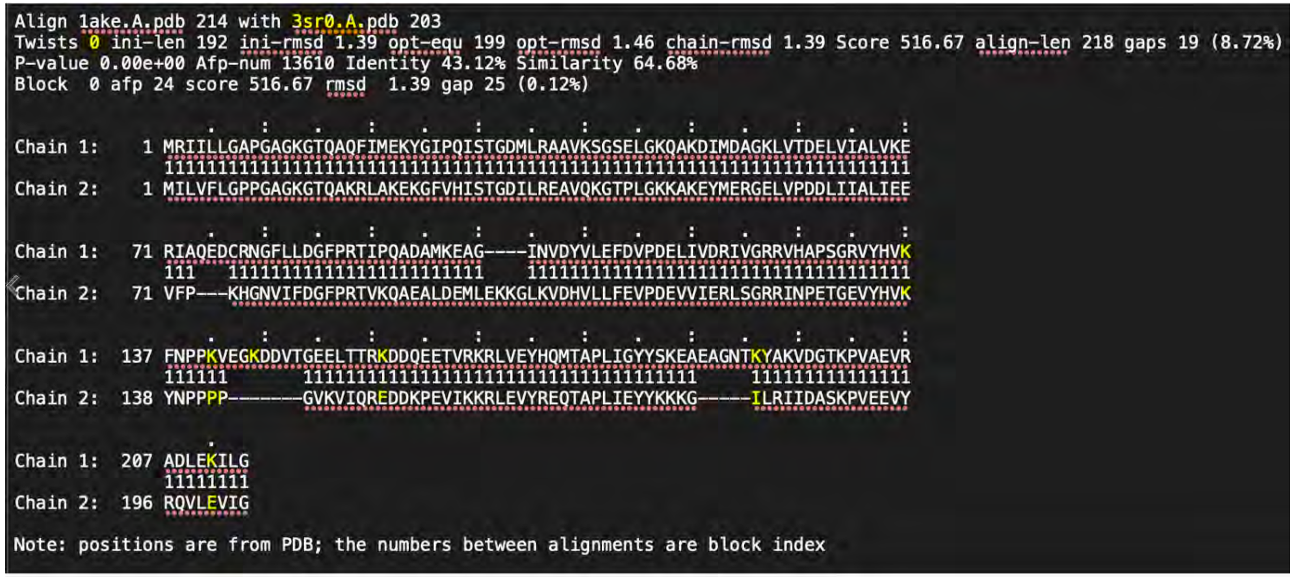

```
Align 1ake.A.pdb 214 with 3sr0.A.pdb 203
Twists 0 ini-len 192 ini-rmsd 1.39 opt-equ 199 opt-rmsd 1.46 chain-rmsd 1.39 Score 516.67 align-len 218 gaps 19 (8.72%)
P-value 0.00e+00 Afp-num 13610 Identity 43.12% Similarity 64.68%
Block  0 afp 24 score 516.67 rmsd  1.39 gap 25 (0.12%)

Chain 1:    1 MRIILLGAPGAGKGTQAQFIMEKYGIPQISTGDMLRAAVKSGSELGKQAKDIMDAGKLVTDELVIALVKE
              1111111111111111111111111111111111111111111111111111111111111111111111
Chain 2:    1 MILVFLGPPGAGKGTQAKRLAKEKGFVHISTGDILREAVQKGTPLGKKAKEYMERGELVPDDLIIALIEE

Chain 1:   71 RIAQEDCRNGFLLDGFPRTIPQADAMKEAG----INVDYVLEFDVPDELIVDRIVGRRVHAPSGRVYHVK
              111    1111111111111111111111111    1111111111111111111111111111111111111
Chain 2:   71 VFP---KHGNVIFDGFPRTVKQAEALDEMLEKKGLKVDHVLLFEVPDEVVIERLSGRRINPETGEVYHVK

Chain 1:  137 FNPPKVEGKDDVTGEELTTRKDDQEETVRKRLVEYHQMTAPLIGYYSKEAEAGNTKYAKVDGTKPVAEVR
              111111          1111111111111111111111111111111111111111       11111111111111
Chain 2:  138 YNPPPP-------GVKVIQREDDKPEVIKKRLEVYREQTAPLIEYYKKKG-----ILRIIDASKPVEEVY

Chain 1:  207 ADLEKILG
              11111111
Chain 2:  196 RQVLEVIG

Note: positions are from PDB; the numbers between alignments are block index
```

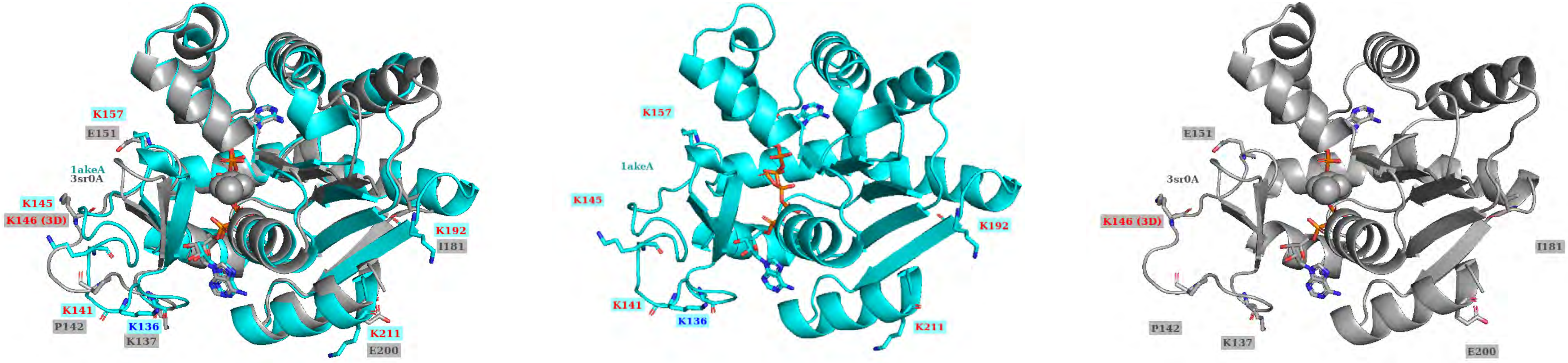

UniProt ID: O66490

PDB ID: 4CF7_B

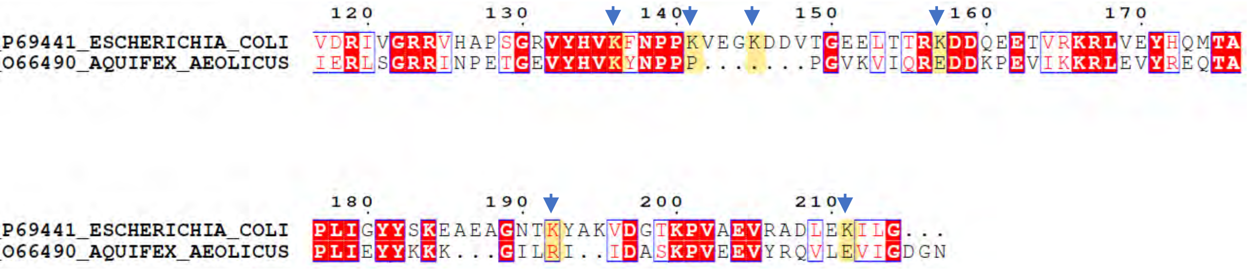


Full sequences in supplemental file.

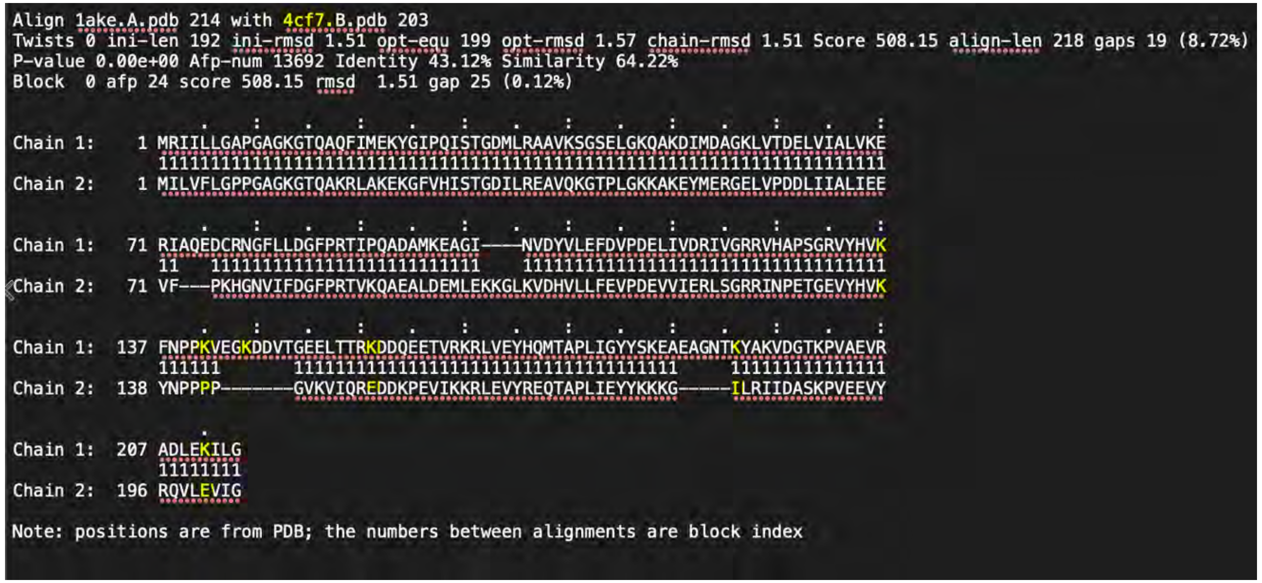

```
Align 1ake.A.pdb 214 with 4cf7.B.pdb 203
Twists 0 ini-len 192 ini-rmsd 1.51 opt-equ 199 opt-rmsd 1.57 chain-rmsd 1.51 Score 508.15 align-len 218 gaps 19 (8.72%)
P-value 0.00e+00 Afp-num 13692 Identity 43.12% Similarity 64.22%
Block  0 afp 24 score 508.15 rmsd  1.51 gap 25 (0.12%)

                .    :    .    :    .    :    .    :    .    :    .    :    .    :
Chain 1:    1 MRIILLGAPGAGKGTQAQFIMEKYGIPQISTGDMLRAAVKSGSELGKQAKDIMDAGKLVTDELVIALVKE
              1111111111111111111111111111111111111111111111111111111111111111111111
Chain 2:    1 MILVFLGPPGAGKGTQAKRLAKEKGFVHISTGDILREAVQKGTPLGKKAKEYMERGELVPDDLIIALIEE

                .    :    .    :    .    :    .    :    .    :    .    :    .    :
Chain 1:   71 RIAQEDCRNGFLLDGFPRTIPQADAMKEAGI----NVDYVLEFDVPDELIVDRIVGRRVHAPSGRVYHVK
              11   11111111111111111111111111    111111111111111111111111111111111111
Chain 2:   71 VF---PKHGNVIFDGFPRTVKQAEALDEMLEKKGLKVDHVLLFEVPDEVVIERLSGRRINPETGEVYHVK

                .    :    .    :    .    :    .    :    .    :    .    :    .    :
Chain 1:  137 FNPPKVEGKDDVTGEELTTRKDDQEETVRKRLVEYHQMTAPLIGYYSKEAEAGNTKYAKVDGTKPVAEVR
              111111       1111111111111111111111111111111111111     111111111111111
Chain 2:  138 YNPPPP-------GVKVIQREDDKPEVIKKRLEVYREQTAPLIEYYKKKG-----ILRIIDASKPVEEVY

                .
Chain 1:  207 ADLEKILG
              11111111
Chain 2:  196 RQVLEVIG

Note: positions are from PDB; the numbers between alignments are block index
```

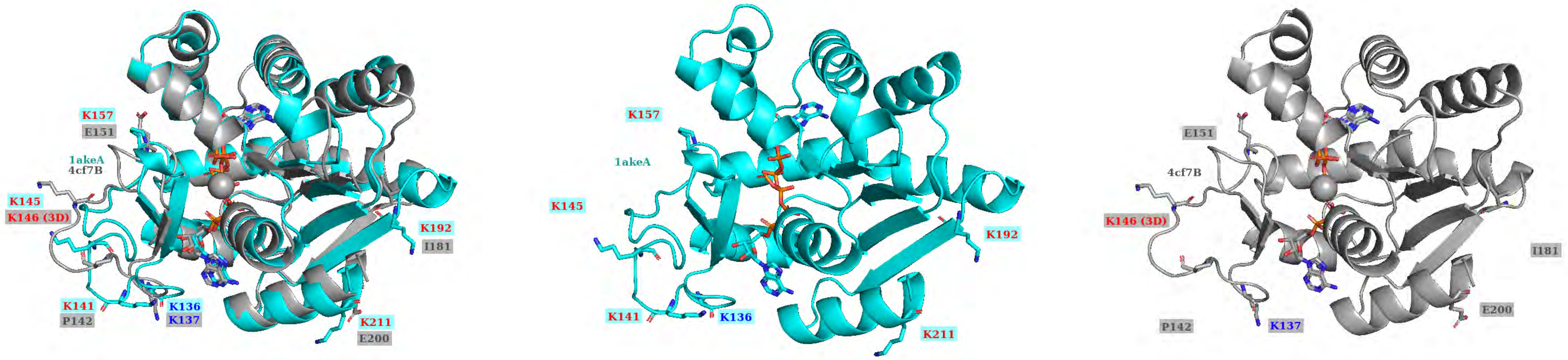

UniProt ID: O66490

PDB ID: 4IKE_A

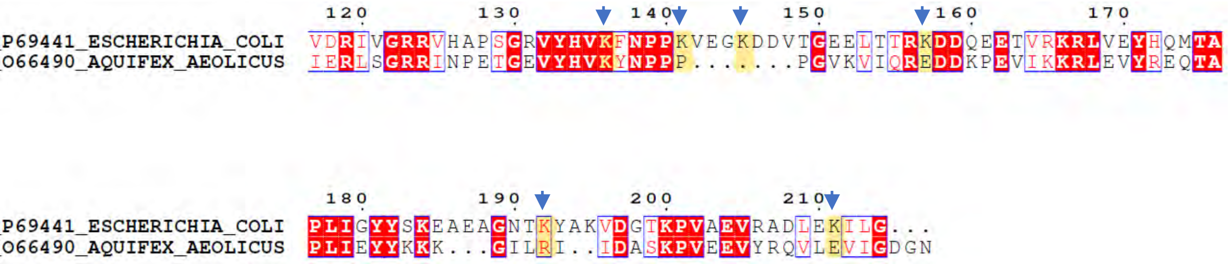


Full sequences in supplemental file.

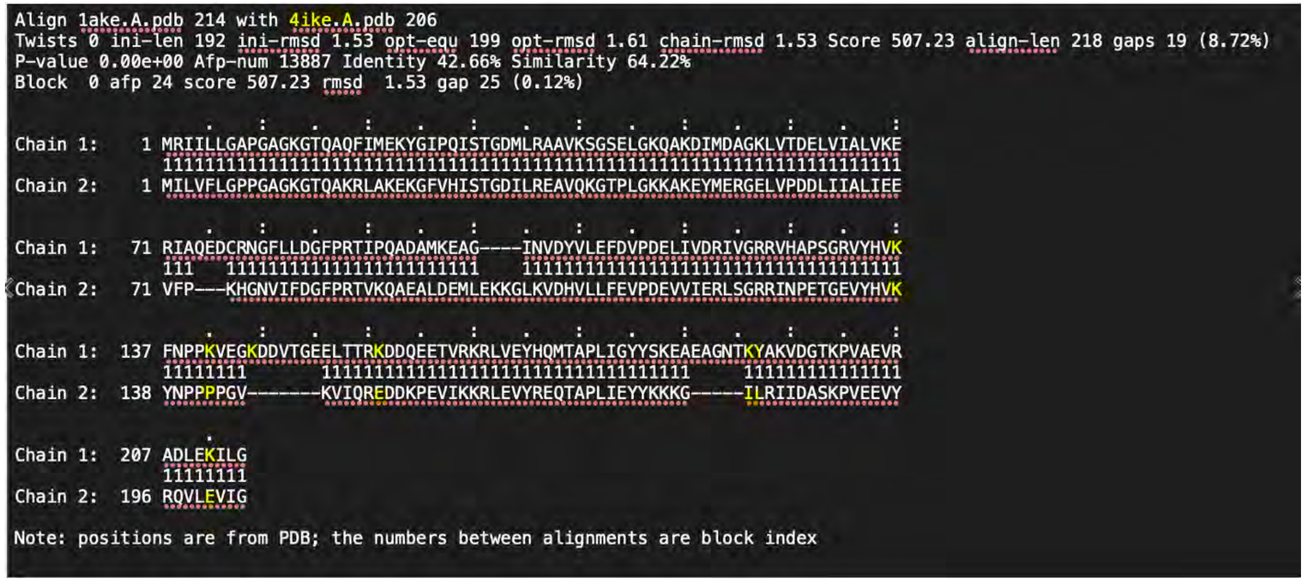

```
Align 1ake.A.pdb 214 with 4ike.A.pdb 206
Twists 0 ini-len 192 ini-rmsd 1.53 opt-equ 199 opt-rmsd 1.61 chain-rmsd 1.53 Score 507.23 align-len 218 gaps 19 (8.72%)
P-value 0.00e+00 Afp-num 13887 Identity 42.66% Similarity 64.22%
Block  0 afp 24 score 507.23 rmsd  1.53 gap 25 (0.12%)

                .    :    .    :    .    :    .    :    .    :    .    :    .    :
Chain 1:    1 MRIILLGAPGAGKGTQAQFIMEKYGIPQISTGDMLRAAVKSGSELGKQAKDIMDAGKLVTDELVIALVKE
              1111111111111111111111111111111111111111111111111111111111111111111111
Chain 2:    1 MILVFLGPPGAGKGTQAKRLAKEKGFVHISTGDILREAVQKGTPLGKKAKEYMERGELVPDDLIIALIEE

                .    :    .    :    .    :    .    :    .    :    .    :    .    :
Chain 1:   71 RIAQEDCRNGFLLDGFPRTIPQADAMKEAG----INVDYVLEFDVPDELIVDRIVGRRVHAPSGRVYHVK
              111   111111111111111111111111    111111111111111111111111111111111111
Chain 2:   71 VFP---KHGNVIFDGFPRTVKQAEALDEMLEKKGLKVDHVLLFEVPDEVVIERLSGRRINPETGEVYHVK

                .    :    .    :    .    :    .    :    .    :    .    :    .    :
Chain 1:  137 FNPPKVEGKDDVTGEELTTRKDDQEETVRKRLVEYHQMTAPLIGYYSKEAEAGNTKYAKVDGTKPVAEVR
              11111111       1111111111111111111111111111111111     111111111111111
Chain 2:  138 YNPPPPGV-------KVIQREDDKPEVIKKRLEVYREQTAPLIEYYKKKG-----ILRIIDASKPVEEVY

                .
Chain 1:  207 ADLEKILG
              11111111
Chain 2:  196 RQVLEVIG

Note: positions are from PDB; the numbers between alignments are block index
```

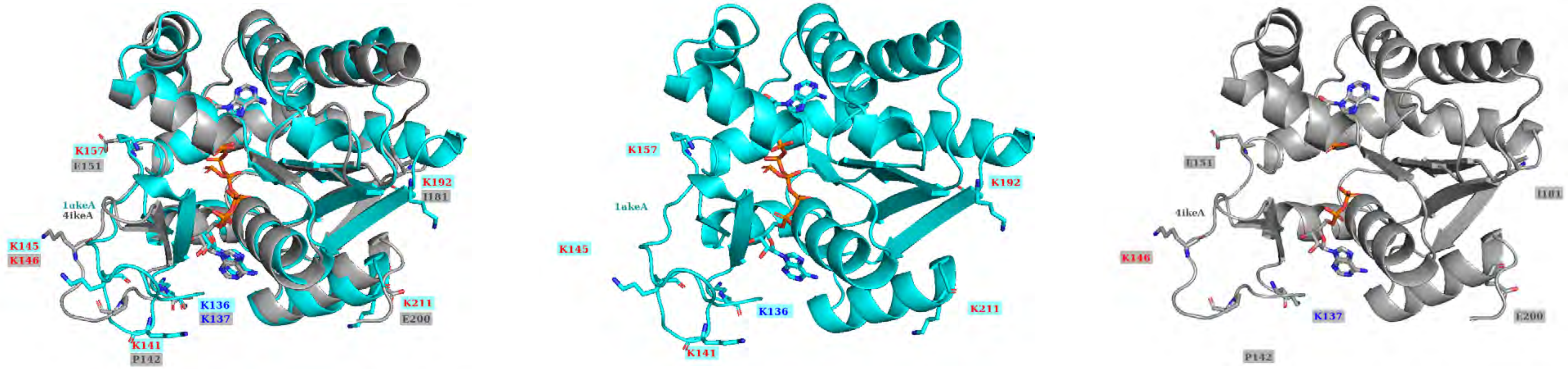

UniProt ID: O66490

PDB ID: 4JKY_A

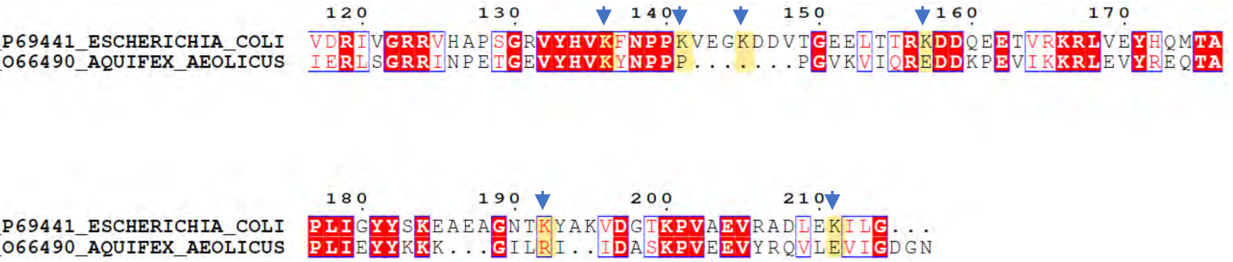


Full sequences in supplemental file.

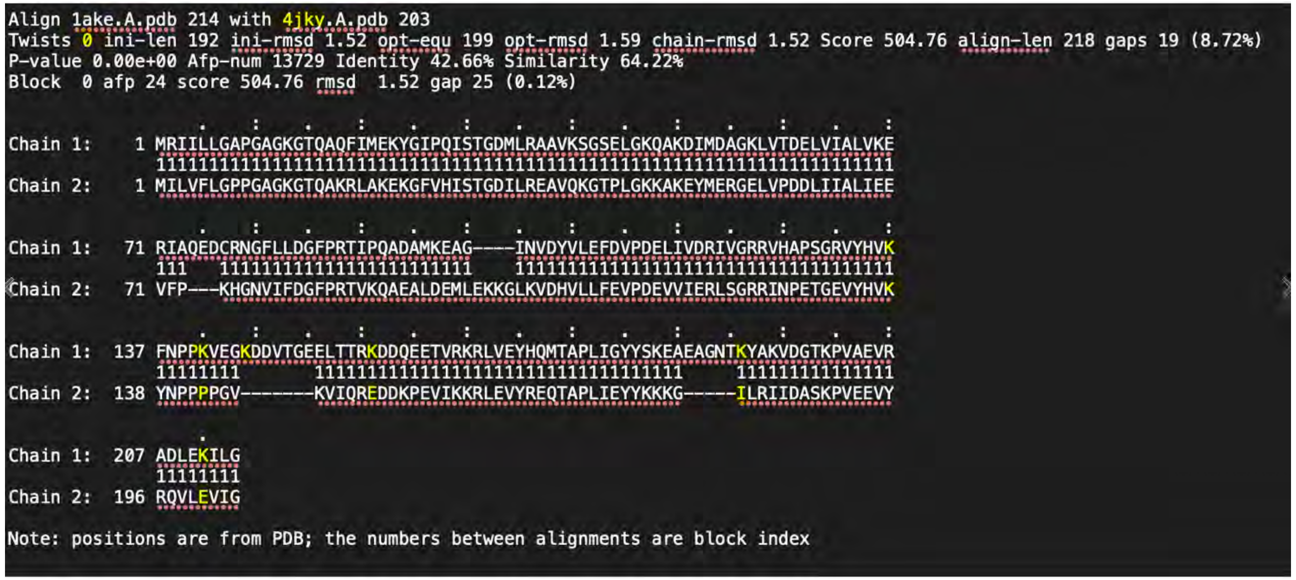

```
Align 1ake.A.pdb 214 with 4jky.A.pdb 203
Twists 0 ini-len 192 ini-rmsd 1.52 opt-equ 199 opt-rmsd 1.59 chain-rmsd 1.52 Score 504.76 align-len 218 gaps 19 (8.72%)
P-value 0.00e+00 Afp-num 13729 Identity 42.66% Similarity 64.22%
Block  0 afp 24 score 504.76 rmsd  1.52 gap 25 (0.12%)

Chain 1:    1 MRIILLGAPGAGKGTQAQFIMEKYGIPQISTGDMLRAAVKSGSELGKQAKDIMDAGKLVTDELVIALVKE
                1111111111111111111111111111111111111111111111111111111111111111111111
Chain 2:    1 MILVFLGPPGAGKGTQAKRLAKEKGFVHISTGDILREAVQKGTPLGKKAKEYMERGELVPDDLIIALIEE

Chain 1:   71 RIAQEDCRNGFLLDGFPRTIPQADAMKEAG----INVDYVLEFDVPDELIVDRIVGRRVHAPSGRVYHVK
              111   1111111111111111111111111     111111111111111111111111111111111111
Chain 2:   71 VFP---KHGNVIFDGFPRTVKQAEALDEMLEKKGLKVDHVLLFEVPDEVVIERLSGRRINPETGEVYHVK

Chain 1:  137 FNPPKVEGKDDVTGEELTTRKDDQEETVRKRLVEYHQMTAPLIGYYSKEAEAGNTKYAKVDGTKPVAEVR
              11111111          1111111111111111111111111111111111111        11111111111111
Chain 2:  138 YNPPPPGV-------KVIQREDDKPEVIKKRLEVYREQTAPLIEYYKKKG-----ILRIIDASKPVEEVY

Chain 1:  207 ADLEKILG
              11111111
Chain 2:  196 RQVLEVIG

Note: positions are from PDB; the numbers between alignments are block index
```

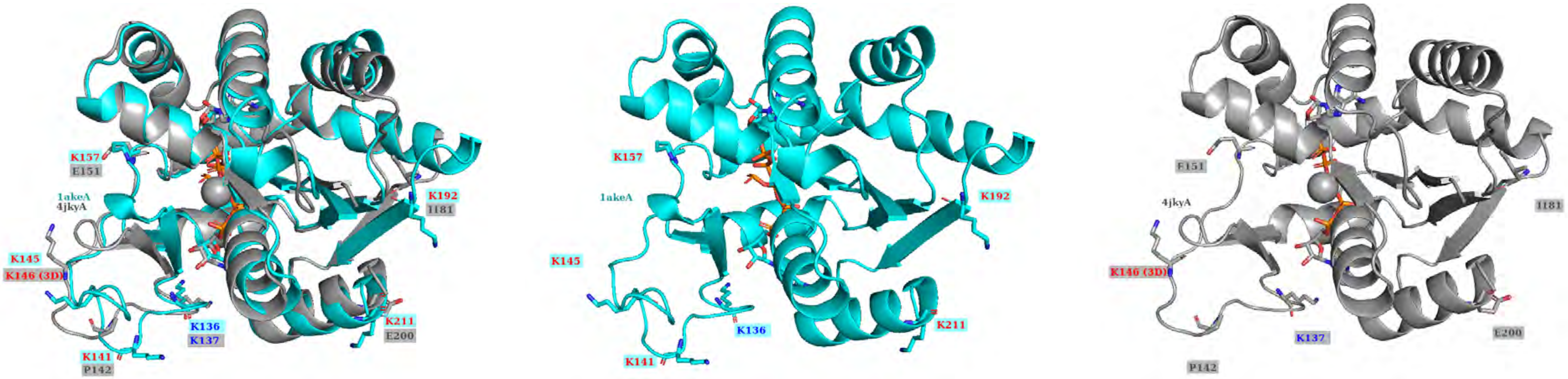

UniProt ID: O66490

PDB ID: 4JL5_A

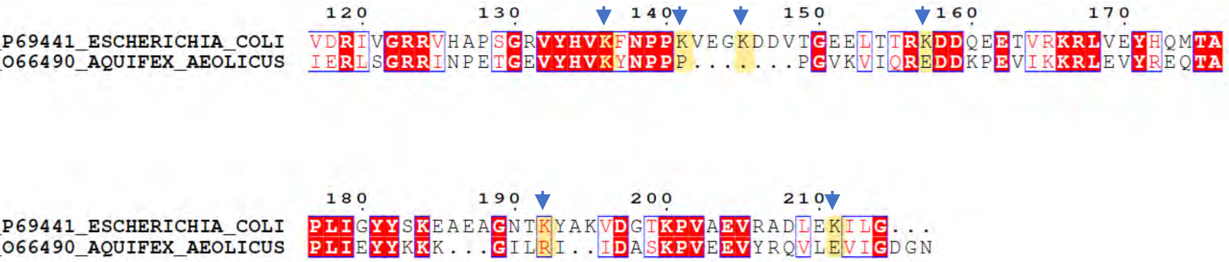


Full sequences in supplemental file.

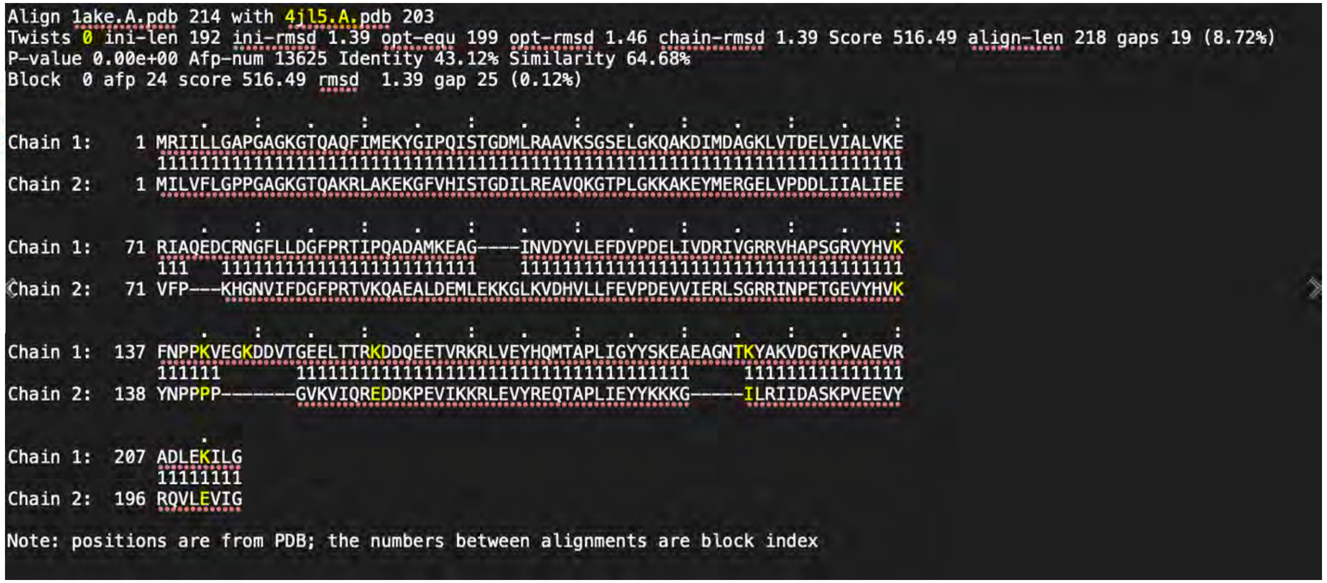

```
Align 1ake.A.pdb 214 with 4jl5.A.pdb 203
Twists 0 ini-len 192 ini-rmsd 1.39 opt-equ 199 opt-rmsd 1.46 chain-rmsd 1.39 Score 516.49 align-len 218 gaps 19 (8.72%)
P-value 0.00e+00 Afp-num 13625 Identity 43.12% Similarity 64.68%
Block  0 afp 24 score 516.49 rmsd  1.39 gap 25 (0.12%)

                .    :    .    :    .    :    .    :    .    :    .    :    .    :
Chain 1:    1 MRIILLGAPGAGKGTQAQFIMEKYGIPQISTGDMLRAAVKSGSELGKQAKDIMDAGKLVTDELVIALVKE
              1111111111111111111111111111111111111111111111111111111111111111111111
Chain 2:    1 MILVFLGPPGAGKGTQAKRLAKEKGFVHISTGDILREAVQKGTPLGKKAKEYMERGELVPDDLIIALIEE

                .    :    .    :    .    :    .    :    .    :    .    :    .    :
Chain 1:   71 RIAQEDCRNGFLLDGFPRTIPQADAMKEAG----INVDYVLEFDVPDELIVDRIVGRRVHAPSGRVYHVK
              111   111111111111111111111111    111111111111111111111111111111111111
Chain 2:   71 VFP---KHGNVIFDGFPRTVKQAEALDEMLEKKGLKVDHVLLFEVPDEVVIERLSGRRINPETGEVYHVK

                .    :    .    :    .    :    .    :    .    :    .    :    .    :
Chain 1:  137 FNPPKVEGKDDVTGEELTTRKDDQEETVRKRLVEYHQMTAPLIGYYSKEAEAGNTKYAKVDGTKPVAEVR
              111111       111111111111111111111111111111111111     111111111111111
Chain 2:  138 YNPPPP-------GVKVIQREDDKPEVIKKRLEVYREQTAPLIEYYKKKG-----ILRIIDASKPVEEVY

                .
Chain 1:  207 ADLEKILG
              11111111
Chain 2:  196 RQVLEVIG

Note: positions are from PDB; the numbers between alignments are block index
```

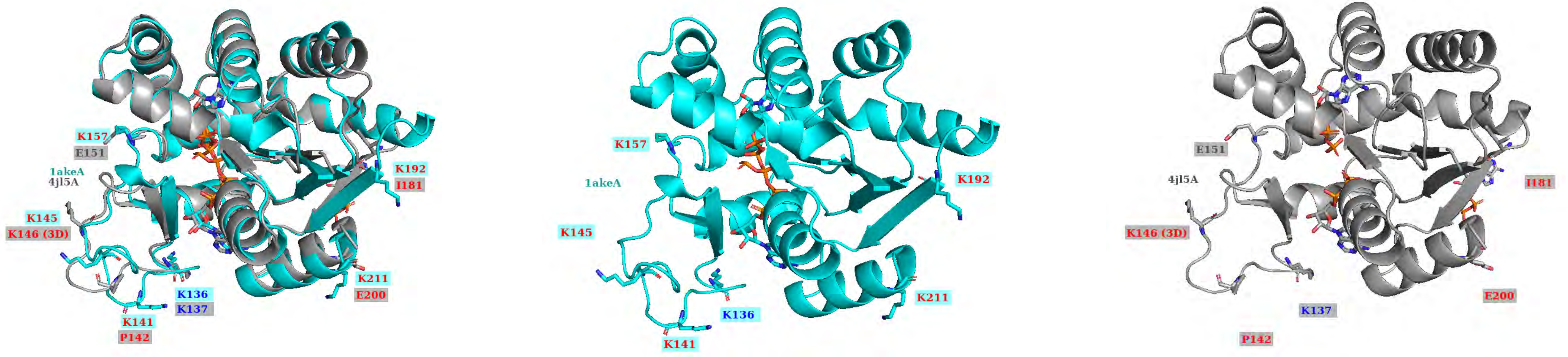

UniProt ID: O66490

PDB ID: 4JL6_A

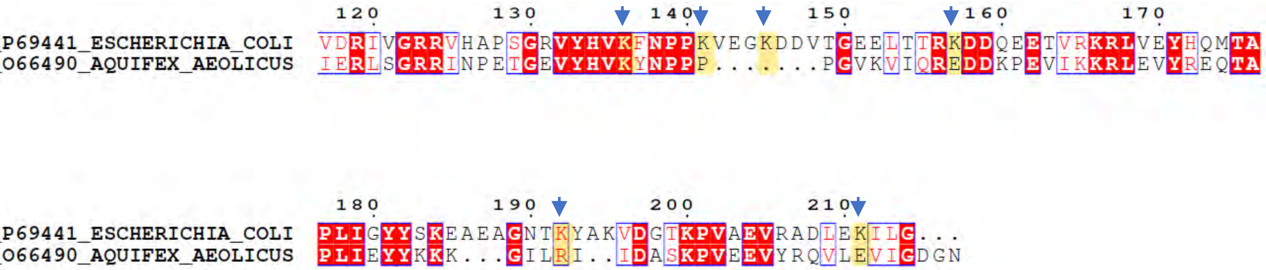


Full sequences in supplemental file.

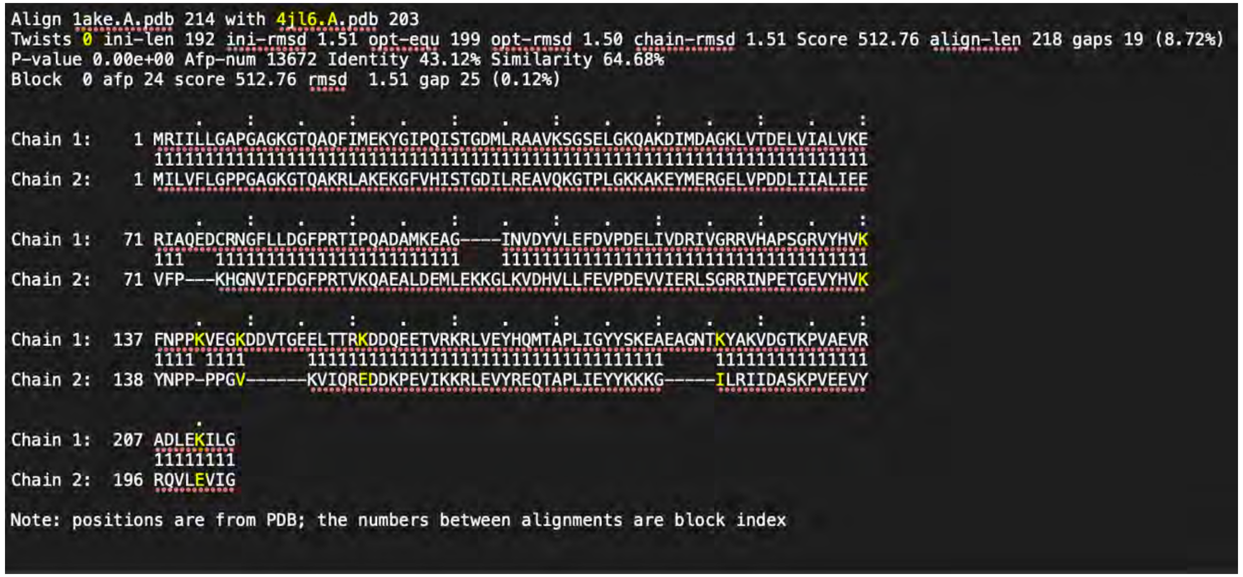

```
Align 1ake.A.pdb 214 with 4jl6.A.pdb 203
Twists 0 ini-len 192 ini-rmsd 1.51 opt-equ 199 opt-rmsd 1.50 chain-rmsd 1.51 Score 512.76 align-len 218 gaps 19 (8.72%)
P-value 0.00e+00 Afp-num 13672 Identity 43.12% Similarity 64.68%
Block  0 afp 24 score 512.76 rmsd  1.51 gap 25 (0.12%)

                   .    :    .    :    .    :    .    :    .    :    .    :    .    :
Chain 1:     1 MRIILLGAPGAGKGTQAQFIMEKYGIPQISTGDMLRAAVKSGSELGKQAKDIMDAGKLVTDELVIALVKE
               111111111111111111111111111111111111111111111111111111111111111111111111
Chain 2:     1 MILVFLGPPGAGKGTQAKRLAKEKGFVHISTGDILREAVQKGTPLGKKAKEYMERGELVPDDLIIALIEE

                   .    :    .    :    .    :    .    :    .    :    .    :    .    :
Chain 1:    71 RIAQEDCRNGFLLDGFPRTIPQADAMKEAG----INVDYVLEFDVPDELIVDRIVGRRVHAPSGRVYHVK
               111   111111111111111111111111    1111111111111111111111111111111111111
Chain 2:    71 VFP---KHGNVIFDGFPRTVKQAEALDEMLEKKGLKVDHVLLFEVPDEVVIERLSGRRINPETGEVYHVK

                   .    :    .    :    .    :    .    :    .    :    .    :    .    :
Chain 1:   137 FNPPKVEGKDDVTGEELTTRKDDQEETVRKRLVEYHQMTAPLIGYYSKEAEAGNTKYAKVDGTKPVAEVR
               1111 1111      11111111111111111111111111111111111     111111111111111
Chain 2:   138 YNPP-PPGV------KVIQREDDKPEVIKKRLEVYREQTAPLIEYYKKKG-----ILRIIDASKPVEEVY

                   .
Chain 1:   207 ADLEKILG
               11111111
Chain 2:   196 RQVLEVIG

Note: positions are from PDB; the numbers between alignments are block index
```

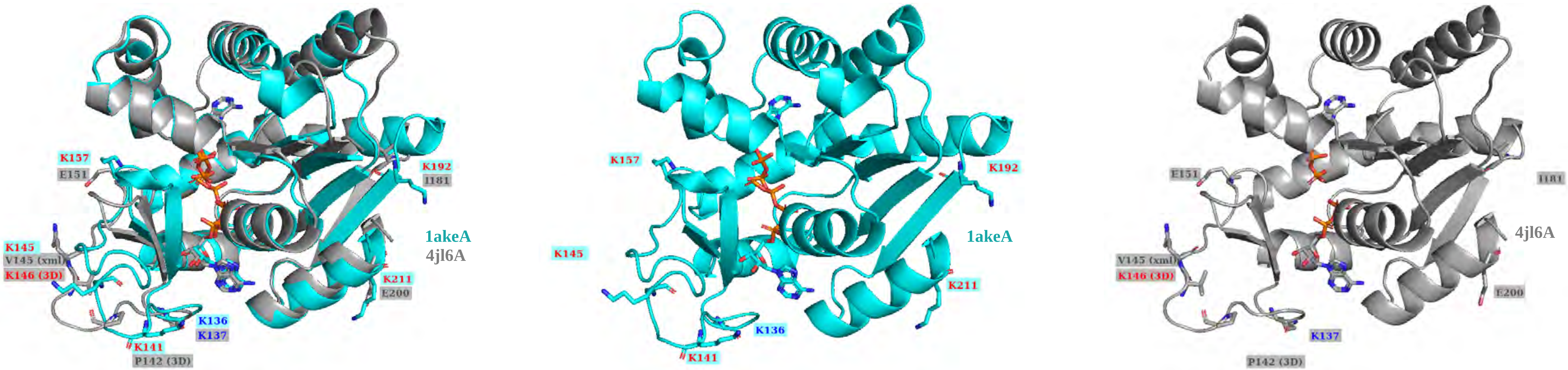

UniProt ID: O66490

PDB ID: 4JL8_B

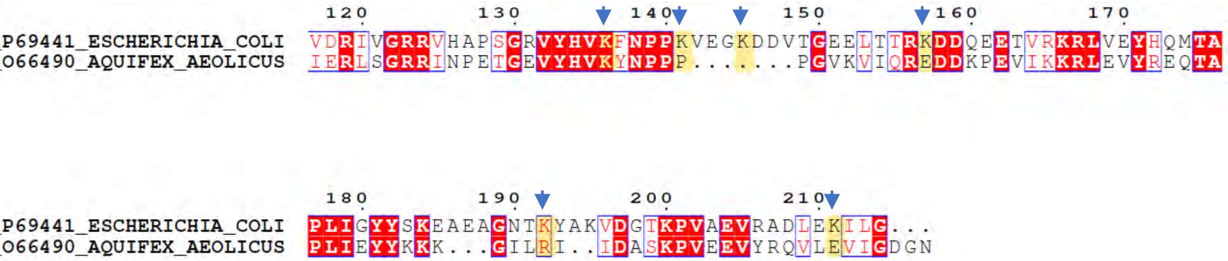


Full sequences in supplemental file.

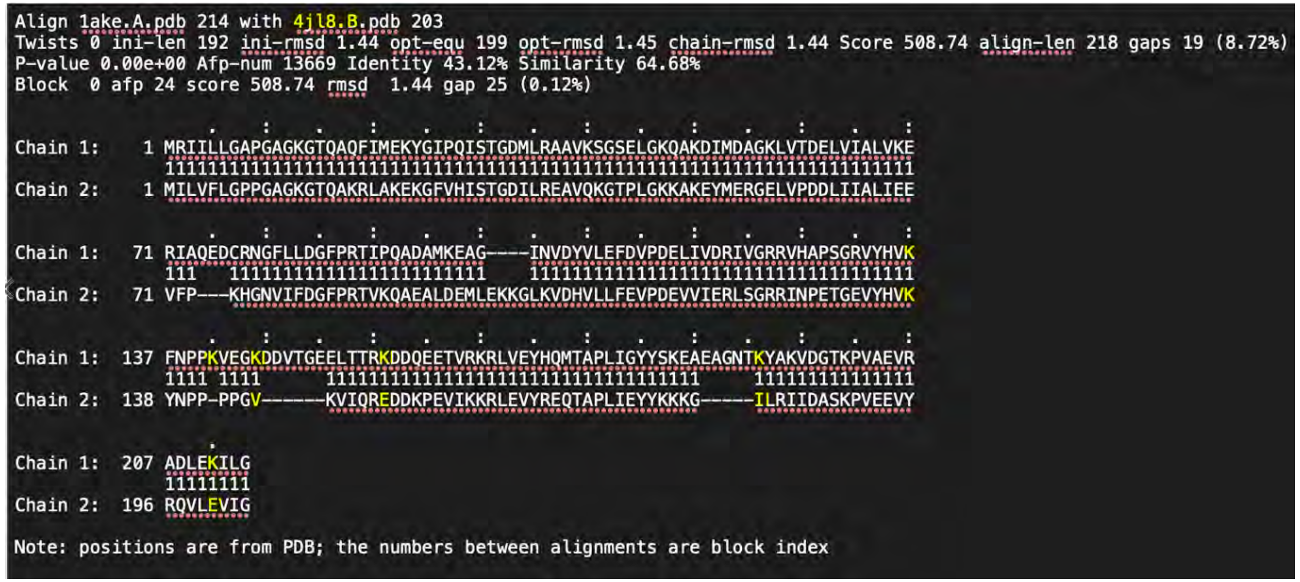

```
Align 1ake.A.pdb 214 with 4jl8.B.pdb 203
Twists 0 ini-len 192 ini-rmsd 1.44 opt-equ 199 opt-rmsd 1.45 chain-rmsd 1.44 Score 508.74 align-len 218 gaps 19 (8.72%)
P-value 0.00e+00 Afp-num 13669 Identity 43.12% Similarity 64.68%
Block  0 afp 24 score 508.74 rmsd  1.44 gap 25 (0.12%)

                 .    :    .    :    .    :    .    :    .    :    .    :    .    :
Chain 1:    1 MRIILLGAPGAGKGTQAQFIMEKYGIPQISTGDMLRAAVKSGSELGKQAKDIMDAGKLVTDELVIALVKE
              1111111111111111111111111111111111111111111111111111111111111111111111
Chain 2:    1 MILVFLGPPGAGKGTQAKRLAKEKGFVHISTGDILREAVQKGTPLGKKAKEYMERGELVPDDLIIALIEE

                 .    :    .    :    .    :    .    :    .    :    .    :    .    :
Chain 1:   71 RIAQEDCRNGFLLDGFPRTIPQADAMKEAG----INVDYVLEFDVPDELIVDRIVGRRVHAPSGRVYHVK
              111   111111111111111111111111    111111111111111111111111111111111111
Chain 2:   71 VFP---KHGNVIFDGFPRTVKQAEALDEMLEKKGLKVDHVLLFEVPDEVVIERLSGRRINPETGEVYHVK

                 .    :    .    :    .    :    .    :    .    :    .    :    .    :
Chain 1:  137 FNPPKVEGKDDVTGEELTTRKDDQEETVRKRLVEYHQMTAPLIGYYSKEAEAGNTKYAKVDGTKPVAEVR
              1111 1111      111111111111111111111111111111111111     111111111111111
Chain 2:  138 YNPP-PPGV------KVIQREDDKPEVIKKRLEVYREQTAPLIEYYKKKG-----ILRIIDASKPVEEVY

                 .
Chain 1:  207 ADLEKILG
              11111111
Chain 2:  196 RQVLEVIG

Note: positions are from PDB; the numbers between alignments are block index
```

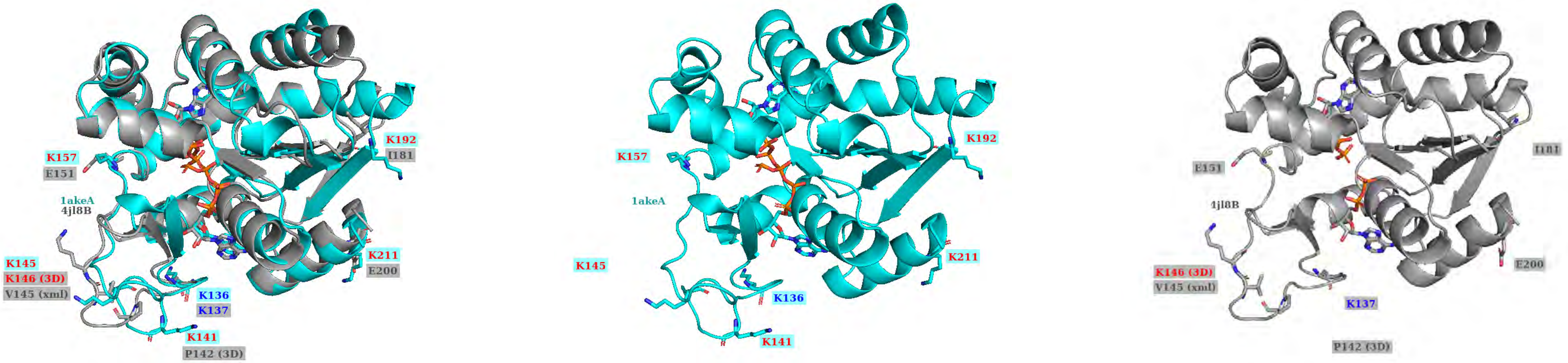

UniProt ID: O66490

PDB ID: 4JLA_B

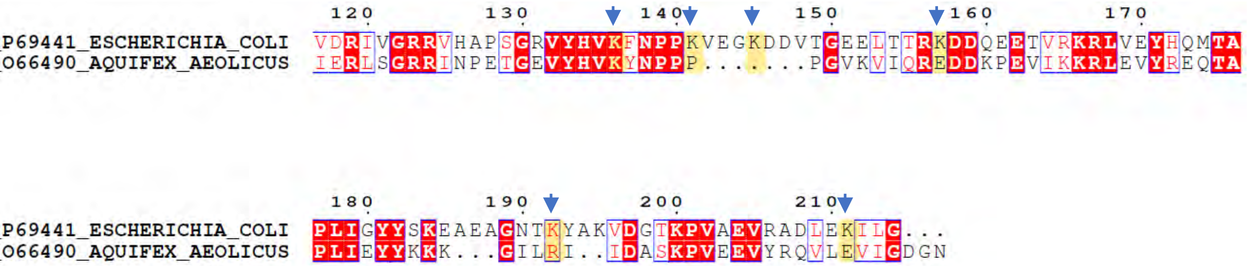


Full sequences in supplemental file.

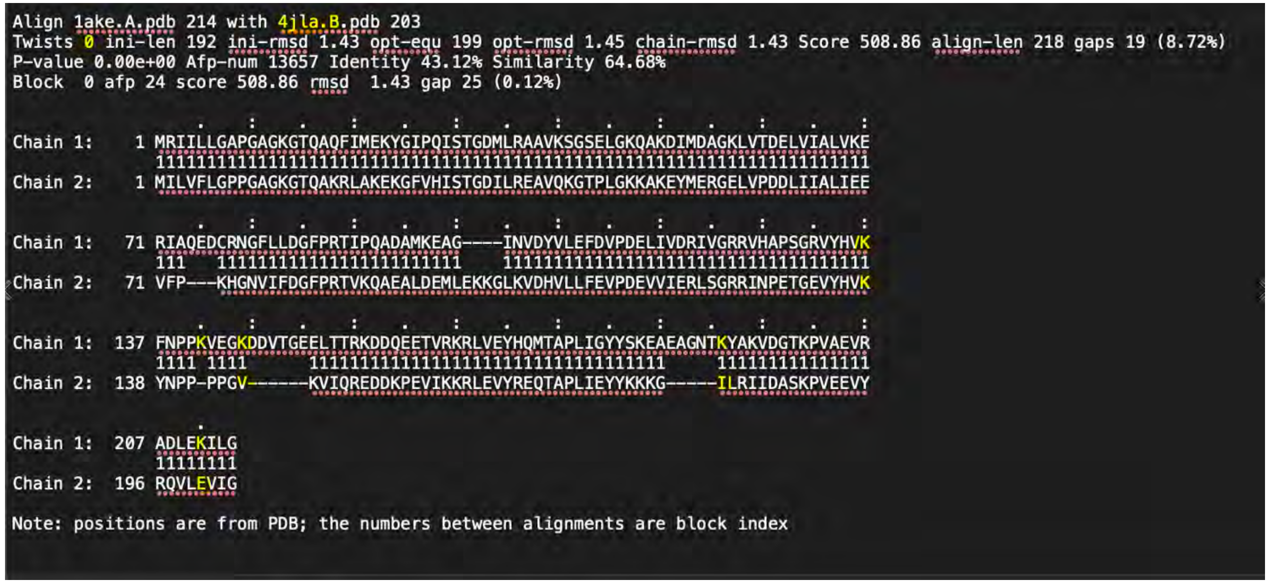


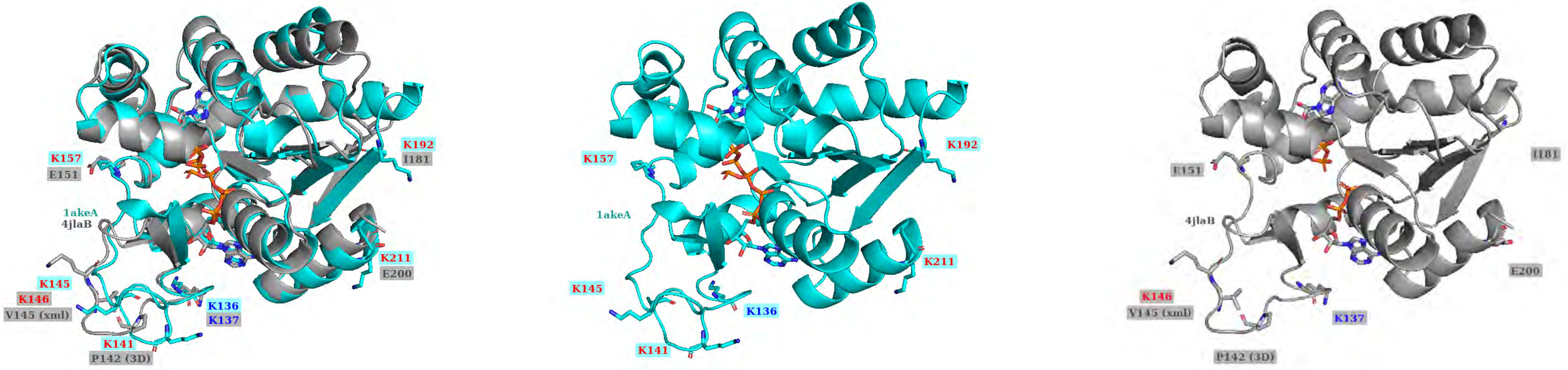

UniProt ID: O66490

PDB ID: 4JLB_B

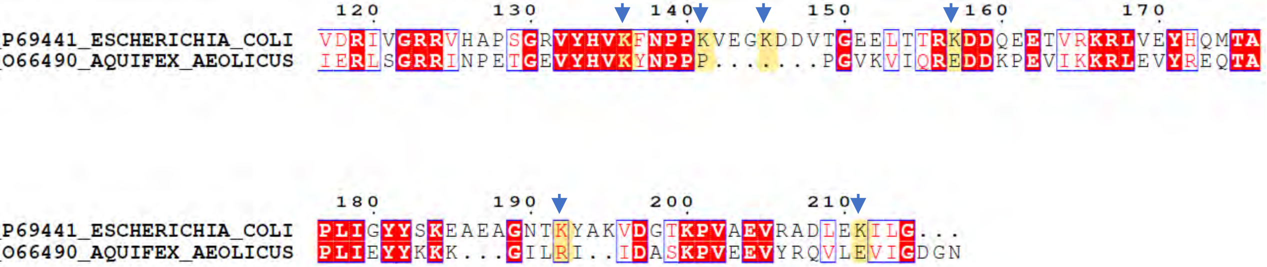


Full sequences in supplemental file.

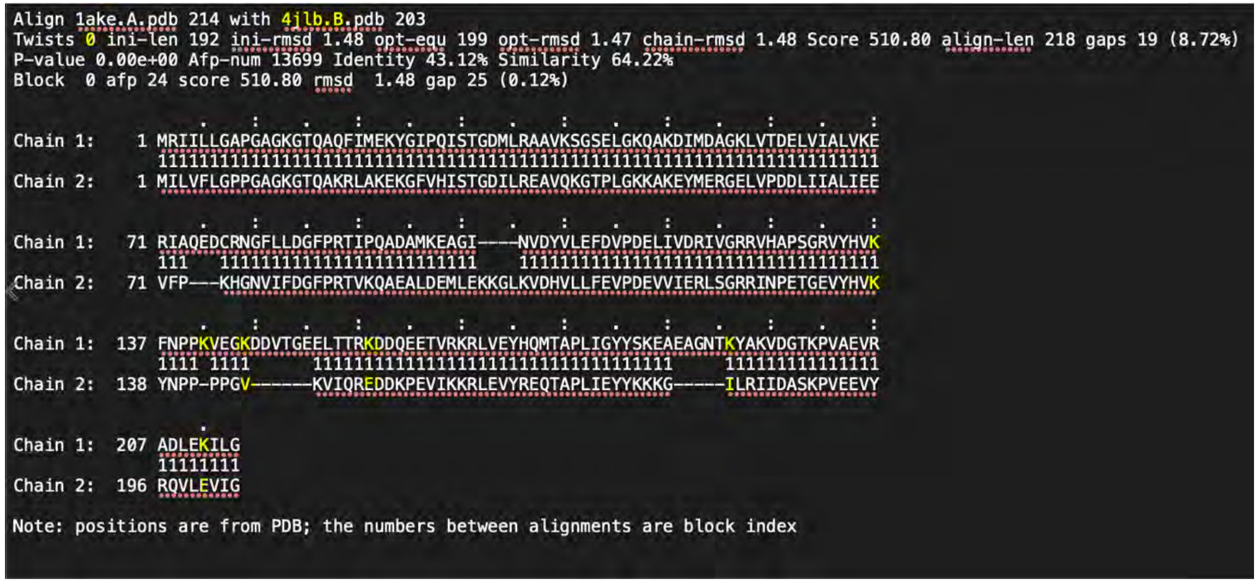

```
Align 1ake.A.pdb 214 with 4jlb.B.pdb 203
Twists 0 ini-len 192 ini-rmsd 1.48 opt-equ 199 opt-rmsd 1.47 chain-rmsd 1.48 Score 510.80 align-len 218 gaps 19 (8.72%)
P-value 0.00e+00 Afp-num 13699 Identity 43.12% Similarity 64.22%
Block  0 afp 24 score 510.80 rmsd  1.48 gap 25 (0.12%)

                .    :    .    :    .    :    .    :    .    :    .    :    .    :
Chain 1:    1 MRIILLGAPGAGKGTQAQFIMEKYGIPQISTGDMLRAAVKSGSELGKQAKDIMDAGKLVTDELVIALVKE
              11111111111111111111111111111111111111111111111111111111111111111111111
Chain 2:    1 MILVFLGPPGAGKGTQAKRLAKEKGFVHISTGDILREAVQKGTPLGKKAKEYMERGELVPDDLIIALIEE

                .    :    .    :    .    :    .    :    .    :    .    :    .    :
Chain 1:   71 RIAQEDCRNGFLLDGFPRTIPQADAMKEAGI----NVDYVLEFDVPDELIVDRIVGRRVHAPSGRVYHVK
              111   1111111111111111111111111    111111111111111111111111111111111111
Chain 2:   71 VFP---KHGNVIFDGFPRTVKQAEALDEMLEKKGLKVDHVLLFEVPDEVVIERLSGRRINPETGEVYHVK

                .    :    .    :    .    :    .    :    .    :    .    :    .    :
Chain 1:  137 FNPPKVEGKDDVTGEELTTRKDDQEETVRKRLVEYHQMTAPLIGYYSKEAEAGNTKYAKVDGTKPVAEVR
              1111 1111      111111111111111111111111111111111111     111111111111111
Chain 2:  138 YNPP-PPGV------KVIQREDDKPEVIKKRLEVYREQTAPLIEYYKKKG-----ILRIIDASKPVEEVY

                .
Chain 1:  207 ADLEKILG
              11111111
Chain 2:  196 RQVLEVIG

Note: positions are from PDB; the numbers between alignments are block index
```

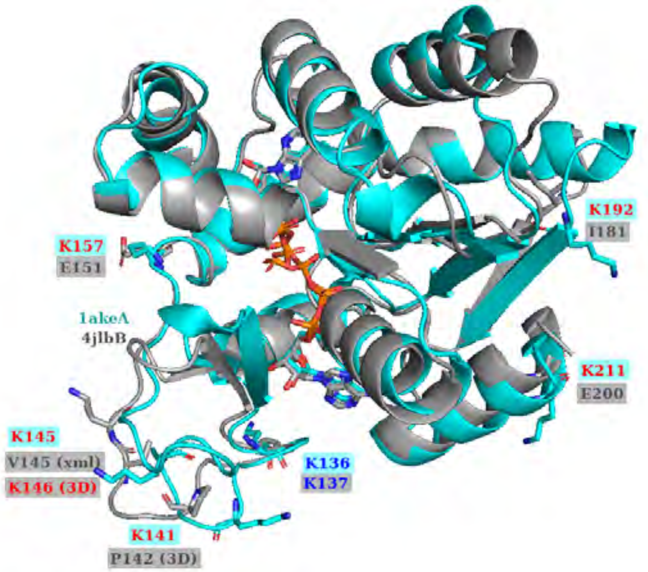


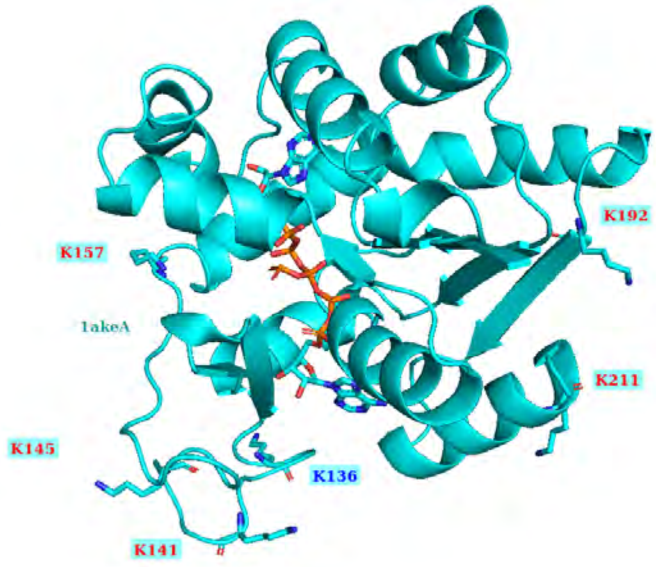


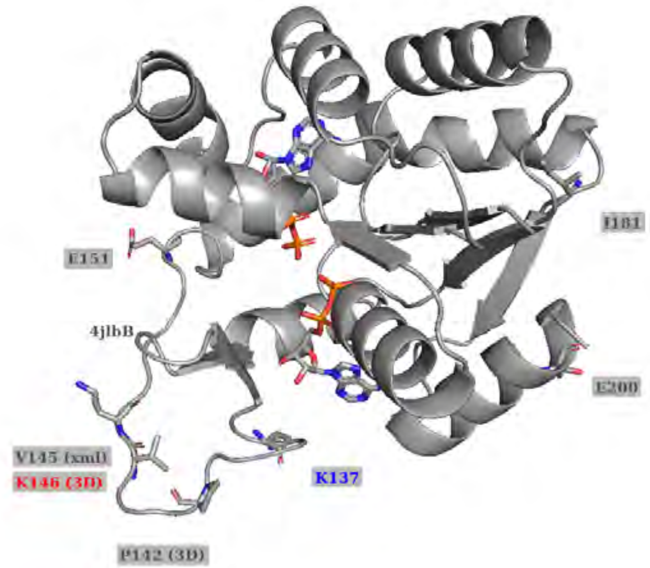

UniProt ID: O66490

PDB ID: 4JLD_A

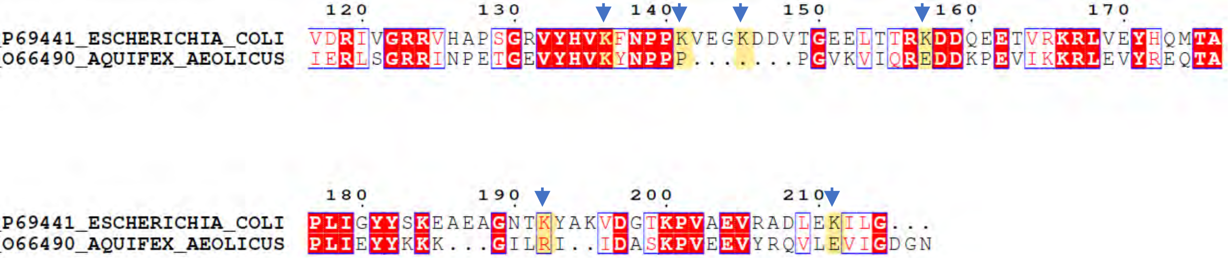


Full sequences in supplemental file.

```
Align 1ake.A.pdb 214 with 4jld.A.pdb 203
Twists 0 ini-len 192 ini-rmsd 1.48 opt-equ 199 opt-rmsd 1.49 chain-rmsd 1.48 Score 508.34 align-len 218 gaps 19 (8.72%)
P-value 0.00e+00 Afp-num 13626 Identity 43.12% Similarity 64.68%
Block  0 afp 24 score 508.34 rmsd  1.48 gap 25 (0.12%)

                .    :    .    :    .    :    .    :    .    :    .    :    .    :
Chain 1:    1 MRIILLGAPGAGKGTQAQFIMEKYGIPQISTGDMLRAAVKSGSELGKQAKDIMDAGKLVTDELVIALVKE
              1111111111111111111111111111111111111111111111111111111111111111111111
Chain 2:    1 MILVFLGPPGAGKGTQAKRLAKEKGFVHISTGDILREAVQKGTPLGKKAKEYMERGELVPDDLIIALIEE

                .    :    .    :    .    :    .    :    .    :    .    :    .    :
Chain 1:   71 RIAQEDCRNGFLLDGFPRTIPQADAMKEAG----INVDYVLEFDVPDELIVDRIVGRRVHAPSGRVYHVK
              111   111111111111111111111111    111111111111111111111111111111111111
Chain 2:   71 VFP---KHGNVIFDGFPRTVKQAEALDEMLEKKGLKVDHVLLFEVPDEVVIERLSGRRINPETGEVYHVK

                .    :    .    :    .    :    .    :    .    :    .    :    .    :
Chain 1:  137 FNPPKVEGKDDVTGEELTTRKDDQEETVRKRLVEYHQMTAPLIGYYSKEAEAGNTKYAKVDGTKPVAEVR
              1111 1111      11111111111111111111111111111111111     111111111111111
Chain 2:  138 YNPP-PPGV------KVIQREDDKPEVIKKRLEVYREQTAPLIEYYKKKG-----ILRIIDASKPVEEVY

                .
Chain 1:  207 ADLEKILG
              11111111
Chain 2:  196 RQVLEVIG

Note: positions are from PDB; the numbers between alignments are block index
```

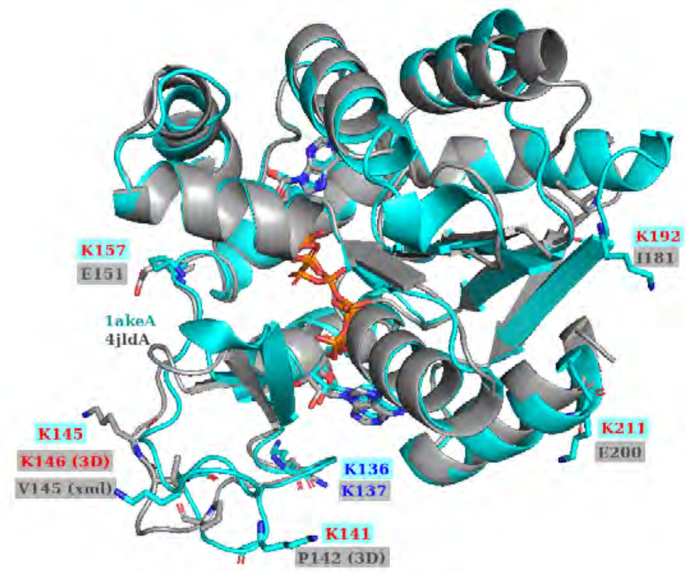


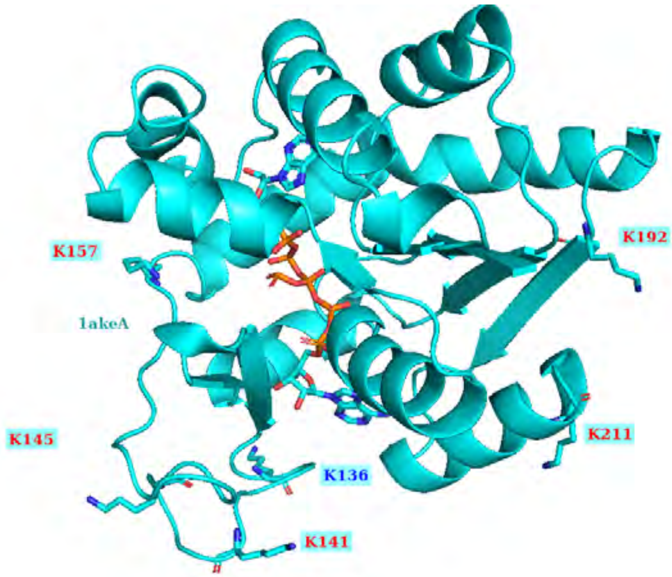


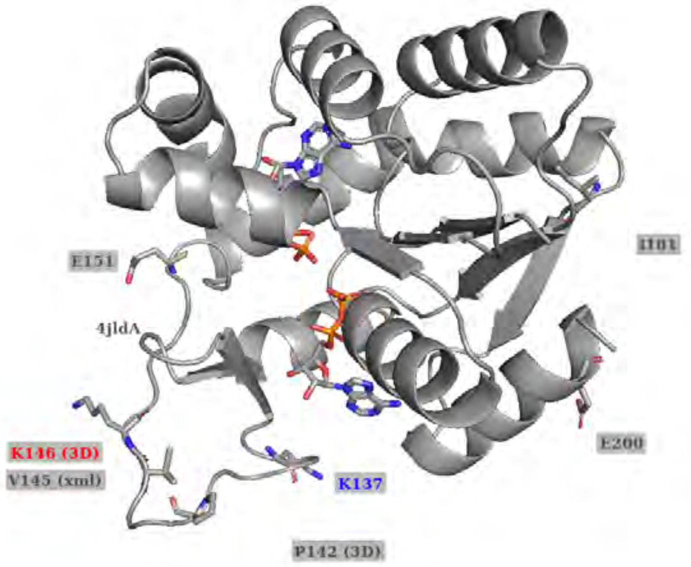

UniProt ID: O66490

PDB ID: 4JLO_A

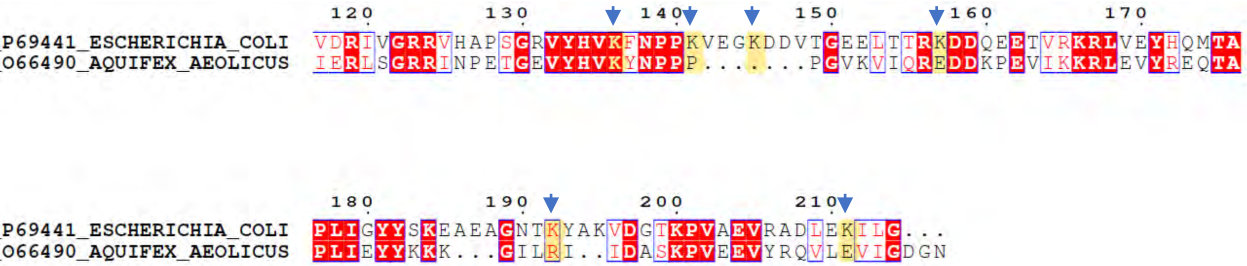


Full sequences in supplemental file.

```
Align 1ake.A.pdb 214 with 4jlo.A.pdb 203
Twists 0 ini-len 192 ini-rmsd 1.48 opt-equ 199 opt-rmsd 1.48 chain-rmsd 1.48 Score 512.29 align-len 218 gaps 19 (8.72%)
P-value 0.00e+00 Afp-num 13627 Identity 42.66% Similarity 64.68%
Block  0 afp 24 score 512.29 rmsd  1.48 gap 25 (0.12%)

                .    :    .    :    .    :    .    :    .    :    .    :    .    :
Chain 1:    1 MRIILLGAPGAGKGTQAQFIMEKYGIPQISTGDMLRAAVKSGSELGKQAKDIMDAGKLVTDELVIALVKE
              1111111111111111111111111111111111111111111111111111111111111111111111
Chain 2:    1 MILVFLGPPGAGKGTQAKRLAKEKGFVHISTGDILREAVQKGTPLGKKAKEYMERGELVPDDLIIALIEE

                .    :    .    :    .    :    .    :    .    :    .    :    .    :
Chain 1:   71 RIAQEDCRNGFLLDGFPRTIPQADAMKEAG----INVDYVLEFDVPDELIVDRIVGRRVHAPSGRVYHVK
              111   11111111111111111111111111    11111111111111111111111111111111111
Chain 2:   71 VFP---KHGNVIFDGFPRTVKQAEALDEMLEKKGLKVDHVLLFEVPDEVVIERLSGRRINPETGEVYHVK

                .    :    .    :    .    :    .    :    .    :    .    :    .    :
Chain 1:  137 FNPPKVEGKDDVTGEELTTRKDDQEETVRKRLVEYHQMTAPLIGYYSKEAEAGNTKYAKVDGTKPVAEVR
              1111 1111      11111111111111111111111111111111111111      111111111111111
Chain 2:  138 YNPP-PPGV------KVIQKEDDKPEVIKKRLEVYREQTAPLIEYYKKKG-----ILRIIDASKPVEEVY

                .
Chain 1:  207 ADLEKILG
              11111111
Chain 2:  196 RQVLEVIG

Note: positions are from PDB; the numbers between alignments are block index
```

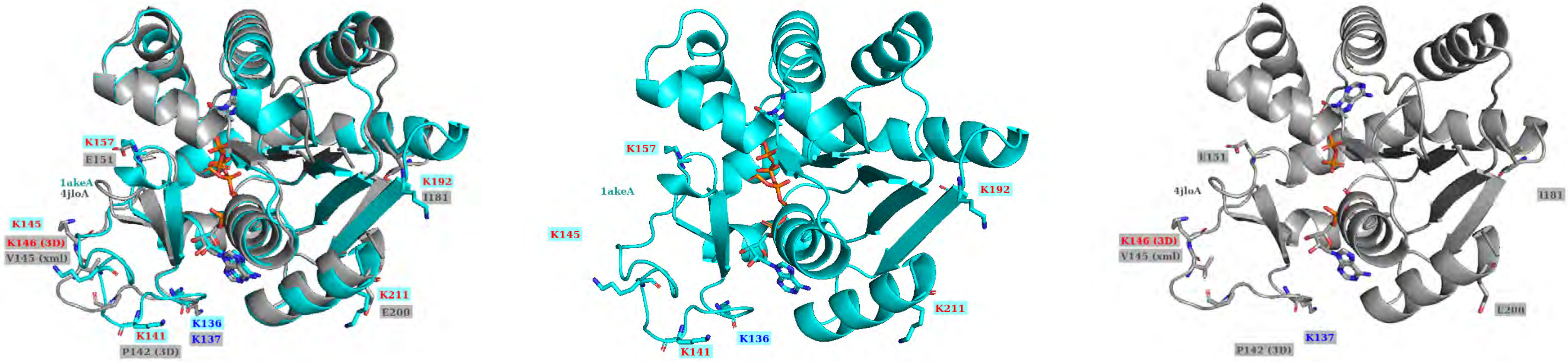

UniProt ID: O66490

PDB ID: 4JLP_A

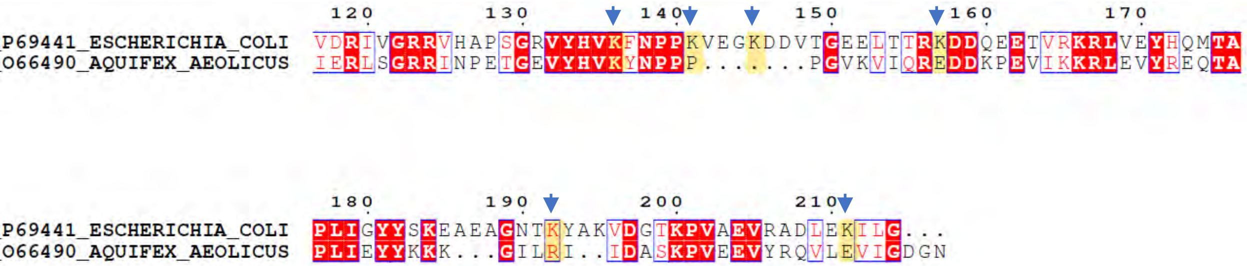


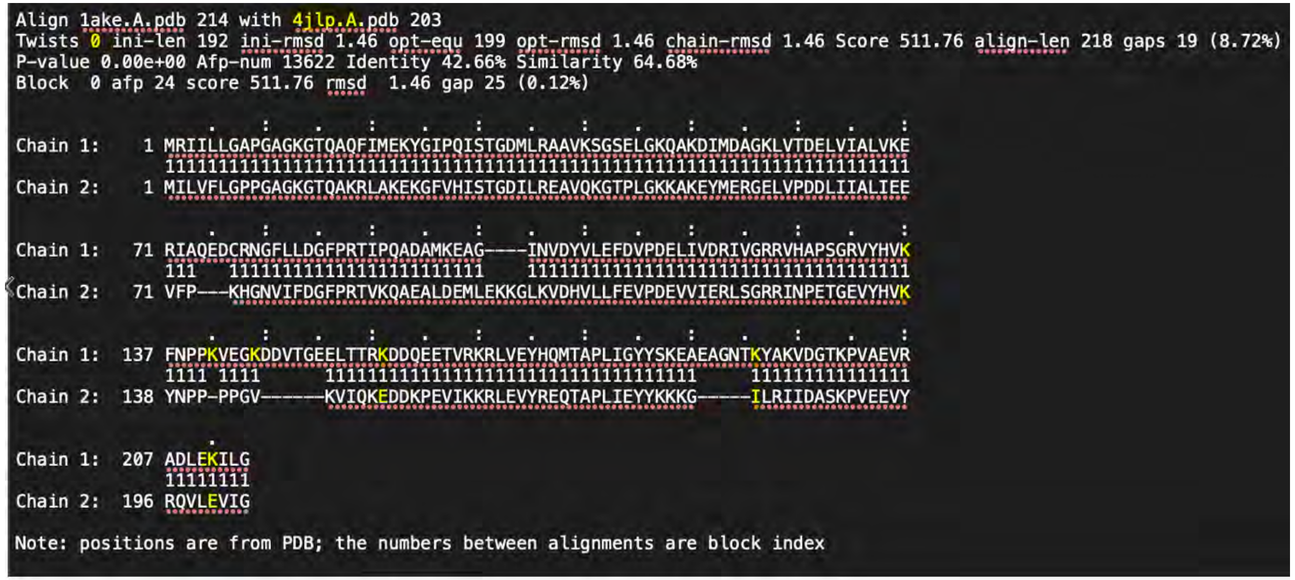


Full sequences in supplemental file.

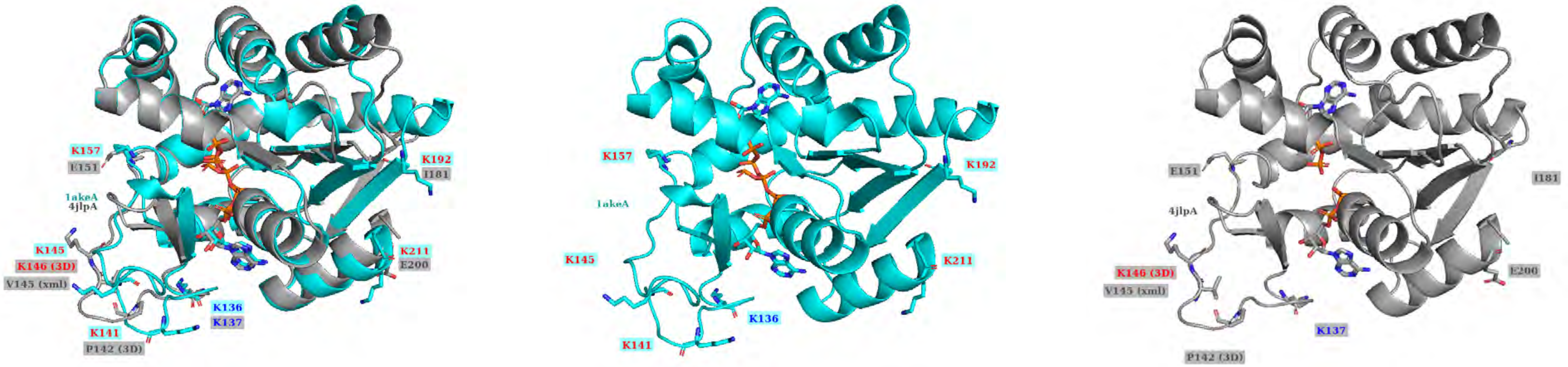

UniProt ID: P00568

PDB ID: 1Z83_C

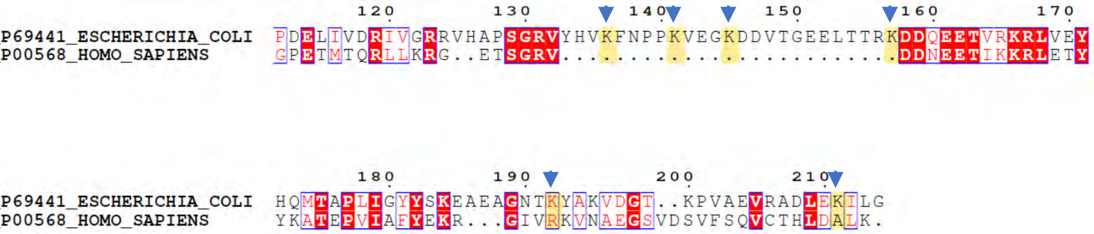


Full sequences in supplemental file.

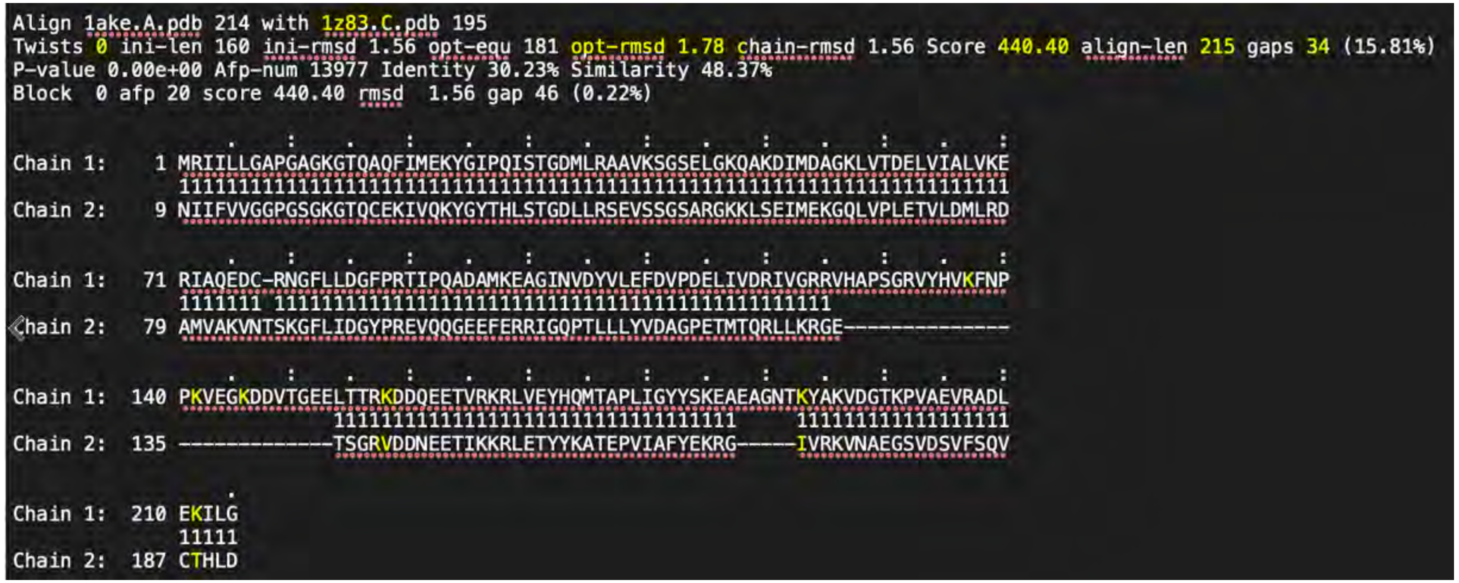


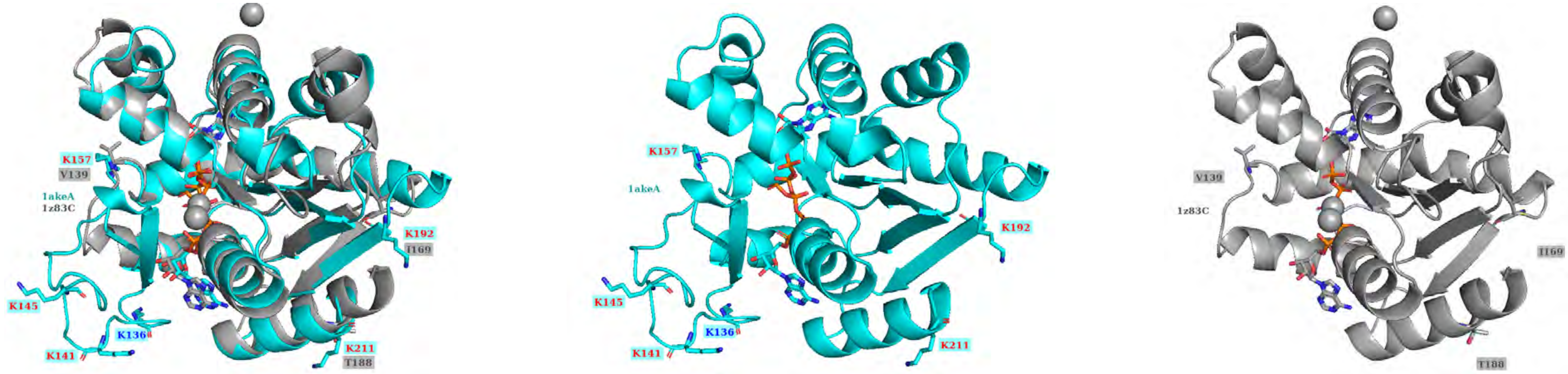

UniProt ID: P00568

PDB ID: 2C95_A

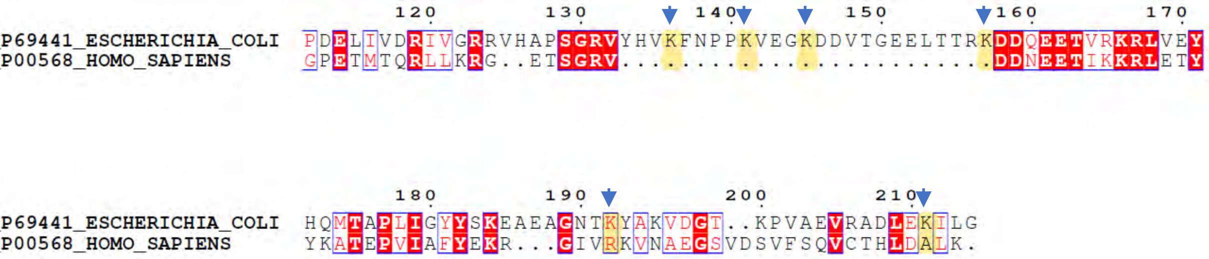


Full sequences in supplemental file.

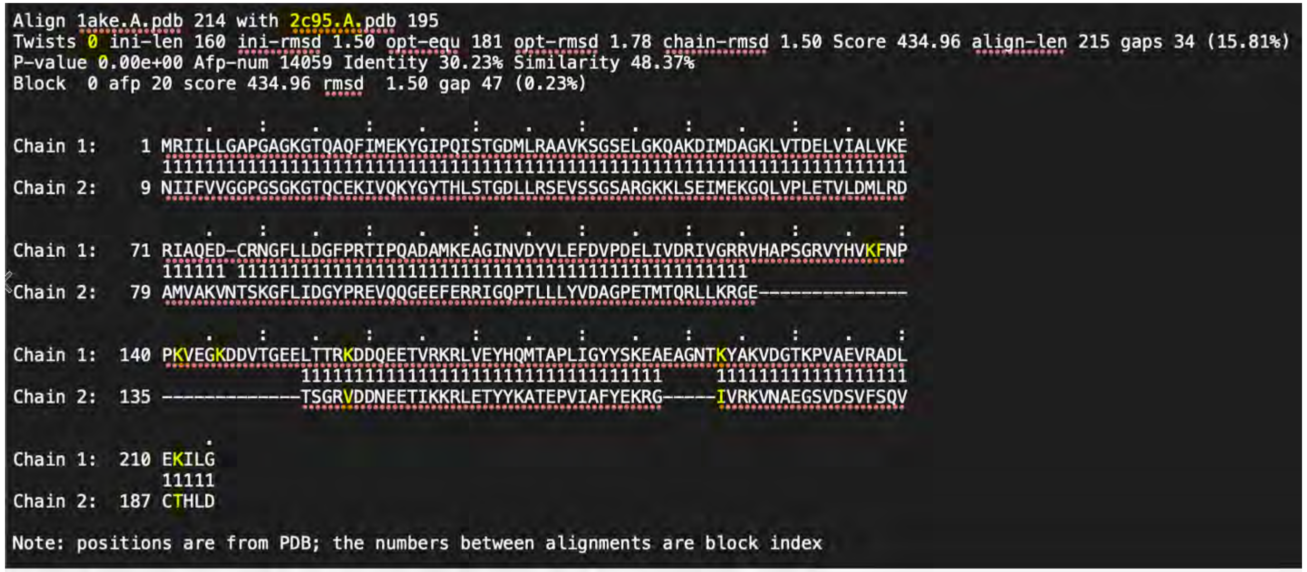

```
Align 1ake.A.pdb 214 with 2c95.A.pdb 195
Twists 0 ini-len 160 ini-rmsd 1.50 opt-equ 181 opt-rmsd 1.78 chain-rmsd 1.50 Score 434.96 align-len 215 gaps 34 (15.81%)
P-value 0.00e+00 Afp-num 14059 Identity 30.23% Similarity 48.37%
Block  0 afp 20 score 434.96 rmsd  1.50 gap 47 (0.23%)

              .    :    .    :    .    :    .    :    .    :    .    :    .    :
Chain 1:    1 MRIILLGAPGAGKGTQAQFIMEKYGIPQISTGDMLRAAVKSGSELGKQAKDIMDAGKLVTDELVIALVKE
              1111111111111111111111111111111111111111111111111111111111111111111111
Chain 2:    9 NIIFVVGGPGSGKGTQCEKIVQKYGYTHLSTGDLLRSEVSSGSARGKKLSEIMEKGQLVPLETVLDMLRD

              .    :    .    :    .    :    .    :    .    :    .    :    .    :
Chain 1:   71 RIAQED-CRNGFLLDGFPRTIPQADAMKEAGINVDYVLEFDVPDELIVDRIVGRRVHAPSGRVYHVKFNP
              111111 1111111111111111111111111111111111111111111111111
Chain 2:   79 AMVAKVNTSKGFLIDGYPREVQQGEEFERRIGQPTLLLYVDAGPETMTQRLLKRGE--------------

              .    :    .    :    .    :    .    :    .    :    .    :    .    :
Chain 1:  140 PKVEGKDDVTGEELTTRKDDQEETVRKRLVEYHQMTAPLIGYYSKEAEAGNTKYAKVDGTKPVAEVRADL
                           111111111111111111111111111111111     111111111111111111
Chain 2:  135 -------------TSGRVDDNEETIKKRLETYYKATEPVIAFYEKRG-----IVRKVNAEGSVDSVFSQV

              .
Chain 1:  210 EKILG
              11111
Chain 2:  187 CTHLD

Note: positions are from PDB; the numbers between alignments are block index
```

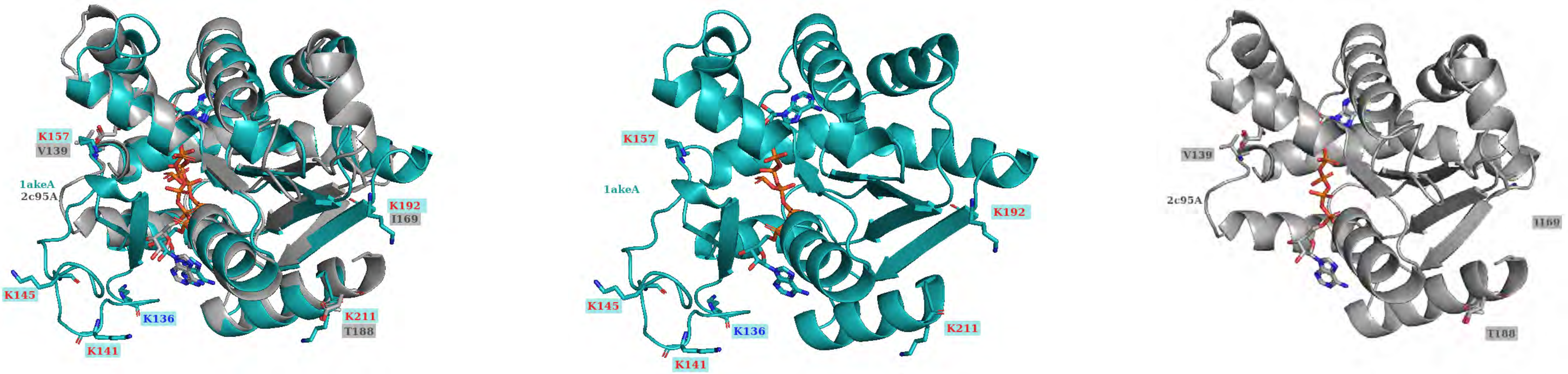

UniProt ID: P00571

PDB ID: 3ADK_A

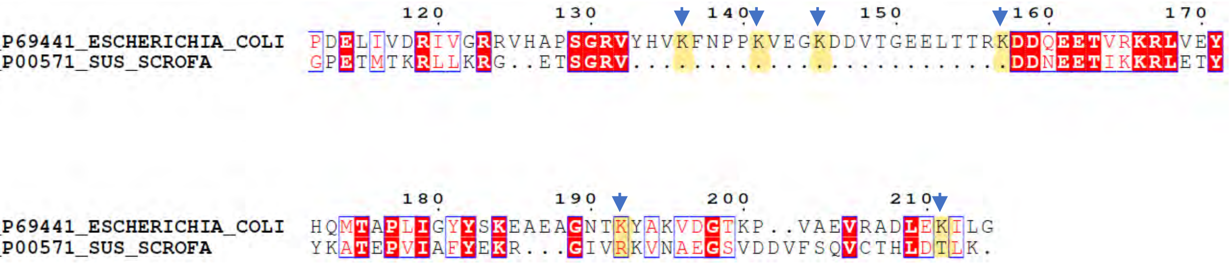


Full sequences in supplemental file.

```
Align 1ake.A.pdb 214 with 3adk.A.pdb 194
Twists 0 ini-len 160 ini-rmsd 2.73 opt-equ 179 opt-rmsd 3.06 chain-rmsd 2.73 Score 405.42 align-len 215 gaps 36 (16.74%)
P-value 1.27e-14 Afp-num 13923 Identity 30.70% Similarity 48.37%
Block  0 afp 20 score 405.42 rmsd  2.73 gap 51 (0.24%)

                  .    :    .    :    .    :    .    :    .    :    .    :    .    :
Chain 1:    1 MRIILLGAPGAGKGTQAQFIMEKYGIPQISTGDMLRAAVKSGSELGKQAKDIMDAGKLVTDELVIALVKE
              1111111111111111111111111111111111111111111111111111111 11111111111111
Chain 2:    9 KIIFVVGGPGSGKGTQCEKIVQKYGYTHLSTGDLLRAEVSSGSARGKMLSEIMEKGQLVPLETVLDMLRD

                  .    :    .    :    .    :    .    :    .    :    .    :    .    :
Chain 1:   71 RIAQEDC-RNGFLLDGFPRTIPQADAMKEAGINVDYVLEFDVPDELIVDRIVGRRVHAPSGRVYHVKFNP
              1111111 11111111111111111111111111111111111111111111111
Chain 2:   79 AMVAKVDTSKGFLIDGYPREVKQGEEFERKIGQPTLLLYVDAGPETMTKRLLKRGE--------------

                  .    :    .    :    .    :    .    :    .    :    .    :    .    :
Chain 1:  140 PKVEGKDDVTGEELTTRKDDQEETVRKRLVEYHQMTAPLIGYYSKEAEAGNTKYAKVDGTKPVAEVRADL
                            111111 11111111111111111111111111     11111111111111111
Chain 2:  135 --------------TSGRVDDNEETIKKRLETYYKATEPVIAFYEKRG-----IVRKVNAEGSVDDVFSQV

                  .
Chain 1:  210 EKILG
              11111
Chain 2:  187 CTHLD

Note: positions are from PDB; the numbers between alignments are block index
```

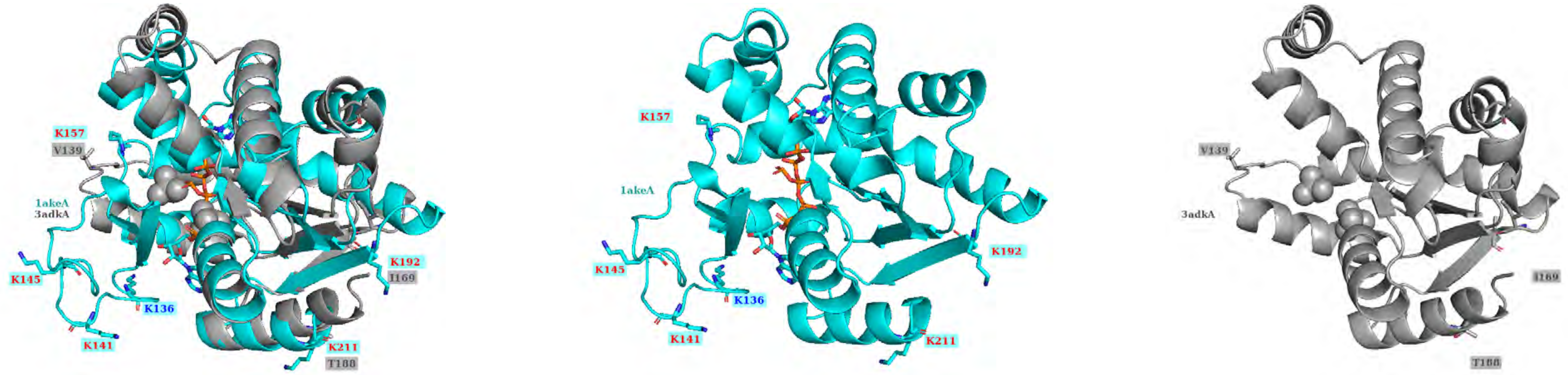

UniProt ID: P07170

PDB ID: 1AKY_A

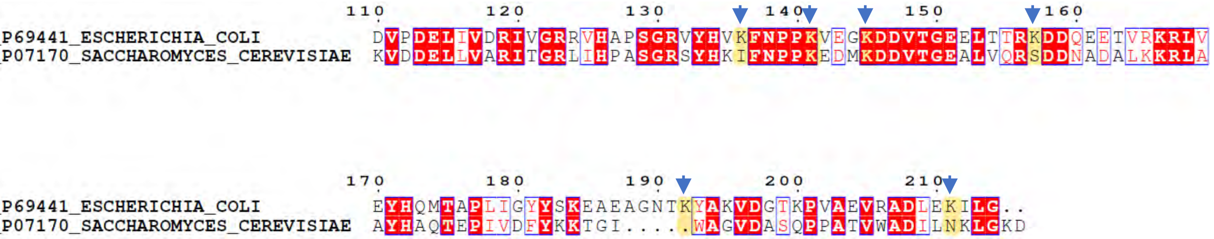


Full sequences in supplemental file.

```
Align 1ake.A.pdb 214 with 1aky.A.pdb 218
Twists 0 ini-len 200 ini-rmsd 1.40 opt-equ 209 opt-rmsd 1.41 chain-rmsd 1.40 Score 552.61 align-len 219 gaps 10 (4.57%)
P-value 0.00e+00 Afp-num 14407 Identity 44.75% Similarity 64.84%
Block  0 afp 25 score 552.61 rmsd  1.40 gap 18 (0.08%)

                 .    :    .    :    .    :    .    :    .    :    .    :    .    :
Chain 1:    1 MRIILLGAPGAGKGTQAQFIMEKYGIPQISTGDMLRAAVKSGSELGKQAKDIMDAGKLVTDELVIALVKE
              1111111111111111111111111111111111111111111111111111111111111111111111
Chain 2:    5 IRMVLIGPPGAGKGTQAPNLQERFHAAHLATGDMLRSQIAKGTQLGLEAKKIMDQGGLVSDDIMVNMIKD

                 .    :    .    :    .    :    .    :    .    :    .    :    .    :
Chain 1:   71 RIA-QEDCRNGFLLDGFPRTIPQADAMKEAGI----NVDYVLEFDVPDELIVDRIVGRRVHAPSGRVYHV
              111 1111111111111111111111111111    111111111111111111111111111111111111
Chain 2:   75 ELTNNPACKNGFILDGFPRTIPQAEKLDQMLKEQGTPLEKAIELKVDDELLVARITGRLIHPASGRSYHK

                 .    :    .    :    .    :    .    :    .    :    .    :    .    :
Chain 1:  136 KFNPPKVEGKDDVTGEELTTRKDDQEETVRKRLVEYHQMTAPLIGYYSKEAEAGNTKYAKVDGTKPVAEV
              11111111111111111111111111111111111111111111111111     11111111111111
Chain 2:  145 IFNPPKEDMKDDVTGEALVQRSDDNADALKKRLAAYHAQTEPIVDFYKKTG-----IWAGVDASQPPATV

                 .
Chain 1:  206 RADLEKILG
              111111111
Chain 2:  210 WADILNKLG

Note: positions are from PDB; the numbers between alignments are block index
```

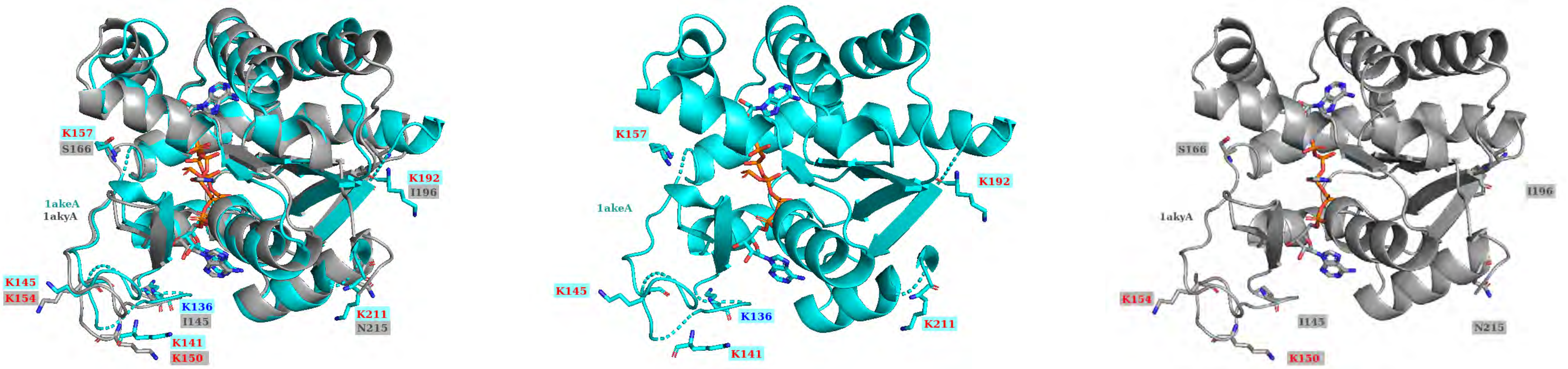

UniProt ID: P07170

PDB ID: 1DVR_B

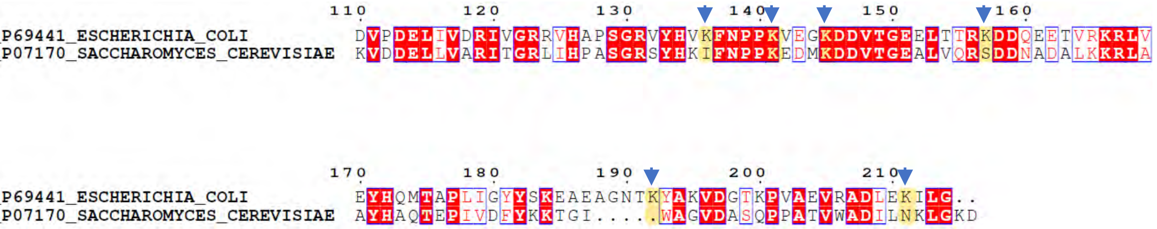


Full sequences in supplemental file.

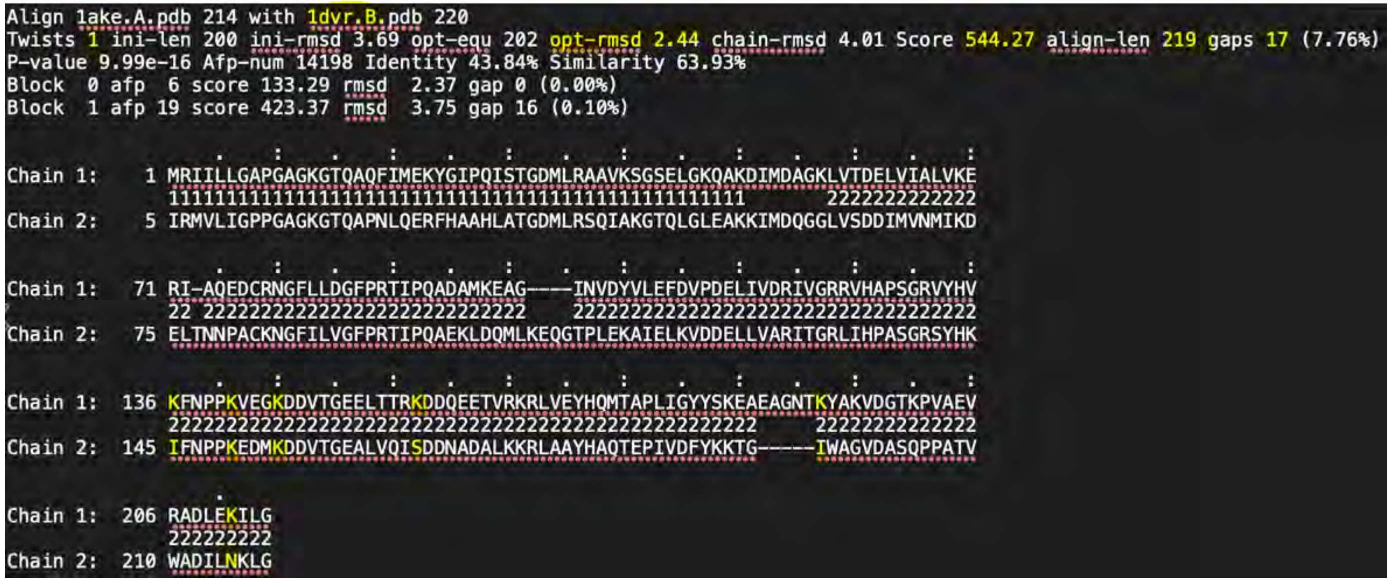

```
Align 1ake.A.pdb 214 with 1dvr.B.pdb 220
Twists 1 ini-len 200 ini-rmsd 3.69 opt-equ 202 opt-rmsd 2.44 chain-rmsd 4.01 Score 544.27 align-len 219 gaps 17 (7.76%)
P-value 9.99e-16 Afp-num 14198 Identity 43.84% Similarity 63.93%
Block  0 afp  6 score 133.29 rmsd  2.37 gap 0 (0.00%)
Block  1 afp 19 score 423.37 rmsd  3.75 gap 16 (0.10%)

                 .    :    .    :    .    :    .    :    .    :    .    :    .    :
Chain 1:    1 MRIILLGAPGAGKGTQAQFIMEKYGIPQISTGDMLRAAVKSGSELGKQAKDIMDAGKLVTDELVIALVKE
              11111111111111111111111111111111111111111111111111       222222222222
Chain 2:    5 IRMVLIGPPGAGKGTQAPNLQERFHAAHLATGDMLRSQIAKGTQLGLEAKKIMDQGGLVSDDIMVNMIKD

                 .    :    .    :    .    :    .    :    .    :    .    :    .    :
Chain 1:   71 RI-AQEDCRNGFLLDGFPRTIPQADAMKEAG----INVDYVLEFDVPDELIVDRIVGRRVHAPSGRVYHV
              22 222222222222222222222222222    222222222222222222222222222222222222
Chain 2:   75 ELTNNPACKNGFILVGFPRTIPQAEKLDQMLKEQGTPLEKAIELKVDDELLVARITGRLIHPASGRSYHK

                 .    :    .    :    .    :    .    :    .    :    .    :    .    :
Chain 1:  136 KFNPPKVEGKDDVTGEELTTRKDDQEETVRKRLVEYHQMTAPLIGYYSKEAEAGNTKYAKVDGTKPVAEV
              2222222222222222222222222222222222222222222222222222     22222222222222
Chain 2:  145 IFNPPKEDMKDDVTGEALVQISDDNADALKKRLAAYHAQTEPIVDFYKKTG-----IWAGVDASQPPATV

                 .
Chain 1:  206 RADLEKILG
              222222222
Chain 2:  210 WADILNKLG
```

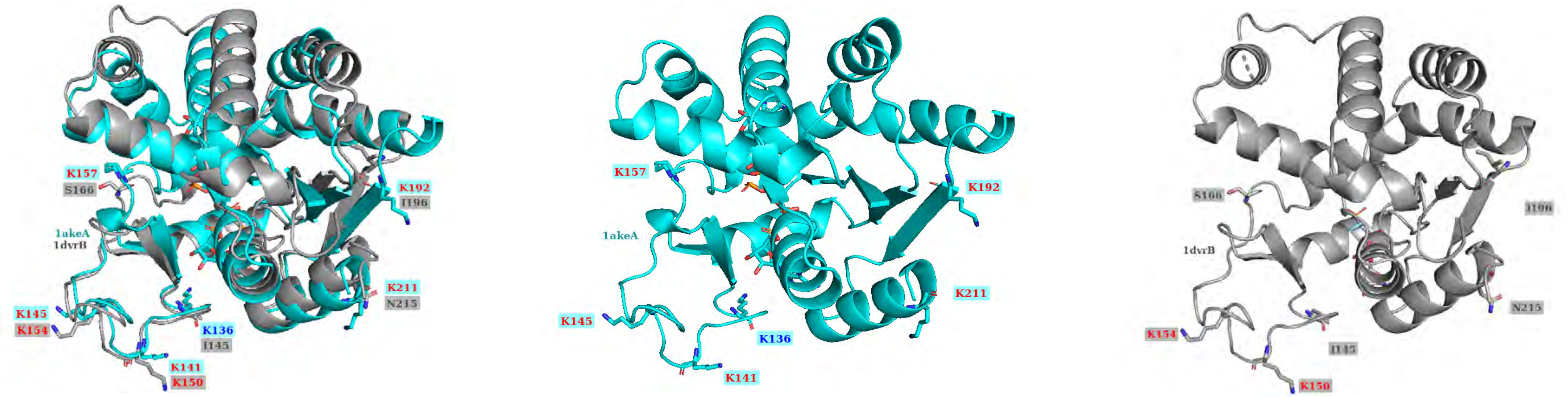

UniProt ID: P07170

PDB ID: 2AKY_A

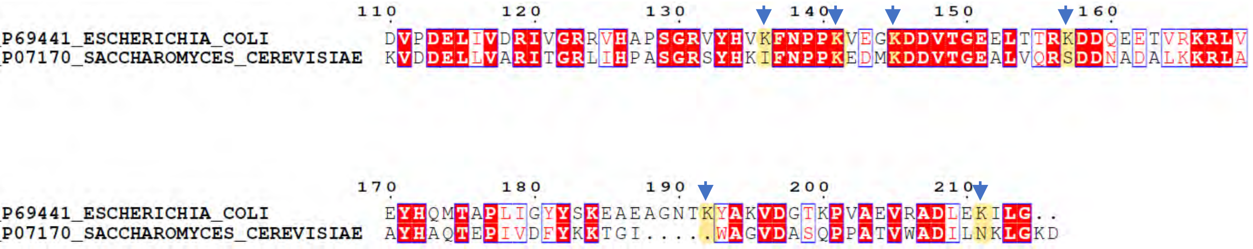


Full sequences in supplemental file.

```
Align 1ake.A.pdb 214 with 2aky.A.pdb 218
Twists 0 ini-len 200 ini-rmsd 1.23 opt-equ 209 opt-rmsd 1.26 chain-rmsd 1.23 Score 554.83 align-len 219 gaps 10 (4.57%)
P-value 0.00e+00 Afp-num 14488 Identity 44.75% Similarity 64.84%
Block  0 afp 25 score 554.83 rmsd  1.23 gap 18 (0.08%)

                .    :    .    :    .    :    .    :    .    :    .    :    .    :
Chain 1:    1 MRIILLGAPGAGKGTQAQFIMEKYGIPQISTGDMLRAAVKSGSELGKQAKDIMDAGKLVTDELVIALVKE
              1111111111111111111111111111111111111111111111111111111111111111111111
Chain 2:    5 IRMVLIGPPGAGKGTQAPNLQERFHAAHLATGDMLRSQIAKGTQLGLEAKKIMDQGGLVSDDIMVNMIKD

                .    :    .    :    .    :    .    :    .    :    .    :    .    :
Chain 1:   71 RIA-QEDCRNGFLLDGFPRTIPQADAMKEAGI----NVDYVLEFDVPDELIVDRIVGRRVHAPSGRVYHV
              111 1111111111111111111111111111    111111111111111111111111111111111
Chain 2:   75 ELTNNPACKNGFILDGFPRTIPQAEKLDQMLKEQGTPLEKAIELKVDDELLVARITGRLIHPASGRSYHK

                .    :    .    :    .    :    .    :    .    :    .    :    .    :
Chain 1:  136 KFNPPKVEGKDDVTGEELTTRKDDQEETVRKRLVEYHQMTAPLIGYYSKEAEAGNTKYAKVDGTKPVAEV
              11111111111111111111111111111111111111111111111111111     11111111111111
Chain 2:  145 IFNPPKEDMKDDVTGEALVQRSDDNADALKKRLAAYHAQTEPIVDFYKKTG-----IWAGVDASQPPATV

                .
Chain 1:  206 RADLEKILG
              111111111
Chain 2:  210 WADILNKLG

Note: positions are from PDB; the numbers between alignments are block index
```

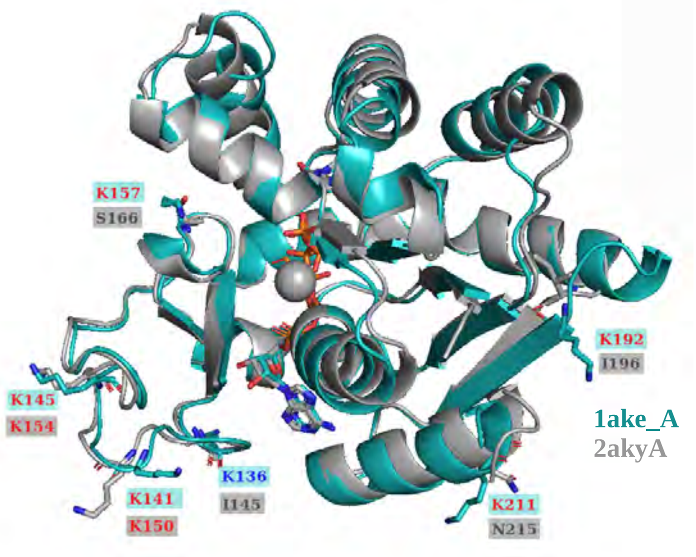


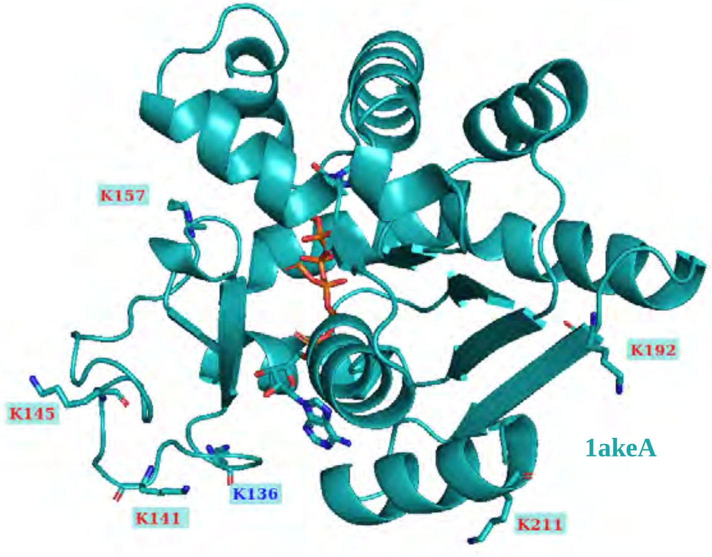


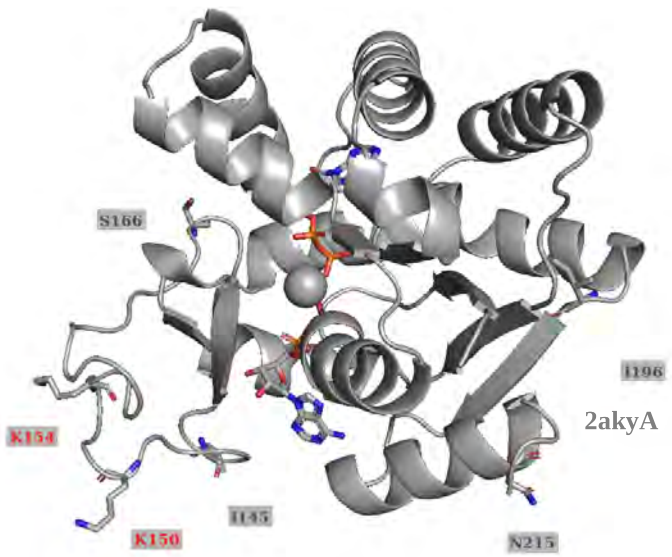

UniProt ID: P07170

PDB ID: 3AKY_A

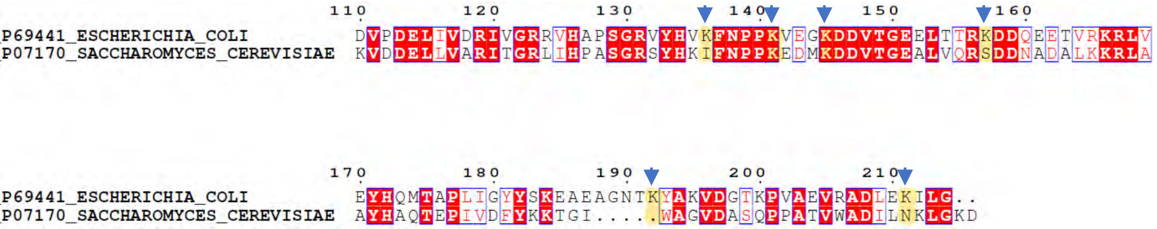


Full sequences in supplemental file.

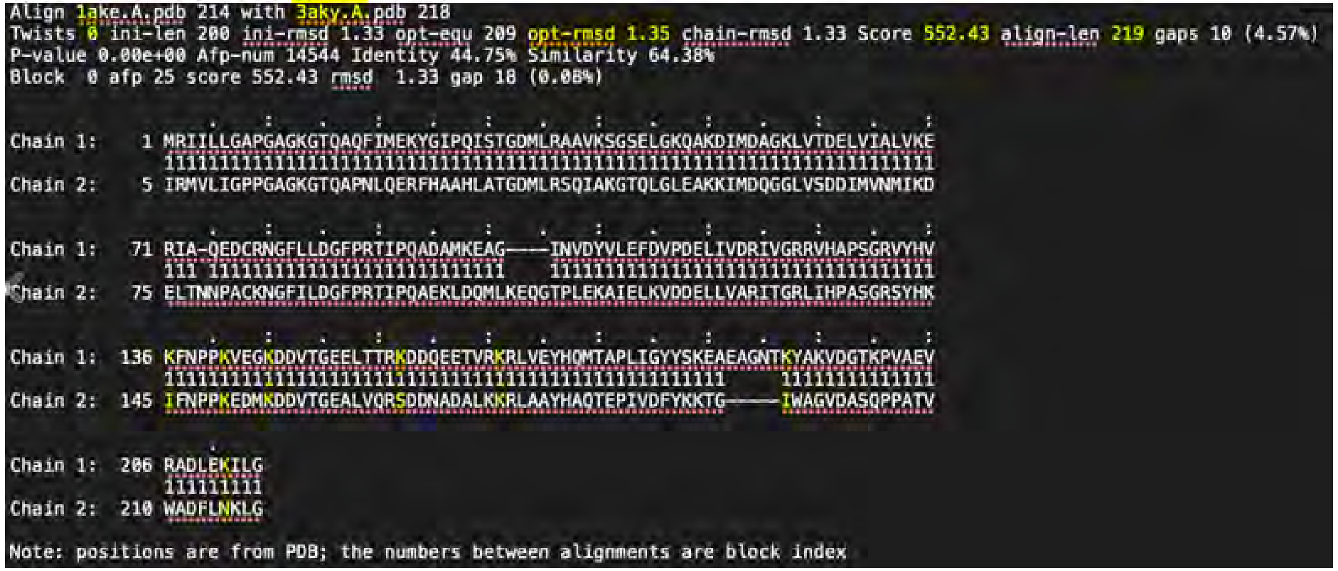


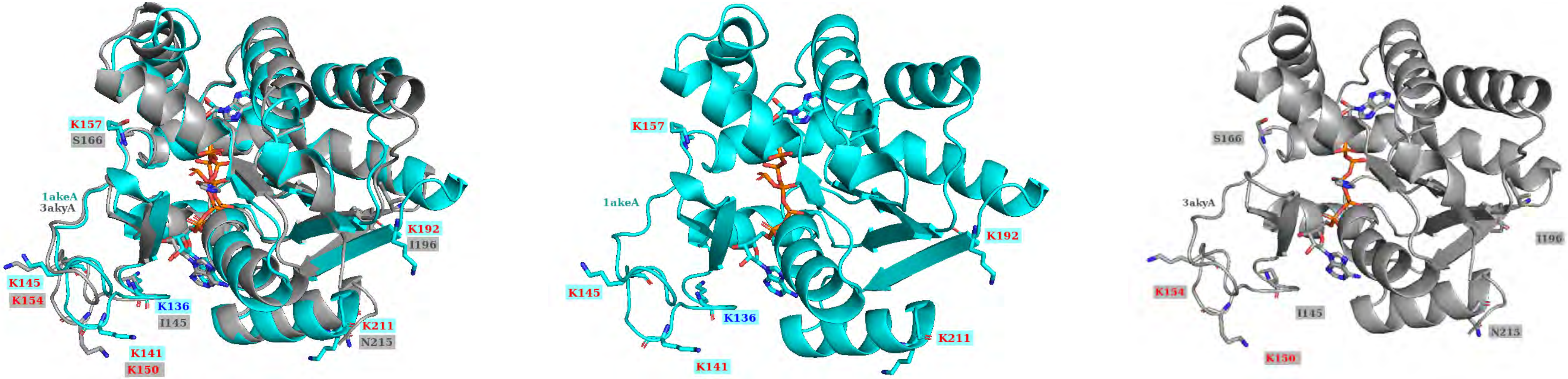

UniProt ID: P08166

PDB ID: 1AK2_A

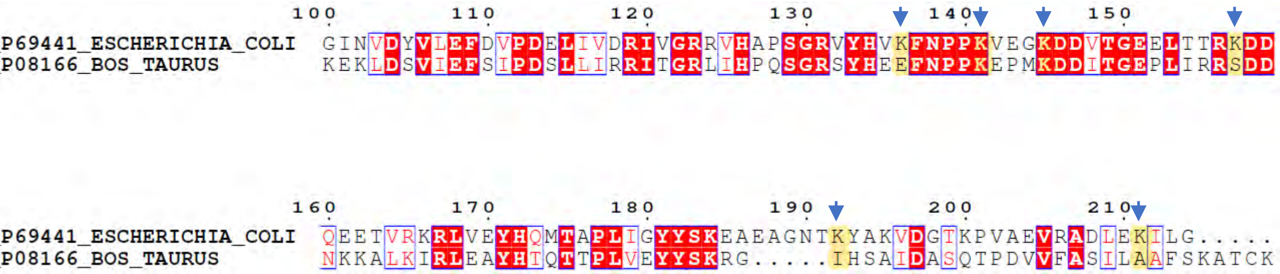


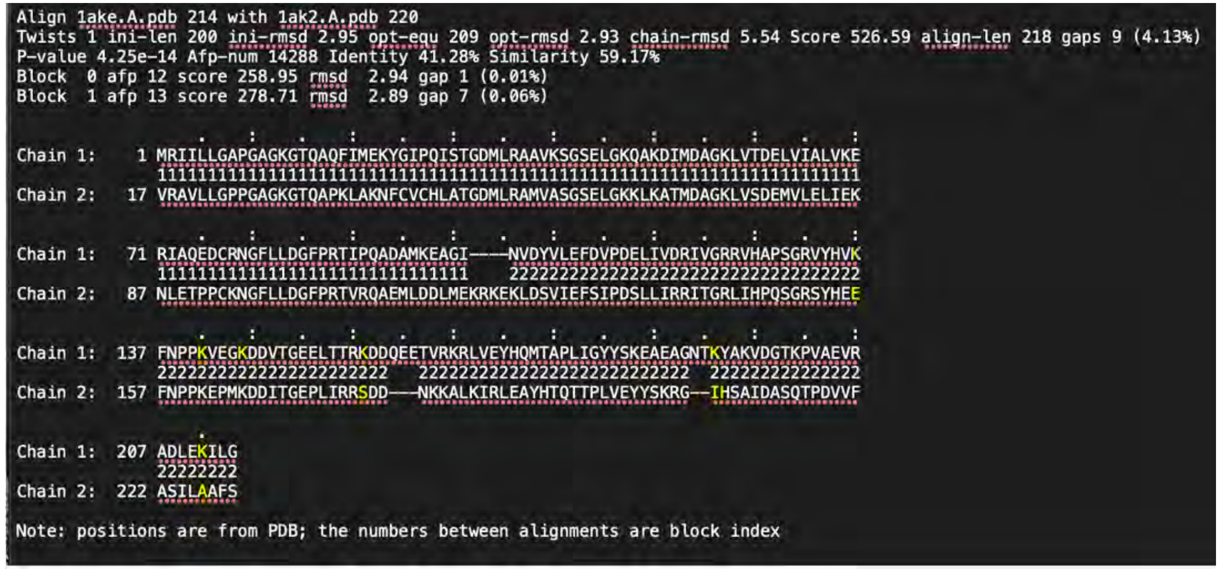


Full sequences in supplemental file.

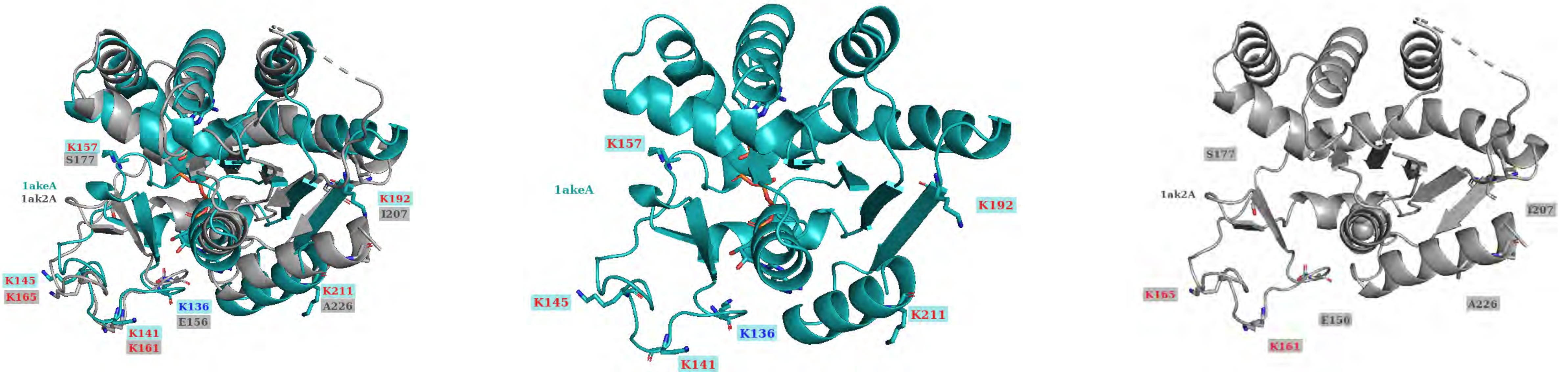

UniProt ID: P08166

PDB ID: 2AK2_A

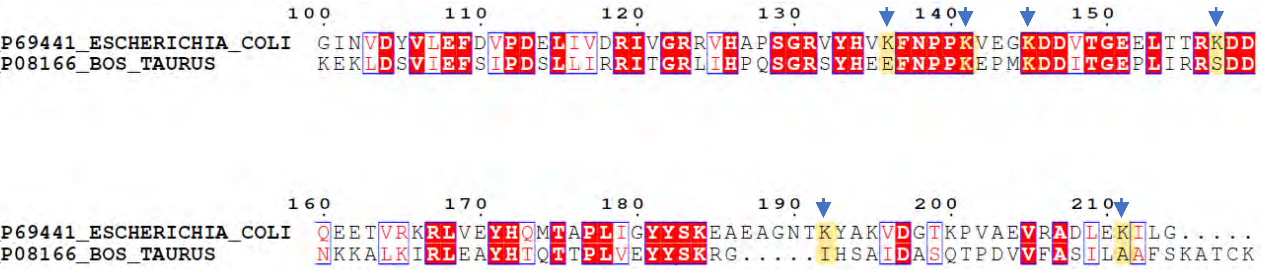


Full sequences in supplemental file.

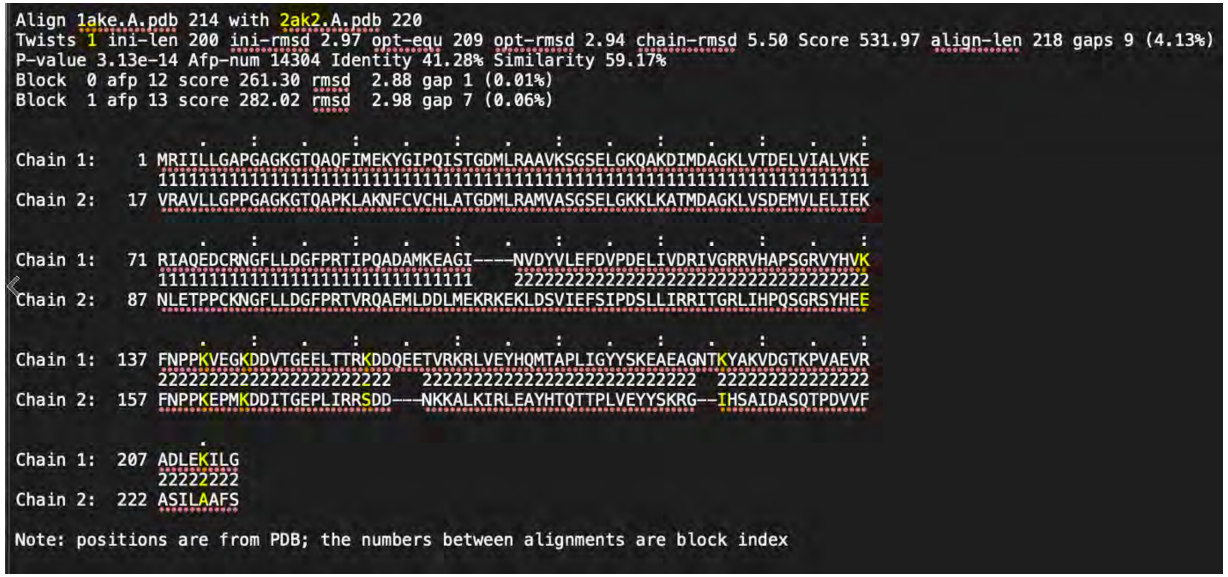

```
Align 1ake.A.pdb 214 with 2ak2.A.pdb 220
Twists 1 ini-len 200 ini-rmsd 2.97 opt-equ 209 opt-rmsd 2.94 chain-rmsd 5.50 Score 531.97 align-len 218 gaps 9 (4.13%)
P-value 3.13e-14 Afp-num 14304 Identity 41.28% Similarity 59.17%
Block  0 afp 12 score 261.30 rmsd  2.88 gap 1 (0.01%)
Block  1 afp 13 score 282.02 rmsd  2.98 gap 7 (0.06%)

                  .    :    .    :    .    :    .    :    .    :    .    :    .    :
Chain 1:    1 MRIILLGAPGAGKGTQAQFIMEKYGIPQISTGDMLRAAVKSGSELGKQAKDIMDAGKLVTDELVIALVKE
              1111111111111111111111111111111111111111111111111111111111111111111111
Chain 2:   17 VRAVLLGPPGAGKGTQAPKLAKNFCVCHLATGDMLRAMVASGSELGKKLKATMDAGKLVSDEMVLELIEK

                  .    :    .    :    .    :    .    :    .    :    .    :    .    :
Chain 1:   71 RIAQEDCRNGFLLDGFPRTIPQADAMKEAGI----NVDYVLEFDVPDELIVDRIVGRRVHAPSGRVYHVK
              1111111111111111111111111111111    22222222222222222222222222222222222
Chain 2:   87 NLETPPCKNGFLLDGFPRTVRQAEMLDDLMEKRKEKLDSVIEFSIPDSLLIRRITGRLIHPQSGRSYHEE

                  .    :    .    :    .    :    .    :    .    :    .    :    .    :
Chain 1:  137 FNPPKVEGKDDVTGEELTTRKDDQEETVRKRLVEYHQMTAPLIGYYSKEAEAGNTKYAKVDGTKPVAEVR
              22222222222222222222222   222222222222222222222222222  222222222222222
Chain 2:  157 FNPPKEPMKDDITGEPLIRRSDD---NKKALKIRLEAYHTQTTPLVEYYSKRG--IHSAIDASQTPDVVF

                  .
Chain 1:  207 ADLEKILG
              22222222
Chain 2:  222 ASILAAFS

Note: positions are from PDB; the numbers between alignments are block index
```

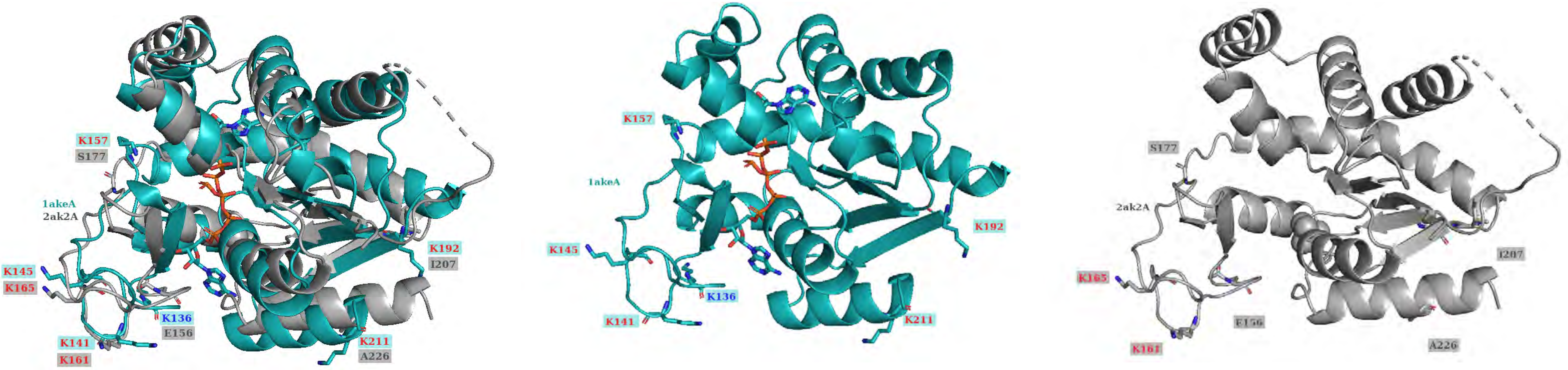

UniProt ID: P08760

PDB ID: 2AK3_A

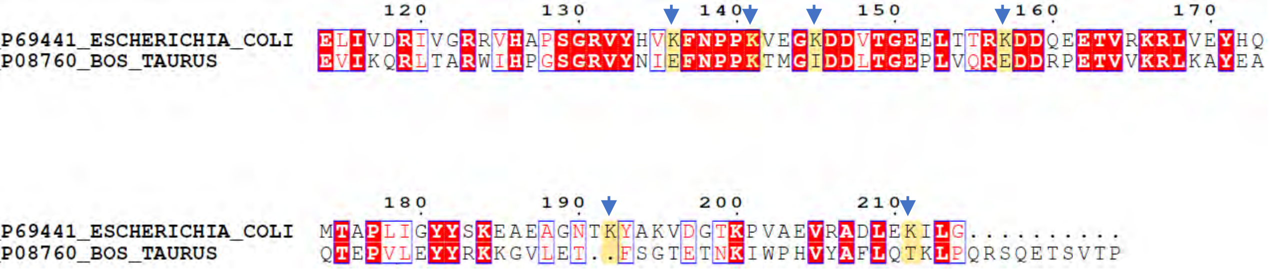


```
Align 1ake.A.pdb 214 with 2ak3.A.pdb 226
Twists 2 ini-len 192 ini-rmsd 3.25 opt-equ 202 opt-rmsd 1.54 chain-rmsd 6.97 Score 489.70 align-len 214 gaps 12 (5.61%)
P-value 2.23e-14 Afp-num 14798 Identity 39.25% Similarity 59.35%
Block  0 afp 14 score 292.13 rmsd  1.69 gap 7 (0.06%)
Block  1 afp  4 score 95.13 rmsd  0.76 gap 0 (0.00%)
Block  2 afp  6 score 127.30 rmsd  2.73 gap 9 (0.16%)

                  .    :    .    :    .    :    .    :    .    :    .    :    .    :
Chain 1:    1 MRIILLGAPGAGKGTQAQFIMEKYGIPQISTGDMLRAAVKSGSELGKQAKDIMDAGKLVTDELVIALVKE
              1111111111111111111111111111111111111111111111111111111111111111111111
Chain 2:    6 LRAAIMGAPGSGKGTVSSRITKHFELKHLSSGDLLRDNMLRGTEIGVLAKTFIDQGKLIPDDVMTRLVLH

                  .    :    .    :    .    :    .    :    .    :    .    :    .    :
Chain 1:   71 RIAQEDCRNGFLLDGFPRTIPQADAMKEAGINVDYVLEFDVPDELIVDRIVGRRVHAPSGRVYHVKFNPP
              1111 1111111111111111111111111 1111111111111111111112222222222222222222
Chain 2:   76 ELKN-LTQYNWLLDGFPRTLPQAEALDRA-YQIDTVINLNVPFEVIKQRLTARWIHPGSGRVYNIEFNPP

                  .    :    .    :    .    :    .    :    .    :    .    :    .    :
Chain 1:  141 KVEGKDDVTGEELTTRKDDQEETVRKRLVEYHQMTAPLIGYYSKEAEAGNTKYAKVDGTKPVAEVRADLE
              2222222222222    333333333333333333333333333333     33333333 3333333333
Chain 2:  144 KTMGIDDLTGEPLVQREDDRPETVVKRLKAYEAQTEPVLEYYRKKG-----VLETFSGT-ETNKIWPHVY

Chain 1:  211 KILG
              3333
Chain 2:  208 AFLQ

Note: positions are from PDB; the numbers between alignments are block index
```

Full sequences in supplemental file.

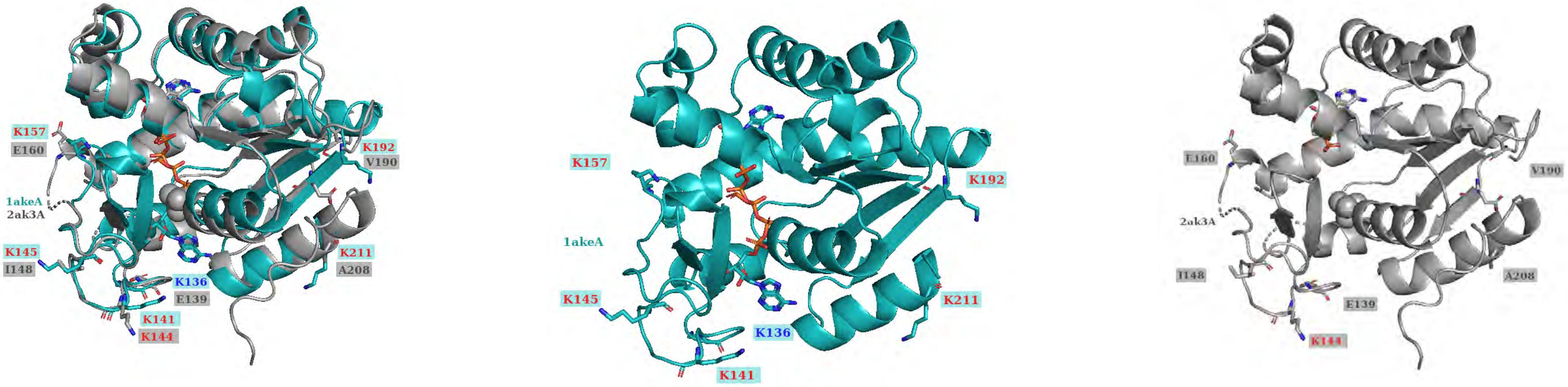

UniProt ID: P16304

PDB ID: 1P3J_A

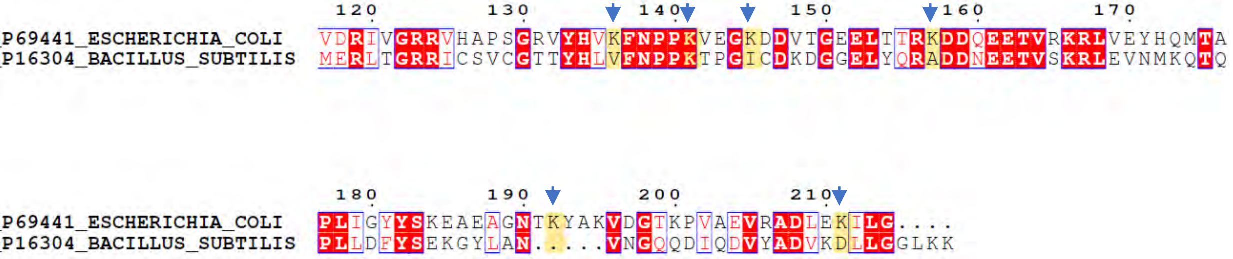


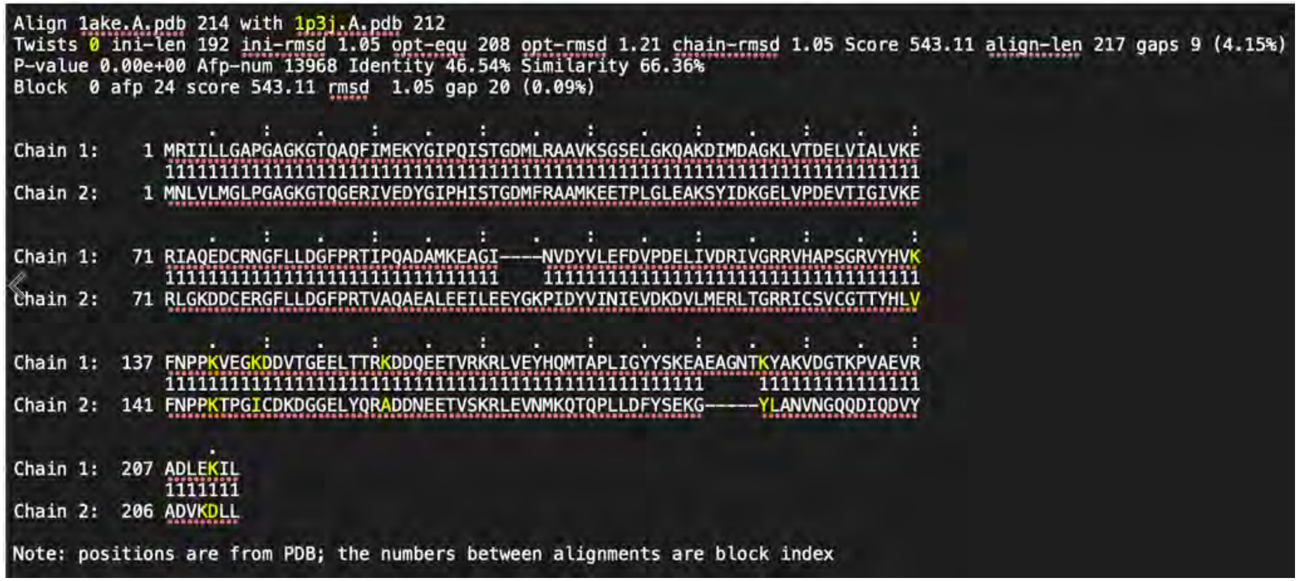

```
Align 1ake.A.pdb 214 with 1p3j.A.pdb 212
Twists 0 ini-len 192 ini-rmsd 1.05 opt-equ 208 opt-rmsd 1.21 chain-rmsd 1.05 Score 543.11 align-len 217 gaps 9 (4.15%)
P-value 0.00e+00 Afp-num 13968 Identity 46.54% Similarity 66.36%
Block  0 afp 24 score 543.11 rmsd  1.05 gap 20 (0.09%)

Chain 1:    1 MRIILLGAPGAGKGTQAQFIMEKYGIPQISTGDMLRAAVKSGSELGKQAKDIMDAGKLVTDELVIALVKE
Chain 2:    1 MNLVLMGLPGAGKGTQGERIVEDYGIPHISTGDMFRAAMKEETPLGLEAKSYIDKGELVPDEVTIGIVKE

Chain 1:   71 RIAQEDCRNGFLLDGFPRTIPQADAMKEAGI----NVDYVLEFDVPDELIVDRIVGRRVHAPSGRVYHVK
Chain 2:   71 RLGKDDCERGFLLDGFPRTVAQAEALEEILEEYGKPIDYVINIEVDKDVLMERLTGRRICSVCGTTYHLV

Chain 1:  137 FNPPKVEGKDDVTGEELTTRKDDQEETVRKRLVEYHQMTAPLIGYYSKEAEAGNTKYAKVDGTKPVAEVR
Chain 2:  141 FNPPKTPGICDKDGGELYQRADDNEETVSKRLEVNMKQTQPLLDFYSEKG------YLANVNGQQDIQDVY

Chain 1:  207 ADLEKIL
Chain 2:  206 ADVKDLL

Note: positions are from PDB; the numbers between alignments are block index
```

Full sequences in supplemental file.

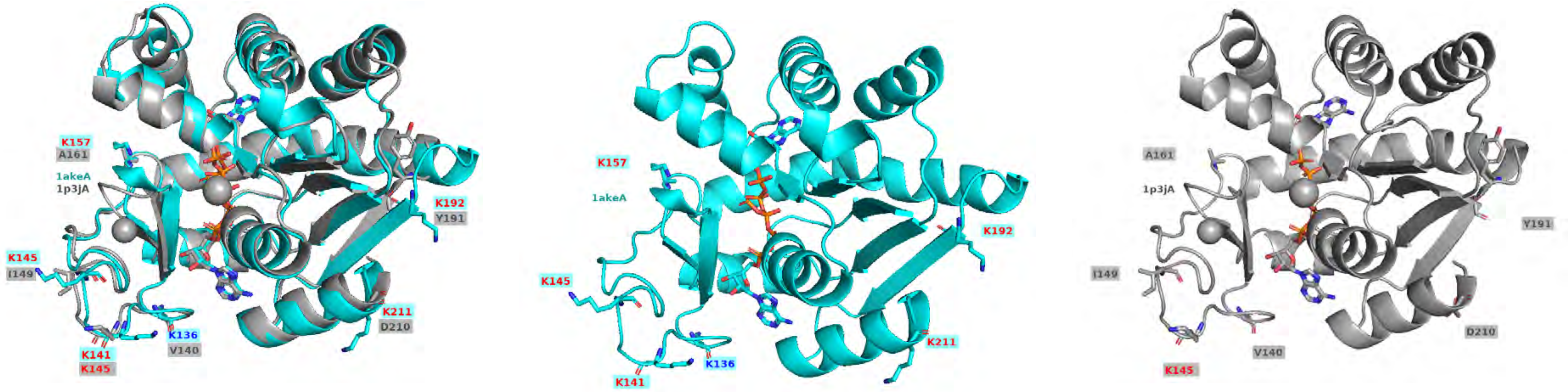

UniProt ID: P16304

PDB ID: 2EU8_B

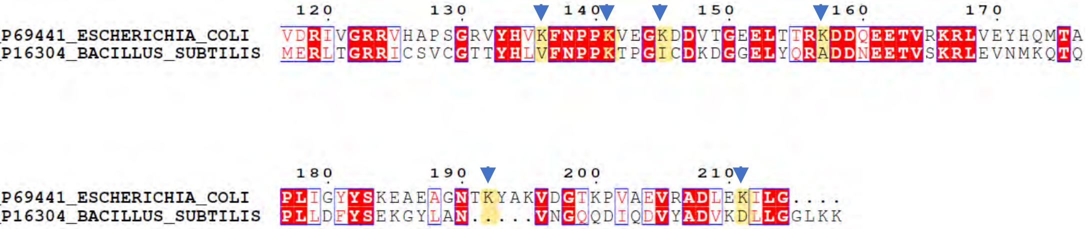


Full sequences in supplemental file.

```
Align 1ake.A.pdb 214 with 2eu8.B.pdb 216
Twists 0 ini-len 200 ini-rmsd 1.27 opt-equ 209 opt-rmsd 1.25 chain-rmsd 1.27 Score 551.23 align-len 218 gaps 9 (4.13%)
P-value 0.00e+00 Afp-num 14401 Identity 46.79% Similarity 66.51%
Block  0 afp 25 score 551.23 rmsd  1.27 gap 17 (0.08%)

Chain 1:    1 MRIILLGAPGAGKGTQAQFIMEKYGIPQISTGDMLRAAVKSGSELGKQAKDIMDAGKLVTDELVIALVKE
                1111111111111111111111111111111111111111111111111111111111111111111111
Chain 2:    1 MNLVLMGLPGAGKGTQGERIVEDYGIPHISTGDMFRAAMKEETPLGLEAKSYIDKGELVPDEVTIGIVKE

Chain 1:   71 RIAQEDCRNGFLLDGFPRTIPQADAMKEAGI----NVDYVLEFDVPDELIVDRIVGRRVHAPSGRVYHVK
                1111111111111111111111111111111    11111111111111111111111111111111111
Chain 2:   71 RLGKDDCERGFLLDGFPRTVAQAEALEEILEEYGKPIDYVINIEVDKDVLMERLTGRRICSVCGTTYHLV

Chain 1:  137 FNPPKVEGKDDVTGEELTTRKDDQEETVRKRLVEYHQMTAPLIGYYSKEAEAGNTKYAKVDGTKPVAEVR
                11111111111111111111111111111111111111111111111111     111111111111111
Chain 2:  141 FNPPKTPGICDKDGGELYQRADDNEETVSKRLEVNMKQTQPLLDFYSEKG-----YLANVNGQRDIQDVY

Chain 1:  207 ADLEKILG
                11111111
Chain 2:  206 ADVKDLLG
```

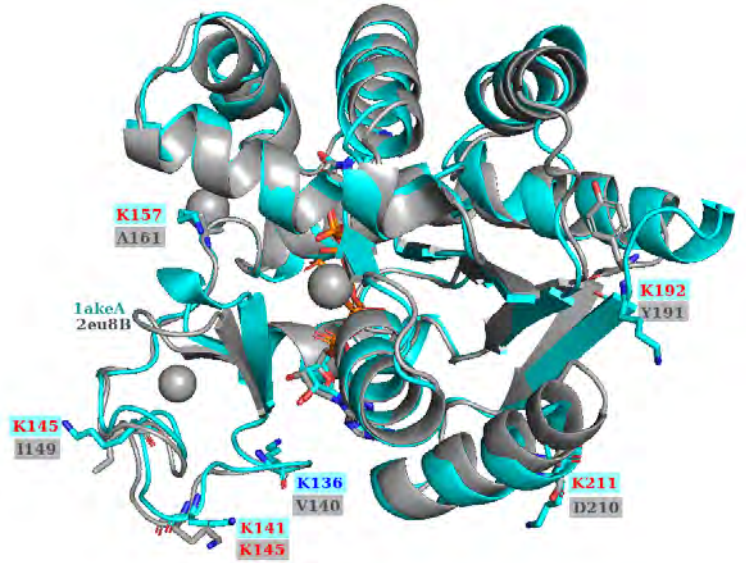


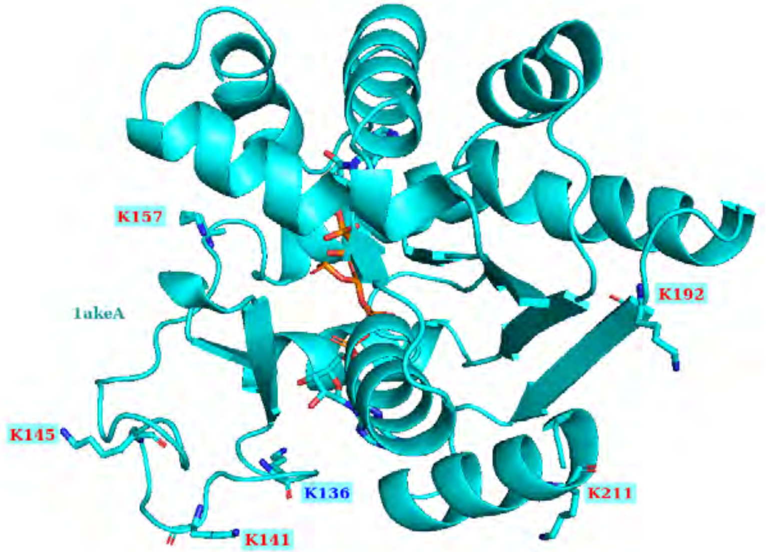


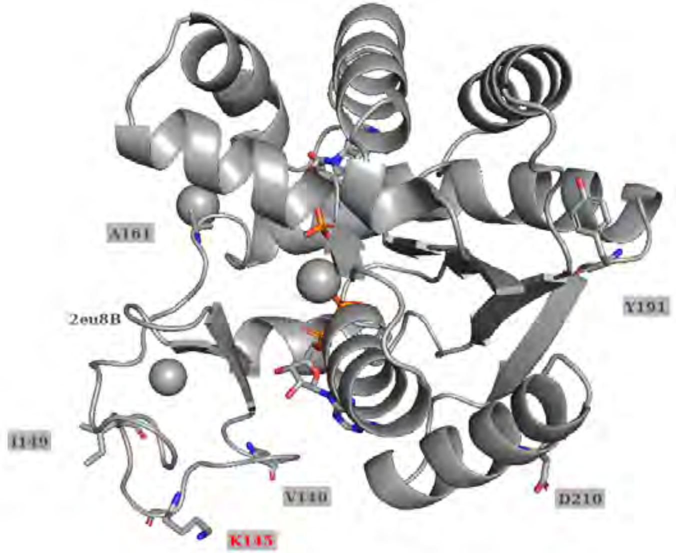

UniProt ID: P16304

PDB ID: 2OO7_B

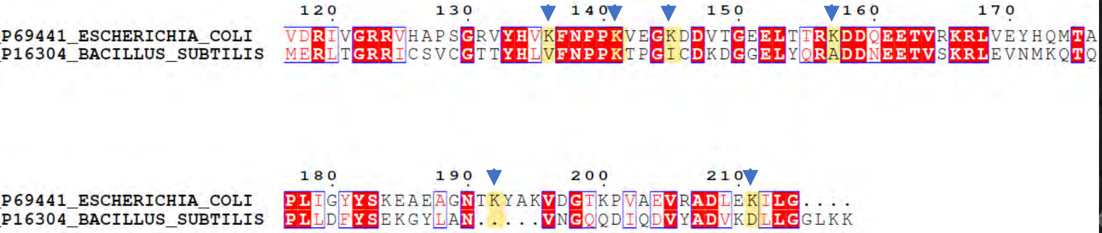


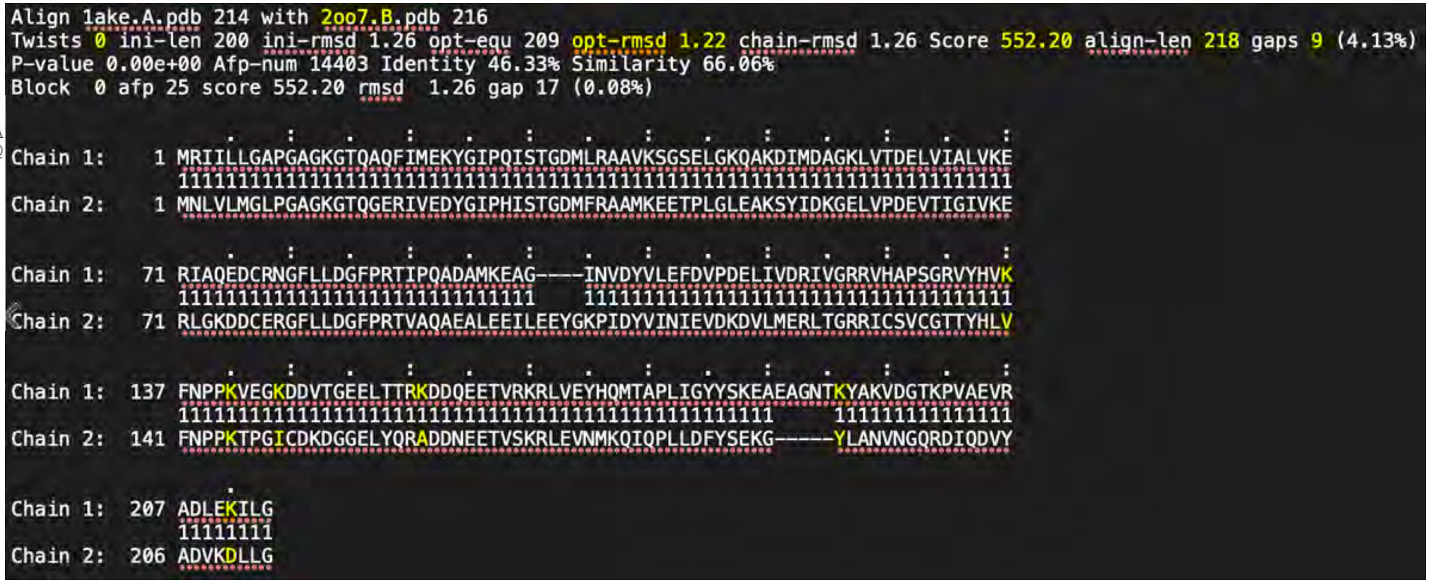

```
Align 1ake.A.pdb 214 with 2oo7.B.pdb 216
Twists 0 ini-len 200 ini-rmsd 1.26 opt-equ 209 opt-rmsd 1.22 chain-rmsd 1.26 Score 552.20 align-len 218 gaps 9 (4.13%)
P-value 0.00e+00 Afp-num 14403 Identity 46.33% Similarity 66.06%
Block  0 afp 25 score 552.20 rmsd  1.26 gap 17 (0.08%)

                .    :    .    :    .    :    .    :    .    :    .    :    .    :
Chain 1:    1 MRIILLGAPGAGKGTQAQFIMEKYGIPQISTGDMLRAAVKSGSELGKQAKDIMDAGKLVTDELVIALVKE
              1111111111111111111111111111111111111111111111111111111111111111111111
Chain 2:    1 MNLVLMGLPGAGKGTQGERIVEDYGIPHISTGDMFRAAMKEETPLGLEAKSYIDKGELVPDEVTIGIVKE

                .    :    .    :    .    :    .    :    .    :    .    :    .    :
Chain 1:   71 RIAQEDCRNGFLLDGFPRTIPQADAMKEAG----INVDYVLEFDVPDELIVDRIVGRRVHAPSGRVYHVK
              111111111111111111111111111111    111111111111111111111111111111111111
Chain 2:   71 RLGKDDCERGFLLDGFPRTVAQAEALEEILEEYGKPIDYVINIEVDKDVLMERLTGRRICSVCGTTYHLV

                .    :    .    :    .    :    .    :    .    :    .    :    .    :
Chain 1:  137 FNPPKVEGKDDVTGEELTTRKDDQEETVRKRLVEYHQMTAPLIGYYSKEAEAGNTKYAKVDGTKPVAEVR
              11111111111111111111111111111111111111111111111111     111111111111111
Chain 2:  141 FNPPKTPGICDKDGGELYQRADDNEETVSKRLEVNMKQIQPLLDFYSEKG-----YLANVNGQRDIQDVY

                .
Chain 1:  207 ADLEKILG
              11111111
Chain 2:  206 ADVKDLLG
```

Full sequences in supplemental file.

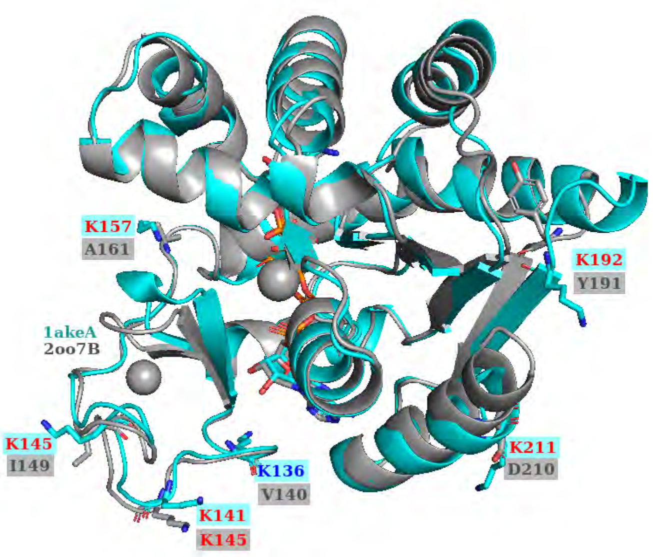


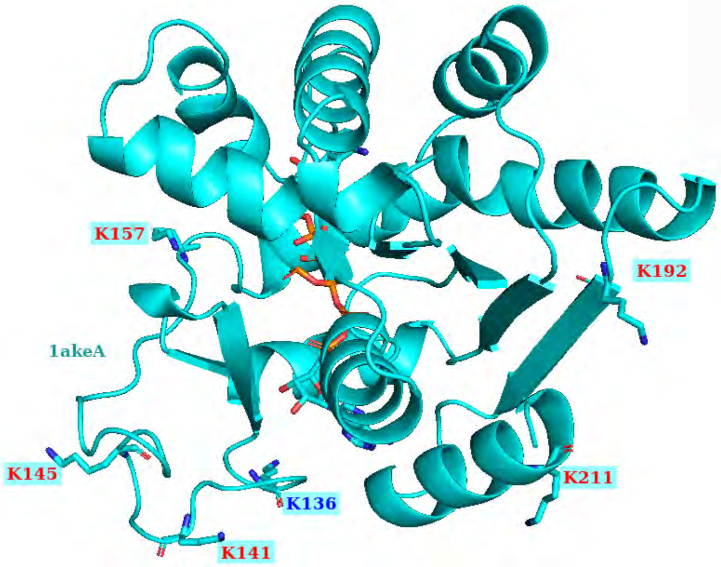


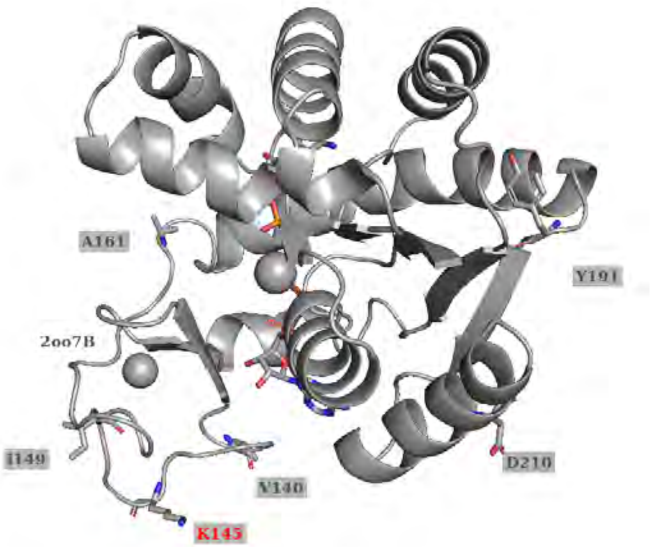

UniProt ID: P16304

PDB ID: 2ORI_B

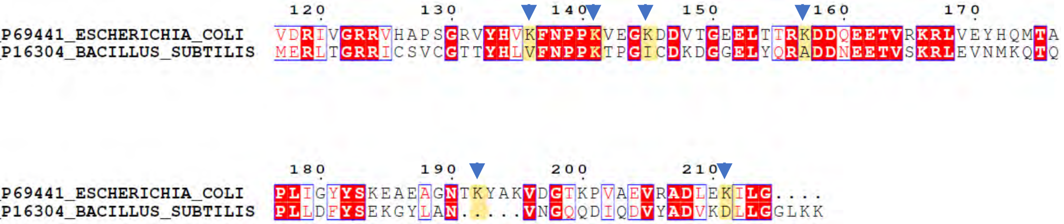


Full sequences in supplemental file.

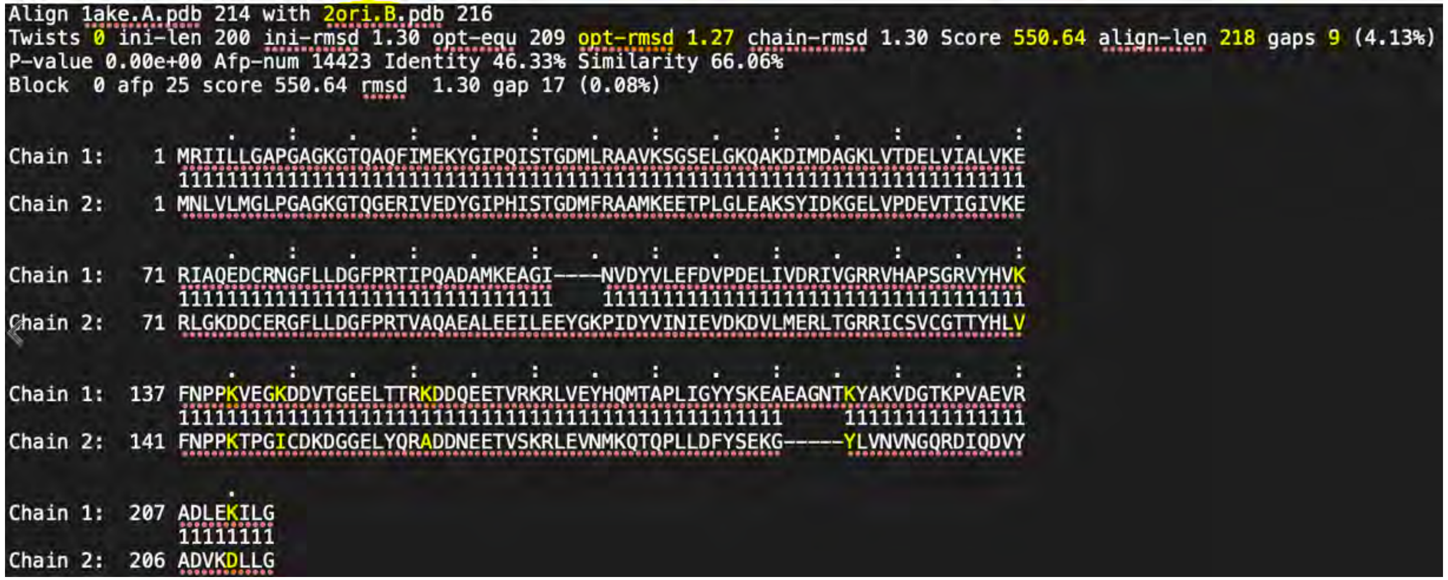


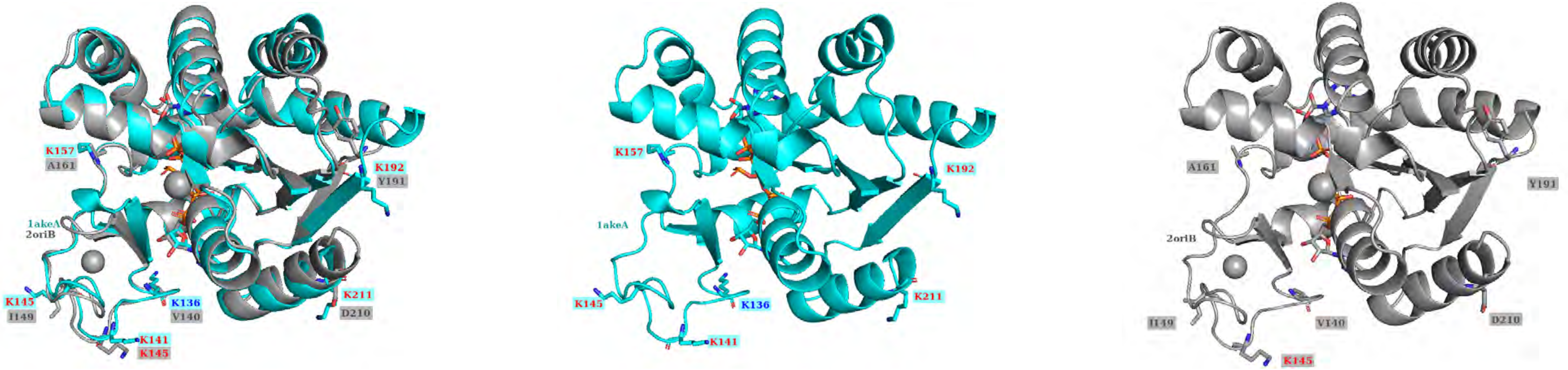

UniProt ID: P16304

PDB ID: 2OSB_B

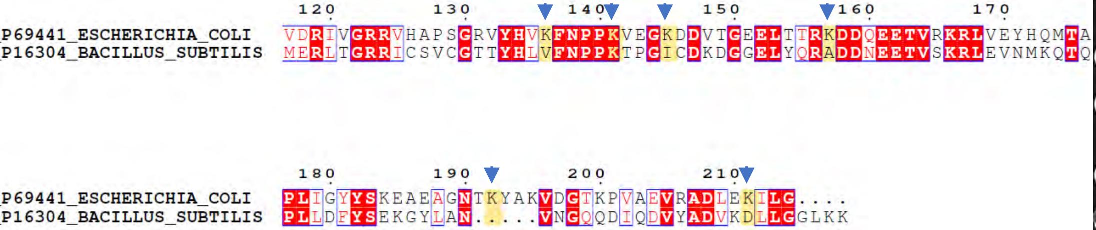


Full sequences in supplemental file.

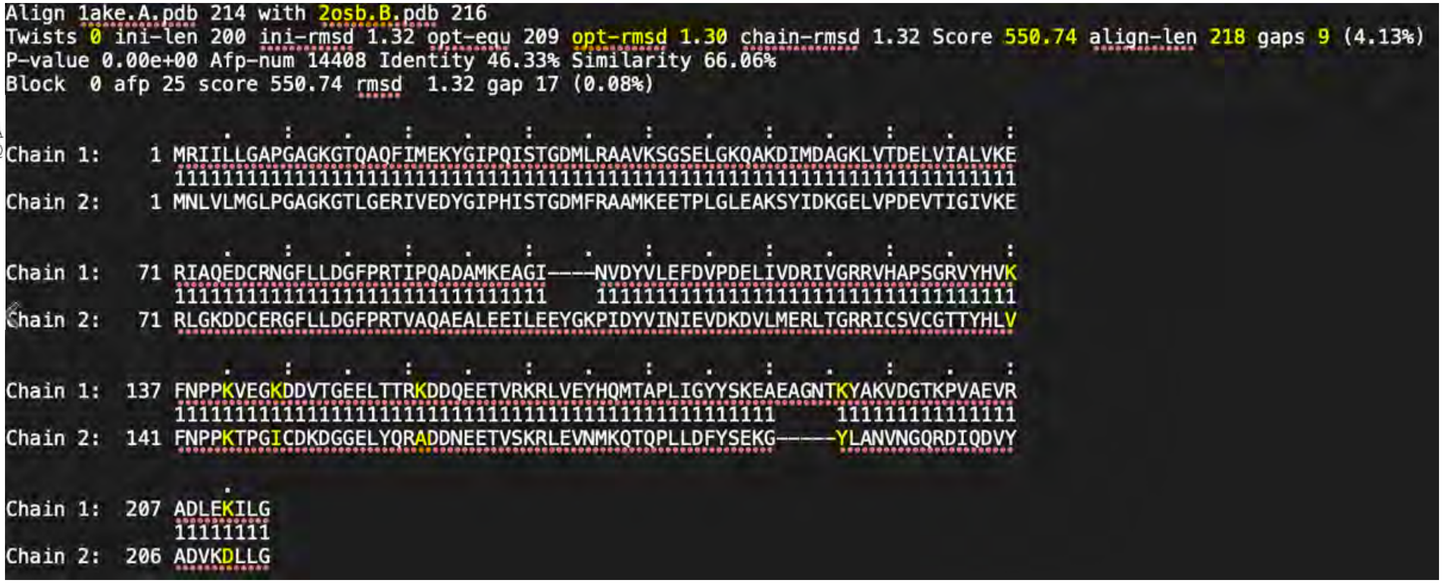

```
Align 1ake.A.pdb 214 with 2osb.B.pdb 216
Twists 0 ini-len 200 ini-rmsd 1.32 opt-equ 209 opt-rmsd 1.30 chain-rmsd 1.32 Score 550.74 align-len 218 gaps 9 (4.13%)
P-value 0.00e+00 Afp-num 14408 Identity 46.33% Similarity 66.06%
Block  0 afp 25 score 550.74 rmsd  1.32 gap 17 (0.08%)

                .    :    .    :    .    :    .    :    .    :    .    :    .    :
Chain 1:    1 MRIILLGAPGAGKGTQAQFIMEKYGIPQISTGDMLRAAVKSGSELGKQAKDIMDAGKLVTDELVIALVKE
              1111111111111111111111111111111111111111111111111111111111111111111111
Chain 2:    1 MNLVLMGLPGAGKGTLGERIVEDYGIPHISTGDMFRAAMKEETPLGLEAKSYIDKGELVPDEVTIGIVKE

                .    :    .    :    .    :    .    :    .    :    .    :    .    :
Chain 1:   71 RIAQEDCRNGFLLDGFPRTIPQADAMKEAGI----NVDYVLEFDVPDELIVDRIVGRRVHAPSGRVYHVK
              1111111111111111111111111111111    11111111111111111111111111111111111
Chain 2:   71 RLGKDDCERGFLLDGFPRTVAQAEALEEILEEYGKPIDYVINIEVDKDVLMERLTGRRICSVCGTTYHLV

                .    :    .    :    .    :    .    :    .    :    .    :    .    :
Chain 1:  137 FNPPKVEGKDDVTGEELTTRKDDQEETVRKRLVEYHQMTAPLIGYYSKEAEAGNTKYAKVDGTKPVAEVR
              11111111111111111111111111111111111111111111111111     111111111111111
Chain 2:  141 FNPPKTPGICDKDGGELYQRADDNEETVSKRLEVNMKQTQPLLDFYSEKG-----YLANVNGQRDIQDVY

                .
Chain 1:  207 ADLEKILG
              11111111
Chain 2:  206 ADVKDLLG
```

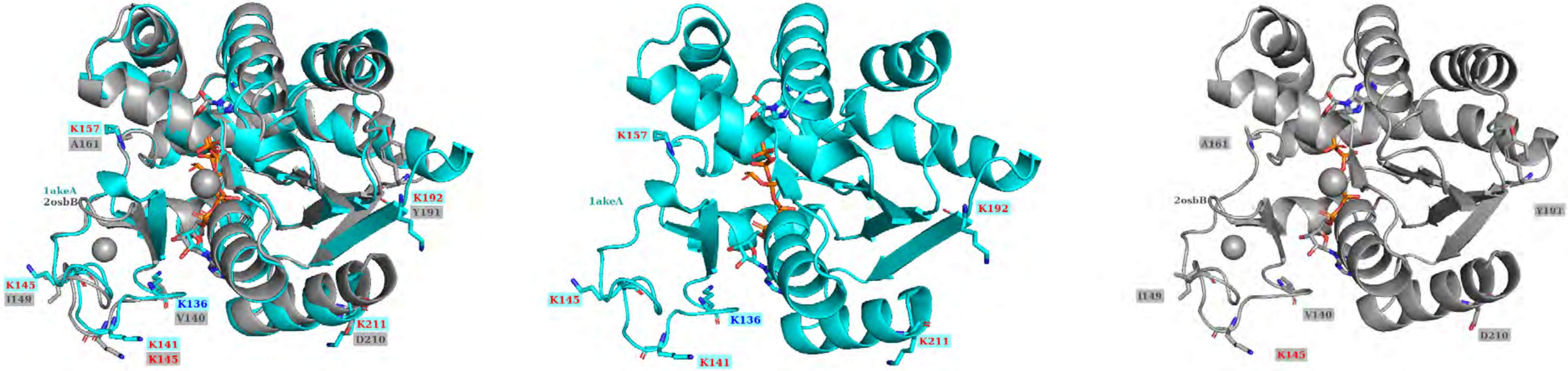

UniProt ID: P16304

PDB ID: 2P3S_A

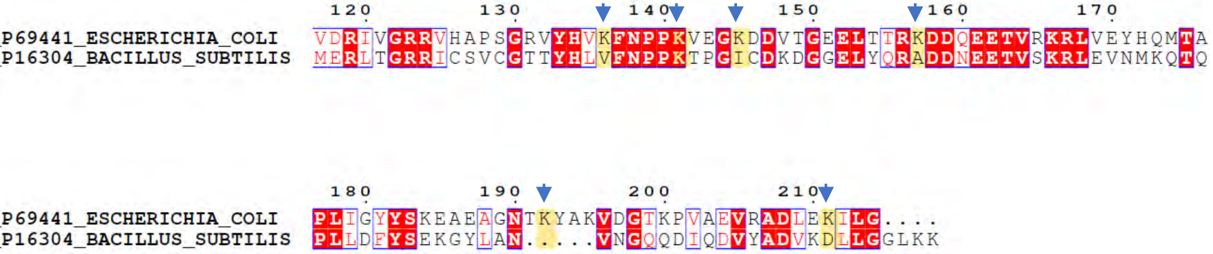


Full sequences in supplemental file.

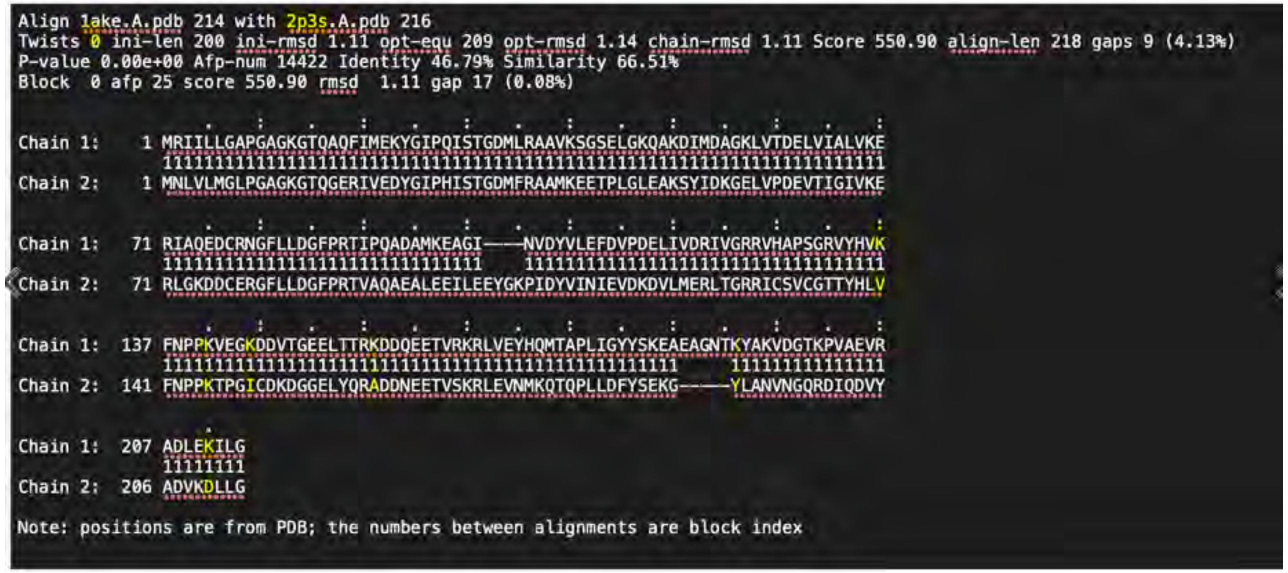

```
Align 1ake.A.pdb 214 with 2p3s.A.pdb 216
Twists 0 ini-len 200 ini-rmsd 1.11 opt-equ 209 opt-rmsd 1.14 chain-rmsd 1.11 Score 550.90 align-len 218 gaps 9 (4.13%)
P-value 0.00e+00 Afp-num 14422 Identity 46.79% Similarity 66.51%
Block  0 afp 25 score 550.90 rmsd  1.11 gap 17 (0.08%)

Chain 1:    1 MRIILLGAPGAGKGTQAQFIMEKYGIPQISTGDMLRAAVKSGSELGKQAKDIMDAGKLVTDELVIALVKE
              11111111111111111111111111111111111111111111111111111111111111111111111
Chain 2:    1 MNLVLMGLPGAGKGTQGERIVEDYGIPHISTGDMFRAAMKEETPLGLEAKSYIDKGELVPDEVTIGIVKE

Chain 1:   71 RIAQEDCRNGFLLDGFPRTIPQADAMKEAGI----NVDYVLEFDVPDELIVDRIVGRRVHAPSGRVYHVK
              1111111111111111111111111111111    11111111111111111111111111111111111
Chain 2:   71 RLGKDDCERGFLLDGFPRTVAQAEALEEILEEYGKPIDYVINIEVDKDVLMERLTGRRICSVCGTTYHLV

Chain 1:  137 FNPPKVEGKDDVTGEELTTRKDDQEETVRKRLVEYHQMTAPLIGYYSKEAEAGNTKYAKVDGTKPVAEVR
              11111111111111111111111111111111111111111111111111      11111111111111
Chain 2:  141 FNPPKTPGICDKDGGELYQRADDNEETVSKRLEVNMKQTQPLLDFYSEKG------YLANVNGQRDIQDVY

Chain 1:  207 ADLEKILG
              11111111
Chain 2:  206 ADVKDLLG

Note: positions are from PDB; the numbers between alignments are block index
```

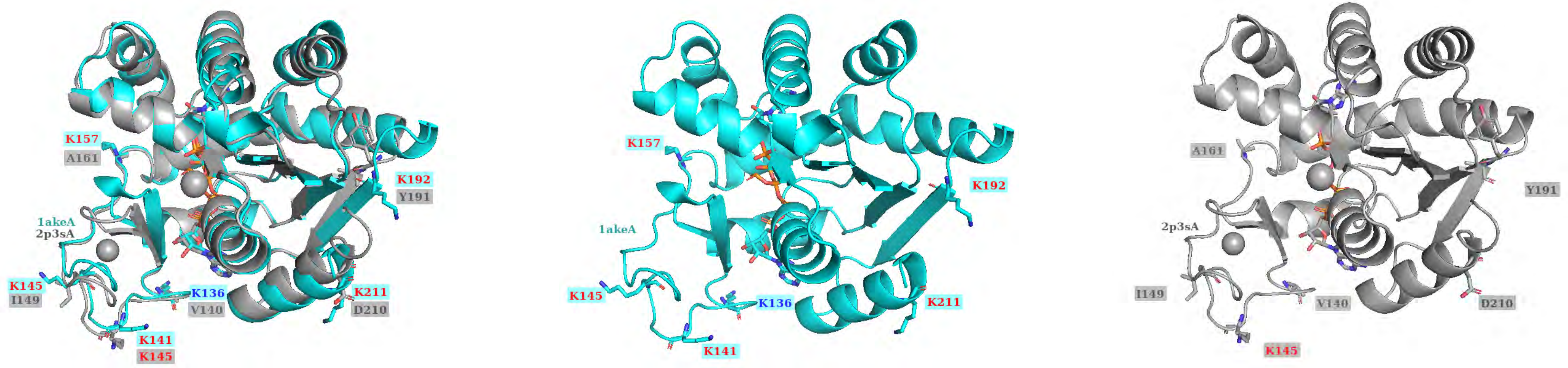

UniProt ID: P16304

PDB ID: 2QAJ_B

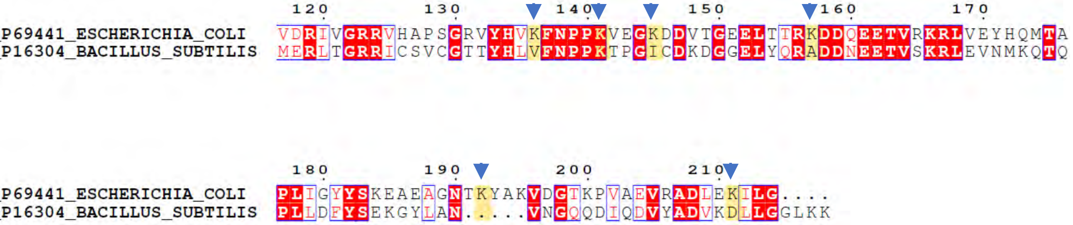


Full sequences in supplemental file.

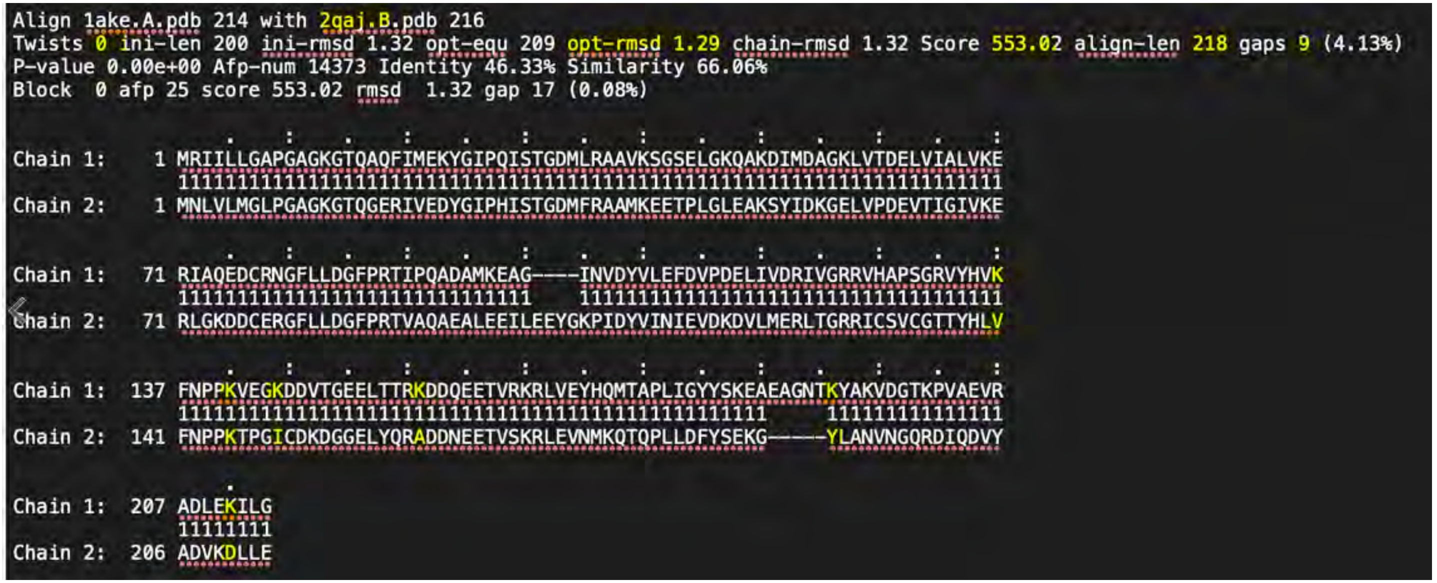


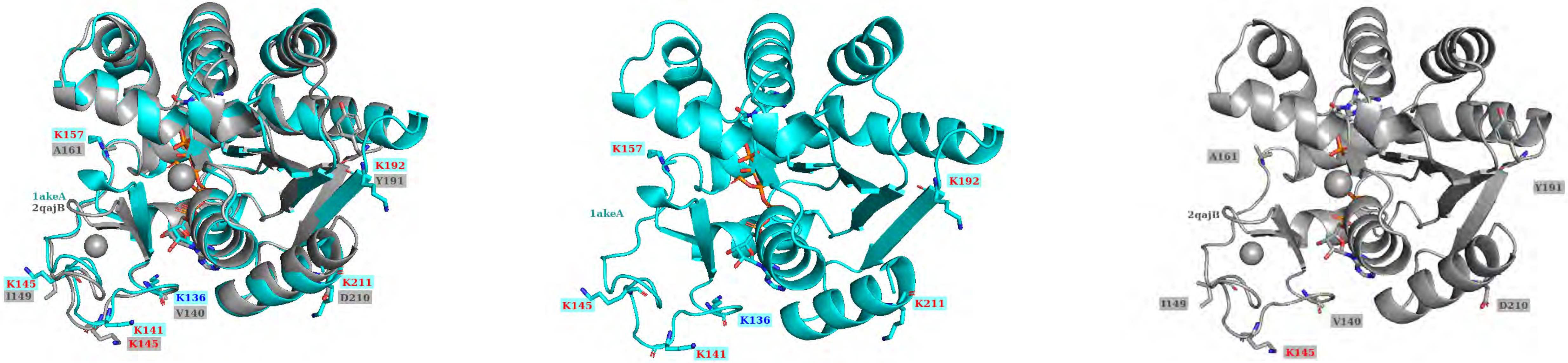

UniProt ID: P16304

PDB ID: 3DKV_A

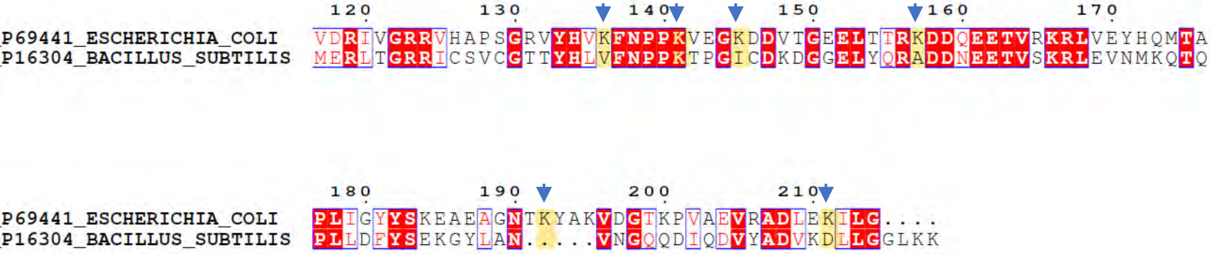


Full sequences in supplemental file.

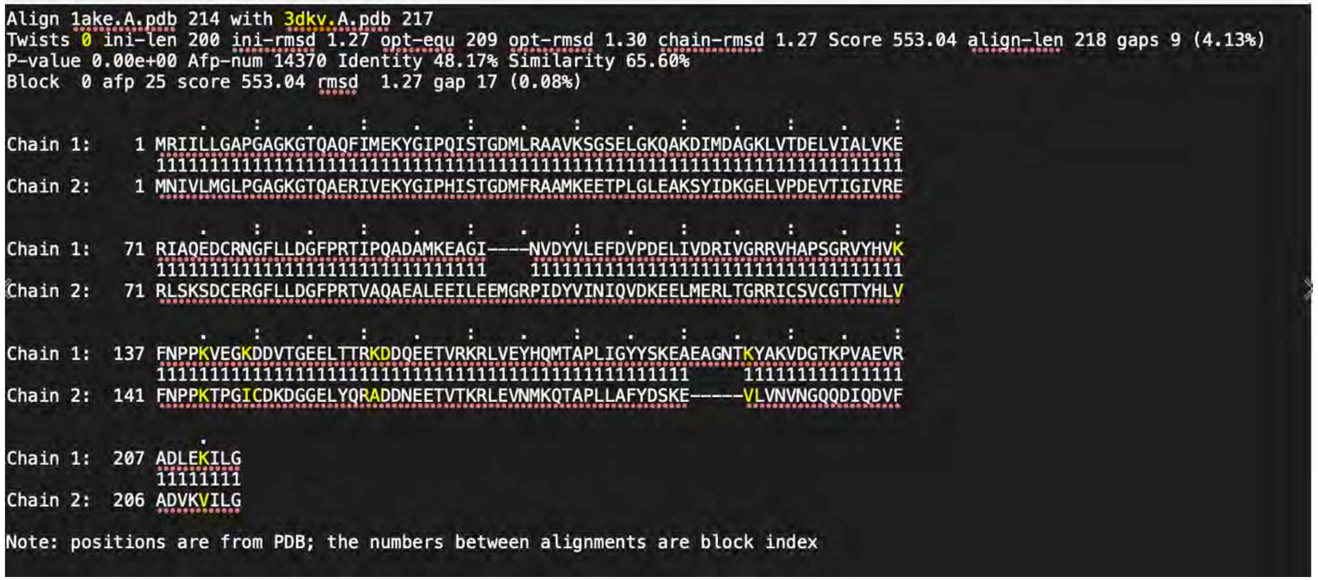

```
Align 1ake.A.pdb 214 with 3dkv.A.pdb 217
Twists 0 ini-len 200 ini-rmsd 1.27 opt-equ 209 opt-rmsd 1.30 chain-rmsd 1.27 Score 553.04 align-len 218 gaps 9 (4.13%)
P-value 0.00e+00 Afp-num 14370 Identity 48.17% Similarity 65.60%
Block  0 afp 25 score 553.04 rmsd  1.27 gap 17 (0.08%)

Chain 1:    1 MRIILLGAPGAGKGTQAQFIMEKYGIPQISTGDMLRAAVKSGSELGKQAKDIMDAGKLVTDELVIALVKE
              1111111111111111111111111111111111111111111111111111111111111111111111
Chain 2:    1 MNIVLMGLPGAGKGTQAERIVEKYGIPHISTGDMFRAAMKEETPLGLEAKSYIDKGELVPDEVTIGIVRE

Chain 1:   71 RIAQEDCRNGFLLDGFPRTIPQADAMKEAGI----NVDYVLEFDVPDELIVDRIVGRRVHAPSGRVYHVK
              1111111111111111111111111111111    11111111111111111111111111111111111
Chain 2:   71 RLSKSDCERGFLLDGFPRTVAQAEALEEILEEMGRPIDYVINIQVDKEELMERLTGRRICSVCGTTYHLV

Chain 1:  137 FNPPKVEGKDDVTGEELTTRKDDQEETVRKRLVEYHQMTAPLIGYYSKEAEAGNTKYAKVDGTKPVAEVR
              11111111111111111111111111111111111111111111111111     11111111111111
Chain 2:  141 FNPPKTPGICDKDGGELYQRADDNEETVTKRLEVNMKQTAPLLAFYDSKE-----VLVNVNGQQDIQDVF

Chain 1:  207 ADLEKILG
              11111111
Chain 2:  206 ADVKVILG

Note: positions are from PDB; the numbers between alignments are block index
```

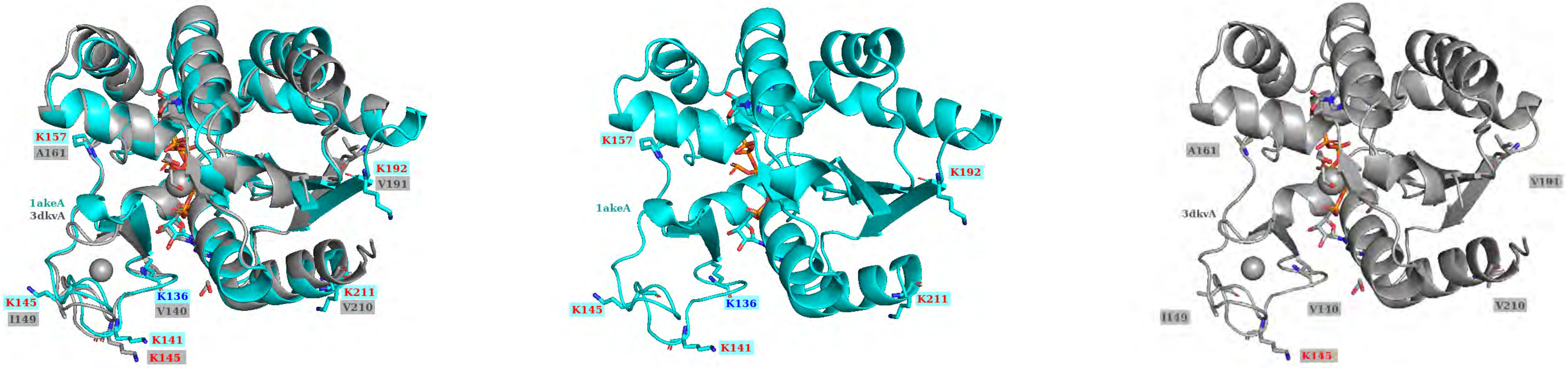

UniProt ID: P16304

PDB ID: 3DL0_A

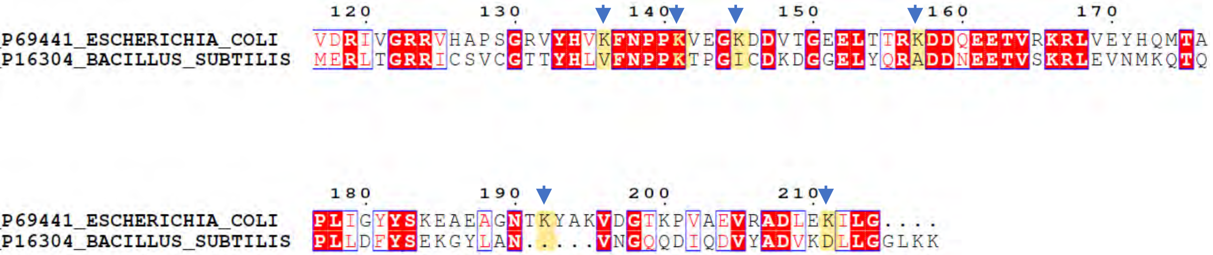


Full sequences in supplemental file.

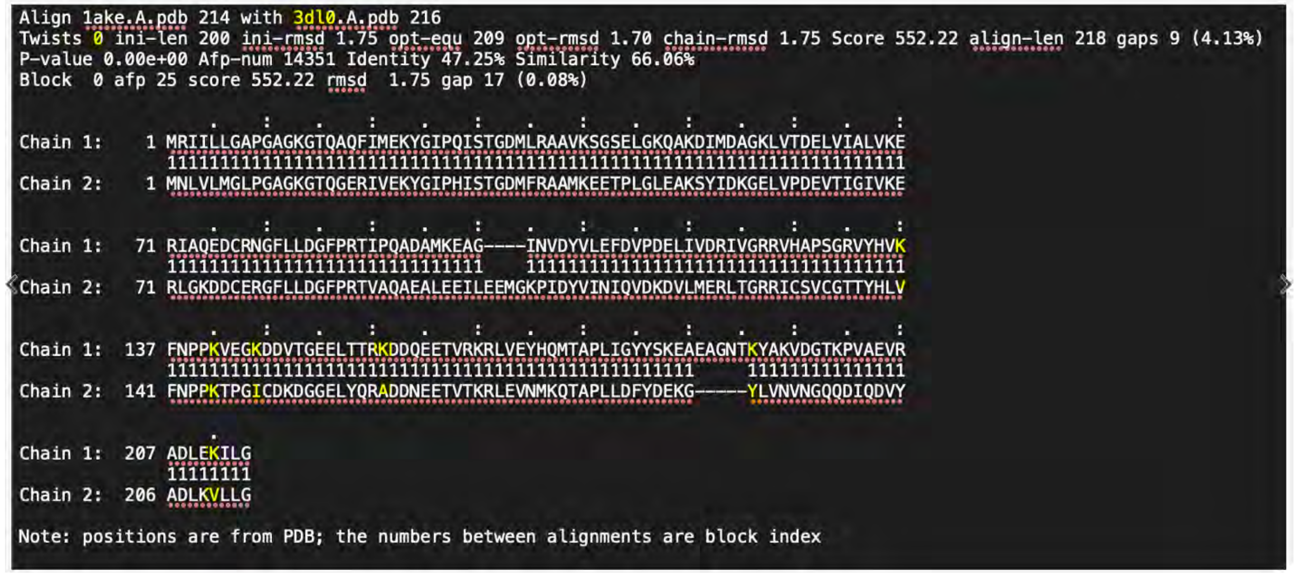

```
Align 1ake.A.pdb 214 with 3dl0.A.pdb 216
Twists 0 ini-len 200 ini-rmsd 1.75 opt-equ 209 opt-rmsd 1.70 chain-rmsd 1.75 Score 552.22 align-len 218 gaps 9 (4.13%)
P-value 0.00e+00 Afp-num 14351 Identity 47.25% Similarity 66.06%
Block  0 afp 25 score 552.22 rmsd  1.75 gap 17 (0.08%)

                .    :    .    :    .    :    .    :    .    :    .    :    .    :
Chain 1:    1 MRIILLGAPGAGKGTQAQFIMEKYGIPQISTGDMLRAAVKSGSELGKQAKDIMDAGKLVTDELVIALVKE
              1111111111111111111111111111111111111111111111111111111111111111111111
Chain 2:    1 MNLVLMGLPGAGKGTQGERIVEKYGIPHISTGDMFRAAMKEETPLGLEAKSYIDKGELVPDEVTIGIVKE

                .    :    .    :    .    :    .    :    .    :    .    :    .    :
Chain 1:   71 RIAQEDCRNGFLLDGFPRTIPQADAMKEAG----INVDYVLEFDVPDELIVDRIVGRRVHAPSGRVYHVK
              111111111111111111111111111111    111111111111111111111111111111111111
Chain 2:   71 RLGKDDCERGFLLDGFPRTVAQAEALEEILEEMGKPIDYVINIQVDKDVLMERLTGRRICSVCGTTYHLV

                .    :    .    :    .    :    .    :    .    :    .    :    .    :
Chain 1:  137 FNPPKVEGKDDVTGEELTTRKDDQEETVRKRLVEYHQMTAPLIGYYSKEAEAGNTKYAKVDGTKPVAEVR
              11111111111111111111111111111111111111111111111111     111111111111111
Chain 2:  141 FNPPKTPGICDKDGGELYQRADDNEETVTKRLEVNMKQTAPLLDFYDEKG-----YLVNVNGQQDIQDVY

                .
Chain 1:  207 ADLEKILG
              11111111
Chain 2:  206 ADLKVLLG

Note: positions are from PDB; the numbers between alignments are block index
```

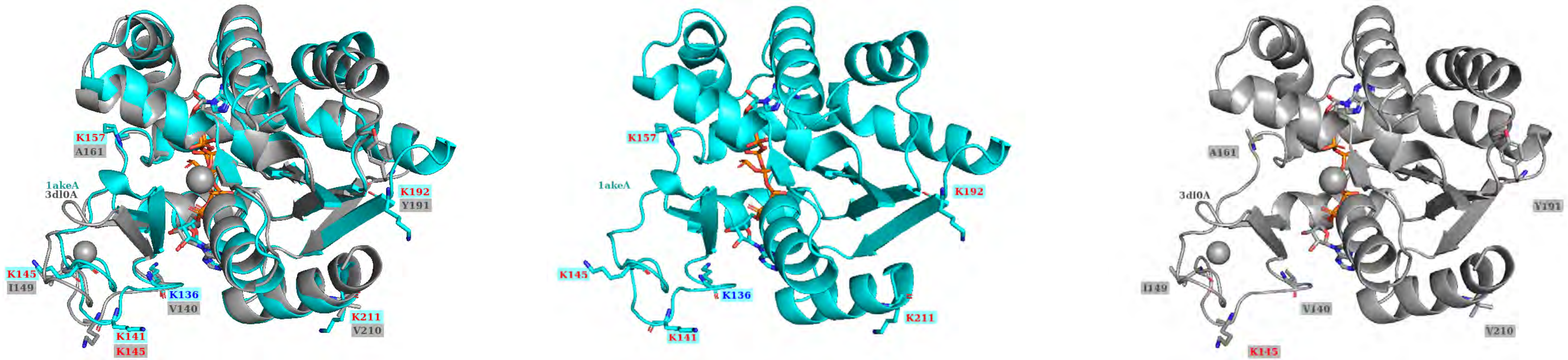

UniProt ID: P16304

PDB ID: 4MKF_A

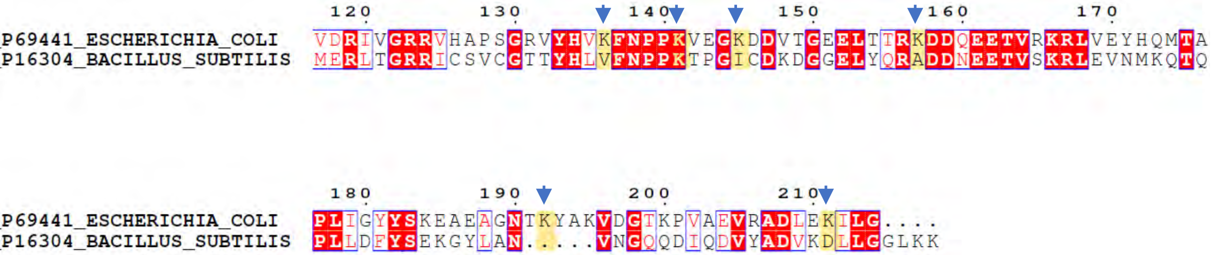


Full sequences in supplemental file.

```
Align 1ake.A.pdb 214 with 4mkf.A.pdb 217
Twists 0 ini-len 200 ini-rmsd 1.33 opt-equ 209 opt-rmsd 1.30 chain-rmsd 1.33 Score 552.02 align-len 218 gaps 9 (4.13%)
P-value 0.00e+00 Afp-num 14436 Identity 46.33% Similarity 66.06%
Block  0 afp 25 score 552.02 rmsd  1.33 gap 17 (0.08%)

                  .    :    .    :    .    :    .    :    .    :    .    :    .    :
Chain 1:    1 MRIILLGAPGAGKGTQAQFIMEKYGIPQISTGDMLRAAVKSGSELGKQAKDIMDAGKLVTDELVIALVKE
              1111111111111111111111111111111111111111111111111111111111111111111111
Chain 2:    1 MNLVLMGLPGAGKGTQGEKIVEDYGIPHISTGDMFRAAMKEETPLGLEAKSYIDKGELVPDEVTIGIVKE

                  .    :    .    :    .    :    .    :    .    :    .    :    .    :
Chain 1:   71 RIAQEDCRNGFLLDGFPRTIPQADAMKEAG----INVDYVLEFDVPDELIVDRIVGRRVHAPSGRVYHVK
              111111111111111111111111111111    111111111111111111111111111111111111
Chain 2:   71 RLGKDDCERGFLLDGFPRTVAQAEALEEILEEYGKPIDYVINIEVRKDVLMERLTGRRICSVCGTTYHLV

                  .    :    .    :    .    :    .    :    .    :    .    :    .    :
Chain 1:  137 FNPPKVEGKDDVTGEELTTRKDDQEETVRKRLVEYHQMTAPLIGYYSKEAEAGNTKYAKVDGTKPVAEVR
              11111111111111111111111111111111111111111111111111     111111111111111
Chain 2:  141 FNPPKTPGICDKDGGELYQRADDNEETVSKRLEVNMKQMQPLLDFYSEKG-----YLANVNGEQDIEDVY

                  .
Chain 1:  207 ADLEKILG
              11111111
Chain 2:  206 ADVKDLLG

Note: positions are from PDB; the numbers between alignments are block index
```

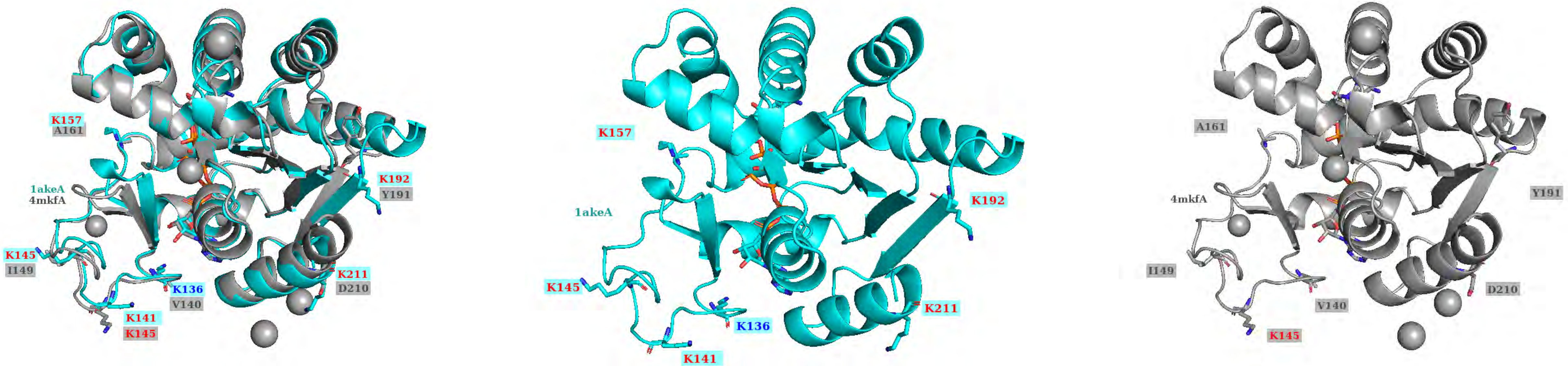

UniProt ID: P16304

PDB ID: 4MKG_A

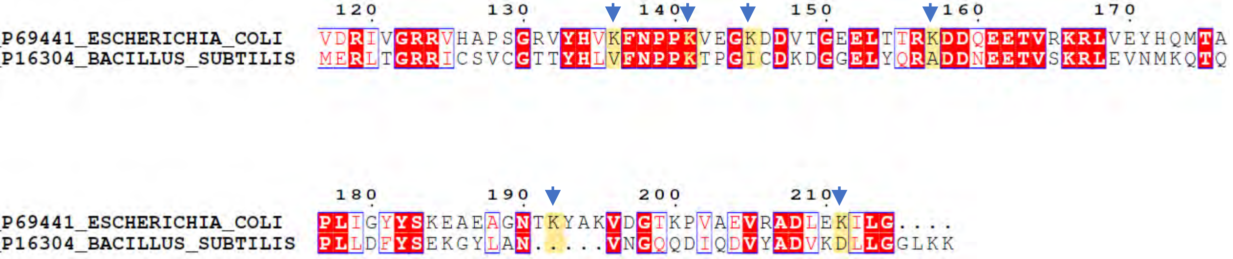


Full sequences in supplemental file.

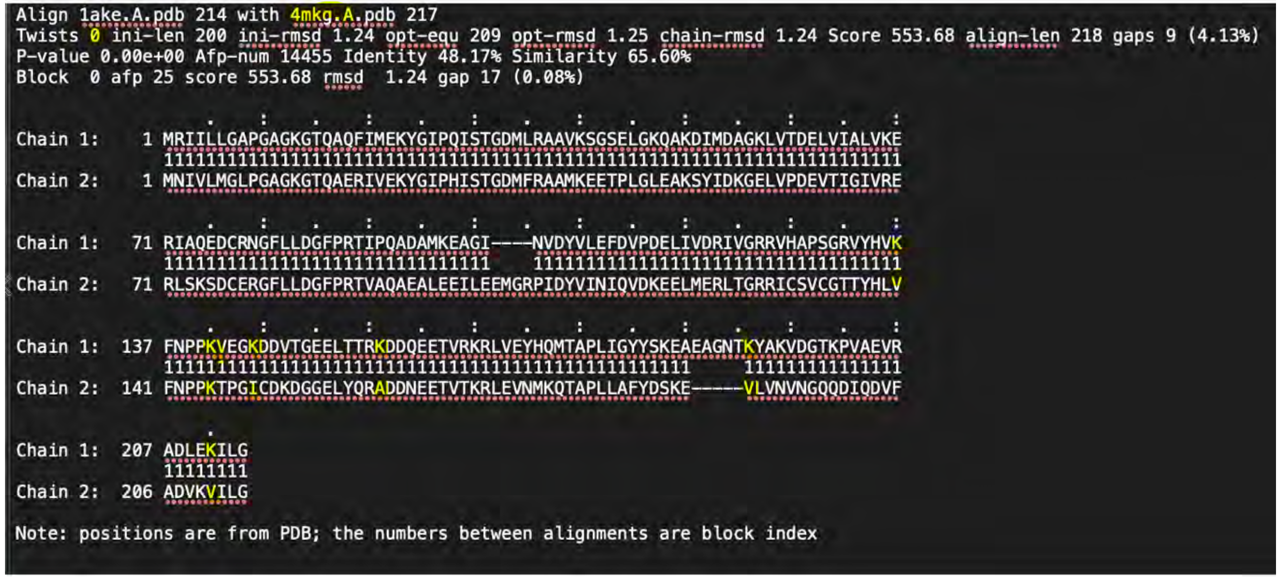


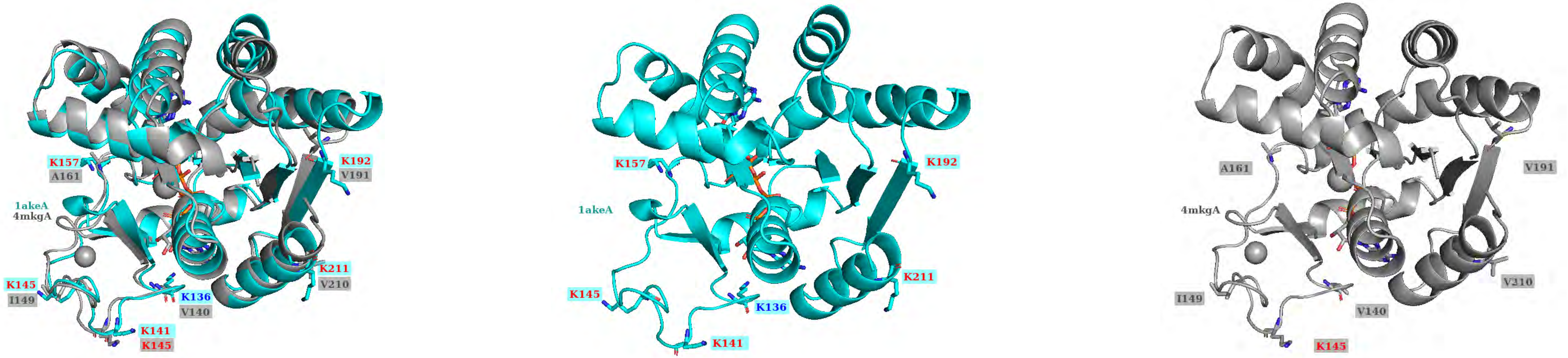

UniProt ID: P16304

PDB ID: 4MKH_A

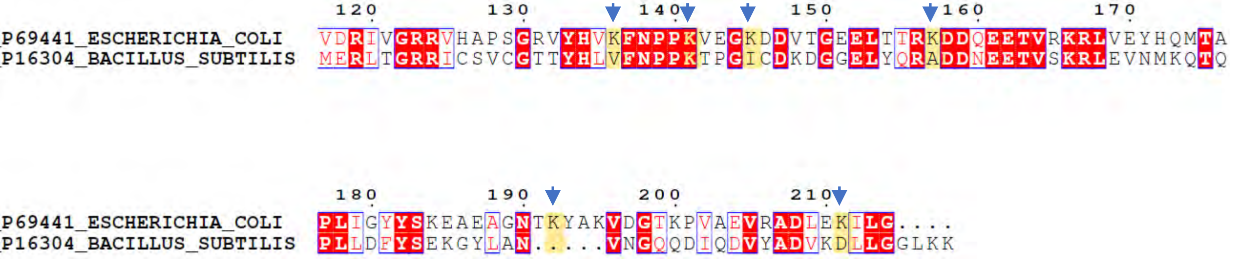


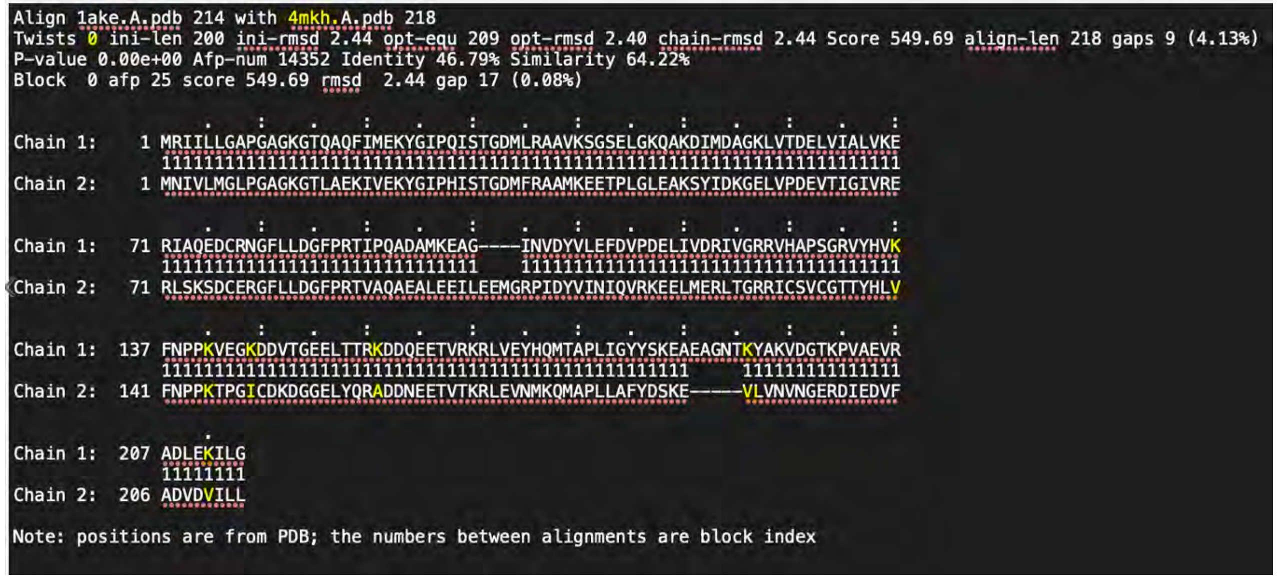

```
Align 1ake.A.pdb 214 with 4mkh.A.pdb 218
Twists 0 ini-len 200 ini-rmsd 2.44 opt-equ 209 opt-rmsd 2.40 chain-rmsd 2.44 Score 549.69 align-len 218 gaps 9 (4.13%)
P-value 0.00e+00 Afp-num 14352 Identity 46.79% Similarity 64.22%
Block  0 afp 25 score 549.69 rmsd  2.44 gap 17 (0.08%)

                  .    :    .    :    .    :    .    :    .    :    .    :    .    :
Chain 1:    1 MRIILLGAPGAGKGTQAQFIMEKYGIPQISTGDMLRAAVKSGSELGKQAKDIMDAGKLVTDELVIALVKE
              1111111111111111111111111111111111111111111111111111111111111111111111
Chain 2:    1 MNIVLMGLPGAGKGTLAEKIVEKYGIPHISTGDMFRAAMKEETPLGLEAKSYIDKGELVPDEVTIGIVRE

                  .    :    .    :    .    :    .    :    .    :    .    :    .    :
Chain 1:   71 RIAQEDCRNGFLLDGFPRTIPQADAMKEAG----INVDYVLEFDVPDELIVDRIVGRRVHAPSGRVYHVK
              111111111111111111111111111111    111111111111111111111111111111111111
Chain 2:   71 RLSKSDCERGFLLDGFPRTVAQAEALEEILEEMGRPIDYVINIQVRKEELMERLTGRRICSVCGTTYHLV

                  .    :    .    :    .    :    .    :    .    :    .    :    .    :
Chain 1:  137 FNPPKVEGKDDVTGEELTTRKDDQEETVRKRLVEYHQMTAPLIGYYSKEAEAGNTKYAKVDGTKPVAEVR
              11111111111111111111111111111111111111111111111111     111111111111111
Chain 2:  141 FNPPKTPGICDKDGGELYQRADDNEETVTKRLEVNMKQMAPLLAFYDSKE-----VLVNVNGERDIEDVF

                  .
Chain 1:  207 ADLEKILG
              11111111
Chain 2:  206 ADVDVILL

Note: positions are from PDB; the numbers between alignments are block index
```

Full sequences in supplemental file.

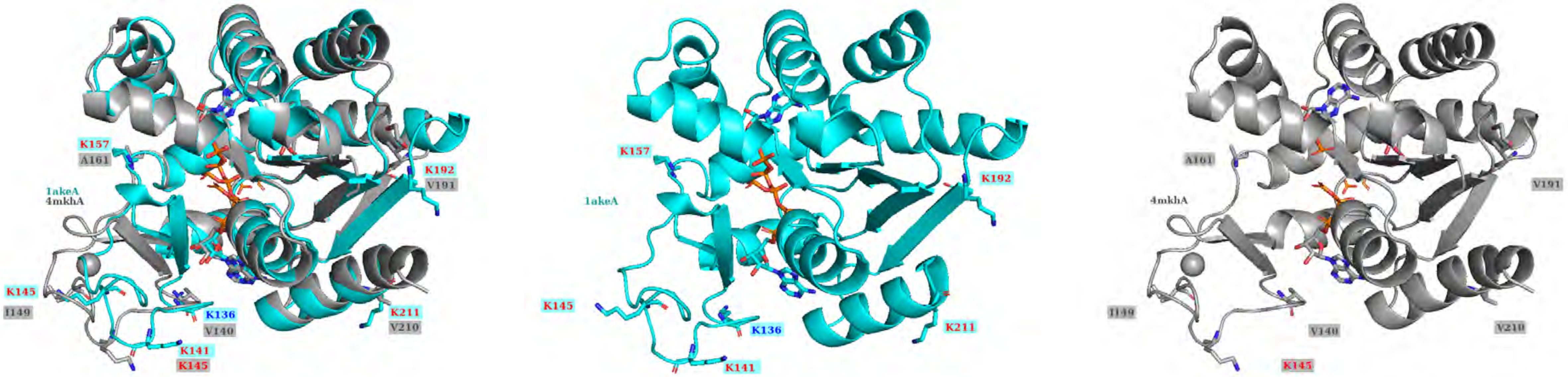

UniProt ID: P16304
PDB ID: 4QBF_A

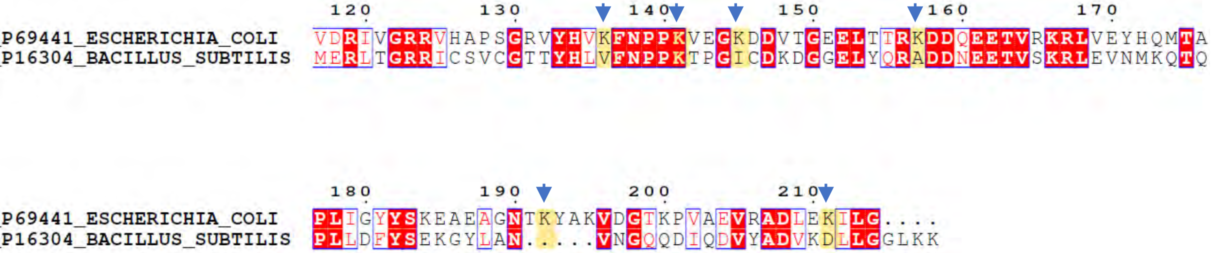


Full sequences in supplemental file.

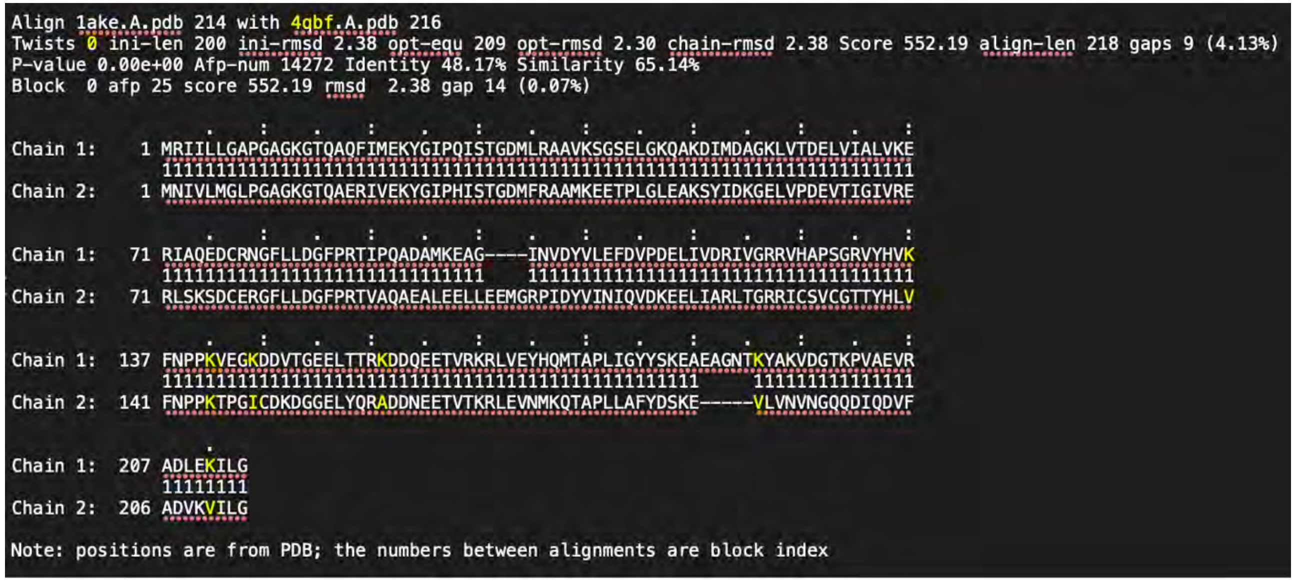

```
Align 1ake.A.pdb 214 with 4qbf.A.pdb 216
Twists 0 ini-len 200 ini-rmsd 2.38 opt-equ 209 opt-rmsd 2.30 chain-rmsd 2.38 Score 552.19 align-len 218 gaps 9 (4.13%)
P-value 0.00e+00 Afp-num 14272 Identity 48.17% Similarity 65.14%
Block  0 afp 25 score 552.19 rmsd  2.38 gap 14 (0.07%)

                .    :    .    :    .    :    .    :    .    :    .    :    .    :
Chain 1:    1 MRIILLGAPGAGKGTQAQFIMEKYGIPQISTGDMLRAAVKSGSELGKQAKDIMDAGKLVTDELVIALVKE
              1111111111111111111111111111111111111111111111111111111111111111111111
Chain 2:    1 MNIVLMGLPGAGKGTQAERIVEKYGIPHISTGDMFRAAMKEETPLGLEAKSYIDKGELVPDEVTIGIVRE

                .    :    .    :    .    :    .    :    .    :    .    :    .    :
Chain 1:   71 RIAQEDCRNGFLLDGFPRTIPQADAMKEAG----INVDYVLEFDVPDELIVDRIVGRRVHAPSGRVYHVK
              111111111111111111111111111111    111111111111111111111111111111111111
Chain 2:   71 RLSKSDCERGFLLDGFPRTVAQAEALEELLEEMGRPIDYVINIQVDKEELIARLTGRRICSVCGTTYHLV

                .    :    .    :    .    :    .    :    .    :    .    :    .    :
Chain 1:  137 FNPPKVEGKDDVTGEELTTRKDDQEETVRKRLVEYHQMTAPLIGYYSKEAEAGNTKYAKVDGTKPVAEVR
              11111111111111111111111111111111111111111111111111     111111111111111
Chain 2:  141 FNPPKTPGICDKDGGELYQRADDNEETVTKRLEVNMKQTAPLLAFYDSKE-----VLVNVNGQQDIQDVF

                .
Chain 1:  207 ADLEKILG
              11111111
Chain 2:  206 ADVKVILG

Note: positions are from PDB; the numbers between alignments are block index
```

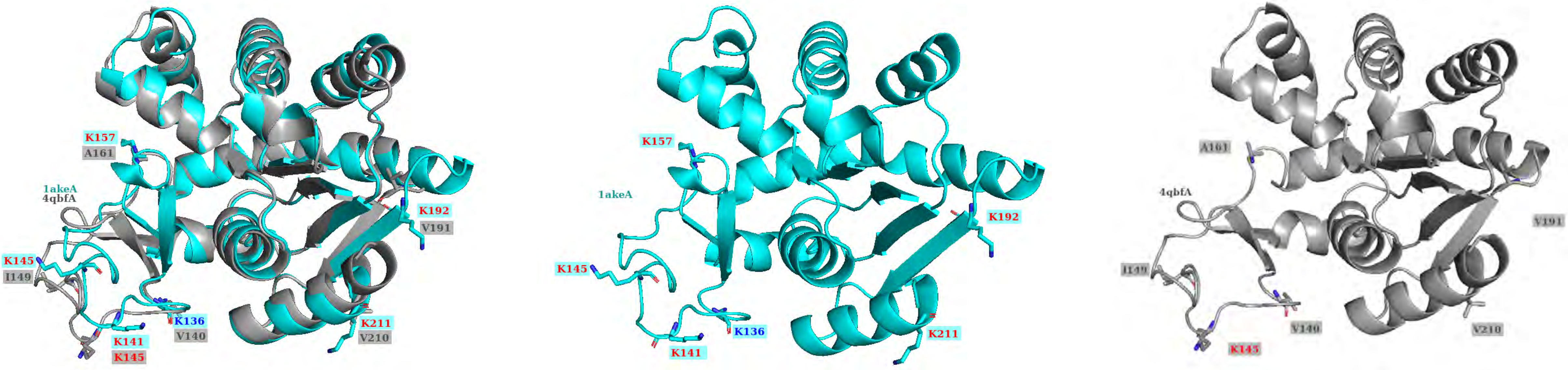

UniProt ID: P16304

PDB ID: 4QBG_B

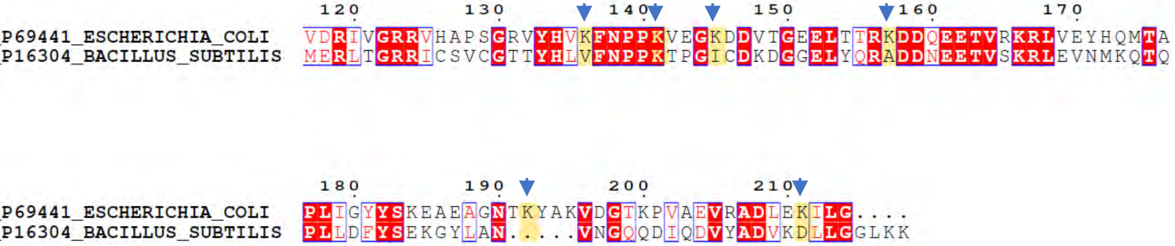


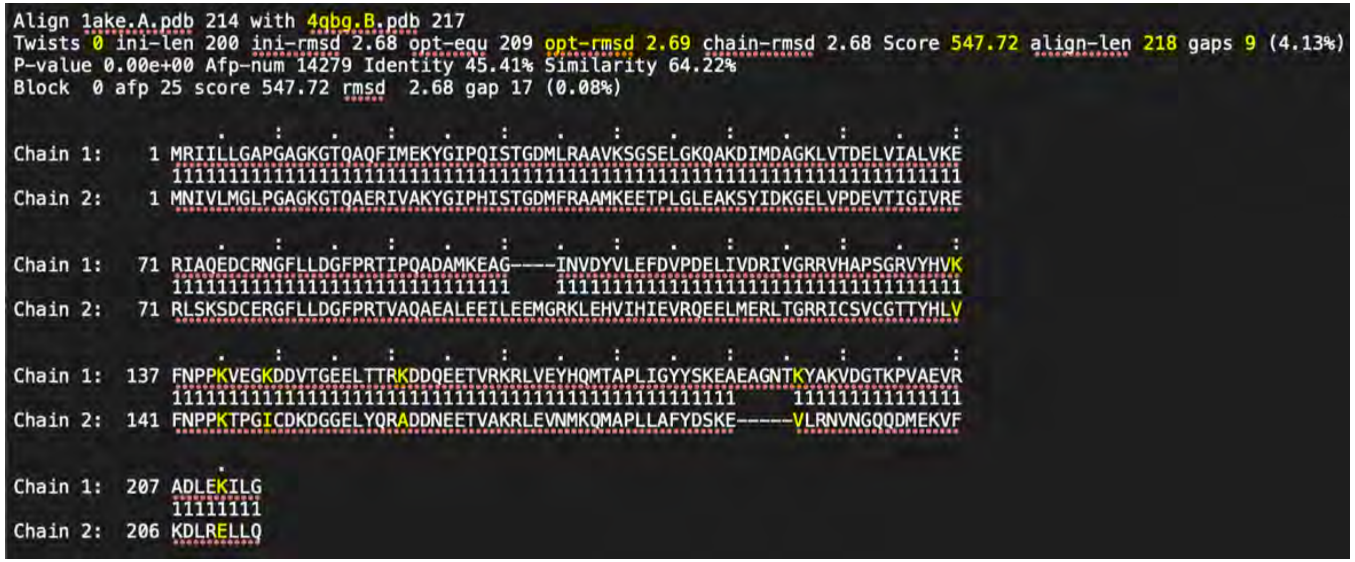

```
Align 1ake.A.pdb 214 with 4qbg.B.pdb 217
Twists 0 ini-len 200 ini-rmsd 2.68 opt-equ 209 opt-rmsd 2.69 chain-rmsd 2.68 Score 547.72 align-len 218 gaps 9 (4.13%)
P-value 0.00e+00 Afp-num 14279 Identity 45.41% Similarity 64.22%
Block  0 afp 25 score 547.72 rmsd  2.68 gap 17 (0.08%)

                  .    :    .    :    .    :    .    :    .    :    .    :    .    :
Chain 1:    1 MRIILLGAPGAGKGTQAQFIMEKYGIPQISTGDMLRAAVKSGSELGKQAKDIMDAGKLVTDELVIALVKE
              1111111111111111111111111111111111111111111111111111111111111111111111
Chain 2:    1 MNIVLMGLPGAGKGTQAERIVAKYGIPHISTGDMFRAAMKEETPLGLEAKSYIDKGELVPDEVTIGIVRE

                  .    :    .    :    .    :    .    :    .    :    .    :    .    :
Chain 1:   71 RIAQEDCRNGFLLDGFPRTIPQADAMKEAG----INVDYVLEFDVPDELIVDRIVGRRVHAPSGRVYHVK
              111111111111111111111111111111    111111111111111111111111111111111111
Chain 2:   71 RLSKSDCERGFLLDGFPRTVAQAEALEEILEEMGRKLEHVIHIEVRQEELMERLTGRRICSVCGTTYHLV

                  .    :    .    :    .    :    .    :    .    :    .    :    .    :
Chain 1:  137 FNPPKVEGKDDVTGEELTTRKDDQEETVRKRLVEYHQMTAPLIGYYSKEAEAGNTKYAKVDGTKPVAEVR
              11111111111111111111111111111111111111111111111111     11111111111111
Chain 2:  141 FNPPKTPGICDKDGGELYQRADDNEETVAKRLEVNMKQMAPLLAFYDSKE-----VLRNVNGQQDMEKVF

                  .
Chain 1:  207 ADLEKILG
              11111111
Chain 2:  206 KDLRELLQ
```

Full sequences in supplemental file.

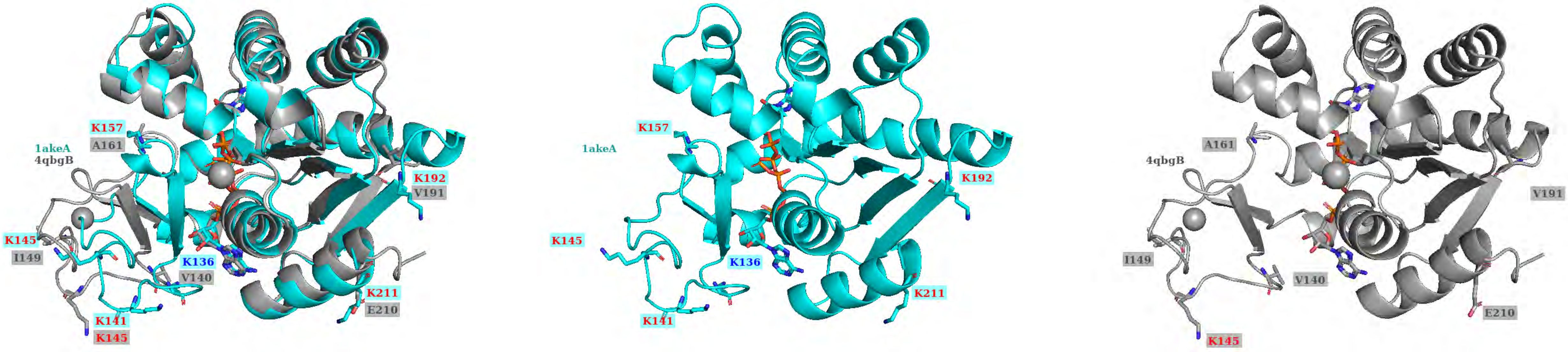

UniProt ID: P16304

PDB ID: 4TYP_C

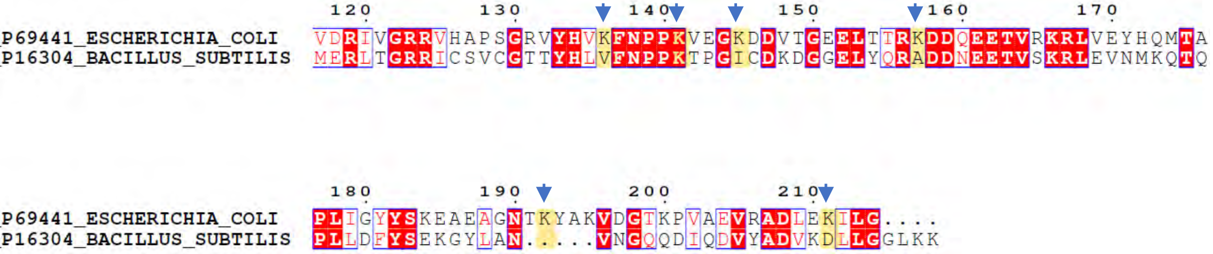


Full sequences in supplemental file.

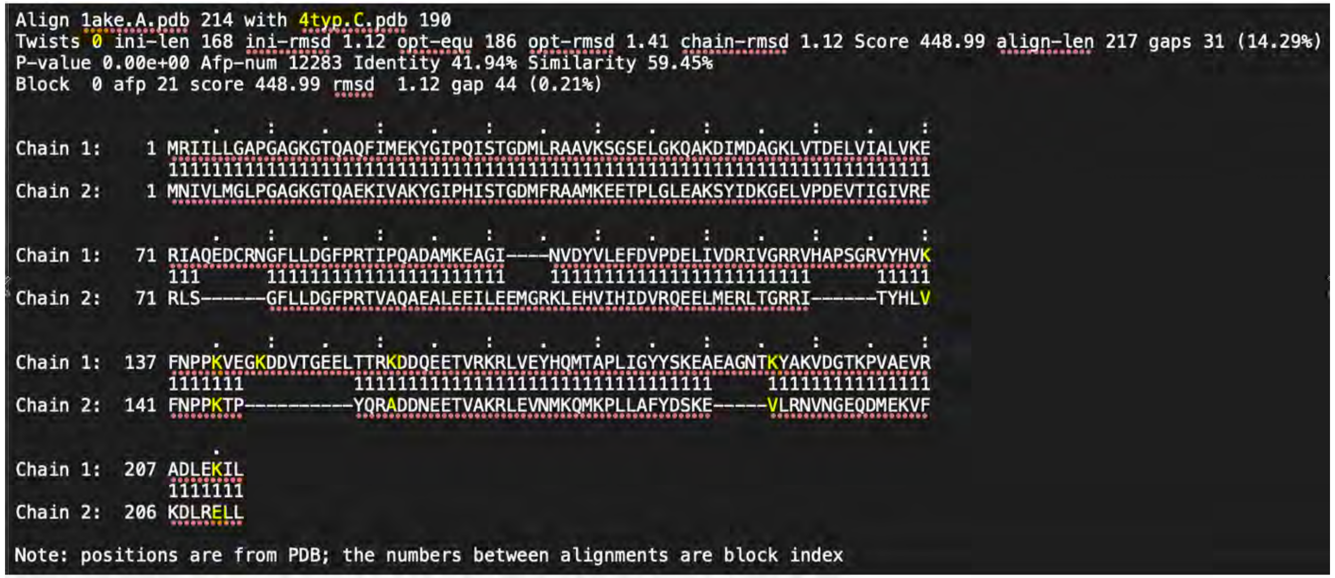

```
Align 1ake.A.pdb 214 with 4typ.C.pdb 190
Twists 0 ini-len 168 ini-rmsd 1.12 opt-equ 186 opt-rmsd 1.41 chain-rmsd 1.12 Score 448.99 align-len 217 gaps 31 (14.29%)
P-value 0.00e+00 Afp-num 12283 Identity 41.94% Similarity 59.45%
Block  0 afp 21 score 448.99 rmsd  1.12 gap 44 (0.21%)

Chain 1:    1 MRIILLGAPGAGKGTQAQFIMEKYGIPQISTGDMLRAAVKSGSELGKQAKDIMDAGKLVTDELVIALVKE
                111111111111111111111111111111111111111111111111111111111111111111111
Chain 2:    1 MNIVLMGLPGAGKGTQAEKIVAKYGIPHISTGDMFRAAMKEETPLGLEAKSYIDKGELVPDEVTIGIVRE

Chain 1:   71 RIAQEDCRNGFLLDGFPRTIPQADAMKEAGI----NVDYVLEFDVPDELIVDRIVGRRVHAPSGRVYHVK
                111      1111111111111111111111     11111111111111111111111111      11111
Chain 2:   71 RLS------GFLLDGFPRTVAQAEALEEILEEMGRKLEHVIHIDVRQEELMERLTGRRI------TYHLV

Chain 1:  137 FNPPKVEGKDDVTGEELTTRKDDQEETVRKRLVEYHQMTAPLIGYYSKEAEAGNTKYAKVDGTKPVAEVR
                1111111            111111111111111111111111111111111      11111111111111
Chain 2:  141 FNPPKTP-----------YQRADDNEETVAKRLEVNMKQMKPLLAFYDSKE-----VLRNVNGEQDMEKVF

Chain 1:  207 ADLEKIL
                1111111
Chain 2:  206 KDLRELL

Note: positions are from PDB; the numbers between alignments are block index
```

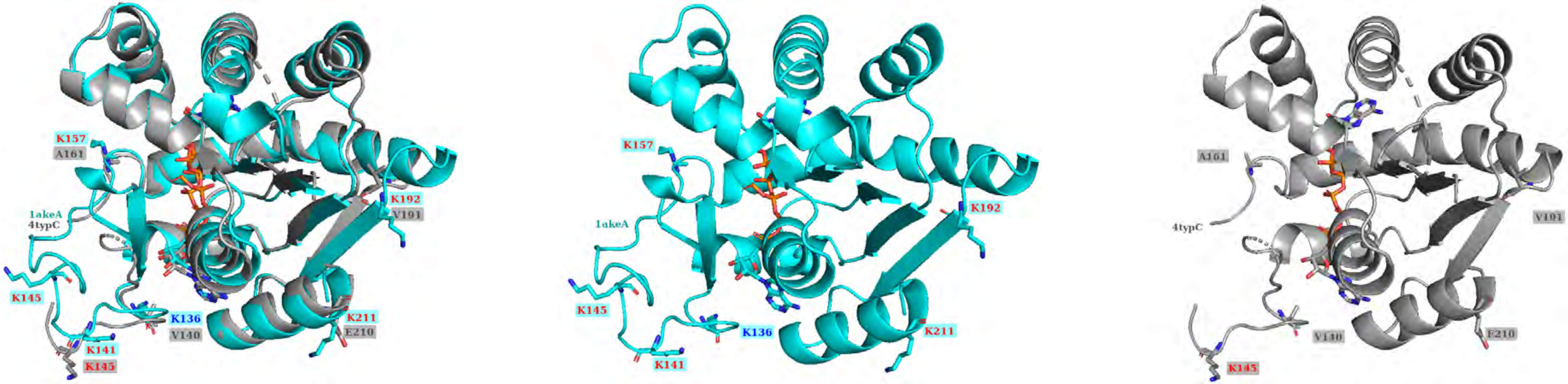

UniProt ID: P16304

PDB ID: 4TYQ_A

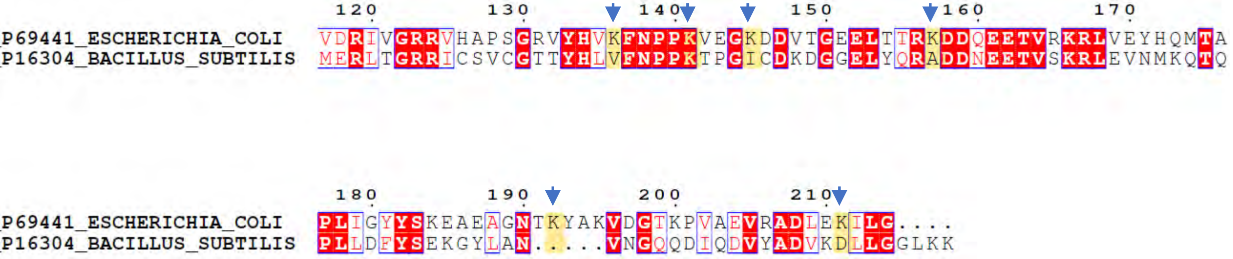


Full sequences in supplemental file.

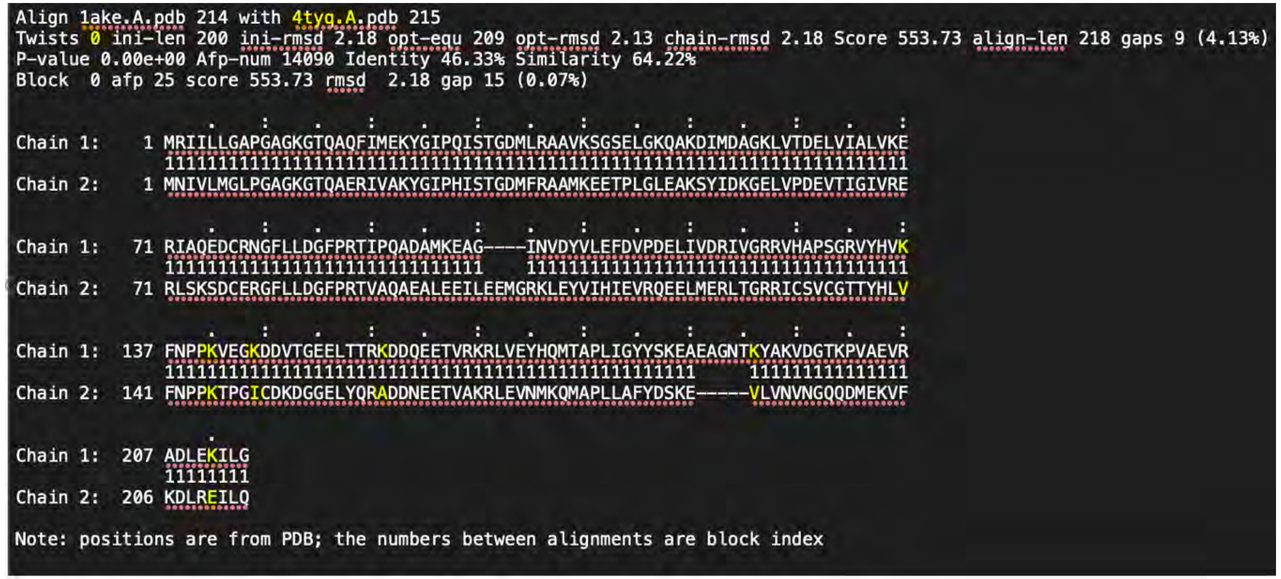

```
Align 1ake.A.pdb 214 with 4tyq.A.pdb 215
Twists 0 ini-len 200 ini-rmsd 2.18 opt-equ 209 opt-rmsd 2.13 chain-rmsd 2.18 Score 553.73 align-len 218 gaps 9 (4.13%)
P-value 0.00e+00 Afp-num 14090 Identity 46.33% Similarity 64.22%
Block  0 afp 25 score 553.73 rmsd  2.18 gap 15 (0.07%)

Chain 1:    1 MRIILLGAPGAGKGTQAQFIMEKYGIPQISTGDMLRAAVKSGSELGKQAKDIMDAGKLVTDELVIALVKE
              1111111111111111111111111111111111111111111111111111111111111111111111
Chain 2:    1 MNIVLMGLPGAGKGTQAERIVAKYGIPHISTGDMFRAAMKEETPLGLEAKSYIDKGELVPDEVTIGIVRE

Chain 1:   71 RIAQEDCRNGFLLDGFPRTIPQADAMKEAG----INVDYVLEFDVPDELIVDRIVGRRVHAPSGRVYHVK
              111111111111111111111111111111    11111111111111111111111111111111111
Chain 2:   71 RLSKSDCERGFLLDGFPRTVAQAEALEEILEEMGRKLEYVIHIEVRQEELMERLTGRRICSVCGTTYHLV

Chain 1:  137 FNPPKVEGKDDVTGEELTTRKDDQEETVRKRLVEYHQMTAPLIGYYSKEAEAGNTKYAKVDGTKPVAEVR
              11111111111111111111111111111111111111111111111111      11111111111111
Chain 2:  141 FNPPKTPGICDKDGGELYQRADDNEETVAKRLEVNMKQMAPLLAFYDSKE-----VLVNVNGQQDMEKVF

Chain 1:  207 ADLEKILG
              11111111
Chain 2:  206 KDLREILQ

Note: positions are from PDB; the numbers between alignments are block index
```

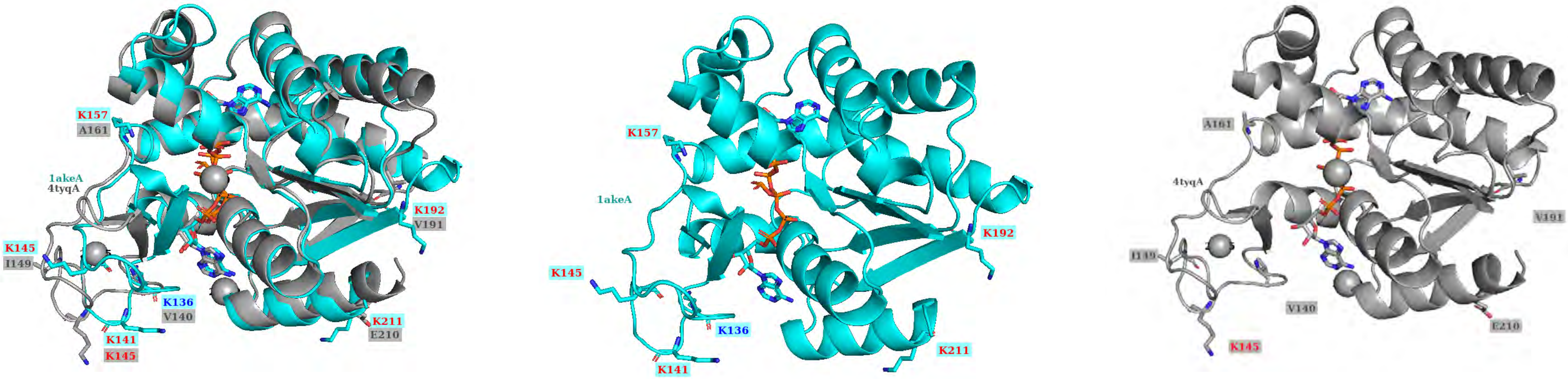

UniProt ID: P16304

PDB ID: 5X6I_A

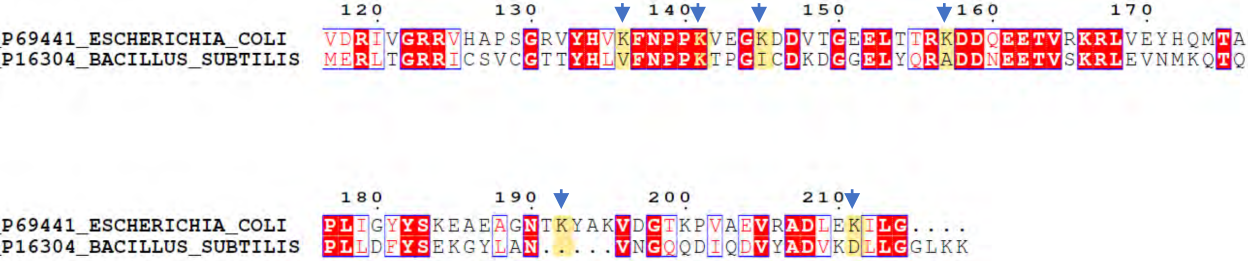


Full sequences in supplemental file.

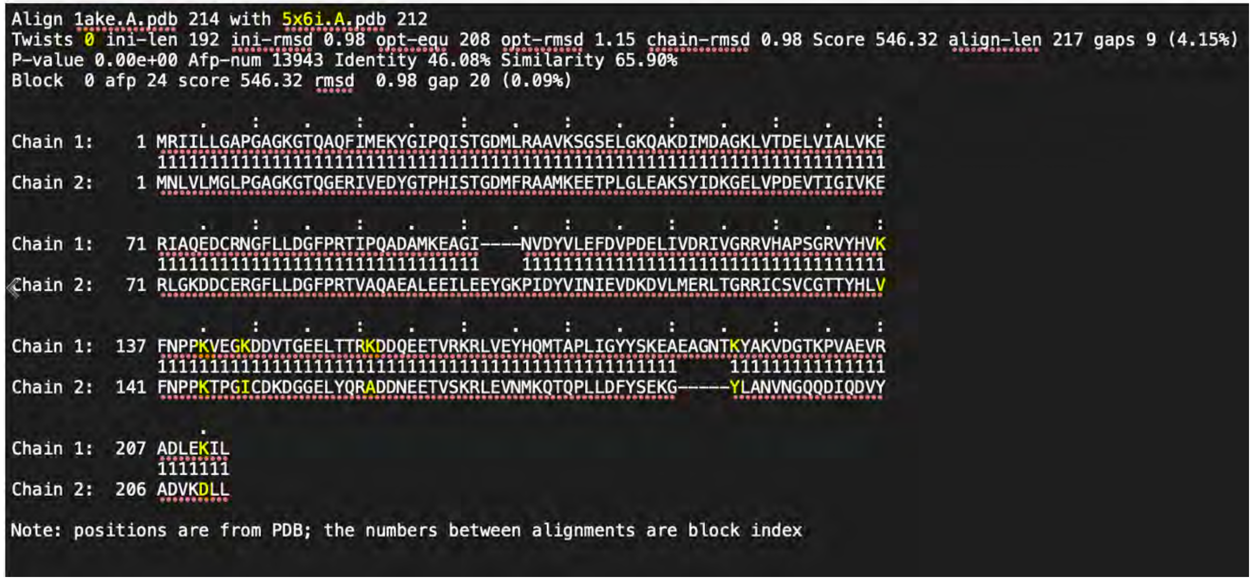

```
Align 1ake.A.pdb 214 with 5x6i.A.pdb 212
Twists 0 ini-len 192 ini-rmsd 0.98 opt-equ 208 opt-rmsd 1.15 chain-rmsd 0.98 Score 546.32 align-len 217 gaps 9 (4.15%)
P-value 0.00e+00 Afp-num 13943 Identity 46.08% Similarity 65.90%
Block  0 afp 24 score 546.32 rmsd  0.98 gap 20 (0.09%)

                .    :    .    :    .    :    .    :    .    :    .    :    .    :
Chain 1:    1 MRIILLGAPGAGKGTQAQFIMEKYGIPQISTGDMLRAAVKSGSELGKQAKDIMDAGKLVTDELVIALVKE
              1111111111111111111111111111111111111111111111111111111111111111111111
Chain 2:    1 MNLVLMGLPGAGKGTQGERIVEDYGTPHISTGDMFRAAMKEETPLGLEAKSYIDKGELVPDEVTIGIVKE

                .    :    .    :    .    :    .    :    .    :    .    :    .    :
Chain 1:   71 RIAQEDCRNGFLLDGFPRTIPQADAMKEAGI----NVDYVLEFDVPDELIVDRIVGRRVHAPSGRVYHVK
              1111111111111111111111111111111    111111111111111111111111111111111111
Chain 2:   71 RLGKDDCERGFLLDGFPRTVAQAEALEEILEEYGKPIDYVINIEVDKDVLMERLTGRRICSVCGTTYHLV

                .    :    .    :    .    :    .    :    .    :    .    :    .    :
Chain 1:  137 FNPPKVEGKDDVTGEELTTRKDDQEETVRKRLVEYHQMTAPLIGYYSKEAEAGNTKYAKVDGTKPVAEVR
              11111111111111111111111111111111111111111111111111     111111111111111
Chain 2:  141 FNPPKTPGICDKDGGELYQRADDNEETVSKRLEVNMKQTQPLLDFYSEKG-----YLANVNGQQDIQDVY

                .
Chain 1:  207 ADLEKIL
              1111111
Chain 2:  206 ADVKDLL

Note: positions are from PDB; the numbers between alignments are block index
```

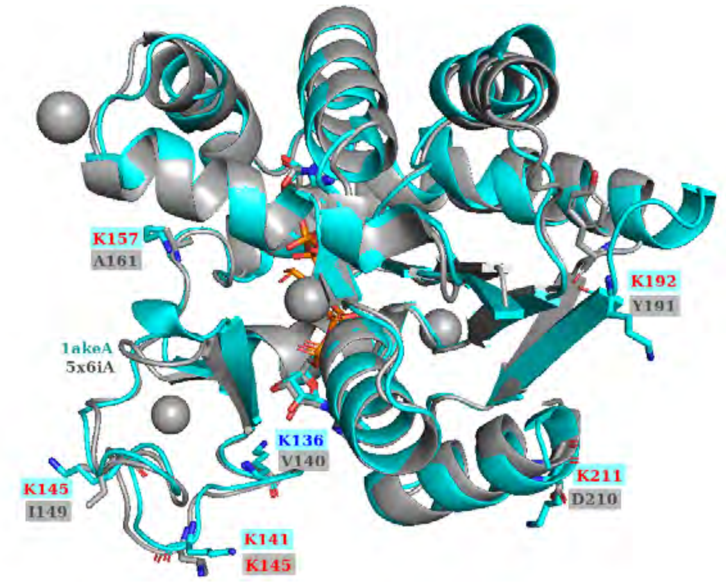


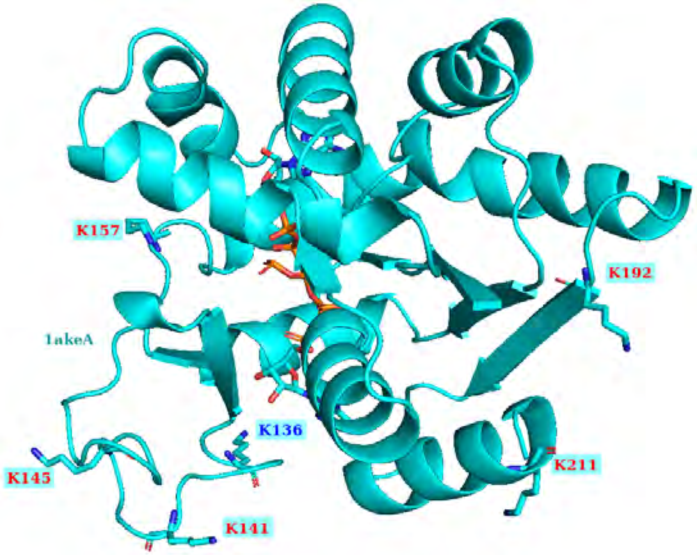


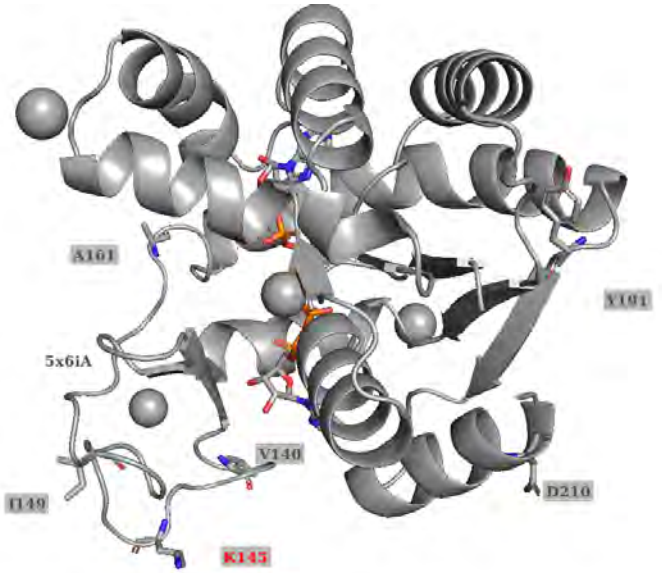

UniProt ID: P27142

PDB ID: 1ZIN_A

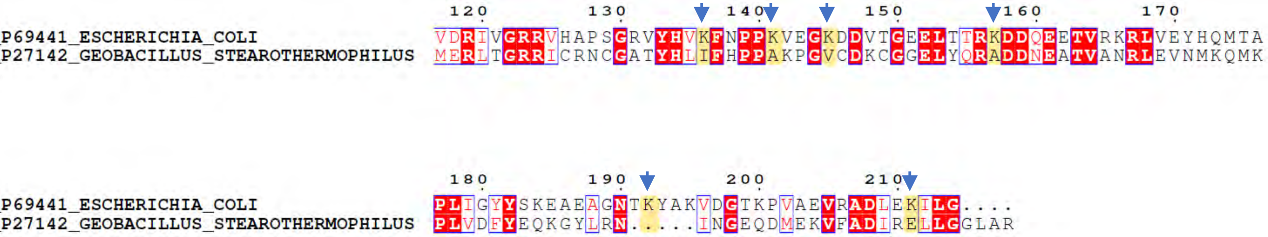


Full sequences in supplemental file.

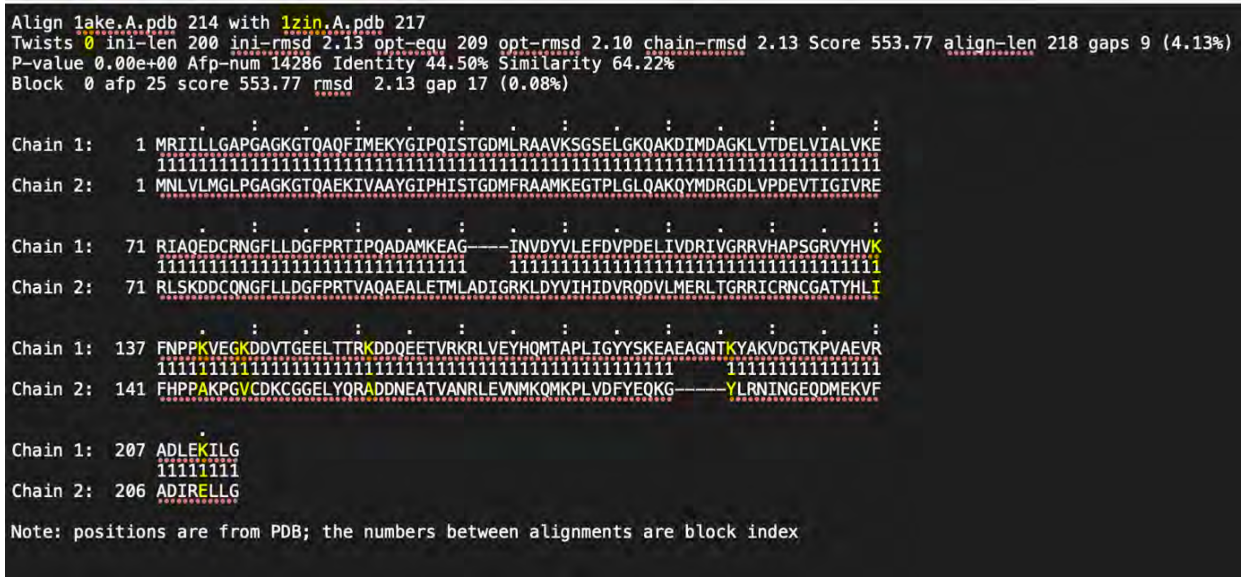

```
Align 1ake.A.pdb 214 with 1zin.A.pdb 217
Twists 0 ini-len 200 ini-rmsd 2.13 opt-equ 209 opt-rmsd 2.10 chain-rmsd 2.13 Score 553.77 align-len 218 gaps 9 (4.13%)
P-value 0.00e+00 Afp-num 14286 Identity 44.50% Similarity 64.22%
Block  0 afp 25 score 553.77 rmsd  2.13 gap 17 (0.08%)

                .    :    .    :    .    :    .    :    .    :    .    :    .    :
Chain 1:    1 MRIILLGAPGAGKGTQAQFIMEKYGIPQISTGDMLRAAVKSGSELGKQAKDIMDAGKLVTDELVIALVKE
              1111111111111111111111111111111111111111111111111111111111111111111111
Chain 2:    1 MNLVLMGLPGAGKGTQAEKIVAAYGIPHISTGDMFRAAMKEGTPLGLQAKQYMDRGDLVPDEVTIGIVRE

                .    :    .    :    .    :    .    :    .    :    .    :    .    :
Chain 1:   71 RIAQEDCRNGFLLDGFPRTIPQADAMKEAG----INVDYVLEFDVPDELIVDRIVGRRVHAPSGRVYHVK
              111111111111111111111111111111    111111111111111111111111111111111111
Chain 2:   71 RLSKDDCQNGFLLDGFPRTVAQAEALETMLADIGRKLDYVIHIDVRQDVLMERLTGRRICRNCGATYHLI

                .    :    .    :    .    :    .    :    .    :    .    :    .    :
Chain 1:  137 FNPPKVEGKDDVTGEELTTRKDDQEETVRKRLVEYHQMTAPLIGYYSKEAEAGNTKYAKVDGTKPVAEVR
              1111111111111111111111111111111111111111111111111111     11111111111111
Chain 2:  141 FHPPAKPGVCDKCGGELYQRADDNEATVANRLEVNMKQMKPLVDFYEQKG-----YLRNINGEQDMEKVF

                .
Chain 1:  207 ADLEKILG
              11111111
Chain 2:  206 ADIRELLG

Note: positions are from PDB; the numbers between alignments are block index
```

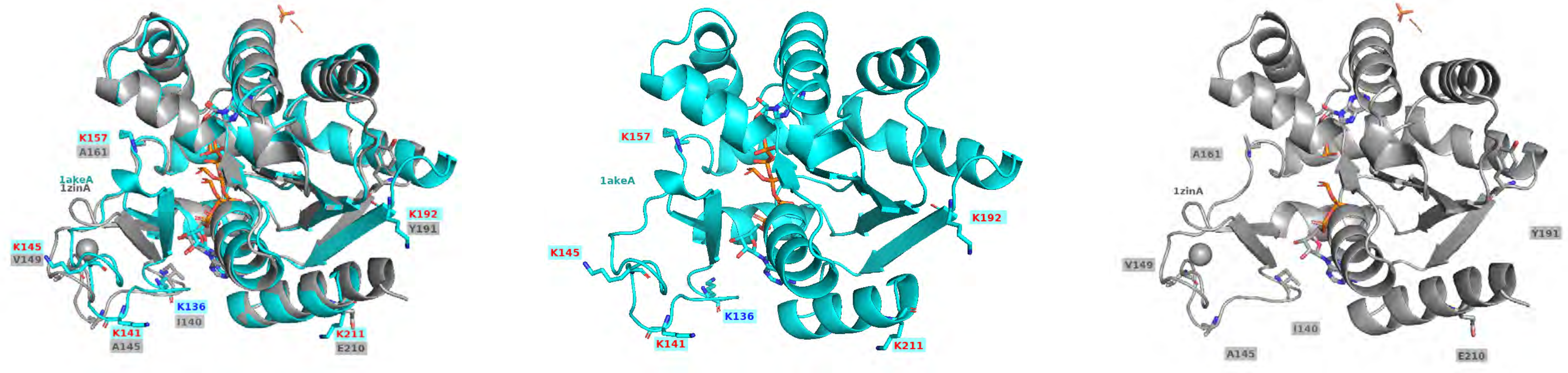

UniProt ID: P27142

PDB ID: 1ZIO_A

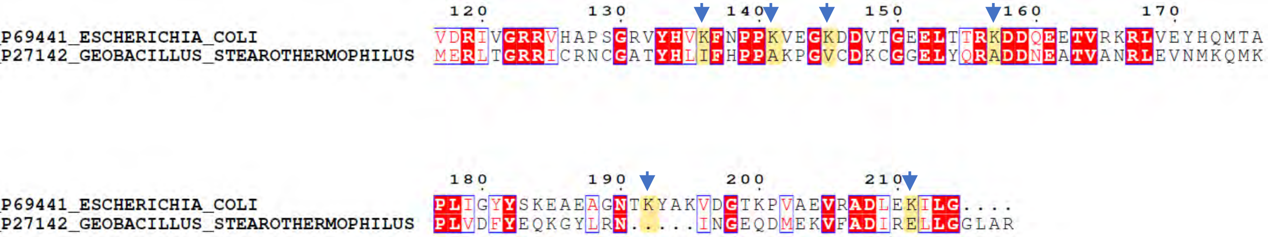


Full sequences in supplemental file.

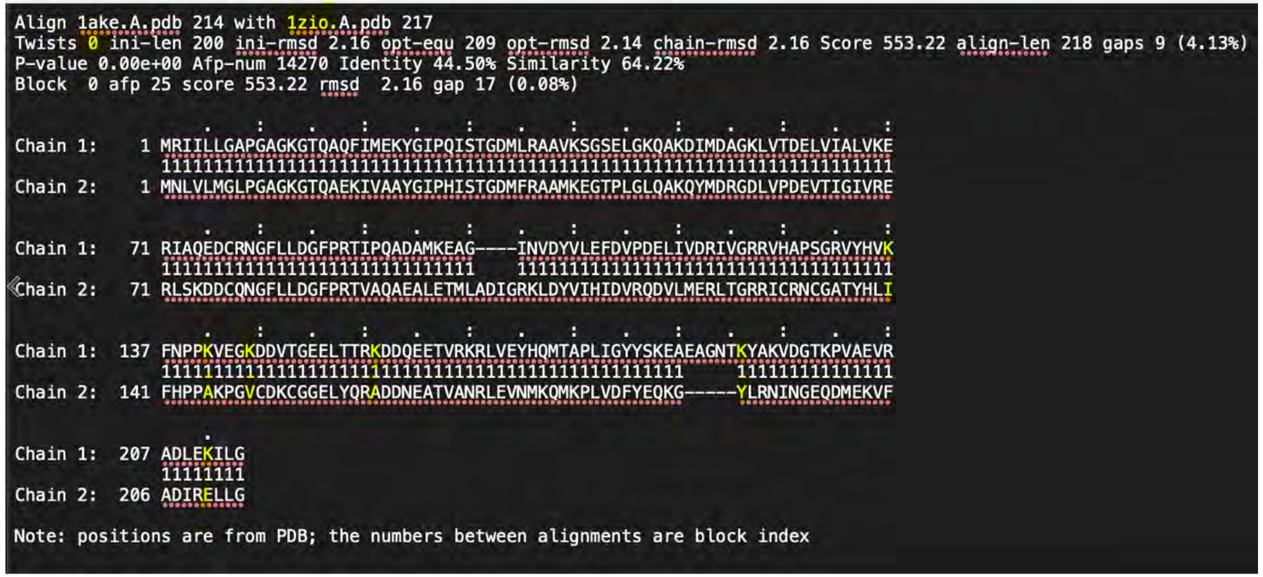

```
Align 1ake.A.pdb 214 with 1zio.A.pdb 217
Twists 0 ini-len 200 ini-rmsd 2.16 opt-equ 209 opt-rmsd 2.14 chain-rmsd 2.16 Score 553.22 align-len 218 gaps 9 (4.13%)
P-value 0.00e+00 Afp-num 14270 Identity 44.50% Similarity 64.22%
Block  0 afp 25 score 553.22 rmsd  2.16 gap 17 (0.08%)

Chain 1:    1 MRIILLGAPGAGKGTQAQFIMEKYGIPQISTGDMLRAAVKSGSELGKQAKDIMDAGKLVTDELVIALVKE
              1111111111111111111111111111111111111111111111111111111111111111111111
Chain 2:    1 MNLVLMGLPGAGKGTQAEKIVAAYGIPHISTGDMFRAAMKEGTPLGLQAKQYMDRGDLVPDEVTIGIVRE

Chain 1:   71 RIAQEDCRNGFLLDGFPRTIPQADAMKEAG----INVDYVLEFDVPDELIVDRIVGRRVHAPSGRVYHVK
              11111111111111111111111111111    111111111111111111111111111111111111
Chain 2:   71 RLSKDDCQNGFLLDGFPRTVAQAEALETMLADIGRKLDYVIHIDVRQDVLMERLTGRRICRNCGATYHLI

Chain 1:  137 FNPPKVEGKDDVTGEELTTRKDDQEETVRKRLVEYHQMTAPLIGYYSKEAEAGNTKYAKVDGTKPVAEVR
              11111111111111111111111111111111111111111111111111     111111111111111
Chain 2:  141 FHPPAKPGVCDKCGGELYQRADDNEATVANRLEVNMKQMKPLVDFYEQKG-----YLRNINGEQDMEKVF

Chain 1:  207 ADLEKILG
              11111111
Chain 2:  206 ADIRELLG

Note: positions are from PDB; the numbers between alignments are block index
```

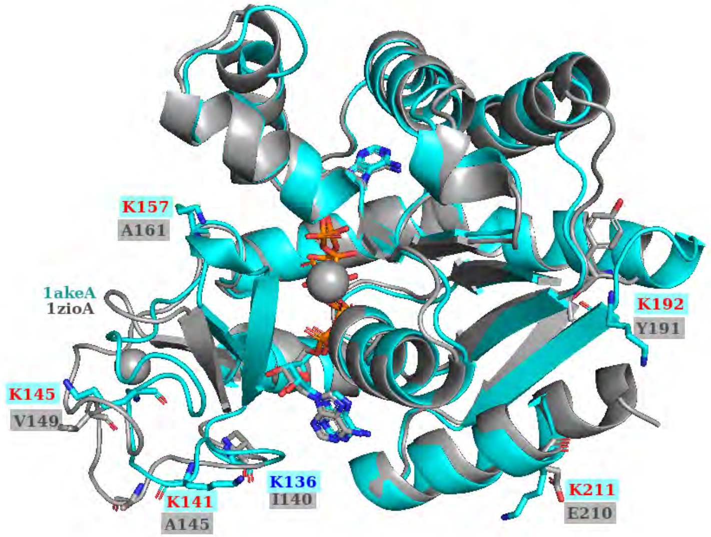


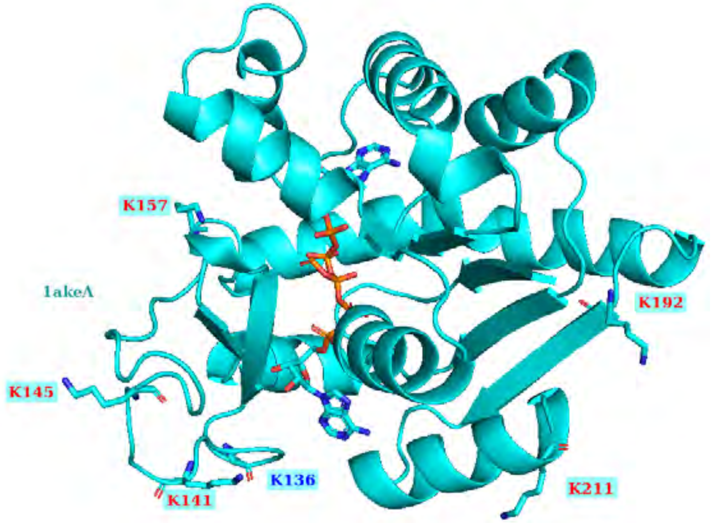


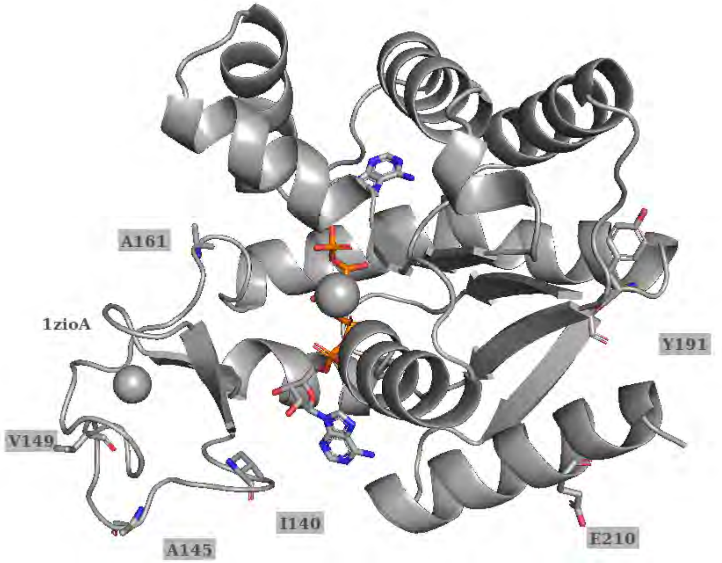

UniProt ID: P27142

PDB ID: 1ZIP_A

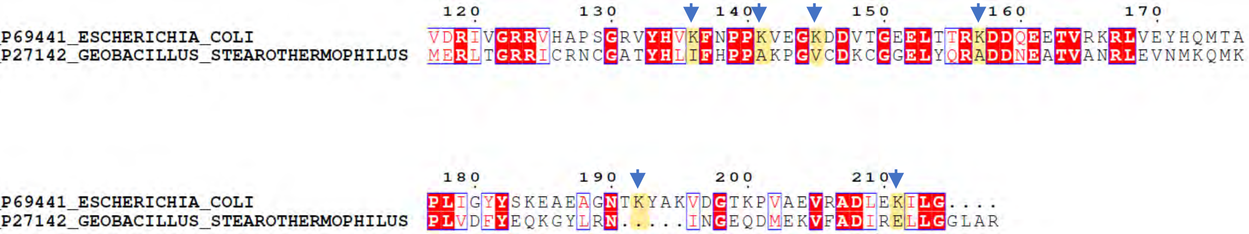


Full sequences in supplemental file.

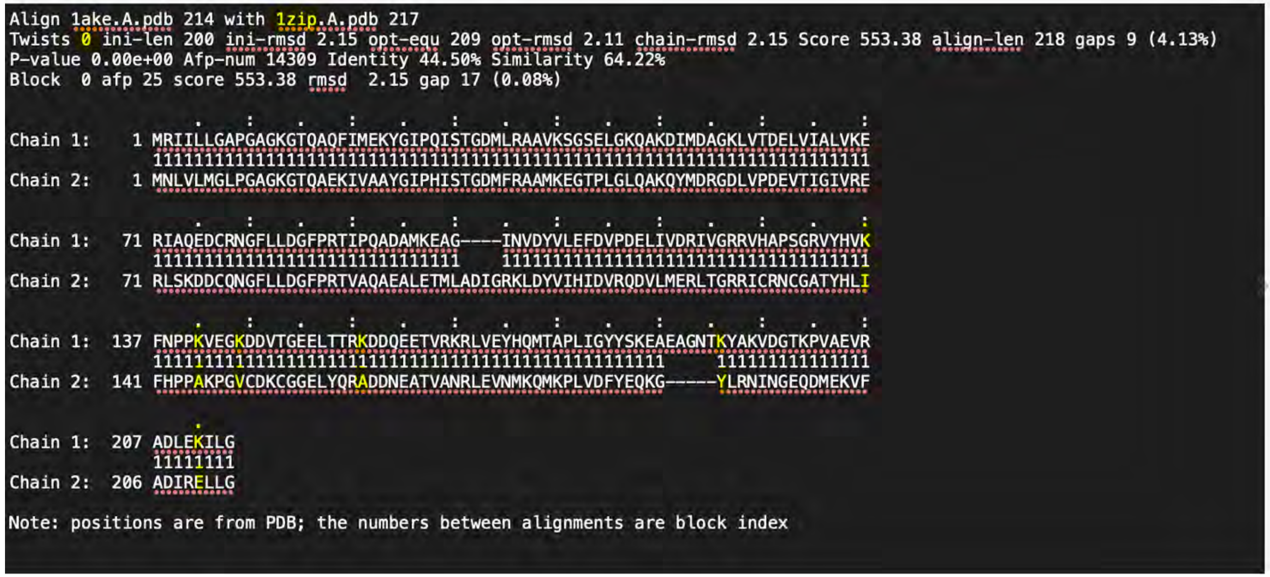

```
Align 1ake.A.pdb 214 with 1zip.A.pdb 217
Twists 0 ini-len 200 ini-rmsd 2.15 opt-equ 209 opt-rmsd 2.11 chain-rmsd 2.15 Score 553.38 align-len 218 gaps 9 (4.13%)
P-value 0.00e+00 Afp-num 14309 Identity 44.50% Similarity 64.22%
Block  0 afp 25 score 553.38 rmsd  2.15 gap 17 (0.08%)

                .    :    .    :    .    :    .    :    .    :    .    :    .    :
Chain 1:    1 MRIILLGAPGAGKGTQAQFIMEKYGIPQISTGDMLRAAVKSGSELGKQAKDIMDAGKLVTDELVIALVKE
              111111111111111111111111111111111111111111111111111111111111111111111111
Chain 2:    1 MNLVLMGLPGAGKGTQAEKIVAAYGIPHISTGDMFRAAMKEGTPLGLQAKQYMDRGDLVPDEVTIGIVRE

                .    :    .    :    .    :    .    :    .    :    .    :    .    :
Chain 1:   71 RIAQEDCRNGFLLDGFPRTIPQADAMKEAG----INVDYVLEFDVPDELIVDRIVGRRVHAPSGRVYHVK
              111111111111111111111111111111    111111111111111111111111111111111111
Chain 2:   71 RLSKDDCQNGFLLDGFPRTVAQAEALETMLADIGRKLDYVIHIDVRQDVLMERLTGRRICRNCGATYHLI

                .    :    .    :    .    :    .    :    .    :    .    :    .    :
Chain 1:  137 FNPPKVEGKDDVTGEELTTRKDDQEETVRKRLVEYHQMTAPLIGYYSKEAEAGNTKYAKVDGTKPVAEVR
              11111111111111111111111111111111111111111111111111     11111111111111
Chain 2:  141 FHPPAKPGVCDKCGGELYQRADDNEATVANRLEVNMKQMKPLVDFYEQKG-----YLRNINGEQDMEKVF

                  .
Chain 1:  207 ADLEKILG
              11111111
Chain 2:  206 ADIRELLG

Note: positions are from PDB; the numbers between alignments are block index
```

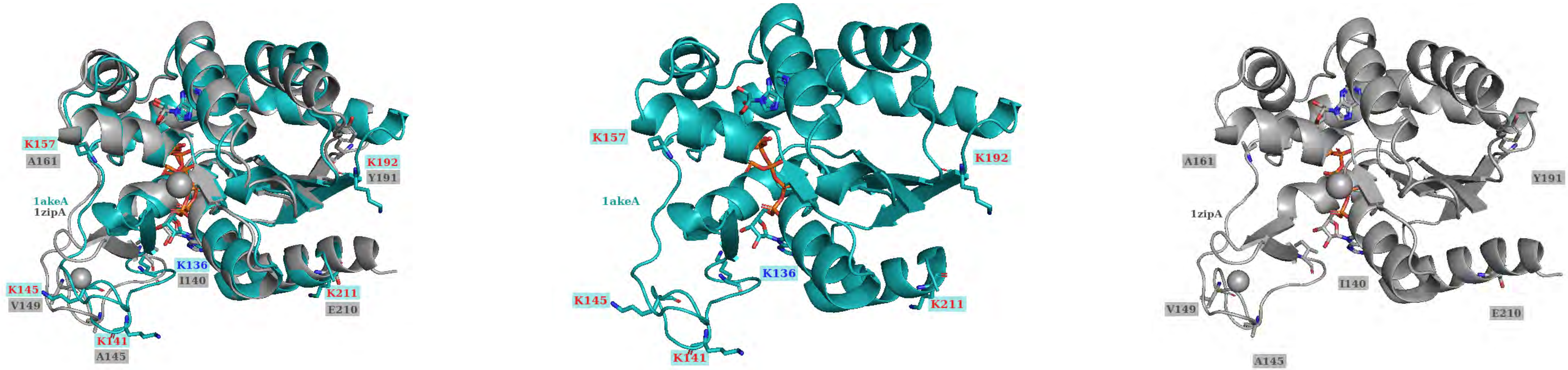

UniProt ID: P27142

PDB ID: 4QBH_A

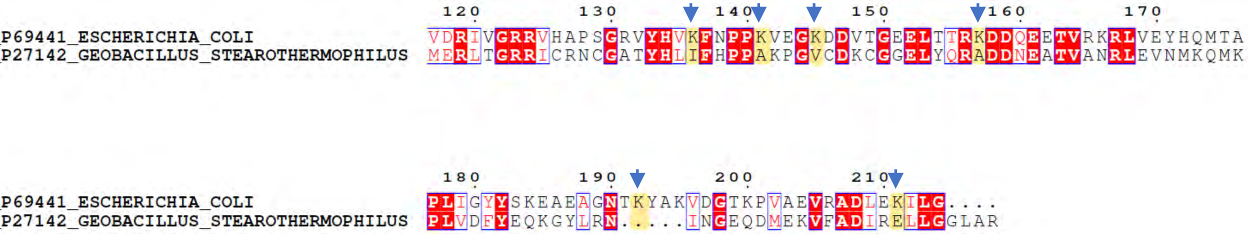


Full sequences in supplemental file.

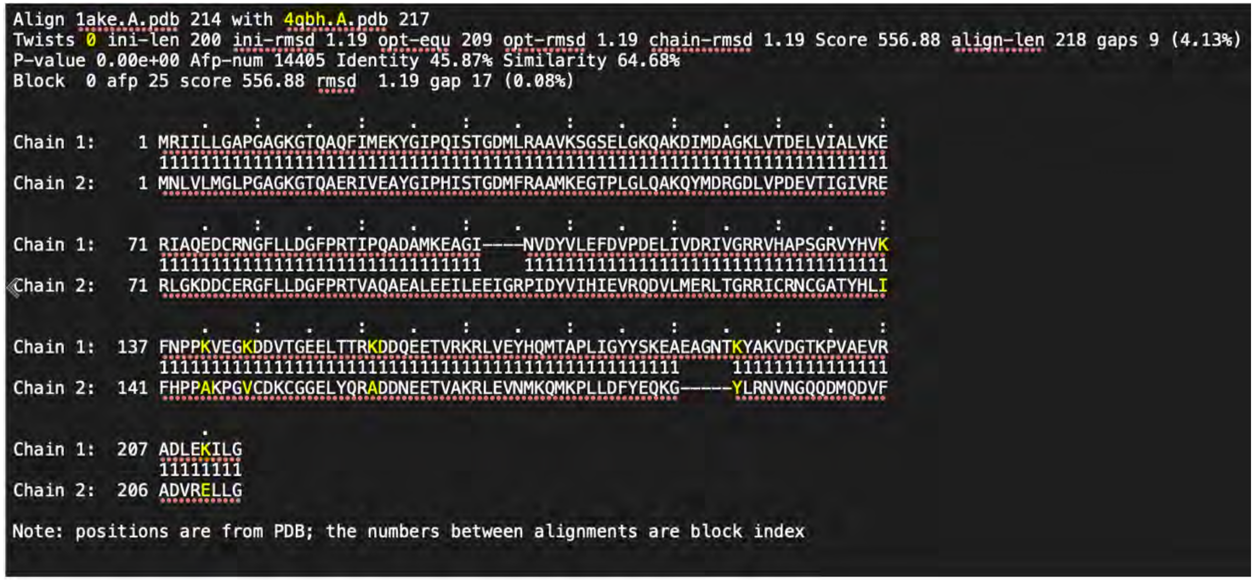


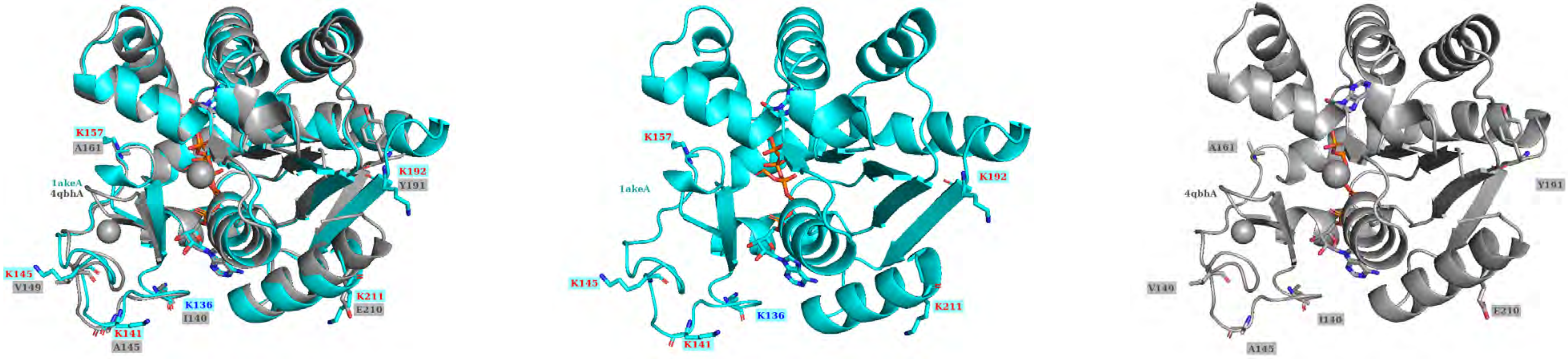

UniProt ID: P27142

PDB ID: 4QBI_A

```
                                     120       130       140       150       160       170
P69441_ESCHERICHIA_COLI              VDRIVGRRVHAPSGRVYHVKFNPPKVEGKDDVTGEELTTRKDDQEETVRKRLVEYHQMTA
P27142_GEOBACILLUS_STEAROTHERMOPHILUS MERLTGRRICRNCGATYHLIFHPPAKPGVCDKCGGELYQRADDNEATVANRLEVNMKQMK

                                     180       190       200       210
P69441_ESCHERICHIA_COLI              PLIGYYSKEAEAGNTKYAKVDGTKPVAEVRADLEKILG....
P27142_GEOBACILLUS_STEAROTHERMOPHILUS PLVDFYEQKGYLRN.....INGEQDMEKVFADIRELLGGLAR
```

Full sequences in supplemental file.

```
Align 1ake.A.pdb 214 with 4qbi.A.pdb 217
Twists 0 ini-len 200 ini-rmsd 1.36 opt-equ 209 opt-rmsd 1.22 chain-rmsd 1.36 Score 557.31 align-len 218 gaps 9 (4.13%)
P-value 0.00e+00 Afp-num 14086 Identity 46.33% Similarity 64.68%
Block  0 afp 25 score 557.31 rmsd  1.36 gap 17 (0.08%)

                  .    :    .    :    .    :    .    :    .    :    .    :    .    :
Chain 1:    1 MRIILLGAPGAGKGTQAQFIMEKYGIPQISTGDMLRAAVKSGSELGKQAKDIMDAGKLVTDELVIALVKE
              1111111111111111111111111111111111111111111111111111111111111111111111
Chain 2:    1 MNLVLMGLPGAGKGTQAEKIVEAYGIPHISTGDMFRAAMKEGTPLGLQAKQYMDRGDLVPDEVTIGIVRE

                  .    :    .    :    .    :    .    :    .    :    .    :    .    :
Chain 1:   71 RIAQEDCRNGFLLDGFPRTIPQADAMKEAGI----NVDYVLEFDVPDELIVDRIVGRRVHAPSGRVYHVK
              1111111111111111111111111111111    11111111111111111111111111111111111
Chain 2:   71 RLGKDDCERGFLLDGFPRTVAQAEALEEILEDIGRKIDYVIHIDVRQDVLMERLTGRRICRNCGATYHLI

                  .    :    .    :    .    :    .    :    .    :    .    :    .    :
Chain 1:  137 FNPPKVEGKDDVTGEELTTRKDDQEETVRKRLVEYHQMTAPLIGYYSKEAEAGNTKYAKVDGTKPVAEVR
              11111111111111111111111111111111111111111111111111     111111111111111
Chain 2:  141 FHPPAKPGVCDKCGGELYQRADDNEETVAKRLEVNMKQMKPLLDFYEQKG-----YLRNVNGEQDIEKVF

                  .
Chain 1:  207 ADLEKILG
              11111111
Chain 2:  206 ADVRELLG

Note: positions are from PDB; the numbers between alignments are block index
```

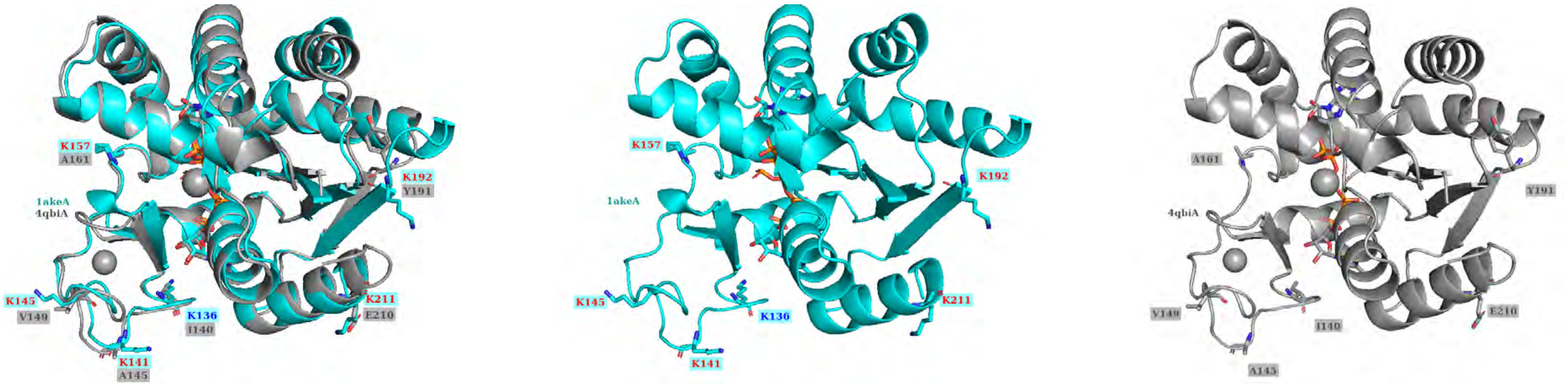

UniProt ID: P27144

PDB ID: 2AR7_B

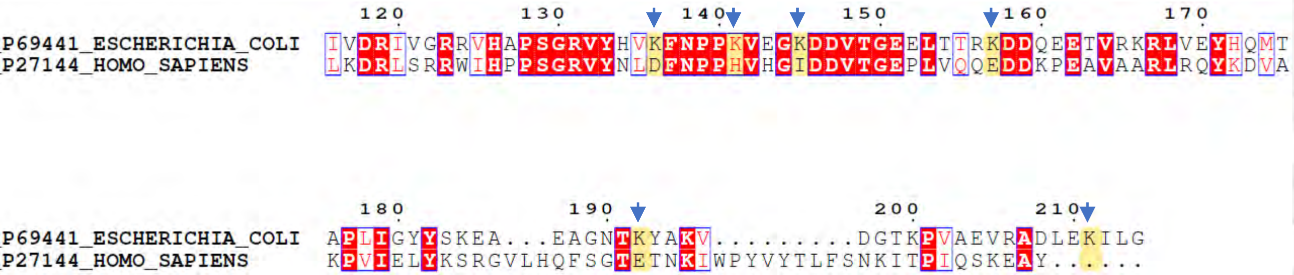


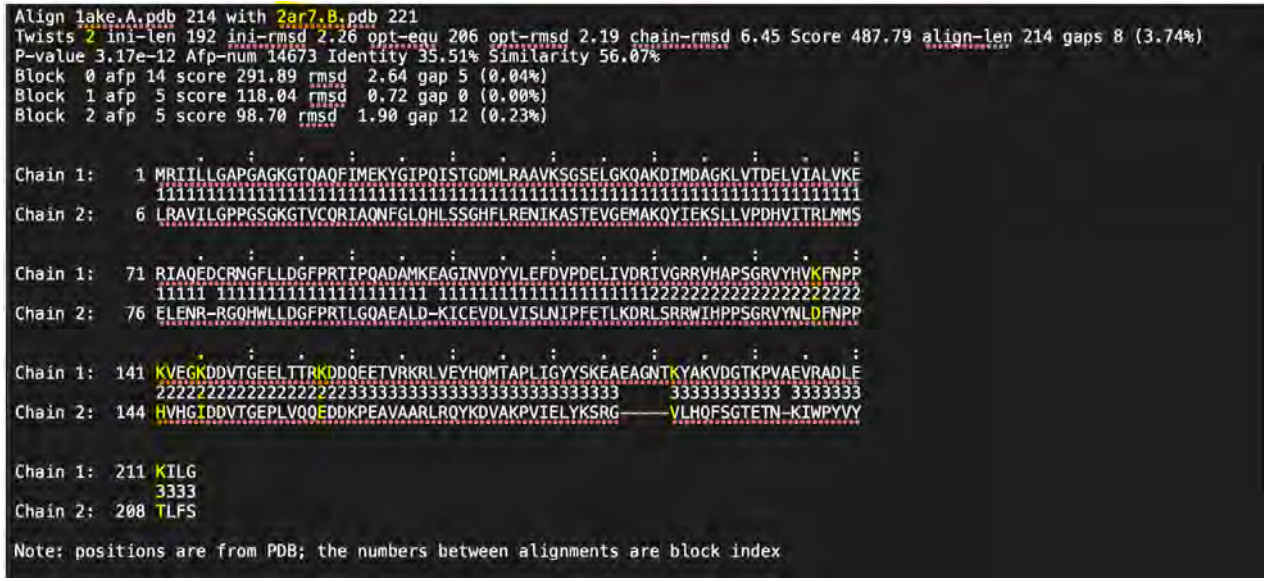


Full sequences in supplemental file.

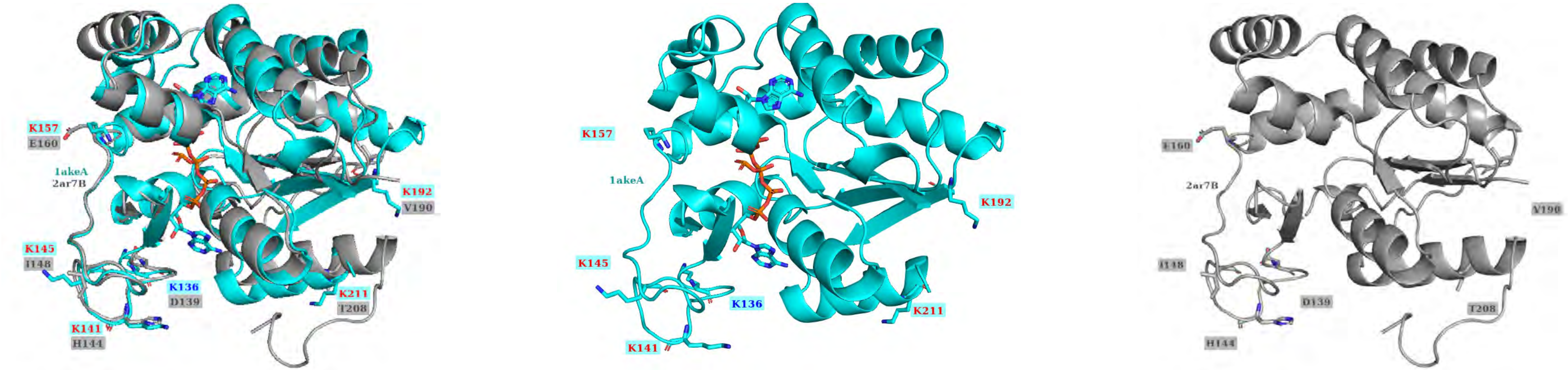

UniProt ID: P27144

PDB ID: 2BBW_B

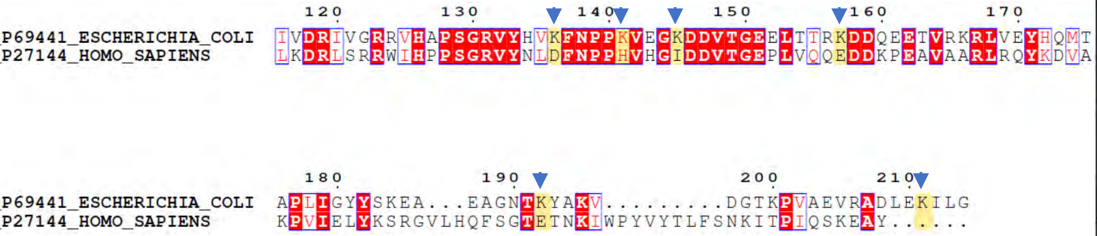


Full sequences in supplemental file.

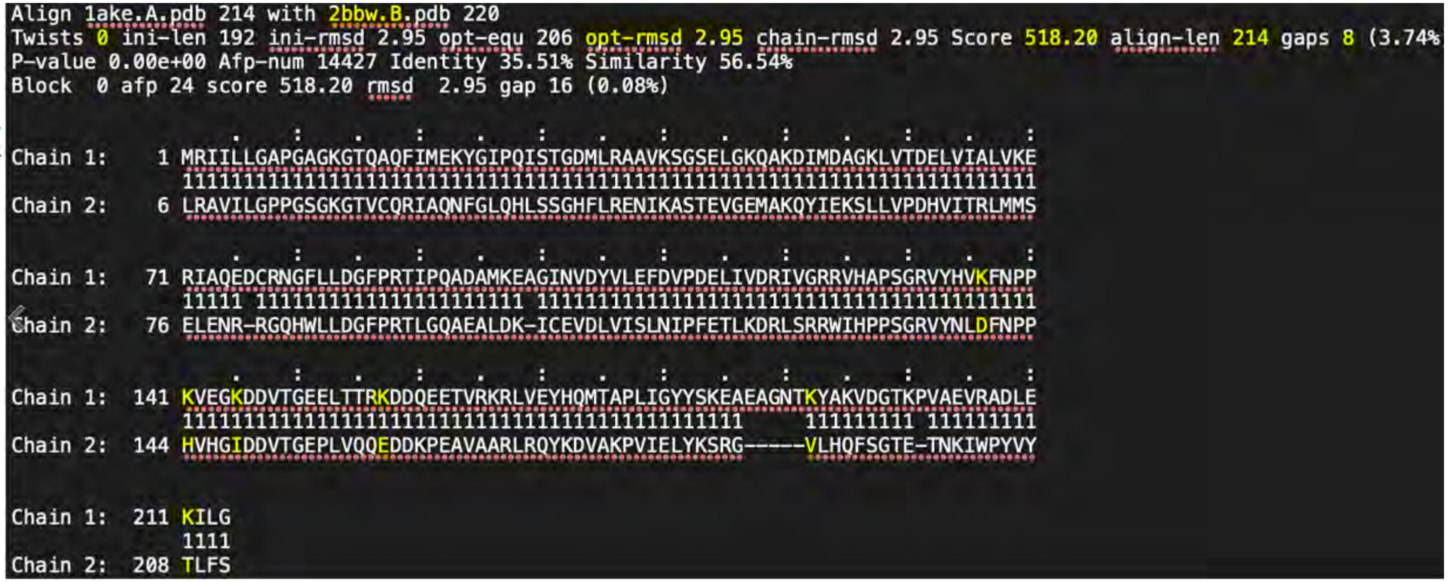


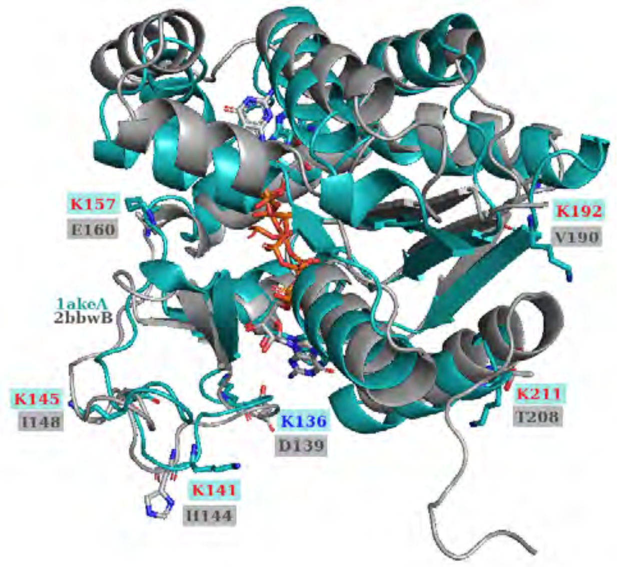


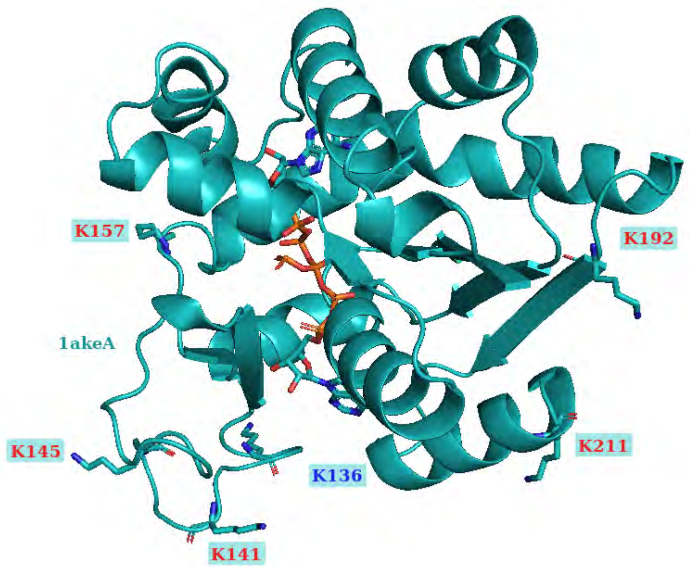


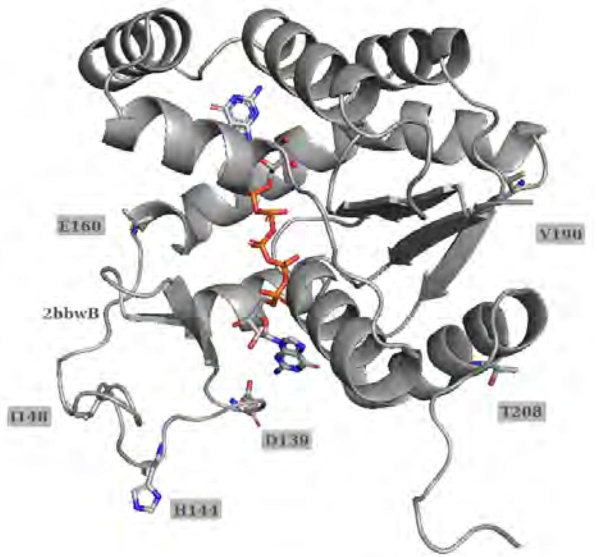

UniProt ID: P27144

PDB ID: 3NDP_B

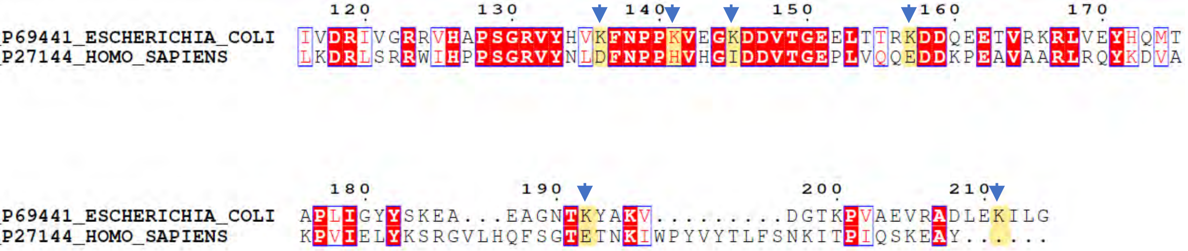


Full sequences in supplemental file.

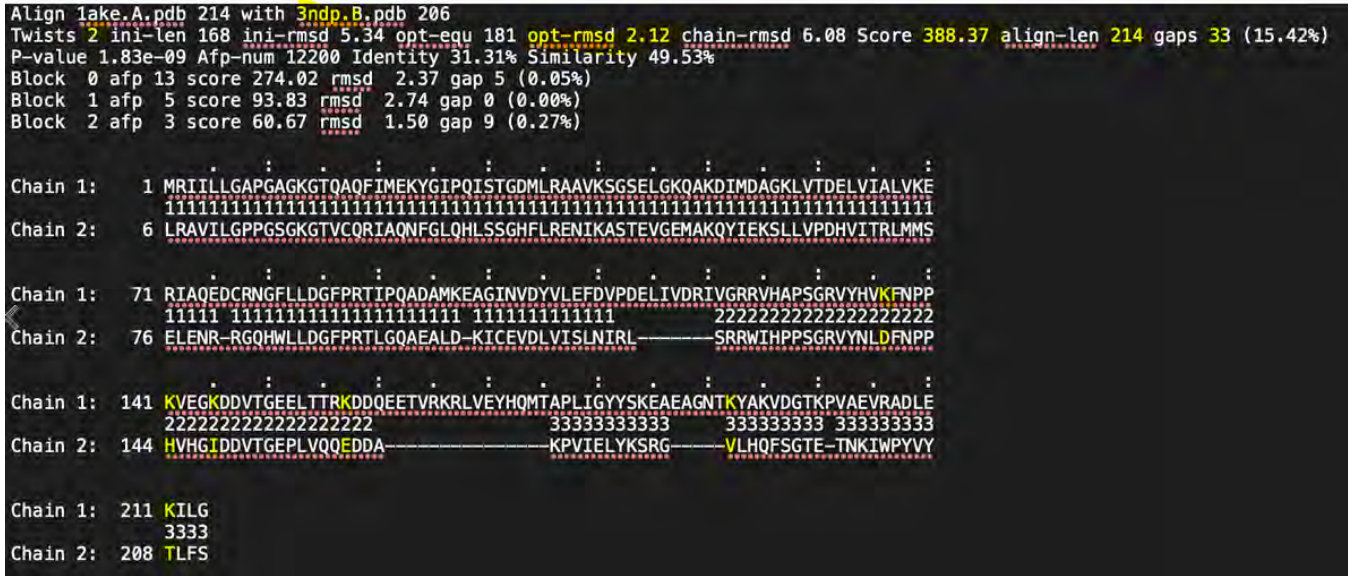


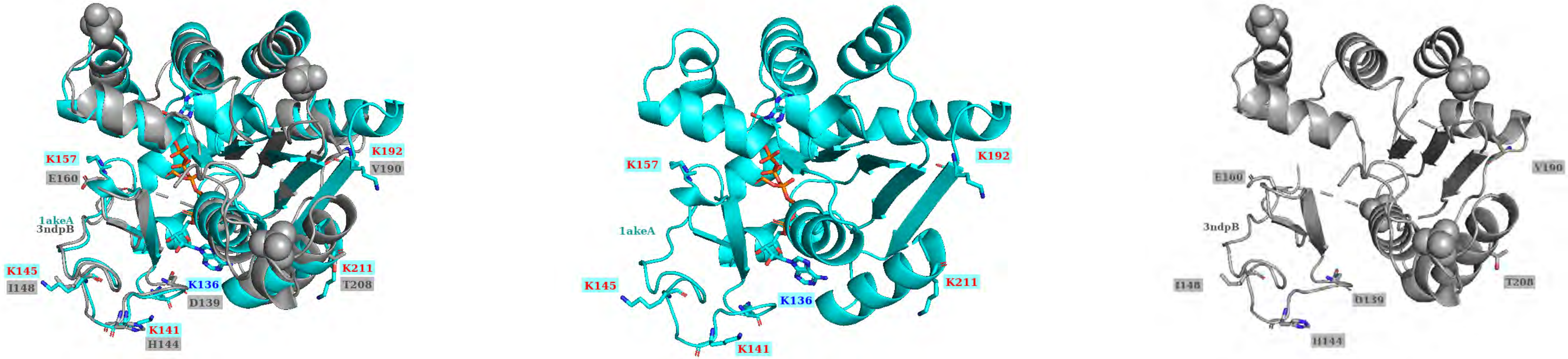

UniProt ID: P30085

PDB ID: 1TEV_A

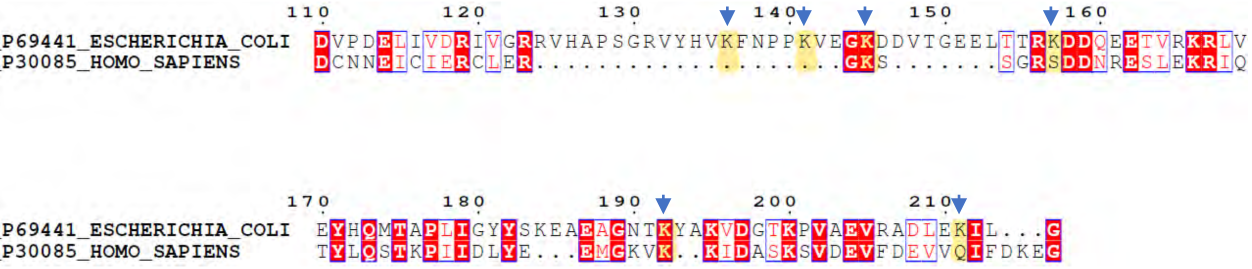


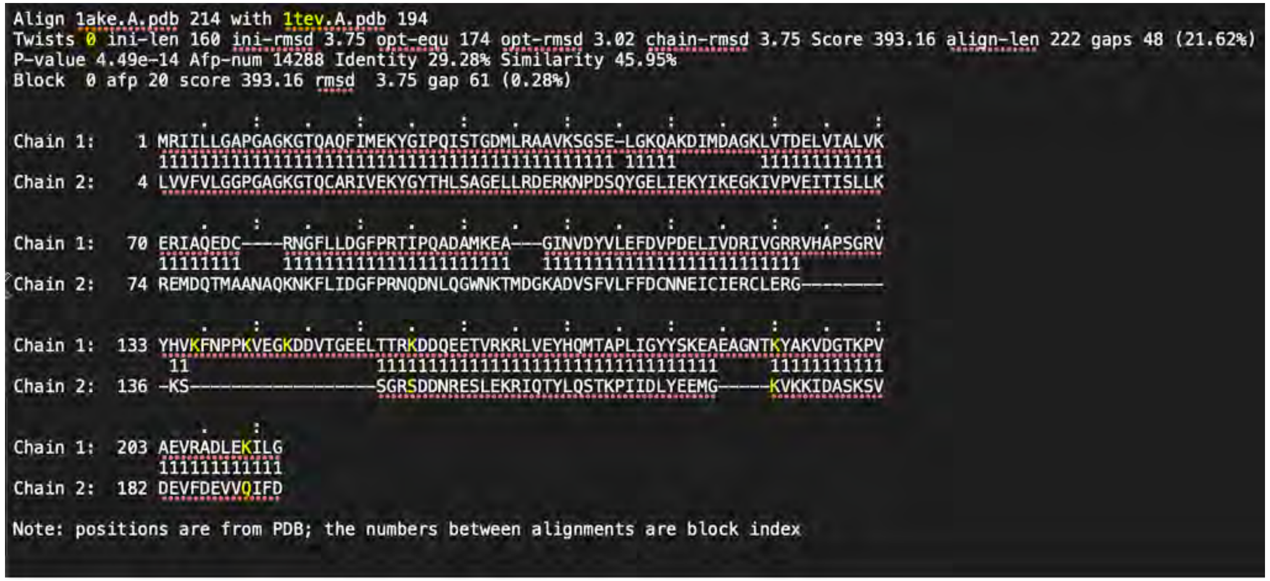

```
Align 1ake.A.pdb 214 with 1tev.A.pdb 194
Twists 0 ini-len 160 ini-rmsd 3.75 opt-equ 174 opt-rmsd 3.02 chain-rmsd 3.75 Score 393.16 align-len 222 gaps 48 (21.62%)
P-value 4.49e-14 Afp-num 14288 Identity 29.28% Similarity 45.95%
Block  0 afp 20 score 393.16 rmsd  3.75 gap 61 (0.28%)

               .    :    .    :    .    :    .    :    .    :    .    :    .    :
Chain 1:    1 MRIILLGAPGAGKGTQAQFIMEKYGIPQISTGDMLRAAVKSGSE-LGKQAKDIMDAGKLVTDELVIALVK
              111111111111111111111111111111111111111111111 11111         111111111111
Chain 2:    4 LVVFVLGGPGAGKGTQCARIVEKYGYTHLSAGELLRDERKNPDSQYGELIEKYIKEGKIVPVEITISLLK

               .    :    .    :    .    :    .    :    .    :    .    :    .    :
Chain 1:   70 ERIAQEDC----RNGFLLDGFPRTIPQADAMKEA---GINVDYVLEFDVPDELIVDRIVGRRVHAPSGRV
              11111111    1111111111111111111111   11111111111111111111111111
Chain 2:   74 REMDQTMAANAQKNKFLIDGFPRNQDNLQGWNKTMDGKADVSFVLFFDCNNEICIERCLERG--------

               .    :    .    :    .    :    .    :    .    :    .    :    .    :
Chain 1:  133 YHVKFNPPKVEGKDDVTGEELTTRKDDQEETVRKRLVEYHQMTAPLIGYYSKEAEAGNTKYAKVDGTKPV
               11                 111111111111111111111111111111111     11111111111
Chain 2:  136 -KS-----------------SGRSDDNRESLEKRIQTYLQSTKPIIDLYEEMG-----KVKKIDASKSV

               .    :
Chain 1:  203 AEVRADLEKILG
              111111111111
Chain 2:  182 DEVFDEVVQIFD

Note: positions are from PDB; the numbers between alignments are block index
```

Full sequences in supplemental file.

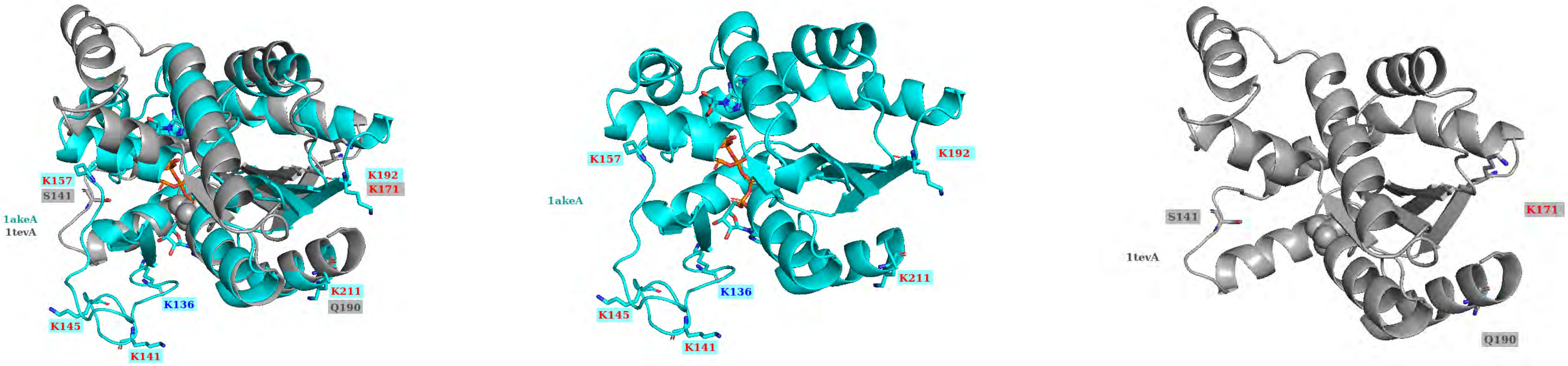

UniProt ID: P43188

PDB ID: 1ZAK_B

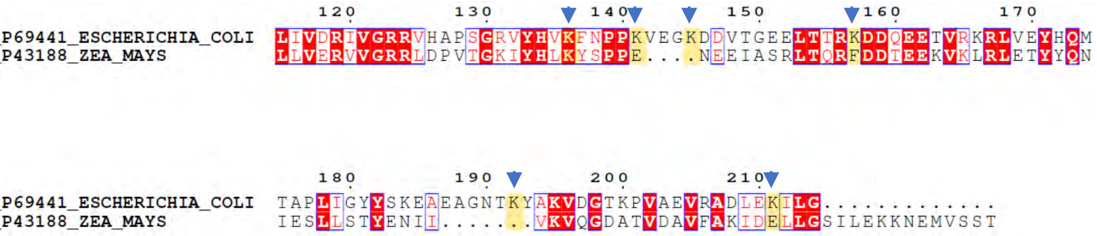


Full sequences in supplemental file.

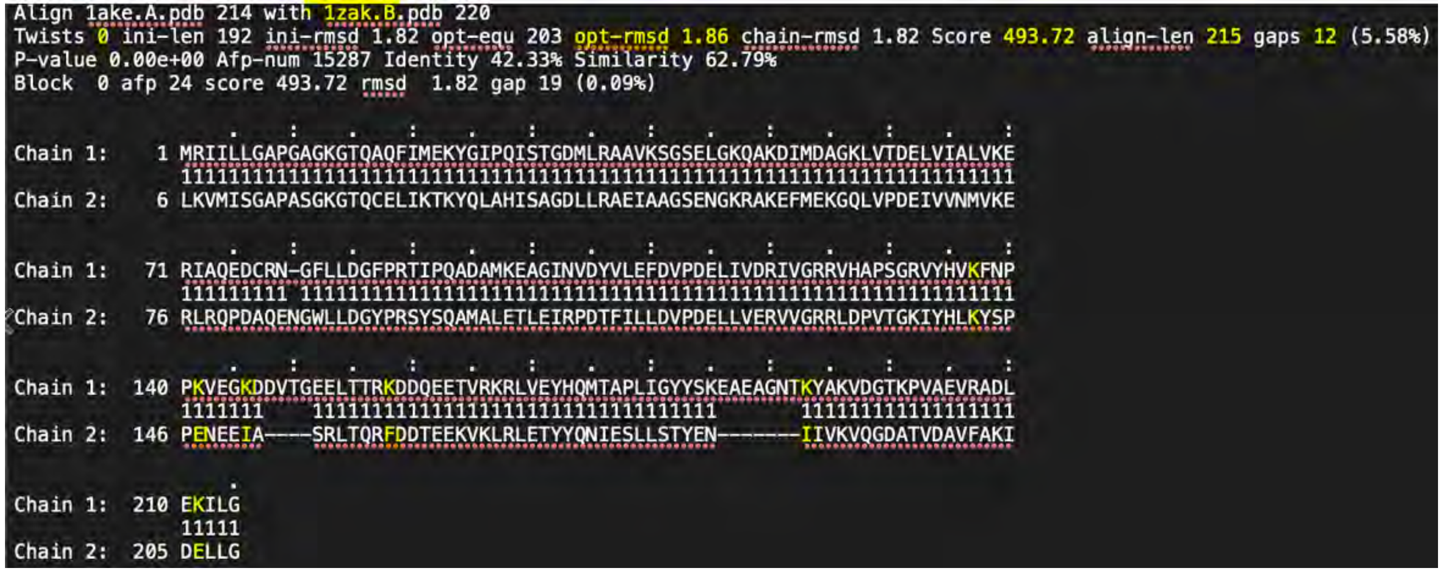

```
Align 1ake.A.pdb 214 with 1zak.B.pdb 220
Twists 0 ini-len 192 ini-rmsd 1.82 opt-equ 203 opt-rmsd 1.86 chain-rmsd 1.82 Score 493.72 align-len 215 gaps 12 (5.58%)
P-value 0.00e+00 Afp-num 15287 Identity 42.33% Similarity 62.79%
Block  0 afp 24 score 493.72 rmsd  1.82 gap 19 (0.09%)

                  .    :    .    :    .    :    .    :    .    :    .    :    .    :
Chain 1:    1 MRIILLGAPGAGKGTQAQFIMEKYGIPQISTGDMLRAAVKSGSELGKQAKDIMDAGKLVTDELVIALVKE
              1111111111111111111111111111111111111111111111111111111111111111111111
Chain 2:    6 LKVMISGAPASGKGTQCELIKTKYQLAHISAGDLLRAEIAAGSENGKRAKEFMEKGQLVPDEIVVNMVKE

                  .    :    .    :    .    :    .    :    .    :    .    :    .    :
Chain 1:   71 RIAQEDCRN-GFLLDGFPRTIPQADAMKEAGINVDYVLEFDVPDELIVDRIVGRRVHAPSGRVYHVKFNP
              111111111 111111111111111111111111111111111111111111111111111111111111
Chain 2:   76 RLRQPDAQENGWLLDGYPRSYSQAMALETLEIRPDTFILLDVPDELLVERVVGRRLDPVTGKIYHLKYSP

                  .    :    .    :    .    :    .    :    .    :    .    :    .    :
Chain 1:  140 PKVEGKDDVTGEELTTRKDDQEETVRKRLVEYHQMTAPLIGYYSKEAEAGNTKYAKVDGTKPVAEVRADL
              1111111    111111111111111111111111111111111       11111111111111111
Chain 2:  146 PENEEIA----SRLTQRFDDTEEKVKLRLETYYQNIESLLSTYEN-------IIVKVQGDATVDAVFAKI

                  .
Chain 1:  210 EKILG
              11111
Chain 2:  205 DELLG
```

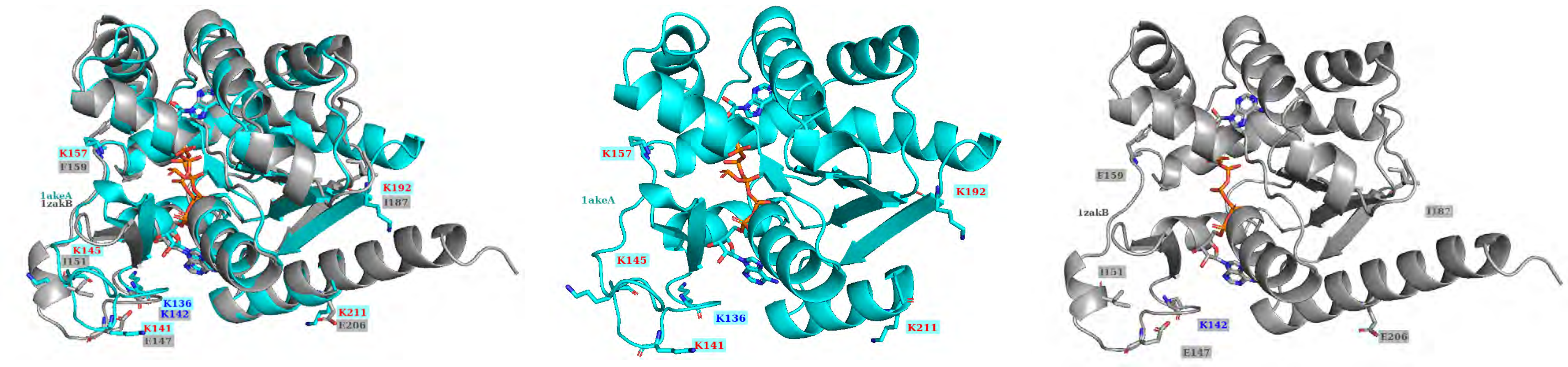

UniProt ID: P54819

PDB ID: 2C9Y_A

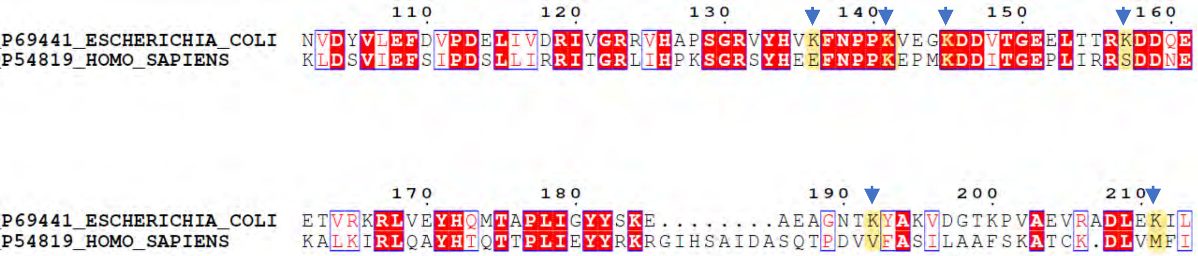


Full sequences in supplemental file.

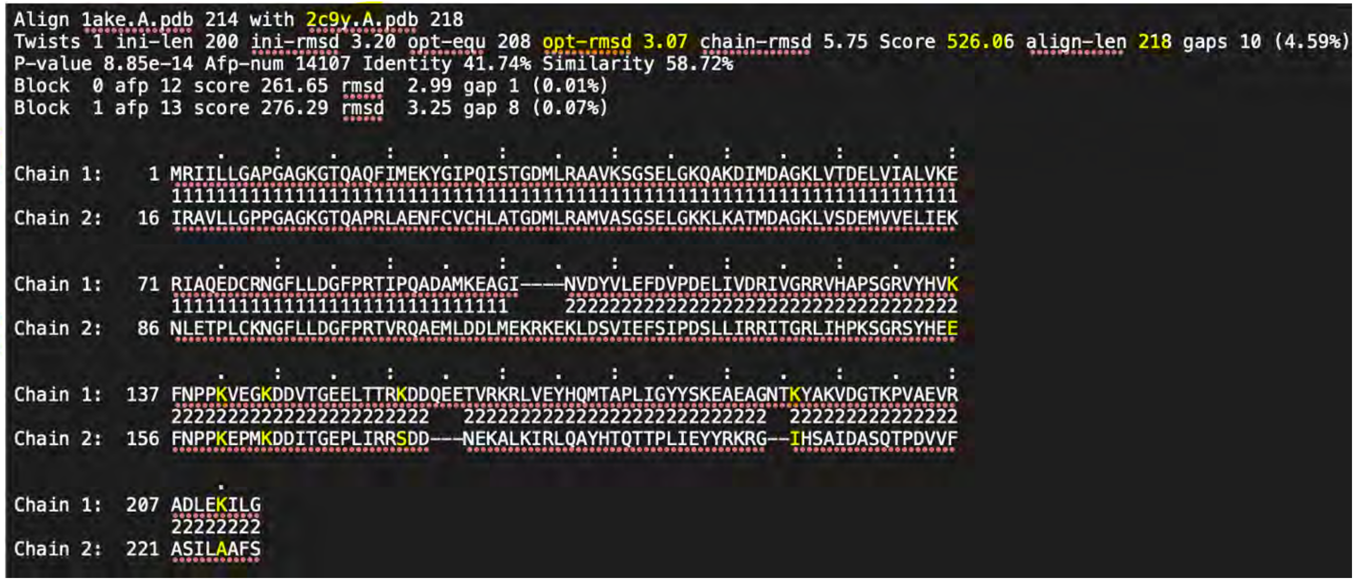


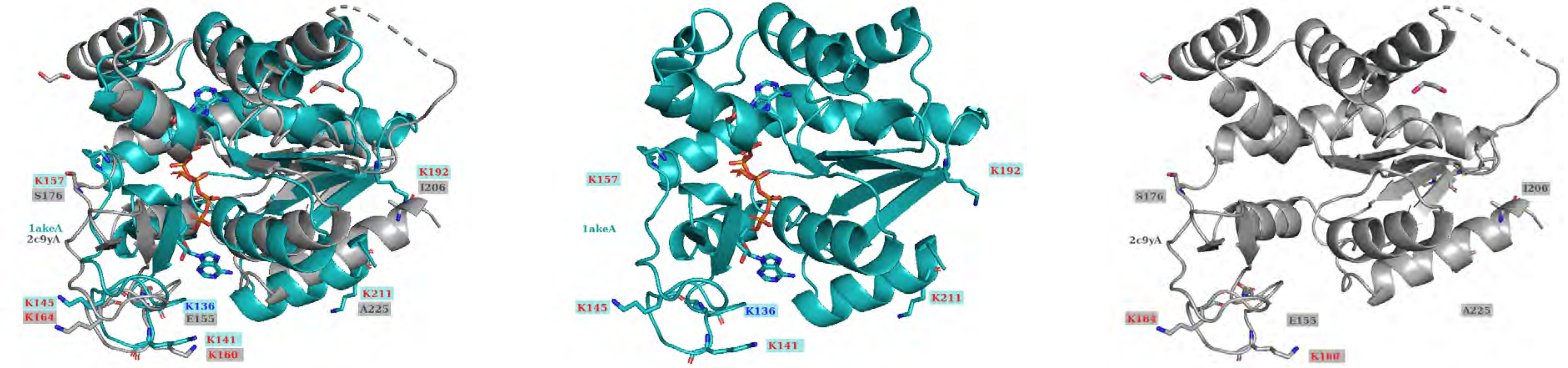

UniProt ID: P69441

PDB ID: 1ANK_A

```
Align 1ake.A.pdb 214 with 1ank.A.pdb 214
Twists 0 ini-len 208 ini-rmsd 0.46 opt-equ 214 opt-rmsd 0.45 chain-rmsd 0.46 Score 621.63 align-len 214 gaps 0 (0.00%)
P-value 0.00e+00 Afp-num 14054 Identity 100.00% Similarity 100.00%
Block  0 afp 26 score 621.63 rmsd  0.46 gap 0 (0.00%)

                  .    :    .    :    .    :    .    :    .    :    .    :    .    :
Chain 1:     1 MRIILLGAPGAGKGTQAQFIMEKYGIPQISTGDMLRAAVKSGSELGKQAKDIMDAGKLVTDELVIALVKE
               1111111111111111111111111111111111111111111111111111111111111111111111
Chain 2:     1 MRIILLGAPGAGKGTQAQFIMEKYGIPQISTGDMLRAAVKSGSELGKQAKDIMDAGKLVTDELVIALVKE

                  .    :    .    :    .    :    .    :    .    :    .    :    .    :
Chain 1:    71 RIAQEDCRNGFLLDGFPRTIPQADAMKEAGINVDYVLEFDVPDELIVDRIVGRRVHAPSGRVYHVKFNPP
               1111111111111111111111111111111111111111111111111111111111111111111111
Chain 2:    71 RIAQEDCRNGFLLDGFPRTIPQADAMKEAGINVDYVLEFDVPDELIVDRIVGRRVHAPSGRVYHVKFNPP

                  .    :    .    :    .    :    .    :    .    :    .    :    .    :
Chain 1:   141 KVEGKDDVTGEELTTRKDDQEETVRKRLVEYHQMTAPLIGYYSKEAEAGNTKYAKVDGTKPVAEVRADLE
               1111111111111111111111111111111111111111111111111111111111111111111111
Chain 2:   141 KVEGKDDVTGEELTTRKDDQEETVRKRLVEYHQMTAPLIGYYSKEAEAGNTKYAKVDGTKPVAEVRADLE

Chain 1:   211 KILG
               1111
Chain 2:   211 KILG

Note: positions are from PDB; the numbers between alignments are block index
```

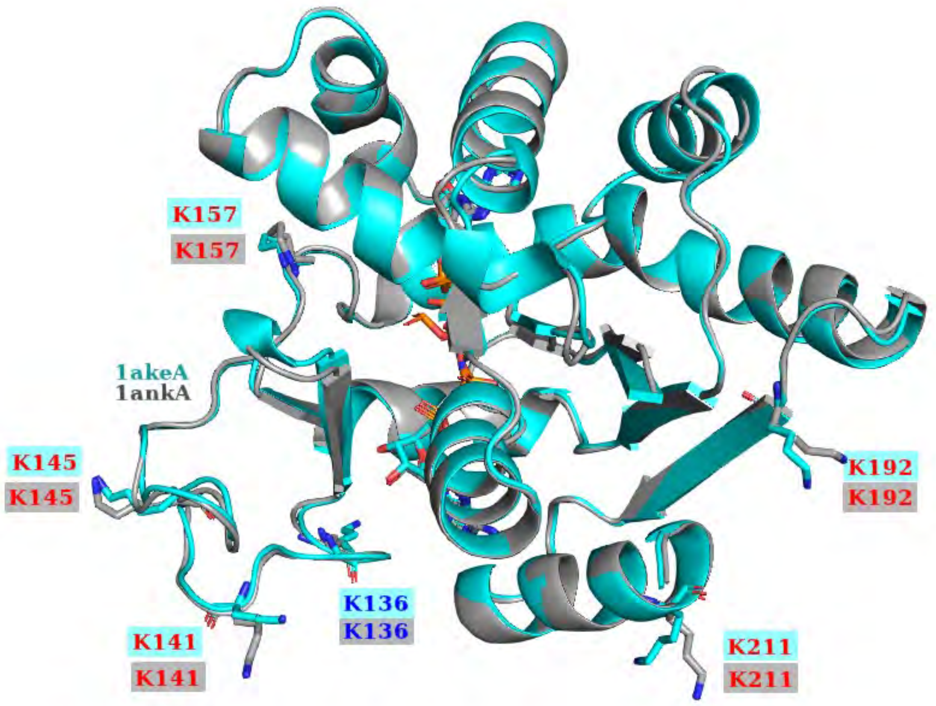

UniProt ID: P69441

PDB ID: 1E4V_A

```
Align 1ake.A.pdb 214 with 1e4v.A.pdb 214
Twists 0 ini-len 208 ini-rmsd 0.25 opt-equ 214 opt-rmsd 0.25 chain-rmsd 0.25 Score 623.32 align-len 214 gaps 0 (0.00%)
P-value 0.00e+00 Afp-num 14113 Identity 99.53% Similarity 99.53%
Block  0 afp 26 score 623.32 rmsd  0.25 gap 0 (0.00%)

                    .    :    .    :    .    :    .    :    .    :    .    :    .    :
Chain 1:    1 MRIILLGAPGAGKGTQAQFIMEKYGIPQISTGDMLRAAVKSGSELGKQAKDIMDAGKLVTDELVIALVKE
              1111111111111111111111111111111111111111111111111111111111111111111111
Chain 2:    1 MRIILLGAPVAGKGTQAQFIMEKYGIPQISTGDMLRAAVKSGSELGKQAKDIMDAGKLVTDELVIALVKE

                    .    :    .    :    .    :    .    :    .    :    .    :    .    :
Chain 1:   71 RIAQEDCRNGFLLDGFPRTIPQADAMKEAGINVDYVLEFDVPDELIVDRIVGRRVHAPSGRVYHVKFNPP
              1111111111111111111111111111111111111111111111111111111111111111111111
Chain 2:   71 RIAQEDCRNGFLLDGFPRTIPQADAMKEAGINVDYVLEFDVPDELIVDRIVGRRVHAPSGRVYHVKFNPP

                    .    :    .    :    .    :    .    :    .    :    .    :    .    :
Chain 1:  141 KVEGKDDVTGEELTTRKDDQEETVRKRLVEYHQMTAPLIGYYSKEAEAGNTKYAKVDGTKPVAEVRADLE
              1111111111111111111111111111111111111111111111111111111111111111111111
Chain 2:  141 KVEGKDDVTGEELTTRKDDQEETVRKRLVEYHQMTAPLIGYYSKEAEAGNTKYAKVDGTKPVAEVRADLE

Chain 1:  211 KILG
              1111
Chain 2:  211 KILG

Note: positions are from PDB; the numbers between alignments are block index
```

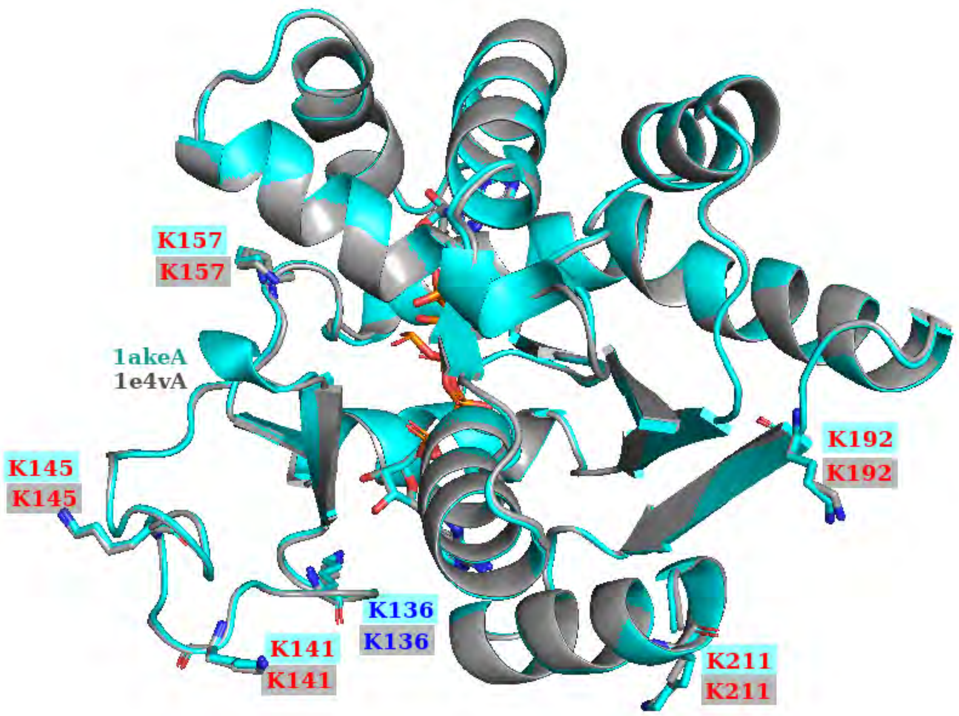

UniProt ID: P69441

PDB ID: 1E4Y_A

```
Align 1ake.A.pdb 214 with 1e4y.A.pdb 214
Twists 0 ini-len 208 ini-rmsd 0.91 opt-equ 214 opt-rmsd 0.93 chain-rmsd 0.91 Score 613.00 align-len 214 gaps 0 (0.00%)
P-value 0.00e+00 Afp-num 14164 Identity 99.07% Similarity 99.07%
Block  0 afp 26 score 613.00 rmsd  0.91 gap 2 (0.01%)

                .    :    .    :    .    :    .    :    .    :    .    :    .    :
Chain 1:    1 MRIILLGAPGAGKGTQAQFIMEKYGIPQISTGDMLRAAVKSGSELGKQAKDIMDAGKLVTDELVIALVKE
              1111111111111111111111111111111111111111111111111111111111111111111111
Chain 2:    1 MRIILLGALVAGKGTQAQFIMEKYGIPQISTGDMLRAAVKSGSELGKQAKDIMDAGKLVTDELVIALVKE

                .    :    .    :    .    :    .    :    .    :    .    :    .    :
Chain 1:   71 RIAQEDCRNGFLLDGFPRTIPQADAMKEAGINVDYVLEFDVPDELIVDRIVGRRVHAPSGRVYHVKFNPP
              1111111111111111111111111111111111111111111111111111111111111111111111
Chain 2:   71 RIAQEDCRNGFLLDGFPRTIPQADAMKEAGINVDYVLEFDVPDELIVDRIVGRRVHAPSGRVYHVKFNPP

                .    :    .    :    .    :    .    :    .    :    .    :    .    :
Chain 1:  141 KVEGKDDVTGEELTTRKDDQEETVRKRLVEYHQMTAPLIGYYSKEAEAGNTKYAKVDGTKPVAEVRADLE
              1111111111111111111111111111111111111111111111111111111111111111111111
Chain 2:  141 KVEGKDDVTGEELTTRKDDQEETVRKRLVEYHQMTAPLIGYYSKEAEAGNTKYAKVDGTKPVAEVRADLE

Chain 1:  211 KILG
              1111
Chain 2:  211 KILG

Note: positions are from PDB; the numbers between alignments are block index
```

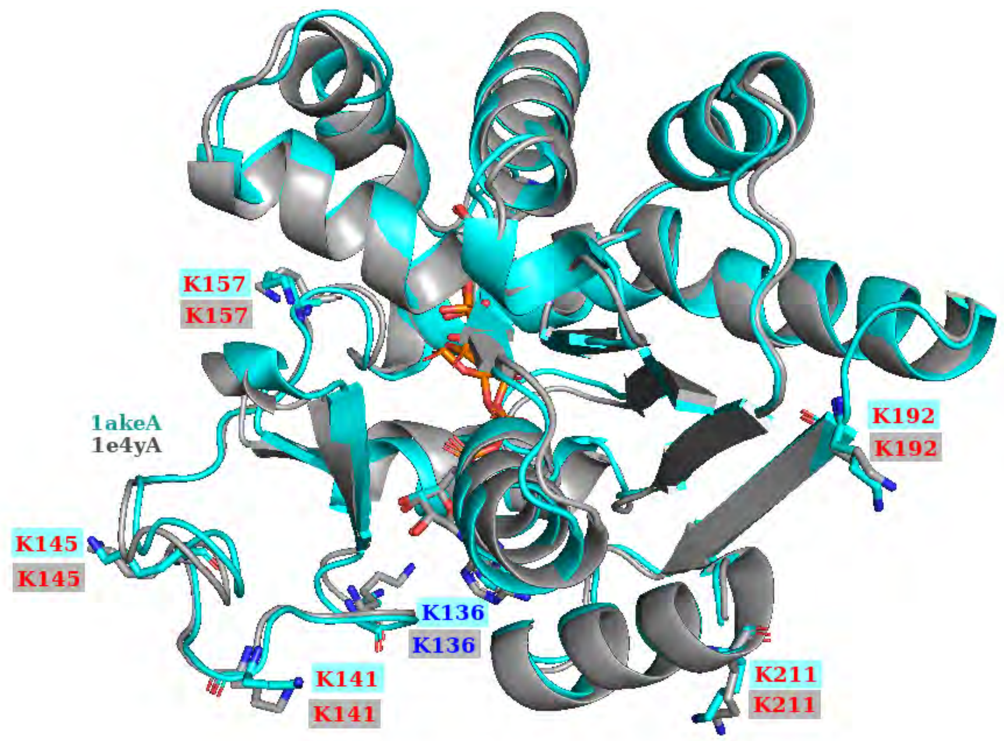

UniProt ID: P69441

PDB ID: 2ECK_A

```
Align 1ake.A.pdb 214 with 2eck.A.pdb 214
Twists 0 ini-len 208 ini-rmsd 0.28 opt-equ 214 opt-rmsd 0.28 chain-rmsd 0.28 Score 622.90 align-len 214 gaps 0 (0.00%)
P-value 0.00e+00 Afp-num 14158 Identity 100.00% Similarity 100.00%
Block  0 afp 26 score 622.90 rmsd  0.28 gap 0 (0.00%)

                      .    :    .    :    .    :    .    :    .    :    .    :    .    :
Chain 1:    1 MRIILLGAPGAGKGTQAQFIMEKYGIPQISTGDMLRAAVKSGSELGKQAKDIMDAGKLVTDELVIALVKE
              1111111111111111111111111111111111111111111111111111111111111111111111
Chain 2:    1 MRIILLGAPGAGKGTQAQFIMEKYGIPQISTGDMLRAAVKSGSELGKQAKDIMDAGKLVTDELVIALVKE

                      .    :    .    :    .    :    .    :    .    :    .    :    .    :
Chain 1:   71 RIAQEDCRNGFLLDGFPRTIPQADAMKEAGINVDYVLEFDVPDELIVDRIVGRRVHAPSGRVYHVKFNPP
              1111111111111111111111111111111111111111111111111111111111111111111111
Chain 2:   71 RIAQEDCRNGFLLDGFPRTIPQADAMKEAGINVDYVLEFDVPDELIVDRIVGRRVHAPSGRVYHVKFNPP

                      .    :    .    :    .    :    .    :    .    :    .    :    .    :
Chain 1:  141 KVEGKDDVTGEELTTRKDDQEETVRKRLVEYHQMTAPLIGYYSKEAEAGNTKYAKVDGTKPVAEVRADLE
              1111111111111111111111111111111111111111111111111111111111111111111111
Chain 2:  141 KVEGKDDVTGEELTTRKDDQEETVRKRLVEYHQMTAPLIGYYSKEAEAGNTKYAKVDGTKPVAEVRADLE

Chain 1:  211 KILG
              1111
Chain 2:  211 KILG

Note: positions are from PDB; the numbers between alignments are block index
```

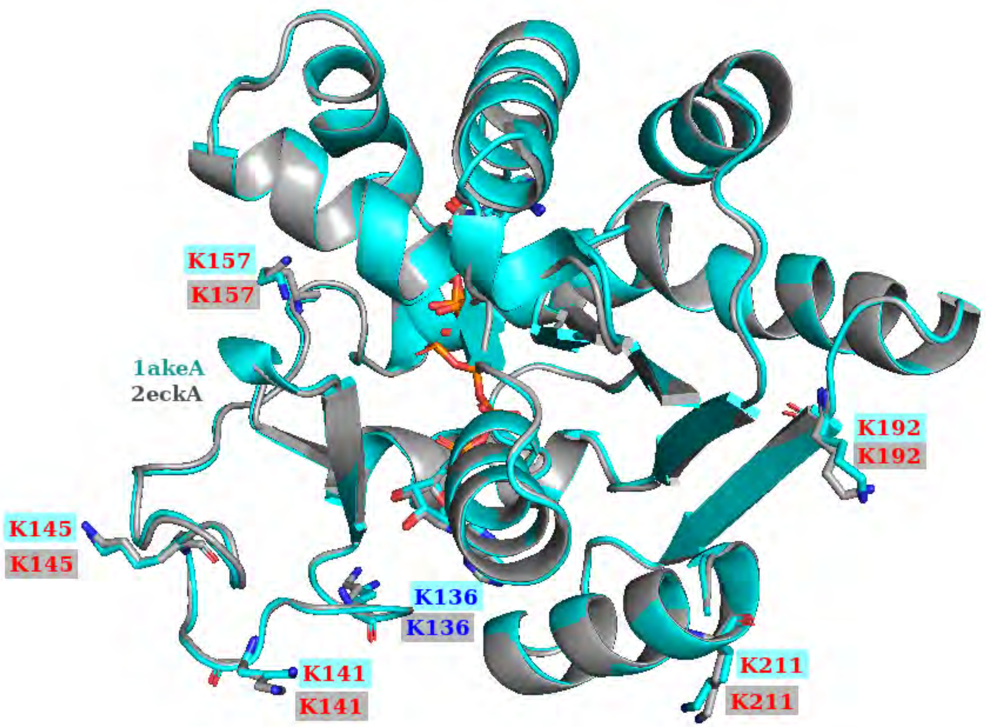

UniProt ID: P69441

PDB ID: 3HPQ_B

```
Align 1ake.A.pdb 214 with 3hpq.B.pdb 214
Twists 0 ini-len 208 ini-rmsd 0.33 opt-equ 214 opt-rmsd 0.35 chain-rmsd 0.33 Score 623.00 align-len 214 gaps 0 (0.00%)
P-value 0.00e+00 Afp-num 14138 Identity 100.00% Similarity 100.00%
Block  0 afp 26 score 623.00 rmsd  0.33 gap 0 (0.00%)

                    .    :    .    :    .    :    .    :    .    :    .    :    .    :
Chain 1:    1 MRIILLGAPGAGKGTQAQFIMEKYGIPQISTGDMLRAAVKSGSELGKQAKDIMDAGKLVTDELVIALVKE
              1111111111111111111111111111111111111111111111111111111111111111111111
Chain 2:    1 MRIILLGAPGAGKGTQAQFIMEKYGIPQISTGDMLRAAVKSGSELGKQAKDIMDAGKLVTDELVIALVKE

                    .    :    .    :    .    :    .    :    .    :    .    :    .    :
Chain 1:   71 RIAQEDCRNGFLLDGFPRTIPQADAMKEAGINVDYVLEFDVPDELIVDRIVGRRVHAPSGRVYHVKFNPP
              1111111111111111111111111111111111111111111111111111111111111111111111
Chain 2:   71 RIAQEDCRNGFLLDGFPRTIPQADAMKEAGINVDYVLEFDVPDELIVDRIVGRRVHAPSGRVYHVKFNPP

                    .    :    .    :    .    :    .    :    .    :    .    :    .    :
Chain 1:  141 KVEGKDDVTGEELTTRKDDQEETVRKRLVEYHQMTAPLIGYYSKEAEAGNTKYAKVDGTKPVAEVRADLE
              1111111111111111111111111111111111111111111111111111111111111111111111
Chain 2:  141 KVEGKDDVTGEELTTRKDDQEETVRKRLVEYHQMTAPLIGYYSKEAEAGNTKYAKVDGTKPVAEVRADLE


Chain 1:  211 KILG
              1111
Chain 2:  211 KILG
```

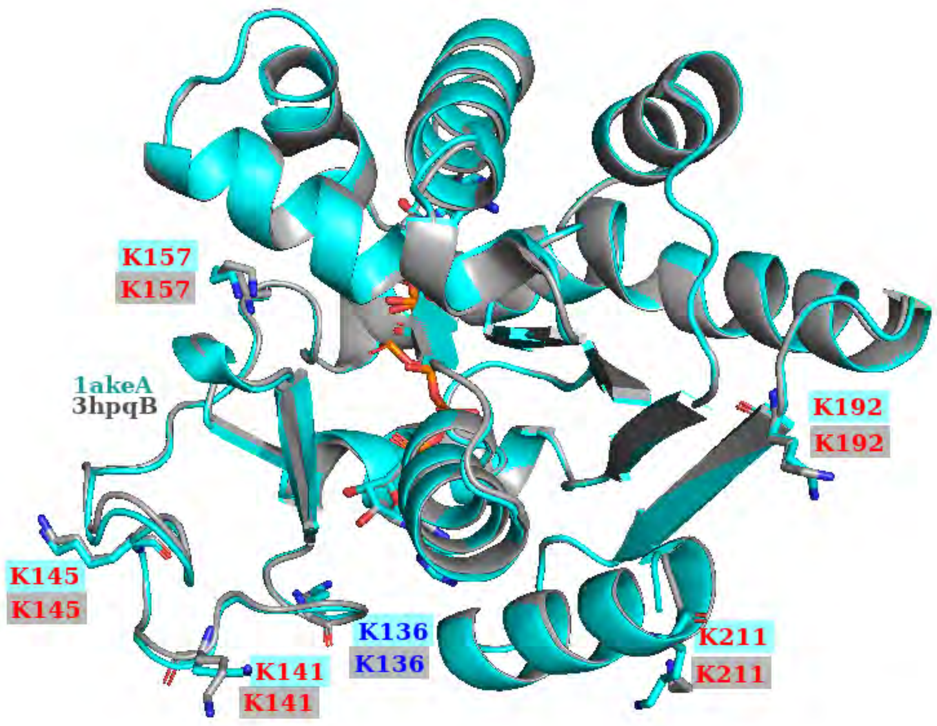

UniProt ID: P69441

PDB ID: 3HPR_A

```
Align 1ake.A.pdb 214 with 3hpr.A.pdb 214
Twists 0 ini-len 208 ini-rmsd 0.30 opt-equ 214 opt-rmsd 0.31 chain-rmsd 0.30 Score 623.16 align-len 214 gaps 0 (0.00%)
P-value 0.00e+00 Afp-num 14189 Identity 99.53% Similarity 99.53%
Block  0 afp 26 score 623.16 rmsd  0.30 gap 0 (0.00%)

                   .    :    .    :    .    :    .    :    .    :    .    :    .    :
Chain 1:     1 MRIILLGAPGAGKGTQAQFIMEKYGIPQISTGDMLRAAVKSGSELGKQAKDIMDAGKLVTDELVIALVKE
               1111111111111111111111111111111111111111111111111111111111111111111111
Chain 2:     1 MRIILLGAPGAGKGTQAQFIMEKYGIPQISTGDMLRAAVKSGSELGKQAKDIMDAGKLVTDELVIALVKE

                   .    :    .    :    .    :    .    :    .    :    .    :    .    :
Chain 1:    71 RIAQEDCRNGFLLDGFPRTIPQADAMKEAGINVDYVLEFDVPDELIVDRIVGRRVHAPSGRVYHVKFNPP
               1111111111111111111111111111111111111111111111111111111111111111111111
Chain 2:    71 RIAQEDCRNGFLLDGFPRTIPQADAMKEAGINVDYVLEFDVPDELIVDRIVGRRVHAPSGRVYHVKFNPP

                   .    :    .    :    .    :    .    :    .    :    .    :    .    :
Chain 1:   141 KVEGKDDVTGEELTTRKDDQEETVRKRLVEYHQMTAPLIGYYSKEAEAGNTKYAKVDGTKPVAEVRADLE
               1111111111111111111111111111111111111111111111111111111111111111111111
Chain 2:   141 KVEGKDDGTGEELTTRKDDQEETVRKRLVEYHQMTAPLIGYYSKEAEAGNTKYAKVDGTKPVAEVRADLE

Chain 1:   211 KILG
               1111
Chain 2:   211 KILG

Note: positions are from PDB; the numbers between alignments are block index
```

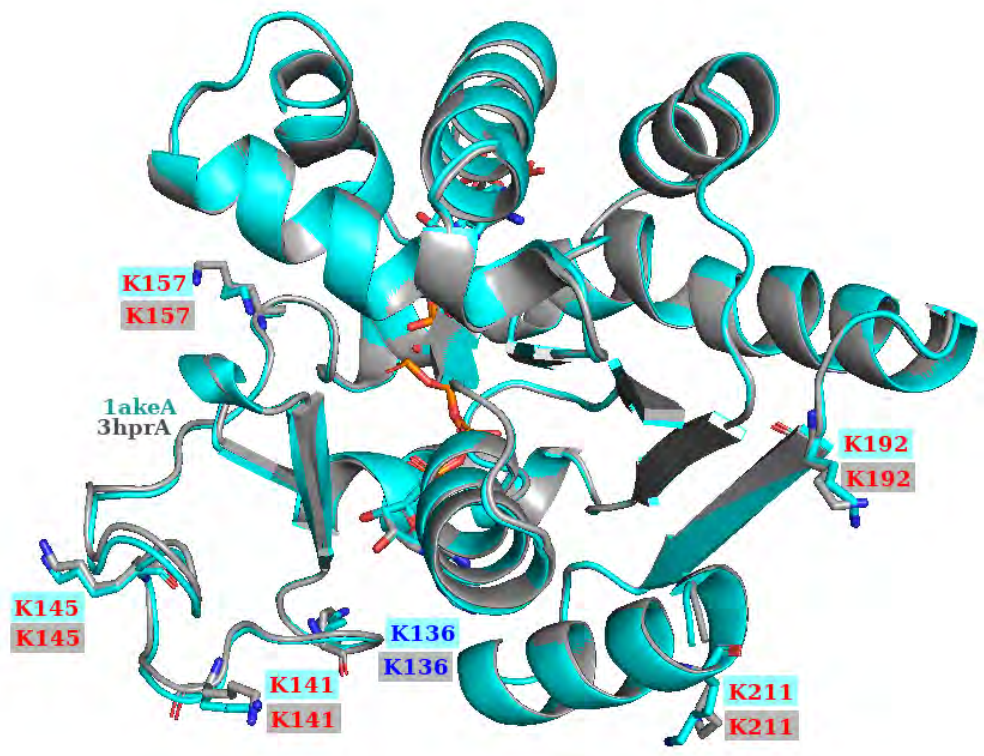

UniProt ID: P69441

PDB ID: 4AKE_B

```
Align 1ake.A.pdb 214 with 4ake.B.pdb 214
Twists 2 ini-len 208 ini-rmsd 2.88 opt-equ 210 opt-rmsd 2.35 chain-rmsd 6.94 Score 592.04 align-len 217 gaps 7 (3.23%)
P-value 1.11e-14 Afp-num 14043 Identity 92.63% Similarity 93.09%
Block  0 afp 14 score 314.17 rmsd  3.72 gap 2 (0.02%)
Block  1 afp  5 score 119.10 rmsd  0.53 gap 0 (0.00%)
Block  2 afp  7 score 165.80 rmsd  1.35 gap 0 (0.00%)

                    .    :    .    :    .    :    .    :    .    :    .    :    .    :
Chain 1:    1 MRIILLGAPGAGKGTQAQFIMEKYGIPQISTGDMLRAAVKSG---SELGKQAKDIMDAGKLVTDELVIAL
              111111111111111111111111111111111111111111   11111111111    1111111111
Chain 2:    1 MRIILLGAPGAGKGTQAQFIMEKYGIPQISTGDMLRAAVKSGSELGKQAKDIMDAGK---LVTDELVIAL

                    .    :    .    :    .    :    .    :    .    :    .    :    .    :
Chain 1:   68 VKERIAQEDCRNGFLLDGFPRTIPQADAMKEAGINVDYVLEFDVPDELIVDRIVGRRVHAPSGRVYHVKF
              1111111111111111111111111111111111111111111111111112222222222222222222
Chain 2:   68 VKERIAQEDCRNGFLLDGFPRTIPQADAMKEAGINVDYVLEFDVPDELIVDRIVGRRVHAPSGRVYHVKF

                    .    :    .    :    .    :    .    :    .    :    .    :    .    :
Chain 1:  138 NPPKVEGKDDVTGEELTTRKDDQEETVRKRLVEYHQMTAPLIGYYSKEAEAGNTKYAKVDGTKPVAEVRA
              2222222222222222222233333333333333333333333333333333333333333333333333
Chain 2:  138 NPPKVEGKDDVTGEELTTRKDDQEETVRKRLVEYHQMTAPLIGYYSKEAEAGNTKYAKVDGTKPVAEVRA

                    .
Chain 1:  208 DLEKILG
              3333333
Chain 2:  208 DLEKILG
```

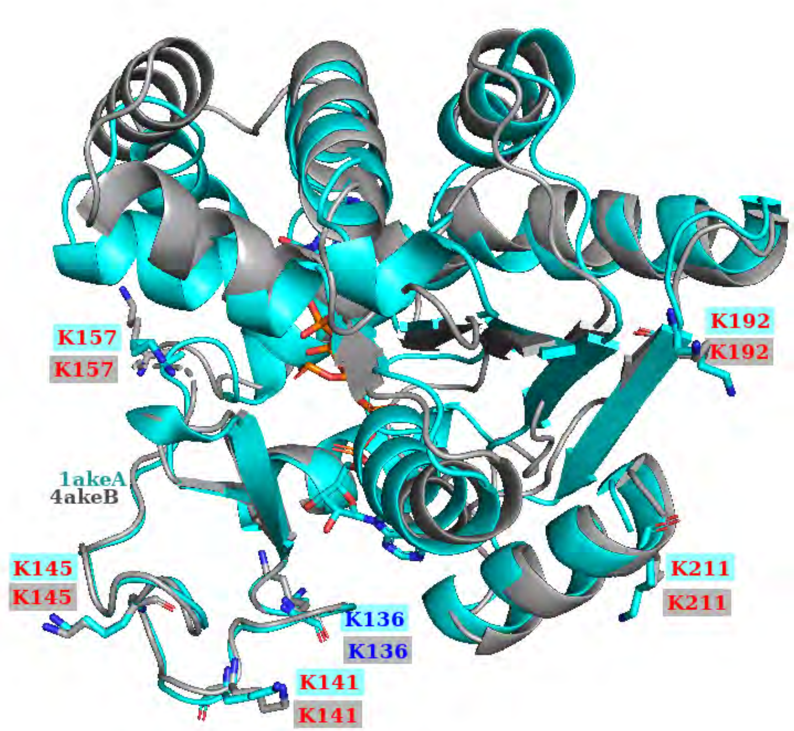

UniProt ID: P69441

PDB ID: 4X8H_A

```
Align 1ake.A.pdb 214 with 4x8h.A.pdb 214
Twists 2 ini-len 208 ini-rmsd 2.92 opt-equ 210 opt-rmsd 2.37 chain-rmsd 7.08 Score 583.05 align-len 217 gaps 7 (3.23%)
P-value 2.23e-14 Afp-num 13929 Identity 92.63% Similarity 93.09%
Block  0 afp 14 score 311.84 rmsd  3.74 gap 2 (0.02%)
Block  1 afp  5 score 119.03 rmsd  0.68 gap 0 (0.00%)
Block  2 afp  7 score 165.54 rmsd  1.29 gap 0 (0.00%)

                  .    :    .    :    .    :    .    :    .    :    .    :    .    :
Chain 1:    1 MRIILLGAPGAGKGTQAQFIMEKYGIPQISTGDMLRAAVKSG---SELGKQAKDIMDAGKLVTDELVIAL
              11111111111111111111111111111111111111111    11111111111   11111111111
Chain 2:    1 MRIILLGAPGAGKGTQAQFIMEKYGIPQISTGDMLRAAVKSGSELGKQAKDIMDAG---KLVTDELVIAL

                  .    :    .    :    .    :    .    :    .    :    .    :    .    :
Chain 1:   68 VKERIAQEDCRNGFLLDGFPRTIPQADAMKEAGINVDYVLEFDVPDELIVDRIVGRRVHAPSGRVYHVKF
              11111111111111111111111111111111111111111111111111111122222222222222
Chain 2:   68 VKERIAQEDCRNGFLLDGFPRTIPQADAMKEAGINVDYVLEFDVPDELIVDRIVGRRVHAPSGRVYHVKF

                  .    :    .    :    .    :    .    :    .    :    .    :    .    :
Chain 1:  138 NPPKVEGKDDVTGEELTTRKDDQEETVRKRLVEYHQMTAPLIGYYSKEAEAGNTKYAKVDGTKPVAEVRA
              2222222222222222222223333333333333333333333333333333333333333333333333
Chain 2:  138 NPPKVEGKDDVTGEELTTRKDDQEETVRKRLVEYHQMTAALIGYYSKEAEAGNTKYAKVDGTKPVAEVRA

                  .
Chain 1:  208 DLEKILG
              3333333
Chain 2:  208 DLEKILG

Note: positions are from PDB; the numbers between alignments are block index
```

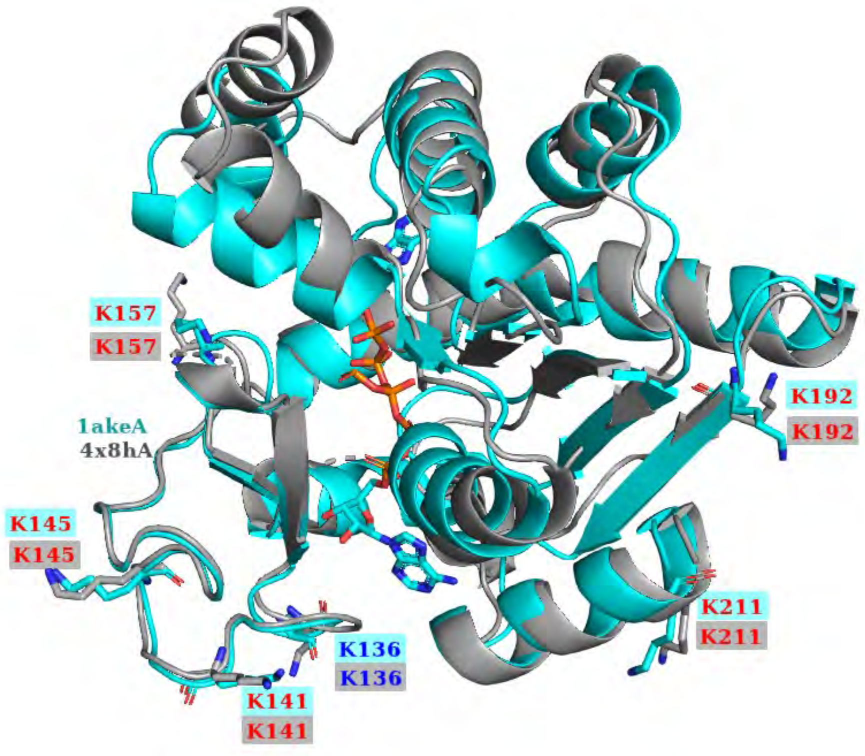

UniProt ID: P69441
PDB ID: 4X8L_B

```
Align 1ake.A.pdb 214 with 4x8l.B.pdb 214
Twists 0 ini-len 208 ini-rmsd 0.39 opt-equ 214 opt-rmsd 0.42 chain-rmsd 0.39 Score 622.75 align-len 214 gaps 0 (0.00%)
P-value 0.00e+00 Afp-num 14108 Identity 99.53% Similarity 99.53%
Block  0 afp 26 score 622.75 rmsd  0.39 gap 0 (0.00%)

                    .    :    .    :    .    :    .    :    .    :    .    :    .    :
Chain 1:    1 MRIILLGAPGAGKGTQAQFIMEKYGIPQISTGDMLRAAVKSGSELGKQAKDIMDAGKLVTDELVIALVKE
              1111111111111111111111111111111111111111111111111111111111111111111111
Chain 2:    1 MRIILLGAPGAGKGTQAQFIMEKYGIPQISTGDMLRAAVKSGSELGKQAKDIMDAGKLVTDELVIALVKE

                    .    :    .    :    .    :    .    :    .    :    .    :    .    :
Chain 1:   71 RIAQEDCRNGFLLDGFPRTIPQADAMKEAGINVDYVLEFDVPDELIVDRIVGRRVHAPSGRVYHVKFNPP
              1111111111111111111111111111111111111111111111111111111111111111111111
Chain 2:   71 RIAQEDCRNGFLLDGFPRTIPQADAMKEAGINVDYVLEFDVPDELIVDRIVGRRVHAPSGRVYHVKFNPP

                    .    :    .    :    .    :    .    :    .    :    .    :    .    :
Chain 1:  141 KVEGKDDVTGEELTTRKDDQEETVRKRLVEYHQMTAPLIGYYSKEAEAGNTKYAKVDGTKPVAEVRADLE
              1111111111111111111111111111111111111111111111111111111111111111111111
Chain 2:  141 KVEGKDDVTGEELTTRKDDQEETVRKRLVEYHQMTAALIGYYSKEAEAGNTKYAKVDGTKPVAEVRADLE

Chain 1:  211 KILG
              1111
Chain 2:  211 KILG
```

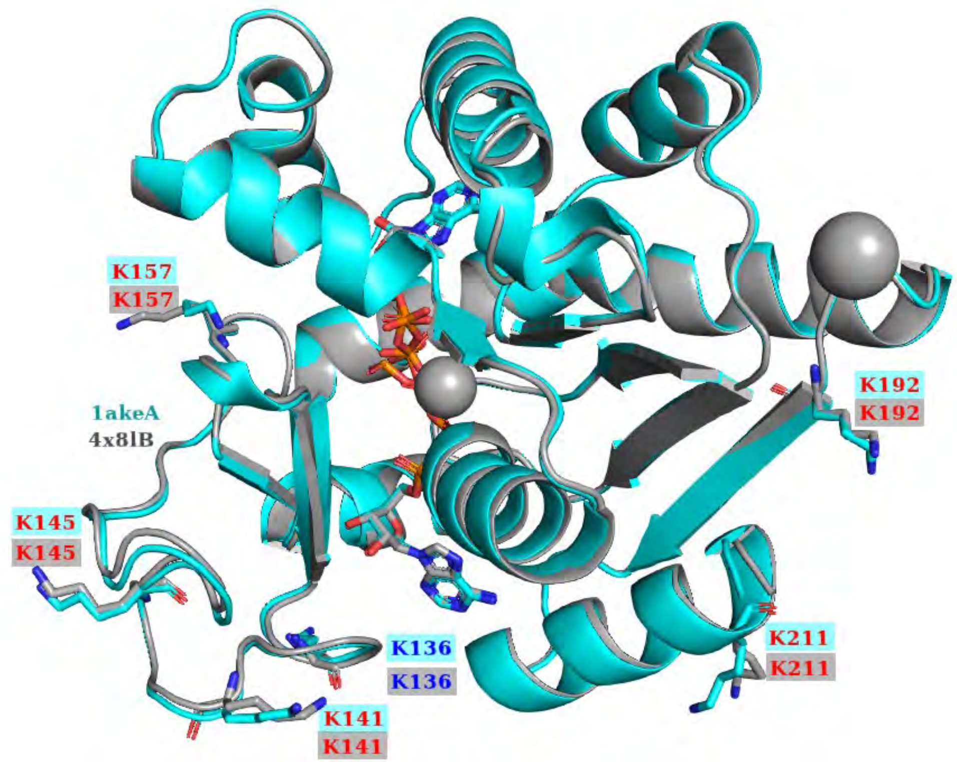

UniProt ID: P69441
PDB ID: 4X8M_A

```
Align 1ake.A.pdb 214 with 4x8m.A.pdb 214
Twists 2 ini-len 208 ini-rmsd 2.82 opt-equ 214 opt-rmsd 2.62 chain-rmsd 7.04 Score 584.38 align-len 214 gaps 0 (0.00%)
P-value 7.75e-14 Afp-num 13892 Identity 99.53% Similarity 100.00%
Block  0 afp 14 score 314.51 rmsd  3.44 gap 2 (0.02%)
Block  1 afp  5 score 118.13 rmsd  0.80 gap 0 (0.00%)
Block  2 afp  7 score 165.61 rmsd  1.47 gap 0 (0.00%)

                  .    :    .    :    .    :    .    :    .    :    .    :    .    :
Chain 1:    1 MRIILLGAPGAGKGTQAQFIMEKYGIPQISTGDMLRAAVKSGSELGKQAKDIMDAGKLVTDELVIALVKE
              1111111111111111111111111111111111111111111111111111111111111111111111
Chain 2:    1 MRIILLGAPGAGKGTQAQFIMEKYGIPQISTGDMLRAAVKSGSELGKQAKDIMDAGKLVTDELVIALVKE

                  .    :    .    :    .    :    .    :    .    :    .    :    .    :
Chain 1:   71 RIAQEDCRNGFLLDGFPRTIPQADAMKEAGINVDYVLEFDVPDELIVDRIVGRRVHAPSGRVYHVKFNPP
              11111111111111111111111111111111111111111111111222222222222222222222222
Chain 2:   71 RIAQEDCRNGFLLDGFPRTIPQADAMKEAGINVDYVLEFDVPDELIVDRIVGRRVHAPSGRVYHVKFNPP

                  .    :    .    :    .    :    .    :    .    :    .    :    .    :
Chain 1:  141 KVEGKDDVTGEELTTRKDDQEETVRKRLVEYHQMTAPLIGYYSKEAEAGNTKYAKVDGTKPVAEVRADLE
              22222222222222222333333333333333333333333333333333333333333333333333333
Chain 2:  141 KVEGKDDVTGEELTTRKDDQEETVRKRLVEWHQMTAPLIGYYSKEAEAGNTKYAKVDGTKPVAEVRADLE

Chain 1:  211 KILG
              3333
Chain 2:  211 KILG

Note: positions are from PDB; the numbers between alignments are block index
```

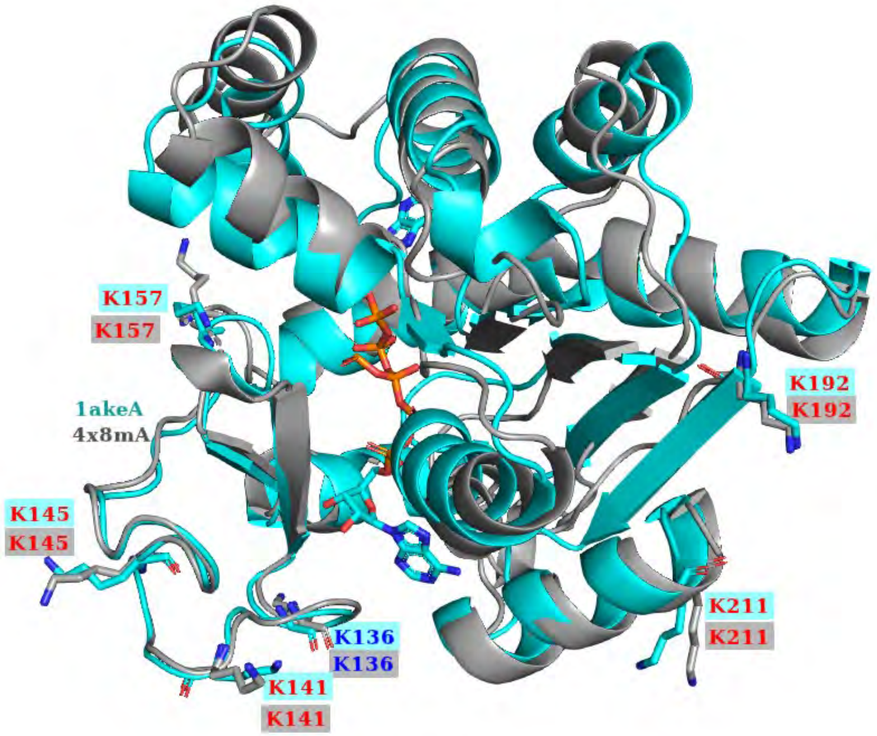

UniProt ID: P69441
PDB ID: 4X8O_B

```
Align 1ake.A.pdb 214 with 4x8o.B.pdb 214
Twists 0 ini-len 208 ini-rmsd 0.37 opt-equ 214 opt-rmsd 0.40 chain-rmsd 0.37 Score 622.87 align-len 214 gaps 0 (0.00%)
P-value 0.00e+00 Afp-num 14103 Identity 99.53% Similarity 100.00%
Block  0 afp 26 score 622.87 rmsd  0.37 gap 0 (0.00%)

Chain 1:    1 MRIILLGAPGAGKGTQAQFIMEKYGIPQISTGDMLRAAVKSGSELGKQAKDIMDAGKLVTDELVIALVKE
              1111111111111111111111111111111111111111111111111111111111111111111111
Chain 2:    1 MRIILLGAPGAGKGTQAQFIMEKYGIPQISTGDMLRAAVKSGSELGKQAKDIMDAGKLVTDELVIALVKE

Chain 1:   71 RIAQEDCRNGFLLDGFPRTIPQADAMKEAGINVDYVLEFDVPDELIVDRIVGRRVHAPSGRVYHVKFNPP
              1111111111111111111111111111111111111111111111111111111111111111111111
Chain 2:   71 RIAQEDCRNGFLLDGFPRTIPQADAMKEAGINVDYVLEFDVPDELIVDRIVGRRVHAPSGRVYHVKFNPP

Chain 1:  141 KVEGKDDVTGEELTTRKDDQEETVRKRLVEYHQMTAPLIGYYSKEAEAGNTKYAKVDGTKPVAEVRADLE
              1111111111111111111111111111111111111111111111111111111111111111111111
Chain 2:  141 KVEGKDDVTGEELTTRKDDQEETVRKRLVEWHQMTAPLIGYYSKEAEAGNTKYAKVDGTKPVAEVRADLE

Chain 1:  211 KILG
              1111
Chain 2:  211 KILG
```

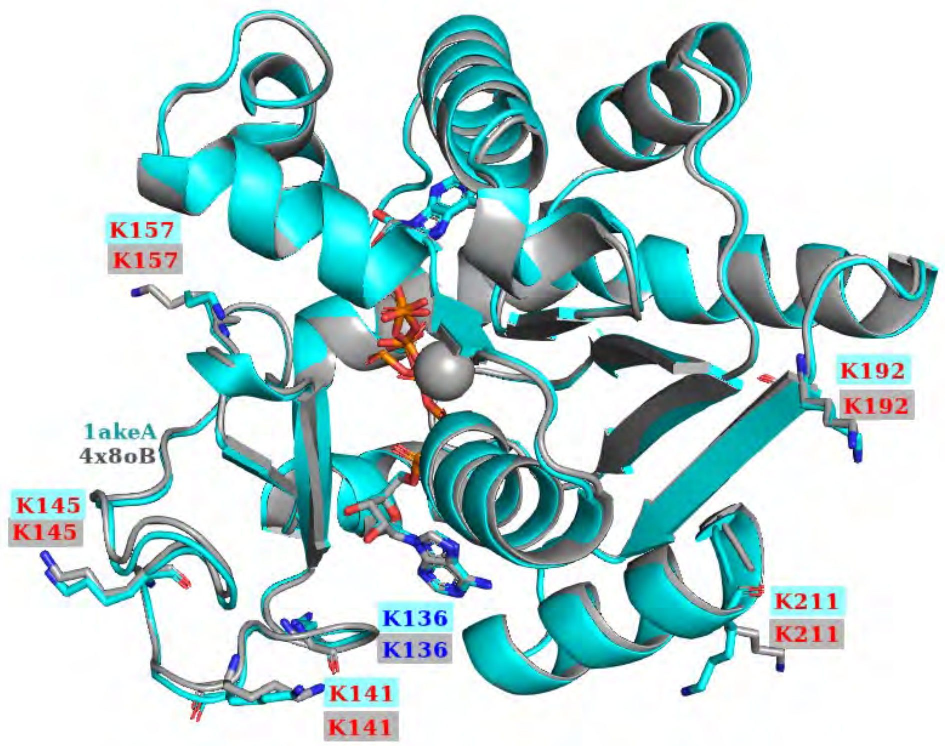

UniProt ID: P69441

PDB ID: 5EJE_B

```
Align 1ake.A.pdb 214 with 5eje.B.pdb 214
Twists 0 ini-len 208 ini-rmsd 0.40 opt-equ 214 opt-rmsd 0.40 chain-rmsd 0.40 Score 623.18 align-len 214 gaps 0 (0.00%)
P-value 0.00e+00 Afp-num 14168 Identity 99.07% Similarity 99.07%
Block  0 afp 26 score 623.18 rmsd  0.40 gap 0 (0.00%)

                  .    :    .    :    .    :    .    :    .    :    .    :    .    :
Chain 1:    1 MRIILLGAPGAGKGTQAQFIMEKYGIPQISTGDMLRAAVKSGSELGKQAKDIMDAGKLVTDELVIALVKE
              1111111111111111111111111111111111111111111111111111111111111111111111
Chain 2:    1 MRIILLGAPGAGKGTQAQFIMEKYGIPQISTGDMLRAAVKSGSELGKQAKDIMDACKLVTDELVIALVKE

                  .    :    .    :    .    :    .    :    .    :    .    :    .    :
Chain 1:   71 RIAQEDCRNGFLLDGFPRTIPQADAMKEAGINVDYVLEFDVPDELIVDRIVGRRVHAPSGRVYHVKFNPP
              1111111111111111111111111111111111111111111111111111111111111111111111
Chain 2:   71 RIAQEDCRNGFLLDGFPRTIPQADAMKEAGINVDYVLEFDVPDELIVDRIVGRRVHAPSGRVYHVKFNPP

                  .    :    .    :    .    :    .    :    .    :    .    :    .    :
Chain 1:  141 KVEGKDDVTGEELTTRKDDQEETVRKRLVEYHQMTAPLIGYYSKEAEAGNTKYAKVDGTKPVAEVRADLE
              1111111111111111111111111111111111111111111111111111111111111111111111
Chain 2:  141 KVEGKDDVTGEELTTRKDDQEECVRKRLVEYHQMTAPLIGYYSKEAEAGNTKYAKVDGTKPVAEVRADLE


Chain 1:  211 KILG
              1111
Chain 2:  211 KILG
```

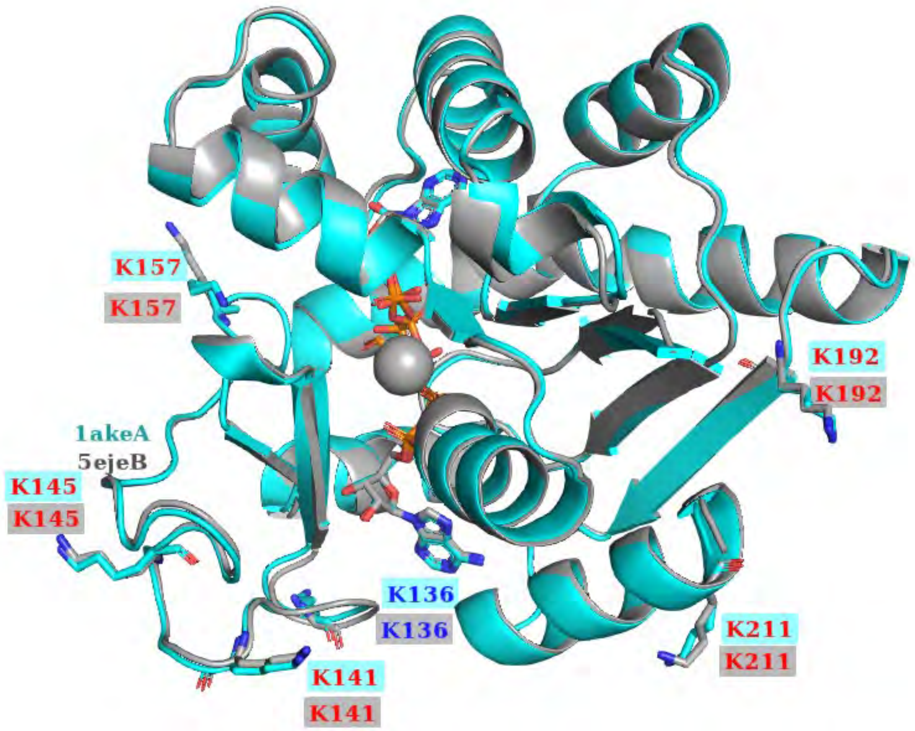

UniProt ID: P69441
PDB ID: 6F7U_A

```
Align 1ake.A.pdb 214 with 6f7u.A.pdb 214
Twists 2 ini-len 208 ini-rmsd 2.77 opt-equ 211 opt-rmsd 2.37 chain-rmsd 6.34 Score 580.35 align-len 217 gaps 6 (2.76%)
P-value 2.48e-14 Afp-num 13859 Identity 93.09% Similarity 93.55%
Block  0 afp 14 score 309.00 rmsd  3.61 gap 4 (0.03%)
Block  1 afp  5 score 119.02 rmsd  0.58 gap 0 (0.00%)
Block  2 afp  7 score 166.05 rmsd  1.32 gap 0 (0.00%)

                  .    :    .    :    .    :    .    :    .    :    .    :    .    :
Chain 1:    1 MRIILLGAPGAGKGTQAQFIMEKYGIPQISTGDMLRAAVKSG---SELGKQAKDIMDAGKLVTDELVIAL
              111111111111111111111111111111111111111111   11111111111   11111111111
Chain 2:    1 MRIILLGAPGAGKGTQAQFIMEKYGIPQISTGDMLRAAVKSGSELGKQAKDIMDAG---KLVTDELVIAL

                  .    :    .    :    .    :    .    :    .    :    .    :    .    :
Chain 1:   68 VKERIAQEDCRNGFLLDGFPRTIPQADAMKEAGINVDYVLEFDVPDELIVDRIVGRRVHAPSGRVYHVKF
              1111111111111111111111111111111111111111111111111122222222222222222222
Chain 2:   68 VKERIAQEDCRNGFLLDGFPRTIPQADAMKEAGINVDYVLEFDVPDELIVDRIVGRRVHAPSGRVYHVKF

                  .    :    .    :    .    :    .    :    .    :    .    :    .    :
Chain 1:  138 NPPKVEGKDDVTGEELTTRKDDQEETVRKRLVEYHQMTAPLIGYYSKEAEAGNTKYAKVDGTKPVAEVRA
              2222222222222222222233333333333333333333333333333333333333333333333333
Chain 2:  138 NPPKVEGKDDVTGEELTTRKDDQEETVRKRLVEYHQMTAPLIGYYSKEAEAGNTKYAKVDGTKPVAEVRA

                  .
Chain 1:  208 DLEKILG
              3333333
Chain 2:  208 DLEKILG

Note: positions are from PDB; the numbers between alignments are block index
```

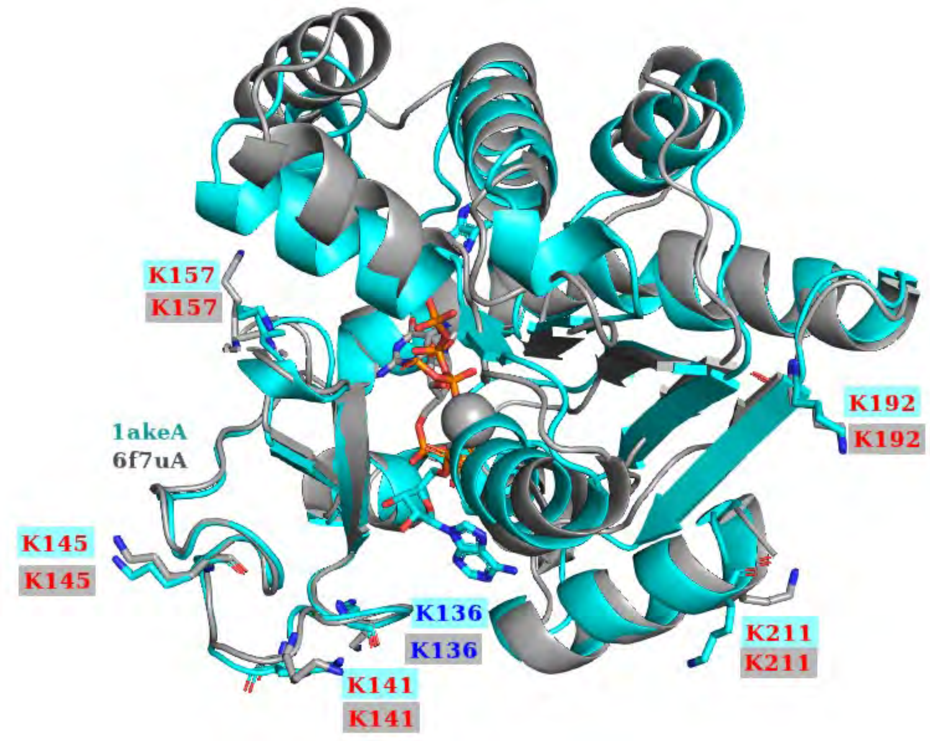

UniProt ID: P69441

PDB ID: 6HAM_A

```
Align 1ake.A.pdb 214 with 6ham.A.pdb 214
Twists 0 ini-len 208 ini-rmsd 0.79 opt-equ 214 opt-rmsd 0.83 chain-rmsd 0.79 Score 611.19 align-len 214 gaps 0 (0.00%)
P-value 0.00e+00 Afp-num 13946 Identity 97.20% Similarity 98.60%
Block  0 afp 26 score 611.19 rmsd  0.79 gap 2 (0.01%)

                  .    :    .    :    .    :    .    :    .    :    .    :    .    :
Chain 1:     1 MRIILLGAPGAGKGTQAQFIMEKYGIPQISTGDMLRAAVKSGSELGKQAKDIMDAGKLVTDELVIALVKE
               1111111111111111111111111111111111111111111111111111111111111111111111
Chain 2:     1 MRIILLGAPGAGKGTQAQFIMEKYGIPQISTGDMLRAAIKSGSELGKQAKDIMDAGKLVTDEIIIALVKE

                  .    :    .    :    .    :    .    :    .    :    .    :    .    :
Chain 1:    71 RIAQEDCRNGFLLDGFPRTIPQADAMKEAGINVDYVLEFDVPDELIVDRIVGRRVHAPSGRVYHVKFNPP
               1111111111111111111111111111111111111111111111111111111111111111111111
Chain 2:    71 RICQEDSRNGFLLDGFPRTIPQADAMKEAGINVDYVLEFDVPDELIVDRIVGRRVHAPSGRVYHVKFNPP

                  .    :    .    :    .    :    .    :    .    :    .    :    .    :
Chain 1:   141 KVEGKDDVTGEELTTRKDDQEETVRKRLVEYHQMTAPLIGYYSKEAEAGNTKYAKVDGTKPVAEVRADLE
               1111111111111111111111111111111111111111111111111111111111111111111111
Chain 2:   141 KVEGKDDVTGEELTTRKDDQEETVRKRLVEYHQMTAPLIGYYSKEAEAGNTKYAKVDGTKPVCEVRADLE

Chain 1:   211 KILG
               1111
Chain 2:   211 KILG

Note: positions are from PDB; the numbers between alignments are block index
```

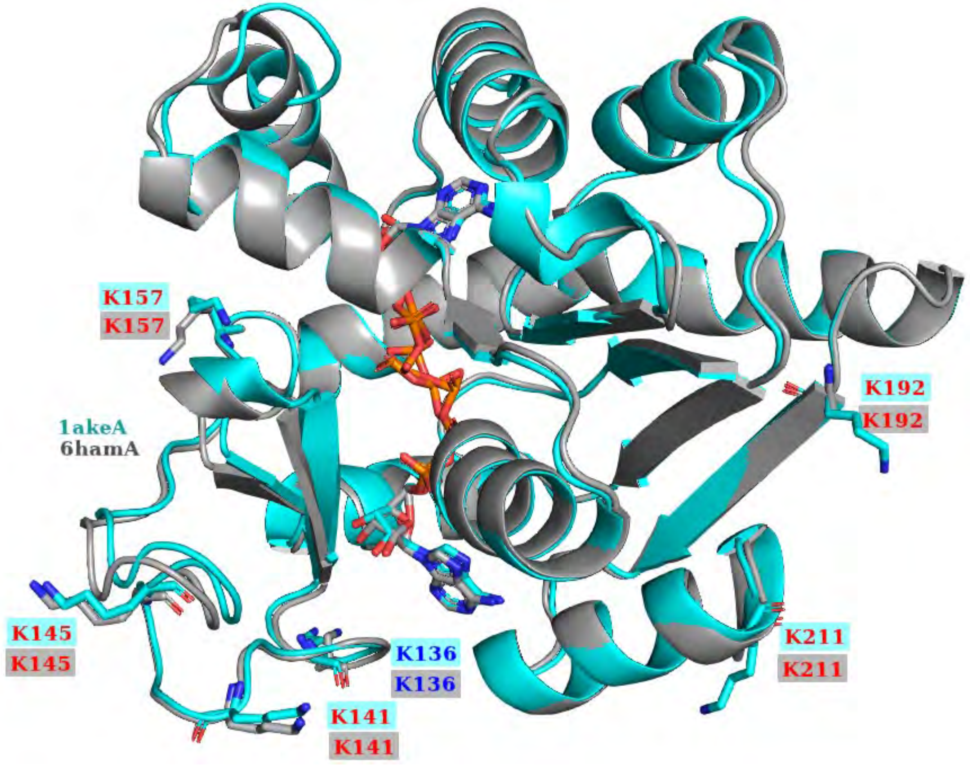

UniProt ID: P69441

PDB ID: 6RZE_A

```
Align 1ake.A.pdb 214 with 6rze.A.pdb 214
Twists 2 ini-len 208 ini-rmsd 2.86 opt-equ 211 opt-rmsd 2.39 chain-rmsd 7.10 Score 575.03 align-len 217 gaps 6 (2.76%)
P-value 3.73e-14 Afp-num 13985 Identity 92.63% Similarity 93.09%
Block  0 afp 14 score 301.43 rmsd  3.62 gap 4 (0.03%)
Block  1 afp  5 score 119.41 rmsd  0.42 gap 0 (0.00%)
Block  2 afp  7 score 165.90 rmsd  1.42 gap 0 (0.00%)

                   .    :    .    :    .    :    .    :    .    :    .    :    .    :
Chain 1:    1 MRIILLGAPGAGKGTQAQFIMEKYGIPQISTGDMLRAAVKSG---SELGKQAKDIMDAGKLVTDELVIAL
              111111111111111111111111111111111111111111   11111111111   11111111111
Chain 2:    1 MRIILLGAPGAGKGTQAQFIMEKYGIPQISTGDMLRAAVKSGSELGKQAKDIMDAG---KLVTDELVIAL

                   .    :    .    :    .    :    .    :    .    :    .    :    .    :
Chain 1:   68 VKERIAQEDCRNGFLLDGFPRTIPQADAMKEAGINVDYVLEFDVPDELIVDRIVGRRVHAPSGRVYHVKF
              1111111111111111111111111111111111111111111111111112222222222222222222
Chain 2:   68 VKERIAQEDCRNGFLLDGFPRTIPQADAMKEAGINVDYVLEFDVPDELIVDAIVGRRVHAPSGRVYHVKF

                   .    :    .    :    .    :    .    :    .    :    .    :    .    :
Chain 1:  138 NPPKVEGKDDVTGEELTTRKDDQEETVRKRLVEYHQMTAPLIGYYSKEAEAGNTKYAKVDGTKPVAEVRA
              2222222222222222222233333333333333333333333333333333333333333333333333
Chain 2:  138 NPPKVEGKDDVTGEELTTRKDDQEETVRKRLVEYHQMTAPLIGYYSKEAEAGNTKYAKVDGTKPVAEVRA

                   .
Chain 1:  208 DLEKILG
              3333333
Chain 2:  208 DLEKILG

Note: positions are from PDB; the numbers between alignments are block index
```

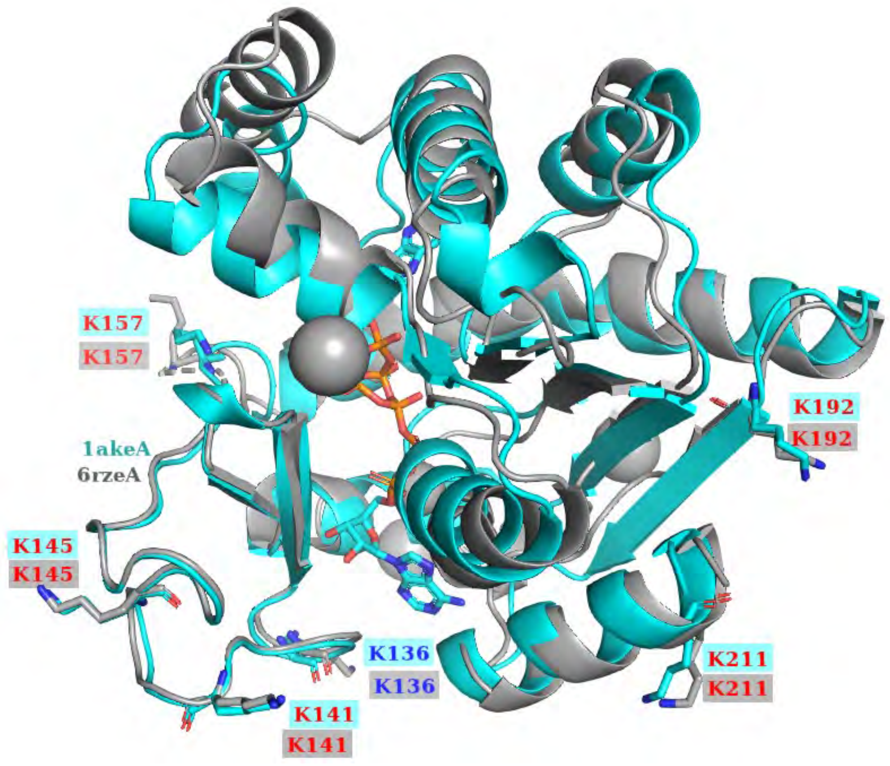

UniProt ID: P69441
PDB ID: 6S36_A

```
Align 1ake.A.pdb 214 with 6s36.A.pdb 214
Twists 2 ini-len 208 ini-rmsd 2.88 opt-equ 210 opt-rmsd 2.34 chain-rmsd 7.00 Score 581.91 align-len 217 gaps 7 (3.23%)
P-value 1.99e-14 Afp-num 13944 Identity 92.17% Similarity 93.09%
Block  0 afp 14 score 310.46 rmsd  3.70 gap 4 (0.03%)
Block  1 afp  5 score 119.31 rmsd  0.58 gap 0 (0.00%)
Block  2 afp  7 score 165.91 rmsd  1.28 gap 0 (0.00%)

                  .    :    .    :    .    :    .    :    .    :    .    :    .    :
Chain 1:    1 MRIILLGAPGAGKGTQAQFIMEKYGIPQISTGDMLRAAVKSG---SELGKQAKDIMDAGKLVTDELVIAL
                111111111111111111111111111111111111111111   111111111111   1111111111
Chain 2:    1 MRIILLGAPGAGKGTQAQFIMEKYGIPQISTGDMLRAAVKSGSELGKQAKDIMDAGK---LVTDELVIAL

                  .    :    .    :    .    :    .    :    .    :    .    :    .    :
Chain 1:   68 VKERIAQEDCRNGFLLDGFPRTIPQADAMKEAGINVDYVLEFDVPDELIVDRIVGRRVHAPSGRVYHVKF
              1111111111111111111111111111111111111111111111111112222222222222222222
Chain 2:   68 VKERIAQEDCRNGFLLDGFPRTIPQADAMKEAGINVDYVLEFDVPDELIVDKIVGRRVHAPSGRVYHVKF

                  .    :    .    :    .    :    .    :    .    :    .    :    .    :
Chain 1:  138 NPPKVEGKDDVTGEELTTRKDDQEETVRKRLVEYHQMTAPLIGYYSKEAEAGNTKYAKVDGTKPVAEVRA
              2222222222222222222233333333333333333333333333333333333333333333333333
Chain 2:  138 NPPKVEGKDDVTGEELTTRKDDQEETVRKRLVEYHQMTAPLIGYYSKEAEAGNTKYAKVDGTKPVAEVRA

                  .
Chain 1:  208 DLEKILG
              3333333
Chain 2:  208 DLEKILG

Note: positions are from PDB; the numbers between alignments are block index
```

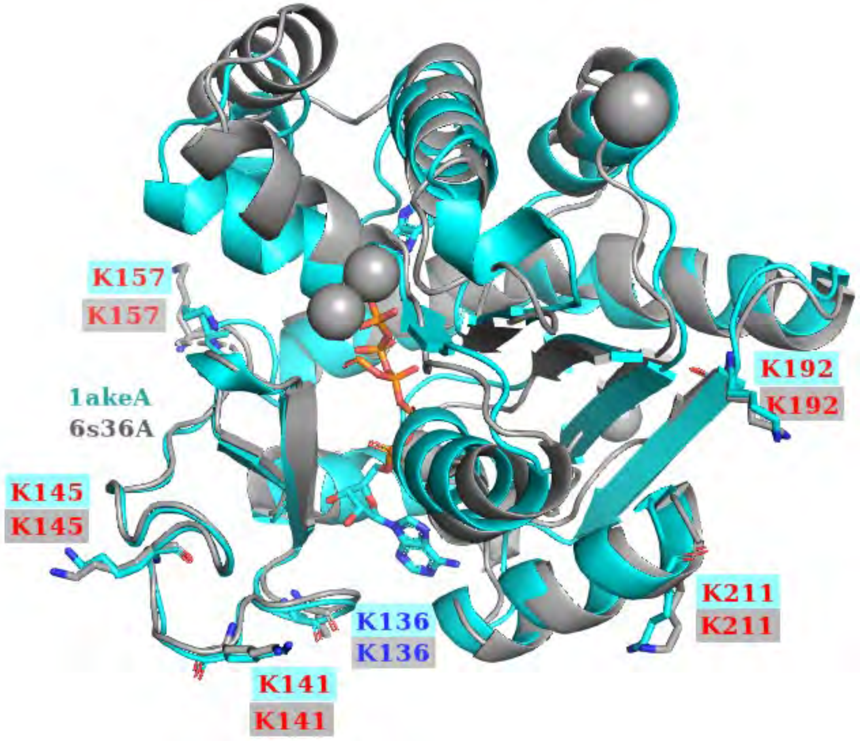

UniProt ID: P84139

PDB ID: 1S3G_A

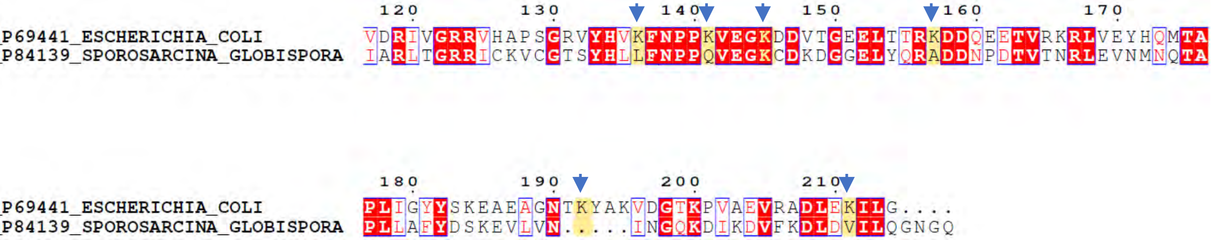


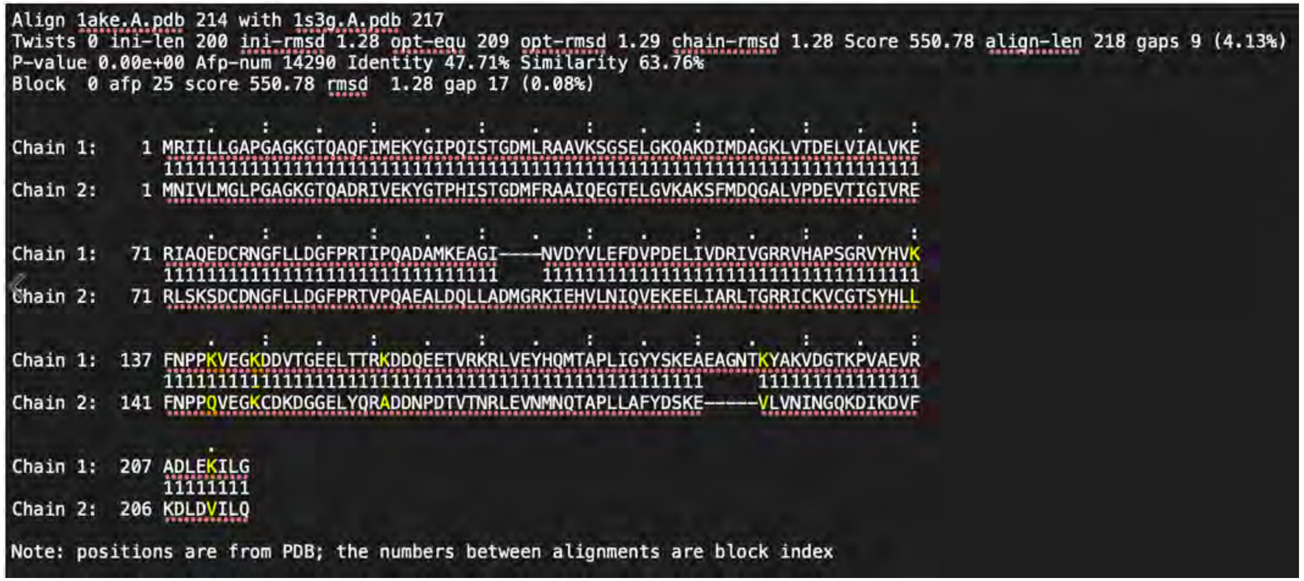

```
Align 1ake.A.pdb 214 with 1s3g.A.pdb 217
Twists 0 ini-len 200 ini-rmsd 1.28 opt-equ 209 opt-rmsd 1.29 chain-rmsd 1.28 Score 550.78 align-len 218 gaps 9 (4.13%)
P-value 0.00e+00 Afp-num 14290 Identity 47.71% Similarity 63.76%
Block  0 afp 25 score 550.78 rmsd  1.28 gap 17 (0.08%)

Chain 1:    1 MRIILLGAPGAGKGTQAQFIMEKYGIPQISTGDMLRAAVKSGSELGKQAKDIMDAGKLVTDELVIALVKE
              1111111111111111111111111111111111111111111111111111111111111111111111
Chain 2:    1 MNIVLMGLPGAGKGTQADRIVEKYGTPHISTGDMFRAAIQEGTELGVKAKSFMDQGALVPDEVTIGIVRE

Chain 1:   71 RIAQEDCRNGFLLDGFPRTIPQADAMKEAGI----NVDYVLEFDVPDELIVDRIVGRRVHAPSGRVYHVK
              1111111111111111111111111111111    11111111111111111111111111111111111
Chain 2:   71 RLSKSDCDNGFLLDGFPRTVPQAEALDQLLADMGRKIEHVLNIQVEKEELIARLTGRRICKVCGTSYHLL

Chain 1:  137 FNPPKVEGKDDVTGEELTTRKDDQEETVRKRLVEYHQMTAPLIGYYSKEAEAGNTKYAKVDGTKPVAEVR
              11111111111111111111111111111111111111111111111111     111111111111111
Chain 2:  141 FNPPQVEGKCDKDGGELYQRADDNPDTVTNRLEVNMNQTAPLLAFYDSKE-----VLVNINGQKDIKDVF

Chain 1:  207 ADLEKILG
              11111111
Chain 2:  206 KDLDVILQ

Note: positions are from PDB; the numbers between alignments are block index
```

Full sequences in supplemental file.

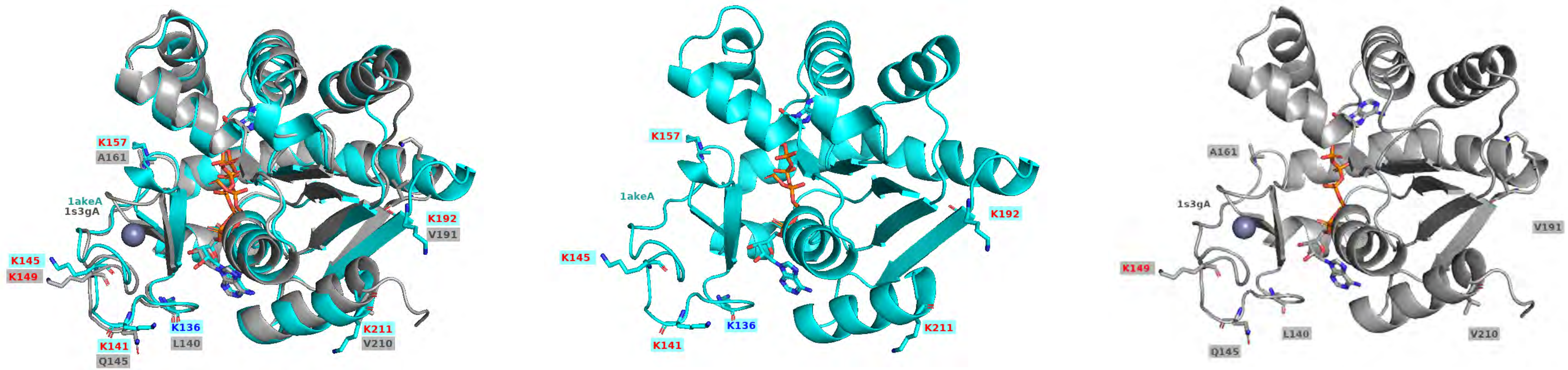

# UniProt ID: P84139
# PDB ID: 5X6J_A

```
                                120       130       140       150       160       170
P69441_ESCHERICHIA_COLI         VDRIVGRRVHAPSGRVYHVKFNPPKVEGKDDVTGEELTTRKDDQEETVRKRLVEYHQMTA
P84139_SPOROSARCINA_GLOBISPORA  IARLTGRRICKVCGTSYHLLFNPPQVEGKCDKDGGELYQRADDNPDTVTNRLEVNMNQTA

                                180       190       200       210
P69441_ESCHERICHIA_COLI         PLIGYYSKEAEAGNTKYAKVDGTKPVAEVRADLEKILG....
P84139_SPOROSARCINA_GLOBISPORA  PLLAFYDSKEVLVN.....INGQKDIKDVFKDLDVILQGNGQ
```

Full sequences in supplemental file.

```
Align 1ake.A.pdb 214 with 5x6j.A.pdb 213
Twists 0 ini-len 200 ini-rmsd 1.27 opt-equ 209 opt-rmsd 1.28 chain-rmsd 1.27 Score 551.38 align-len 218 gaps 9 (4.13%)
P-value 0.00e+00 Afp-num 13919 Identity 48.17% Similarity 64.22%
Block  0 afp 25 score 551.38 rmsd  1.27 gap 17 (0.08%)

                   .    :    .    :    .    :    .    :    .    :    .    :    .    :
Chain 1:    1 MRIILLGAPGAGKGTQAQFIMEKYGIPQISTGDMLRAAVKSGSELGKQAKDIMDAGKLVTDELVIALVKE
              1111111111111111111111111111111111111111111111111111111111111111111111
Chain 2:    1 MNIVLMGLPGAGKGTQADRIVEKYGIPHISTGDMFRAAIQEGTELGVKAKSFMDQGALVPDEVTIGIVRE

                   .    :    .    :    .    :    .    :    .    :    .    :    .    :
Chain 1:   71 RIAQEDCRNGFLLDGFPRTIPQADAMKEAGI----NVDYVLEFDVPDELIVDRIVGRRVHAPSGRVYHVK
              1111111111111111111111111111111    11111111111111111111111111111111111
Chain 2:   71 RLSKSDCDNGFLLDGFPRTVPQAEALDQLLADMGRKIEHVLNIQVEKEELIARLTGRRICKVCGTSYHLL

                   .    :    .    :    .    :    .    :    .    :    .    :    .    :
Chain 1:  137 FNPPKVEGKDDVTGEELTTRKDDQEETVRKRLVEYHQMTAPLIGYYSKEAEAGNTKYAKVDGTKPVAEVR
              11111111111111111111111111111111111111111111111111     111111111111111
Chain 2:  141 FNPPQVEGKCDKDGGELYQRADDNPDTVTNRLEVNMNQTAPLLAFYDSKE-----VLVNINGQKDIKDVF

                   .
Chain 1:  207 ADLEKILG
              11111111
Chain 2:  206 KDLDVILQ

Note: positions are from PDB; the numbers between alignments are block index
```

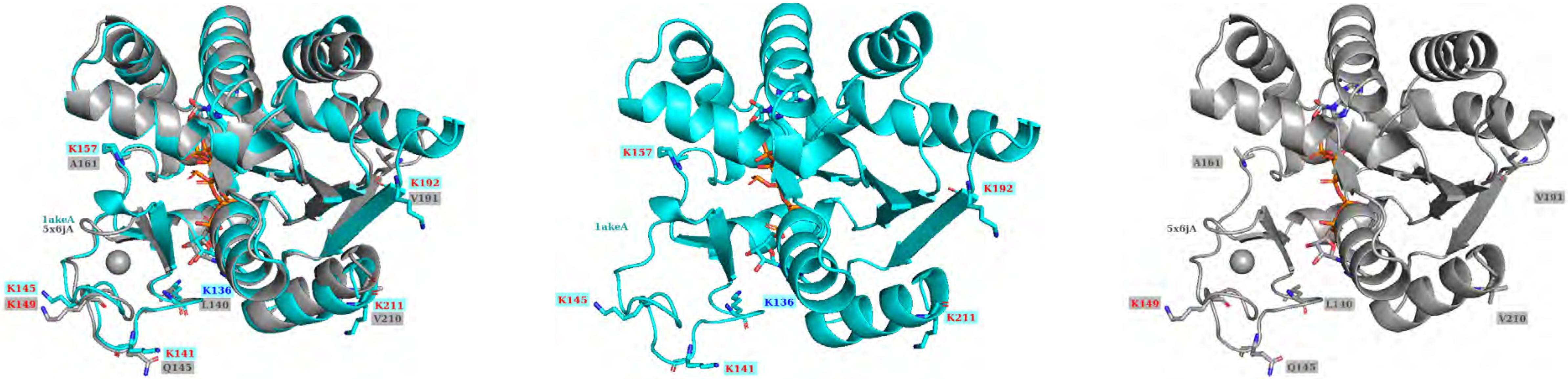

# UniProt ID: P9WKF5

# PDB ID: 1P4S_A

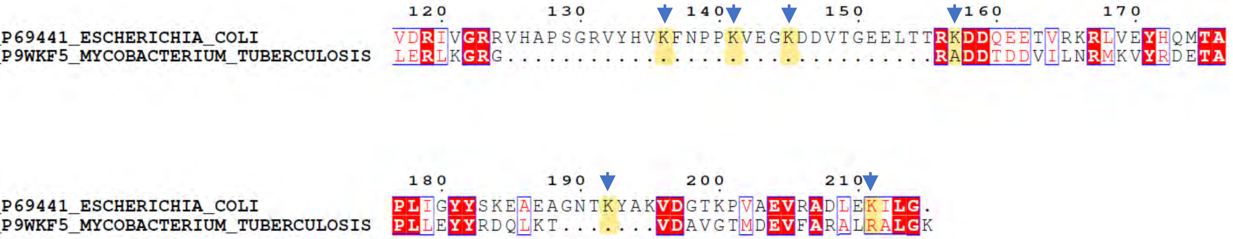


Full sequences in supplemental file.

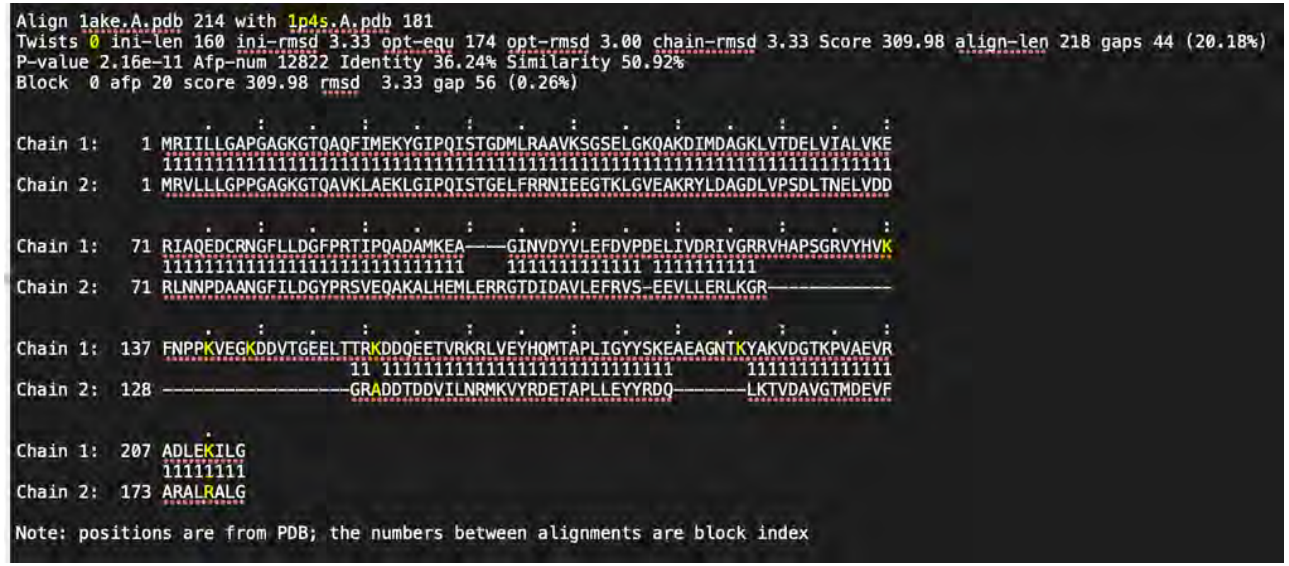

```
Align 1ake.A.pdb 214 with 1p4s.A.pdb 181
Twists 0 ini-len 160 ini-rmsd 3.33 opt-equ 174 opt-rmsd 3.00 chain-rmsd 3.33 Score 309.98 align-len 218 gaps 44 (20.18%)
P-value 2.16e-11 Afp-num 12822 Identity 36.24% Similarity 50.92%
Block  0 afp 20 score 309.98 rmsd  3.33 gap 56 (0.26%)

                .    :    .    :    .    :    .    :    .    :    .    :    .    :
Chain 1:    1 MRIILLGAPGAGKGTQAQFIMEKYGIPQISTGDMLRAAVKSGSELGKQAKDIMDAGKLVTDELVIALVKE
              1111111111111111111111111111111111111111111111111111111111111111111111
Chain 2:    1 MRVLLLGPPGAGKGTQAVKLAEKLGIPQISTGELFRRNIEEGTKLGVEAKRYLDAGDLVPSDLTNELVDD

                .    :    .    :    .    :    .    :    .    :    .    :    .    :
Chain 1:   71 RIAQEDCRNGFLLDGFPRTIPQADAMKEA---GINVDYVLEFDVPDELIVDRIVGRRVHAPSGRVYHVK
              11111111111111111111111111111    1111111111111 1111111111
Chain 2:   71 RLNNPDAANGFILDGYPRSVEQAKALHEMLERRGTDIDAVLEFRVS-EEVLLERLKGR-----------

                .    :    .    :    .    :    .    :    .    :    .    :    .    :
Chain 1:  137 FNPPKVEGKDDVTGEELTTRKDDQEETVRKRLVEYHQMTAPLIGYYSKEAEAGNTKYAKVDGTKPVAEVR
                                 11 1111111111111111111111111111       11111111111111
Chain 2:  128 -----------------GRADDTDDVILNRMKVYRDETAPLLEYYRDQ-------LKTVDAVGTMDEVF

                .
Chain 1:  207 ADLEKILG
              11111111
Chain 2:  173 ARALRALG

Note: positions are from PDB; the numbers between alignments are block index
```

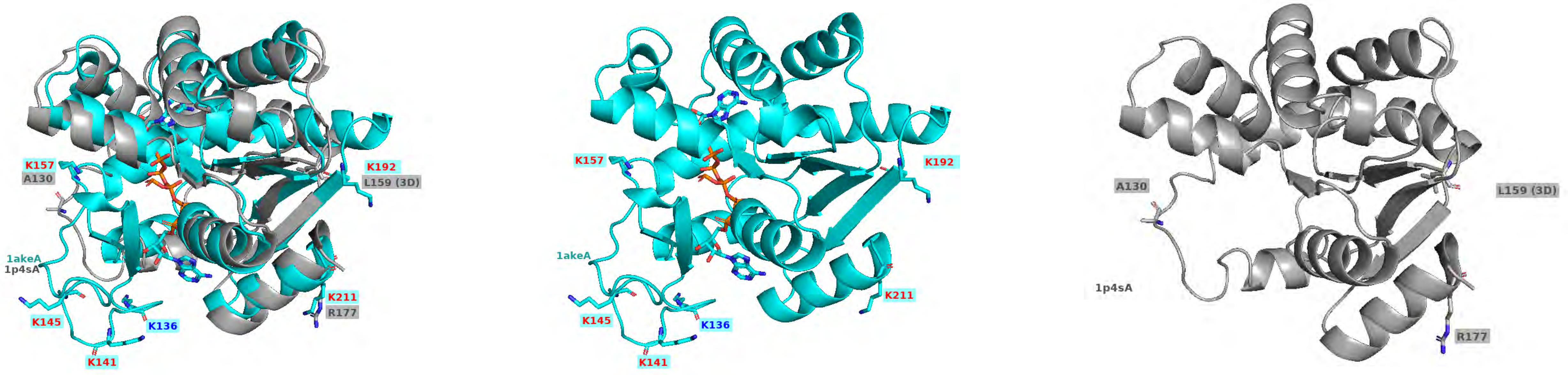

UniProt ID: P9WKF5

PDB ID: 2CDN_A

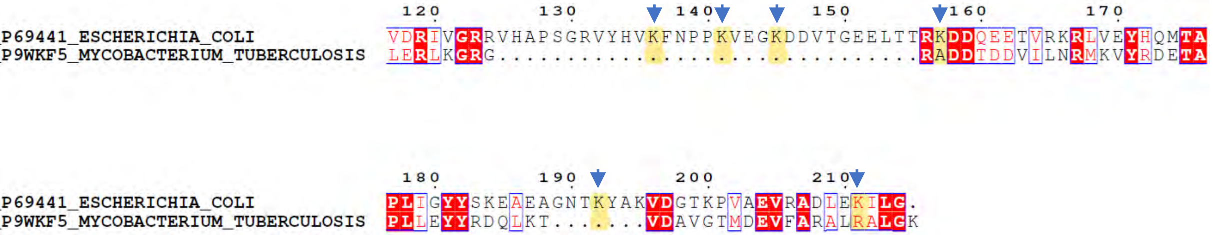


Full sequences in supplemental file.

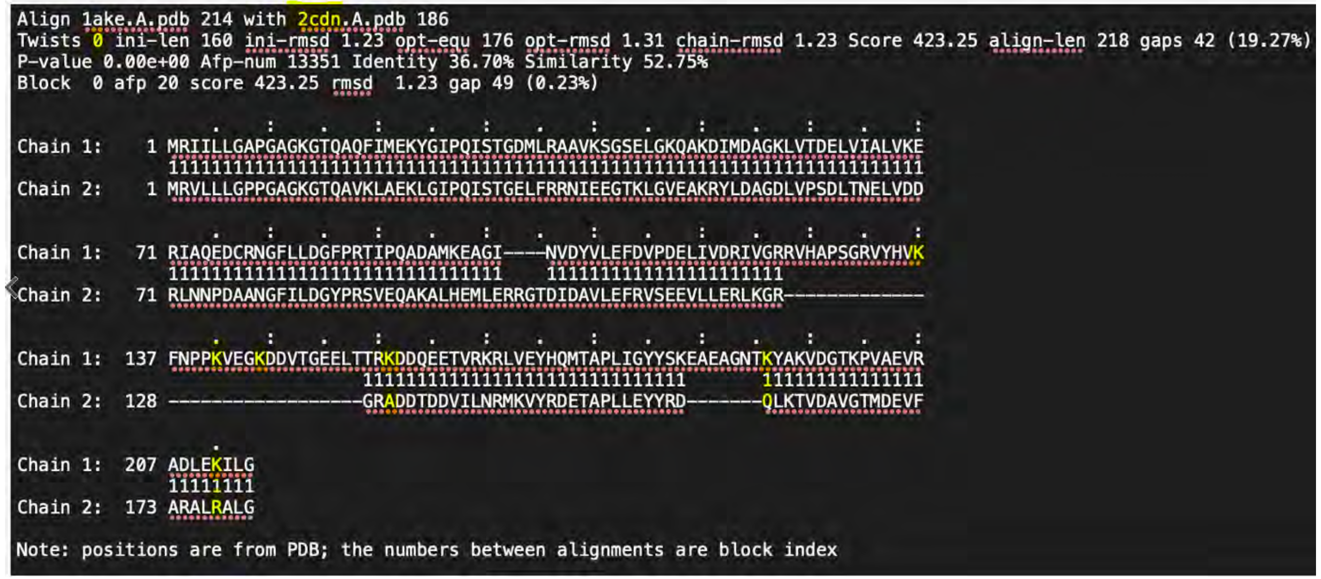


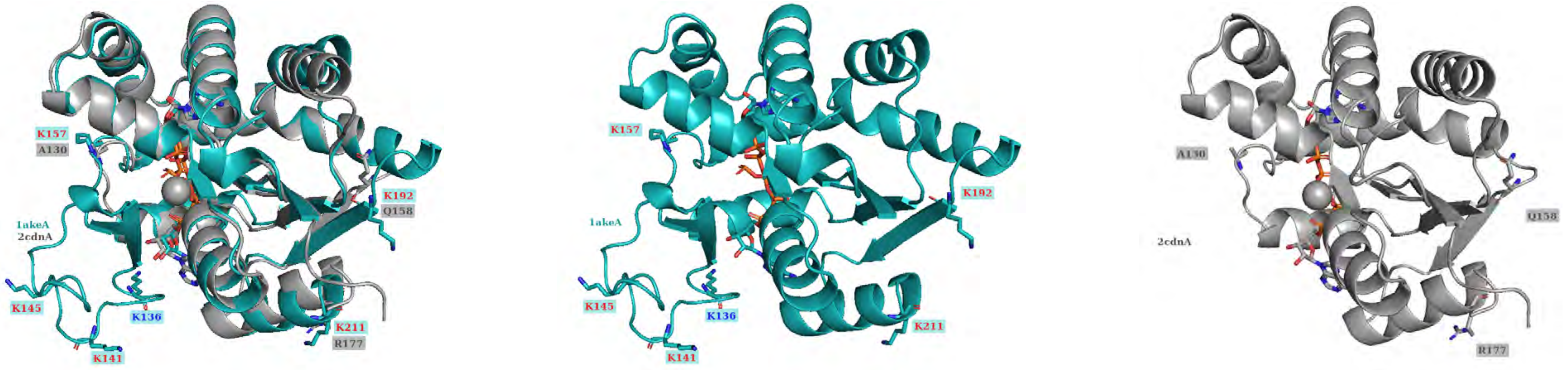

UniProt ID: Q04ML5
PDB ID: 4NTZ_A

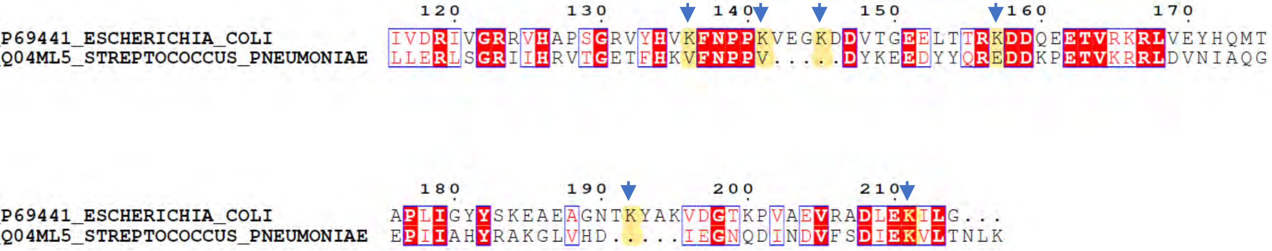


Full sequences in supplemental file.

```
Align 1ake.A.pdb 214 with 4ntz.A.pdb 213
Twists 2 ini-len 184 ini-rmsd 3.08 opt-equ 201 opt-rmsd 2.68 chain-rmsd 7.52 Score 463.64 align-len 222 gaps 21 (9.46%)
P-value 1.71e-10 Afp-num 14101 Identity 35.14% Similarity 57.66%
Block  0 afp 13 score 291.10 rmsd  3.75 gap 18 (0.15%)
Block  1 afp  4 score 77.49 rmsd  1.65 gap 7 (0.18%)
Block  2 afp  6 score 120.78 rmsd  1.75 gap 5 (0.09%)

                  .    :    .    :    .    :    .    :    .    :    .    :    .    :
Chain 1:    1 MRIILLGAPGAGKGTQAQFIMEKYGIPQISTGDMLRAAVKSG---SELGKQAKDIMDAGKLVTDELVIAL
              111111111111111111111111111111111111111111   111111111111   1111111111
Chain 2:    1 MNLLIMGLPGAGKGTQAAKIVEQFHVAHISTGDMFRAAMANQTEMGVLAKSYIDKGE---LVPDEVTNGI

                  .    :    .    :    .    :    .    :    .    :    .    :    .    :
Chain 1:   68 VKERIAQEDCRN-GFLLDGFPRTIPQADAMKEAG----INVDYVLEFDVPDELIVDRIVGRRVHAPSGRV
              111111111111 1111111111111111111111    1111111111111111111112222222222222
Chain 2:   68 VKERLSQDDIKETGFLLDGYPRTIEQAHALDKTLAELGIELEGIINIEVNPDSLLERLSGRIIHRVTGET

                  .    :    .    :    .    :    .    :    .    :    .    :    .    :
Chain 1:  133 YHVKFNPPKVEGKDDVTGEELTTRKDDQEETVRKRLVEYHQMTAPLIGYYSKEAEAGNTKYAKVDGTKPV
              2222222222222     222222222333333333333333333333333333     33333333333
Chain 2:  138 FHKVFNPPVDYKE-----EDYYQREDDKPETVKRRLDVNIAQGEPIIAHYRAKG-----LVHDIEGNQDI

                  .    :
Chain 1:  203 AEVRADLEKILG
              333333333333
Chain 2:  198 NDVFSDIEKVLT

Note: positions are from PDB; the numbers between alignments are block index
```

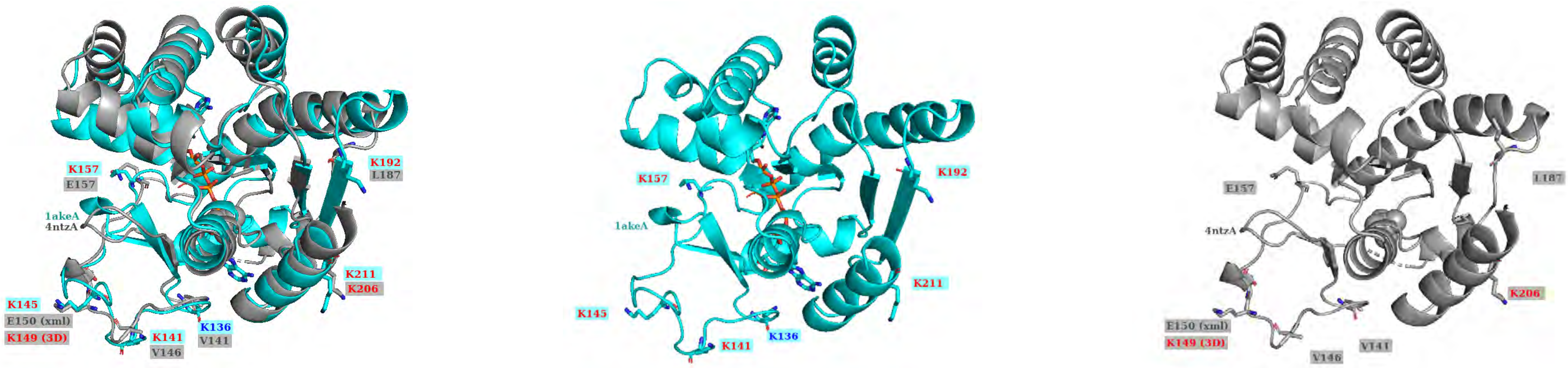

UniProt ID: Q04ML5

PDB ID: 4NU0_B

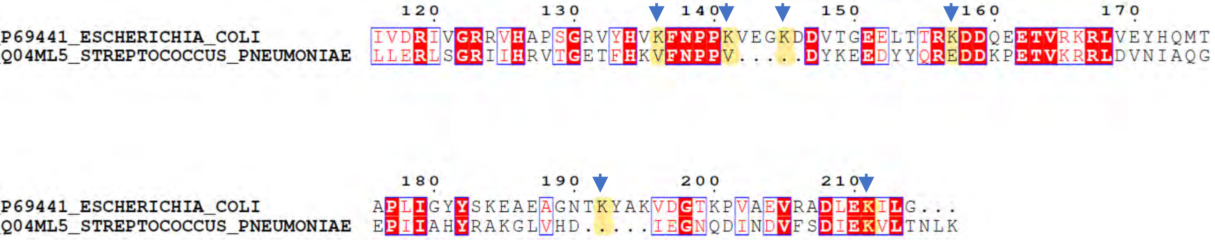


Full sequences in supplemental file.

```
Align 1ake.A.pdb 214 with 4nu0.B.pdb 212
Twists 0 ini-len 184 ini-rmsd 1.26 opt-equ 204 opt-rmsd 1.45 chain-rmsd 1.26 Score 513.71 align-len 219 gaps 15 (6.85%)
P-value 0.00e+00 Afp-num 14474 Identity 36.99% Similarity 61.19%
Block  0 afp 23 score 513.71 rmsd  1.26 gap 31 (0.14%)

                .    :    .    :    .    :    .    :    .    :    .    :    .    :
Chain 1:    1 MRIILLGAPGAGKGTQAQFIMEKYGIPQISTGDMLRAAVKSGSELGKQAKDIMDAGKLVTDELVIALVKE
              1111111111111111111111111111111111111111111111111111111111111111111111
Chain 2:    1 MNLLIMGLPGAGKGTQAAKIVEQFHVAHISTGDMFRAAMANQTEMGVLAKSYIDKGELVPDEVTNGIVKE

                .    :    .    :    .    :    .    :    .    :    .    :    .    :
Chain 1:   71 RIAQEDCRN-GFLLDGFPRTIPQADAMKEAG----INVDYVLEFDVPDELIVDRIVGRRVHAPSGRVYHV
              111111111 111111111111111111111    11111111111111111111111111111111111
Chain 2:   71 RLSQDDIKETGFLLDGYPRTIEQAHALDKTLAELGIELEGIINIEVNPDSLLERLSGRIIHRVTGETFHK

                .    :    .    :    .    :    .    :    .    :    .    :    .    :
Chain 1:  136 KFNPPKVEGKDDVTGEELTTRKDDQEETVRKRLVEYHQMTAPLIGYYSKEAEAGNTKYAKVDGTKPVAEV
              1111111111     111111111111111111111111111111111111     11111111111111
Chain 2:  141 VFNPPVDYKE-----EDYYQREDDKPETVKRRLDVNIAQGEPIIAHYRAKG-----LVHDIEGNQDINDV

                .
Chain 1:  206 RADLEKILG
              111111111
Chain 2:  201 FSDIEKVLT

Note: positions are from PDB; the numbers between alignments are block index
```

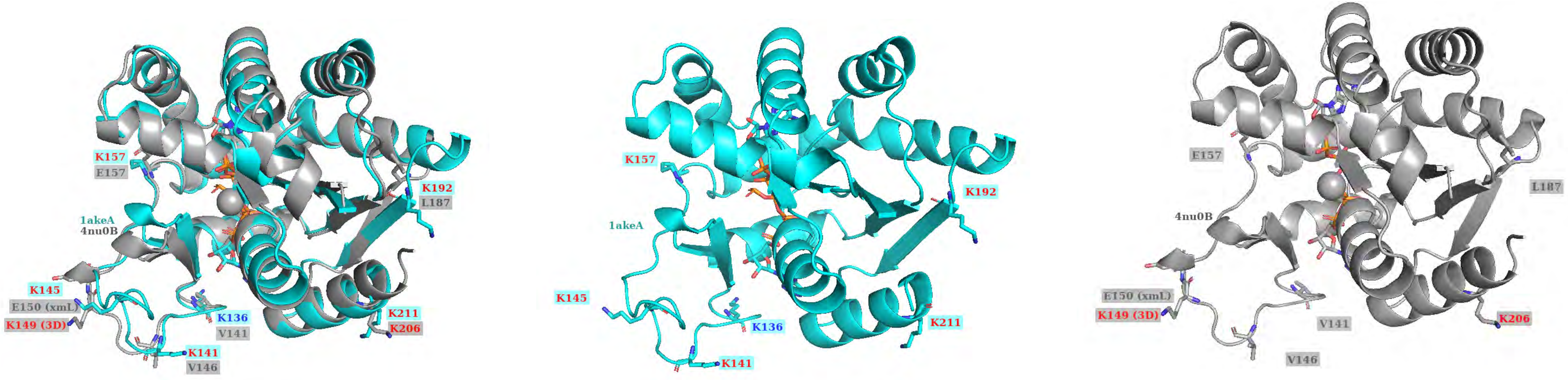

UniProt ID: Q04ML5

PDB ID: 4W5H_A

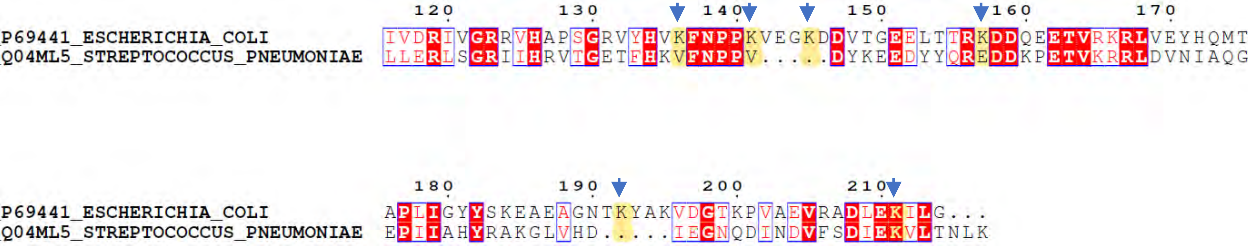


Full sequences in supplemental file.

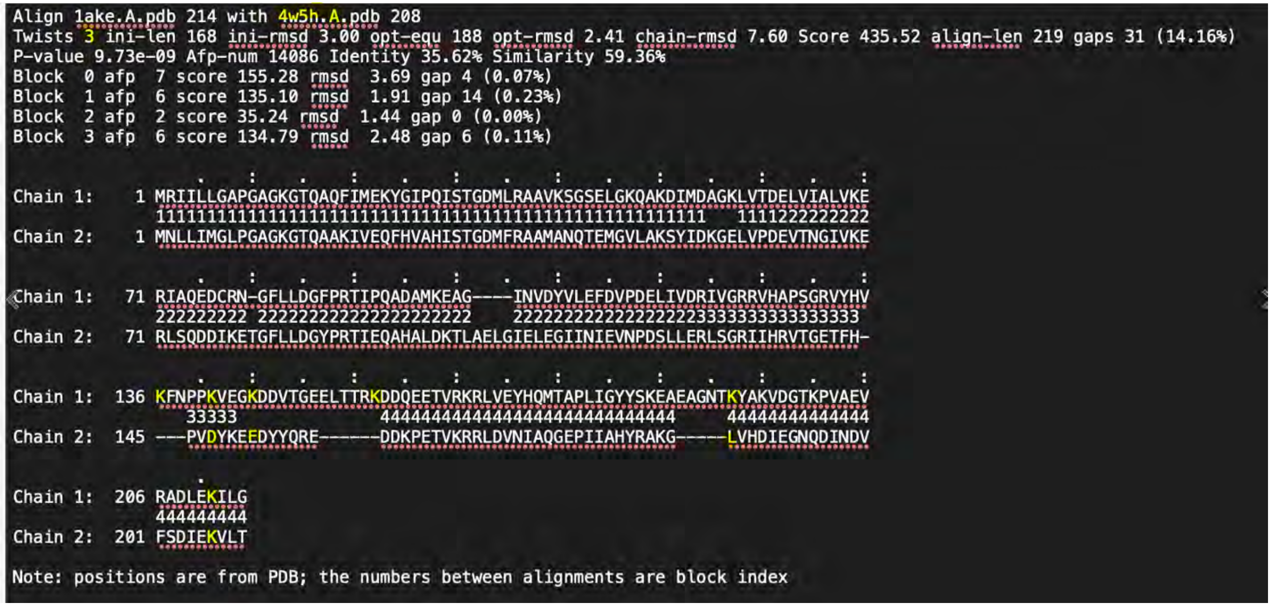

```
Align 1ake.A.pdb 214 with 4w5h.A.pdb 208
Twists 3 ini-len 168 ini-rmsd 3.00 opt-equ 188 opt-rmsd 2.41 chain-rmsd 7.60 Score 435.52 align-len 219 gaps 31 (14.16%)
P-value 9.73e-09 Afp-num 14086 Identity 35.62% Similarity 59.36%
Block  0 afp  7 score 155.28 rmsd  3.69 gap 4 (0.07%)
Block  1 afp  6 score 135.10 rmsd  1.91 gap 14 (0.23%)
Block  2 afp  2 score 35.24 rmsd  1.44 gap 0 (0.00%)
Block  3 afp  6 score 134.79 rmsd  2.48 gap 6 (0.11%)

                 .    :    .    :    .    :    .    :    .    :    .    :    .    :
Chain 1:    1 MRIILLGAPGAGKGTQAQFIMEKYGIPQISTGDMLRAAVKSGSELGKQAKDIMDAGKLVTDELVIALVKE
              1111111111111111111111111111111111111111111111111111111   1111222222222
Chain 2:    1 MNLLIMGLPGAGKGTQAAKIVEQFHVAHISTGDMFRAAMANQTEMGVLAKSYIDKGELVPDEVTNGIVKE

                 .    :    .    :    .    :    .    :    .    :    .    :    .    :
Chain 1:   71 RIAQEDCRN-GFLLDGFPRTIPQADAMKEAG----INVDYVLEFDVPDELIVDRIVGRRVHAPSGRVYHV
              222222222 222222222222222222222    2222222222222222222233333333333333
Chain 2:   71 RLSQDDIKETGFLLDGYPRTIEQAHALDKTLAELGIELEGIINIEVNPDSLLERLSGRIIHRVTGETFH-

                 .    :    .    :    .    :    .    :    .    :    .    :    .    :
Chain 1:  136 KFNPPKVEGKDDVTGEELTTRKDDQEETVRKRLVEYHQMTAPLIGYYSKEAEAGNTKYAKVDGTKPVAEV
                 33333               44444444444444444444444444444     44444444444444
Chain 2:  145 ---PVDYKEEDYYQRE------DDKPETVKRRLDVNIAQGEPIIAHYRAKG-----LVHDIEGNQDINDV

                 .
Chain 1:  206 RADLEKILG
              444444444
Chain 2:  201 FSDIEKVLT

Note: positions are from PDB; the numbers between alignments are block index
```

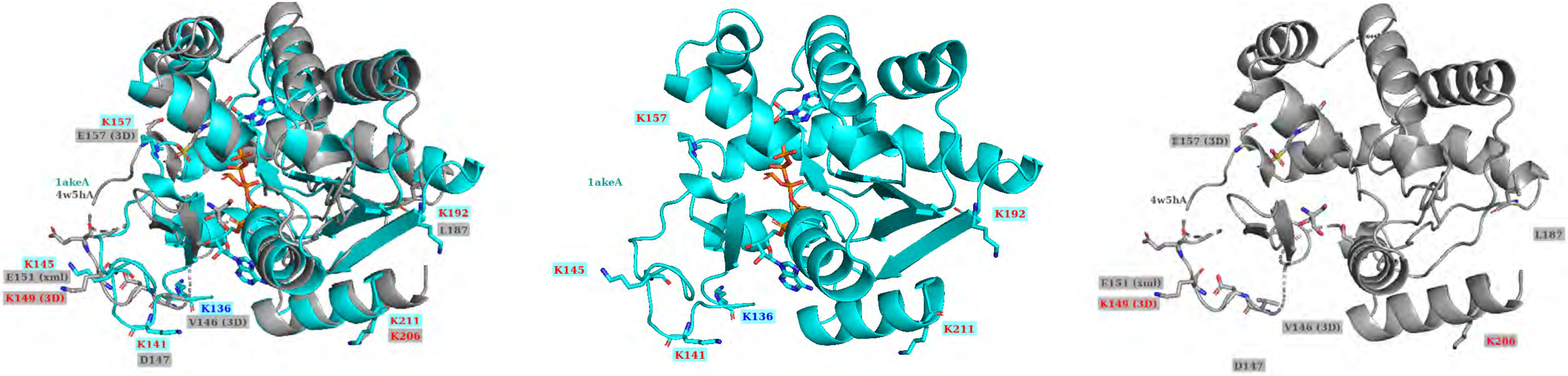

UniProt ID: Q04ML5

PDB ID: 4W5J_B

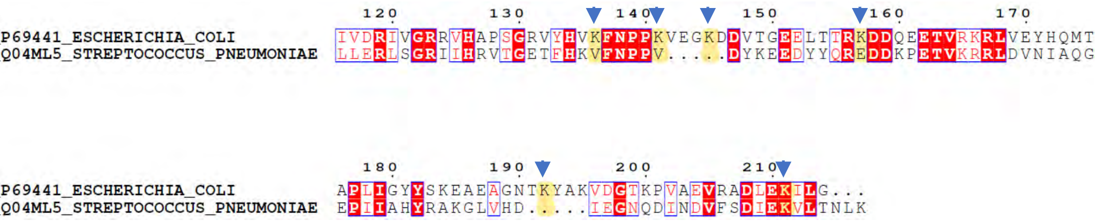


Full sequences in supplemental file.

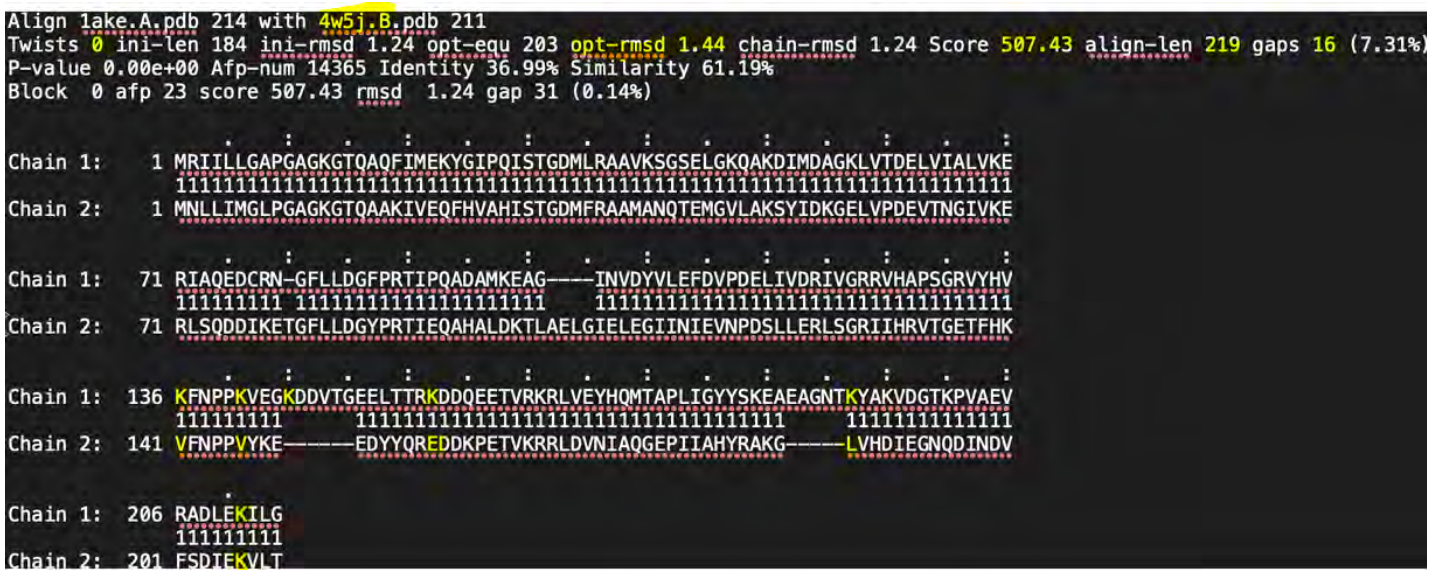

```
Align 1ake.A.pdb 214 with 4w5j.B.pdb 211
Twists 0 ini-len 184 ini-rmsd 1.24 opt-equ 203 opt-rmsd 1.44 chain-rmsd 1.24 Score 507.43 align-len 219 gaps 16 (7.31%)
P-value 0.00e+00 Afp-num 14365 Identity 36.99% Similarity 61.19%
Block  0 afp 23 score 507.43 rmsd  1.24 gap 31 (0.14%)

Chain 1:    1 MRIILLGAPGAGKGTQAQFIMEKYGIPQISTGDMLRAAVKSGSELGKQAKDIMDAGKLVTDELVIALVKE
                1111111111111111111111111111111111111111111111111111111111111111111111
Chain 2:    1 MNLLIMGLPGAGKGTQAAKIVEQFHVAHISTGDMFRAAMANQTEMGVLAKSYIDKGELVPDEVTNGIVKE

Chain 1:   71 RIAQEDCRN-GFLLDGFPRTIPQADAMKEAG----INVDYVLEFDVPDELIVDRIVGRRVHAPSGRVYHV
                111111111 111111111111111111111    111111111111111111111111111111111111
Chain 2:   71 RLSQDDIKETGFLLDGYPRTIEQAHALDKTLAELGIELEGINIEVNPDSLLERLSGRIIHRVTGETFHK

Chain 1:  136 KFNPPKVEGKDDVTGEELTTRKDDQEETVRKRLVEYHQMTAPLIGYYSKEAEAGNTKYAKVDGTKPVAEV
                111111111      1111111111111111111111111111111111111111      11111111111111
Chain 2:  141 VFNPPVYKE-----EDYYQREDDKPETVKRRLDVNIAQGEPIIAHYRAKG-----LVHDIEGNQDINDV

Chain 1:  206 RADLEKILG
                111111111
Chain 2:  201 FSDIEKVLT
```

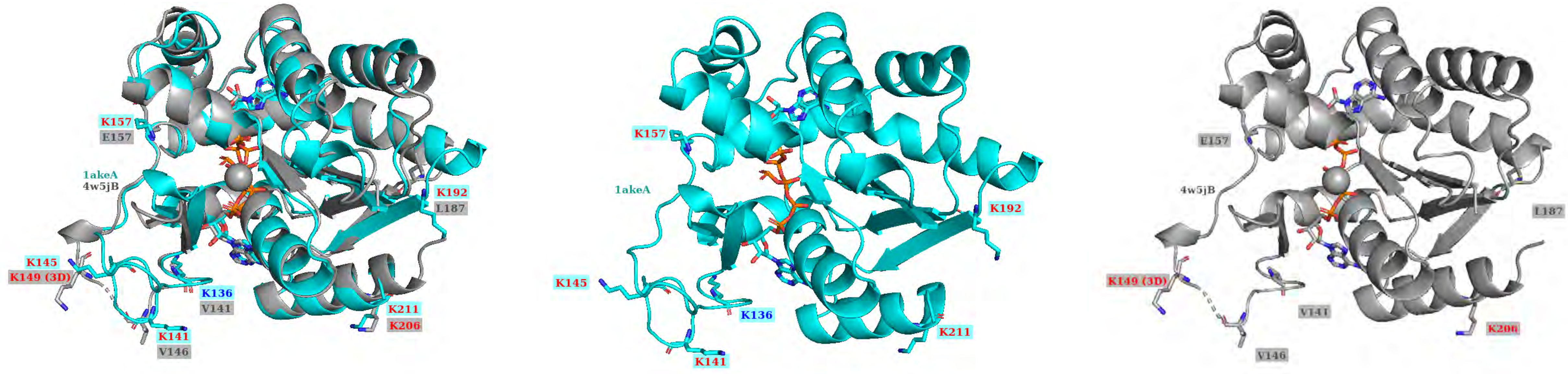

UniProt ID: Q3JVB1

PDB ID: 3GMT_A

```
                                   130       140       150       160       170       180
P69441_ESCHERICHIA_COLI           VGRRVHAPSGRVYHVKFNPPKVEGKDDVTGEELTTRKDDQEETVRKRLVEYHQMTAPLIG
Q3JVB1_BURKHOLDERIA_PSEUDOMALLEI  SGRRTHPASGRTYHVKFNPPKVEGKDDVTGEPLVQRDDDKEETVKKRLDVYEAQTKPLIT

                                   190       200       210
P69441_ESCHERICHIA_COLI           YYSKEAEAGNTK......YAKVDGTKPVAEVRADLEKILG
Q3JVB1_BURKHOLDERIA_PSEUDOMALLEI  YYGDWARRGAENGLKAPAYRKISGLGAVEEIRARVFDALK
```

Full sequences in supplemental file.

```
Align 1ake.A.pdb 214 with 3gmt.A.pdb 204
Twists 3 ini-len 192 ini-rmsd 2.98 opt-equ 197 opt-rmsd 2.42 chain-rmsd 9.47 Score 516.72 align-len 204 gaps 7 (3.43%)
P-value 1.00e-10 Afp-num 13097 Identity 65.69% Similarity 74.02%
Block  0 afp 10 score 220.95 rmsd  3.24 gap 1 (0.01%)
Block  1 afp  4 score 94.26 rmsd  1.44 gap 0 (0.00%)
Block  2 afp  5 score 118.44 rmsd  0.82 gap 0 (0.00%)
Block  3 afp  5 score 102.36 rmsd  2.36 gap 6 (0.13%)

                     .    :    .    :    .    :    .    :    .    :    .    :    .    :
Chain 1:    1 MRIILLGAPGAGKGTQAQFIMEKYGIPQISTGDMLRAAVKSGSELGKQAKDIMDAGKLVTDELVIALVKE
              111111111 111111111111111111111111111111111111111111111111111111111111
Chain 2:    1 MRLILLGAPGAGKGTQANFIKEKFGIPQISTGDMLRAAVKAGTPLGVEAKTYMDEGKLVPDSLIIGLVKE

                     .    :    .    :    .    :    .    :    .    :    .    :    .    :
Chain 1:   71 RIAQEDCRNGFLLDGFPRTIPQADAMKEAGINVDYVLEFDVPDELIVDRIVGRRVHAPSGRVYHVKFNPP
              1111111111111112222222222222222222222222222222223333333333333333333333
Chain 2:   71 RLKEADCANGYLFDGFPRTIAQADAMKEAGVAIDYVLEIDVPFSEIIERMSGRRTHPASGRTYHVKFNPP

                     .    :    .    :    .    :    .    :    .    :    .    :
Chain 1:  141 KVEGKDDVTGEELTTRKDDQEETVRKRLVEYHQMTAPLIGYYSKEAEA------GNTKYAKVDG
              333333333333333334444444444444444444444444444444      4444444444
Chain 2:  141 KVEGKDDVTGEPLVQRDDDKEETVKKRLDVYEAQTKPLITYYGDWARRGAENGLKAPAYRKISG

Note: positions are from PDB; the numbers between alignments are block index
```

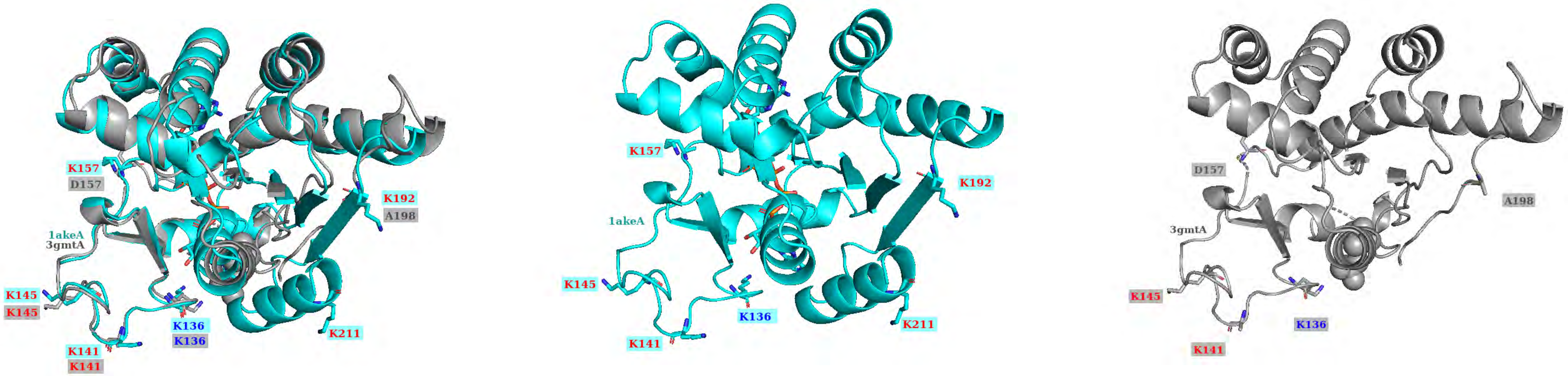

UniProt ID: Q5CRC5

PDB ID: 3BE4_A

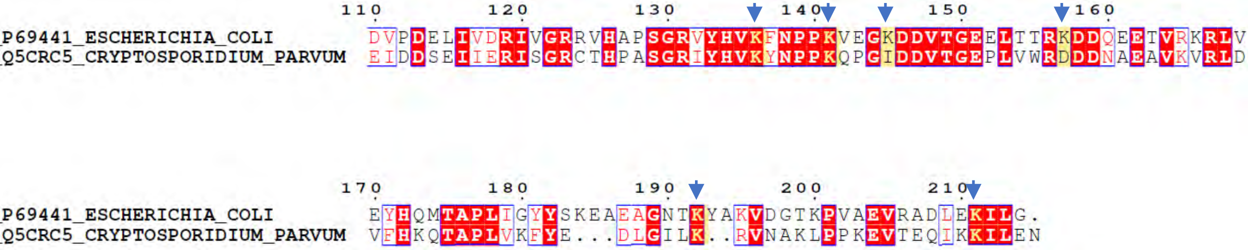


Full sequences in supplemental file.

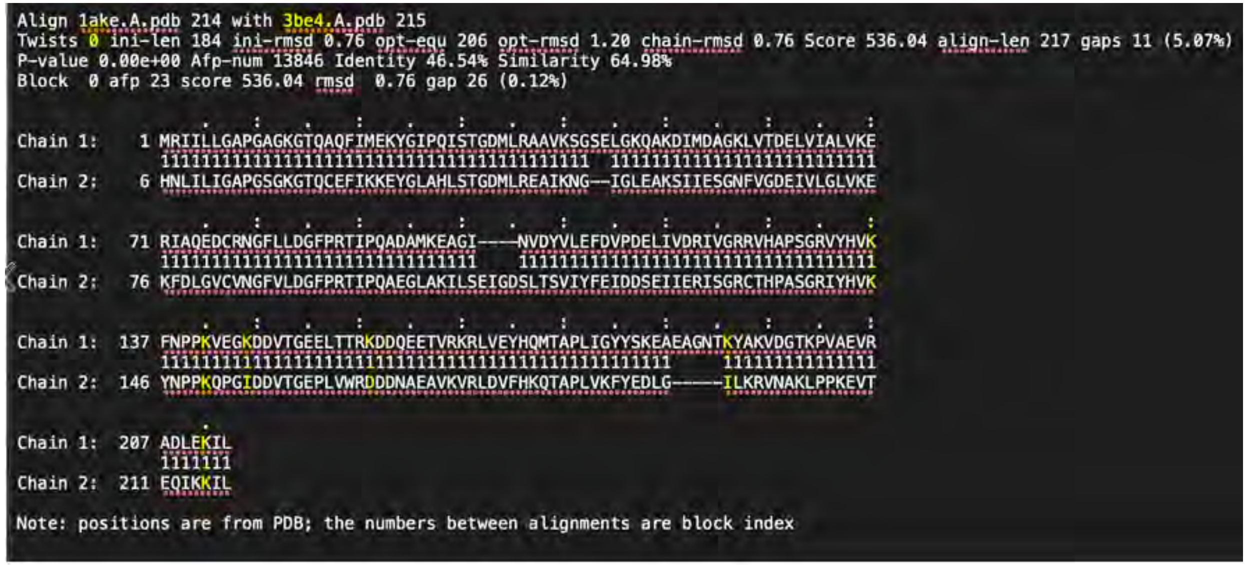

```
Align 1ake.A.pdb 214 with 3be4.A.pdb 215
Twists 0 ini-len 184 ini-rmsd 0.76 opt-equ 206 opt-rmsd 1.20 chain-rmsd 0.76 Score 536.04 align-len 217 gaps 11 (5.07%)
P-value 0.00e+00 Afp-num 13846 Identity 46.54% Similarity 64.98%
Block  0 afp 23 score 536.04 rmsd  0.76 gap 26 (0.12%)

Chain 1:    1 MRIILLGAPGAGKGTQAQFIMEKYGIPQISTGDMLRAAVKSGSELGKQAKDIMDAGKLVTDELVIALVKE
              111111111111111111111111111111111111111111  111111111111111111111111111
Chain 2:    6 HNLILIGAPGSGKGTQCEFIKKEYGLAHLSTGDMLREAIKNG--IGLEAKSIIESGNFVGDEIVLGLVKE

Chain 1:   71 RIAQEDCRNGFLLDGFPRTIPQADAMKEAGI----NVDYVLEFDVPDELIVDRIVGRRVHAPSGRVYHVK
              1111111111111111111111111111111    11111111111111111111111111111111111
Chain 2:   76 KFDLGVCVNGFVLDGFPRTIPQAEGLAKILSEIGDSLTSVIYFEIDDSEIIERISGRCTHPASGRIYHVK

Chain 1:  137 FNPPKVEGKDDVTGEELTTRKDDQEETVRKRLVEYHQMTAPLIGYYSKEAEAGNTKYAKVDGTKPVAEVR
              1111111111111111111111111111111111111111111111111111     111111111111111
Chain 2:  146 YNPPKQPGIDDVTGEPLVWRDDDNAEAVKVRLDVFHKQTAPLVKFYEDLG-----ILKRVNAKLPPKEVT

Chain 1:  207 ADLEKIL
              1111111
Chain 2:  211 EQIKKIL

Note: positions are from PDB; the numbers between alignments are block index
```

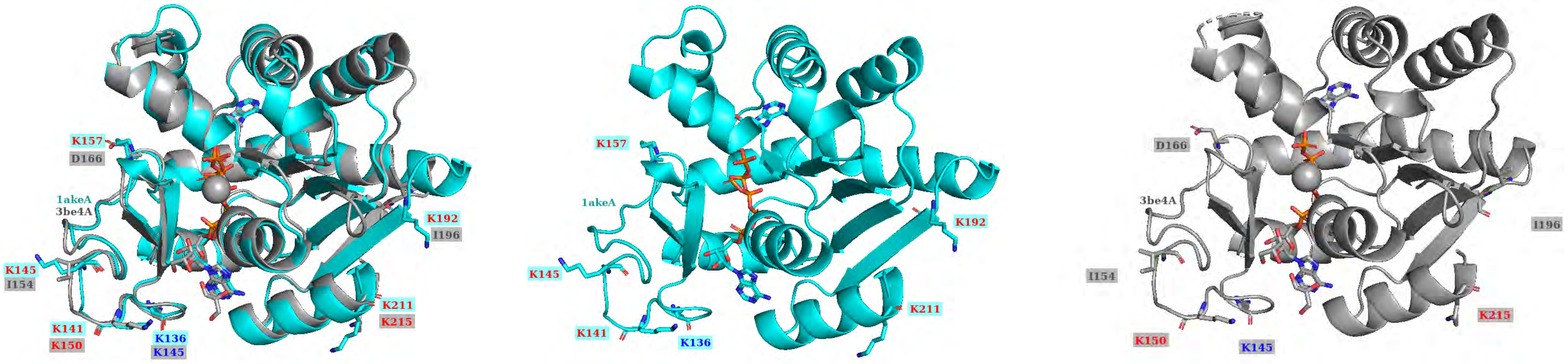

UniProt ID: Q5NFR4

PDB ID: 4PZL_D

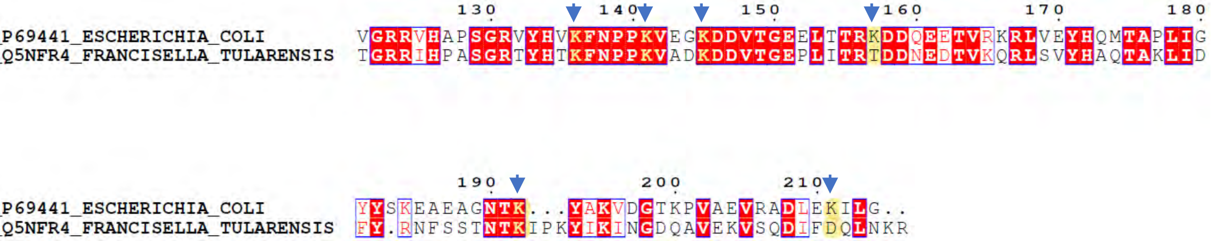


Full sequences in supplemental file.

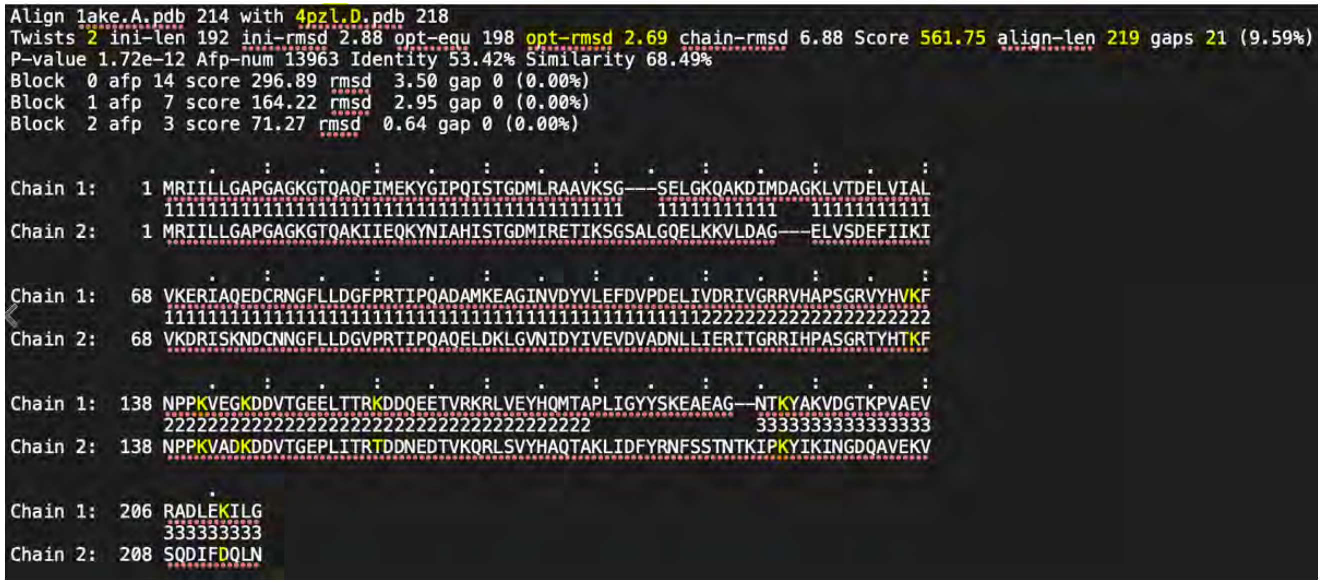

```
Align 1ake.A.pdb 214 with 4pzl.D.pdb 218
Twists 2 ini-len 192 ini-rmsd 2.88 opt-equ 198 opt-rmsd 2.69 chain-rmsd 6.88 Score 561.75 align-len 219 gaps 21 (9.59%)
P-value 1.72e-12 Afp-num 13963 Identity 53.42% Similarity 68.49%
Block  0 afp 14 score 296.89 rmsd  3.50 gap 0 (0.00%)
Block  1 afp  7 score 164.22 rmsd  2.95 gap 0 (0.00%)
Block  2 afp  3 score 71.27 rmsd  0.64 gap 0 (0.00%)

                 .    :    .    :    .    :    .    :    .    :    .    :    .    :
Chain 1:    1 MRIILLGAPGAGKGTQAQFIMEKYGIPQISTGDMLRAAVKSG---SELGKQAKDIMDAGKLVTDELVIAL
              111111111111111111111111111111111111111111   11111111111   11111111111
Chain 2:    1 MRIILLGAPGAGKGTQAKIIEQKYNIAHISTGDMIRETIKSGSALGQELKKVLDAG---ELVSDEFIIKI

                 .    :    .    :    .    :    .    :    .    :    .    :    .    :
Chain 1:   68 VKERIAQEDCRNGFLLDGFPRTIPQADAMKEAGINVDYVLEFDVPDELIVDRIVGRRVHAPSGRVYHVKF
              1111111111111111111111111111111111111111111111111122222222222222222222
Chain 2:   68 VKDRISKNDCNNGFLLDGVPRTIPQAQELDKLGVNIDYIVEVDVADNLLIERITGRRIHPASGRTYHTKF

                 .    :    .    :    .    :    .    :    .    :    .    :    .    :
Chain 1:  138 NPPKVEGKDDVTGEELTTRKDDQEETVRKRLVEYHQMTAPLIGYYSKEAEAG--NTKYAKVDGTKPVAEV
              222222222222222222222222222222222222222222                 3333333333333333
Chain 2:  138 NPPKVADKDDVTGEPLITRTDDNEDTVKQRLSVYHAQTAKLIDFYRNFSSTNTKIPKYIKINGDQAVEKV

                 .
Chain 1:  206 RADLEKILG
              333333333
Chain 2:  208 SQDIFDQLN
```

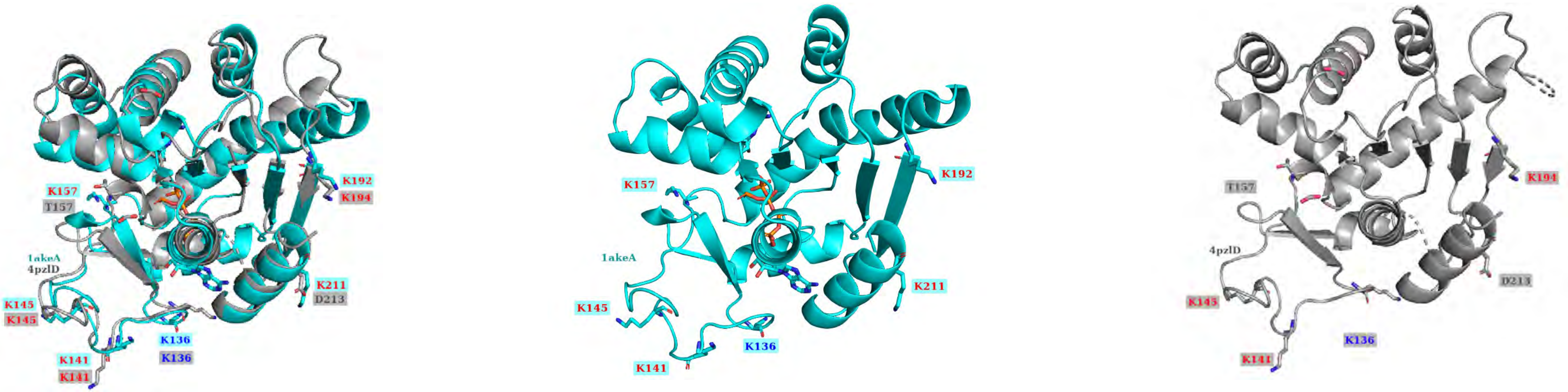

UniProt ID: Q5SHQ9

PDB ID: 3CM0_A

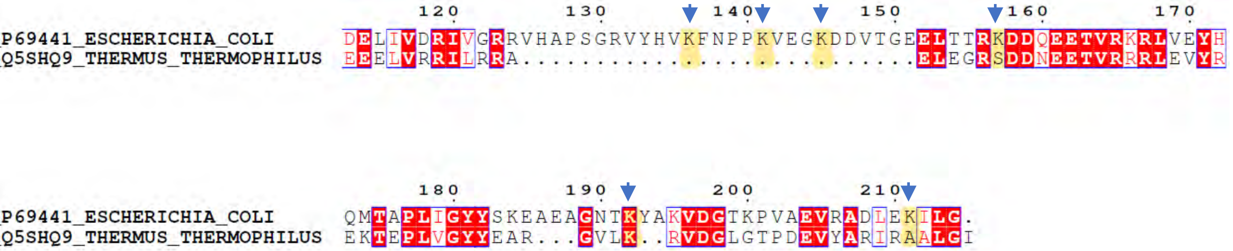


Full sequences in supplemental file.

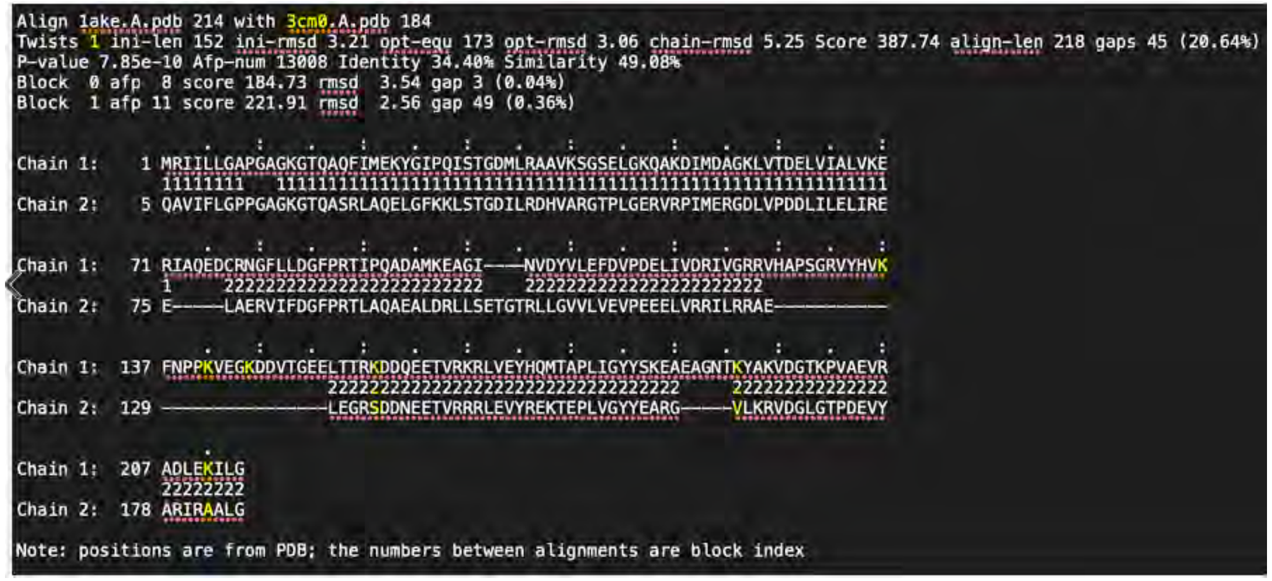

```
Align 1ake.A.pdb 214 with 3cm0.A.pdb 184
Twists 1 ini-len 152 ini-rmsd 3.21 opt-equ 173 opt-rmsd 3.06 chain-rmsd 5.25 Score 387.74 align-len 218 gaps 45 (20.64%)
P-value 7.85e-10 Afp-num 13008 Identity 34.40% Similarity 49.08%
Block  0 afp  8 score 184.73 rmsd  3.54 gap 3 (0.04%)
Block  1 afp 11 score 221.91 rmsd  2.56 gap 49 (0.36%)

Chain 1:    1 MRIILLGAPGAGKGTQAQFIMEKYGIPQISTGDMLRAAVKSGSELGKQAKDIMDAGKLVTDELVIALVKE
              11111111   11111111111111111111111111111111111111111111111111111111111
Chain 2:    5 QAVIFLGPPGAGKGTQASRLAQELGFKKLSTGDILRDHVARGTPLGERVRPIMERGDLVPDDLILELIRE

Chain 1:   71 RIAQEDCRNGFLLDGFPRTIPQADAMKEAGI---NVDYVLEFDVPDELIVDRIVGRRVHAPSGRVYHVK
              1     2222222222222222222222222    22222222222222222222222
Chain 2:   75 E-----LAERVIFDGFPRTLAQAEALDRLLSETGTRLLGVVLVEVPEEELVRRILRRAE-----------

Chain 1:  137 FNPPKVEGKDDVTGEELTTRKDDQEETVRKRLVEYHQMTAPLIGYYSKEAEAGNTKYAKVDGTKPVAEVR
                              2222222222222222222222222222222222     222222222222222
Chain 2:  129 ----------------LEGRSDDNEETVRRRLEVYREKTEPLVGYYEARG-----VLKRVDGLGTPDEVY

Chain 1:  207 ADLEKILG
              22222222
Chain 2:  178 ARIRAALG

Note: positions are from PDB; the numbers between alignments are block index
```

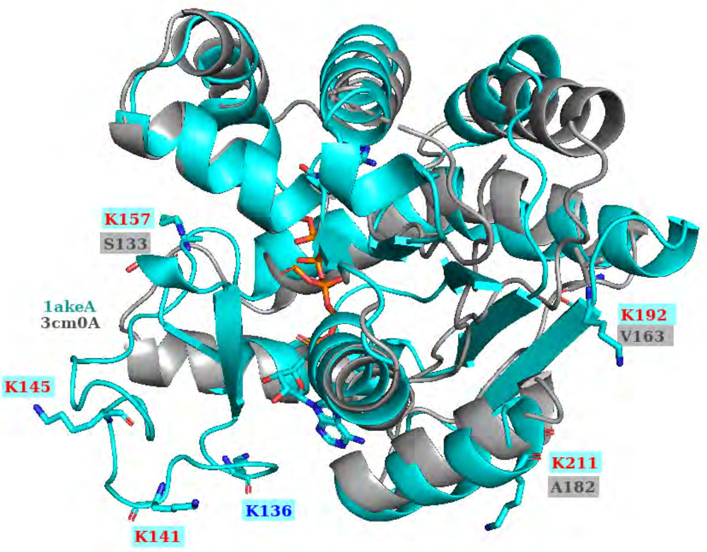


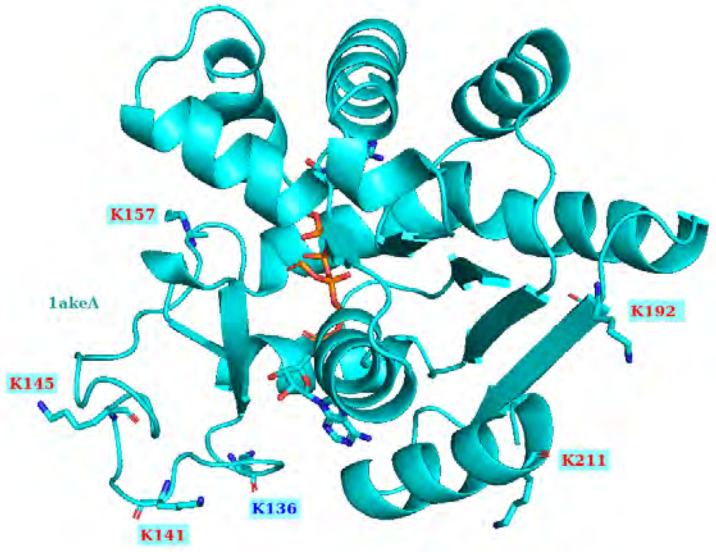


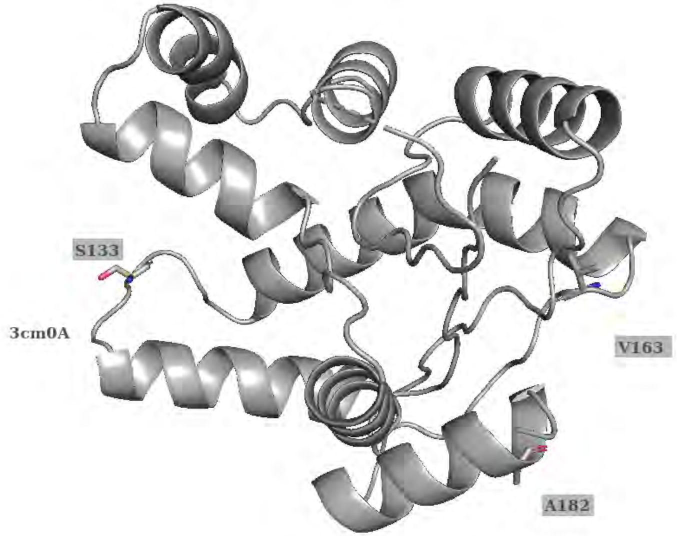

UniProt ID: Q68EH2

PDB ID: 5XZ2_A

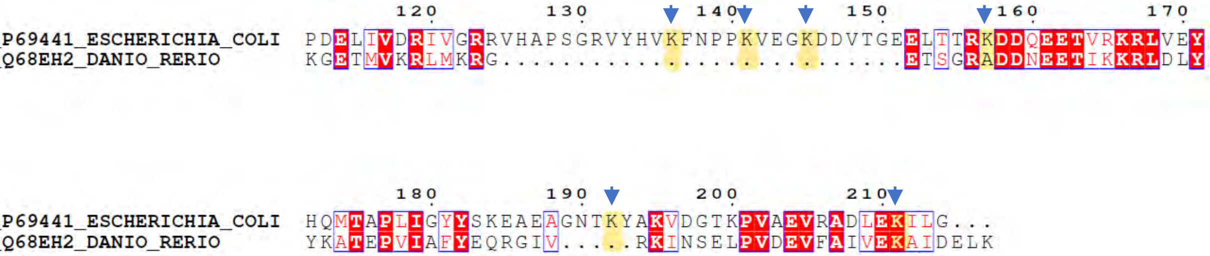


Full sequences in supplemental file.

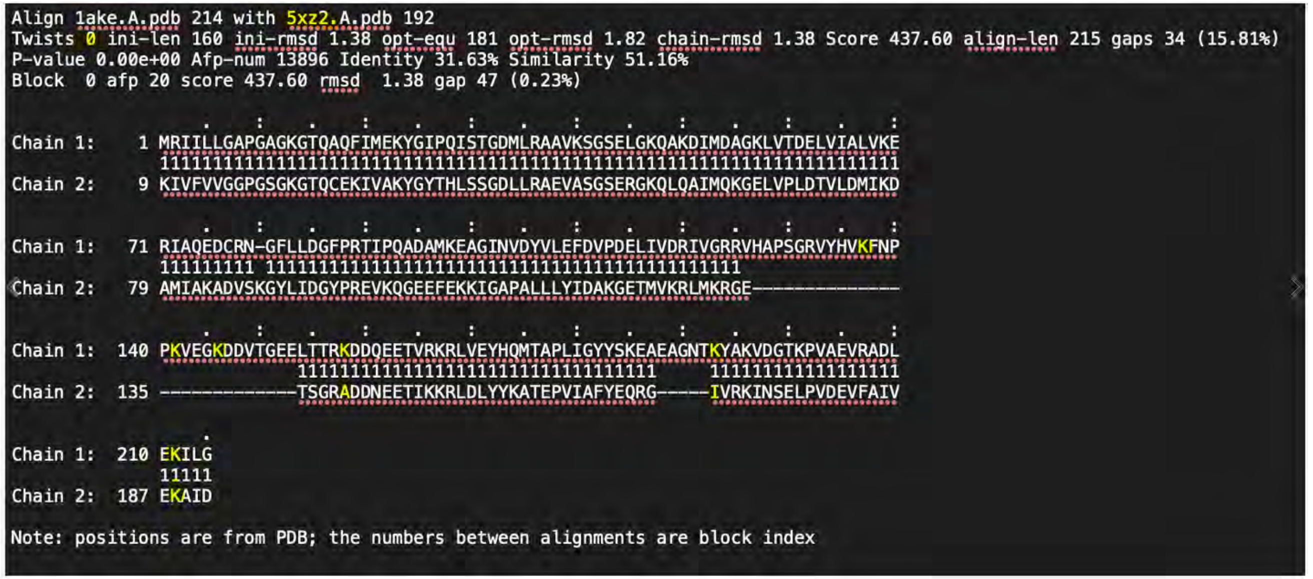


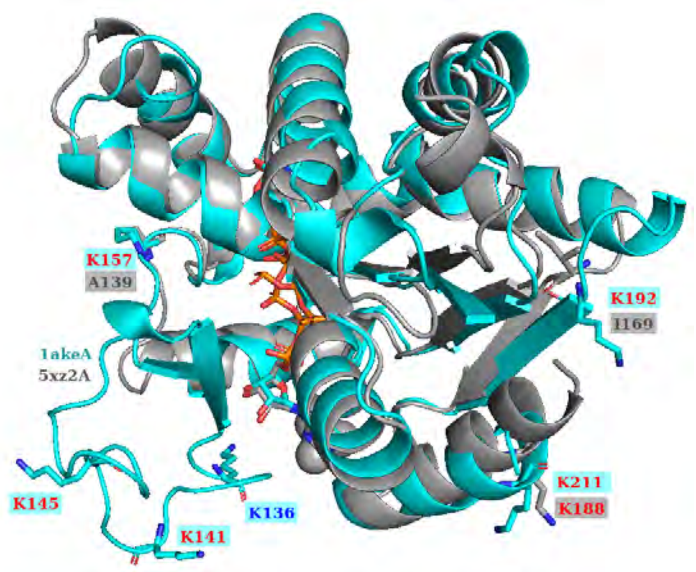


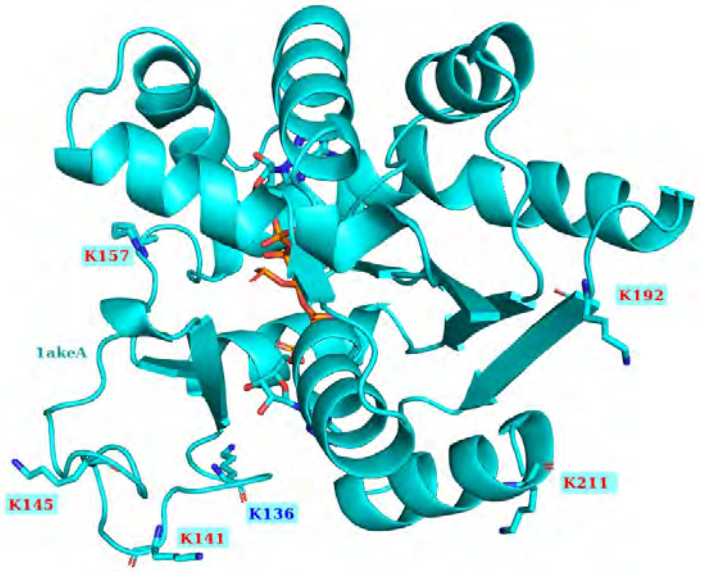


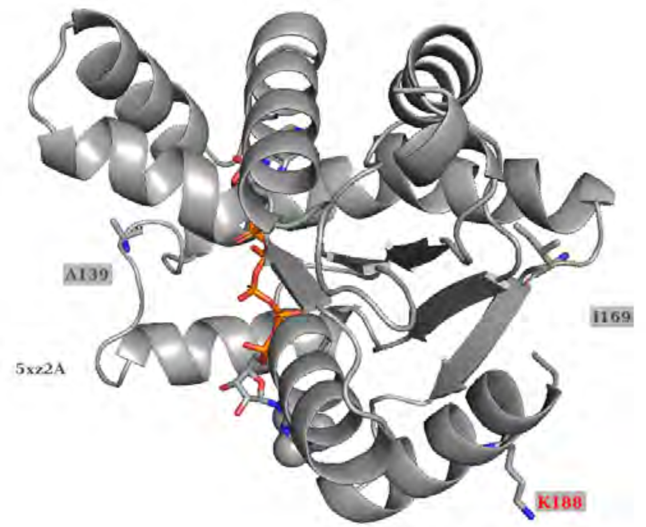

UniProt ID: Q6B341
PDB ID: 3FB4_A

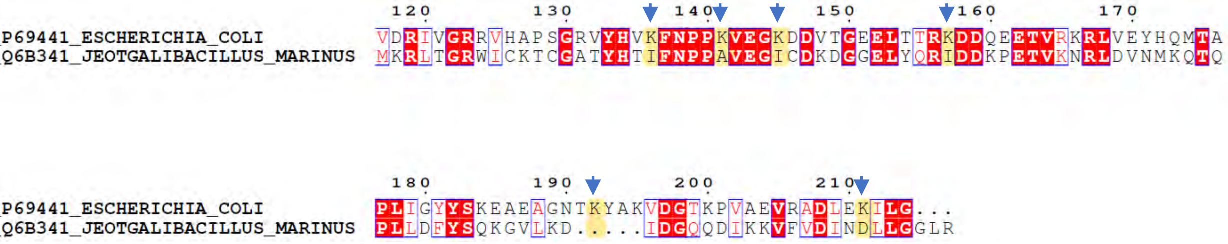


Full sequences in supplemental file.

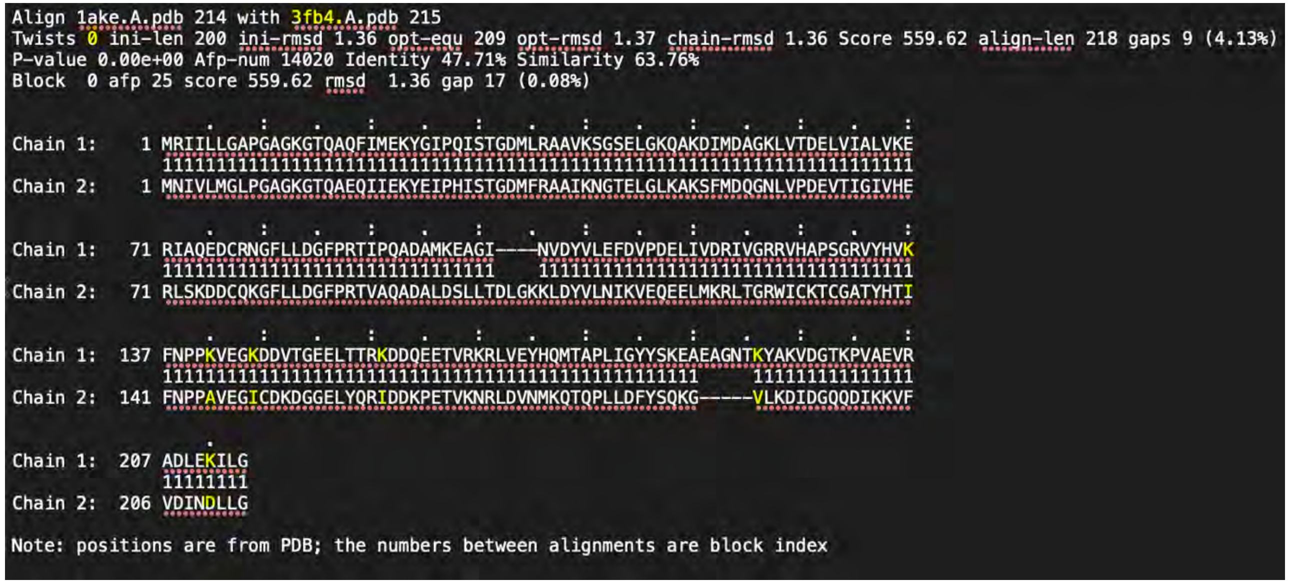


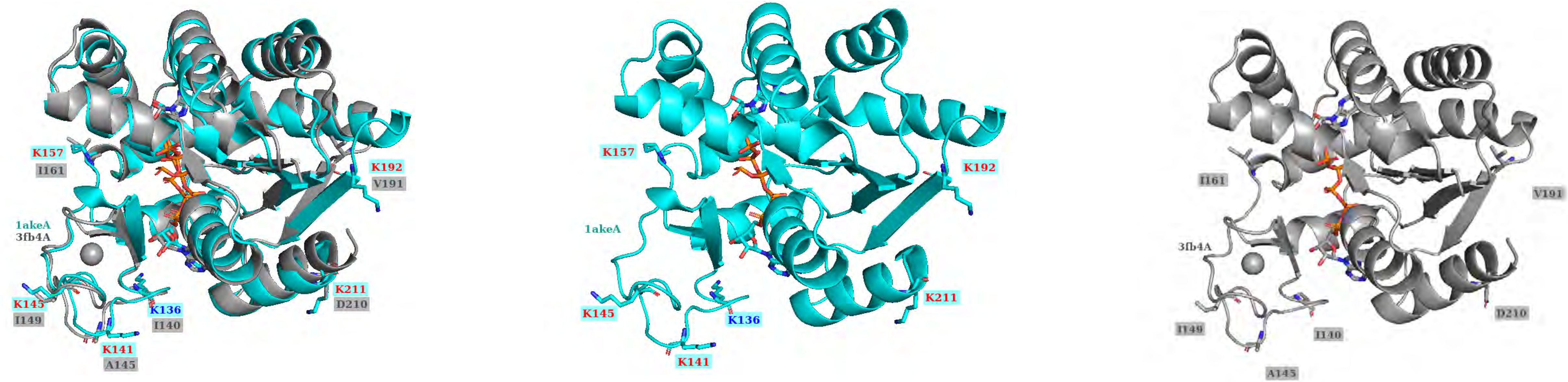

UniProt ID: Q6LTE1

PDB ID: 4K46_A

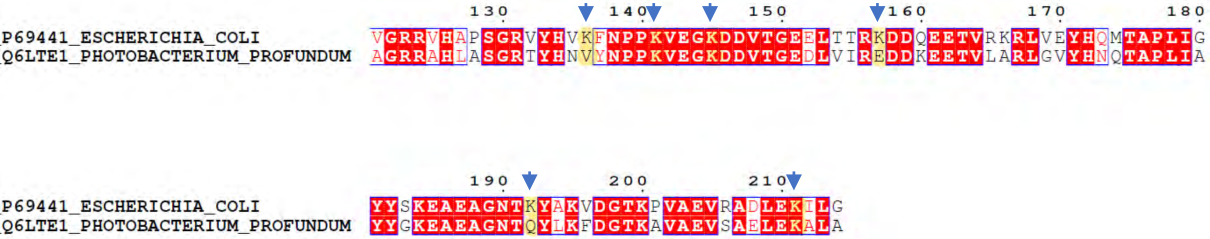


Full sequences in supplemental file.

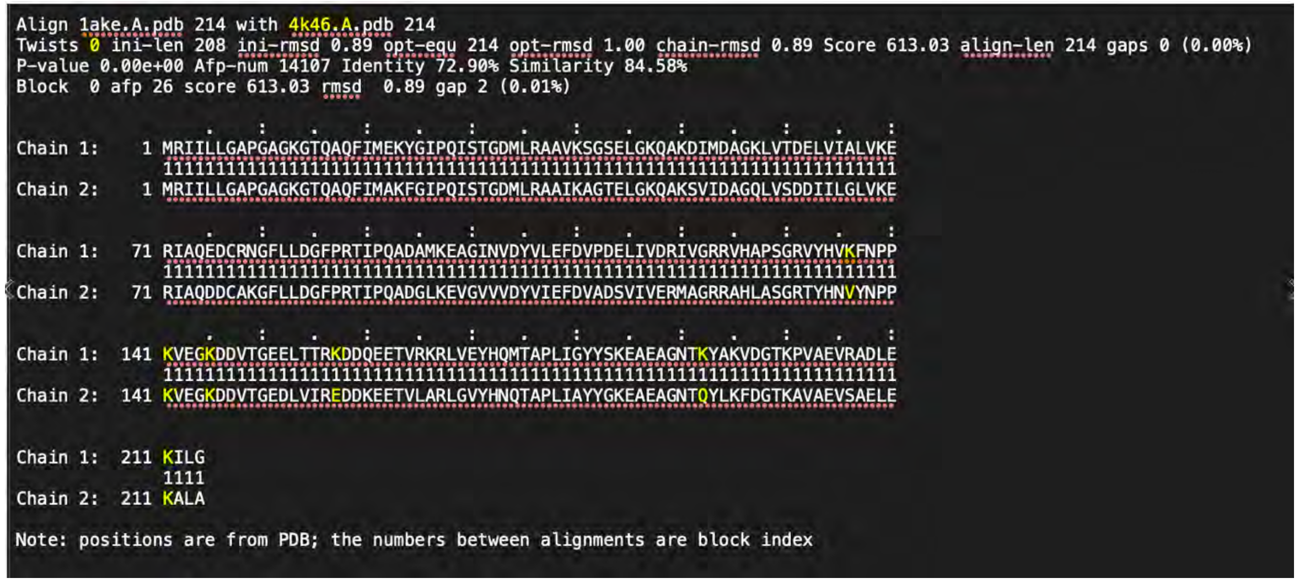

```
Align 1ake.A.pdb 214 with 4k46.A.pdb 214
Twists 0 ini-len 208 ini-rmsd 0.89 opt-equ 214 opt-rmsd 1.00 chain-rmsd 0.89 Score 613.03 align-len 214 gaps 0 (0.00%)
P-value 0.00e+00 Afp-num 14107 Identity 72.90% Similarity 84.58%
Block  0 afp 26 score 613.03 rmsd  0.89 gap 2 (0.01%)

                 .    :    .    :    .    :    .    :    .    :    .    :    .    :
Chain 1:    1 MRIILLGAPGAGKGTQAQFIMEKYGIPQISTGDMLRAAVKSGSELGKQAKDIMDAGKLVTDELVIALVKE
              1111111111111111111111111111111111111111111111111111111111111111111111
Chain 2:    1 MRIILLGAPGAGKGTQAQFIMAKFGIPQISTGDMLRAAIKAGTELGKQAKSVIDAGQLVSDDIILGLVKE

                 .    :    .    :    .    :    .    :    .    :    .    :    .    :
Chain 1:   71 RIAQEDCRNGFLLDGFPRTIPQADAMKEAGINVDYVLEFDVPDELIVDRIVGRRVHAPSGRVYHVKFNPP
              1111111111111111111111111111111111111111111111111111111111111111111111
Chain 2:   71 RIAQDDCAKGFLLDGFPRTIPQADGLKEVGVVVDYVIEFDVADSVIVERMAGRRAHLASGRTYHNVYNPP

                 .    :    .    :    .    :    .    :    .    :    .    :    .    :
Chain 1:  141 KVEGKDDVTGEELTTRKDDQEETVRKRLVEYHQMTAPLIGYYSKEAEAGNTKYAKVDGTKPVAEVRADLE
              1111111111111111111111111111111111111111111111111111111111111111111111
Chain 2:  141 KVEGKDDVTGEDLVIREDDKEETVLARLGVYHNQTAPLIAYYGKEAEAGNTQYLKFDGTKAVAEVSAELE

Chain 1:  211 KILG
              1111
Chain 2:  211 KALA

Note: positions are from PDB; the numbers between alignments are block index
```

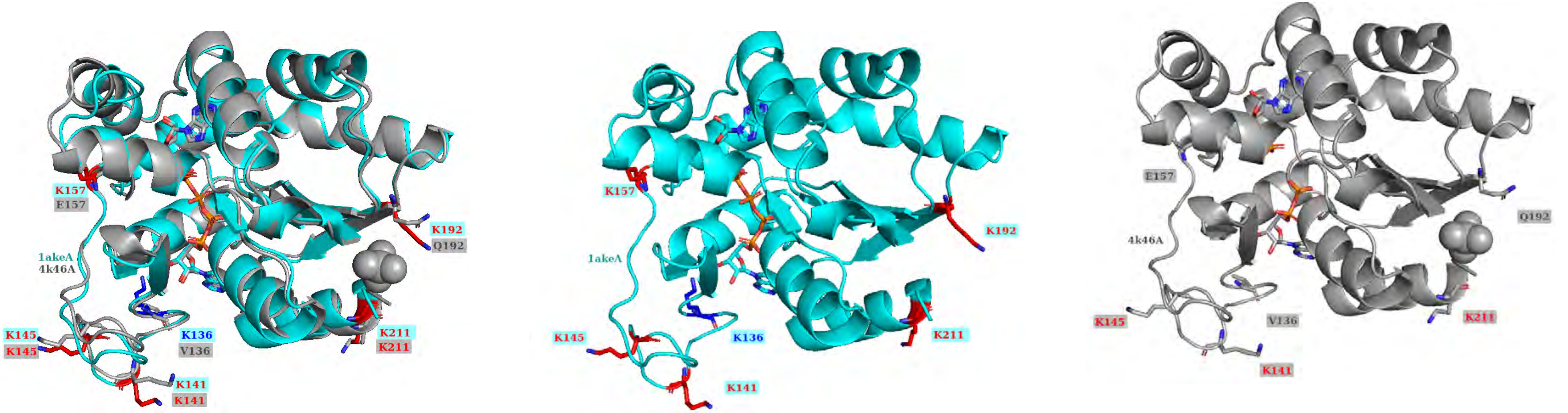

UniProt ID: Q7Z0H0

PDB ID: 3TLX_A

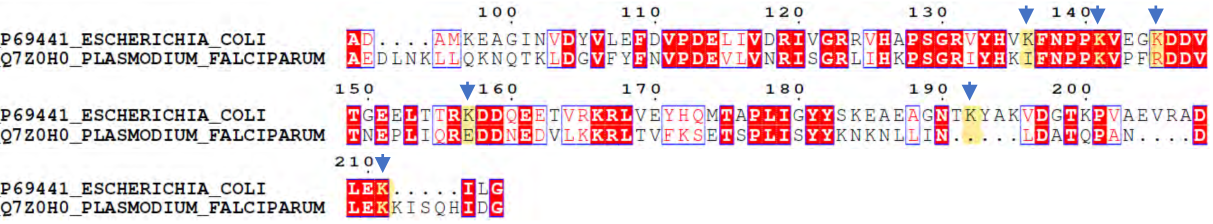


Full sequences in supplemental file.

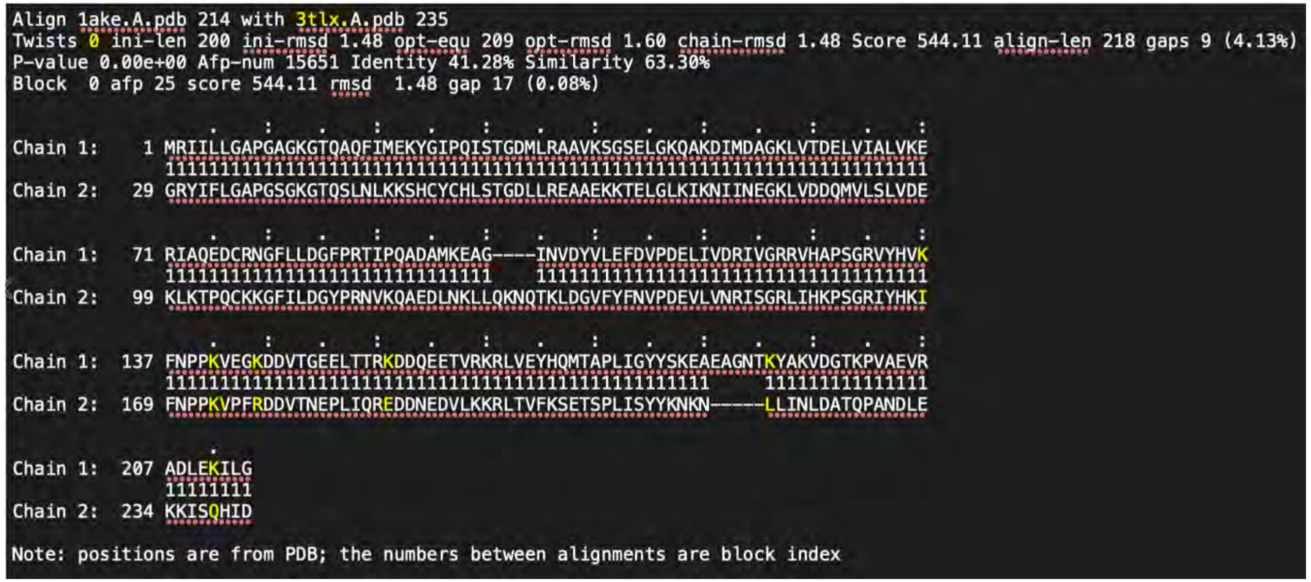

```
Align 1ake.A.pdb 214 with 3tlx.A.pdb 235
Twists 0 ini-len 200 ini-rmsd 1.48 opt-equ 209 opt-rmsd 1.60 chain-rmsd 1.48 Score 544.11 align-len 218 gaps 9 (4.13%)
P-value 0.00e+00 Afp-num 15651 Identity 41.28% Similarity 63.30%
Block  0 afp 25 score 544.11 rmsd  1.48 gap 17 (0.08%)

                  .    :    .    :    .    :    .    :    .    :    .    :    .    :
Chain 1:    1 MRIILLGAPGAGKGTQAQFIMEKYGIPQISTGDMLRAAVKSGSELGKQAKDIMDAGKLVTDELVIALVKE
              1111111111111111111111111111111111111111111111111111111111111111111111
Chain 2:   29 GRYIFLGAPGSGKGTQSLNLKKSHCYCHLSTGDLLREAAEKKTELGLKIKNIINEGKLVDDQMVLSLVDE

                  .    :    .    :    .    :    .    :    .    :    .    :    .    :
Chain 1:   71 RIAQEDCRNGFLLDGFPRTIPQADAMKEAG----INVDYVLEFDVPDELIVDRIVGRRVHAPSGRVYHVK
              111111111111111111111111111111    111111111111111111111111111111111111
Chain 2:   99 KLKTPQCKKGFILDGYPRNVKQAEDLNKLLQKNQTKLDGVFYFNVPDEVLVNRISGRLIHKPSGRIYHKI

                  .    :    .    :    .    :    .    :    .    :    .    :    .    :
Chain 1:  137 FNPPKVEGKDDVTGEELTTRKDDQEETVRKRLVEYHQMTAPLIGYYSKEAEAGNTKYAKVDGTKPVAEVR
              11111111111111111111111111111111111111111111111111     111111111111111
Chain 2:  169 FNPPKVPFRDDVTNEPLIQREDDNEDVLKKRLTVFKSETSPLISYYKNKN-----LLINLDATQPANDLE

                  .
Chain 1:  207 ADLEKILG
              11111111
Chain 2:  234 KKISQHID

Note: positions are from PDB; the numbers between alignments are block index
```

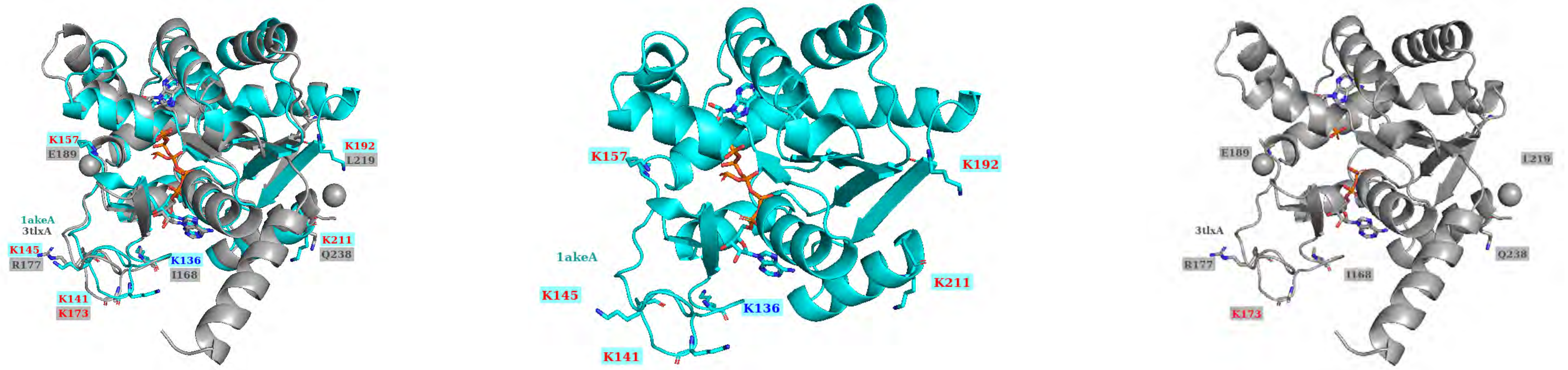

UniProt ID: Q9KTB7
PDB ID: 4NP6_B

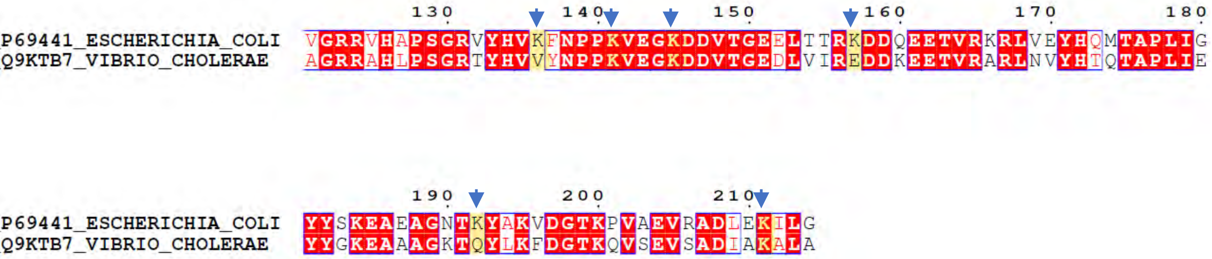


Full sequences in supplemental file.

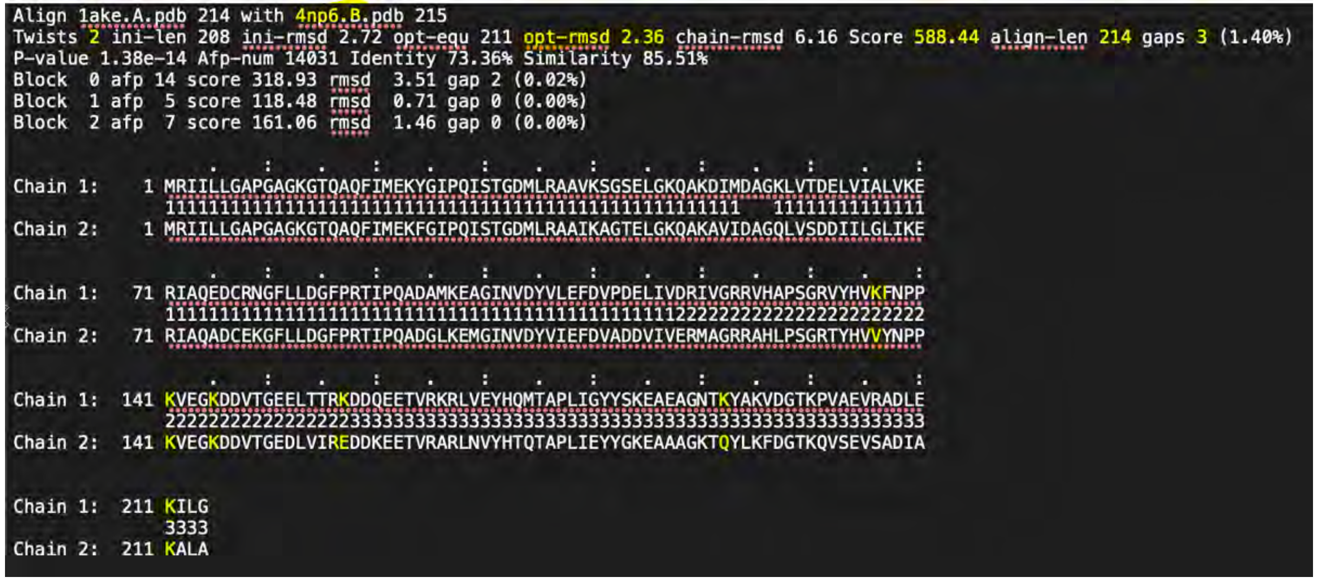

```
Align 1ake.A.pdb 214 with 4np6.B.pdb 215
Twists 2 ini-len 208 ini-rmsd 2.72 opt-equ 211 opt-rmsd 2.36 chain-rmsd 6.16 Score 588.44 align-len 214 gaps 3 (1.40%)
P-value 1.38e-14 Afp-num 14031 Identity 73.36% Similarity 85.51%
Block  0 afp 14 score 318.93 rmsd  3.51 gap 2 (0.02%)
Block  1 afp  5 score 118.48 rmsd  0.71 gap 0 (0.00%)
Block  2 afp  7 score 161.06 rmsd  1.46 gap 0 (0.00%)

                .    :    .    :    .    :    .    :    .    :    .    :    .    :
Chain 1:    1 MRIILLGAPGAGKGTQAQFIMEKYGIPQISTGDMLRAAVKSGSELGKQAKDIMDAGKLVTDELVIALVKE
              11111111111111111111111111111111111111111111111111111   11111111111111
Chain 2:    1 MRIILLGAPGAGKGTQAQFIMEKFGIPQISTGDMLRAAIKAGTELGKQAKAVIDAGQLVSDDIILGLIKE

                .    :    .    :    .    :    .    :    .    :    .    :    .    :
Chain 1:   71 RIAQEDCRNGFLLDGFPRTIPQADAMKEAGINVDYVLEFDVPDELIVDRIVGRRVHAPSGRVYHVKFNPP
              1111111111111111111111111111111111111111111111222222222222222222222222
Chain 2:   71 RIAQADCEKGFLLDGFPRTIPQADGLKEMGINVDYVIEFDVADDVIVERMAGRRAHLPSGRTYHVVYNPP

                .    :    .    :    .    :    .    :    .    :    .    :    .    :
Chain 1:  141 KVEGKDDVTGEELTTRKDDQEETVRKRLVEYHQMTAPLIGYYSKEAEAGNTKYAKVDGTKPVAEVRADLE
              2222222222222222233333333333333333333333333333333333333333333333333333
Chain 2:  141 KVEGKDDVTGEDLVIREDDKEETVRARLNVYHTQTAPLIEYYGKEAAAGKTQYLKFDGTKQVSEVSADIA


Chain 1:  211 KILG
              3333
Chain 2:  211 KALA
```

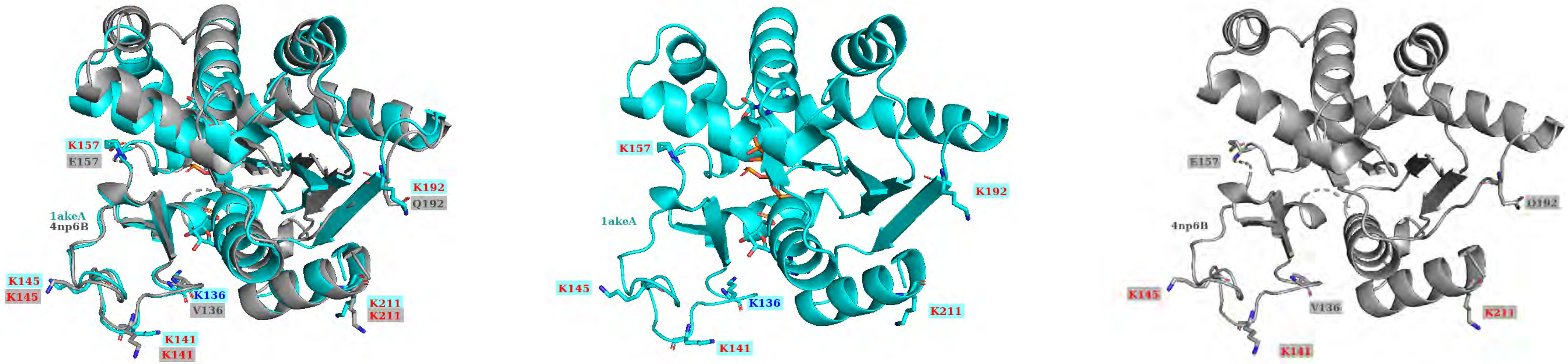

UniProt ID: Q9UIJ7

PDB ID: 1ZD8_A

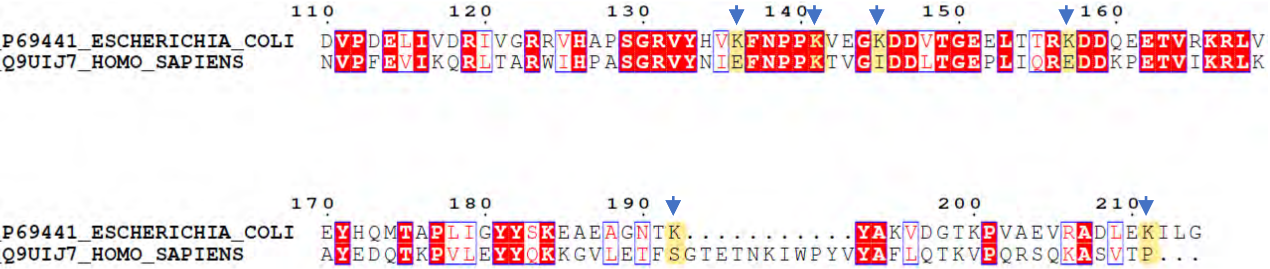


Full sequences in supplemental file.

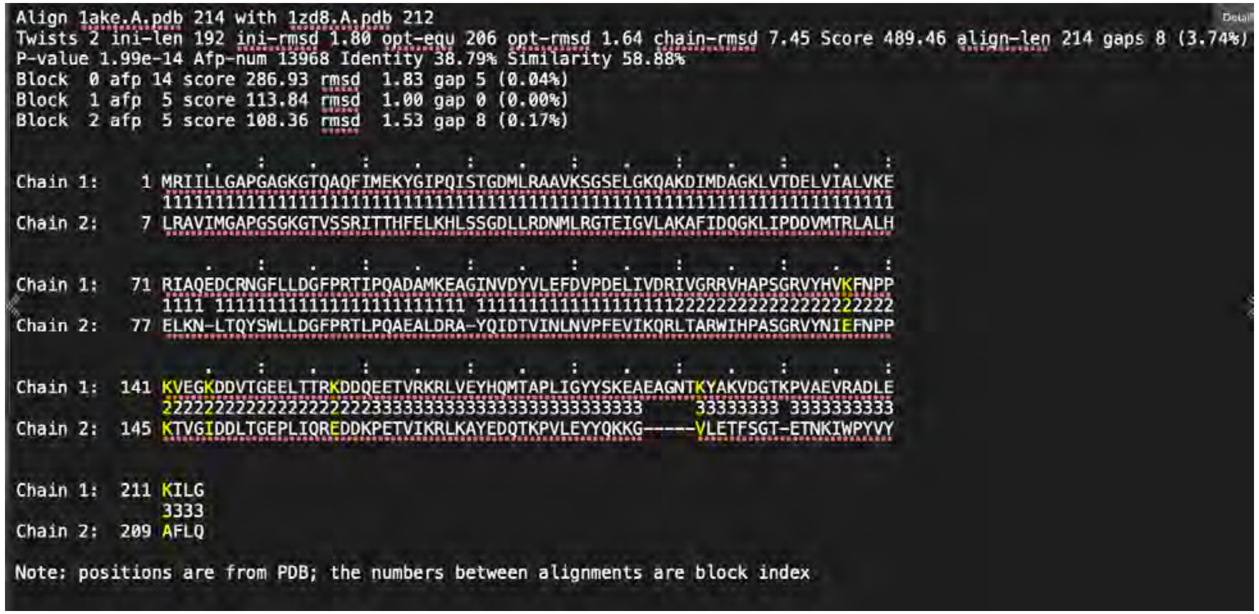

```
Align 1ake.A.pdb 214 with 1zd8.A.pdb 212
Twists 2 ini-len 192 ini-rmsd 1.80 opt-equ 206 opt-rmsd 1.64 chain-rmsd 7.45 Score 489.46 align-len 214 gaps 8 (3.74%)
P-value 1.99e-14 Afp-num 13968 Identity 38.79% Similarity 58.88%
Block  0 afp 14 score 286.93 rmsd  1.83 gap 5 (0.04%)
Block  1 afp  5 score 113.84 rmsd  1.00 gap 0 (0.00%)
Block  2 afp  5 score 108.36 rmsd  1.53 gap 8 (0.17%)

Chain 1:    1 MRIILLGAPGAGKGTQAQFIMEKYGIPQISTGDMLRAAVKSGSELGKQAKDIMDAGKLVTDELVIALVKE
              1111111111111111111111111111111111111111111111111111111111111111111111
Chain 2:    7 LRAVIMGAPGSGKGTVSSRITTHFELKHLSSGDLLRDNMLRGTEIGVLAKAFIDQGKLIPDDVMTRLALH

Chain 1:   71 RIAQEDCRNGFLLDGFPRTIPQADAMKEAGINVDYVLEFDVPDELIVDRIVGRRVHAPSGRVYHVKFNPP
              1111 111111111111111111111111 11111111111111111111222222222222222222222
Chain 2:   77 ELKN-LTQYSWLLDGFPRTLPQAEALDRA-YQIDTVINLNVPFEVIKQRLTARWIHPASGRVYNIEFNPP

Chain 1:  141 KVEGKDDVTGEELTTRKDDQEETVRKRLVEYHQMTAPLIGYYSKEAEAGNTKYAKVDGTKPVAEVRADLE
              2222222222222222222333333333333333333333333333     33333333 3333333333
Chain 2:  145 KTVGIDDLTGEPLIQREDDKPETVIKRLKAYEDQTKPVLEYYQKKG-----VLETFSGT-ETNKIWPYVY

Chain 1:  211 KILG
              3333
Chain 2:  209 AFLQ

Note: positions are from PDB; the numbers between alignments are block index
```

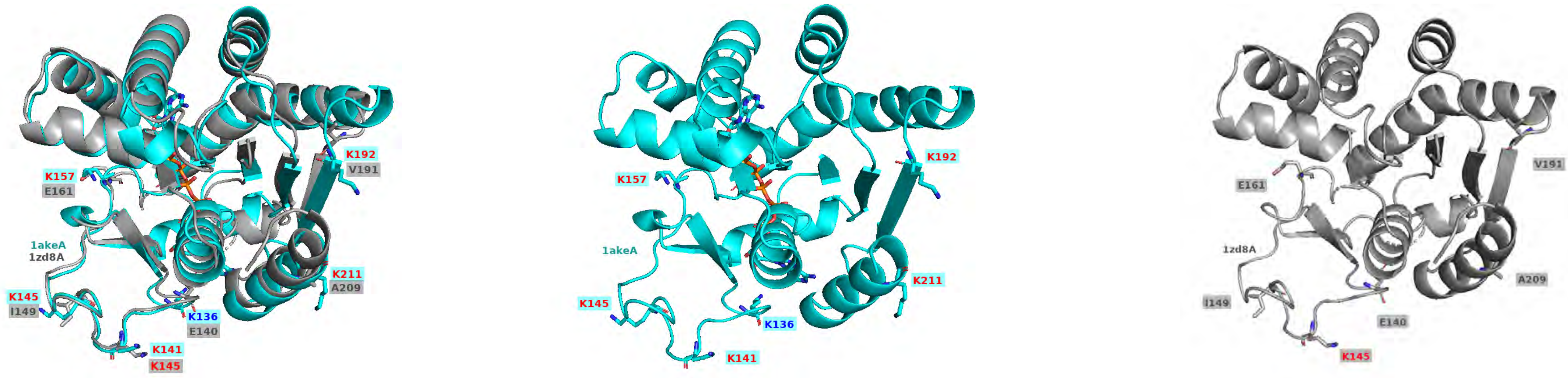

UniProt ID: Q9UIJ7

PDB ID: 6ZJB_A

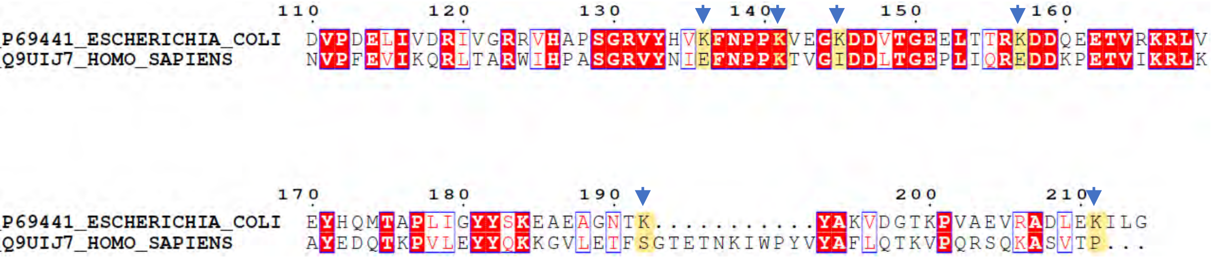


Full sequences in supplemental file.

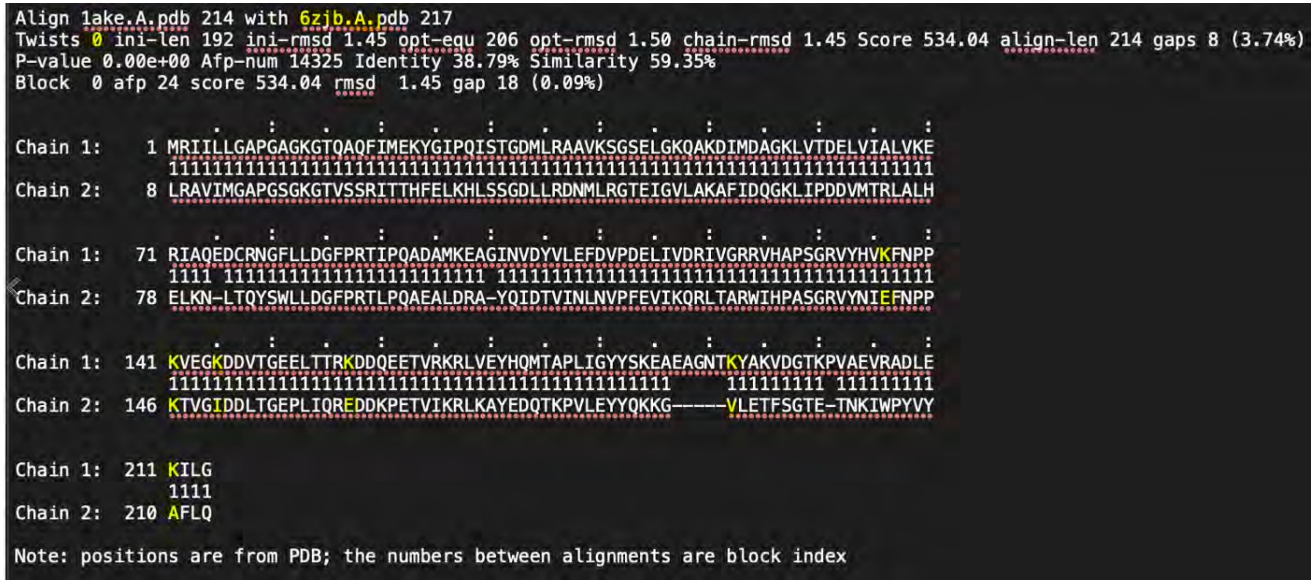

```
Align 1ake.A.pdb 214 with 6zjb.A.pdb 217
Twists 0 ini-len 192 ini-rmsd 1.45 opt-equ 206 opt-rmsd 1.50 chain-rmsd 1.45 Score 534.04 align-len 214 gaps 8 (3.74%)
P-value 0.00e+00 Afp-num 14325 Identity 38.79% Similarity 59.35%
Block  0 afp 24 score 534.04 rmsd  1.45 gap 18 (0.09%)

                 .    :    .    :    .    :    .    :    .    :    .    :    .    :
Chain 1:    1 MRIILLGAPGAGKGTQAQFIMEKYGIPQISTGDMLRAAVKSGSELGKQAKDIMDAGKLVTDELVIALVKE
              1111111111111111111111111111111111111111111111111111111111111111111111
Chain 2:    8 LRAVIMGAPGSGKGTVSSRITTHFELKHLSSGDLLRDNMLRGTEIGVLAKAFIDQGKLIPDDVMTRLALH

                 .    :    .    :    .    :    .    :    .    :    .    :    .    :
Chain 1:   71 RIAQEDCRNGFLLDGFPRTIPQADAMKEAGINVDYVLEFDVPDELIVDRIVGRRVHAPSGRVYHVKFNPP
              1111 1111111111111111111111111 1111111111111111111111111111111111111111
Chain 2:   78 ELKN-LTQYSWLLDGFPRTLPQAEALDRA-YQIDTVINLNVPFEVIKQRLTARWIHPASGRVYNIEFNPP

                 .    :    .    :    .    :    .    :    .    :    .    :    .    :
Chain 1:  141 KVEGKDDVTGEELTTRKDDQEETVRKRLVEYHQMTAPLIGYYSKEAEAGNTKYAKVDGTKPVAEVRADLE
              1111111111111111111111111111111111111111111111111111     111111111 111111111
Chain 2:  146 KTVGIDDLTGEPLIQREDDKPETVIKRLKAYEDQTKPVLEYYQKKG-----VLETFSGTE-TNKIWPYVY

Chain 1:  211 KILG
              1111
Chain 2:  210 AFLQ

Note: positions are from PDB; the numbers between alignments are block index
```

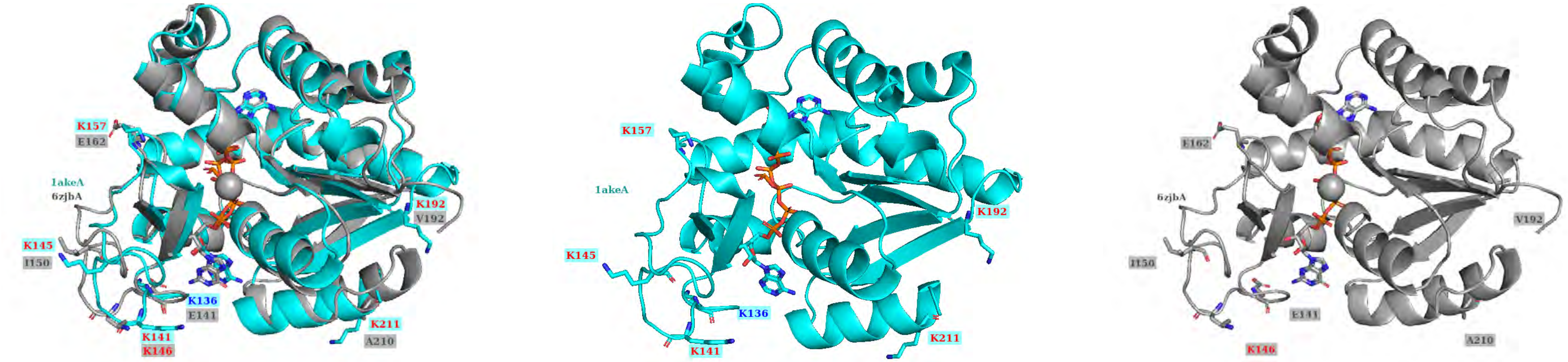

UniProt ID: Q9UIJ7

PDB ID: 6ZJD_A

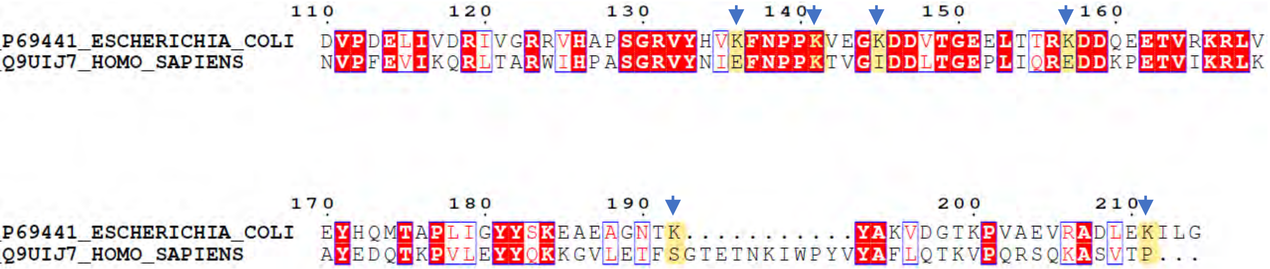


Full sequences in supplemental file.

```
Align 1ake.A.pdb 214 with 6zjd.A.pdb 214
Twists 2 ini-len 192 ini-rmsd 1.90 opt-equ 206 opt-rmsd 1.74 chain-rmsd 7.39 Score 490.37 align-len 214 gaps 8 (3.74%)
P-value 5.91e-14 Afp-num 14009 Identity 38.79% Similarity 58.88%
Block  0 afp 14 score 286.36 rmsd  2.02 gap 5 (0.04%)
Block  1 afp  5 score 113.86 rmsd  0.99 gap 0 (0.00%)
Block  2 afp  5 score 110.94 rmsd  1.47 gap 7 (0.15%)

                .    :    .    :    .    :    .    :    .    :    .    :    .    :
Chain 1:    1 MRIILLGAPGAGKGTQAQFIMEKYGIPQISTGDMLRAAVKSGSELGKQAKDIMDAGKLVTDELVIALVKE
              1111111111111111111111111111111111111111111111111111111111111111111111
Chain 2:    8 LRAVIMGAPGSGKGTVSSRITTHFELKHLSSGDLLRDNMLRGTEIGVLAKAFIDQGKLIPDDVMTRLALH

                .    :    .    :    .    :    .    :    .    :    .    :    .    :
Chain 1:   71 RIAQEDCRNGFLLDGFPRTIPQADAMKEAGINVDYVLEFDVPDELIVDRIVGRRVHAPSGRVYHVKFNPP
              1111 111111111111111111111111 1111111111111111111122222222222222222222
Chain 2:   78 ELKN-LTQYSWLLDGFPRTLPQAEALDRA-YQIDTVINLNVPFEVIKQRLTARWIHPASGRVYNIEFNPP

                .    :    .    :    .    :    .    :    .    :    .    :    .    :
Chain 1:  141 KVEGKDDVTGEELTTRKDDQEETVRKRLVEYHQMTAPLIGYYSKEAEAGNTKYAKVDGTKPVAEVRADLE
              22222222222222222223333333333333333333333333333     33333333 3333333333
Chain 2:  146 KTVGIDDLTGEPLIQREDDKPETVIKRLKAYEDQTKPVLEYYQKKG-----VLETFSGT-ETNKIWPYVY

Chain 1:  211 KILG
              3333
Chain 2:  210 AFLQ

Note: positions are from PDB; the numbers between alignments are block index
```

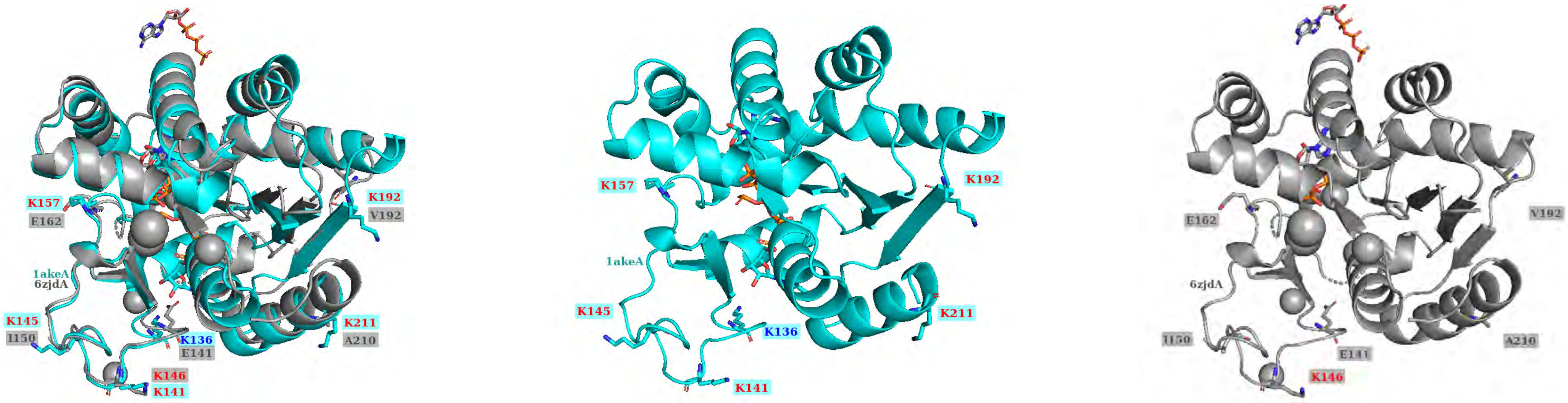

UniProt ID: Q9UIJ7

PDB ID: 6ZJE_A

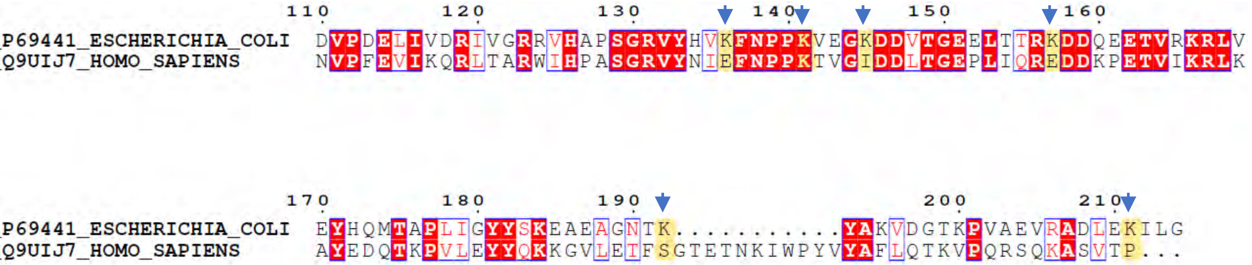


Full sequences in supplemental file.

```
Align 1ake.A.pdb 214 with 6zje.A.pdb 214
Twists 2 ini-len 192 ini-rmsd 3.73 opt-equ 204 opt-rmsd 1.92 chain-rmsd 7.49 Score 493.23 align-len 214 gaps 10 (4.67%)
P-value 2.62e-13 Afp-num 13934 Identity 38.79% Similarity 58.88%
Block  0 afp 14 score 291.55 rmsd  1.96 gap 6 (0.05%)
Block  1 afp  4 score 95.42 rmsd  0.58 gap 0 (0.00%)
Block  2 afp  6 score 126.03 rmsd  3.49 gap 9 (0.16%)

                .    :    .    :    .    :    .    :    .    :    .    :    .    :
Chain 1:    1 MRIILLGAPGAGKGTQAQFIMEKYGIPQISTGDMLRAAVKSGSELGKQAKDIMDAGKLVTDELVIALVKE
              111111111111111111111111111111111111111111111111111111111111111111111111
Chain 2:    8 LRAVIMGAPGSGKGTVSSRITTHFELKHLSSGDLLRDNMLRGTEIGVLAKAFIDQGKLIPDDVMTRLALH

                .    :    .    :    .    :    .    :    .    :    .    :    .    :
Chain 1:   71 RIAQEDCRNGFLLDGFPRTIPQADAMKEAGINVDYVLEFDVPDELIVDRIVGRRVHAPSGRVYHVKFNPP
              1111 11111111111111111111111 1111111111111111111122222222222222222222
Chain 2:   78 ELKN-LTQYSWLLDGFPRTLPQAEALDRA-YQIDTVINLNVPFEVIKQRLTARWIHPASGRVYNIEFNPP

                .    :    .    :    .    :    .    :    .    :    .    :    .    :
Chain 1:  141 KVEGKDDVTGEELTTRKDDQEETVRKRLVEYHQMTAPLIGYYSKEAEAGNTKYAKVDGTKPVAEVRADLE
              222222222222   33333333333333333333333333333333      333333333 3333333333
Chain 2:  146 KTVGIDDLTGEPLIQREDDKPETVIKRLKAYEDQTKPVLEYYQKKG-----VLETFSGT-ETNKIWPYVY

Chain 1:  211 KILG
              3333
Chain 2:  210 AFLQ

Note: positions are from PDB; the numbers between alignments are block index
```

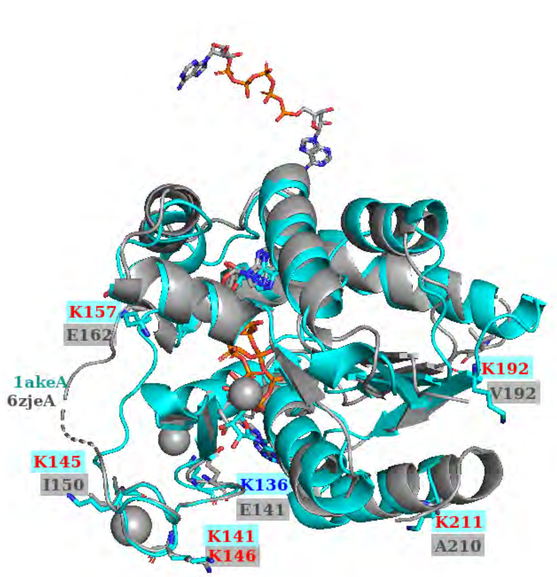


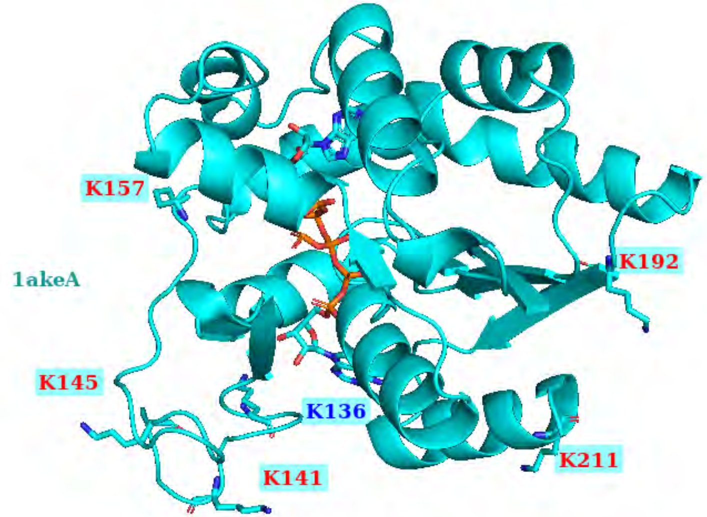


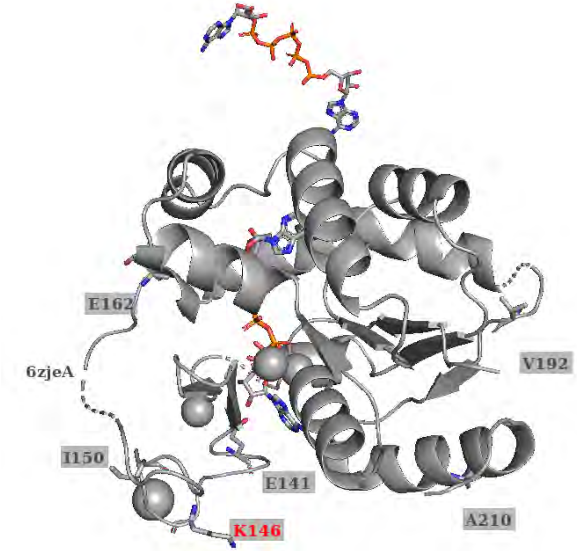

UniProt ID: Q9Y6K8

PDB ID: 2BWJ_A

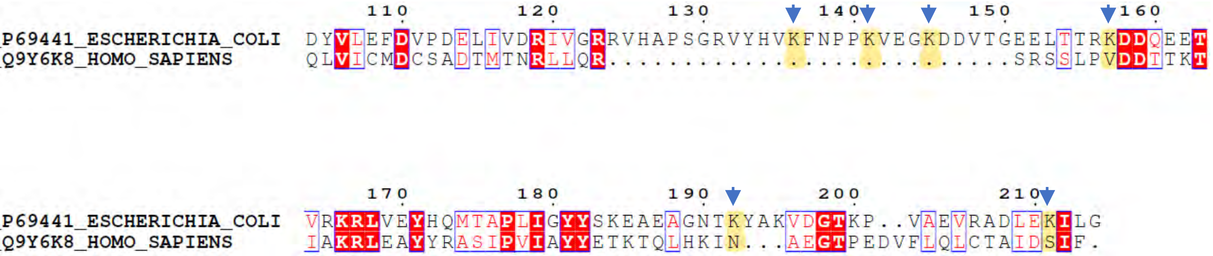


Full sequences in supplemental file.

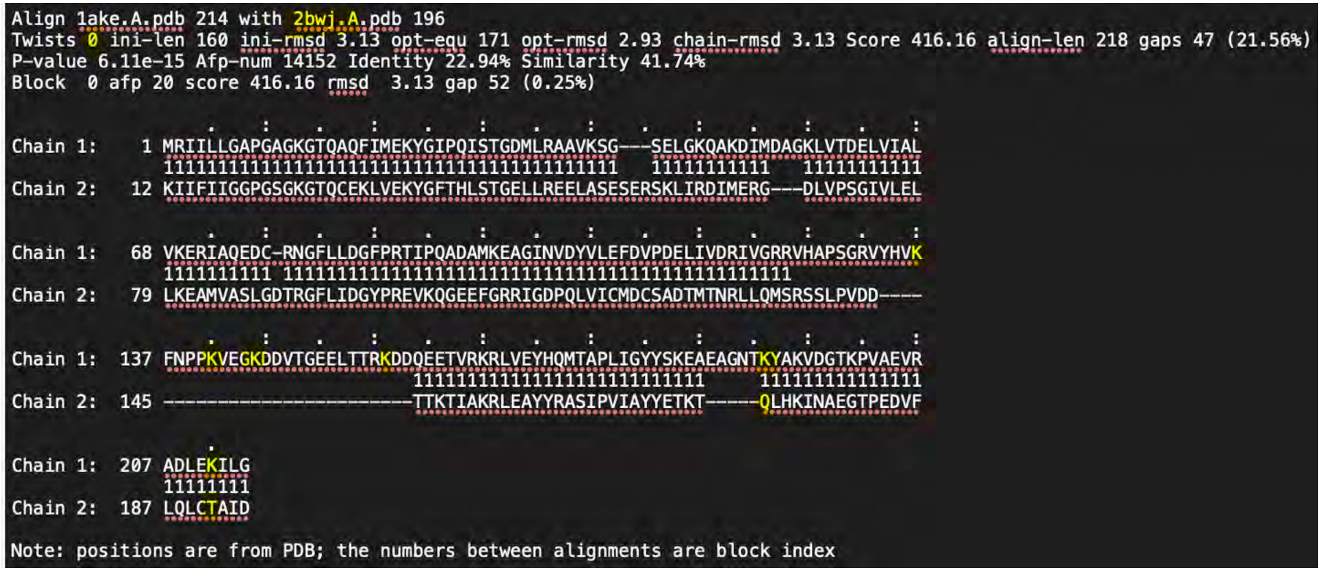

```
Align 1ake.A.pdb 214 with 2bwj.A.pdb 196
Twists 0 ini-len 160 ini-rmsd 3.13 opt-equ 171 opt-rmsd 2.93 chain-rmsd 3.13 Score 416.16 align-len 218 gaps 47 (21.56%)
P-value 6.11e-15 Afp-num 14152 Identity 22.94% Similarity 41.74%
Block  0 afp 20 score 416.16 rmsd  3.13 gap 52 (0.25%)

                  .    :    .    :    .    :    .    :    .    :    .    :    .    :
Chain 1:    1 MRIILLGAPGAGKGTQAQFIMEKYGIPQISTGDMLRAAVKSG---SELGKQAKDIMDAGKLVTDELVIAL
              111111111111111111111111111111111111111111   11111111111   1111111111
Chain 2:   12 KIIFIIGGPGSGKGTQCEKLVEKYGFTHLSTGELLREELASESERSKLIRDIMERG---DLVPSGIVLEL

                  .    :    .    :    .    :    .    :    .    :    .    :    .    :
Chain 1:   68 VKERIAQEDC-RNGFLLDGFPRTIPQADAMKEAGINVDYVLEFDVPDELIVDRIVGRRVHAPSGRVYHVK
              1111111111 11111111111111111111111111111111111111111111111
Chain 2:   79 LKEAMVASLGDTRGFLIDGYPREVKQGEEFGRRIGDPQLVICMDCSADTMTNRLLQMSRSSLPVDD----

                  .    :    .    :    .    :    .    :    .    :    .    :    .    :
Chain 1:  137 FNPPKVEGKDDVTGEELTTRKDDQEETVRKRLVEYHQMTAPLIGYYSKEAEAGNTKYAKVDGTKPVAEVR
                                     1111111111111111111111111111     111111111111111
Chain 2:  145 ----------------------TTKTIAKRLEAYYRASIPVIAYYETKT-----QLHKINAEGTPEDVF

                  .
Chain 1:  207 ADLEKILG
              11111111
Chain 2:  187 LQLCTAID

Note: positions are from PDB; the numbers between alignments are block index
```

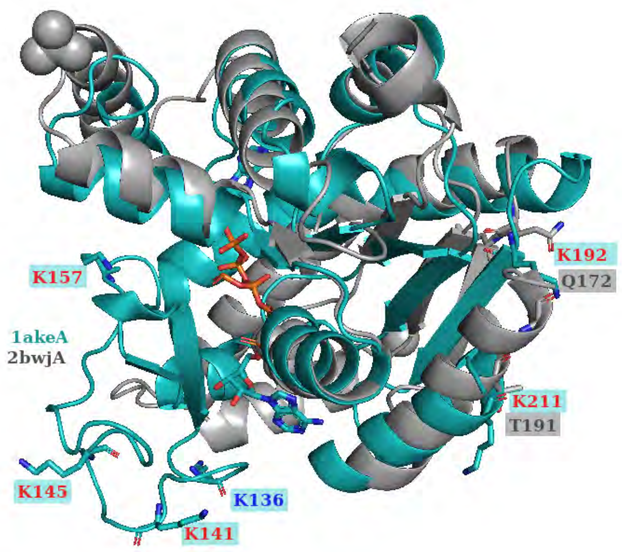


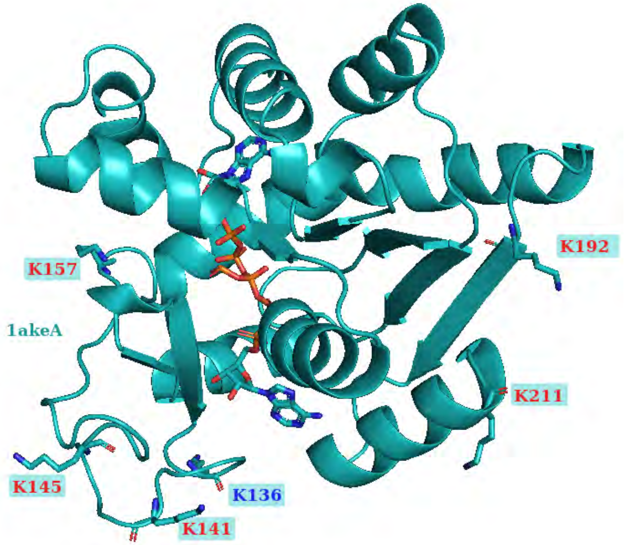


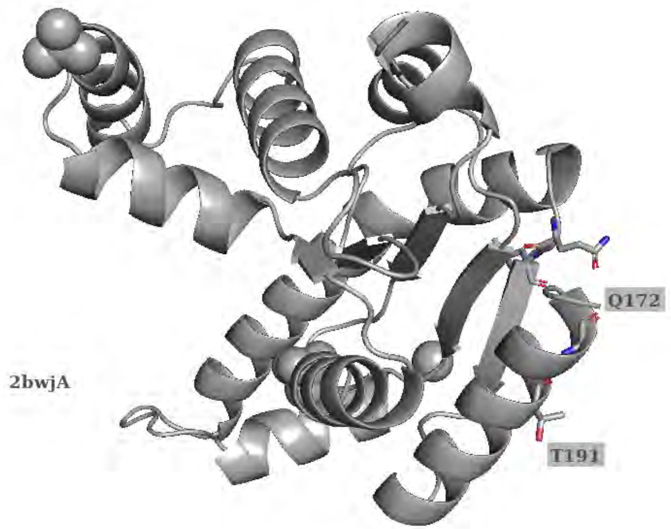

UniProt ID: synthetic construct

PDB ID: 5G3Y_A

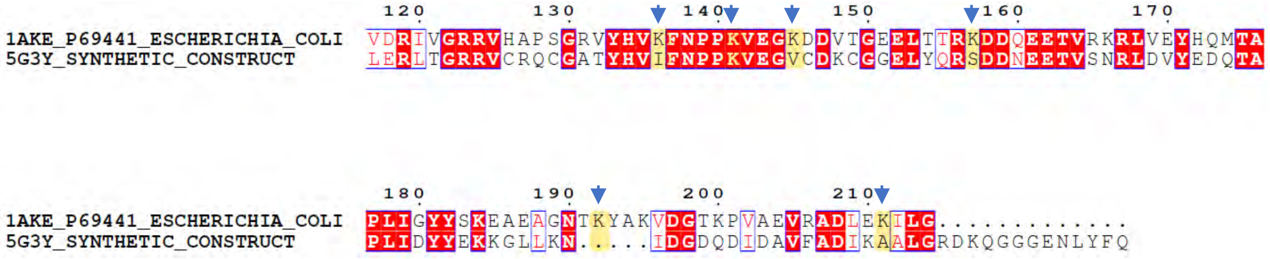


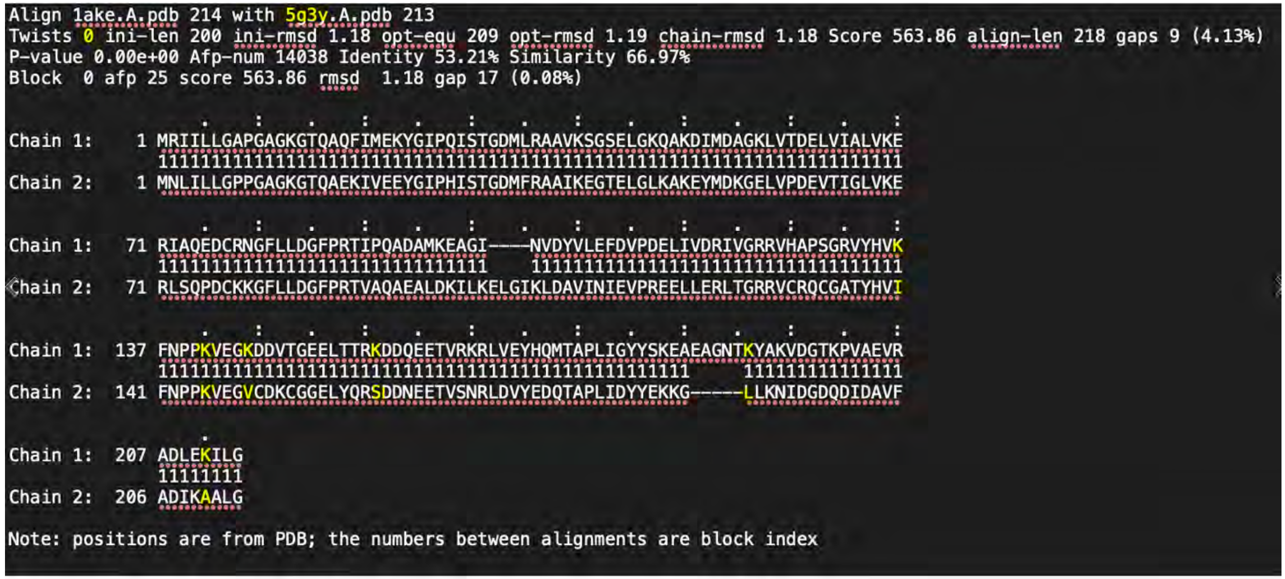


Full sequences in supplemental file.

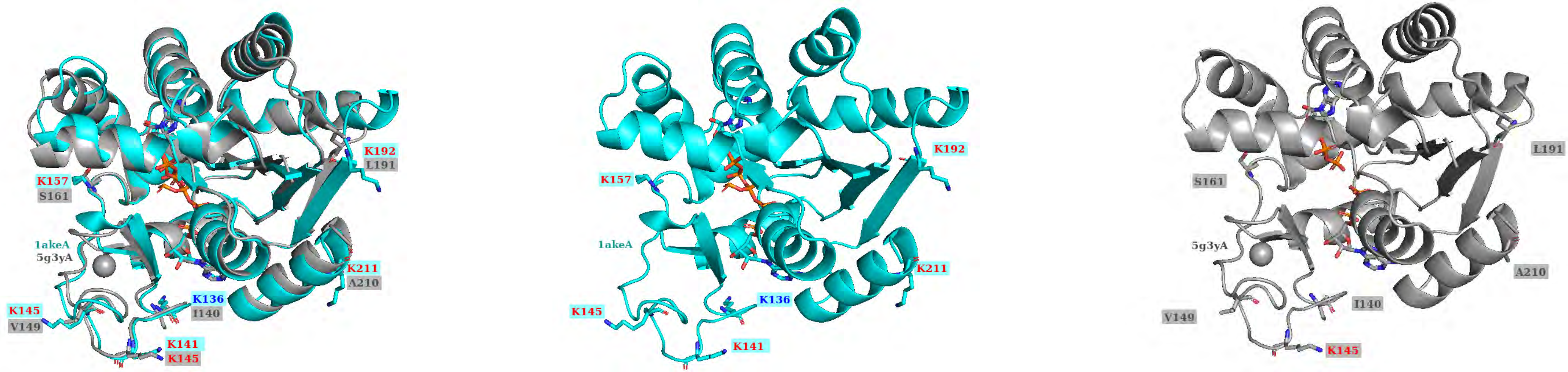

UniProt ID: synthetic construct

PDB ID: 5G3Z_A

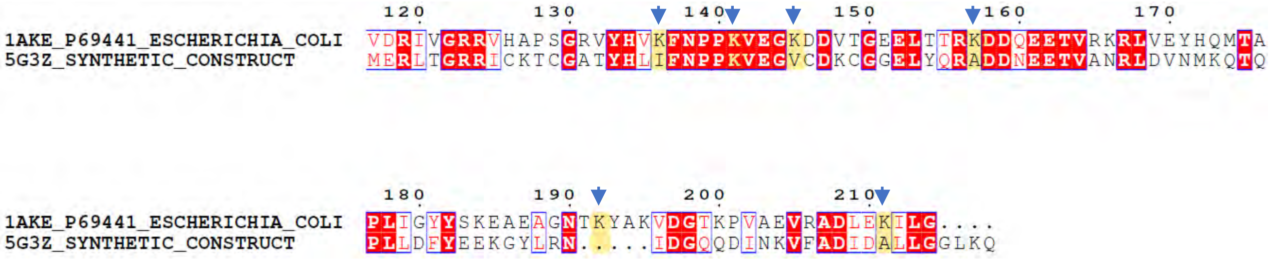


Full sequences in supplemental file.

```
Align 1ake.A.pdb 214 with 5g3z.A.pdb 215
Twists 0 ini-len 200 ini-rmsd 1.49 opt-equ 209 opt-rmsd 1.49 chain-rmsd 1.49 Score 553.79 align-len 218 gaps 9 (4.13%)
P-value 0.00e+00 Afp-num 14221 Identity 48.62% Similarity 67.43%
Block  0 afp 25 score 553.79 rmsd  1.49 gap 15 (0.07%)

                .    :    .    :    .    :    .    :    .    :    .    :    .    :
Chain 1:     1 MRIILLGAPGAGKGTQAQFIMEKYGIPQISTGDMLRAAVKSGSELGKQAKDIMDAGKLVTDELVIALVKE
               1111111111111111111111111111111111111111111111111111111111111111111111
Chain 2:     1 MNLVLMGLPGAGKGTQAEKIVEKYGIPHISTGDMFRAAIKEGTELGLKAKSFMDKGELVPDEVTIGIVRE

                .    :    .    :    .    :    .    :    .    :    .    :    .    :
Chain 1:    71 RIAQEDCRNGFLLDGFPRTIPQADAMKEAG----INVDYVLEFDVPDELIVDRIVGRRVHAPSGRVYHVK
               111111111111111111111111111111    111111111111111111111111111111111111
Chain 2:    71 RLSKDDCKKGFLLDGFPRTVAQAEALDNILKELGKKLDYVINIEVPKEELMERLTGRRICKTCGATYHLI

                .    :    .    :    .    :    .    :    .    :    .    :    .    :
Chain 1:   137 FNPPKVEGKDDVTGEELTTRKDDQEETVRKRLVEYHQMTAPLIGYYSKEAEAGNTKYAKVDGTKPVAEVR
               1111111111111111111111111111111111111111111111111     111111111111111
Chain 2:   141 FNPPKVEGVCDKCGGELYQRADDNEETVANRLDVNMKQTQPLLDFYEEKG-----YLRNIDGQQDINKVF

                .
Chain 1:   207 ADLEKILG
               11111111
Chain 2:   206 ADIDALLG

Note: positions are from PDB; the numbers between alignments are block index
```

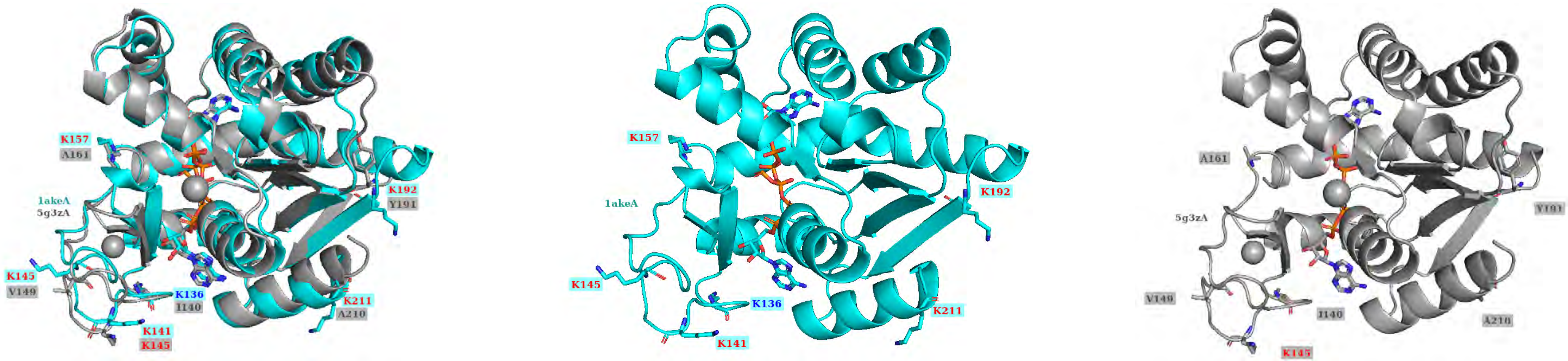

UniProt ID: synthetic construct

PDB ID: 5G40_A

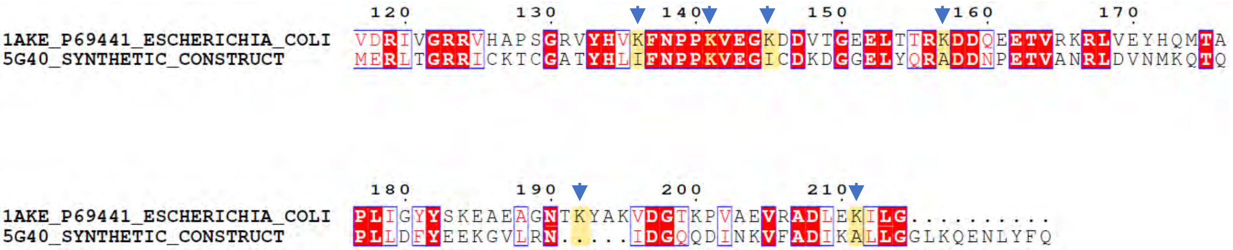


Full sequences in supplemental file.

```
Align 1ake.A.pdb 214 with 5g40.A.pdb 215
Twists 0 ini-len 200 ini-rmsd 1.05 opt-equ 209 opt-rmsd 1.09 chain-rmsd 1.05 Score 555.45 align-len 218 gaps 9 (4.13%)
P-value 0.00e+00 Afp-num 14184 Identity 48.17% Similarity 66.97%
Block  0 afp 25 score 555.45 rmsd  1.05 gap 17 (0.08%)

                   .    :    .    :    .    :    .    :    .    :    .    :    .    :
Chain 1:    1 MRIILLGAPGAGKGTQAQFIMEKYGIPQISTGDMLRAAVKSGSELGKQAKDIMDAGKLVTDELVIALVKE
              1111111111111111111111111111111111111111111111111111111111111111111111
Chain 2:    1 MNLVLMGLPGAGKGTQAEKIVEKYGIPHISTGDMFRAAIKEGTELGLKAKSFMDKGELVPDEVTIGIVRE

                   .    :    .    :    .    :    .    :    .    :    .    :    .    :
Chain 1:   71 RIAQEDCRNGFLLDGFPRTIPQADAMKEAGI----NVDYVLEFDVPDELIVDRIVGRRVHAPSGRVYHVK
              1111111111111111111111111111111    11111111111111111111111111111111111
Chain 2:   71 RLSKDDCKKGFLLDGFPRTVAQAEALDNILSELGKKLDYVINIEVPKEELMERLTGRRICKTCGATYHLI

                   .    :    .    :    .    :    .    :    .    :    .    :    .    :
Chain 1:  137 FNPPKVEGKDDVTGEELTTRKDDQEETVRKRLVEYHQMTAPLIGYYSKEAEAGNTKYAKVDGTKPVAEVR
              11111111111111111111111111111111111111111111111111     111111111111111
Chain 2:  141 FNPPKVEGICDKDGGELYQRADDNPETVANRLDVNMKQTQPLLDFYEEKG-----VLRNIDGQQDINKVF

                   .
Chain 1:  207 ADLEKILG
              11111111
Chain 2:  206 ADIKALLG

Note: positions are from PDB; the numbers between alignments are block index
```

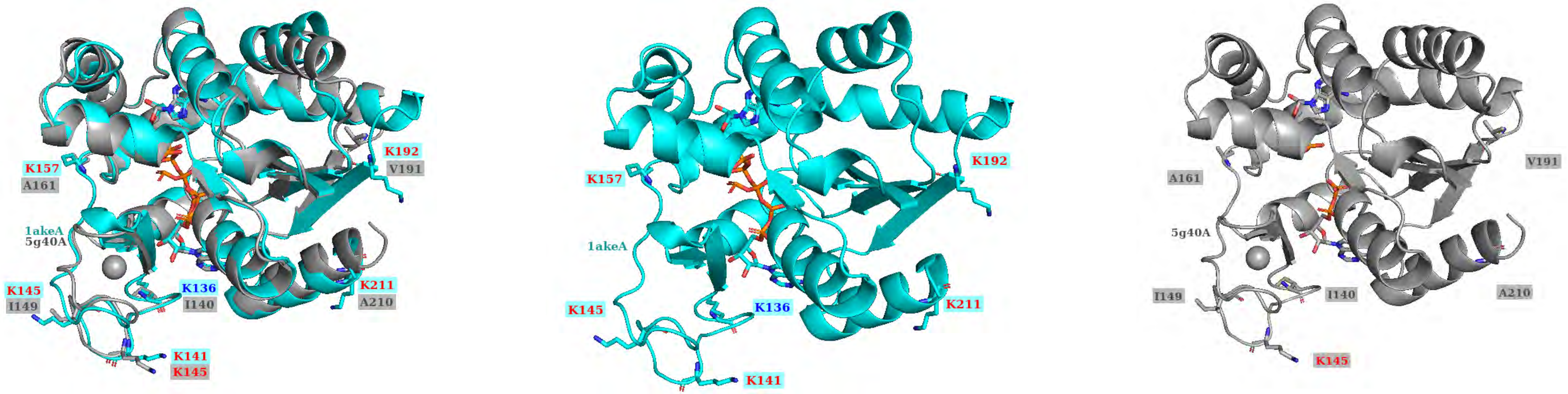

UniProt ID: synthetic construct

PDB ID: 5G41_A

```
                             120         130        140         150        160         170
1AKE_P69441_ESCHERICHIA_COLI VDRIVGRRVHAPSGRVYHVKFNPPKVEGKDDVTGEELTTRKDDQEETVRKRLVEYHQMTA
5G41_SYNTHETIC_CONSTRUCT     MERLTGRRICKTCGATYHLIFNPPKVEGICDKDGGELYQRADDNPETVANRLDVNMKQTQ

                             180         190        200        210
1AKE_P69441_ESCHERICHIA_COLI PLIGYYSKEAEAGNTKYAKVDGTKPVAEVRADLEKILG..........
5G41_SYNTHETIC_CONSTRUCT     PLLDFYEEKGVLRN.....IDGQQDINKVFADIKALLGGLKQENLYFQ
```

Full sequences in supplemental file.

```
Align 1ake.A.pdb 214 with 5g41.A.pdb 214
Twists 0 ini-len 200 ini-rmsd 1.99 opt-equ 209 opt-rmsd 1.96 chain-rmsd 1.99 Score 555.70 align-len 218 gaps 9 (4.13%)
P-value 0.00e+00 Afp-num 13922 Identity 48.17% Similarity 66.97%
Block  0 afp 25 score 555.70 rmsd  1.99 gap 17 (0.08%)

                .    :    .    :    .    :    .    :    .    :    .    :    .    :
Chain 1:    1 MRIILLGAPGAGKGTQAQFIMEKYGIPQISTGDMLRAAVKSGSELGKQAKDIMDAGKLVTDELVIALVKE
              1111111111111111111111111111111111111111111111111111111111111111111111
Chain 2:    1 MNLVLMGLPGAGKGTQAEKIVEKYGIPHISTGDMFRAAIKEGTELGLKAKSFMDKGELVPDEVTIGIVRE

                .    :    .    :    .    :    .    :    .    :    .    :    .    :
Chain 1:   71 RIAQEDCRNGFLLDGFPRTIPQADAMKEAG----INVDYVLEFDVPDELIVDRIVGRRVHAPSGRVYHVK
              111111111111111111111111111111    111111111111111111111111111111111111
Chain 2:   71 RLSKDDCKKGFLLDGFPRTVAQAEALDNILSELGKKLDYVINIEVPKEELMERLTGRRICKTCGATYHLI

                .    :    .    :    .    :    .    :    .    :    .    :    .    :
Chain 1:  137 FNPPKVEGKDDVTGEELTTRKDDQEETVRKRLVEYHQMTAPLIGYYSKEAEAGNTKYAKVDGTKPVAEVR
              111111111111111111111111111111111111111111111111111      111111111111111
Chain 2:  141 FNPPKVEGICDKDGGELYQRADDNPETVANRLDVNMKQTQPLLDFYEEKG-----VLRNIDGQQDINKVF

                .
Chain 1:  207 ADLEKILG
              11111111
Chain 2:  206 ADIKALLG
```

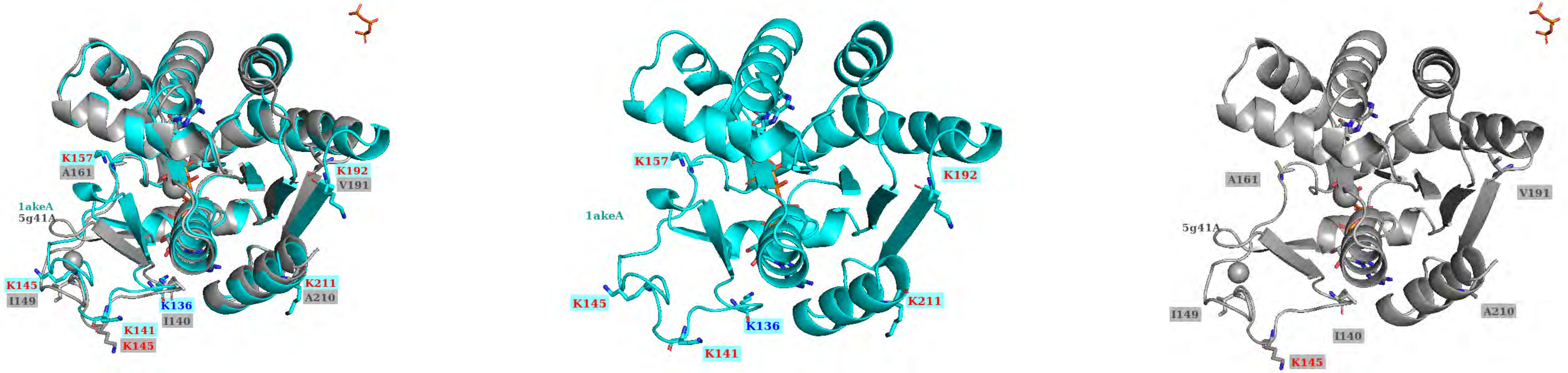

Supplement: Supplementary file 2 [file Data_Sheet_2.PDF]
