# Supplementary material for: Investigation of the Importance of Protein 3D Structure for Assessing Conservation of Lysine Acetylation Sites in Protein Homologs
Source: Front Microbiol. 2022 Jan 31;12:805181. doi: 10.3389/fmicb.2021.805181 (PMC8843374; doi:10.3389/fmicb.2021.805181)

Supplemental Figure SF2B. Compiled pairwise sequence (Cobalt) and structural (FATCAT) alignments between the *E. coli* substrate protein target (Icd-isocitrate dehydrogenase; PDB ID: 1ai2) and homologs sorted by UniProt ID. Lysine residues previously identified as acetylated in the target protein are highlighted in yellow in the sequence alignments and FATCAT structural alignment xml files to examine conservation. 3D protein structures are shown as ribbon representations with the target protein in cyan and the homolog protein in gray. Blue lysine residues correspond to KAT (lysine acetyltransferase) acetylation sites, red lysine residues correspond to AcP (acetyl phosphate) acetylation sites, and purple lysine residues correspond to sites acetylated by both KAT and AcP. 1D sequence alignments are not shown between *E. coli* proteins because sequences were identical.

UniProt ID: O29610

PDB ID: 2IV0_B

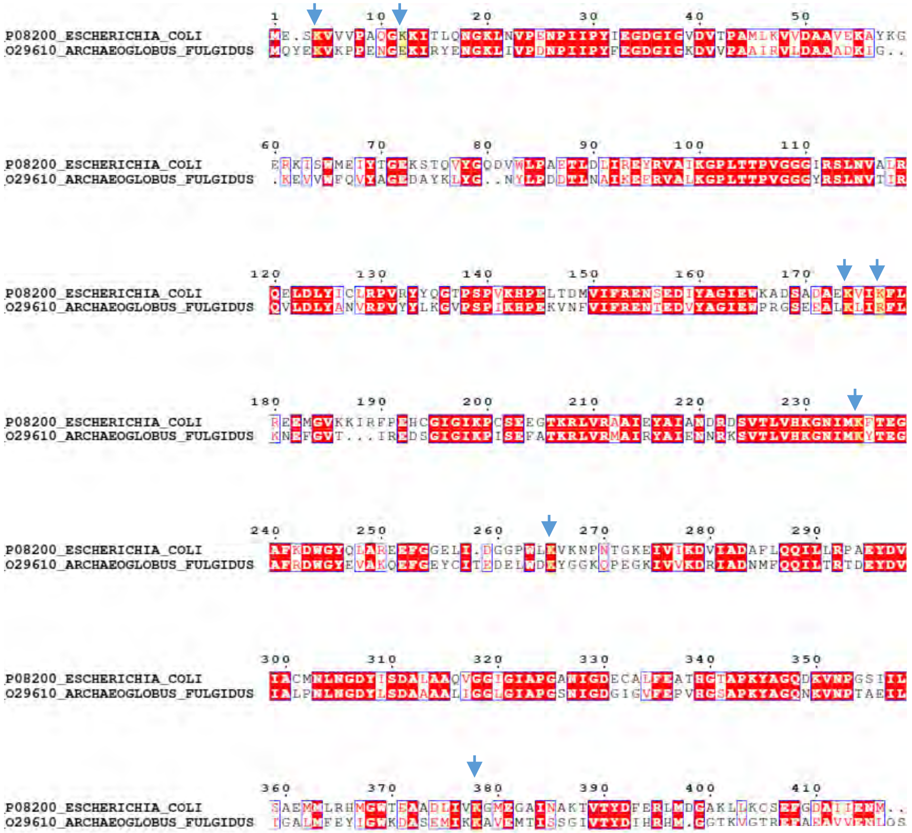


```
Align 1ai2.A.pdb 414 with 2iv0.B.pdb 412
Twists 0 ini-len 368 ini-rmsd 1.86 opt-equ 404 opt-rmsd 2.62 chain-rmsd 1.86 Score 1023.68 align-len 416 gaps 12 (2.88%)
P-value 0.00e+00 Afp-num 51049 Identity 55.29% Similarity 70.91%
Block  0 afp 46 score 1023.68 rmsd  1.86 gap 46 (0.11%)

Chain 1:    3 SKVVVPAQGKKITLQNGKLNVPENPIIPYIEGDGIGVDVTPAMLKVVDAAVEKAYKGERKISWMEIYTGE
              1111111111111111111111111111111111111111111111111111   11111111111
Chain 2:    4 EKVKPPENGEKIRYENGKLIVPDNPIIPYFEGDGIGKDVVPAAIRVLDAAADKIG---KEVVWFQVYAGE

Chain 1:   73 KSTQVYGQDVWLPAETLDLIREYRVAIKGPLTTPVGGGIRSLNVALRQELDLYICLRPVRYYQGTPSPVK
              1111111  1111111111111111111111111111111111111111111111111111111111111
Chain 2:   71 DAYKLYG--NYLPDDTLNAIKEFRVALKGPLTTPVGGGYRSLNVTIRQVLDLYANVRPVYYLKGVPSPIK

Chain 1:  143 HPELTDMVIFRENSEDIYAGIEWKADSADAEKVIKFLREEMGVKKIRFPEHCGIGIKPCSEEGTKRLVRA
              111111111111111111111111111111111111111111111   1111111111111111111111
Chain 2:  139 HPEKVNFVIFRENTEDVYAGIEWPRGSEEALKLIRFLKNEFGVTI---REDSGIGIKPISEFATKRLVRM

Chain 1:  213 AIEYAIANDRDSVTLVHKGNIMKFTEGAFKDWGYQLAREEFG--GELIDGGPWLKVKNPNTGKEIVIKDV
              111111111111111111111111111111111111111111  1111111111111111 1111111111
Chain 2:  206 AIRYAIENNRKSVTLVHKGNIMKYTEGAFRDWGYEVAKQEFGEYCITEDELWDKYGGKQ-PEGKIVVKDR

Chain 1:  281 IADAFLQQILLRPAEYDVIACMNLNGDYISDALAAQVGGIGIAPGANIGDECALFEATHGTAPKYAGQDK
              1111111111111111111111111111111111111111111111111111111111111111111111
Chain 2:  275 IADNMFQQILTRTDEYDVIALPNLNGDYLSDAAAALIGGLGIAPGSNIGDGIGVFEPVHGSAPKYAGQNK

Chain 1:  351 VNPGSIILSAEMMLRHMGWTEAADLIVKGMEGAINAKTVTYDFERLMDGAKLLKCSEFGDAIIENM
              11111111111111111111111111111111111111111111111111 1111111111111111
Chain 2:  345 VNPTAEILTGALMFEYIGWKDASEMIKKAVEMTISSGIVTYDIHRHMGG-TKVGTREFAEAVVENL
```

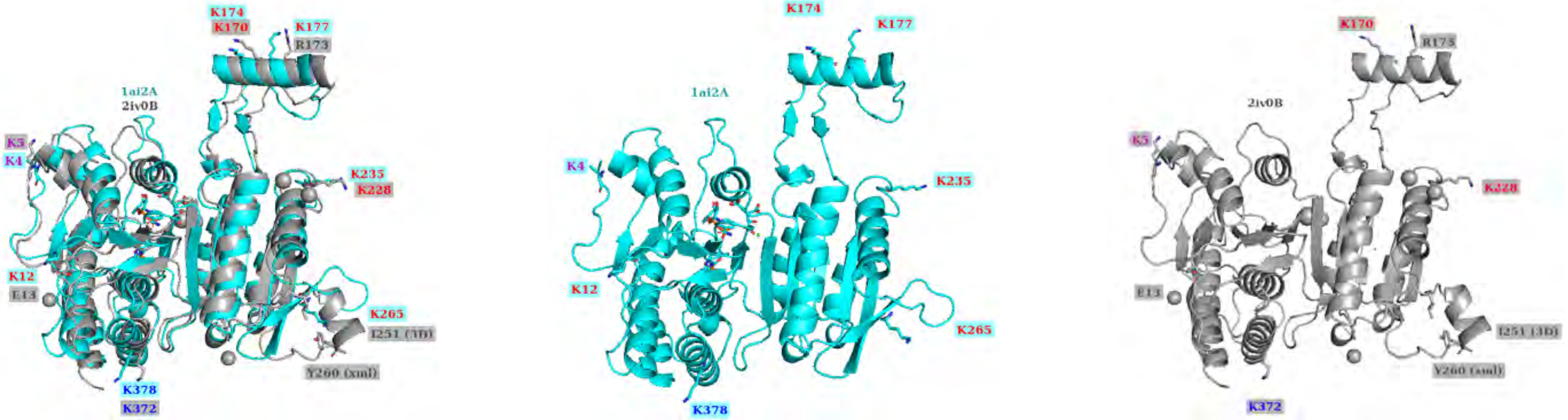

UniProt ID: P08200

PDB ID: 1AI3_A

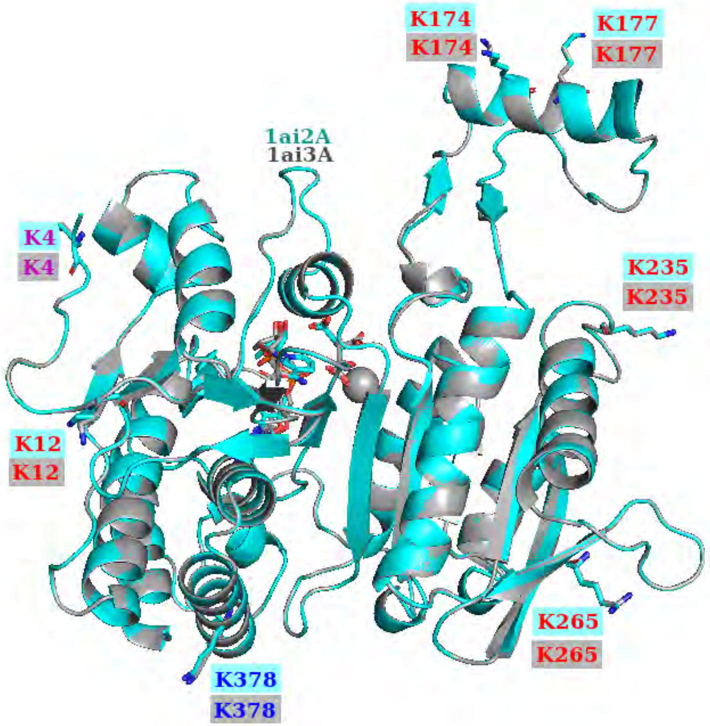


```
Align 1ai2.A.pdb 414 with 1ai3.A.pdb 414
Twists 0 ini-len 408 ini-rmsd 0.11 opt-equ 414 opt-rmsd 0.11 chain-rmsd 0.11 Score 1223.58 align-len 414 gaps 0 (0.00%)
P-value 0.00e+00 Afp-num 51765 Identity 100.00% Similarity 100.00%
Block  0 afp 51 score 1223.58 rmsd  0.11 gap 0 (0.00%)

                       .         :         .         :         .         :         .         :         .         :         .         :         .         :
Chain 1:    3 SKVVVPAQGKKITLQNGKLNVPENPIIPYIEGDGIGVDVTPAMLKVVDAAVEKAYKGERKISWMEIYTGE
              1111111111111111111111111111111111111111111111111111111111111111111111
Chain 2:    3 SKVVVPAQGKKITLQNGKLNVPENPIIPYIEGDGIGVDVTPAMLKVVDAAVEKAYKGERKISWMEIYTGE

                       .         :         .         :         .         :         .         :         .         :         .         :         .         :
Chain 1:   73 KSTQVYGQDVWLPAETLDLIREYRVAIKGPLTTPVGGGIRSLNVALRQELDLYICLRPVRYYQGTPSPVK
              1111111111111111111111111111111111111111111111111111111111111111111111
Chain 2:   73 KSTQVYGQDVWLPAETLDLIREYRVAIKGPLTTPVGGGIRSLNVALRQELDLYICLRPVRYYQGTPSPVK

                       .         :         .         :         .         :         .         :         .         :         .         :         .         :
Chain 1:  143 HPELTDMVIFRENSEDIYAGIEWKADSADAEKVIKFLREEMGVKKIRFPEHCGIGIKPCSEEGTKRLVRA
              1111111111111111111111111111111111111111111111111111111111111111111111
Chain 2:  143 HPELTDMVIFRENSEDIYAGIEWKADSADAEKVIKFLREEMGVKKIRFPEHCGIGIKPCSEEGTKRLVRA

                       .         :         .         :         .         :         .         :         .         :         .         :         .         :
Chain 1:  213 AIEYAIANDRDSVTLVHKGNIMKFTEGAFKDWGYQLAREEFGGELIDGGPWLKVKNPNTGKEIVIKDVIA
              1111111111111111111111111111111111111111111111111111111111111111111111
Chain 2:  213 AIEYAIANDRDSVTLVHKGNIMKFTEGAFKDWGYQLAREEFGGELIDGGPWLKVKNPNTGKEIVIKDVIA

                       .         :         .         :         .         :         .         :         .         :         .         :         .         :
Chain 1:  283 DAFLQQILLRPAEYDVIACMNLNGDYISDALAAQVGGIGIAPGANIGDECALFEATHGTAPKYAGQDKVN
              1111111111111111111111111111111111111111111111111111111111111111111111
Chain 2:  283 DAFLQQILLRPAEYDVIACMNLNGDYISDALAAQVGGIGIAPGANIGDECALFEATHGTAPKYAGQDKVN

                       .         :         .         :         .         :         .         :         .         :         .         :
Chain 1:  353 PGSIILSAEMMLRHMGWTEAADLIVKGMEGAINAKTVTYDFERLMDGAKLLKCSEFGDAIIENM
              111111111111111111111111111111111111111111111111111111111111111
Chain 2:  353 PGSIILSAEMMLRHMGWTEAADLIVKGMEGAINAKTVTYDFERLMDGAKLLKCSEFGDAIIENM
```

UniProt ID: P08200

PDB ID: 1BL5_A

```
Align 1ai2.A.pdb 414 with 1bl5.A.pdb 414
Twists 0 ini-len 408 ini-rmsd 0.55 opt-equ 414 opt-rmsd 0.56 chain-rmsd 0.55 Score 1218.52 align-len 414 gaps 0 (0.00%)
P-value 0.00e+00 Afp-num 51903 Identity 100.00% Similarity 100.00%
Block  0 afp 51 score 1218.52 rmsd  0.55 gap 0 (0.00%)

Chain 1:    3 SKVVVPAQGKKITLQNGKLNVPENPIIPYIEGDGIGVDVTPAMLKVVDAAVEKAYKGERKISWMEIYTGE
              1111111111111111111111111111111111111111111111111111111111111111111111
Chain 2:    3 SKVVVPAQGKKITLQNGKLNVPENPIIPYIEGDGIGVDVTPAMLKVVDAAVEKAYKGERKISWMEIYTGE

Chain 1:   73 KSTQVYGQDVWLPAETLDLIREYRVAIKGPLTTPVGGGIRSLNVALRQELDLYICLRPVRYYQGTPSPVK
              1111111111111111111111111111111111111111111111111111111111111111111111
Chain 2:   73 KSTQVYGQDVWLPAETLDLIREYRVAIKGPLTTPVGGGIRSLNVALRQELDLYICLRPVRYYQGTPSPVK

Chain 1:  143 HPELTDMVIFRENSEDIYAGIEWKADSADAEKVIKFLREEMGVKKIRFPEHCGIGIKPCSEEGTKRLVRA
              1111111111111111111111111111111111111111111111111111111111111111111111
Chain 2:  143 HPELTDMVIFRENSEDIYAGIEWKADSADAEKVIKFLREEMGVKKIRFPEHCGIGIKPCSEEGTKRLVRA

Chain 1:  213 AIEYAIANDRDSVTLVHKGNIMKFTEGAFKDWGYQLAREEFGGELIDGGPWLKVKNPNTGKEIVIKDVIA
              1111111111111111111111111111111111111111111111111111111111111111111111
Chain 2:  213 AIEYAIANDRDSVTLVHKGNIMKFTEGAFKDWGYQLAREEFGGELIDGGPWLKVKNPNTGKEIVIKDVIA

Chain 1:  283 DAFLQQILLRPAEYDVIACMNLNGDYISDALAAQVGGIGIAPGANIGDECALFEATHGTAPKYAGQDKVN
              1111111111111111111111111111111111111111111111111111111111111111111111
Chain 2:  283 DAFLQQILLRPAEYDVIACMNLNGDYISDALAAQVGGIGIAPGANIGDECALFEATHGTAPKYAGQDKVN

Chain 1:  353 PGSIILSAEMMLRHMGWTEAADLIVKGMEGAINAKTVTYDFERLMDGAKLLKCSEFGDAIIENM
              1111111111111111111111111111111111111111111111111111111111111111
Chain 2:  353 PGSIILSAEMMLRHMGWTEAADLIVKGMEGAINAKTVTYDFERLMDGAKLLKCSEFGDAIIENM

Note: positions are from PDB; the numbers between alignments are block index
```

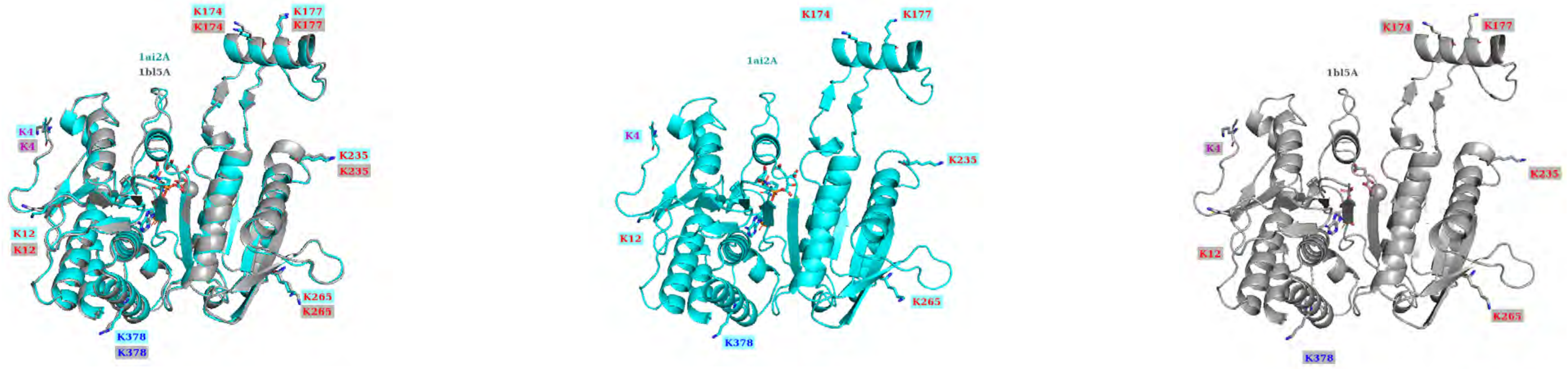

UniProt ID: P08200

PDB ID: 1CW1_A

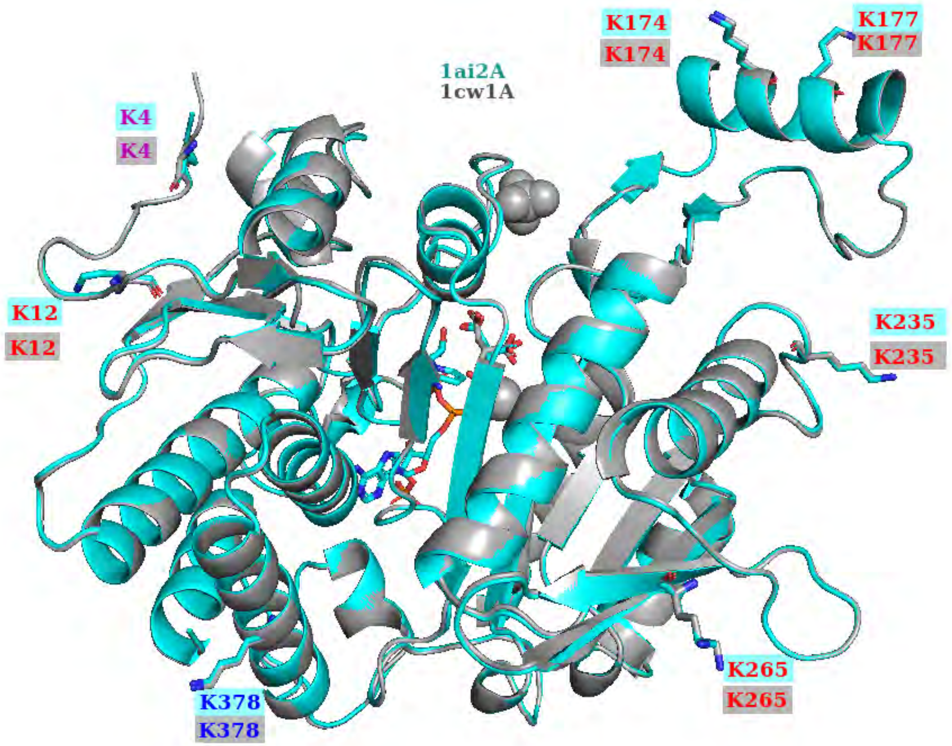


```
Align 1ai2.A.pdb 414 with 1cw1.A.pdb 415
Twists 0 ini-len 408 ini-rmsd 0.26 opt-equ 414 opt-rmsd 0.26 chain-rmsd 0.26 Score 1222.85 align-len 414 gaps 0 (0.00%)
P-value 0.00e+00 Afp-num 51816 Identity 99.76% Similarity 99.76%
Block  0 afp 51 score 1222.85 rmsd  0.26 gap 0 (0.00%)

                    .    :    .    :    .    :    .    :    .    :    .    :    .    :
Chain 1:    3 SKVVVPAQGKKITLQNGKLNVPENPIIPYIEGDGIGVDVTPAMLKVVDAAVEKAYKGERKISWMEIYTGE
              1111111111111111111111111111111111111111111111111111111111111111111111
Chain 2:    3 SKVVVPAQGKKITLQNGKLNVPENPIIPYIEGDGIGVDVTPAMLKVVDAAVEKAYKGERKISWMEIYTGE

                    .    :    .    :    .    :    .    :    .    :    .    :    .    :
Chain 1:   73 KSTQVYGQDVWLPAETLDLIREYRVAIKGPLTTPVGGGIRSLNVALRQELDLYICLRPVRYYQGTPSPVK
              1111111111111111111111111111111111111111111111111111111111111111111111
Chain 2:   73 KSTQVYGQDVWLPAETLDLIREYRVAIKGPLTTPVGGGIRSLNVALRQELDLYICLRPVRYYQGTPSPVK

                    .    :    .    :    .    :    .    :    .    :    .    :    .    :
Chain 1:  143 HPELTDMVIFRENSEDIYAGIEWKADSADAEKVIKFLREEMGVKKIRFPEHCGIGIKPCSEEGTKRLVRA
              1111111111111111111111111111111111111111111111111111111111111111111111
Chain 2:  143 HPELTDMVIFRENSEDIYAGIEWKADSADAEKVIKFLREEMGVKKIRFPEHCGIGIKPCSEEGTKRLVRA

                    .    :    .    :    .    :    .    :    .    :    .    :    .    :
Chain 1:  213 AIEYAIANDRDSVTLVHKGNIMKFTEGAFKDWGYQLAREEFGGELIDGGPWLKVKNPNTGKEIVIKDVIA
              1111111111111111111111111111111111111111111111111111111111111111111111
Chain 2:  213 AIEYAIANDRDSVTLVHMGNIMKFTEGAFKDWGYQLAREEFGGELIDGGPWLKVKNPNTGKEIVIKDVIA

                    .    :    .    :    .    :    .    :    .    :    .    :    .    :
Chain 1:  283 DAFLQQILLRPAEYDVIACMNLNGDYISDALAAQVGGIGIAPGANIGDECALFEATHGTAPKYAGQDKVN
              1111111111111111111111111111111111111111111111111111111111111111111111
Chain 2:  283 DAFLQQILLRPAEYDVIACMNLNGDYISDALAAQVGGIGIAPGANIGDECALFEATHGTAPKYAGQDKVN

                    .    :    .    :    .    :    .    :    .    :    .    :
Chain 1:  353 PGSIILSAEMMLRHMGWTEAADLIVKGMEGAINAKTVTYDFERLMDGAKLLKCSEFGDAIIENM
              1111111111111111111111111111111111111111111111111111111111111111
Chain 2:  353 PGSIILSAEMMLRHMGWTEAADLIVKGMEGAINAKTVTYDFERLMDGAKLLKCSEFGDAIIENM

Note: positions are from PDB; the numbers between alignments are block index
```

UniProt ID: P08200
PDB ID: 1CW4_A

```
Align 1ai2.A.pdb 414 with 1cw4.A.pdb 415
Twists 0 ini-len 408 ini-rmsd 0.26 opt-equ 414 opt-rmsd 0.26 chain-rmsd 0.26 Score 1222.95 align-len 414 gaps 0 (0.00%)
P-value 0.00e+00 Afp-num 51779 Identity 99.76% Similarity 99.76%
Block  0 afp 51 score 1222.95 rmsd  0.26 gap 0 (0.00%)

                    .    :    .    :    .    :    .    :    .    :    .    :    .    :
Chain 1:    3 SKVVVPAQGKKITLQNGKLNVPENPIIPYIEGDGIGVDVTPAMLKVVDAAVEKAYKGERKISWMEIYTGE
              1111111111111111111111111111111111111111111111111111111111111111111111
Chain 2:    3 SKVVVPAQGKKITLQNGKLNVPENPIIPYIEGDGIGVDVTPAMLKVVDAAVEKAYKGERKISWMEIYTGE

                    .    :    .    :    .    :    .    :    .    :    .    :    .    :
Chain 1:   73 KSTQVYGQDVWLPAETLDLIREYRVAIKGPLTTPVGGGIRSLNVALRQELDLYICLRPVRYYQGTPSPVK
              1111111111111111111111111111111111111111111111111111111111111111111111
Chain 2:   73 KSTQVYGQDVWLPAETLDLIREYRVAIKGPLTTPVGGGIRSLNVALRQELDLYICLRPVRYYQGTPSPVK

                    .    :    .    :    .    :    .    :    .    :    .    :    .    :
Chain 1:  143 HPELTDMVIFRENSEDIYAGIEWKADSADAEKVIKFLREEMGVKKIRFPEHCGIGIKPCSEEGTKRLVRA
              1111111111111111111111111111111111111111111111111111111111111111111111
Chain 2:  143 HPELTDMVIFRENSEDIYAGIEWKADSADAEKVIKFLREEMGVKKIRFPEHCGIGIKPCSEEGTKRLVRA

                    .    :    .    :    .    :    .    :    .    :    .    :    .    :
Chain 1:  213 AIEYAIANDRDSVTLVHKGNIMKFTEGAFKDWGYQLAREEFGGELIDGGPWLKVKNPNTGKEIVIKDVIA
              1111111111111111111111111111111111111111111111111111111111111111111111
Chain 2:  213 AIEYAIANDRDSVTLVHMGNIMKFTEGAFKDWGYQLAREEFGGELIDGGPWLKVKNPNTGKEIVIKDVIA

                    .    :    .    :    .    :    .    :    .    :    .    :    .    :
Chain 1:  283 DAFLQQILLRPAEYDVIACMNLNGDYISDALAAQVGGIGIAPGANIGDECALFEATHGTAPKYAGQDKVN
              1111111111111111111111111111111111111111111111111111111111111111111111
Chain 2:  283 DAFLQQILLRPAEYDVIACMNLNGDYISDALAAQVGGIGIAPGANIGDECALFEATHGTAPKYAGQDKVN

                    .    :    .    :    .    :    .    :    .    :    .    :
Chain 1:  353 PGSIILSAEMMLRHMGWTEAADLIVKGMEGAINAKTVTYDFERLMDGAKLLKCSEFGDAIIENM
              1111111111111111111111111111111111111111111111111111111111111111
Chain 2:  353 PGSIILSAEMMLRHMGWTEAADLIVKGMEGAINAKTVTYDFERLMDGAKLLKCSEFGDAIIENM

Note: positions are from PDB; the numbers between alignments are block index
```

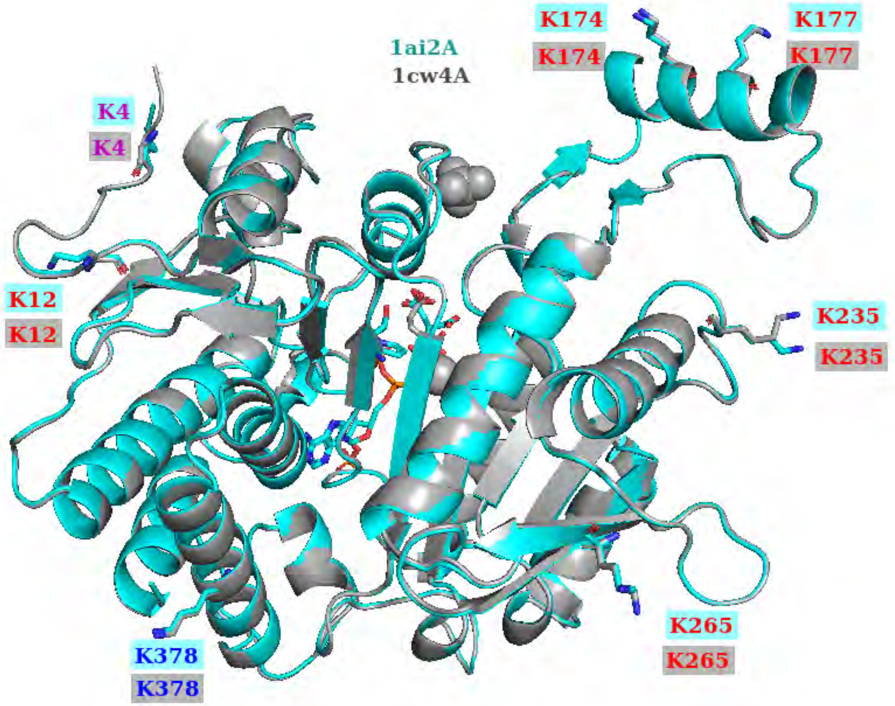

UniProt ID: P08200

PDB ID: 1CW7_A

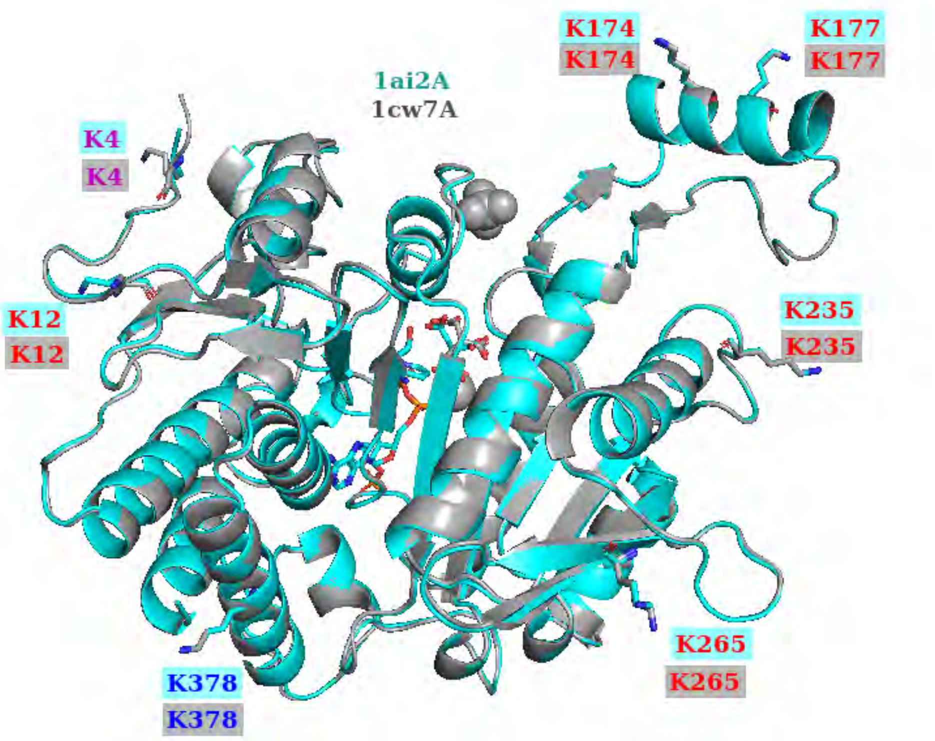


```
Align 1ai2.A.pdb 414 with 1cw7.A.pdb 415
Twists 0 ini-len 408 ini-rmsd 0.29 opt-equ 414 opt-rmsd 0.29 chain-rmsd 0.29 Score 1222.46 align-len 414 gaps 0 (0.00%)
P-value 0.00e+00 Afp-num 51728 Identity 99.76% Similarity 99.76%
Block  0 afp 51 score 1222.46 rmsd  0.29 gap 0 (0.00%)

                    .    :    .    :    .    :    .    :    .    :    .    :    .    :
Chain 1:    3 SKVVVPAQGKKITLQNGKLNVPENPIIPYIEGDGIGVDVTPAMLKVVDAAVEKAYKGERKISWMEIYTGE
              1111111111111111111111111111111111111111111111111111111111111111111111
Chain 2:    3 SKVVVPAQGKKITLQNGKLNVPENPIIPYIEGDGIGVDVTPAMLKVVDAAVEKAYKGERKISWMEIYTGE

                    .    :    .    :    .    :    .    :    .    :    .    :    .    :
Chain 1:   73 KSTQVYGQDVWLPAETLDLIREYRVAIKGPLTTPVGGGIRSLNVALRQELDLYICLRPVRYYQGTPSPVK
              1111111111111111111111111111111111111111111111111111111111111111111111
Chain 2:   73 KSTQVYGQDVWLPAETLDLIREYRVAIKGPLTTPVGGGIRSLNVALRQELDLYICLRPVRYYQGTPSPVK

                    .    :    .    :    .    :    .    :    .    :    .    :    .    :
Chain 1:  143 HPELTDMVIFRENSEDIYAGIEWKADSADAEKVIKFLREEMGVKKIRFPEHCGIGIKPCSEEGTKRLVRA
              1111111111111111111111111111111111111111111111111111111111111111111111
Chain 2:  143 HPELTDMVIFRENSEDIYAGIEWKADSADAEKVIKFLREEMGVKKIRFPEHCGIGIKPCSEEGTKRLVRA

                    .    :    .    :    .    :    .    :    .    :    .    :    .    :
Chain 1:  213 AIEYAIANDRDSVTLVHKGNIMKFTEGAFKDWGYQLAREEFGGELIDGGPWLKVKNPNTGKEIVIKDVIA
              1111111111111111111111111111111111111111111111111111111111111111111111
Chain 2:  213 AIEYAIANDRDSVTLVHKGNIMKFTEGAFKDWGYQLAREEFGGELIDGGPWLKVKNPNTGKEIVIKDVIA

                    .    :    .    :    .    :    .    :    .    :    .    :    .    :
Chain 1:  283 DAFLQQILLRPAEYDVIACMNLNGDYISDALAAQVGGIGIAPGANIGDECALFEATHGTAPKYAGQDKVN
              1111111111111111111111111111111111111111111111111111111111111111111111
Chain 2:  283 DAFLQQILLRPAEYDVIACMNLNGDYISDALAAQVGGIGIAPGANIGDECALFEATHGTAPAYAGQDKVN

                    .    :    .    :    .    :    .    :    .    :    .    :
Chain 1:  353 PGSIILSAEMMLRHMGWTEAADLIVKGMEGAINAKTVTYDFERLMDGAKLLKCSEFGDAIIENM
              1111111111111111111111111111111111111111111111111111111111111111
Chain 2:  353 PGSIILSAEMMLRHMGWTEAADLIVKGMEGAINAKTVTYDFERLMDGAKLLKCSEFGDAIIENM

Note: positions are from PDB; the numbers between alignments are block index
```

UniProt ID: P08200
PDB ID: 1GRO_A

```
Align 1ai2.A.pdb 414 with 1gro.A.pdb 414
Twists 0 ini-len 408 ini-rmsd 0.47 opt-equ 414 opt-rmsd 0.48 chain-rmsd 0.47 Score 1220.75 align-len 414 gaps 0 (0.00%)
P-value 0.00e+00 Afp-num 51713 Identity 99.28% Similarity 99.52%
Block  0 afp 51 score 1220.75 rmsd  0.47 gap 0 (0.00%)

                  .    :    .    :    .    :    .    :    .    :    .    :    .    :
Chain 1:    3 SKVVVPAQGKKITLQNGKLNVPENPIIPYIEGDGIGVDVTPAMLKVVDAAVEKAYKGERKISWMEIYTGE
              1111111111111111111111111111111111111111111111111111111111111111111111
Chain 2:    3 SKVVVPAQGKKITLQNGKLNVPENPIIPYIEGDGIGVDVTPAMLKVVDAAVEKAYKGERKISWMEIYTGE

                  .    :    .    :    .    :    .    :    .    :    .    :    .    :
Chain 1:   73 KSTQVYGQDVWLPAETLDLIREYRVAIKGPLTTPVGGGIRSLNVALRQELDLYICLRPVRYYQGTPSPVK
              1111111111111111111111111111111111111111111111111111111111111111111111
Chain 2:   73 KSTQVYGQDVWLPAETLDLIREYRVAIKGPLTTPVGGGIRELLVALRQELDLYICLRPVRYYQGTPSPVK

                  .    :    .    :    .    :    .    :    .    :    .    :    .    :
Chain 1:  143 HPELTDMVIFRENSEDIYAGIEWKADSADAEKVIKFLREEMGVKKIRFPEHCGIGIKPCSEEGTKRLVRA
              1111111111111111111111111111111111111111111111111111111111111111111111
Chain 2:  143 HPELTDMVIFRENSEDIYAGIEWKADSADAEKVIKFLREEMGVKKIRFPDHCGIGIKPCSEEGTKRLVRA

                  .    :    .    :    .    :    .    :    .    :    .    :    .    :
Chain 1:  213 AIEYAIANDRDSVTLVHKGNIMKFTEGAFKDWGYQLAREEFGGELIDGGPWLKVKNPNTGKEIVIKDVIA
              1111111111111111111111111111111111111111111111111111111111111111111111
Chain 2:  213 AIEYAIANDRDSVTLVHKGNIMKFTEGAFKDWGYQLAREEFGGELIDGGPWLKVKNPNTGKEIVIKDVIA

                  .    :    .    :    .    :    .    :    .    :    .    :    .    :
Chain 1:  283 DAFLQQILLRPAEYDVIACMNLNGDYISDALAAQVGGIGIAPGANIGDECALFEATHGTAPKYAGQDKVN
              1111111111111111111111111111111111111111111111111111111111111111111111
Chain 2:  283 DAFLQQILLRPAEYDVIACMNLNGDYISDALAAQVGGIGIAPGANIGDECALFEATHGTAPKYAGQDKVN

                  .    :    .    :    .    :    .    :    .    :    .    :
Chain 1:  353 PGSIILSAEMMLRHMGWTEAADLIVKGMEGAINAKTVTYDFERLMDGAKLLKCSEFGDAIIENM
              1111111111111111111111111111111111111111111111111111111111111111
Chain 2:  353 PGSIILSAEMMLRHMGWTEAADLIVKGMEGAINAKTVTYDFERLMDGAKLLKCSEFGDAIIENM

Note: positions are from PDB; the numbers between alignments are block index
```

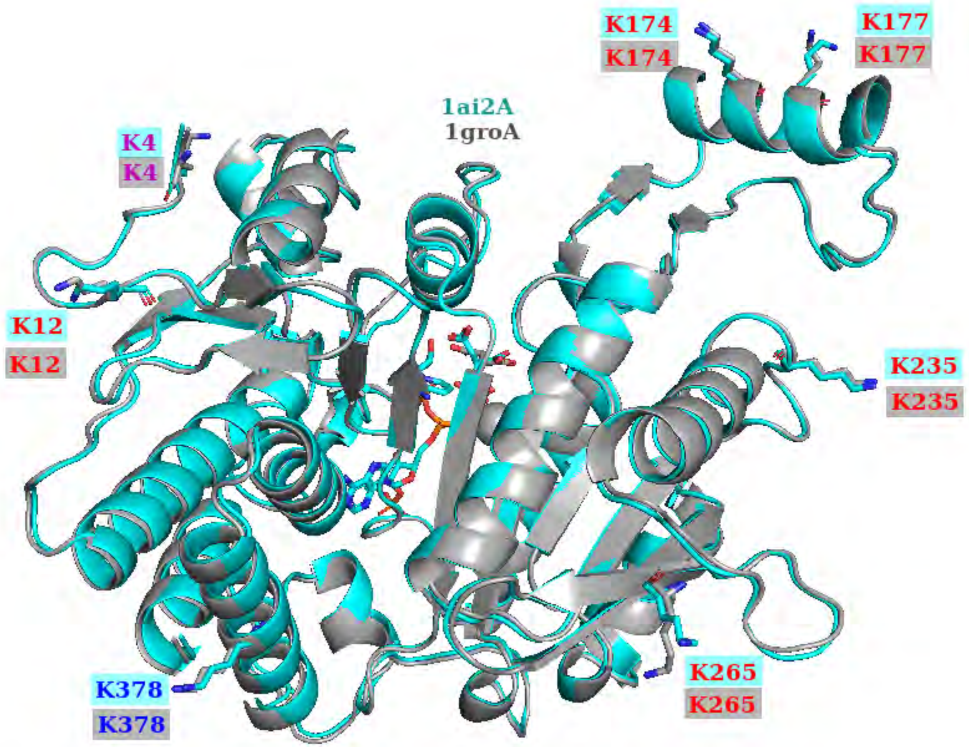

UniProt ID: P08200
PDB ID: 1GRP_A

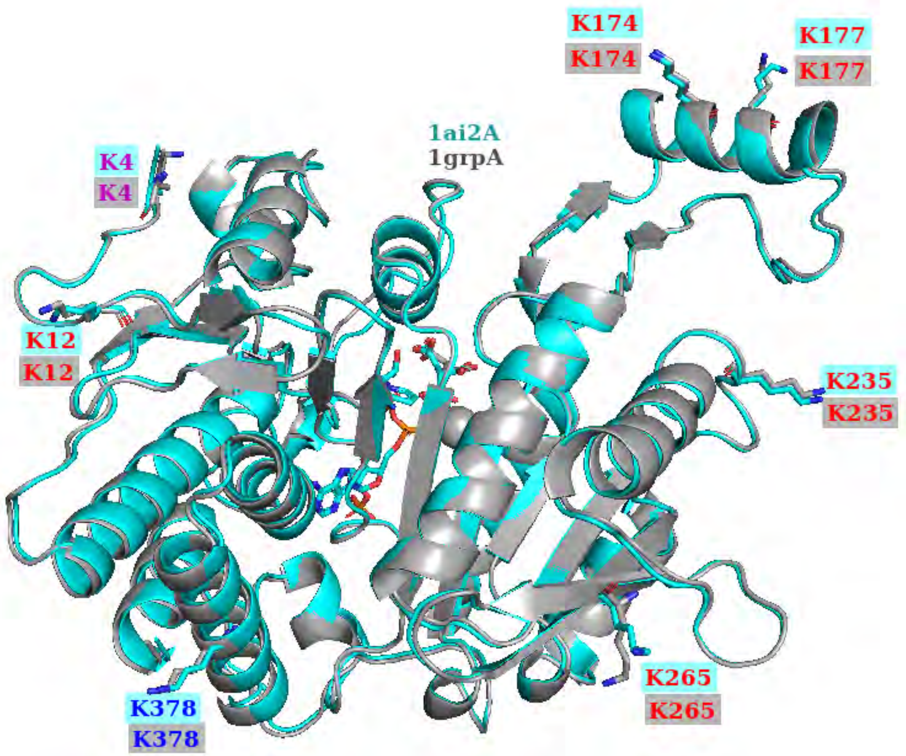


```
Align 1ai2.A.pdb 414 with 1grp.A.pdb 414
Twists 0 ini-len 408 ini-rmsd 0.48 opt-equ 414 opt-rmsd 0.48 chain-rmsd 0.48 Score 1220.69 align-len 414 gaps 0
(0.00%)
P-value 0.00e+00 Afp-num 51694 Identity 99.52% Similarity 99.76%
Block  0 afp 51 score 1220.69 rmsd  0.48 gap 0 (0.00%)

                    .    :    .    :    .    :    .    :    .    :    .    :    .    :
Chain 1:    3 SKVVVPAQGKKITLQNGKLNVPENPIIPYIEGDGIGVDVTPAMLKVVDAAVEKAYKGERKISWMEIYTGE
              1111111111111111111111111111111111111111111111111111111111111111111111
Chain 2:    3 SKVVVPAQGKKITLQNGKLNVPENPIIPYIEGDGIGVDVTPAMLKVVDAAVEKAYKGERKISWMEIYTGE

                    .    :    .    :    .    :    .    :    .    :    .    :    .    :
Chain 1:   73 KSTQVYGQDVWLPAETLDLIREYRVAIKGPLTTPVGGGIRSLNVALRQELDLYICLRPVRYYQGTPSPVK
              1111111111111111111111111111111111111111111111111111111111111111111111
Chain 2:   73 KSTQVYGQDVWLPAETLDLIREYRVAIKGPLTTPVGGGIRSLLVALRQELDLYICLRPVRYYQGTPSPVK

                    .    :    .    :    .    :    .    :    .    :    .    :    .    :
Chain 1:  143 HPELTDMVIFRENSEDIYAGIEWKADSADAEKVIKFLREEMGVKKIRFPEHCGIGIKPCSEEGTKRLVRA
              1111111111111111111111111111111111111111111111111111111111111111111111
Chain 2:  143 HPELTDMVIFRENSEDIYAGIEWKADSADAEKVIKFLREEMGVKKIRFPDHCGIGIKPCSEEGTKRLVRA

                    .    :    .    :    .    :    .    :    .    :    .    :    .    :
Chain 1:  213 AIEYAIANDRDSVTLVHKGNIMKFTEGAFKDWGYQLAREEFGGELIDGGPWLKVKNPNTGKEIVIKDVIA
              1111111111111111111111111111111111111111111111111111111111111111111111
Chain 2:  213 AIEYAIANDRDSVTLVHKGNIMKFTEGAFKDWGYQLAREEFGGELIDGGPWLKVKNPNTGKEIVIKDVIA

                    .    :    .    :    .    :    .    :    .    :    .    :    .    :
Chain 1:  283 DAFLQQILLRPAEYDVIACMNLNGDYISDALAAQVGGIGIAPGANIGDECALFEATHGTAPKYAGQDKVN
              1111111111111111111111111111111111111111111111111111111111111111111111
Chain 2:  283 DAFLQQILLRPAEYDVIACMNLNGDYISDALAAQVGGIGIAPGANIGDECALFEATHGTAPKYAGQDKVN

                    .    :    .    :    .    :    .    :    .    :    .    :
Chain 1:  353 PGSIILSAEMMLRHMGWTEAADLIVKGMEGAINAKTVTYDFERLMDGAKLLKCSEFGDAIIENM
              1111111111111111111111111111111111111111111111111111111111111111
Chain 2:  353 PGSIILSAEMMLRHMGWTEAADLIVKGMEGAINAKTVTYDFERLMDGAKLLKCSEFGDAIIENM

Note: positions are from PDB; the numbers between alignments are block index
```

UniProt ID: P08200

PDB ID: 1HJ6_A

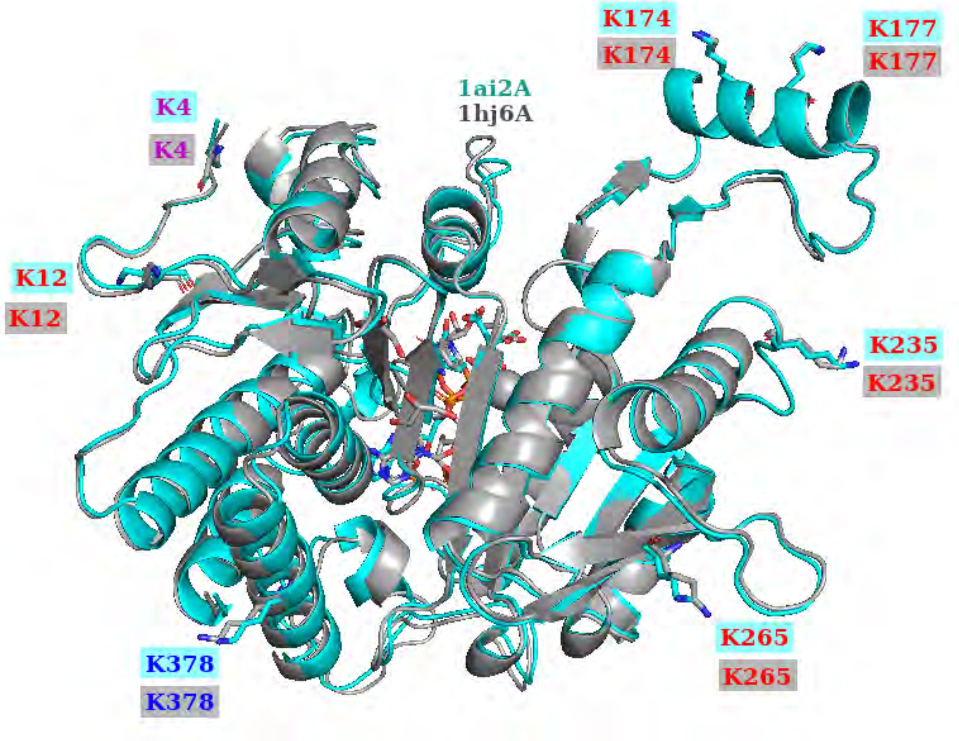


```
Align 1ai2.A.pdb 414 with 1hj6.A.pdb 414
Twists 0 ini-len 408 ini-rmsd 0.88 opt-equ 414 opt-rmsd 0.88 chain-rmsd 0.88 Score 1218.53 align-len 414 gaps 0 (0.00%)
P-value 0.00e+00 Afp-num 51848 Identity 99.76% Similarity 99.76%
Block  0 afp 51 score 1218.53 rmsd  0.88 gap 1 (0.00%)

                   .    :    .    :    .    :    .    :    .    :    .    :    .    :
Chain 1:    3 SKVVVPAQGKKITLQNGKLNVPENPIIPYIEGDGIGVDVTPAMLKVVDAAVEKAYKGERKISWMEIYTGE
              1111111111111111111111111111111111111111111111111111111111111111111111
Chain 2:    3 SKVVVPAQGKKITLQNGKLNVPENPIIPYIEGDGIGVDVTPAMLKVVDAAVEKAYKGERKISWMEIYTGE

                   .    :    .    :    .    :    .    :    .    :    .    :    .    :
Chain 1:   73 KSTQVYGQDVWLPAETLDLIREYRVAIKGPLTTPVGGGIRSLNVALRQELDLYICLRPVRYYQGTPSPVK
              1111111111111111111111111111111111111111111111111111111111111111111111
Chain 2:   73 KSTQVYGQDVWLPAETLDLIREYRVAIKGPLTTPVGGGIRELNVALRQELDLYICLRPVRYYQGTPSPVK

                   .    :    .    :    .    :    .    :    .    :    .    :    .    :
Chain 1:  143 HPELTDMVIFRENSEDIYAGIEWKADSADAEKVIKFLREEMGVKKIRFPEHCGIGIKPCSEEGTKRLVRA
              1111111111111111111111111111111111111111111111111111111111111111111111
Chain 2:  143 HPELTDMVIFRENSEDIYAGIEWKADSADAEKVIKFLREEMGVKKIRFPEHCGIGIKPCSEEGTKRLVRA

                   .    :    .    :    .    :    .    :    .    :    .    :    .    :
Chain 1:  213 AIEYAIANDRDSVTLVHKGNIMKFTEGAFKDWGYQLAREEFGGELIDGGPWLKVKNPNTGKEIVIKDVIA
              1111111111111111111111111111111111111111111111111111111111111111111111
Chain 2:  213 AIEYAIANDRDSVTLVHKGNIMKFTEGAFKDWGYQLAREEFGGELIDGGPWLKVKNPNTGKEIVIKDVIA

                   .    :    .    :    .    :    .    :    .    :    .    :    .    :
Chain 1:  283 DAFLQQILLRPAEYDVIACMNLNGDYISDALAAQVGGIGIAPGANIGDECALFEATHGTAPKYAGQDKVN
              1111111111111111111111111111111111111111111111111111111111111111111111
Chain 2:  283 DAFLQQILLRPAEYDVIACMNLNGDYISDALAAQVGGIGIAPGANIGDECALFEATHGTAPKYAGQDKVN

                   .    :    .    :    .    :    .    :    .    :    .    :
Chain 1:  353 PGSIILSAEMMLRHMGWTEAADLIVKGMEGAINAKTVTYDFERLMDGAKLLKCSEFGDAIIENM
              1111111111111111111111111111111111111111111111111111111111111111
Chain 2:  353 PGSIILSAEMMLRHMGWTEAADLIVKGMEGAINAKTVTYDFERLMDGAKLLKCSEFGDAIIENM

Note: positions are from PDB; the numbers between alignments are block index
```

UniProt ID: P08200

PDB ID: 1IDC_A

```
Align 1ai2.A.pdb 414 with 1idc.A.pdb 414
Twists 0 ini-len 408 ini-rmsd 0.52 opt-equ 414 opt-rmsd 0.53 chain-rmsd 0.52 Score 1218.00 align-len 414 gaps 0 (0.00%)
P-value 0.00e+00 Afp-num 51483 Identity 99.76% Similarity 99.76%
Block  0 afp 51 score 1218.00 rmsd  0.52 gap 0 (0.00%)

                 .    :    .    :    .    :    .    :    .    :    .    :    .    :
Chain 1:     3 SKVVVPAQGKKITLQNGKLNVPENPIIPYIEGDGIGVDVTPAMLKVVDAAVEKAYKGERKISWMEIYTGE
               1111111111111111111111111111111111111111111111111111111111111111111111
Chain 2:     3 SKVVVPAQGKKITLQNGKLNVPENPIIPYIEGDGIGVDVTPAMLKVVDAAVEKAYKGERKISWMEIYTGE

                 .    :    .    :    .    :    .    :    .    :    .    :    .    :
Chain 1:    73 KSTQVYGQDVWLPAETLDLIREYRVAIKGPLTTPVGGGIRSLNVALRQELDLYICLRPVRYYQGTPSPVK
               1111111111111111111111111111111111111111111111111111111111111111111111
Chain 2:    73 KSTQVYGQDVWLPAETLDLIREYRVAIKGPLTTPVGGGIRSLNVALRQELDLYICLRPVRYYQGTPSPVK

                 .    :    .    :    .    :    .    :    .    :    .    :    .    :
Chain 1:   143 HPELTDMVIFRENSEDIYAGIEWKADSADAEKVIKFLREEMGVKKIRFPEHCGIGIKPCSEEGTKRLVRA
               1111111111111111111111111111111111111111111111111111111111111111111111
Chain 2:   143 HPELTDMVIFRENSEDIYAGIEWKADSADAEKVIKFLREEMGVKKIRFPEHCGIGIKPCSEEGTKRLVRA

                 .    :    .    :    .    :    .    :    .    :    .    :    .    :
Chain 1:   213 AIEYAIANDRDSVTLVHKGNIMKFTEGAFKDWGYQLAREEFGGELIDGGPWLKVKNPNTGKEIVIKDVIA
               1111111111111111111111111111111111111111111111111111111111111111111111
Chain 2:   213 AIEYAIANDRDSVTLVHMGNIMKFTEGAFKDWGYQLAREEFGGELIDGGPWLKVKNPNTGKEIVIKDVIA

                 .    :    .    :    .    :    .    :    .    :    .    :    .    :
Chain 1:   283 DAFLQQILLRPAEYDVIACMNLNGDYISDALAAQVGGIGIAPGANIGDECALFEATHGTAPKYAGQDKVN
               1111111111111111111111111111111111111111111111111111111111111111111111
Chain 2:   283 DAFLQQILLRPAEYDVIACMNLNGDYISDALAAQVGGIGIAPGANIGDECALFEATHGTAPKYAGQDKVN

                 .    :    .    :    .    :    .    :    .    :    .    :
Chain 1:   353 PGSIILSAEMMLRHMGWTEAADLIVKGMEGAINAKTVTYDFERLMDGAKLLKCSEFGDAIIENM
               1111111111111111111111111111111111111111111111111111111111111111
Chain 2:   353 PGSIILSAEMMLRHMGWTEAADLIVKGMEGAINAKTVTYDFERLMDGAKLLKCSEFGDAIIENM

Note: positions are from PDB; the numbers between alignments are block index
```

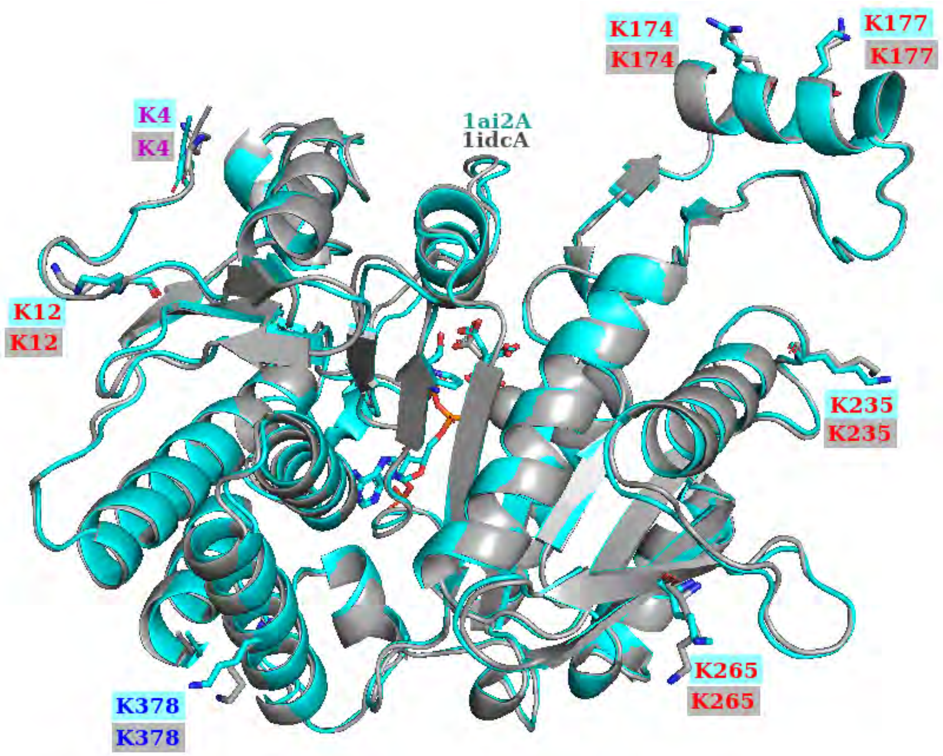

UniProt ID: P08200
PDB ID: 1IDD_A

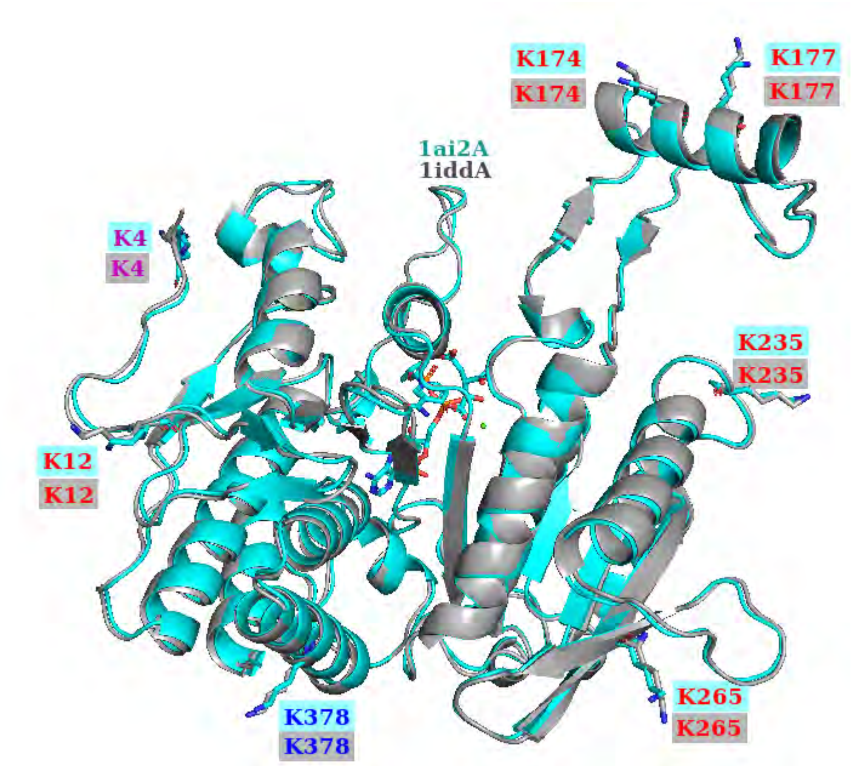


```
Align 1ai2.A.pdb 414 with 1idd.A.pdb 414
Twists 0 ini-len 408 ini-rmsd 0.53 opt-equ 414 opt-rmsd 0.54 chain-rmsd 0.53 Score 1218.56 align-len 414 gaps 0 (0.00%)
P-value 0.00e+00 Afp-num 51519 Identity 99.76% Similarity 100.00%
Block  0 afp 51 score 1218.56 rmsd  0.53 gap 0 (0.00%)

                    .    :    .    :    .    :    .    :    .    :    .    :    .    :
Chain 1:    3 SKVVVPAQGKKITLQNGKLNVPENPIIPYIEGDGIGVDVTPAMLKVVDAAVEKAYKGERKISWMEIYTGE
              1111111111111111111111111111111111111111111111111111111111111111111111
Chain 2:    3 SKVVVPAQGKKITLQNGKLNVPENPIIPYIEGDGIGVDVTPAMLKVVDAAVEKAYKGERKISWMEIYTGE

                    .    :    .    :    .    :    .    :    .    :    .    :    .    :
Chain 1:   73 KSTQVYGQDVWLPAETLDLIREYRVAIKGPLTTPVGGGIRSLNVALRQELDLYICLRPVRYYQGTPSPVK
              1111111111111111111111111111111111111111111111111111111111111111111111
Chain 2:   73 KSTQVYGQDVWLPAETLDLIREYRVAIKGPLTTPVGGGIRSLNVALRQELDLYICLRPVRYYQGTPSPVK

                    .    :    .    :    .    :    .    :    .    :    .    :    .    :
Chain 1:  143 HPELTDMVIFRENSEDIYAGIEWKADSADAEKVIKFLREEMGVKKIRFPEHCGIGIKPCSEEGTKRLVRA
              1111111111111111111111111111111111111111111111111111111111111111111111
Chain 2:  143 HPELTDMVIFRENSEDIFAGIEWKADSADAEKVIKFLREEMGVKKIRFPEHCGIGIKPCSEEGTKRLVRA

                    .    :    .    :    .    :    .    :    .    :    .    :    .    :
Chain 1:  213 AIEYAIANDRDSVTLVHKGNIMKFTEGAFKDWGYQLAREEFGGELIDGGPWLKVKNPNTGKEIVIKDVIA
              1111111111111111111111111111111111111111111111111111111111111111111111
Chain 2:  213 AIEYAIANDRDSVTLVHKGNIMKFTEGAFKDWGYQLAREEFGGELIDGGPWLKVKNPNTGKEIVIKDVIA

                    .    :    .    :    .    :    .    :    .    :    .    :    .    :
Chain 1:  283 DAFLQQILLRPAEYDVIACMNLNGDYISDALAAQVGGIGIAPGANIGDECALFEATHGTAPKYAGQDKVN
              1111111111111111111111111111111111111111111111111111111111111111111111
Chain 2:  283 DAFLQQILLRPAEYDVIACMNLNGDYISDALAAQVGGIGIAPGANIGDECALFEATHGTAPKYAGQDKVN

                    .    :    .    :    .    :    .    :    .    :    .    :
Chain 1:  353 PGSIILSAEMMLRHMGWTEAADLIVKGMEGAINAKTVTYDFERLMDGAKLLKCSEFGDAIIENM
              1111111111111111111111111111111111111111111111111111111111111111
Chain 2:  353 PGSIILSAEMMLRHMGWTEAADLIVKGMEGAINAKTVTYDFERLMDGAKLLKCSEFGDAIIENM

Note: positions are from PDB; the numbers between alignments are block index
```

UniProt ID: P08200

PDB ID: 1IDE_A

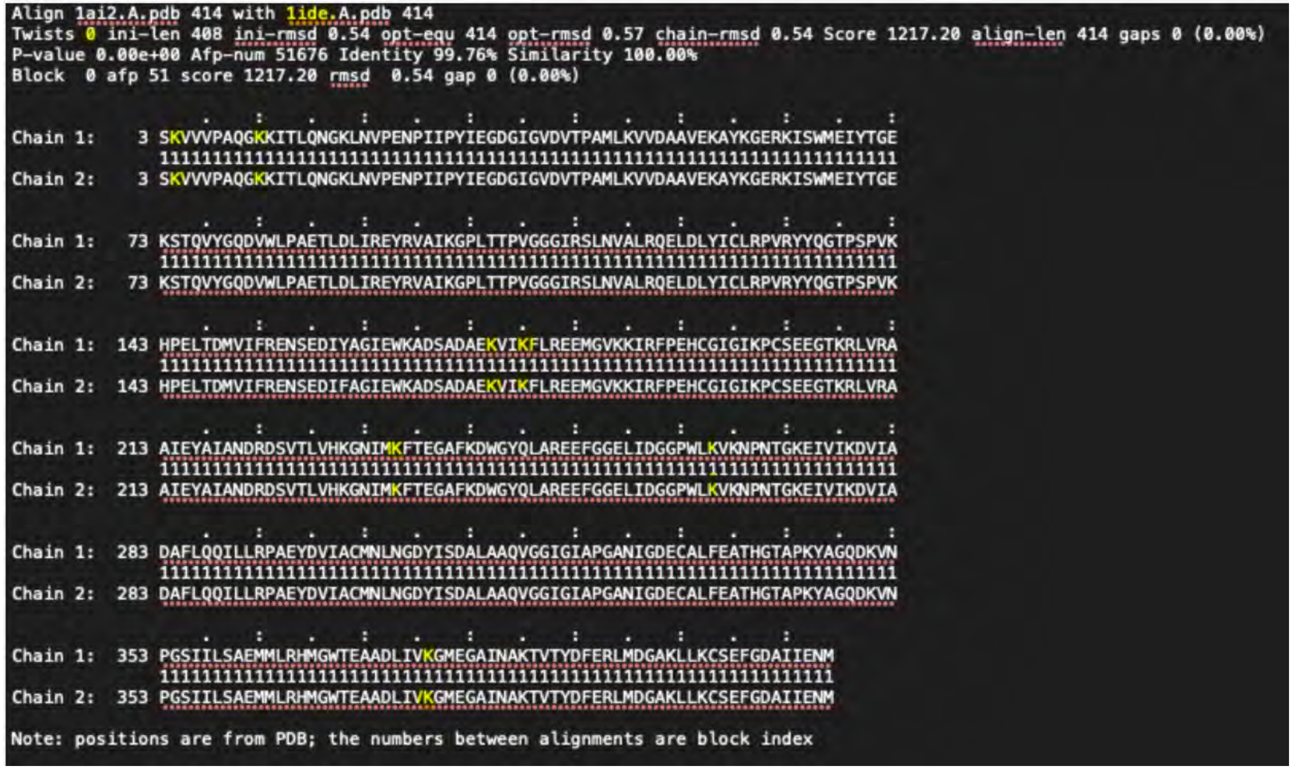

```
Align 1ai2.A.pdb 414 with 1ide.A.pdb 414
Twists 0 ini-len 408 ini-rmsd 0.54 opt-equ 414 opt-rmsd 0.57 chain-rmsd 0.54 Score 1217.20 align-len 414 gaps 0 (0.00%)
P-value 0.00e+00 Afp-num 51676 Identity 99.76% Similarity 100.00%
Block  0 afp 51 score 1217.20 rmsd  0.54 gap 0 (0.00%)

                .    :    .    :    .    :    .    :    .    :    .    :    .    :
Chain 1:    3 SKVVVPAQGKKITLQNGKLNVPENPIIPYIEGDGIGVDVTPAMLKVVDAAVEKAYKGERKISWMEIYTGE
              1111111111111111111111111111111111111111111111111111111111111111111111
Chain 2:    3 SKVVVPAQGKKITLQNGKLNVPENPIIPYIEGDGIGVDVTPAMLKVVDAAVEKAYKGERKISWMEIYTGE

                .    :    .    :    .    :    .    :    .    :    .    :    .    :
Chain 1:   73 KSTQVYGQDVWLPAETLDLIREYRVAIKGPLTTPVGGGIRSLNVALRQELDLYICLRPVRYYQGTPSPVK
              1111111111111111111111111111111111111111111111111111111111111111111111
Chain 2:   73 KSTQVYGQDVWLPAETLDLIREYRVAIKGPLTTPVGGGIRSLNVALRQELDLYICLRPVRYYQGTPSPVK

                .    :    .    :    .    :    .    :    .    :    .    :    .    :
Chain 1:  143 HPELTDMVIFRENSEDIYAGIEWKADSADAEKVIKFLREEMGVKKIRFPEHCGIGIKPCSEEGTKRLVRA
              1111111111111111111111111111111111111111111111111111111111111111111111
Chain 2:  143 HPELTDMVIFRENSEDIFAGIEWKADSADAEKVIKFLREEMGVKKIRFPEHCGIGIKPCSEEGTKRLVRA

                .    :    .    :    .    :    .    :    .    :    .    :    .    :
Chain 1:  213 AIEYAIANDRDSVTLVHKGNIMKFTEGAFKDWGYQLAREEFGGELIDGGPWLKVKNPNTGKEIVIKDVIA
              1111111111111111111111111111111111111111111111111111111111111111111111
Chain 2:  213 AIEYAIANDRDSVTLVHKGNIMKFTEGAFKDWGYQLAREEFGGELIDGGPWLKVKNPNTGKEIVIKDVIA

                .    :    .    :    .    :    .    :    .    :    .    :    .    :
Chain 1:  283 DAFLQQILLRPAEYDVIACMNLNGDYISDALAAQVGGIGIAPGANIGDECALFEATHGTAPKYAGQDKVN
              1111111111111111111111111111111111111111111111111111111111111111111111
Chain 2:  283 DAFLQQILLRPAEYDVIACMNLNGDYISDALAAQVGGIGIAPGANIGDECALFEATHGTAPKYAGQDKVN

                .    :    .    :    .    :    .    :    .    :    .    :
Chain 1:  353 PGSIILSAEMMLRHMGWTEAADLIVKGMEGAINAKTVTYDFERLMDGAKLLKCSEFGDAIIENM
              11111111111111111111111111111111111111111111111111111111111111
Chain 2:  353 PGSIILSAEMMLRHMGWTEAADLIVKGMEGAINAKTVTYDFERLMDGAKLLKCSEFGDAIIENM

Note: positions are from PDB; the numbers between alignments are block index
```

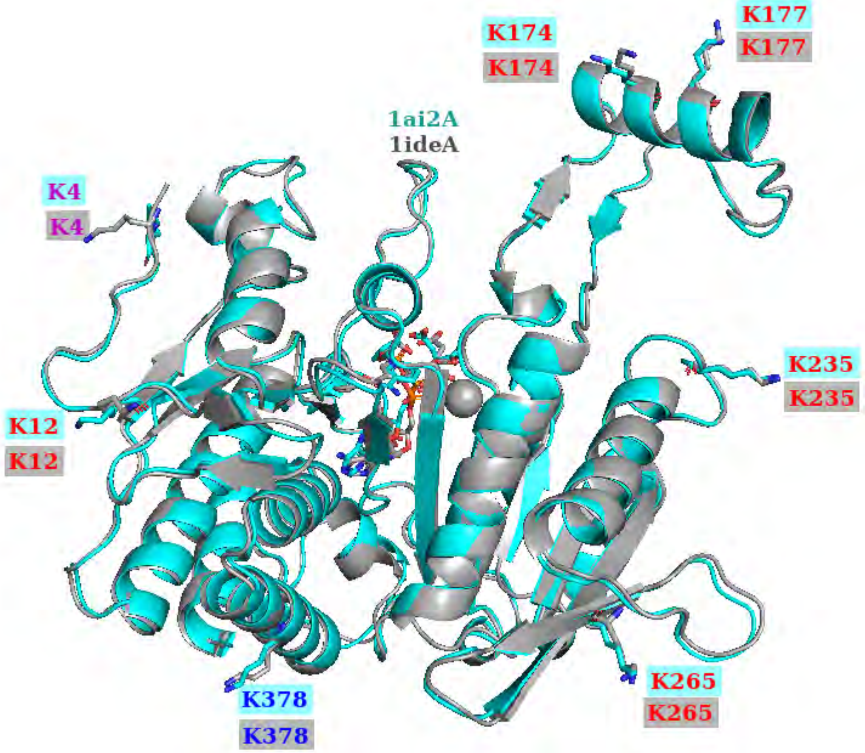

UniProt ID: P08200

PDB ID: 1IDF_A

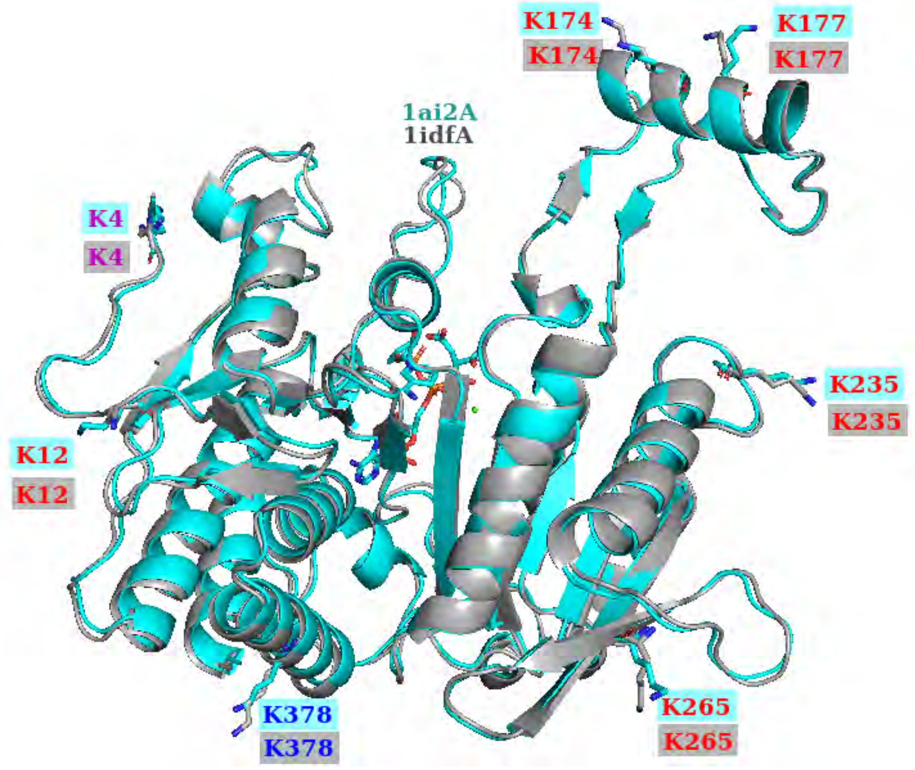


```
Align 1ai2.A.pdb 414 with 1idf.A.pdb 414
Twists 0 ini-len 408 ini-rmsd 0.57 opt-equ 414 opt-rmsd 0.57 chain-rmsd 0.57 Score 1217.65 align-len 414 gaps 0 (0.00%)
P-value 0.00e+00 Afp-num 51371 Identity 99.76% Similarity 99.76%
Block  0 afp 51 score 1217.65 rmsd  0.57 gap 0 (0.00%)

                  .    :    .    :    .    :    .    :    .    :    .    :    .    :
Chain 1:    3 SKVVVPAQGKKITLQNGKLNVPENPIIPYIEGDGIGVDVTPAMLKVVDAAVEKAYKGERKISWMEIYTGE
              1111111111111111111111111111111111111111111111111111111111111111111111
Chain 2:    3 SKVVVPAQGKKITLQNGKLNVPENPIIPYIEGDGIGVDVTPAMLKVVDAAVEKAYKGERKISWMEIYTGE

                  .    :    .    :    .    :    .    :    .    :    .    :    .    :
Chain 1:   73 KSTQVYGQDVWLPAETLDLIREYRVAIKGPLTTPVGGGIRSLNVALRQELDLYICLRPVRYYQGTPSPVK
              1111111111111111111111111111111111111111111111111111111111111111111111
Chain 2:   73 KSTQVYGQDVWLPAETLDLIREYRVAIKGPLTTPVGGGIRSLNVALRQELDLYICLRPVRYYQGTPSPVK

                  .    :    .    :    .    :    .    :    .    :    .    :    .    :
Chain 1:  143 HPELTDMVIFRENSEDIYAGIEWKADSADAEKVIKFLREEMGVKKIRFPEHCGIGIKPCSEEGTKRLVRA
              1111111111111111111111111111111111111111111111111111111111111111111111
Chain 2:  143 HPELTDMVIFRENSEDIYAGIEWKADSADAEKVIKFLREEMGVKKIRFPEHCGIGIKPCSEEGTKRLVRA

                  .    :    .    :    .    :    .    :    .    :    .    :    .    :
Chain 1:  213 AIEYAIANDRDSVTLVHKGNIMKFTEGAFKDWGYQLAREEFGGELIDGGPWLKVKNPNTGKEIVIKDVIA
              1111111111111111111111111111111111111111111111111111111111111111111111
Chain 2:  213 AIEYAIANDRDSVTLVHMGNIMKFTEGAFKDWGYQLAREEFGGELIDGGPWLKVKNPNTGKEIVIKDVIA

                  .    :    .    :    .    :    .    :    .    :    .    :    .    :
Chain 1:  283 DAFLQQILLRPAEYDVIACMNLNGDYISDALAAQVGGIGIAPGANIGDECALFEATHGTAPKYAGQDKVN
              1111111111111111111111111111111111111111111111111111111111111111111111
Chain 2:  283 DAFLQQILLRPAEYDVIACMNLNGDYISDALAAQVGGIGIAPGANIGDECALFEATHGTAPKYAGQDKVN

                  .    :    .    :    .    :    .    :    .    :    .    :
Chain 1:  353 PGSIILSAEMMLRHMGWTEAADLIVKGMEGAINAKTVTYDFERLMDGAKLLKCSEFGDAIIENM
              1111111111111111111111111111111111111111111111111111111111111111
Chain 2:  353 PGSIILSAEMMLRHMGWTEAADLIVKGMEGAINAKTVTYDFERLMDGAKLLKCSEFGDAIIENM

Note: positions are from PDB; the numbers between alignments are block index
```

UniProt ID: P08200

PDB ID: 1IKA_A

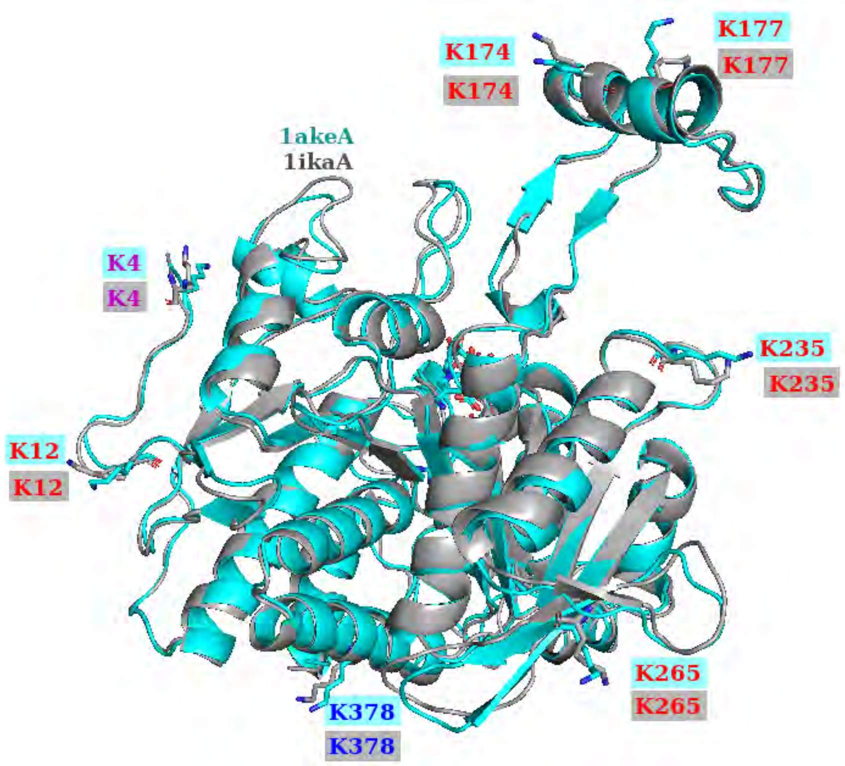


```
Align 1ai2.A.pdb 414 with 1ika.A.pdb 414
Twists 0 ini-len 408 ini-rmsd 0.91 opt-equ 414 opt-rmsd 0.91 chain-rmsd 0.91 Score 1190.73 align-len 414 gaps 0 (0.00%)
P-value 0.00e+00 Afp-num 51092 Identity 100.00% Similarity 100.00%
Block  0 afp 51 score 1190.73 rmsd  0.91 gap 1 (0.00%)

                   .    :    .    :    .    :    .    :    .    :    .    :    .    :
Chain 1:     3 SKVVVPAQGKKITLQNGKLNVPENPIIPYIEGDGIGVDVTPAMLKVVDAAVEKAYKGERKISWMEIYTGE
               1111111111111111111111111111111111111111111111111111111111111111111111
Chain 2:     3 SKVVVPAQGKKITLQNGKLNVPENPIIPYIEGDGIGVDVTPAMLKVVDAAVEKAYKGERKISWMEIYTGE

                   .    :    .    :    .    :    .    :    .    :    .    :    .    :
Chain 1:    73 KSTQVYGQDVWLPAETLDLIREYRVAIKGPLTTPVGGGIRSLNVALRQELDLYICLRPVRYYQGTPSPVK
               1111111111111111111111111111111111111111111111111111111111111111111111
Chain 2:    73 KSTQVYGQDVWLPAETLDLIREYRVAIKGPLTTPVGGGIRSLNVALRQELDLYICLRPVRYYQGTPSPVK

                   .    :    .    :    .    :    .    :    .    :    .    :    .    :
Chain 1:   143 HPELTDMVIFRENSEDIYAGIEWKADSADAEKVIKFLREEMGVKKIRFPEHCGIGIKPCSEEGTKRLVRA
               1111111111111111111111111111111111111111111111111111111111111111111111
Chain 2:   143 HPELTDMVIFRENSEDIYAGIEWKADSADAEKVIKFLREEMGVKKIRFPEHCGIGIKPCSEEGTKRLVRA

                   .    :    .    :    .    :    .    :    .    :    .    :    .    :
Chain 1:   213 AIEYAIANDRDSVTLVHKGNIMKFTEGAFKDWGYQLAREEFGGELIDGGPWLKVKNPNTGKEIVIKDVIA
               1111111111111111111111111111111111111111111111111111111111111111111111
Chain 2:   213 AIEYAIANDRDSVTLVHKGNIMKFTEGAFKDWGYQLAREEFGGELIDGGPWLKVKNPNTGKEIVIKDVIA

                   .    :    .    :    .    :    .    :    .    :    .    :    .    :
Chain 1:   283 DAFLQQILLRPAEYDVIACMNLNGDYISDALAAQVGGIGIAPGANIGDECALFEATHGTAPKYAGQDKVN
               1111111111111111111111111111111111111111111111111111111111111111111111
Chain 2:   283 DAFLQQILLRPAEYDVIACMNLNGDYISDALAAQVGGIGIAPGANIGDECALFEATHGTAPKYAGQDKVN

                   .    :    .    :    .    :    .    :    .    :    .    :
Chain 1:   353 PGSIILSAEMMLRHMGWTEAADLIVKGMEGAINAKTVTYDFERLMDGAKLLKCSEFGDAIIENM
               1111111111111111111111111111111111111111111111111111111111111111
Chain 2:   353 PGSIILSAEMMLRHMGWTEAADLIVKGMEGAINAKTVTYDFERLMDGAKLLKCSEFGDAIIENM

Note: positions are from PDB; the numbers between alignments are block index
```

UniProt ID: P08200

PDB ID: 1ISO_A

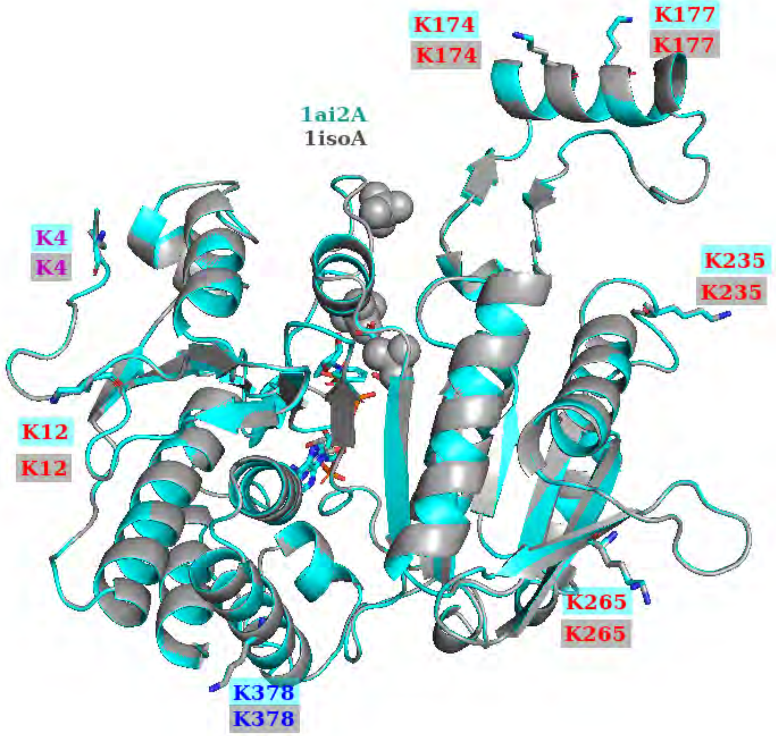


```
Align 1ai2.A.pdb 414 with 1iso.A.pdb 414
Twists 0 ini-len 408 ini-rmsd 0.09 opt-equ 414 opt-rmsd 0.09 chain-rmsd 0.09 Score 1223.67 align-len 414 gaps 0 (0.00%)
P-value 0.00e+00 Afp-num 51499 Identity 98.31% Similarity 98.31%
Block  0 afp 51 score 1223.67 rmsd  0.09 gap 0 (0.00%)

                  .    :    .    :    .    :    .    :    .    :    .    :    .    :
Chain 1:    3 SKVVVPAQGKKITLQNGKLNVPENPIIPYIEGDGIGVDVTPAMLKVVDAAVEKAYKGERKISWMEIYTGE
              1111111111111111111111111111111111111111111111111111111111111111111111
Chain 2:    3 SKVVVPAQGKKITLQNGKLNVPENPIIPYIEGDGIGVDVTPAMLKVVDAAVEKAYKGERKISWMEIYTGE

                  .    :    .    :    .    :    .    :    .    :    .    :    .    :
Chain 1:   73 KSTQVYGQDVWLPAETLDLIREYRVAIKGPLTTPVGGGIRSLNVALRQELDLYICLRPVRYYQGTPSPVK
              1111111111111111111111111111111111111111111111111111111111111111111111
Chain 2:   73 KSTQVYGQDVWLPAETLDLIREYRVAIKGPLTTPVGGGIRSLNVALRQELDLYICLRPVRYYQGTPSPVK

                  .    :    .    :    .    :    .    :    .    :    .    :    .    :
Chain 1:  143 HPELTDMVIFRENSEDIYAGIEWKADSADAEKVIKFLREEMGVKKIRFPEHCGIGIKPCSEEGTKRLVRA
              1111111111111111111111111111111111111111111111111111111111111111111111
Chain 2:  143 HPELTDMVIFRENSEDIYAGIEWKADSADAEKVIKFLREEMGVKKIRFPEHCGIGIKPMSEEGTKRLVRA

                  .    :    .    :    .    :    .    :    .    :    .    :    .    :
Chain 1:  213 AIEYAIANDRDSVTLVHKGNIMKFTEGAFKDWGYQLAREEFGGELIDGGPWLKVKNPNTGKEIVIKDVIA
              1111111111111111111111111111111111111111111111111111111111111111111111
Chain 2:  213 AIEYAIANDRDSVTLVHKGNIMKFTEGAFKDWGYQLAREEFGGELIDGGPWLKVKNPNTGKEIVIKDVIA

                  .    :    .    :    .    :    .    :    .    :    .    :    .    :
Chain 1:  283 DAFLQQILLRPAEYDVIACMNLNGDYISDALAAQVGGIGIAPGANIGDECALFEATHGTAPKYAGQDKVN
              1111111111111111111111111111111111111111111111111111111111111111111111
Chain 2:  283 DAFLQQILLRPAEYDVIACMNLNGDYISDALAAQVGGIGIAPGANIGDEYALFEATHGTAPDIAGQDKAN

                  .    :    .    :    .    :    .    :    .    :    .    :
Chain 1:  353 PGSIILSAEMMLRHMGWTEAADLIVKGMEGAINAKTVTYDFERLMDGAKLLKCSEFGDAIIENM
              11111111111111111111111111111111111111111111111111111111111111
Chain 2:  353 PGSIILSAEMMLRHMGWTEAADLIVKGMEGAINAKTVTKDFESLMDGAKLLKCSEFGDAIIENM

Note: positions are from PDB; the numbers between alignments are block index
```

UniProt ID: P08200

PDB ID: 1P8F_A

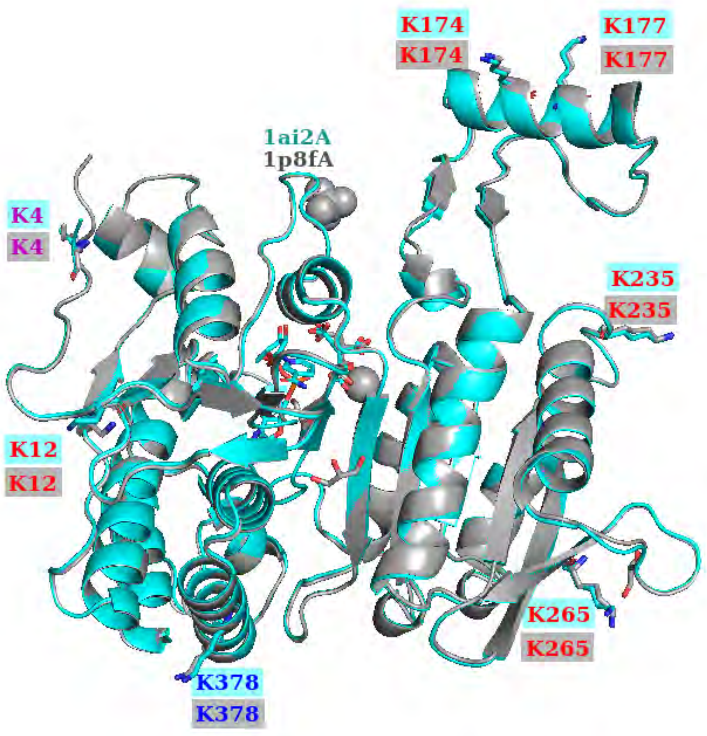


```
Align 1ai2.A.pdb 414 with 1p8f.A.pdb 416
Twists 0 ini-len 408 ini-rmsd 0.35 opt-equ 414 opt-rmsd 0.35 chain-rmsd 0.35 Score 1222.73 align-len 414 gaps 0 (0.00%)
P-value 0.00e+00 Afp-num 51979 Identity 100.00% Similarity 100.00%
Block  0 afp 51 score 1222.73 rmsd  0.35 gap 0 (0.00%)

                  .    :    .    :    .    :    .    :    .    :    .    :    .    :
Chain 1:    3 SKVVVPAQGKKITLQNGKLNVPENPIIPYIEGDGIGVDVTPAMLKVVDAAVEKAYKGERKISWMEIYTGE
              1111111111111111111111111111111111111111111111111111111111111111111111
Chain 2:    3 SKVVVPAQGKKITLQNGKLNVPENPIIPYIEGDGIGVDVTPAMLKVVDAAVEKAYKGERKISWMEIYTGE

                  .    :    .    :    .    :    .    :    .    :    .    :    .    :
Chain 1:   73 KSTQVYGQDVWLPAETLDLIREYRVAIKGPLTTPVGGGIRSLNVALRQELDLYICLRPVRYYQGTPSPVK
              1111111111111111111111111111111111111111111111111111111111111111111111
Chain 2:   73 KSTQVYGQDVWLPAETLDLIREYRVAIKGPLTTPVGGGIRSLNVALRQELDLYICLRPVRYYQGTPSPVK

                  .    :    .    :    .    :    .    :    .    :    .    :    .    :
Chain 1:  143 HPELTDMVIFRENSEDIYAGIEWKADSADAEKVIKFLREEMGVKKIRFPEHCGIGIKPCSEEGTKRLVRA
              1111111111111111111111111111111111111111111111111111111111111111111111
Chain 2:  143 HPELTDMVIFRENSEDIYAGIEWKADSADAEKVIKFLREEMGVKKIRFPEHCGIGIKPCSEEGTKRLVRA

                  .    :    .    :    .    :    .    :    .    :    .    :    .    :
Chain 1:  213 AIEYAIANDRDSVTLVHKGNIMKFTEGAFKDWGYQLAREEFGGELIDGGPWLKVKNPNTGKEIVIKDVIA
              1111111111111111111111111111111111111111111111111111111111111111111111
Chain 2:  213 AIEYAIANDRDSVTLVHKGNIMKFTEGAFKDWGYQLAREEFGGELIDGGPWLKVKNPNTGKEIVIKDVIA

                  .    :    .    :    .    :    .    :    .    :    .    :    .    :
Chain 1:  283 DAFLQQILLRPAEYDVIACMNLNGDYISDALAAQVGGIGIAPGANIGDECALFEATHGTAPKYAGQDKVN
              1111111111111111111111111111111111111111111111111111111111111111111111
Chain 2:  283 DAFLQQILLRPAEYDVIACMNLNGDYISDALAAQVGGIGIAPGANIGDECALFEATHGTAPKYAGQDKVN

                  .    :    .    :    .    :    .    :    .    :    .    :
Chain 1:  353 PGSIILSAEMMLRHMGWTEAADLIVKGMEGAINAKTVTYDFERLMDGAKLLKCSEFGDAIIENM
              11111111111111111111111111111111111111111111111111111111111111
Chain 2:  353 PGSIILSAEMMLRHMGWTEAADLIVKGMEGAINAKTVTYDFERLMDGAKLLKCSEFGDAIIENM

Note: positions are from PDB; the numbers between alignments are block index
```

UniProt ID: P08200
PDB ID: 1PB1_A

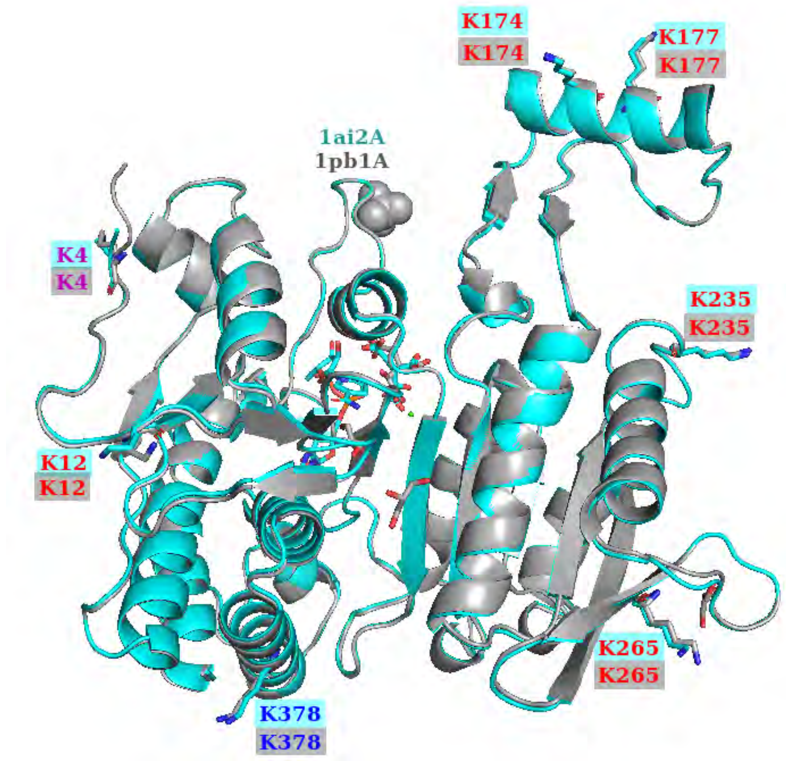

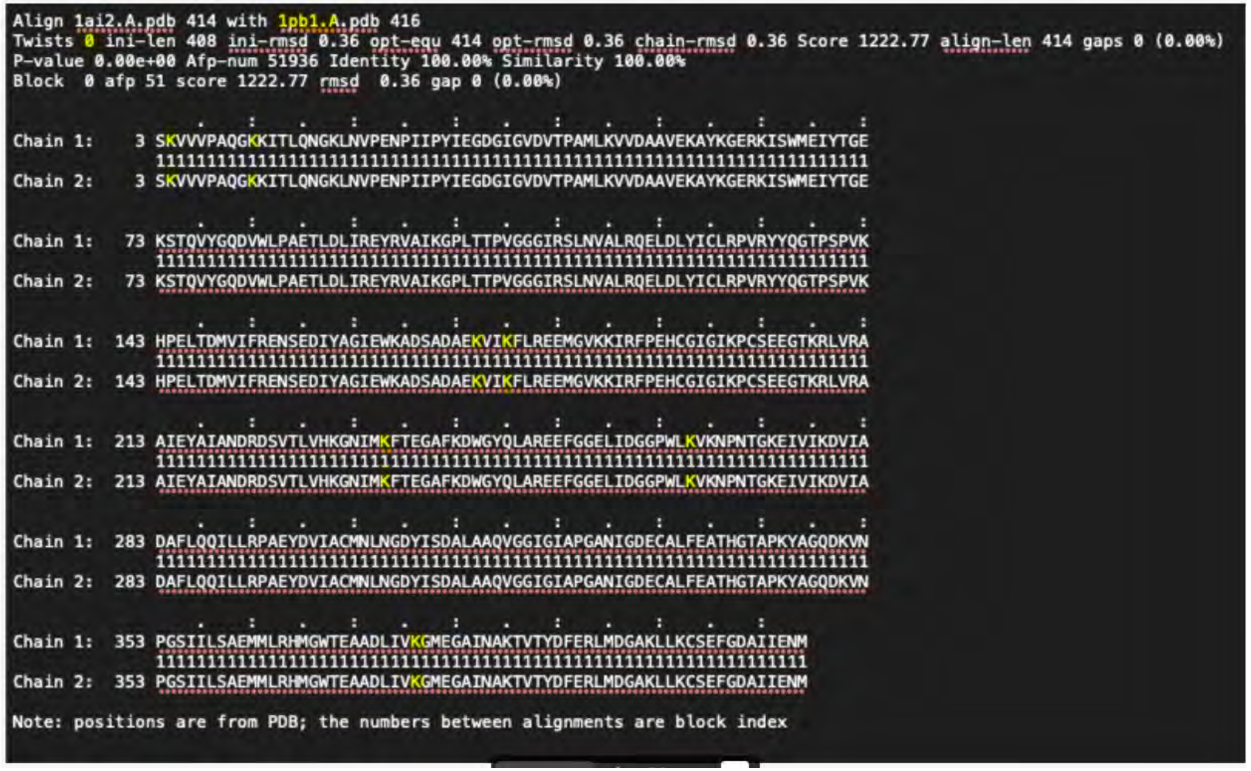

```
Align 1ai2.A.pdb 414 with 1pb1.A.pdb 416
Twists 0 ini-len 408 ini-rmsd 0.36 opt-equ 414 opt-rmsd 0.36 chain-rmsd 0.36 Score 1222.77 align-len 414 gaps 0 (0.00%)
P-value 0.00e+00 Afp-num 51936 Identity 100.00% Similarity 100.00%
Block  0 afp 51 score 1222.77 rmsd  0.36 gap 0 (0.00%)

                    .    :    .    :    .    :    .    :    .    :    .    :    .    :
Chain 1:    3 SKVVVPAQGKKITLQNGKLNVPENPIIPYIEGDGIGVDVTPAMLKVVDAAVEKAYKGERKISWMEIYTGE
              1111111111111111111111111111111111111111111111111111111111111111111111
Chain 2:    3 SKVVVPAQGKKITLQNGKLNVPENPIIPYIEGDGIGVDVTPAMLKVVDAAVEKAYKGERKISWMEIYTGE

                    .    :    .    :    .    :    .    :    .    :    .    :    .    :
Chain 1:   73 KSTQVYGQDVWLPAETLDLIREYRVAIKGPLTTPVGGGIRSLNVALRQELDLYICLRPVRYYQGTPSPVK
              1111111111111111111111111111111111111111111111111111111111111111111111
Chain 2:   73 KSTQVYGQDVWLPAETLDLIREYRVAIKGPLTTPVGGGIRSLNVALRQELDLYICLRPVRYYQGTPSPVK

                    .    :    .    :    .    :    .    :    .    :    .    :    .    :
Chain 1:  143 HPELTDMVIFRENSEDIYAGIEWKADSADAEKVIKFLREEMGVKKIRFPEHCGIGIKPCSEEGTKRLVRA
              1111111111111111111111111111111111111111111111111111111111111111111111
Chain 2:  143 HPELTDMVIFRENSEDIYAGIEWKADSADAEKVIKFLREEMGVKKIRFPEHCGIGIKPCSEEGTKRLVRA

                    .    :    .    :    .    :    .    :    .    :    .    :    .    :
Chain 1:  213 AIEYAIANDRDSVTLVHKGNIMKFTEGAFKDWGYQLAREEFGGELIDGGPWLKVKNPNTGKEIVIKDVIA
              1111111111111111111111111111111111111111111111111111111111111111111111
Chain 2:  213 AIEYAIANDRDSVTLVHKGNIMKFTEGAFKDWGYQLAREEFGGELIDGGPWLKVKNPNTGKEIVIKDVIA

                    .    :    .    :    .    :    .    :    .    :    .    :    .    :
Chain 1:  283 DAFLQQILLRPAEYDVIACMNLNGDYISDALAAQVGGIGIAPGANIGDECALFEATHGTAPKYAGQDKVN
              1111111111111111111111111111111111111111111111111111111111111111111111
Chain 2:  283 DAFLQQILLRPAEYDVIACMNLNGDYISDALAAQVGGIGIAPGANIGDECALFEATHGTAPKYAGQDKVN

                    .    :    .    :    .    :    .    :    .    :    .    :
Chain 1:  353 PGSIILSAEMMLRHMGWTEAADLIVKGMEGAINAKTVTYDFERLMDGAKLLKCSEFGDAIIENM
              1111111111111111111111111111111111111111111111111111111111111111
Chain 2:  353 PGSIILSAEMMLRHMGWTEAADLIVKGMEGAINAKTVTYDFERLMDGAKLLKCSEFGDAIIENM

Note: positions are from PDB; the numbers between alignments are block index
```

UniProt ID: P08200

PDB ID: 1PB3_A

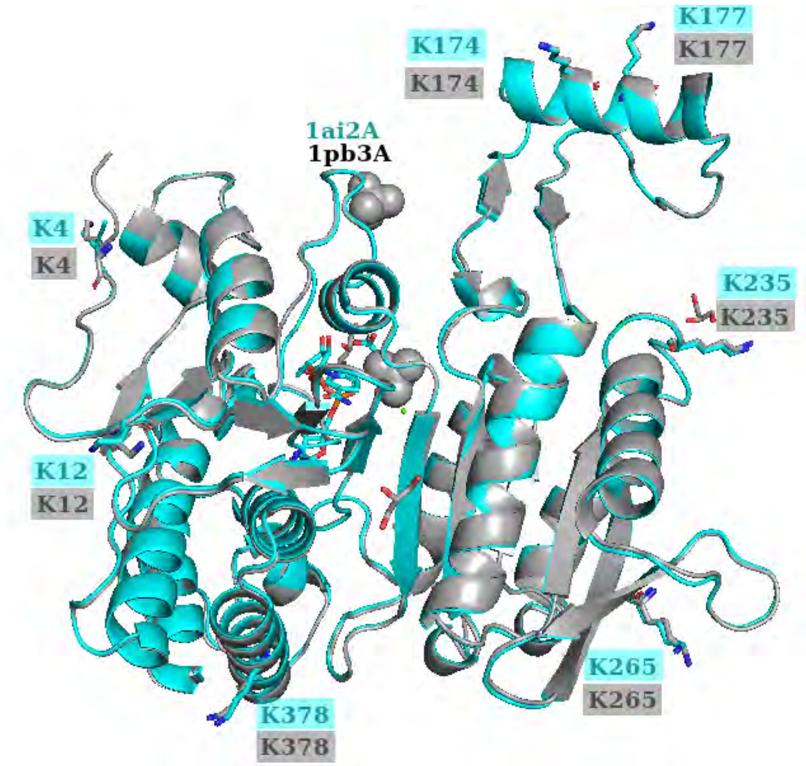


```
Align 1ai2.A.pdb 414 with 1pb3.A.pdb 416
Twists 0 ini-len 408 ini-rmsd 0.32 opt-equ 414 opt-rmsd 0.32 chain-rmsd 0.32 Score 1222.80 align-len 414 gaps 0 (0.00%)
P-value 0.00e+00 Afp-num 51828 Identity 100.00% Similarity 100.00%
Block  0 afp 51 score 1222.80 rmsd  0.32 gap 0 (0.00%)

                    .    :    .    :    .    :    .    :    .    :    .    :    .    :
Chain 1:    3 SKVVVPAQGKKITLQNGKLNVPENPIIPYIEGDGIGVDVTPAMLKVVDAAVEKAYKGERKISWMEIYTGE
              1111111111111111111111111111111111111111111111111111111111111111111111
Chain 2:    3 SKVVVPAQGKKITLQNGKLNVPENPIIPYIEGDGIGVDVTPAMLKVVDAAVEKAYKGERKISWMEIYTGE

                    .    :    .    :    .    :    .    :    .    :    .    :    .    :
Chain 1:   73 KSTQVYGQDVWLPAETLDLIREYRVAIKGPLTTPVGGGIRSLNVALRQELDLYICLRPVRYYQGTPSPVK
              1111111111111111111111111111111111111111111111111111111111111111111111
Chain 2:   73 KSTQVYGQDVWLPAETLDLIREYRVAIKGPLTTPVGGGIRSLNVALRQELDLYICLRPVRYYQGTPSPVK

                    .    :    .    :    .    :    .    :    .    :    .    :    .    :
Chain 1:  143 HPELTDMVIFRENSEDIYAGIEWKADSADAEKVIKFLREEMGVKKIRFPEHCGIGIKPCSEEGTKRLVRA
              1111111111111111111111111111111111111111111111111111111111111111111111
Chain 2:  143 HPELTDMVIFRENSEDIYAGIEWKADSADAEKVIKFLREEMGVKKIRFPEHCGIGIKPCSEEGTKRLVRA

                    .    :    .    :    .    :    .    :    .    :    .    :    .    :
Chain 1:  213 AIEYAIANDRDSVTLVHKGNIMKFTEGAFKDWGYQLAREEFGGELIDGGPWLKVKNPNTGKEIVIKDVIA
              1111111111111111111111111111111111111111111111111111111111111111111111
Chain 2:  213 AIEYAIANDRDSVTLVHKGNIMKFTEGAFKDWGYQLAREEFGGELIDGGPWLKVKNPNTGKEIVIKDVIA

                    .    :    .    :    .    :    .    :    .    :    .    :    .    :
Chain 1:  283 DAFLQQILLRPAEYDVIACMNLNGDYISDALAAQVGGIGIAPGANIGDECALFEATHGTAPKYAGQDKVN
              1111111111111111111111111111111111111111111111111111111111111111111111
Chain 2:  283 DAFLQQILLRPAEYDVIACMNLNGDYISDALAAQVGGIGIAPGANIGDECALFEATHGTAPKYAGQDKVN

                    .    :    .    :    .    :    .    :    .    :    .    :
Chain 1:  353 PGSIILSAEMMLRHMGWTEAADLIVKGMEGAINAKTVTYDFERLMDGAKLLKCSEFGDAIIENM
              1111111111111111111111111111111111111111111111111111111111111111
Chain 2:  353 PGSIILSAEMMLRHMGWTEAADLIVKGMEGAINAKTVTYDFERLMDGAKLLKCSEFGDAIIENM

Note: positions are from PDB; the numbers between alignments are block index
```

UniProt ID: P08200
PDB ID: 1SJS_A

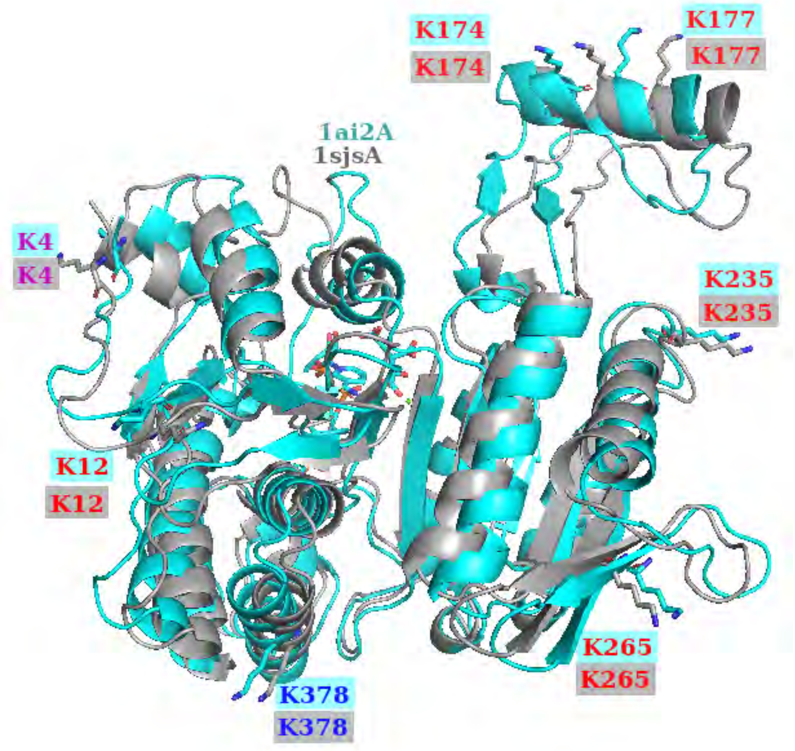


```
Align 1ai2.A.pdb 414 with 1sjs.A.pdb 415
Twists 0 ini-len 408 ini-rmsd 2.35 opt-equ 414 opt-rmsd 2.38 chain-rmsd 2.35 Score 1218.28 align-len 414 gaps 0 (0.00%)
P-value 0.00e+00 Afp-num 51738 Identity 100.00% Similarity 100.00%
Block  0 afp 51 score 1218.28 rmsd  2.35 gap 1 (0.00%)

                  .    :    .    :    .    :    .    :    .    :    .    :    .    :
Chain 1:    3 SKVVVPAQGKKITLQNGKLNVPENPIIPYIEGDGIGVDVTPAMLKVVDAAVEKAYKGERKISWMEIYTGE
              1111111111111111111111111111111111111111111111111111111111111111111111
Chain 2:    3 SKVVVPAQGKKITLQNGKLNVPENPIIPYIEGDGIGVDVTPAMLKVVDAAVEKAYKGERKISWMEIYTGE

                  .    :    .    :    .    :    .    :    .    :    .    :    .    :
Chain 1:   73 KSTQVYGQDVWLPAETLDLIREYRVAIKGPLTTPVGGGIRSLNVALRQELDLYICLRPVRYYQGTPSPVK
              1111111111111111111111111111111111111111111111111111111111111111111111
Chain 2:   73 KSTQVYGQDVWLPAETLDLIREYRVAIKGPLTTPVGGGIRSLNVALRQELDLYICLRPVRYYQGTPSPVK

                  .    :    .    :    .    :    .    :    .    :    .    :    .    :
Chain 1:  143 HPELTDMVIFRENSEDIYAGIEWKADSADAEKVIKFLREEMGVKKIRFPEHCGIGIKPCSEEGTKRLVRA
              1111111111111111111111111111111111111111111111111111111111111111111111
Chain 2:  143 HPELTDMVIFRENSEDIYAGIEWKADSADAEKVIKFLREEMGVKKIRFPEHCGIGIKPCSEEGTKRLVRA

                  .    :    .    :    .    :    .    :    .    :    .    :    .    :
Chain 1:  213 AIEYAIANDRDSVTLVHKGNIMKFTEGAFKDWGYQLAREEFGGELIDGGPWLKVKNPNTGKEIVIKDVIA
              1111111111111111111111111111111111111111111111111111111111111111111111
Chain 2:  213 AIEYAIANDRDSVTLVHKGNIMKFTEGAFKDWGYQLAREEFGGELIDGGPWLKVKNPNTGKEIVIKDVIA

                  .    :    .    :    .    :    .    :    .    :    .    :    .    :
Chain 1:  283 DAFLQQILLRPAEYDVIACMNLNGDYISDALAAQVGGIGIAPGANIGDECALFEATHGTAPKYAGQDKVN
              1111111111111111111111111111111111111111111111111111111111111111111111
Chain 2:  283 DAFLQQILLRPAEYDVIACMNLNGDYISDALAAQVGGIGIAPGANIGDECALFEATHGTAPKYAGQDKVN

                  .    :    .    :    .    :    .    :    .    :    .    :
Chain 1:  353 PGSIILSAEMMLRHMGWTEAADLIVKGMEGAINAKTVTYDFERLMDGAKLLKCSEFGDAIIENM
              1111111111111111111111111111111111111111111111111111111111111111
Chain 2:  353 PGSIILSAEMMLRHMGWTEAADLIVKGMEGAINAKTVTYDFERLMDGAKLLKCSEFGDAIIENM

Note: positions are from PDB; the numbers between alignments are block index
```

UniProt ID: P08200
PDB ID: 3ICD_A

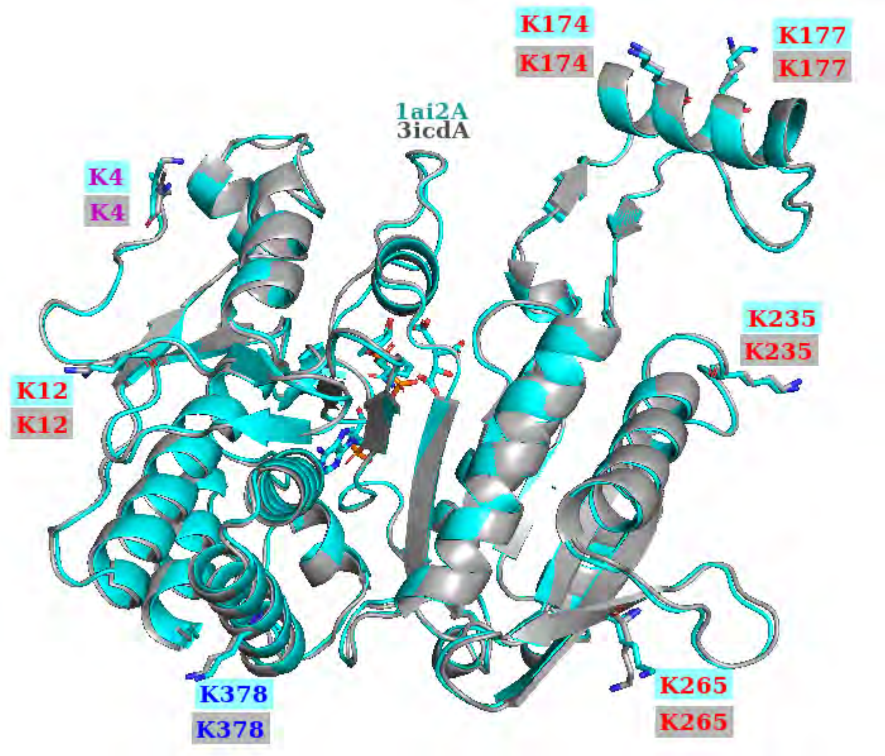


```
Align 1ai2.A.pdb 414 with 3icd.A.pdb 414
Twists 0 ini-len 408 ini-rmsd 0.45 opt-equ 414 opt-rmsd 0.46 chain-rmsd 0.45 Score 1220.74 align-len 414 gaps 0 (0.00%)
P-value 0.00e+00 Afp-num 51539 Identity 100.00% Similarity 100.00%
Block  0 afp 51 score 1220.74 rmsd  0.45 gap 0 (0.00%)

                   .    :    .    :    .    :    .    :    .    :    .    :    .    :
Chain 1:     3 SKVVVPAQGKKITLQNGKLNVPENPIIPYIEGDGIGVDVTPAMLKVVDAAVEKAYKGERKISWMEIYTGE
               1111111111111111111111111111111111111111111111111111111111111111111111
Chain 2:     3 SKVVVPAQGKKITLQNGKLNVPENPIIPYIEGDGIGVDVTPAMLKVVDAAVEKAYKGERKISWMEIYTGE

                   .    :    .    :    .    :    .    :    .    :    .    :    .    :
Chain 1:    73 KSTQVYGQDVWLPAETLDLIREYRVAIKGPLTTPVGGGIRSLNVALRQELDLYICLRPVRYYQGTPSPVK
               1111111111111111111111111111111111111111111111111111111111111111111111
Chain 2:    73 KSTQVYGQDVWLPAETLDLIREYRVAIKGPLTTPVGGGIRSLNVALRQELDLYICLRPVRYYQGTPSPVK

                   .    :    .    :    .    :    .    :    .    :    .    :    .    :
Chain 1:   143 HPELTDMVIFRENSEDIYAGIEWKADSADAEKVIKFLREEMGVKKIRFPEHCGIGIKPCSEEGTKRLVRA
               1111111111111111111111111111111111111111111111111111111111111111111111
Chain 2:   143 HPELTDMVIFRENSEDIYAGIEWKADSADAEKVIKFLREEMGVKKIRFPEHCGIGIKPCSEEGTKRLVRA

                   .    :    .    :    .    :    .    :    .    :    .    :    .    :
Chain 1:   213 AIEYAIANDRDSVTLVHKGNIMKFTEGAFKDWGYQLAREEFGGELIDGGPWLKVKNPNTGKEIVIKDVIA
               1111111111111111111111111111111111111111111111111111111111111111111111
Chain 2:   213 AIEYAIANDRDSVTLVHKGNIMKFTEGAFKDWGYQLAREEFGGELIDGGPWLKVKNPNTGKEIVIKDVIA

                   .    :    .    :    .    :    .    :    .    :    .    :    .    :
Chain 1:   283 DAFLQQILLRPAEYDVIACMNLNGDYISDALAAQVGGIGIAPGANIGDECALFEATHGTAPKYAGQDKVN
               1111111111111111111111111111111111111111111111111111111111111111111111
Chain 2:   283 DAFLQQILLRPAEYDVIACMNLNGDYISDALAAQVGGIGIAPGANIGDECALFEATHGTAPKYAGQDKVN

                   .    :    .    :    .    :    .    :    .    :    .    :
Chain 1:   353 PGSIILSAEMMLRHMGWTEAADLIVKGMEGAINAKTVTYDFERLMDGAKLLKCSEFGDAIIENM
               1111111111111111111111111111111111111111111111111111111111111111
Chain 2:   353 PGSIILSAEMMLRHMGWTEAADLIVKGMEGAINAKTVTYDFERLMDGAKLLKCSEFGDAIIENM

Note: positions are from PDB; the numbers between alignments are block index
```

UniProt ID: P08200
PDB ID: 4AJ3_A

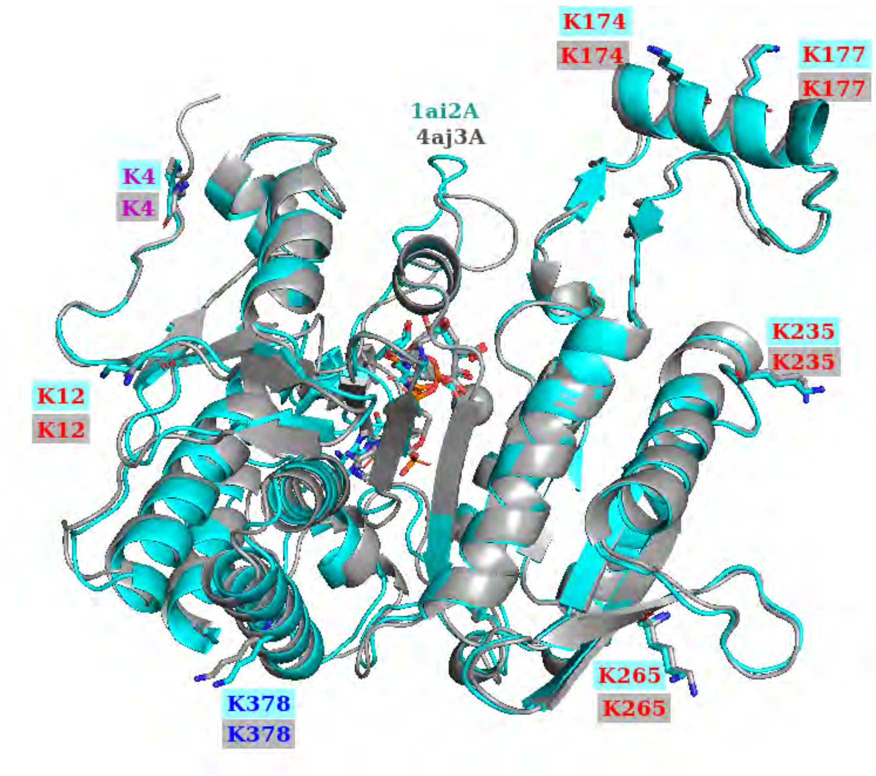


```
Align 1ai2.A.pdb 414 with 4aj3.A.pdb 416
Twists 0 ini-len 408 ini-rmsd 1.18 opt-equ 414 opt-rmsd 1.19 chain-rmsd 1.18 Score 1217.74 align-len 414 gaps 0 (0.00%)
P-value 0.00e+00 Afp-num 51837 Identity 100.00% Similarity 100.00%
Block  0 afp 51 score 1217.74 rmsd  1.18 gap 2 (0.00%)

                  .    :    .    :    .    :    .    :    .    :    .    :    .    :
Chain 1:    3 SKVVVPAQGKKITLQNGKLNVPENPIIPYIEGDGIGVDVTPAMLKVVDAAVEKAYKGERKISWMEIYTGE
              1111111111111111111111111111111111111111111111111111111111111111111111
Chain 2:    3 SKVVVPAQGKKITLQNGKLNVPENPIIPYIEGDGIGVDVTPAMLKVVDAAVEKAYKGERKISWMEIYTGE

                  .    :    .    :    .    :    .    :    .    :    .    :    .    :
Chain 1:   73 KSTQVYGQDVWLPAETLDLIREYRVAIKGPLTTPVGGGIRSLNVALRQELDLYICLRPVRYYQGTPSPVK
              1111111111111111111111111111111111111111111111111111111111111111111111
Chain 2:   73 KSTQVYGQDVWLPAETLDLIREYRVAIKGPLTTPVGGGIRSLNVALRQELDLYICLRPVRYYQGTPSPVK

                  .    :    .    :    .    :    .    :    .    :    .    :    .    :
Chain 1:  143 HPELTDMVIFRENSEDIYAGIEWKADSADAEKVIKFLREEMGVKKIRFPEHCGIGIKPCSEEGTKRLVRA
              1111111111111111111111111111111111111111111111111111111111111111111111
Chain 2:  143 HPELTDMVIFRENSEDIYAGIEWKADSADAEKVIKFLREEMGVKKIRFPEHCGIGIKPCSEEGTKRLVRA

                  .    :    .    :    .    :    .    :    .    :    .    :    .    :
Chain 1:  213 AIEYAIANDRDSVTLVHKGNIMKFTEGAFKDWGYQLAREEFGGELIDGGPWLKVKNPNTGKEIVIKDVIA
              1111111111111111111111111111111111111111111111111111111111111111111111
Chain 2:  213 AIEYAIANDRDSVTLVHKGNIMKFTEGAFKDWGYQLAREEFGGELIDGGPWLKVKNPNTGKEIVIKDVIA

                  .    :    .    :    .    :    .    :    .    :    .    :    .    :
Chain 1:  283 DAFLQQILLRPAEYDVIACMNLNGDYISDALAAQVGGIGIAPGANIGDECALFEATHGTAPKYAGQDKVN
              1111111111111111111111111111111111111111111111111111111111111111111111
Chain 2:  283 DAFLQQILLRPAEYDVIACMNLNGDYISDALAAQVGGIGIAPGANIGDECALFEATHGTAPKYAGQDKVN

                  .    :    .    :    .    :    .    :    .    :    .    :
Chain 1:  353 PGSIILSAEMMLRHMGWTEAADLIVKGMEGAINAKTVTYDFERLMDGAKLLKCSEFGDAIIENM
              1111111111111111111111111111111111111111111111111111111111111111
Chain 2:  353 PGSIILSAEMMLRHMGWTEAADLIVKGMEGAINAKTVTYDFERLMDGAKLLKCSEFGDAIIENM

Note: positions are from PDB; the numbers between [illegible] dex
```

UniProt ID: P08200
PDB ID: 3LCB_D

```
Align 1ai2.A.pdb 414 with 3lcb.D.pdb 415
Twists 0 ini-len 408 ini-rmsd 2.50 opt-equ 414 opt-rmsd 2.51 chain-rmsd 2.50 Score 1214.46 align-len 415 gaps 1 (0.24%)
P-value 0.00e+00 Afp-num 51559 Identity 98.31% Similarity 98.31%
Block  0 afp 51 score 1214.46 rmsd  2.50 gap 4 (0.01%)

                  .    :    .    :    .    :    .    :    .    :    .    :    .    :
Chain 1:    3 SKVVVPAQ-GKKITLQNGKLNVPENPIIPYIEGDGIGVDVTPAMLKVVDAAVEKAYKGERKISWMEIYTG
              11111111 1111111111111111111111111111111111111111111111111111111111111
Chain 2:    2 ESKVVVPAQGKKITLQNGKLNVPENPIIPYIEGDGIGVDVTPAMLKVVDAAVEKAYKGERKISWMEIYTG

                  .    :    .    :    .    :    .    :    .    :    .    :    .    :
Chain 1:   72 EKSTQVYGQDVWLPAETLDLIREYRVAIKGPLTTPVGGGIRSLNVALRQELDLYICLRPVRYYQGTPSPV
              1111111111111111111111111111111111111111111111111111111111111111111111
Chain 2:   72 EKSTQVYGQDVWLPAETLDLIREYRVAIKGPLTTPVGGGIRSLNVALRQELDLYICLRPVRYYQGTPSPV

                  .    :    .    :    .    :    .    :    .    :    .    :    .    :
Chain 1:  142 KHPELTDMVIFRENSEDIYAGIEWKADSADAEKVIKFLREEMGVKKIRFPEHCGIGIKPCSEEGTKRLVR
              1111111111111111111111111111111111111111111111111111111111111111111111
Chain 2:  142 KHPELTDMVIFRENSEDIYAGIEWKADSADAEKVIKFLREEMGVKKIRFPEHCGIGIKPCSEEGTKRLVR

                  .    :    .    :    .    :    .    :    .    :    .    :    .    :
Chain 1:  212 AAIEYAIANDRDSVTLVHKGNIMKFTEGAFKDWGYQLAREEFGGELIDGGPWLKVKNPNTGKEIVIKDVI
              1111111111111111111111111111111111111111111111111111111111111111111111
Chain 2:  212 AAIEYAIANDRDSVTLVHKGNIMKFTEGAFKDWGYQLAREEFGGELIDGGPWLKVKNPNTGKEIVIKDVI

                  .    :    .    :    .    :    .    :    .    :    .    :    .    :
Chain 1:  282 ADAFLQQILLRPAEYDVIACMNLNGDYISDALAAQVGGIGIAPGANIGDECALFEATHGTAPKYAGQDKV
              1111111111111111111111111111111111111111111111111111111111111111111111
Chain 2:  282 ADAFLQQILLRPAEYDVIACMNLNGDYISDALAAQVGGIGIAPGANIGDECALFEATHGTAPKYAGQDKV

                  .    :    .    :    .    :    .    :    .    :    .    :    .
Chain 1:  352 NPGSIILSAEMMLRHMGWTEAADLIVKGMEGAINAKTVTYDFERLMDGAKLLKCSEFGDAIIENM
              11111111111111111111111111111111111111111111111111111111111111111
Chain 2:  352 NPGSIILSAEMMLRHMGWTEAADLIVKGMEGAINAKTVTYDFERLMDGAKLLKCSEFGDAIIENM
```

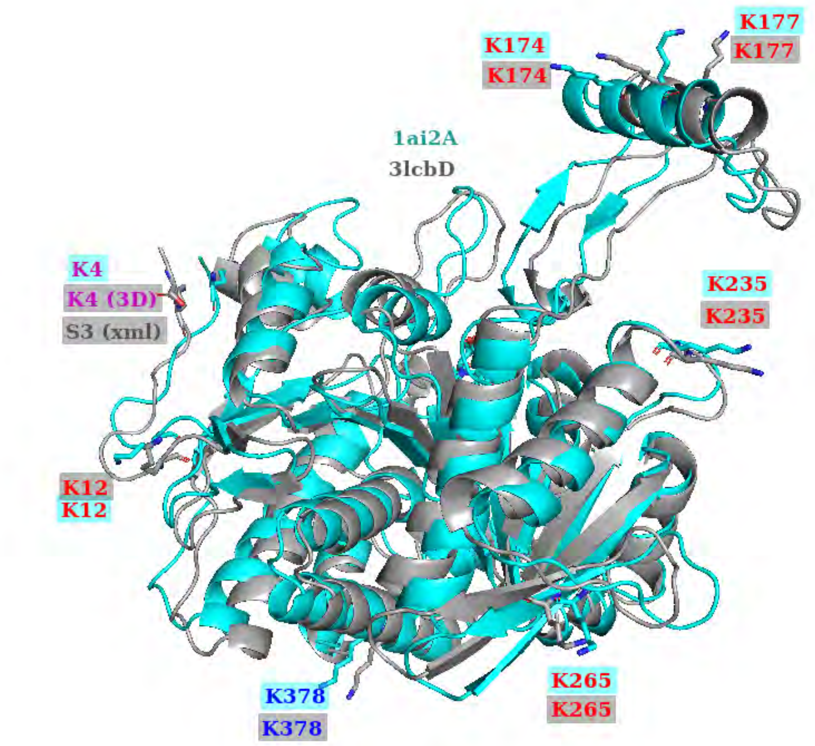

UniProt ID: P08200
PDB ID: 4P69_D

```
Align 1ai2.A.pdb 414 with 4p69.D.pdb 415
Twists 0 ini-len 408 ini-rmsd 2.57 opt-equ 414 opt-rmsd 2.57 chain-rmsd 2.57 Score 1207.46 align-len 415 gaps 1 (0.24%)
P-value 0.00e+00 Afp-num 53453 Identity 98.31% Similarity 98.31%
Block  0 afp 51 score 1207.46 rmsd  2.57 gap 5 (0.01%)

                       .         :         .         :         .         :         .         :
Chain 1:    3 SKVVVPAQ-GKKITLQNGKLNVPENPIIPYIEGDGIGVDVTPAMLKVVDAAVEKAYKGERKISWMEIYTG
              11111111 1111111111111111111111111111111111111111111111111111111111111
Chain 2:    2 ESKVVVPAQGKKITLQNGKLNVPENPIIPYIEGDGIGVDVTPAMLKVVDAAVEKAYKGERKISWMEIYTG

                       .         :         .         :         .         :         .         :
Chain 1:   72 EKSTQVYGQDVWLPAETLDLIREYRVAIKGPLTTPVGGGIRSLNVALRQELDLYICLRPVRYYQGTPSPV
              1111111111111111111111111111111111111111111111111111111111111111111111
Chain 2:   72 EKSTQVYGQDVWLPAETLDLIREYRVAIKGPLTTPVGGGIRSLNVALRQELDLYICLRPVRYYQGTPSPV

                       .         :         .         :         .         :         .         :
Chain 1:  142 KHPELTDMVIFRENSEDIYAGIEWKADSADAEKVIKFLREEMGVKKIRFPEHCGIGIKPCSEEGTKRLVR
              1111111111111111111111111111111111111111111111111111111111111111111111
Chain 2:  142 KHPELTDMVIFRENSEDIYAGIEWKADSADAEKVIKFLREEMGVKKIRFPEHCGIGIKPCSEEGTKRLVR

                       .         :         .         :         .         :         .         :
Chain 1:  212 AAIEYAIANDRDSVTLVHKGNIMKFTEGAFKDWGYQLAREEFGGELIDGGPWLKVKNPNTGKEIVIKDVI
              1111111111111111111111111111111111111111111111111111111111111111111111
Chain 2:  212 AAIEYAIANDRDSVTLVHKGNIMKFTEGAFKDWGYQLAREEFGGELIDGGPWLKVKNPNTGKEIVIKDVI

                       .         :         .         :         .         :         .         :
Chain 1:  282 ADAFLQQILLRPAEYDVIACMNLNGDYISDALAAQVGGIGIAPGANIGDECALFEATHGTAPKYAGQDKV
              1111111111111111111111111111111111111111111111111111111111111111111111
Chain 2:  282 ADAFLQQILLRPAEYDVIACMNLNGDYISDALAAQVGGIGIAPGANIGDECALFEATHGTAPKYAGQDKV

                       .         :         .         :         .         :         .
Chain 1:  352 NPGSIILSAEMMLRHMGWTEAADLIVKGMEGAINAKTVTYDFERLMDGAKLLKCSEFGDAIIENM
              11111111111111111111111111111111111111111111111111111111111111111
Chain 2:  352 NPGSIILSAEMMLRHMGWTEAADLIVKGMEGAINAKTVTYDFERLMDGAKLLKCSEFGDAIIENM
```

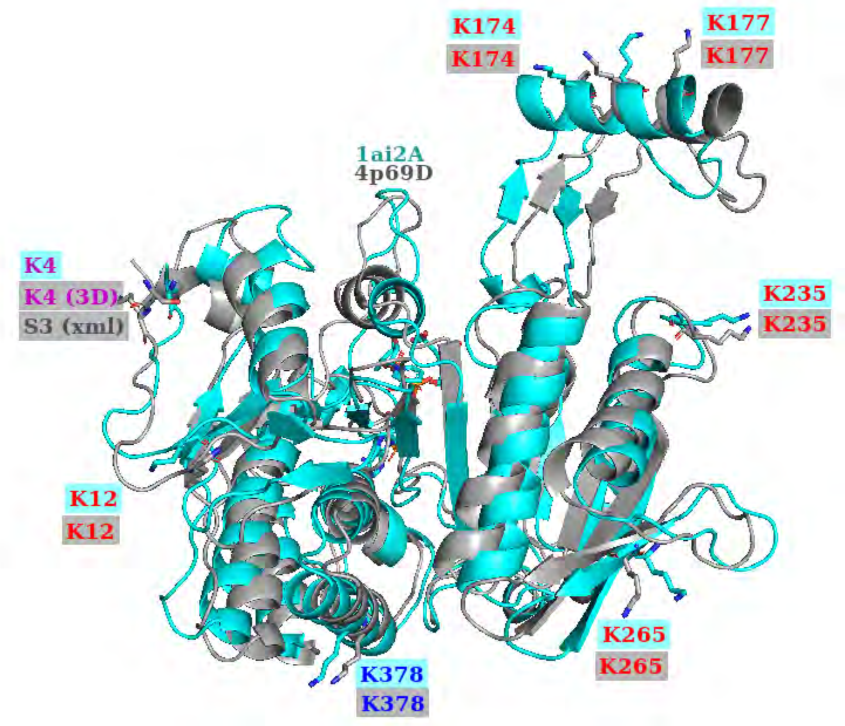

UniProt ID: P08200
PDB ID: 4AJA_A

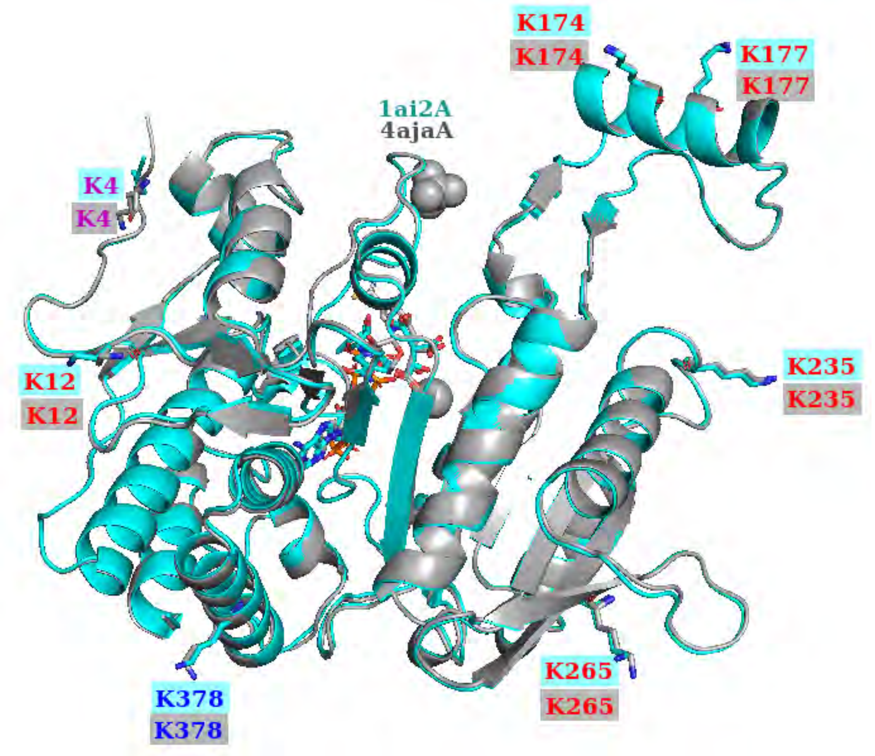


```
Align 1ai2.A.pdb 414 with 4aja.A.pdb 415
Twists 0 ini-len 408 ini-rmsd 0.40 opt-equ 414 opt-rmsd 0.40 chain-rmsd 0.40 Score 1222.16 align-len 414 gaps 0 (0.00%)
P-value 0.00e+00 Afp-num 51851 Identity 100.00% Similarity 100.00%
Block  0 afp 51 score 1222.16 rmsd  0.40 gap 0 (0.00%)

                    .    :    .    :    .    :    .    :    .    :    .    :    .    :
Chain 1:    3 SKVVVPAQGKKITLQNGKLNVPENPIIPYIEGDGIGVDVTPAMLKVVDAAVEKAYKGERKISWMEIYTGE
              1111111111111111111111111111111111111111111111111111111111111111111111
Chain 2:    3 SKVVVPAQGKKITLQNGKLNVPENPIIPYIEGDGIGVDVTPAMLKVVDAAVEKAYKGERKISWMEIYTGE

                    .    :    .    :    .    :    .    :    .    :    .    :    .    :
Chain 1:   73 KSTQVYGQDVWLPAETLDLIREYRVAIKGPLTTPVGGGIRSLNVALRQELDLYICLRPVRYYQGTPSPVK
              1111111111111111111111111111111111111111111111111111111111111111111111
Chain 2:   73 KSTQVYGQDVWLPAETLDLIREYRVAIKGPLTTPVGGGIRSLNVALRQELDLYICLRPVRYYQGTPSPVK

                    .    :    .    :    .    :    .    :    .    :    .    :    .    :
Chain 1:  143 HPELTDMVIFRENSEDIYAGIEWKADSADAEKVIKFLREEMGVKKIRFPEHCGIGIKPCSEEGTKRLVRA
              1111111111111111111111111111111111111111111111111111111111111111111111
Chain 2:  143 HPELTDMVIFRENSEDIYAGIEWKADSADAEKVIKFLREEMGVKKIRFPEHCGIGIKPCSEEGTKRLVRA

                    .    :    .    :    .    :    .    :    .    :    .    :    .    :
Chain 1:  213 AIEYAIANDRDSVTLVHKGNIMKFTEGAFKDWGYQLAREEFGGELIDGGPWLKVKNPNTGKEIVIKDVIA
              1111111111111111111111111111111111111111111111111111111111111111111111
Chain 2:  213 AIEYAIANDRDSVTLVHKGNIMKFTEGAFKDWGYQLAREEFGGELIDGGPWLKVKNPNTGKEIVIKDVIA

                    .    :    .    :    .    :    .    :    .    :    .    :    .    :
Chain 1:  283 DAFLQQILLRPAEYDVIACMNLNGDYISDALAAQVGGIGIAPGANIGDECALFEATHGTAPKYAGQDKVN
              1111111111111111111111111111111111111111111111111111111111111111111111
Chain 2:  283 DAFLQQILLRPAEYDVIACMNLNGDYISDALAAQVGGIGIAPGANIGDECALFEATHGTAPKYAGQDKVN

                    .    :    .    :    .    :    .    :    .    :    .    :
Chain 1:  353 PGSIILSAEMMLRHMGWTEAADLIVKGMEGAINAKTVTYDFERLMDGAKLLKCSEFGDAIIENM
              11111111111111111111111111111111111111111111111111111111111111
Chain 2:  353 PGSIILSAEMMLRHMGWTEAADLIVKGMEGAINAKTVTYDFERLMDGAKLLKCSEFGDAIIENM

Note: positions are from PDB; the numbers between alignments are block index
```

UniProt ID: P08200

PDB ID: 4AJB_A

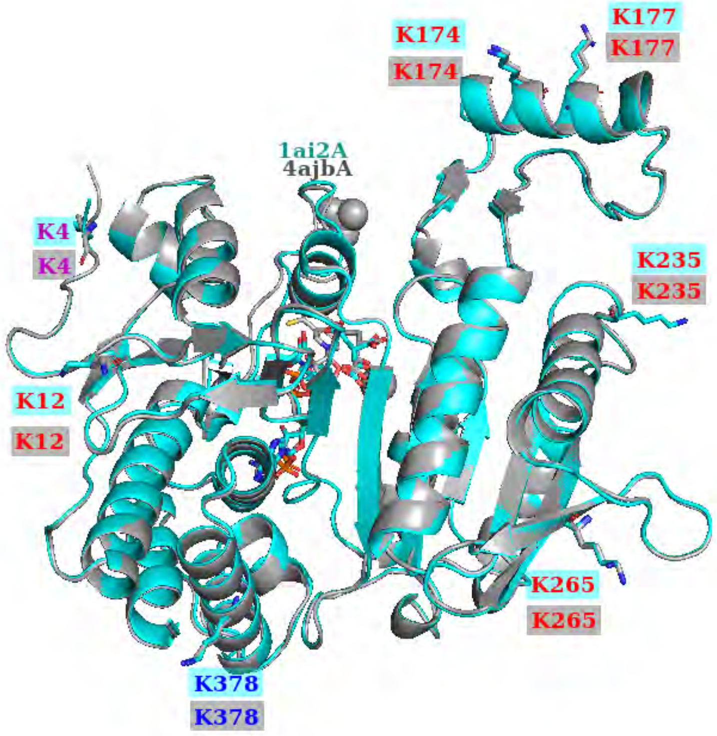


```
Align 1ai2.A.pdb 414 with 4ajb.A.pdb 415
Twists 0 ini-len 408 ini-rmsd 0.42 opt-equ 414 opt-rmsd 0.42 chain-rmsd 0.42 Score 1221.86 align-len 414 gaps 0 (0.00%)
P-value 0.00e+00 Afp-num 51956 Identity 99.76% Similarity 99.76%
Block  0 afp 51 score 1221.86 rmsd  0.42 gap 0 (0.00%)

                    .    :    .    :    .    :    .    :    .    :    .    :    .    :
Chain 1:    3 SKVVVPAQGKKITLQNGKLNVPENPIIPYIEGDGIGVDVTPAMLKVVDAAVEKAYKGERKISWMEIYTGE
              1111111111111111111111111111111111111111111111111111111111111111111111
Chain 2:    3 SKVVVPAQGKKITLQNGKLNVPENPIIPYIEGDGIGVDVTPAMLKVVDAAVEKAYKGERKISWMEIYTGE

                    .    :    .    :    .    :    .    :    .    :    .    :    .    :
Chain 1:   73 KSTQVYGQDVWLPAETLDLIREYRVAIKGPLTTPVGGGIRSLNVALRQELDLYICLRPVRYYQGTPSPVK
              1111111111111111111111111111111111111111111111111111111111111111111111
Chain 2:   73 KSTQVYGQDVWLPAETLDLIREYRVAIMGPLTTPVGGGIRSLNVALRQELDLYICLRPVRYYQGTPSPVK

                    .    :    .    :    .    :    .    :    .    :    .    :    .    :
Chain 1:  143 HPELTDMVIFRENSEDIYAGIEWKADSADAEKVIKFLREEMGVKKIRFPEHCGIGIKPCSEEGTKRLVRA
              1111111111111111111111111111111111111111111111111111111111111111111111
Chain 2:  143 HPELTDMVIFRENSEDIYAGIEWKADSADAEKVIKFLREEMGVKKIRFPEHCGIGIKPCSEEGTKRLVRA

                    .    :    .    :    .    :    .    :    .    :    .    :    .    :
Chain 1:  213 AIEYAIANDRDSVTLVHKGNIMKFTEGAFKDWGYQLAREEFGGELIDGGPWLKVKNPNTGKEIVIKDVIA
              1111111111111111111111111111111111111111111111111111111111111111111111
Chain 2:  213 AIEYAIANDRDSVTLVHKGNIMKFTEGAFKDWGYQLAREEFGGELIDGGPWLKVKNPNTGKEIVIKDVIA

                    .    :    .    :    .    :    .    :    .    :    .    :    .    :
Chain 1:  283 DAFLQQILLRPAEYDVIACMNLNGDYISDALAAQVGGIGIAPGANIGDECALFEATHGTAPKYAGQDKVN
              1111111111111111111111111111111111111111111111111111111111111111111111
Chain 2:  283 DAFLQQILLRPAEYDVIACMNLNGDYISDALAAQVGGIGIAPGANIGDECALFEATHGTAPKYAGQDKVN

                    .    :    .    :    .    :    .    :    .    :    .    :
Chain 1:  353 PGSIILSAEMMLRHMGWTEAADLIVKGMEGAINAKTVTYDFERLMDGAKLLKCSEFGDAIIENM
              111111111111111111111111111111111111111111111111111111111111111
Chain 2:  353 PGSIILSAEMMLRHMGWTEAADLIVKGMEGAINAKTVTYDFERLMDGAKLLKCSEFGDAIIENM

Note: positions are from PDB; the numbers between alignments are block index
```

UniProt ID: P08200

PDB ID: 4AJC_A

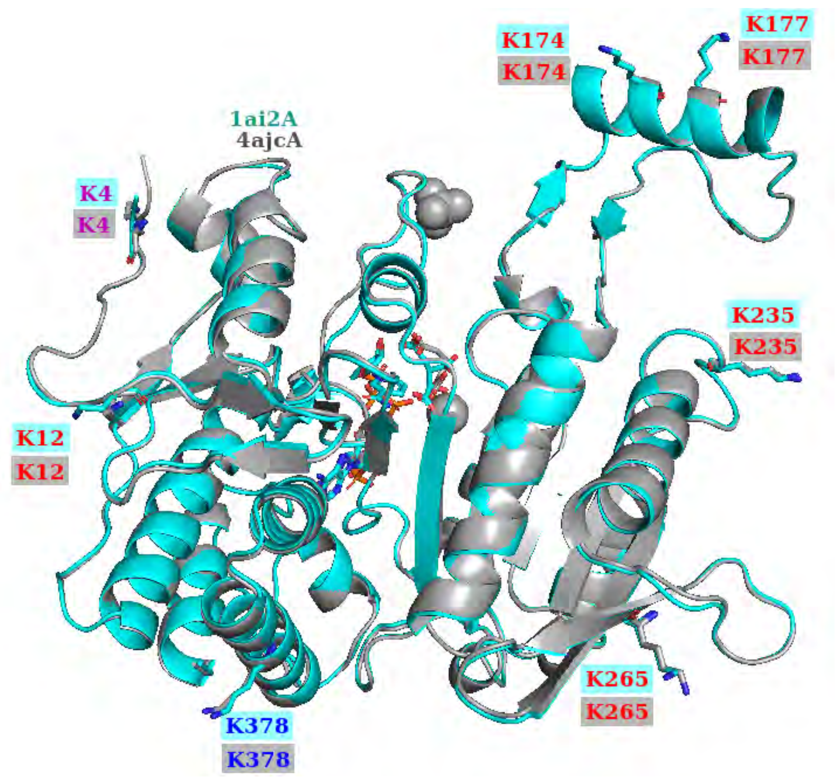


```
Align 1ai2.A.pdb 414 with 4ajc.A.pdb 415
Twists 0 ini-len 408 ini-rmsd 0.35 opt-equ 414 opt-rmsd 0.35 chain-rmsd 0.35 Score 1222.49 align-len 414 gaps 0 (0.00%)
P-value 0.00e+00 Afp-num 51815 Identity 99.76% Similarity 99.76%
Block  0 afp 51 score 1222.49 rmsd  0.35 gap 0 (0.00%)

                        .    :    .    :    .    :    .    :    .    :    .    :    .    :
Chain 1:    3 SKVVVPAQGKKITLQNGKLNVPENPIIPYIEGDGIGVDVTPAMLKVVDAAVEKAYKGERKISWMEIYTGE
              1111111111111111111111111111111111111111111111111111111111111111111111
Chain 2:    3 SKVVVPAQGKKITLQNGKLNVPENPIIPYIEGDGIGVDVTPAMLKVVDAAVEKAYKGERKISWMEIYTGE

                        .    :    .    :    .    :    .    :    .    :    .    :    .    :
Chain 1:   73 KSTQVYGQDVWLPAETLDLIREYRVAIKGPLTTPVGGGIRSLNVALRQELDLYICLRPVRYYQGTPSPVK
              1111111111111111111111111111111111111111111111111111111111111111111111
Chain 2:   73 KSTQVYGQDVWLPAETLDLIREYRVAIMGPLTTPVGGGIRSLNVALRQELDLYICLRPVRYYQGTPSPVK

                        .    :    .    :    .    :    .    :    .    :    .    :    .    :
Chain 1:  143 HPELTDMVIFRENSEDIYAGIEWKADSADAEKVIKFLREEMGVKKIRFPEHCGIGIKPCSEEGTKRLVRA
              1111111111111111111111111111111111111111111111111111111111111111111111
Chain 2:  143 HPELTDMVIFRENSEDIYAGIEWKADSADAEKVIKFLREEMGVKKIRFPEHCGIGIKPCSEEGTKRLVRA

                        .    :    .    :    .    :    .    :    .    :    .    :    .    :
Chain 1:  213 AIEYAIANDRDSVTLVHKGNIMKFTEGAFKDWGYQLAREEFGGELIDGGPWLKVKNPNTGKEIVIKDVIA
              1111111111111111111111111111111111111111111111111111111111111111111111
Chain 2:  213 AIEYAIANDRDSVTLVHKGNIMKFTEGAFKDWGYQLAREEFGGELIDGGPWLKVKNPNTGKEIVIKDVIA

                        .    :    .    :    .    :    .    :    .    :    .    :    .    :
Chain 1:  283 DAFLQQILLRPAEYDVIACMNLNGDYISDALAAQVGGIGIAPGANIGDECALFEATHGTAPKYAGQDKVN
              1111111111111111111111111111111111111111111111111111111111111111111111
Chain 2:  283 DAFLQQILLRPAEYDVIACMNLNGDYISDALAAQVGGIGIAPGANIGDECALFEATHGTAPKYAGQDKVN

                        .    :    .    :    .    :    .    :    .    :    .    :
Chain 1:  353 PGSIILSAEMMLRHMGWTEAADLIVKGMEGAINAKTVTYDFERLMDGAKLLKCSEFGDAIIENM
              1111111111111111111111111111111111111111111111111111111111111111
Chain 2:  353 PGSIILSAEMMLRHMGWTEAADLIVKGMEGAINAKTVTYDFERLMDGAKLLKCSEFGDAIIENM

Note: positions are from PDB; the numbers between alignments are block index
```

UniProt ID: P08200

PDB ID: 4AJR_A

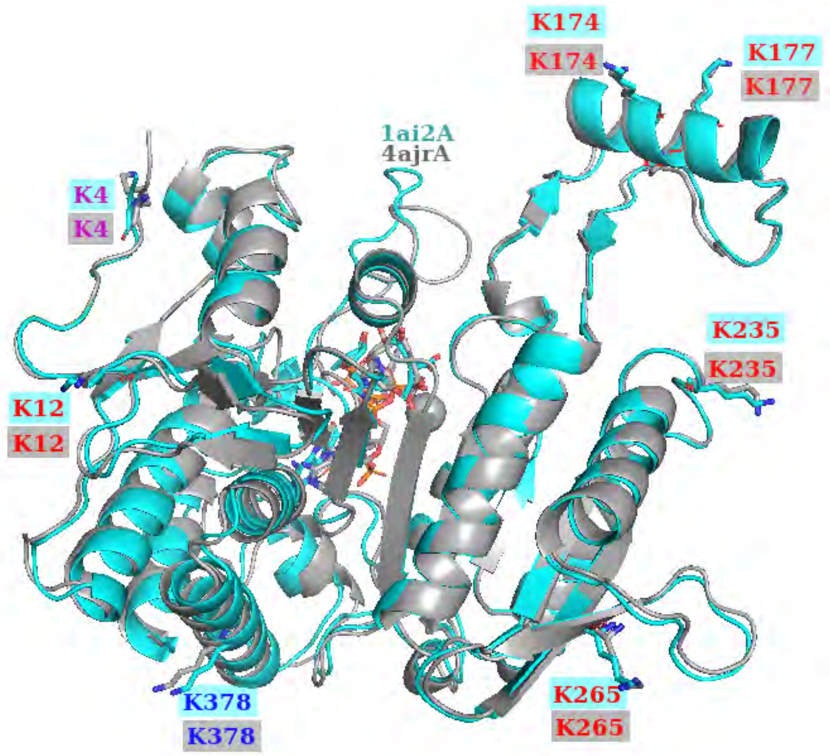


```
Align 1ai2.A.pdb 414 with 4ajr.A.pdb 415
Twists 0 ini-len 408 ini-rmsd 1.19 opt-equ 414 opt-rmsd 1.20 chain-rmsd 1.19 Score 1214.83 align-len 414 gaps 0 (0.00%)
P-value 0.00e+00 Afp-num 51781 Identity 99.76% Similarity 99.76%
Block  0 afp 51 score 1214.83 rmsd  1.19 gap 1 (0.00%)

                    .    :    .    :    .    :    .    :    .    :    .    :    .    :
Chain 1:    3 SKVVVPAQGKKITLQNGKLNVPENPIIPYIEGDGIGVDVTPAMLKVVDAAVEKAYKGERKISWMEIYTGE
              1111111111111111111111111111111111111111111111111111111111111111111111
Chain 2:    3 SKVVVPAQGKKITLQNGKLNVPENPIIPYIEGDGIGVDVTPAMLKVVDAAVEKAYKGERKISWMEIYTGE

                    .    :    .    :    .    :    .    :    .    :    .    :    .    :
Chain 1:   73 KSTQVYGQDVWLPAETLDLIREYRVAIKGPLTTPVGGGIRSLNVALRQELDLYICLRPVRYYQGTPSPVK
              1111111111111111111111111111111111111111111111111111111111111111111111
Chain 2:   73 KSTQVYGQDVWLPAETLDLIREYRVAIMGPLTTPVGGGIRSLNVALRQELDLYICLRPVRYYQGTPSPVK

                    .    :    .    :    .    :    .    :    .    :    .    :    .    :
Chain 1:  143 HPELTDMVIFRENSEDIYAGIEWKADSADAEKVIKFLREEMGVKKIRFPEHCGIGIKPCSEEGTKRLVRA
              1111111111111111111111111111111111111111111111111111111111111111111111
Chain 2:  143 HPELTDMVIFRENSEDIYAGIEWKADSADAEKVIKFLREEMGVKKIRFPEHCGIGIKPCSEEGTKRLVRA

                    .    :    .    :    .    :    .    :    .    :    .    :    .    :
Chain 1:  213 AIEYAIANDRDSVTLVHKGNIMKFTEGAFKDWGYQLAREEFGGELIDGGPWLKVKNPNTGKEIVIKDVIA
              1111111111111111111111111111111111111111111111111111111111111111111111
Chain 2:  213 AIEYAIANDRDSVTLVHKGNIMKFTEGAFKDWGYQLAREEFGGELIDGGPWLKVKNPNTGKEIVIKDVIA

                    .    :    .    :    .    :    .    :    .    :    .    :    .    :
Chain 1:  283 DAFLQQILLRPAEYDVIACMNLNGDYISDALAAQVGGIGIAPGANIGDECALFEATHGTAPKYAGQDKVN
              1111111111111111111111111111111111111111111111111111111111111111111111
Chain 2:  283 DAFLQQILLRPAEYDVIACMNLNGDYISDALAAQVGGIGIAPGANIGDECALFEATHGTAPKYAGQDKVN

                    .    :    .    :    .    :    .    :    .    :    .    :
Chain 1:  353 PGSIILSAEMMLRHMGWTEAADLIVKGMEGAINAKTVTYDFERLMDGAKLLKCSEFGDAIIENM
              1111111111111111111111111111111111111111111111111111111111111111
Chain 2:  353 PGSIILSAEMMLRHMGWTEAADLIVKGMEGAINAKTVTYDFERLMDGAKLLKCSEFGDAIIENM

Note: positions are from PDB; the numbers between alignments are block index
```

UniProt ID: P08200
PDB ID: 4AJS_A

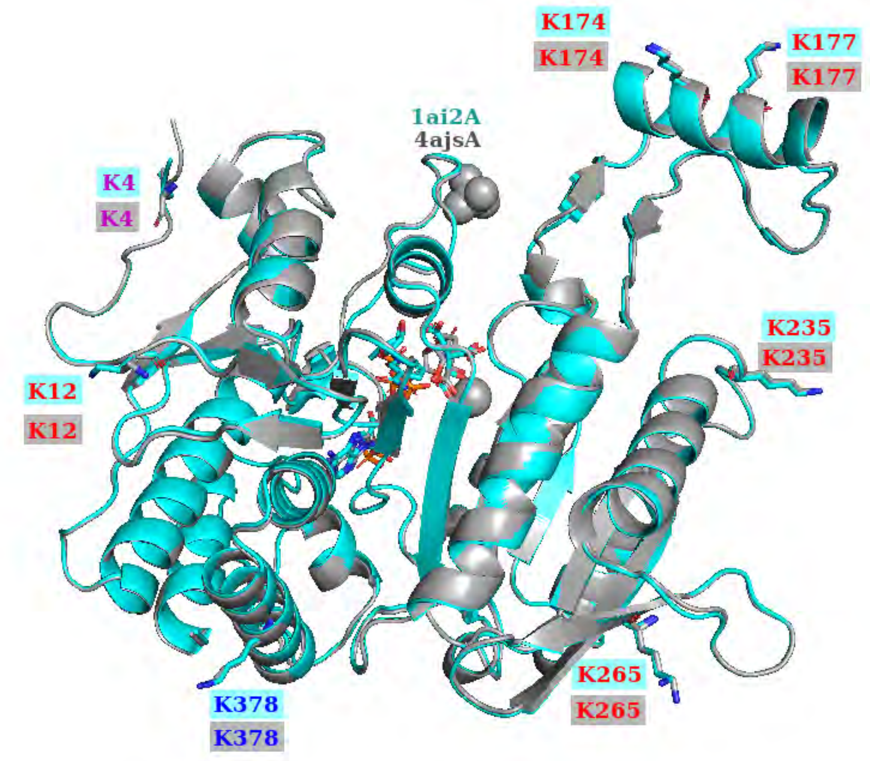


```
Align 1ai2.A.pdb 414 with 4ajs.A.pdb 415
Twists 0 ini-len 408 ini-rmsd 0.38 opt-equ 414 opt-rmsd 0.38 chain-rmsd 0.38 Score 1222.38 align-len 414 gaps 0 (0.00%)
P-value 0.00e+00 Afp-num 51906 Identity 99.76% Similarity 99.76%
Block  0 afp 51 score 1222.38 rmsd  0.38 gap 0 (0.00%)

                   .    :    .    :    .    :    .    :    .    :    .    :    .    :
Chain 1:     3 SKVVVPAQGKKITLQNGKLNVPENPIIPYIEGDGIGVDVTPAMLKVVDAAVEKAYKGERKISWMEIYTGE
               1111111111111111111111111111111111111111111111111111111111111111111111
Chain 2:     3 SKVVVPAQGKKITLQNGKLNVPENPIIPYIEGDGIGVDVTPAMLKVVDAAVEKAYKGERKISWMEIYTGE

                   .    :    .    :    .    :    .    :    .    :    .    :    .    :
Chain 1:    73 KSTQVYGQDVWLPAETLDLIREYRVAIKGPLTTPVGGGIRSLNVALRQELDLYICLRPVRYYQGTPSPVK
               1111111111111111111111111111111111111111111111111111111111111111111111
Chain 2:    73 KSTQVYGQDVWLPAETLDLIREYRVAIMGPLTTPVGGGIRSLNVALRQELDLYICLRPVRYYQGTPSPVK

                   .    :    .    :    .    :    .    :    .    :    .    :    .    :
Chain 1:   143 HPELTDMVIFRENSEDIYAGIEWKADSADAEKVIKFLREEMGVKKIRFPEHCGIGIKPCSEEGTKRLVRA
               1111111111111111111111111111111111111111111111111111111111111111111111
Chain 2:   143 HPELTDMVIFRENSEDIYAGIEWKADSADAEKVIKFLREEMGVKKIRFPEHCGIGIKPCSEEGTKRLVRA

                   .    :    .    :    .    :    .    :    .    :    .    :    .    :
Chain 1:   213 AIEYAIANDRDSVTLVHKGNIMKFTEGAFKDWGYQLAREEFGGELIDGGPWLKVKNPNTGKEIVIKDVIA
               1111111111111111111111111111111111111111111111111111111111111111111111
Chain 2:   213 AIEYAIANDRDSVTLVHKGNIMKFTEGAFKDWGYQLAREEFGGELIDGGPWLKVKNPNTGKEIVIKDVIA

                   .    :    .    :    .    :    .    :    .    :    .    :    .    :
Chain 1:   283 DAFLQQILLRPAEYDVIACMNLNGDYISDALAAQVGGIGIAPGANIGDECALFEATHGTAPKYAGQDKVN
               1111111111111111111111111111111111111111111111111111111111111111111111
Chain 2:   283 DAFLQQILLRPAEYDVIACMNLNGDYISDALAAQVGGIGIAPGANIGDECALFEATHGTAPKYAGQDKVN

                   .    :    .    :    .    :    .    :    .    :    .    :
Chain 1:   353 PGSIILSAEMMLRHMGWTEAADLIVKGMEGAINAKTVTYDFERLMDGAKLLKCSEFGDAIIENM
               1111111111111111111111111111111111111111111111111111111111111111
Chain 2:   353 PGSIILSAEMMLRHMGWTEAADLIVKGMEGAINAKTVTYDFERLMDGAKLLKCSEFGDAIIENM

Note: positions are from PDB; the numbers between alignments are block index
```

UniProt ID: P08200

PDB ID: 4BNP_A

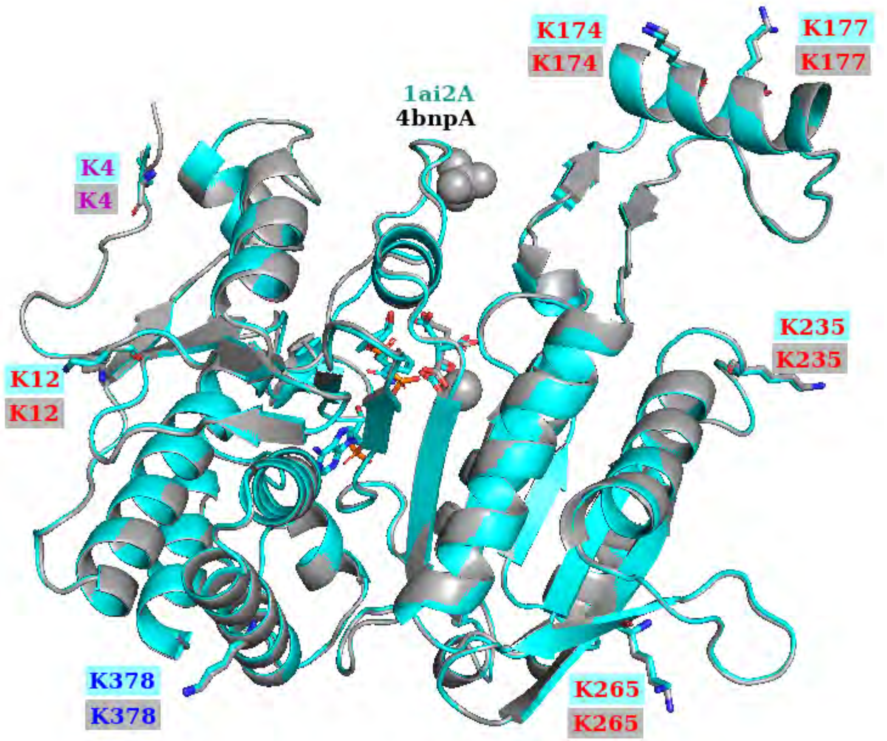


```
Align 1ai2.A.pdb 414 with 4bnp.A.pdb 415
Twists 0 ini-len 408 ini-rmsd 0.31 opt-equ 414 opt-rmsd 0.31 chain-rmsd 0.31 Score 1222.57 align-len 414 gaps 0 (0.00%)
P-value 0.00e+00 Afp-num 51905 Identity 99.76% Similarity 99.76%
Block  0 afp 51 score 1222.57 rmsd  0.31 gap 0 (0.00%)

                    .    :    .    :    .    :    .    :    .    :    .    :    .    :
Chain 1:    3 SKVVVPAQGKKITLQNGKLNVPENPIIPYIEGDGIGVDVTPAMLKVVDAAVEKAYKGERKISWMEIYTGE
              1111111111111111111111111111111111111111111111111111111111111111111111
Chain 2:    3 SKVVVPAQGKKITLQNGKLNVPENPIIPYIEGDGIGVDVTPAMLKVVDAAVEKAYKGERKISWMEIYTGE

                    .    :    .    :    .    :    .    :    .    :    .    :    .    :
Chain 1:   73 KSTQVYGQDVWLPAETLDLIREYRVAIKGPLTTPVGGGIRSLNVALRQELDLYICLRPVRYYQGTPSPVK
              1111111111111111111111111111111111111111111111111111111111111111111111
Chain 2:   73 KSTQVYGQDVWLPAETLDLIREYRVAIMGPLTTPVGGGIRSLNVALRQELDLYICLRPVRYYQGTPSPVK

                    .    :    .    :    .    :    .    :    .    :    .    :    .    :
Chain 1:  143 HPELTDMVIFRENSEDIYAGIEWKADSADAEKVIKFLREEMGVKKIRFPEHCGIGIKPCSEEGTKRLVRA
              1111111111111111111111111111111111111111111111111111111111111111111111
Chain 2:  143 HPELTDMVIFRENSEDIYAGIEWKADSADAEKVIKFLREEMGVKKIRFPEHCGIGIKPCSEEGTKRLVRA

                    .    :    .    :    .    :    .    :    .    :    .    :    .    :
Chain 1:  213 AIEYAIANDRDSVTLVHKGNIMKFTEGAFKDWGYQLAREEFGGELIDGGPWLKVKNPNTGKEIVIKDVIA
              1111111111111111111111111111111111111111111111111111111111111111111111
Chain 2:  213 AIEYAIANDRDSVTLVHKGNIMKFTEGAFKDWGYQLAREEFGGELIDGGPWLKVKNPNTGKEIVIKDVIA

                    .    :    .    :    .    :    .    :    .    :    .    :    .    :
Chain 1:  283 DAFLQQILLRPAEYDVIACMNLNGDYISDALAAQVGGIGIAPGANIGDECALFEATHGTAPKYAGQDKVN
              1111111111111111111111111111111111111111111111111111111111111111111111
Chain 2:  283 DAFLQQILLRPAEYDVIACMNLNGDYISDALAAQVGGIGIAPGANIGDECALFEATHGTAPKYAGQDKVN

                    .    :    .    :    .    :    .    :    .    :    .    :
Chain 1:  353 PGSIILSAEMMLRHMGWTEAADLIVKGMEGAINAKTVTYDFERLMDGAKLLKCSEFGDAIIENM
              11111111111111111111111111111111111111111111111111111111111111
Chain 2:  353 PGSIILSAEMMLRHMGWTEAADLIVKGMEGAINAKTVTYDFERLMDGAKLLKCSEFGDAIIENM

Note: positions are from PDB; the numbers between alignments are block index
```

UniProt ID: P08200
PDB ID: 4ICD_A

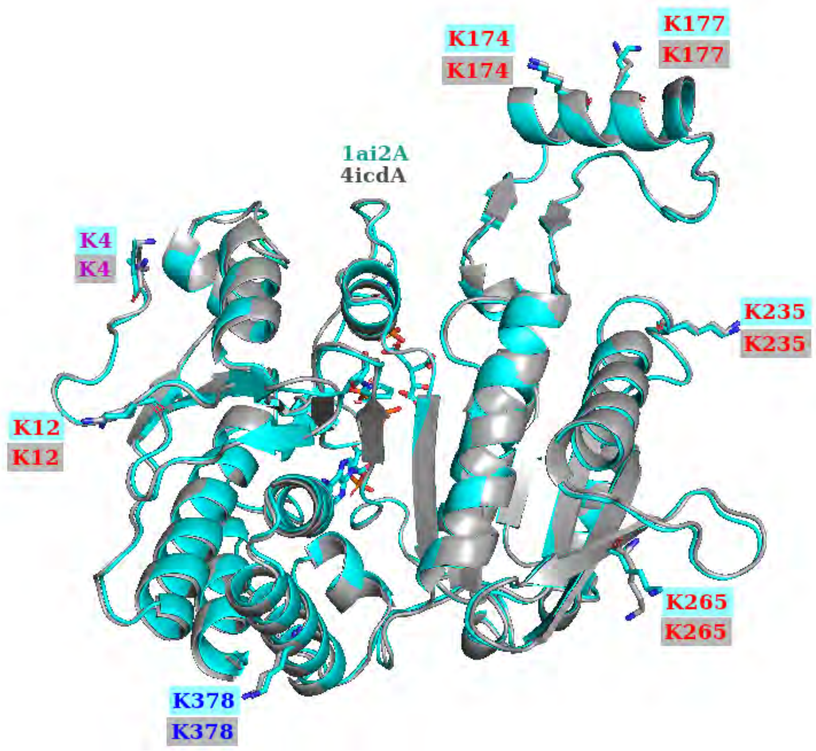


```
Align 1ai2.A.pdb 414 with 4icd.A.pdb 414
Twists 0 ini-len 408 ini-rmsd 0.46 opt-equ 414 opt-rmsd 0.47 chain-rmsd 0.46 Score 1220.74 align-len 414 gaps 0 (0.00%)
P-value 0.00e+00 Afp-num 51647 Identity 99.76% Similarity 99.76%
Block  0 afp 51 score 1220.74 rmsd  0.46 gap 0 (0.00%)

                    .    :    .    :    .    :    .    :    .    :    .    :    .    :
Chain 1:    3 SKVVVPAQGKKITLQNGKLNVPENPIIPYIEGDGIGVDVTPAMLKVVDAAVEKAYKGERKISWMEIYTGE
              1111111111111111111111111111111111111111111111111111111111111111111111
Chain 2:    3 SKVVVPAQGKKITLQNGKLNVPENPIIPYIEGDGIGVDVTPAMLKVVDAAVEKAYKGERKISWMEIYTGE

                    .    :    .    :    .    :    .    :    .    :    .    :    .    :
Chain 1:   73 KSTQVYGQDVWLPAETLDLIREYRVAIKGPLTTPVGGGIRSLNVALRQELDLYICLRPVRYYQGTPSPVK
              1111111111111111111111111111111111111111111111111111111111111111111111
Chain 2:   73 KSTQVYGQDVWLPAETLDLIREYRVAIKGPLTTPVGGGIRxLNVALRQELDLYICLRPVRYYQGTPSPVK

                    .    :    .    :    .    :    .    :    .    :    .    :    .    :
Chain 1:  143 HPELTDMVIFRENSEDIYAGIEWKADSADAEKVIKFLREEMGVKKIRFPEHCGIGIKPCSEEGTKRLVRA
              1111111111111111111111111111111111111111111111111111111111111111111111
Chain 2:  143 HPELTDMVIFRENSEDIYAGIEWKADSADAEKVIKFLREEMGVKKIRFPEHCGIGIKPCSEEGTKRLVRA

                    .    :    .    :    .    :    .    :    .    :    .    :    .    :
Chain 1:  213 AIEYAIANDRDSVTLVHKGNIMKFTEGAFKDWGYQLAREEFGGELIDGGPWLKVKNPNTGKEIVIKDVIA
              1111111111111111111111111111111111111111111111111111111111111111111111
Chain 2:  213 AIEYAIANDRDSVTLVHKGNIMKFTEGAFKDWGYQLAREEFGGELIDGGPWLKVKNPNTGKEIVIKDVIA

                    .    :    .    :    .    :    .    :    .    :    .    :    .    :
Chain 1:  283 DAFLQQILLRPAEYDVIACMNLNGDYISDALAAQVGGIGIAPGANIGDECALFEATHGTAPKYAGQDKVN
              1111111111111111111111111111111111111111111111111111111111111111111111
Chain 2:  283 DAFLQQILLRPAEYDVIACMNLNGDYISDALAAQVGGIGIAPGANIGDECALFEATHGTAPKYAGQDKVN

                    .    :    .    :    .    :    .    :    .    :    .    :
Chain 1:  353 PGSIILSAEMMLRHMGWTEAADLIVKGMEGAINAKTVTYDFERLMDGAKLLKCSEFGDAIIENM
              1111111111111111111111111111111111111111111111111111111111111111
Chain 2:  353 PGSIILSAEMMLRHMGWTEAADLIVKGMEGAINAKTVTYDFERLMDGAKLLKCSEFGDAIIENM

Note: positions are from PDB; the numbers between alignments are block index
```

UniProt ID: P08200

PDB ID: 5ICD_A

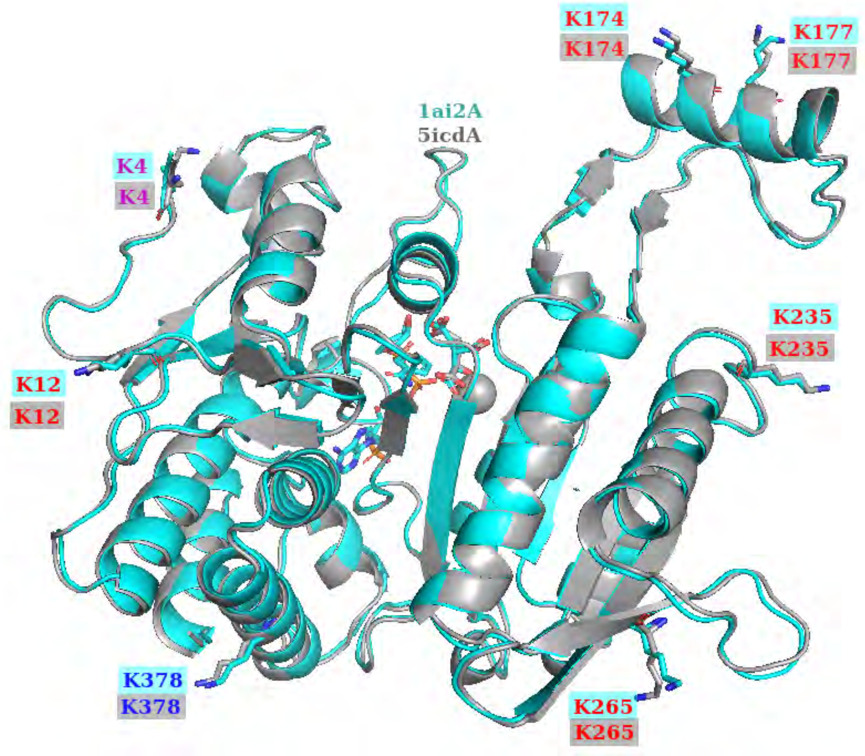

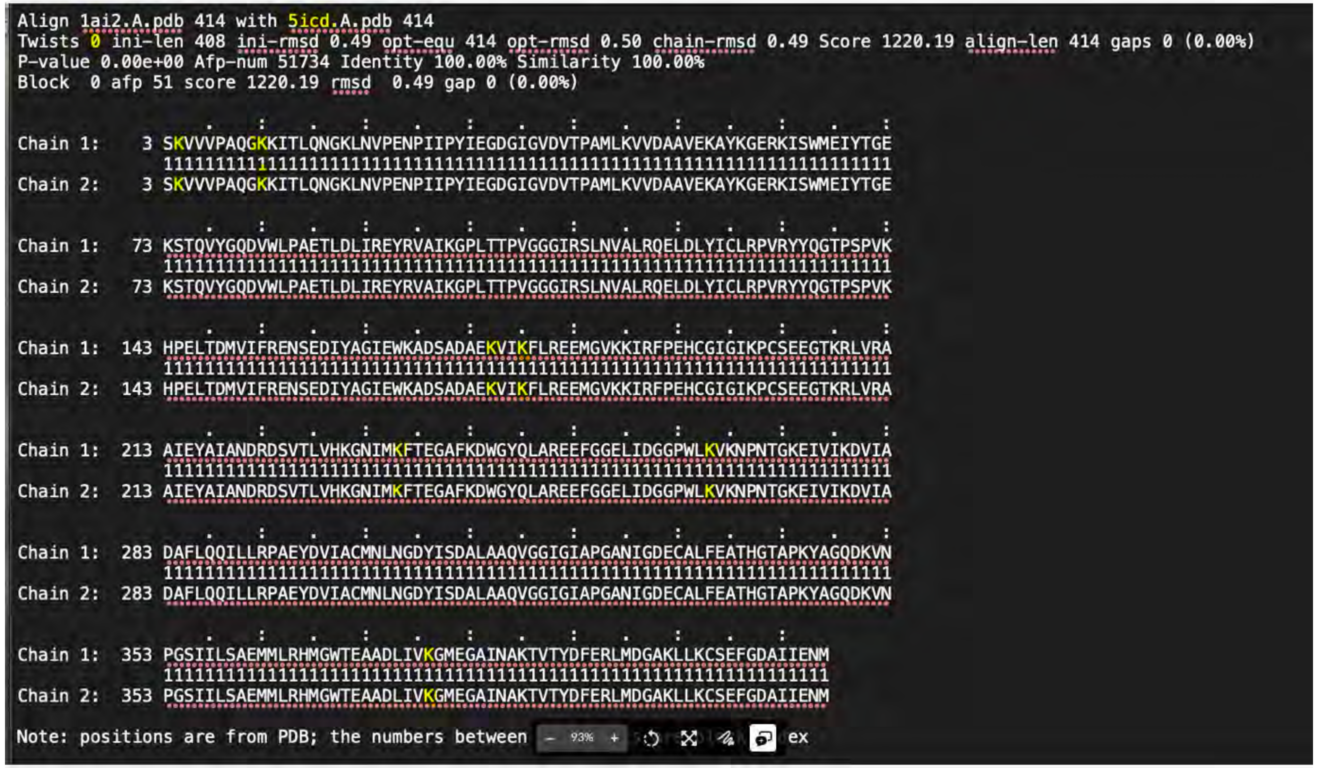

```
Align 1ai2.A.pdb 414 with 5icd.A.pdb 414
Twists 0 ini-len 408 ini-rmsd 0.49 opt-equ 414 opt-rmsd 0.50 chain-rmsd 0.49 Score 1220.19 align-len 414 gaps 0 (0.00%)
P-value 0.00e+00 Afp-num 51734 Identity 100.00% Similarity 100.00%
Block  0 afp 51 score 1220.19 rmsd  0.49 gap 0 (0.00%)

                    .    :    .    :    .    :    .    :    .    :    .    :    .    :
Chain 1:    3 SKVVVPAQGKKITLQNGKLNVPENPIIPYIEGDGIGVDVTPAMLKVVDAAVEKAYKGERKISWMEIYTGE
              1111111111111111111111111111111111111111111111111111111111111111111111
Chain 2:    3 SKVVVPAQGKKITLQNGKLNVPENPIIPYIEGDGIGVDVTPAMLKVVDAAVEKAYKGERKISWMEIYTGE

                    .    :    .    :    .    :    .    :    .    :    .    :    .    :
Chain 1:   73 KSTQVYGQDVWLPAETLDLIREYRVAIKGPLTTPVGGGIRSLNVALRQELDLYICLRPVRYYQGTPSPVK
              1111111111111111111111111111111111111111111111111111111111111111111111
Chain 2:   73 KSTQVYGQDVWLPAETLDLIREYRVAIKGPLTTPVGGGIRSLNVALRQELDLYICLRPVRYYQGTPSPVK

                    .    :    .    :    .    :    .    :    .    :    .    :    .    :
Chain 1:  143 HPELTDMVIFRENSEDIYAGIEWKADSADAEKVIKFLREEMGVKKIRFPEHCGIGIKPCSEEGTKRLVRA
              1111111111111111111111111111111111111111111111111111111111111111111111
Chain 2:  143 HPELTDMVIFRENSEDIYAGIEWKADSADAEKVIKFLREEMGVKKIRFPEHCGIGIKPCSEEGTKRLVRA

                    .    :    .    :    .    :    .    :    .    :    .    :    .    :
Chain 1:  213 AIEYAIANDRDSVTLVHKGNIMKFTEGAFKDWGYQLAREEFGGELIDGGPWLKVKNPNTGKEIVIKDVIA
              1111111111111111111111111111111111111111111111111111111111111111111111
Chain 2:  213 AIEYAIANDRDSVTLVHKGNIMKFTEGAFKDWGYQLAREEFGGELIDGGPWLKVKNPNTGKEIVIKDVIA

                    .    :    .    :    .    :    .    :    .    :    .    :    .    :
Chain 1:  283 DAFLQQILLRPAEYDVIACMNLNGDYISDALAAQVGGIGIAPGANIGDECALFEATHGTAPKYAGQDKVN
              1111111111111111111111111111111111111111111111111111111111111111111111
Chain 2:  283 DAFLQQILLRPAEYDVIACMNLNGDYISDALAAQVGGIGIAPGANIGDECALFEATHGTAPKYAGQDKVN

                    .    :    .    :    .    :    .    :    .    :    .    :
Chain 1:  353 PGSIILSAEMMLRHMGWTEAADLIVKGMEGAINAKTVTYDFERLMDGAKLLKCSEFGDAIIENM
              11111111111111111111111111111111111111111111111111111111111111
Chain 2:  353 PGSIILSAEMMLRHMGWTEAADLIVKGMEGAINAKTVTYDFERLMDGAKLLKCSEFGDAIIENM

Note: positions are from PDB; the numbers between  – 93% +  ex
```

UniProt ID: P08200
PDB ID: 6ICD_A

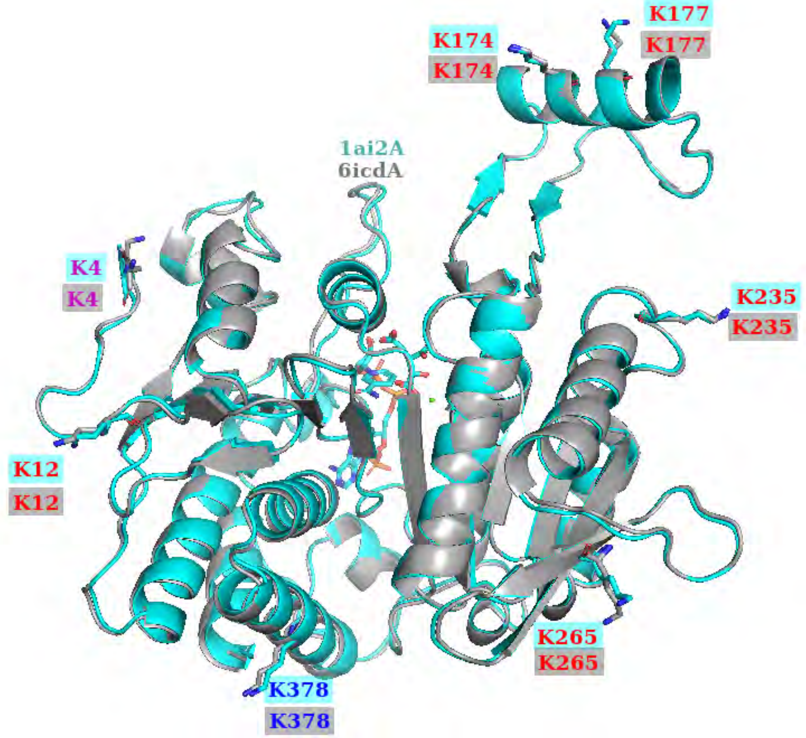


```
Align 1ai2.A.pdb 414 with 6icd.A.pdb 414
Twists 0 ini-len 408 ini-rmsd 0.46 opt-equ 414 opt-rmsd 0.47 chain-rmsd 0.46 Score 1220.22 align-len 414 gaps 0 (0.00%)
P-value 0.00e+00 Afp-num 51636 Identity 99.76% Similarity 99.76%
Block  0 afp 51 score 1220.22 rmsd  0.46 gap 0 (0.00%)

                         .    :    .    :    .    :    .    :    .    :    .    :    .    :
Chain 1:    3 SKVVVPAQGKKITLQNGKLNVPENPIIPYIEGDGIGVDVTPAMLKVVDAAVEKAYKGERKISWMEIYTGE
                1111111111111111111111111111111111111111111111111111111111111111111111
Chain 2:    3 SKVVVPAQGKKITLQNGKLNVPENPIIPYIEGDGIGVDVTPAMLKVVDAAVEKAYKGERKISWMEIYTGE

                         .    :    .    :    .    :    .    :    .    :    .    :    .    :
Chain 1:   73 KSTQVYGQDVWLPAETLDLIREYRVAIKGPLTTPVGGGIRSLNVALRQELDLYICLRPVRYYQGTPSPVK
                1111111111111111111111111111111111111111111111111111111111111111111111
Chain 2:   73 KSTQVYGQDVWLPAETLDLIREYRVAIKGPLTTPVGGGIRDLNVALRQELDLYICLRPVRYYQGTPSPVK

                         .    :    .    :    .    :    .    :    .    :    .    :    .    :
Chain 1:  143 HPELTDMVIFRENSEDIYAGIEWKADSADAEKVIKFLREEMGVKKIRFPEHCGIGIKPCSEEGTKRLVRA
                1111111111111111111111111111111111111111111111111111111111111111111111
Chain 2:  143 HPELTDMVIFRENSEDIYAGIEWKADSADAEKVIKFLREEMGVKKIRFPEHCGIGIKPCSEEGTKRLVRA

                         .    :    .    :    .    :    .    :    .    :    .    :    .    :
Chain 1:  213 AIEYAIANDRDSVTLVHKGNIMKFTEGAFKDWGYQLAREEFGGELIDGGPWLKVKNPNTGKEIVIKDVIA
                1111111111111111111111111111111111111111111111111111111111111111111111
Chain 2:  213 AIEYAIANDRDSVTLVHKGNIMKFTEGAFKDWGYQLAREEFGGELIDGGPWLKVKNPNTGKEIVIKDVIA

                         .    :    .    :    .    :    .    :    .    :    .    :    .    :
Chain 1:  283 DAFLQQILLRPAEYDVIACMNLNGDYISDALAAQVGGIGIAPGANIGDECALFEATHGTAPKYAGQDKVN
                1111111111111111111111111111111111111111111111111111111111111111111111
Chain 2:  283 DAFLQQILLRPAEYDVIACMNLNGDYISDALAAQVGGIGIAPGANIGDECALFEATHGTAPKYAGQDKVN

                         .    :    .    :    .    :    .    :    .    :    .    :
Chain 1:  353 PGSIILSAEMMLRHMGWTEAADLIVKGMEGAINAKTVTYDFERLMDGAKLLKCSEFGDAIIENM
                11111111111111111111111111111111111111111111111111111111111111
Chain 2:  353 PGSIILSAEMMLRHMGWTEAADLIVKGMEGAINAKTVTYDFERLMDGAKLLKCSEFGDAIIENM

Note: positions are from PDB; the numbers between alignments are block index
```

UniProt ID: P08200

PDB ID: 7ICD_A

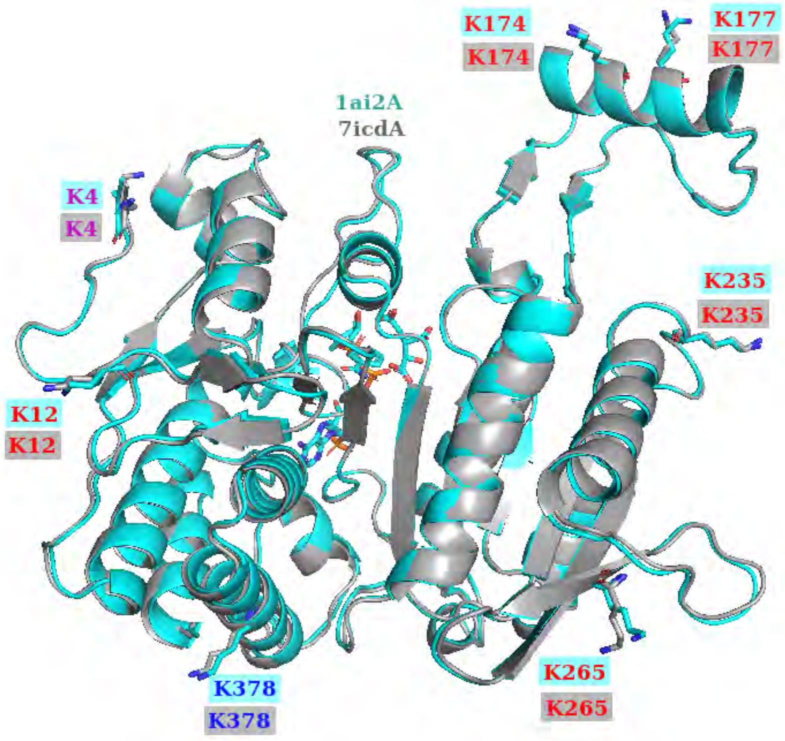


```
Align 1ai2.A.pdb 414 with 7icd.A.pdb 414
Twists 0 ini-len 408 ini-rmsd 0.45 opt-equ 414 opt-rmsd 0.46 chain-rmsd 0.45 Score 1220.53 align-len 414 gaps 0 (0.00%)
P-value 0.00e+00 Afp-num 51629 Identity 99.76% Similarity 99.76%
Block  0 afp 51 score 1220.53 rmsd  0.45 gap 0 (0.00%)

                   .    :    .    :    .    :    .    :    .    :    .    :    .    :
Chain 1:    3 SKVVVPAQGKKITLQNGKLNVPENPIIPYIEGDGIGVDVTPAMLKVVDAAVEKAYKGERKISWMEIYTGE
              1111111111111111111111111111111111111111111111111111111111111111111111
Chain 2:    3 SKVVVPAQGKKITLQNGKLNVPENPIIPYIEGDGIGVDVTPAMLKVVDAAVEKAYKGERKISWMEIYTGE

                   .    :    .    :    .    :    .    :    .    :    .    :    .    :
Chain 1:   73 KSTQVYGQDVWLPAETLDLIREYRVAIKGPLTTPVGGGIRSLNVALRQELDLYICLRPVRYYQGTPSPVK
              1111111111111111111111111111111111111111111111111111111111111111111111
Chain 2:   73 KSTQVYGQDVWLPAETLDLIREYRVAIKGPLTTPVGGGIRELNVALRQELDLYICLRPVRYYQGTPSPVK

                   .    :    .    :    .    :    .    :    .    :    .    :    .    :
Chain 1:  143 HPELTDMVIFRENSEDIYAGIEWKADSADAEKVIKFLREEMGVKKIRFPEHCGIGIKPCSEEGTKRLVRA
              1111111111111111111111111111111111111111111111111111111111111111111111
Chain 2:  143 HPELTDMVIFRENSEDIYAGIEWKADSADAEKVIKFLREEMGVKKIRFPEHCGIGIKPCSEEGTKRLVRA

                   .    :    .    :    .    :    .    :    .    :    .    :    .    :
Chain 1:  213 AIEYAIANDRDSVTLVHKGNIMKFTEGAFKDWGYQLAREEFGGELIDGGPWLKVKNPNTGKEIVIKDVIA
              1111111111111111111111111111111111111111111111111111111111111111111111
Chain 2:  213 AIEYAIANDRDSVTLVHKGNIMKFTEGAFKDWGYQLAREEFGGELIDGGPWLKVKNPNTGKEIVIKDVIA

                   .    :    .    :    .    :    .    :    .    :    .    :    .    :
Chain 1:  283 DAFLQQILLRPAEYDVIACMNLNGDYISDALAAQVGGIGIAPGANIGDECALFEATHGTAPKYAGQDKVN
              1111111111111111111111111111111111111111111111111111111111111111111111
Chain 2:  283 DAFLQQILLRPAEYDVIACMNLNGDYISDALAAQVGGIGIAPGANIGDECALFEATHGTAPKYAGQDKVN

                   .    :    .    :    .    :    .    :    .    :    .    :
Chain 1:  353 PGSIILSAEMMLRHMGWTEAADLIVKGMEGAINAKTVTYDFERLMDGAKLLKCSEFGDAIIENM
              11111111111111111111111111111111111111111111111111111111111111
Chain 2:  353 PGSIILSAEMMLRHMGWTEAADLIVKGMEGAINAKTVTYDFERLMDGAKLLKCSEFGDAIIENM

Note: positions are from PDB; the numbers between alignments are block index
```

UniProt ID: P08200

PDB ID: 8ICD_A

```
Align 1ai2.A.pdb 414 with 8icd.A.pdb 414
Twists 0 ini-len 408 ini-rmsd 0.47 opt-equ 414 opt-rmsd 0.47 chain-rmsd 0.47 Score 1220.35 align-len 414 gaps 0 (0.00%)
P-value 0.00e+00 Afp-num 51648 Identity 99.76% Similarity 99.76%
Block  0 afp 51 score 1220.35 rmsd  0.47 gap 0 (0.00%)

                  .    :    .    :    .    :    .    :    .    :    .    :    .    :
Chain 1:    3 SKVVVPAQGKKITLQNGKLNVPENPIIPYIEGDGIGVDVTPAMLKVVDAAVEKAYKGERKISWMEIYTGE
              1111111111111111111111111111111111111111111111111111111111111111111111
Chain 2:    3 SKVVVPAQGKKITLQNGKLNVPENPIIPYIEGDGIGVDVTPAMLKVVDAAVEKAYKGERKISWMEIYTGE

                  .    :    .    :    .    :    .    :    .    :    .    :    .    :
Chain 1:   73 KSTQVYGQDVWLPAETLDLIREYRVAIKGPLTTPVGGGIRSLNVALRQELDLYICLRPVRYYQGTPSPVK
              1111111111111111111111111111111111111111111111111111111111111111111111
Chain 2:   73 KSTQVYGQDVWLPAETLDLIREYRVAIKGPLTTPVGGGIRELNVALRQELDLYICLRPVRYYQGTPSPVK

                  .    :    .    :    .    :    .    :    .    :    .    :    .    :
Chain 1:  143 HPELTDMVIFRENSEDIYAGIEWKADSADAEKVIKFLREEMGVKKIRFPEHCGIGIKPCSEEGTKRLVRA
              1111111111111111111111111111111111111111111111111111111111111111111111
Chain 2:  143 HPELTDMVIFRENSEDIYAGIEWKADSADAEKVIKFLREEMGVKKIRFPEHCGIGIKPCSEEGTKRLVRA

                  .    :    .    :    .    :    .    :    .    :    .    :    .    :
Chain 1:  213 AIEYAIANDRDSVTLVHKGNIMKFTEGAFKDWGYQLAREEFGGELIDGGPWLKVKNPNTGKEIVIKDVIA
              1111111111111111111111111111111111111111111111111111111111111111111111
Chain 2:  213 AIEYAIANDRDSVTLVHKGNIMKFTEGAFKDWGYQLAREEFGGELIDGGPWLKVKNPNTGKEIVIKDVIA

                  .    :    .    :    .    :    .    :    .    :    .    :    .    :
Chain 1:  283 DAFLQQILLRPAEYDVIACMNLNGDYISDALAAQVGGIGIAPGANIGDECALFEATHGTAPKYAGQDKVN
              1111111111111111111111111111111111111111111111111111111111111111111111
Chain 2:  283 DAFLQQILLRPAEYDVIACMNLNGDYISDALAAQVGGIGIAPGANIGDECALFEATHGTAPKYAGQDKVN

                  .    :    .    :    .    :    .    :    .    :    .    :
Chain 1:  353 PGSIILSAEMMLRHMGWTEAADLIVKGMEGAINAKTVTYDFERLMDGAKLLKCSEFGDAIIENM
              1111111111111111111111111111111111111111111111111111111111111111
Chain 2:  353 PGSIILSAEMMLRHMGWTEAADLIVKGMEGAINAKTVTYDFERLMDGAKLLKCSEFGDAIIENM

Note: positions are from PDB; the numbers between alignments are block index
```

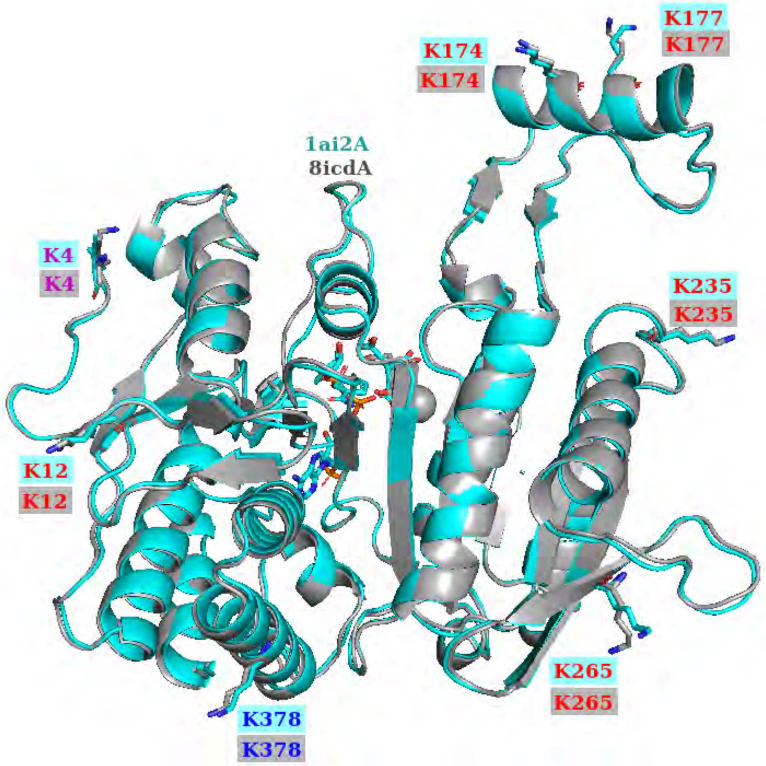

UniProt ID: P08200
PDB ID: 9ICD_A

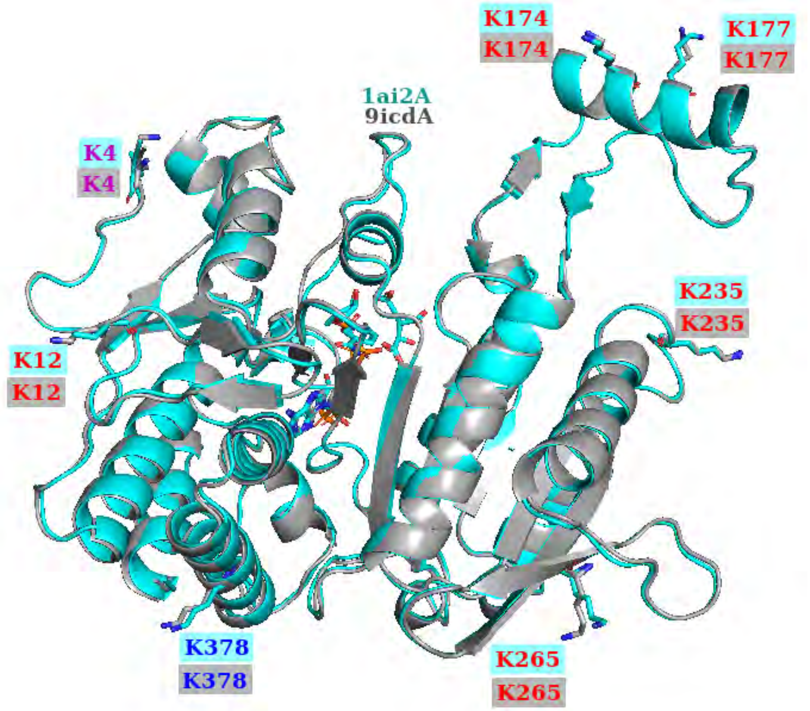


```
Align 1ai2.A.pdb 414 with 9icd.A.pdb 414
Twists 0 ini-len 408 ini-rmsd 0.44 opt-equ 414 opt-rmsd 0.45 chain-rmsd 0.44 Score 1220.91 align-len 414 gaps 0 (0.00%)
P-value 0.00e+00 Afp-num 51536 Identity 100.00% Similarity 100.00%
Block  0 afp 51 score 1220.91 rmsd  0.44 gap 0 (0.00%)

                    .    :    .    :    .    :    .    :    .    :    .    :    .    :
Chain 1:    3 SKVVVPAQGKKITLQNGKLNVPENPIIPYIEGDGIGVDVTPAMLKVVDAAVEKAYKGERKISWMEIYTGE
              1111111111111111111111111111111111111111111111111111111111111111111111
Chain 2:    3 SKVVVPAQGKKITLQNGKLNVPENPIIPYIEGDGIGVDVTPAMLKVVDAAVEKAYKGERKISWMEIYTGE

                    .    :    .    :    .    :    .    :    .    :    .    :    .    :
Chain 1:   73 KSTQVYGQDVWLPAETLDLIREYRVAIKGPLTTPVGGGIRSLNVALRQELDLYICLRPVRYYQGTPSPVK
              1111111111111111111111111111111111111111111111111111111111111111111111
Chain 2:   73 KSTQVYGQDVWLPAETLDLIREYRVAIKGPLTTPVGGGIRSLNVALRQELDLYICLRPVRYYQGTPSPVK

                    .    :    .    :    .    :    .    :    .    :    .    :    .    :
Chain 1:  143 HPELTDMVIFRENSEDIYAGIEWKADSADAEKVIKFLREEMGVKKIRFPEHCGIGIKPCSEEGTKRLVRA
              1111111111111111111111111111111111111111111111111111111111111111111111
Chain 2:  143 HPELTDMVIFRENSEDIYAGIEWKADSADAEKVIKFLREEMGVKKIRFPEHCGIGIKPCSEEGTKRLVRA

                    .    :    .    :    .    :    .    :    .    :    .    :    .    :
Chain 1:  213 AIEYAIANDRDSVTLVHKGNIMKFTEGAFKDWGYQLAREEFGGELIDGGPWLKVKNPNTGKEIVIKDVIA
              1111111111111111111111111111111111111111111111111111111111111111111111
Chain 2:  213 AIEYAIANDRDSVTLVHKGNIMKFTEGAFKDWGYQLAREEFGGELIDGGPWLKVKNPNTGKEIVIKDVIA

                    .    :    .    :    .    :    .    :    .    :    .    :    .    :
Chain 1:  283 DAFLQQILLRPAEYDVIACMNLNGDYISDALAAQVGGIGIAPGANIGDECALFEATHGTAPKYAGQDKVN
              1111111111111111111111111111111111111111111111111111111111111111111111
Chain 2:  283 DAFLQQILLRPAEYDVIACMNLNGDYISDALAAQVGGIGIAPGANIGDECALFEATHGTAPKYAGQDKVN

                    .    :    .    :    .    :    .    :    .    :    .    :
Chain 1:  353 PGSIILSAEMMLRHMGWTEAADLIVKGMEGAINAKTVTYDFERLMDGAKLLKCSEFGDAIIENM
              11111111111111111111111111111111111111111111111111111111111111
Chain 2:  353 PGSIILSAEMMLRHMGWTEAADLIVKGMEGAINAKTVTYDFERLMDGAKLLKCSEFGDAIIENM

Note: positions are from PDB; the numbers between alignments are block index
```

UniProt ID: P28834

PDB ID: 3BLV_G

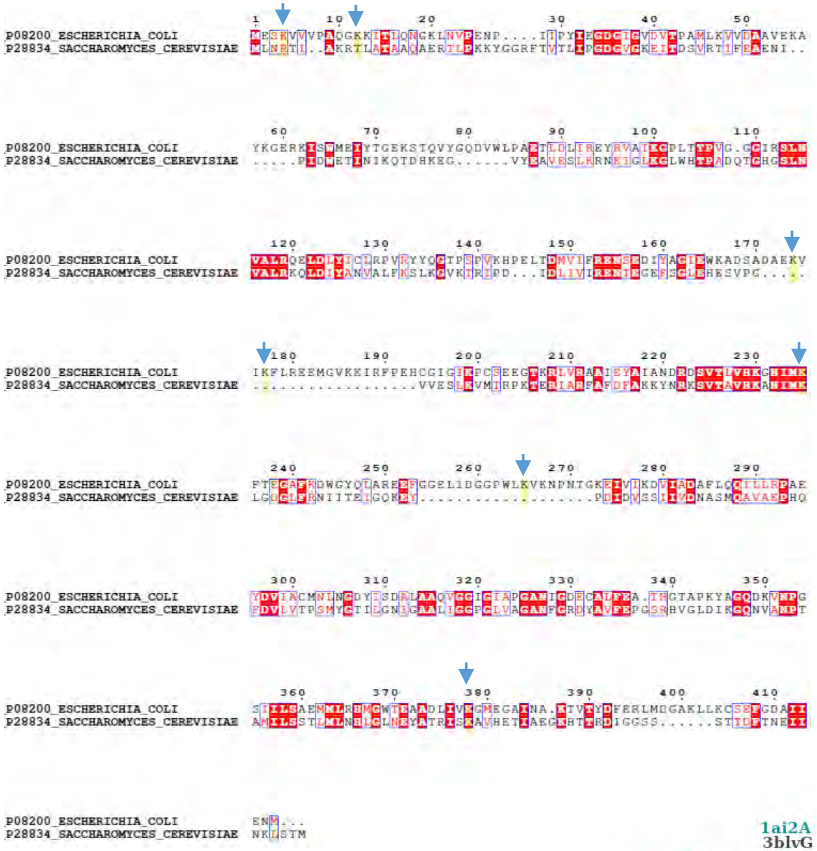

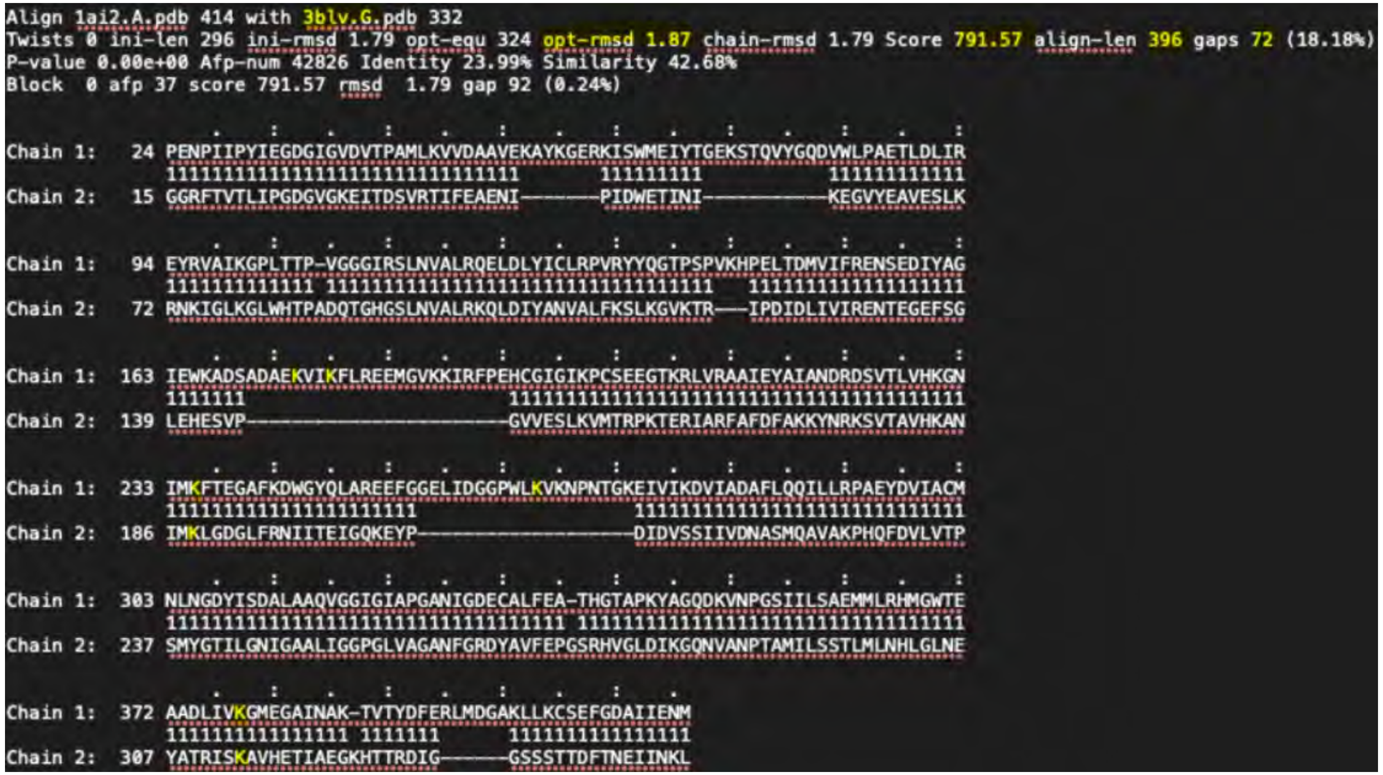

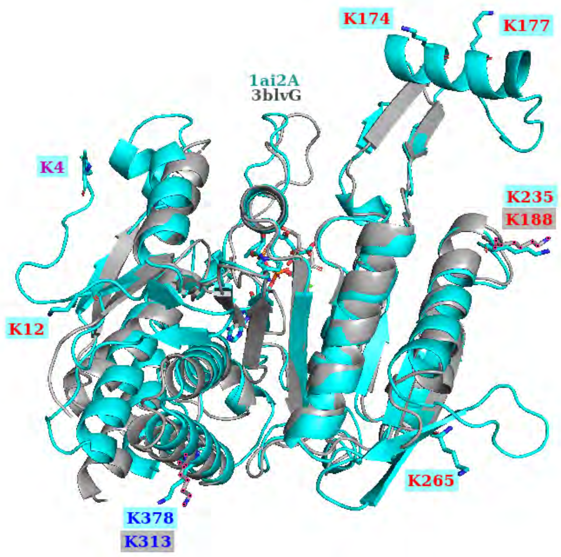

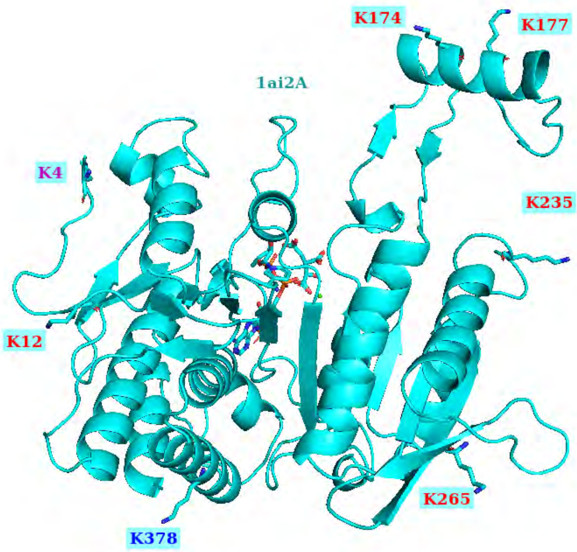

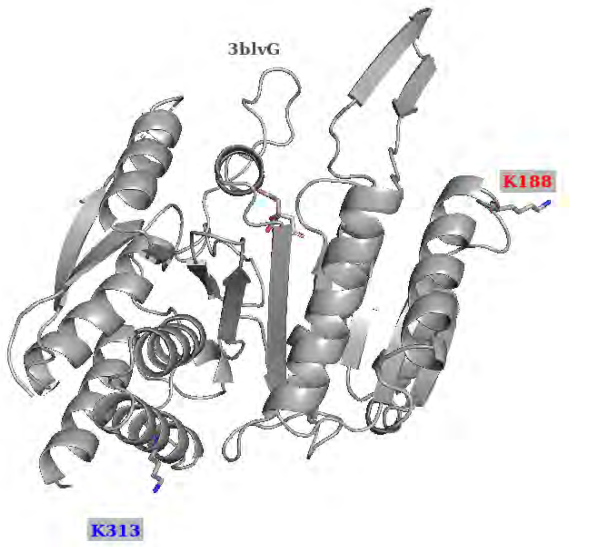

UniProt ID: P28834
PDB ID: 3BLW_A

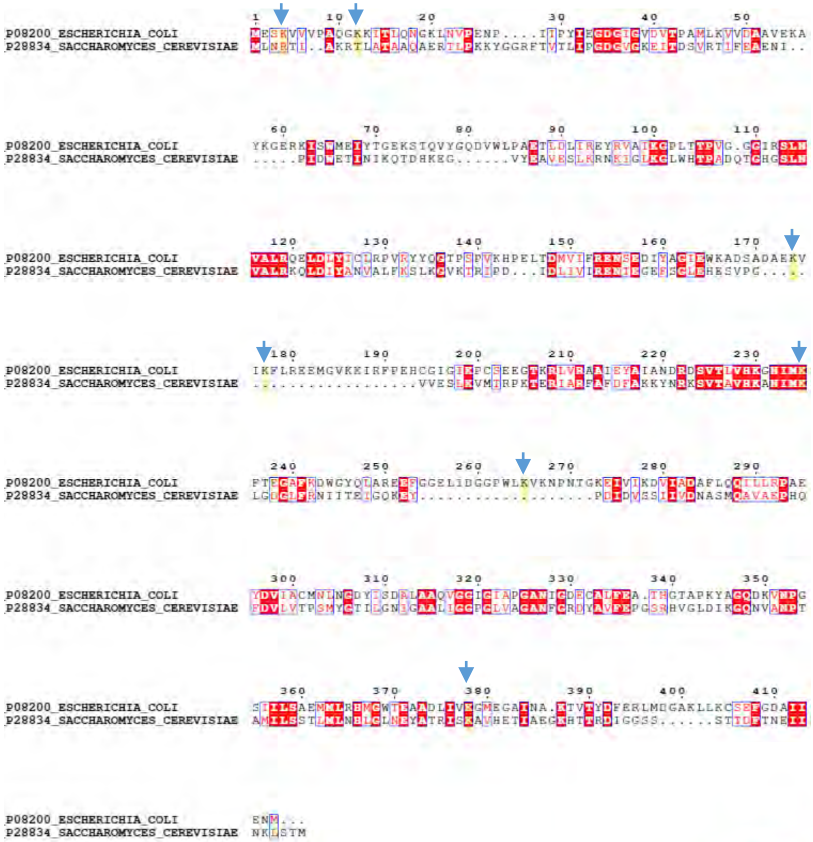

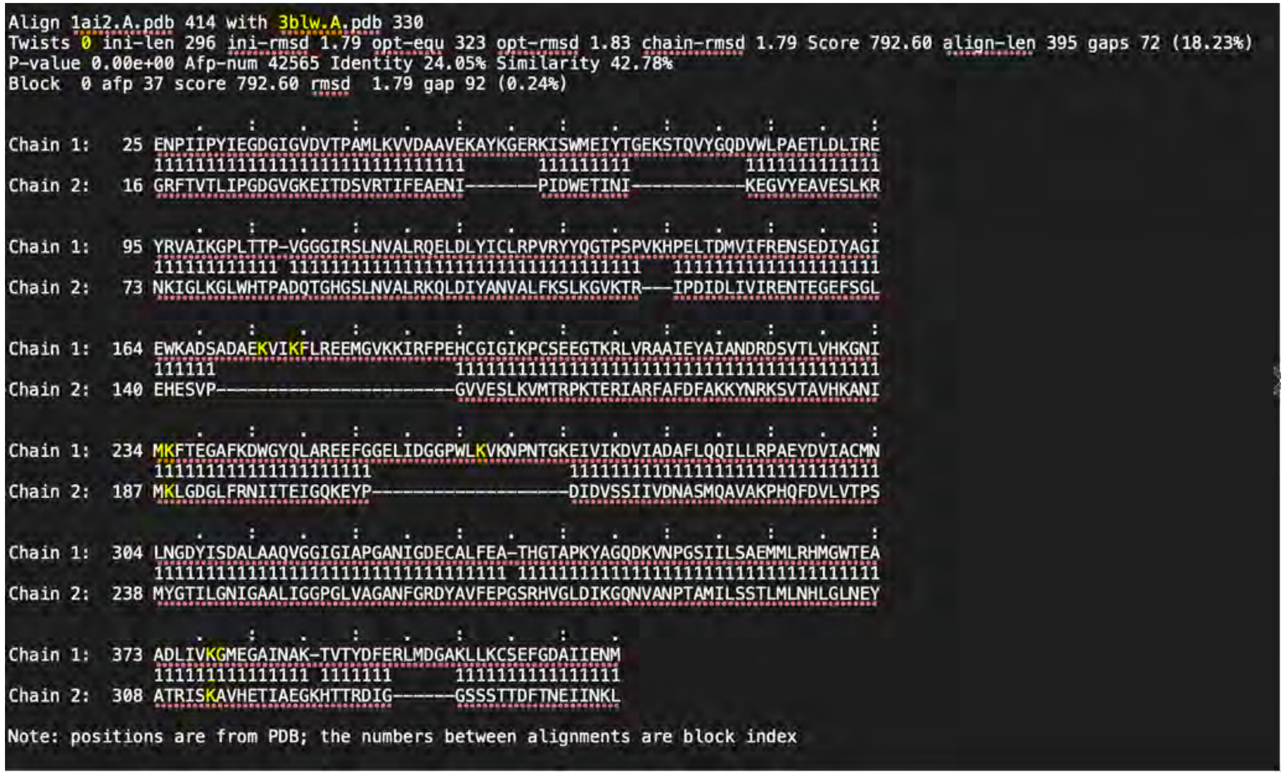

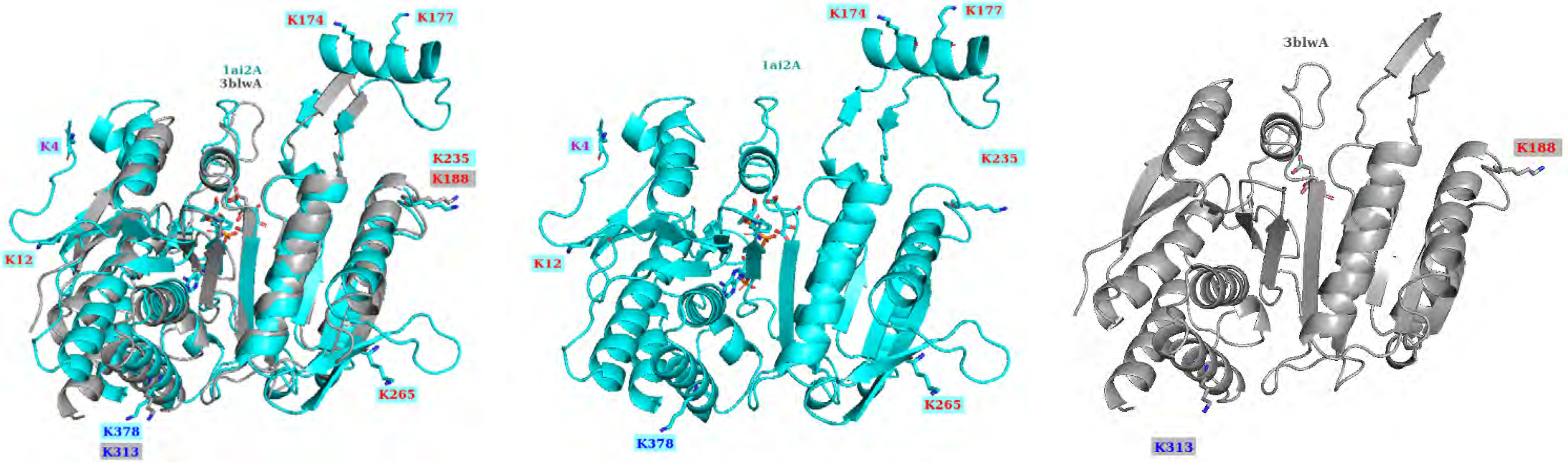

UniProt ID: P28834

PDB ID: 3BLX_O

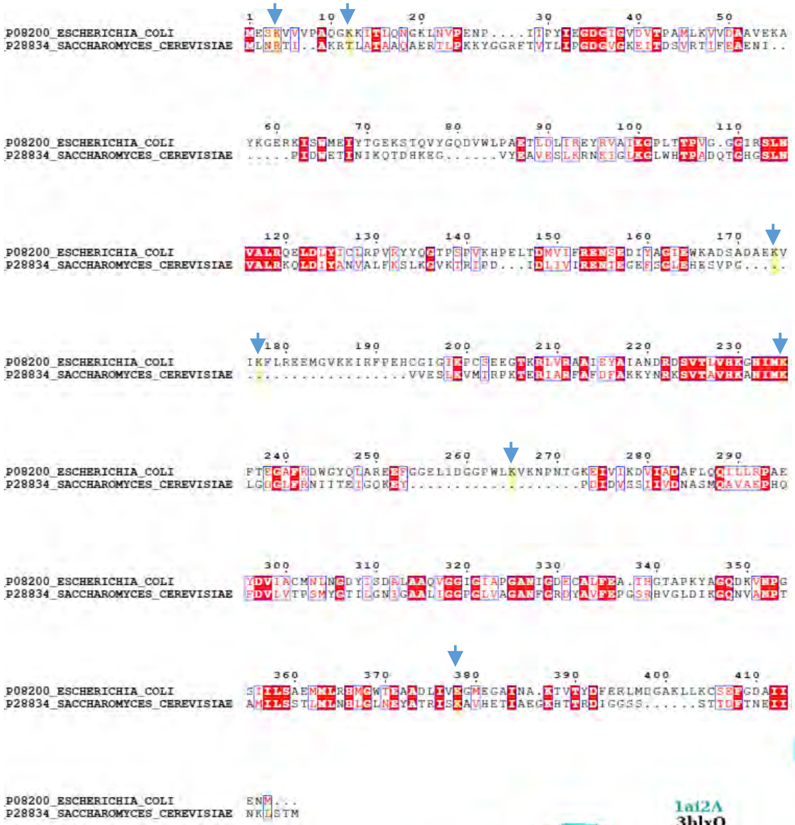


```
Align 1ai2.A.pdb 414 with 3blx.O.pdb 332
Twists 0 ini-len 272 ini-rmsd 1.70 opt-equ 328 opt-rmsd 2.88 chain-rmsd 1.70 Score 697.45 align-len 394 gaps 66 (16.75%)
P-value 0.00e+00 Afp-num 41478 Identity 23.86% Similarity 42.64%
Block  0 afp 34 score 697.45 rmsd  1.70 gap 115 (0.30%)

                  .    :    .    :    .    :    .    :    .    :    .    :    .    :
Chain 1:   24 PENPIIPYIEGDGIGVDVTPAMLKVVDAAVEKAYKGERKISWMEIYTGEKSTQVYGQDVWLPAETLDLIR
              1111111111111111111111111111111       1111111111111      1111111111111
Chain 2:   15 GGRFTVTLIPGDGVGKEITDSVRTIFEAENI-------PIDWETINIKQTD------HKEGVYEAVESLK

                  .    :    .    :    .    :    .    :    .    :    .    :    .    :
Chain 1:   94 EYRVAIKGPLTTPVG-GGIRSLNVALRQELDLYICLRPVRYYQGTPSPVKHPELTDMVIFRENSEDIYAG
              111111111111111 11111111111111111111111111111111   1111111111111111111
Chain 2:   72 RNKIGLKGLWHTPADQTGHGSLNVALRKQLDIYANVALFKSLKGVKTR---IPDIDLIVIRENTEGEFSG

                  .    :    .    :    .    :    .    :    .    :    .    :    .    :
Chain 1:  163 IEWKADSADAEKVIKFLREEMGVKKIRFPEHCGIGIKPCSEEGTKRLVRAAIEYAIANDRDSVTLVHKGN
              111111                       11111111111111111111111111111111111111111
Chain 2:  139 LEHESV-----------------------PGVVESLKVMTRPKTERIARFAFDFAKKYNRKSVTAVHKAN

                  .    :    .    :    .    :    .    :    .    :    .    :    .    :
Chain 1:  233 IMKFTEGAFKDWGYQLAREEFGGELIDGGPWLKVKNPNTGKEIVIKDVIADAFLQQILLRPAEYDVIACM
              1111111111111111111111                   11111111111111111111111111111
Chain 2:  186 IMKLGDGLFRNIITEIGQKEYP-------------------DIDVSSIIVDNASMQAVAKPHQFDVLVTP

                  .    :    .    :    .    :    .    :    .    :    .    :    .    :
Chain 1:  303 NLNGDYISDALAAQVGGIGIAPGANIGDECALFEATHGTAPKYAGQDKVNPGSIILSAEMMLRHMGWTEA
              1111111111111111111111111111111111111111111  1111111111111111111111111
Chain 2:  237 SMYGTILGNIGAALIGGPGLVAGANFGRDYAVFEPGSRHVGKG--QNVANPTAMILSSTLMLNHLGLNEY

                  .    :    .    :    .    :    .    :
Chain 1:  373 ADLIVKGMEGAINAKTVTYDFERLMDGAKLLKCSEFGDAIIENM
              11111111111111111111111     1111111111111111
Chain 2:  308 ATRISKAVHETIAEGKHTTRDIG-----GSSSTTDFTNEIINKL
```

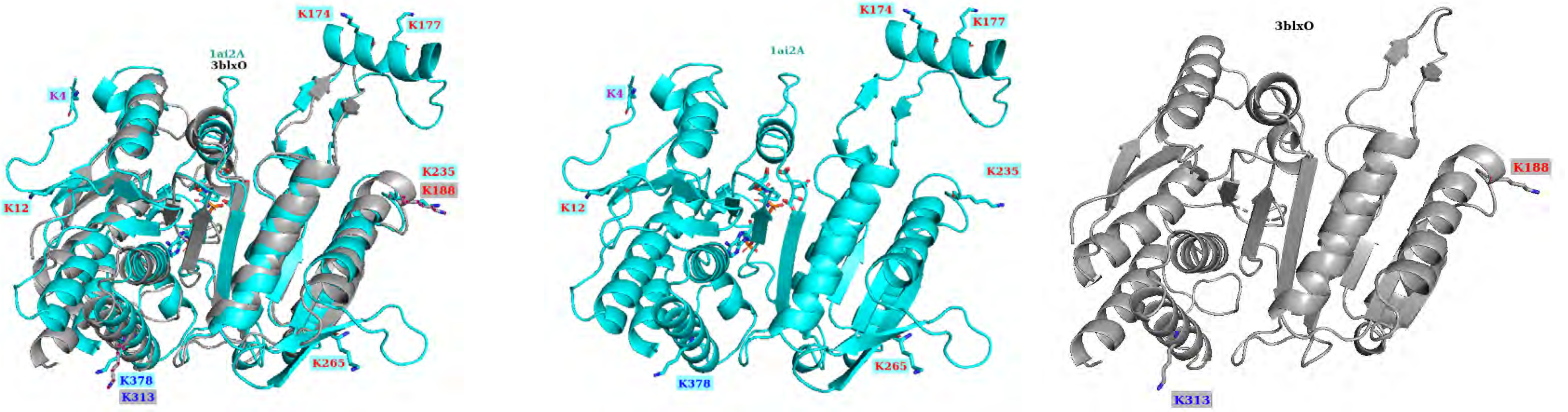

UniProt ID: P33197

PDB ID: 2D1C_B

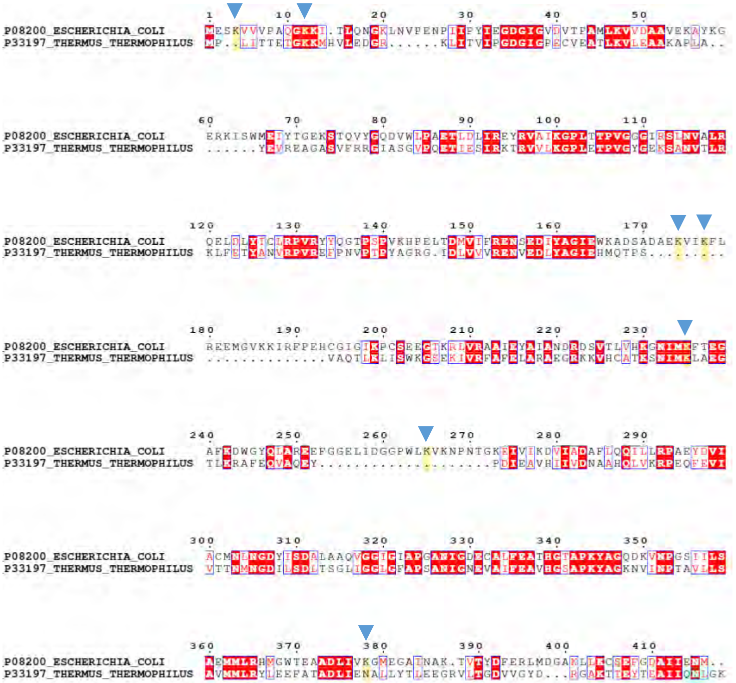


```
Align 1ai2.A.pdb 414 with 2d1c.B.pdb 495
Twists 0 ini-len 312 ini-rmsd 1.78 opt-equ 350 opt-rmsd 2.27 chain-rmsd 1.78 Score 841.44 align-len 409 gaps 59 (14.43%)
P-value 0.00e+00 Afp-num 62759 Identity 33.25% Similarity 48.66%
Block  0 afp 39 score 841.44 rmsd  1.78 gap 90 (0.22%)

                .    :    .    :    .    :    .    :    .    :    .    :    .    :
Chain 1:   12 KKITLQNGKLNVP---ENPIIPYIEGDGIGVDVTPAMLKVVDAAVEKAYKGERKISWMEIYTGEKSTQVY
              1111111111111   111111111111111111111111111111       111111111111111 1
Chain 2:    2 PLITTETGKKMHVLEDGRKLITVIPGDGIGPECVEATLKVLEAAKA-------PLAYEVREAGASVFR-R

                .    :    .    :    .    :    .    :    .    :    .    :    .    :
Chain 1:   79 GQDVWLPAETLDLIREYRVAIKGPLTTPVGGGIRSLNVALRQELDLYICLRPVRYYQGTPSPVKHPELTD
              111111111111111111111111111111111111111111111111111111111111 11111111
Chain 2:   64 GIASGVPQETIESIRKTRVVLKGPLETPVGYGEKSANVTLRKLFETYANVRPVREFPNVPT-PYAGRGID

                .    :    .    :    .    :    .    :    .    :    .    :    .    :
Chain 1:  149 MVIFRENSEDIYAGIEWKADSADAEKVIKFLREEMGVKKIRFPEHCGIGIKPCSEEGTKRLVRAAIEYAI
              111111111111111111111                       11111111111111111111111111
Chain 2:  133 LVVVRENVEDLYAGIEHMQTP-----------------------SVAQTLKLISWKGSEKIVRFAFELAR

                .    :    .    :    .    :    .    :    .    :    .    :    .    :
Chain 1:  219 ANDRDSVTLVHKGNIMKFTEGAFKDWGYQLAREEFGGELIDGGPWLKVKNPNTGKEIVIKDVIADAFLQQ
              11111111111111111111111111111111  111                  111111111111111
Chain 2:  180 AEGRKKVHCATKSNIMKLAEGTLKRAFEQVAQ--EYP------------------DIEAVHIIVDNAAHQ

                .    :    .    :    .    :    .    :    .    :    .    :    .    :
Chain 1:  289 ILLRPAEYDVIACMNLNGDYISDALAAQVGGIGIAPGANIGDECALFEATHGTAPKYAGQDKVNPGSIIL
              1111111111111111111111111111111111111111111111111111111111111111111111
Chain 2:  230 LVKRPEQFEVIVTTNMNGDILSDLTSGLIGGLGFAPSANIGNEVAIFEAVHGSAPKYAGKNVINPTAVLL

                .    :    .    :    .    :    .    :    .    :    .
Chain 1:  359 SAEMMLRHMGWTEAADLIVKGMEGAINAK-TVTYDFERLMDGAKLLKCSEFGDAIIENM
              1111111111111111111111111111 1111111111   1111111111111111
Chain 2:  300 SAVMMLRYLEEFATADLIENALLYTLEEGRVLTGDVVGYD---RGAKTTEYTEAIIQNL
```

Full sequences in supplemental file.

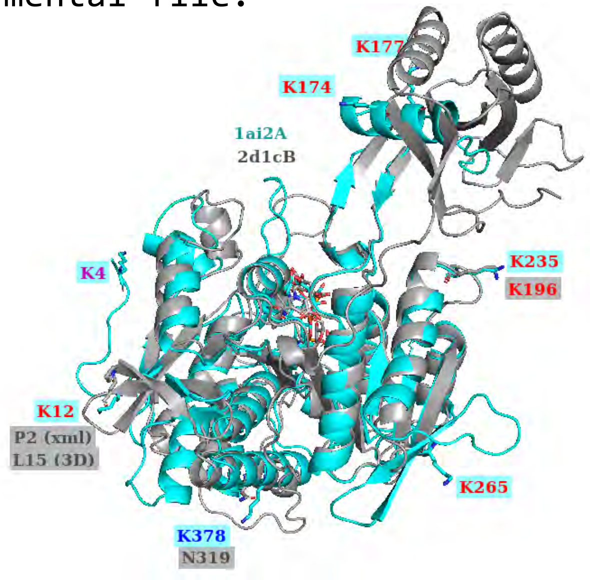

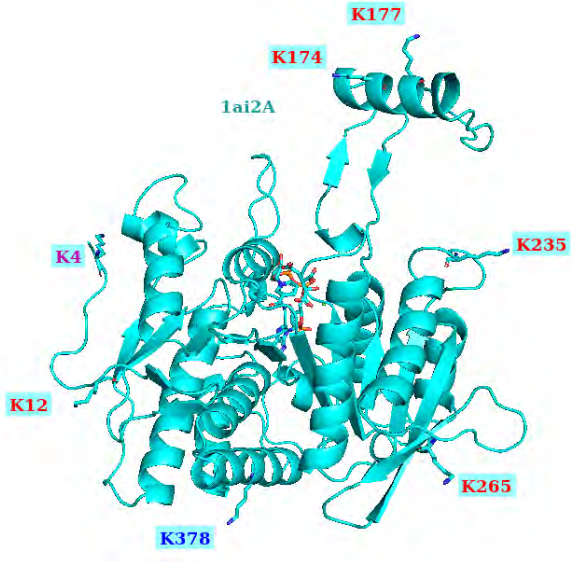

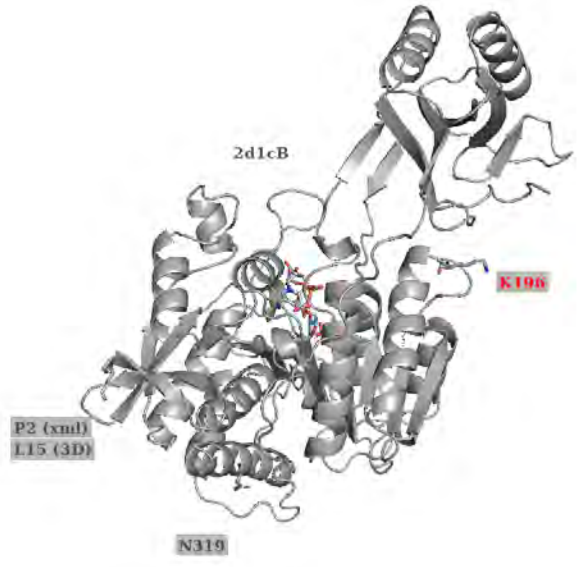

UniProt ID: P39126

PDB ID: 1HQS_B

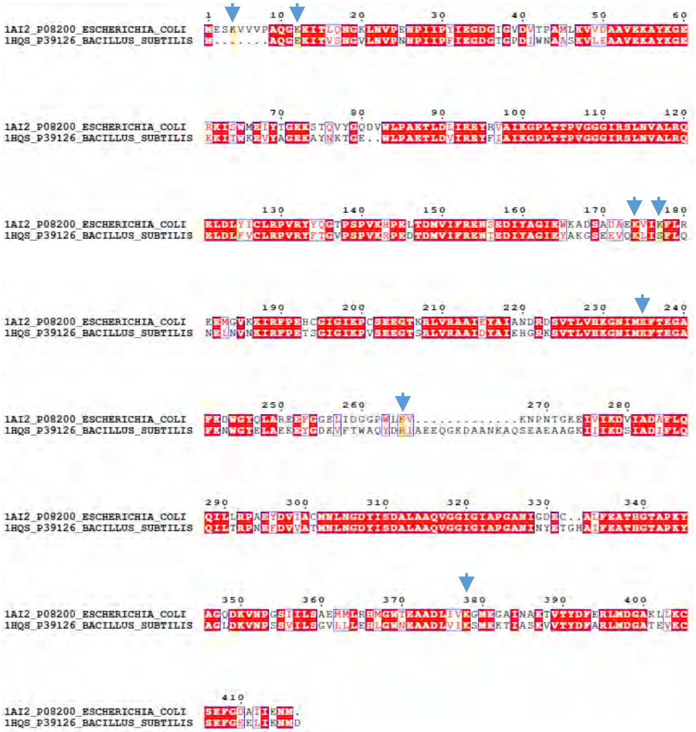

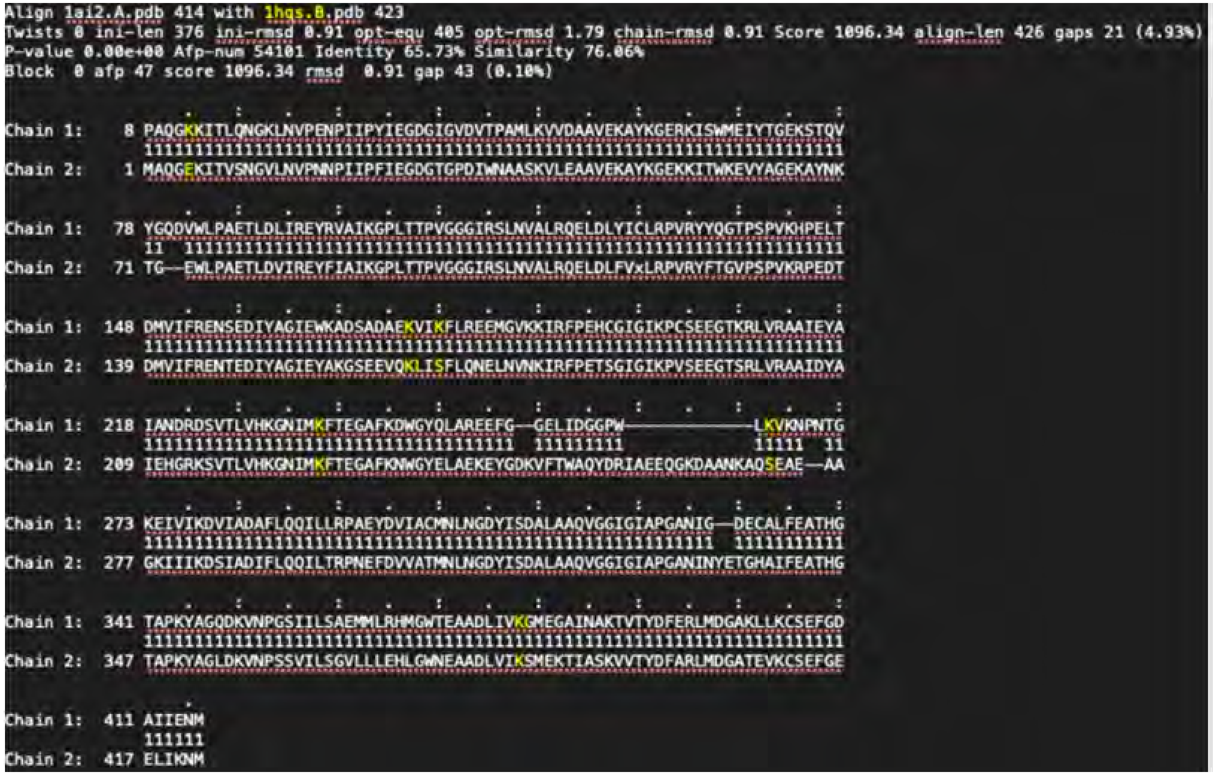

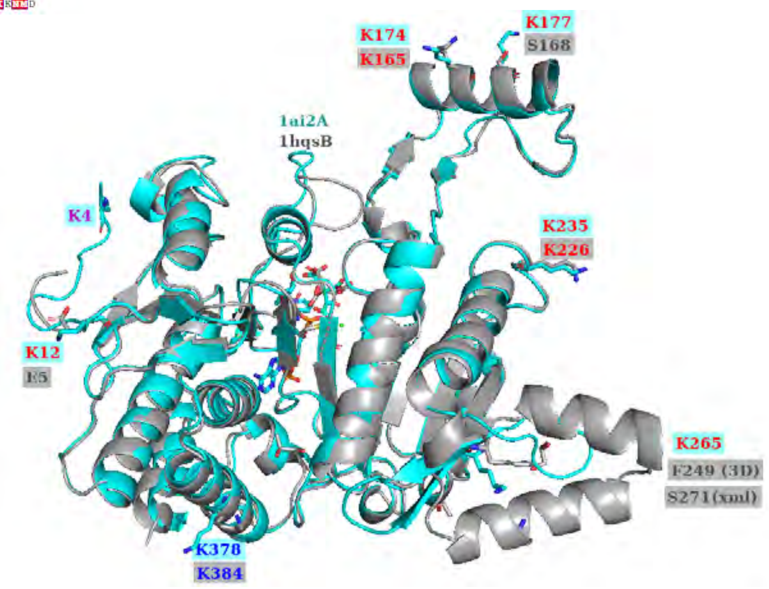

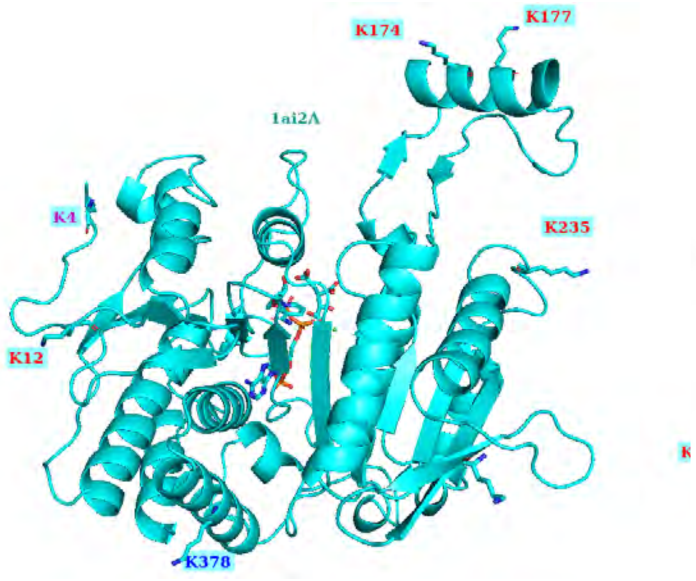

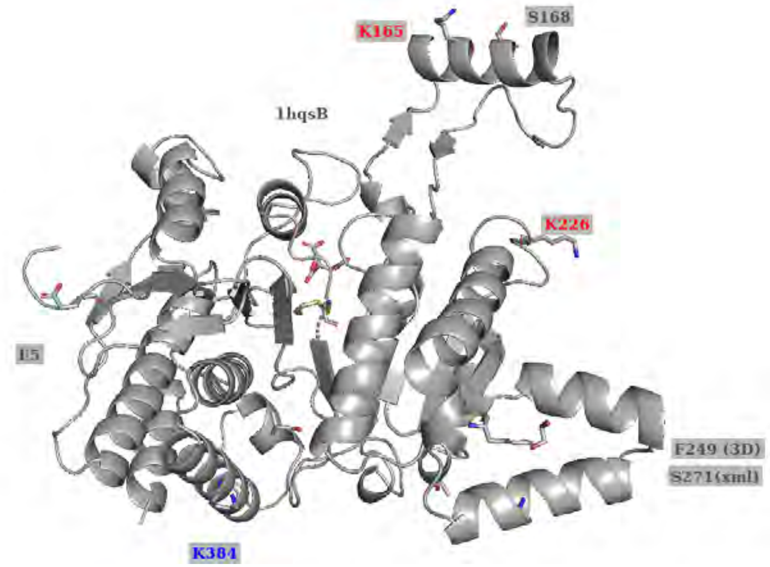

UniProt ID: P50213

PDB ID: 5GRE_A

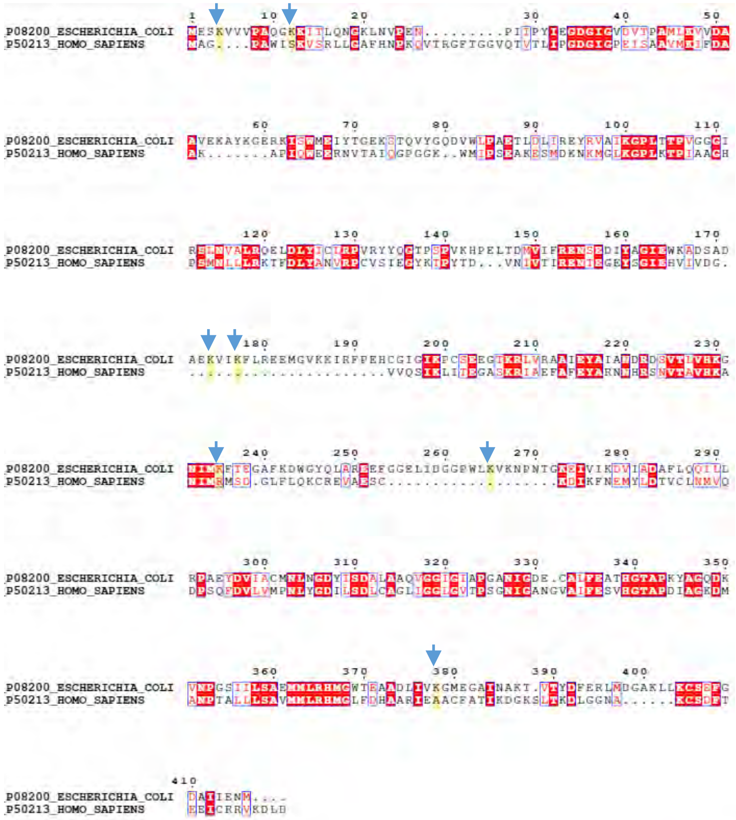

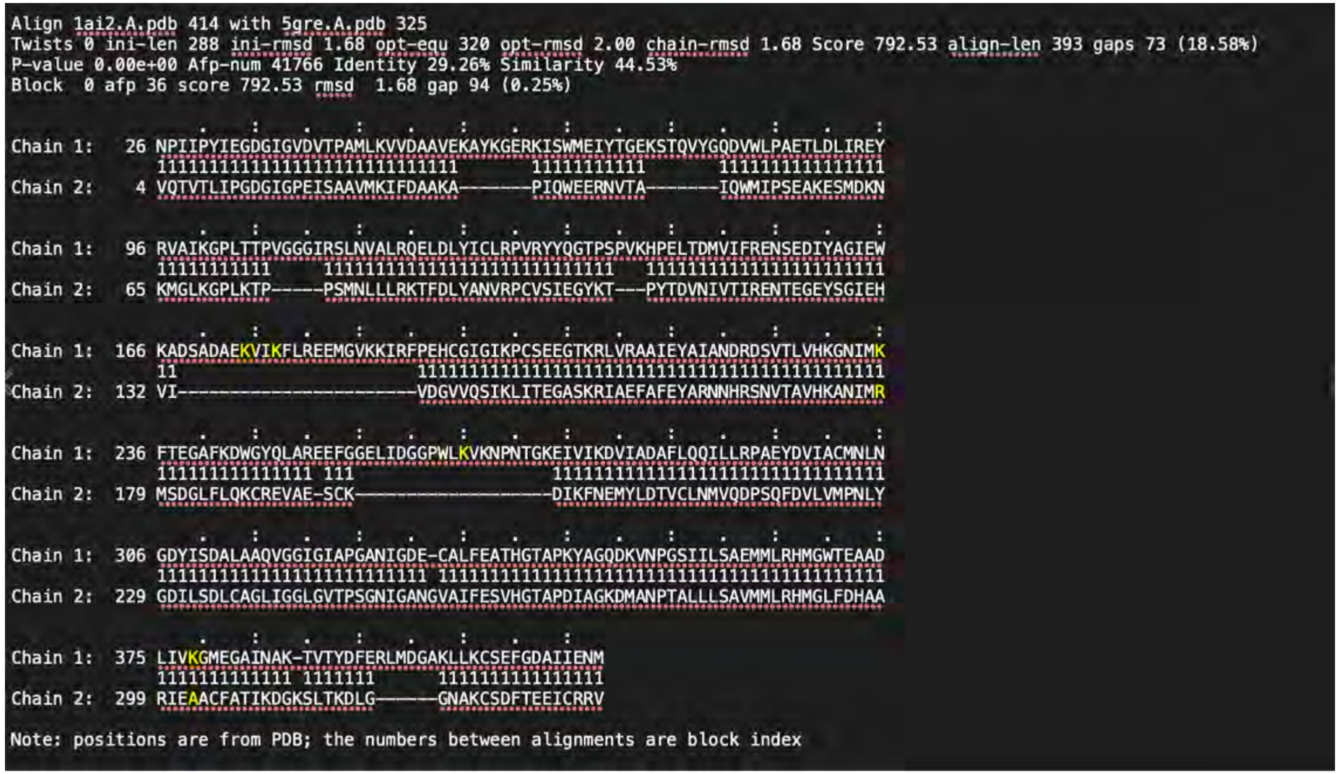

```
Align 1ai2.A.pdb 414 with 5gre.A.pdb 325
Twists 0 ini-len 288 ini-rmsd 1.68 opt-equ 320 opt-rmsd 2.00 chain-rmsd 1.68 Score 792.53 align-len 393 gaps 73 (18.58%)
P-value 0.00e+00 Afp-num 41766 Identity 29.26% Similarity 44.53%
Block  0 afp 36 score 792.53 rmsd  1.68 gap 94 (0.25%)

                       .    :    .    :    .    :    .    :    .    :    .    :    .    :
Chain 1:   26 NPIIPYIEGDGIGVDVTPAMLKVVDAAVEKAYKGERKISWMEIYTGEKSTQVYGQDVWLPAETLDLIREY
              11111111111111111111111111111       11111111111       1111111111111111
Chain 2:    4 VQTVTLIPGDGIGPEISAAVMKIFDAAKA-------PIQWEERNVTA-------IQWMIPSEAKESMDKN

                       .    :    .    :    .    :    .    :    .    :    .    :    .    :
Chain 1:   96 RVAIKGPLTTPVGGGIRSLNVALRQELDLYICLRPVRYYQGTPSPVKHPELTDMVIFRENSEDIYAGIEW
              11111111111     1111111111111111111111111111   11111111111111111111111
Chain 2:   65 KMGLKGPLKTP-----PSMNLLLRKTFDLYANVRPCVSIEGYKT---PYTDVNIVTIRENTEGEYSGIEH

                       .    :    .    :    .    :    .    :    .    :    .    :    .    :
Chain 1:  166 KADSADAEKVIKFLREEMGVKKIRFPEHCGIGIKPCSEEGTKRLVRAAIEYAIANDRDSVTLVHKGNIMK
              11                       111111111111111111111111111111111111111111111
Chain 2:  132 VI-----------------------VDGVVQSIKLITEGASKRIAEFAFEYARNNHRSNVTAVHKANIMR

                       .    :    .    :    .    :    .    :    .    :    .    :    .    :
Chain 1:  236 FTEGAFKDWGYQLAREEFGGELIDGGPWLKVKNPNTGKEIVIKDVIADAFLQQILLRPAEYDVIACMNLN
              111111111111111 111                   11111111111111111111111111111111
Chain 2:  179 MSDGLFLQKCREVAE-SCK-------------------DIKFNEMYLDTVCLNMVQDPSQFDVLVMPNLY

                       .    :    .    :    .    :    .    :    .    :    .    :    .    :
Chain 1:  306 GDYISDALAAQVGGIGIAPGANIGDE-CALFEATHGTAPKYAGQDKVNPGSIILSAEMMLRHMGWTEAAD
              11111111111111111111111111 1111111111111111111111111111111111111111111
Chain 2:  229 GDILSDLCAGLIGGLGVTPSGNIGANGVAIFESVHGTAPDIAGKDMANPTALLLSAVMMLRHMGLFDHAA

                       .    :    .    :    .    :    .    :
Chain 1:  375 LIVKGMEGAINAK-TVTYDFERLMDGAKLLKCSEFGDAIIENM
              11111111111111 1111111     1111111111111111
Chain 2:  299 RIEAACFATIKDGKSLTKDLG------GNAKCSDFTEEICRRV

Note: positions are from PDB; the numbers between alignments are block index
```

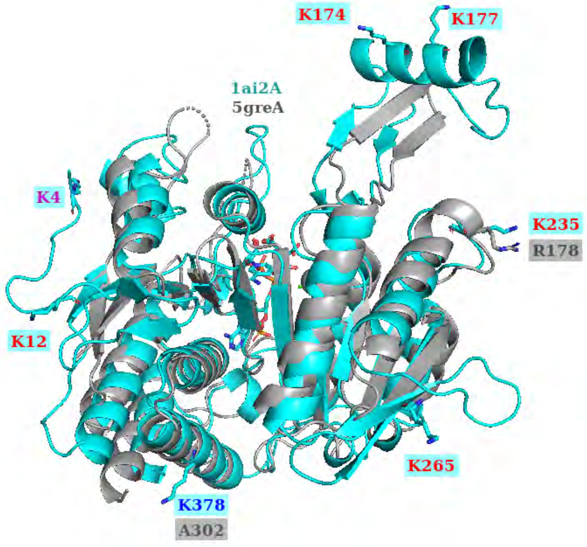

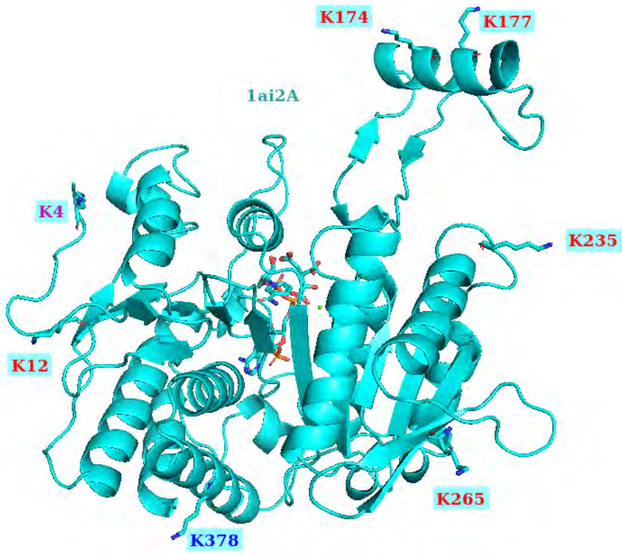

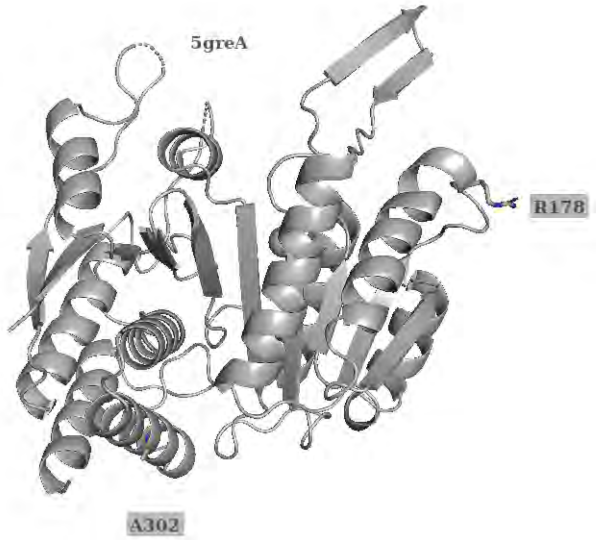

UniProt ID: P50213

PDB ID: 5GRF_A

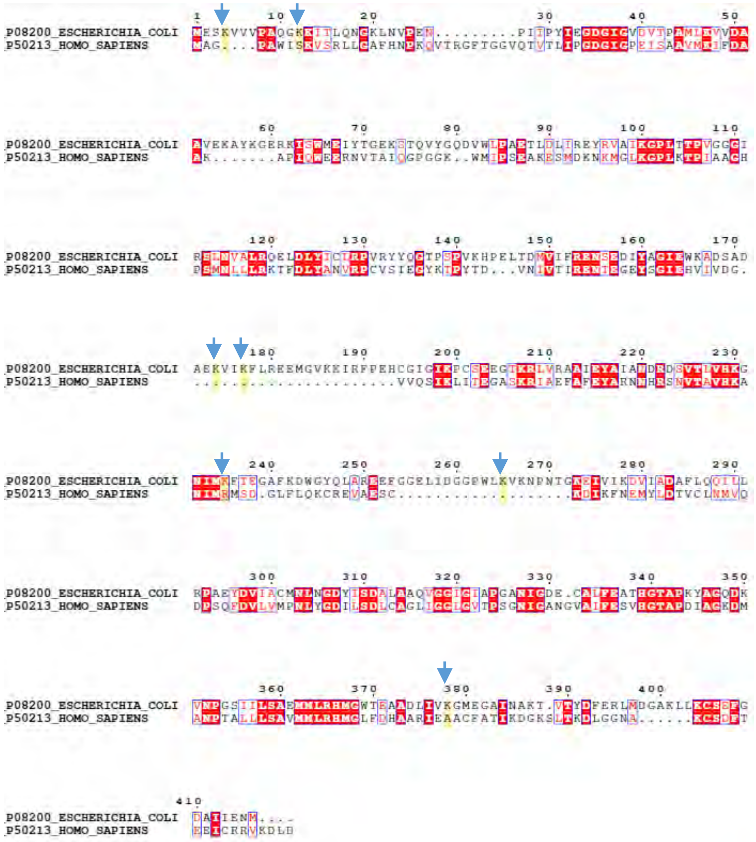

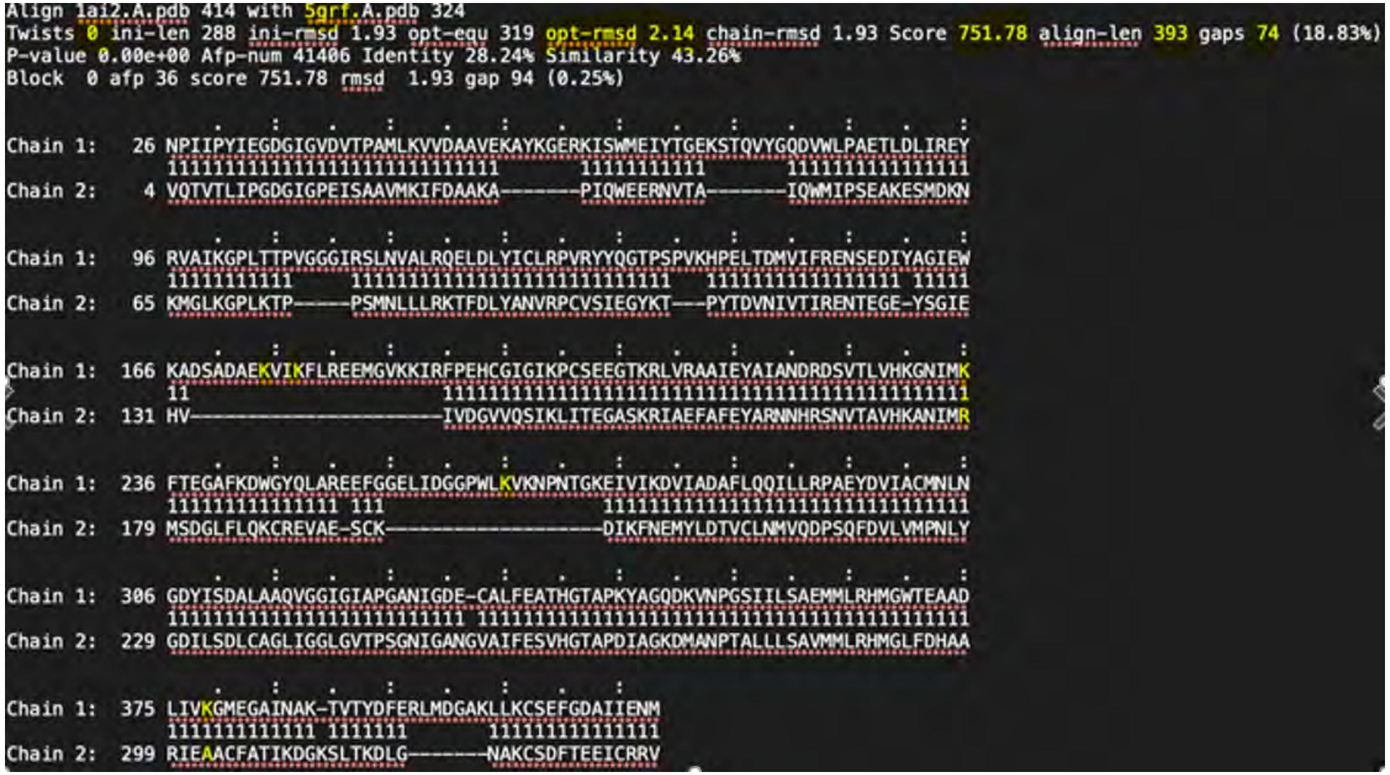

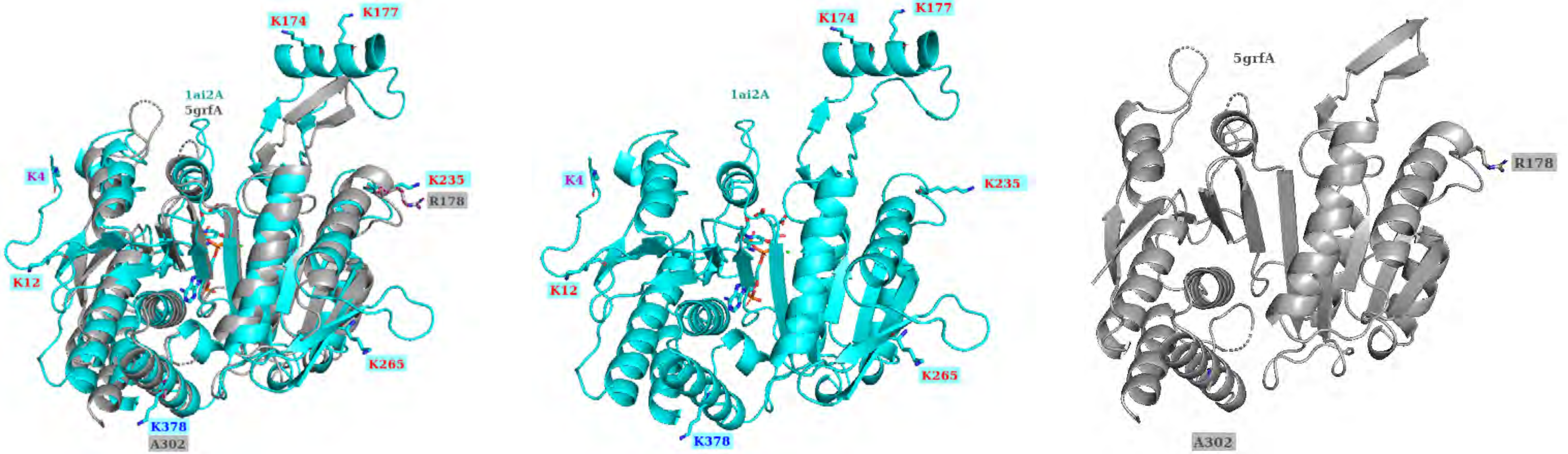

UniProt ID: P50213

PDB ID: 5GRH_A

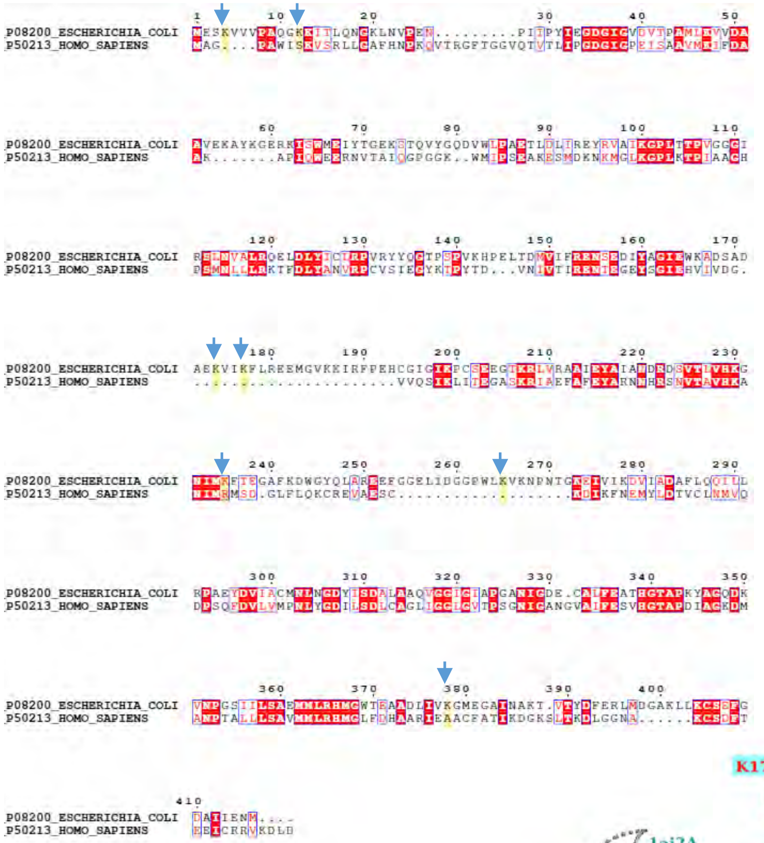

```
Align 1ai2.A.pdb 414 with 5grh.A.pdb 327
Twists 0 ini-len 280 ini-rmsd 1.66 opt-equ 324 opt-rmsd 2.27 chain-rmsd 1.66 Score 765.27 align-len 393 gaps 69 (17.56%)
P-value 0.00e+00 Afp-num 42040 Identity 28.24% Similarity 43.26%
Block  0 afp 35 score 765.27 rmsd  1.66 gap 101 (0.27%)

                   .    :    .    :    .    :    .    :    .    :    .    :    .    :
Chain 1:   25 ENPIIPYIEGDGIGVDVTPAMLKVVDAAVEKAYKGERKISWMEIYTGEKSTQVYGQDVWLPAETLDLIRE
              111111111111111111111111111111       11111111111     11111111111111111
Chain 2:    3 GVQTVTLIPGDGIGPEISAAVMKIFDAAKA-------PIQWEERNVTA-----IQGKWMIPSEAKESMDK

                   .    :    .    :    .    :    .    :    .    :    .    :    .    :
Chain 1:   95 YRVAIKGPLTTPVGGGIRSLNVALRQELDLYICLRPVRYYQGTPSPVKHPELTDMVIFRENSEDIYAGIE
              111111111111    11111111111111111111111111111   1111111111111111 11111
Chain 2:   64 NKMGLKGPLKTP----HPSMNLLLRKTFDLYANVRPCVSIEGYKT---PYTDVNIVTIRENTEG-EYSGI

                   .    :    .    :    .    :    .    :    .    :    .    :    .    :
Chain 1:  165 WKADSADAEKVIKFLREEMGVKKIRFPEHCGIGIKPCSEEGTKRLVRAAIEYAIANDRDSVTLVHKGNIM
              1111                      11111111111111111111111111111111111111111111
Chain 2:  130 EHVI----------------------VDGVVQSIKLITEGASKRIAEFAFEYARNNHRSNVTAVHKANIM

                   .    :    .    :    .    :    .    :    .    :    .    :    .    :
Chain 1:  235 KFTEGAFKDWGYQLAREEFGGELIDGGPWLKVKNPNTGKEIVIKDVIADAFLQQILLRPAEYDVIACMNL
              1111111111111111 111                   1111111111111111111111111111111
Chain 2:  178 RMSDGLFLQKCREVAE-SCK-------------------DIKFNEMYLDTVCLNMVQDPSQFDVLVMPNL

                   .    :    .    :    .    :    .    :    .    :    .    :    .    :
Chain 1:  305 NGDYISDALAAQVGGIGIAPGANIGDE-CALFEATHGTAPKYAGQDKVNPGSIILSAEMMLRHMGWTEAA
              111111111111111111111111111 111111111111111111111111111111111111111111
Chain 2:  228 YGDILSDLCAGLIGGLGVTPSGNIGANGVAIFESVHGTAPDIAGKDMANPTALLLSAVMMLRHMGLFDHA

                   .    :    .    :    .    :    .    :
Chain 1:  374 DLIVKGMEGAINAKTVTYDFERLMDGAKLLKCSEFGDAIIENM
              111111111111111111111      1111111111111111
Chain 2:  298 ARIEAACFATIKDGSLTKDLG------GNAKCSDFTEEICRRV
```

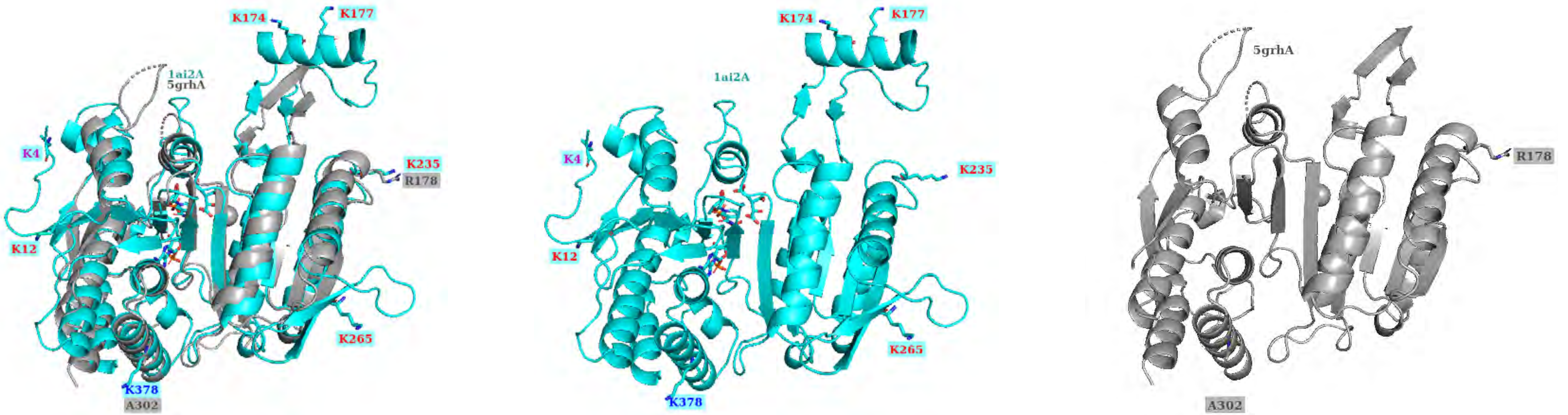

UniProt ID: P50213

PDB ID: 5GRI_A

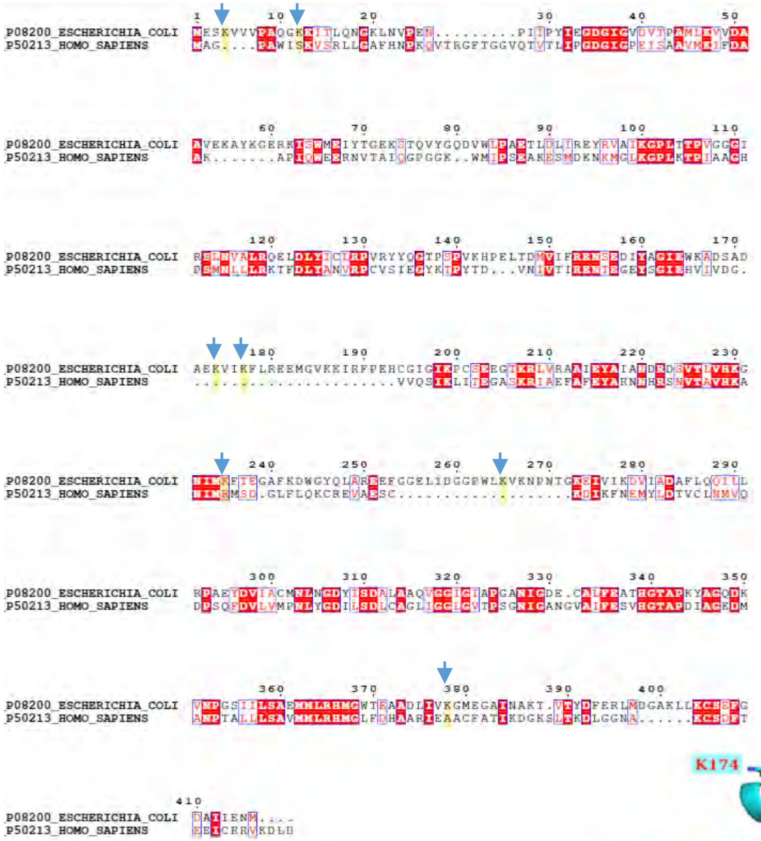


```
Align 1ai2.A.pdb 414 with 5gri.A.pdb 323
Twists 0 ini-len 288 ini-rmsd 1.91 opt-equ 318 opt-rmsd 2.08 chain-rmsd 1.91 Score 774.96 align-len 393 gaps 75 (19.08%)
P-value 0.00e+00 Afp-num 40931 Identity 29.26% Similarity 44.53%
Block  0 afp 36 score 774.96 rmsd  1.91 gap 100 (0.26%)

                   .    :    .    :    .    :    .    :    .    :    .    :    .    :
Chain 1:   26 NPIIPYIEGDGIGVDVTPAMLKVVDAAVEKAYKGERKISWMEIYTGEKSTQVYGQDVWLPAETLDLIREY
              11111111111111111111111111111       11111111111       1111111111111111
Chain 2:    4 VQTVTLIPGDGIGPEISAAVMKIFDAAKA-------PIQWEERNVTA-------IQWMIPSEAKESMDKN

                   .    :    .    :    .    :    .    :    .    :    .    :    .    :
Chain 1:   96 RVAIKGPLTTPVGGGIRSLNVALRQELDLYICLRPVRYYQGTPSPVKHPELTDMVIFRENSEDIYAGIEW
              1111111111      11111111111111111111111111111   1111111111111111111111
Chain 2:   65 KMGLKGPLKT------PSMNLLLRKTFDLYANVRPCVSIEGYKTP---YTDVNIVTIRENTEGEYSGIEH

                   .    :    .    :    .    :    .    :    .    :    .    :    .    :
Chain 1:  166 KADSADAEKVIKFLREEMGVKKIRFPEHCGIGIKPCSEEGTKRLVRAAIEYAIANDRDSVTLVHKGNIMK
                    11                 111111111111111111111111111111111111111111111
Chain 2:  132 ------VI-----------------VDGVVQSIKLITEGASKRIAEFAFEYARNNHRSNVTAVHKANIMR

                   .    :    .    :    .    :    .    :    .    :    .    :    .    :
Chain 1:  236 FTEGAFKDWGYQLAREEFGGELIDGGPWLKVKNPNTGKEIVIKDVIADAFLQQILLRPAEYDVIACMNLN
              111111111111111 111                   11111111111111111111111111111111
Chain 2:  179 MSDGLFLQKCREVAE-SCK-------------------DIKFNEMYLDTVCLNMVQDPSQFDVLVMPNLY

                   .    :    .    :    .    :    .    :    .    :    .    :    .    :
Chain 1:  306 GDYISDALAAQVGGIGIAPGANIGDE-CALFEATHGTAPKYAGQDKVNPGSIILSAEMMLRHMGWTEAAD
              11111111111111111111111111 1111111111111111111111111111111111111111111
Chain 2:  229 GDILSDLCAGLIGGLGVTPSGNIGANGVAIFESVHGTAPDIAGKDMANPTALLLSAVMMLRHMGLFDHAA

                   .    :    .    :    .    :    .    :
Chain 1:  375 LIVKGMEGAINAK-TVTYDFERLMDGAKLLKCSEFGDAIIENM
              11111111111111 1111111      111111111111111
Chain 2:  299 RIEAACFATIKDGKSLTKDLG-------NAKCSDFTEEICRRV
```

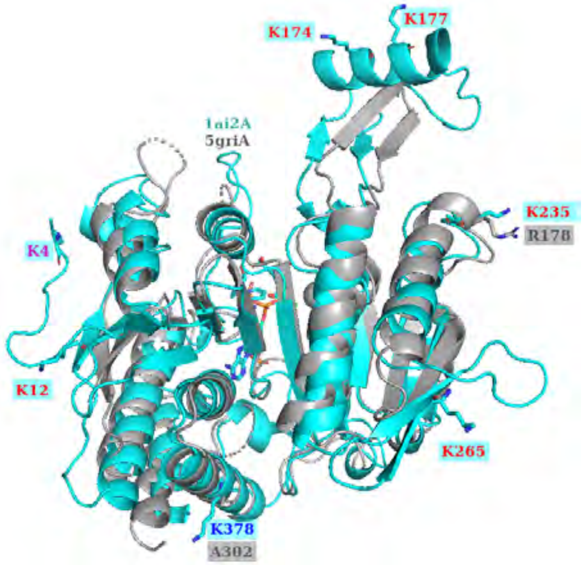

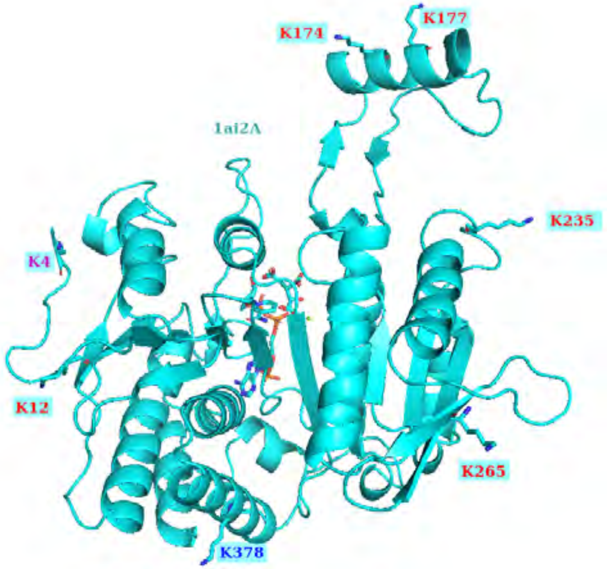

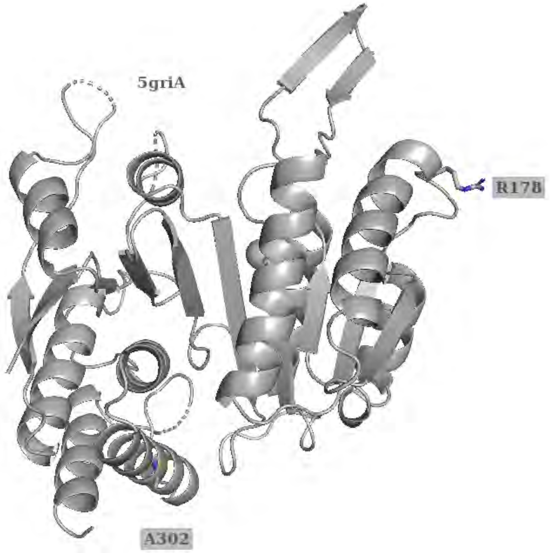

UniProt ID: P50213

PDB ID: 5GRL_A

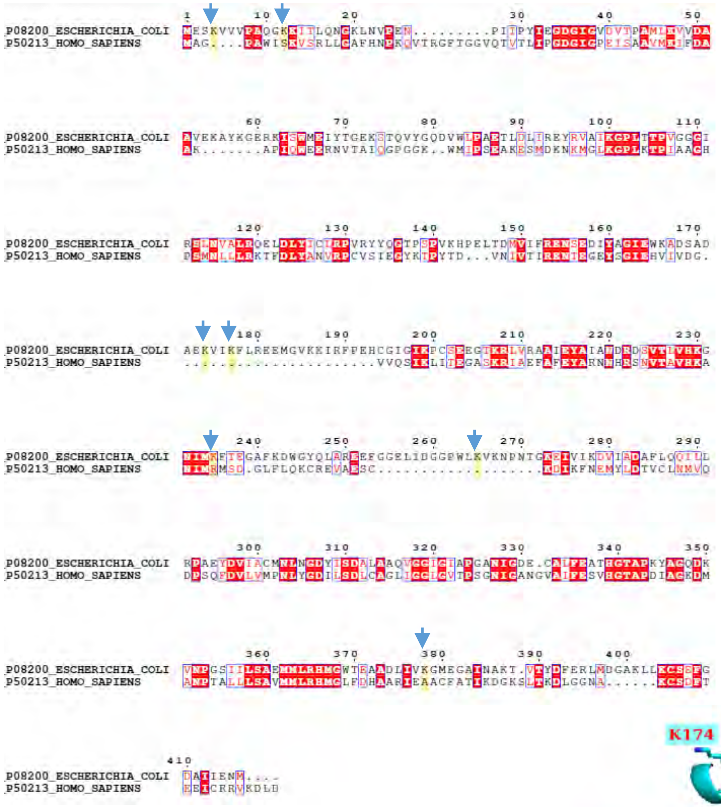

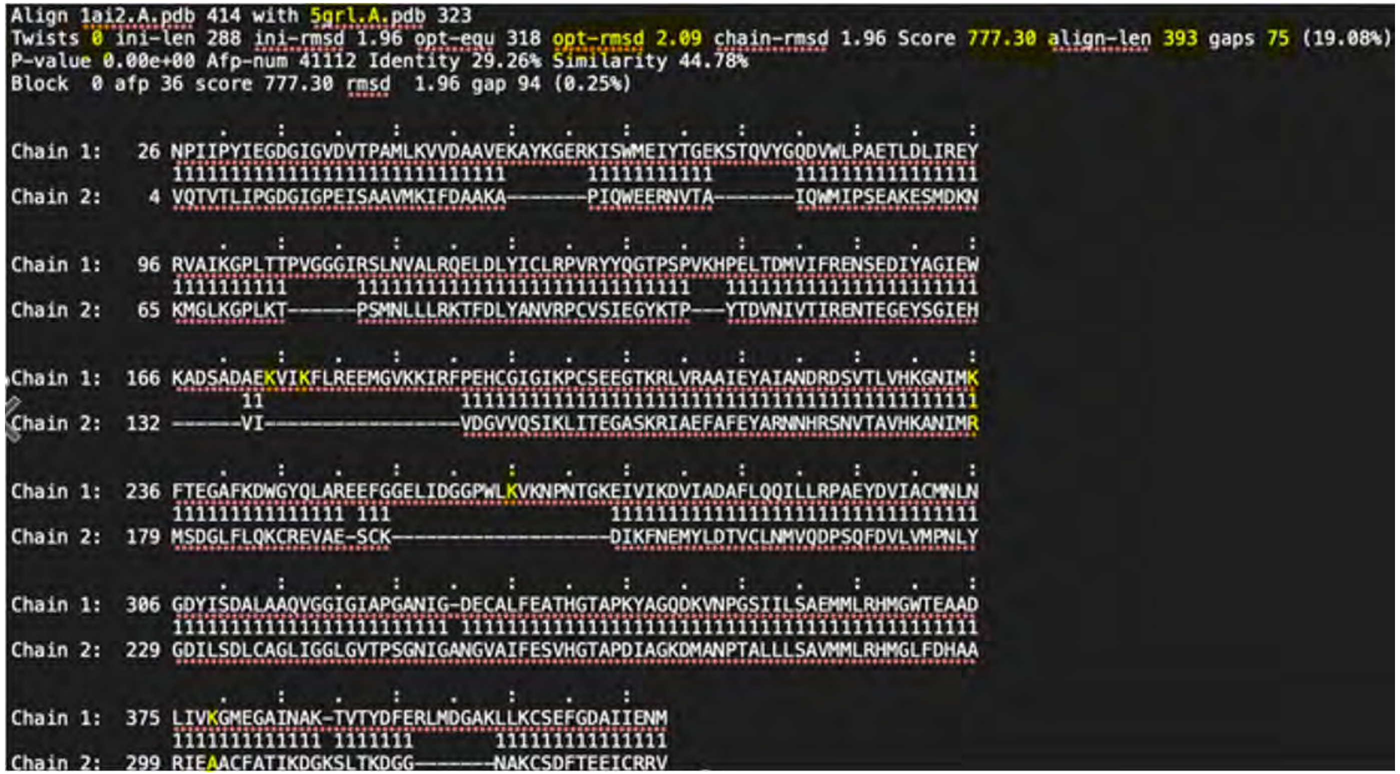

```
Align 1ai2.A.pdb 414 with 5grl.A.pdb 323
Twists 0 ini-len 288 ini-rmsd 1.96 opt-equ 318 opt-rmsd 2.09 chain-rmsd 1.96 Score 777.30 align-len 393 gaps 75 (19.08%)
P-value 0.00e+00 Afp-num 41112 Identity 29.26% Similarity 44.78%
Block  0 afp 36 score 777.30 rmsd  1.96 gap 94 (0.25%)

              .    :    .    :    .    :    .    :    .    :    .    :    .    :
Chain 1:   26 NPIIPYIEGDGIGVDVTPAMLKVVDAAVEKAYKGERKISWMEIYTGEKSTQVYGQDVWLPAETLDLIREY
              11111111111111111111111111111              11111111111        1111111111111111
Chain 2:    4 VQTVTLIPGDGIGPEISAAVMKIFDAAKA--------PIQWEERNVTA--------IQWMIPSEAKESMDKN

              .    :    .    :    .    :    .    :    .    :    .    :    .    :
Chain 1:   96 RVAIKGPLTTPVGGGIRSLNVALRQELDLYICLRPVRYYQGTPSPVKHPELTDMVIFRENSEDIYAGIEW
              1111111111      11111111111111111111111111111111   111111111111111111111111
Chain 2:   65 KMGLKGPLKT------PSMNLLLRKTFDLYANVRPCVSIEGYKTP---YTDVNIVTIRENTEGEYSGIEH

              .    :    .    :    .    :    .    :    .    :    .    :    .    :
Chain 1:  166 KADSADAEKVIKFLREEMGVKKIRFPEHCGIGIKPCSEEGTKRLVRAAIEYAIANDRDSVTLVHKGNIMK
                     11                  11111111111111111111111111111111111111111111
Chain 2:  132 ------VI-----------------VDGVVQSIKLITEGASKRIAEFAFEYARNNHRSNVTAVHKANIMR

              .    :    .    :    .    :    .    :    .    :    .    :    .    :
Chain 1:  236 FTEGAFKDWGYQLAREEFGGELIDGGPWLKVKNPNTGKEIVIKDVIADAFLQQILLRPAEYDVIACMNLN
              111111111111111 111                  11111111111111111111111111111111111
Chain 2:  179 MSDGLFLQKCREVAE-SCK------------------DIKFNEMYLDTVCLNMVQDPSQFDVLVMPNLY

              .    :    .    :    .    :    .    :    .    :    .    :    .    :
Chain 1:  306 GDYISDALAAQVGGIGIAPGANIG-DECALFEATHGTAPKYAGQDKVNPGSIILSAEMMLRHMGWTEAAD
              1111111111111111111111111 11111111111111111111111111111111111111111111111
Chain 2:  229 GDILSDLCAGLIGGLGVTPSGNIGANGVAIFESVHGTAPDIAGKDMANPTALLLSAVMMLRHMGLFDHAA

              .    :    .    :    .    :    .    :
Chain 1:  375 LIVKGMEGAINAK-TVTYDFERLMDGAKLLKCSEFGDAIIENM
              11111111111111 1111111       111111111111111
Chain 2:  299 RIEAACFATIKDGKSLTKDGG-------NAKCSDFTEEICRRV
```

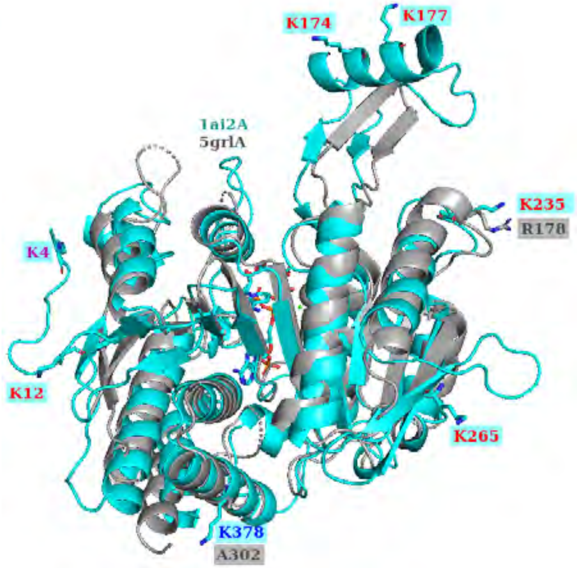

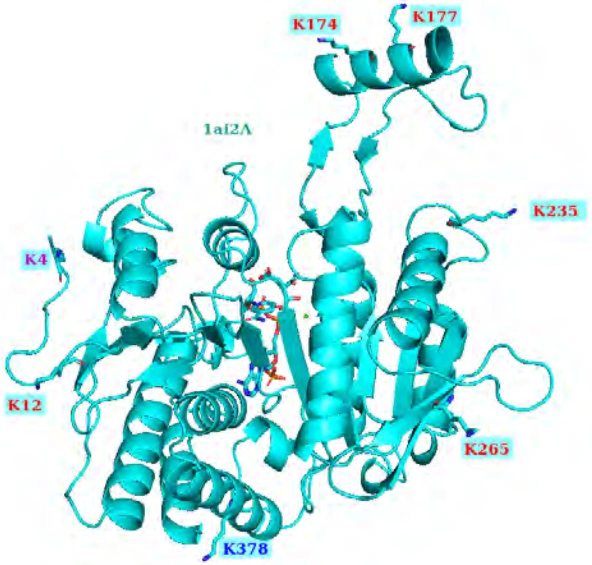

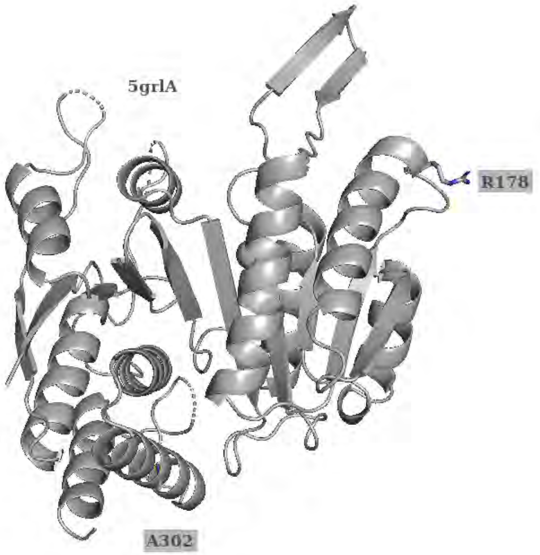

UniProt ID: P50213

PDB ID: 5YVT_A

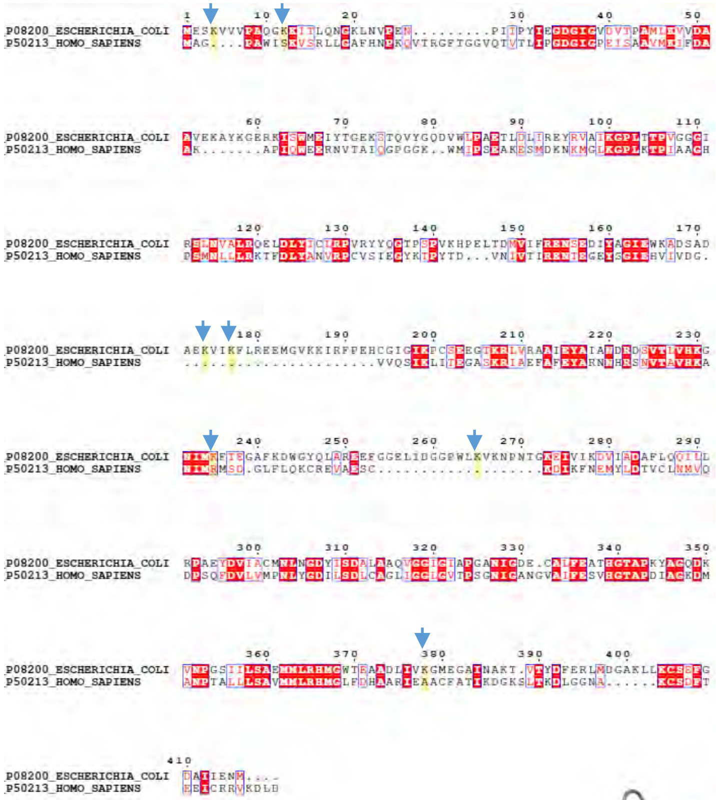

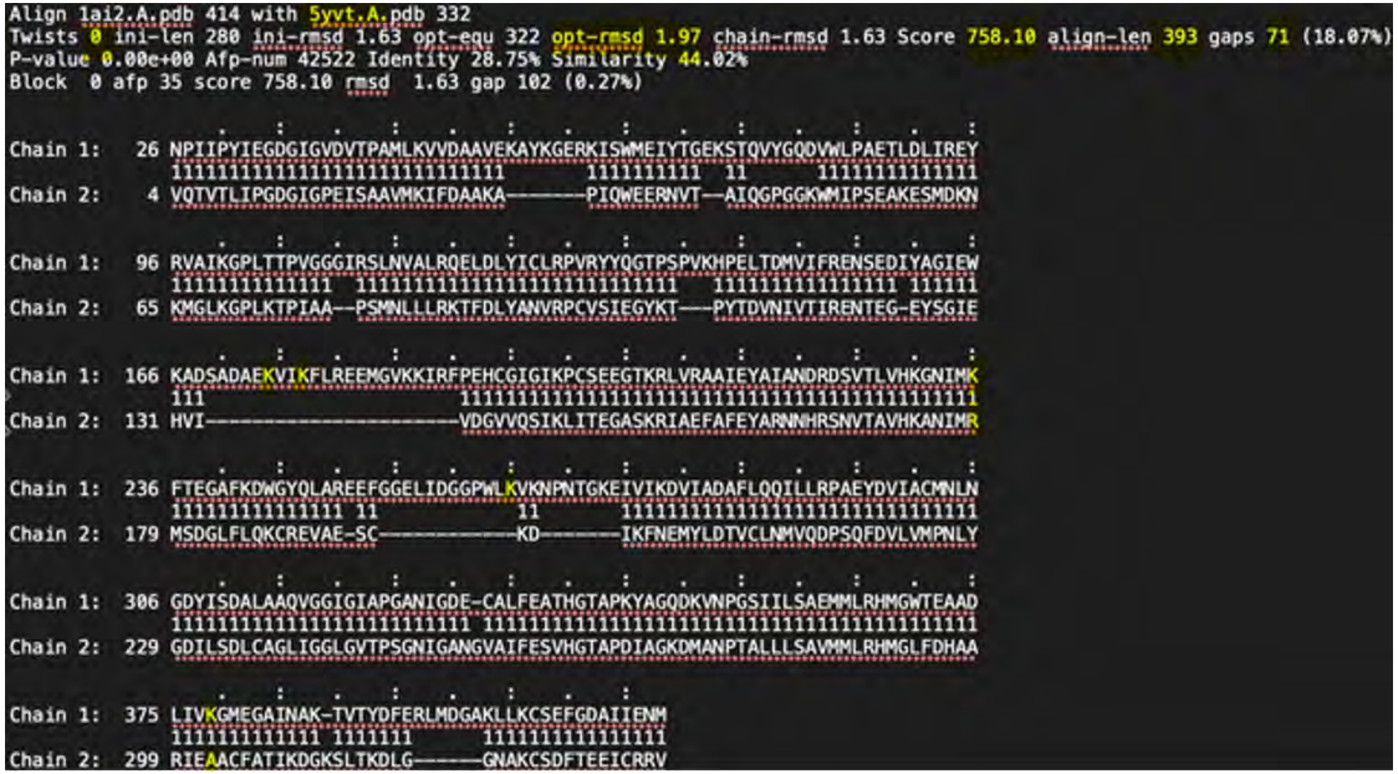

```
Align 1ai2.A.pdb 414 with 5yvt.A.pdb 332
Twists 0 ini-len 280 ini-rmsd 1.63 opt-equ 322 opt-rmsd 1.97 chain-rmsd 1.63 Score 758.10 align-len 393 gaps 71 (18.07%)
P-value 0.00e+00 Afp-num 42522 Identity 28.75% Similarity 44.02%
Block  0 afp 35 score 758.10 rmsd  1.63 gap 102 (0.27%)

                   .    :    .    :    .    :    .    :    .    :    .    :    .    :
Chain 1:   26 NPIIPYIEGDGIGVDVTPAMLKVVDAAVEKAYKGERKISWMEIYTGEKSTQVYGQDVWLPAETLDLIREY
                11111111111111111111111111111       1111111111 11       11111111111111
Chain 2:    4 VQTVTLIPGDGIGPEISAAVMKIFDAAKA-------PIQWEERNVT--AIQGPGGKWMIPSEAKESMDKN

                   .    :    .    :    .    :    .    :    .    :    .    :    .    :
Chain 1:   96 RVAIKGPLTTPVGGGIRSLNVALRQELDLYICLRPVRYYQGTPSPVKHPELTDMVIFRENSEDIYAGIEW
                11111111111111  1111111111111111111111111111   1111111111111111 111111
Chain 2:   65 KMGLKGPLKTPIAA--PSMNLLLRKTFDLYANVRPCVSIEGYKT---PYTDVNIVTIRENTEG-EYSGIE

                   .    :    .    :    .    :    .    :    .    :    .    :    .    :
Chain 1:  166 KADSADAEKVIKFLREEMGVKKIRFPEHCGIGIKPCSEEGTKRLVRAAIEYAIANDRDSVTLVHKGNIMK
                111                      111111111111111111111111111111111111111111111
Chain 2:  131 HVI----------------------VDGVVQSIKLITEGASKRIAEFAFEYARNNHRSNVTAVHKANIMR

                   .    :    .    :    .    :    .    :    .    :    .    :    .    :
Chain 1:  236 FTEGAFKDWGYQLAREEFGGELIDGGPWLKVKNPNTGKEIVIKDVIADAFLQQILLRPAEYDVIACMNLN
                111111111111111 11            11       1111111111111111111111111111111
Chain 2:  179 MSDGLFLQKCREVAE-SC------------KD-------IKFNEMYLDTVCLNMVQDPSQFDVLVMPNLY

                   .    :    .    :    .    :    .    :    .    :    .    :    .    :
Chain 1:  306 GDYISDALAAQVGGIGIAPGANIGDE-CALFEATHGTAPKYAGQDKVNPGSIILSAEMMLRHMGWTEAAD
                11111111111111111111111111 1111111111111111111111111111111111111111111
Chain 2:  229 GDILSDLCAGLIGGLGVTPSGNIGANGVAIFESVHGTAPDIAGKDMANPTALLLSAVMMLRHMGLFDHAA

                   .    :    .    :    .    :    .    :
Chain 1:  375 LIVKGMEGAINAK-TVTYDFERLMDGAKLLKCSEFGDAIIENM
                11111111111111 1111111     1111111111111111
Chain 2:  299 RIEAACFATIKDGKSLTKDLG------GNAKCSDFTEEICRRV
```

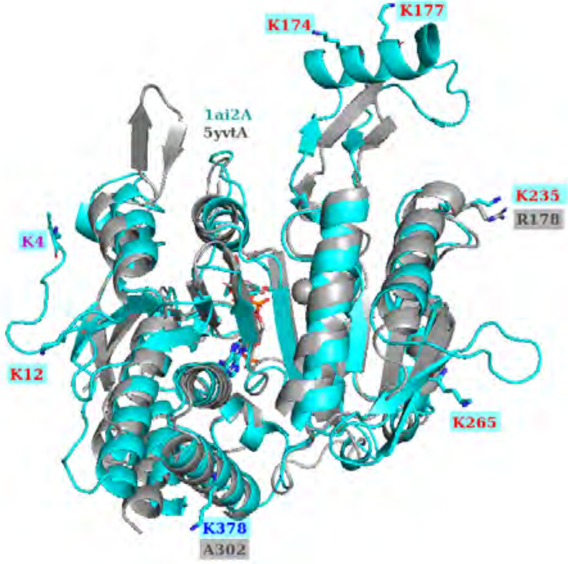

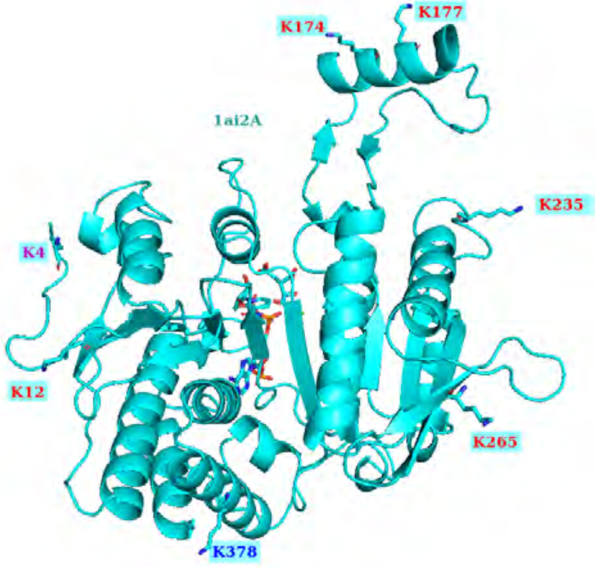

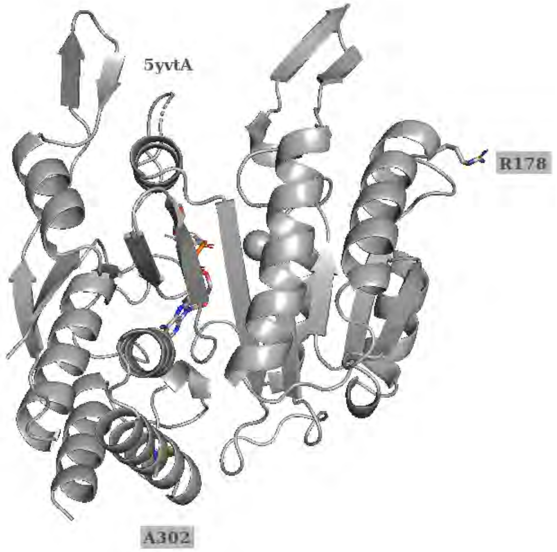

UniProt ID: P50213

PDB ID: 6KDE_C

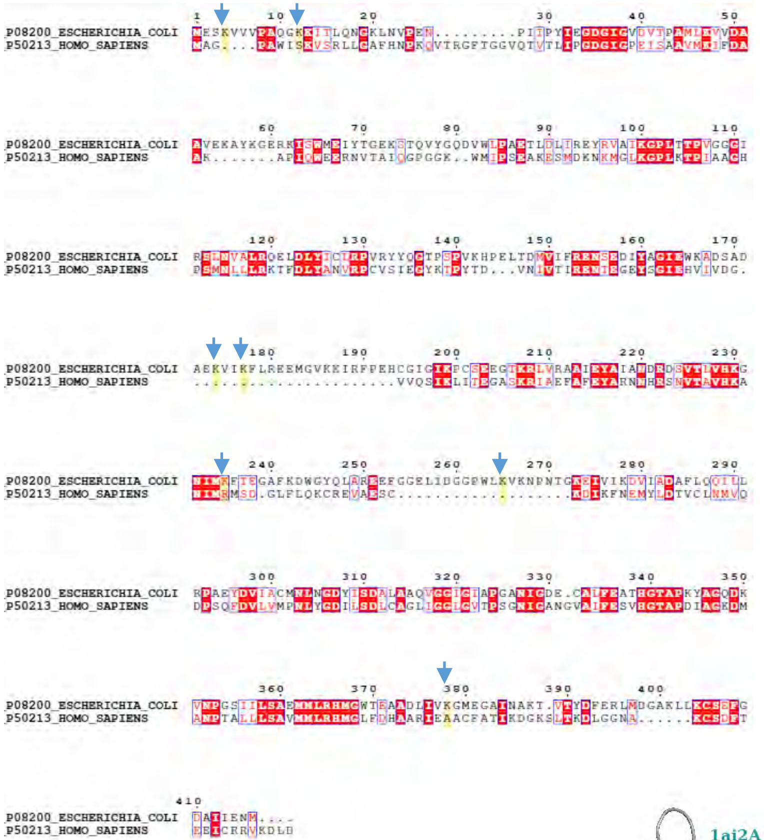

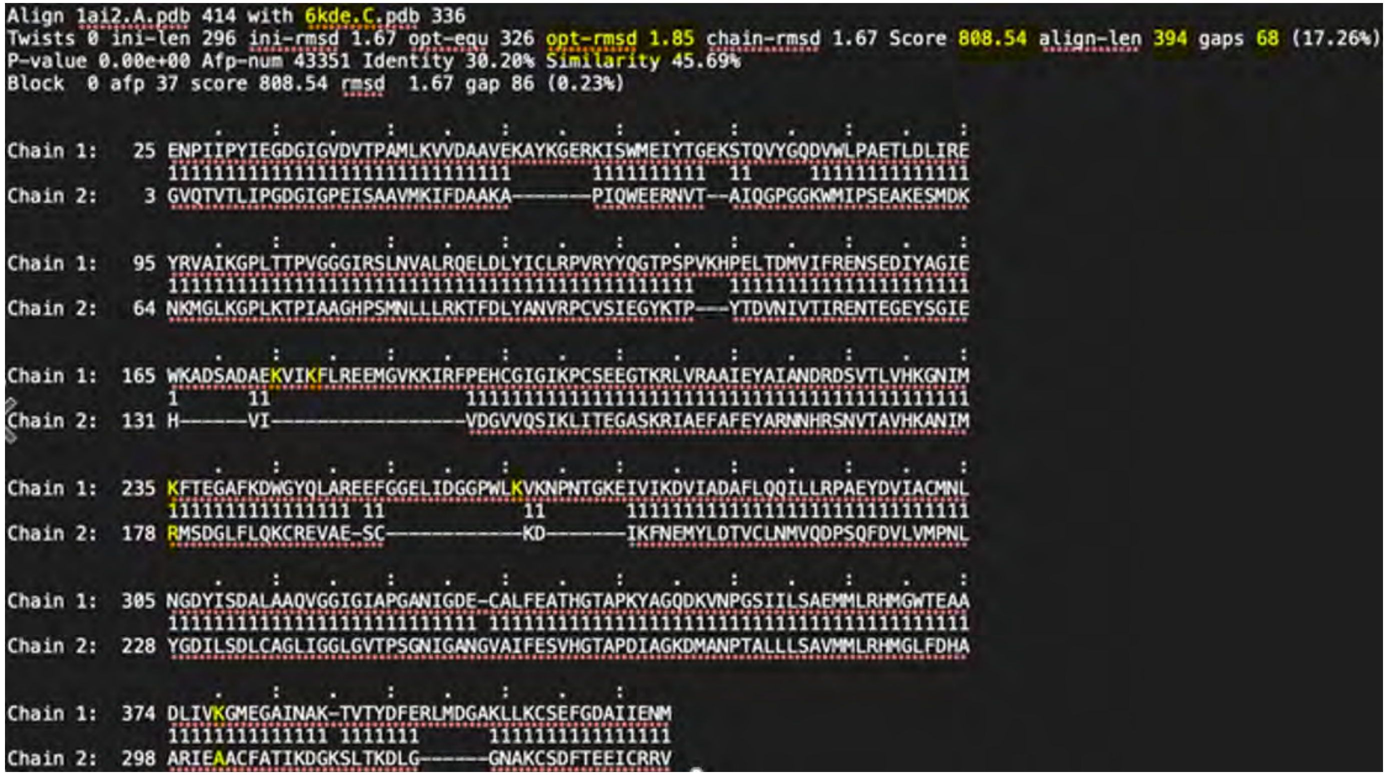

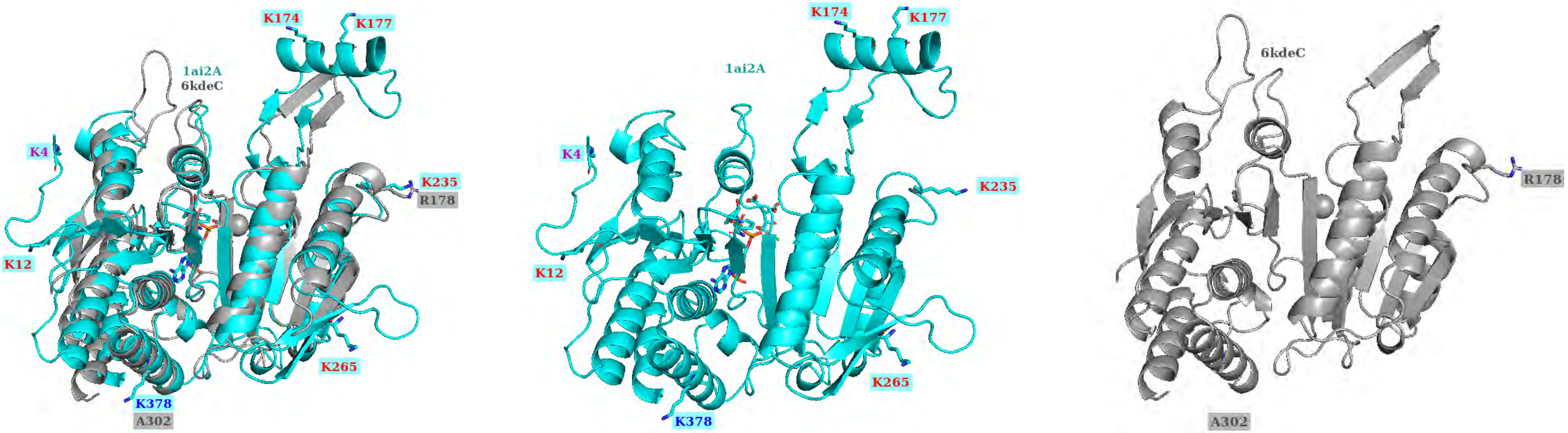

UniProt ID: P50213
PDB ID: 6KDF_I

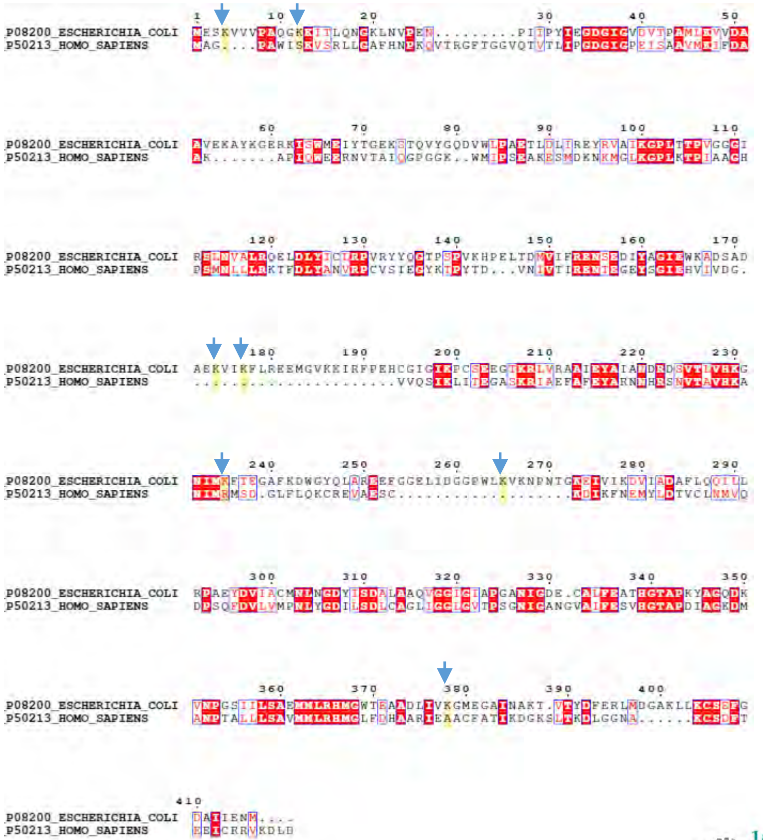

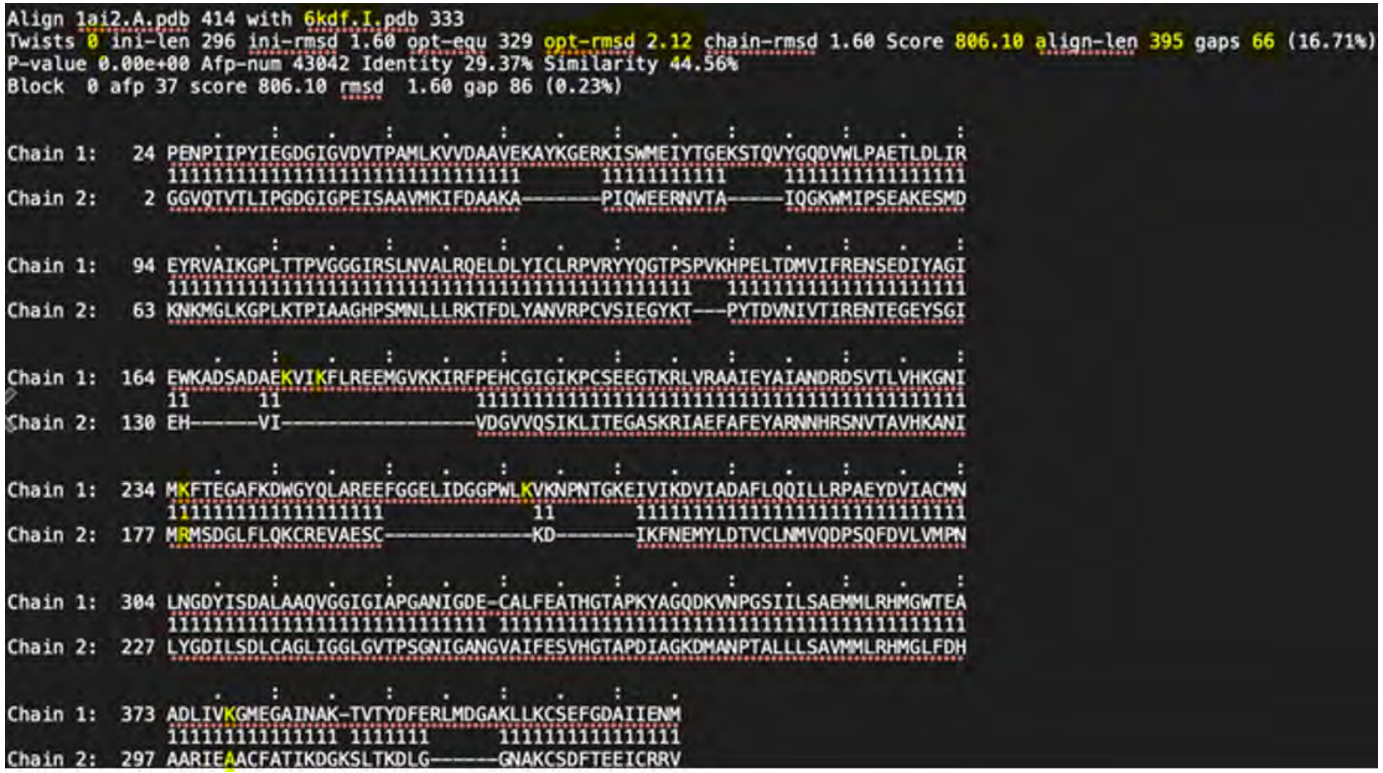

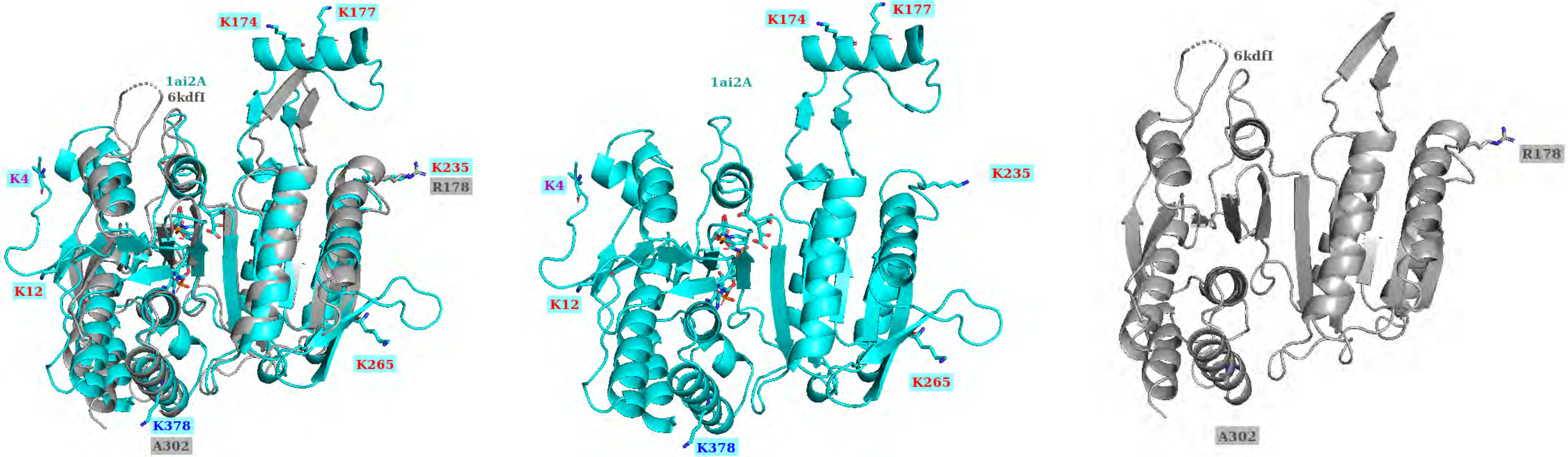

UniProt ID: P50213
PDB ID: 6KDY_E

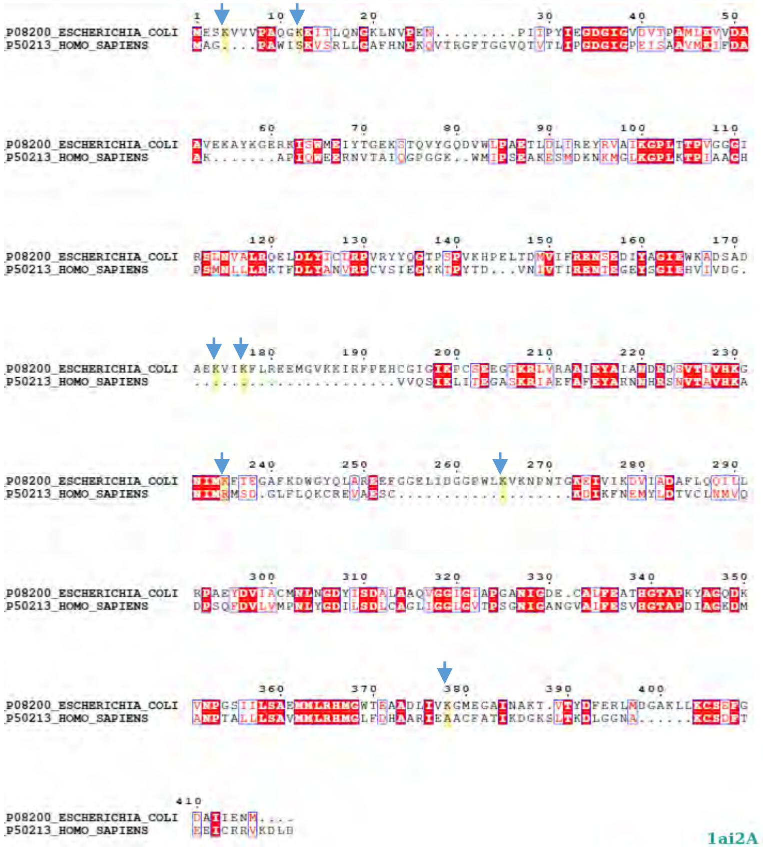

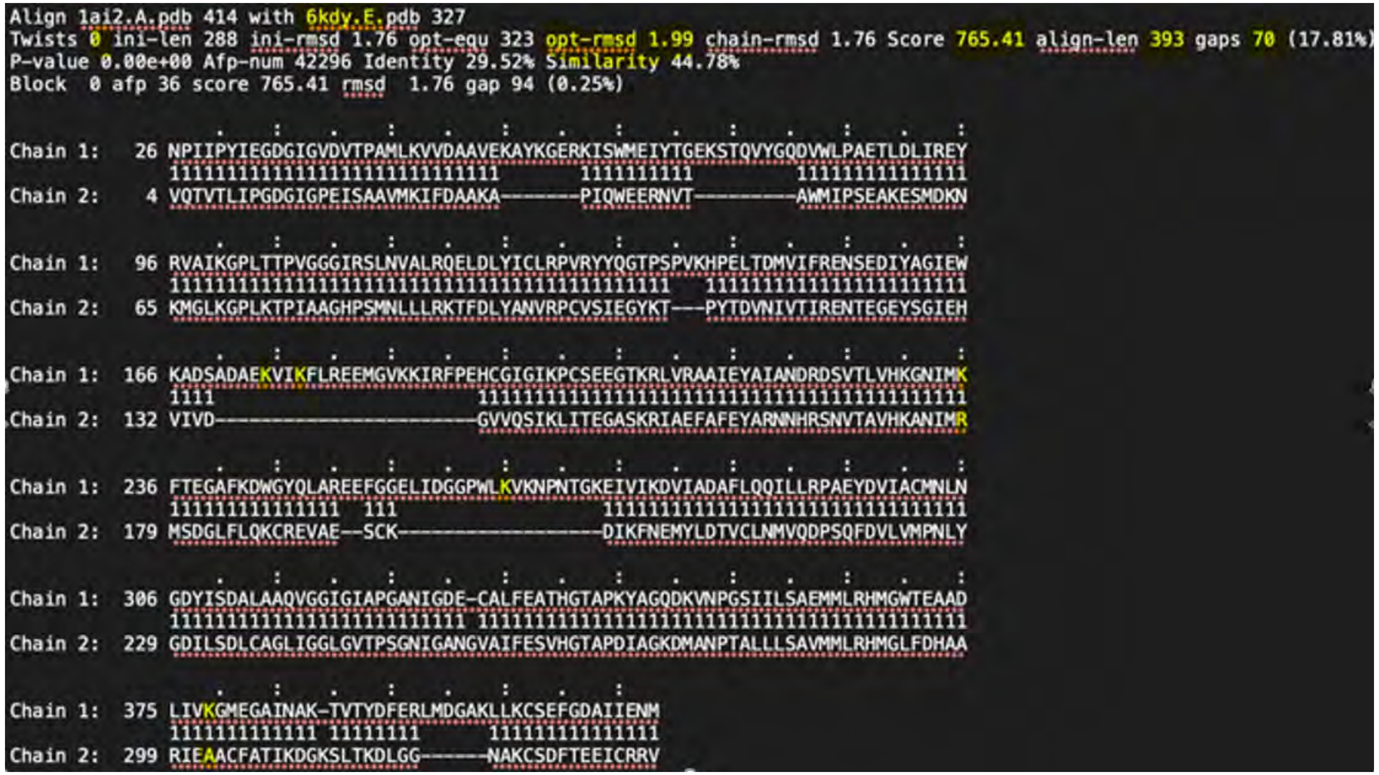

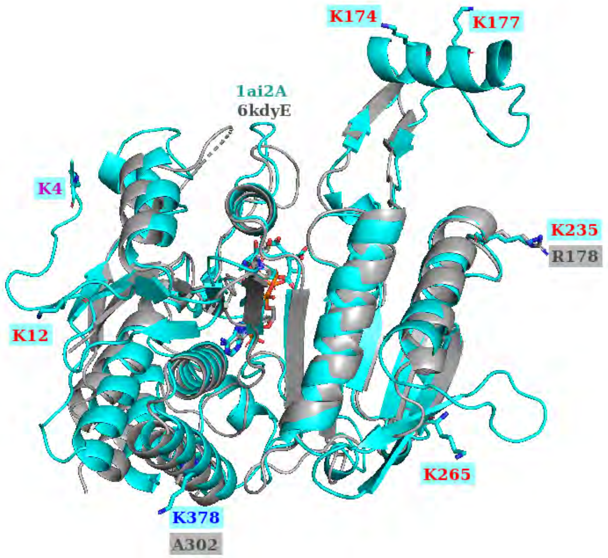

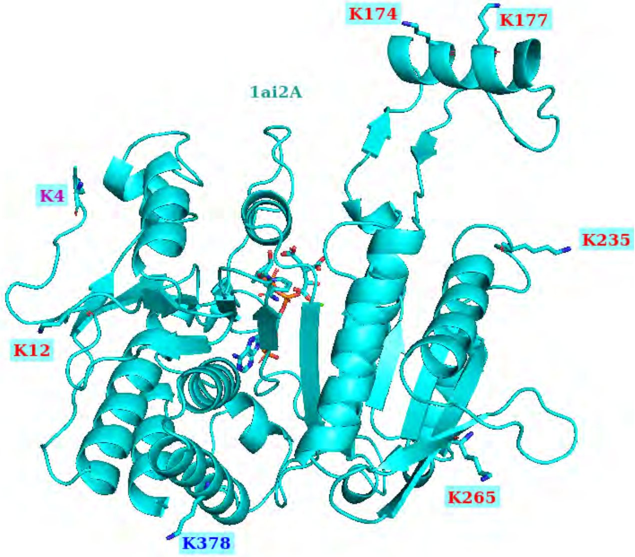

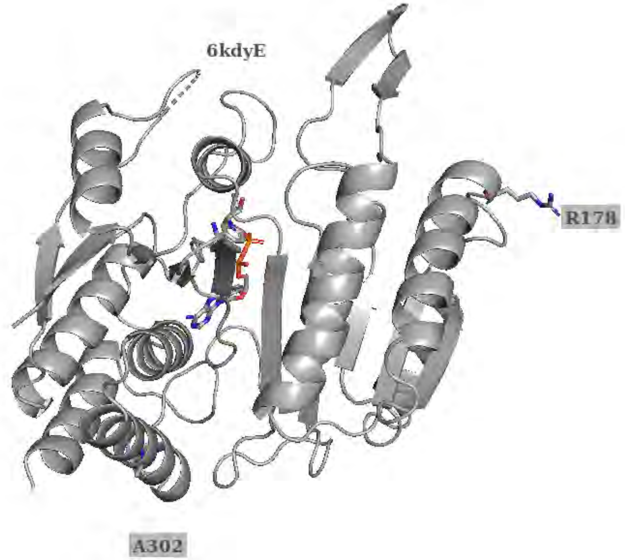

UniProt ID: P50213

PDB ID: 6KE3_E

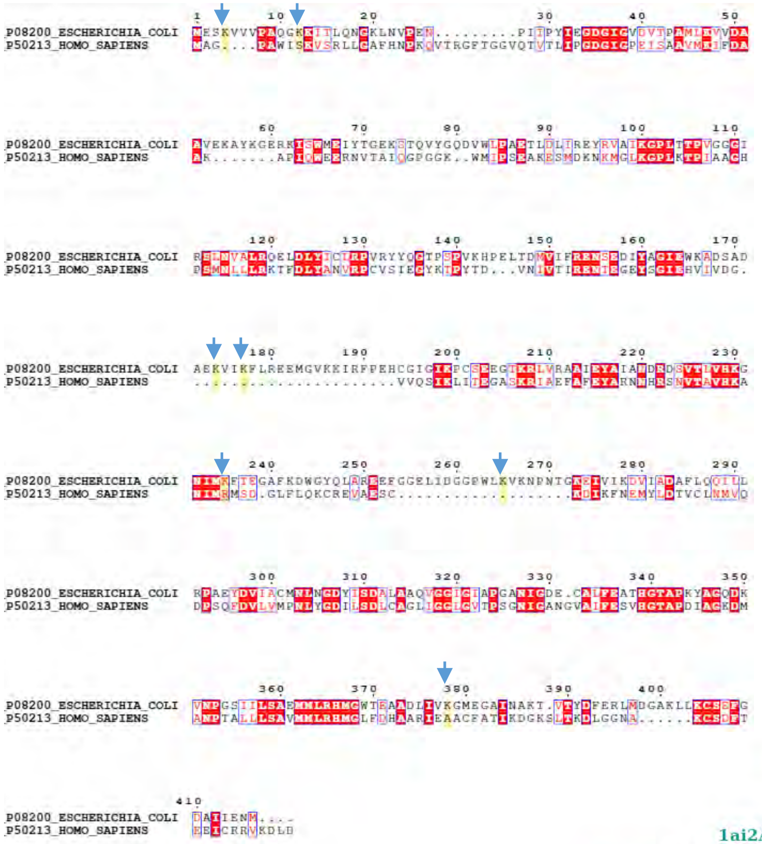

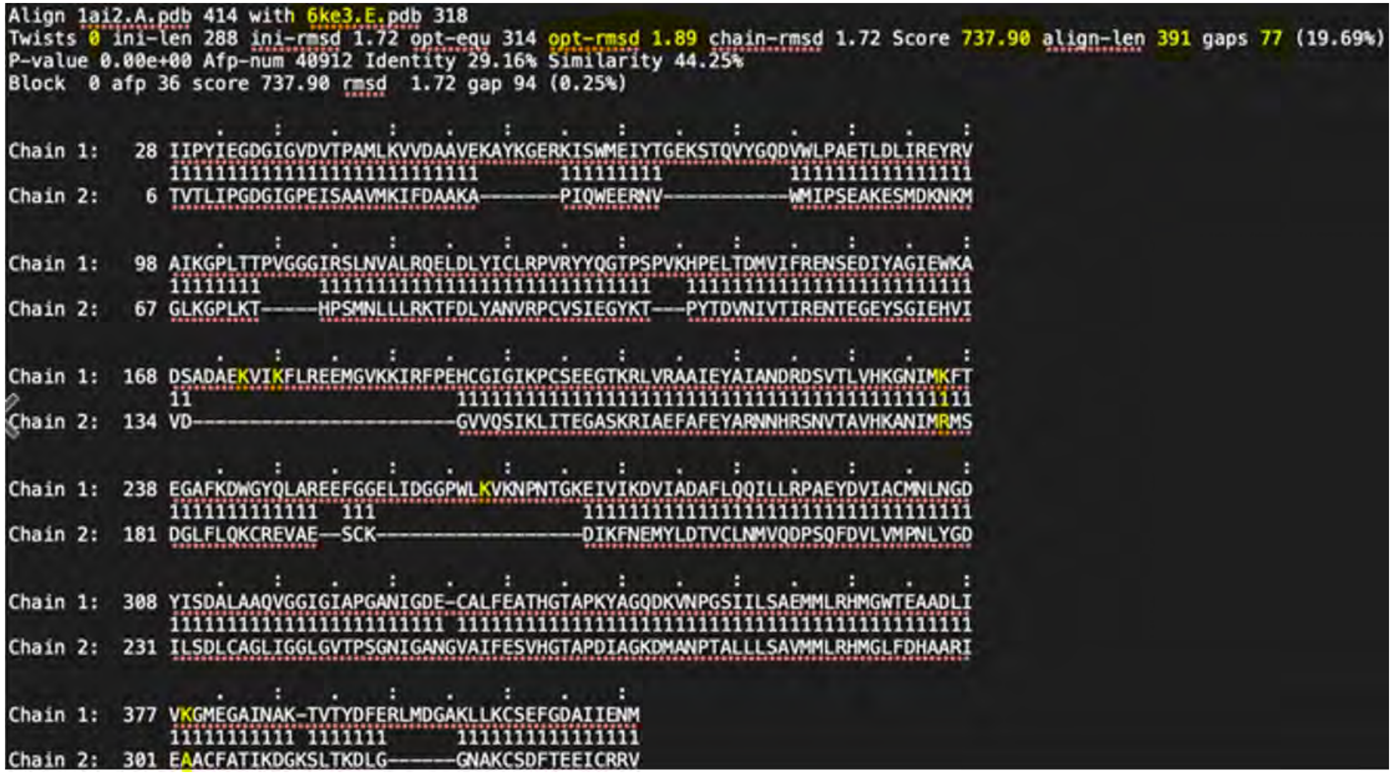

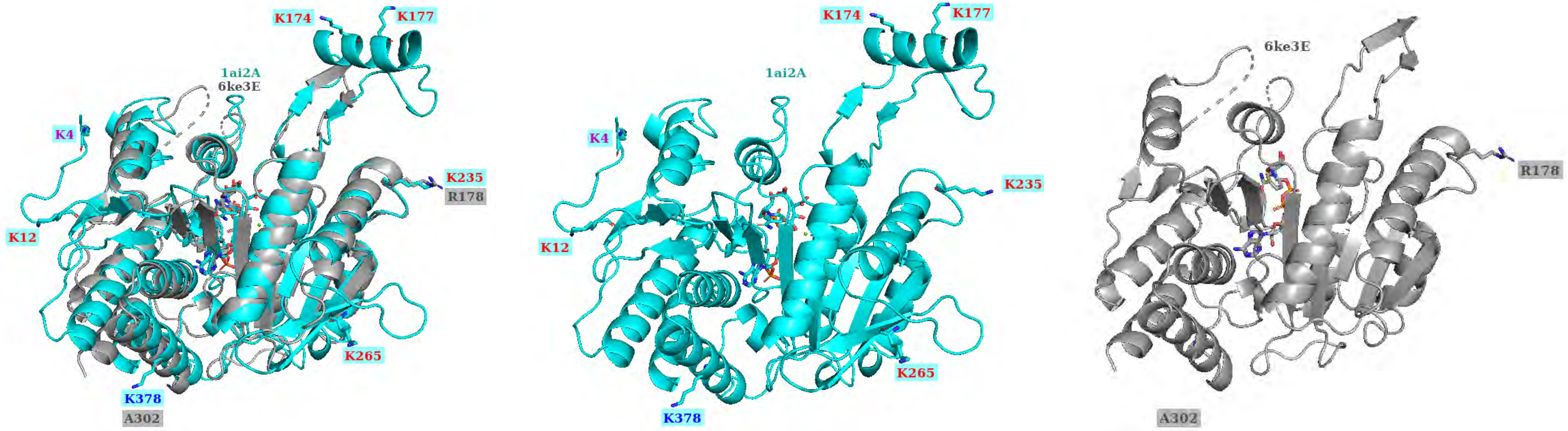

UniProt ID: P50213

PDB ID: 6L57_A

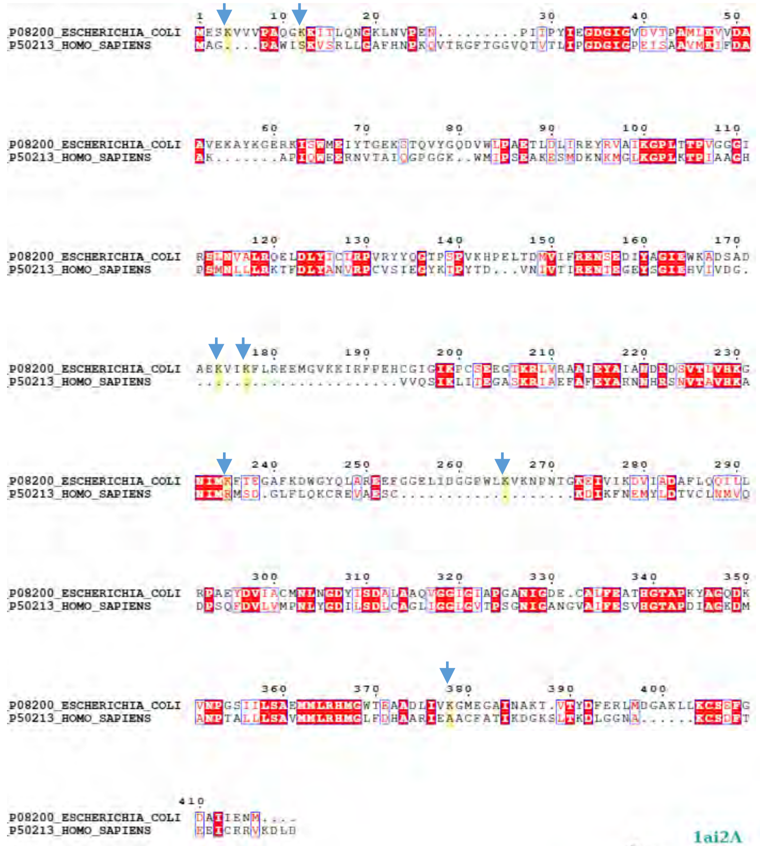

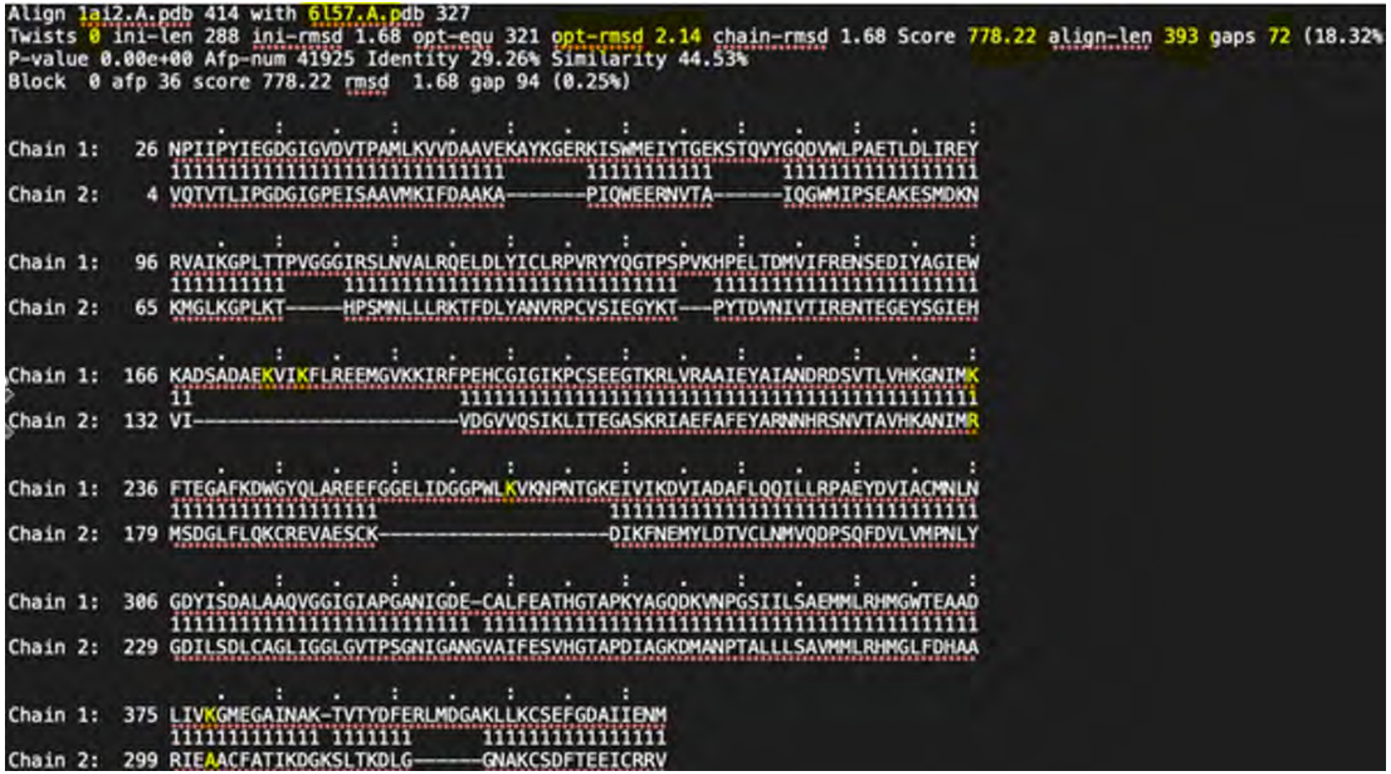

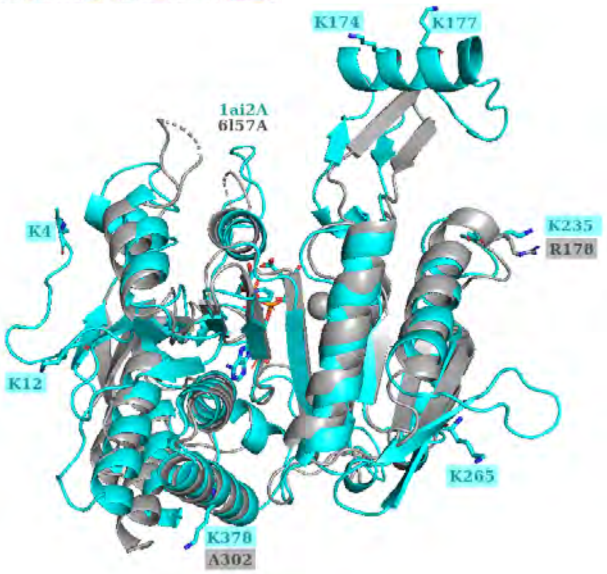

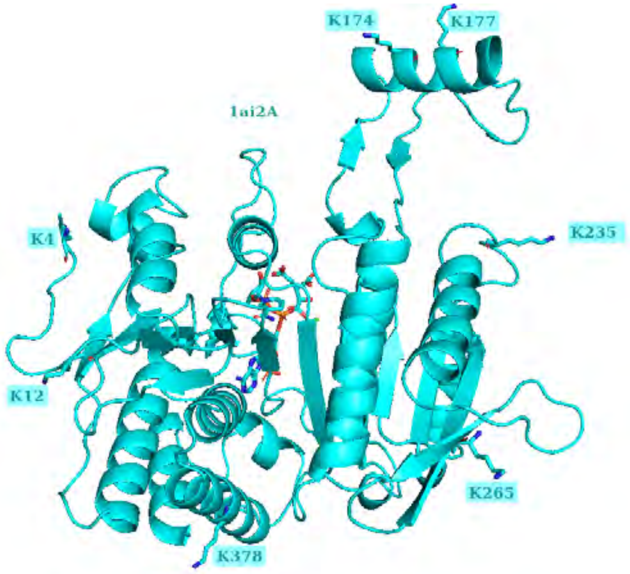

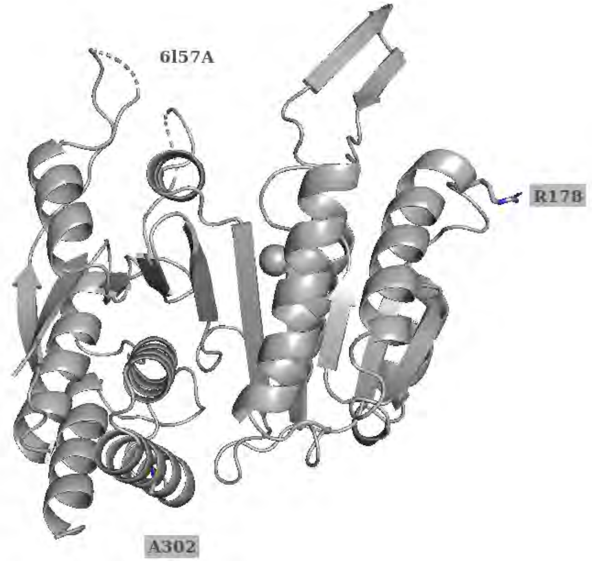

UniProt ID: P50213

PDB ID: 6L59_A

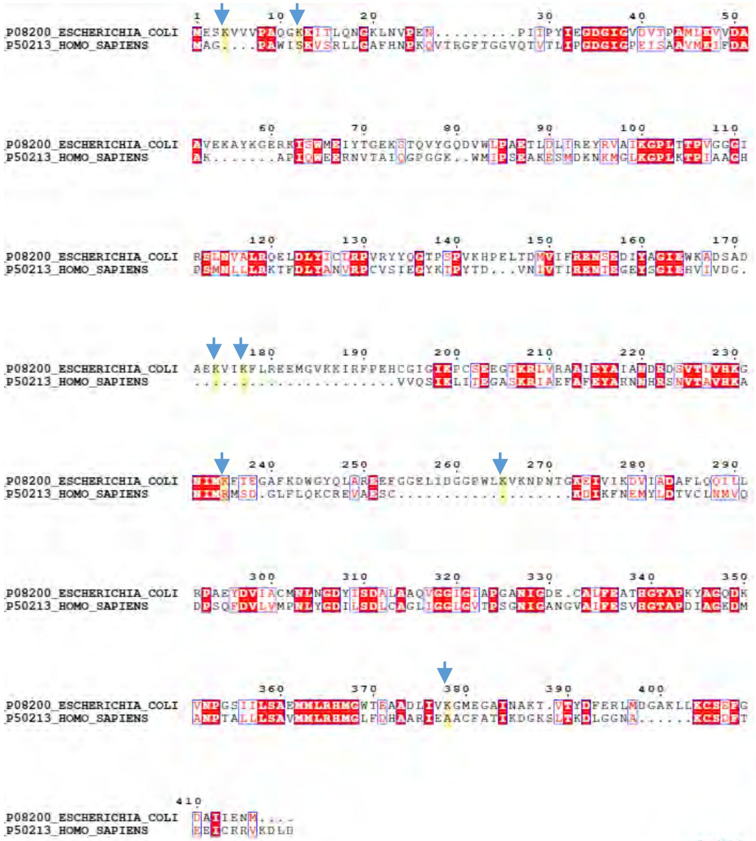


```
Align 1ai2.A.pdb 414 with 6l59.A.pdb 325
Twists 0 ini-len 280 ini-rmsd 1.77 opt-equ 319 opt-rmsd 2.02 chain-rmsd 1.77 Score 784.22 align-len 393 gaps 74 (18.83%)
P-value 0.00e+00 Afp-num 41528 Identity 29.26% Similarity 44.27%
Block  0 afp 35 score 784.22 rmsd  1.77 gap 102 (0.27%)

                  .    :    .    :    .    :    .    :    .    :    .    :    .    :
Chain 1:   26 NPIIPYIEGDGIGVDVTPAMLKVVDAAVEKAYKGERKISWMEIYTGEKSTQVYGQDVWLPAETLDLIREY
              11111111111111111111111111111       11111111111       1111111111111111
Chain 2:    4 VQTVTLIPGDGIGPEISAAVMKIFDAAKA-------PIQWEERNVTA-------IQWMIPSEAKESMDKN

                  .    :    .    :    .    :    .    :    .    :    .    :    .    :
Chain 1:   96 RVAIKGPLTTPVGGGIRSLNVALRQELDLYICLRPVRYYQGTPSPVKHPELTDMVIFRENSEDIYAGIEW
              1111111111      11111111111111111111111111111   1111111111111111111111
Chain 2:   65 KMGLKGPLKT------PSMNLLLRKTFDLYANVRPCVSIEGYKTP---YTDVNIVTIRENTEGEYSGIEH

                  .    :    .    :    .    :    .    :    .    :    .    :    .    :
Chain 1:  166 KADSADAEKVIKFLREEMGVKKIRFPEHCGIGIKPCSEEGTKRLVRAAIEYAIANDRDSVTLVHKGNIMK
                    11                 111111111111111111111111111111111111111111111
Chain 2:  132 ------VI-----------------VDGVVQSIKLITEGASKRIAEFAFEYARNNHRSNVTAVHKANIMR

                  .    :    .    :    .    :    .    :    .    :    .    :    .    :
Chain 1:  236 FTEGAFKDWGYQLAREEFGGELIDGGPWLKVKNPNTGKEIVIKDVIADAFLQQILLRPAEYDVIACMNLN
              111111111111111 11            11       1111111111111111111111111111111
Chain 2:  179 MSDGLFLQKCREVAE-SC------------KD-------IKFNEMYLDTVCLNMVQDPSQFDVLVMPNLY

                  .    :    .    :    .    :    .    :    .    :    .    :    .    :
Chain 1:  306 GDYISDALAAQVGGIGIAPGANIGD-ECALFEATHGTAPKYAGQDKVNPGSIILSAEMMLRHMGWTEAAD
              1111111111111111111111111 11111111111111111111111111111111111111111111
Chain 2:  229 GDILSDLCAGLIGGLGVTPSGNIGANGVAIFESVHGTAPDIAGKDMANPTALLLSAVMMLRHMGLFDHAA

                  .    :    .    :    .    :    .    :
Chain 1:  375 LIVKGMEGAINAK-TVTYDFERLMDGAKLLKCSEFGDAIIENM
              1111111111111 1111111      1111111111111111
Chain 2:  299 RIEAACFATIKDGKSLTKDLG------GNAKCSDFTEEICRRV
```

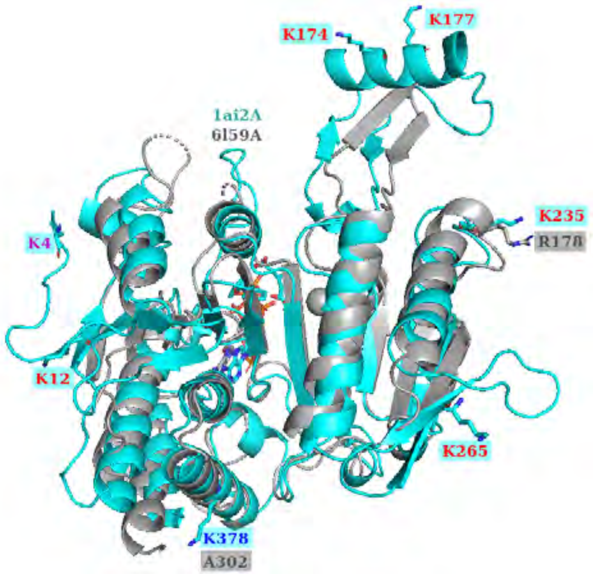

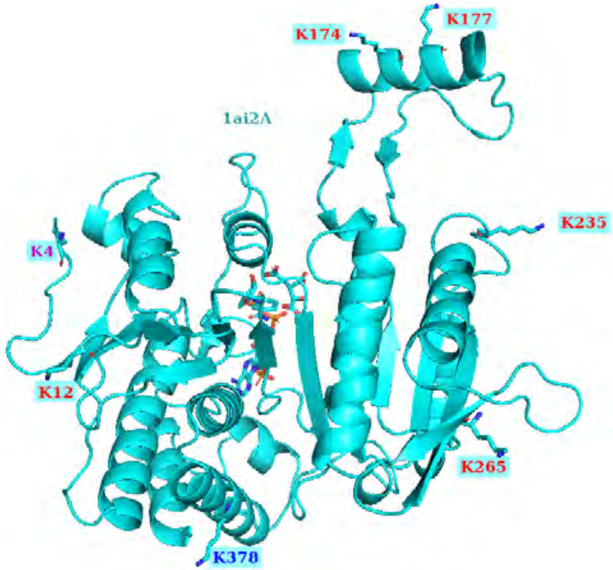

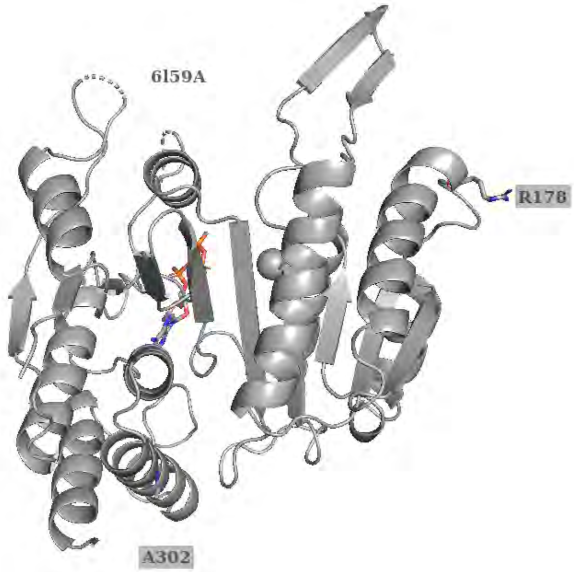

UniProt ID: Q02NB5
PDB ID: 5M2E_D

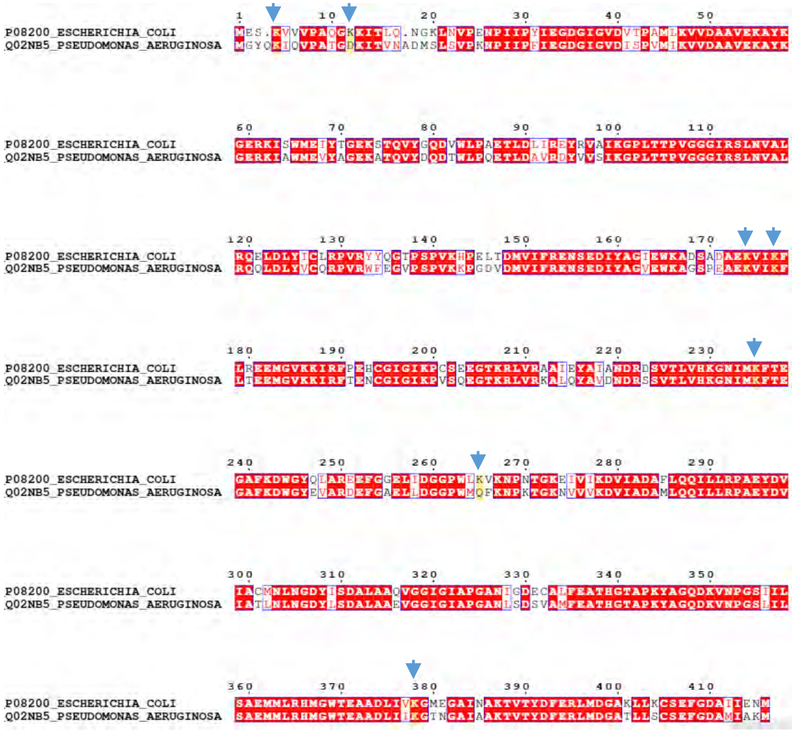


```
Align 1ai2.A.pdb 414 with 5m2e.D.pdb 418
Twists 0 ini-len 408 ini-rmsd 2.05 opt-equ 414 opt-rmsd 2.08 chain-rmsd 2.05 Score 1205.29 align-len 415 gaps 1 (0.24%)
P-value 0.00e+00 Afp-num 52101 Identity 78.80% Similarity 88.92%
Block  0 afp 51 score 1205.29 rmsd  2.05 gap 6 (0.01%)

                  .    :    .    :    .    :    .    :    .    :    .    :    .    :
Chain 1:    3 SKVVVPAQGKKITLQN-GKLNVPENPIIPYIEGDGIGVDVTPAMLKVVDAAVEKAYKGERKISWMEIYTG
              1111111111111111 11111111111111111111111111111111111111111111111111111
Chain 2:    4 QKIQVPATGDKITVNADMSLSVPKNPIIPFIEGDGIGVDISPVMIKVVDAAVEKAYKGERKIAWMEVYAG

                  .    :    .    :    .    :    .    :    .    :    .    :    .    :
Chain 1:   72 EKSTQVYGQDVWLPAETLDLIREYRVAIKGPLTTPVGGGIRSLNVALRQELDLYICLRPVRYYQGTPSPV
              1111111111111111111111111111111111111111111111111111111111111111111111
Chain 2:   74 EKATQVYDQDTWLPQETLDAVRDYVVSIKGPLTTPVGGGIRSLNVALRQQLDLYVCQRPVRWFEGVPSPV

                  .    :    .    :    .    :    .    :    .    :    .    :    .    :
Chain 1:  142 KHPELTDMVIFRENSEDIYAGIEWKADSADAEKVIKFLREEMGVKKIRFPEHCGIGIKPCSEEGTKRLVR
              1111111111111111111111111111111111111111111111111111111111111111111111
Chain 2:  144 KKPGDVDMVIFRENSEDIYAGVEWKAGSPEAEKVIKFLTEEMGVKKIRFTENCGIGIKPVSQEGTKRLVR

                  .    :    .    :    .    :    .    :    .    :    .    :    .    :
Chain 1:  212 AAIEYAIANDRDSVTLVHKGNIMKFTEGAFKDWGYQLAREEFGGELIDGGPWLKVKNPNTGKEIVIKDVI
              1111111111111111111111111111111111111111111111111111111111111111111111
Chain 2:  214 KALQYAVDNDRSSVTLVHKGNIMKFTEGAFKDWGYEVARDEFGAELLDGGPWMQFKNPKTGKNVVVKDVI

                  .    :    .    :    .    :    .    :    .    :    .    :    .    :
Chain 1:  282 ADAFLQQILLRPAEYDVIACMNLNGDYISDALAAQVGGIGIAPGANIGDECALFEATHGTAPKYAGQDKV
              1111111111111111111111111111111111111111111111111111111111111111111111
Chain 2:  284 ADAMLQQILLRPAEYDVIATLNLNGDYLSDALAAEVGGIGIAPGANLSDSVAMFEATHGTAPKYAGQDKV

                  .    :    .    :    .    :    .    :    .    :    .    :    .
Chain 1:  352 NPGSIILSAEMMLRHMGWTEAADLIVKGMEGAINAKTVTYDFERLMDGAKLLKCSEFGDAIIENM
              11111111111111111111111111111111111111111111111111111111111111111
Chain 2:  354 NPGSLILSAEMMLRHMGWTEAADLIIKGTNGAIAAKTVTYDFERLMDGATLLSCSEFGDAMIAKM
```

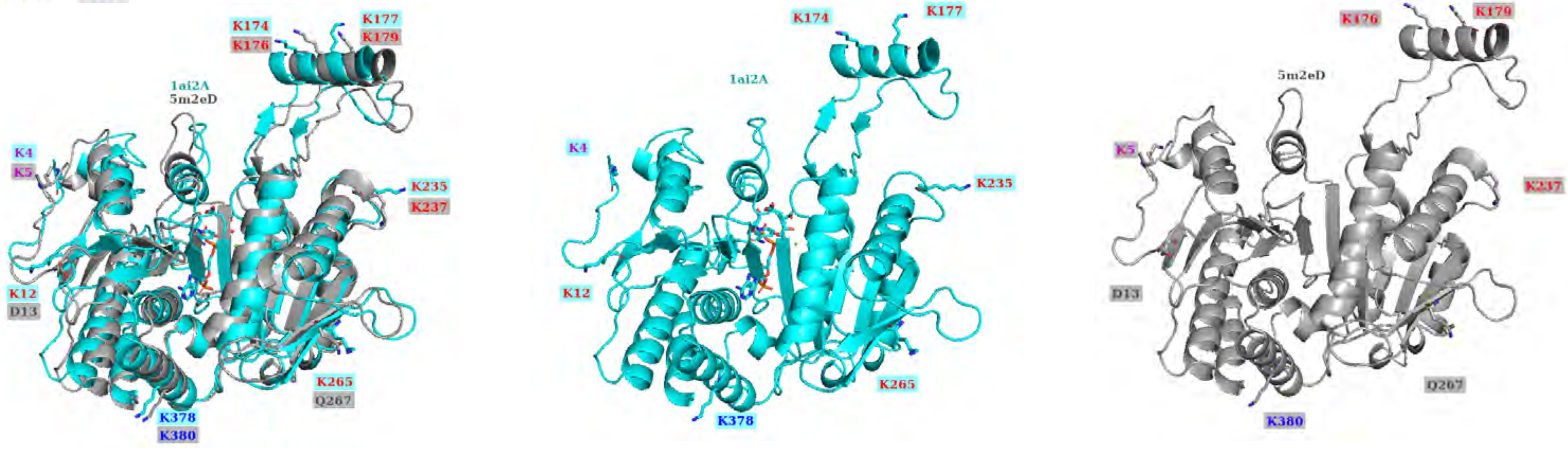

UniProt ID: Q3JV82
PDB ID: 3DMS_A

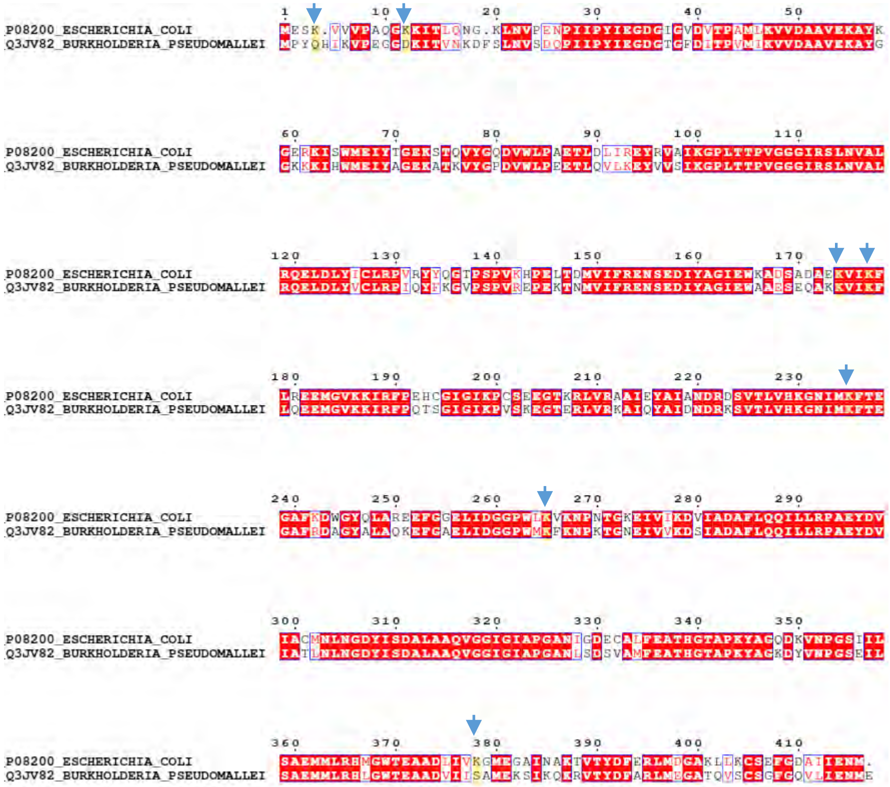


```
Align 1ai2.A.pdb 414 with 3dms.A.pdb 413
Twists 0 ini-len 408 ini-rmsd 0.74 opt-equ 410 opt-rmsd 0.75 chain-rmsd 0.74 Score 1215.35 align-len 415 gaps 5 (1.20%)
P-value 0.00e+00 Afp-num 50641 Identity 73.98% Similarity 84.34%
Block  0 afp 51 score 1215.35 rmsd  0.74 gap 6 (0.01%)

Chain 1:    3 SKVVVPAQGKKITLQN-GKLNVPENPIIPYIEGDGIGVDVTPAMLKVVDAAVEKAYKGERKISWMEIYTG
                1111111111111111 11111111111111111111111111111111111111111111111111111
Chain 2:    4 QHIKVPEGGDKITVNKDFSLNVSDQPIIPYIEGDGTGFDITPVMIKVVDAAVEKAYGGKKKIHWMEIYAG

Chain 1:   72 EKSTQVYGQDVWLPAETLDLIREYRVAIKGPLTTPVGGGIRSLNVALRQELDLYICLRPVRYYQGTPSPV
                111111111111111111111111111111111111    111111111111111111111111111111
Chain 2:   74 EKATKVYGPDVWLPEETLQVLKEYVVSIKGPLTTPV----RSLNVALRQELDLYVCLRPIQYFKGVPSPV

Chain 1:  142 KHPELTDMVIFRENSEDIYAGIEWKADSADAEKVIKFLREEMGVKKIRFPEHCGIGIKPCSEEGTKRLVR
                1111111111111111111111111111111111111111111111111111111111111111111111
Chain 2:  144 REPEKTNMVIFRENSEDIYAGIEWAAESEQAKKVIKFLQEEMGVKKIRFPQTSGIGIKPVSKEGTERLVR

Chain 1:  212 AAIEYAIANDRDSVTLVHKGNIMKFTEGAFKDWGYQLAREEFGGELIDGGPWLKVKNPNTGKEIVIKDVI
                1111111111111111111111111111111111111111111111111111111111111111111111
Chain 2:  214 KAIQYAIDNDRKSVTLVHKGNIMKFTEGAFRDAGYALAQKEFGAELIDGGPWMKFKNPKTGNEIVVKDSI

Chain 1:  282 ADAFLQQILLRPAEYDVIACMNLNGDYISDALAAQVGGIGIAPGANIGDECALFEATHGTAPKYAGQDKV
                1111111111111111111111111111111111111111111111111111111111111111111111
Chain 2:  284 ADAFLQQILLRPAEYDVIATLNLNGDYISDALAAQVGGIGIAPGANLSDSVAMFEATHGTAPKYAGKDYV

Chain 1:  352 NPGSIILSAEMMLRHMGWTEAADLIVKGMEGAINAKTVTYDFERLMDGAKLLKCSEFGDAIIENM
                11111111111111111111111111111111111111111111111111111111111111111
Chain 2:  354 NPGSEILSAEMMLRHLGWTEAADVIISAMEKSIKQKRVTYDFARLMEGATQVSCSGFGQVLIENM

Note: positions are from PDB; the numbers between alignments are block index
```

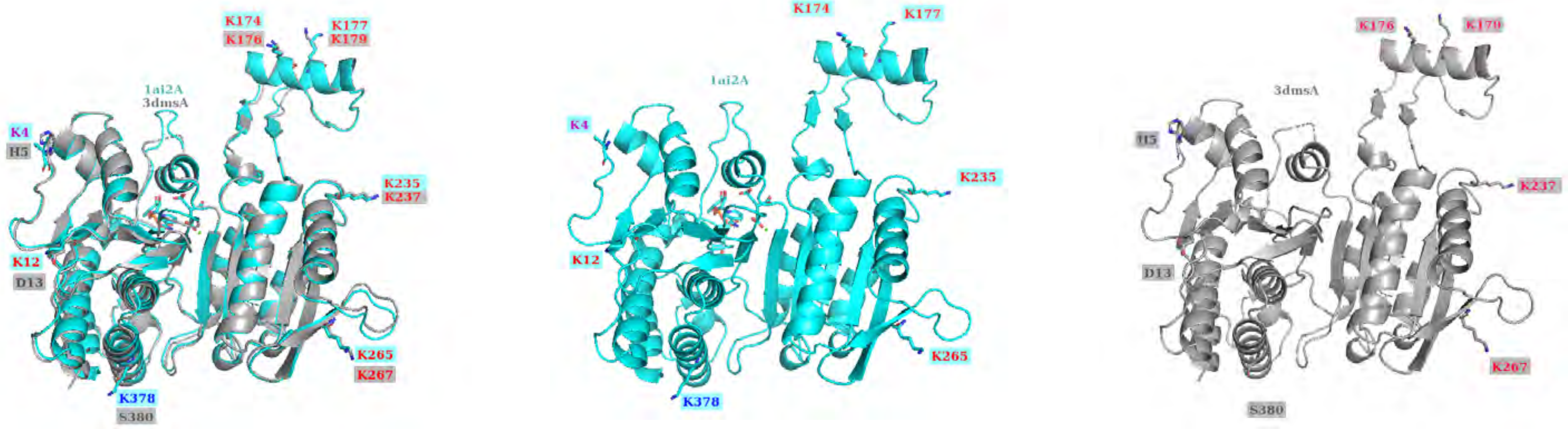

UniProt ID: Q5JFV8
PDB ID: 5HN3_A

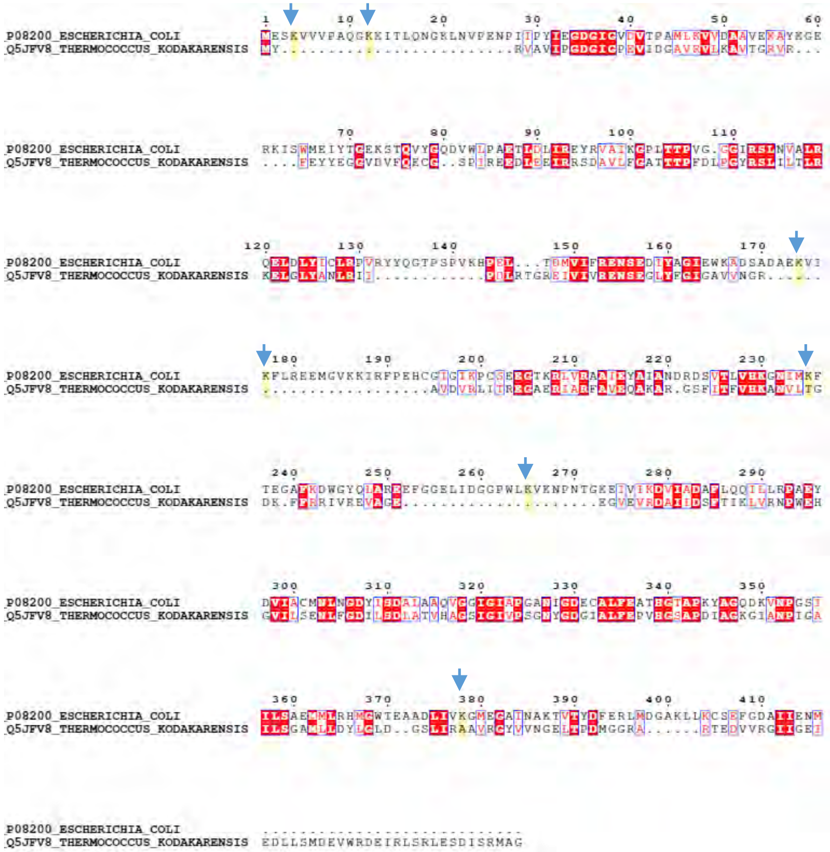

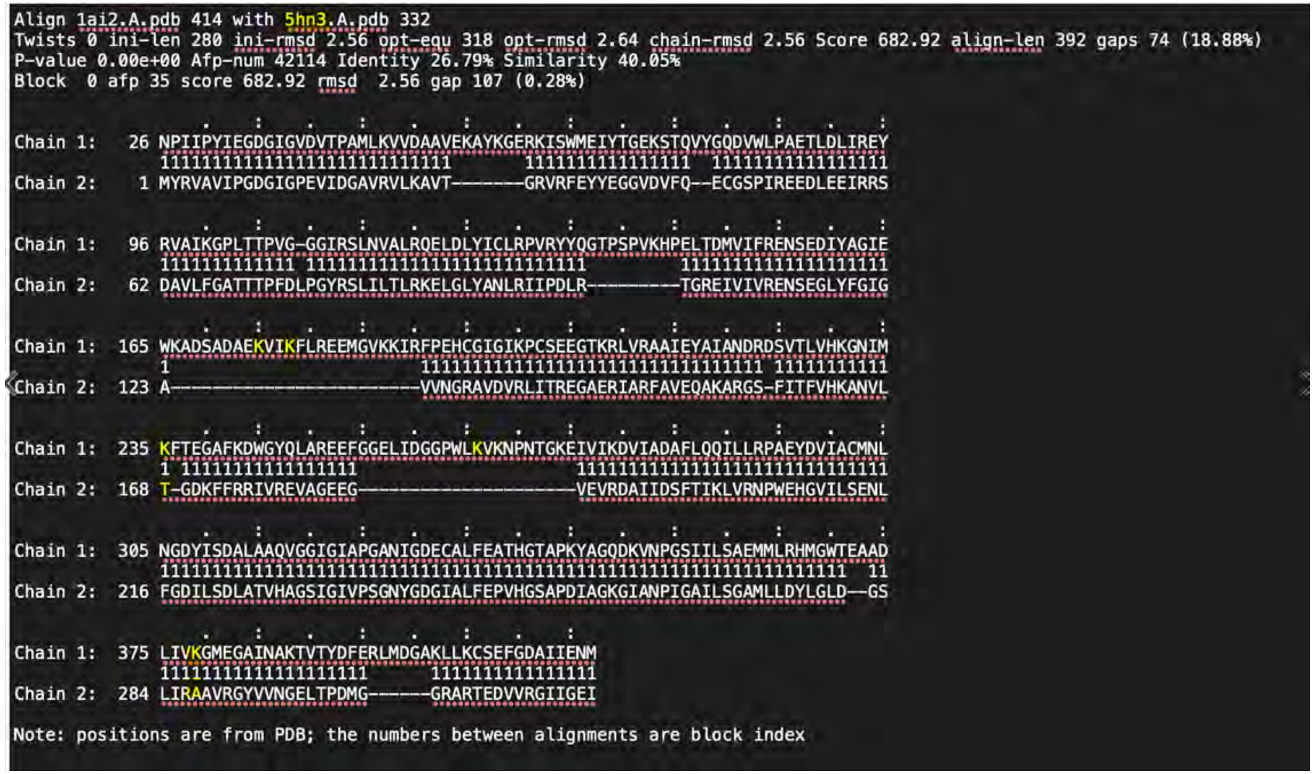

```
Align 1ai2.A.pdb 414 with 5hn3.A.pdb 332
Twists 0 ini-len 280 ini-rmsd 2.56 opt-equ 318 opt-rmsd 2.64 chain-rmsd 2.56 Score 682.92 align-len 392 gaps 74 (18.88%)
P-value 0.00e+00 Afp-num 42114 Identity 26.79% Similarity 40.05%
Block  0 afp 35 score 682.92 rmsd  2.56 gap 107 (0.28%)

                  .    :    .    :    .    :    .    :    .    :    .    :    .    :
Chain 1:   26 NPIIPYIEGDGIGVDVTPAMLKVVDAAVEKAYKGERKISWMEIYTGEKSTQVYGQDVWLPAETLDLIREY
              1111111111111111111111111111       1111111111111111  11111111111111111
Chain 2:    1 MYRVAVIPGDGIGPEVIDGAVRVLKAVT-------GRVRFEYYEGGVDVFQ--ECGSPIREEDLEEIRRS

                  .    :    .    :    .    :    .    :    .    :    .    :    .    :
Chain 1:   96 RVAIKGPLTTPVG-GGIRSLNVALRQELDLYICLRPVRYYQGTPSPVKHPELTDMVIFRENSEDIYAGIE
              1111111111111 111111111111111111111111111         11111111111111111111
Chain 2:   62 DAVLFGATTTPFDLPGYRSLILTLRKELGLYANLRIIPDLR---------TGREIVIVRENSEGLYFGIG

                  .    :    .    :    .    :    .    :    .    :    .    :    .    :
Chain 1:  165 WKADSADAEKVIKFLREEMGVKKIRFPEHCGIGIKPCSEEGTKRLVRAAIEYAIANDRDSVTLVHKGNIM
              1                        111111111111111111111111111111111 11111111111
Chain 2:  123 A------------------------VVNGRAVDVRLITREGAERIARFAVEQAKARGS-FITFVHKANVL

                  .    :    .    :    .    :    .    :    .    :    .    :    .    :
Chain 1:  235 KFTEGAFKDWGYQLAREEFGGELIDGGPWLKVKNPNTGKEIVIKDVIADAFLQQILLRPAEYDVIACMNL
              1 11111111111111111                     111111111111111111111111111111
Chain 2:  168 T-GDKFFRRIVREVAGEEG---------------------VEVRDAIIDSFTIKLVRNPWEHGVILSENL

                  .    :    .    :    .    :    .    :    .    :    .    :    .    :
Chain 1:  305 NGDYISDALAAQVGGIGIAPGANIGDECALFEATHGTAPKYAGQDKVNPGSIILSAEMMLRHMGWTEAAD
              111111111111111111111111111111111111111111111111111111111111111111  11
Chain 2:  216 FGDILSDLATVHAGSIGIVPSGNYGDGIALFEPVHGSAPDIAGKGIANPIGAILSGAMLLDYLGLD--GS

                  .    :    .    :    .    :    .    :
Chain 1:  375 LIVKGMEGAINAKTVTYDFERLMDGAKLLKCSEFGDAIIENM
              11111111111111111111      1111111111111111
Chain 2:  284 LIRAAVRGYVVNGELTPDMG------GRARTEDVVRGIIGEI

Note: positions are from PDB; the numbers between alignments are block index
```

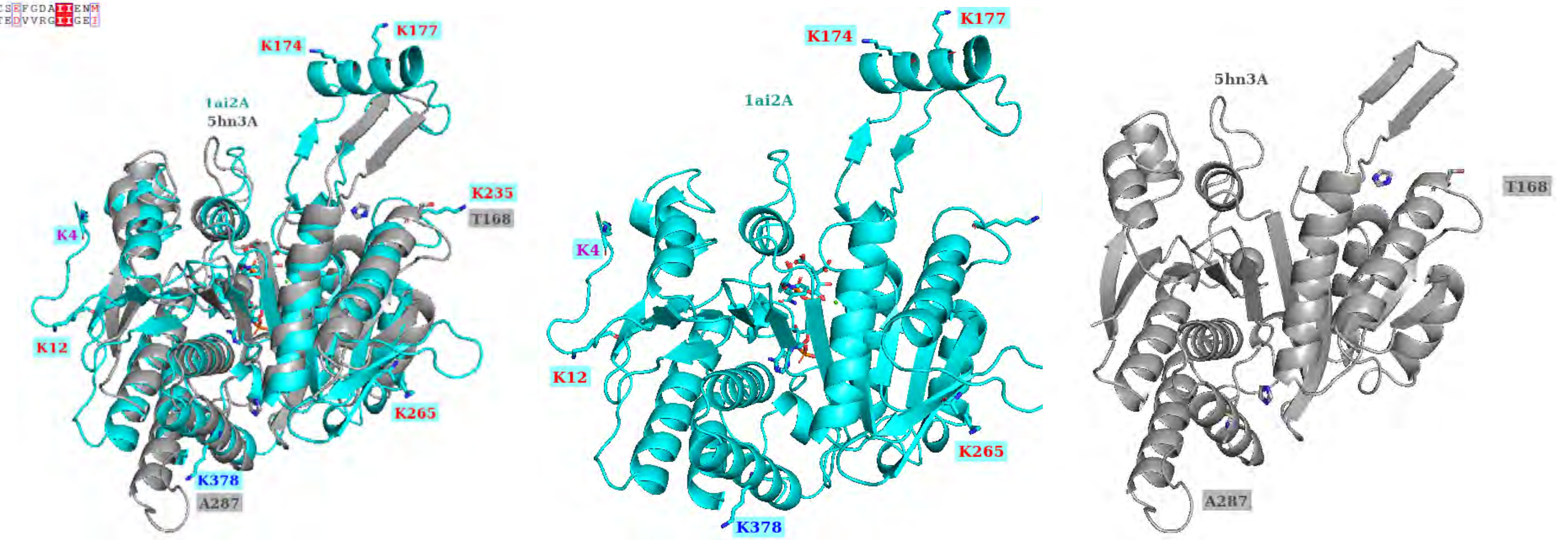

UniProt ID: Q5JFV8
PDB ID: 5HN4_A

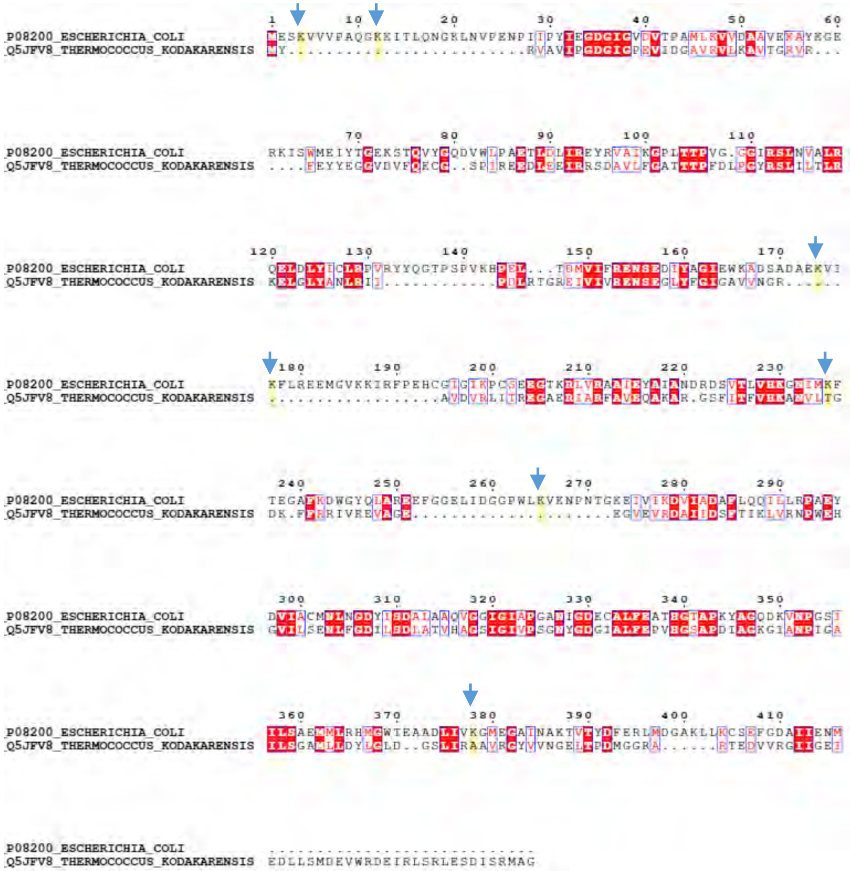

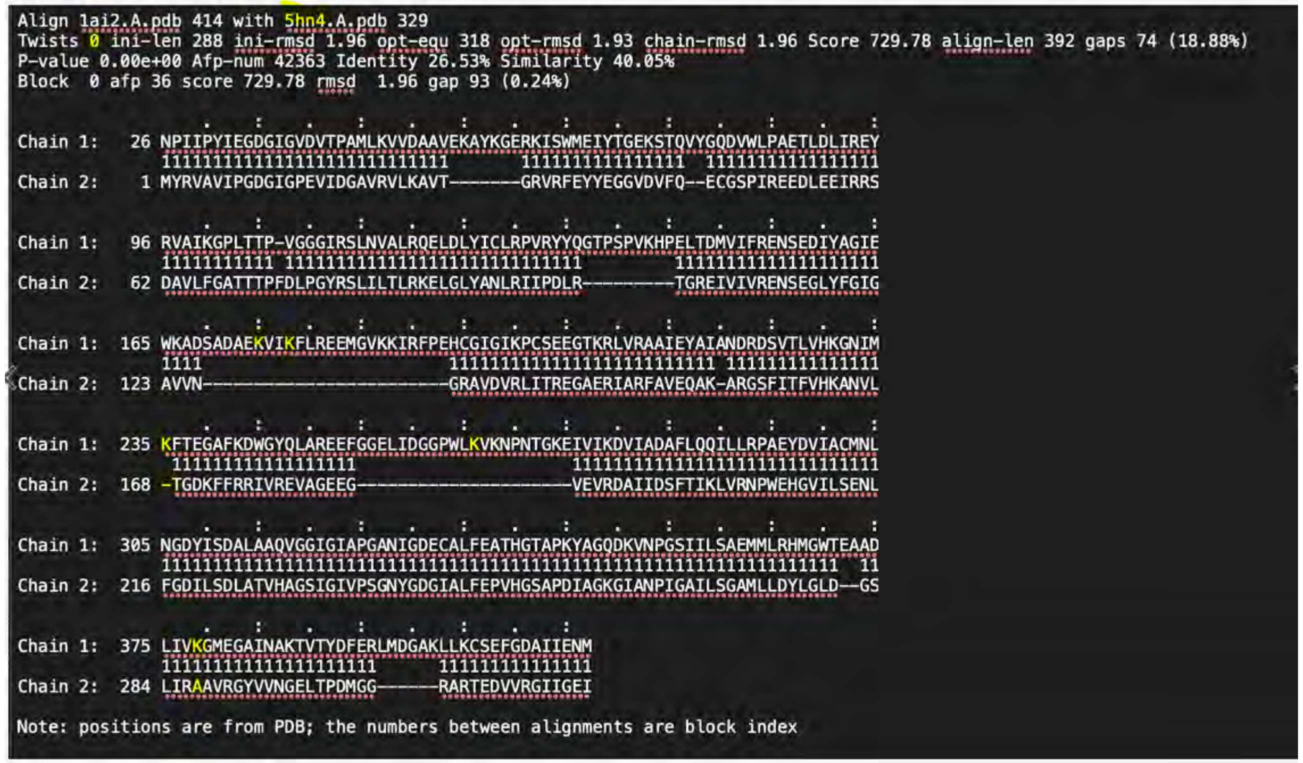

```
Align 1ai2.A.pdb 414 with 5hn4.A.pdb 329
Twists 0 ini-len 288 ini-rmsd 1.96 opt-equ 318 opt-rmsd 1.93 chain-rmsd 1.96 Score 729.78 align-len 392 gaps 74 (18.88%)
P-value 0.00e+00 Afp-num 42363 Identity 26.53% Similarity 40.05%
Block  0 afp 36 score 729.78 rmsd  1.96 gap 93 (0.24%)

Chain 1:   26 NPIIPYIEGDGIGVDVTPAMLKVVDAAVEKAYKGERKISWMEIYTGEKSTQVYGQDVWLPAETLDLIREY
              1111111111111111111111111111       1111111111111111  11111111111111111
Chain 2:    1 MYRVAVIPGDGIGPEVIDGAVRVLKAVT-------GRVRFEYYEGGVDVFQ--ECGSPIREEDLEEIRRS

Chain 1:   96 RVAIKGPLTTP-VGGGIRSLNVALRQELDLYICLRPVRYYQGTPSPVKHPELTDMVIFRENSEDIYAGIE
              11111111111 11111111111111111111111111111        11111111111111111111
Chain 2:   62 DAVLFGATTTPFDLPGYRSLILTLRKELGLYANLRIIPDLR--------TGREIVIVRENSEGLYFGIG

Chain 1:  165 WKADSADAEKVIKFLREEMGVKKIRFPEHCGIGIKPCSEEGTKRLVRAAIEYAIANDRDSVTLVHKGNIM
              1111                        11111111111111111111111111 111111111111111
Chain 2:  123 AVVN------------------------GRAVDVRLITREGAERIARFAVEQAK-ARGSFITFVHKANVL

Chain 1:  235 KFTEGAFKDWGYQLAREEFGGELIDGGPWLKVKNPNTGKEIVIKDVIADAFLQQILLRPAEYDVIACMNL
               111111111111111111                     111111111111111111111111111111
Chain 2:  168 -TGDKFFRRIVREVAGEEG---------------------VEVRDAIIDSFTIKLVRNPWEHGVILSENL

Chain 1:  305 NGDYISDALAAQVGGIGIAPGANIGDECALFEATHGTAPKYAGQDKVNPGSIILSAEMMLRHMGWTEAAD
              111111111111111111111111111111111111111111111111111111111111111111  11
Chain 2:  216 FGDILSDLATVHAGSIGIVPSGNYGDGIALFEPVHGSAPDIAGKGIANPIGAILSGAMLLDYLGLD--GS

Chain 1:  375 LIVKGMEGAINAKTVTYDFERLMDGAKLLKCSEFGDAIIENM
              111111111111111111111      111111111111111
Chain 2:  284 LIRAAVRGYVVNGELTPDMGG------RARTEDVVRGIIGEI

Note: positions are from PDB; the numbers between alignments are block index
```

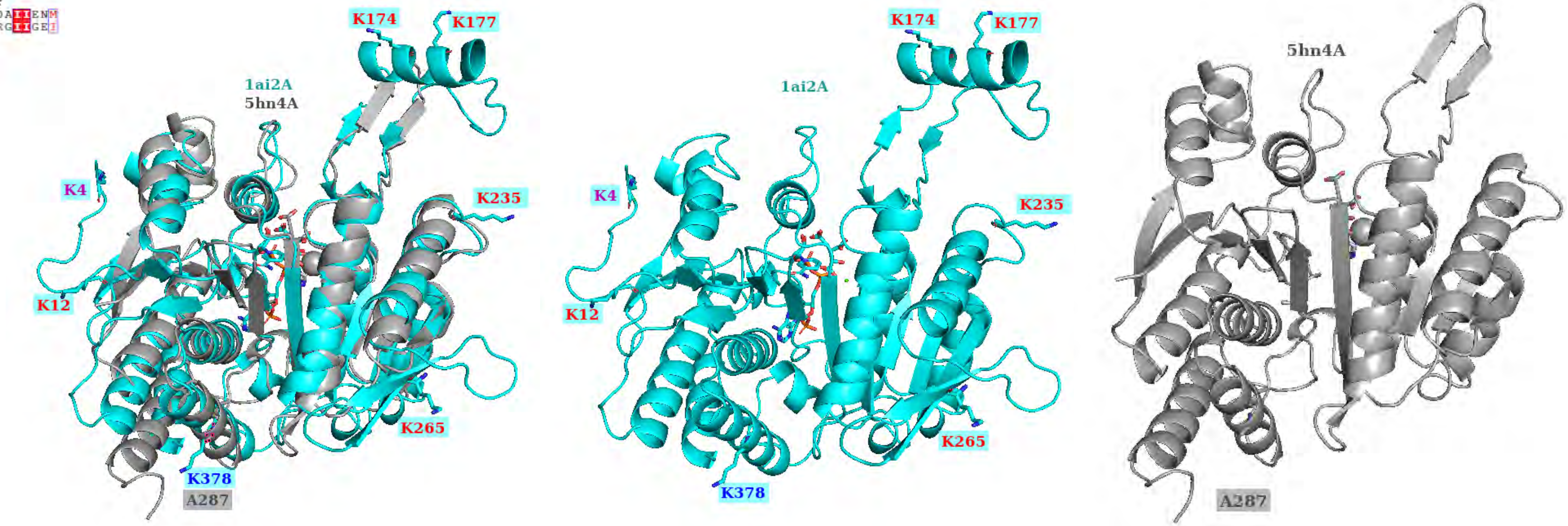

UniProt ID: Q5JFV8

PDB ID: 5HN5_A

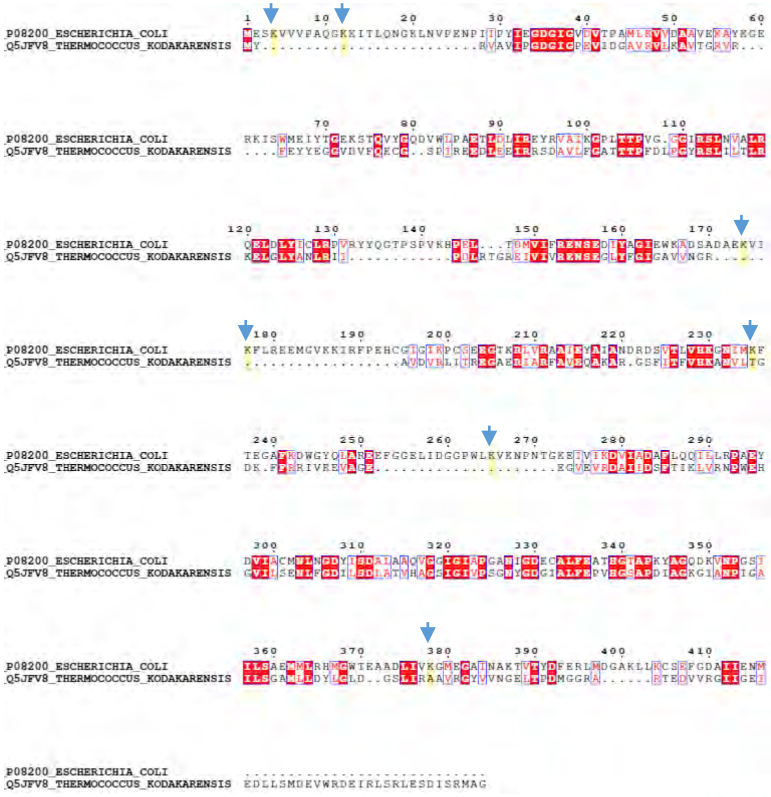


```
Align 1ai2.A.pdb 414 with 5hn5.A.pdb 327
Twists 0 ini-len 288 ini-rmsd 1.98 opt-equ 318 opt-rmsd 1.88 chain-rmsd 1.98 Score 726.39 align-len 392 gaps 74 (18.88%)
P-value 0.00e+00 Afp-num 41828 Identity 26.53% Similarity 40.05%
Block  0 afp 36 score 726.39 rmsd  1.98 gap 99 (0.26%)

Chain 1:   26 NPIIPYIEGDGIGVDVTPAMLKVVDAAVEKAYKGERKISWMEIYTGEKSTQVYGQDVWLPAETLDLIREY
              1111111111111111111111111111       1111111111111111  11111111111111111
Chain 2:    1 MYRVAVIPGDGIGPEVIDGAVRVLKAVT-------GRVRFEYYEGGVDVFQ--ECGSPIREEDLEEIRRS

Chain 1:   96 RVAIKGPLTTP-VGGGIRSLNVALRQELDLYICLRPVRYYQGTPSPVKHPELTDMVIFRENSEDIYAGIE
              11111111111 11111111111111111111111111111         11111111111111111111
Chain 2:   62 DAVLFGATTTPFDLPGYRSLILTLRKELGLYANLRIIPDLR---------TGREIVIVRENSEGLYFGIG

Chain 1:  165 WKADSADAEKVIKFLREEMGVKKIRFPEHCGIGIKPCSEEGTKRLVRAAIEYAIANDRDSVTLVHKGNIM
              1111                        11111111111111111111111111 111111111111111
Chain 2:  123 AVVN------------------------GRAVDVRLITREGAERIARFAVEQAK-ARGSFITFVHKANVL

Chain 1:  235 KFTEGAFKDWGYQLAREEFGGELIDGGPWLKVKNPNTGKEIVIKDVIADAFLQQILLRPAEYDVIACMNL
               111111111111111111                     111111111111111111111111111111
Chain 2:  168 -TGDKFFRRIVREVAGEEG---------------------VEVRDAIIDSFTIKLVRNPWEHGVILSENL

Chain 1:  305 NGDYISDALAAQVGGIGIAPGANIGDECALFEATHGTAPKYAGQDKVNPGSIILSAEMMLRHMGWTEAAD
              111111111111111111111111111111111111111111111111111111111111111111  11
Chain 2:  216 FGDILSDLATVHAGSIGIVPSGNYGDGIALFEPVHGSAPDIAGKGIANPIGAILSGAMLLDYLGLD--GS

Chain 1:  375 LIVKGMEGAINAKTVTYDFERLMDGAKLLKCSEFGDAIIENM
              111111111111111111111      111111111111111
Chain 2:  284 LIRAAVRGYVVNGELTPDMGG------RARTEDVVRGIIGEI

Note: positions are from PDB; the numbers between alignments are block index
```

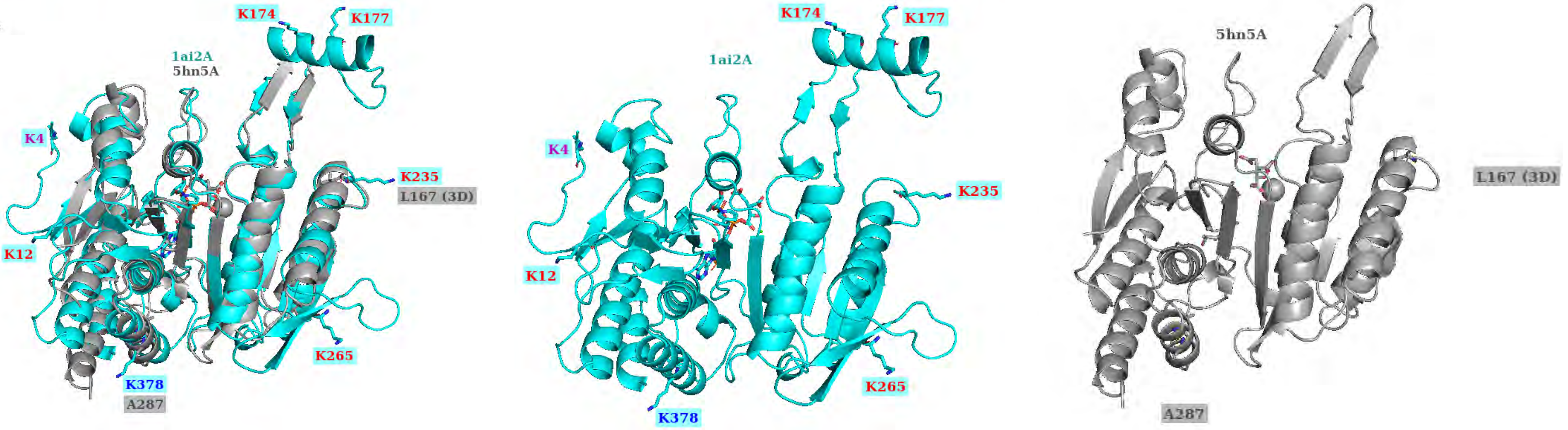

UniProt ID: Q5JFV8
PDB ID: 5HN6_A

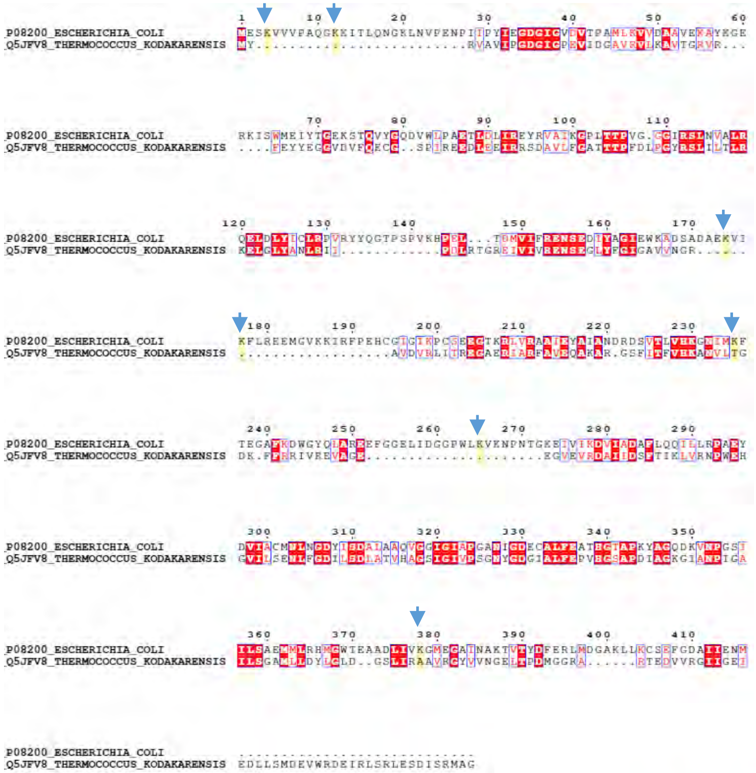


```
Align 1ai2.A.pdb 414 with 5hn6.A.pdb 329
Twists 0 ini-len 288 ini-rmsd 1.95 opt-equ 318 opt-rmsd 1.87 chain-rmsd 1.95 Score 725.27 align-len 392 gaps 74 (18.88%)
P-value 0.00e+00 Afp-num 42083 Identity 26.53% Similarity 40.05%
Block  0 afp 36 score 725.27 rmsd  1.95 gap 99 (0.26%)

Chain 1:   26 NPIIPYIEGDGIGVDVTPAMLKVVDAAVEKAYKGERKISWMEIYTGEKSTQVYGQDVWLPAETLDLIREY
Chain 2:    1 MYRVAVIPGDGIGPEVIDGAVRVLKAVT-------GRVRFEYYEGGVDVFQ--ECGSPIREEDLEEIRRS

Chain 1:   96 RVAIKGPLTTP-VGGGIRSLNVALRQELDLYICLRPVRYYQGTPSPVKHPELTDMVIFRENSEDIYAGIE
Chain 2:   62 DAVLFGATTTPFDLPGYRSLILTLRKELGLYANLRIIPDLR---------TGREIVIVRENSEGLYFGIG

Chain 1:  165 WKADSADAEKVIKFLREEMGVKKIRFPEHCGIGIKPCSEEGTKRLVRAAIEYAIANDRDSVTLVHKGNIM
Chain 2:  123 AVVN---------------------GRAVDVRLITREGAERIARFAVEQAK-ARGSFITFVHKANVL

Chain 1:  235 KFTEGAFKDWGYQLAREEFGGELIDGGPWLKVKNPNTGKEIVIKDVIADAFLQQILLRPAEYDVIACMNL
Chain 2:  168 -TGDKFFRRIVREVAGEEG-----------------VEVRDAIIDSFTIKLVRNPWEHGVILSENL

Chain 1:  305 NGDYISDALAAQVGGIGIAPGANIGDECALFEATHGTAPKYAGQDKVNPGSIILSAEMMLRHMGWTEAAD
Chain 2:  216 FGDILSDLATVHAGSIGIVPSGNYGDGIALFEPVHGSAPDIAGKGIANPIGAILSGAMLLDYLGLD--GS

Chain 1:  375 LIVKGMEGAINAKTVTYDFERLMDGAKLLKCSEFGDAIIENM
Chain 2:  284 LIRAAVRGYVVNGELTPDMGG------RARTEDVVRGIIGEI

Note: positions are from PDB; the numbers between alignments are block index
```

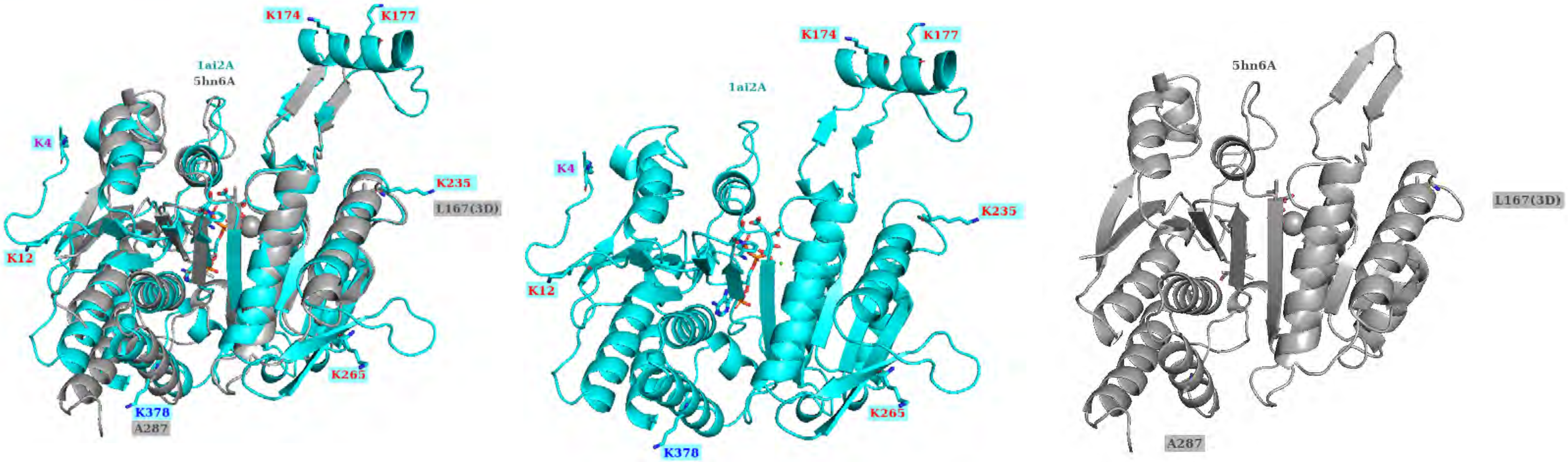

UniProt ID: Q5SIJ1
PDB ID: 3ASJ_C

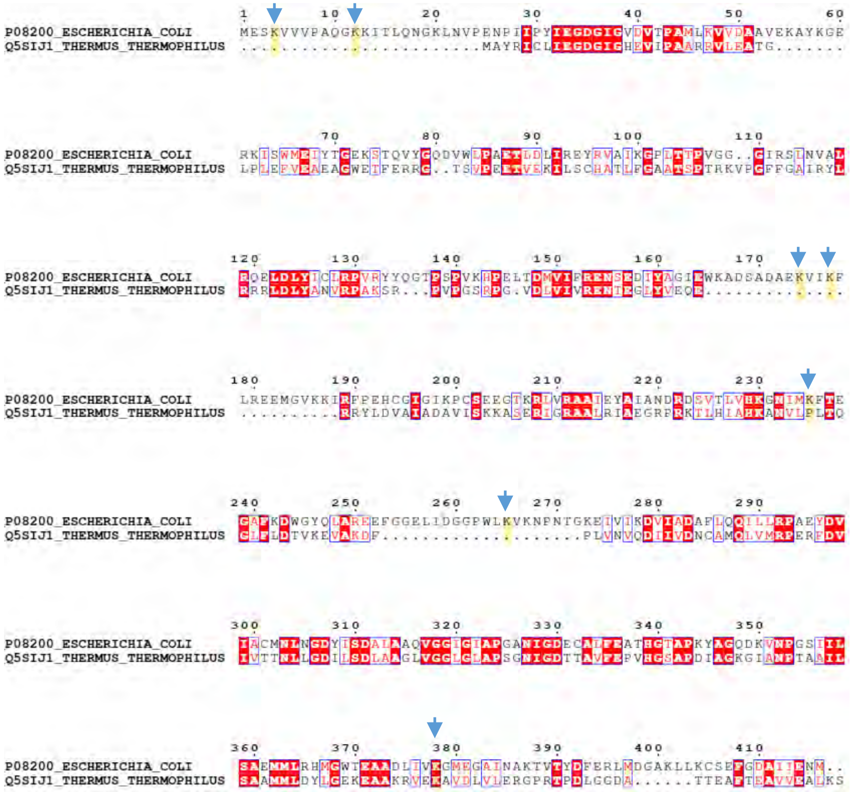


```
Align 1ai2.A.pdb 414 with 3asj.C.pdb 333
Twists 0 ini-len 304 ini-rmsd 1.53 opt-equ 328 opt-rmsd 1.61 chain-rmsd 1.53 Score 808.86 align-len 393 gaps 65 (16.54%)
P-value 0.00e+00 Afp-num 42912 Identity 28.50% Similarity 44.78%
Block  0 afp 38 score 808.86 rmsd  1.53 gap 81 (0.21%)

                  .    :    .    :    .    :    .    :    .    :    .    :    .    :
Chain 1:   26 NPIIPYIEGDGIGVDVTPAMLKVVDAAVEKAYKGERKISWMEIYTGEKSTQVYGQDVWLPAETLDLIREY
              1111111111111111111111111111       1111111111111111  11111111111111111
Chain 2:    2 AYRICLIEGDGIGHEVIPAARRVLEATG-------LPLEFVEAEAGWETFE--RRGTSVPEETVEKILSC

                  .    :    .    :    .    :    .    :    .    :    .    :    .    :
Chain 1:   96 RVAIKGPLTTP--VGGGIRSLNVALRQELDLYICLRPVRYYQGTPSPVKHPELTDMVIFRENSEDIYAGI
              11111111111  11111111111111111111111111111111     11111111111111111111
Chain 2:   63 HATLFGAATSPTRKVPGFFGAIRYLRRLDLYANVRPAKSRPVPGS-----RPGVDLVIVRENTEGLYVEQ

                  .    :    .    :    .    :    .    :    .    :    .    :    .    :
Chain 1:  164 EWKADSADAEKVIKFLREEMGVKKIRFPEHCGIGIKPCSEEGTKRLVRAAIEYAIANDRDSVTLVHKGNI
              11111                        11111111111111111111111111111111111111111
Chain 2:  129 ERRYL------------------------DVAIADAVISKKASERIGRAALRIAEGRPRKTLHIAHKANV

                  .    :    .    :    .    :    .    :    .    :    .    :    .    :
Chain 1:  234 MKFTEGAFKDWGYQLAREEFGGELIDGGPWLKVKNPNTGKEIVIKDVIADAFLQQILLRPAEYDVIACMN
              11111111111111111 111                   111111111111111111111111111111
Chain 2:  175 LPLTQGLFLDTVKEVAK-DFP-------------------LVNVQDIIVDNCAMQLVMRPERFDVIVTTN

                  .    :    .    :    .    :    .    :    .    :    .    :    .    :
Chain 1:  304 LNGDYISDALAAQVGGIGIAPGANIGDECALFEATHGTAPKYAGQDKVNPGSIILSAEMMLRHMGWTEAA
              1111111111111111111111111111111111111111111111111111111111111111111111
Chain 2:  225 LLGDILSDLAAGLVGGLGLAPSGNIGDTTAVFEPVHGSAPDIAGKGIANPTAAILSAAMMLDYLGEKEAA

                  .    :    .    :    .    :    .    :
Chain 1:  374 DLIVKGMEGAINAKTVTYDFERLMDGAKLLKCSEFGDAIIENM
              111111111111111111111      1111111111111111
Chain 2:  295 KRVEKAVDLVLERGPRTPDLG------GDATTEAFTEAVVEAL
```

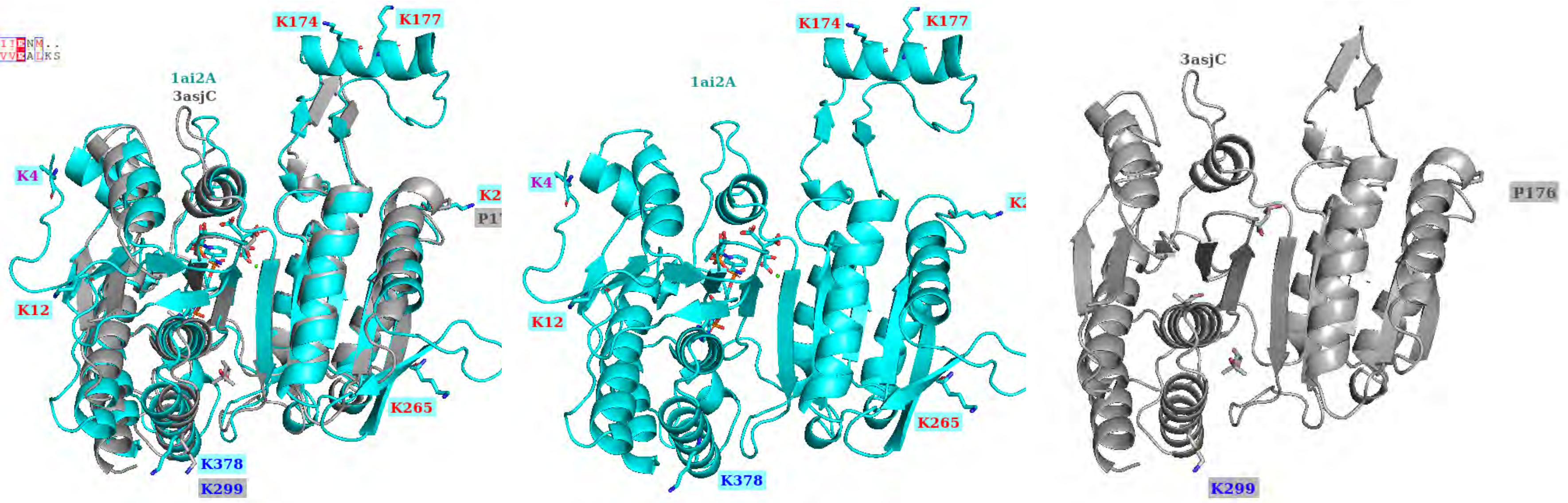

UniProt ID: Q5ZXB6

PDB ID: 6C0E_B

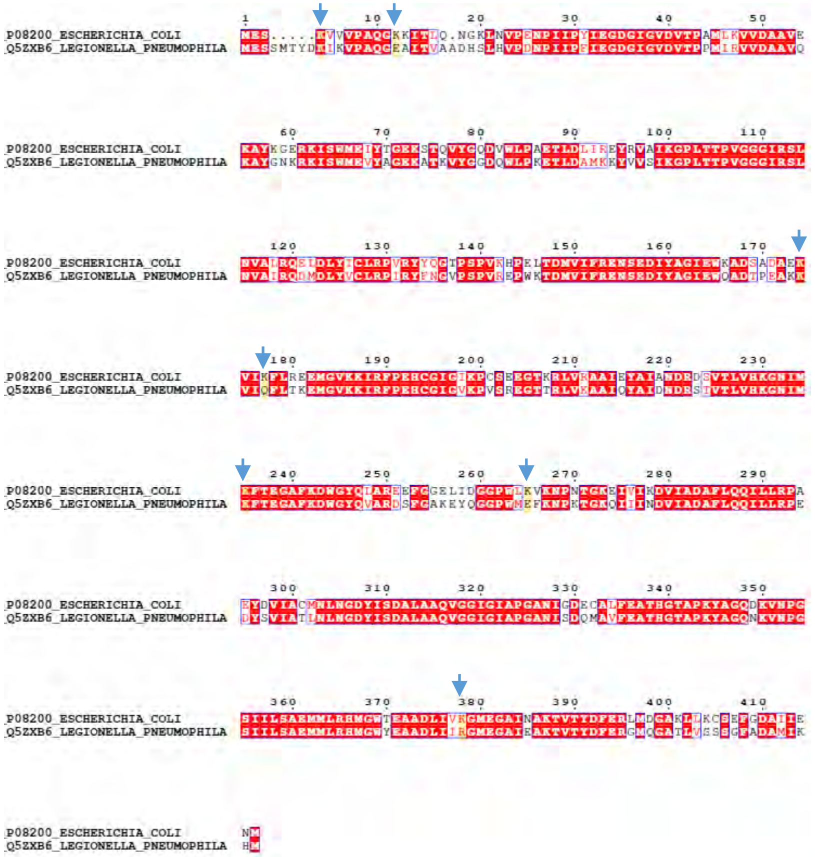

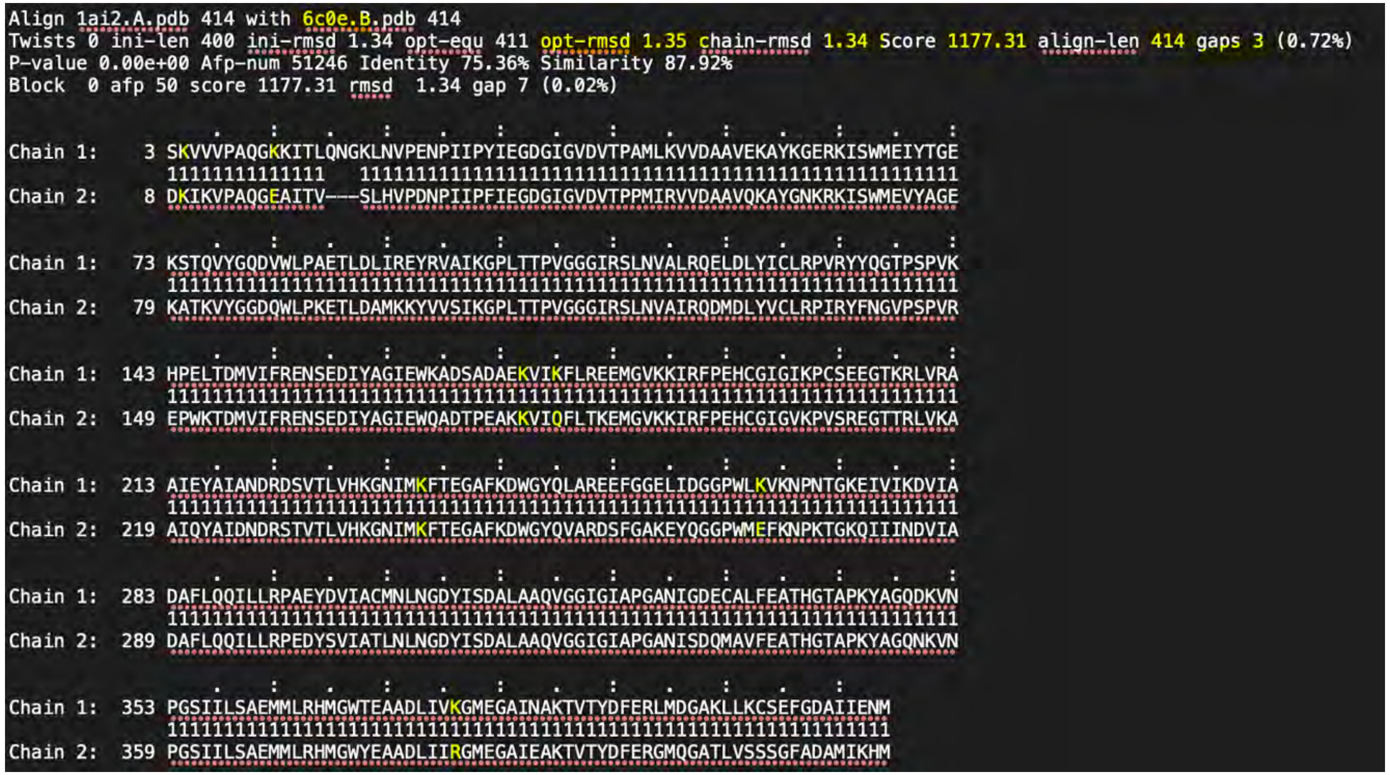

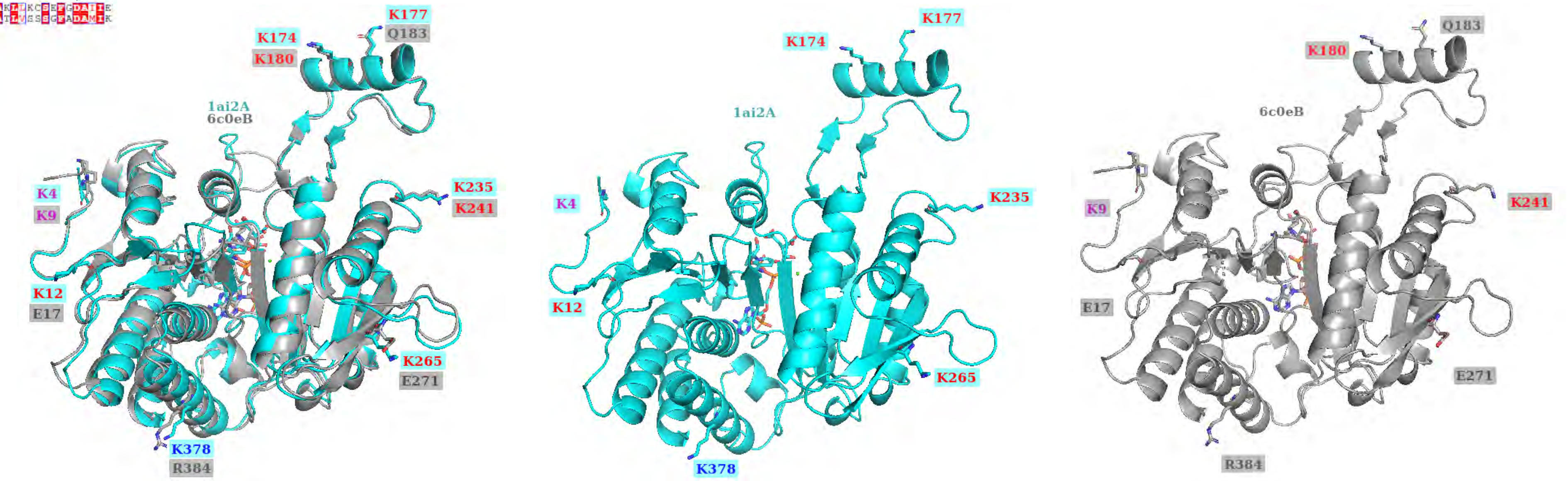

UniProt ID: Q72IW9

PDB ID: 1X0L_A

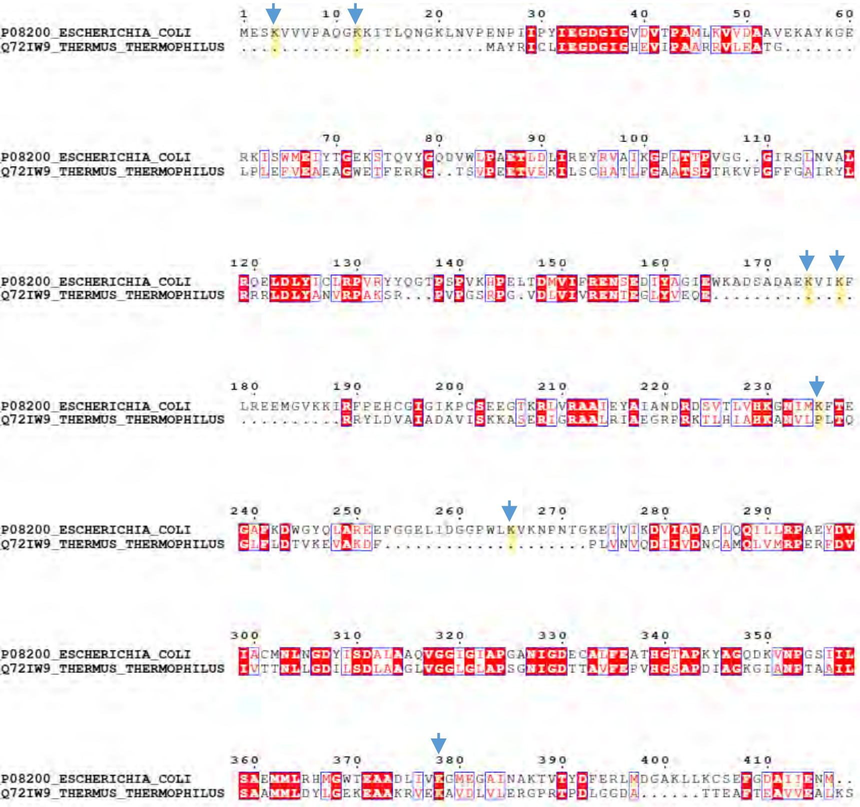

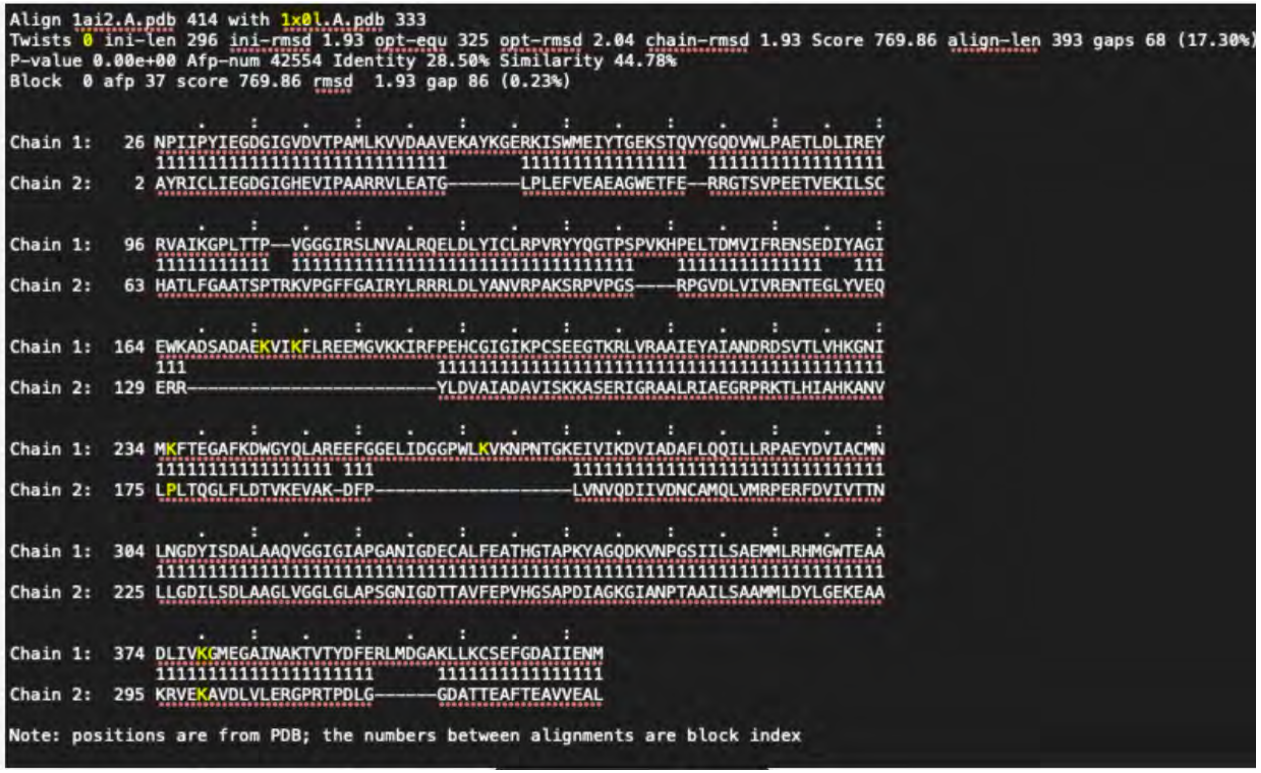

```
Align 1ai2.A.pdb 414 with 1x0l.A.pdb 333
Twists 0 ini-len 296 ini-rmsd 1.93 opt-equ 325 opt-rmsd 2.04 chain-rmsd 1.93 Score 769.86 align-len 393 gaps 68 (17.30%)
P-value 0.00e+00 Afp-num 42554 Identity 28.50% Similarity 44.78%
Block  0 afp 37 score 769.86 rmsd  1.93 gap 86 (0.23%)

Chain 1:   26 NPIIPYIEGDGIGVDVTPAMLKVVDAAVEKAYKGERKISWMEIYTGEKSTQVYGQDVWLPAETLDLIREY
              1111111111111111111111111111       1111111111111111  11111111111111111
Chain 2:    2 AYRICLIEGDGIGHEVIPAARRVLEATG-------LPLEFVEAEAGWETFE--RRGTSVPEETVEKILSC

Chain 1:   96 RVAIKGPLTTP--VGGGIRSLNVALRQELDLYICLRPVRYYQGTPSPVKHPELTDMVIFRENSEDIYAGI
              11111111111  11111111111111111111111111111111     11111111111111   111
Chain 2:   63 HATLFGAATSPTRKVPGFFGAIRYLRRLDLYANVRPAKSRPVPGS-----RPGVDLVIVRENTEGLYVEQ

Chain 1:  164 EWKADSADAEKVIKFLREEMGVKKIRFPEHCGIGIKPCSEEGTKRLVRAAIEYAIANDRDSVTLVHKGNI
              111                        1111111111111111111111111111111111111111111
Chain 2:  129 ERR------------------------YLDVAIADAVISKKASERIGRAALRIAEGRPRKTLHIAHKANV

Chain 1:  234 MKFTEGAFKDWGYQLAREEFGGELIDGGPWLKVKNPNTGKEIVIKDVIADAFLQQILLRPAEYDVIACMN
              11111111111111111 111                   111111111111111111111111111111
Chain 2:  175 LPLTQGLFLDTVKEVAK-DFP-------------------LVNVQDIIVDNCAMQLVMRPERFDVIVTTN

Chain 1:  304 LNGDYISDALAAQVGGIGIAPGANIGDECALFEATHGTAPKYAGQDKVNPGSIILSAEMMLRHMGWTEAA
              1111111111111111111111111111111111111111111111111111111111111111111111
Chain 2:  225 LLGDILSDLAAGLVGGLGLAPSGNIGDTTAVFEPVHGSAPDIAGKGIANPTAAILSAAMMLDYLGEKEAA

Chain 1:  374 DLIVKGMEGAINAKTVTYDFERLMDGAKLLKCSEFGDAIIENM
              111111111111111111111      1111111111111111
Chain 2:  295 KRVEKAVDLVLERGPRTPDLG------GDATTEAFTEAVVEAL

Note: positions are from PDB; the numbers between alignments are block index
```

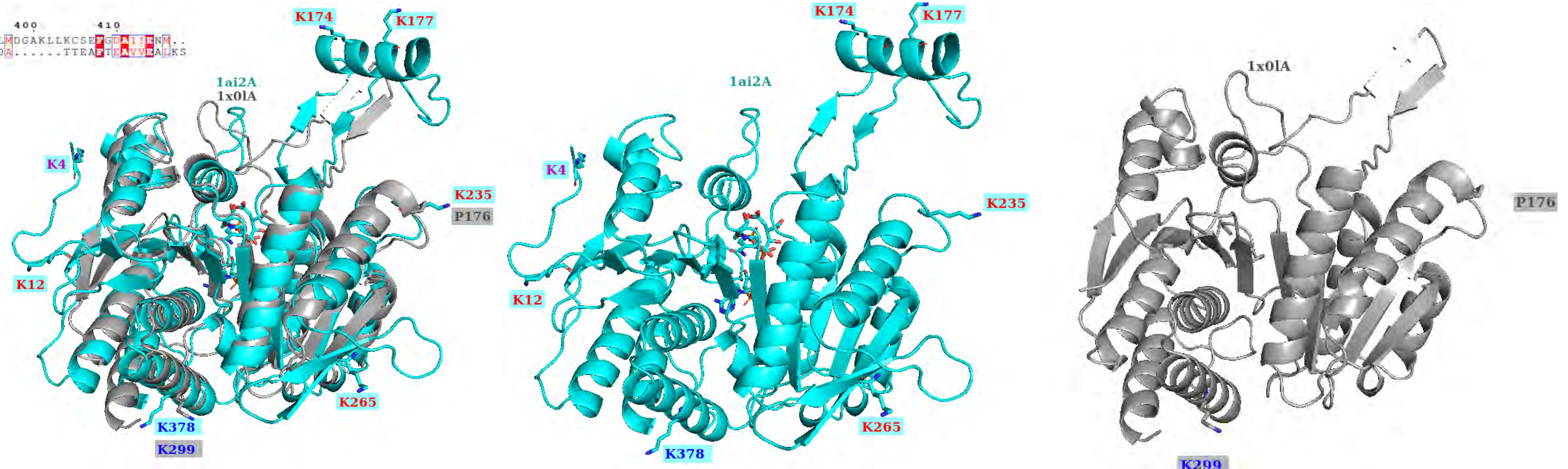

UniProt ID: Q72IW9

PDB ID: 3AH3_C

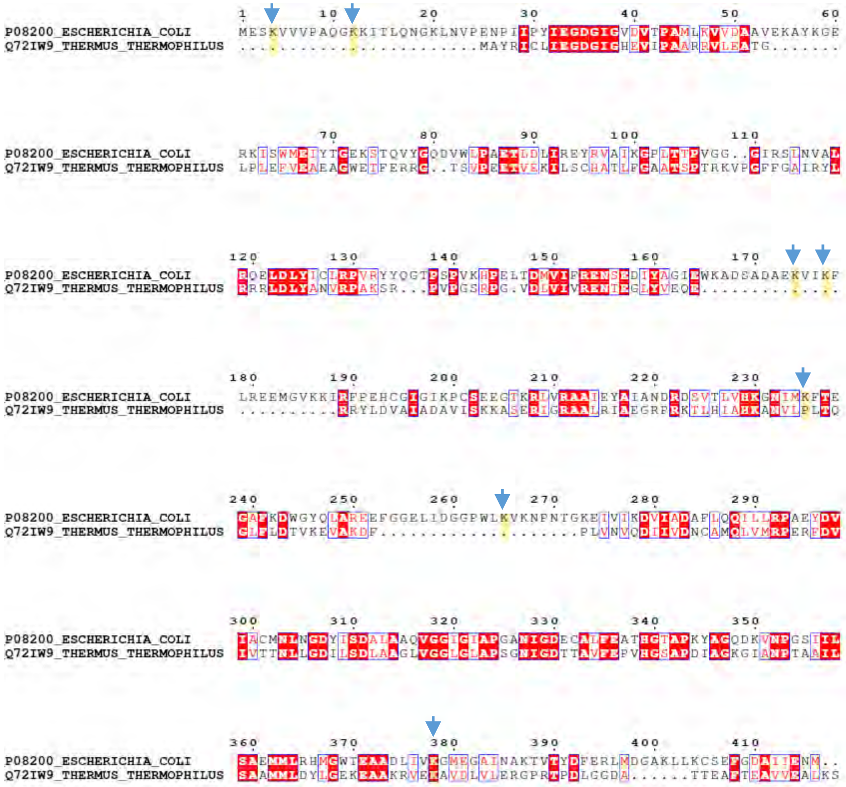

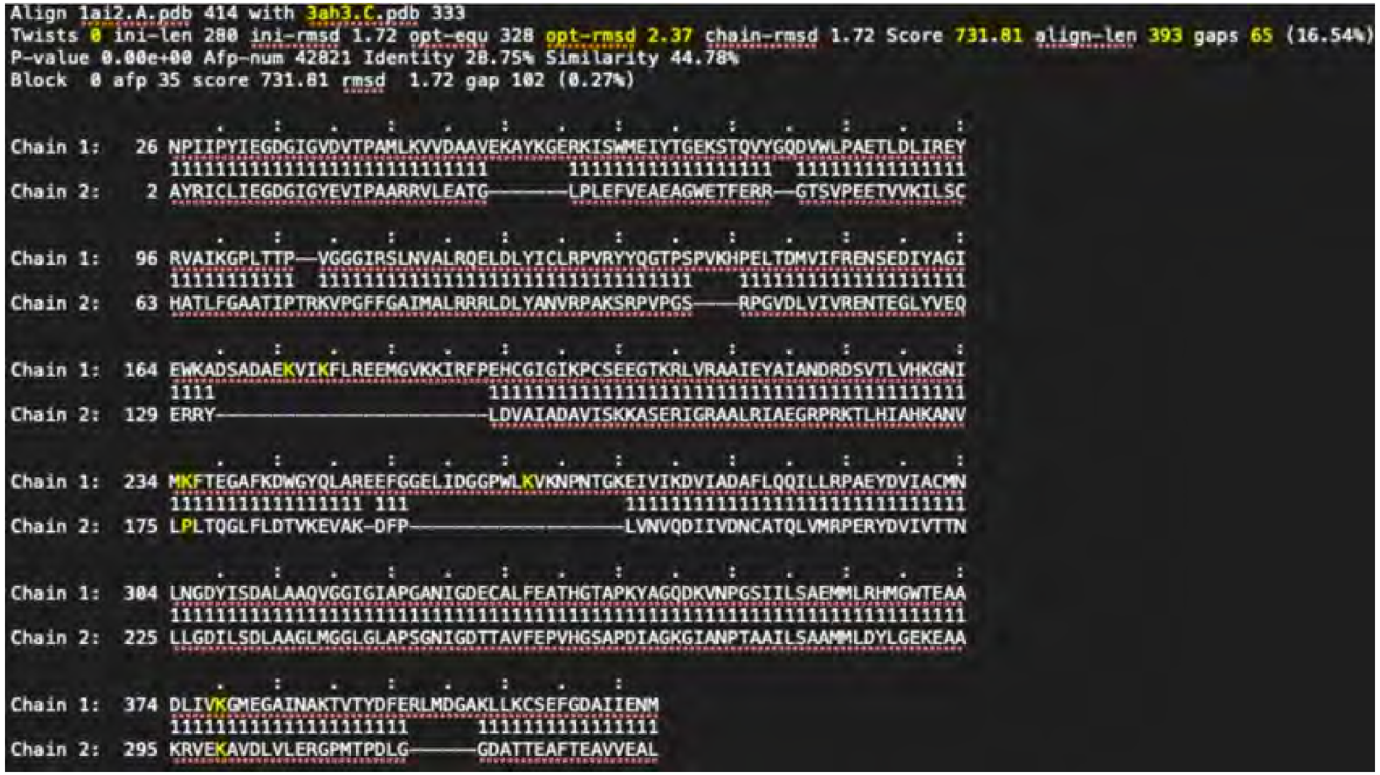

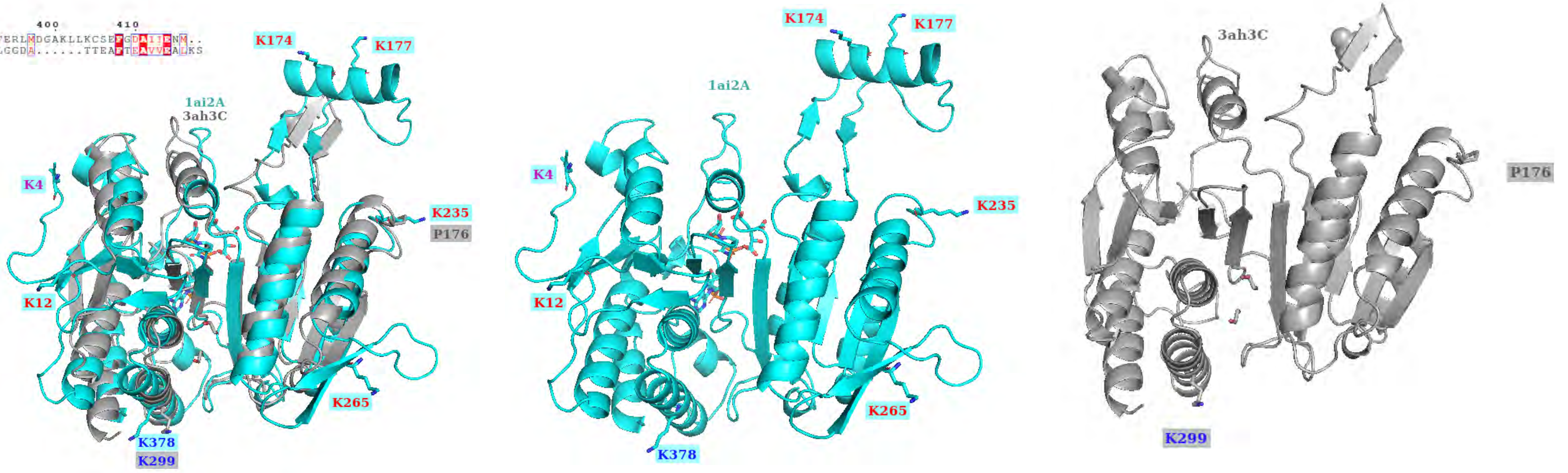

UniProt ID: Q72IW9

PDB ID: 4YB4_A

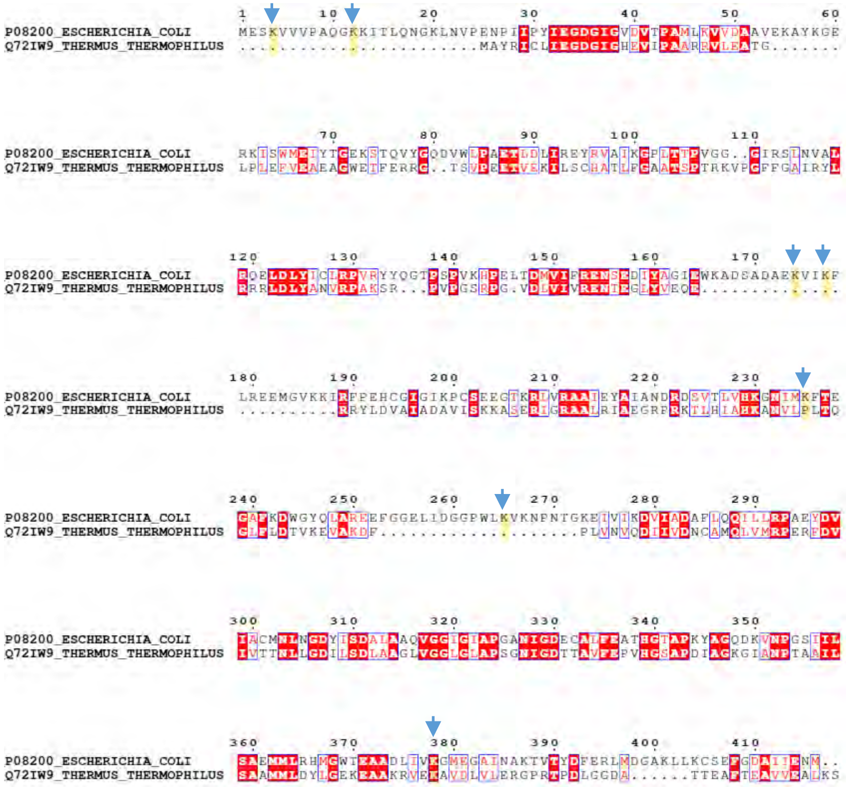


```
Align 1ai2.A.pdb 414 with 4yb4.A.pdb 333
Twists 0 ini-len 304 ini-rmsd 1.61 opt-equ 328 opt-rmsd 1.65 chain-rmsd 1.61 Score 811.66 align-len 393 gaps 65 (16.54%)
P-value 0.00e+00 Afp-num 42695 Identity 28.50% Similarity 44.78%
Block  0 afp 38 score 811.66 rmsd  1.61 gap 79 (0.21%)

Chain 1:   26 NPIIPYIEGDGIGVDVTPAMLKVVDAAVEKAYKGERKISWMEIYTGEKSTQVYGQDVWLPAETLDLIREY
              1111111111111111111111111111       1111111111111111  11111111111111111
Chain 2:    2 AYRICLIEGDGIGHEVIPAARRVLEATG-------LPLEFVEAEAGWETFE--RRGTSVPEETVEKILSC

Chain 1:   96 RVAIKGPLTTPV--GGGIRSLNVALRQELDLYICLRPVRYYQGTPSPVKHPELTDMVIFRENSEDIYAGI
              111111111111  111111111111111111111111111111111    11111111111111111111
Chain 2:   63 HATLFGAATSPTRKVPGFFGAIRYLRRLDLYANVRPAKSRPVPGS----RPGVDLVIVRENTEGLYVEQ

Chain 1:  164 EWKADSADAEKVIKFLREEMGVKKIRFPEHCGIGIKPCSEEGTKRLVRAAIEYAIANDRDSVTLVHKGNI
              11111                       11111111111111111111111111111111111111111
Chain 2:  129 ERRYL-----------------------DVAIADAVISKKASERIGRAALRIAEGRPRKTLHIAHKANV

Chain 1:  234 MKFTEGAFKDWGYQLAREEFGGELIDGGPWLKVKNPNTGKEIVIKDVIADAFLQQILLRPAEYDVIACMN
              11111111111111111 111                  1111111111111111111111111111111
Chain 2:  175 LPLTQGLFLDTVKEVAK-DFP------------------LVNVQDIIVDNCAMQLVMRPERFDVIVTTN

Chain 1:  304 LNGDYISDALAAQVGGIGIAPGANIGDECALFEATHGTAPKYAGQDKVNPGSILLSAEMMLRHMGWTEAA
              1111111111111111111111111111111111111111111111111111111111111111111111
Chain 2:  225 LLGDILSDLAAGLVGGLGLAPSGNIGDTTAVFEPVHGSAPDIAGKGIANPTAAILSAAMMLDYLGEKEAA

Chain 1:  374 DLIVKGMEGAINAKTVTYDFERLMDGAKLLKCSEFGDAIIENM
              111111111111111111111      1111111111111111
Chain 2:  295 KRVEKAVDLVLERGPRTPDLG------GDATTEAFTEAVVEAL

Note: positions are from PDB; the numbers between alignments are block index
```

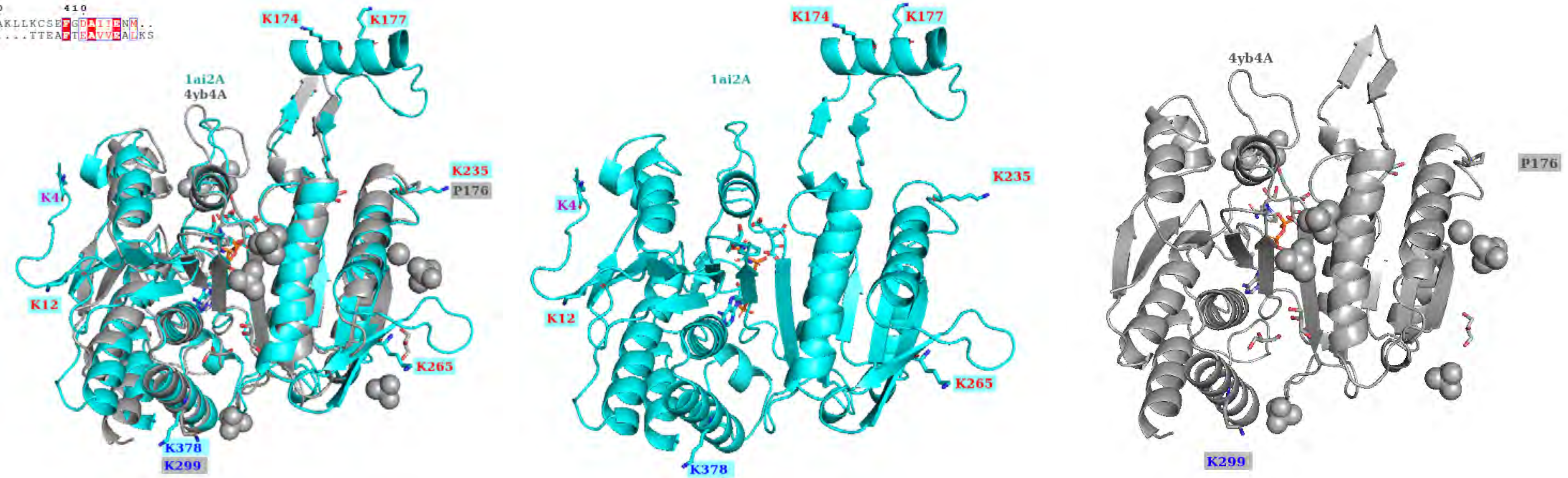

UniProt ID: Q8GAX0

PDB ID: 2D4V_C

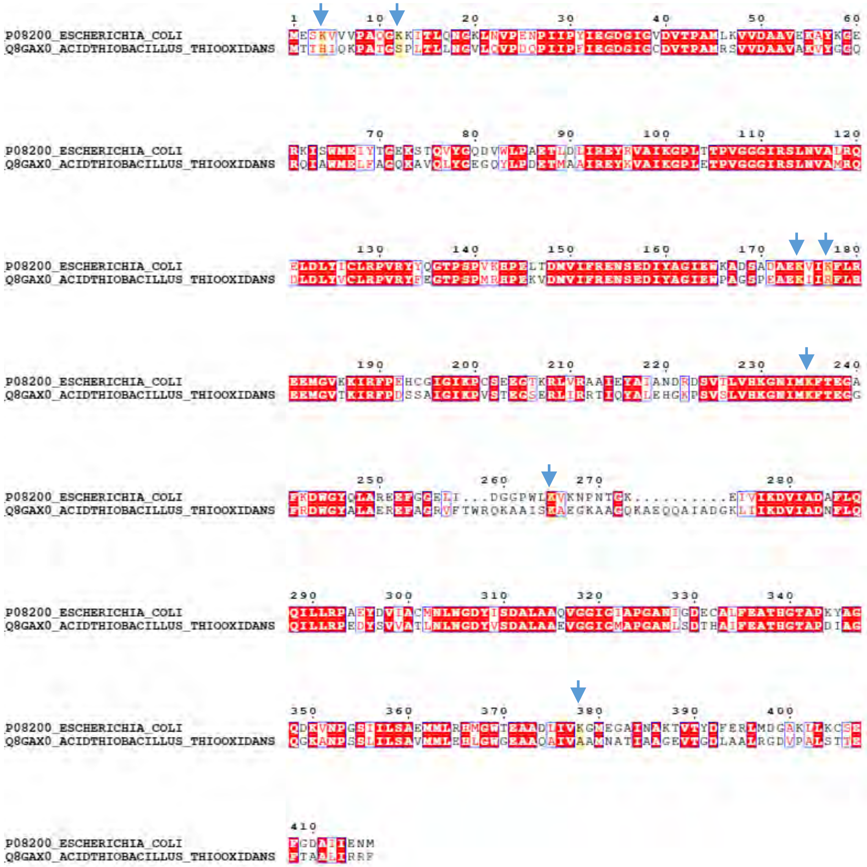

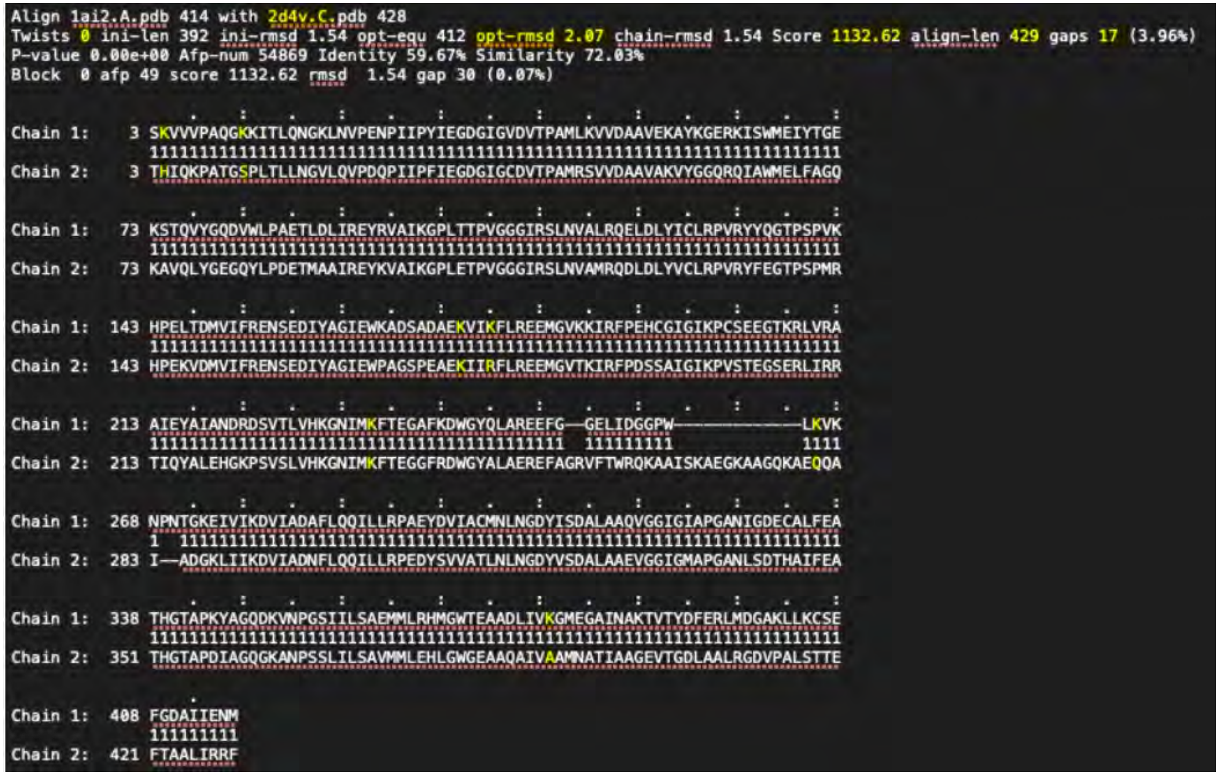

```
Align 1ai2.A.pdb 414 with 2d4v.C.pdb 428
Twists 0 ini-len 392 ini-rmsd 1.54 opt-equ 412 opt-rmsd 2.07 chain-rmsd 1.54 Score 1132.62 align-len 429 gaps 17 (3.96%)
P-value 0.00e+00 Afp-num 54869 Identity 59.67% Similarity 72.03%
Block  0 afp 49 score 1132.62 rmsd  1.54 gap 30 (0.07%)

             .    :    .    :    .    :    .    :    .    :    .    :    .    :
Chain 1:    3 SKVVVPAQGKKITLQNGKLNVPENPIIPYIEGDGIGVDVTPAMLKVVDAAVEKAYKGERKISWMEIYTGE
              1111111111111111111111111111111111111111111111111111111111111111111111
Chain 2:    3 THIQKPATGSPLTLLNGVLQVPDQPIIPFIEGDGIGCDVTPAMRSVVDAAVAKVYGGQRQIAWMELFAGQ

             .    :    .    :    .    :    .    :    .    :    .    :    .    :
Chain 1:   73 KSTQVYGQDVWLPAETLDLIREYRVAIKGPLTTPVGGGIRSLNVALRQELDLYICLRPVRYYQGTPSPVK
              1111111111111111111111111111111111111111111111111111111111111111111111
Chain 2:   73 KAVQLYGEGQYLPDETMAAIREYKVAIKGPLETPVGGGIRSLNVAMRQDLDLYVCLRPVRYFEGTPSPMR

             .    :    .    :    .    :    .    :    .    :    .    :    .    :
Chain 1:  143 HPELTDMVIFRENSEDIYAGIEWKADSADAEKVIKFLREEMGVKKIRFPEHCGIGIKPCSEEGTKRLVRA
              1111111111111111111111111111111111111111111111111111111111111111111111
Chain 2:  143 HPEKVDMVIFRENSEDIYAGIEWPAGSPEAEKIIRFLREEMGVTKIRFPDSSAIGIKPVSTEGSERLIRR

             .    :    .    :    .    :    .    :    .    :    .    :    .    :
Chain 1:  213 AIEYAIANDRDSVTLVHKGNIMKFTEGAFKDWGYQLAREEFG--GELIDGGPW-------------LKVK
              1111111111111111111111111111111111111111  111111111             1111
Chain 2:  213 TIQYALEHGKPSVSLVHKGNIMKFTEGGFRDWGYALAEREFAGRVFTWRQKAAISKAEGKAAGQKAEQQA

             .    :    .    :    .    :    .    :    .    :    .    :    .    :
Chain 1:  268 NPNTGKEIVIKDVIADAFLQQILLRPAEYDVIACMNLNGDYISDALAAQVGGIGIAPGANIGDECALFEA
              1  11111111111111111111111111111111111111111111111111111111111111111
Chain 2:  283 I--ADGKLIIKDVIADNFLQQILLRPEDYSVVATLNLNGDYVSDALAAEVGGIGMAPGANLSDTHAIFEA

             .    :    .    :    .    :    .    :    .    :    .    :    .    :
Chain 1:  338 THGTAPKYAGQDKVNPGSIILSAEMMLRHMGWTEAADLIVKGMEGAINAKTVTYDFERLMDGAKLLKCSE
              1111111111111111111111111111111111111111111111111111111111111111111111
Chain 2:  351 THGTAPDIAGQGKANPSSLILSAVMMLEHLGWGEAAQAIVAAMNATIAAGEVTGDLAALRGDVPALSTTE

             .
Chain 1:  408 FGDAIIENM
              111111111
Chain 2:  421 FTAALIRRF
```

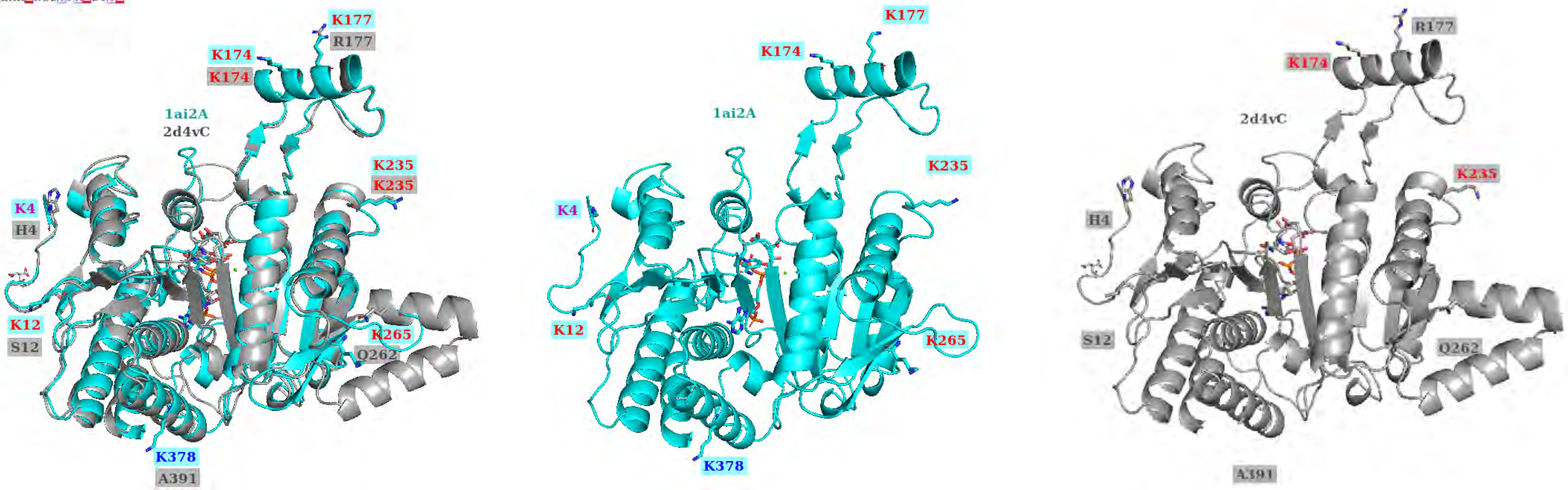

UniProt ID: Q96YK6

PDB ID: 2DHT_A

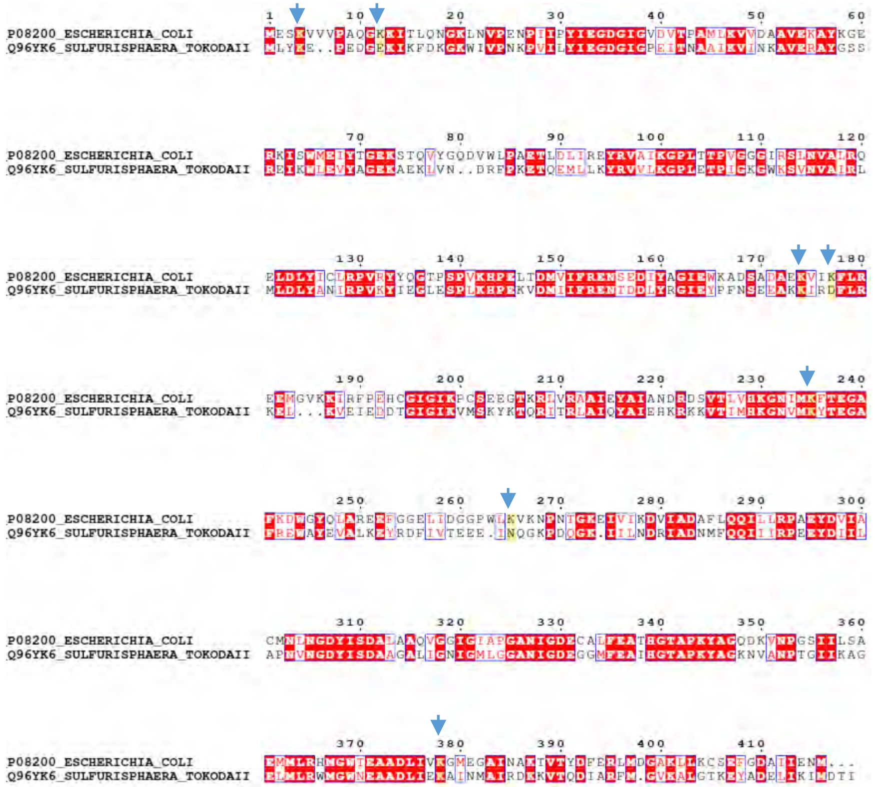

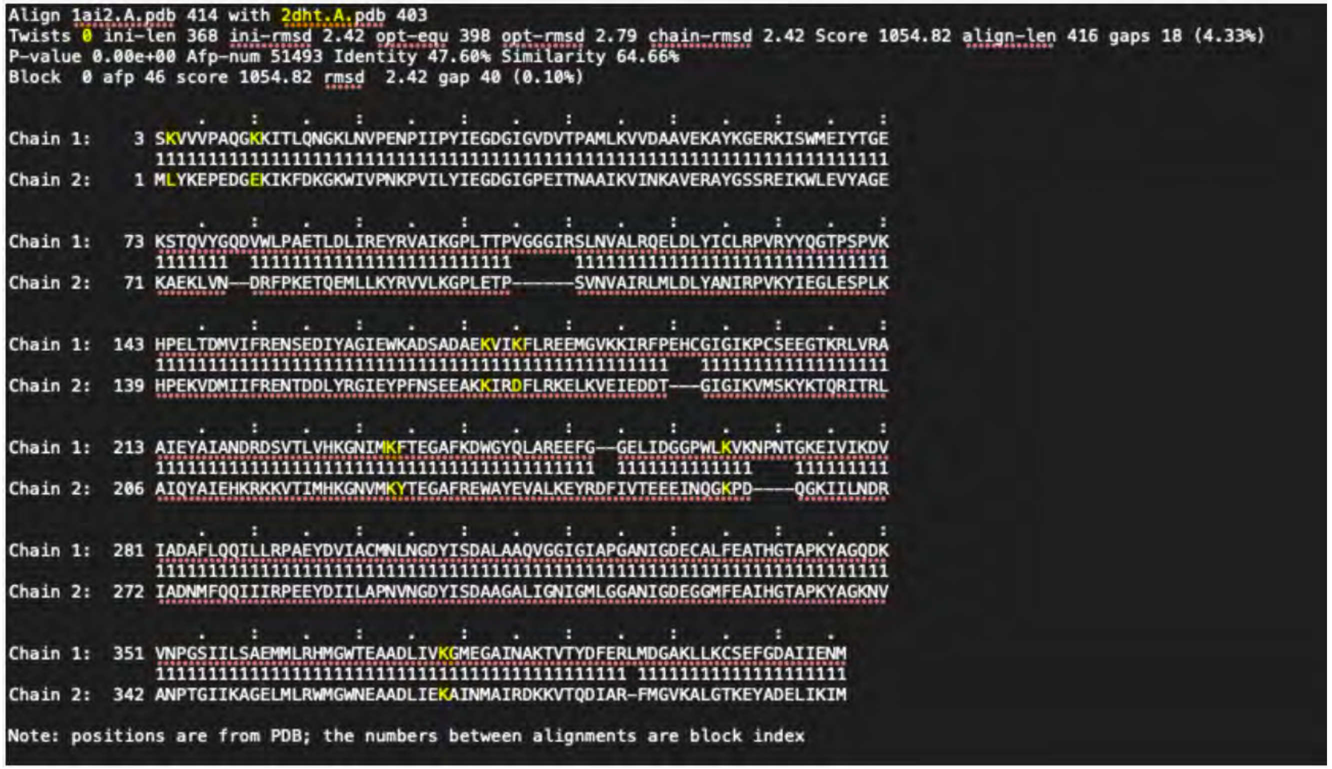

```
Align 1ai2.A.pdb 414 with 2dht.A.pdb 403
Twists 0 ini-len 368 ini-rmsd 2.42 opt-equ 398 opt-rmsd 2.79 chain-rmsd 2.42 Score 1054.82 align-len 416 gaps 18 (4.33%)
P-value 0.00e+00 Afp-num 51493 Identity 47.60% Similarity 64.66%
Block  0 afp 46 score 1054.82 rmsd  2.42 gap 40 (0.10%)

                  .    :    .    :    .    :    .    :    .    :    .    :    .    :
Chain 1:    3 SKVVVPAQGKKITLQNGKLNVPENPIIPYIEGDGIGVDVTPAMLKVVDAAVEKAYKGERKISWMEIYTGE
              1111111111111111111111111111111111111111111111111111111111111111111111
Chain 2:    1 MLYKEPEDGEKIKFDKGKWIVPNKPVILYIEGDGIGPEITNAAIKVINKAVERAYGSSREIKWLEVYAGE

                  .    :    .    :    .    :    .    :    .    :    .    :    .    :
Chain 1:   73 KSTQVYGQDVWLPAETLDLIREYRVAIKGPLTTPVGGGIRSLNVALRQELDLYICLRPVRYYQGTPSPVK
              1111111  1111111111111111111111111      111111111111111111111111111111
Chain 2:   71 KAEKLVN--DRFPKETQEMLLKYRVVLKGPLETP------SVNVAIRLMLDLYANIRPVKYIEGLESPLK

                  .    :    .    :    .    :    .    :    .    :    .    :    .    :
Chain 1:  143 HPELTDMVIFRENSEDIYAGIEWKADSADAEKVIKFLREEMGVKKIRFPEHCGIGIKPCSEEGTKRLVRA
              11111111111111111111111111111111111111111111111111   111111111111111111
Chain 2:  139 HPEKVDMIIFRENTDDLYRGIEYPFNSEEAKKIRDFLRKELKVEIEDDT---GIGIKVMSKYKTQRITRL

                  .    :    .    :    .    :    .    :    .    :    .    :    .    :
Chain 1:  213 AIEYAIANDRDSVTLVHKGNIMKFTEGAFKDWGYQLAREEFG--GELIDGGPWLKVKNPNTGKEIVIKDV
              111111111111111111111111111111111111111111  1111111111111     111111111
Chain 2:  206 AIQYAIEHKRKKVTIMHKGNVMKYTEGAFREWAYEVALKEYRDFIVTEEEINQGKPD-----QGKIILNDR

                  .    :    .    :    .    :    .    :    .    :    .    :    .    :
Chain 1:  281 IADAFLQQILLRPAEYDVIACMNLNGDYISDALAAQVGGIGIAPGANIGDECALFEATHGTAPKYAGQDK
              1111111111111111111111111111111111111111111111111111111111111111111111
Chain 2:  272 IADNMFQQIIIRPEEYDIILAPNVNGDYISDAAGALIGNIGMLGGANIGDEGGMFEAIHGTAPKYAGKNV

                  .    :    .    :    .    :    .    :    .    :    .    :    .
Chain 1:  351 VNPGSIILSAEMMLRHMGWTEAADLIVKGMEGAINAKTVTYDFERLMDGAKLLKCSEFGDAIIENM
              111111111111111111111111111111111111111111111 111111111111111111111
Chain 2:  342 ANPTGIIKAGELMLRWMGWNEAADLIEKAINMAIRDKKVTQDIAR-FMGVKALGTKEYADELIKIM

Note: positions are from PDB; the numbers between alignments are block index
```

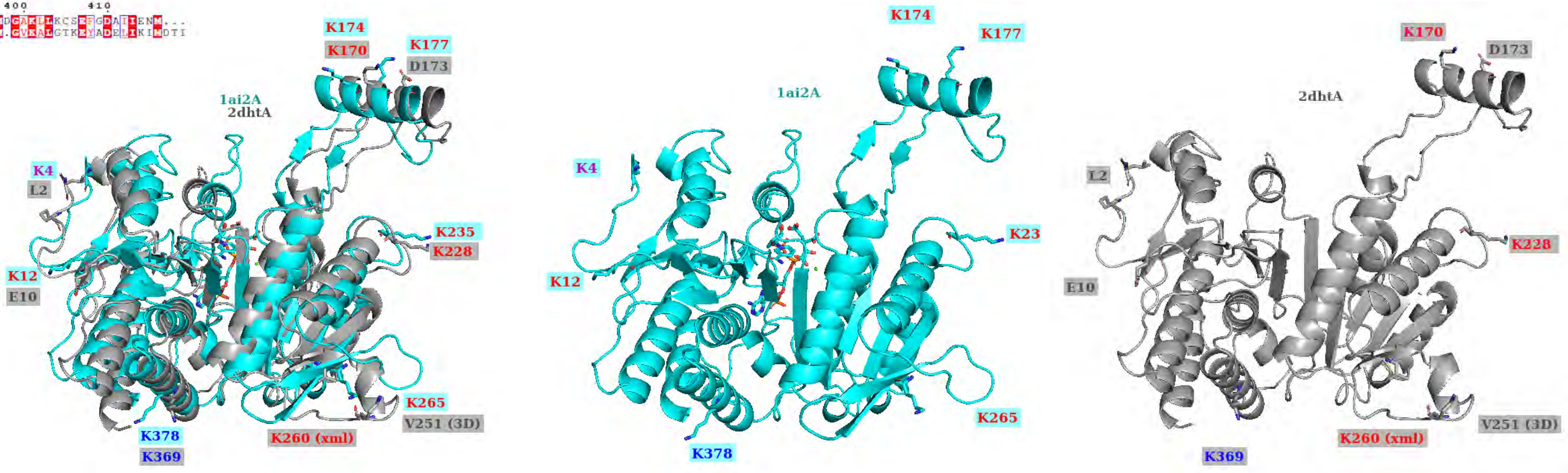

UniProt ID: Q96YK6

PDB ID: 2E0C_A

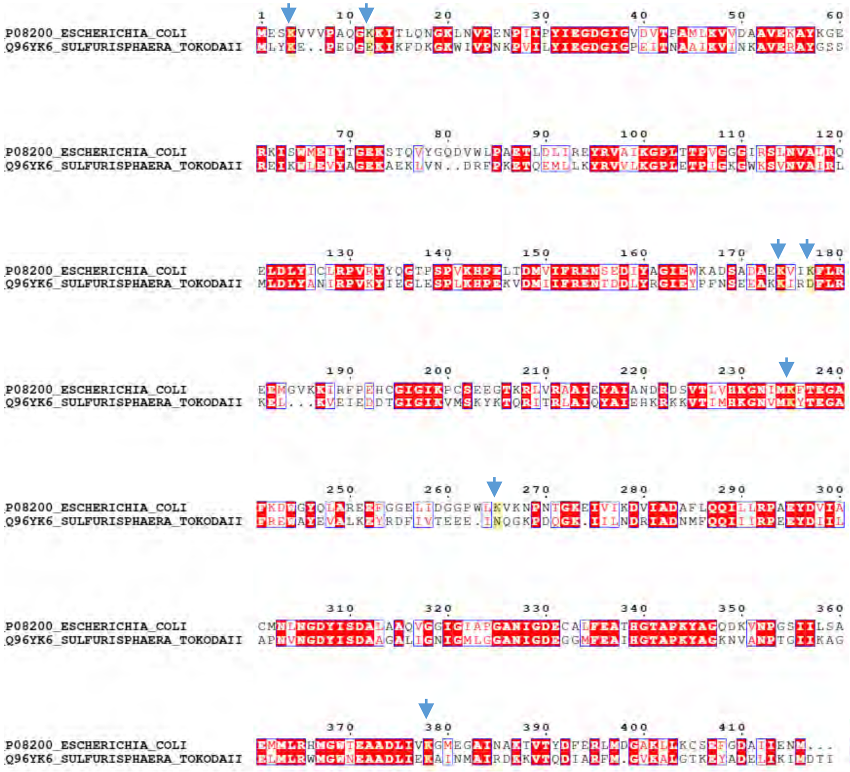

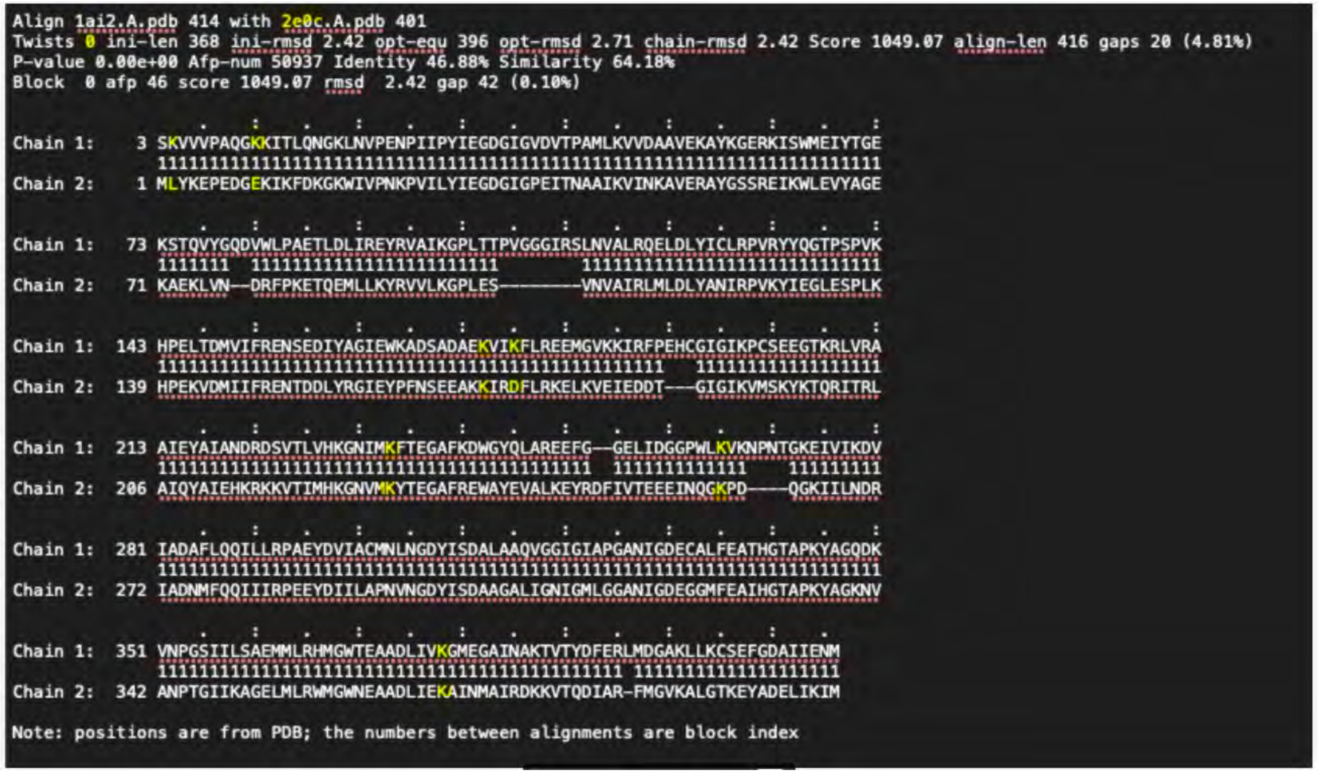

```
Align 1ai2.A.pdb 414 with 2e0c.A.pdb 401
Twists 0 ini-len 368 ini-rmsd 2.42 opt-equ 396 opt-rmsd 2.71 chain-rmsd 2.42 Score 1049.07 align-len 416 gaps 20 (4.81%)
P-value 0.00e+00 Afp-num 50937 Identity 46.88% Similarity 64.18%
Block  0 afp 46 score 1049.07 rmsd  2.42 gap 42 (0.10%)

                  .    :    .    :    .    :    .    :    .    :    .    :    .    :
Chain 1:    3 SKVVVPAQGKKITLQNGKLNVPENPIIPYIEGDGIGVDVTPAMLKVVDAAVEKAYKGERKISWMEIYTGE
              1111111111111111111111111111111111111111111111111111111111111111111111
Chain 2:    1 MLYKEPEDGEKIKFDKGKWIVPNKPVILYIEGDGIGPEITNAAIKVINKAVERAYGSSREIKWLEVYAGE

                  .    :    .    :    .    :    .    :    .    :    .    :    .    :
Chain 1:   73 KSTQVYGQDVWLPAETLDLIREYRVAIKGPLTTPVGGGIRSLNVALRQELDLYICLRPVRYYQGTPSPVK
              1111111  111111111111111111111111         1111111111111111111111111111
Chain 2:   71 KAEKLVN--DRFPKETQEMLLKYRVVLKGPLES---------VNVAIRLMLDLYANIRPVKYIEGLESPLK

                  .    :    .    :    .    :    .    :    .    :    .    :    .    :
Chain 1:  143 HPELTDMVIFRENSEDIYAGIEWKADSADAEKVIKFLREEMGVKKIRFPEHCGIGIKPCSEEGTKRLVRA
              1111111111111111111111111111111111111111111111111   111111111111111111
Chain 2:  139 HPEKVDMIIFRENTDDLYRGIEYPFNSEEAKKIRDFLRKELKVEIEDDT---GIGIKVMSKYKTQRITRL

                  .    :    .    :    .    :    .    :    .    :    .    :    .    :
Chain 1:  213 AIEYAIANDRDSVTLVHKGNIMKFTEGAFKDWGYQLAREEFG--GELIDGGPWLKVKNPNTGKEIVIKDV
              111111111111111111111111111111111111111111  1111111111111    111111111
Chain 2:  206 AIQYAIEHKRKKVTIMHKGNVMKYTEGAFREWAYEVALKEYRDFIVTEEEINQGKPD----QGKIILNDR

                  .    :    .    :    .    :    .    :    .    :    .    :    .    :
Chain 1:  281 IADAFLQQILLRPAEYDVIACMNLNGDYISDALAAQVGGIGIAPGANIGDECALFEATHGTAPKYAGQDK
              1111111111111111111111111111111111111111111111111111111111111111111111
Chain 2:  272 IADNMFQQIIIRPEEYDIILAPNVNGDYISDAAGALIGNIGMLGGANIGDEGGMFEAIHGTAPKYAGKNV

                  .    :    .    :    .    :    .    :    .    :    .    :    .
Chain 1:  351 VNPGSIILSAEMMLRHMGWTEAADLIVKGMEGAINAKTVTYDFERLMDGAKLLKCSEFGDAIIENM
              111111111111111111111111111111111111111111111 11111111111111111111
Chain 2:  342 ANPTGIIKAGELMLRWMGWNEAADLIEKAINMAIRDKKVTQDIAR-FMGVKALGTKEYADELIKIM

Note: positions are from PDB; the numbers between alignments are block index
```

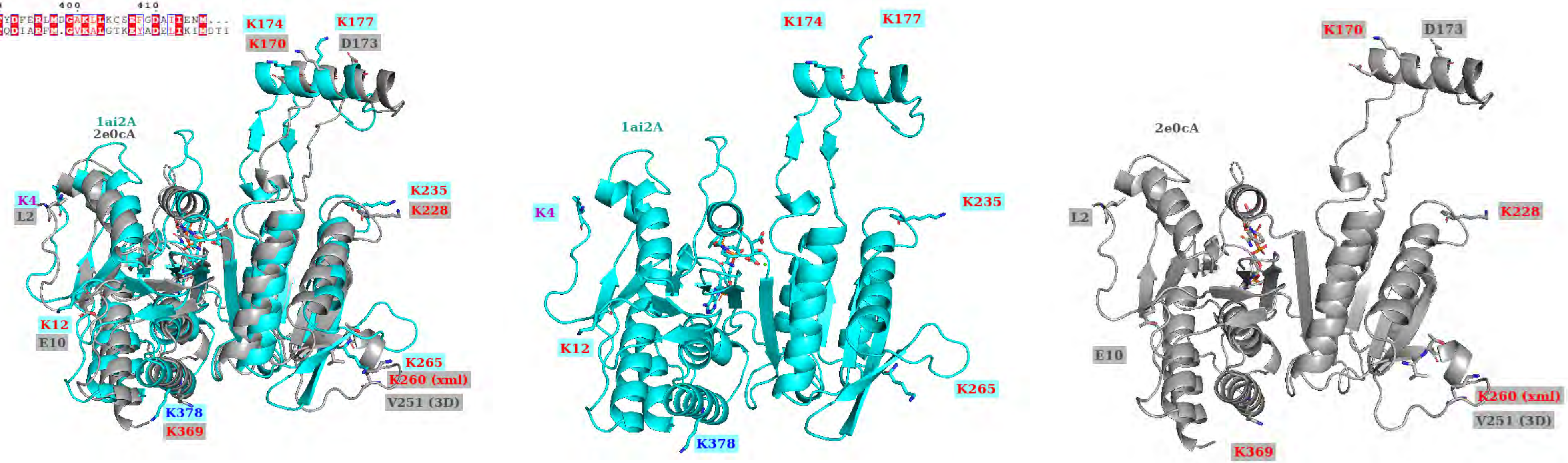

UniProt ID: Q96YK6

PDB ID: 2E5M_A

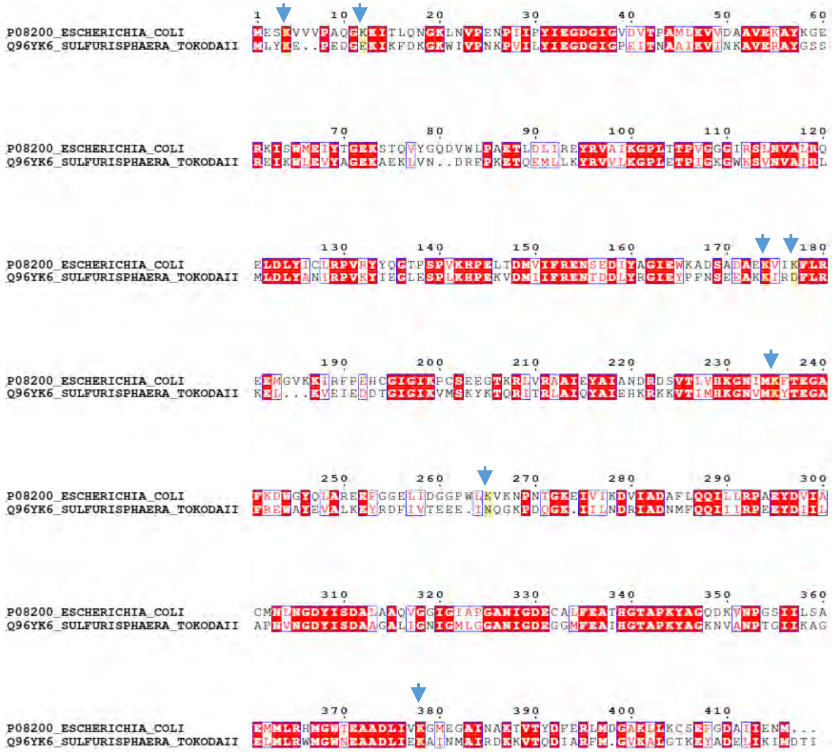

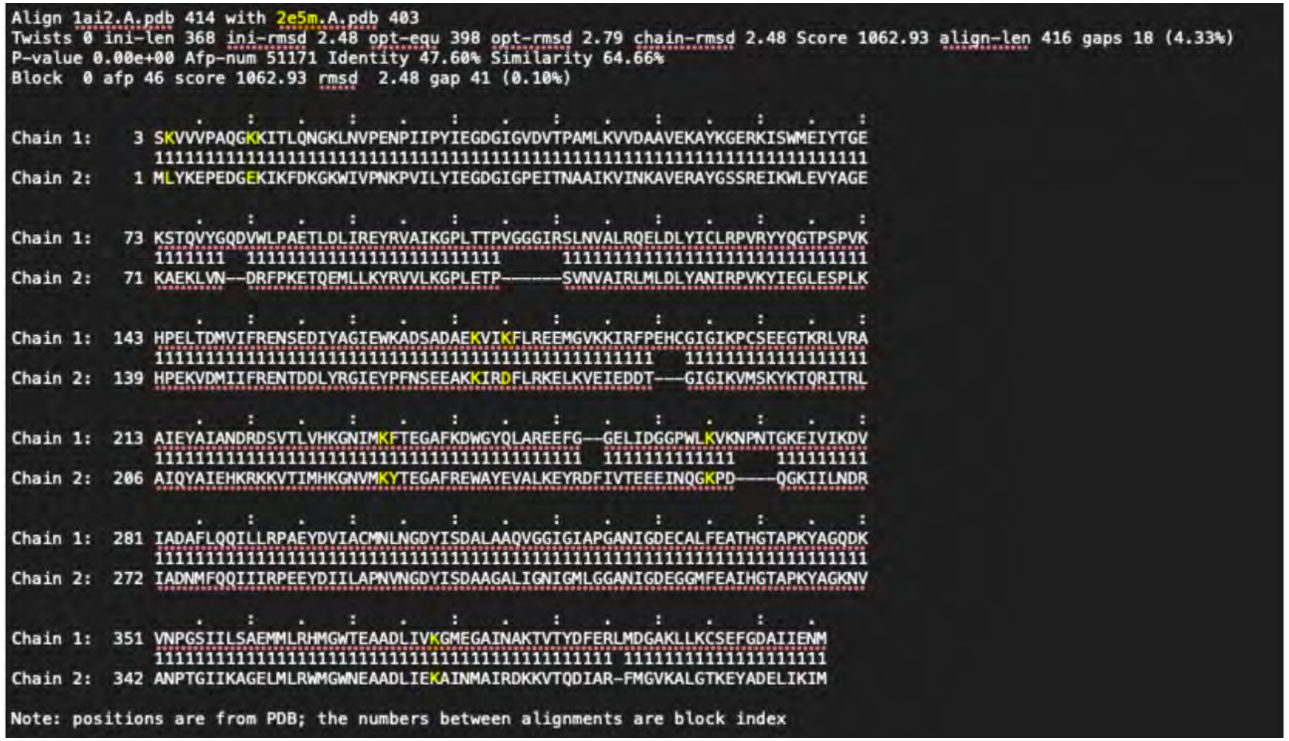

```
Align 1ai2.A.pdb 414 with 2e5m.A.pdb 403
Twists 0 ini-len 368 ini-rmsd 2.48 opt-equ 398 opt-rmsd 2.79 chain-rmsd 2.48 Score 1062.93 align-len 416 gaps 18 (4.33%)
P-value 0.00e+00 Afp-num 51171 Identity 47.60% Similarity 64.66%
Block  0 afp 46 score 1062.93 rmsd  2.48 gap 41 (0.10%)

                  .    :    .    :    .    :    .    :    .    :    .    :    .    :
Chain 1:    3 SKVVVPAQGKKITLQNGKLNVPENPIIPYIEGDGIGVDVTPAMLKVVDAAVEKAYKGERKISWMEIYTGE
              1111111111111111111111111111111111111111111111111111111111111111111111
Chain 2:    1 MLYKEPEDGEKIKFDKGKWIVPNKPVILYIEGDGIGPEITNAAIKVINKAVERAYGSSREIKWLEVYAGE

                  .    :    .    :    .    :    .    :    .    :    .    :    .    :
Chain 1:   73 KSTQVYGQDVWLPAETLDLIREYRVAIKGPLTTPVGGGIRSLNVALRQELDLYICLRPVRYYQGTPSPVK
              1111111  1111111111111111111111111      111111111111111111111111111111
Chain 2:   71 KAEKLVN--DRFPKETQEMLLKYRVVLKGPLETP------SVNVAIRLMLDLYANIRPVKYIEGLESPLK

                  .    :    .    :    .    :    .    :    .    :    .    :    .    :
Chain 1:  143 HPELTDMVIFRENSEDIYAGIEWKADSADAEKVIKFLREEMGVKKIRFPEHCGIGIKPCSEEGTKRLVRA
              1111111111111111111111111111111111111111111111111   111111111111111111
Chain 2:  139 HPEKVDMIIFRENTDDLYRGIEYPFNSEEAKKIRDFLRKELKVEIEDDT---GIGIKVMSKYKTQRITRL

                  .    :    .    :    .    :    .    :    .    :    .    :    .    :
Chain 1:  213 AIEYAIANDRDSVTLVHKGNIMKFTEGAFKDWGYQLAREEFG--GELIDGGPWLKVKNPNTGKEIVIKDV
              111111111111111111111111111111111111111111  1111111111111    111111111
Chain 2:  206 AIQYAIEHKRKKVTIMHKGNVMKYTEGAFREWAYEVALKEYRDFIVTEEEINQGKPD----QGKIILNDR

                  .    :    .    :    .    :    .    :    .    :    .    :    .    :
Chain 1:  281 IADAFLQQILLRPAEYDVIACMNLNGDYISDALAAQVGGIGIAPGANIGDECALFEATHGTAPKYAGQDK
              1111111111111111111111111111111111111111111111111111111111111111111111
Chain 2:  272 IADNMFQQIIIRPEEYDIILAPNVNGDYISDAAGALIGNIGMLGGANIGDEGGMFEAIHGTAPKYAGKNV

                  .    :    .    :    .    :    .    :    .    :    .    :    .
Chain 1:  351 VNPGSIILSAEMMLRHMGWTEAADLIVKGMEGAINAKTVTYDFERLMDGAKLLKCSEFGDAIIENM
              111111111111111111111111111111111111111111111 11111111111111111111
Chain 2:  342 ANPTGIIKAGELMLRWMGWNEAADLIEKAINMAIRDKKVTQDIAR-FMGVKALGTKEYADELIKIM

Note: positions are from PDB; the numbers between alignments are block index
```

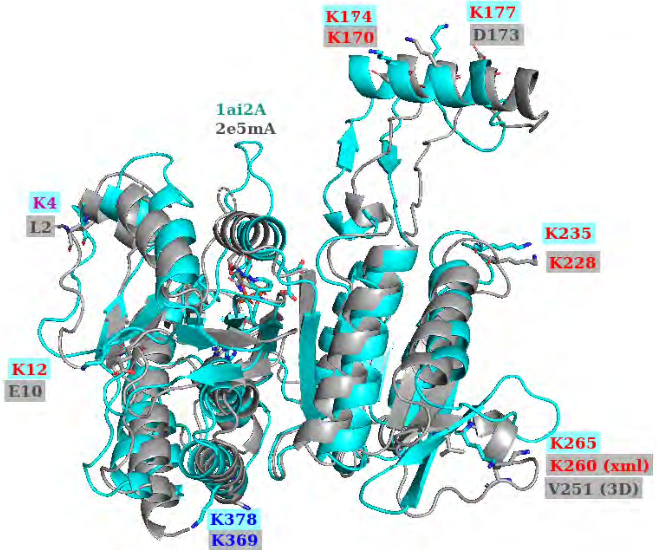

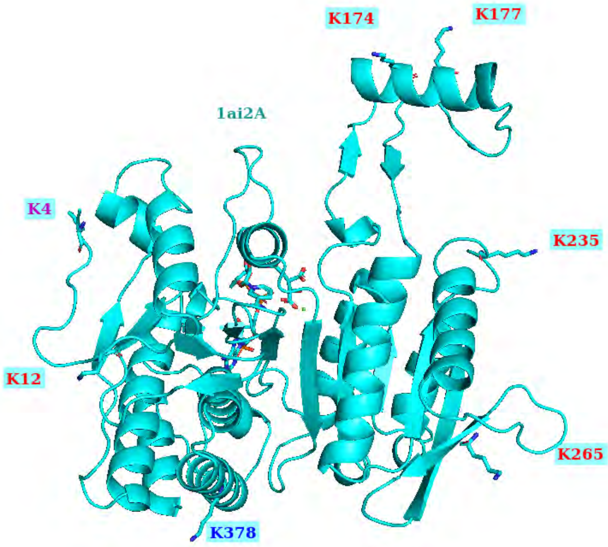

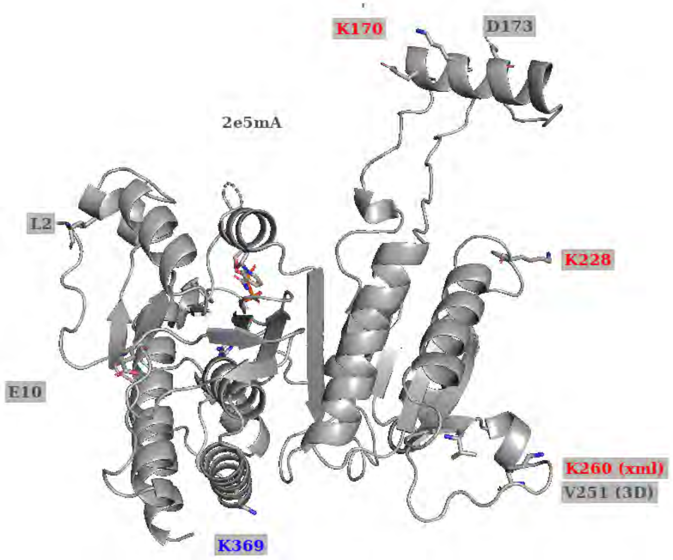

UniProt ID: Q9YE81
PDB ID: 1TYO_A

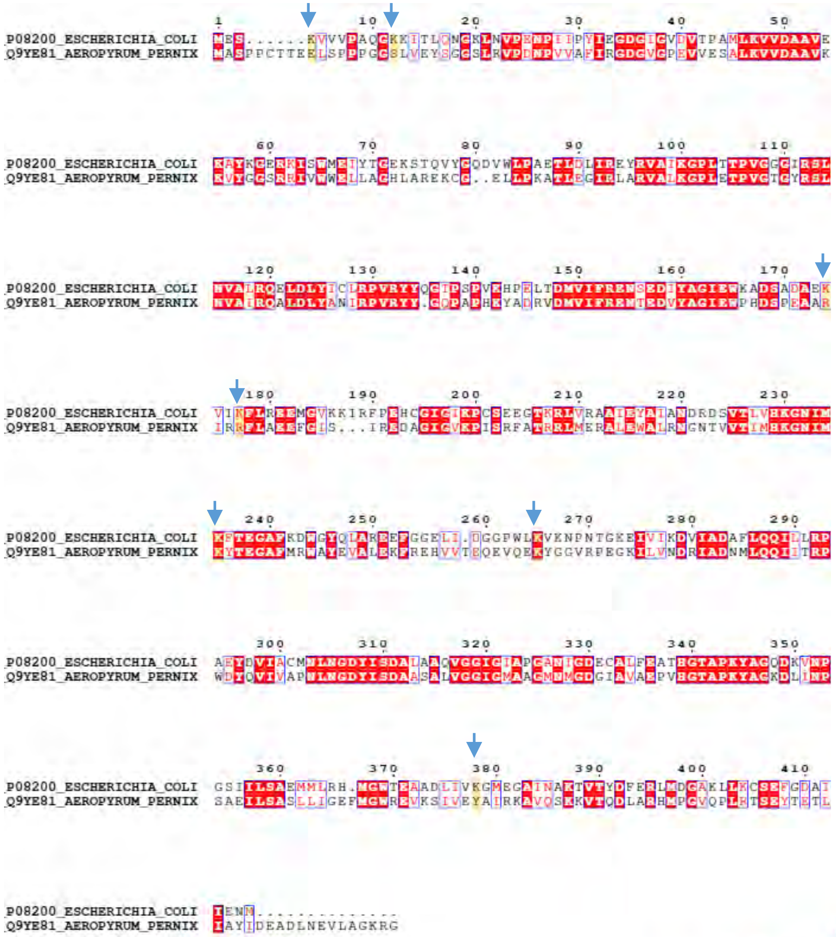

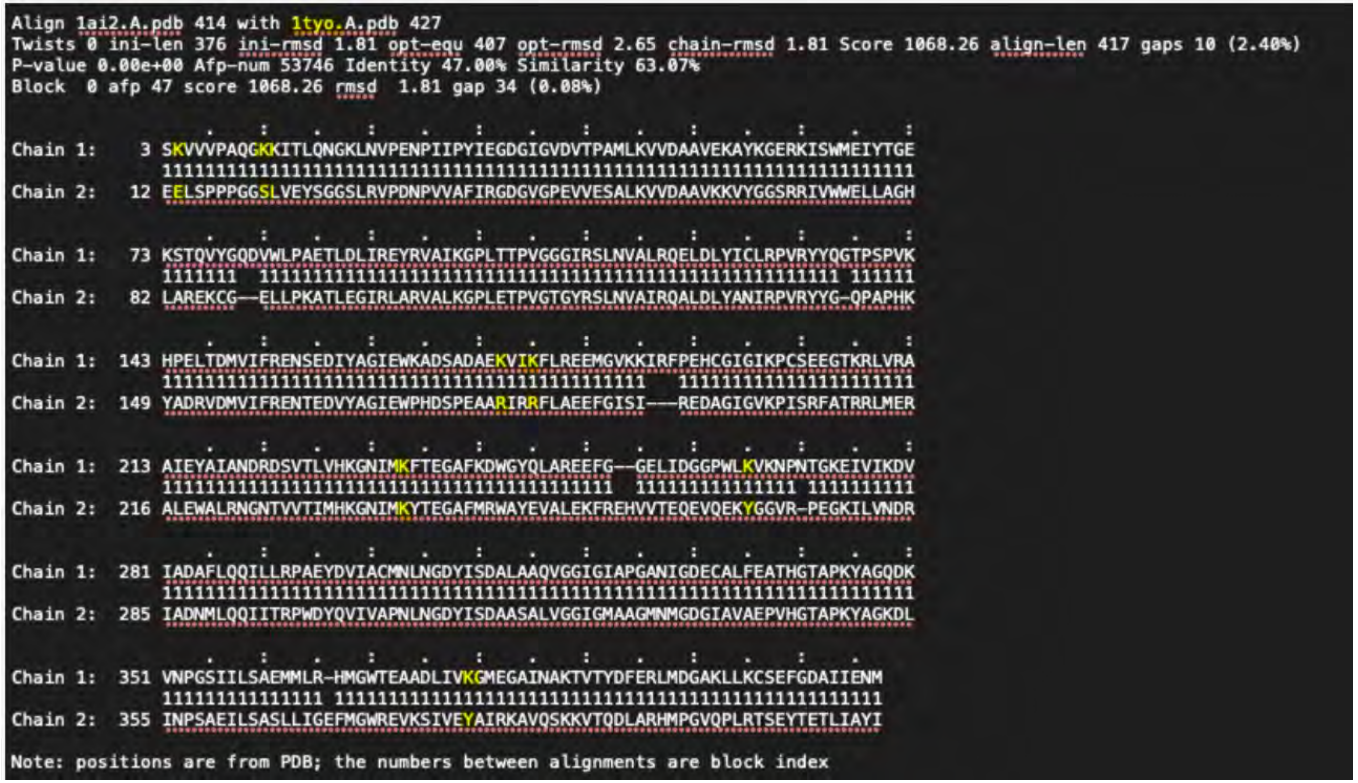

```
Align 1ai2.A.pdb 414 with 1tyo.A.pdb 427
Twists 0 ini-len 376 ini-rmsd 1.81 opt-equ 407 opt-rmsd 2.65 chain-rmsd 1.81 Score 1068.26 align-len 417 gaps 10 (2.40%)
P-value 0.00e+00 Afp-num 53746 Identity 47.00% Similarity 63.07%
Block  0 afp 47 score 1068.26 rmsd  1.81 gap 34 (0.08%)

Chain 1:    3 SKVVVPAQGKKITLQNGKLNVPENPIIPYIEGDGIGVDVTPAMLKVVDAAVEKAYKGERKISWMEIYTGE
              11111111111111111111111111111111111111111111111111111111111111111111111
Chain 2:   12 EELSPPPGGSLVEYSGGSLRVPDNPVVAFIRGDGVGPEVVESALKVVDAAVKKVYGGSRRIVWWELLAGH

Chain 1:   73 KSTQVYGQDVWLPAETLDLIREYRVAIKGPLTTPVGGGIRSLNVALRQELDLYICLRPVRYYQGTPSPVK
              1111111  11111111111111111111111111111111111111111111111111111 111111
Chain 2:   82 LAREKCG--ELLPKATLEGIRLARVALKGPLETPVGTGYRSLNVAIRQALDLYANIRPVRYYG-QPAPHK

Chain 1:  143 HPELTDMVIFRENSEDIYAGIEWKADSADAEKVIKFLREEMGVKKIRFPEHCGIGIKPCSEEGTKRLVRA
              11111111111111111111111111111111111111111111111   11111111111111111111111
Chain 2:  149 YADRVDMVIFRENTEDVYAGIEWPHDSPEAARIRRFLAEEFGISI---REDAGIGVKPISRFATRRLMER

Chain 1:  213 AIEYAIANDRDSVTLVHKGNIMKFTEGAFKDWGYQLAREEFG--GELIDGGPWLKVKNPNTGKEIVIKDV
              111111111111111111111111111111111111111111  111111111111111 1111111111
Chain 2:  216 ALEWALRNGNTVVTIMHKGNIMKYTEGAFMRWAYEVALEKFREHVVTEQEVQEKYGGVR-PEGKILVNDR

Chain 1:  281 IADAFLQQILLRPAEYDVIACMNLNGDYISDALAAQVGGIGIAPGANIGDECALFEATHGTAPKYAGQDK
              1111111111111111111111111111111111111111111111111111111111111111111111
Chain 2:  285 IADNMLQQIITRPWDYQVIVAPNLNGDYISDAASALVGGIGMAAGMNMGDGIAVAEPVHGTAPKYAGKDL

Chain 1:  351 VNPGSIILSAEMMLR-HMGWTEAADLIVKGMEGAINAKTVTYDFERLMDGAKLLKCSEFGDAIIENM
              111111111111111 111111111111111111111111111111111111111111111111111
Chain 2:  355 INPSAEILSASLLIGEFMGWREVKSIVEYAIRKAVQSKKVTQDLARHMPGVQPLRTSEYTETLIAYI

Note: positions are from PDB; the numbers between alignments are block index
```

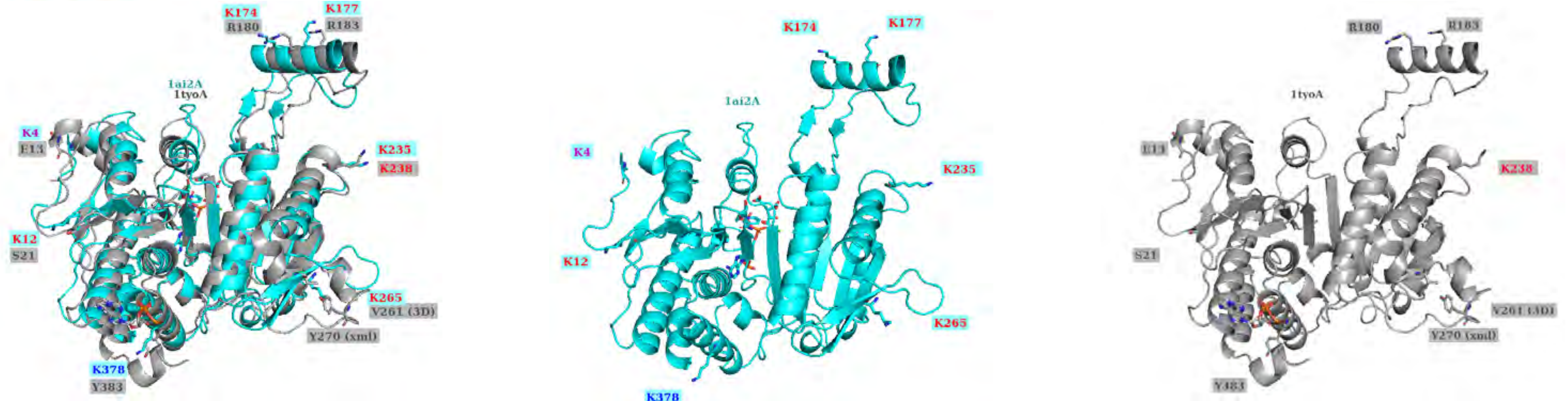

UniProt ID: Q9YE81
PDB ID: 1V94_B

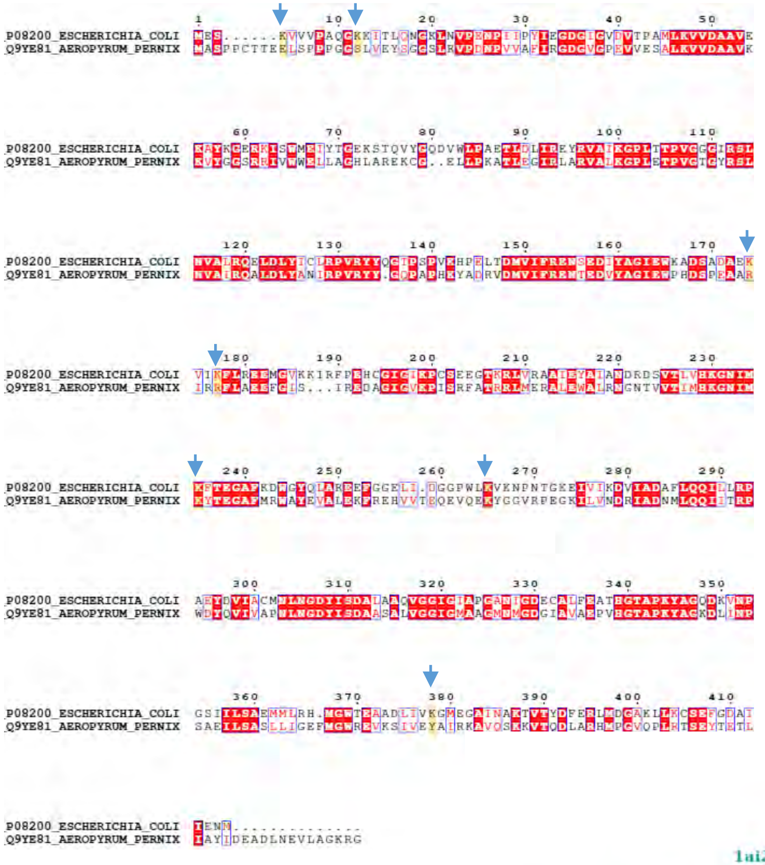

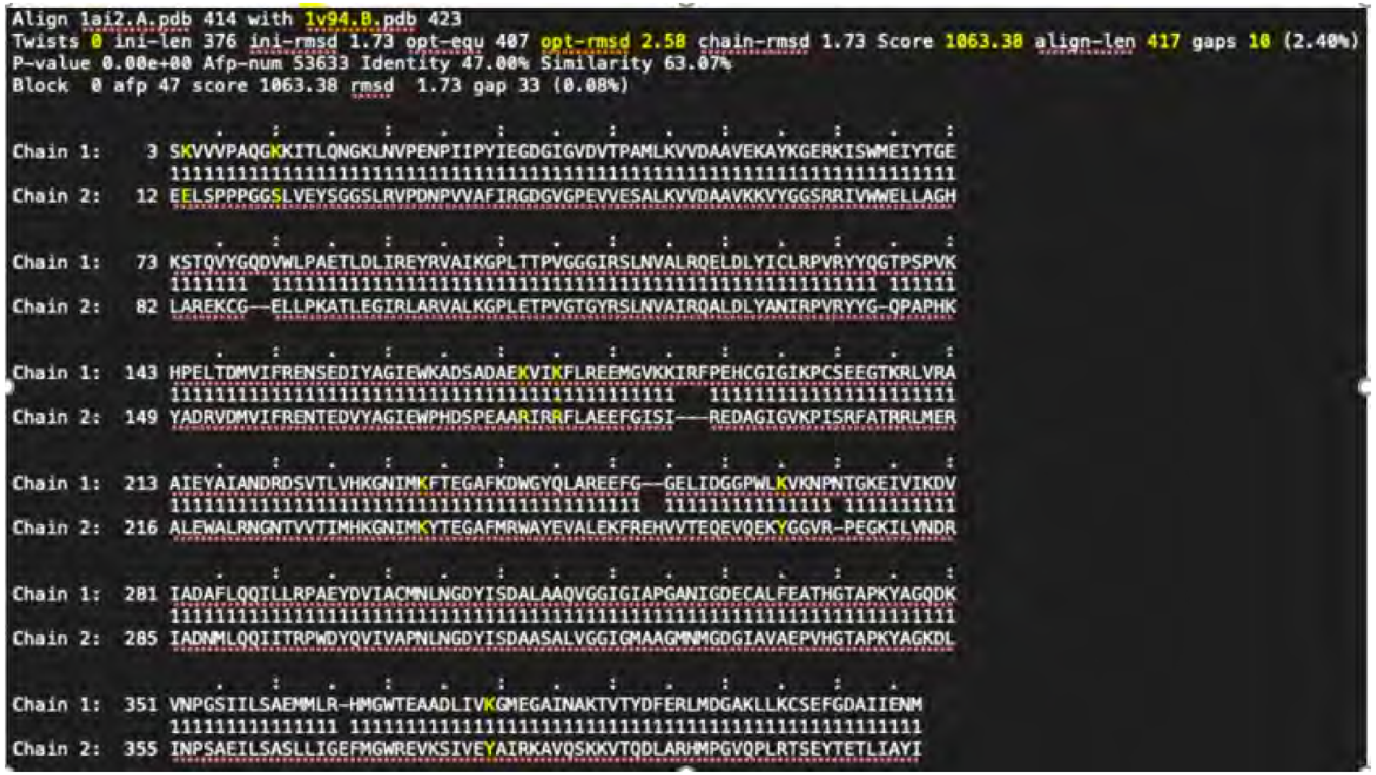

```
Align 1ai2.A.pdb 414 with 1v94.B.pdb 423
Twists 0 ini-len 376 ini-rmsd 1.73 opt-equ 407 opt-rmsd 2.58 chain-rmsd 1.73 Score 1063.38 align-len 417 gaps 10 (2.40%)
P-value 0.00e+00 Afp-num 53633 Identity 47.00% Similarity 63.07%
Block  0 afp 47 score 1063.38 rmsd  1.73 gap 33 (0.08%)

Chain 1:    3 SKVVVPAQGKKITLQNGKLNVPENPIIPYIEGDGIGVDVTPAMLKVVDAAVEKAYKGERKISWMEIYTGE
Chain 2:   12 EELSPPPGGSLVEYSGGSLRVPDNPVVAFIRGDGVGPEVVESALKVVDAAVKKVYGGSRRIVWWELLAGH

Chain 1:   73 KSTQVYGQDVWLPAETLDLIREYRVAIKGPLTTPVGGGIRSLNVALRQELDLYICLRPVRYYQGTPSPVK
Chain 2:   82 LAREKCG--ELLPKATLEGIRLARVALKGPLETPVGTGYRSLNVAIRQALDLYANIRPVRYYG-QPAPHK

Chain 1:  143 HPELTDMVIFRENSEDIYAGIEWKADSADAEKVIKFLREEMGVKKIRFPEHCGIGIKPCSEEGTKRLVRA
Chain 2:  149 YADRVDMVIFRENTEDVYAGIEWPHDSPEAARIRRFLAEEFGISI---REDAGIGVKPISRFATRRLMER

Chain 1:  213 AIEYAIANDRDSVTLVHKGNIMKFTEGAFKDWGYQLAREEFG--GELIDGGPWLKVKNPNTGKEIVIKDV
Chain 2:  216 ALEWALRNGNTVVTIMHKGNIMKYTEGAFMRWAYEVALEKFREHVVTEQEVQEKYGGVR-PEGKILVNDR

Chain 1:  281 IADAFLQQILLRPAEYDVIACMNLNGDYISDALAAQVGGIGIAPGANIGDECALFEATHGTAPKYAGQDK
Chain 2:  285 IADNMLQQIITRPWDYQVIVAPNLNGDYISDAASALVGGIGMAAGMNMGDGIAVAEPVHGTAPKYAGKDL

Chain 1:  351 VNPGSIILSAEMMLR-HMGWTEAADLIVKGMEGAINAKTVTYDFERLMDGAKLLKCSEFGDAIIENM
Chain 2:  355 INPSAEILSASLLIGEFMGWREVKSIVEYAIRKAVQSKKVTQDLARHMPGVQPLRTSEYTETLIAYI
```

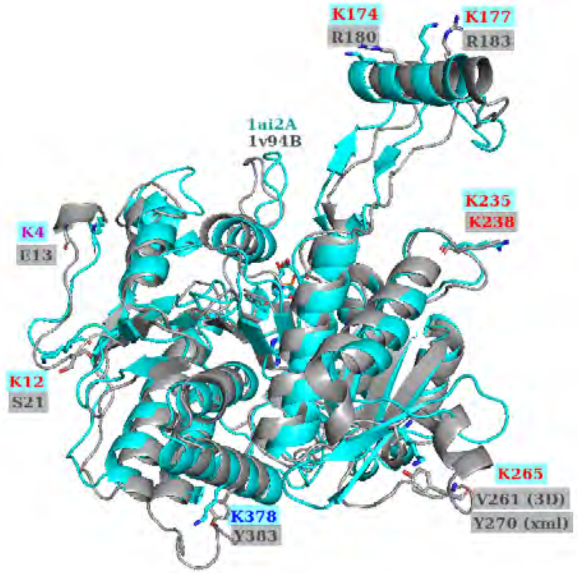

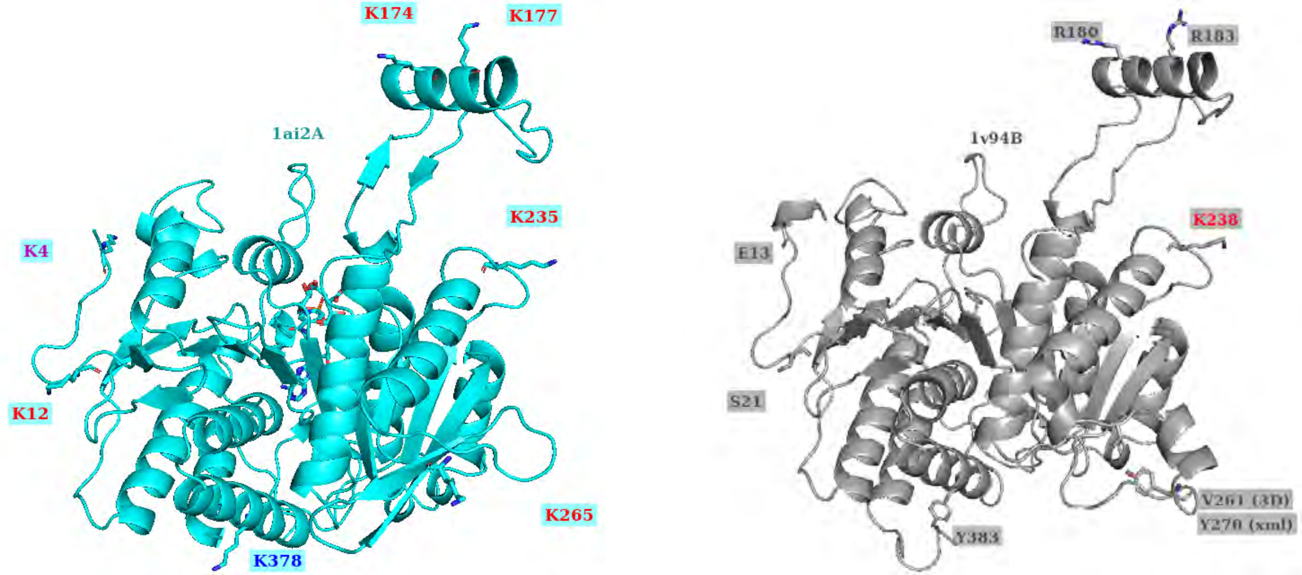

UniProt ID: Q9YE81

PDB ID: 1XGV_A

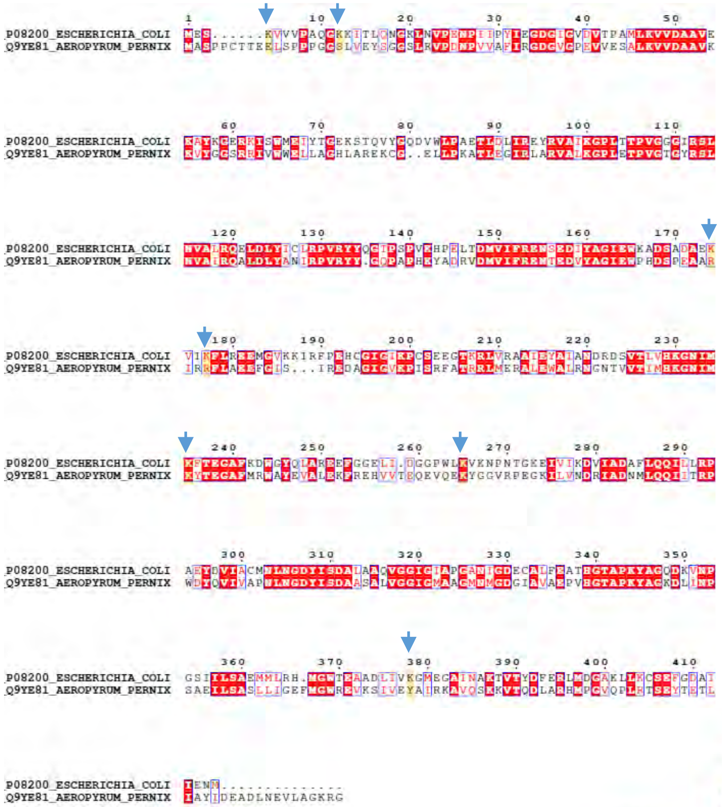

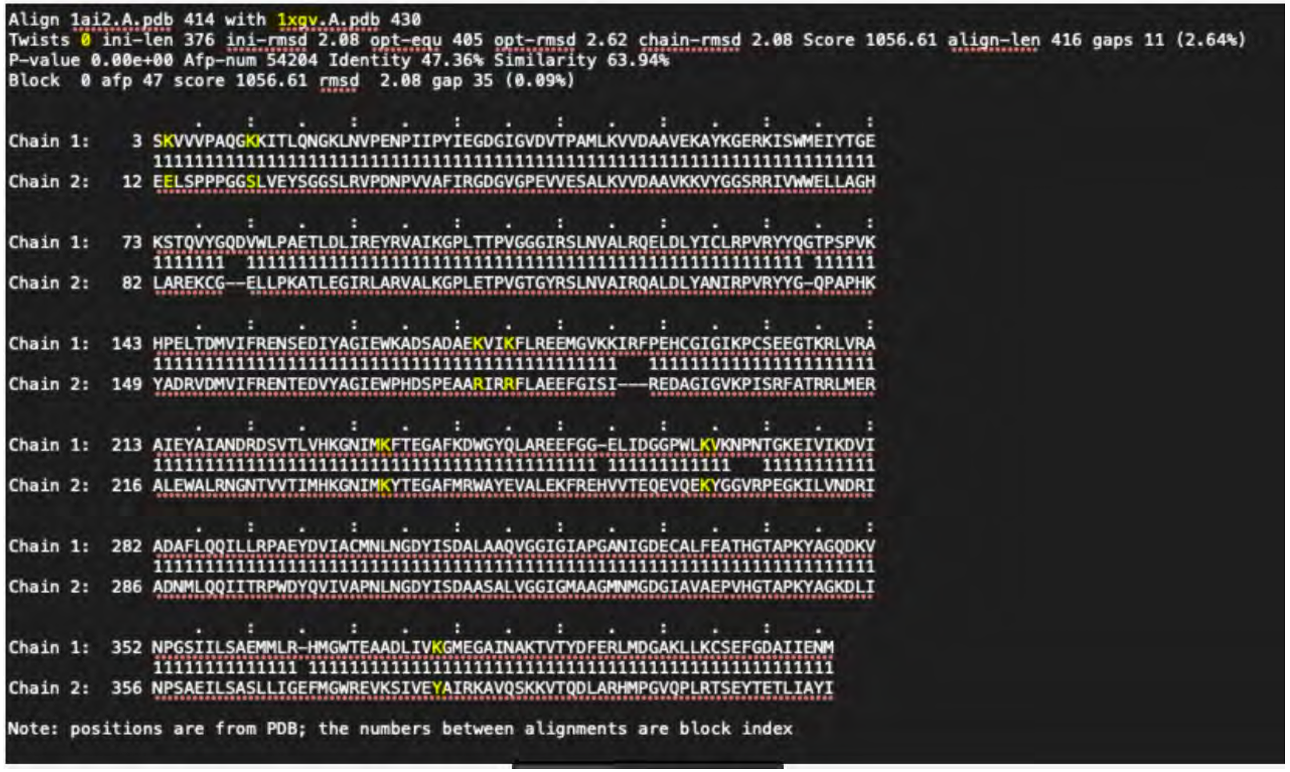

```
Align 1ai2.A.pdb 414 with 1xgv.A.pdb 430
Twists 0 ini-len 376 ini-rmsd 2.08 opt-equ 405 opt-rmsd 2.62 chain-rmsd 2.08 Score 1056.61 align-len 416 gaps 11 (2.64%)
P-value 0.00e+00 Afp-num 54204 Identity 47.36% Similarity 63.94%
Block  0 afp 47 score 1056.61 rmsd  2.08 gap 35 (0.09%)

Chain 1:    3 SKVVVPAQGKKITLQNGKLNVPENPIIPYIEGDGIGVDVTPAMLKVVDAAVEKAYKGERKISWMEIYTGE
              1111111111111111111111111111111111111111111111111111111111111111111111
Chain 2:   12 EELSPPPGGSLVEYSGGSLRVPDNPVVAFIRGDGVGPEVVESALKVVDAAVKKVYGGSRRIVWWELLAGH

Chain 1:   73 KSTQVYGQDVWLPAETLDLIREYRVAIKGPLTTPVGGIRSLNVALRQELDLYICLRPVRYYQGTPSPVK
              1111111  111111111111111111111111111111111111111111111111111111 111111
Chain 2:   82 LAREKCG--ELLPKATLEGIRLARVALKGPLETPVGTGYRSLNVAIRQALDLYANIRPVRYYG-QPAPHK

Chain 1:  143 HPELTDMVIFRENSEDIYAGIEWKADSADAEKVIKFLREEMGVKKIRFPEHCGIGIKPCSEEGTKRLVRA
              111111111111111111111111111111111111111111111   1111111111111111111111
Chain 2:  149 YADRVDMVIFRENTEDVYAGIEWPHDSPEAARIRRFLAEEFGISI---REDAGIGVKPISRFATRRLMER

Chain 1:  213 AIEYAIANDRDSVTLVHKGNIMKFTEGAFKDWGYQLAREEFGG-ELIDGGPWLKVKNPNTGKEIVIKDVI
              1111111111111111111111111111111111111111111 111111111111   11111111111
Chain 2:  216 ALEWALRNGNTVVTIMHKGNIMKYTEGAFMRWAYEVALEKFREHVVTEQEVQEKYGGVRPEGKILVNDRI

Chain 1:  282 ADAFLQQILLRPAEYDVIACMNLNGDYISDALAAQVGGIGIAPGANIGDECALFEATHGTAPKYAGQDKV
              1111111111111111111111111111111111111111111111111111111111111111111111
Chain 2:  286 ADNMLQQIITRPWDYQVIVAPNLNGDYISDAASALVGGIGMAAGMNMGDGIAVAEPVHGTAPKYAGKDLI

Chain 1:  352 NPGSIILSAEMMLR-HMGWTEAADLIVKGMEGAINAKTVTYDFERLMDGAKLLKCSEFGDAIIENM
              11111111111111 111111111111111111111111111111111111111111111111111
Chain 2:  356 NPSAEILSASLLIGEFMGWREVKSIVEYAIRKAVQSKKVTQDLARHMPGVQPLRTSEYTETLIAYI

Note: positions are from PDB; the numbers between alignments are block index
```

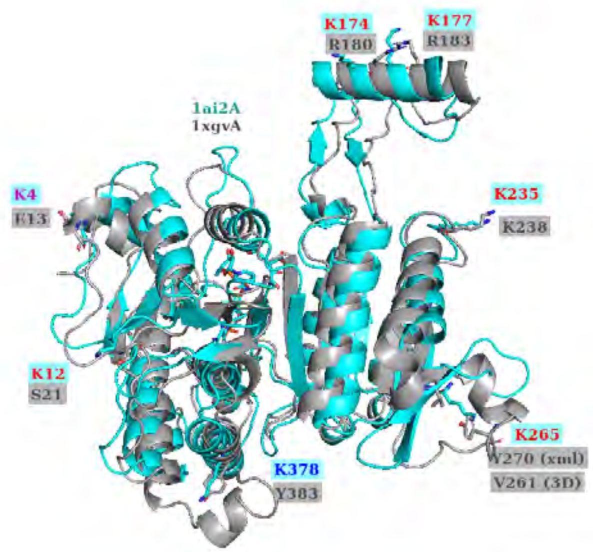

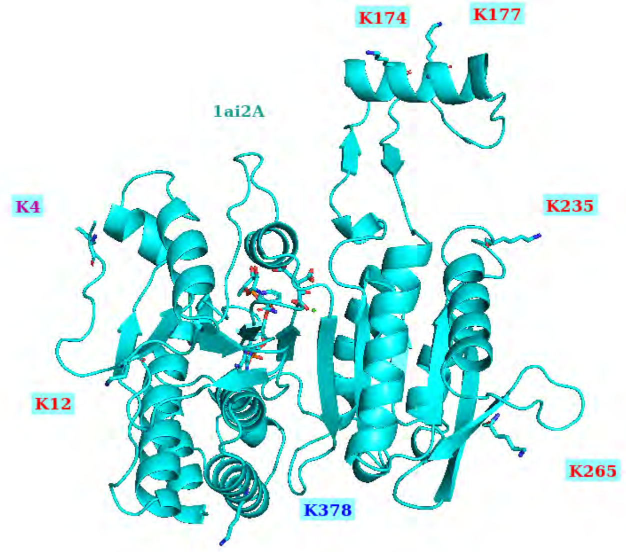

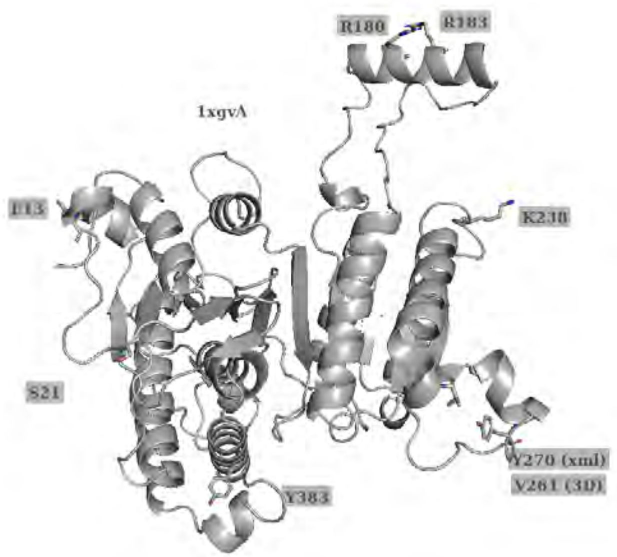

UniProt ID: Q9YE81

PDB ID: 1XKD_B

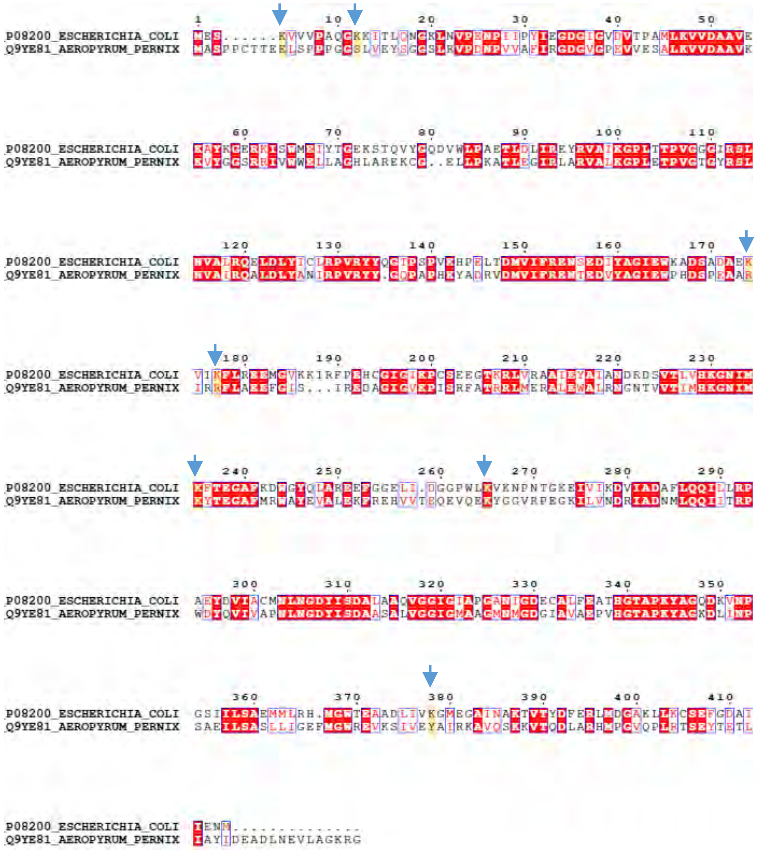

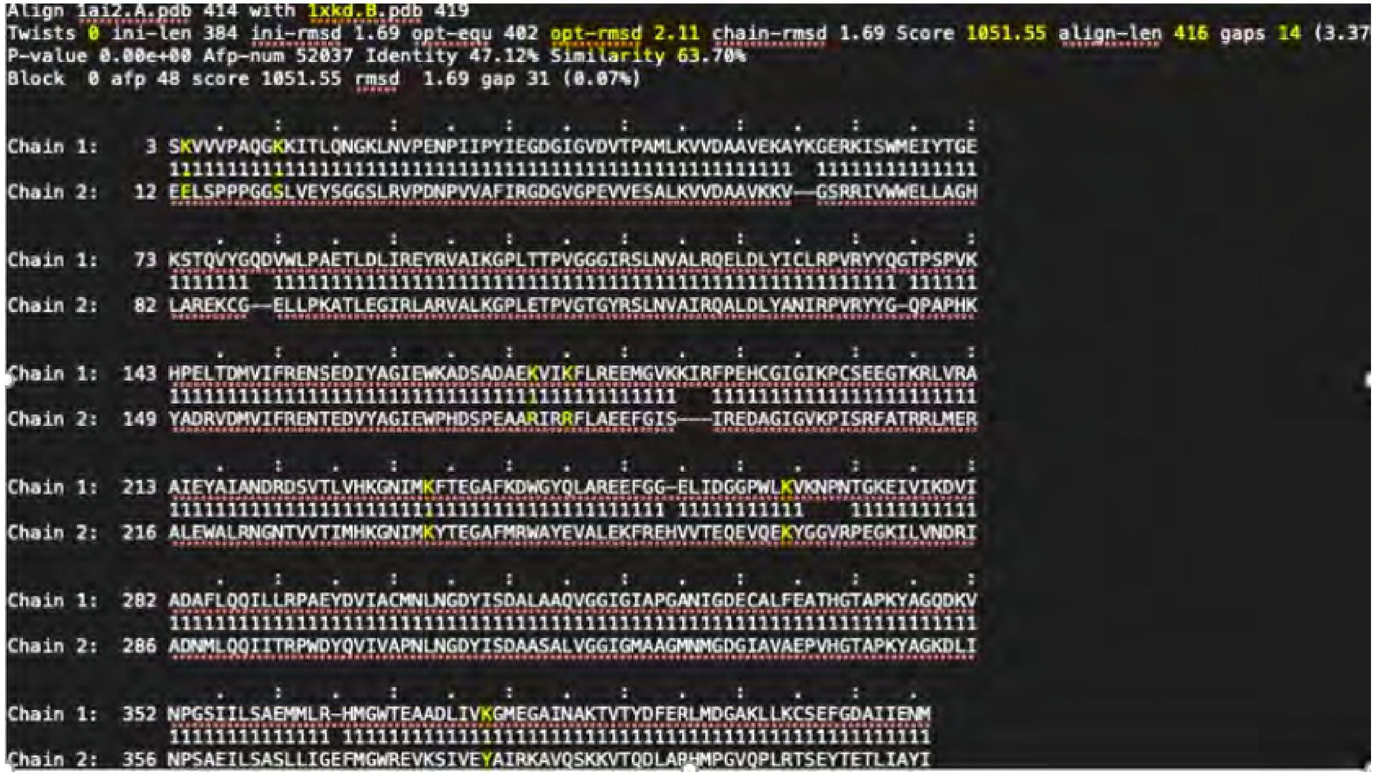

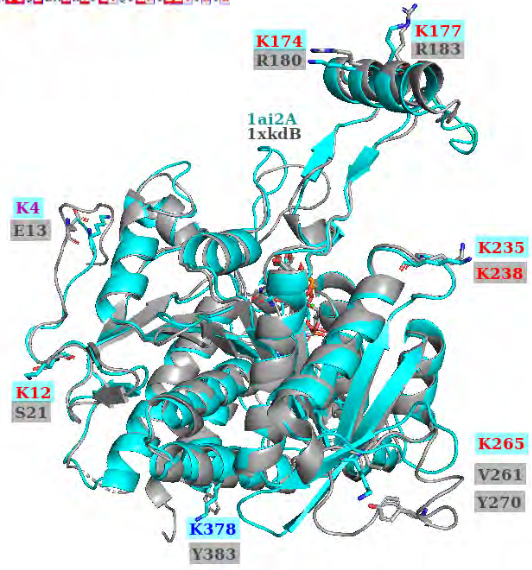

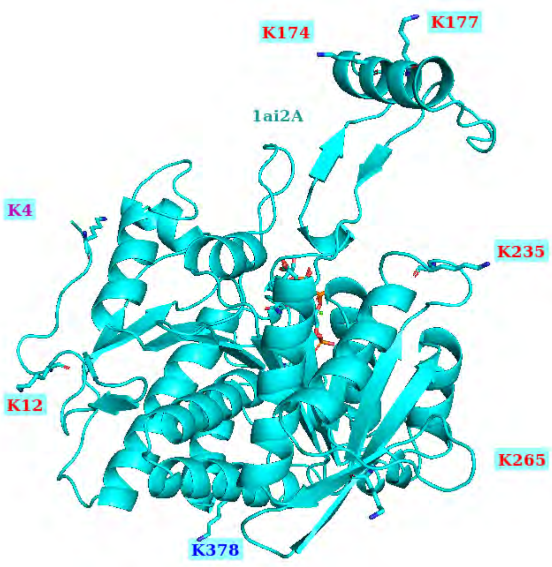

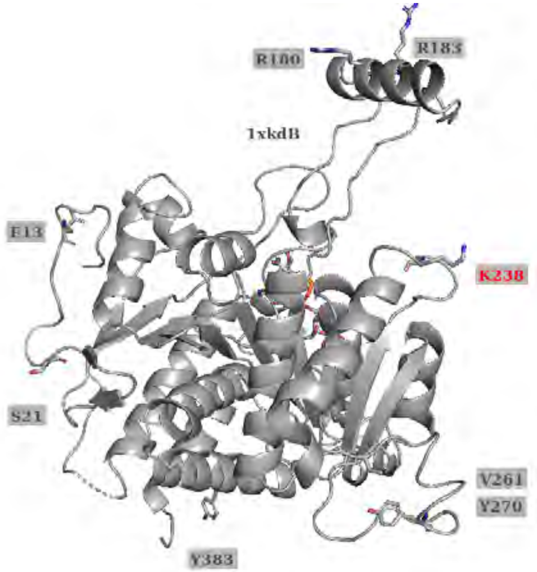

Supplement: Supplementary file 3 [file Data_Sheet_3.PDF]
