# Supplementary material for: Investigation of the Importance of Protein 3D Structure for Assessing Conservation of Lysine Acetylation Sites in Protein Homologs
Source: Front Microbiol. 2022 Jan 31;12:805181. doi: 10.3389/fmicb.2021.805181 (PMC8843374; doi:10.3389/fmicb.2021.805181)

Supplemental Figure SF2C. Compiled pairwise sequence (Cobalt) and structural (FATCAT) alignments between the *E. coli* substrate protein target (KatE-catalase HPII; PDB ID: 1cf9) and homologs sorted by UniProt ID. Lysine residues previously identified as acetylated in the target protein are highlighted in yellow in the sequence alignments and FATCAT structural alignment xml files to examine conservation. 3D protein structures are shown as ribbon representations with the target protein in cyan and the homolog protein in gray. Blue lysine residues correspond to KAT (lysine acetyltransferase) acetylation sites, red lysine residues correspond to AcP (acetyl phosphate) acetylation sites, and purple lysine residues correspond to sites acetylated by both KAT and AcP. 1D sequence alignments are not shown between *E. coli* proteins because sequences were identical.

UniProt ID: A0A031LXI5

PDB ID: 6PT7_A

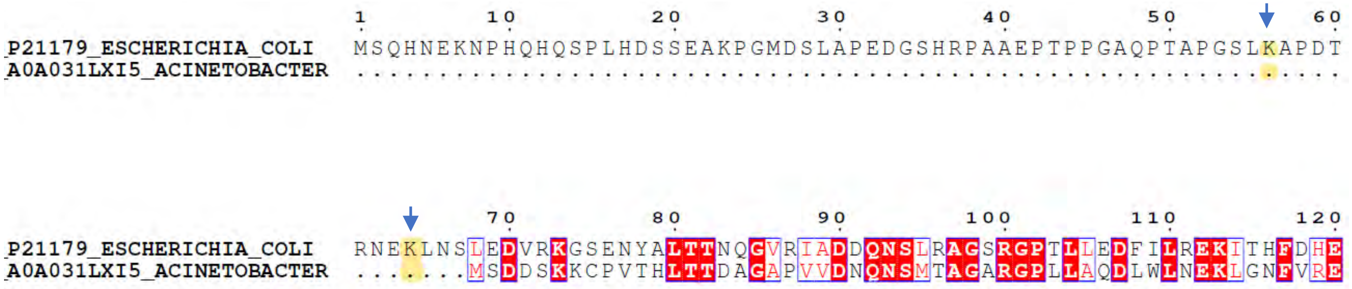

Full sequences in supplemental file.

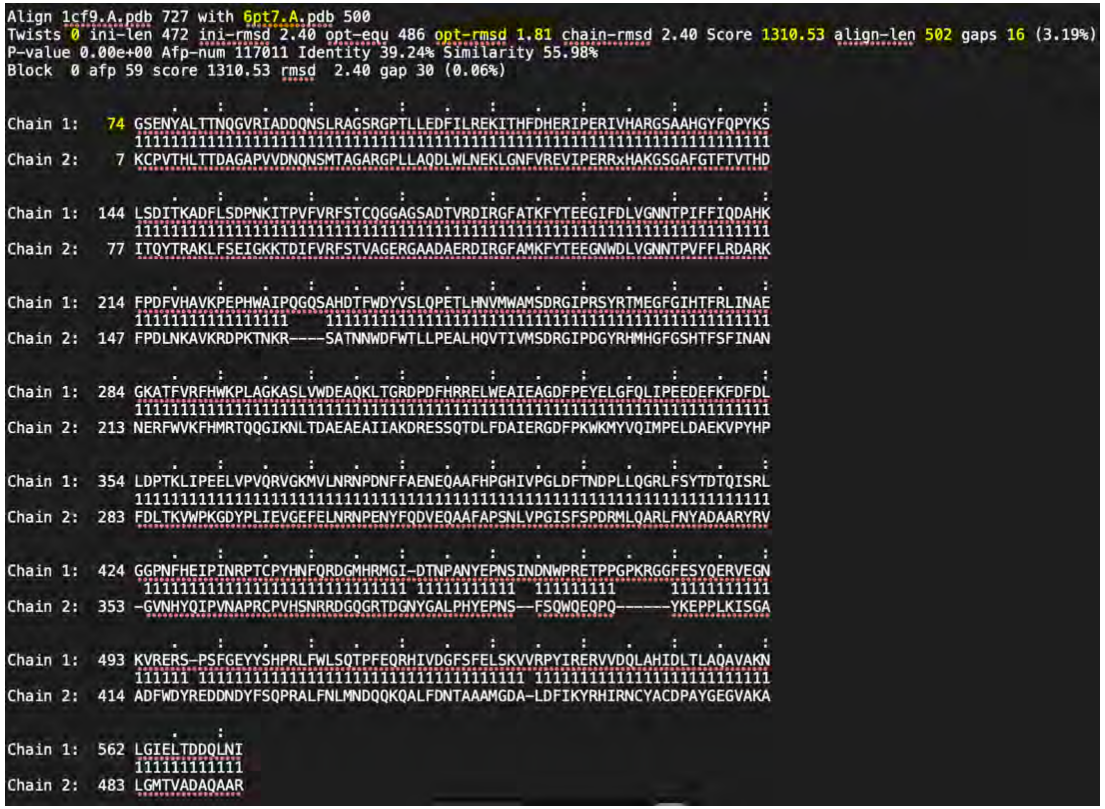

```
Align 1cf9.A.pdb 727 with 6pt7.A.pdb 500
Twists 0 ini-len 472 ini-rmsd 2.40 opt-equ 486 opt-rmsd 1.81 chain-rmsd 2.40 Score 1310.53 align-len 502 gaps 16 (3.19%)
P-value 0.00e+00 Afp-num 117011 Identity 39.24% Similarity 55.98%
Block  0 afp 59 score 1310.53 rmsd  2.40 gap 30 (0.06%)

                .    :    .    :    .    :    .    :    .    :    .    :    .    :
Chain 1:   74 GSENYALTTNQGVRIADDQNSLRAGSRGPTLLEDFILREKITHFDHERIPERIVHARGSAAHGYFQPYKS
              1111111111111111111111111111111111111111111111111111111111111111111111
Chain 2:    7 KCPVTHLTTDAGAPVVDNQNSMTAGARGPLLAQDLWLNEKLGNFVREVIPERRxHAKGSGAFGTFTVTHD

                .    :    .    :    .    :    .    :    .    :    .    :    .    :
Chain 1:  144 LSDITKADFLSDPNKITPVFVRFSTCQGGAGSADTVRDIRGFATKFYTEEGIFDLVGNNTPIFFIQDAHK
              1111111111111111111111111111111111111111111111111111111111111111111111
Chain 2:   77 ITQYTRAKLFSEIGKKTDIFVRFSTVAGERGAADAERDIRGFAMKFYTEEGNWDLVGNNTPVFFLRDARK

                .    :    .    :    .    :    .    :    .    :    .    :    .    :
Chain 1:  214 FPDFVHAVKPEPHWAIPQGQSAHDTFWDYVSLQPETLHNVMWAMSDRGIPRSYRTMEGFGIHTFRLINAE
              11111111111111111    11111111111111111111111111111111111111111111111111
Chain 2:  147 FPDLNKAVKRDPKTNKR----SATNNWDFWTLLPEALHQVTIVMSDRGIPDGYRHMHGFGSHTFSFINAN

                .    :    .    :    .    :    .    :    .    :    .    :    .    :
Chain 1:  284 GKATFVRFHWKPLAGKASLVWDEAQKLTGRDPDFHRRELWEAIEAGDFPEYELGFQLIPEEDEFKFDFDL
              1111111111111111111111111111111111111111111111111111111111111111111111
Chain 2:  213 NERFWVKFHMRTQQGIKNLTDAEAEAIIAKDRESSQTDLFDAIERGDFPKWKMYVQIMPELDAEKVPYHP

                .    :    .    :    .    :    .    :    .    :    .    :    .    :
Chain 1:  354 LDPTKLIPEELVPVQRVGKMVLNRNPDNFFAENEQAAFHPGHIVPGLDFTNDPLLQGRLFSYTDTQISRL
              1111111111111111111111111111111111111111111111111111111111111111111111
Chain 2:  283 FDLTKVWPKGDYPLIEVGEFELNRNPENYFQDVEQAAFAPSNLVPGISFSPDRMLQARLFNYADAARYRV

                .    :    .    :    .    :    .    :    .    :    .    :    .    :
Chain 1:  424 GGPNFHEIPINRPTCPYHNFQRDGMHRMGI-DTNPANYEPNSINDNWPRETPPGPKRGGFESYQERVEGN
               11111111111111111111111111111 11111111111  111111111      11111111111
Chain 2:  353 -GVNHYQIPVNAPRCPVHSNRRDGQGRTDGNYGALPHYEPNS--FSQWQEQPQ------YKEPPLKISGA

                .    :    .    :    .    :    .    :    .    :    .    :    .    :
Chain 1:  493 KVRERS-PSFGEYYSHPRLFWLSQTPFEQRHIVDGFSFELSKVVRPYIRERVVDQLAHIDLTLAQAVAKN
              111111 11111111111111111111111111111111111111 111111111111111111111111
Chain 2:  414 ADFWDYREDDNDYFSQPRALFNLMNDQQKQALFDNTAAAMGDA-LDFIKYRHIRNCYACDPAYGEGVAKA

                .    :
Chain 1:  562 LGIELTDDQLNI
              111111111111
Chain 2:  483 LGMTVADAQAAR
```

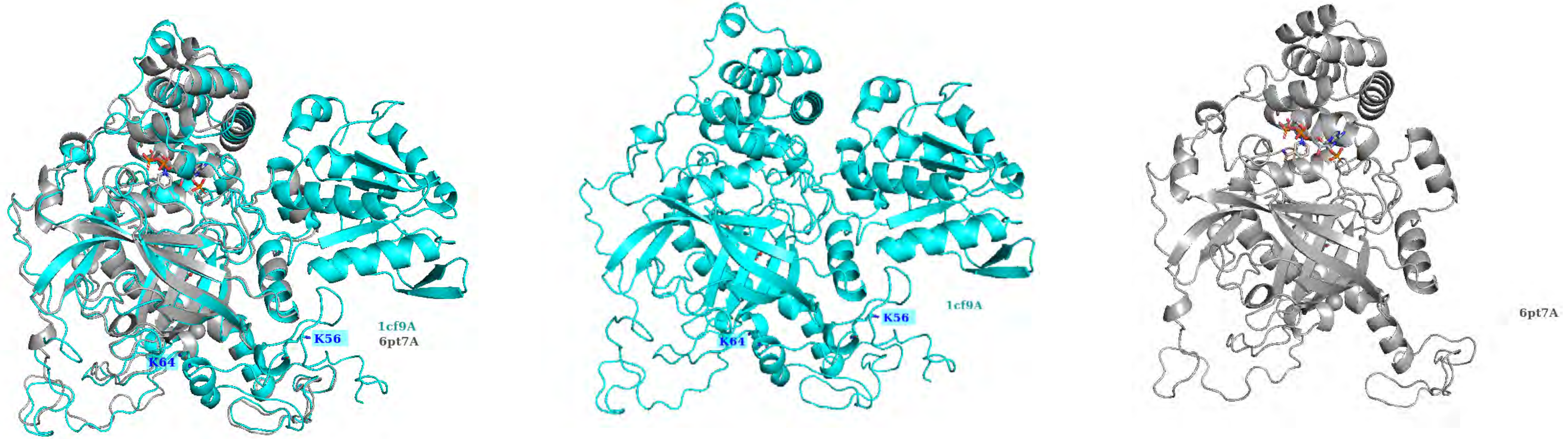

UniProt ID: A0A0U4WRC5

PDB ID: 4B7F_C

```
                                      1         10        20        30        40        50        60
P21179_ESCHERICHIA_COLI               MSQHNEKNPHQHQSPLHDSSEAKPGMDSLAPEDGSHRPAAEPTPPGAQPTAPGSLKAPDT
AOAOU4WRC5_CORYNEBACTERIUM_GLUTAMICUM ............................................................

                                                70        80        90        100       110
P21179_ESCHERICHIA_COLI               RNEKLNSLEDVRKGSE...NYALTTNQGVRIADDQNSLRAGSRGPTLLEDFILREKITHF
AOAOU4WRC5_CORYNEBACTERIUM_GLUTAMICUM MSEKSAADQIVDRGMRPKLSGNTTRHNGAPVPSENISATAGPQGPNVLNDIHLIEKLAHF
```

Full sequences in supplemental file.

```
Align 1cf9.A.pdb 727 with 4b7f.C.pdb 514
Twists 0 ini-len 464 ini-rmsd 1.31 opt-equ 491 opt-rmsd 1.70 chain-rmsd 1.31 Score 1269.34 align-len 520 gaps 29 (5.58%)
P-value 0.00e+00 Afp-num 121478 Identity 33.65% Similarity 50.77%
Block  0 afp 58 score 1269.34 rmsd  1.31 gap 51 (0.10%)

Chain 1:   60 TRNEKLNSLEDVRKGSENYALTTNQGVRIADDQNSLRAGSRGPTLLEDFILREKITHFDHERIPERIVHA
              1111111111111111111111111111111111111111111111111111111111111111111111
Chain 2:    3 EKSAADQIVDRGMRPKLSGNTTRHNGAPVPSENISATAGPQGPNVLNDIHLIEKLAHFNRENVPERIPHA

Chain 1:  130 RGSAAHGYFQPYKSLSDITKADFLSDPNKITPVFVRFSTCQGGAGSADTVRDIRGFATKFYTEEGIFDLV
              111111111111111111111111111 111111111111111111111111111111111111111111
Chain 2:   73 KGHGAFGELHITEDVSEYTKADLFQPG-KVTPLAVRFSTVAGEQGSPDTWRDVHGFALRFYTEEGNYDIV

Chain 1:  200 GNNTPIFFIQDAHKFPDFVHAVKPEPHWAIPQGQSAHDTFWDYVSLQPETLHNVMWAMSDRGIPRSYRTM
              111111111111111111111111111111111    11111111111111111111111111111111
Chain 2:  142 GNNTPTFFLRDGMKFPDFIHSQKRLNKNGLR----DADMQWDFWTRAPESAHQVTYLMGDRGTPKTSRHQ

Chain 1:  270 EGFGIHTFRLINAEGKATFVRFHWKPLAGKASLVWDEAQKLTGRDPDFHRRELWEAIEAGDFPEYELGFQ
              1111111111111111111111111111111111111111111111111111111111111111111111
Chain 2:  208 DGFGSHTFQWINAEGKPVWVKYHFKTRQGWDCFTDAEAAKVAGENADYQREDLYNAIENGDFPIWDVKVQ

Chain 1:  340 LIPEEDEFKFDFDLLDPTKLIPEELVPVQRVGKMVLNRNPDNFFAENEQAAFHPGHIVPGLDFTNDPLLQ
              1111111111111111111111111111111111111111111111111111111111111111111111
Chain 2:  278 IMPFEDAENYRWNPFDLTKTWSQKDYPLIPVGYFILNRNPRNFFAQIEQIALDPGNIVPGVGLSPDRMLQ

Chain 1:  410 GRLFSYTDTQISRLGGPNFHEIPINRPTCPYHNFQRDGMHRMGID-TNPANYEPNSINDNWPRETPPGPK
              111111111111111 1111111111111111111111111111 111111111  111111111
Chain 2:  348 ARIFAYADQQRYRIG-ANYRDLPVNRPINEVNTYSREGSMQYIFDAEGEPSYSPNR--YDKGAGYLDNGT

Chain 1:  479 RG---------GFESYQERV--EGNKVRERSPSF--GEYYSHPRLFWL-SQTPFEQRHIVDGFSFELSKV
                         111111111  111111111111  111111111111 11111111111111111111
Chain 2:  415 DSSSNHTSYGQADDIYVNPDPHGTDLVRAAYVKHQDDDDFIQPGILYREVLDEGEKERLADNISNAMQGI

Chain 1:  535 VRPYIRERVVDQLAHIDLTLAQAVAKNLGI
               11111111111111111111111111111
Chain 2:  485 -SEATEPRVYDYWNNVDENLGARVKELYLQ
```

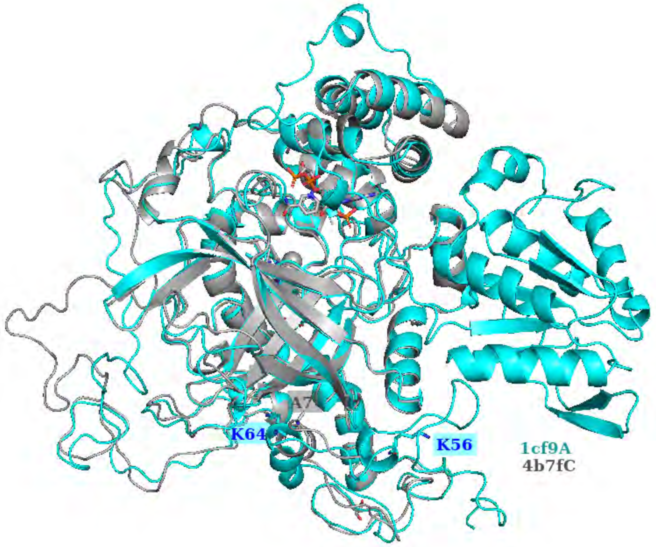

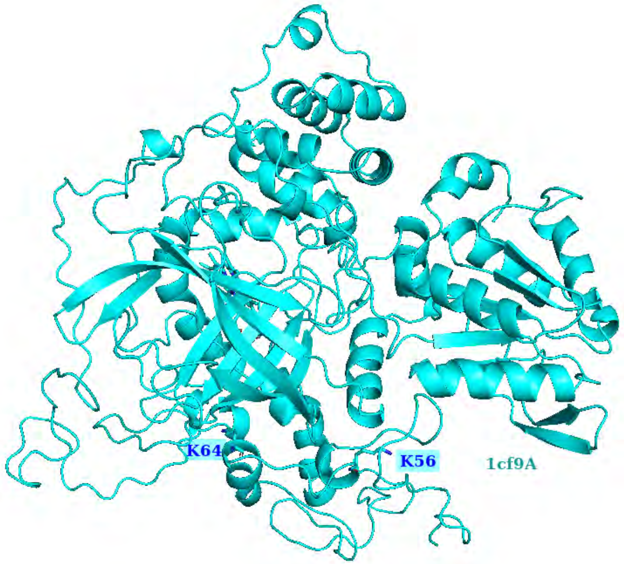

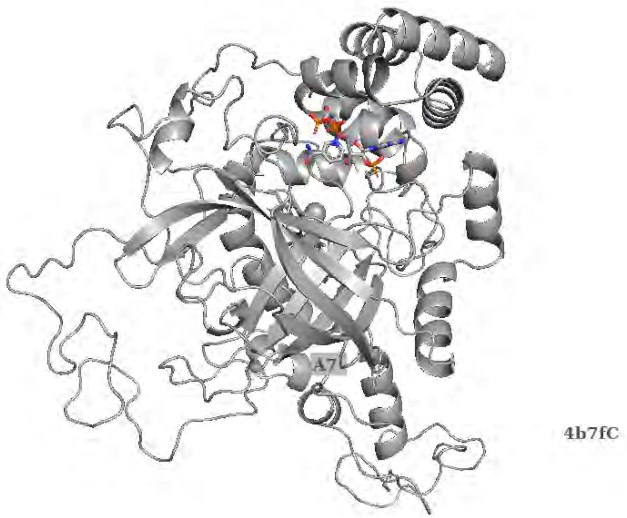

UniProt ID: A0A0U4WRC5

PDB ID: 4B7G_C

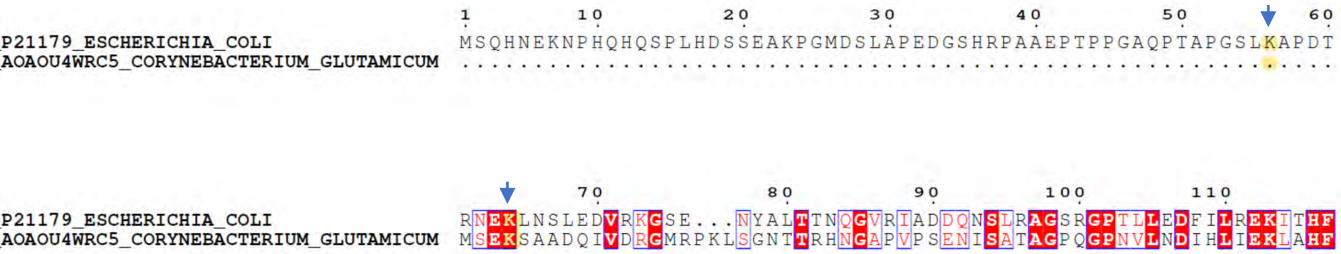

Full sequences in supplemental file.

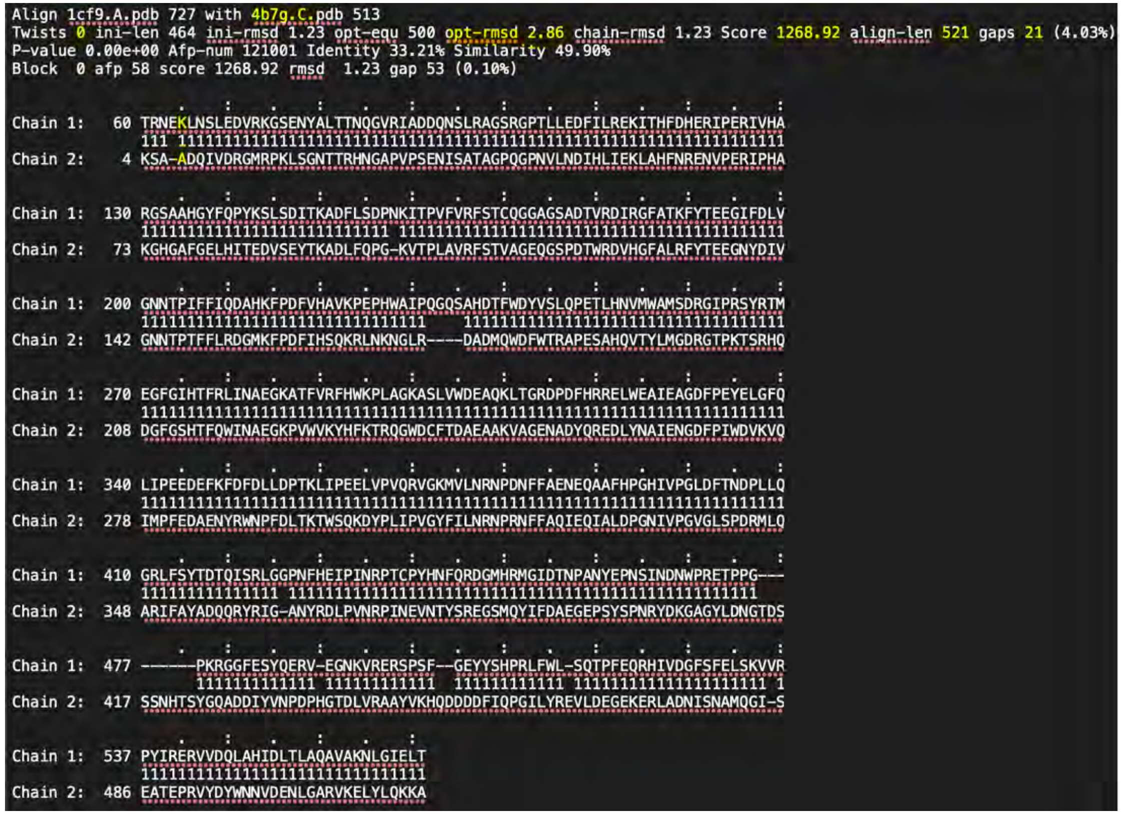

```
Align 1cf9.A.pdb 727 with 4b7g.C.pdb 513
Twists 0 ini-len 464 ini-rmsd 1.23 opt-equ 500 opt-rmsd 2.86 chain-rmsd 1.23 Score 1268.92 align-len 521 gaps 21 (4.03%)
P-value 0.00e+00 Afp-num 121001 Identity 33.21% Similarity 49.90%
Block  0 afp 58 score 1268.92 rmsd  1.23 gap 53 (0.10%)

                  .    :    .    :    .    :    .    :    .    :    .    :    .    :
Chain 1:   60 TRNEKLNSLEDVRKGSENYALTTNQGVRIADDQNSLRAGSRGPTLLEDFILREKITHFDHERIPERIVHA
              111 1111111111111111111111111111111111111111111111111111111111111111111
Chain 2:    4 KSA-ADQIVDRGMRPKLSGNTTRHNGAPVPSENISATAGPQGPNVLNDIHLIEKLAHFNRENVPERIPHA

                  .    :    .    :    .    :    .    :    .    :    .    :    .    :
Chain 1:  130 RGSAAHGYFQPYKSLSDITKADFLSDPNKITPVFVRFSTCQGGAGSADTVRDIRGFATKFYTEEGIFDLV
              111111111111111111111111111 111111111111111111111111111111111111111111
Chain 2:   73 KGHGAFGELHITEDVSEYTKADLFQPG-KVTPLAVRFSTVAGEQGSPDTWRDVHGFALRFYTEEGNYDIV

                  .    :    .    :    .    :    .    :    .    :    .    :    .    :
Chain 1:  200 GNNTPIFFIQDAHKFPDFVHAVKPEPHWAIPQGQSAHDTFWDYVSLQPETLHNVMWAMSDRGIPRSYRTM
              1111111111111111111111111111111    11111111111111111111111111111111111
Chain 2:  142 GNNTPTFFLRDGMKFPDFIHSQKRLNKNGLR----DADMQWDFWTRAPESAHQVTYLMGDRGTPKTSRHQ

                  .    :    .    :    .    :    .    :    .    :    .    :    .    :
Chain 1:  270 EGFGIHTFRLINAEGKATFVRFHWKPLAGKASLVWDEAQKLTGRDPDFHRRELWEAIEAGDFPEYELGFQ
              1111111111111111111111111111111111111111111111111111111111111111111111
Chain 2:  208 DGFGSHTFQWINAEGKPVWVKYHFKTRQGWDCFTDAEAAKVAGENADYQREDLYNAIENGDFPIWDVKVQ

                  .    :    .    :    .    :    .    :    .    :    .    :    .    :
Chain 1:  340 LIPEEDEFKFDFDLLDPTKLIPEELVPVQRVGKMVLNRNPDNFFAENEQAAFHPGHIVPGLDFTNDPLLQ
              1111111111111111111111111111111111111111111111111111111111111111111111
Chain 2:  278 IMPFEDAENYRWNPFDLTKTWSQKDYPLIPVGYFILNRNPRNFFAQIEQIALDPGNIVPGVGLSPDRMLQ

                  .    :    .    :    .    :    .    :    .    :    .    :    .    :
Chain 1:  410 GRLFSYTDTQISRLGGPNFHEIPINRPTCPYHNFQRDGMHRMGIDTNPANYEPNSINDNWPRETPPG---
              111111111111111 1111111111111111111111111111111111111111111111111111
Chain 2:  348 ARIFAYADQQRYRIG-ANYRDLPVNRPINEVNTYSREGSMQYIFDAEGEPSYSPNRYDKGAGYLDNGTDS

                  .    :    .    :    .    :    .    :    .    :    .    :    .    :
Chain 1:  477 ------PKRGGFESYQERV-EGNKVRERSPSF--GEYYSHPRLFWL-SQTPFEQRHIVDGFSFELSKVVR
                    1111111111111 111111111111  111111111111 111111111111111111111 1
Chain 2:  417 SSNHTSYGQADDIYVNPDPHGTDLVRAAYVKHQDDDDFIQPGILYREVLDEGEKERLADNISNAMQGI-S

                  .    :    .    :    .    :
Chain 1:  537 PYIRERVVDQLAHIDLTLAQAVAKNLGIELT
              1111111111111111111111111111111
Chain 2:  486 EATEPRVYDYWNNVDENLGARVKELYLQKKA
```

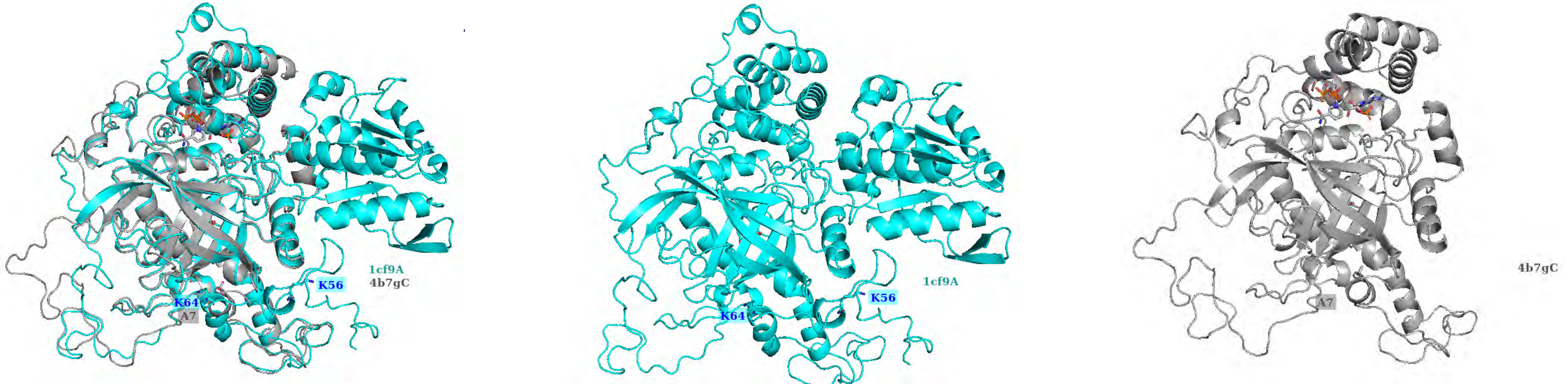

UniProt ID: A0A0U4WRC5

PDB ID: 4B7H_C

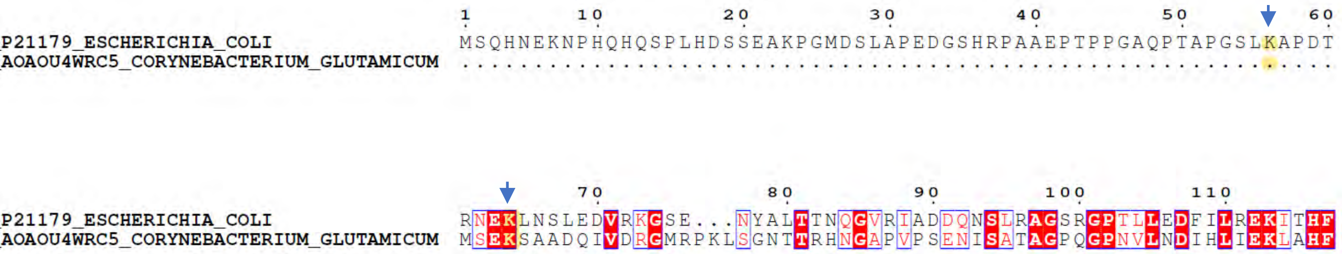

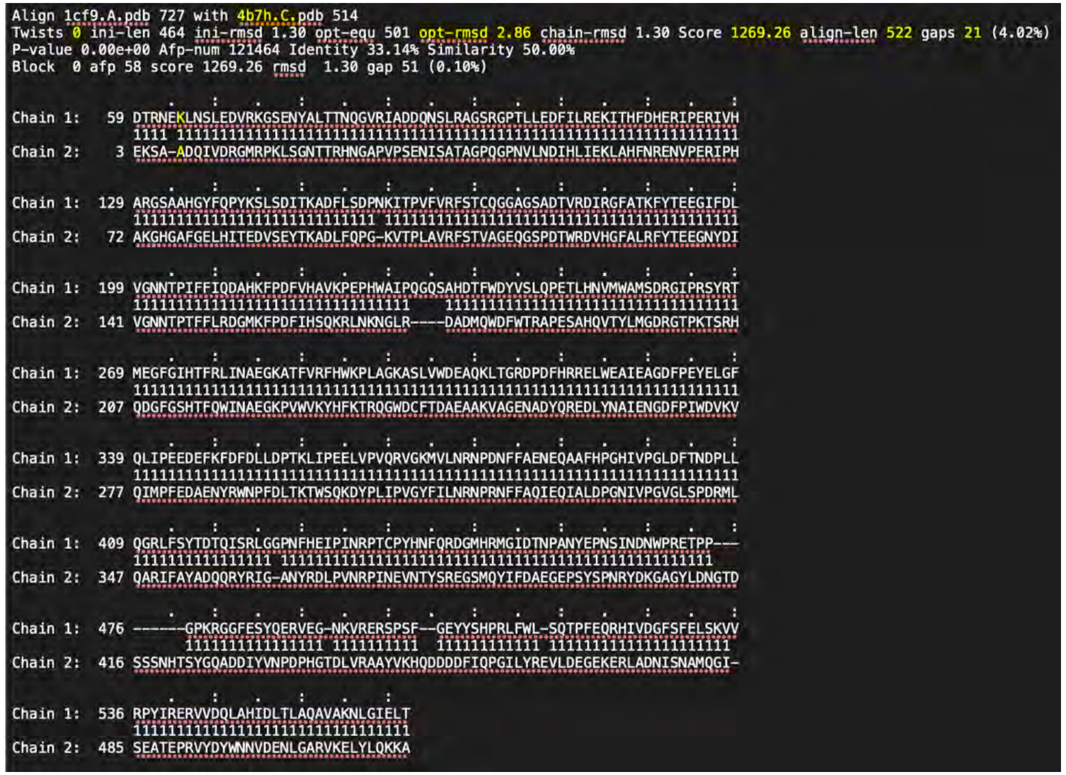

Full sequences in supplemental file.

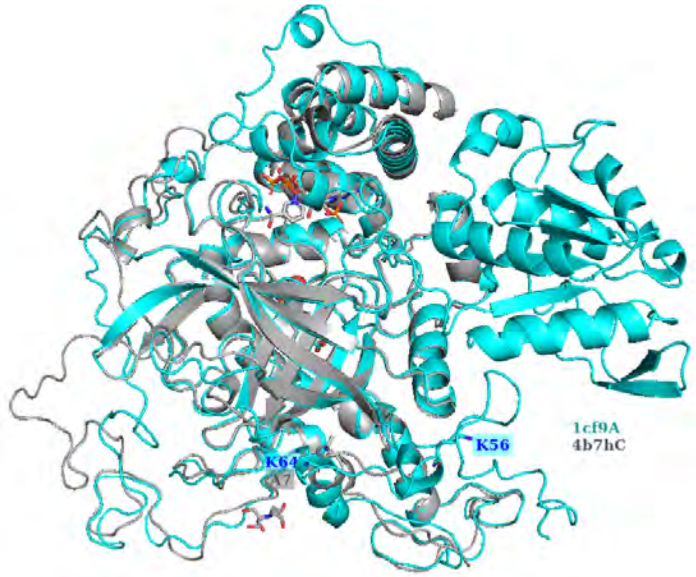

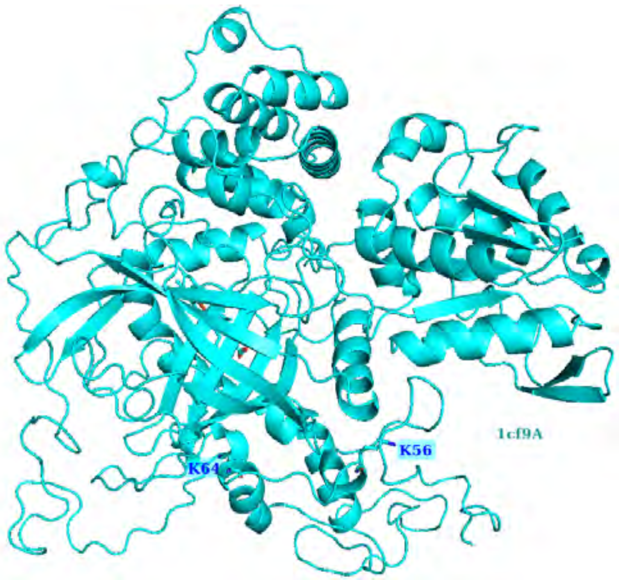

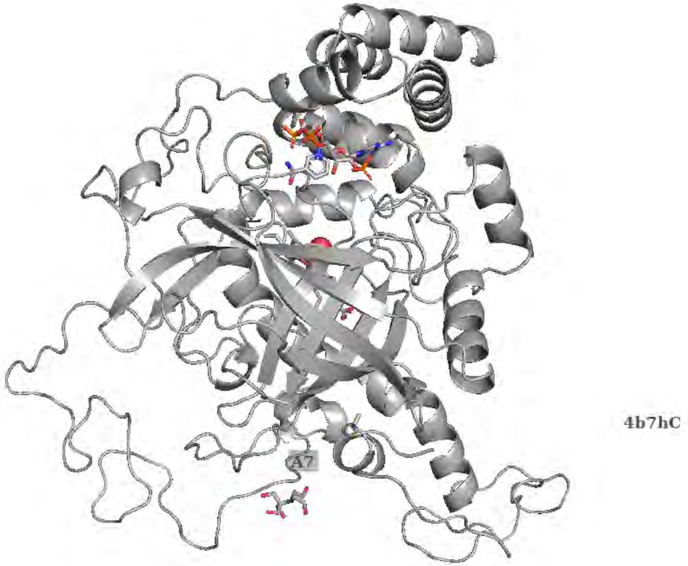

UniProt ID: A0A6I8WFM0

PDB ID: 6LFK_D

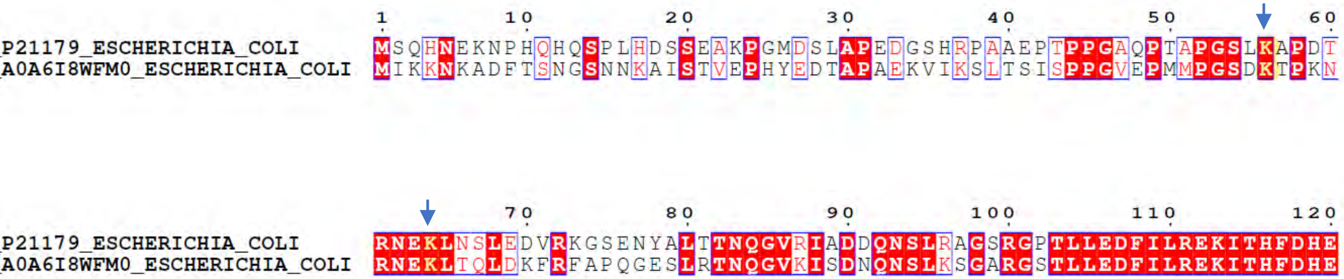

Full sequences in supplemental file.

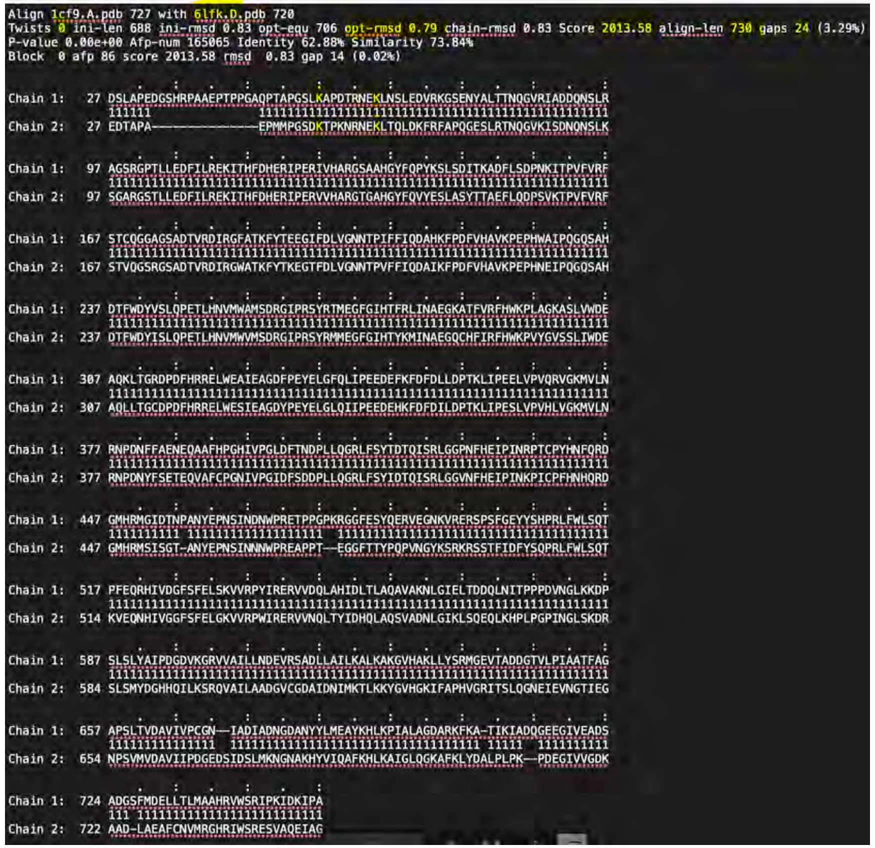

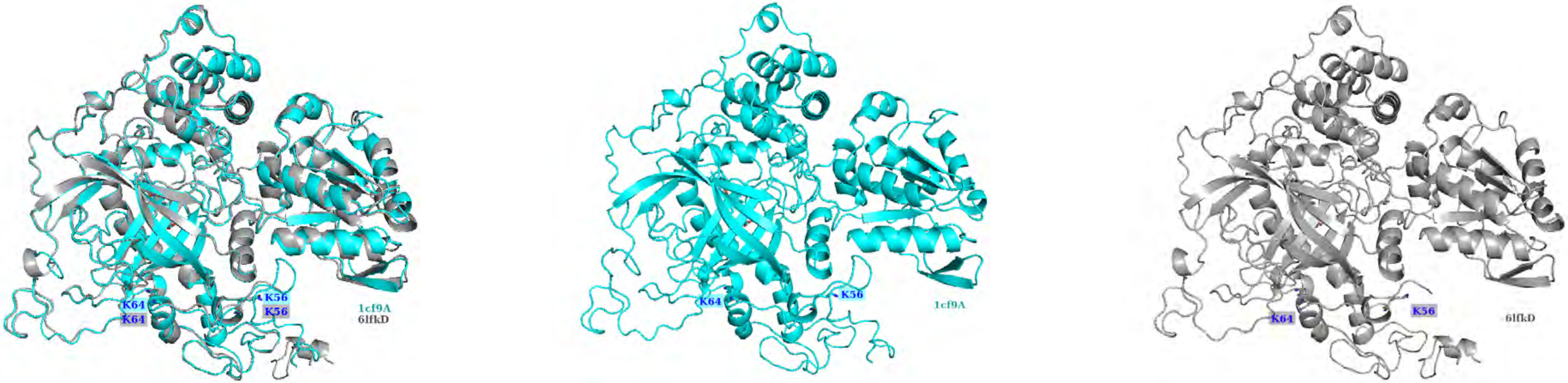

UniProt ID: A2A136
PDB ID: 2J2M_D

```
P21179_ESCHERICHIA_COLI              MSQHNEKNPHQHQSPLHDSSEAKPGMDSLAPEDGSHRPAAEPTPPGAQPTAPGSLKAPDT
A2A136_EXIGUOBACTERIUM_OXIDOTOLERANS ............................................................

P21179_ESCHERICHIA_COLI              RNEKLNSLEDVRKGSENYALTTNQGVRIADDQNSLRAGSRGPTLLEDFILREKITHFDHE
A2A136_EXIGUOBACTERIUM_OXIDOTOLERANS ............MNENEKKLTTNQGVPIGDNQNSRTAGRRGPTLLEDYQLIEKIAHFDRE
```

Full sequences in supplemental file.

```
Align 1cf9.A.pdb 727 with 2j2m.D.pdb 480
Twists 0 ini-len 464 ini-rmsd 1.15 opt-equ 480 opt-rmsd 1.28 chain-rmsd 1.15 Score 1321.53 align-len 492 gaps 12 (2.44%)
P-value 0.00e+00 Afp-num 113518 Identity 42.07% Similarity 58.54%
Block  0 afp 58 score 1321.53 rmsd  1.15 gap 26 (0.05%)

Chain 1:   78 YALTTNQGVRIADDQNSLRAGSRGPTLLEDFILREKITHFDHERIPERIVHARGSAAHGYFQPYKSLSDI
              1111111111111111111111111111111111111111111111111111111111111111111111
Chain 2:    6 KKLTTNQGVPIGDNQNSRTAGRRGPTLLEDYQLIEKIAHFDRERVPERVVHARGFGAHGVFKVKNSMKKY

Chain 1:  148 TKADFLSDPNKITPVFVRFSTCQGGAGSADTVRDIRGFATKFYTEEGIFDLVGNNTPIFFIQDAHKFPDF
              1111111111111111111111111111111111111111111111111111111111111111111111
Chain 2:   76 TKAAFLQEEGTEVPVFARFSTVIHGTHSPETLRDPRGFSVKFYTEEGNWDFVGNNLPVFFIRDAMKFPDM

Chain 1:  218 VHAVKPEPHWAIPQGQSAHDTFWDYVSLQPETLHNVMWAMSDRGIPRSYRTMEGFGIHTFRLINAEGKAT
              111111111111    11111111111111111111111111111111111111111111111111111
Chain 2:  146 VHSLKPDPRTNIQ----DPDRYWDFMTLRPESTNMLMHIFTDEGIPASYRKMRGSSVHSFKWVNAHGNTV

Chain 1:  288 FVRFHWKPLAGKASLVWDEAQKLTGRDPDFHRRELWEAIEAGDFPEYELGFQLIPEEDEFKFDFDLLDPT
              1111111111111111111111111111111111111111111111111111111111111111111111
Chain 2:  212 YIKLRWVPKEGVHNLSADEATEVQGKDFNHASNDTFQAIENGDFPEWDLFVQVLDPADVENFDFDPLDAT

Chain 1:  358 KLIPEELVPVQRVGKMVLNRNPDNFFAENEQAAFHPGHIVPGLDFTNDPLLQGRLFSYTDTQISRLGGPN
              111111111111111111111111111111111111111111111111111111111111111111 1
Chain 2:  282 KDWFEDVIPFQHVGTMTLNKNVDNYFAETESVGFNPGVLVPGMLPSEDKLLQGRLFSYSDTQRHRIGP-N

Chain 1:  428 FHEIPINRPTCPYHNFQRDGMHRMGIDTNPANYEPNSINDNWPRETPPGPKRGGFESYQERVEGNKVRER
              11111111111111111111111111111111111 1111111111     11111111111111111
Chain 2:  351 YQQLPINCPFAQVNNYQRDGAMPFKQQTSSVNYEPNR-YQDEPKQTPE-----YTEDTQPLHDDIHGRLE

Chain 1:  498 SPSFGEYYSHPRLFWLSQTPFEQRHIVDGFSFELSKVVRPYIRERVVDQLAHIDLTLAQAVAKNLGIELT
              111 111111111111111111111111111111111111111111111111111111111111111111
Chain 2:  415 IEK-TNNFGQAGEVYRRMTEEEQMALLNNLVNDLQQVRHENTVLLAICNFYRADASLGEKLSEALNVDIK

Chain 1:  568 DD
              11
Chain 2:  484 PF
```

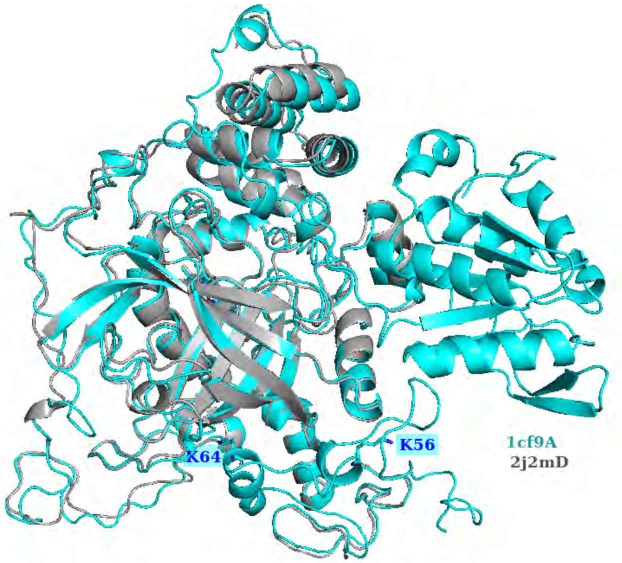

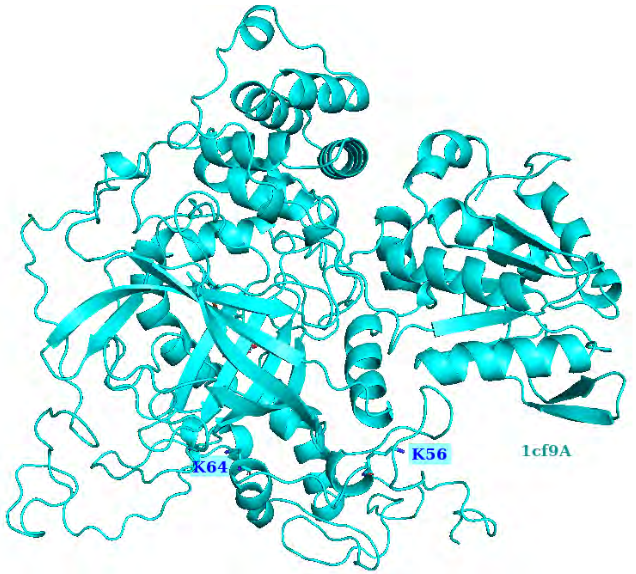

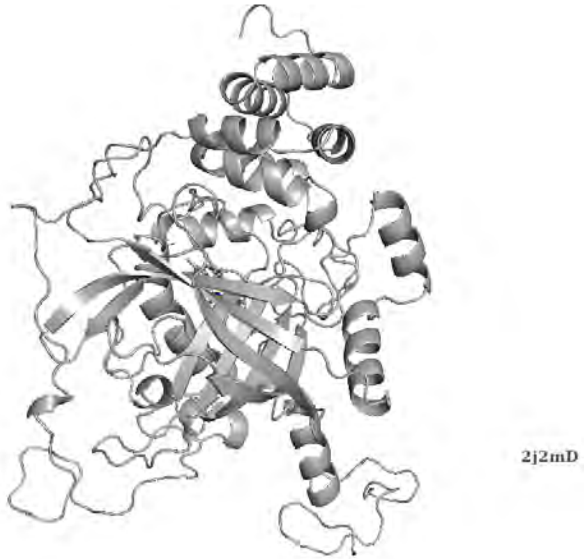

UniProt ID: C1PHG1
PDB ID: 6RJN_C

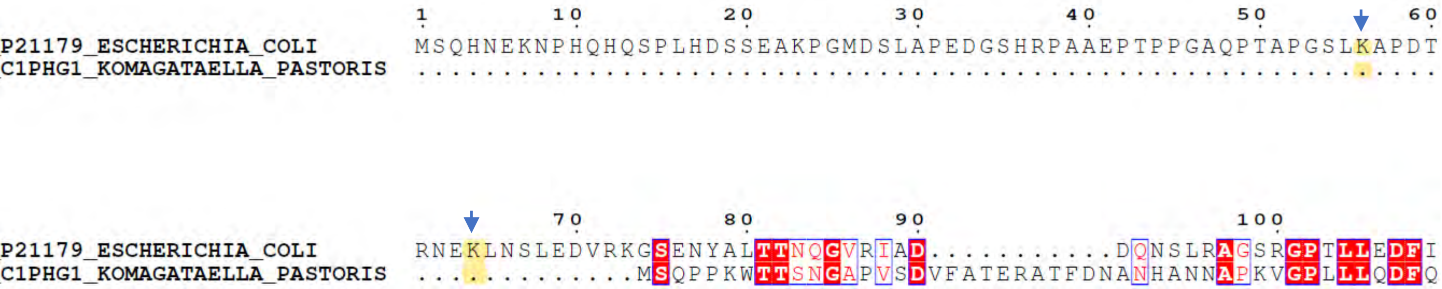

Full sequences in supplemental file.

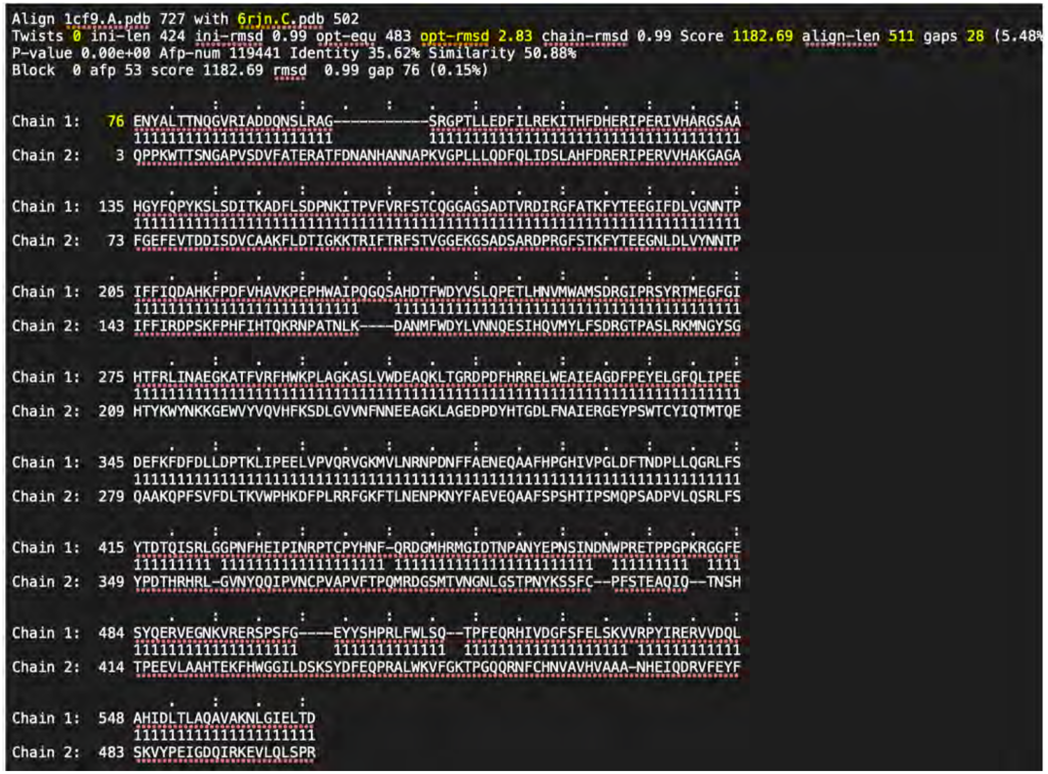

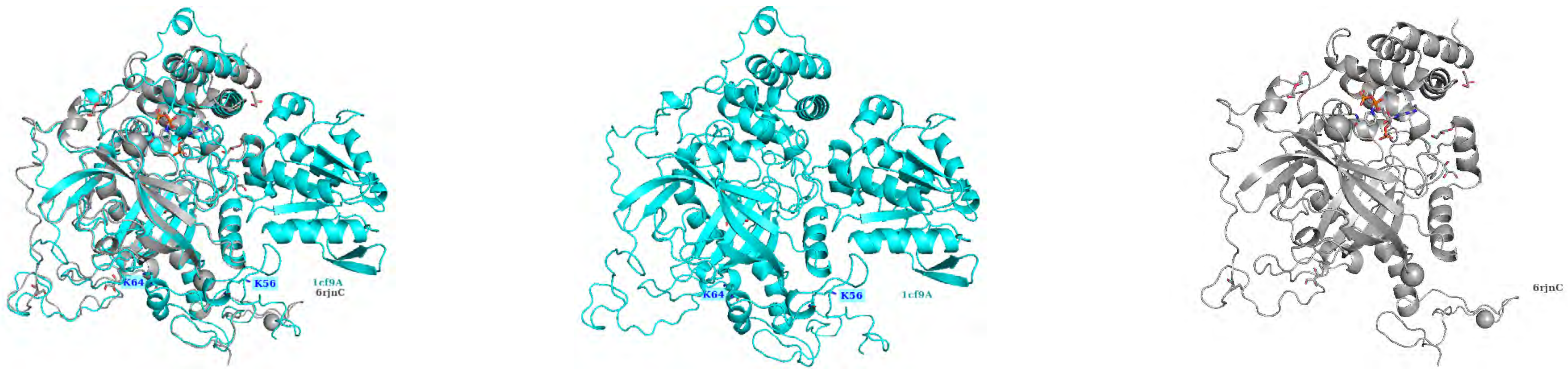

UniProt ID: D9N167

PDB ID: 2IUF_E

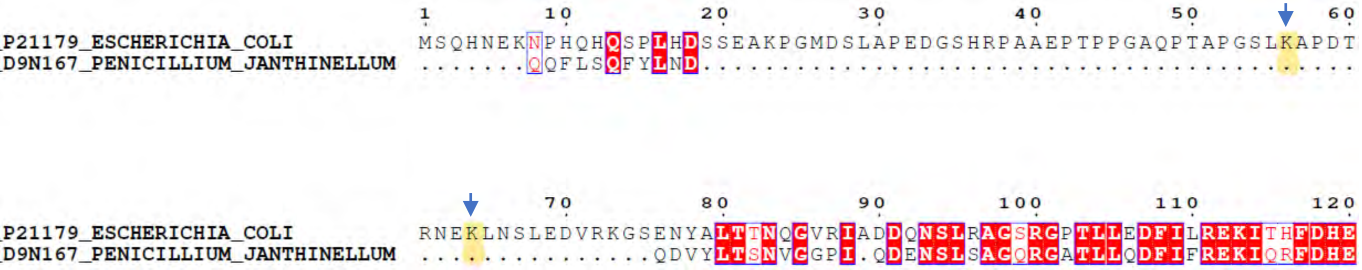

Full sequences in supplemental file.

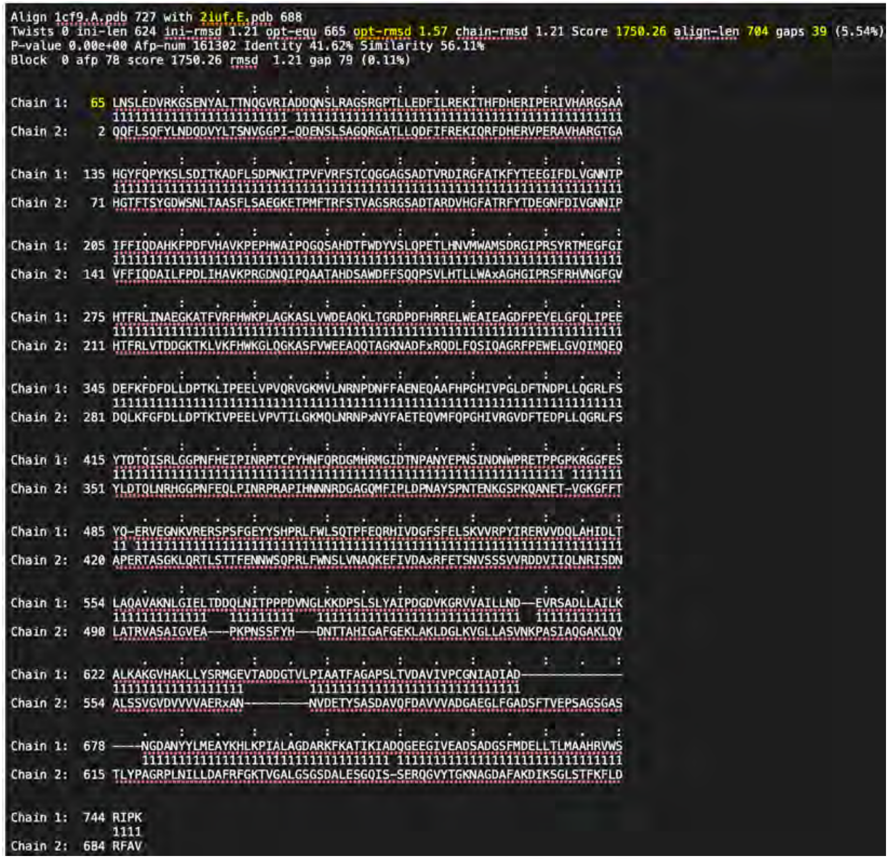

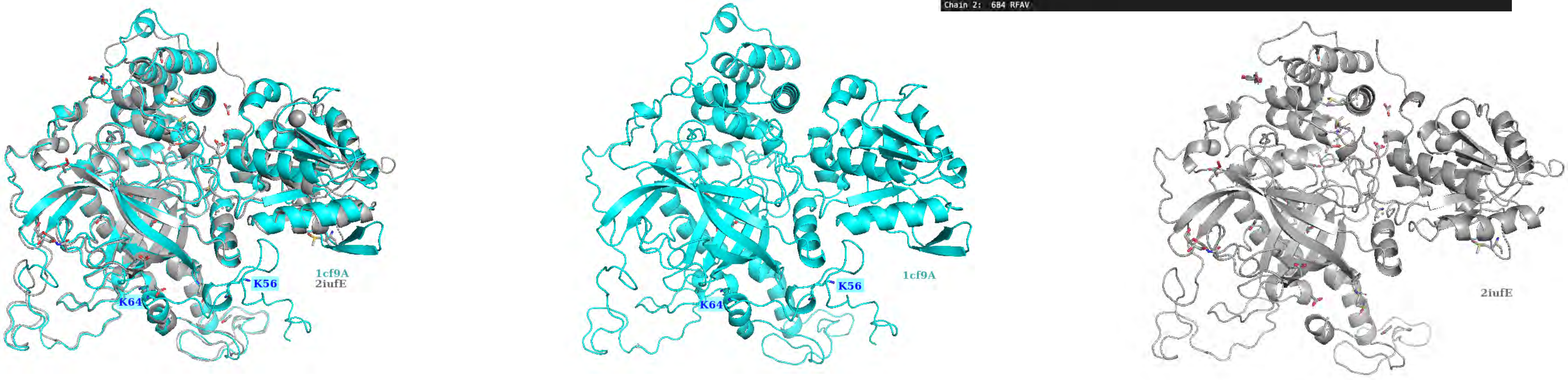

UniProt ID: D9N167

PDB ID: 2XF2_A

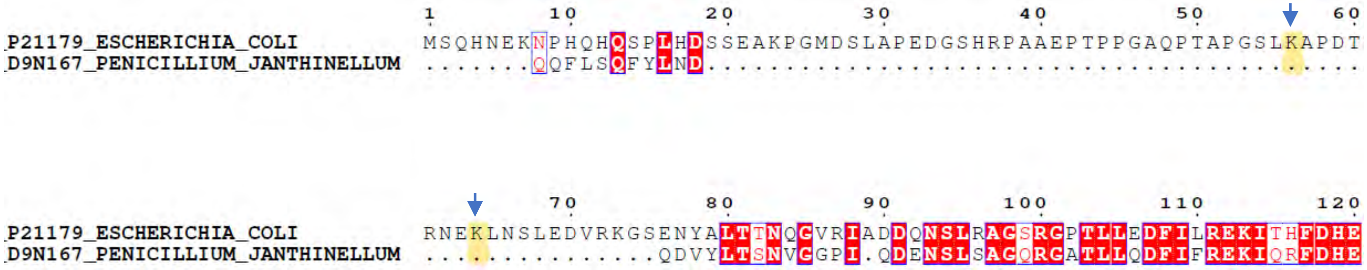

Full sequences in supplemental file.

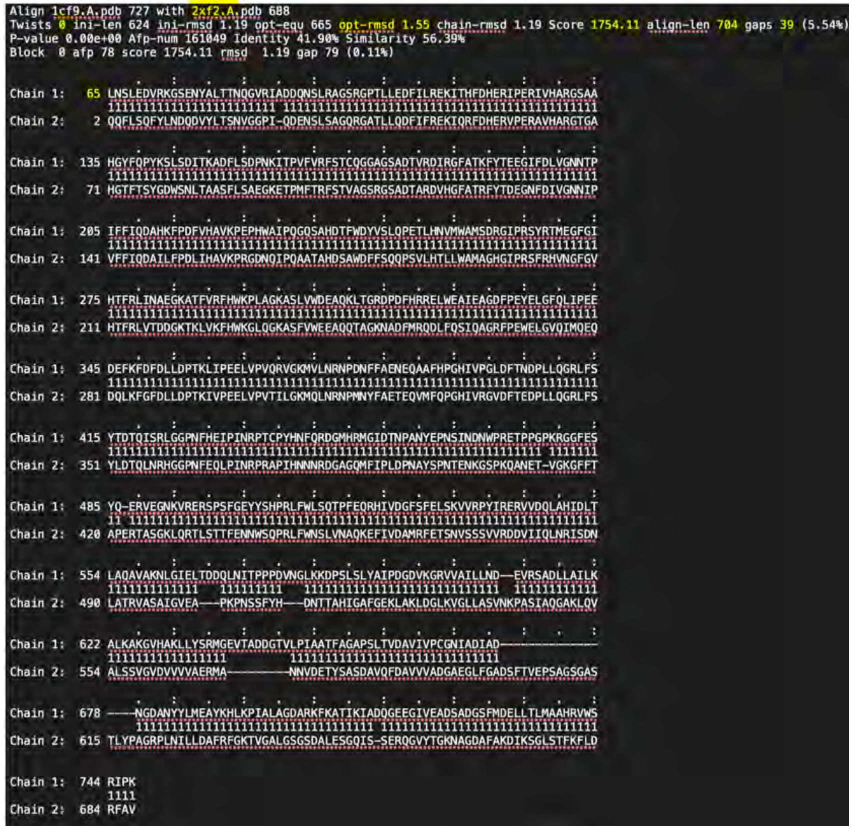

```
Align 1cf9.A.pdb 727 with 2xf2.A.pdb 688
Twists 0 ini-len 624 ini-rmsd 1.19 opt-equ 665 opt-rmsd 1.55 chain-rmsd 1.19 Score 1754.11 align-len 704 gaps 39 (5.54%)
P-value 0.00e+00 Afp-num 161049 Identity 41.90% Similarity 56.39%
Block  0 afp 78 score 1754.11 rmsd  1.19 gap 79 (0.11%)

Chain 1:   65 LNSLEDVRKGSENYALTTNQGVRIADDQNSLRAGSRGPTLLEDFILREKITHFDHERIPERIVHARGSAA
              111111111111111111111111 1111111111111111111111111111111111111111111111
Chain 2:    2 QQFLSQFYLNDQDVYLTSNVGGPI-QDENSLSAGQRGATLLQDFIFREKIQRFDHERVPERAVHARGTGA

Chain 1:  135 HGYFQPYKSLSDITKADFLSDPNKITPVFVRFSTCQGGAGSADTVRDIRGFATKFYTEEGIFDLVGNNTP
              1111111111111111111111111111111111111111111111111111111111111111111111
Chain 2:   71 HGTFTSYGDWSNLTAASFLSAEGKETPMFTRFSTVAGSRGSADTARDVHGFATRFYTDEGNFDIVGNNIP

Chain 1:  205 IFFIQDAHKFPDFVHAVKPEPHWAIPQGQSAHDTFWDYVSLQPETLHNVMWAMSDRGIPRSYRTMEGFGI
              1111111111111111111111111111111111111111111111111111111111111111111111
Chain 2:  141 VFFIQDAILFPDLIHAVKPRGDNQIPQAATAHDSAWDFFSQQPSVLHTLLWAMAGHGIPRSFRHVNGFGV

Chain 1:  275 HTFRLINAEGKATFVRFHWKPLAGKASLVWDEAQKLTGRDPDFHRRELWEAIEAGDFPEYELGFQLIPEE
              1111111111111111111111111111111111111111111111111111111111111111111111
Chain 2:  211 HTFRLVTDDGKTKLVKFHWKGLQGKASFVWEEAQQTAGKNADFMRQDLFQSIQAGRFPEWELGVQIMQEQ

Chain 1:  345 DEFKFDFDLLDPTKLIPEELVPVQRVGKMVLNRNPDNFFAENEQAAFHPGHIVPGLDFTNDPLLQGRLFS
              1111111111111111111111111111111111111111111111111111111111111111111111
Chain 2:  281 DQLKFGFDLLDPTKIVPEELVPVTILGKMQLNRNPMNYFAETEQVMFQPGHIVRGVDFTEDPLLQGRLFS

Chain 1:  415 YTDTQISRLGGPNFHEIPINRPTCPYHNFQRDGMHRMGIDTNPANYEPNSINDNWPRETPPGPKRGGFES
              111111111111111111111111111111111111111111111111111111111111111 1111111
Chain 2:  351 YLDTQLNRHGGPNFEQLPINRPRAPIHNNNRDGAGQMFIPLDPNAYSPNTENKGSPKQANET-VGKGFFT

Chain 1:  485 YQ-ERVEGNKVRERSPSFGEYYSHPRLFWLSQTPFEQRHIVDGFSFELSKVVRPYIRERVVDQLAHIDLT
              11 11111111111111111111111111111111111111111111111111111111111111111111
Chain 2:  420 APERTASGKLQRTLSTTFENNWSQPRLFWNSLVNAQKEFIVDAMRFETSNVSSSVVRDDVIIQLNRISDN

Chain 1:  554 LAQAVAKNLGIELTDDQLNITPPPDVNGLKKDPSLSLYAIPDGDVKGRVVAILLND--EVRSADLLAILK
              1111111111111   111111111   111111111111111111111111111111  11111111111
Chain 2:  490 LATRVASAIGVEA---PKPNSSFYH---DNTTAHIGAFGEKLAKLDGLKVGLLASVNKPASIAQGAKLQV

Chain 1:  622 ALKAKGVHAKLLYSRMGEVTADDGTVLPIAATFAGAPSLTVDAVIVPCGNIADIAD--------------
              11111111111111111          11111111111111111111111111111111
Chain 2:  554 ALSSVGVDVVVVAERMA----------NNVDETYSASDAVQFDAVVVADGAEGLFGADSFTVEPSAGSGAS

Chain 1:  678 ----NGDANYYLMEAYKHLKPIALAGDARKFKATIKIADQGEEGIVEADSADGSFMDELLTLMAAHRVWS
                  111111111111111111111111111111111111 11111111111111111111111111111111
Chain 2:  615 TLYPAGRPLNILLDAFRFGKTVGALGSGSDALESGQIS-SERQGVYTGKNAGDAFAKDIKSGLSTFKFLD

Chain 1:  744 RIPK
              1111
Chain 2:  684 RFAV
```

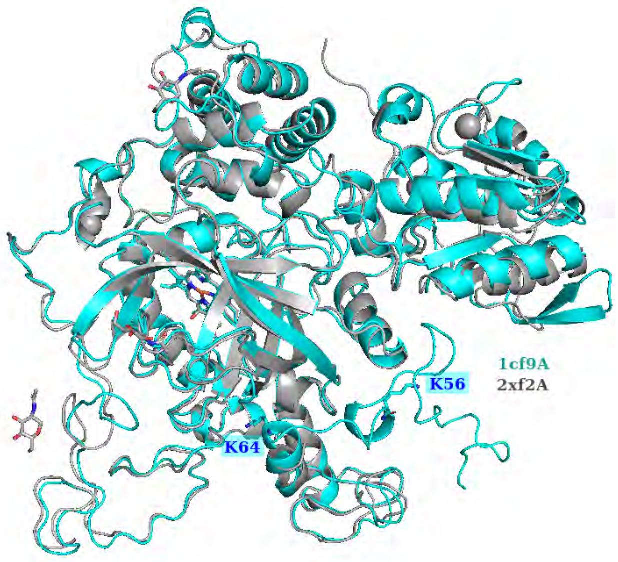

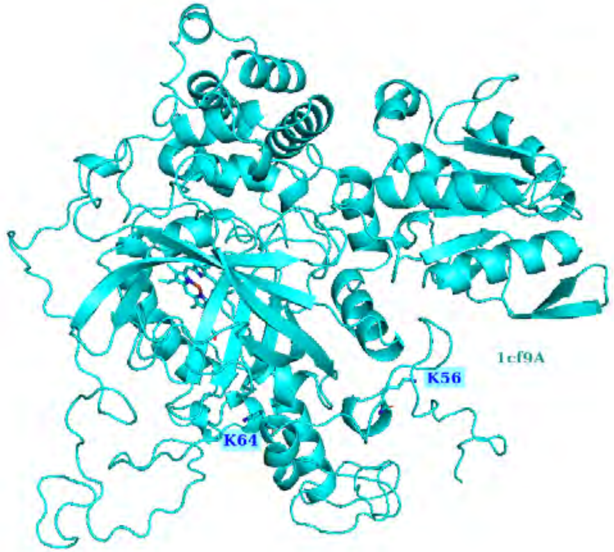

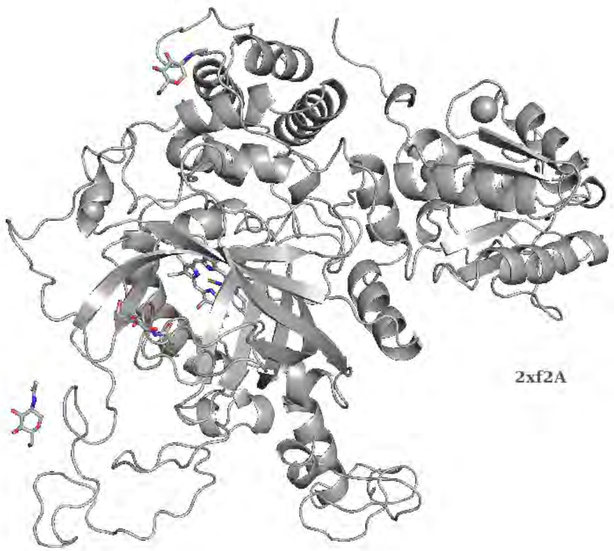

UniProt ID: M4GGR5

PDB ID: 4AUE_A

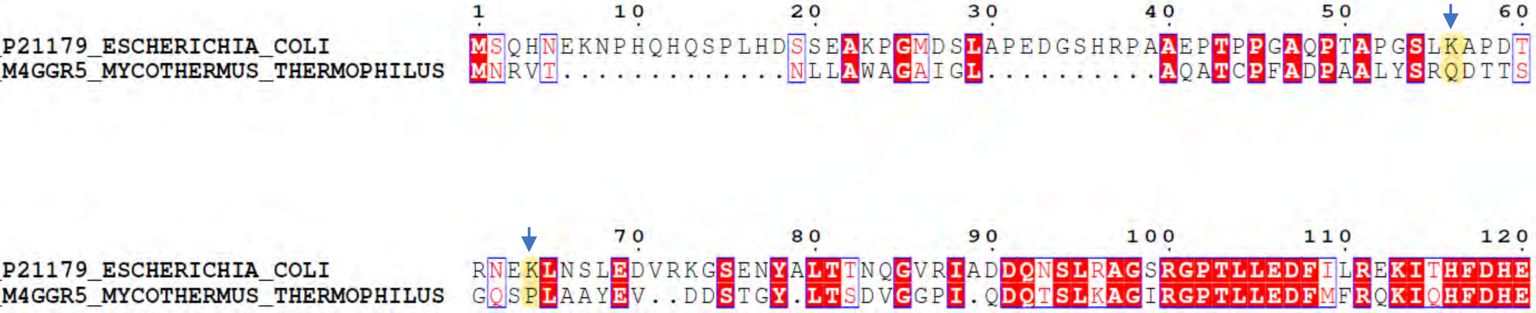

Full sequences in supplemental file.

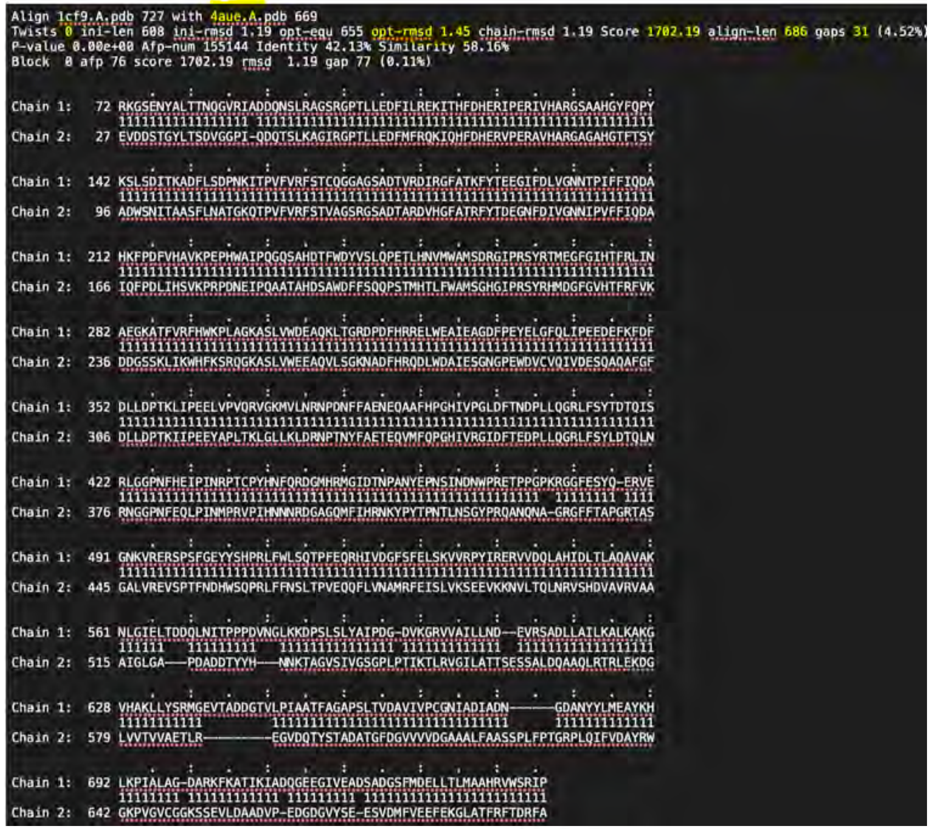

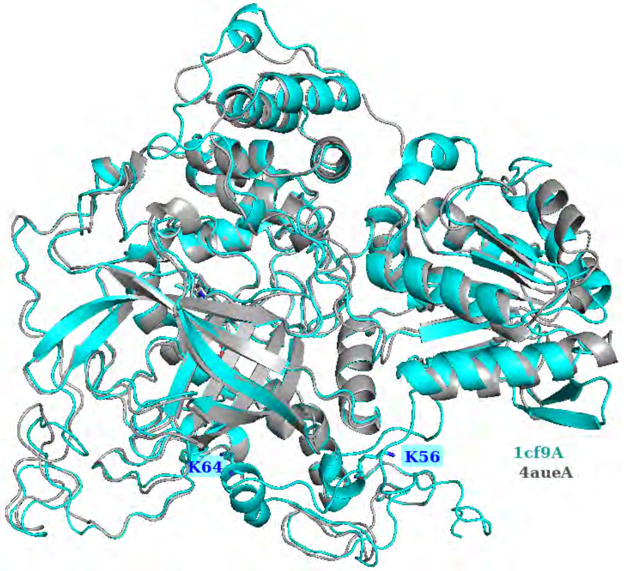

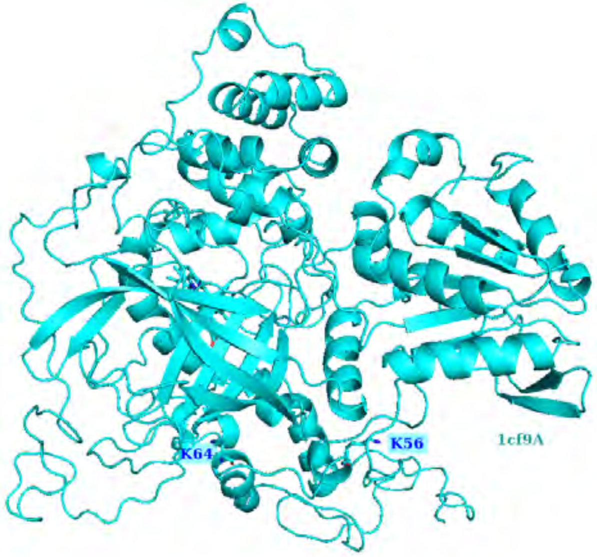

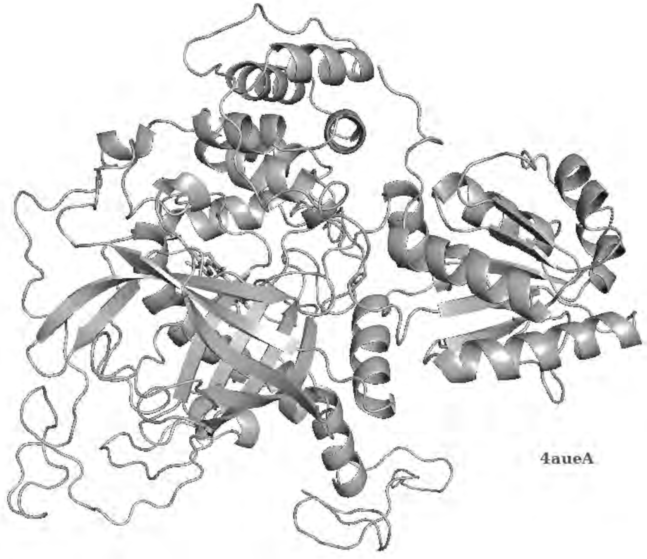

UniProt ID: M4GGR5

PDB ID: 4B7A_D

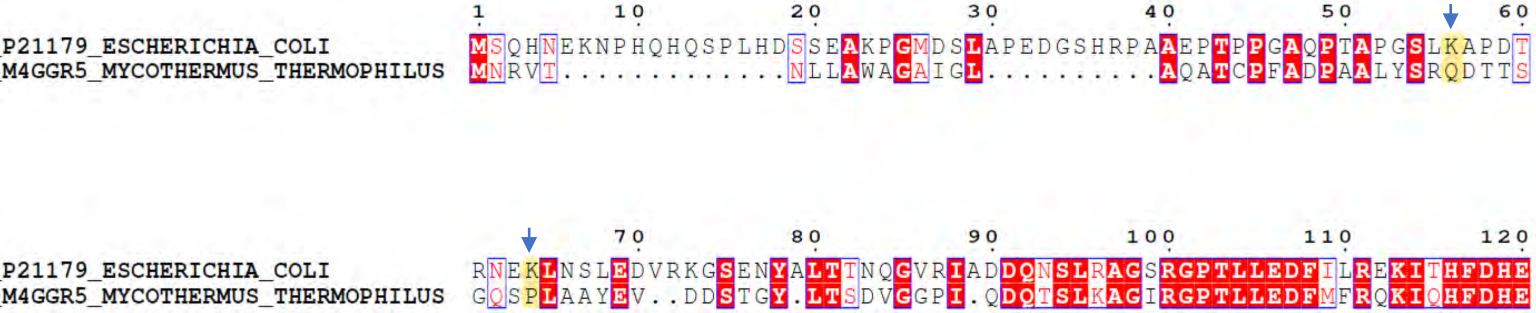

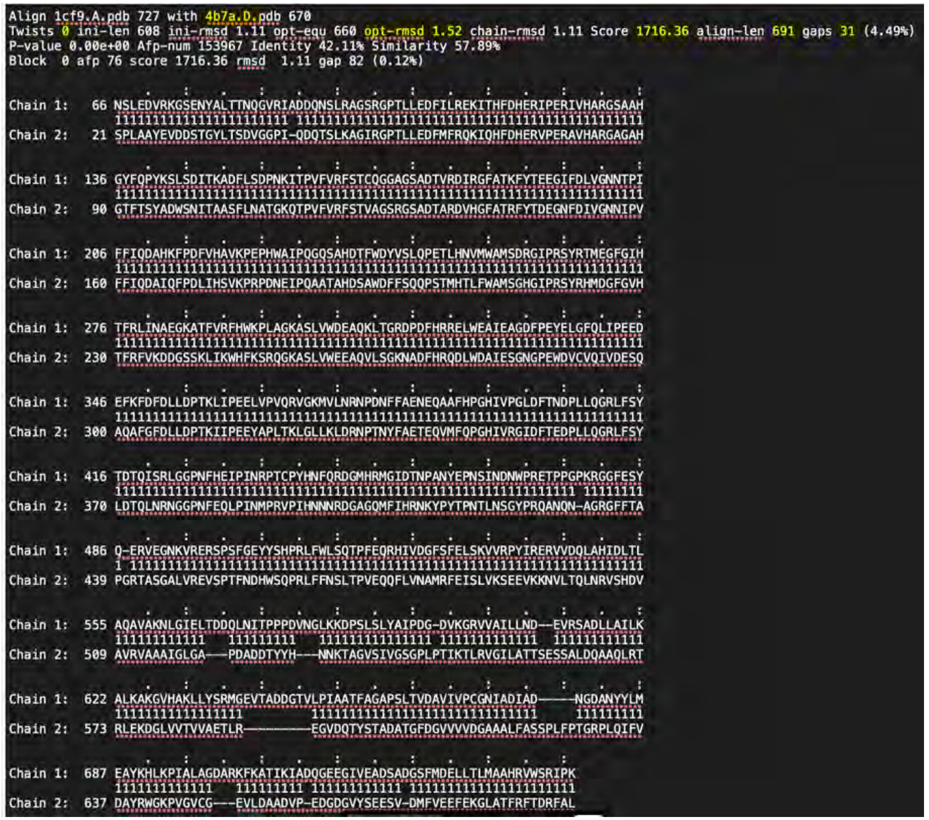

Full sequences in supplemental file.

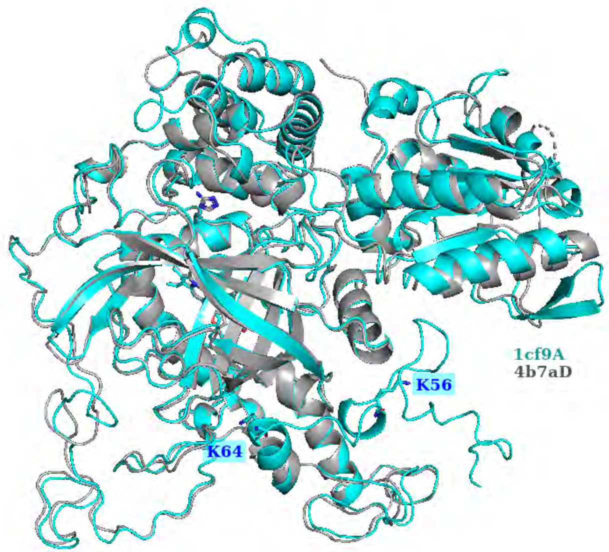

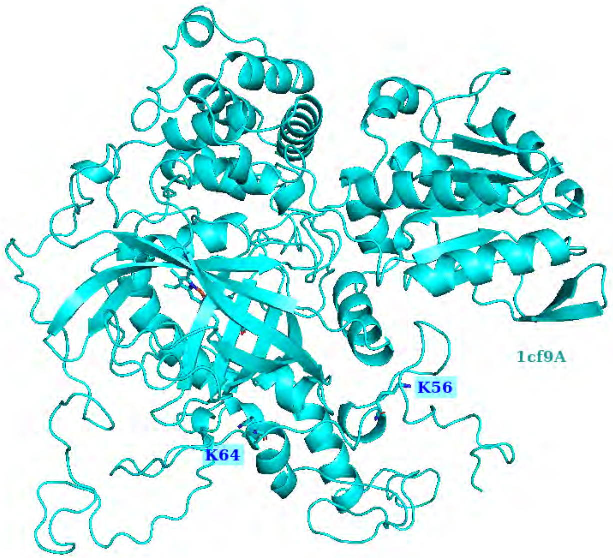

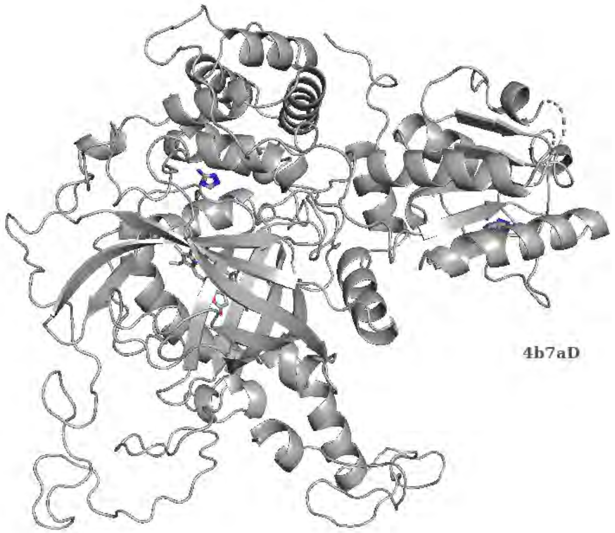

UniProt ID: M4GGR5

PDB ID: 5YEM_B

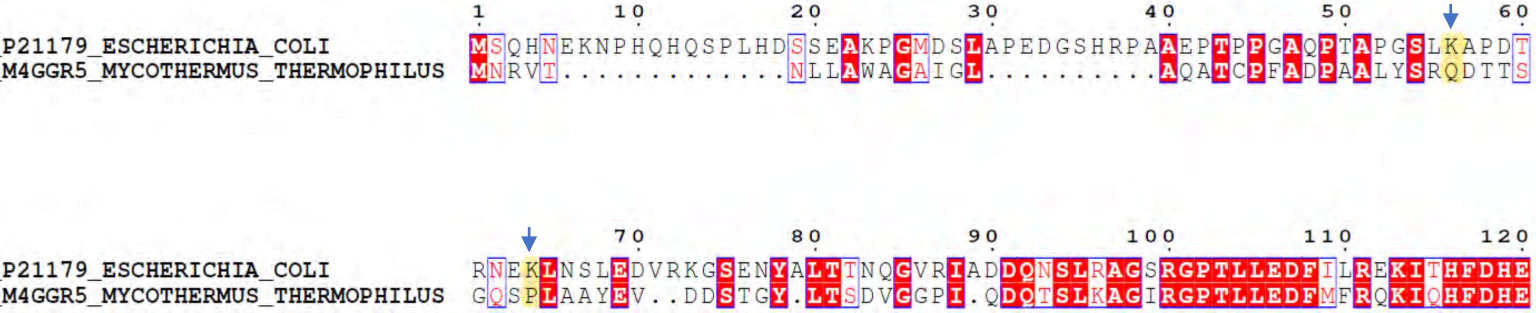

Full sequences in supplemental file.

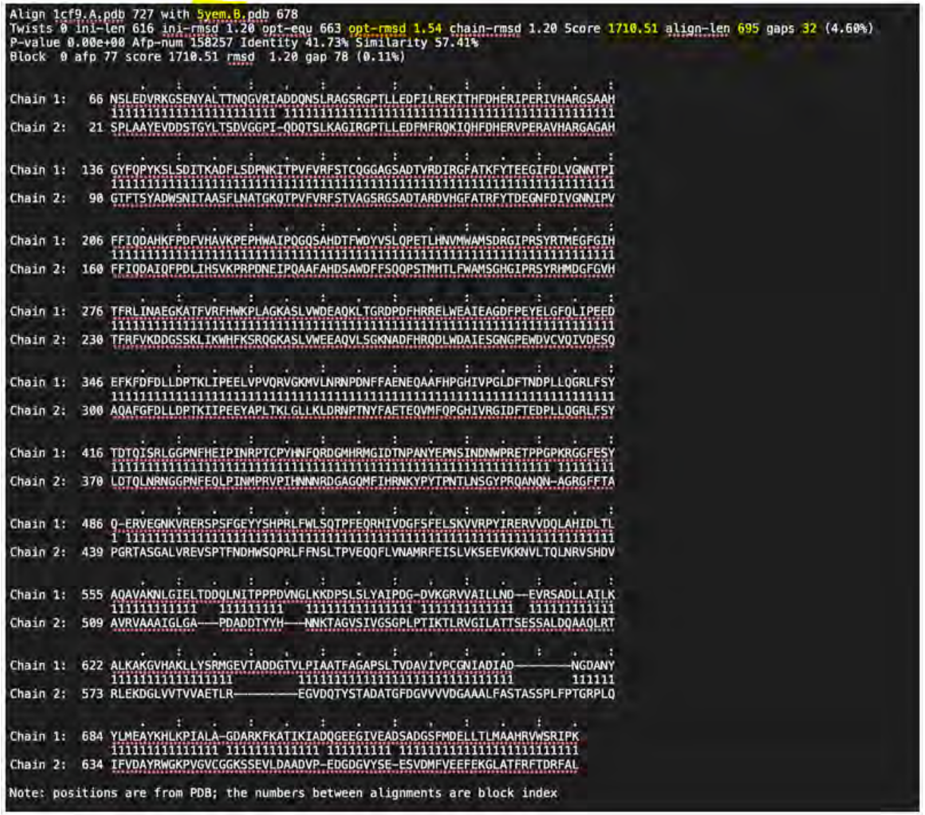

```
Align 1cf9.A.pdb 727 with 5yem.B.pdb 678
Twists 0 ini-len 616 ini-rmsd 1.20 opt-equ 663 opt-rmsd 1.54 chain-rmsd 1.20 Score 1710.51 align-len 695 gaps 32 (4.60%)
P-value 0.00e+00 Afp-num 158257 Identity 41.73% Similarity 57.41%
Block  0 afp 77 score 1710.51 rmsd  1.20 gap 78 (0.11%)

Chain 1:   66 NSLEDVRKGSENYALTTNQGVRIADDQNSLRAGSRGPTLLEDFILREKITHFDHERIPERIVHARGSAAH
Chain 2:   21 SPLAAYEVDDSTGYLTSDVGGPI-QDQTSLKAGIRGPTLLEDFMFRQKIQHFDHERVPERAVHARGAGAH

Chain 1:  136 GYFQPYKSLSDITKADFLSDPNKITPVFVRFSTCQGGAGSADTVRDIRGFATKFYTEEGIFDLVGNNTPI
Chain 2:   90 GTFTSYADWSNITAASFLNATGKQTPVFVRFSTVAGSRGSADTARDVHGFATRFYTDEGNFDIVGNNIPV

Chain 1:  206 FFIQDAHKFPDFVHAVKPEPHWAIPQGQSAHDTFWDYVSLQPETLHNVMWAMSDRGIPRSYRTMEGFGIH
Chain 2:  160 FFIQDAIQFPDLIHSVKPRPDNEIPQAAFAHDSAWDFFSQQPSTMHTLFWAMSGHGIPRSYRHMDGFGVH

Chain 1:  276 TFRLINAEGKATFVRFHWKPLAGKASLVWDEAQKLTGRDPDFHRRELWEAIEAGDFPEYELGFQLIPEED
Chain 2:  230 TFRFVKDDGSSKLIKWHFKSRQGKASLVWEEAQVLSGKNADFHRQDLWDAIESGNGPEWDVCVQIVDESQ

Chain 1:  346 EFKFDFDLLDPTKLIPEELVPVQRVGKMVLNRNPDNFFAENEQAAFHPGHIVPGLDFTNDPLLQGRLFSY
Chain 2:  300 AQAFGFDLLDPTKIIPEEYAPLTKLGLLKLDRNPTNYFAETEQVMFQPGHIVRGIDFTEDPLLQGRLFSY

Chain 1:  416 TDTQISRLGGPNFHEIPINRPTCPYHNFQRDGMHRMGIDTNPANYEPNSINDNWPRETPPGPKRGGFESY
Chain 2:  370 LDTQLNRNGGPNFEQLPINMPRVPIHNNNRDGAGQMFIHRNKYPYTPNTLNSGYPRQANQN-AGRGFFTA

Chain 1:  486 Q-ERVEGNKVRERSPSFGEYYSHPRLFWLSQTPFEQRHIVDGFSFELSKVVRPYIRERVVDQLAHIDLTL
Chain 2:  439 PGRTASGALVREVSPTFNDHWSQPRLFFNSLTPVEQQFLVNAMRFEISLVKSEEVKKNVLTQLNRVSHDV

Chain 1:  555 AQAVAKNLGIELTDDQLNITPPPDVNGLKKDPSLSLYAIPDG-DVKGRVVAILLND--EVRSADLLAILK
Chain 2:  509 AVRVAAAIGLGA---PDADDTYYH---NNKTAGVSIVGSGPLPTIKTLRVGILATTSESSALDQAAQLRT

Chain 1:  622 ALKAKGVHAKLLYSRMGEVTADDGTVLPIAATFAGAPSLTVDAVIVPCGNIADIAD---------NGDANY
Chain 2:  573 RLEKDGLVVTVVAETLR----------EGVDQTYSTADATGFDGVVVVDGAAALFASTASSPLFPTGRPLQ

Chain 1:  684 YLMEAYKHLKPIALA-GDARKFKATIKIADQGEEGIVEADSADGSFMDELLTLMAAHRVWSRIPK
Chain 2:  634 IFVDAYRWGKPVGVCGGKSSEVLDAADVP-EDGDGVYSE-ESVDMFVEEFEKGLATFRFTDRFAL

Note: positions are from PDB; the numbers between alignments are block index
```

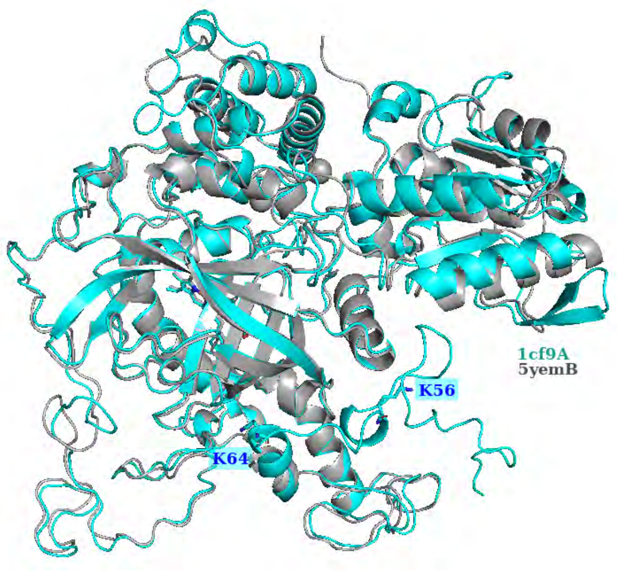

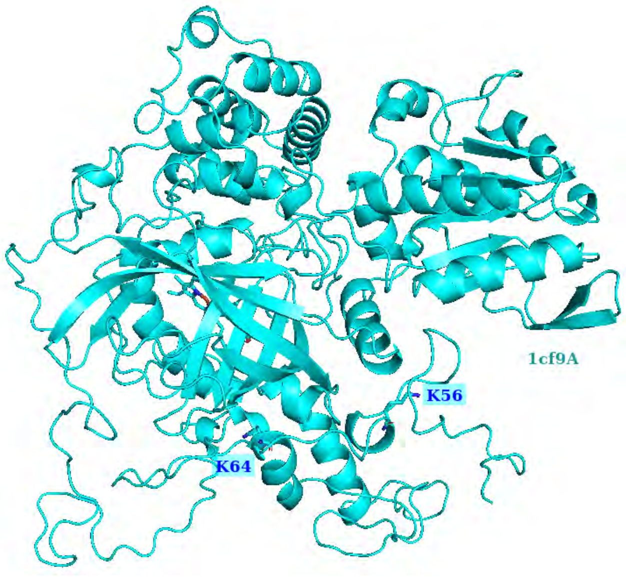

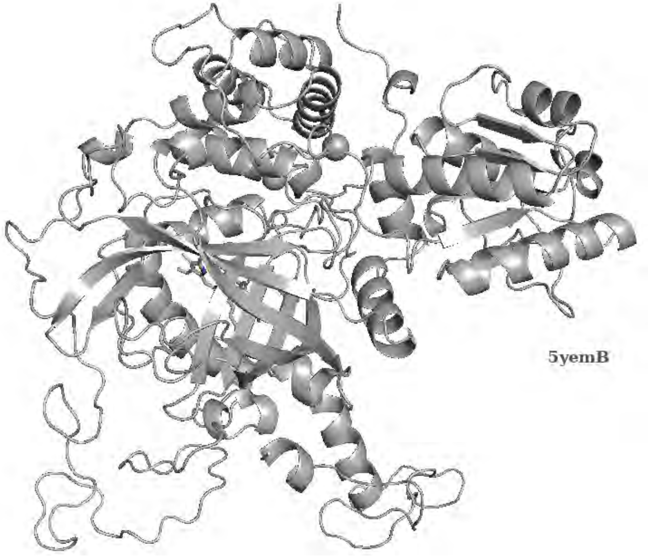

UniProt ID: M4GGR6
PDB ID: 4AUL_B

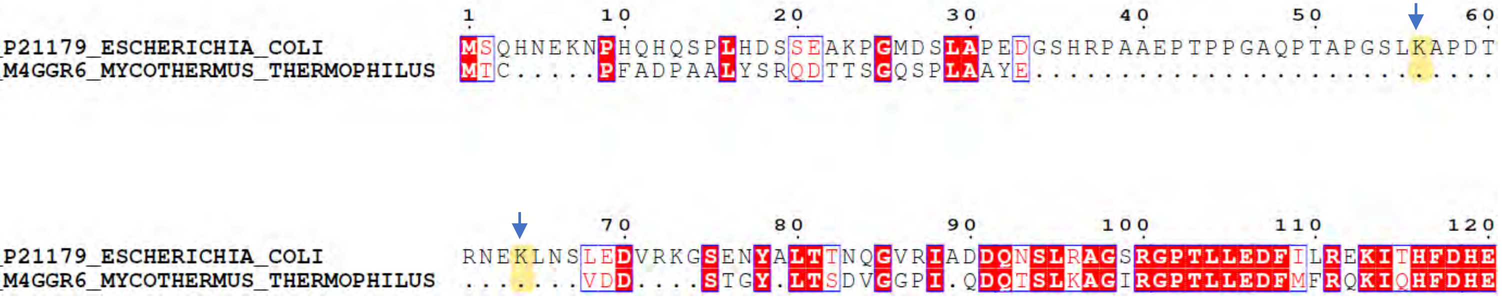

Full sequences in supplemental file.

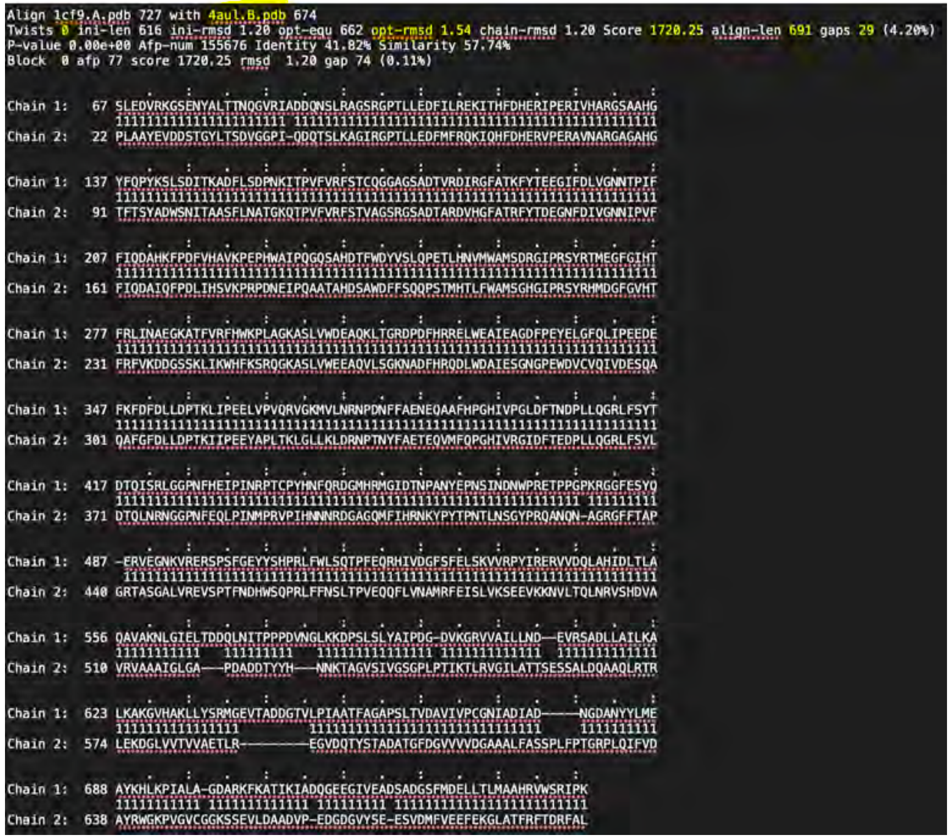

```
Align 1cf9.A.pdb 727 with 4aul.B.pdb 674
Twists 0 ini-len 616 ini-rmsd 1.20 opt-equ 662 opt-rmsd 1.54 chain-rmsd 1.20 Score 1720.25 align-len 691 gaps 29 (4.20%)
P-value 0.00e+00 Afp-num 155676 Identity 41.82% Similarity 57.74%
Block  0 afp 77 score 1720.25 rmsd  1.20 gap 74 (0.11%)

Chain 1:   67 SLEDVRKGSENYALTTNQGVRIADDQNSLRAGSRGPTLLEDFILREKITHFDHERIPERIVHARGSAAHG
              1111111111111111111111 111111111111111111111111111111111111111111111111
Chain 2:   22 PLAAYEVDDSTGYLTSDVGGPI-QDQTSLKAGIRGPTLLEDFMFRQKIQHFDHERVPERAVNARGAGAHG

Chain 1:  137 YFQPYKSLSDITKADFLSDPNKITPVFVRFSTCQGGAGSADTVRDIRGFATKFYTEEGIFDLVGNNTPIF
              1111111111111111111111111111111111111111111111111111111111111111111111
Chain 2:   91 TFTSYADWSNITAASFLNATGKQTPVFVRFSTVAGSRGSADTARDVHGFATRFYTDEGNFDIVGNNIPVF

Chain 1:  207 FIQDAHKFPDFVHAVKPEPHWAIPQGQSAHDTFWDYVSLQPETLHNVMWAMSDRGIPRSYRTMEGFGIHT
              1111111111111111111111111111111111111111111111111111111111111111111111
Chain 2:  161 FIQDAIQFPDLIHSVKPRPDNEIPQAATAHDSAWDFFSQQPSTMHTLFWAMSGHGIPRSYRHMDGFGVHT

Chain 1:  277 FRLINAEGKATFVRFHWKPLAGKASLVWDEAQKLTGRDPDFHRRELWEAIEAGDFPEYELGFQLIPEEDE
              1111111111111111111111111111111111111111111111111111111111111111111111
Chain 2:  231 FRFVKDDGSSKLIKWHFKSRQGKASLVWEEAQVLSGKNADFHRQDLWDAIESGNGPEWDVCVQIVDESQA

Chain 1:  347 FKFDFDLLDPTKLIPEELVPVQRVGKMVLNRNPDNFFAENEQAAFHPGHIVPGLDFTNDPLLQGRLFSYT
              1111111111111111111111111111111111111111111111111111111111111111111111
Chain 2:  301 QAFGFDLLDPTKIIPEEYAPLTKLGLLKLDRNPTNYFAETEQVMFQPGHIVRGIDFTEDPLLQGRLFSYL

Chain 1:  417 DTQISRLGGPNFHEIPINRPTCPYHNFQRDGMHRMGIDTNPANYEPNSINDNWPRETPPGPKRGGFESYQ
              111111111111111111111111111111111111111111111111111111111111 111111111
Chain 2:  371 DTQLNRNGGPNFEQLPINMPRVPIHNNNRDGAGQMFIHRNKYPYTPNTLNSGYPRQANQN-AGRGFFTAP

Chain 1:  487 -ERVEGNKVRERSPSFGEYYSHPRLFWLSQTPFEQRHIVDGFSFELSKVVRPYIRERVVDQLAHIDLTLA
               111111111111111111111111111111111111111111111111111111111111111111111
Chain 2:  440 GRTASGALVREVSPTFNDHWSQPRLFFNSLTPVEQQFLVNAMRFEISLVKSEEVKKNVLTQLNRVSHDVA

Chain 1:  556 QAVAKNLGIELTDDQLNITPPPDVNGLKKDPSLSLYAIPDG-DVKGRVVAILLND--EVRSADLLAILKA
              11111111111   111111111   111111111111111 1111111111111  1111111111111
Chain 2:  510 VRVAAAIGLGA---PDADDTYYH---NNKTAGVSIVGSGPLPTIKTLRVGILATTSESSALDQAAQLRTR

Chain 1:  623 LKAKGVHAKLLYSRMGEVTADDGTVLPIAATFAGAPSLTVDAVIVPCGNIADIAD------NGDANYYLME
              1111111111111111         111111111111111111111111111111      1111111111
Chain 2:  574 LEKDGLVVTVVAETLR---------EGVDQTYSTADATGFDGVVVVDGAAALFASSPLFPTGRPLQIFVD

Chain 1:  688 AYKHLKPIALA-GDARKFKATIKIADQGEEGIVEADSADGSFMDELLTLMAAHRVWSRIPK
              11111111111 1111111111111 111111111 111111111111111111111111111
Chain 2:  638 AYRWGKPVGVCGGKSSEVLDAADVP-EDGDGVYSE-ESVDMFVEEFEKGLATFRFTDRFAL
```

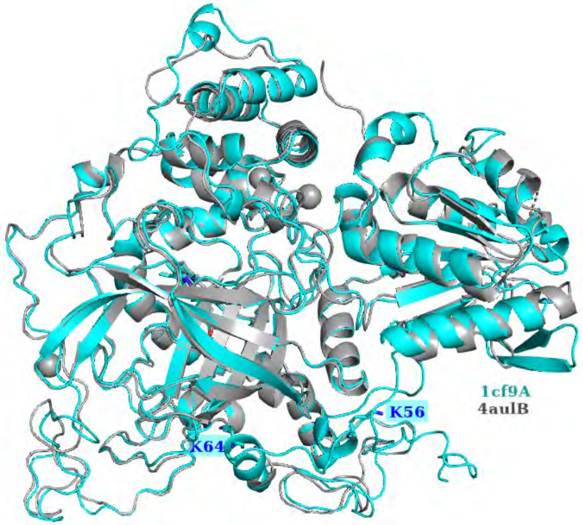

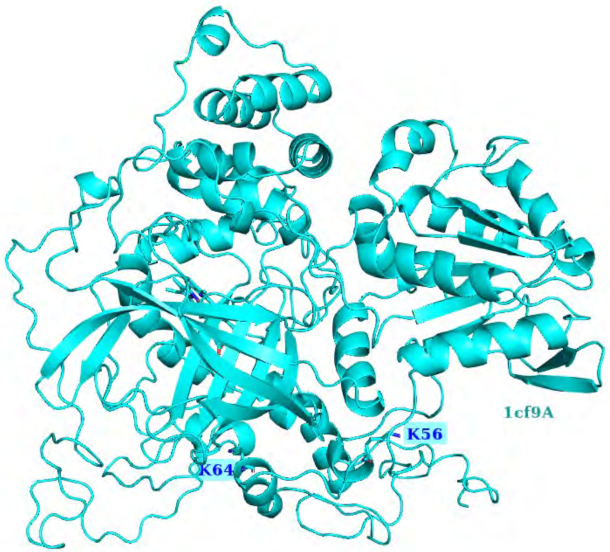

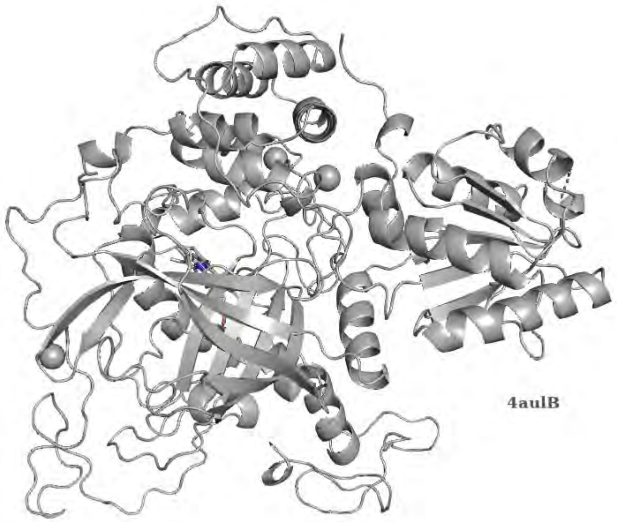

UniProt ID: M4GGR7
PDB ID: 4AUM_D

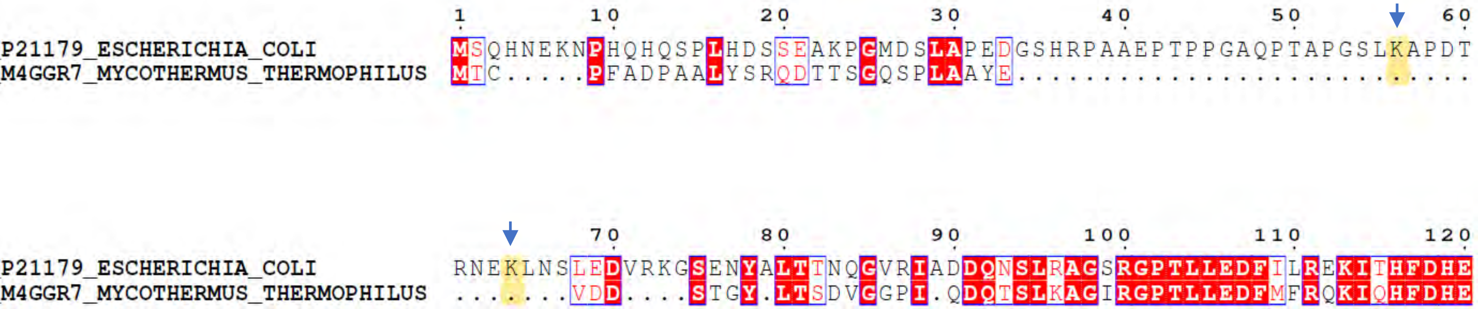

Full sequences in supplemental file.

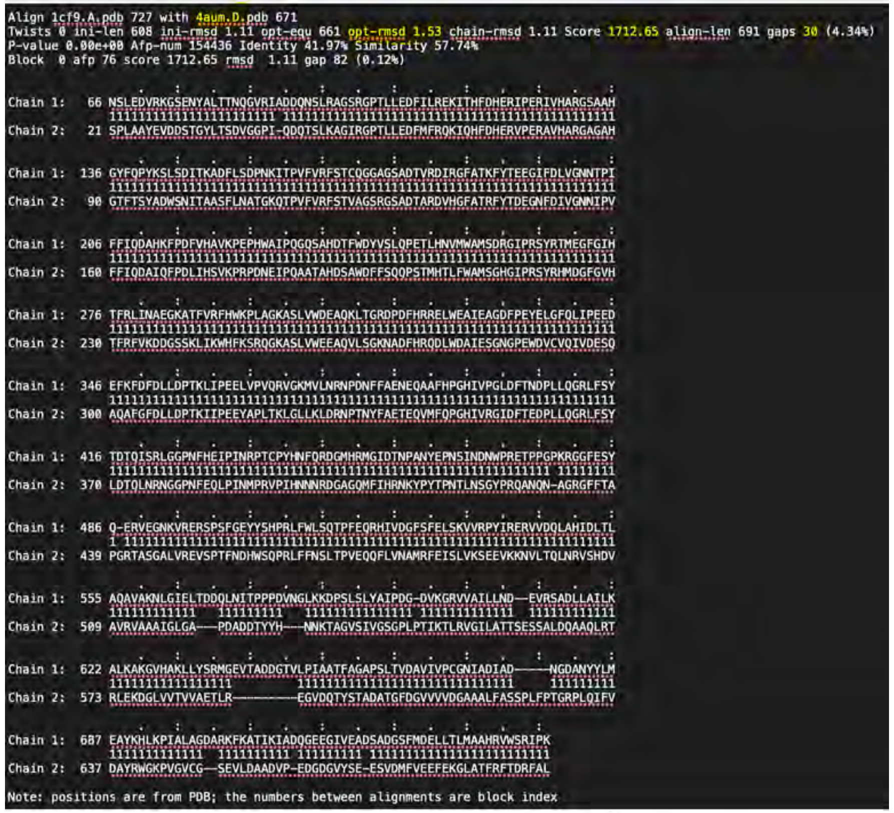
```
Align 1cf9.A.pdb 727 with 4aum.D.pdb 671
Twists 0 ini-len 608 ini-rmsd 1.11 opt-equ 661 opt-rmsd 1.53 chain-rmsd 1.11 Score 1712.65 align-len 691 gaps 30 (4.34%)
P-value 0.00e+00 Afp-num 154436 Identity 41.97% Similarity 57.74%
Block  0 afp 76 score 1712.65 rmsd  1.11 gap 82 (0.12%)

Chain 1:   66 NSLEDVRKGSENYALTTNQGVRIADDQNSLRAGSRGPTLLEDFILREKITHFDHERIPERIVHARGSAAH
Chain 2:   21 SPLAAYEVDDSTGYLTSDVGGPI-QDQTSLKAGIRGPTLLEDFMFRQKIQHFDHERVPERAVHARGAGAH

Chain 1:  136 GYFQPYKSLSDITKADFLSDPNKITPVFVRFSTCQGGAGSADTVRDIRGFATKFYTEEGIFDLVGNNTPI
Chain 2:   90 GTFTSYADWSNITAASFLNATGKQTPVFVRFSTVAGSRGSADTARDVHGFATRFYTDEGNFDIVGNNIPV

Chain 1:  206 FFIQDAHKFPDFVHAVKPEPHWAIPQGQSAHDTFWDYVSLQPETLHNVMWAMSDRGIPRSYRTMEGFGIH
Chain 2:  160 FFIQDAIQFPDLIHSVKPRPDNEIPQAATAHDSAWDFFSQQPSTMHTLFWAMSGHGIPRSYRHMDGFGVH

Chain 1:  276 TFRLINAEGKATFVRFHWKPLAGKASLVWDEAQKLTGRDPDFHRRELWEAIEAGDFPEYELGFQLIPEED
Chain 2:  230 TFRFVKDDGSSKLIKWHFKSRQGKASLVWEEAQVLSGKNADFHRQDLWDAIESGNGPEWDVCVQIVDESQ

Chain 1:  346 EFKFDFDLLDPTKLIPEELVPVQRVGKMVLNRNPDNFFAENEQAAFHPGHIVPGLDFTNDPLLQGRLFSY
Chain 2:  300 AQAFGFDLLDPTKIIPEEYAPLTKLGLLKLDRNPTNYFAETEQVMFQPGHIVRGIDFTEDPLLQGRLFSY

Chain 1:  416 TDTQISRLGGPNFHEIPINRPTCPYHNFQRDGMHRMGIDTNPANYEPNSINDNWPRETPPGPKRGGFESY
Chain 2:  370 LDTQLNRNGGPNFEQLPINMPRVPIHNNNRDGAGQMFIHRNKYPYTPNTLNSGYPRQANQN-AGRGFFTA

Chain 1:  486 Q-ERVEGNKVRERSPSFGEYYSHPRLFWLSQTPFEQRHIVDGFSFELSKVVRPYIRERVVDQLAHIDLTL
Chain 2:  439 PGRTASGALVREVSPTFNDHWSQPRLFFNSLTPVEQQFLVNAMRFEISLVKSEEVKKNVLTQLNRVSHDV

Chain 1:  555 AQAVAKNLGIELTDDQLNITPPPDVNGLKKDPSLSLYAIPDG-DVKGRVVAILLND--EVRSADLLAILK
Chain 2:  509 AVRVAAAIGLGA---PDADDTYYH---NNKTAGVSIVGSGPLPTIKTLRVGILATTSESSALDQAAQLRT

Chain 1:  622 ALKAKGVHAKLLYSRMGEVTADDGTVLPIAATFAGAPSLTVDAVIVPCGNIADIAD------NGDANYYLM
Chain 2:  573 RLEKDGLVVTVVAETLR----------EGVDQTYSTADATGFDGVVVVDGAAALFASSPLFPTGRPLQIFV

Chain 1:  687 EAYKHLKPIALAGDARKFKATIKIADQGEEGIVEADSADGSFMDELLTLMAAHRVWSRIPK
Chain 2:  637 DAYRWGKPVGVCG--SEVLDAADVP-EDGDGVYSE-ESVDMFVEEFEKGLATFRFTDRFAL

Note: positions are from PDB; the numbers between alignments are block index
```

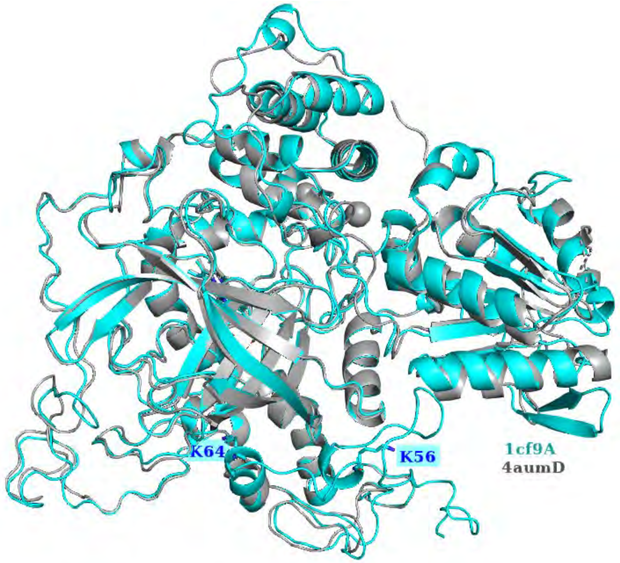

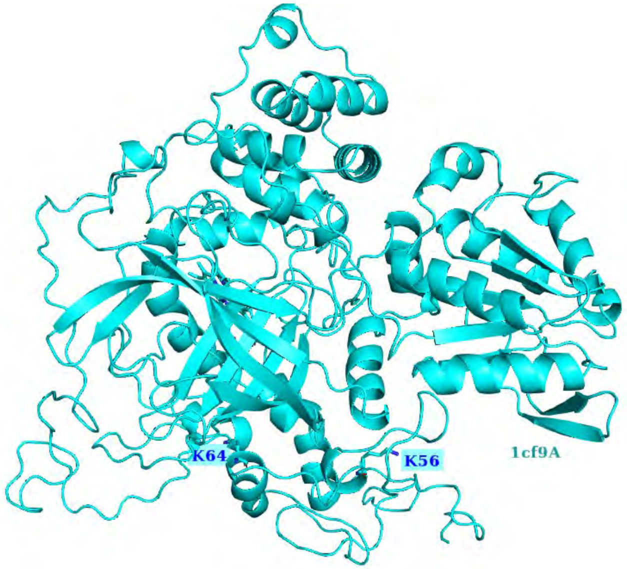

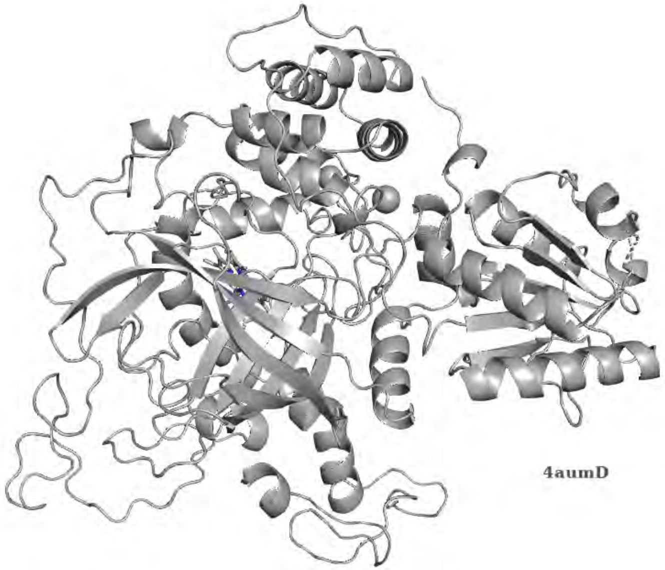

UniProt ID: M4GGR7

PDB ID: 5XVZ_B

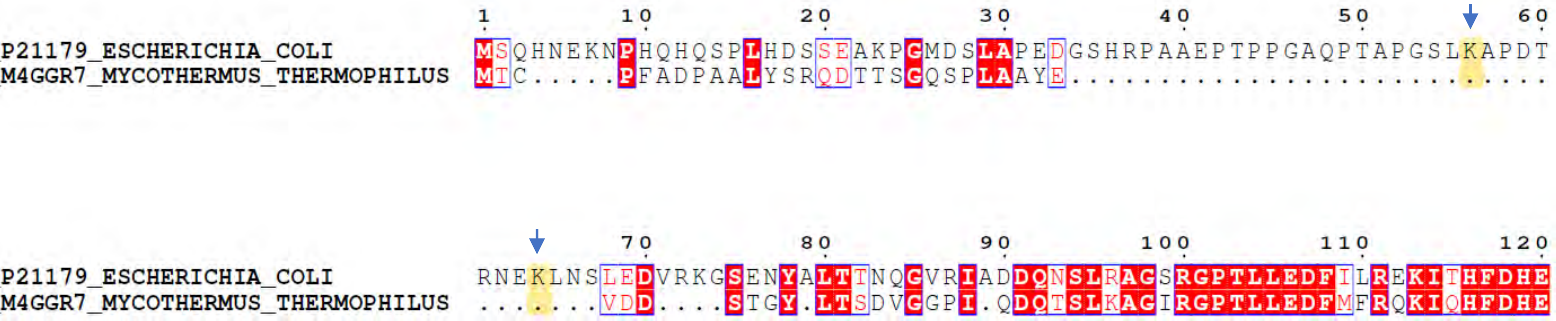

Full sequences in supplemental file.

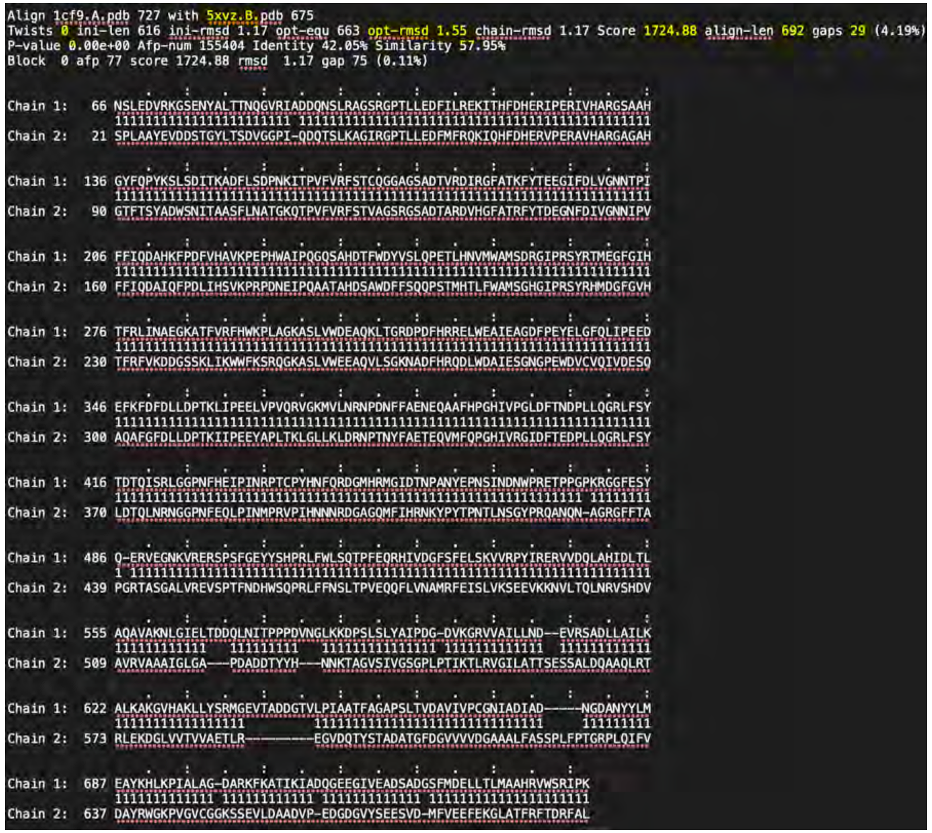

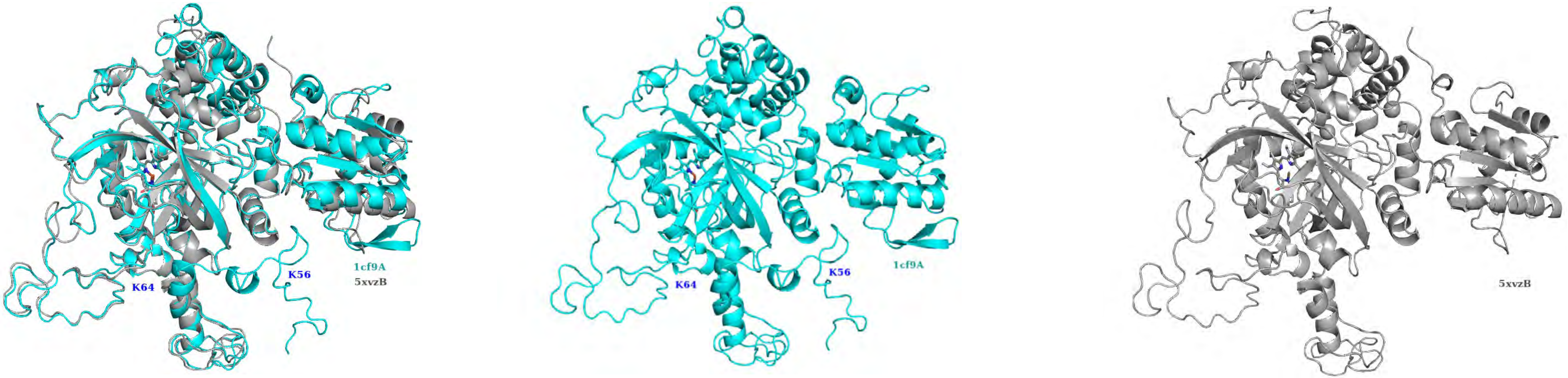

UniProt ID: M4GGR7

PDB ID: 5XY4_B

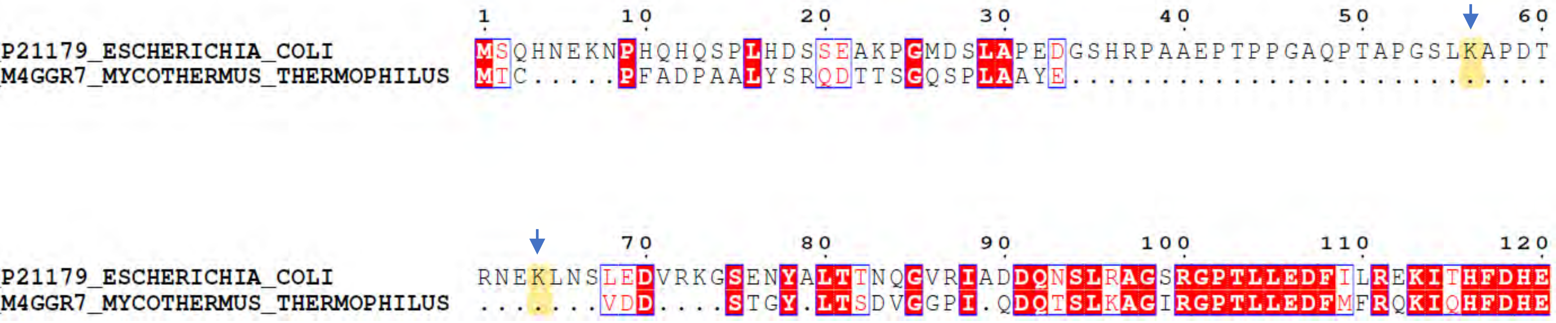

Full sequences in supplemental file.

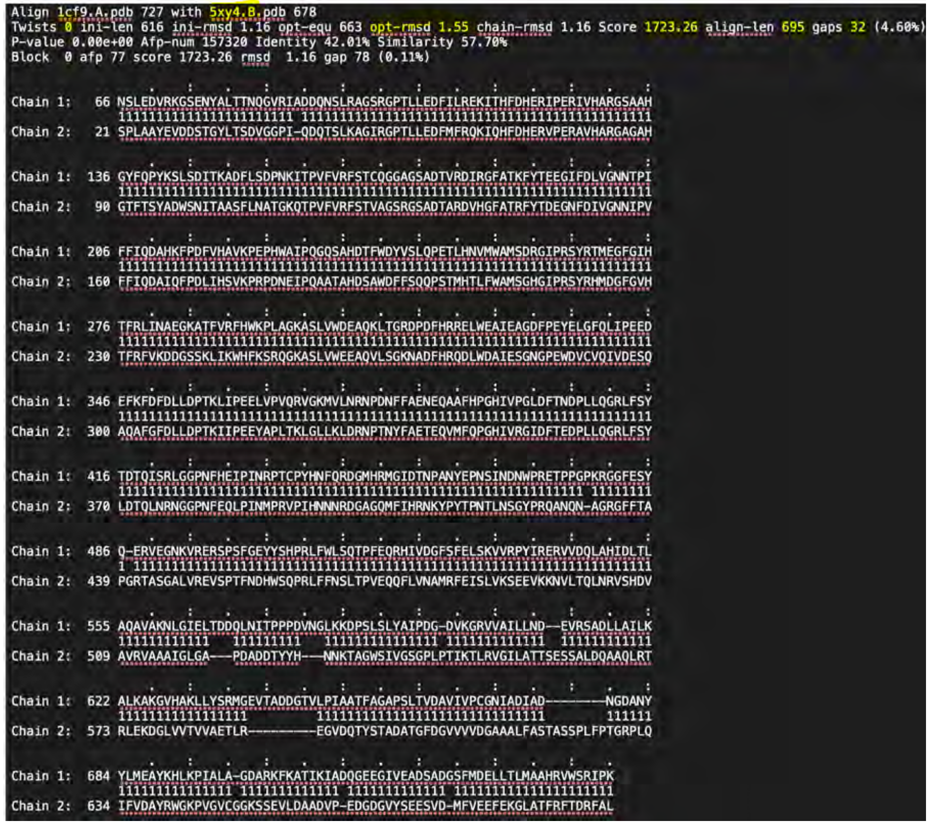

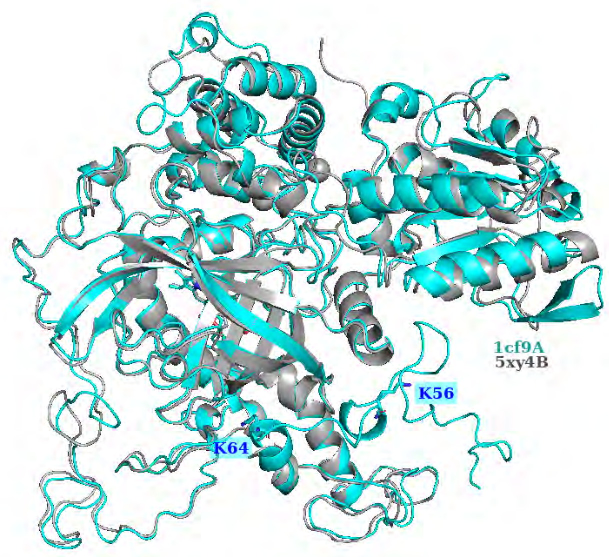

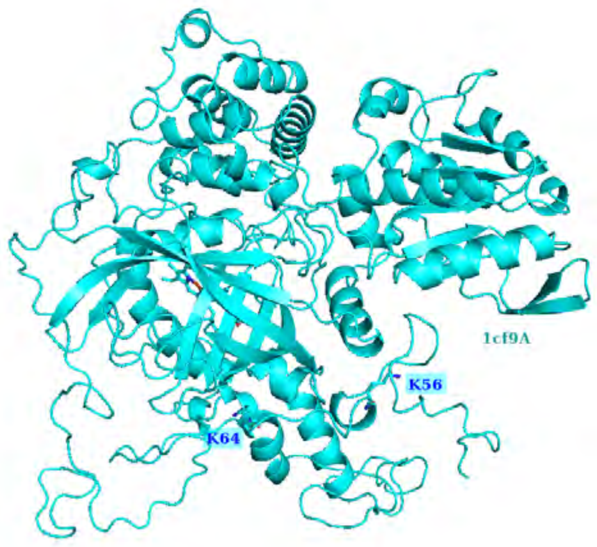

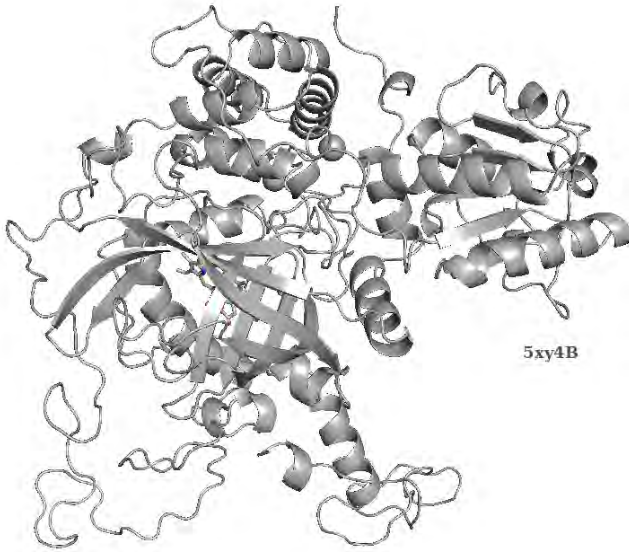

UniProt ID: M4GGR7
PDB ID: 5XZM_B

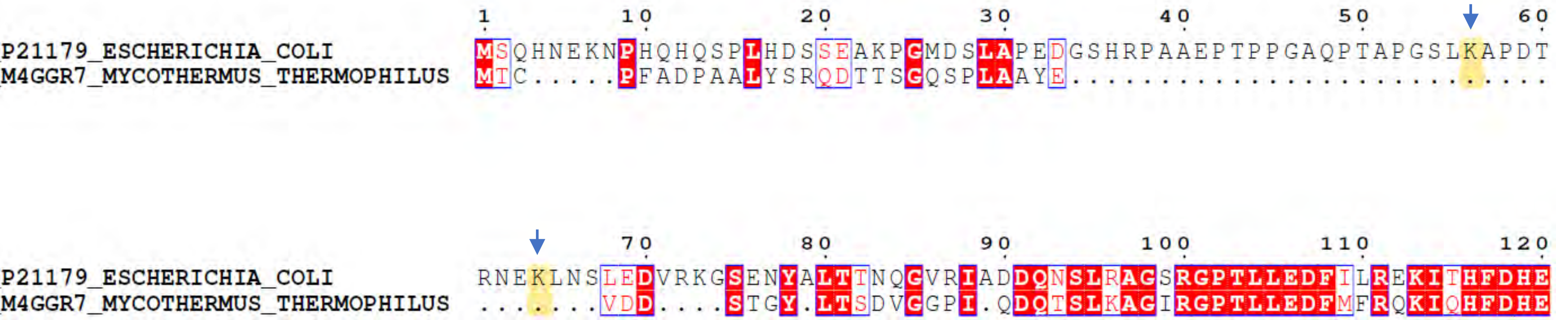

Full sequences in supplemental file.

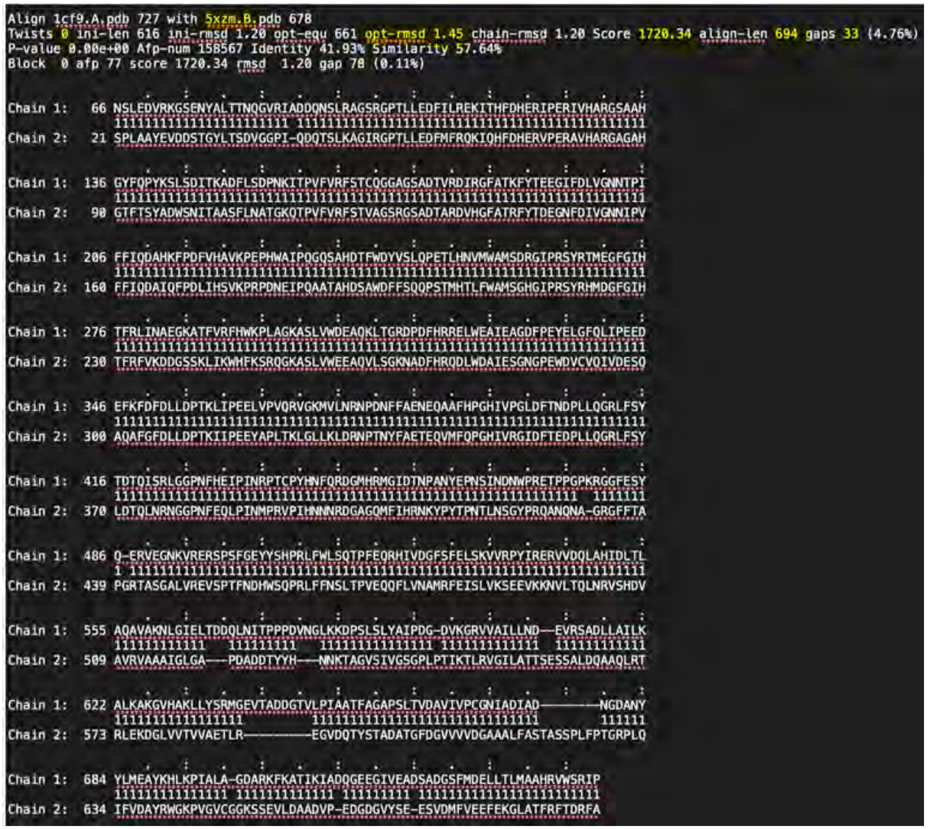

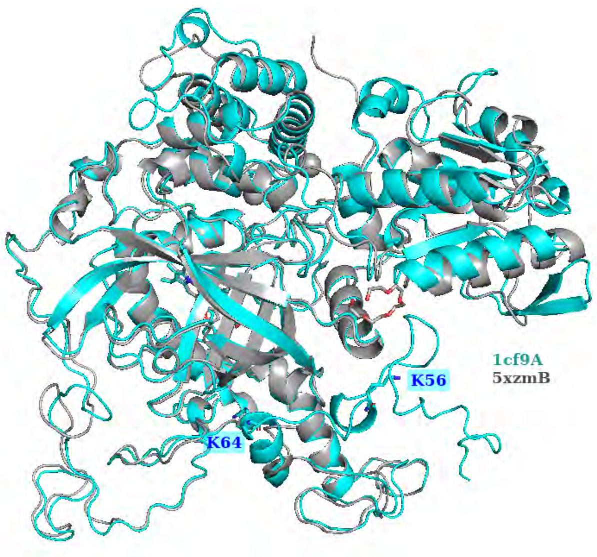

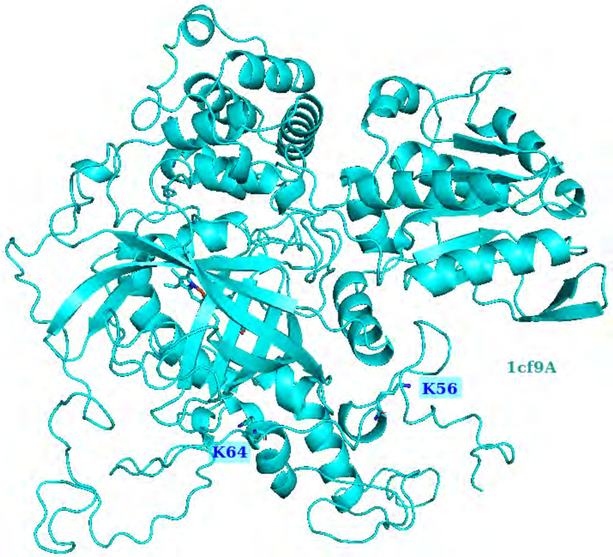

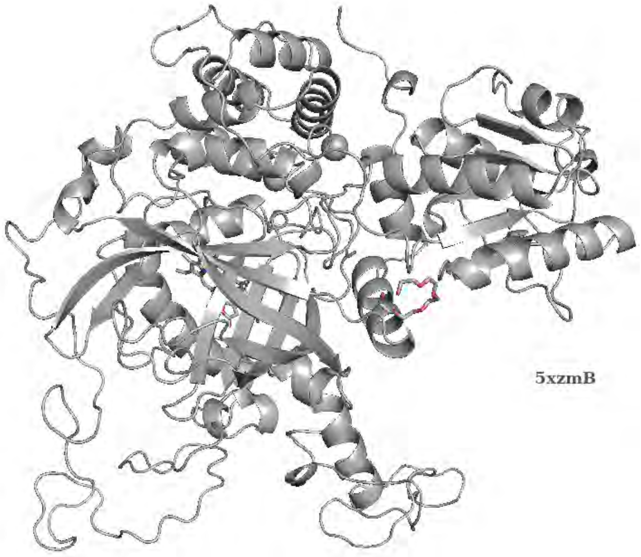

UniProt ID: M4GGR7

PDB ID: 5XZN_B

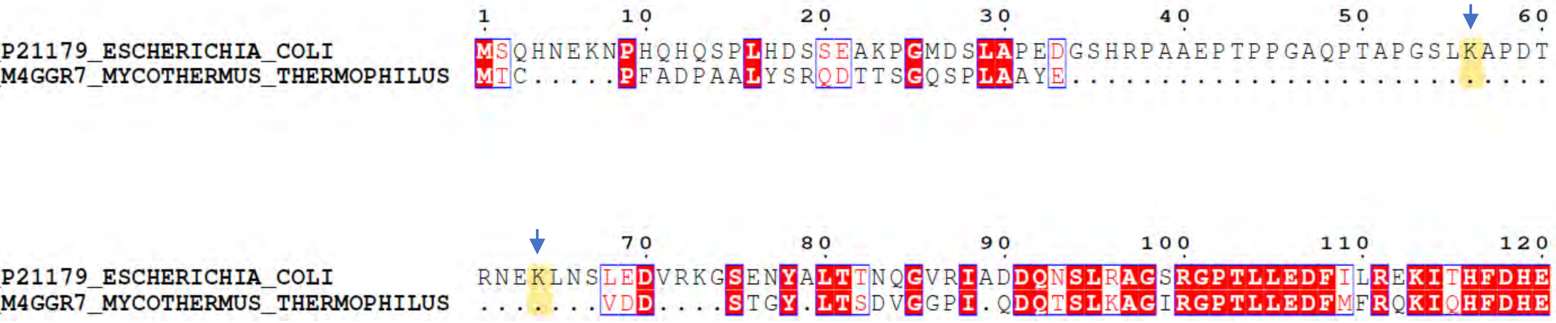

Full sequences in supplemental file.

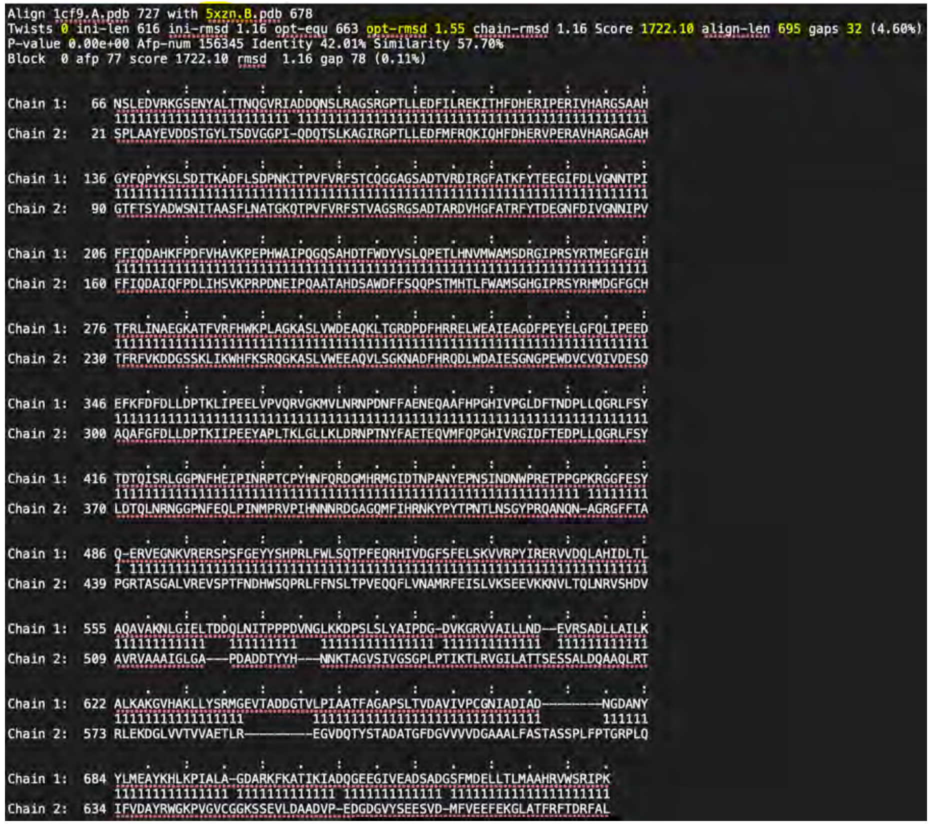

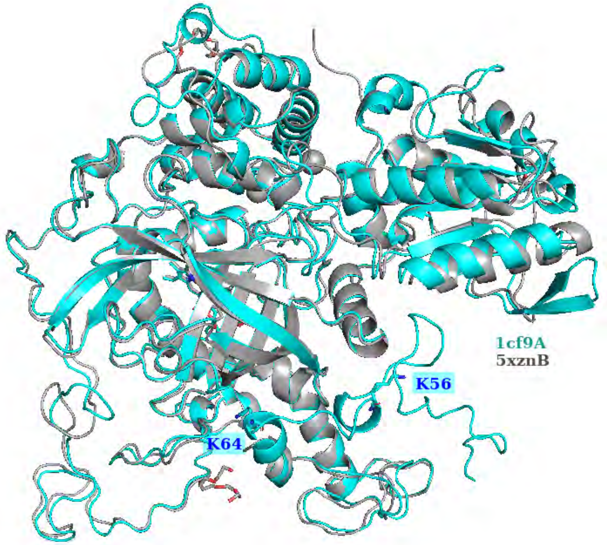

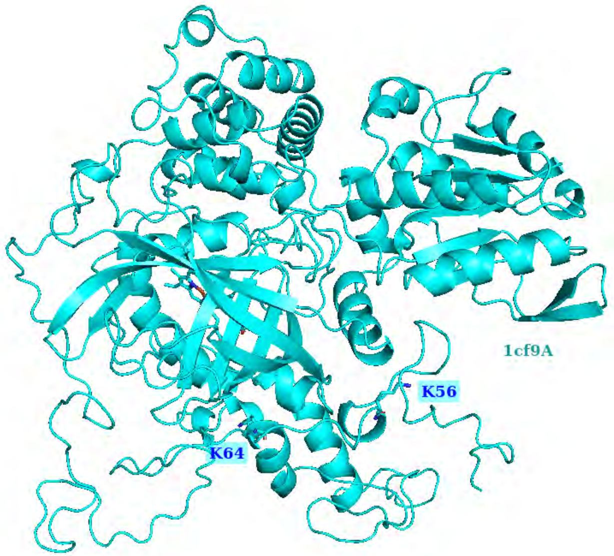

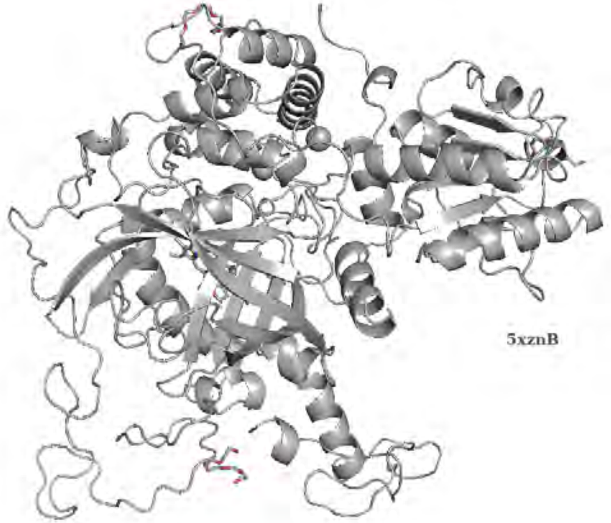

UniProt ID: M4GGR7

PDB ID: 5Y17_D

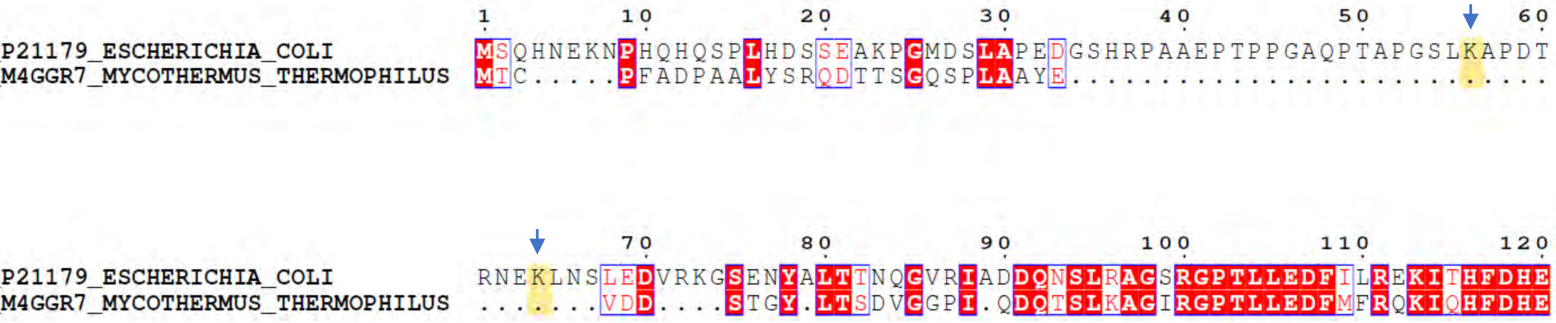

Full sequences in supplemental file.

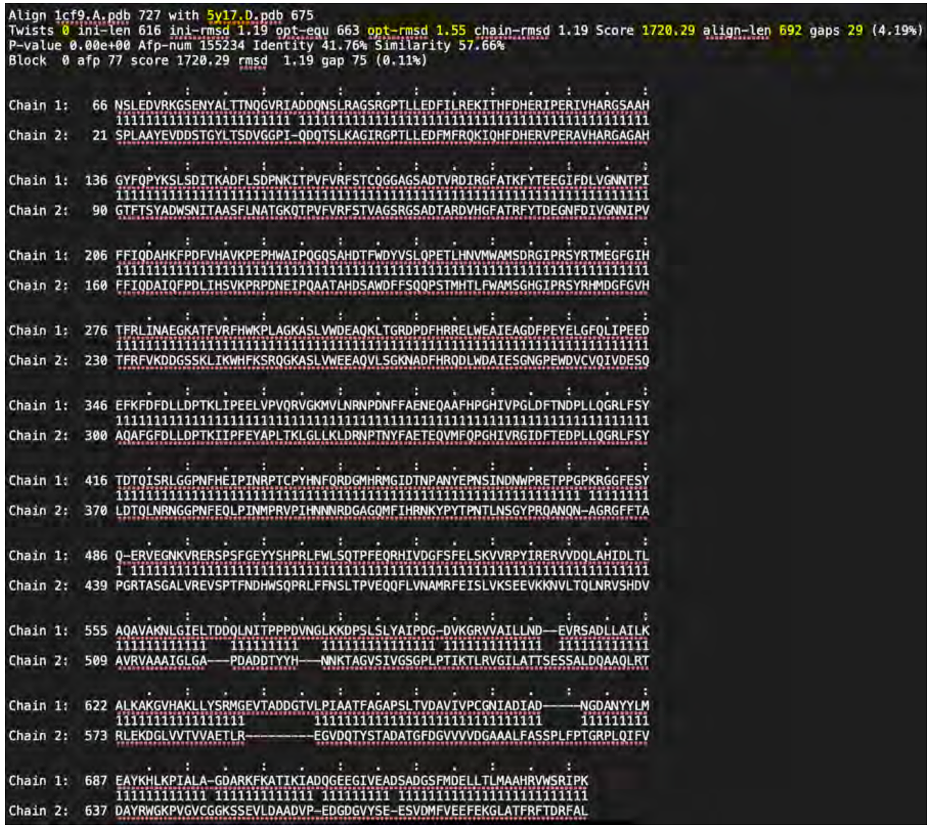

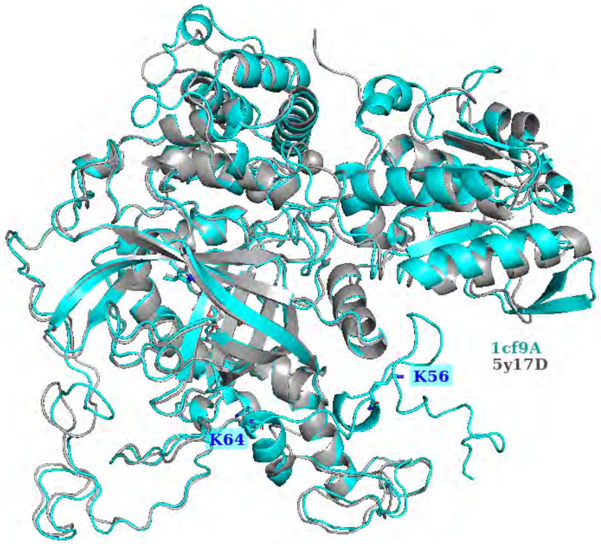

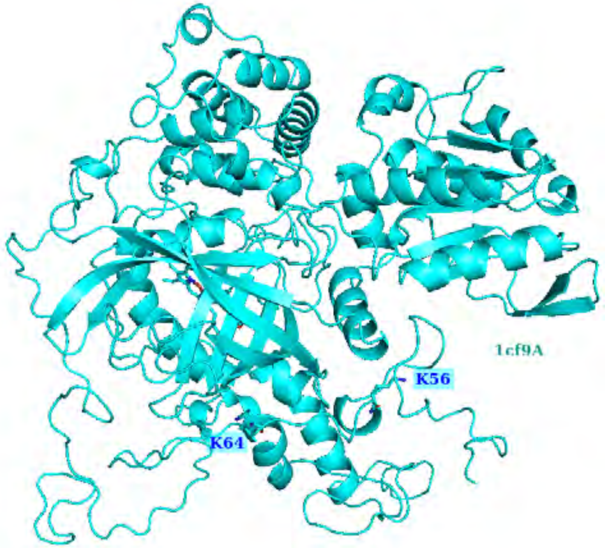

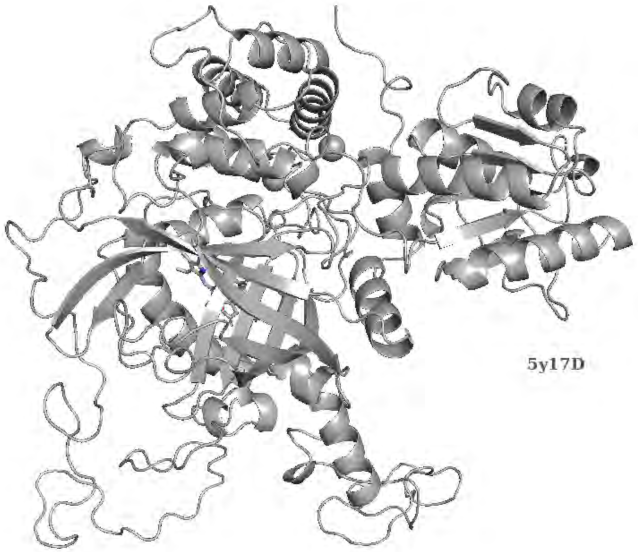

UniProt ID: M4GGR7
PDB ID: 5ZZ1_B

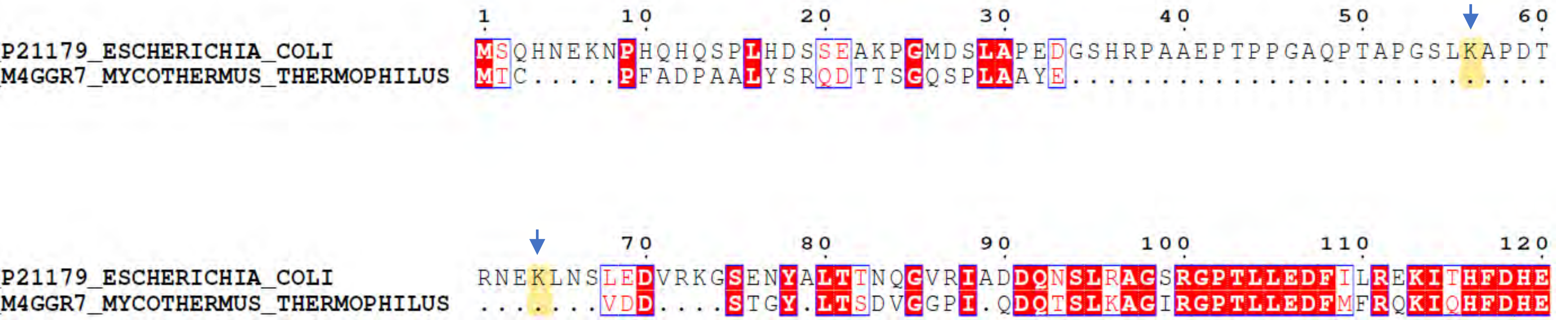

Full sequences in supplemental file.

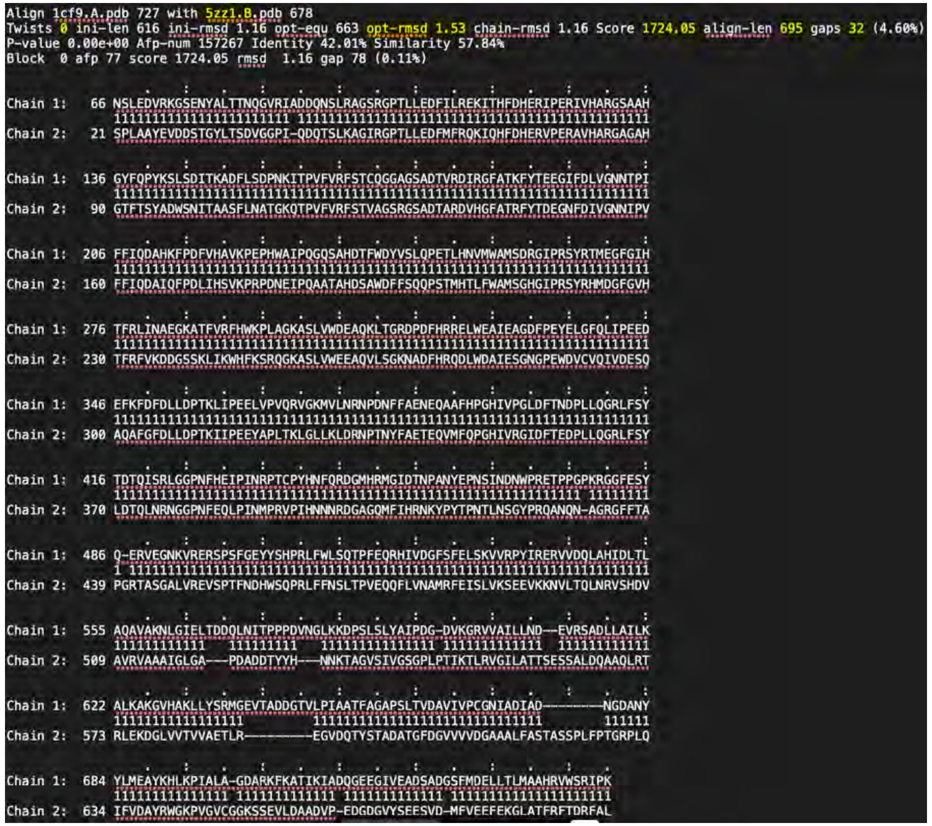

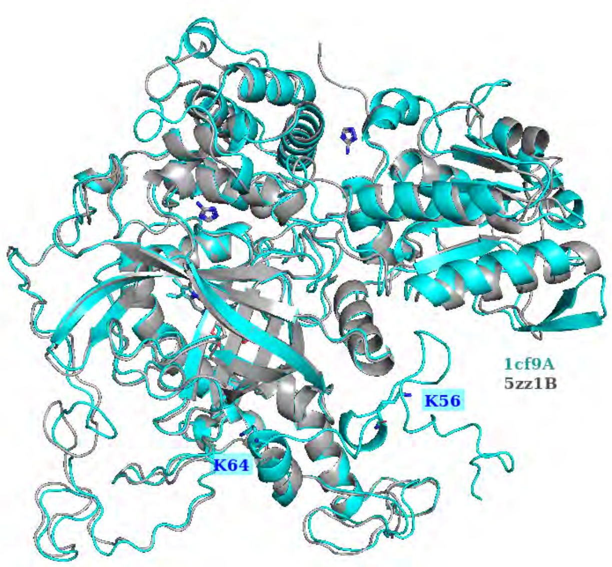

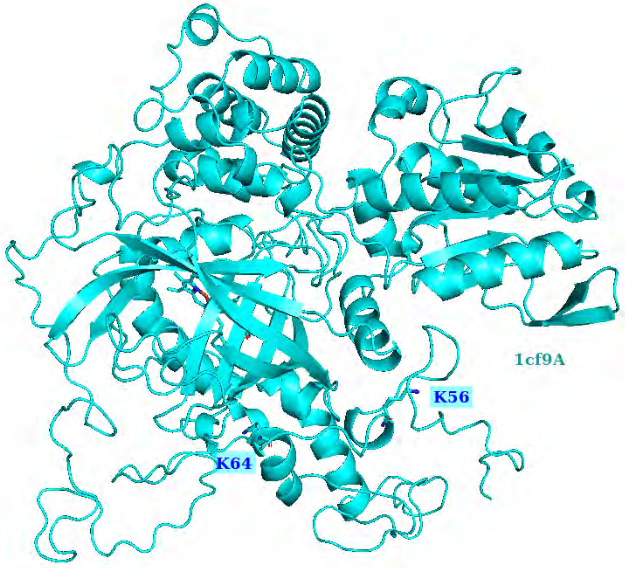

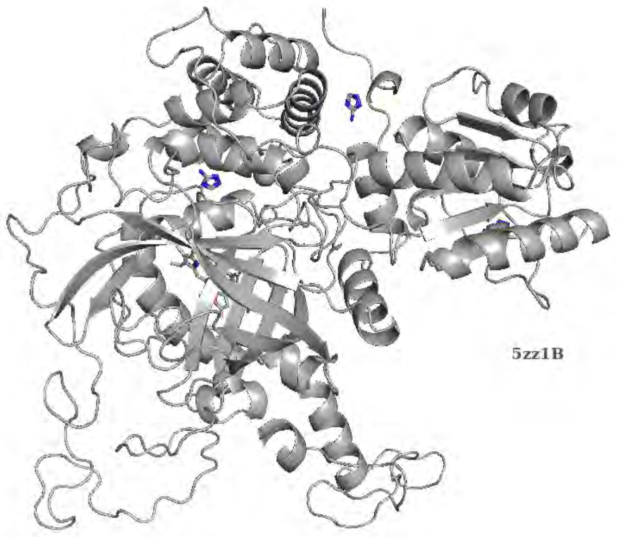

UniProt ID: M4GGR8

PDB ID: 4AUN_A

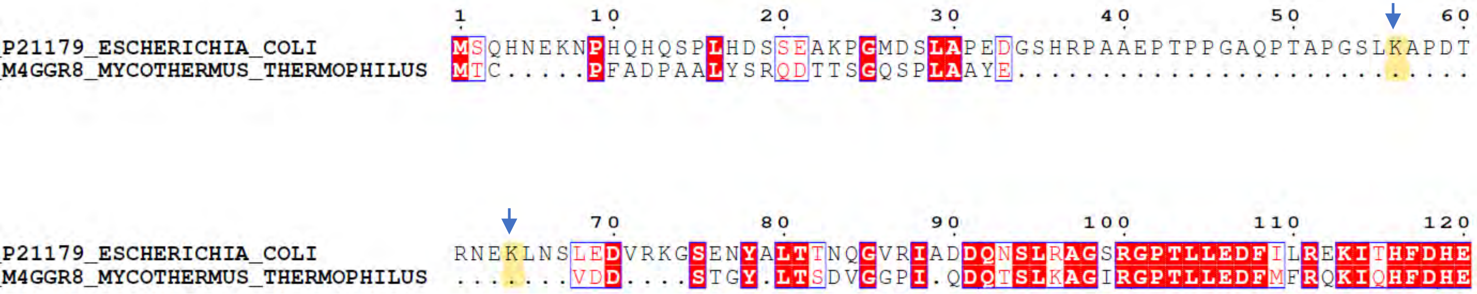

Full sequences in supplemental file.

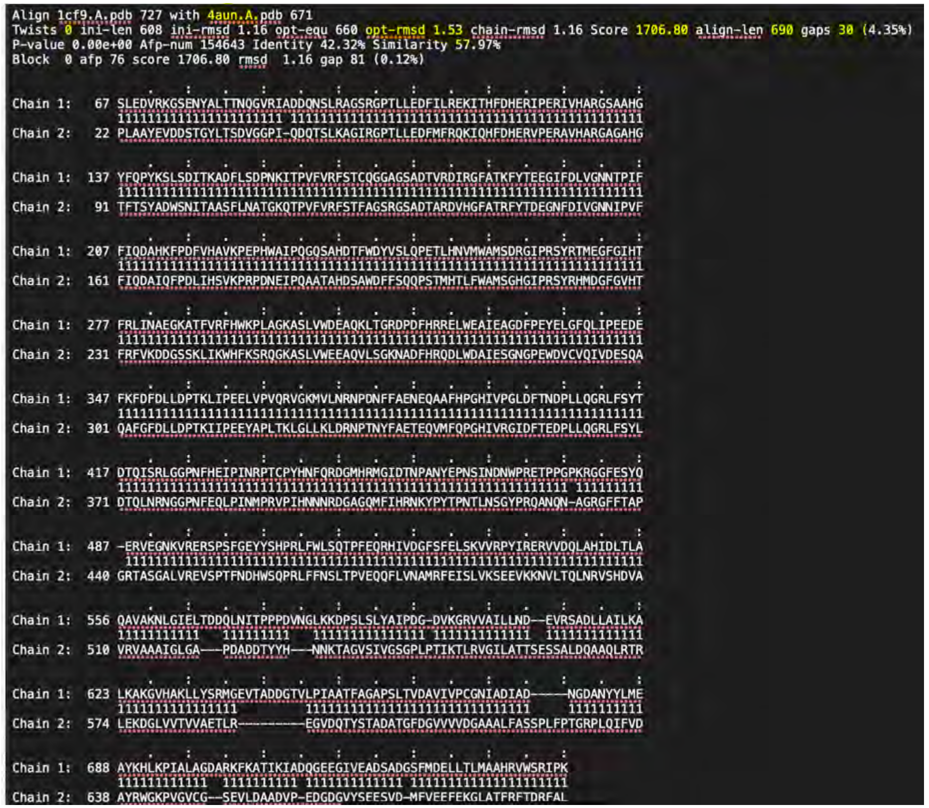

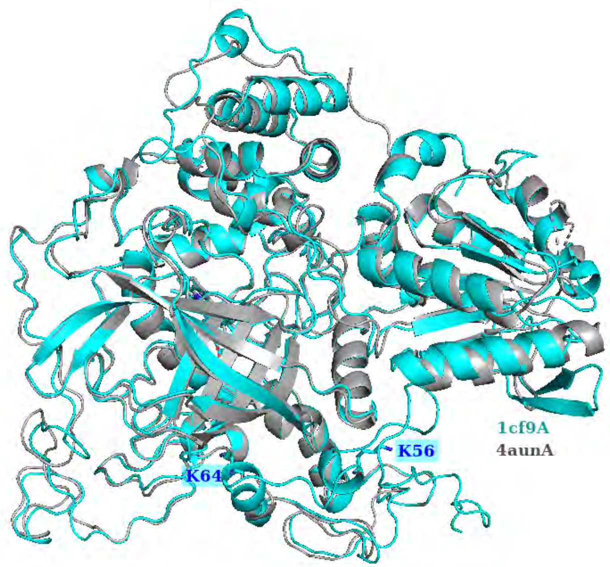

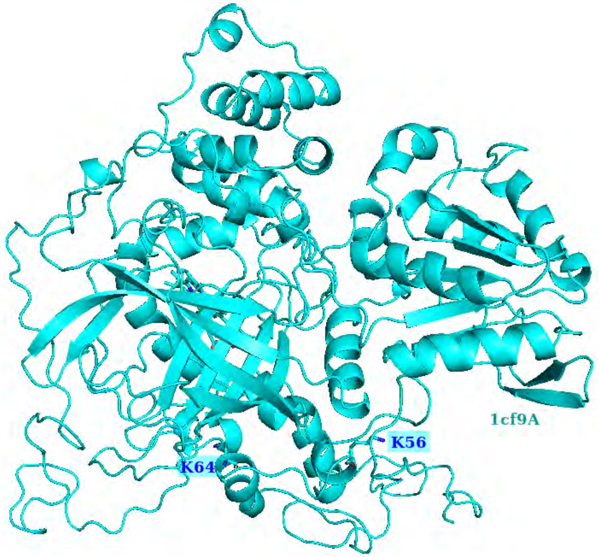

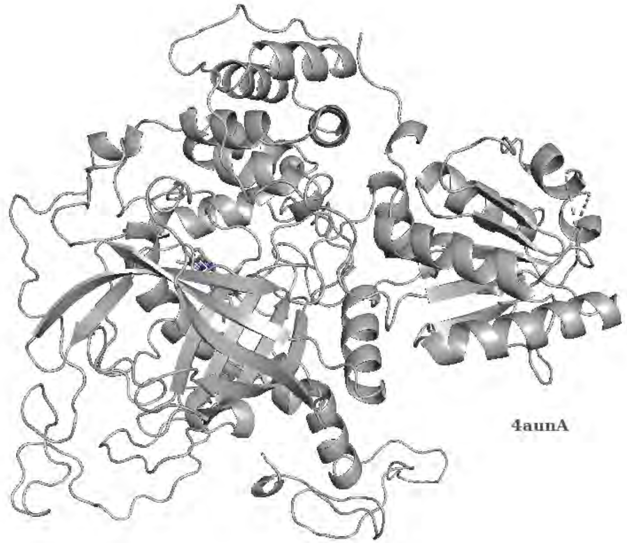

UniProt ID: none
PDB ID: 4QOL_B

```
                         1        10        20        30        40        50        60
P21179_ESCHERICHIA_COLI  MSQHNEKNPHQHQSPLHDSSEAKPGMDSLAPEDGSHRPAAEPTPPGAQPTAPGSLKAPDT
BACILLUS_PUMILUS         ............................................................

                                   70        80        90       100       110       120
P21179_ESCHERICHIA_COLI  RNEKLNSLEDVRKGSENYALTTNQGVRIADDQNSLRAGSRGPTLLEDFILREKITHFDHE
BACILLUS_PUMILUS         ...........MTNSNHKNLTTNQGVPVGDNQNSRTAGHRGPSFLDDYHLIEKLAHFDRE
```

Full sequences in supplemental file.

```
Align 1cf9.A.pdb 727 with 4qol.B.pdb 480
Twists 0 ini-len 464 ini-rmsd 1.17 opt-equ 479 opt-rmsd 1.23 chain-rmsd 1.17 Score 1307.51 align-len 494 gaps 15 (3.04%)
P-value 0.00e+00 Afp-num 113981 Identity 43.32% Similarity 60.53%
Block  0 afp 58 score 1307.51 rmsd  1.17 gap 27 (0.05%)

                  .    :    .    :    .    :    .    :    .    :    .    :    .    :
Chain 1:   77 NYALTTNQGVRIADDQNSLRAGSRGPTLLEDFILREKITHFDHERIPERIVHARGSAAHGYFQPYKSLSD
              1111111111111111111111111111111111111111111111111111111111111111111111
Chain 2:    6 HKNLTTNQGVPVGDNQNSRTAGHRGPSFLDDYHLIEKLAHFDRERIPERVVHARGAGAYGVFEVENSMEK

                  .    :    .    :    .    :    .    :    .    :    .    :    .    :
Chain 1:  147 ITKADFLSDPNKITPVFVRFSTCQGGAGSADTVRDIRGFATKFYTEEGIFDLVGNNTPIFFIQDAHKFPD
              1111111111111111111111111111111111111111111111111111111111111111111111
Chain 2:   76 HTRAAFLSEEGKQTDVFVRFSTVIHPKGSPETLRDPRGFAVKFYTEEGNYDLVGNNLPIFFIRDALKFPD

                  .    :    .    :    .    :    .    :    .    :    .    :    .    :
Chain 1:  217 FVHAVKPEPHWAIPQGQSAHDTFWDYVSLQPETLHNVMWAMSDRGIPRSYRTMEGFGIHTFRLINAEGKA
              11111111111111    111111111111111111111111111111111111111111111111111
Chain 2:  146 MVHSLKPDPVTNIQ----DPDRYWDFMTLTPESTHMLTWLFSDEGIPANYAEMRGSGVHTFRWVNKYGET

                  .    :    .    :    .    :    .    :    .    :    .    :    .    :
Chain 1:  287 TFVRFHWKPLAGKASLVWDEAQKLTGRDPDFHRRELWEAIEAGDFPEYELGFQLIPEEDEFKFDFDLLDP
              1111111111111111111111111111111111111111111111111111111111111111111111
Chain 2:  212 KYVKYHWRPSEGIRNLSMEEAAEIQANDFQHATRDLYDRIEKGNYPAWDLYVQLMPLSDYDELDYDPCDP

                  .    :    .    :    .    :    .    :    .    :    .    :    .    :
Chain 1:  357 TKLIPEELVPVQRVGKMVLNRNPDNFFAENEQAAFHPGHIVPGLDFTNDPLLQGRLFSYTDTQISRLGGP
              1111111111111111111111111111111111111111111111111111111111111111111 11
Chain 2:  282 TKTWSEEDYPLQKVGRMTLNRNPENFFAETEQAAFTPSALVPGIEASEDKLLQGRLFSYPDTQRHRL-GA

                  .    :    .    :    .    :    .    :    .    :    .    :    .    :
Chain 1:  427 NFHEIPINRPTCPYHNFQRDGMHRMGIDTNPANYEPNSINDNWPRETPPGPKRGGFESYQERV-EGNKVR
              111111111111111111111111111111111111111 1111111111      11111111 111111
Chain 2:  351 NYMRIPVNCPYAPVHNNQQDGFMTTTRPSGHINYEPNR-YDDQPKENPH------YKESEPVLHGDRMVR

                  .    :    .    :    .    :    .    :    .    :    .    :    .    :
Chain 1:  496 ERSPSFGEYYSHPRLFWLSQTPFEQRHIVDGFSFELSKVVRPYIRERVVDQLAHIDLTLAQAVAKNLGIE
              111111 11111111111111111111111111111111 11111111111111111111111111111111
Chain 2:  414 QKIEKP-NDFKQAGEKYRSYSEEEKQALIKNLTADLKGV-NEKTKLLAICNFYRADEDYGQRLADSLGVD

Chain 1:  566 LTDD
              1111
Chain 2:  482 IRSY
```

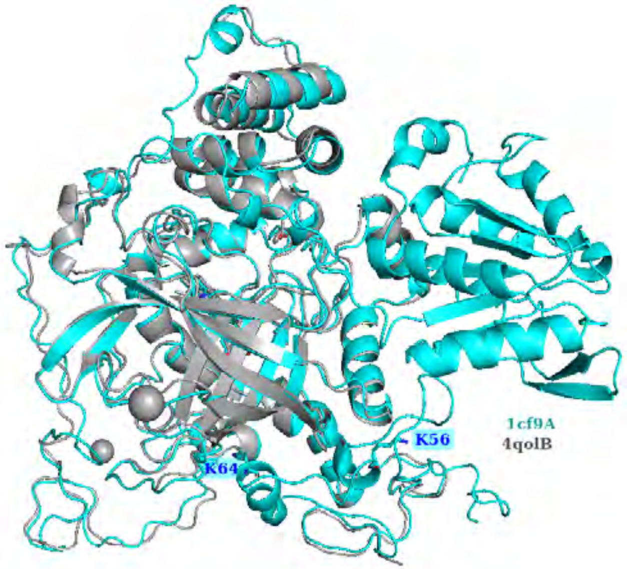

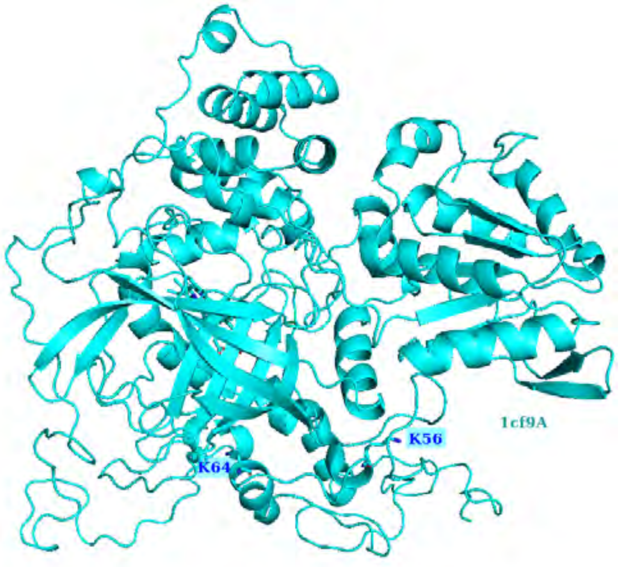

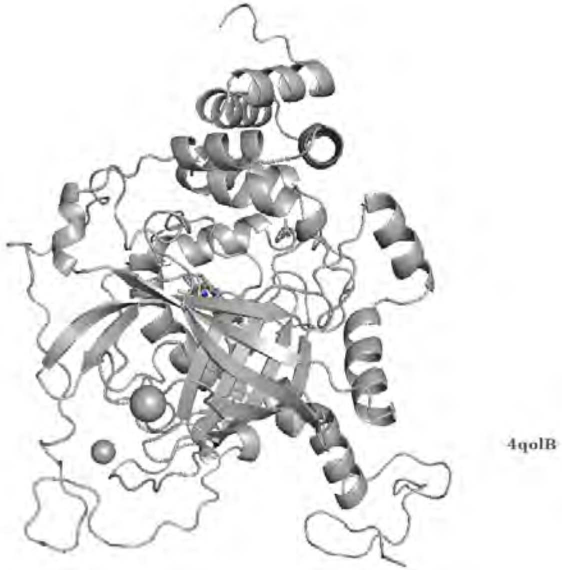

UniProt ID: none
PDB ID: 4QOM_D

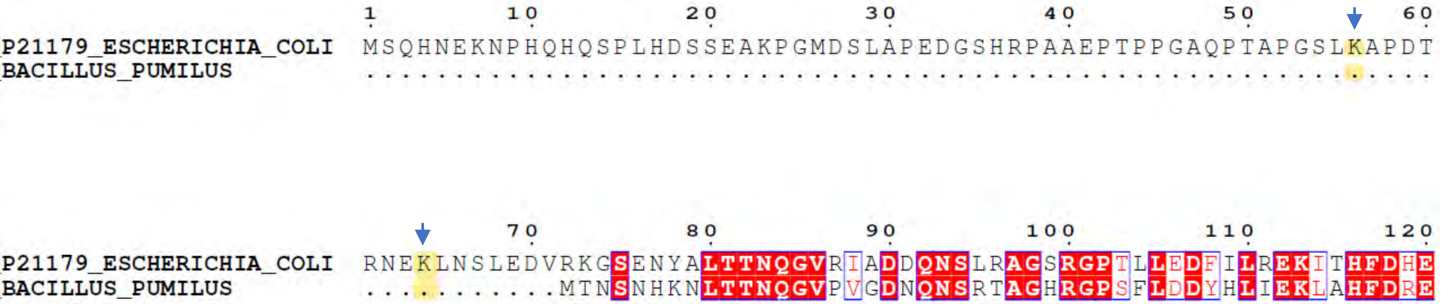

Full sequences in supplemental file.

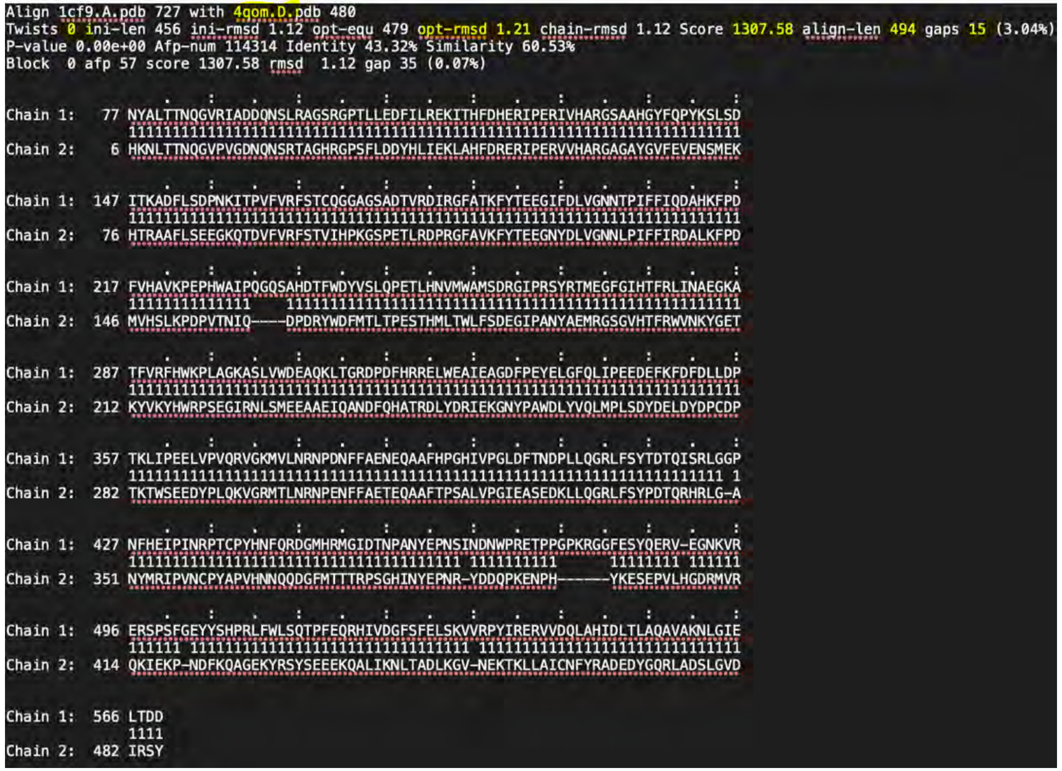

```
Align 1cf9.A.pdb 727 with 4qom.D.pdb 480
Twists 0 ini-len 456 ini-rmsd 1.12 opt-equ 479 opt-rmsd 1.21 chain-rmsd 1.12 Score 1307.58 align-len 494 gaps 15 (3.04%)
P-value 0.00e+00 Afp-num 114314 Identity 43.32% Similarity 60.53%
Block  0 afp 57 score 1307.58 rmsd  1.12 gap 35 (0.07%)

Chain 1:   77 NYALTTNQGVRIADDQNSLRAGSRGPTLLEDFILREKITHFDHERIPERIVHARGSAAHGYFQPYKSLSD
              1111111111111111111111111111111111111111111111111111111111111111111111
Chain 2:    6 HKNLTTNQGVPVGDNQNSRTAGHRGPSFLDDYHLIEKLAHFDRERIPERVVHARGAGAYGVFEVENSMEK

Chain 1:  147 ITKADFLSDPNKITPVFVRFSTCQGGAGSADTVRDIRGFATKFYTEEGIFDLVGNNTPIFFIQDAHKFPD
              1111111111111111111111111111111111111111111111111111111111111111111111
Chain 2:   76 HTRAAFLSEEGKQTDVFVRFSTVIHPKGSPETLRDPRGFAVKFYTEEGNYDLVGNNLPIFFIRDALKFPD

Chain 1:  217 FVHAVKPEPHWAIPQGQSAHDTFWDYVSLQPETLHNVMWAMSDRGIPRSYRTMEGFGIHTFRLINAEGKA
              11111111111111    1111111111111111111111111111111111111111111111111111
Chain 2:  146 MVHSLKPDPVTNIQ----DPDRYWDFMTLTPESTHMLTWLFSDEGIPANYAEMRGSGVHTFRWVNKYGET

Chain 1:  287 TFVRFHWKPLAGKASLVWDEAQKLTGRDPDFHRRELWEAIEAGDFPEYELGFQLIPEEDEFKFDFDLLDP
              1111111111111111111111111111111111111111111111111111111111111111111111
Chain 2:  212 KYVKYHWRPSEGIRNLSMEEAAEIQANDFQHATRDLYDRIEKGNYPAWDLYVQLMPLSDYDELDYDPCDP

Chain 1:  357 TKLIPEELVPVQRVGKMVLNRNPDNFFAENEQAAFHPGHIVPGLDFTNDPLLQGRLFSYTDTQISRLGGP
              111111111111111111111111111111111111111111111111111111111111111111111 1
Chain 2:  282 TKTWSEEDYPLQKVGRMTLNRNPENFFAETEQAAFTPSALVPGIEASEDKLLQGRLFSYPDTQRHRLG-A

Chain 1:  427 NFHEIPINRPTCPYHNFQRDGMHRMGIDTNPANYEPNSINDNWPRETPPGPKRGGFESYQERV-EGNKVR
              11111111111111111111111111111111111111 1111111111      11111111 111111
Chain 2:  351 NYMRIPVNCPYAPVHNNQQDGFMTTTRPSGHINYEPNR-YDDQPKENPH------YKESEPVLHGDRMVR

Chain 1:  496 ERSPSFGEYYSHPRLFWLSQTPFEQRHIVDGFSFELSKVVRPYIRERVVDQLAHIDLTLAQAVAKNLGIE
              111111 111111111111111111111111111111111 111111111111111111111111111111
Chain 2:  414 QKIEKP-NDFKQAGEKYRSYSEEEKQALIKNLTADLKGV-NEKTKLLAICNFYRADEDYGQRLADSLGVD

Chain 1:  566 LTDD
              1111
Chain 2:  482 IRSY
```

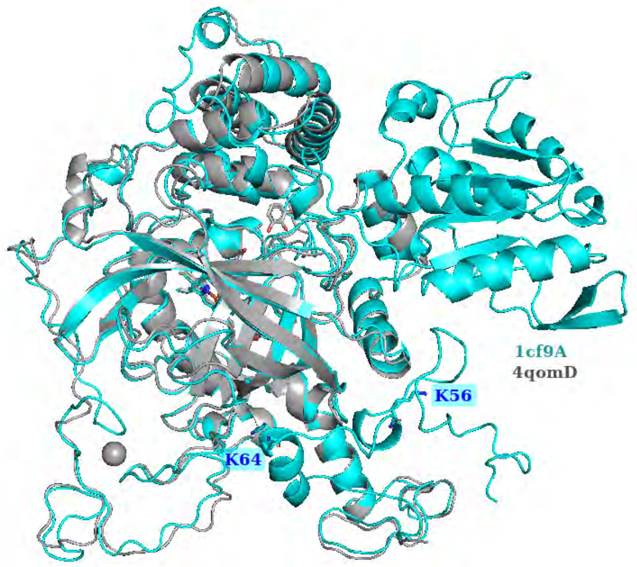

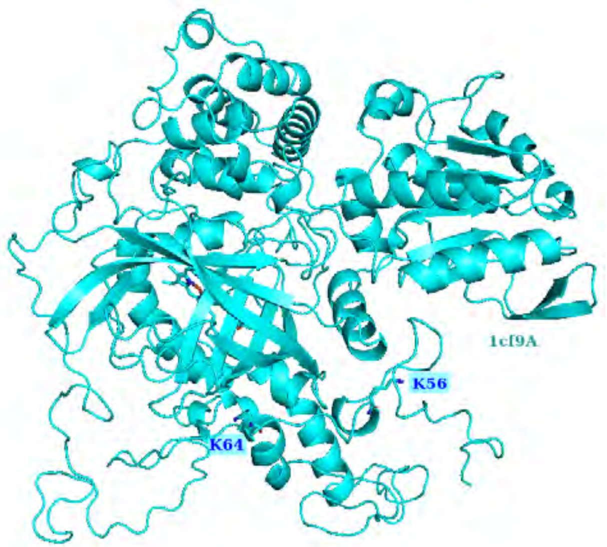

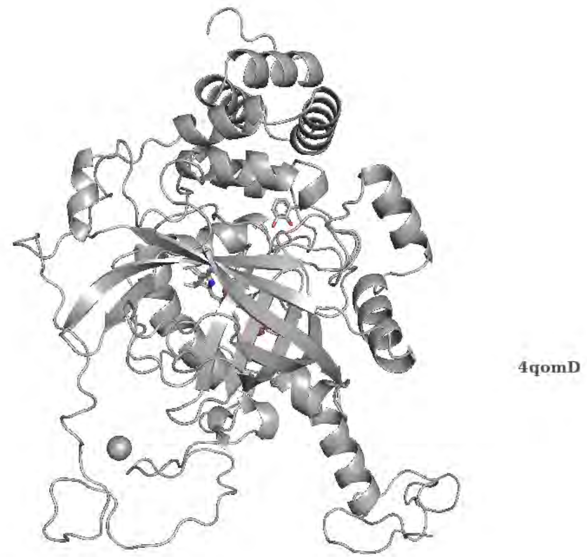

UniProt ID: none
PDB ID: 4QON_D

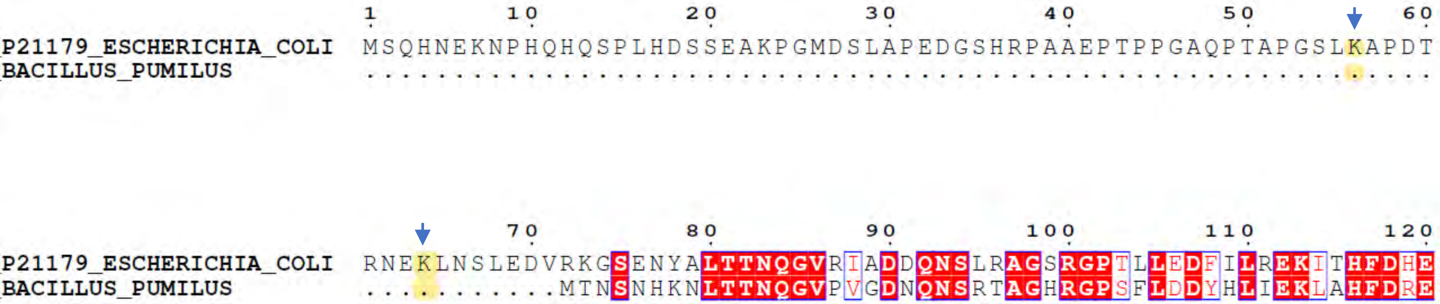

Full sequences in supplemental file.

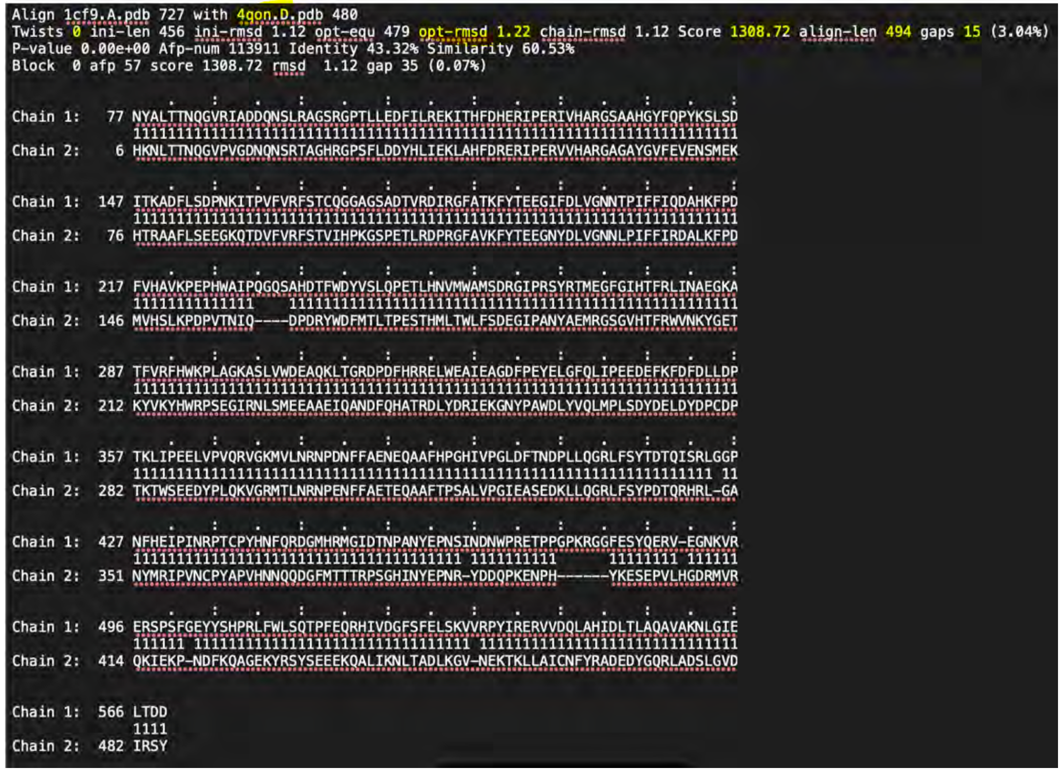

```
Align 1cf9.A.pdb 727 with 4qon.D.pdb 480
Twists 0 ini-len 456 ini-rmsd 1.12 opt-equ 479 opt-rmsd 1.22 chain-rmsd 1.12 Score 1308.72 align-len 494 gaps 15 (3.04%)
P-value 0.00e+00 Afp-num 113911 Identity 43.32% Similarity 60.53%
Block  0 afp 57 score 1308.72 rmsd  1.12 gap 35 (0.07%)

                  .    :    .    :    .    :    .    :    .    :    .    :    .    :
Chain 1:   77 NYALTTNQGVRIADDQNSLRAGSRGPTLLEDFILREKITHFDHERIPERIVHARGSAAHGYFQPYKSLSD
              1111111111111111111111111111111111111111111111111111111111111111111111
Chain 2:    6 HKNLTTNQGVPVGDNQNSRTAGHRGPSFLDDYHLIEKLAHFDRERIPERVVHARGAGAYGVFEVENSMEK

                  .    :    .    :    .    :    .    :    .    :    .    :    .    :
Chain 1:  147 ITKADFLSDPNKITPVFVRFSTCQGGAGSADTVRDIRGFATKFYTEEGIFDLVGNNTPIFFIQDAHKFPD
              1111111111111111111111111111111111111111111111111111111111111111111111
Chain 2:   76 HTRAAFLSEEGKQTDVFVRFSTVIHPKGSPETLRDPRGFAVKFYTEEGNYDLVGNNLPIFFIRDALKFPD

                  .    :    .    :    .    :    .    :    .    :    .    :    .    :
Chain 1:  217 FVHAVKPEPHWAIPQGQSAHDTFWDYVSLQPETLHNVMWAMSDRGIPRSYRTMEGFGIHTFRLINAEGKA
              11111111111111    1111111111111111111111111111111111111111111111111111
Chain 2:  146 MVHSLKPDPVTNIQ----DPDRYWDFMTLTPESTHMLTWLFSDEGIPANYAEMRGSGVHTFRWVNKYGET

                  .    :    .    :    .    :    .    :    .    :    .    :    .    :
Chain 1:  287 TFVRFHWKPLAGKASLVWDEAQKLTGRDPDFHRRELWEAIEAGDFPEYELGFQLIPEEDEFKFDFDLLDP
              1111111111111111111111111111111111111111111111111111111111111111111111
Chain 2:  212 KYVKYHWRPSEGIRNLSMEEAAEIQANDFQHATRDLYDRIEKGNYPAWDLYVQLMPLSDYDELDYDPCDP

                  .    :    .    :    .    :    .    :    .    :    .    :    .    :
Chain 1:  357 TKLIPEELVPVQRVGKMVLNRNPDNFFAENEQAAFHPGHIVPGLDFTNDPLLQGRLFSYTDTQISRLGGP
              1111111111111111111111111111111111111111111111111111111111111111111 11
Chain 2:  282 TKTWSEEDYPLQKVGRMTLNRNPENFFAETEQAAFTPSALVPGIEASEDKLLQGRLFSYPDTQRHRL-GA

                  .    :    .    :    .    :    .    :    .    :    .    :    .    :
Chain 1:  427 NFHEIPINRPTCPYHNFQRDGMHRMGIDTNPANYEPNSINDNWPRETPPGPKRGGFESYQERV-EGNKVR
              11111111111111111111111111111111111111 1111111111       1111111 111111
Chain 2:  351 NYMRIPVNCPYAPVHNNQQDGFMTTTRPSGHINYEPNR-YDDQPKENPH-------YKESEPVLHGDRMVR

                  .    :    .    :    .    :    .    :    .    :    .    :    .    :
Chain 1:  496 ERSPSFGEYYSHPRLFWLSQTPFEQRHIVDGFSFELSKVVRPYIRERVVDQLAHIDLTLAQAVAKNLGIE
              111111 11111111111111111111111111111111 111111111111111111111111111111
Chain 2:  414 QKIEKP-NDFKQAGEKYRSYSEEEKQALIKNLTADLKGV-NEKTKLLAICNFYRADEDYGQRLADSLGVD

Chain 1:  566 LTDD
              1111
Chain 2:  482 IRSY
```

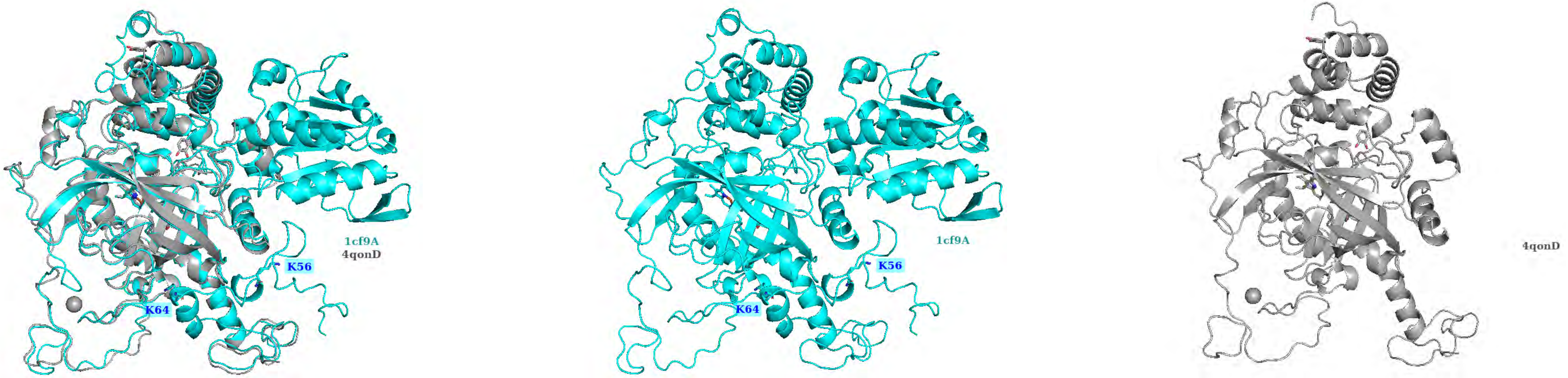

UniProt ID: none
PDB ID: 4QOO_D

```
                      1        10        20        30        40        50        60
P21179_ESCHERICHIA_COLI MSQHNEKNPHQHQSPLHDSSEAKPGMDSLAPEDGSHRPAAEPTPPGAQPTAPGSLKAPDT
BACILLUS_PUMILUS        ............................................................

                              70        80        90       100       110       120
P21179_ESCHERICHIA_COLI RNEKLNSLEDVRKGSENYALTTNQGVRIADDQNSLRAGSRGPTLLEDFILREKITHFDHE
BACILLUS_PUMILUS        ...........MTNSNHKNLTTNQGVPVGDNQNSRTAGHRGPSFLDDYHLIEKLAHFDRE
```

Full sequences in supplemental file.

```
Align 1cf9.A.pdb 727 with 4qoo.D.pdb 480
Twists 0 ini-len 456 ini-rmsd 1.12 opt-equ 479 opt-rmsd 1.20 chain-rmsd 1.12 Score 1308.74 align-len 494 gaps 15 (3.04%)
P-value 0.00e+00 Afp-num 114300 Identity 43.32% Similarity 60.53%
Block  0 afp 57 score 1308.74 rmsd  1.12 gap 35 (0.07%)

Chain 1:   77 NYALTTNQGVRIADDQNSLRAGSRGPTLLEDFILREKITHFDHERIPERIVHARGSAAHGYFQPYKSLSD
              1111111111111111111111111111111111111111111111111111111111111111111111
Chain 2:    6 HKNLTTNQGVPVGDNQNSRTAGHRGPSFLDDYHLIEKLAHFDRERIPERVVHARGAGAYGVFEVENSMEK

Chain 1:  147 ITKADFLSDPNKITPVFVRFSTCQGGAGSADTVRDIRGFATKFYTEEGIFDLVGNNTPIFFIQDAHKFPD
              1111111111111111111111111111111111111111111111111111111111111111111111
Chain 2:   76 HTRAAFLSEEGKQTDVFVRFSTVIHPKGSPETLRDPRGFAVKFYTEEGNYDLVGNNLPIFFIRDALKFPD

Chain 1:  217 FVHAVKPEPHWAIPQGQSAHDTFWDYVSLQPETLHNVMWAMSDRGIPRSYRTMEGFGIHTFRLINAEGKA
              11111111111111    111111111111111111111111111111111111111111111111111
Chain 2:  146 MVHSLKPDPVTNIQ----DPDRYWDFMTLTPESTHMLTWLFSDEGIPANYAEMRGSGVHTFRWVNKYGET

Chain 1:  287 TFVRFHWKPLAGKASLVWDEAQKLTGRDPDFHRRELWEAIEAGDFPEYELGFQLIPEEDEFKFDFDLLDP
              1111111111111111111111111111111111111111111111111111111111111111111111
Chain 2:  212 KYVKYHWRPSEGIRNLSMEEAAEIQANDFQHATRDLYDRIEKGNYPAWDLYVQLMPLSDYDELDYDPCDP

Chain 1:  357 TKLIPEELVPVQRVGKMVLNRNPDNFFAENEQAAFHPGHIVPGLDFTNDPLLQGRLFSYTDTQISRLGGP
              1111111111111111111111111111111111111111111111111111111111111111111 11
Chain 2:  282 TKTWSEEDYPLQKVGRMTLNRNPENFFAETEQAAFTPSALVPGIEASEDKLLQGRLFSYPDTQRHRL-GA

Chain 1:  427 NFHEIPINRPTCPYHNFQRDGMHRMGIDTNPANYEPNSINDNWPRETPPGPKRGGFESYQERV-EGNKVR
              11111111111111111111111111111111111111111 1111111111       11111111 111111
Chain 2:  351 NYMRIPVNCPYAPVHNNQQDGFMTTTRPSGHINYEPNR-YDDQPKENPH-------YKESEPVLHGDRMVR

Chain 1:  496 ERSPSFGEYYSHPRLFWLSQTPFEQRHIVDGFSFELSKVVRPYIRERVVDQLAHIDLTLAQAVAKNLGIE
              111111 11111111111111111111111111111111 111111111111111111111111111111
Chain 2:  414 QKIEKP-NDFKQAGEKYRSYSEEEKQALIKNLTADLKGV-NEKTKLLAICNFYRADEDYGQRLADSLGVD

Chain 1:  566 LTDD
              1111
Chain 2:  482 IRSY
```

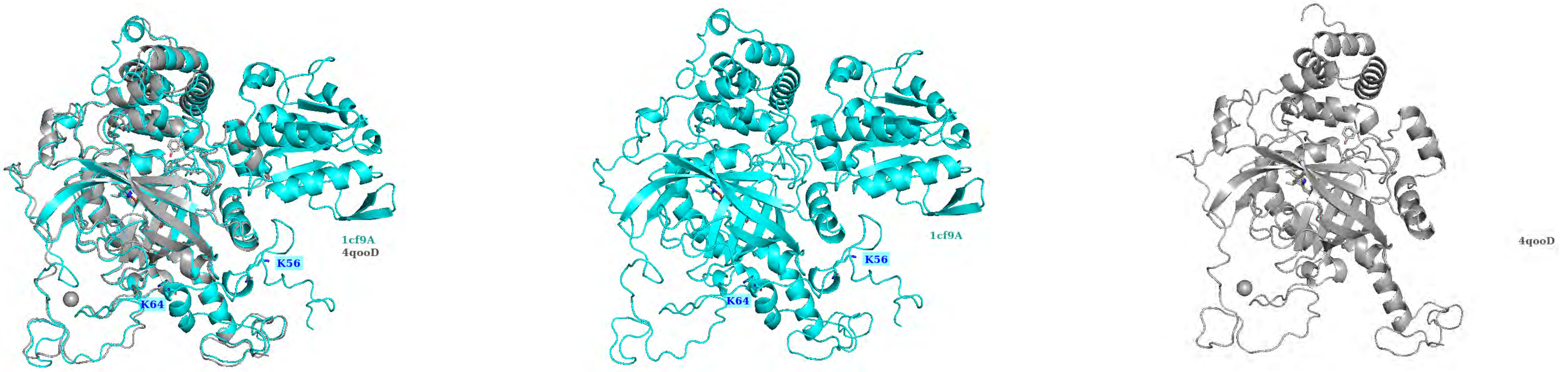

UniProt ID: none
PDB ID: 4QOP_D

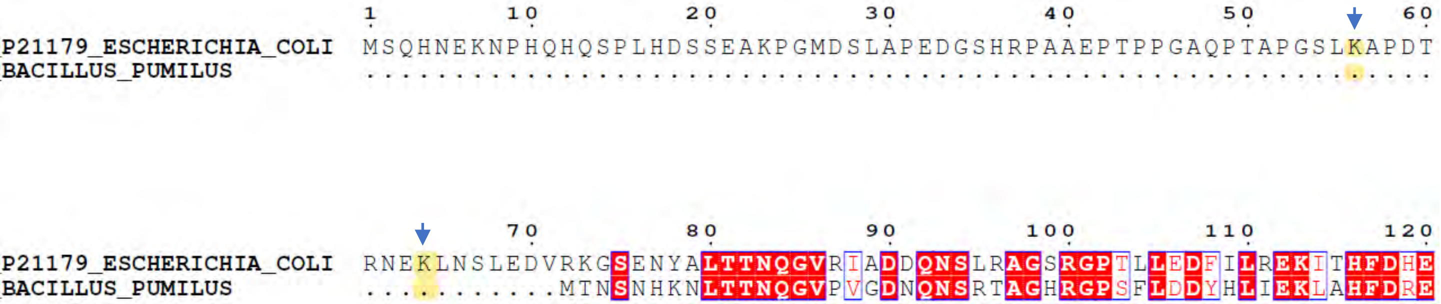

Full sequences in supplemental file.

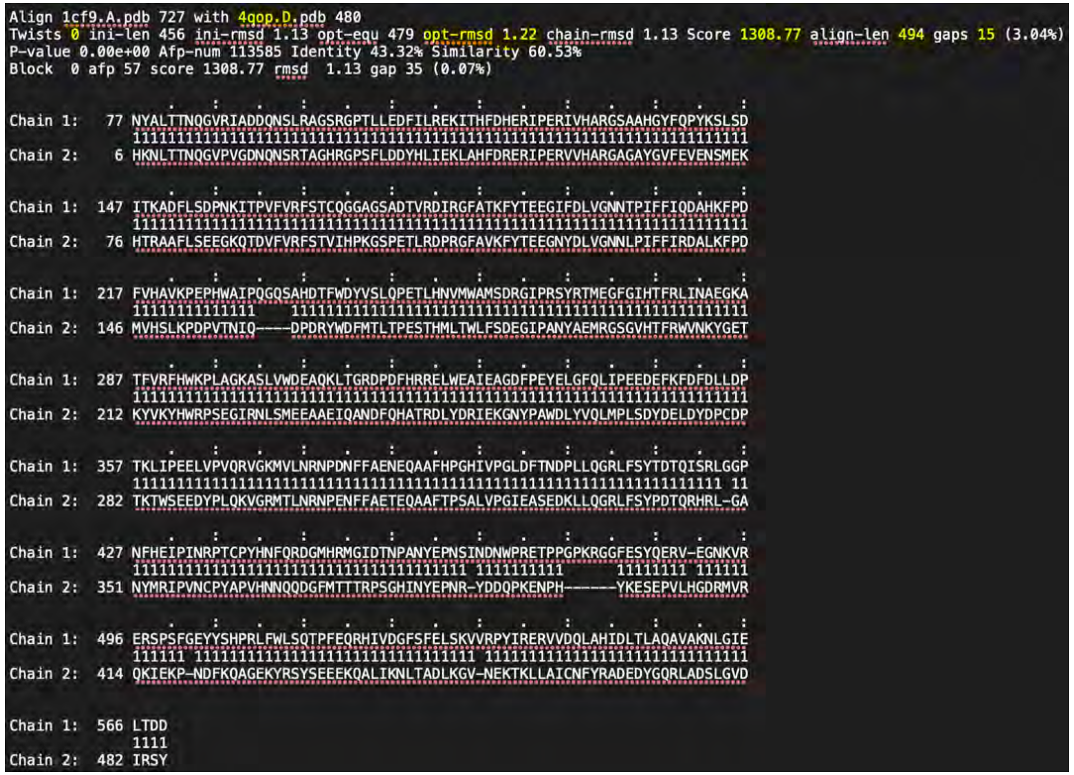
```
Align 1cf9.A.pdb 727 with 4qop.D.pdb 480
Twists 0 ini-len 456 ini-rmsd 1.13 opt-equ 479 opt-rmsd 1.22 chain-rmsd 1.13 Score 1308.77 align-len 494 gaps 15 (3.04%)
P-value 0.00e+00 Afp-num 113585 Identity 43.32% Similarity 60.53%
Block  0 afp 57 score 1308.77 rmsd  1.13 gap 35 (0.07%)

              .    :    .    :    .    :    .    :    .    :    .    :    .    :
Chain 1:   77 NYALTTNQGVRIADDQNSLRAGSRGPTLLEDFILREKITHFDHERIPERIVHARGSAAHGYFQPYKSLSD
              1111111111111111111111111111111111111111111111111111111111111111111111
Chain 2:    6 HKNLTTNQGVPVGDNQNSRTAGHRGPSFLDDYHLIEKLAHFDRERIPERVVHARGAGAYGVFEVENSMEK

              .    :    .    :    .    :    .    :    .    :    .    :    .    :
Chain 1:  147 ITKADFLSDPNKITPVFVRFSTCQGGAGSADTVRDIRGFATKFYTEEGIFDLVGNNTPIFFIQDAHKFPD
              1111111111111111111111111111111111111111111111111111111111111111111111
Chain 2:   76 HTRAAFLSEEGKQTDVFVRFSTVIHPKGSPETLRDPRGFAVKFYTEEGNYDLVGNNLPIFFIRDALKFPD

              .    :    .    :    .    :    .    :    .    :    .    :    .    :
Chain 1:  217 FVHAVKPEPHWAIPQGQSAHDTFWDYVSLQPETLHNVMWAMSDRGIPRSYRTMEGFGIHTFRLINAEGKA
              11111111111111    11111111111111111111111111111111111111111111111111
Chain 2:  146 MVHSLKPDPVTNIQ----DPDRYWDFMTLTPESTHMLTWLFSDEGIPANYAEMRGSGVHTFRWVNKYGET

              .    :    .    :    .    :    .    :    .    :    .    :    .    :
Chain 1:  287 TFVRFHWKPLAGKASLVWDEAQKLTGRDPDFHRRELWEAIEAGDFPEYELGFQLIPEEDEFKFDFDLLDP
              1111111111111111111111111111111111111111111111111111111111111111111111
Chain 2:  212 KYVKYHWRPSEGIRNLSMEEAAEIQANDFQHATRDLYDRIEKGNYPAWDLYVQLMPLSDYDELDYDPCDP

              .    :    .    :    .    :    .    :    .    :    .    :    .    :
Chain 1:  357 TKLIPEELVPVQRVGKMVLNRNPDNFFAENEQAAFHPGHIVPGLDFTNDPLLQGRLFSYTDTQISRLGGP
              111111111111111111111111111111111111111111111111111111111111111111 11
Chain 2:  282 TKTWSEEDYPLQKVGRMTLNRNPENFFAETEQAAFTPSALVPGIEASEDKLLQGRLFSYPDTQRHRL-GA

              .    :    .    :    .    :    .    :    .    :    .    :    .    :
Chain 1:  427 NFHEIPINRPTCPYHNFQRDGMHRMGIDTNPANYEPNSINDNWPRETPPGPKRGGFESYQERV-EGNKVR
              1111111111111111111111111111111111111 1111111111      11111111 111111
Chain 2:  351 NYMRIPVNCPYAPVHNNQQDGFMTTTRPSGHINYEPNR-YDDQPKENPH------YKESEPVLHGDRMVR

              .    :    .    :    .    :    .    :    .    :    .    :    .    :
Chain 1:  496 ERSPSFGEYYSHPRLFWLSQTPFEQRHIVDGFSFELSKVVRPYIRERVVDQLAHIDLTLAQAVAKNLGIE
              111111 111111111111111111111111111111111 111111111111111111111111111111
Chain 2:  414 QKIEKP-NDFKQAGEKYRSYSEEEKQALIKNLTADLKGV-NEKTKLLAICNFYRADEDYGQRLADSLGVD

Chain 1:  566 LTDD
              1111
Chain 2:  482 IRSY
```

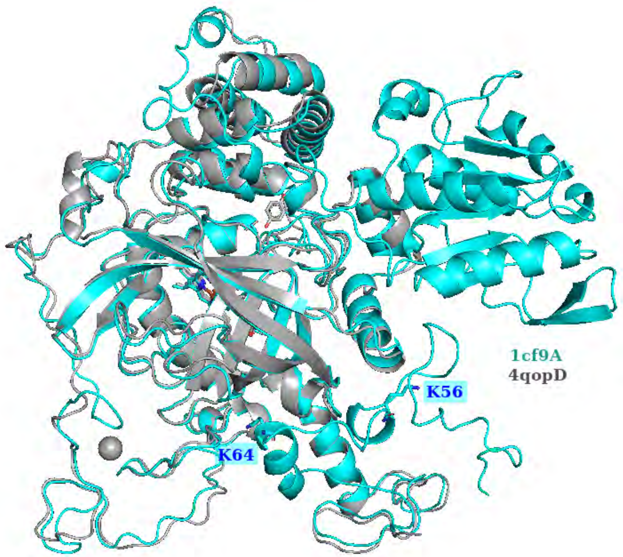

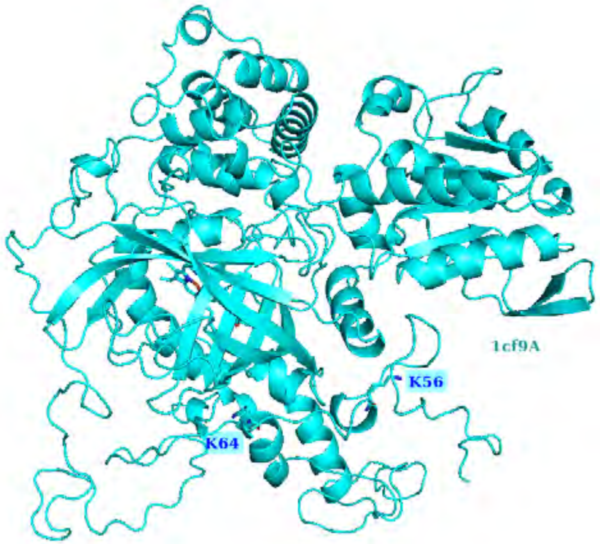

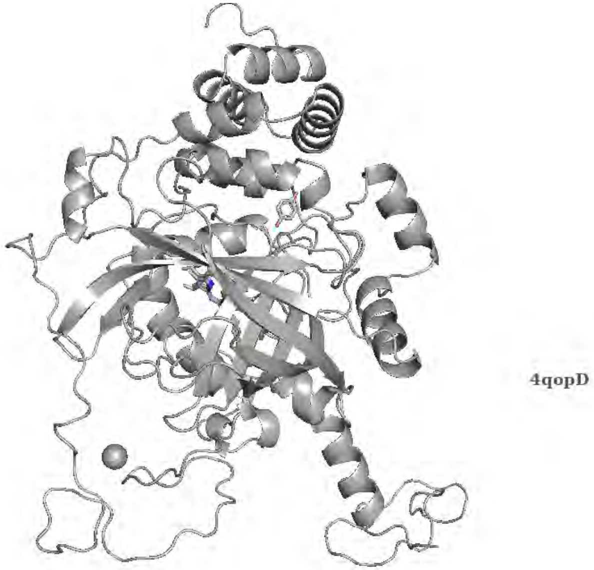

UniProt ID: none

PDB ID: 4QOQ_D

```
                        1        10        20        30        40        50        60
P21179_ESCHERICHIA_COLI MSQHNEKNPHQHQSPLHDSSEAKPGMDSLAPEDGSHRPAAEPTPPGAQPTAPGSLKAPDT
BACILLUS_PUMILUS        ............................................................

                                 70        80        90       100       110       120
P21179_ESCHERICHIA_COLI RNEKLNSLEDVRKGSENYALTTNQGVRIADDQNSLRAGSRGPTLLEDFILREKITHFDHE
BACILLUS_PUMILUS        ...........MTNSNHKNLTTNQGVPVGDNQNSRTAGHRGPSFLDDYHLIEKLAHFDRE
```

Full sequences in supplemental file.

```
Align 1cf9.A.pdb 727 with 4qoq.D.pdb 481
Twists 0 ini-len 456 ini-rmsd 1.12 opt-equ 480 opt-rmsd 1.22 chain-rmsd 1.12 Score 1308.83 align-len 495 gaps 15 (3.03%)
P-value 0.00e+00 Afp-num 114765 Identity 43.23% Similarity 60.40%
Block  0 afp 57 score 1308.83 rmsd  1.12 gap 35 (0.07%)

Chain 1:   77 NYALTTNQGVRIADDQNSLRAGSRGPTLLEDFILREKITHFDHERIPERIVHARGSAAHGYFQPYKSLSD
              1111111111111111111111111111111111111111111111111111111111111111111111
Chain 2:    6 HKNLTTNQGVPVGDNQNSRTAGHRGPSFLDDYHLIEKLAHFDRERIPERVVHARGAGAYGVFEVENSMEK

Chain 1:  147 ITKADFLSDPNKITPVFVRFSTCQGGAGSADTVRDIRGFATKFYTEEGIFDLVGNNTPIFFIQDAHKFPD
              1111111111111111111111111111111111111111111111111111111111111111111111
Chain 2:   76 HTRAAFLSEEGKQTDVFVRFSTVIHPKGSPETLRDPRGFAVKFYTEEGNYDLVGNNLPIFFIRDALKFPD

Chain 1:  217 FVHAVKPEPHWAIPQGQSAHDTFWDYVSLQPETLHNVMWAMSDRGIPRSYRTMEGFGIHTFRLINAEGKA
              11111111111111    1111111111111111111111111111111111111111111111111111
Chain 2:  146 MVHSLKPDPVTNIQ----DPDRYWDFMTLTPESTHMLTWLFSDEGIPANYAEMRGSGVHTFRWVNKYGET

Chain 1:  287 TFVRFHWKPLAGKASLVWDEAQKLTGRDPDFHRRELWEAIEAGDFPEYELGFQLIPEEDEFKFDFDLLDP
              1111111111111111111111111111111111111111111111111111111111111111111111
Chain 2:  212 KYVKYHWRPSEGIRNLSMEEAAEIQANDFQHATRDLYDRIEKGNYPAWDLYVQLMPLSDYDELDYDPCDP

Chain 1:  357 TKLIPEELVPVQRVGKMVLNRNPDNFFAENEQAAFHPGHIVPGLDFTNDPLLQGRLFSYTDTQISRLGGP
              111111111111111111111111111111111111111111111111111111111111111111 1
Chain 2:  282 TKTWSEEDYPLQKVGRMTLNRNPENFFAETEQAAFTPSALVPGIEASEDKLLQGRLFSYPDTQRHRLG-A

Chain 1:  427 NFHEIPINRPTCPYHNFQRDGMHRMGIDTNPANYEPNSINDNWPRETPPGPKRGGFESYQERV-EGNKVR
              11111111111111111111111111111111111111 1111111111      11111111 111111
Chain 2:  351 NYMRIPVNCPYAPVHNNQQDGFMTTTRPSGHINYEPNR-YDDQPKENPH------YKESEPVLHGDRMVR

Chain 1:  496 ERSPSFGEYYSHPRLFWLSQTPFEQRHIVDGFSFELSKVVRPYIRERVVDQLAHIDLTLAQAVAKNLGIE
              111111 11111111111111111111111111111111 111111111111111111111111111111
Chain 2:  414 QKIEKP-NDFKQAGEKYRSYSEEEKQALIKNLTADLKGV-NEKTKLLAICNFYRADEDYGQRLADSLGVD

Chain 1:  566 LTDDQ
              11111
Chain 2:  482 IRSYL
```

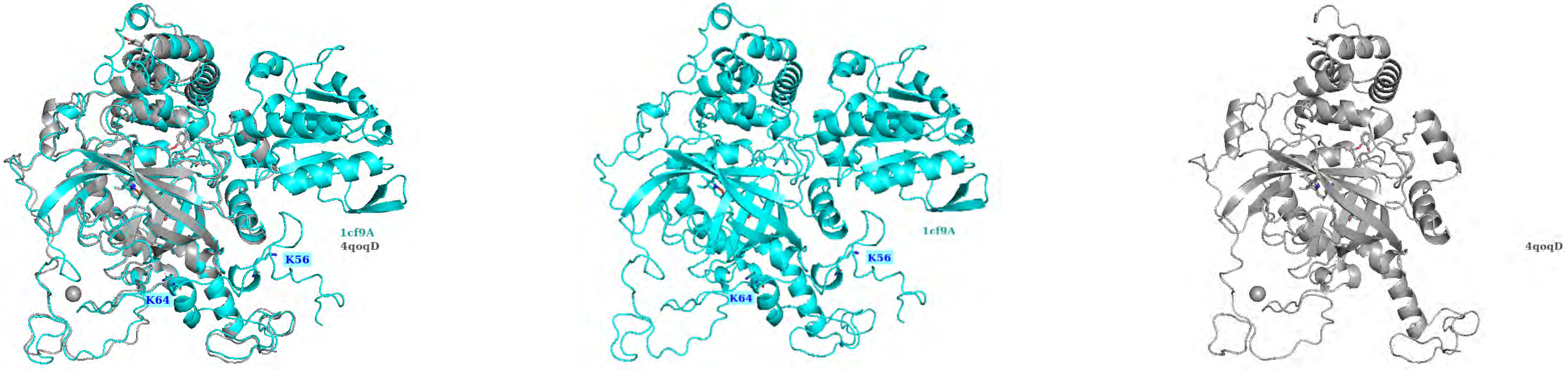

UniProt ID: none

PDB ID: 4QOR_B

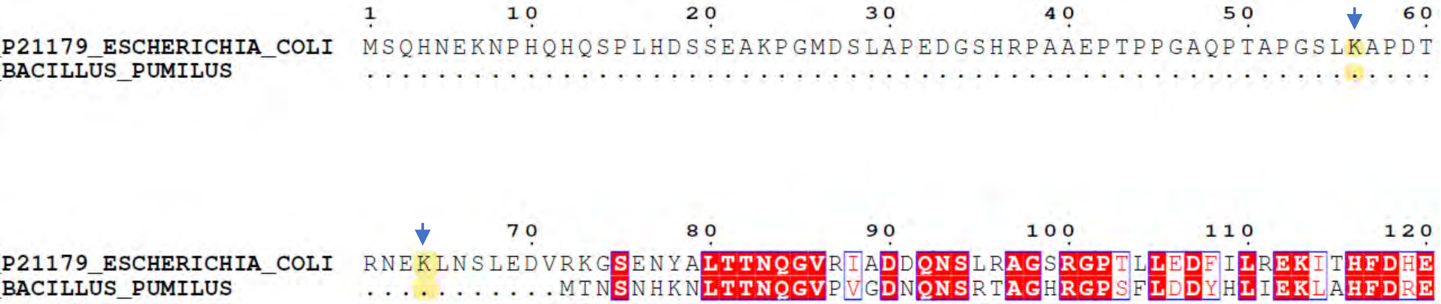

Full sequences in supplemental file.

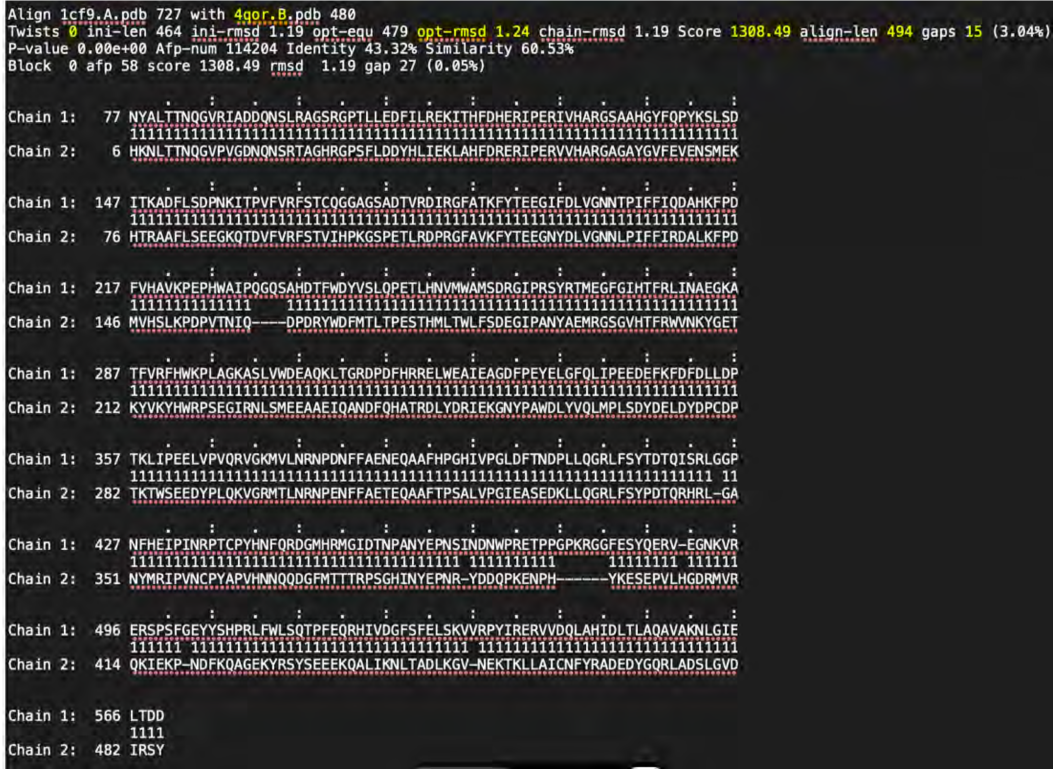

```
Align 1cf9.A.pdb 727 with 4qor.B.pdb 480
Twists 0 ini-len 464 ini-rmsd 1.19 opt-equ 479 opt-rmsd 1.24 chain-rmsd 1.19 Score 1308.49 align-len 494 gaps 15 (3.04%)
P-value 0.00e+00 Afp-num 114204 Identity 43.32% Similarity 60.53%
Block  0 afp 58 score 1308.49 rmsd  1.19 gap 27 (0.05%)

Chain 1:   77 NYALTTNQGVRIADDQNSLRAGSRGPTLLEDFILREKITHFDHERIPERIVHARGSAAHGYFQPYKSLSD
              1111111111111111111111111111111111111111111111111111111111111111111111
Chain 2:    6 HKNLTTNQGVPVGDNQNSRTAGHRGPSFLDDYHLIEKLAHFDRERIPERVVHARGAGAYGVFEVENSMEK

Chain 1:  147 ITKADFLSDPNKITPVFVRFSTCQGGAGSADTVRDIRGFATKFYTEEGIFDLVGNNTPIFFIQDAHKFPD
              1111111111111111111111111111111111111111111111111111111111111111111111
Chain 2:   76 HTRAAFLSEEGKQTDVFVRFSTVIHPKGSPETLRDPRGFAVKFYTEEGNYDLVGNNLPIFFIRDALKFPD

Chain 1:  217 FVHAVKPEPHWAIPQGQSAHDTFWDYVSLQPETLHNVMWAMSDRGIPRSYRTMEGFGIHTFRLINAEGKA
              11111111111111    1111111111111111111111111111111111111111111111111111
Chain 2:  146 MVHSLKPDPVTNIQ----DPDRYWDFMTLTPESTHMLTWLFSDEGIPANYAEMRGSGVHTFRWVNKYGET

Chain 1:  287 TFVRFHWKPLAGKASLVWDEAQKLTGRDPDFHRRELWEAIEAGDFPEYELGFQLIPEEDEFKFDFDLLDP
              1111111111111111111111111111111111111111111111111111111111111111111111
Chain 2:  212 KYVKYHWRPSEGIRNLSMEEAAEIQANDFQHATRDLYDRIEKGNYPAWDLYVQLMPLSDYDELDYDPCDP

Chain 1:  357 TKLIPEELVPVQRVGKMVLNRNPDNFFAENEQAAFHPGHIVPGLDFTNDPLLQGRLFSYTDTQISRLGGP
              111111111111111111111111111111111111111111111111111111111111111111 11
Chain 2:  282 TKTWSEEDYPLQKVGRMTLNRNPENFFAETEQAAFTPSALVPGIEASEDKLLQGRLFSYPDTQRHRL-GA

Chain 1:  427 NFHEIPINRPTCPYHNFQRDGMHRMGIDTNPANYEPNSINDNWPRETPPGPKRGGFESYQERV-EGNKVR
              1111111111111111111111111111111111111 1111111111      11111111 111111
Chain 2:  351 NYMRIPVNCPYAPVHNNQQDGFMTTTRPSGHINYEPNR-YDDQPKENPH------YKESEPVLHGDRMVR

Chain 1:  496 ERSPSFGEYYSHPRLFWLSQTPFEQRHIVDGFSFELSKVVRPYIRERVVDQLAHIDLTLAQAVAKNLGIE
              111111 111111111111111111111111111111111 111111111111111111111111111111
Chain 2:  414 QKIEKP-NDFKQAGEKYRSYSEEEKQALIKNLTADLKGV-NEKTKLLAICNFYRADEDYGQRLADSLGVD

Chain 1:  566 LTDD
              1111
Chain 2:  482 IRSY
```

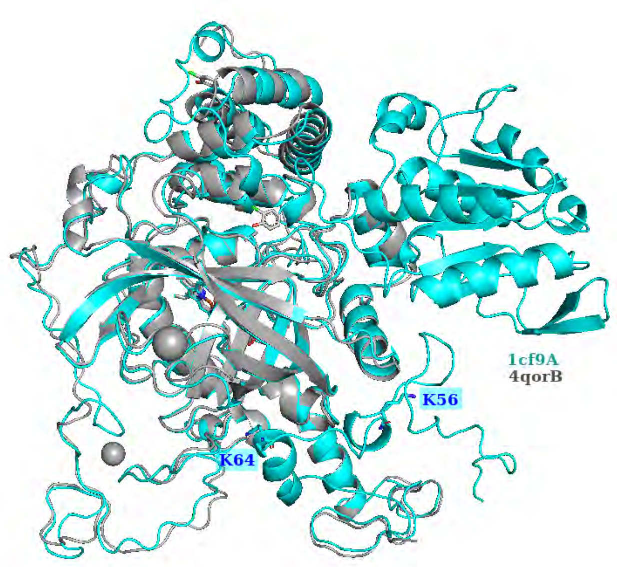

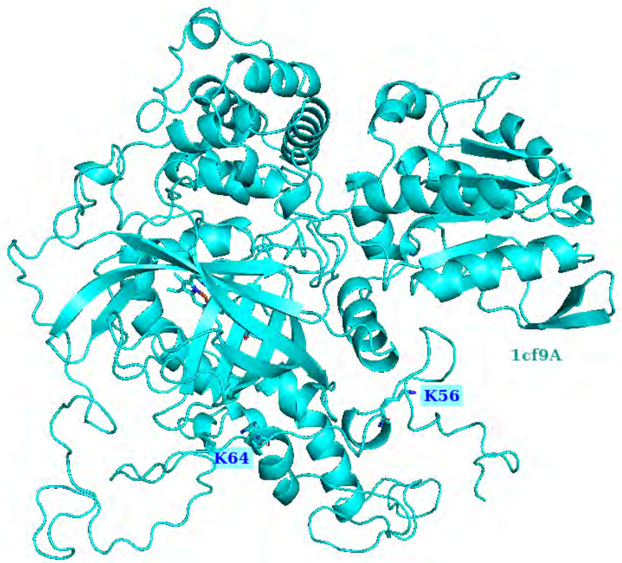

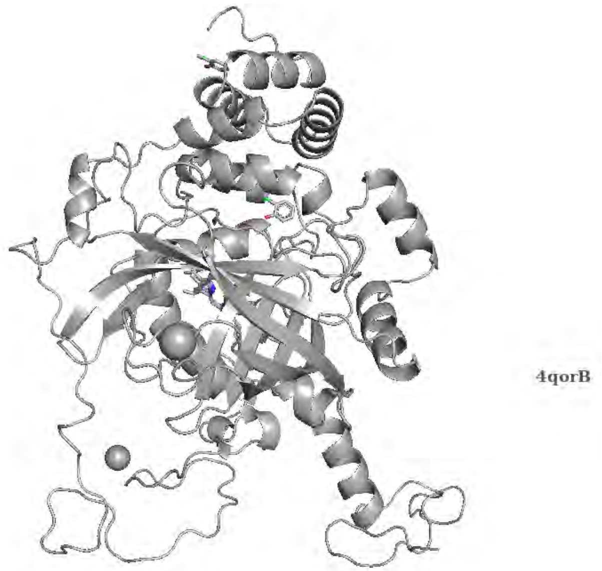

UniProt ID: O52762

PDB ID: 4E37_D

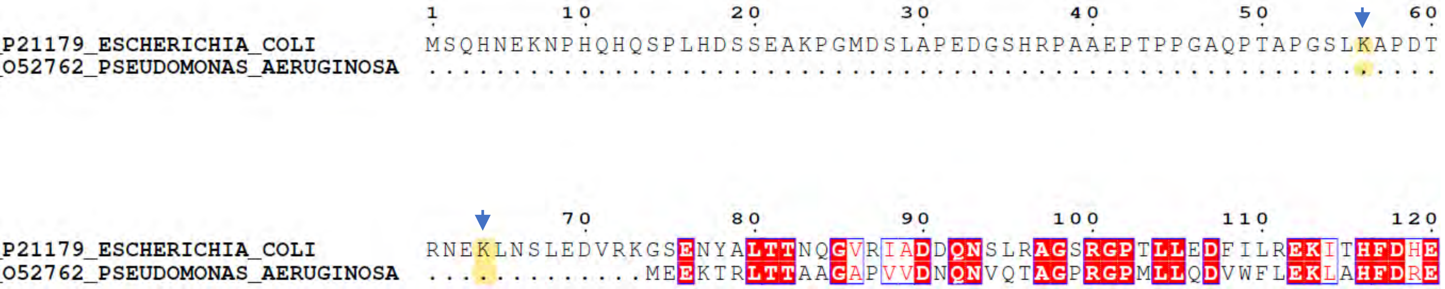

Full sequences in supplemental file.

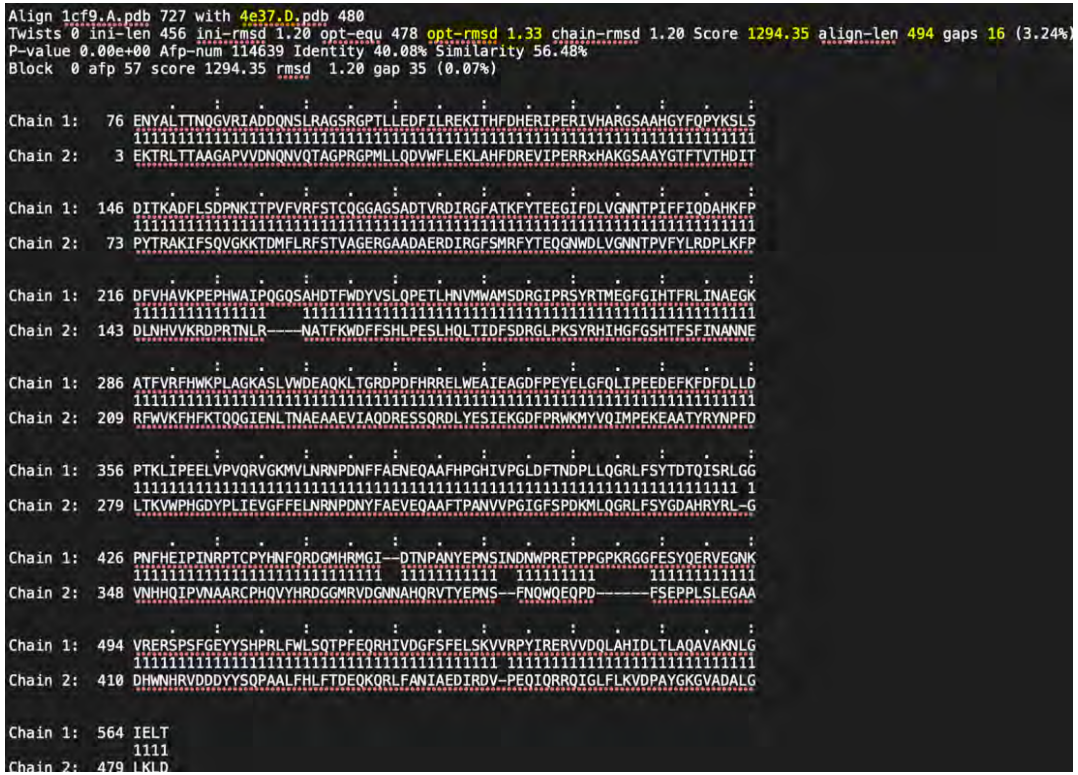

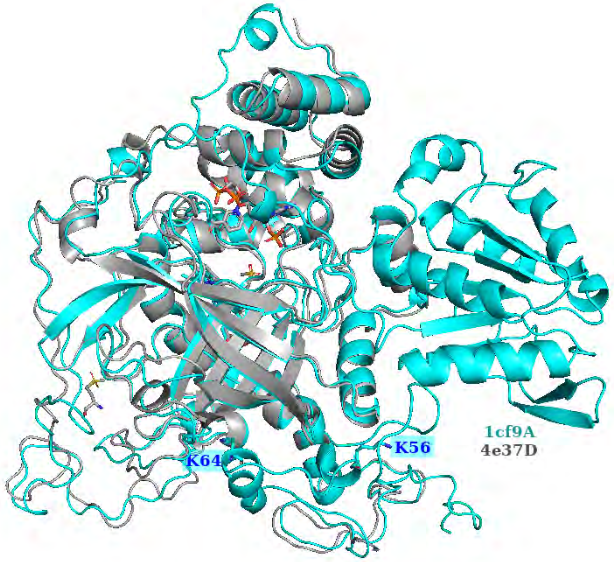

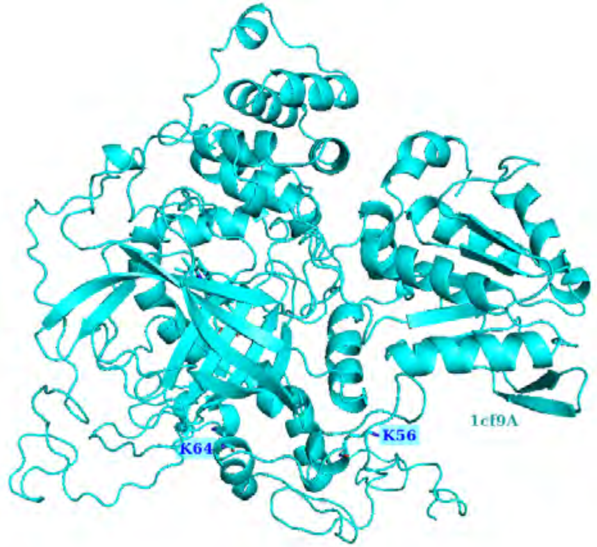

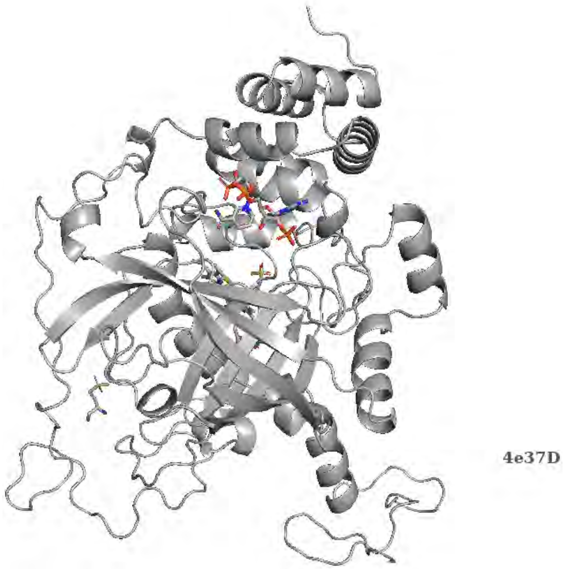

UniProt ID: P00432

PDB ID: 1TGU_B

```
                          1        10        20        30        40        50       60
P21179_ESCHERICHIA_COLI   MSQHNEKNPHQHQSPLHDSSEAKPGMDSLAPEDGSHRPAAEPTPPGAQPTAPGSLKAPDT
P00432_BOS_TAURUS         .....................................................MADNRDP

                                   70        80        90       100       110       120
P21179_ESCHERICHIA_COLI   RNEKLNSLEDVRKGSENYALTTNQGVRIADDQNSLRAGSRGPTLLEDFILREKITHFDHE
P00432_BOS_TAURUS         ASDQMKHWKEQRAAQKPDVLTTGGGNPVGDKLNSLTVGPRGPLLVQDVVFTDEMAHFDRE
```

Full sequences in supplemental file.

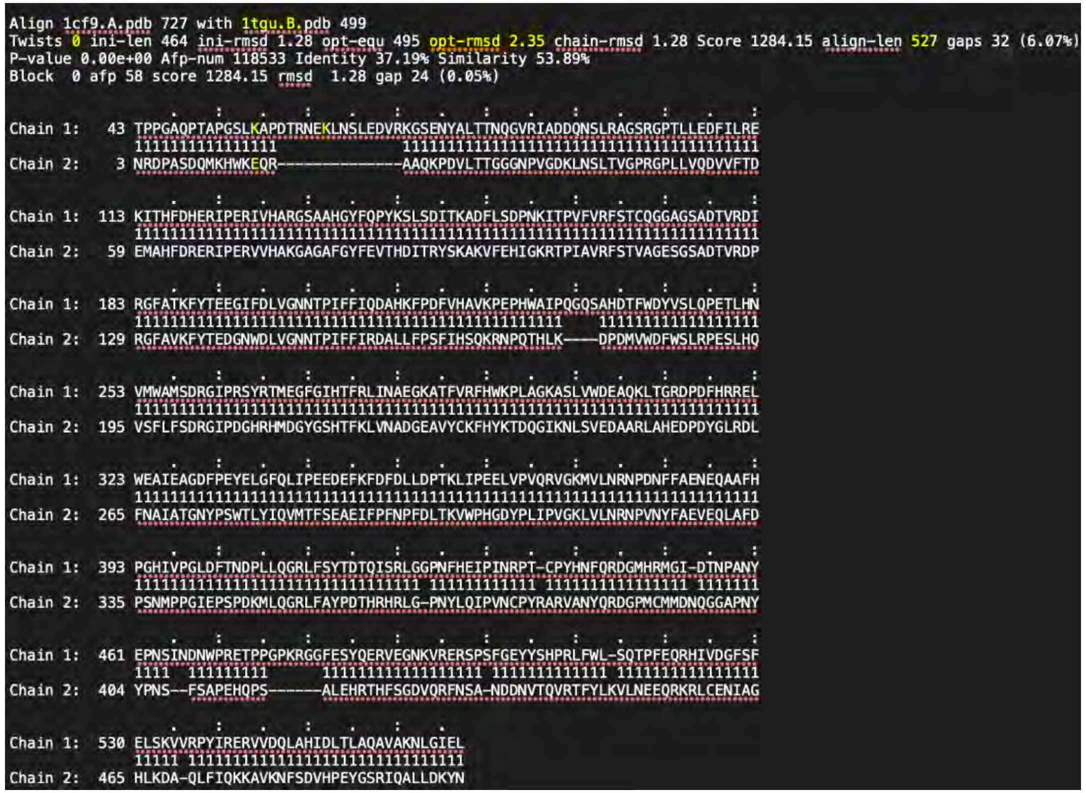

```
Align 1cf9.A.pdb 727 with 1tgu.B.pdb 499
Twists 0 ini-len 464 ini-rmsd 1.28 opt-equ 495 opt-rmsd 2.35 chain-rmsd 1.28 Score 1284.15 align-len 527 gaps 32 (6.07%)
P-value 0.00e+00 Afp-num 118533 Identity 37.19% Similarity 53.89%
Block  0 afp 58 score 1284.15 rmsd  1.28 gap 24 (0.05%)

                  .    :    .    :    .    :    .    :    .    :    .    :    .    :
Chain 1:   43 TPPGAQPTAPGSLKAPDTRNEKLNSLEDVRKGSENYALTTNQGVRIADDQNSLRAGSRGPTLLEDFILRE
              1111111111111111              1111111111111111111111111111111111111111
Chain 2:    3 NRDPASDQMKHWKEQR--------------AAQKPDVLTTGGGNPVGDKLNSLTVGPRGPLLVQDVVFTD

                  .    :    .    :    .    :    .    :    .    :    .    :    .    :
Chain 1:  113 KITHFDHERIPERIVHARGSAAHGYFQPYKSLSDITKADFLSDPNKITPVFVRFSTCQGGAGSADTVRDI
              1111111111111111111111111111111111111111111111111111111111111111111111
Chain 2:   59 EMAHFDRERIPERVVHAKGAGAFGYFEVTHDITRYSKAKVFEHIGKRTPIAVRFSTVAGESGSADTVRDP

                  .    :    .    :    .    :    .    :    .    :    .    :    .    :
Chain 1:  183 RGFATKFYTEEGIFDLVGNNTPIFFIQDAHKFPDFVHAVKPEPHWAIPQGQSAHDTFWDYVSLQPETLHN
              11111111111111111111111111111111111111111111111111    11111111111111111
Chain 2:  129 RGFAVKFYTEDGNWDLVGNNTPIFFIRDALLFPSFIHSQKRNPQTHLK----DPDMVWDFWSLRPESLHQ

                  .    :    .    :    .    :    .    :    .    :    .    :    .    :
Chain 1:  253 VMWAMSDRGIPRSYRTMEGFGIHTFRLINAEGKATFVRFHWKPLAGKASLVWDEAQKLTGRDPDFHRREL
              1111111111111111111111111111111111111111111111111111111111111111111111
Chain 2:  195 VSFLFSDRGIPDGHRHMDGYGSHTFKLVNADGEAVYCKFHYKTDQGIKNLSVEDAARLAHEDPDYGLRDL

                  .    :    .    :    .    :    .    :    .    :    .    :    .    :
Chain 1:  323 WEAIEAGDFPEYELGFQLIPEEDEFKFDFDLLDPTKLIPEELVPVQRVGKMVLNRNPDNFFAENEQAAFH
              1111111111111111111111111111111111111111111111111111111111111111111111
Chain 2:  265 FNAIATGNYPSWTLYIQVMTFSEAEIFPFNPFDLTKVWPHGDYPLIPVGKLVLNRNPVNYFAEVEQLAFD

                  .    :    .    :    .    :    .    :    .    :    .    :    .    :
Chain 1:  393 PGHIVPGLDFTNDPLLQGRLFSYTDTQISRLGGPNFHEIPINRPT-CPYHNFQRDGMHRMGI-DTNPANY
              1111111111111111111111111111111111 111111111111 11111111111111111 1111111
Chain 2:  335 PSNMPPGIEPSPDKMLQGRLFAYPDTHRHRLG-PNYLQIPVNCPYRARVANYQRDGPMCMMDNQGGAPNY

                  .    :    .    :    .    :    .    :    .    :    .    :    .    :
Chain 1:  461 EPNSINDNWPRETPPGPKRGGFESYQERVEGNKVRERSPSFGEYYSHPRLFWL-SQTPFEQRHIVDGFSF
              1111  111111111      111111111111111111 1111111111111 111111111111111
Chain 2:  404 YPNS--FSAPEHQPS------ALEHRTHFSGDVQRFNSA-NDDNVTQVRTFYLKVLNEEQRKRLCENIAG

                  .    :    .    :    .    :    .
Chain 1:  530 ELSKVVRPYIRERVVDQLAHIDLTLAQAVAKNLGIEL
              11111 11111111111111111111111111111111
Chain 2:  465 HLKDA-QLFIQKKAVKNFSDVHPEYGSRIQALLDKYN
```

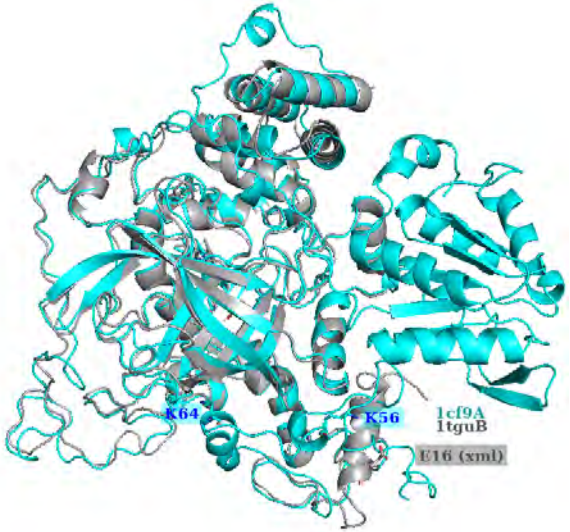

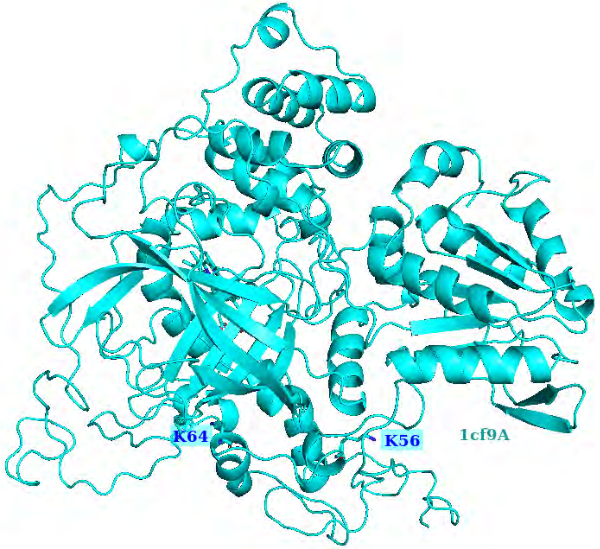

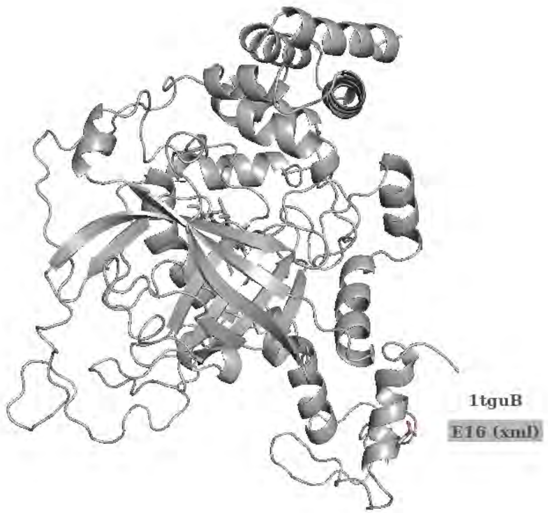

UniProt ID: P00432

PDB ID: 1TH2_A

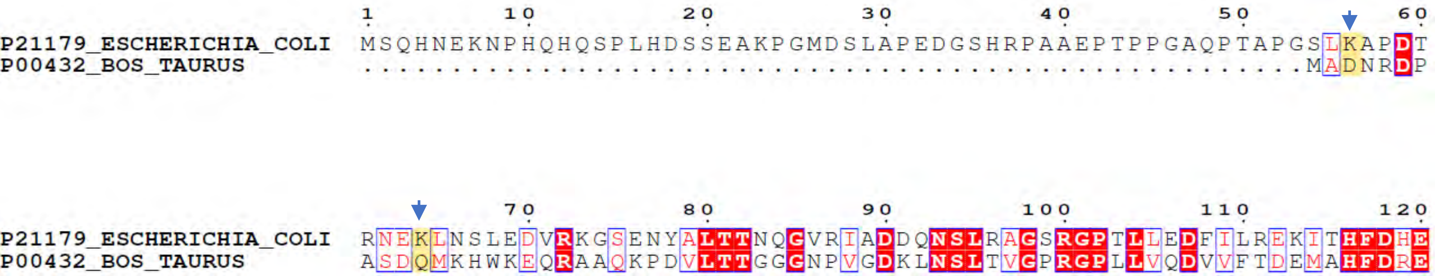

Full sequences in supplemental file.

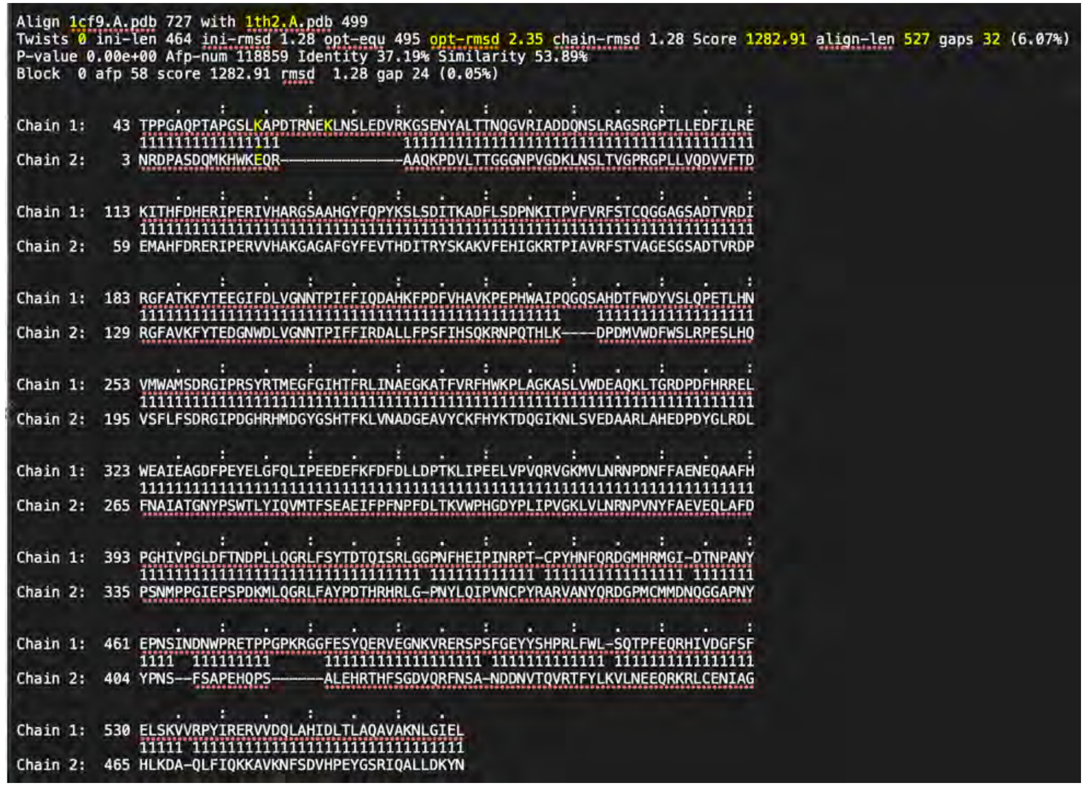

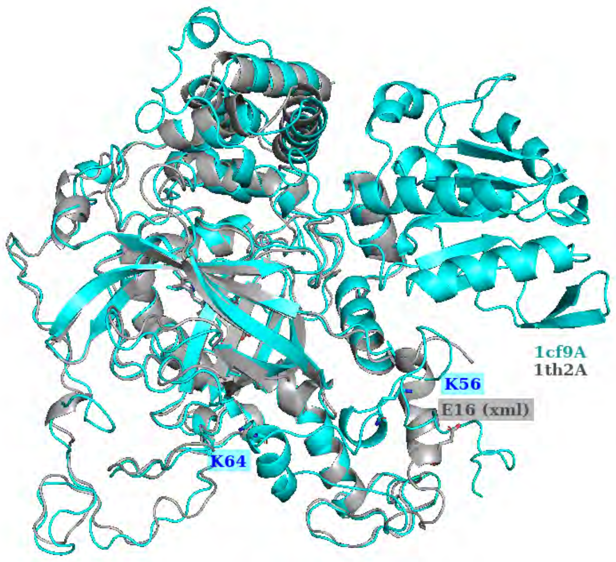

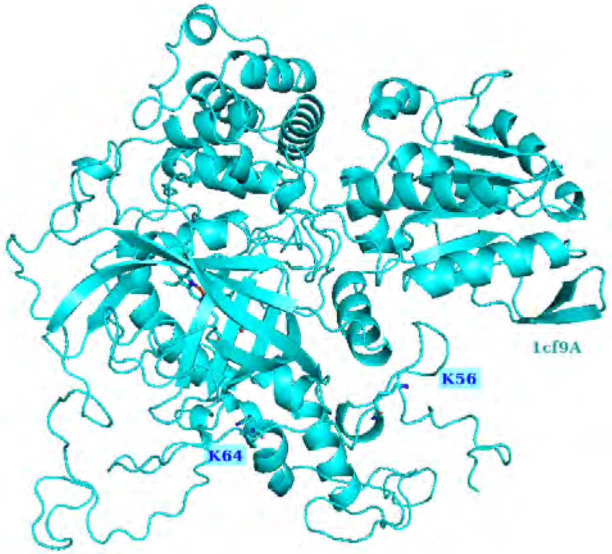

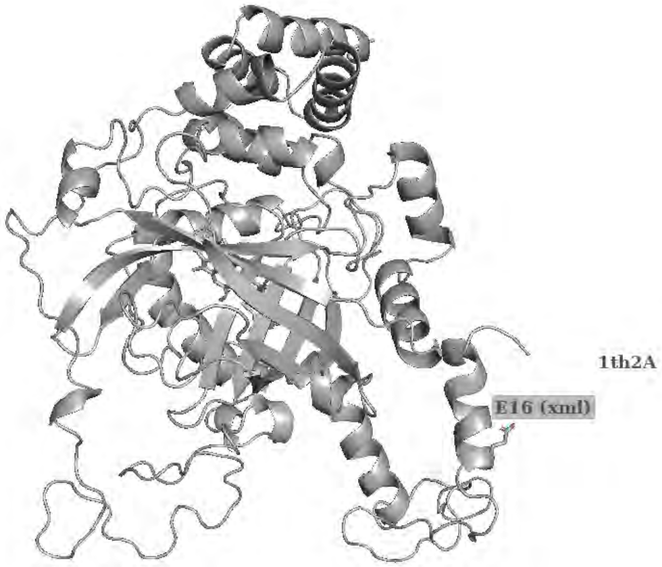

UniProt ID: P00432

PDB ID: 1TH3_B

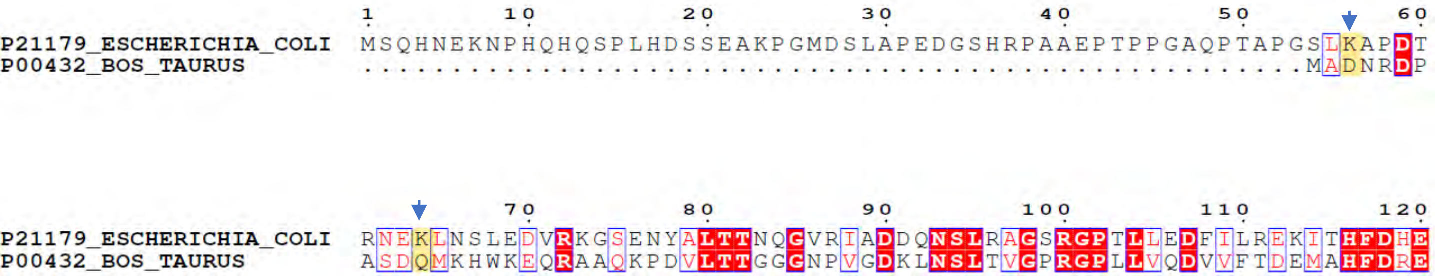

Full sequences in supplemental file.

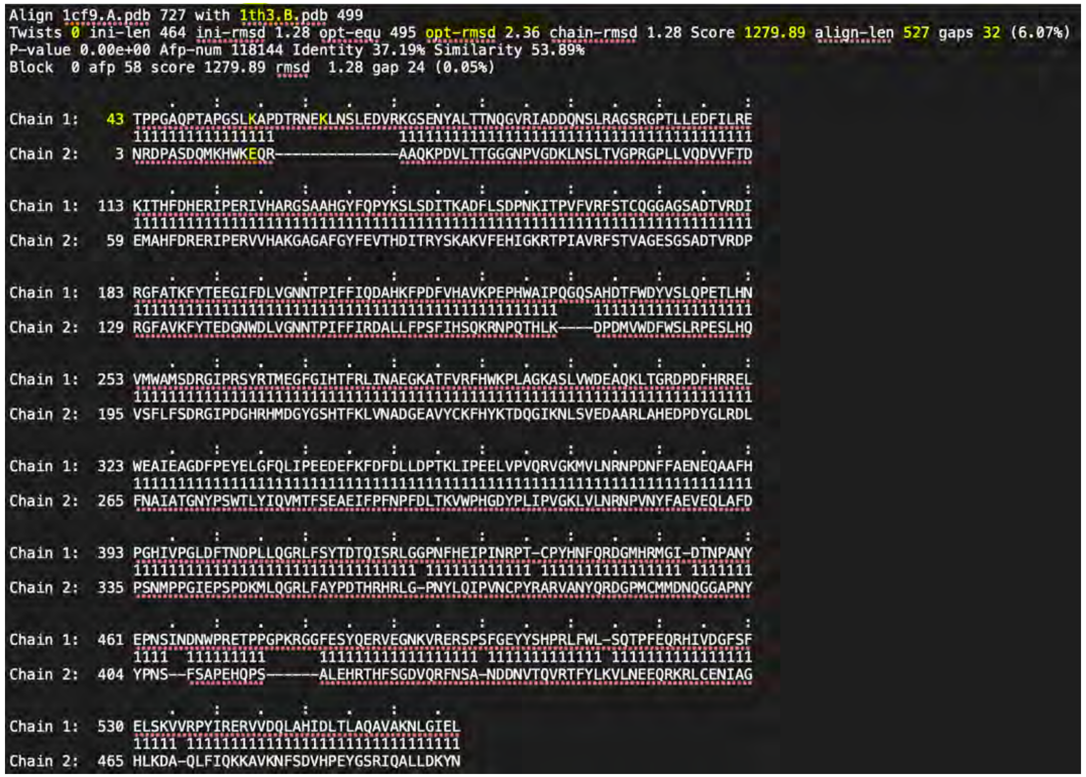

```
Align 1cf9.A.pdb 727 with 1th3.B.pdb 499
Twists 0 ini-len 464 ini-rmsd 1.28 opt-equ 495 opt-rmsd 2.36 chain-rmsd 1.28 Score 1279.89 align-len 527 gaps 32 (6.07%)
P-value 0.00e+00 Afp-num 118144 Identity 37.19% Similarity 53.89%
Block  0 afp 58 score 1279.89 rmsd  1.28 gap 24 (0.05%)

Chain 1:   43 TPPGAQPTAPGSLKAPDTRNEKLNSLEDVRKGSENYALTTNQGVRIADDQNSLRAGSRGPTLLEDFILRE
              1111111111111111              11111111111111111111111111111111111111111
Chain 2:    3 NRDPASDQMKHWKEQR--------------AAQKPDVLTTGGGNPVGDKLNSLTVGPRGPLLVQDVVFTD

Chain 1:  113 KITHFDHERIPERIVHARGSAAHGYFQPYKSLSDITKADFLSDPNKITPVFVRFSTCQGGAGSADTVRDI
              1111111111111111111111111111111111111111111111111111111111111111111111
Chain 2:   59 EMAHFDRERIPERVVHAKGAGAFGYFEVTHDITRYSKAKVFEHIGKRTPIAVRFSTVAGESGSADTVRDP

Chain 1:  183 RGFATKFYTEEGIFDLVGNNTPIFFIQDAHKFPDFVHAVKPEPHWAIPQGQSAHDTFWDYVSLQPETLHN
              1111111111111111111111111111111111111111111111111    111111111111111111
Chain 2:  129 RGFAVKFYTEDGNWDLVGNNTPIFFIRDALLFPSFIHSQKRNPQTHLK----DPDMVWDFWSLRPESLHQ

Chain 1:  253 VMWAMSDRGIPRSYRTMEGFGIHTFRLINAEGKATFVRFHWKPLAGKASLVWDEAQKLTGRDPDFHRREL
              1111111111111111111111111111111111111111111111111111111111111111111111
Chain 2:  195 VSFLFSDRGIPDGHRHMDGYGSHTFKLVNADGEAVYCKFHYKTDQGIKNLSVEDAARLAHEDPDYGLRDL

Chain 1:  323 WEAIEAGDFPEYELGFQLIPEEDEFKFDFDLLDPTKLIPEELVPVQRVGKMVLNRNPDNFFAENEQAAFH
              1111111111111111111111111111111111111111111111111111111111111111111111
Chain 2:  265 FNAIATGNYPSWTLYIQVMTFSEAEIFPFNPFDLTKVWPHGDYPLIPVGKLVLNRNPVNYFAEVEQLAFD

Chain 1:  393 PGHIVPGLDFTNDPLLQGRLFSYTDTQISRLGGPNFHEIPINRPT-CPYHNFQRDGMHRMGI-DTNPANY
              11111111111111111111111111111111 111111111111 1111111111111111 1111111
Chain 2:  335 PSNMPPGIEPSPDKMLQGRLFAYPDTHRHRLG-PNYLQIPVNCPYRARVANYQRDGPMCMMDNQGGAPNY

Chain 1:  461 EPNSINDNWPRETPPGPKRGGFESYQERVEGNKVRERSPSFGEYYSHPRLFWL-SQTPFEQRHIVDGFSF
              1111  111111111      111111111111111111 1111111111111 111111111111111
Chain 2:  404 YPNS--FSAPEHQPS------ALEHRTHFSGDVQRFNSA-NDDNVTQVRTFYLKVLNEEQRKRLCENIAG

Chain 1:  530 ELSKVVRPYIRERVVDQLAHIDLTLAQAVAKNLGIEL
              11111 1111111111111111111111111111111
Chain 2:  465 HLKDA-QLFIQKKAVKNFSDVHPEYGSRIQALLDKYN
```

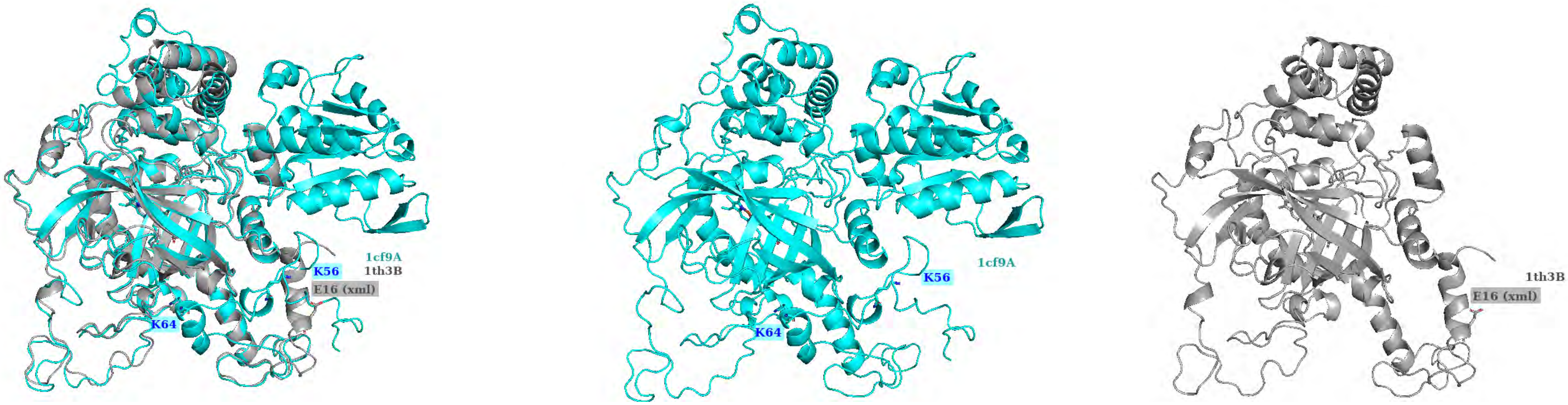

UniProt ID: P00432

PDB ID: 1TH4_A

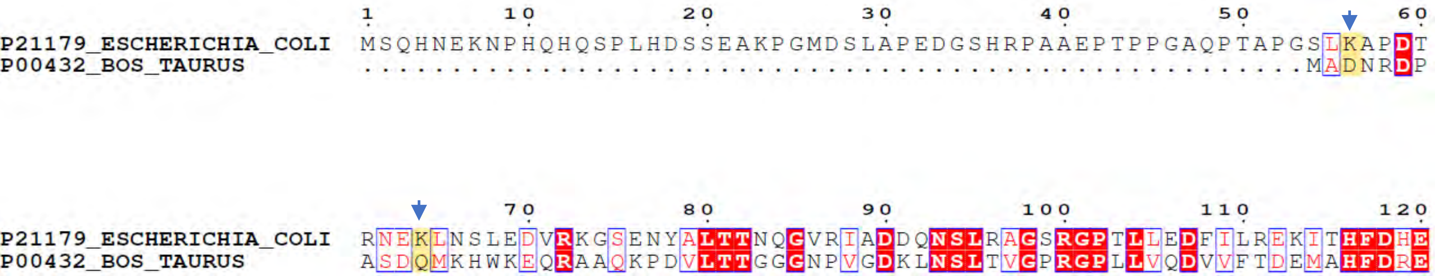

Full sequences in supplemental file.

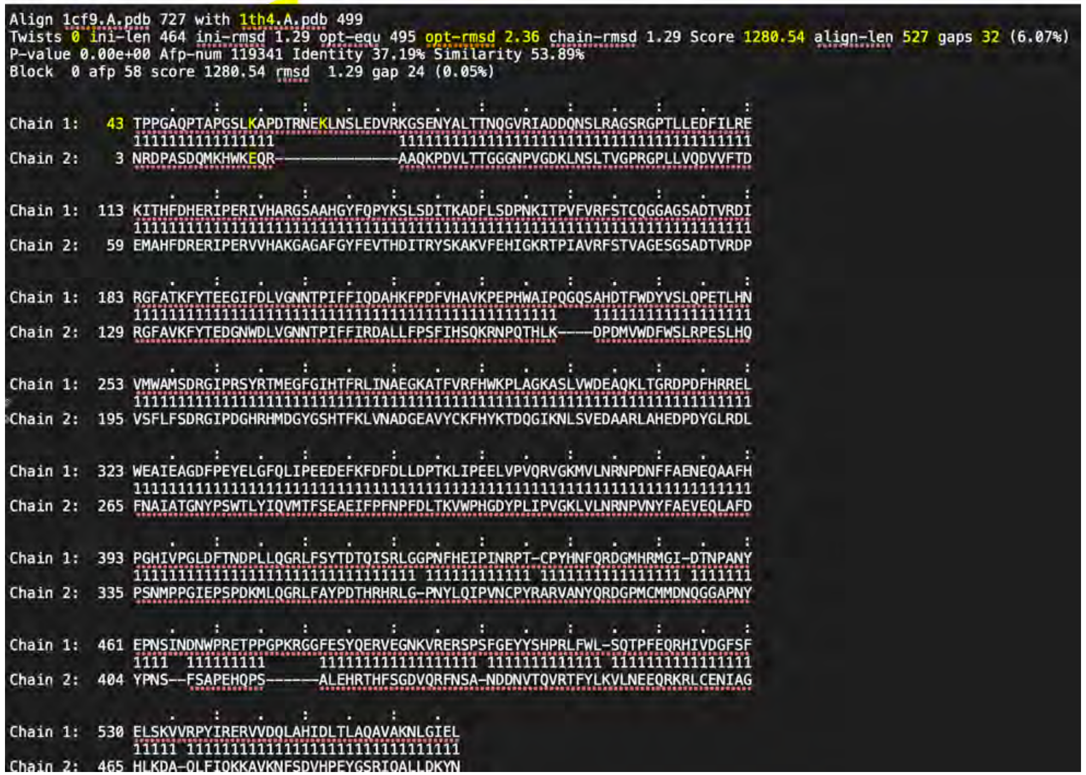

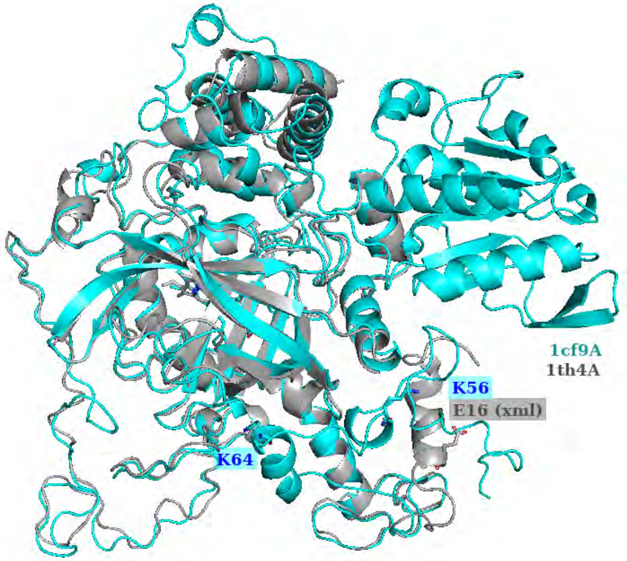

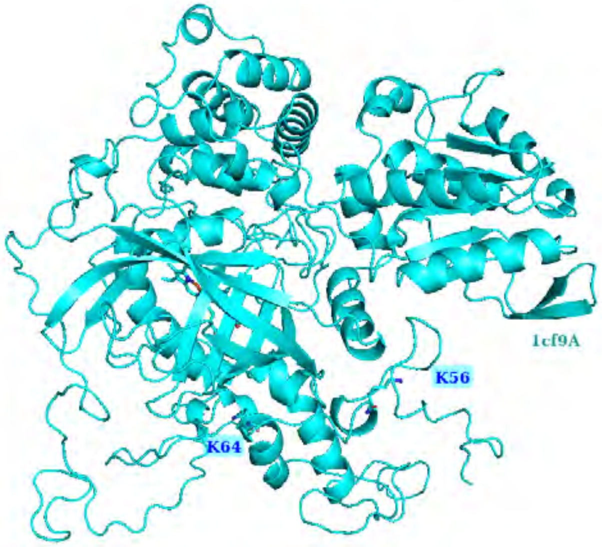

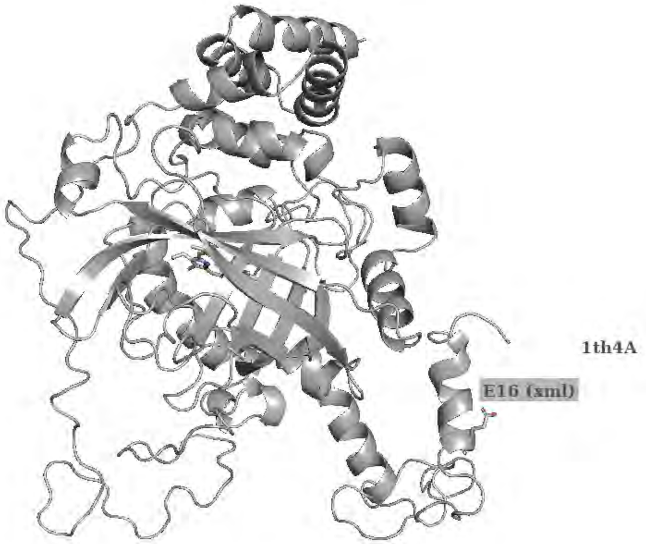

UniProt ID: P00432
PDB ID: 3J7B_C

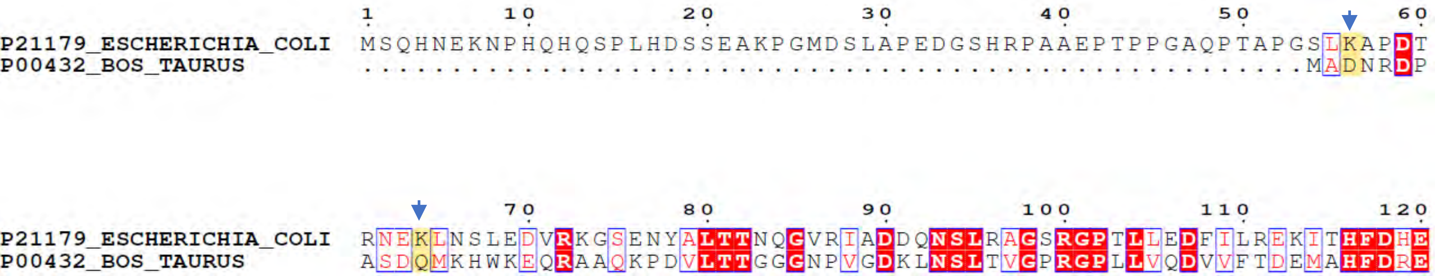

Full sequences in supplemental file.

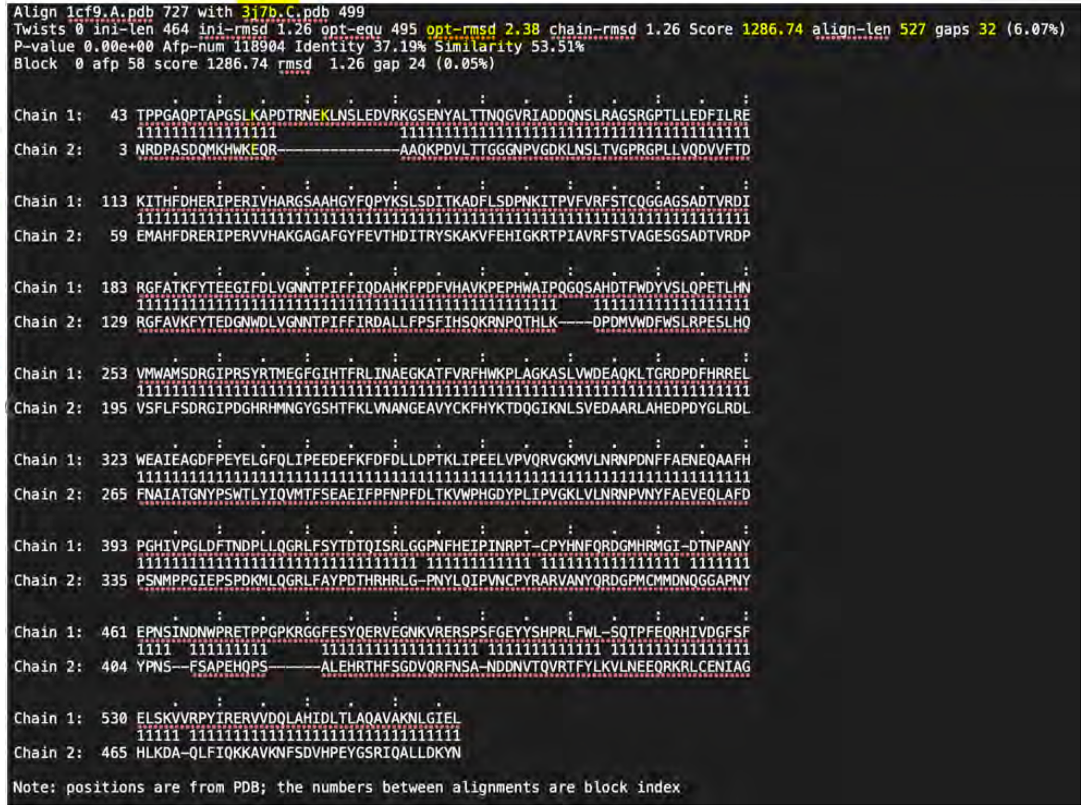

```
Align 1cf9.A.pdb 727 with 3j7b.C.pdb 499
Twists 0 ini-len 464 ini-rmsd 1.26 opt-equ 495 opt-rmsd 2.38 chain-rmsd 1.26 Score 1286.74 align-len 527 gaps 32 (6.07%)
P-value 0.00e+00 Afp-num 118904 Identity 37.19% Similarity 53.51%
Block  0 afp 58 score 1286.74 rmsd  1.26 gap 24 (0.05%)

Chain 1:   43 TPPGAQPTAPGSLKAPDTRNEKLNSLEDVRKGSENYALTTNQGVRIADDQNSLRAGSRGPTLLEDFILRE
              1111111111111111              1111111111111111111111111111111111111111
Chain 2:    3 NRDPASDQMKHWKEQR--------------AAQKPDVLTTGGGNPVGDKLNSLTVGPRGPLLVQDVVFTD

Chain 1:  113 KITHFDHERIPERIVHARGSAAHGYFQPYKSLSDITKADFLSDPNKITPVFVRFSTCQGGAGSADTVRDI
              1111111111111111111111111111111111111111111111111111111111111111111111
Chain 2:   59 EMAHFDRERIPERVVHAKGAGAFGYFEVTHDITRYSKAKVFEHIGKRTPIAVRFSTVAGESGSADTVRDP

Chain 1:  183 RGFATKFYTEEGIFDLVGNNTPIFFIQDAHKFPDFVHAVKPEPHWAIPQGQSAHDTFWDYVSLQPETLHN
              111111111111111111111111111111111111111111111111    111111111111111111
Chain 2:  129 RGFAVKFYTEDGNWDLVGNNTPIFFIRDALLFPSFIHSQKRNPQTHLK----DPDMVWDFWSLRPESLHQ

Chain 1:  253 VMWAMSDRGIPRSYRTMEGFGIHTFRLINAEGKATFVRFHWKPLAGKASLVWDEAQKLTGRDPDFHRREL
              1111111111111111111111111111111111111111111111111111111111111111111111
Chain 2:  195 VSFLFSDRGIPDGHRHMNGYGSHTFKLVNANGEAVYCKFHYKTDQGIKNLSVEDAARLAHEDPDYGLRDL

Chain 1:  323 WEAIEAGDFPEYELGFQLIPEEDEFKFDFDLLDPTKLIPEELVPVQRVGKMVLNRNPDNFFAENEQAAFH
              1111111111111111111111111111111111111111111111111111111111111111111111
Chain 2:  265 FNAIATGNYPSWTLYIQVMTFSEAEIFPFNPFDLTKVWPHGDYPLIPVGKLVLNRNPVNYFAEVEQLAFD

Chain 1:  393 PGHIVPGLDFTNDPLLQGRLFSYTDTQISRLGGPNFHEIPINRPT-CPYHNFQRDGMHRMGI-DTNPANY
              11111111111111111111111111111111 111111111111 1111111111111111 1111111
Chain 2:  335 PSNMPPGIEPSPDKMLQGRLFAYPDTHRHRLG-PNYLQIPVNCPYRARVANYQRDGPMCMMDNQGGAPNY

Chain 1:  461 EPNSINDNWPRETPPGPKRGGFESYQERVEGNKVRERSPSFGEYYSHPRLFWL-SQTPFEQRHIVDGFSF
              1111  111111111      111111111111111111 1111111111111 1111111111111111
Chain 2:  404 YPNS--FSAPEHQPS------ALEHRTHFSGDVQRFNSA-NDDNVTQVRTFYLKVLNEEQRKRLCENIAG

Chain 1:  530 ELSKVVRPYIRERVVDQLAHIDLTLAQAVAKNLGIEL
              11111 1111111111111111111111111111111
Chain 2:  465 HLKDA-QLFIQKKAVKNFSDVHPEYGSRIQALLDKYN

Note: positions are from PDB; the numbers between alignments are block index
```

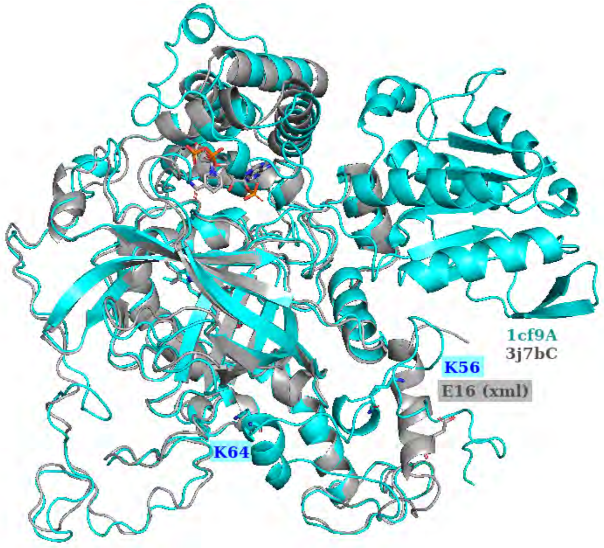

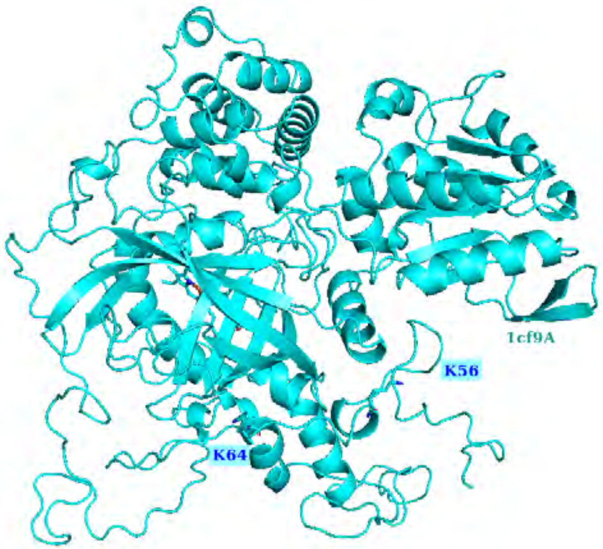

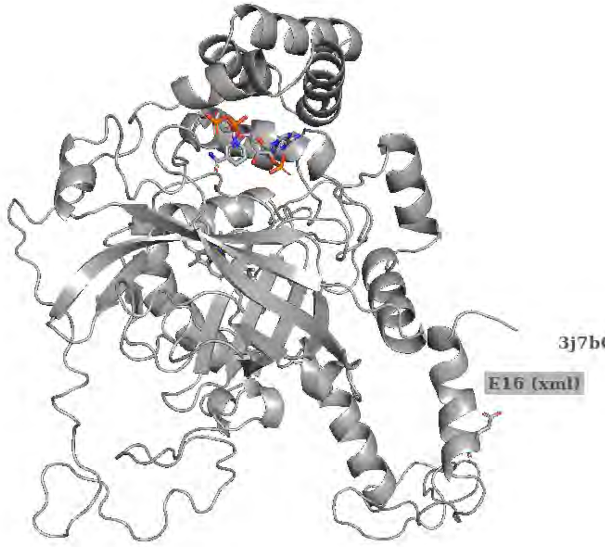

UniProt ID: P00432

PDB ID: 3NWL_C

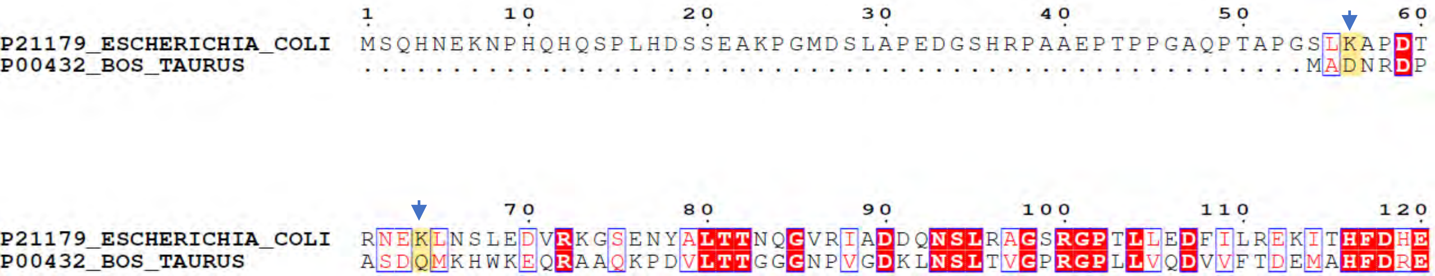

Full sequences in supplemental file.

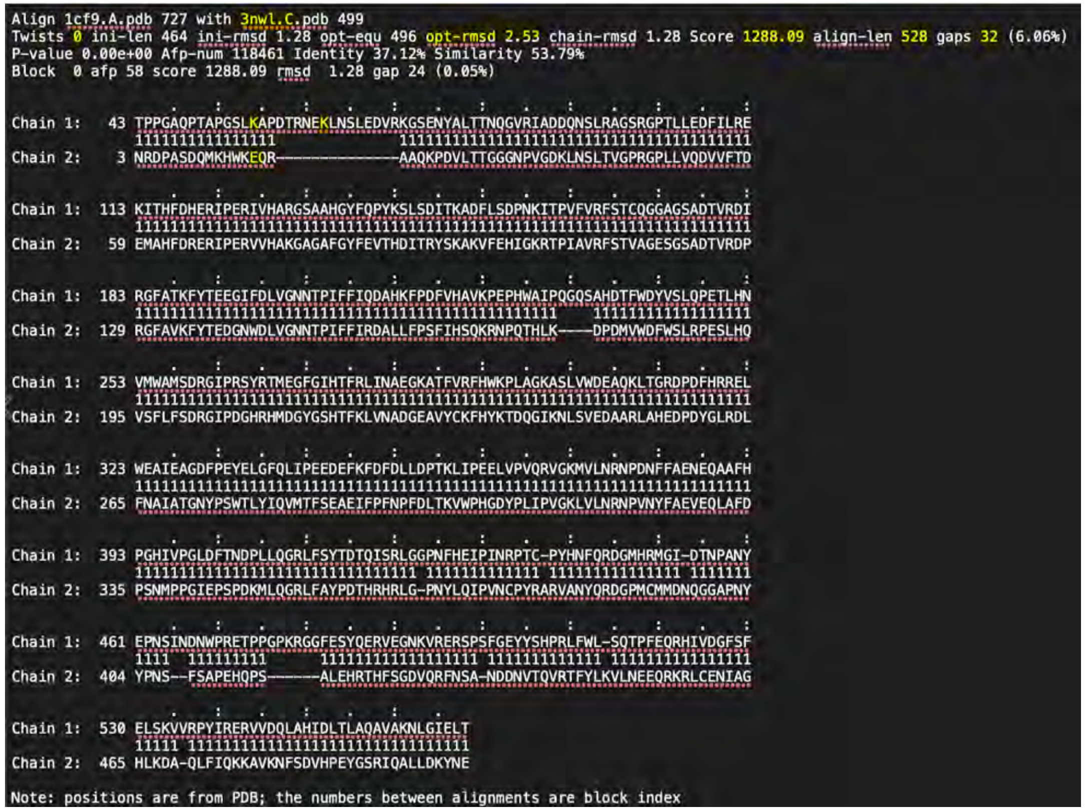

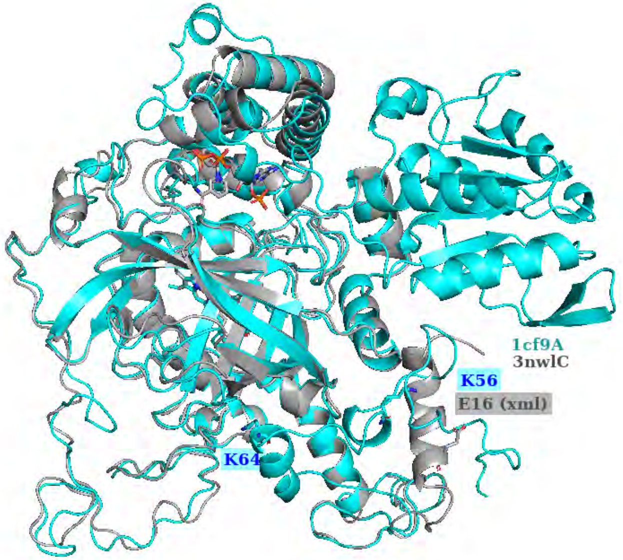

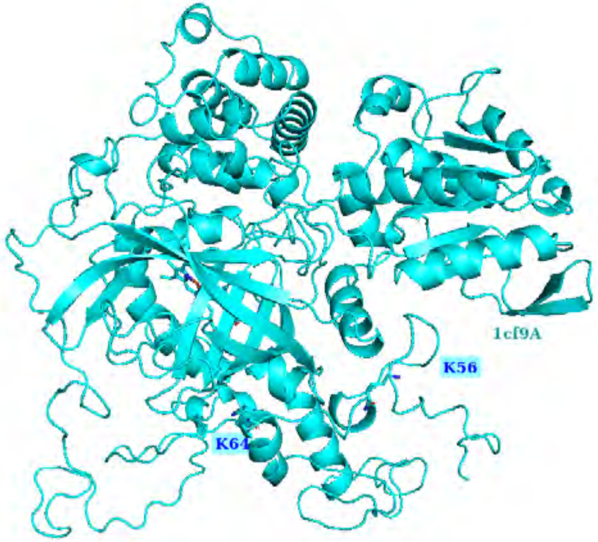

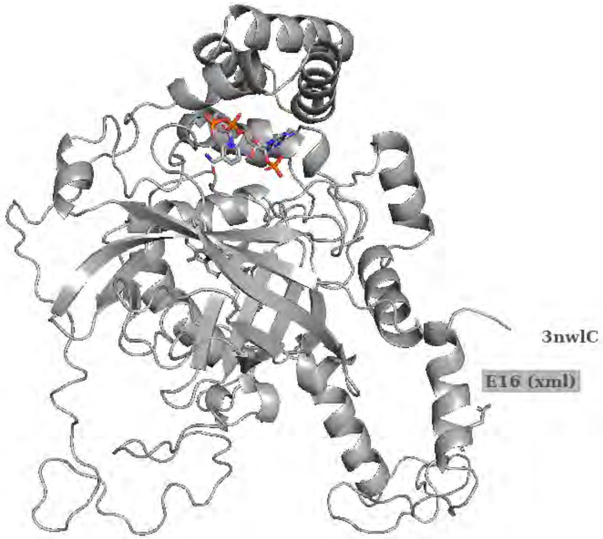

UniProt ID: P00432

PDB ID: 3RE8_C

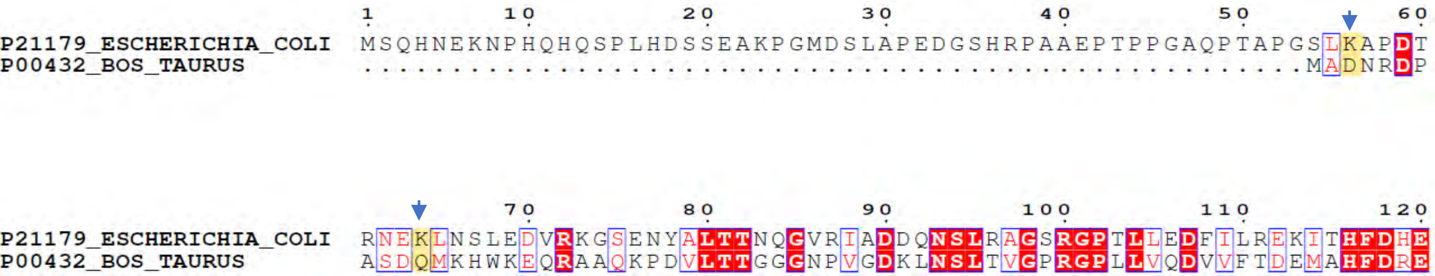

Full sequences in supplemental file.

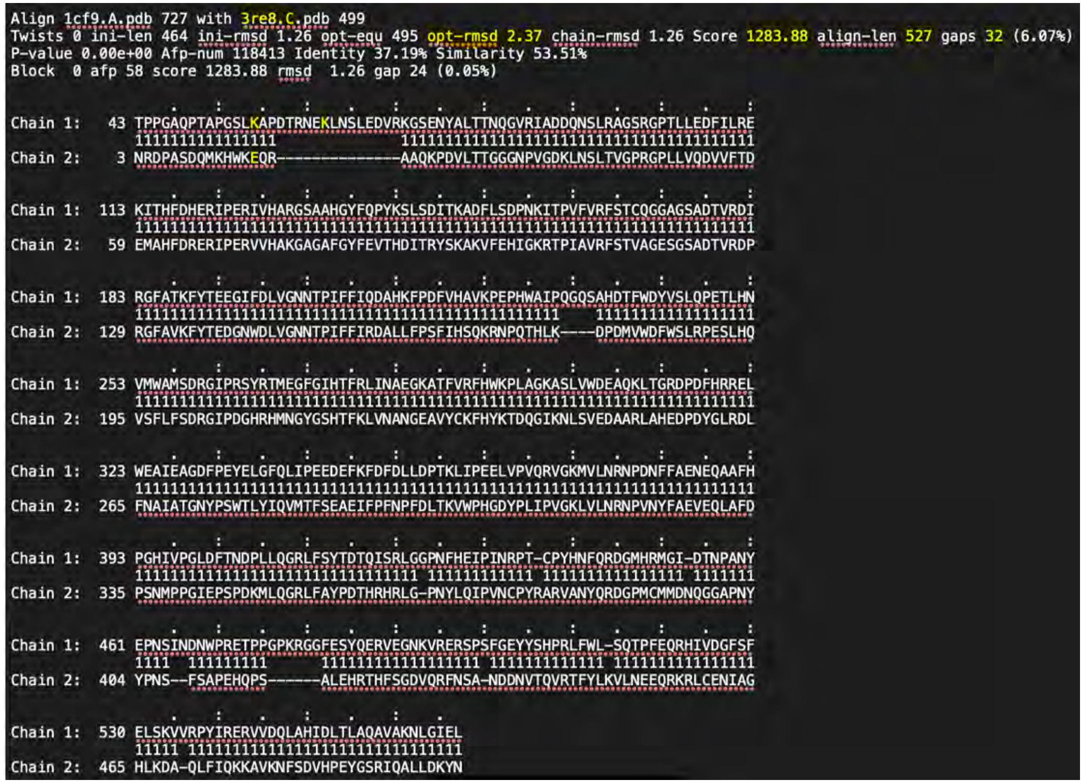

```
Align 1cf9.A.pdb 727 with 3re8.C.pdb 499
Twists 0 ini-len 464 ini-rmsd 1.26 opt-equ 495 opt-rmsd 2.37 chain-rmsd 1.26 Score 1283.88 align-len 527 gaps 32 (6.07%)
P-value 0.00e+00 Afp-num 118413 Identity 37.19% Similarity 53.51%
Block  0 afp 58 score 1283.88 rmsd  1.26 gap 24 (0.05%)

                  .    :    .    :    .    :    .    :    .    :    .    :    .    :
Chain 1:   43 TPPGAQPTAPGSLKAPDTRNEKLNSLEDVRKGSENYALTTNQGVRIADDQNSLRAGSRGPTLLEDFILRE
              1111111111111111              111111111111111111111111111111111111111
Chain 2:    3 NRDPASDQMKHWKEQR--------------AAQKPDVLTTGGGNPVGDKLNSLTVGPRGPLLVQDVVFTD

                  .    :    .    :    .    :    .    :    .    :    .    :    .    :
Chain 1:  113 KITHFDHERIPERIVHARGSAAHGYFQPYKSLSDITKADFLSDPNKITPVFVRFSTCQGGAGSADTVRDI
              1111111111111111111111111111111111111111111111111111111111111111111111
Chain 2:   59 EMAHFDRERIPERVVHAKGAGAFGYFEVTHDITRYSKAKVFEHIGKRTPIAVRFSTVAGESGSADTVRDP

                  .    :    .    :    .    :    .    :    .    :    .    :    .    :
Chain 1:  183 RGFATKFYTEEGIFDLVGNNTPIFFIQDAHKFPDFVHAVKPEPHWAIPQGQSAHDTFWDYVSLQPETLHN
              11111111111111111111111111111111111111111111111111    111111111111111111
Chain 2:  129 RGFAVKFYTEDGNWDLVGNNTPIFFIRDALLFPSFIHSQKRNPQTHLK----DPDMVWDFWSLRPESLHQ

                  .    :    .    :    .    :    .    :    .    :    .    :    .    :
Chain 1:  253 VMWAMSDRGIPRSYRTMEGFGIHTFRLINAEGKATFVRFHWKPLAGKASLVWDEAQKLTGRDPDFHRREL
              1111111111111111111111111111111111111111111111111111111111111111111111
Chain 2:  195 VSFLFSDRGIPDGHRHMNGYGSHTFKLVNANGEAVYCKFHYKTDQGIKNLSVEDAARLAHEDPDYGLRDL

                  .    :    .    :    .    :    .    :    .    :    .    :    .    :
Chain 1:  323 WEAIEAGDFPEYELGFQLIPEEDEFKFDFDLLDPTKLIPEELVPVQRVGKMVLNRNPDNFFAENEQAAFH
              1111111111111111111111111111111111111111111111111111111111111111111111
Chain 2:  265 FNAIATGNYPSWTLYIQVMTFSEAEIFPFNPFDLTKVWPHGDYPLIPVGKLVLNRNPVNYFAEVEQLAFD

                  .    :    .    :    .    :    .    :    .    :    .    :    .    :
Chain 1:  393 PGHIVPGLDFTNDPLLQGRLFSYTDTQISRLGGPNFHEIPINRPT-CPYHNFQRDGMHRMGI-DTNPANY
              11111111111111111111111111111111 111111111111 1111111111111111 1111111
Chain 2:  335 PSNMPPGIEPSPDKMLQGRLFAYPDTHRHRLG-PNYLQIPVNCPYRARVANYQRDGPMCMMDNQGGAPNY

                  .    :    .    :    .    :    .    :    .    :    .    :    .    :
Chain 1:  461 EPNSINDNWPRETPPGPKRGGFESYQERVEGNKVRERSPSFGEYYSHPRLFWL-SQTPFEQRHIVDGFSF
              1111  111111111      111111111111111111 11111111111111 1111111111111111
Chain 2:  404 YPNS--FSAPEHQPS------ALEHRTHFSGDVQRFNSA-NDDNVTQVRTFYLKVLNEEQRKRLCENIAG

                  .    :    .    :    .    :    .
Chain 1:  530 ELSKVVRPYIRERVVDQLAHIDLTLAQAVAKNLGIEL
              11111 11111111111111111111111111111111
Chain 2:  465 HLKDA-QLFIQKKAVKNFSDVHPEYGSRIQALLDKYN
```

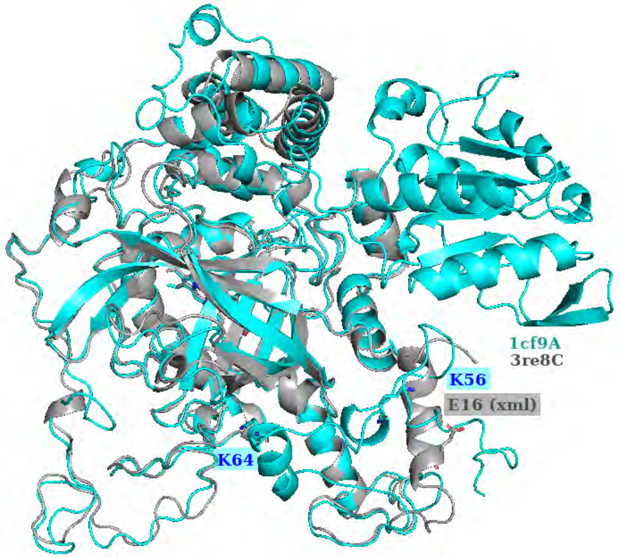

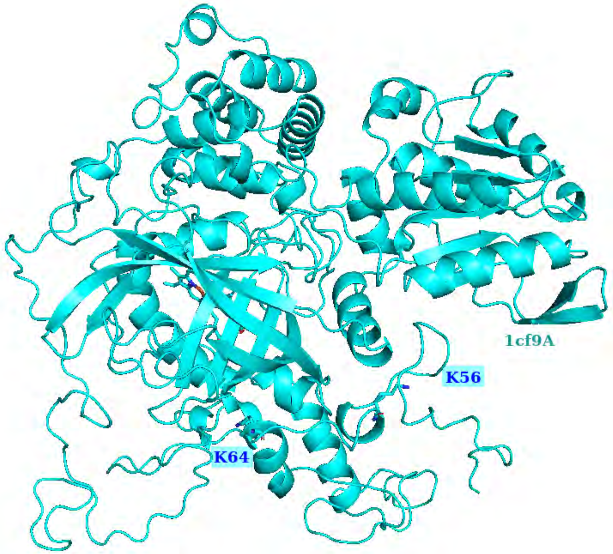

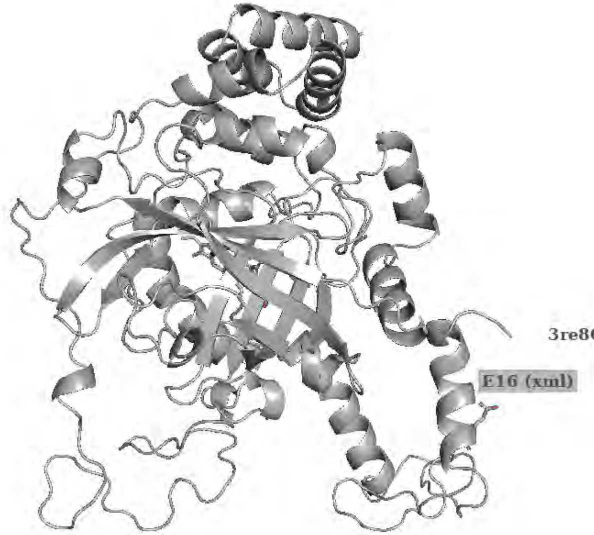

UniProt ID: P00432

PDB ID: 3RGP_D

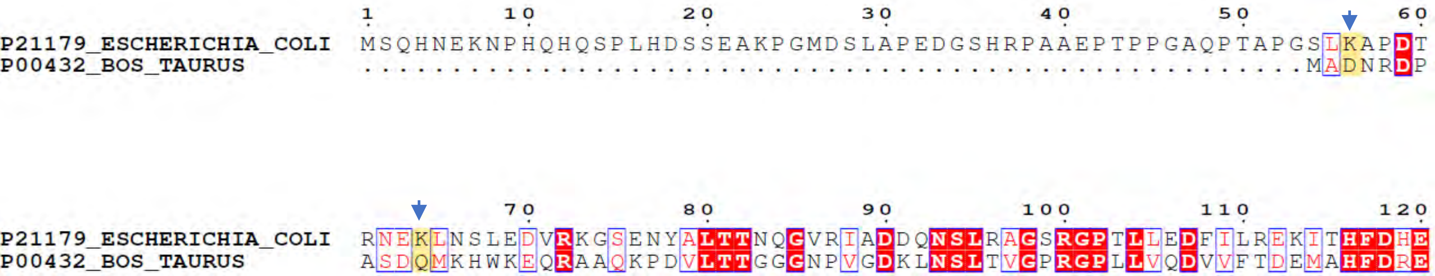

Full sequences in supplemental file.

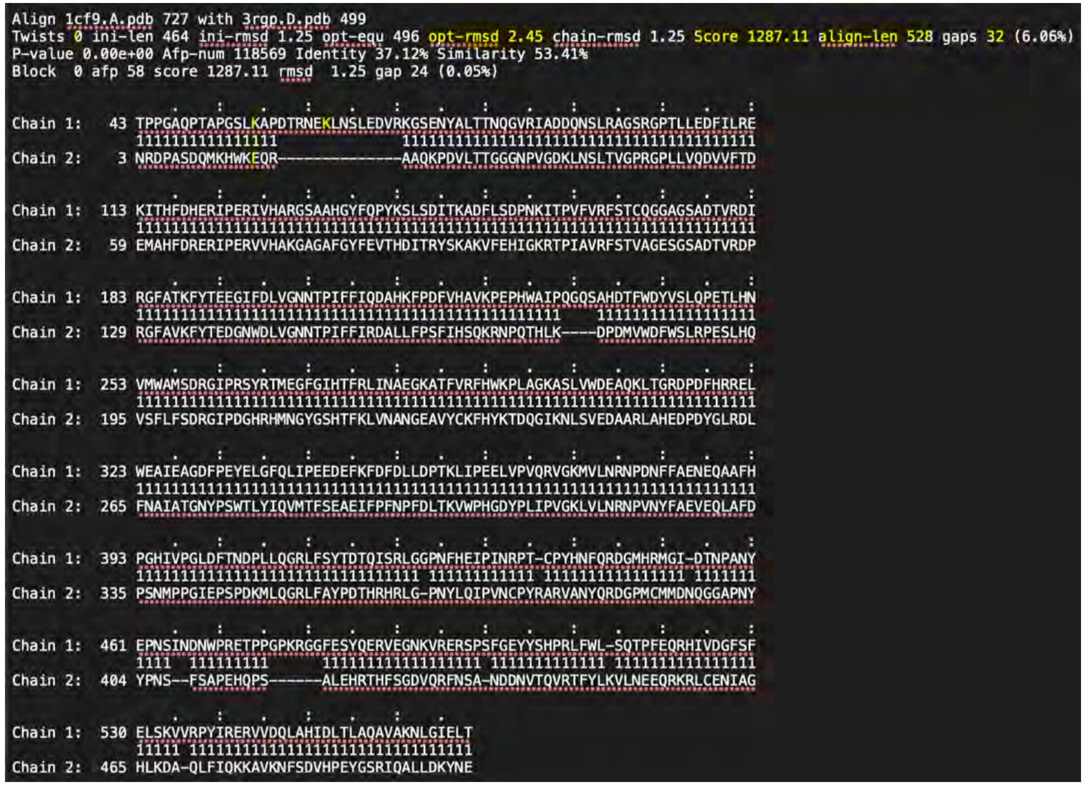

```
Align 1cf9.A.pdb 727 with 3rgp.D.pdb 499
Twists 0 ini-len 464 ini-rmsd 1.25 opt-equ 496 opt-rmsd 2.45 chain-rmsd 1.25 Score 1287.11 align-len 528 gaps 32 (6.06%)
P-value 0.00e+00 Afp-num 118569 Identity 37.12% Similarity 53.41%
Block  0 afp 58 score 1287.11 rmsd  1.25 gap 24 (0.05%)

Chain 1:   43 TPPGAQPTAPGSLKAPDTRNEKLNSLEDVRKGSENYALTTNQGVRIADDQNSLRAGSRGPTLLEDFILRE
Chain 2:    3 NRDPASDQMKHWKEQR--------------AAQKPDVLTTGGGNPVGDKLNSLTVGPRGPLLVQDVVFTD

Chain 1:  113 KITHFDHERIPERIVHARGSAAHGYFQPYKSLSDITKADFLSDPNKITPVFVRFSTCQGGAGSADTVRDI
Chain 2:   59 EMAHFDRERIPERVVHAKGAGAFGYFEVTHDITRYSKAKVFEHIGKRTPIAVRFSTVAGESGSADTVRDP

Chain 1:  183 RGFATKFYTEEGIFDLVGNNTPIFFIQDAHKFPDFVHAVKPEPHWAIPQGQSAHDTFWDYVSLQPETLHN
Chain 2:  129 RGFAVKFYTEDGNWDLVGNNTPIFFIRDALLFPSFIHSQKRNPQTHLK----DPDMVWDFWSLRPESLHQ

Chain 1:  253 VMWAMSDRGIPRSYRTMEGFGIHTFRLINAEGKATFVRFHWKPLAGKASLVWDEAQKLTGRDPDFHRREL
Chain 2:  195 VSFLFSDRGIPDGHRHMNGYGSHTFKLVNANGEAVYCKFHYKTDQGIKNLSVEDAARLAHEDPDYGLRDL

Chain 1:  323 WEAIEAGDFPEYELGFQLIPEEDEFKFDFDLLDPTKLIPEELVPVQRVGKMVLNRNPDNFFAENEQAAFH
Chain 2:  265 FNAIATGNYPSWTLYIQVMTFSEAEIFPFNPFDLTKVWPHGDYPLIPVGKLVLNRNPVNYFAEVEQLAFD

Chain 1:  393 PGHIVPGLDFTNDPLLQGRLFSYTDTQISRLGGPNFHEIPINRPT-CPYHNFQRDGMHRMGI-DTNPANY
Chain 2:  335 PSNMPPGIEPSPDKMLQGRLFAYPDTHRHRLG-PNYLQIPVNCPYRARVANYQRDGPMCMMDNQGGAPNY

Chain 1:  461 EPNSINDNWPRETPPGPKRGGFESYQERVEGNKVRERSPSFGEYYSHPRLFWL-SQTPFEQRHIVDGFSF
Chain 2:  404 YPNS--FSAPEHQPS------ALEHRTHFSGDVQRFNSA-NDDNVTQVRTFYLKVLNEEQRKRLCENIAG

Chain 1:  530 ELSKVVRPYIRERVVDQLAHIDLTLAQAVAKNLGIELT
Chain 2:  465 HLKDA-QLFIQKKAVKNFSDVHPEYGSRIQALLDKYNE
```

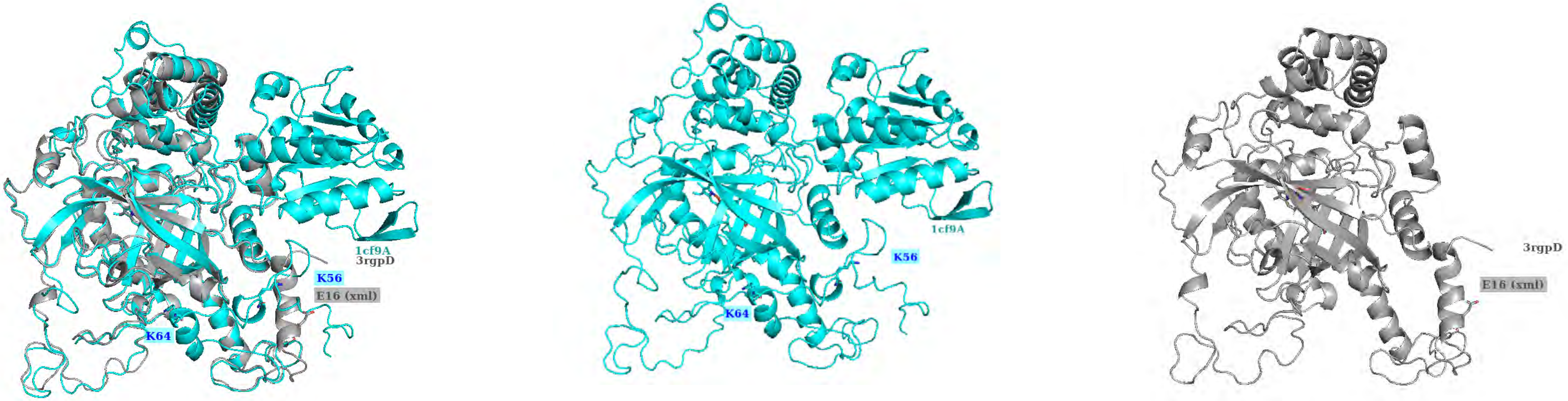

UniProt ID: P00432

PDB ID: 3RGS_C

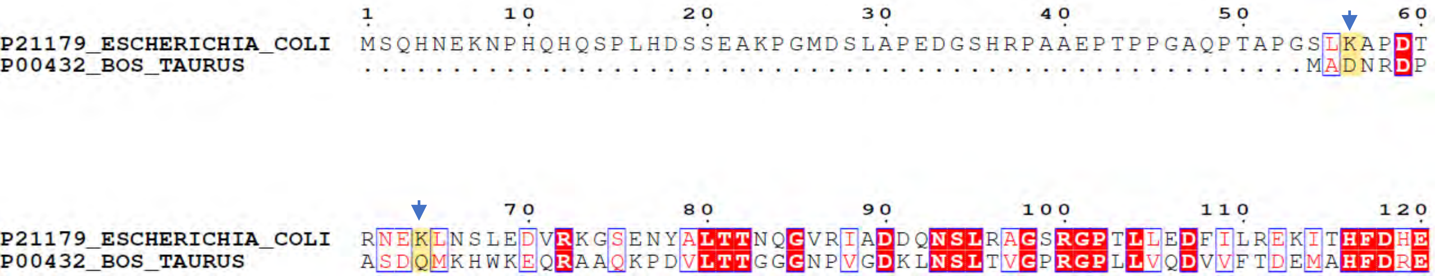

Full sequences in supplemental file.

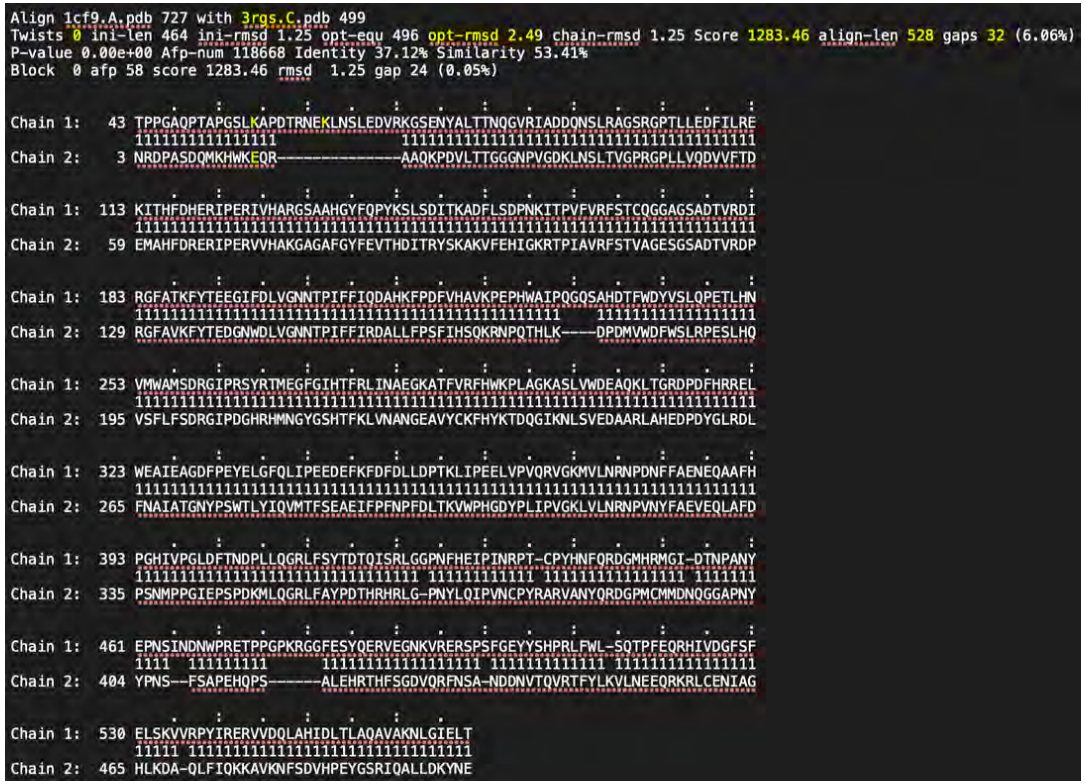

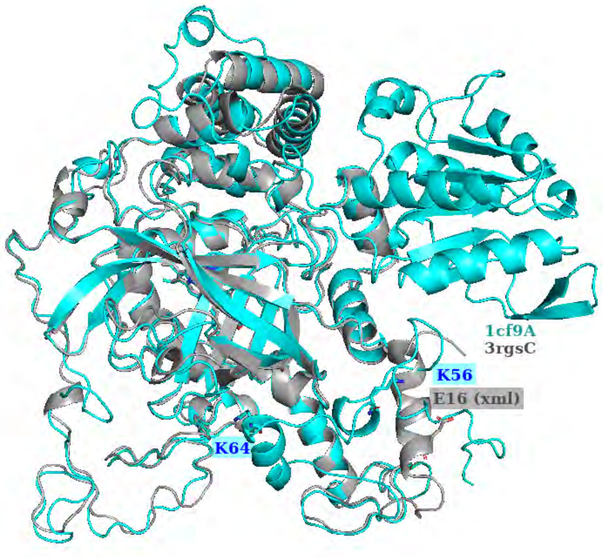

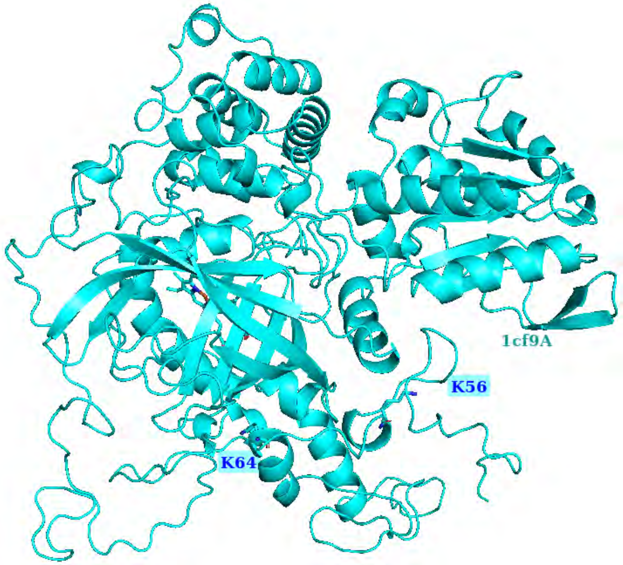

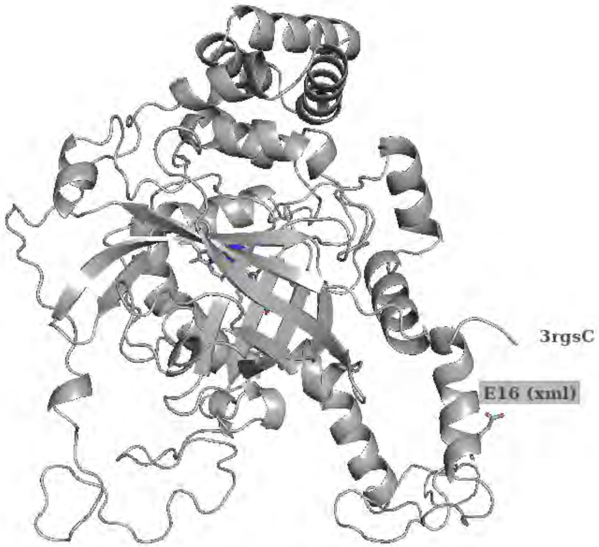

UniProt ID: P00432

PDB ID: 4BLC_A

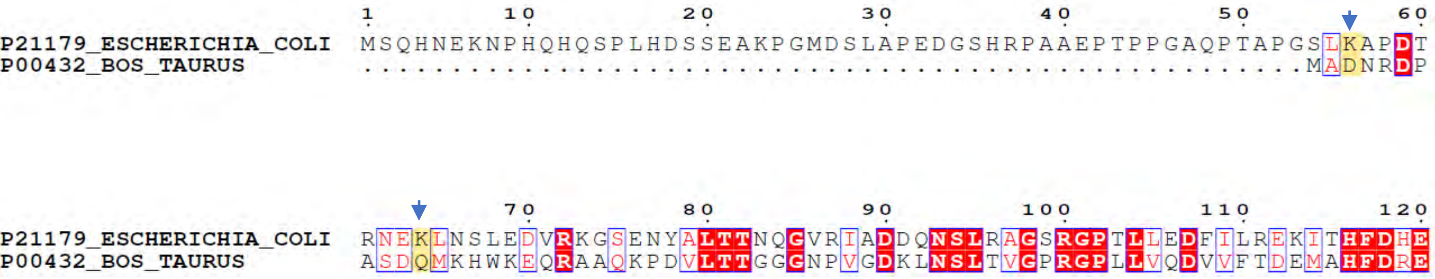

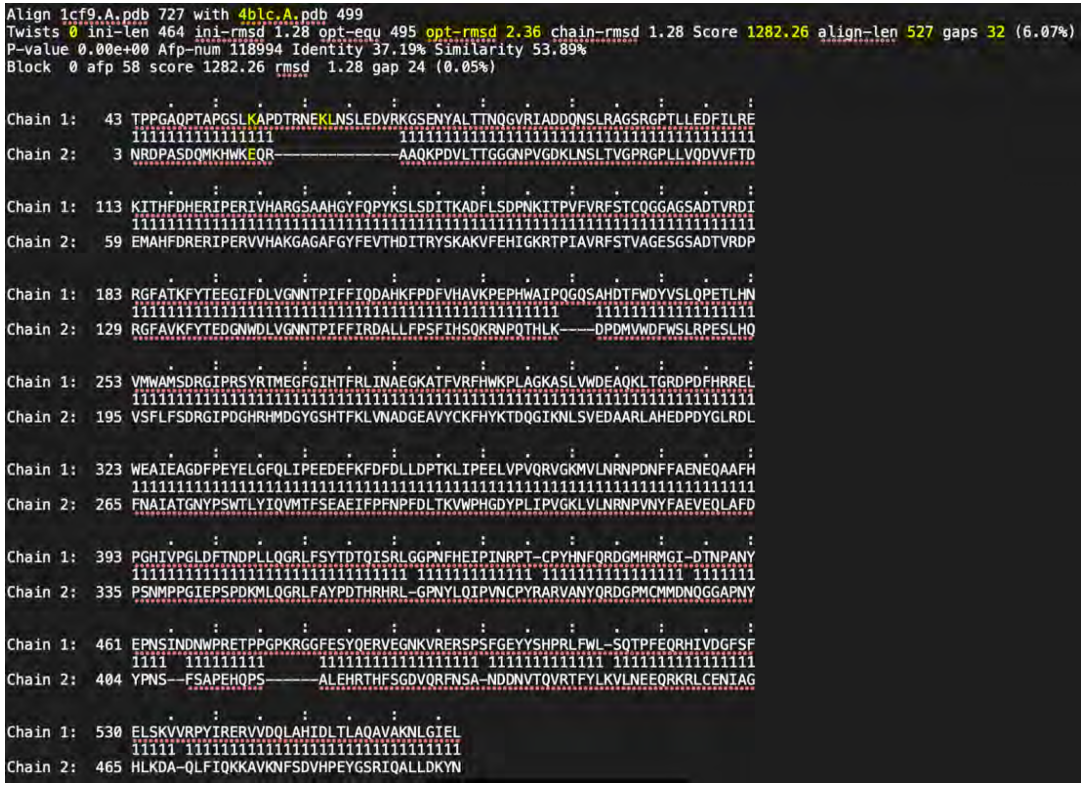

Full sequences in supplemental file.

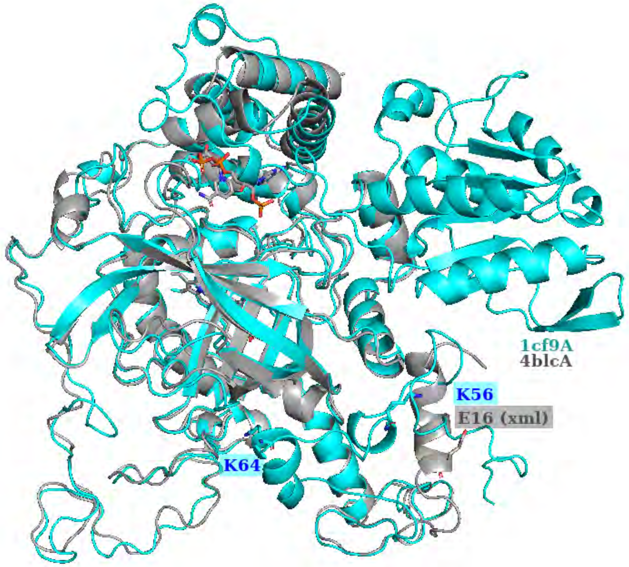

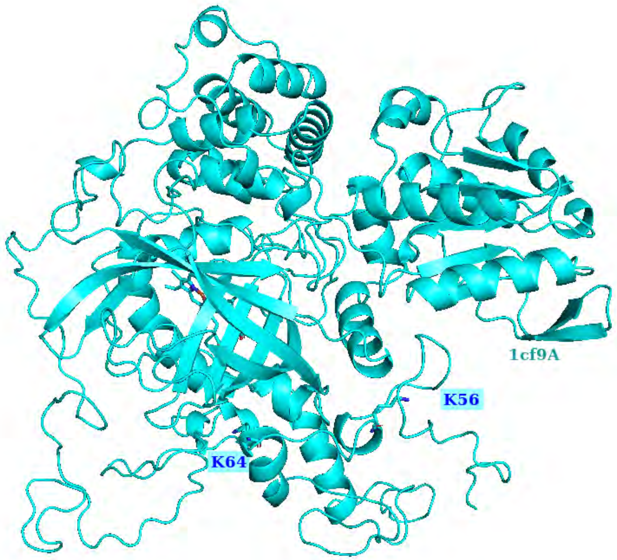

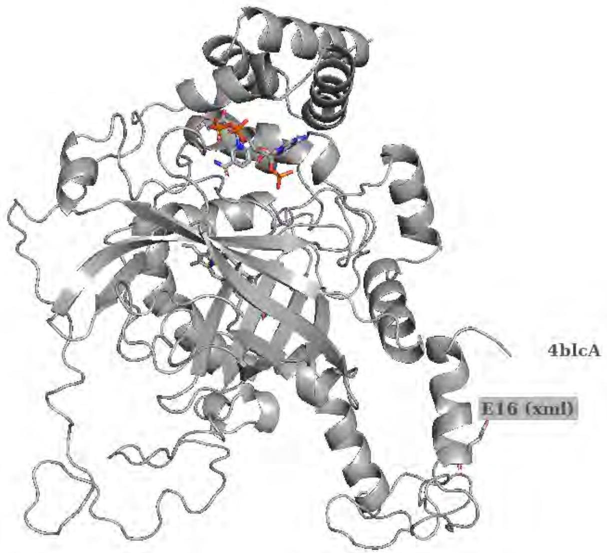

UniProt ID: P00432
PDB ID: 5GKN_C

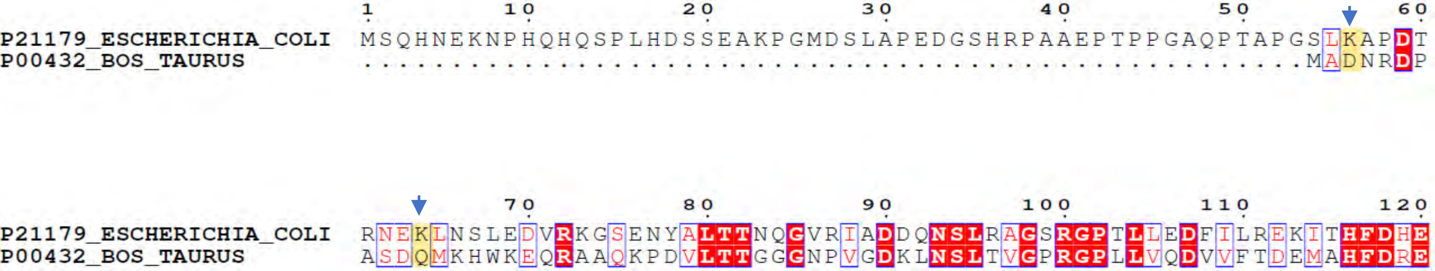

Full sequences in supplemental file.

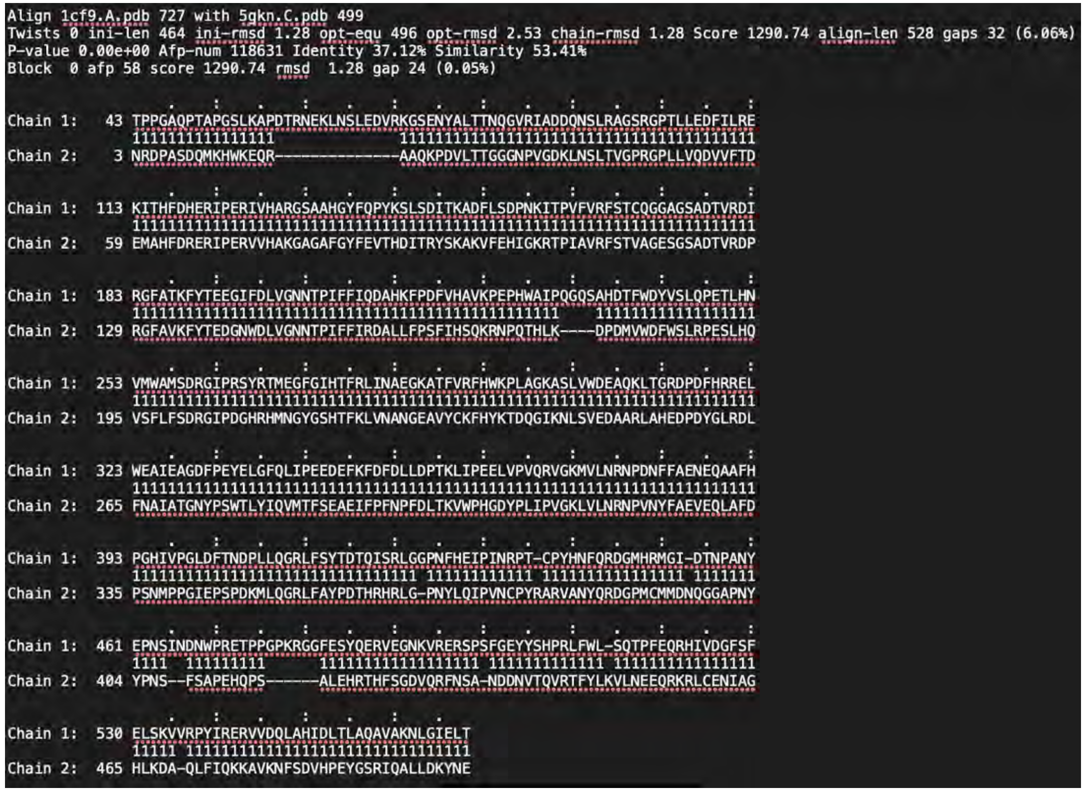

```
Align 1cf9.A.pdb 727 with 5gkn.C.pdb 499
Twists 0 ini-len 464 ini-rmsd 1.28 opt-equ 496 opt-rmsd 2.53 chain-rmsd 1.28 Score 1290.74 align-len 528 gaps 32 (6.06%)
P-value 0.00e+00 Afp-num 118631 Identity 37.12% Similarity 53.41%
Block  0 afp 58 score 1290.74 rmsd  1.28 gap 24 (0.05%)

                 .    :    .    :    .    :    .    :    .    :    .    :    .    :
Chain 1:   43 TPPGAQPTAPGSLKAPDTRNEKLNSLEDVRKGSENYALTTNQGVRIADDQNSLRAGSRGPTLLEDFILRE
              1111111111111111              111111111111111111111111111111111111111111
Chain 2:    3 NRDPASDQMKHWKEQR--------------AAQKPDVLTTGGGNPVGDKLNSLTVGPRGPLLVQDVVFTD

                 .    :    .    :    .    :    .    :    .    :    .    :    .    :
Chain 1:  113 KITHFDHERIPERIVHARGSAAHGYFQPYKSLSDITKADFLSDPNKITPVFVRFSTCQGGAGSADTVRDI
              1111111111111111111111111111111111111111111111111111111111111111111111
Chain 2:   59 EMAHFDRERIPERVVHAKGAGAFGYFEVTHDITRYSKAKVFEHIGKRTPIAVRFSTVAGESGSADTVRDP

                 .    :    .    :    .    :    .    :    .    :    .    :    .    :
Chain 1:  183 RGFATKFYTEEGIFDLVGNNTPIFFIQDAHKFPDFVHAVKPEPHWAIPQGQSAHDTFWDYVSLQPETLHN
              11111111111111111111111111111111111111111111111111    111111111111111111
Chain 2:  129 RGFAVKFYTEDGNWDLVGNNTPIFFIRDALLFPSFIHSQKRNPQTHLK----DPDMVWDFWSLRPESLHQ

                 .    :    .    :    .    :    .    :    .    :    .    :    .    :
Chain 1:  253 VMWAMSDRGIPRSYRTMEGFGIHTFRLINAEGKATFVRFHWKPLAGKASLVWDEAQKLTGRDPDFHRREL
              1111111111111111111111111111111111111111111111111111111111111111111111
Chain 2:  195 VSFLFSDRGIPDGHRHMNGYGSHTFKLVNANGEAVYCKFHYKTDQGIKNLSVEDAARLAHEDPDYGLRDL

                 .    :    .    :    .    :    .    :    .    :    .    :    .    :
Chain 1:  323 WEAIEAGDFPEYELGFQLIPEEDEFKFDFDLLDPTKLIPEELVPVQRVGKMVLNRNPDNFFAENEQAAFH
              1111111111111111111111111111111111111111111111111111111111111111111111
Chain 2:  265 FNAIATGNYPSWTLYIQVMTFSEAEIFPFNPFDLTKVWPHGDYPLIPVGKLVLNRNPVNYFAEVEQLAFD

                 .    :    .    :    .    :    .    :    .    :    .    :    .    :
Chain 1:  393 PGHIVPGLDFTNDPLLQGRLFSYTDTQISRLGGPNFHEIPINRPT-CPYHNFQRDGMHRMGI-DTNPANY
              11111111111111111111111111111111 1111111111 111111111111111 1111111
Chain 2:  335 PSNMPPGIEPSPDKMLQGRLFAYPDTHRHRLG-PNYLQIPVNCPYRARVANYQRDGPMCMMDNQGGAPNY

                 .    :    .    :    .    :    .    :    .    :    .    :    .    :
Chain 1:  461 EPNSINDNWPRETPPGPKRGGFESYQERVEGNKVRERSPSFGEYYSHPRLFWL-SQTPFEQRHIVDGFSF
              1111  111111111      1111111111111111111 111111111111 111111111111111111
Chain 2:  404 YPNS--FSAPEHQPS------ALEHRTHFSGDVQRFNSA-NDDNVTQVRTFYLKVLNEEQRKRLCENIAG

                 .    :    .    :    .    :    .
Chain 1:  530 ELSKVVRPYIRERVVDQLAHIDLTLAQAVAKNLGIELT
              11111 11111111111111111111111111111111
Chain 2:  465 HLKDA-QLFIQKKAVKNFSDVHPEYGSRIQALLDKYNE
```

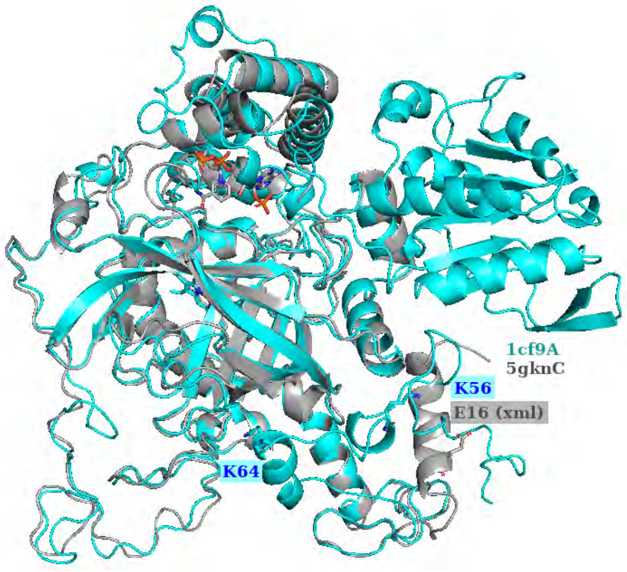

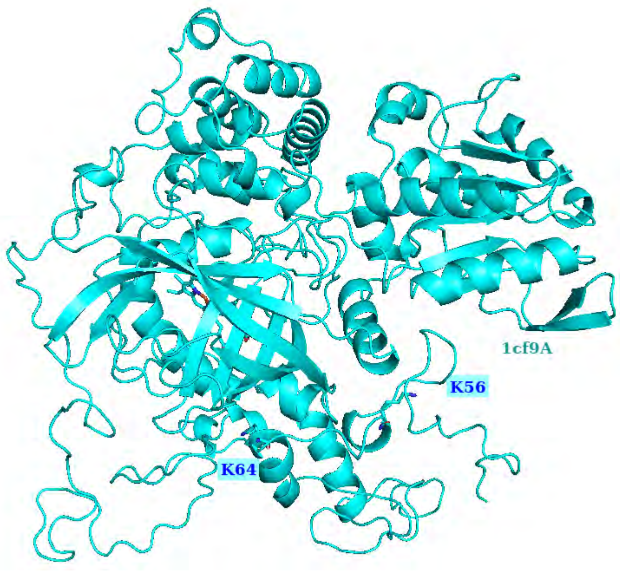

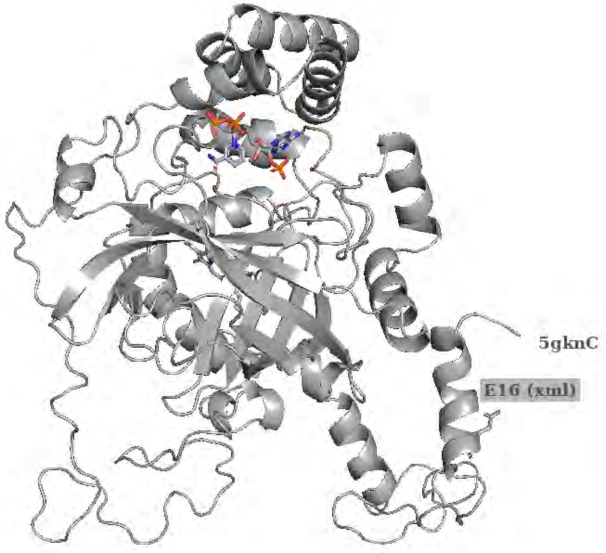

UniProt ID: P00432
PDB ID: 6JNT_A

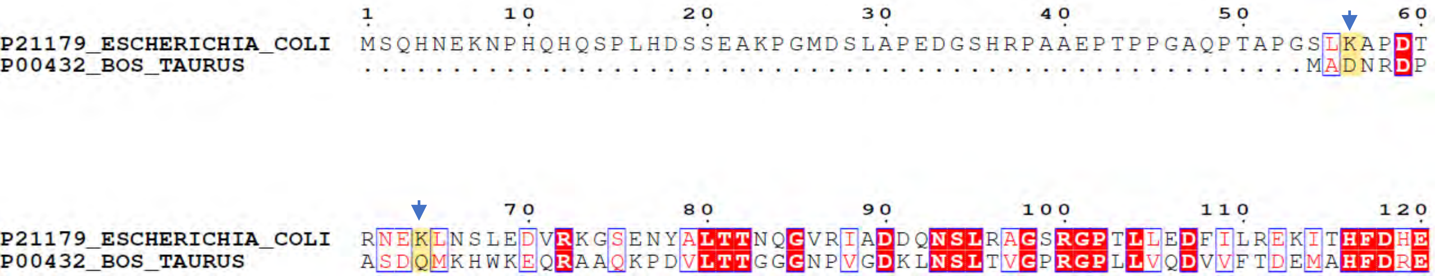

Full sequences in supplemental file.

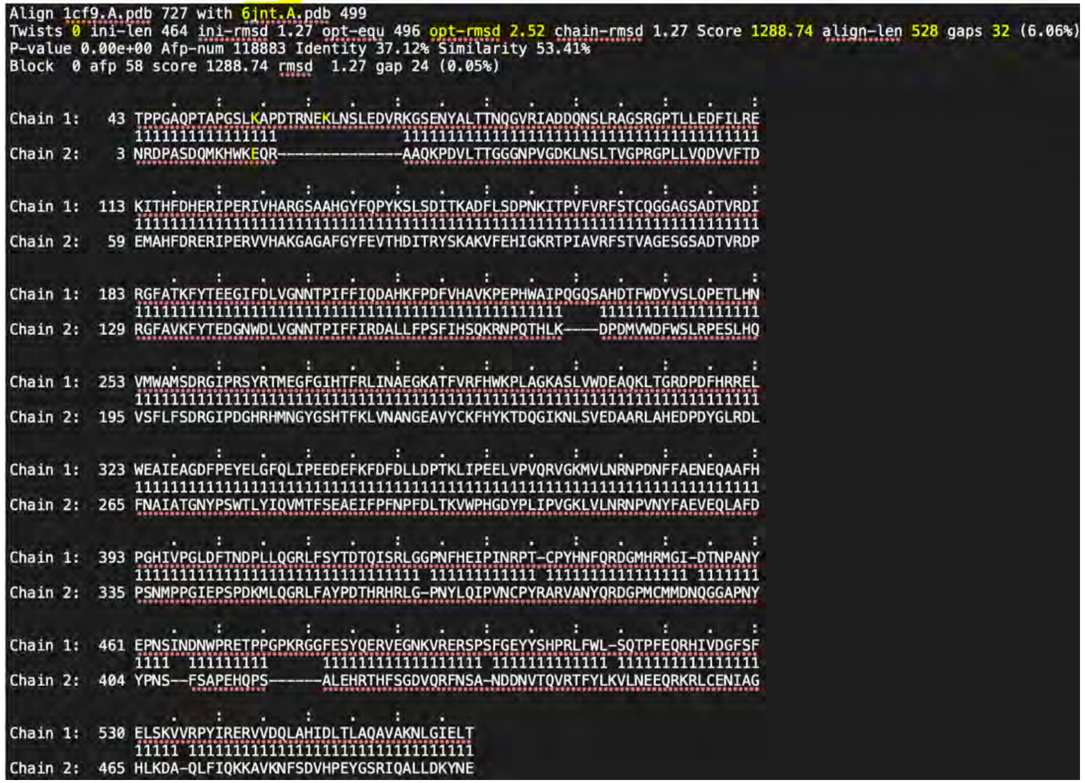

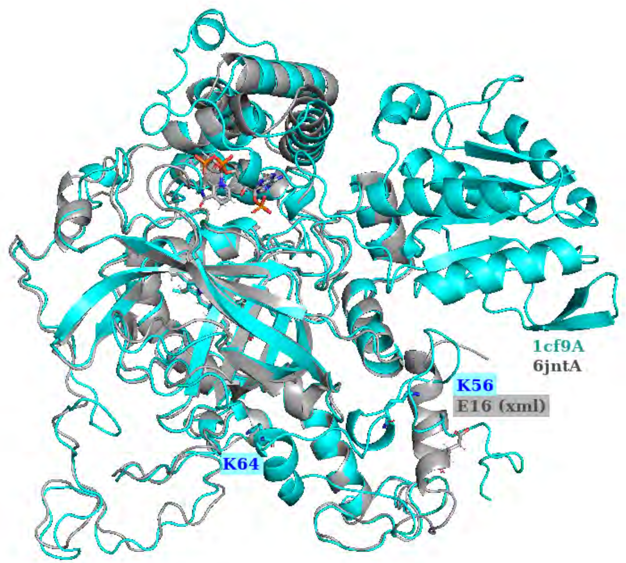

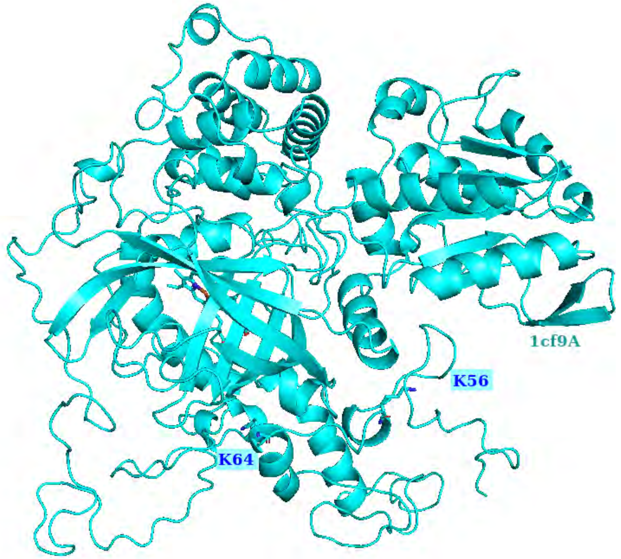

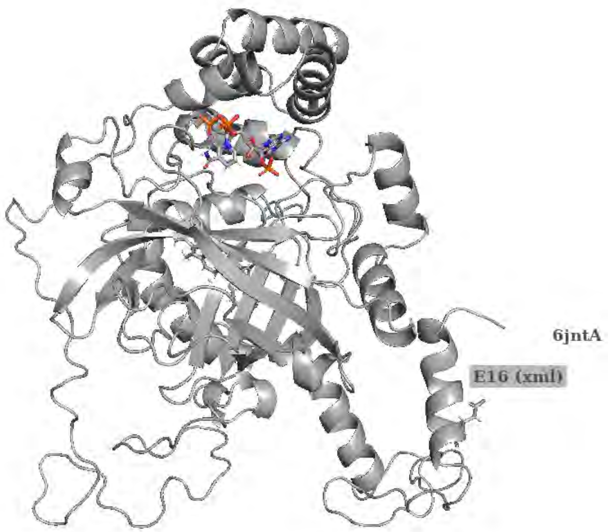

UniProt ID: P00432

PDB ID: 6JNU_C

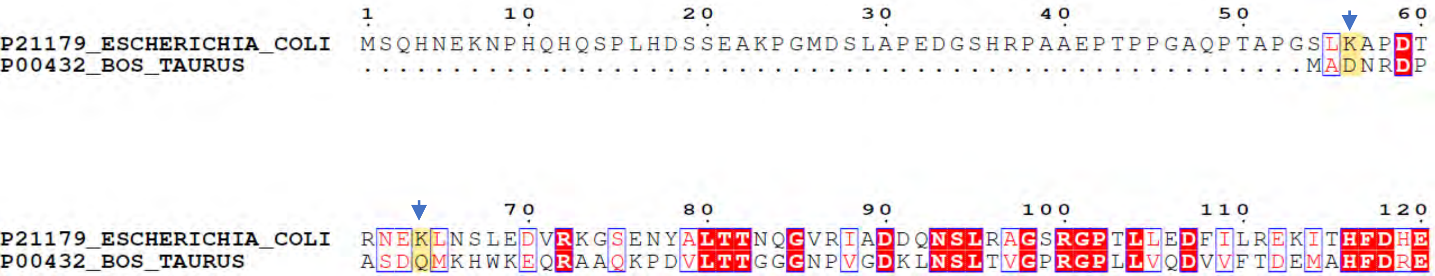

Full sequences in supplemental file.

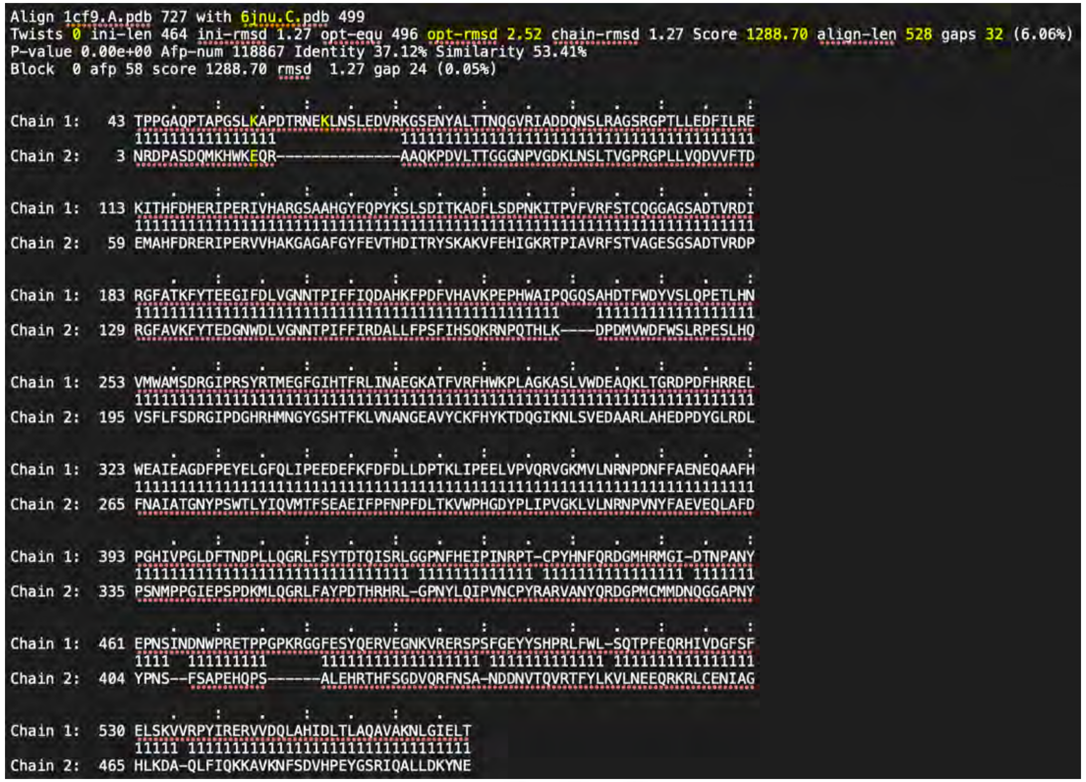

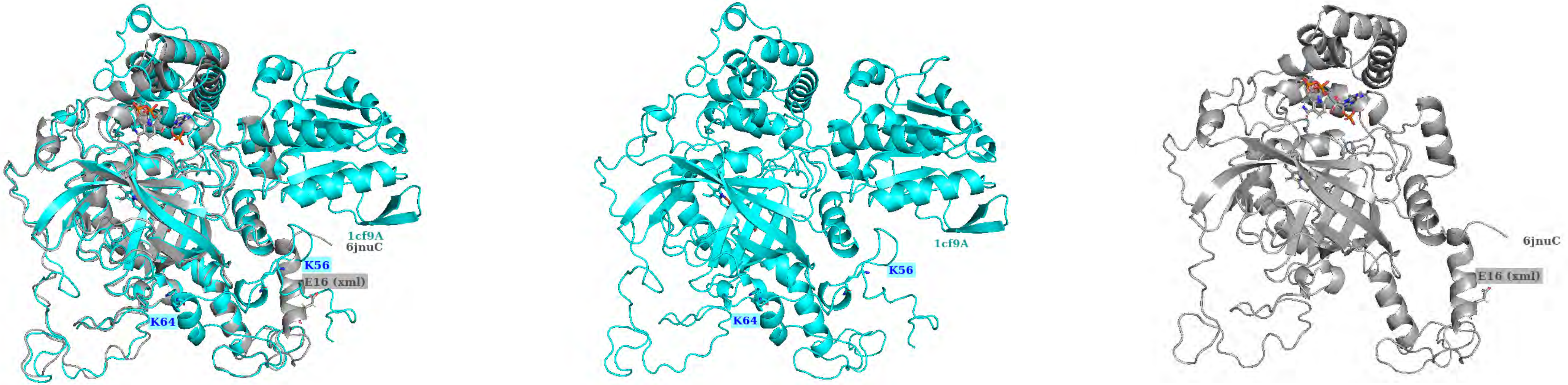

UniProt ID: P00432
PDB ID: 6PM7_B

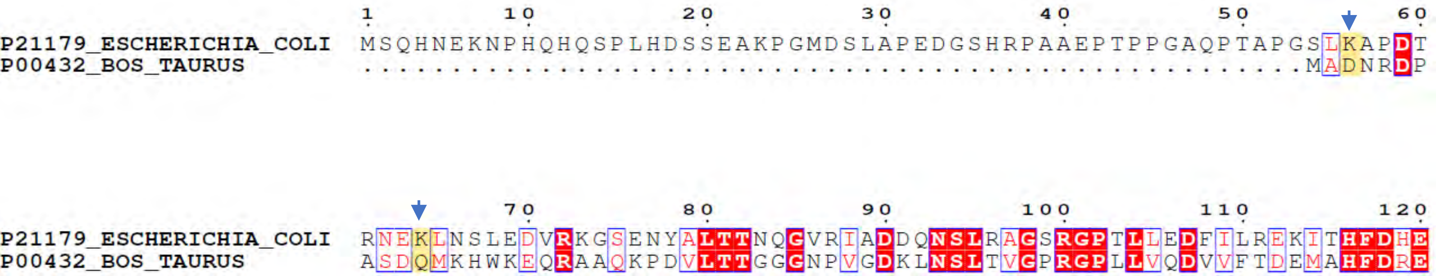

Full sequences in supplemental file.

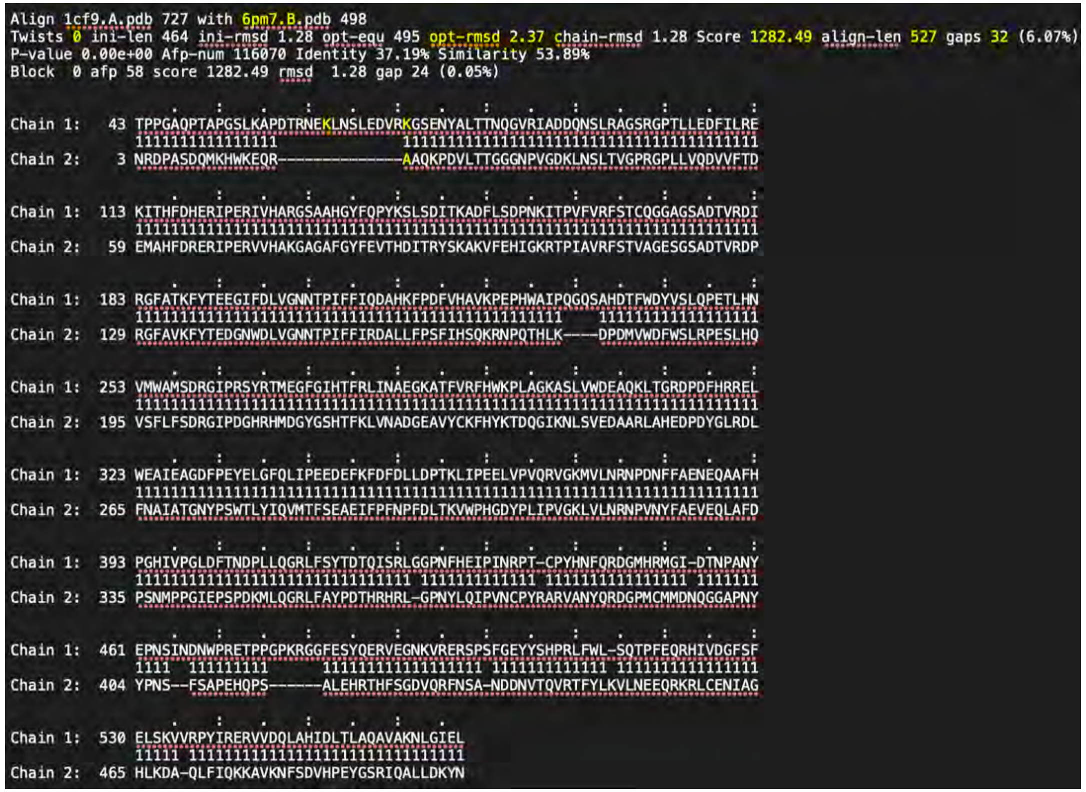

```
Align 1cf9.A.pdb 727 with 6pm7.B.pdb 498
Twists 0 ini-len 464 ini-rmsd 1.28 opt-equ 495 opt-rmsd 2.37 chain-rmsd 1.28 Score 1282.49 align-len 527 gaps 32 (6.07%)
P-value 0.00e+00 Afp-num 116070 Identity 37.19% Similarity 53.89%
Block  0 afp 58 score 1282.49 rmsd  1.28 gap 24 (0.05%)

Chain 1:   43 TPPGAQPTAPGSLKAPDTRNEKLNSLEDVRKGSENYALTTNQGVRIADDQNSLRAGSRGPTLLEDFILRE
              1111111111111111              111111111111111111111111111111111111111
Chain 2:    3 NRDPASDQMKHWKEQR--------------AAQKPDVLTTGGGNPVGDKLNSLTVGPRGPLLVQDVVFTD

Chain 1:  113 KITHFDHERIPERIVHARGSAAHGYFQPYKSLSDITKADFLSDPNKITPVFVRFSTCQGGAGSADTVRDI
              1111111111111111111111111111111111111111111111111111111111111111111111
Chain 2:   59 EMAHFDRERIPERVVHAKGAGAFGYFEVTHDITRYSKAKVFEHIGKRTPIAVRFSTVAGESGSADTVRDP

Chain 1:  183 RGFATKFYTEEGIFDLVGNNTPIFFIQDAHKFPDFVHAVKPEPHWAIPQGQSAHDTFWDYVSLQPETLHN
              11111111111111111111111111111111111111111111111111    111111111111111111
Chain 2:  129 RGFAVKFYTEDGNWDLVGNNTPIFFIRDALLFPSFIHSQKRNPQTHLK----DPDMVWDFWSLRPESLHQ

Chain 1:  253 VMWAMSDRGIPRSYRTMEGFGIHTFRLINAEGKATFVRFHWKPLAGKASLVWDEAQKLTGRDPDFHRREL
              1111111111111111111111111111111111111111111111111111111111111111111111
Chain 2:  195 VSFLFSDRGIPDGHRHMDGYGSHTFKLVNADGEAVYCKFHYKTDQGIKNLSVEDAARLAHEDPDYGLRDL

Chain 1:  323 WEAIEAGDFPEYELGFQLIPEEDEFKFDFDLLDPTKLIPEELVPVQRVGKMVLNRNPDNFFAENEQAAFH
              1111111111111111111111111111111111111111111111111111111111111111111111
Chain 2:  265 FNAIATGNYPSWTLYIQVMTFSEAEIFPFNPFDLTKVWPHGDYPLIPVGKLVLNRNPVNYFAEVEQLAFD

Chain 1:  393 PGHIVPGLDFTNDPLLQGRLFSYTDTQISRLGGPNFHEIPINRPT-CPYHNFQRDGMHRMGI-DTNPANY
              1111111111111111111111111111111 111111111111 11111111111111 1111111
Chain 2:  335 PSNMPPGIEPSPDKMLQGRLFAYPDTHRHRL-GPNYLQIPVNCPYRARVANYQRDGPMCMMDNQGGAPNY

Chain 1:  461 EPNSINDNWPRETPPGPKRGGFESYQERVEGNKVRERSPSFGEYYSHPRLFWL-SQTPFEQRHIVDGFSF
              1111  111111111      11111111111111111 1111111111111 111111111111111
Chain 2:  404 YPNS--FSAPEHQPS------ALEHRTHFSGDVQRFNSA-NDDNVTQVRTFYLKVLNEEQRKRLCENIAG

Chain 1:  530 ELSKVVRPYIRERVVDQLAHIDLTLAQAVAKNLGIEL
              11111 1111111111111111111111111111111
Chain 2:  465 HLKDA-QLFIQKKAVKNFSDVHPEYGSRIQALLDKYN
```

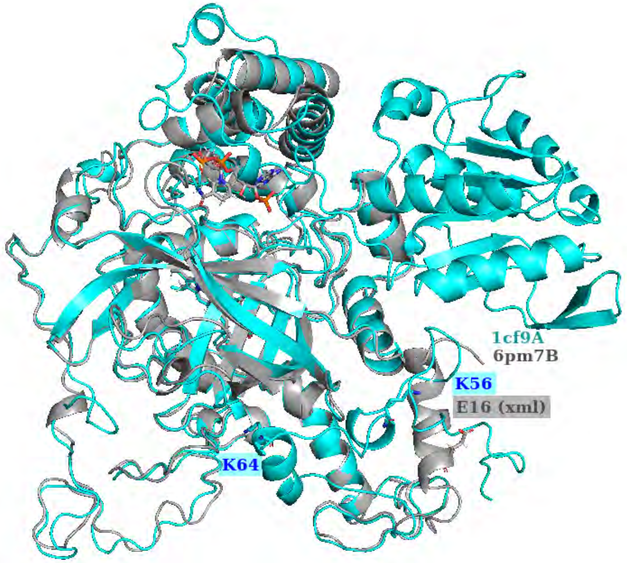

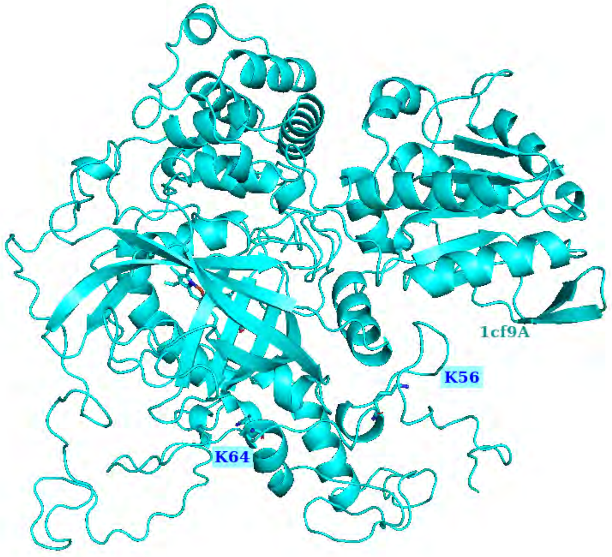

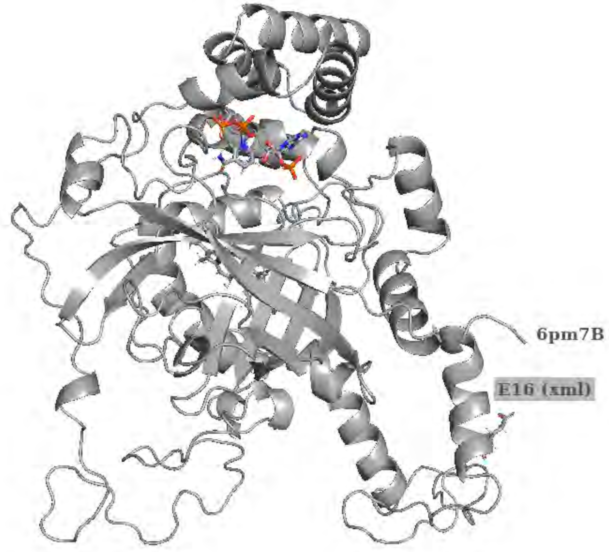

UniProt ID: P00432
PDB ID: 6PO0_A

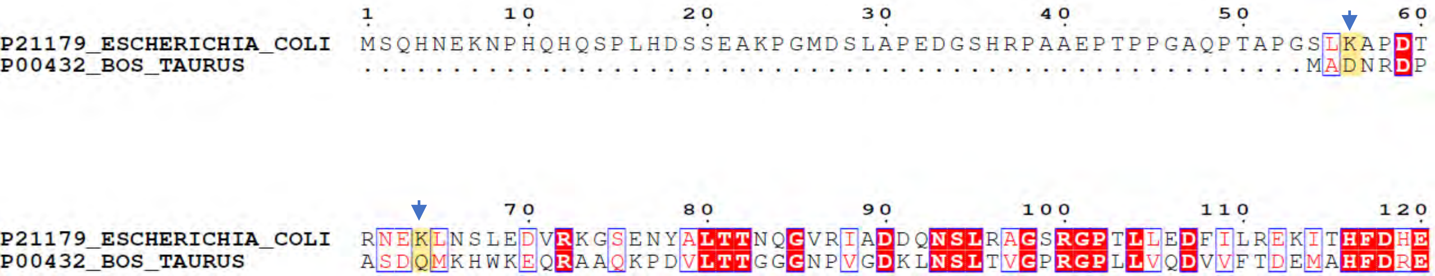

Full sequences in supplemental file.

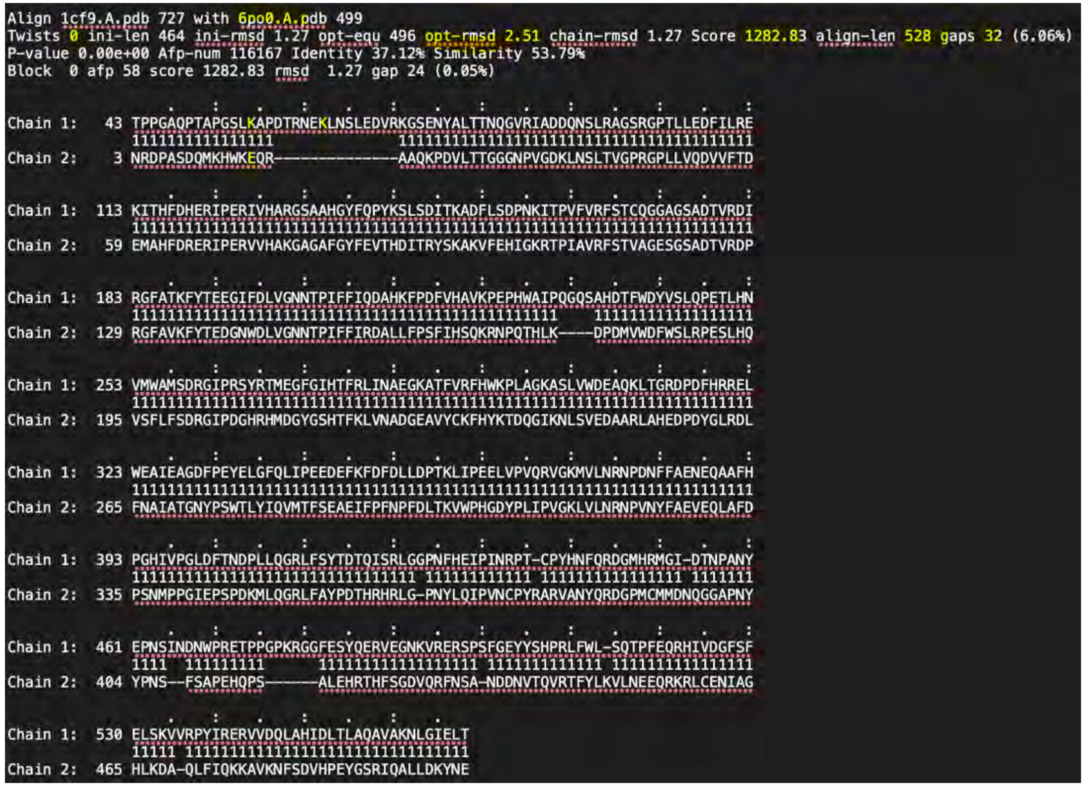

```
Align 1cf9.A.pdb 727 with 6po0.A.pdb 499
Twists 0 ini-len 464 ini-rmsd 1.27 opt-equ 496 opt-rmsd 2.51 chain-rmsd 1.27 Score 1282.83 align-len 528 gaps 32 (6.06%)
P-value 0.00e+00 Afp-num 116167 Identity 37.12% Similarity 53.79%
Block  0 afp 58 score 1282.83 rmsd  1.27 gap 24 (0.05%)

Chain 1:   43 TPPGAQPTAPGSLKAPDTRNEKLNSLEDVRKGSENYALTTNQGVRIADDQNSLRAGSRGPTLLEDFILRE
Chain 2:    3 NRDPASDQMKHWKEQR--------------AAQKPDVLTTGGGNPVGDKLNSLTVGPRGPLLVQDVVFTD

Chain 1:  113 KITHFDHERIPERIVHARGSAAHGYFQPYKSLSDITKADFLSDPNKITPVFVRFSTCQGGAGSADTVRDI
Chain 2:   59 EMAHFDRERIPERVVHAKGAGAFGYFEVTHDITRYSKAKVFEHIGKRTPIAVRFSTVAGESGSADTVRDP

Chain 1:  183 RGFATKFYTEEGIFDLVGNNTPIFFIQDAHKFPDFVHAVKPEPHWAIPQGQSAHDTFWDYVSLQPETLHN
Chain 2:  129 RGFAVKFYTEDGNWDLVGNNTPIFFIRDALLFPSFIHSQKRNPQTHLK----DPDMVWDFWSLRPESLHQ

Chain 1:  253 VMWAMSDRGIPRSYRTMEGFGIHTFRLINAEGKATFVRFHWKPLAGKASLVWDEAQKLTGRDPDFHRREL
Chain 2:  195 VSFLFSDRGIPDGHRHMDGYGSHTFKLVNADGEAVYCKFHYKTDQGIKNLSVEDAARLAHEDPDYGLRDL

Chain 1:  323 WEAIEAGDFPEYELGFQLIPEEDEFKFDFDLLDPTKLIPEELVPVQRVGKMVLNRNPDNFFAENEQAAFH
Chain 2:  265 FNAIATGNYPSWTLYIQVMTFSEAEIFPFNPFDLTKVWPHGDYPLIPVGKLVLNRNPVNYFAEVEQLAFD

Chain 1:  393 PGHIVPGLDFTNDPLLQGRLFSYTDTQISRLGGPNFHEIPINRPT-CPYHNFQRDGMHRMGI-DTNPANY
Chain 2:  335 PSNMPPGIEPSPDKMLQGRLFAYPDTHRHRLG-PNYLQIPVNCPYRARVANYQRDGPMCMMDNQGGAPNY

Chain 1:  461 EPNSINDNWPRETPPGPKRGGFESYQERVEGNKVRERSPSFGEYYSHPRLFWL-SQTPFEQRHIVDGFSF
Chain 2:  404 YPNS--FSAPEHQPS------ALEHRTHFSGDVQRFNSA-NDDNVTQVRTFYLKVLNEEQRKRLCENIAG

Chain 1:  530 ELSKVVRPYIRERVVDQLAHIDLTLAQAVAKNLGIELT
Chain 2:  465 HLKDA-QLFIQKKAVKNFSDVHPEYGSRIQALLDKYNE
```

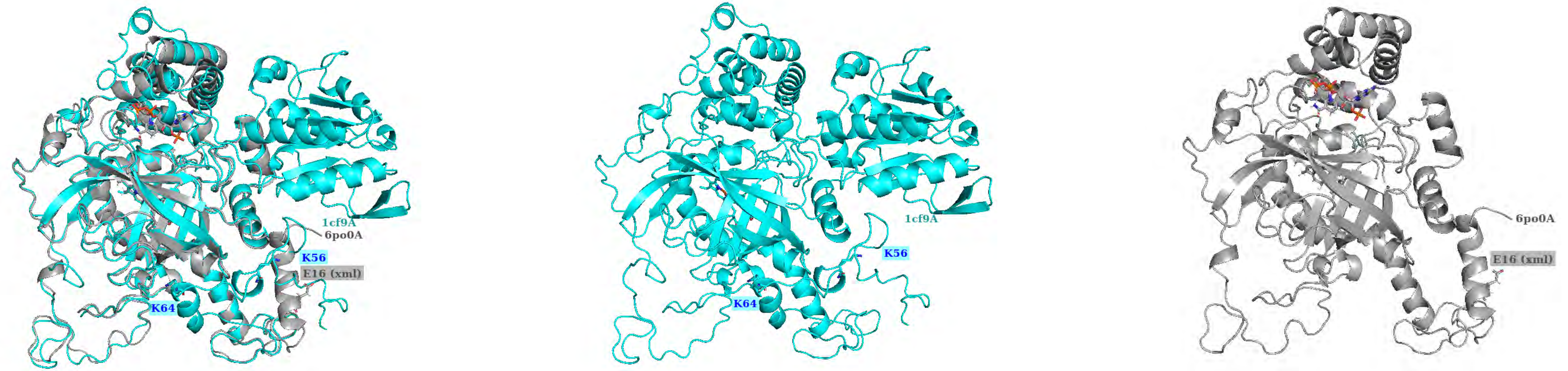

UniProt ID: P00432
PDB ID: 7CAT_A

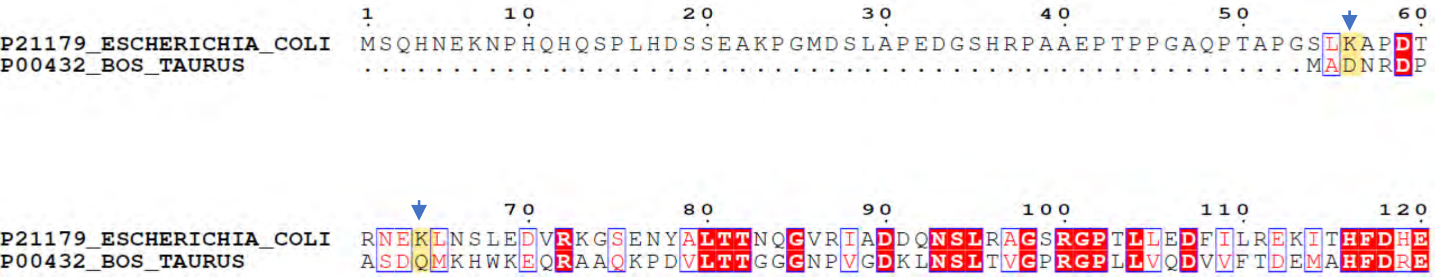

Full sequences in supplemental file.

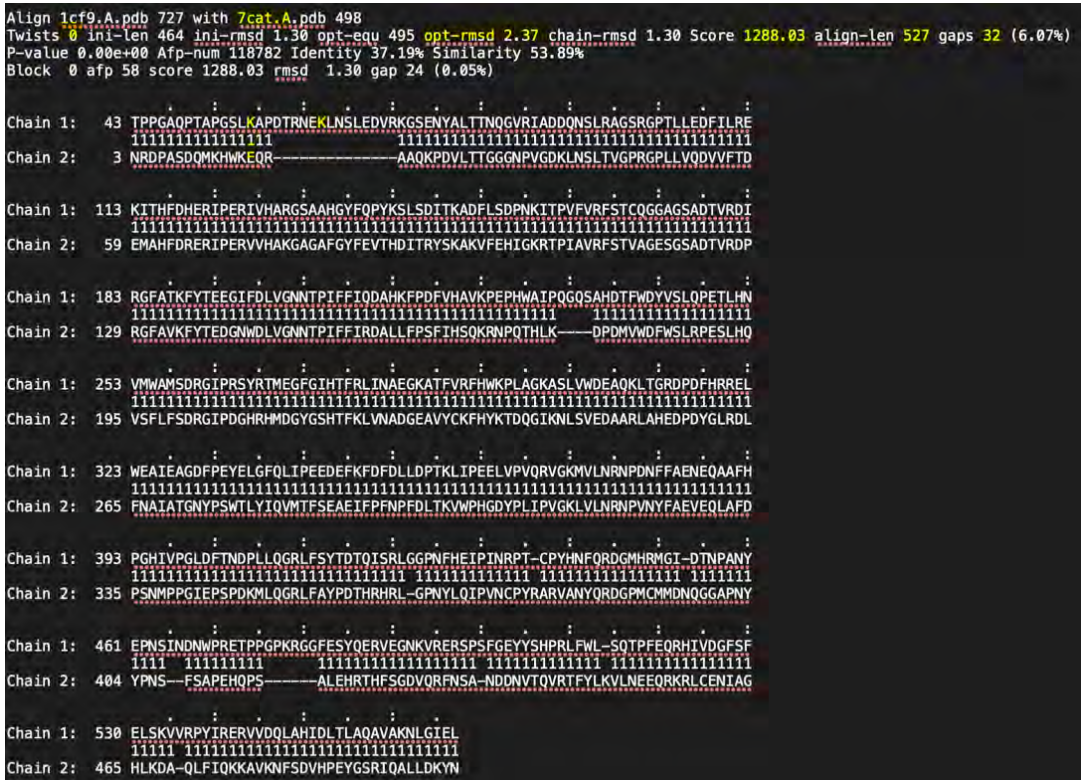

```
Align 1cf9.A.pdb 727 with 7cat.A.pdb 498
Twists 0 ini-len 464 ini-rmsd 1.30 opt-equ 495 opt-rmsd 2.37 chain-rmsd 1.30 Score 1288.03 align-len 527 gaps 32 (6.07%)
P-value 0.00e+00 Afp-num 118782 Identity 37.19% Similarity 53.89%
Block  0 afp 58 score 1288.03 rmsd  1.30 gap 24 (0.05%)

Chain 1:   43 TPPGAQPTAPGSLKAPDTRNEKLNSLEDVRKGSENYALTTNQGVRIADDQNSLRAGSRGPTLLEDFILRE
Chain 2:    3 NRDPASDQMKHWKEQR--------------AAQKPDVLTTGGNPVGDKLNSLTVGPRGPLLVQDVVFTD

Chain 1:  113 KITHFDHERIPERIVHARGSAAHGYFQPYKSLSDITKADFLSDPNKITPVFVRFSTCQGGAGSADTVRDI
Chain 2:   59 EMAHFDRERIPERVVHAKGAGAFGYFEVTHDITRYSKAKVFEHIGKRTPIAVRFSTVAGESGSADTVRDP

Chain 1:  183 RGFATKFYTEEGIFDLVGNNTPIFFIQDAHKFPDFVHAVKPEPHWAIPQGQSAHDTFWDYVSLQPETLHN
Chain 2:  129 RGFAVKFYTEDGNWDLVGNNTPIFFIRDALLFPSFIHSQKRNPQTHLK----DPDMVWDFWSLRPESLHQ

Chain 1:  253 VMWAMSDRGIPRSYRTMEGFGIHTFRLINAEGKATFVRFHWKPLAGKASLVWDEAQKLTGRDPDFHRREL
Chain 2:  195 VSFLFSDRGIPDGHRHMDGYGSHTFKLVNADGEAVYCKFHYKTDQGIKNLSVEDAARLAHEDPDYGLRDL

Chain 1:  323 WEAIEAGDFPEYELGFQLIPEEDEFKFDFDLLDPTKLIPEELVPVQRVGKMVLNRNPDNFFAENEQAAFH
Chain 2:  265 FNAIATGNYPSWTLYIQVMTFSEAEIFPFNPFDLTKVWPHGDYPLIPVGKLVLNRNPVNYFAEVEQLAFD

Chain 1:  393 PGHIVPGLDFTNDPLLQGRLFSYTDTQISRLGGPNFHEIPINRPT-CPYHNFQRDGMHRMGI-DTNPANY
Chain 2:  335 PSNMPPGIEPSPDKMLQGRLFAYPDTHRHRL-GPNYLQIPVNCPYRARVANYQRDGPMCMMDNQGGAPNY

Chain 1:  461 EPNSINDNWPRETPPGPKRGGFESYQERVEGNKVRERSPSFGEYYSHPRLFWL-SQTPFEQRHIVDGFSF
Chain 2:  404 YPNS--FSAPEHQPS------ALEHRTHFSGDVQRFNSA-NDDNVTQVRTFYLKVLNEEQRKRLCENIAG

Chain 1:  530 ELSKVVRPYIRERVVDQLAHIDLTLAQAVAKNLGIEL
Chain 2:  465 HLKDA-QLFIQKKAVKNFSDVHPEYGSRIQALLDKYN
```

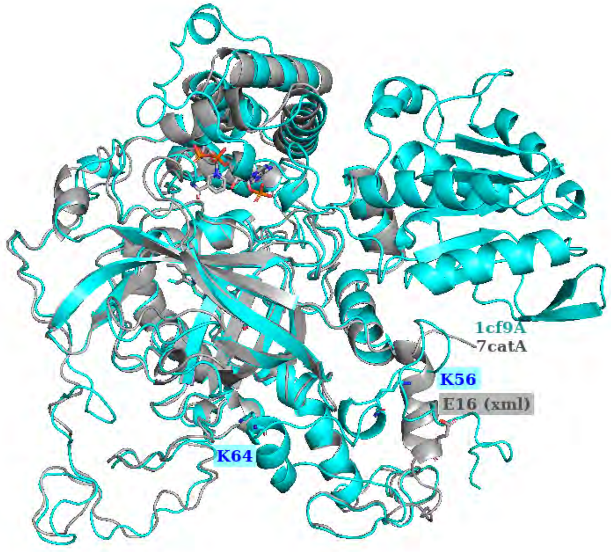

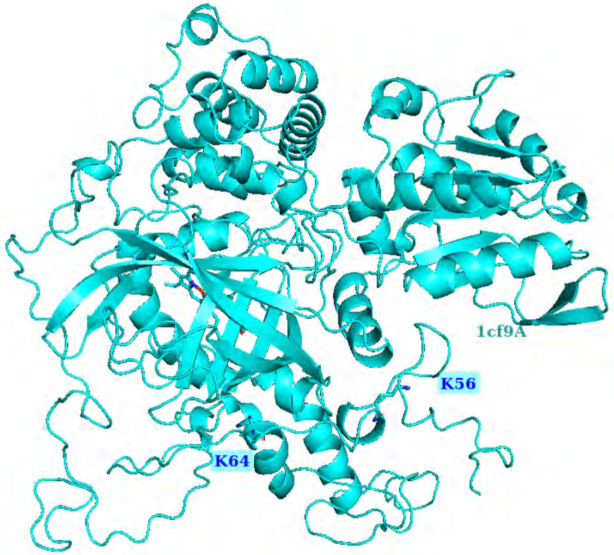

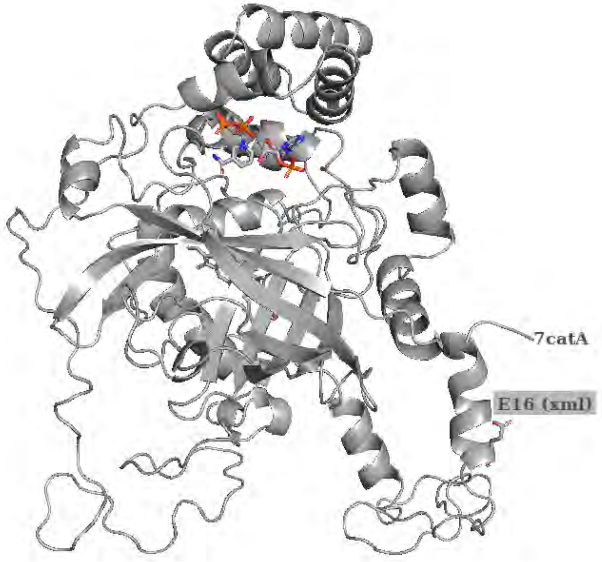

UniProt ID: P00432

PDB ID: 7DI8_D

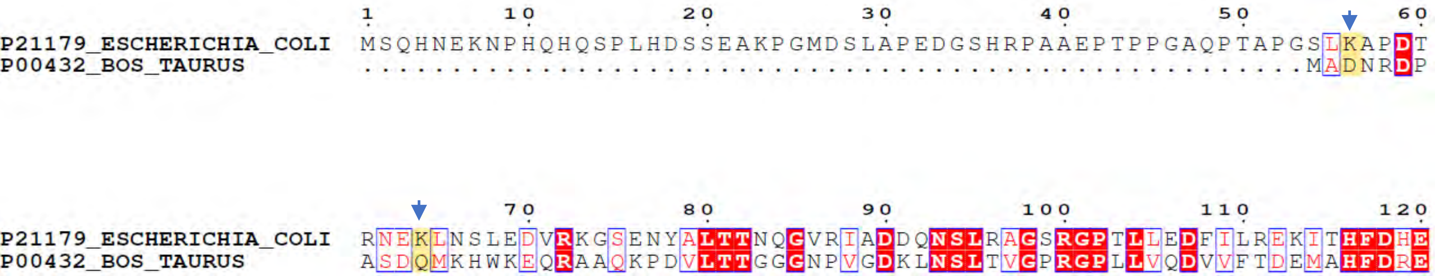

Full sequences in supplemental file.

```
Align 1cf9.A.pdb 727 with 7di8.D.pdb 499
Twists 0 ini-len 464 ini-rmsd 1.28 opt-equ 496 opt-rmsd 2.52 chain-rmsd 1.28 Score 1285.17 align-len 528 gaps 32 (6.06%)
P-value 0.00e+00 Afp-num 118711 Identity 37.12% Similarity 53.41%
Block  0 afp 58 score 1285.17 rmsd  1.28 gap 24 (0.05%)

Chain 1:   43 TPPGAQPTAPGSLKAPDTRNEKLNSLEDVRKGSENYALTTNQGVRIADDQNSLRAGSRGPTLLEDFILRE
              1111111111111111              111111111111111111111111111111111111111
Chain 2:    3 NRDPASDQMKHWKEQR--------------AAQKPDVLTTGGNPVGDKLNSLTVGPRGPLLVQDVVFTD

Chain 1:  113 KITHFDHERIPERIVHARGSAAHGYFQPYKSLSDITKADFLSDPNKITPVFVRFSTCQGGAGSADTVRDI
              1111111111111111111111111111111111111111111111111111111111111111111111
Chain 2:   59 EMAHFDRERIPERVVHAKGAGAFGYFEVTHDITRYSKAKVFEHIGKRTPIAVRFSTVAGESGSADTVRDP

Chain 1:  183 RGFATKFYTEEGIFDLVGNNTPIFFIQDAHKFPDFVHAVKPEPHWAIPQGQSAHDTFWDYVSLQPETLHN
              11111111111111111111111111111111111111111111111111    111111111111111111
Chain 2:  129 RGFAVKFYTEDGNWDLVGNNTPIFFIRDALLFPSFIHSQKRNPQTHLK----DPDMVWDFWSLRPESLHQ

Chain 1:  253 VMWAMSDRGIPRSYRTMEGFGIHTFRLINAEGKATFVRFHWKPLAGKASLVWDEAQKLTGRDPDFHRREL
              1111111111111111111111111111111111111111111111111111111111111111111111
Chain 2:  195 VSFLFSDRGIPDGHRHMNGYGSHTFKLVNANGEAVYCKFHYKTDQGIKNLSVEDAARLAHEDPDYGLRDL

Chain 1:  323 WEAIEAGDFPEYELGFQLIPEEDEFKFDFDLLDPTKLIPEELVPVQRVGKMVLNRNPDNFFAENEQAAFH
              1111111111111111111111111111111111111111111111111111111111111111111111
Chain 2:  265 FNAIATGNYPSWTLYIQVMTFSEAEIFPFNPFDLTKVWPHGDYPLIPVGKLVLNRNPVNYFAEVEQLAFD

Chain 1:  393 PGHIVPGLDFTNDPLLQGRLFSYTDTQISRLGGPNFHEIPINRPTC-PYHNFQRDGMHRMGI-DTNPANY
              11111111111111111111111111111111 1111111111111 111111111111111 1111111
Chain 2:  335 PSNMPPGIEPSPDKMLQGRLFAYPDTHRHRLG-PNYLQIPVNCPYRARVANYQRDGPMCMMDNQGGAPNY

Chain 1:  461 EPNSINDNWPRETPPGPKRGGFESYQERVEGNKVRERSPSFGEYYSHPRLFWL-SQTPFEQRHIVDGFSF
              1111  111111111      111111111111111111 1111111111111 1111111111111111
Chain 2:  404 YPNS--FSAPEHQPS------ALEHRTHFSGDVQRFNSA-NDDNVTQVRTFYLKVLNEEQRKRLCENIAG

Chain 1:  530 ELSKVVRPYIRERVVDQLAHIDLTLAQAVAKNLGIELT
              11111 111111111111111111111111111111111
Chain 2:  465 HLKDA-QLFIQKKAVKNFSDVHPEYGSRIQALLDKYNE
```

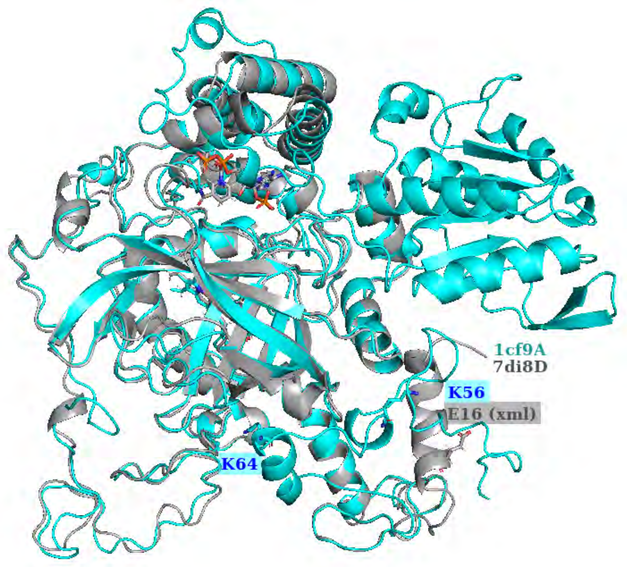

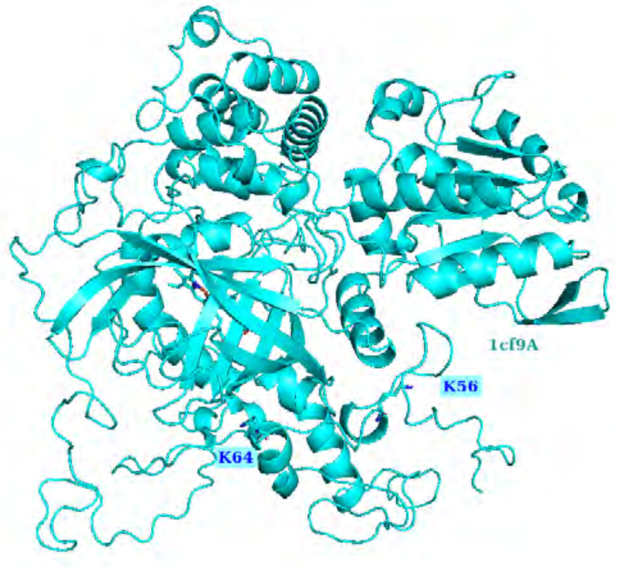

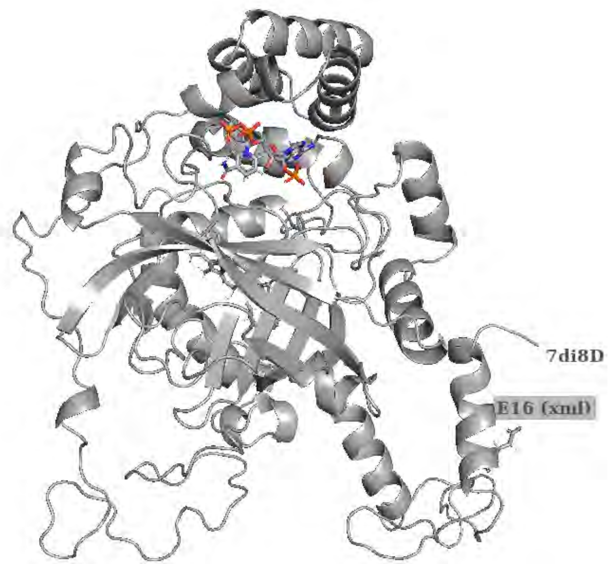

UniProt ID: P00432

PDB ID: 8CAT_A

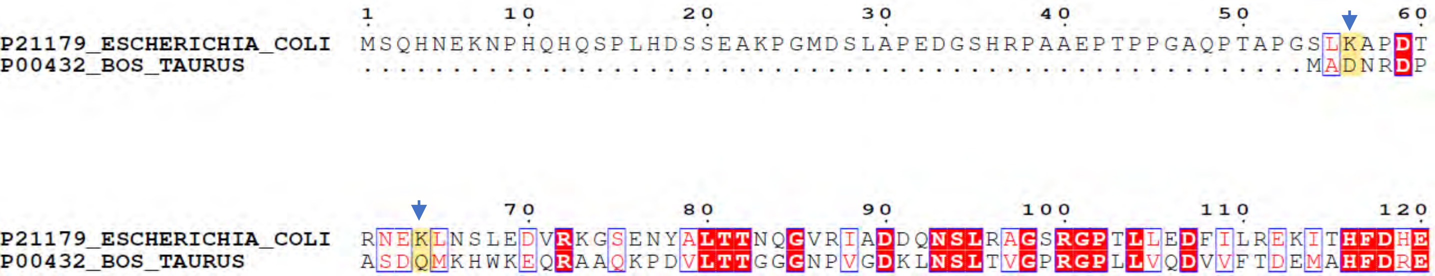

Full sequences in supplemental file.

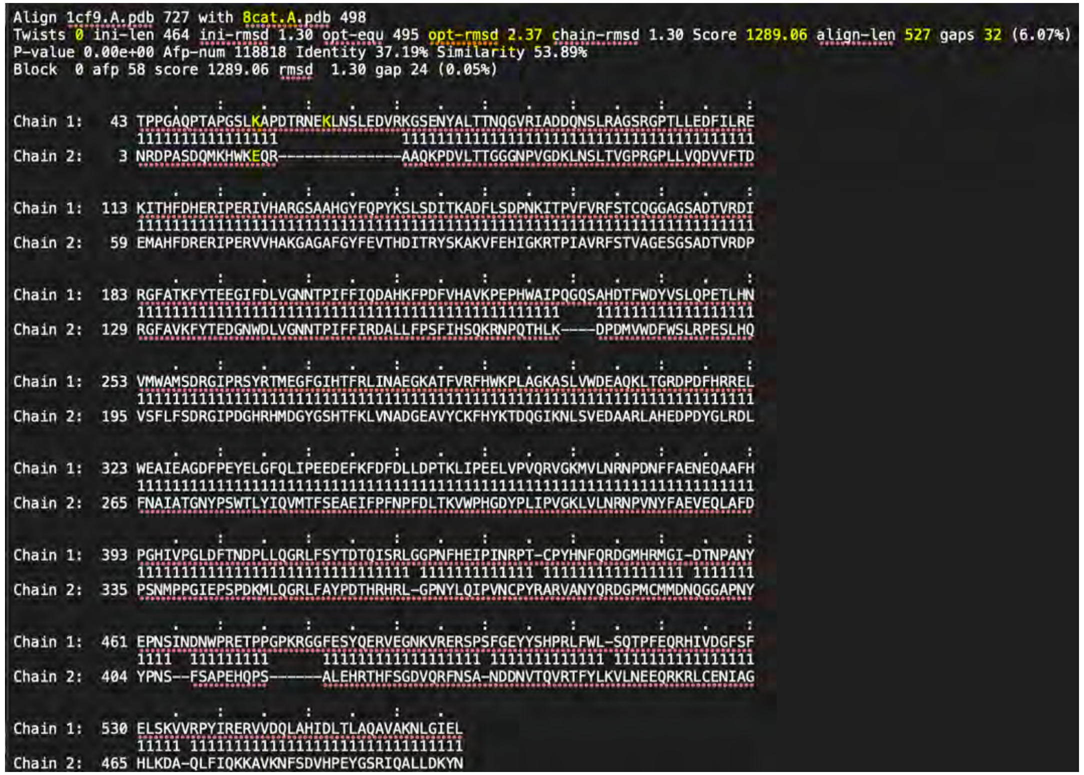

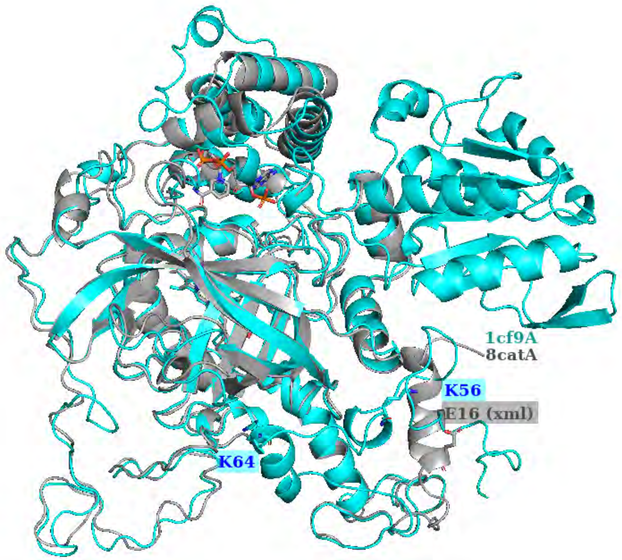

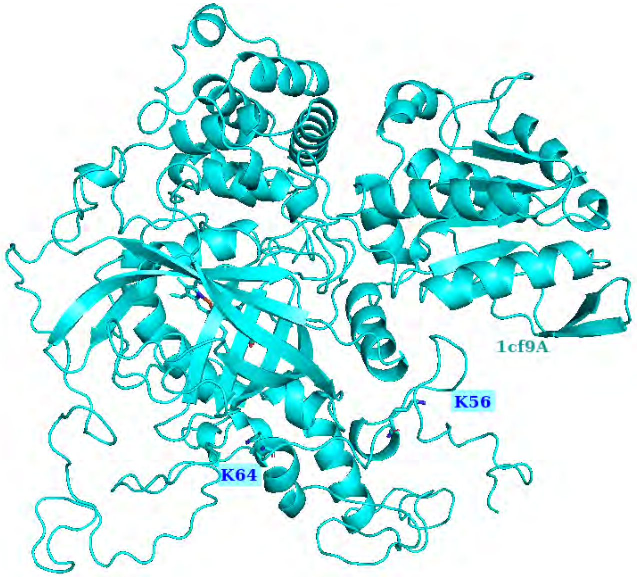

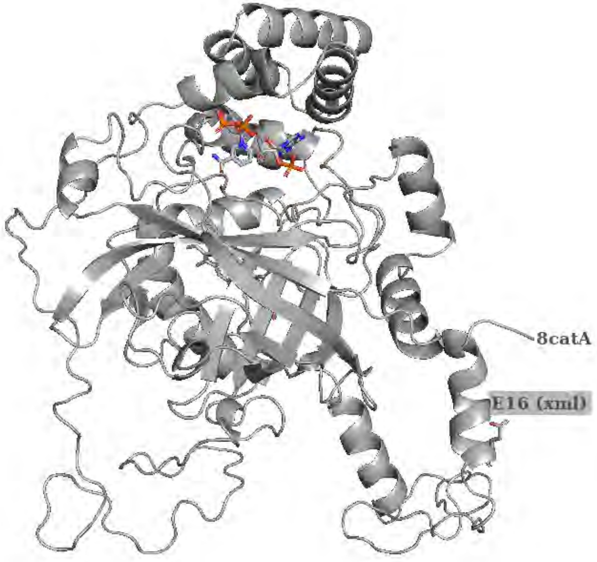

UniProt ID: P04040

PDB ID: 1DGB_D

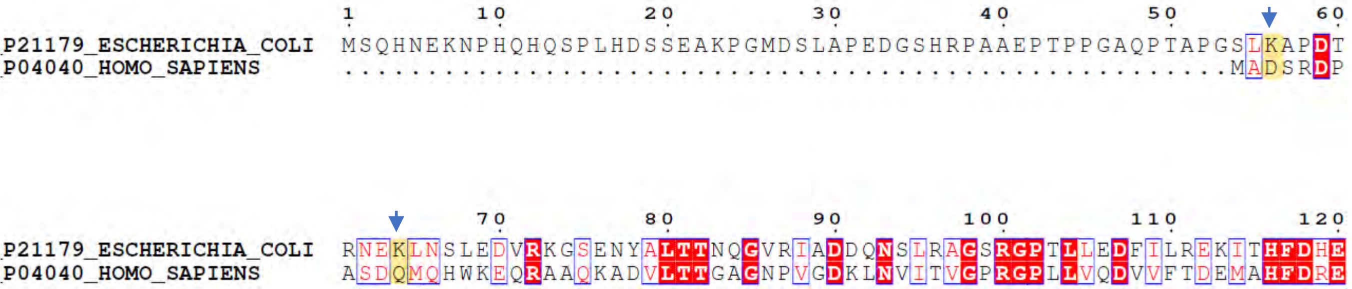

Full sequences in supplemental file.

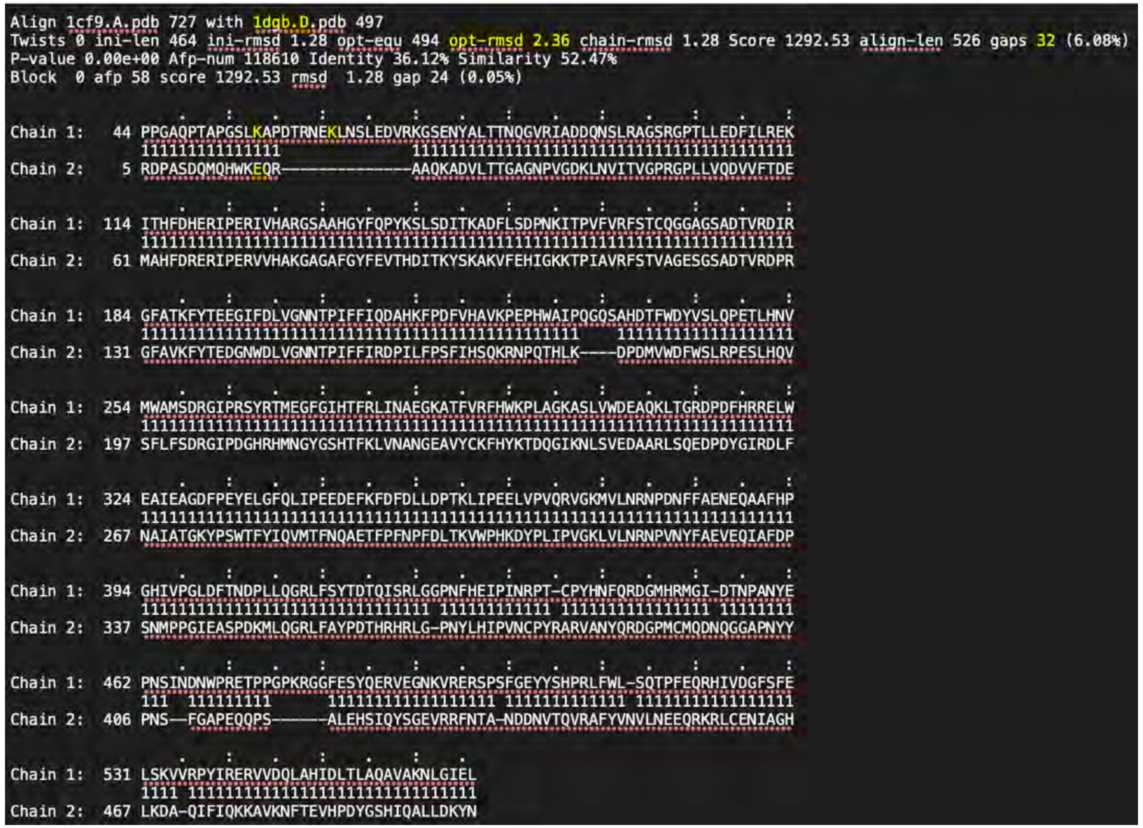

```
Align 1cf9.A.pdb 727 with 1dgb.D.pdb 497
Twists 0 ini-len 464 ini-rmsd 1.28 opt-equ 494 opt-rmsd 2.36 chain-rmsd 1.28 Score 1292.53 align-len 526 gaps 32 (6.08%)
P-value 0.00e+00 Afp-num 118610 Identity 36.12% Similarity 52.47%
Block  0 afp 58 score 1292.53 rmsd  1.28 gap 24 (0.05%)

               .    :    .    :    .    :    .    :    .    :    .    :    .    :
Chain 1:   44 PPGAQPTAPGSLKAPDTRNEKLNSLEDVRKGSENYALTTNQGVRIADDQNSLRAGSRGPTLLEDFILREK
              111111111111111              111111111111111111111111111111111111111111
Chain 2:    5 RDPASDQMQHWKEQR--------------AAQKADVLTTGAGNPVGDKLNVITVGPRGPLLVQDVVFTDE

               .    :    .    :    .    :    .    :    .    :    .    :    .    :
Chain 1:  114 ITHFDHERIPERIVHARGSAAHGYFQPYKSLSDITKADFLSDPNKITPVFVRFSTCQGGAGSADTVRDIR
              1111111111111111111111111111111111111111111111111111111111111111111111
Chain 2:   61 MAHFDRERIPERVVHAKGAGAFGYFEVTHDITKYSKAKVFEHIGKKTPIAVRFSTVAGESGSADTVRDPR

               .    :    .    :    .    :    .    :    .    :    .    :    .    :
Chain 1:  184 GFATKFYTEEGIFDLVGNNTPIFFIQDAHKFPDFVHAVKPEPHWAIPQGQSAHDTFWDYVSLQPETLHNV
              1111111111111111111111111111111111111111111111111    1111111111111111111
Chain 2:  131 GFAVKFYTEDGNWDLVGNNTPIFFIRDPILFPSFIHSQKRNPQTHLK----DPDMVWDFWSLRPESLHQV

               .    :    .    :    .    :    .    :    .    :    .    :    .    :
Chain 1:  254 MWAMSDRGIPRSYRTMEGFGIHTFRLINAEGKATFVRFHWKPLAGKASLVWDEAQKLTGRDPDFHRRELW
              1111111111111111111111111111111111111111111111111111111111111111111111
Chain 2:  197 SFLFSDRGIPDGHRHMNGYGSHTFKLVNANGEAVYCKFHYKTDQGIKNLSVEDAARLSQEDPDYGIRDLF

               .    :    .    :    .    :    .    :    .    :    .    :    .    :
Chain 1:  324 EAIEAGDFPEYELGFQLIPEEDEFKFDFDLLDPTKLIPEELVPVQRVGKMVLNRNPDNFFAENEQAAFHP
              1111111111111111111111111111111111111111111111111111111111111111111111
Chain 2:  267 NAIATGKYPSWTFYIQVMTFNQAETFPFNPFDLTKVWPHKDYPLIPVGKLVLNRNPVNYFAEVEQIAFDP

               .    :    .    :    .    :    .    :    .    :    .    :    .    :
Chain 1:  394 GHIVPGLDFTNDPLLQGRLFSYTDTQISRLGGPNFHEIPINRPT-CPYHNFQRDGMHRMGI-DTNPANYE
              111111111111111111111111111111 111111111111 1111111111111111 11111111
Chain 2:  337 SNMPPGIEASPDKMLQGRLFAYPDTHRHRLG-PNYLHIPVNCPYRARVANYQRDGPMCMQDNQGGAPNYY

               .    :    .    :    .    :    .    :    .    :    .    :    .    :
Chain 1:  462 PNSINDNWPRETPPGPKRGGFESYQERVEGNKVRERSPSFGEYYSHPRLFWL-SQTPFEQRHIVDGFSFE
              111  111111111      111111111111111111 1111111111111 1111111111111111
Chain 2:  406 PNS--FGAPEQQPS------ALEHSIQYSGEVRRFNTA-NDDNVTQVRAFYVNVLNEEQRKRLCENIAGH

               .    :    .    :    .    :    .
Chain 1:  531 LSKVVRPYIRERVVDQLAHIDLTLAQAVAKNLGIEL
              1111 11111111111111111111111111111
Chain 2:  467 LKDA-QIFIQKKAVKNFTEVHPDYGSHIQALLDKYN
```

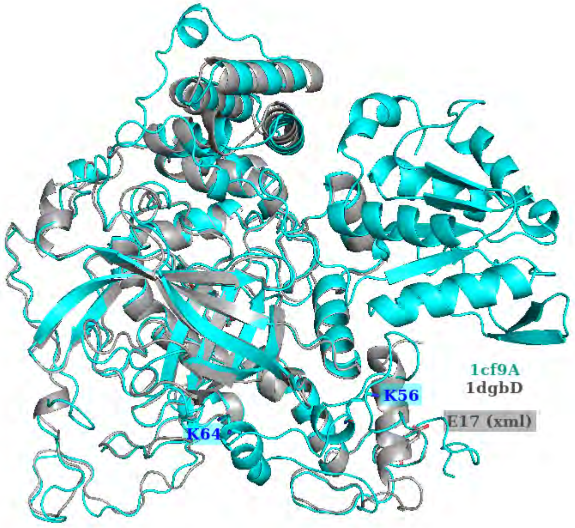

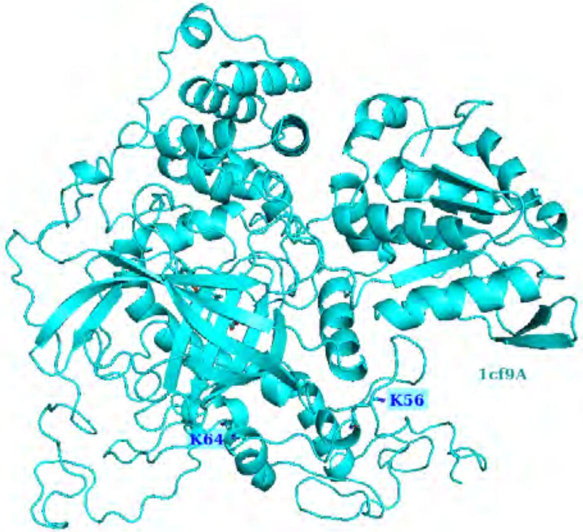

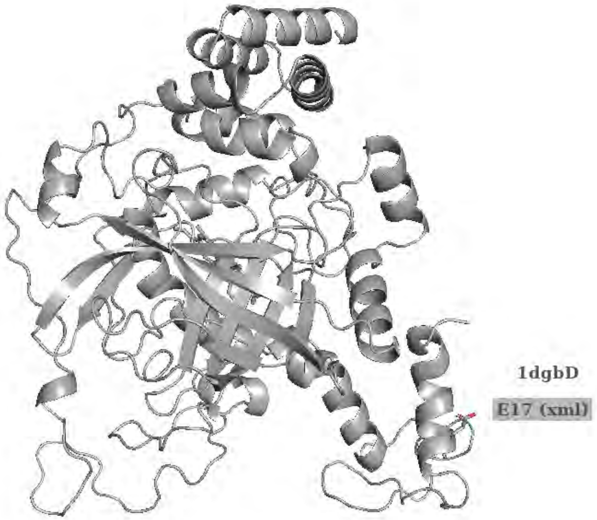

UniProt ID: P04040

PDB ID: 1DGF_C

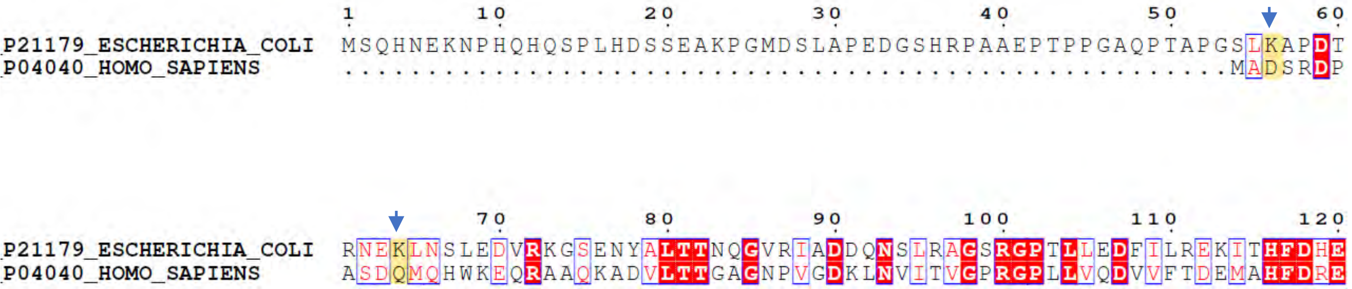

Full sequences in supplemental file.

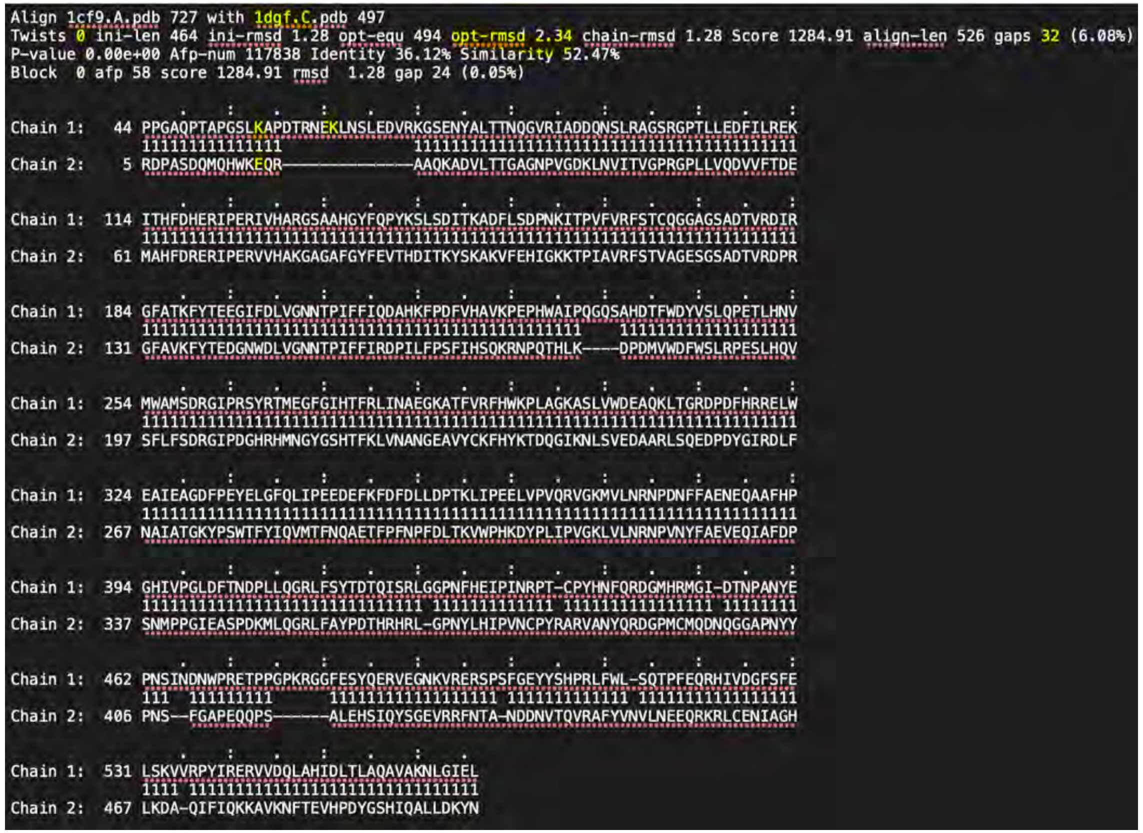

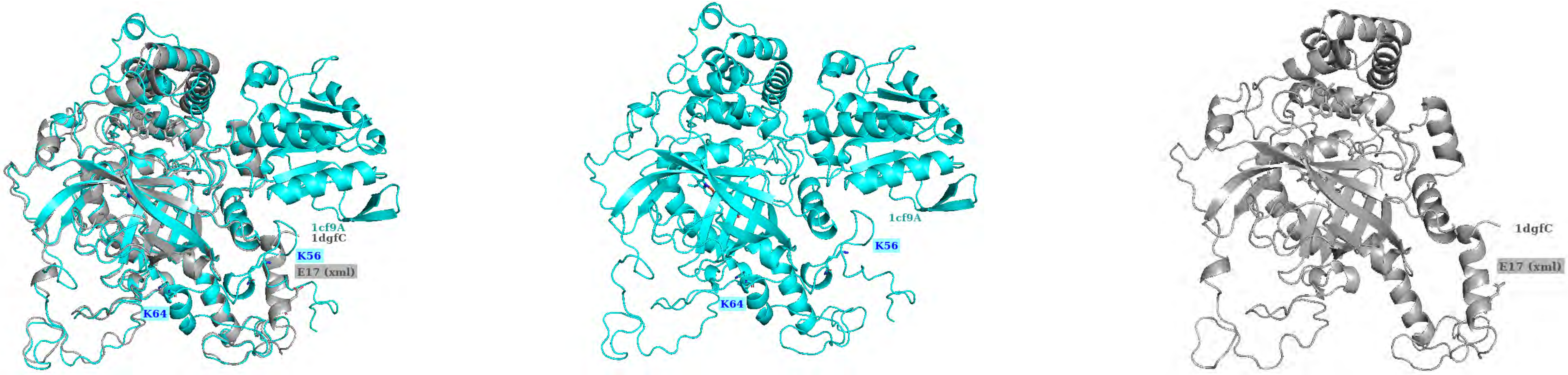

UniProt ID: P04040
PDB ID: 1DGG_A

```
                         1        10        20        30        40        50        60
P21179_ESCHERICHIA_COLI  MSQHNEKNPHQHQSPLHDSSEAKPGMDSLAPEDGSHRPAAEPTPPGAQPTAPGSLKAPDT
P04040_HOMO_SAPIENS      .....................................................MADSRDP

                                  70        80        90       100       110       120
P21179_ESCHERICHIA_COLI  RNEKLNSLEDVRKGSENYALTTNQGVRIADDQNSLRAGSRGPTLLEDFILREKITHFDHE
P04040_HOMO_SAPIENS      ASDQMQHWKEQRAAQKADVLTTGAGNPVGDKLNVITVGPRGPLLVQDVVFTDEMAHFDRE
```

Full sequences in supplemental file.

```
Align 1cf9.A.pdb 727 with 1dgg.A.pdb 497
Twists 0 ini-len 464 ini-rmsd 1.26 opt-equ 494 opt-rmsd 2.35 chain-rmsd 1.26 Score 1283.94 align-len 526 gaps 32 (6.08%)
P-value 0.00e+00 Afp-num 118335 Identity 36.12% Similarity 52.47%
Block  0 afp 58 score 1283.94 rmsd  1.26 gap 24 (0.05%)

                  .    :    .    :    .    :    .    :    .    :    .    :    .    :
Chain 1:   44 PPGAQPTAPGSLKAPDTRNEKLNSLEDVRKGSENYALTTNQGVRIADDQNSLRAGSRGPTLLEDFILREK
              111111111111111              11111111111111111111111111111111111111111
Chain 2:    5 RDPASDQMQHWKEQR--------------AAQKADVLTTGAGNPVGDKLNVITVGPRGPLLVQDVVFTDE

                  .    :    .    :    .    :    .    :    .    :    .    :    .    :
Chain 1:  114 ITHFDHERIPERIVHARGSAAHGYFQPYKSLSDITKADFLSDPNKITPVFVRFSTCQGGAGSADTVRDIR
              1111111111111111111111111111111111111111111111111111111111111111111111
Chain 2:   61 MAHFDRERIPERVVHAKGAGAFGYFEVTHDITKYSKAKVFEHIGKKTPIAVRFSTVAGESGSADTVRDPR

                  .    :    .    :    .    :    .    :    .    :    .    :    .    :
Chain 1:  184 GFATKFYTEEGIFDLVGNNTPIFFIQDAHKFPDFVHAVKPEPHWAIPQGQSAHDTFWDYVSLQPETLHNV
              11111111111111111111111111111111111111111111111    1111111111111111111
Chain 2:  131 GFAVKFYTEDGNWDLVGNNTPIFFIRDPILFPSFIHSQKRNPQTHLK----DPDMVWDFWSLRPESLHQV

                  .    :    .    :    .    :    .    :    .    :    .    :    .    :
Chain 1:  254 MWAMSDRGIPRSYRTMEGFGIHTFRLINAEGKATFVRFHWKPLAGKASLVWDEAQKLTGRDPDFHRRELW
              1111111111111111111111111111111111111111111111111111111111111111111111
Chain 2:  197 SFLFSDRGIPDGHRHMNGYGSHTFKLVNANGEAVYCKFHYKTDQGIKNLSVEDAARLSQEDPDYGIRDLF

                  .    :    .    :    .    :    .    :    .    :    .    :    .    :
Chain 1:  324 EAIEAGDFPEYELGFQLIPEEDEFKFDFDLLDPTKLIPEELVPVQRVGKMVLNRNPDNFFAENEQAAFHP
              1111111111111111111111111111111111111111111111111111111111111111111111
Chain 2:  267 NAIATGKYPSWTFYIQVMTFNQAETFPFNPFDLTKVWPHKDYPLIPVGKLVLNRNPVNYFAEVEQIAFDP

                  .    :    .    :    .    :    .    :    .    :    .    :    .    :
Chain 1:  394 GHIVPGLDFTNDPLLQGRLFSYTDTQISRLGGPNFHEIPINRPT-CPYHNFQRDGMHRMGI-DTNPANYE
              1111111111111111111111111111111 111111111111 1111111111111111 11111111
Chain 2:  337 SNMPPGIEASPDKMLQGRLFAYPDTHRHRLG-PNYLHIPVNCPYRARVANYQRDGPMCMQDNQGGAPNYY

                  .    :    .    :    .    :    .    :    .    :    .    :    .    :
Chain 1:  462 PNSINDNWPRETPPGPKRGGFESYQERVEGNKVRERSPSFGEYYSHPRLFWL-SQTPFEQRHIVDGFSFE
              111  111111111      111111111111111111 1111111111111 11111111111111111
Chain 2:  406 PNS--FGAPEQQPS------ALEHSIQYSGEVRRFNTA-NDDNVTQVRAFYVNVLNEEQRKRLCENIAGH

                  .    :    .    :    .    :    .
Chain 1:  531 LSKVVRPYIRERVVDQLAHIDLTLAQAVAKNLGIEL
              1111 1111111111111111111111111111111
Chain 2:  467 LKDA-QIFIQKKAVKNFTEVHPDYGSHIQALLDKYN
```

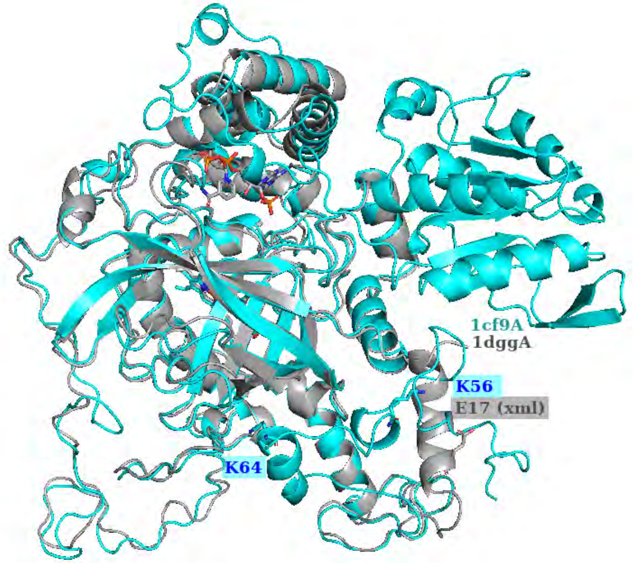

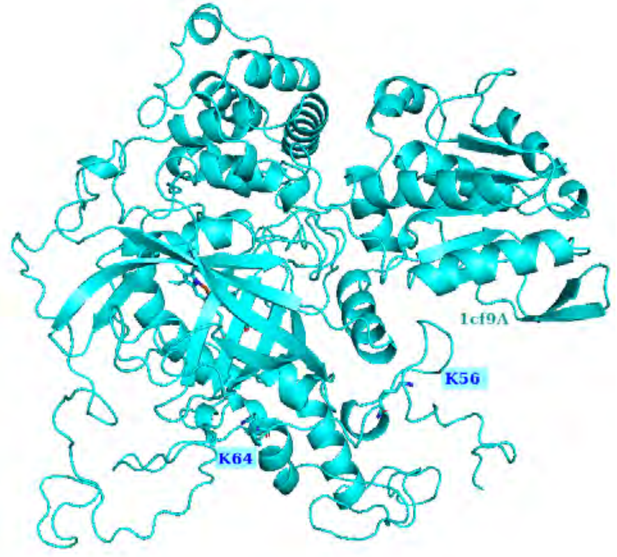

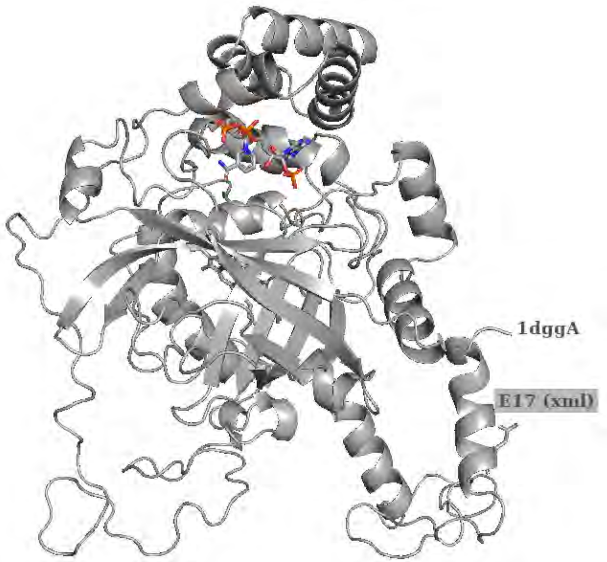

UniProt ID: P04040
PDB ID: 1DGH_B

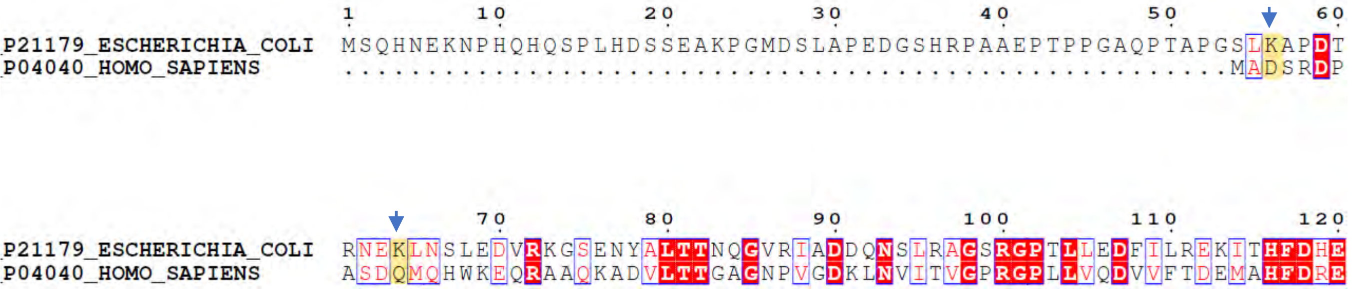

Full sequences in supplemental file.

```
Align 1cf9.A.pdb 727 with 1dgh.B.pdb 498
Twists 0 ini-len 464 ini-rmsd 1.27 opt-equ 494 opt-rmsd 2.28 chain-rmsd 1.27 Score 1286.71 align-len 527 gaps 33 (6.26%)
P-value 0.00e+00 Afp-num 118484 Identity 35.86% Similarity 52.37%
Block  0 afp 58 score 1286.71 rmsd  1.27 gap 24 (0.05%)

Chain 1:   43 TPPGAQPTAPGSLKAPDTRNEKLNSLEDVRKGSENYALTTNQGVRIADDQNSLRAGSRGPTLLEDFILRE
              1111111111111111               111111111111111111111111111111111111111
Chain 2:    4 SRDPASDQMQHWKEQRA--------------AQKADVLTTGAGNPVGDKLNVITVGPRGPLLVQDVVFTD

Chain 1:  113 KITHFDHERIPERIVHARGSAAHGYFQPYKSLSDITKADFLSDPNKITPVFVRFSTCQGGAGSADTVRDI
              1111111111111111111111111111111111111111111111111111111111111111111111
Chain 2:   60 EMAHFDRERIPERVVxAKGAGAFGYFEVTHDITKYSKAKVFEHIGKKTPIAVRFSTVAGESGSADTVRDP

Chain 1:  183 RGFATKFYTEEGIFDLVGNNTPIFFIQDAHKFPDFVHAVKPEPHWAIPQGQSAHDTFWDYVSLQPETLHN
              111111111111111111111111111111111111111111111111    111111111111111111
Chain 2:  130 RGFAVKFYTEDGNWDLVGNNTPIFFIRDPILFPSFIHSQKRNPQTHLK----DPDMVWDFWSLRPESLHQ

Chain 1:  253 VMWAMSDRGIPRSYRTMEGFGIHTFRLINAEGKATFVRFHWKPLAGKASLVWDEAQKLTGRDPDFHRREL
              1111111111111111111111111111111111111111111111111111111111111111111111
Chain 2:  196 VSFLFSDRGIPDGHRHMNGYGSHTFKLVNANGEAVYCKFHYKTDQGIKNLSVEDAARLSQEDPDYGIRDL

Chain 1:  323 WEAIEAGDFPEYELGFQLIPEEDEFKFDFDLLDPTKLIPEELVPVQRVGKMVLNRNPDNFFAENEQAAFH
              1111111111111111111111111111111111111111111111111111111111111111111111
Chain 2:  266 FNAIATGKYPSWTFYIQVMTFNQAETFPFNPFDLTKVWPHKDYPLIPVGKLVLNRNPVNYFAEVEQIAFD

Chain 1:  393 PGHIVPGLDFTNDPLLQGRLFSYTDTQISRLGGPNFHEIPINRPT-CPYHNFQRDGMHRMGI-DTNPANY
              11111111111111111111111111111111 111111111111 1111111111111111 1111111
Chain 2:  336 PSNMPPGIEASPDKMLQGRLFAYPDTHRHRLG-PNYLHIPVNCPYRARVANYQRDGPMCMQDNQGGAPNY

Chain 1:  461 EPNSINDNWPRETPPGPKRGGFESYQERVEGNKVRERSPSFGEYYSHPRLFWL-SQTPFEQRHIVDGFSF
              1111  111111111      111111111111111111 1111111111111 1111111111111111
Chain 2:  405 YPNS--FGAPEQQPS------ALEHSIQYSGEVRRFNTA-NDDNVTQVRAFYVNVLNEEQRKRLCENIAG

Chain 1:  530 ELSKVVRPYIRERVVDQLAHIDLTLAQAVAKNLGIEL
              11111 1111111111111111111111111111111
Chain 2:  466 HLKDA-QIFIQKKAVKNFTEVHPDYGSHIQALLDKYN
```

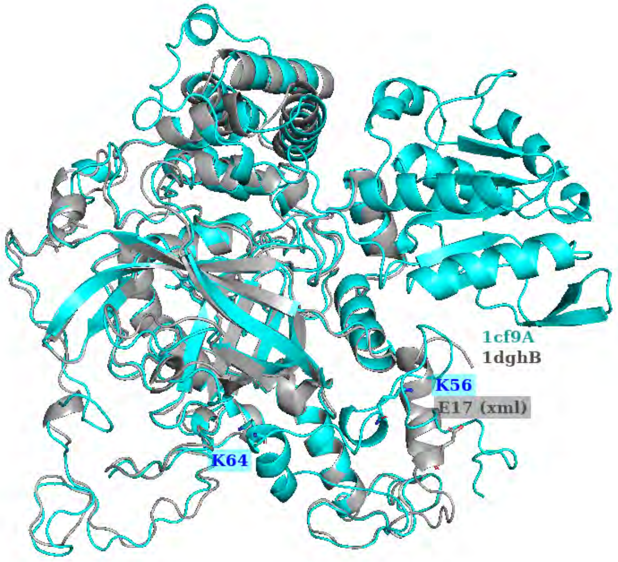

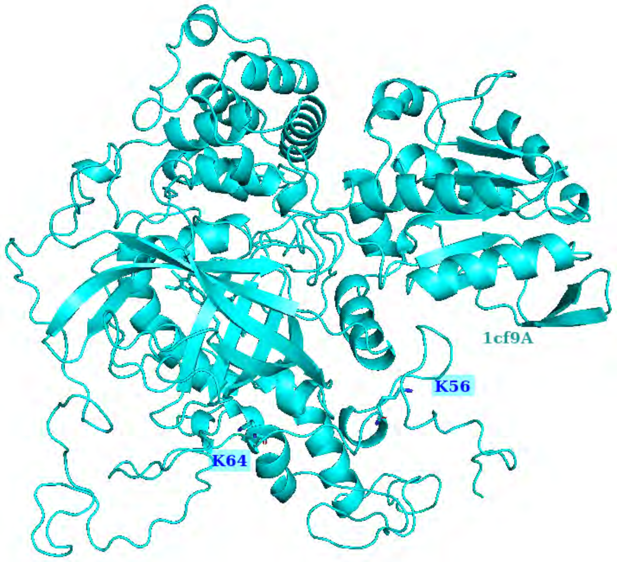

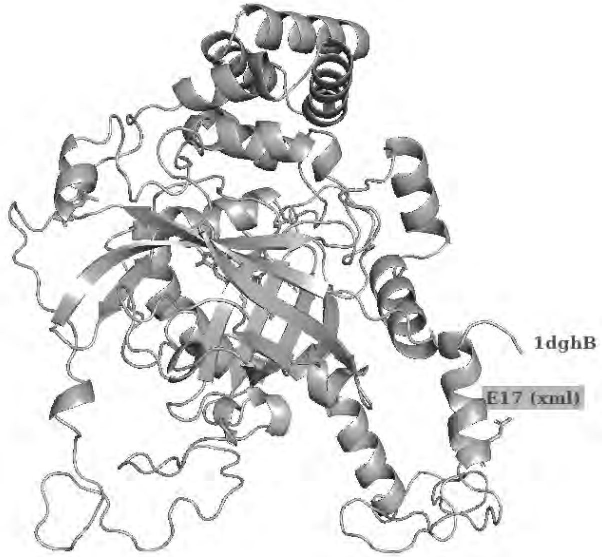

UniProt ID: P04040

PDB ID: 1F4J_B

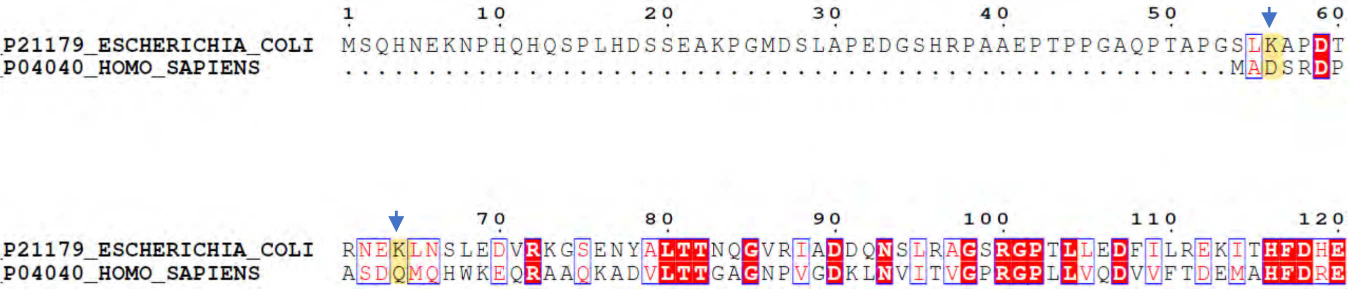

Full sequences in supplemental file.

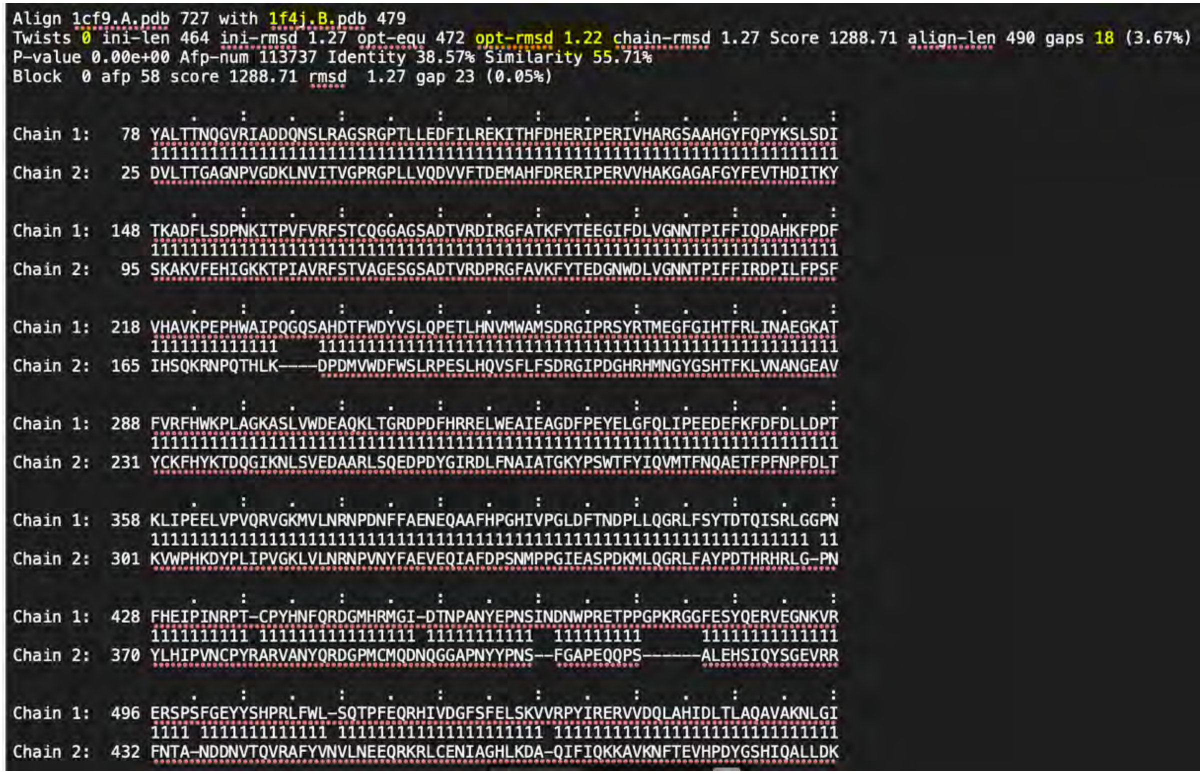

```
Align 1cf9.A.pdb 727 with 1f4j.B.pdb 479
Twists 0 ini-len 464 ini-rmsd 1.27 opt-equ 472 opt-rmsd 1.22 chain-rmsd 1.27 Score 1288.71 align-len 490 gaps 18 (3.67%)
P-value 0.00e+00 Afp-num 113737 Identity 38.57% Similarity 55.71%
Block  0 afp 58 score 1288.71 rmsd  1.27 gap 23 (0.05%)

                 .    :    .    :    .    :    .    :    .    :    .    :    .    :
Chain 1:   78 YALTTNQGVRIADDQNSLRAGSRGPTLLEDFILREKITHFDHERIPERIVHARGSAAHGYFQPYKSLSDI
              1111111111111111111111111111111111111111111111111111111111111111111111
Chain 2:   25 DVLTTGAGNPVGDKLNVITVGPRGPLLVQDVVFTDEMAHFDRERIPERVVHAKGAGAFGYFEVTHDITKY

                 .    :    .    :    .    :    .    :    .    :    .    :    .    :
Chain 1:  148 TKADFLSDPNKITPVFVRFSTCQGGAGSADTVRDIRGFATKFYTEEGIFDLVGNNTPIFFIQDAHKFPDF
              1111111111111111111111111111111111111111111111111111111111111111111111
Chain 2:   95 SKAKVFEHIGKKTPIAVRFSTVAGESGSADTVRDPRGFAVKFYTEDGNWDLVGNNTPIFFIRDPILFPSF

                 .    :    .    :    .    :    .    :    .    :    .    :    .    :
Chain 1:  218 VHAVKPEPHWAIPQGQSAHDTFWDYVSLQPETLHNVMWAMSDRGIPRSYRTMEGFGIHTFRLINAEGKAT
              1111111111111    11111111111111111111111111111111111111111111111111111
Chain 2:  165 IHSQKRNPQTHLK----DPDMVWDFWSLRPESLHQVSFLFSDRGIPDGHRHMNGYGSHTFKLVNANGEAV

                 .    :    .    :    .    :    .    :    .    :    .    :    .    :
Chain 1:  288 FVRFHWKPLAGKASLVWDEAQKLTGRDPDFHRRELWEAIEAGDFPEYELGFQLIPEEDEFKFDFDLLDPT
              1111111111111111111111111111111111111111111111111111111111111111111111
Chain 2:  231 YCKFHYKTDQGIKNLSVEDAARLSQEDPDYGIRDLFNAIATGKYPSWTFYIQVMTFNQAETFPFNPFDLT

                 .    :    .    :    .    :    .    :    .    :    .    :    .    :
Chain 1:  358 KLIPEELVPVQRVGKMVLNRNPDNFFAENEQAAFHPGHIVPGLDFTNDPLLQGRLFSYTDTQISRLGGPN
              111111111111111111111111111111111111111111111111111111111111111111 11
Chain 2:  301 KVWPHKDYPLIPVGKLVLNRNPVNYFAEVEQIAFDPSNMPPGIEASPDKMLQGRLFAYPDTHRHRLG-PN

                 .    :    .    :    .    :    .    :    .    :    .    :    .    :
Chain 1:  428 FHEIPINRPT-CPYHNFQRDGMHRMGI-DTNPANYEPNSINDNWPRETPPGPKRGGFESYQERVEGNKVR
              1111111111 1111111111111111 11111111111  111111111      11111111111111
Chain 2:  370 YLHIPVNCPYRARVANYQRDGPMCMQDNQGGAPNYYPNS--FGAPEQQPS------ALEHSIQYSGEVRR

                 .    :    .    :    .    :    .    :    .    :    .    :    .    :
Chain 1:  496 ERSPSFGEYYSHPRLFWL-SQTPFEQRHIVDGFSFELSKVVRPYIRERVVDQLAHIDLTLAQAVAKNLGI
              1111 1111111111111 1111111111111111111111 111111111111111111111111111111
Chain 2:  432 FNTA-NDDNVTQVRAFYVNVLNEEQRKRLCENIAGHLKDA-QIFIQKKAVKNFTEVHPDYGSHIQALLDK
```

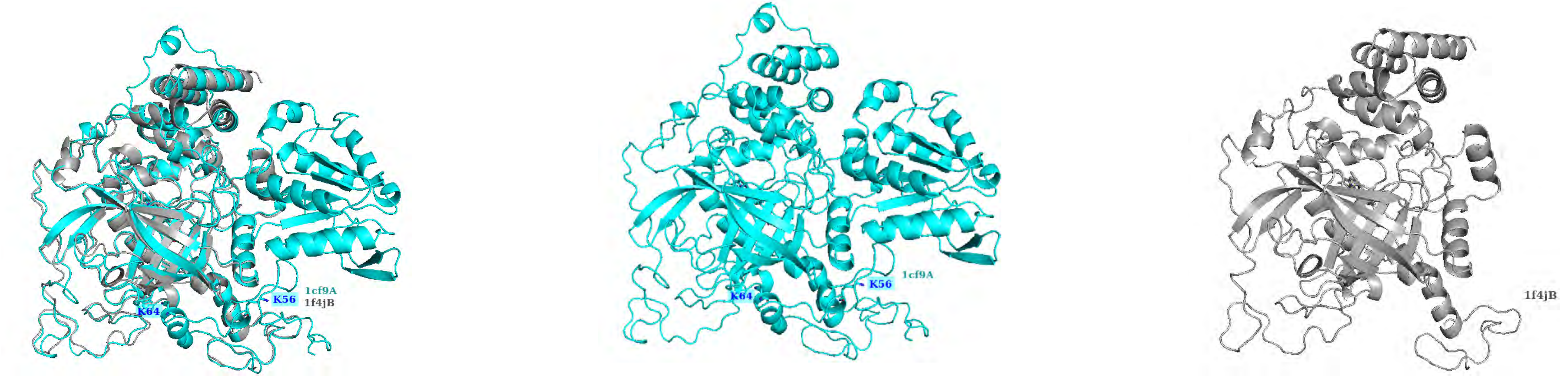

UniProt ID: P04040

PDB ID: 1QQW_D

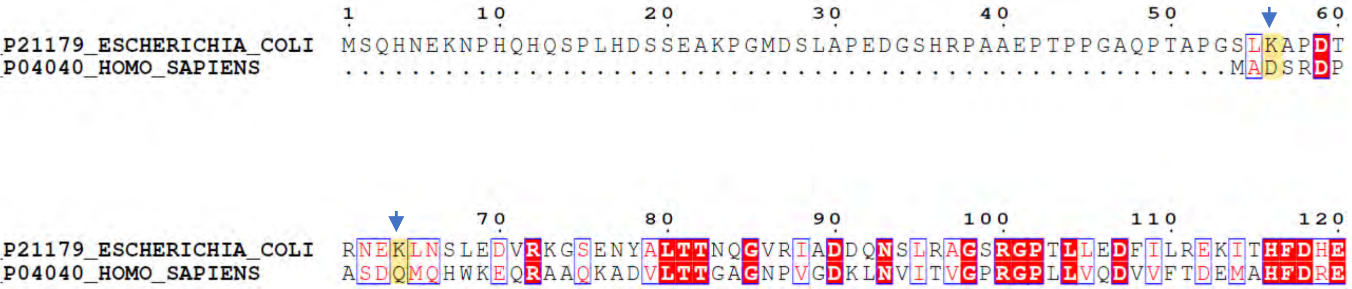

Full sequences in supplemental file.

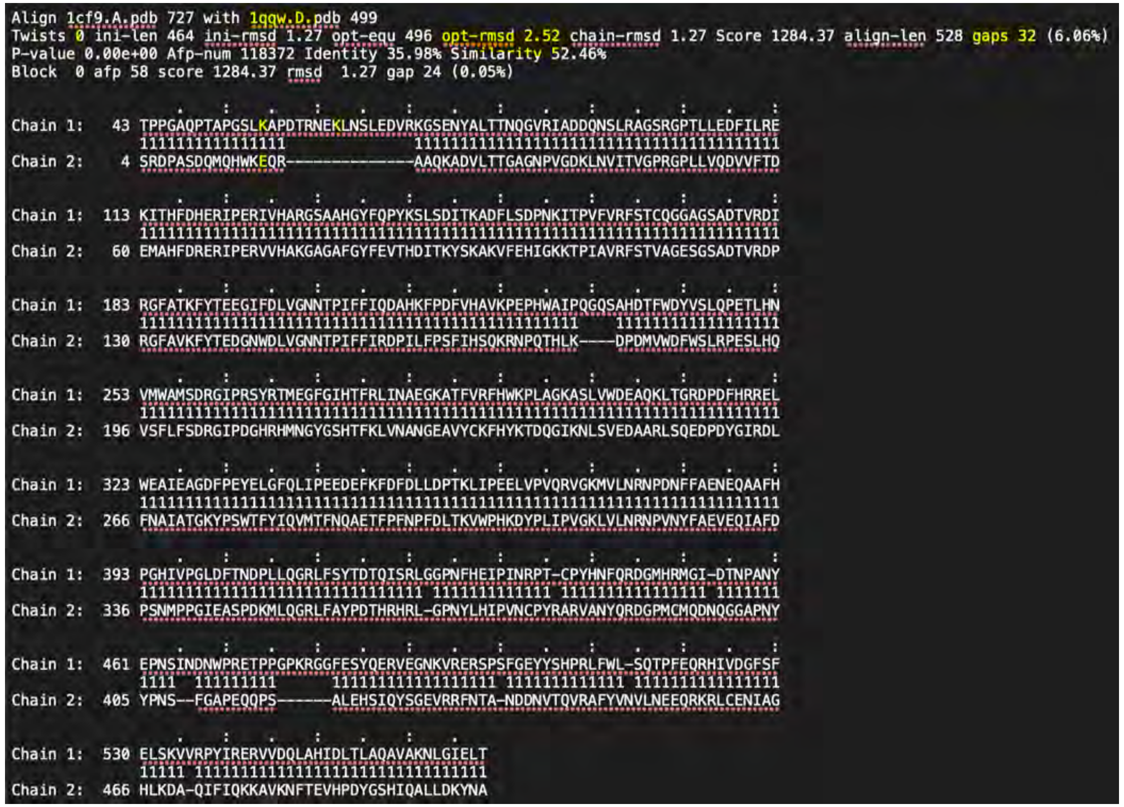

```
Align 1cf9.A.pdb 727 with 1qqw.D.pdb 499
Twists 0 ini-len 464 ini-rmsd 1.27 opt-equ 496 opt-rmsd 2.52 chain-rmsd 1.27 Score 1284.37 align-len 528 gaps 32 (6.06%)
P-value 0.00e+00 Afp-num 118372 Identity 35.98% Similarity 52.46%
Block  0 afp 58 score 1284.37 rmsd  1.27 gap 24 (0.05%)

                  .    :    .    :    .    :    .    :    .    :    .    :    .    :
Chain 1:   43 TPPGAQPTAPGSLKAPDTRNEKLNSLEDVRKGSENYALTTNQGVRIADDQNSLRAGSRGPTLLEDFILRE
              1111111111111111              111111111111111111111111111111111111111
Chain 2:    4 SRDPASDQMQHWKEQR--------------AAQKADVLTTGAGNPVGDKLNVITVGPRGPLLVQDVVFTD

                  .    :    .    :    .    :    .    :    .    :    .    :    .    :
Chain 1:  113 KITHFDHERIPERIVHARGSAAHGYFQPYKSLSDITKADFLSDPNKITPVFVRFSTCQGGAGSADTVRDI
              1111111111111111111111111111111111111111111111111111111111111111111111
Chain 2:   60 EMAHFDRERIPERVVHAKGAGAFGYFEVTHDITKYSKAKVFEHIGKKTPIAVRFSTVAGESGSADTVRDP

                  .    :    .    :    .    :    .    :    .    :    .    :    .    :
Chain 1:  183 RGFATKFYTEEGIFDLVGNNTPIFFIQDAHKFPDFVHAVKPEPHWAIPQGQSAHDTFWDYVSLQPETLHN
              11111111111111111111111111111111111111111111111    111111111111111111
Chain 2:  130 RGFAVKFYTEDGNWDLVGNNTPIFFIRDPILFPSFIHSQKRNPQTHLK----DPDMVWDFWSLRPESLHQ

                  .    :    .    :    .    :    .    :    .    :    .    :    .    :
Chain 1:  253 VMWAMSDRGIPRSYRTMEGFGIHTFRLINAEGKATFVRFHWKPLAGKASLVWDEAQKLTGRDPDFHRREL
              1111111111111111111111111111111111111111111111111111111111111111111111
Chain 2:  196 VSFLFSDRGIPDGHRHMNGYGSHTFKLVNANGEAVYCKFHYKTDQGIKNLSVEDAARLSQEDPDYGIRDL

                  .    :    .    :    .    :    .    :    .    :    .    :    .    :
Chain 1:  323 WEAIEAGDFPEYELGFQLIPEEDEFKFDFDLLDPTKLIPEELVPVQRVGKMVLNRNPDNFFAENEQAAFH
              1111111111111111111111111111111111111111111111111111111111111111111111
Chain 2:  266 FNAIATGKYPSWTFYIQVMTFNQAETFPFNPFDLTKVWPHKDYPLIPVGKLVLNRNPVNYFAEVEQIAFD

                  .    :    .    :    .    :    .    :    .    :    .    :    .    :
Chain 1:  393 PGHIVPGLDFTNDPLLQGRLFSYTDTQISRLGGPNFHEIPINRPT-CPYHNFQRDGMHRMGI-DTNPANY
              111111111111111111111111111111 111111111111 111111111111111 1111111
Chain 2:  336 PSNMPPGIEASPDKMLQGRLFAYPDTHRHRL-GPNYLHIPVNCPYRARVANYQRDGPMCMQDNQGGAPNY

                  .    :    .    :    .    :    .    :    .    :    .    :    .    :
Chain 1:  461 EPNSINDNWPRETPPGPKRGGFESYQERVEGNKVRERSPSFGEYYSHPRLFWL-SQTPFEQRHIVDGFSF
              1111  111111111      111111111111111111 1111111111111 111111111111111
Chain 2:  405 YPNS--FGAPEQQPS------ALEHSIQYSGEVRRFNTA-NDDNVTQVRAFYVNVLNEEQRKRLCENIAG

                  .    :    .    :    .    :    .
Chain 1:  530 ELSKVVRPYIRERVVDQLAHIDLTLAQAVAKNLGIELT
              11111 111111111111111111111111111111
Chain 2:  466 HLKDA-QIFIQKKAVKNFTEVHPDYGSHIQALLDKYNA
```

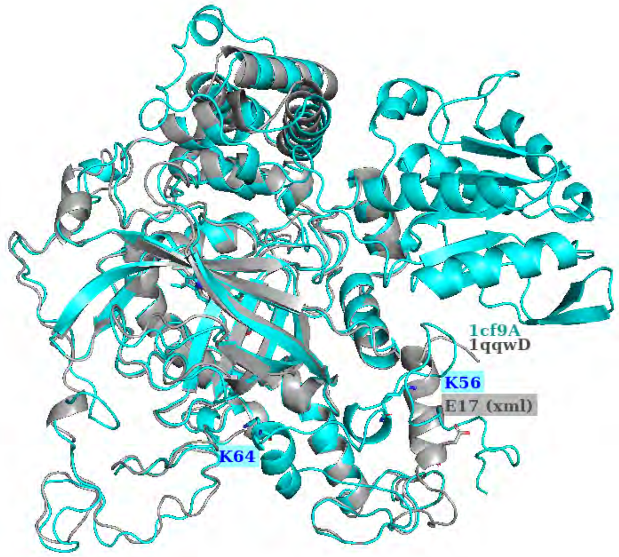

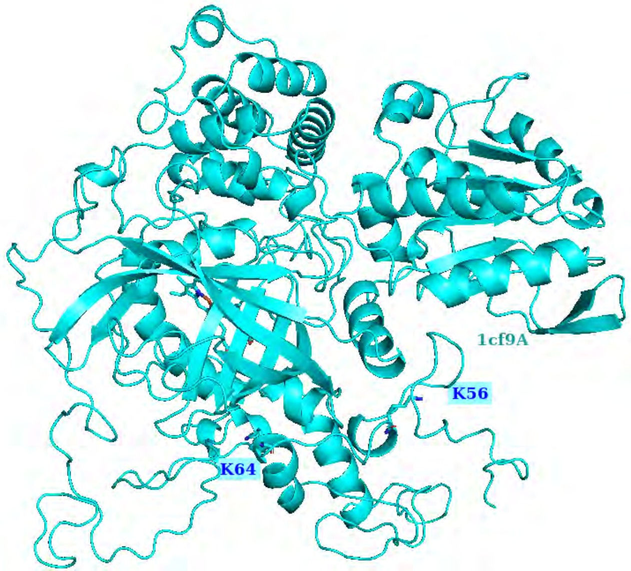

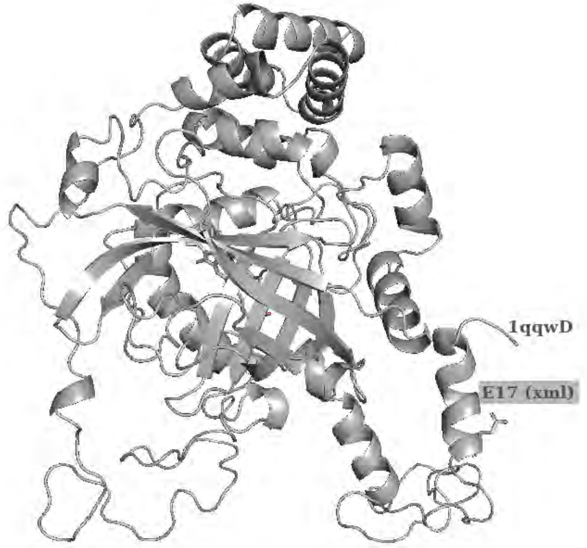

UniProt ID: P15202

PDB ID: 1A4E_C

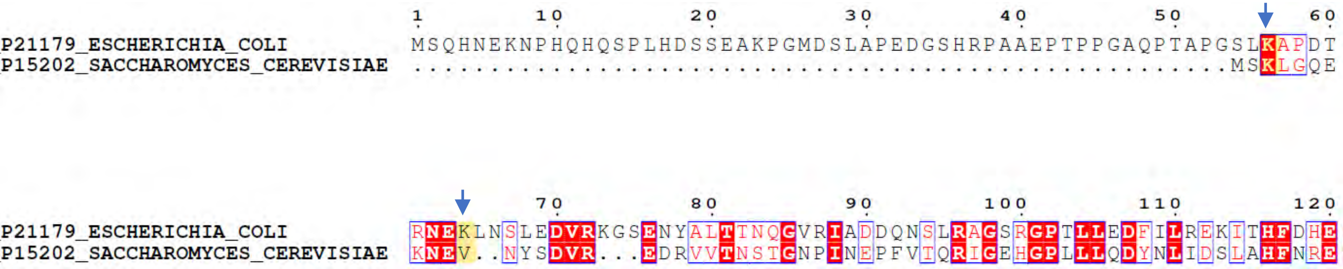

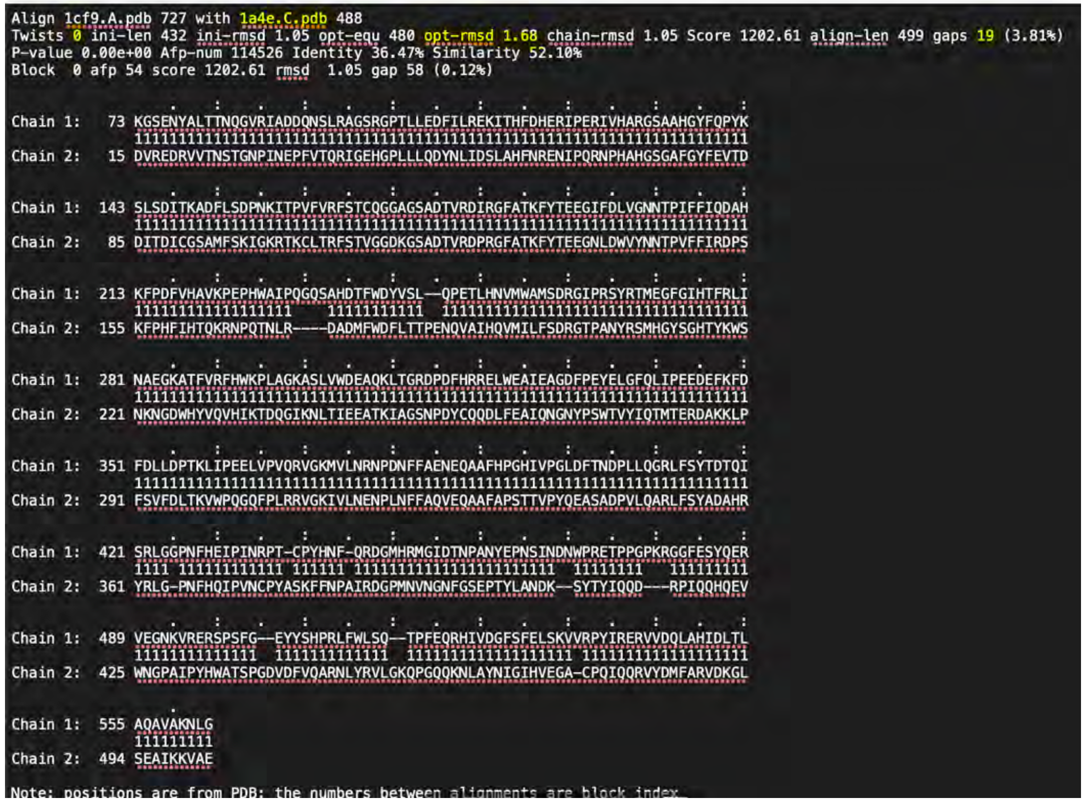

```
Align 1cf9.A.pdb 727 with 1a4e.C.pdb 488
Twists 0 ini-len 432 ini-rmsd 1.05 opt-equ 480 opt-rmsd 1.68 chain-rmsd 1.05 Score 1202.61 align-len 499 gaps 19 (3.81%)
P-value 0.00e+00 Afp-num 114526 Identity 36.47% Similarity 52.10%
Block  0 afp 54 score 1202.61 rmsd  1.05 gap 58 (0.12%)

                  .    :    .    :    .    :    .    :    .    :    .    :    .    :
Chain 1:   73 KGSENYALTTNQGVRIADDQNSLRAGSRGPTLLEDFILREKITHFDHERIPERIVHARGSAAHGYFQPYK
              1111111111111111111111111111111111111111111111111111111111111111111111
Chain 2:   15 DVREDRVVTNSTGNPINEPFVTQRIGEHGPLLLQDYNLIDSLAHFNRENIPQRNPHAHGSGAFGYFEVTD

                  .    :    .    :    .    :    .    :    .    :    .    :    .    :
Chain 1:  143 SLSDITKADFLSDPNKITPVFVRFSTCQGGAGSADTVRDIRGFATKFYTEEGIFDLVGNNTPIFFIQDAH
              1111111111111111111111111111111111111111111111111111111111111111111111
Chain 2:   85 DITDICGSAMFSKIGKRTKCLTRFSTVGGDKGSADTVRDPRGFATKFYTEEGNLDWVYNNTPVFFIRDPS

                  .    :    .    :    .    :    .    :    .    :    .    :    .    :
Chain 1:  213 KFPDFVHAVKPEPHWAIPQGQSAHDTFWDYVSL--QPETLHNVMWAMSDRGIPRSYRTMEGFGIHTFRLI
              111111111111111111    11111111111  11111111111111111111111111111111111
Chain 2:  155 KFPHFIHTQKRNPQTNLR----DADMFWDFLTTPENQVAIHQVMILFSDRGTPANYRSMHGYSGHTYKWS

                  .    :    .    :    .    :    .    :    .    :    .    :    .    :
Chain 1:  281 NAEGKATFVRFHWKPLAGKASLVWDEAQKLTGRDPDFHRRELWEAIEAGDFPEYELGFQLIPEEDEFKFD
              1111111111111111111111111111111111111111111111111111111111111111111111
Chain 2:  221 NKNGDWHYVQVHIKTDQGIKNLTIEEATKIAGSNPDYCQQDLFEAIQNGNYPSWTVYIQTMTERDAKKLP

                  .    :    .    :    .    :    .    :    .    :    .    :    .    :
Chain 1:  351 FDLLDPTKLIPEELVPVQRVGKMVLNRNPDNFFAENEQAAFHPGHIVPGLDFTNDPLLQGRLFSYTDTQI
              1111111111111111111111111111111111111111111111111111111111111111111111
Chain 2:  291 FSVFDLTKVWPQGQFPLRRVGKIVLNENPLNFFAQVEQAAFAPSTTVPYQEASADPVLQARLFSYADAHR

                  .    :    .    :    .    :    .    :    .    :    .    :    .    :
Chain 1:  421 SRLGGPNFHEIPINRPT-CPYHNF-QRDGMHRMGIDTNPANYEPNSINDNWPRETPPGPKRGGFESYQER
              1111 111111111111 111111 11111111111111111111111  11111111   111111111
Chain 2:  361 YRLG-PNFHQIPVNCPYASKFFNPAIRDGPMNVNGNFGSEPTYLANDK--SYTYIQQD---RPIQQHQEV

                  .    :    .    :    .    :    .    :    .    :    .    :    .    :
Chain 1:  489 VEGNKVRERSPSFG--EYYSHPRLFWLSQ--TPFEQRHIVDGFSFELSKVVRPYIRERVVDQLAHIDLTL
              11111111111111  1111111111111  1111111111111111111 1111111111111111111
Chain 2:  425 WNGPAIPYHWATSPGDVDFVQARNLYRVLGKQPGQQKNLAYNIGIHVEGA-CPQIQQRVYDMFARVDKGL

                  .
Chain 1:  555 AQAVAKNLG
              111111111
Chain 2:  494 SEAIKKVAE

Note: positions are from PDB; the numbers between alignments are block index
```

Full sequences in supplemental file.

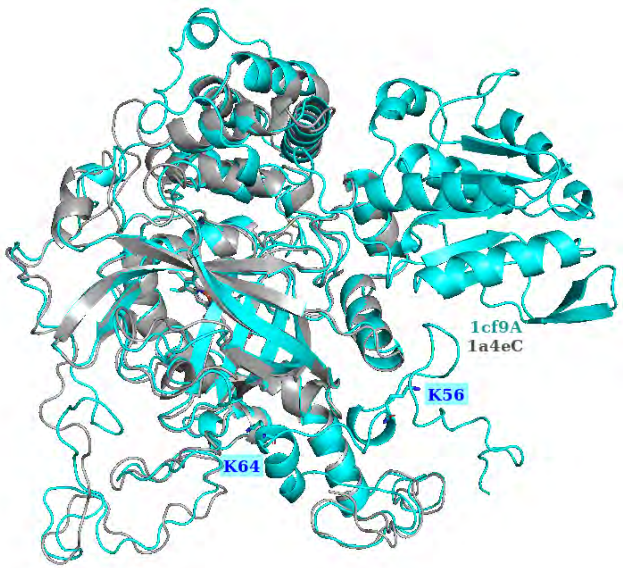

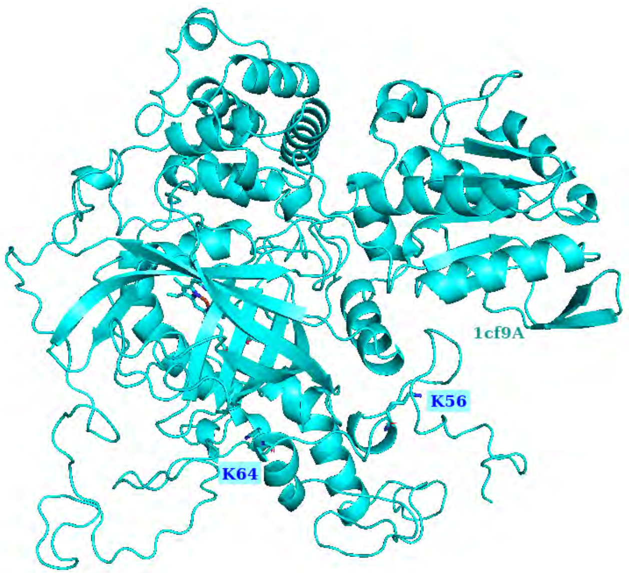

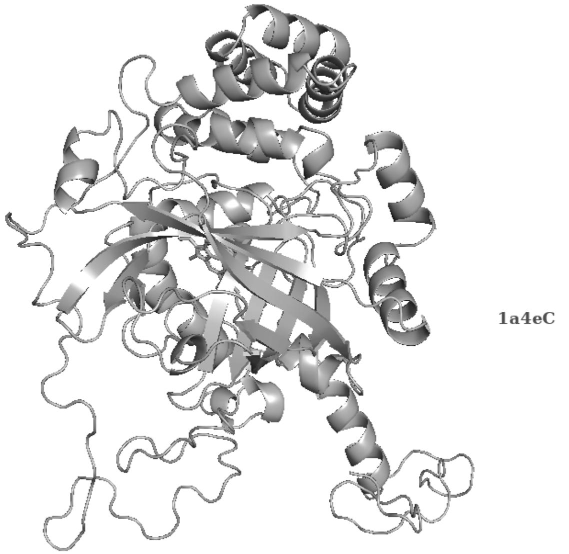

UniProt ID: P21179

PDB ID: 1GG9_B

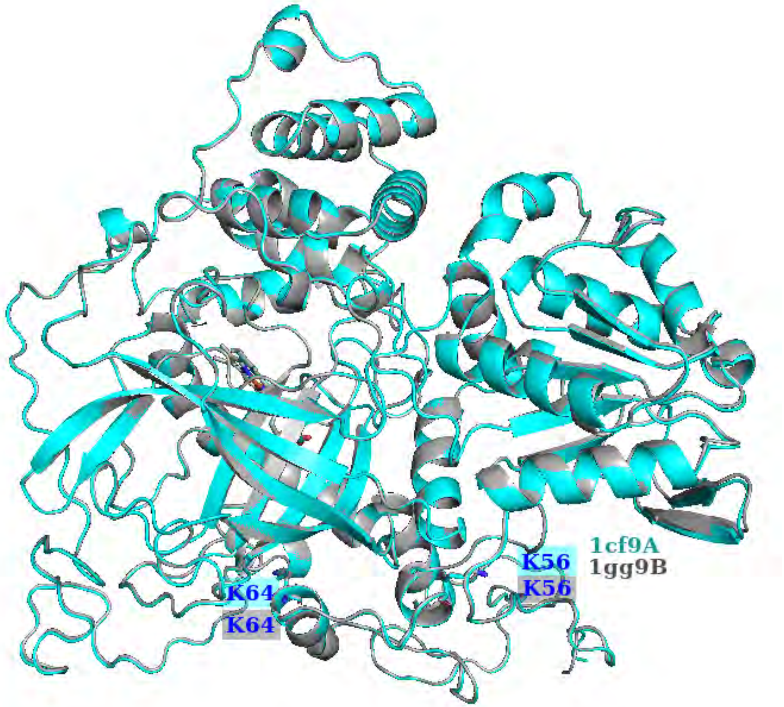


```
Align 1cf9.A.pdb 727 with 1gg9.B.pdb 727
Twists 0 ini-len 720 ini-rmsd 0.25 opt-equ 727 opt-rmsd 0.29 chain-rmsd 0.25 Score 2158.43 align-len 727 gaps 0 (0.00%)
P-value 0.00e+00 Afp-num 167754 Identity 99.72% Similarity 99.86%
Block  0 afp 90 score 2158.43 rmsd  0.25 gap 0 (0.00%)

                   .    :    .    :    .    :    .    :    .    :    .    :    .    :
Chain 1:   27 DSLAPEDGSHRPAAEPTPPGAQPTAPGSLKAPDTRNEKLNSLEDVRKGSENYALTTNQGVRIADDQNSLR
              1111111111111111111111111111111111111111111111111111111111111111111111
Chain 2:   27 DSLAPEDGSHRPAAEPTPPGAQPTAPGSLKAPDTRNEKLNSLEDVRKGSENYALTTNQGVRIADDQNSLR

                   .    :    .    :    .    :    .    :    .    :    .    :    .    :
Chain 1:   97 AGSRGPTLLEDFILREKITHFDHERIPERIVHARGSAAHGYFQPYKSLSDITKADFLSDPNKITPVFVRF
              1111111111111111111111111111111111111111111111111111111111111111111111
Chain 2:   97 AGSRGPTLLEDFILREKITHFDHERIPERIVNARGSAAHGYFQPYKSLSDITKADFLSDPNKITPVFVRF

                   .    :    .    :    .    :    .    :    .    :    .    :    .    :
Chain 1:  167 STCQGGAGSADTVRDIRGFATKFYTEEGIFDLVGNNTPIFFIQDAHKFPDFVHAVKPEPHWAIPQGQSAH
              1111111111111111111111111111111111111111111111111111111111111111111111
Chain 2:  167 STVQGGAGSADTVRDIRGFATKFYTEEGIFDLVGNNTPIFFIQDAHKFPDFVHAVKPEPHWAIPQGQSAH

                   .    :    .    :    .    :    .    :    .    :    .    :    .    :
Chain 1:  237 DTFWDYVSLQPETLHNVMWAMSDRGIPRSYRTMEGFGIHTFRLINAEGKATFVRFHWKPLAGKASLVWDE
              1111111111111111111111111111111111111111111111111111111111111111111111
Chain 2:  237 DTFWDYVSLQPETLHNVMWAMSDRGIPRSYRTMEGFGIHTFRLINAEGKATFVRFHWKPLAGKASLVWDE

                   .    :    .    :    .    :    .    :    .    :    .    :    .    :
Chain 1:  307 AQKLTGRDPDFHRRELWEAIEAGDFPEYELGFQLIPEEDEFKFDFDLLDPTKLIPEELVPVQRVGKMVLN
              1111111111111111111111111111111111111111111111111111111111111111111111
Chain 2:  307 AQKLTGRDPDFHRRELWEAIEAGDFPEYELGFQLIPEEDEFKFDFDLLDPTKLIPEELVPVQRVGKMVLN

                   .    :    .    :    .    :    .    :    .    :    .    :    .    :
Chain 1:  377 RNPDNFFAENEQAAFHPGHIVPGLDFTNDPLLQGRLFSYTDTQISRLGGPNFHEIPINRPTCPYHNFQRD
              1111111111111111111111111111111111111111111111111111111111111111111111
Chain 2:  377 RNPDNFFAENEQAAFHPGHIVPGLDFTNDPLLQGRLFSYTDTQISRLGGPNFHEIPINRPTCPYHNFQRD

                   .    :    .    :    .    :    .    :    .    :    .    :    .    :
Chain 1:  447 GMHRMGIDTNPANYEPNSINDNWPRETPPGPKRGGFESYQERVEGNKVRERSPSFGEYYSHPRLFWLSQT
              1111111111111111111111111111111111111111111111111111111111111111111111
Chain 2:  447 GMHRMGIDTNPANYEPNSINDNWPRETPPGPKRGGFESYQERVEGNKVRERSPSFGEYYSHPRLFWLSQT

                   .    :    .    :    .    :    .    :    .    :    .    :    .    :
Chain 1:  517 PFEQRHIVDGFSFELSKVVRPYIRERVVDQLAHIDLTLAQAVAKNLGIELTDDQLNITPPPDVNGLKKDP
              1111111111111111111111111111111111111111111111111111111111111111111111
Chain 2:  517 PFEQRHIVDGFSFELSKVVRPYIRERVVDQLAHIDLTLAQAVAKNLGIELTDDQLNITPPPDVNGLKKDP

                   .    :    .    :    .    :    .    :    .    :    .    :    .    :
Chain 1:  587 SLSLYAIPDGDVKGRVVAILLNDEVRSADLLAILKALKAKGVHAKLLYSRMGEVTADDGTVLPIAATFAG
              1111111111111111111111111111111111111111111111111111111111111111111111
Chain 2:  587 SLSLYAIPDGDVKGRVVAILLNDEVRSADLLAILKALKAKGVHAKLLYSRMGEVTADDGTVLPIAATFAG

                   .    :    .    :    .    :    .    :    .    :    .    :    .    :
Chain 1:  657 APSLTVDAVIVPCGNIADIADNGDANYYLMEAYKHLKPIALAGDARKFKATIKIADQGEEGIVEADSADG
              1111111111111111111111111111111111111111111111111111111111111111111111
Chain 2:  657 APSLTVDAVIVPCGNIADIADNGDANYYLMEAYKHLKPIALAGDARKFKATIKIADQGEEGIVEADSADG

                   .    :    .    :    .
Chain 1:  727 SFMDELLTLMAAHRVWSRIPKIDKIPA
              111111111111111111111111111
Chain 2:  727 SFMDELLTLMAAHRVWSRIPKIDKIPA

Note: positions are from PDB; the numbers between alignments are block index
```

UniProt ID: P21179
PDB ID: 1GGE_B

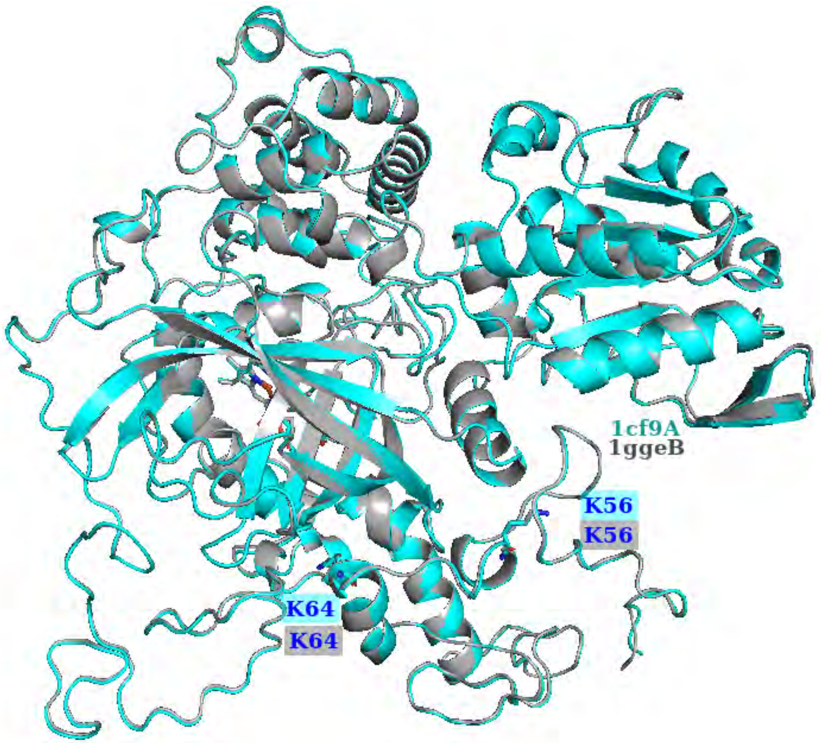


```
Align 1cf9.A.pdb 727 with 1gge.B.pdb 727
Twists 0 ini-len 720 ini-rmsd 0.25 opt-equ 727 opt-rmsd 0.29 chain-rmsd 0.25 Score 2158.07 align-len 727 gaps 0 (0.00%)
P-value 0.00e+00 Afp-num 167771 Identity 99.86% Similarity 99.86%
Block  0 afp 90 score 2158.07 rmsd  0.25 gap 0 (0.00%)

                    .    :    .    :    .    :    .    :    .    :    .    :    .    :
Chain 1:   27 DSLAPEDGSHRPAAEPTPPGAQPTAPGSLKAPDTRNEKLNSLEDVRKGSENYALTTNQGVRIADDQNSLR
              1111111111111111111111111111111111111111111111111111111111111111111111
Chain 2:   27 DSLAPEDGSHRPAAEPTPPGAQPTAPGSLKAPDTRNEKLNSLEDVRKGSENYALTTNQGVRIADDQNSLR

                    .    :    .    :    .    :    .    :    .    :    .    :    .    :
Chain 1:   97 AGSRGPTLLEDFILREKITHFDHERIPERIVHARGSAAHGYFQPYKSLSDITKADFLSDPNKITPVFVRF
              1111111111111111111111111111111111111111111111111111111111111111111111
Chain 2:   97 AGSRGPTLLEDFILREKITHFDHERIPERIVHARGSAAHGYFQPYKSLSDITKADFLSDPNKITPVFVRF

                    .    :    .    :    .    :    .    :    .    :    .    :    .    :
Chain 1:  167 STCQGGAGSADTVRDIRGFATKFYTEEGIFDLVGNNTPIFFIQDAHKFPDFVHAVKPEPHWAIPQGQSAH
              1111111111111111111111111111111111111111111111111111111111111111111111
Chain 2:  167 STVQGGAGSADTVRDIRGFATKFYTEEGIFDLVGNNTPIFFIQDAHKFPDFVHAVKPEPHWAIPQGQSAH

                    .    :    .    :    .    :    .    :    .    :    .    :    .    :
Chain 1:  237 DTFWDYVSLQPETLHNVMWAMSDRGIPRSYRTMEGFGIHTFRLINAEGKATFVRFHWKPLAGKASLVWDE
              1111111111111111111111111111111111111111111111111111111111111111111111
Chain 2:  237 DTFWDYVSLQPETLHNVMWAMSDRGIPRSYRTMEGFGIHTFRLINAEGKATFVRFHWKPLAGKASLVWDE

                    .    :    .    :    .    :    .    :    .    :    .    :    .    :
Chain 1:  307 AQKLTGRDPDFHRRELWEAIEAGDFPEYELGFQLIPEEDEFKFDFDLLDPTKLIPEELVPVQRVGKMVLN
              1111111111111111111111111111111111111111111111111111111111111111111111
Chain 2:  307 AQKLTGRDPDFHRRELWEAIEAGDFPEYELGFQLIPEEDEFKFDFDLLDPTKLIPEELVPVQRVGKMVLN

                    .    :    .    :    .    :    .    :    .    :    .    :    .    :
Chain 1:  377 RNPDNFFAENEQAAFHPGHIVPGLDFTNDPLLQGRLFSYTDTQISRLGGPNFHEIPINRPTCPYHNFQRD
              1111111111111111111111111111111111111111111111111111111111111111111111
Chain 2:  377 RNPDNFFAENEQAAFHPGHIVPGLDFTNDPLLQGRLFSYTDTQISRLGGPNFHEIPINRPTCPYHNFQRD

                    .    :    .    :    .    :    .    :    .    :    .    :    .    :
Chain 1:  447 GMHRMGIDTNPANYEPNSINDNWPRETPPGPKRGGFESYQERVEGNKVRERSPSFGEYYSHPRLFWLSQT
              1111111111111111111111111111111111111111111111111111111111111111111111
Chain 2:  447 GMHRMGIDTNPANYEPNSINDNWPRETPPGPKRGGFESYQERVEGNKVRERSPSFGEYYSHPRLFWLSQT

                    .    :    .    :    .    :    .    :    .    :    .    :    .    :
Chain 1:  517 PFEQRHIVDGFSFELSKVVRPYIRERVVDQLAHIDLTLAQAVAKNLGIELTDDQLNITPPPDVNGLKKDP
              1111111111111111111111111111111111111111111111111111111111111111111111
Chain 2:  517 PFEQRHIVDGFSFELSKVVRPYIRERVVDQLAHIDLTLAQAVAKNLGIELTDDQLNITPPPDVNGLKKDP

                    .    :    .    :    .    :    .    :    .    :    .    :    .    :
Chain 1:  587 SLSLYAIPDGDVKGRVVAILLNDEVRSADLLAILKALKAKGVHAKLLYSRMGEVTADDGTVLPIAATFAG
              1111111111111111111111111111111111111111111111111111111111111111111111
Chain 2:  587 SLSLYAIPDGDVKGRVVAILLNDEVRSADLLAILKALKAKGVHAKLLYSRMGEVTADDGTVLPIAATFAG

                    .    :    .    :    .    :    .    :    .    :    .    :    .    :
Chain 1:  657 APSLTVDAVIVPCGNIADIADNGDANYYLMEAYKHLKPIALAGDARKFKATIKIADQGEEGIVEADSADG
              1111111111111111111111111111111111111111111111111111111111111111111111
Chain 2:  657 APSLTVDAVIVPCGNIADIADNGDANYYLMEAYKHLKPIALAGDARKFKATIKIADQGEEGIVEADSADG

                    .    :    .    :    .
Chain 1:  727 SFMDELLTLMAAHRVWSRIPKIDKIPA
              111111111111111111111111111
Chain 2:  727 SFMDELLTLMAAHRVWSRIPKIDKIPA
```

UniProt ID: P21179
PDB ID: 1GGF_D

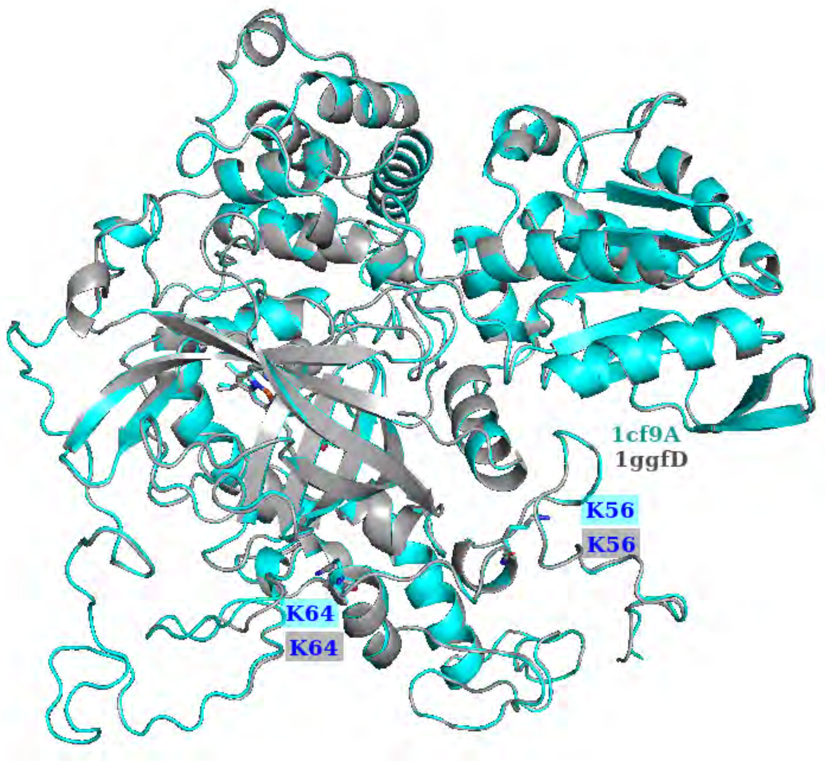

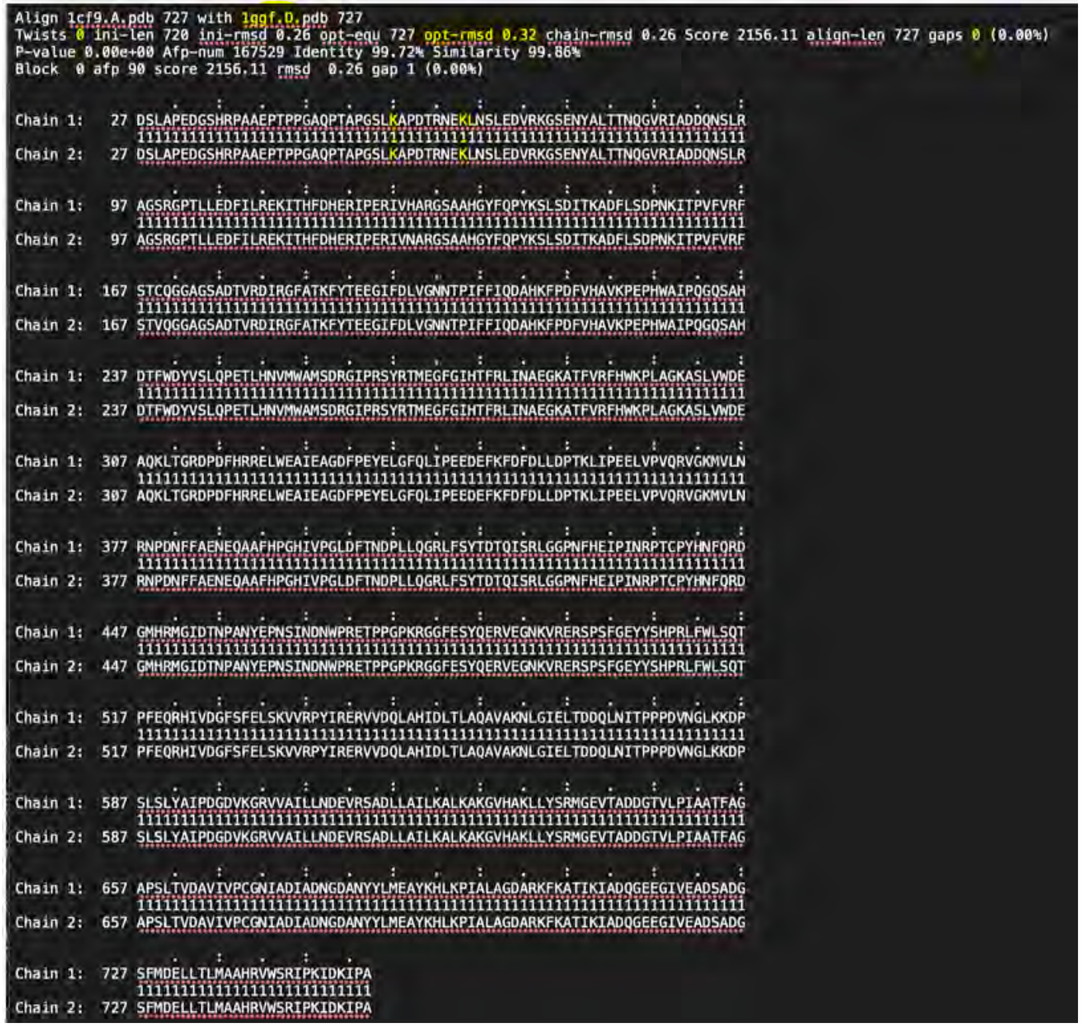

```
Align 1cf9.A.pdb 727 with 1ggf.D.pdb 727
Twists 0 ini-len 720 ini-rmsd 0.26 opt-equ 727 opt-rmsd 0.32 chain-rmsd 0.26 Score 2156.11 align-len 727 gaps 0 (0.00%)
P-value 0.00e+00 Afp-num 167529 Identity 99.72% Similarity 99.86%
Block  0 afp 90 score 2156.11 rmsd  0.26 gap 1 (0.00%)

Chain 1:   27 DSLAPEDGSHRPAAEPTPPGAQPTAPGSLKAPDTRNEKLNSLEDVRKGSENYALTTNQGVRIADDQNSLR
              1111111111111111111111111111111111111111111111111111111111111111111111
Chain 2:   27 DSLAPEDGSHRPAAEPTPPGAQPTAPGSLKAPDTRNEKLNSLEDVRKGSENYALTTNQGVRIADDQNSLR

Chain 1:   97 AGSRGPTLLEDFILREKITHFDHERIPERIVHARGSAAHGYFQPYKSLSDITKADFLSDPNKITPVFVRF
              1111111111111111111111111111111111111111111111111111111111111111111111
Chain 2:   97 AGSRGPTLLEDFILREKITHFDHERIPERIVNARGSAAHGYFQPYKSLSDITKADFLSDPNKITPVFVRF

Chain 1:  167 STCQGGAGSADTVRDIRGFATKFYTEEGIFDLVGNNTPIFFIQDAHKFPDFVHAVKPEPHWAIPQGQSAH
              1111111111111111111111111111111111111111111111111111111111111111111111
Chain 2:  167 STVQGGAGSADTVRDIRGFATKFYTEEGIFDLVGNNTPIFFIQDAHKFPDFVHAVKPEPHWAIPQGQSAH

Chain 1:  237 DTFWDYVSLQPETLHNVMWAMSDRGIPRSYRTMEGFGIHTFRLINAEGKATFVRFHWKPLAGKASLVWDE
              1111111111111111111111111111111111111111111111111111111111111111111111
Chain 2:  237 DTFWDYVSLQPETLHNVMWAMSDRGIPRSYRTMEGFGIHTFRLINAEGKATFVRFHWKPLAGKASLVWDE

Chain 1:  307 AQKLTGRDPDFHRRELWEAIEAGDFPEYELGFQLIPEEDEFKFDFDLLDPTKLIPEELVPVQRVGKMVLN
              1111111111111111111111111111111111111111111111111111111111111111111111
Chain 2:  307 AQKLTGRDPDFHRRELWEAIEAGDFPEYELGFQLIPEEDEFKFDFDLLDPTKLIPEELVPVQRVGKMVLN

Chain 1:  377 RNPDNFFAENEQAAFHPGHIVPGLDFTNDPLLQGRLFSYTDTQISRLGGPNFHEIPINRPTCPYHNFQRD
              1111111111111111111111111111111111111111111111111111111111111111111111
Chain 2:  377 RNPDNFFAENEQAAFHPGHIVPGLDFTNDPLLQGRLFSYTDTQISRLGGPNFHEIPINRPTCPYHNFQRD

Chain 1:  447 GMHRMGIDTNPANYEPNSINDNWPRETPPGPKRGGFESYQERVEGNKVRERSPSFGEYYSHPRLFWLSQT
              1111111111111111111111111111111111111111111111111111111111111111111111
Chain 2:  447 GMHRMGIDTNPANYEPNSINDNWPRETPPGPKRGGFESYQERVEGNKVRERSPSFGEYYSHPRLFWLSQT

Chain 1:  517 PFEQRHIVDGFSFELSKVVRPYIRERVVDQLAHIDLTLAQAVAKNLGIELTDDQLNITPPPDVNGLKKDP
              1111111111111111111111111111111111111111111111111111111111111111111111
Chain 2:  517 PFEQRHIVDGFSFELSKVVRPYIRERVVDQLAHIDLTLAQAVAKNLGIELTDDQLNITPPPDVNGLKKDP

Chain 1:  587 SLSLYAIPDGDVKGRVVAILLNDEVRSADLLAILKALKAKGVHAKLLYSRMGEVTADDGTVLPIAATFAG
              1111111111111111111111111111111111111111111111111111111111111111111111
Chain 2:  587 SLSLYAIPDGDVKGRVVAILLNDEVRSADLLAILKALKAKGVHAKLLYSRMGEVTADDGTVLPIAATFAG

Chain 1:  657 APSLTVDAVIVPCGNIADIADNGDANYYLMEAYKHLKPIALAGDARKFKATIKIADQGEEGIVEADSADG
              1111111111111111111111111111111111111111111111111111111111111111111111
Chain 2:  657 APSLTVDAVIVPCGNIADIADNGDANYYLMEAYKHLKPIALAGDARKFKATIKIADQGEEGIVEADSADG

Chain 1:  727 SFMDELLTLMAAHRVWSRIPKIDKIPA
              111111111111111111111111111
Chain 2:  727 SFMDELLTLMAAHRVWSRIPKIDKIPA
```

UniProt ID: P21179

PDB ID: 1GGH_B

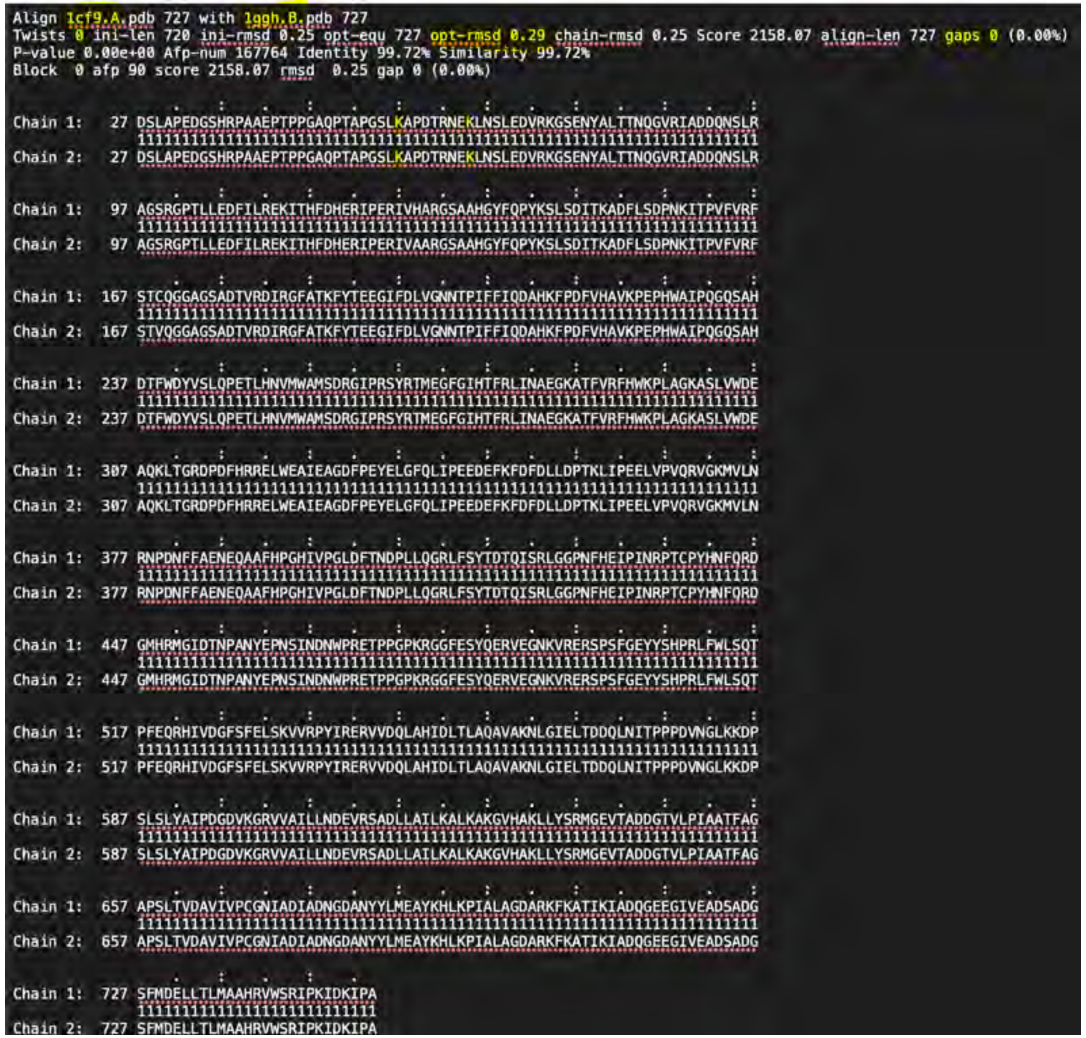

```
Align 1cf9.A.pdb 727 with 1ggh.B.pdb 727
Twists 0 ini-len 720 ini-rmsd 0.25 opt-equ 727 opt-rmsd 0.29 chain-rmsd 0.25 Score 2158.07 align-len 727 gaps 0 (0.00%)
P-value 0.00e+00 Afp-num 167764 Identity 99.72% Similarity 99.72%
Block  0 afp 90 score 2158.07 rmsd  0.25 gap 0 (0.00%)

Chain 1:   27 DSLAPEDGSHRPAAEPTPPGAQPTAPGSLKAPDTRNEKLNSLEDVRKGSENYALTTNQGVRIADDQNSLR
              1111111111111111111111111111111111111111111111111111111111111111111111
Chain 2:   27 DSLAPEDGSHRPAAEPTPPGAQPTAPGSLKAPDTRNEKLNSLEDVRKGSENYALTTNQGVRIADDQNSLR

Chain 1:   97 AGSRGPTLLEDFILREKITHFDHERIPERIVHARGSAAHGYFQPYKSLSDITKADFLSDPNKITPVFVRF
              1111111111111111111111111111111111111111111111111111111111111111111111
Chain 2:   97 AGSRGPTLLEDFILREKITHFDHERIPERIVAARGSAAHGYFQPYKSLSDITKADFLSDPNKITPVFVRF

Chain 1:  167 STCQGGAGSADTVRDIRGFATKFYTEEGIFDLVGNNTPIFFIQDAHKFPDFVHAVKPEPHWAIPQGQSAH
              1111111111111111111111111111111111111111111111111111111111111111111111
Chain 2:  167 STVQGGAGSADTVRDIRGFATKFYTEEGIFDLVGNNTPIFFIQDAHKFPDFVHAVKPEPHWAIPQGQSAH

Chain 1:  237 DTFWDYVSLQPETLHNVMWAMSDRGIPRSYRTMEGFGIHTFRLINAEGKATFVRFHWKPLAGKASLVWDE
              1111111111111111111111111111111111111111111111111111111111111111111111
Chain 2:  237 DTFWDYVSLQPETLHNVMWAMSDRGIPRSYRTMEGFGIHTFRLINAEGKATFVRFHWKPLAGKASLVWDE

Chain 1:  307 AQKLTGRDPDFHRRELWEAIEAGDFPEYELGFQLIPEEDEFKFDFDLLDPTKLIPEELVPVQRVGKMVLN
              1111111111111111111111111111111111111111111111111111111111111111111111
Chain 2:  307 AQKLTGRDPDFHRRELWEAIEAGDFPEYELGFQLIPEEDEFKFDFDLLDPTKLIPEELVPVQRVGKMVLN

Chain 1:  377 RNPDNFFAENEQAAFHPGHIVPGLDFTNDPLLQGRLFSYTDTQISRLGGPNFHEIPINRPTCPYHNFQRD
              1111111111111111111111111111111111111111111111111111111111111111111111
Chain 2:  377 RNPDNFFAENEQAAFHPGHIVPGLDFTNDPLLQGRLFSYTDTQISRLGGPNFHEIPINRPTCPYHNFQRD

Chain 1:  447 GMHRMGIDTNPANYEPNSINDNWPRETPPGPKRGGFESYQERVEGNKVRERSPSFGEYYSHPRLFWLSQT
              1111111111111111111111111111111111111111111111111111111111111111111111
Chain 2:  447 GMHRMGIDTNPANYEPNSINDNWPRETPPGPKRGGFESYQERVEGNKVRERSPSFGEYYSHPRLFWLSQT

Chain 1:  517 PFEQRHIVDGFSFELSKVVRPYIRERVVDQLAHIDLTLAQAVAKNLGIELTDDQLNITPPPDVNGLKKDP
              1111111111111111111111111111111111111111111111111111111111111111111111
Chain 2:  517 PFEQRHIVDGFSFELSKVVRPYIRERVVDQLAHIDLTLAQAVAKNLGIELTDDQLNITPPPDVNGLKKDP

Chain 1:  587 SLSLYAIPDGDVKGRVVAILLNDEVRSADLLAILKALKAKGVHAKLLYSRMGEVTADDGTVLPIAATFAG
              1111111111111111111111111111111111111111111111111111111111111111111111
Chain 2:  587 SLSLYAIPDGDVKGRVVAILLNDEVRSADLLAILKALKAKGVHAKLLYSRMGEVTADDGTVLPIAATFAG

Chain 1:  657 APSLTVDAVIVPCGNIADIADNGDANYYLMEAYKHLKPIALAGDARKFKATIKIADQGEEGIVEADSADG
              1111111111111111111111111111111111111111111111111111111111111111111111
Chain 2:  657 APSLTVDAVIVPCGNIADIADNGDANYYLMEAYKHLKPIALAGDARKFKATIKIADQGEEGIVEADSADG

Chain 1:  727 SFMDELLTLMAAHRVWSRIPKIDKIPA
              111111111111111111111111111
Chain 2:  727 SFMDELLTLMAAHRVWSRIPKIDKIPA
```

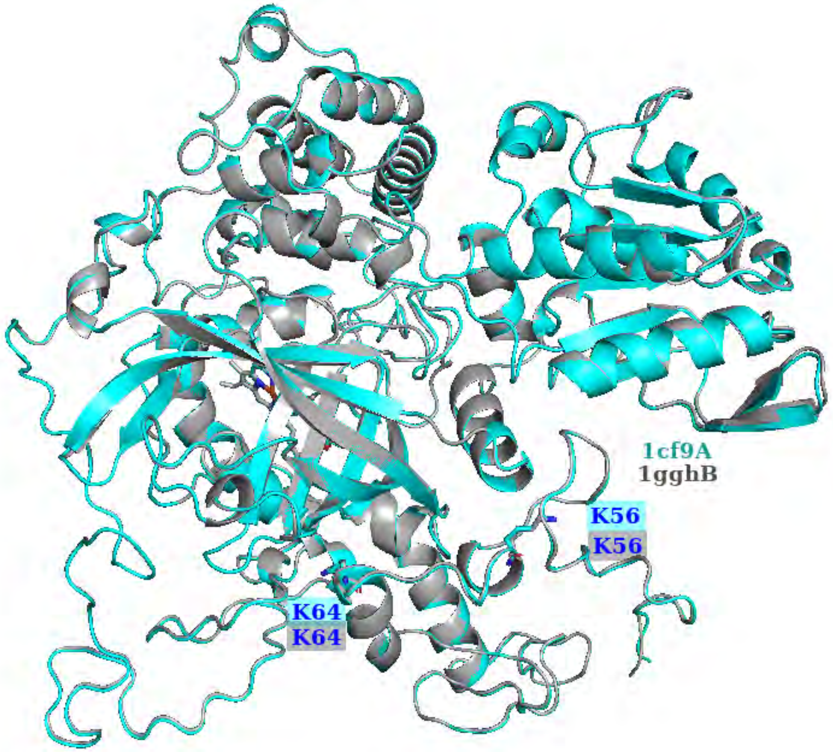

UniProt ID: P21179

PDB ID: 1GGJ_B

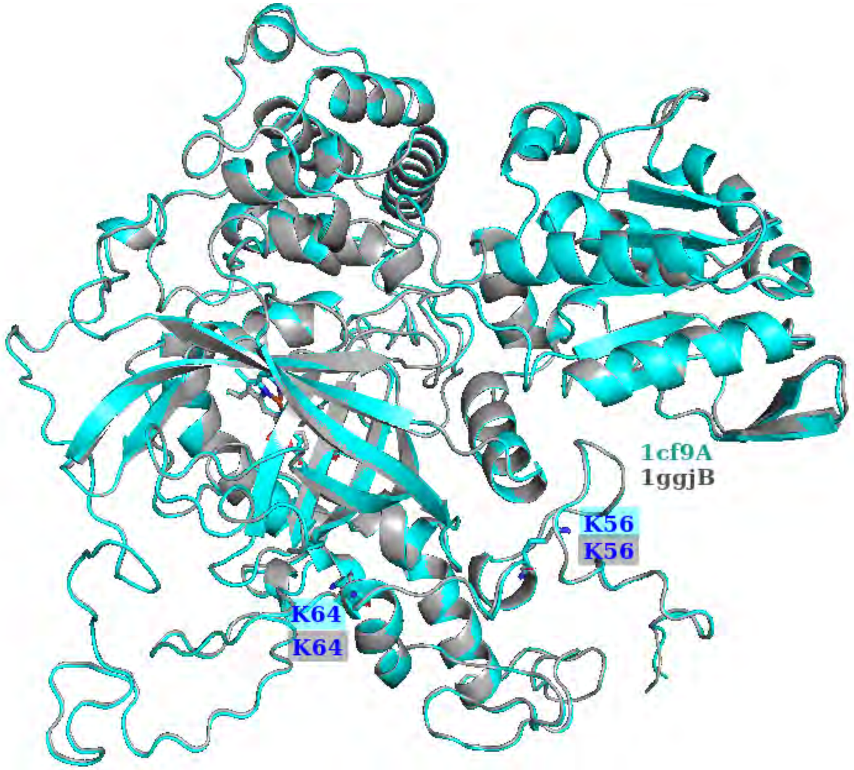


```
Align 1cf9.A.pdb 727 with 1ggj.B.pdb 727
Twists 0 ini-len 720 ini-rmsd 0.25 opt-equ 727 opt-rmsd 0.29 chain-rmsd 0.25 Score 2157.88 align-len 727 gaps 0 (0.00%)
P-value 0.00e+00 Afp-num 167679 Identity 99.72% Similarity 99.72%
Block  0 afp 90 score 2157.88 rmsd  0.25 gap 0 (0.00%)

                   .    :    .    :    .    :    .    :    .    :    .    :    .    :
Chain 1:    27 DSLAPEDGSHRPAAEPTPPGAQPTAPGSLKAPDTRNEKLNSLEDVRKGSENYALTTNQGVRIADDQNSLR
               1111111111111111111111111111111111111111111111111111111111111111111111
Chain 2:    27 DSLAPEDGSHRPAAEPTPPGAQPTAPGSLKAPDTRNEKLNSLEDVRKGSENYALTTNQGVRIADDQNSLR

                   .    :    .    :    .    :    .    :    .    :    .    :    .    :
Chain 1:    97 AGSRGPTLLEDFILREKITHFDHERIPERIVHARGSAAHGYFQPYKSLSDITKADFLSDPNKITPVFVRF
               1111111111111111111111111111111111111111111111111111111111111111111111
Chain 2:    97 AGSRGPTLLEDFILREKITHFDHERIPERIVHARGSAAHGYFQPYKSLSDITKADFLSDPNKITPVFVRF

                   .    :    .    :    .    :    .    :    .    :    .    :    .    :
Chain 1:   167 STCQGGAGSADTVRDIRGFATKFYTEEGIFDLVGNNTPIFFIQDAHKFPDFVHAVKPEPHWAIPQGQSAH
               1111111111111111111111111111111111111111111111111111111111111111111111
Chain 2:   167 STVQGGAGSADTVRDIRGFATKFYTEEGIFDLVGANTPIFFIQDAHKFPDFVHAVKPEPHWAIPQGQSAH

                   .    :    .    :    .    :    .    :    .    :    .    :    .    :
Chain 1:   237 DTFWDYVSLQPETLHNVMWAMSDRGIPRSYRTMEGFGIHTFRLINAEGKATFVRFHWKPLAGKASLVWDE
               1111111111111111111111111111111111111111111111111111111111111111111111
Chain 2:   237 DTFWDYVSLQPETLHNVMWAMSDRGIPRSYRTMEGFGIHTFRLINAEGKATFVRFHWKPLAGKASLVWDE

                   .    :    .    :    .    :    .    :    .    :    .    :    .    :
Chain 1:   307 AQKLTGRDPDFHRRELWEAIEAGDFPEYELGFQLIPEEDEFKFDFDLLDPTKLIPEELVPVQRVGKMVLN
               1111111111111111111111111111111111111111111111111111111111111111111111
Chain 2:   307 AQKLTGRDPDFHRRELWEAIEAGDFPEYELGFQLIPEEDEFKFDFDLLDPTKLIPEELVPVQRVGKMVLN

                   .    :    .    :    .    :    .    :    .    :    .    :    .    :
Chain 1:   377 RNPDNFFAENEQAAFHPGHIVPGLDFTNDPLLQGRLFSYTDTQISRLGGPNFHEIPINRPTCPYHNFQRD
               1111111111111111111111111111111111111111111111111111111111111111111111
Chain 2:   377 RNPDNFFAENEQAAFHPGHIVPGLDFTNDPLLQGRLFSYTDTQISRLGGPNFHEIPINRPTCPYHNFQRD

                   .    :    .    :    .    :    .    :    .    :    .    :    .    :
Chain 1:   447 GMHRMGIDTNPANYEPNSINDNWPRETPPGPKRGGFESYQERVEGNKVRERSPSFGEYYSHPRLFWLSQT
               1111111111111111111111111111111111111111111111111111111111111111111111
Chain 2:   447 GMHRMGIDTNPANYEPNSINDNWPRETPPGPKRGGFESYQERVEGNKVRERSPSFGEYYSHPRLFWLSQT

                   .    :    .    :    .    :    .    :    .    :    .    :    .    :
Chain 1:   517 PFEQRHIVDGFSFELSKVVRPYIRERVVDQLAHIDLTLAQAVAKNLGIELTDDQLNITPPPDVNGLKKDP
               1111111111111111111111111111111111111111111111111111111111111111111111
Chain 2:   517 PFEQRHIVDGFSFELSKVVRPYIRERVVDQLAHIDLTLAQAVAKNLGIELTDDQLNITPPPDVNGLKKDP

                   .    :    .    :    .    :    .    :    .    :    .    :    .    :
Chain 1:   587 SLSLYAIPDGDVKGRVVAILLNDEVRSADLLAILKALKAKGVHAKLLYSRMGEVTADDGTVLPIAATFAG
               1111111111111111111111111111111111111111111111111111111111111111111111
Chain 2:   587 SLSLYAIPDGDVKGRVVAILLNDEVRSADLLAILKALKAKGVHAKLLYSRMGEVTADDGTVLPIAATFAG

                   .    :    .    :    .    :    .    :    .    :    .    :    .    :
Chain 1:   657 APSLTVDAVIVPCGNIADIADNGDANYYLMEAYKHLKPIALAGDARKFKATIKIADQGEEGIVEADSADG
               1111111111111111111111111111111111111111111111111111111111111111111111
Chain 2:   657 APSLTVDAVIVPCGNIADIADNGDANYYLMEAYKHLKPIALAGDARKFKATIKIADQGEEGIVEADSADG

                   .    :    .    :    .
Chain 1:   727 SFMDELLTLMAAHRVWSRIPKIDKIPA
               111111111111111111111111111
Chain 2:   727 SFMDELLTLMAAHRVWSRIPKIDKIPA
```

UniProt ID: P21179
PDB ID: 1GGK_A

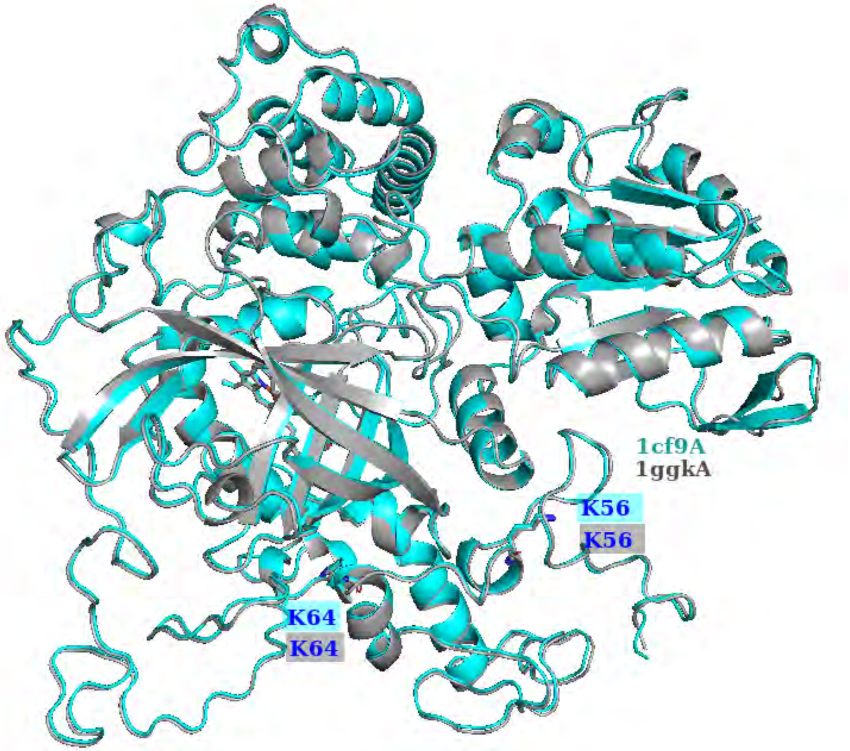


```
Align 1cf9.A.pdb 727 with 1ggk.A.pdb 727
Twists 0 ini-len 720 ini-rmsd 0.40 opt-equ 727 opt-rmsd 0.43 chain-rmsd 0.40 Score 2157.17 align-len 727 gaps 0 (0.00%)
P-value 0.00e+00 Afp-num 166976 Identity 99.72% Similarity 99.86%
Block  0 afp 90 score 2157.17 rmsd  0.40 gap 0 (0.00%)

                    .    :    .    :    .    :    .    :    .    :    .    :    .    :
Chain 1:   27 DSLAPEDGSHRPAAEPTPPGAQPTAPGSLKAPDTRNEKLNSLEDVRKGSENYALTTNQGVRIADDQNSLR
              1111111111111111111111111111111111111111111111111111111111111111111111
Chain 2:   27 DSLAPEDGSHRPAAEPTPPGAQPTAPGSLKAPDTRNEKLNSLEDVRKGSENYALTTNQGVRIADDQNSLR

                    .    :    .    :    .    :    .    :    .    :    .    :    .    :
Chain 1:   97 AGSRGPTLLEDFILREKITHFDHERIPERIVHARGSAAHGYFQPYKSLSDITKADFLSDPNKITPVFVRF
              1111111111111111111111111111111111111111111111111111111111111111111111
Chain 2:   97 AGSRGPTLLEDFILREKITHFDHERIPERIVHARGSAAHGYFQPYKSLSDITKADFLSDPNKITPVFVRF

                    .    :    .    :    .    :    .    :    .    :    .    :    .    :
Chain 1:  167 STCQGGAGSADTVRDIRGFATKFYTEEGIFDLVGNNTPIFFIQDAHKFPDFVHAVKPEPHWAIPQGQSAH
              1111111111111111111111111111111111111111111111111111111111111111111111
Chain 2:  167 STVQGGAGSADTVRDIRGFATKFYTEEGIFDLVGHNTPIFFIQDAHKFPDFVHAVKPEPHWAIPQGQSAH

                    .    :    .    :    .    :    .    :    .    :    .    :    .    :
Chain 1:  237 DTFWDYVSLQPETLHNVMWAMSDRGIPRSYRTMEGFGIHTFRLINAEGKATFVRFHWKPLAGKASLVWDE
              1111111111111111111111111111111111111111111111111111111111111111111111
Chain 2:  237 DTFWDYVSLQPETLHNVMWAMSDRGIPRSYRTMEGFGIHTFRLINAEGKATFVRFHWKPLAGKASLVWDE

                    .    :    .    :    .    :    .    :    .    :    .    :    .    :
Chain 1:  307 AQKLTGRDPDFHRRELWEAIEAGDFPEYELGFQLIPEEDEFKFDFDLLDPTKLIPEELVPVQRVGKMVLN
              1111111111111111111111111111111111111111111111111111111111111111111111
Chain 2:  307 AQKLTGRDPDFHRRELWEAIEAGDFPEYELGFQLIPEEDEFKFDFDLLDPTKLIPEELVPVQRVGKMVLN

                    .    :    .    :    .    :    .    :    .    :    .    :    .    :
Chain 1:  377 RNPDNFFAENEQAAFHPGHIVPGLDFTNDPLLQGRLFSYTDTQISRLGGPNFHEIPINRPTCPYHNFQRD
              1111111111111111111111111111111111111111111111111111111111111111111111
Chain 2:  377 RNPDNFFAENEQAAFHPGHIVPGLDFTNDPLLQGRLFSYTDTQISRLGGPNFHEIPINRPTCPYHNFQRD

                    .    :    .    :    .    :    .    :    .    :    .    :    .    :
Chain 1:  447 GMHRMGIDTNPANYEPNSINDNWPRETPPGPKRGGFESYQERVEGNKVRERSPSFGEYYSHPRLFWLSQT
              1111111111111111111111111111111111111111111111111111111111111111111111
Chain 2:  447 GMHRMGIDTNPANYEPNSINDNWPRETPPGPKRGGFESYQERVEGNKVRERSPSFGEYYSHPRLFWLSQT

                    .    :    .    :    .    :    .    :    .    :    .    :    .    :
Chain 1:  517 PFEQRHIVDGFSFELSKVVRPYIRERVVDQLAHIDLTLAQAVAKNLGIELTDDQLNITPPPDVNGLKKDP
              1111111111111111111111111111111111111111111111111111111111111111111111
Chain 2:  517 PFEQRHIVDGFSFELSKVVRPYIRERVVDQLAHIDLTLAQAVAKNLGIELTDDQLNITPPPDVNGLKKDP

                    .    :    .    :    .    :    .    :    .    :    .    :    .    :
Chain 1:  587 SLSLYAIPDGDVKGRVVAILLNDEVRSADLLAILKALKAKGVHAKLLYSRMGEVTADDGTVLPIAATFAG
              1111111111111111111111111111111111111111111111111111111111111111111111
Chain 2:  587 SLSLYAIPDGDVKGRVVAILLNDEVRSADLLAILKALKAKGVHAKLLYSRMGEVTADDGTVLPIAATFAG

                    .    :    .    :    .    :    .    :    .    :    .    :    .    :
Chain 1:  657 APSLTVDAVIVPCGNIADIADNGDANYYLMEAYKHLKPIALAGDARKFKATIKIADQGEEGIVEADSADG
              1111111111111111111111111111111111111111111111111111111111111111111111
Chain 2:  657 APSLTVDAVIVPCGNIADIADNGDANYYLMEAYKHLKPIALAGDARKFKATIKIADQGEEGIVEADSADG

                    .    :    .    :    .
Chain 1:  727 SFMDELLTLMAAHRVWSRIPKIDKIPA
              111111111111111111111111111
Chain 2:  727 SFMDELLTLMAAHRVWSRIPKIDKIPA
```

UniProt ID: P21179

PDB ID: 1IPH_A

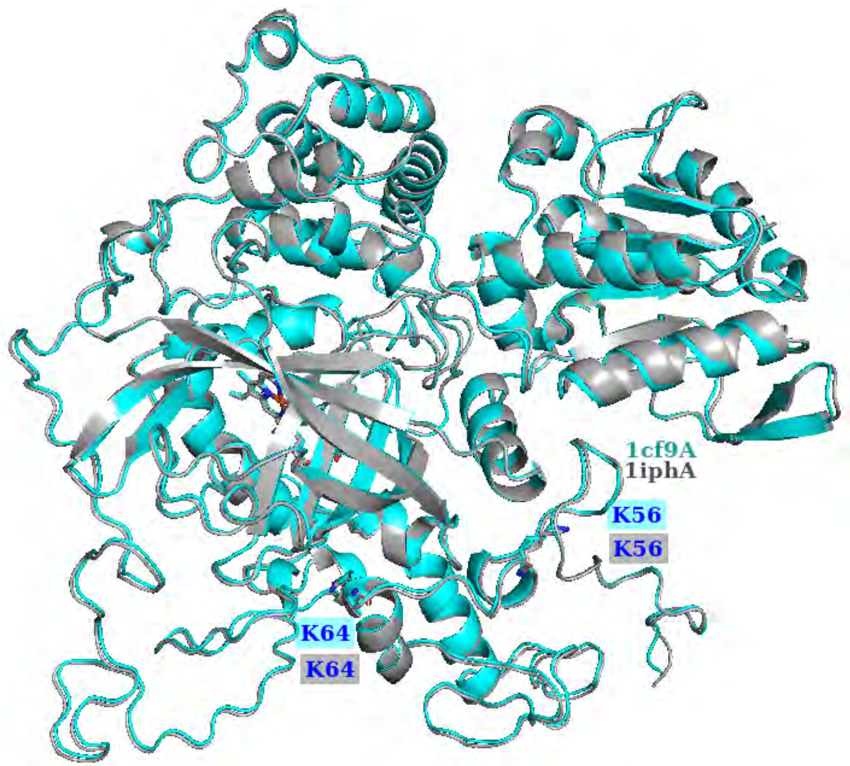

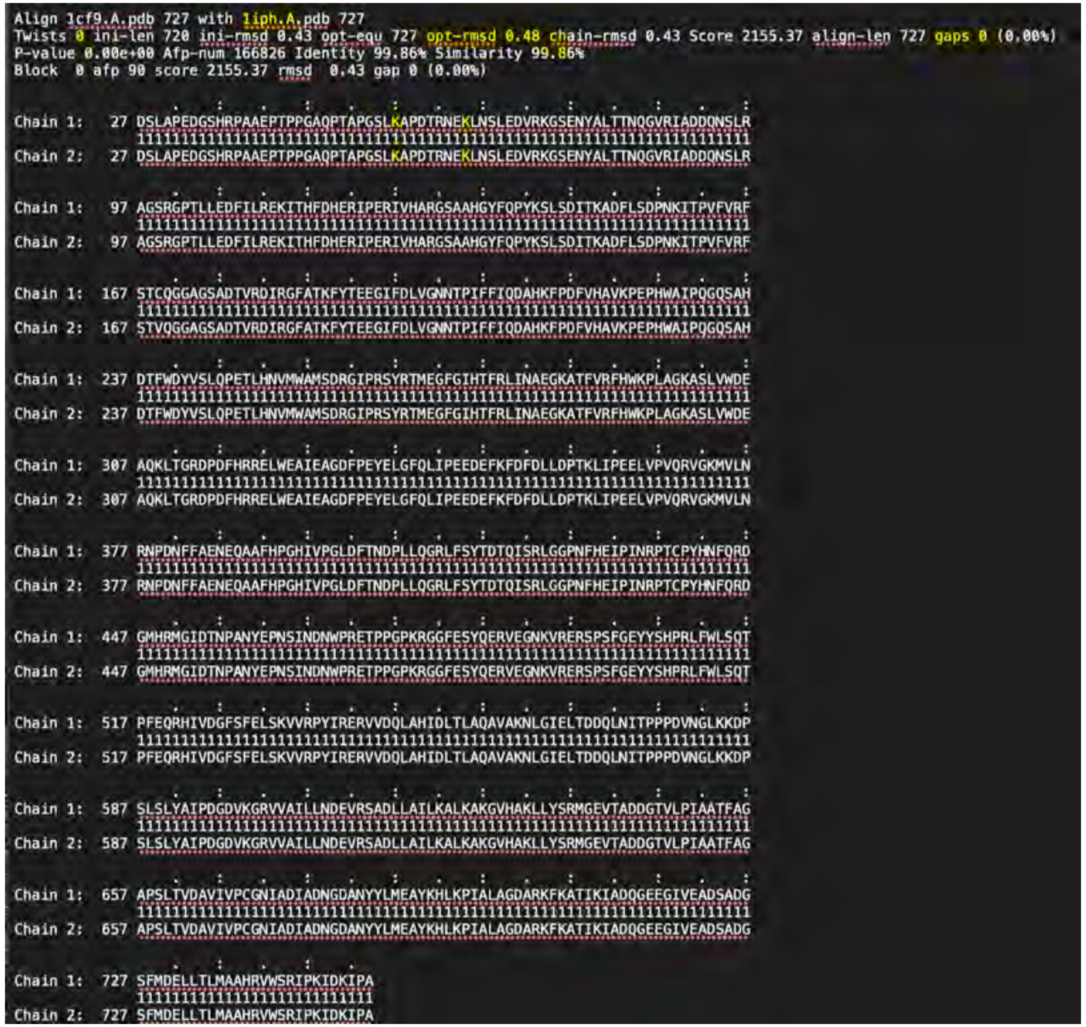

```
Align 1cf9.A.pdb 727 with 1iph.A.pdb 727
Twists 0 ini-len 720 ini-rmsd 0.43 opt-equ 727 opt-rmsd 0.48 chain-rmsd 0.43 Score 2155.37 align-len 727 gaps 0 (0.00%)
P-value 0.00e+00 Afp-num 166826 Identity 99.86% Similarity 99.86%
Block  0 afp 90 score 2155.37 rmsd  0.43 gap 0 (0.00%)

                    .    :    .    :    .    :    .    :    .    :    .    :    .    :
Chain 1:   27 DSLAPEDGSHRPAAEPTPPGAQPTAPGSLKAPDTRNEKLNSLEDVRKGSENYALTTNQGVRIADDQNSLR
              1111111111111111111111111111111111111111111111111111111111111111111111
Chain 2:   27 DSLAPEDGSHRPAAEPTPPGAQPTAPGSLKAPDTRNEKLNSLEDVRKGSENYALTTNQGVRIADDQNSLR

                    .    :    .    :    .    :    .    :    .    :    .    :    .    :
Chain 1:   97 AGSRGPTLLEDFILREKITHFDHERIPERIVHARGSAAHGYFQPYKSLSDITKADFLSDPNKITPVFVRF
              1111111111111111111111111111111111111111111111111111111111111111111111
Chain 2:   97 AGSRGPTLLEDFILREKITHFDHERIPERIVHARGSAAHGYFQPYKSLSDITKADFLSDPNKITPVFVRF

                    .    :    .    :    .    :    .    :    .    :    .    :    .    :
Chain 1:  167 STCQGGAGSADTVRDIRGFATKFYTEEGIFDLVGNNTPIFFIQDAHKFPDFVHAVKPEPHWAIPQGQSAH
              1111111111111111111111111111111111111111111111111111111111111111111111
Chain 2:  167 STVQGGAGSADTVRDIRGFATKFYTEEGIFDLVGNNTPIFFIQDAHKFPDFVHAVKPEPHWAIPQGQSAH

                    .    :    .    :    .    :    .    :    .    :    .    :    .    :
Chain 1:  237 DTFWDYVSLQPETLHNVMWAMSDRGIPRSYRTMEGFGIHTFRLINAEGKATFVRFHWKPLAGKASLVWDE
              1111111111111111111111111111111111111111111111111111111111111111111111
Chain 2:  237 DTFWDYVSLQPETLHNVMWAMSDRGIPRSYRTMEGFGIHTFRLINAEGKATFVRFHWKPLAGKASLVWDE

                    .    :    .    :    .    :    .    :    .    :    .    :    .    :
Chain 1:  307 AQKLTGRDPDFHRRELWEAIEAGDFPEYELGFQLIPEEDEFKFDFDLLDPTKLIPEELVPVQRVGKMVLN
              1111111111111111111111111111111111111111111111111111111111111111111111
Chain 2:  307 AQKLTGRDPDFHRRELWEAIEAGDFPEYELGFQLIPEEDEFKFDFDLLDPTKLIPEELVPVQRVGKMVLN

                    .    :    .    :    .    :    .    :    .    :    .    :    .    :
Chain 1:  377 RNPDNFFAENEQAAFHPGHIVPGLDFTNDPLLQGRLFSYTDTQISRLGGPNFHEIPINRPTCPYHNFQRD
              1111111111111111111111111111111111111111111111111111111111111111111111
Chain 2:  377 RNPDNFFAENEQAAFHPGHIVPGLDFTNDPLLQGRLFSYTDTQISRLGGPNFHEIPINRPTCPYHNFQRD

                    .    :    .    :    .    :    .    :    .    :    .    :    .    :
Chain 1:  447 GMHRMGIDTNPANYEPNSINDNWPRETPPGPKRGGFESYQERVEGNKVRERSPSFGEYYSHPRLFWLSQT
              1111111111111111111111111111111111111111111111111111111111111111111111
Chain 2:  447 GMHRMGIDTNPANYEPNSINDNWPRETPPGPKRGGFESYQERVEGNKVRERSPSFGEYYSHPRLFWLSQT

                    .    :    .    :    .    :    .    :    .    :    .    :    .    :
Chain 1:  517 PFEQRHIVDGFSFELSKVVRPYIRERVVDQLAHIDLTLAQAVAKNLGIELTDDQLNITPPPDVNGLKKDP
              1111111111111111111111111111111111111111111111111111111111111111111111
Chain 2:  517 PFEQRHIVDGFSFELSKVVRPYIRERVVDQLAHIDLTLAQAVAKNLGIELTDDQLNITPPPDVNGLKKDP

                    .    :    .    :    .    :    .    :    .    :    .    :    .    :
Chain 1:  587 SLSLYAIPDGDVKGRVVAILLNDEVRSADLLAILKALKAKGVHAKLLYSRMGEVTADDGTVLPIAATFAG
              1111111111111111111111111111111111111111111111111111111111111111111111
Chain 2:  587 SLSLYAIPDGDVKGRVVAILLNDEVRSADLLAILKALKAKGVHAKLLYSRMGEVTADDGTVLPIAATFAG

                    .    :    .    :    .    :    .    :    .    :    .    :    .    :
Chain 1:  657 APSLTVDAVIVPCGNIADIADNGDANYYLMEAYKHLKPIALAGDARKFKATIKIADQGEEGIVEADSADG
              1111111111111111111111111111111111111111111111111111111111111111111111
Chain 2:  657 APSLTVDAVIVPCGNIADIADNGDANYYLMEAYKHLKPIALAGDARKFKATIKIADQGEEGIVEADSADG

                    .    :    .    :    .
Chain 1:  727 SFMDELLTLMAAHRVWSRIPKIDKIPA
              111111111111111111111111111
Chain 2:  727 SFMDELLTLMAAHRVWSRIPKIDKIPA
```

UniProt ID: P21179
PDB ID: 1P7Y_A

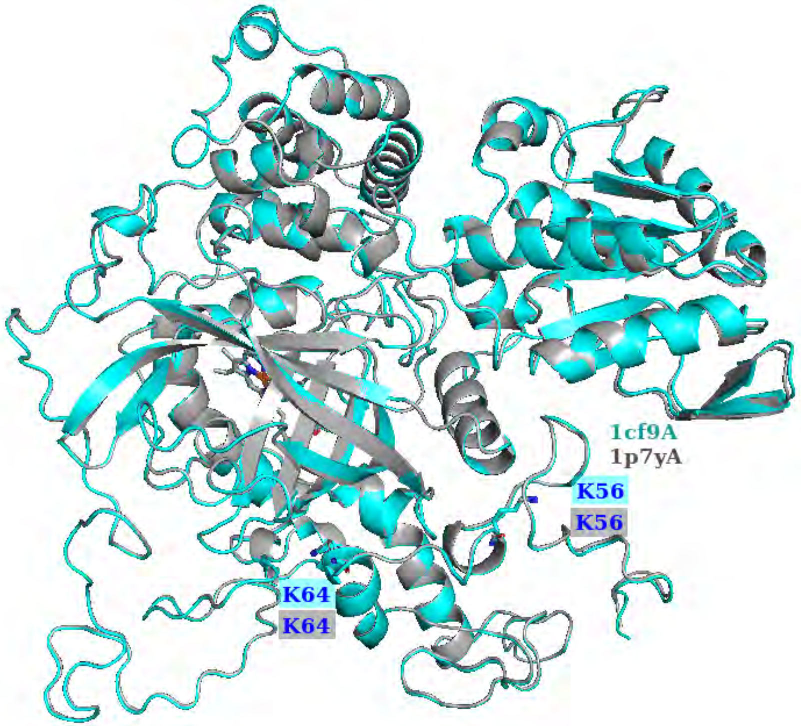

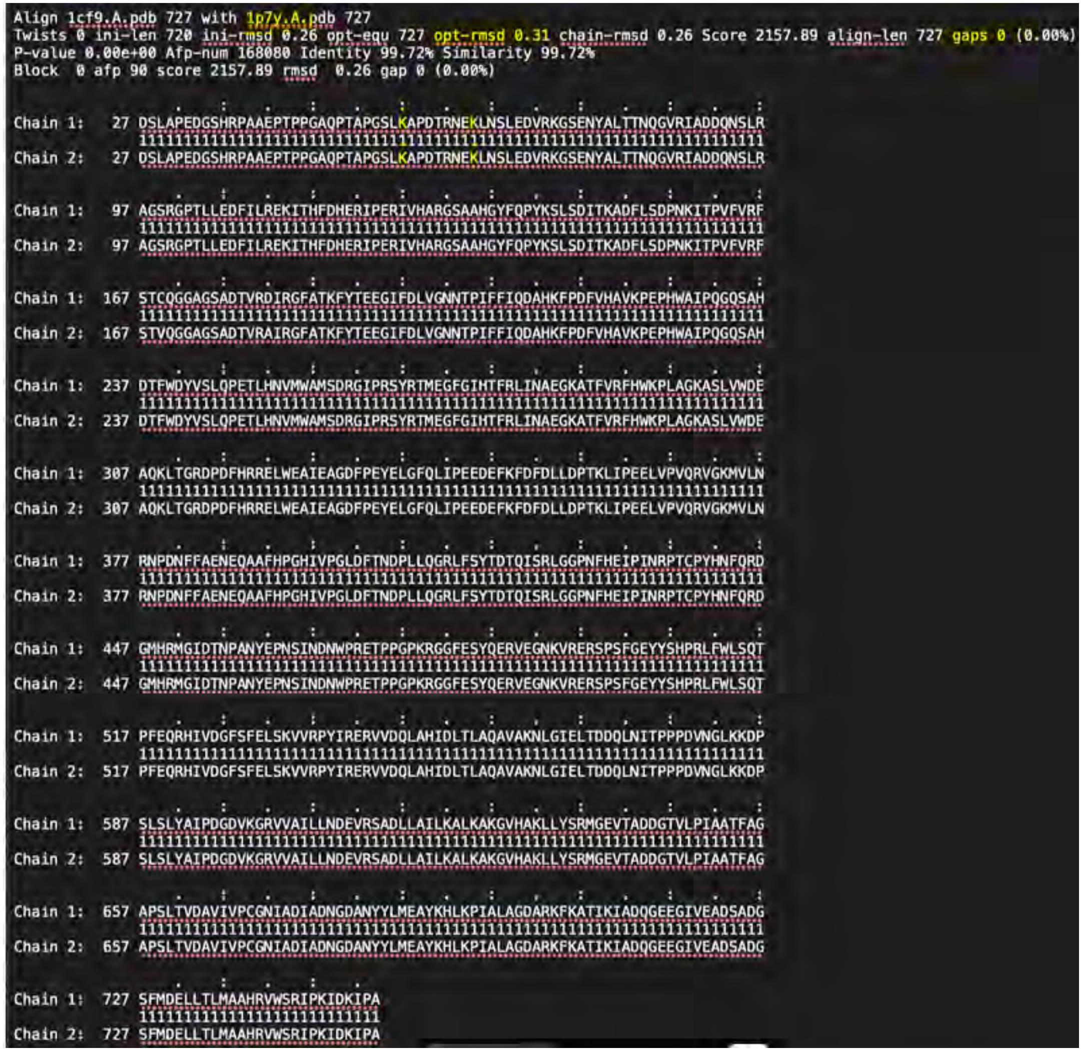

```
Align 1cf9.A.pdb 727 with 1p7y.A.pdb 727
Twists 0 ini-len 720 ini-rmsd 0.26 opt-equ 727 opt-rmsd 0.31 chain-rmsd 0.26 Score 2157.89 align-len 727 gaps 0 (0.00%)
P-value 0.00e+00 Afp-num 168080 Identity 99.72% Similarity 99.72%
Block  0 afp 90 score 2157.89 rmsd  0.26 gap 0 (0.00%)

Chain 1:   27 DSLAPEDGSHRPAAEPTPPGAQPTAPGSLKAPDTRNEKLNSLEDVRKGSENYALTTNQGVRIADDQNSLR
              1111111111111111111111111111111111111111111111111111111111111111111111
Chain 2:   27 DSLAPEDGSHRPAAEPTPPGAQPTAPGSLKAPDTRNEKLNSLEDVRKGSENYALTTNQGVRIADDQNSLR

Chain 1:   97 AGSRGPTLLEDFILREKITHFDHERIPERIVHARGSAAHGYFQPYKSLSDITKADFLSDPNKITPVFVRF
              1111111111111111111111111111111111111111111111111111111111111111111111
Chain 2:   97 AGSRGPTLLEDFILREKITHFDHERIPERIVHARGSAAHGYFQPYKSLSDITKADFLSDPNKITPVFVRF

Chain 1:  167 STCQGGAGSADTVRDIRGFATKFYTEEGIFDLVGNNTPIFFIQDAHKFPDFVHAVKPEPHWAIPQGQSAH
              1111111111111111111111111111111111111111111111111111111111111111111111
Chain 2:  167 STVQGGAGSADTVRAIRGFATKFYTEEGIFDLVGNNTPIFFIQDAHKFPDFVHAVKPEPHWAIPQGQSAH

Chain 1:  237 DTFWDYVSLQPETLHNVMWAMSDRGIPRSYRTMEGFGIHTFRLINAEGKATFVRFHWKPLAGKASLVWDE
              1111111111111111111111111111111111111111111111111111111111111111111111
Chain 2:  237 DTFWDYVSLQPETLHNVMWAMSDRGIPRSYRTMEGFGIHTFRLINAEGKATFVRFHWKPLAGKASLVWDE

Chain 1:  307 AQKLTGRDPDFHRRELWEAIEAGDFPEYELGFQLIPEEDEFKFDFDLLDPTKLIPEELVPVQRVGKMVLN
              1111111111111111111111111111111111111111111111111111111111111111111111
Chain 2:  307 AQKLTGRDPDFHRRELWEAIEAGDFPEYELGFQLIPEEDEFKFDFDLLDPTKLIPEELVPVQRVGKMVLN

Chain 1:  377 RNPDNFFAENEQAAFHPGHIVPGLDFTNDPLLQGRLFSYTDTQISRLGGPNFHEIPINRPTCPYHNFQRD
              1111111111111111111111111111111111111111111111111111111111111111111111
Chain 2:  377 RNPDNFFAENEQAAFHPGHIVPGLDFTNDPLLQGRLFSYTDTQISRLGGPNFHEIPINRPTCPYHNFQRD

Chain 1:  447 GMHRMGIDTNPANYEPNSINDNWPRETPPGPKRGGFESYQERVEGNKVRERSPSFGEYYSHPRLFWLSQT
              1111111111111111111111111111111111111111111111111111111111111111111111
Chain 2:  447 GMHRMGIDTNPANYEPNSINDNWPRETPPGPKRGGFESYQERVEGNKVRERSPSFGEYYSHPRLFWLSQT

Chain 1:  517 PFEQRHIVDGFSFELSKVVRPYIRERVVDQLAHIDLTLAQAVAKNLGIELTDDQLNITPPPDVNGLKKDP
              1111111111111111111111111111111111111111111111111111111111111111111111
Chain 2:  517 PFEQRHIVDGFSFELSKVVRPYIRERVVDQLAHIDLTLAQAVAKNLGIELTDDQLNITPPPDVNGLKKDP

Chain 1:  587 SLSLYAIPDGDVKGRVVAILLNDEVRSADLLAILKALKAKGVHAKLLYSRMGEVTADDGTVLPIAATFAG
              1111111111111111111111111111111111111111111111111111111111111111111111
Chain 2:  587 SLSLYAIPDGDVKGRVVAILLNDEVRSADLLAILKALKAKGVHAKLLYSRMGEVTADDGTVLPIAATFAG

Chain 1:  657 APSLTVDAVIVPCGNIADIADNGDANYYLMEAYKHLKPIALAGDARKFKATIKIADQGEEGIVEADSADG
              1111111111111111111111111111111111111111111111111111111111111111111111
Chain 2:  657 APSLTVDAVIVPCGNIADIADNGDANYYLMEAYKHLKPIALAGDARKFKATIKIADQGEEGIVEADSADG

Chain 1:  727 SFMDELLTLMAAHRVWSRIPKIDKIPA
              111111111111111111111111111
Chain 2:  727 SFMDELLTLMAAHRVWSRIPKIDKIPA
```

UniProt ID: P21179
PDB ID: 1P7Z_A

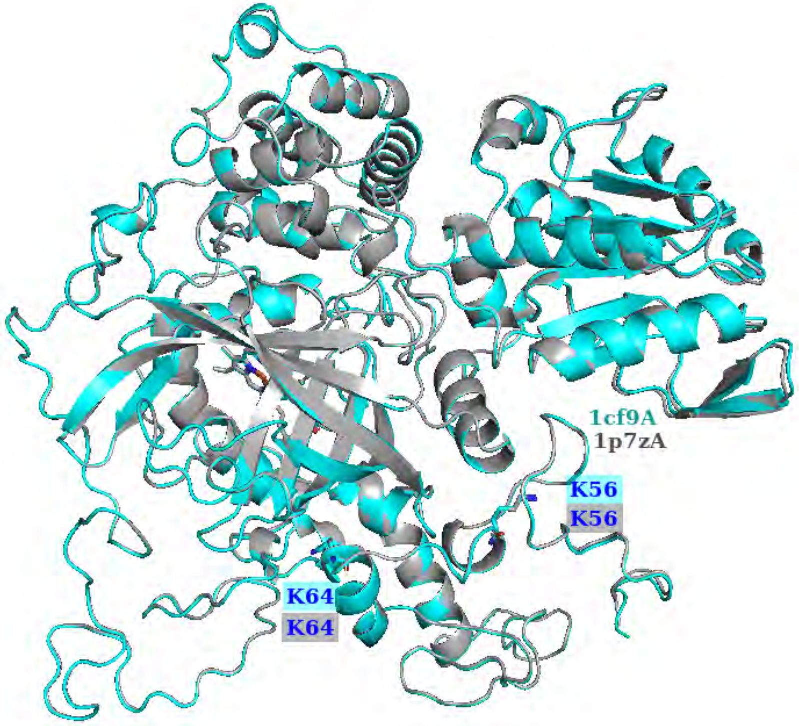

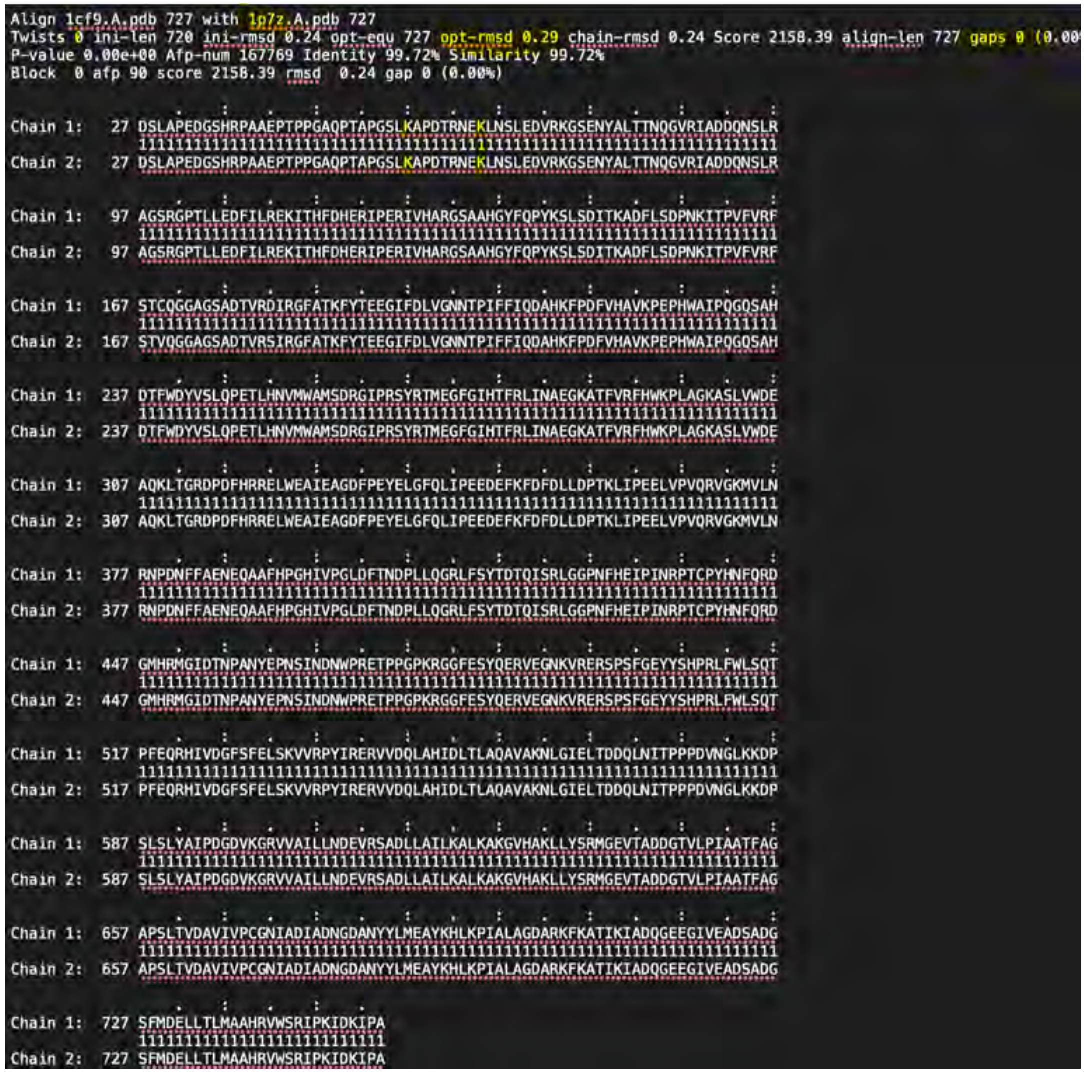

UniProt ID: P21179

PDB ID: 1P80_A

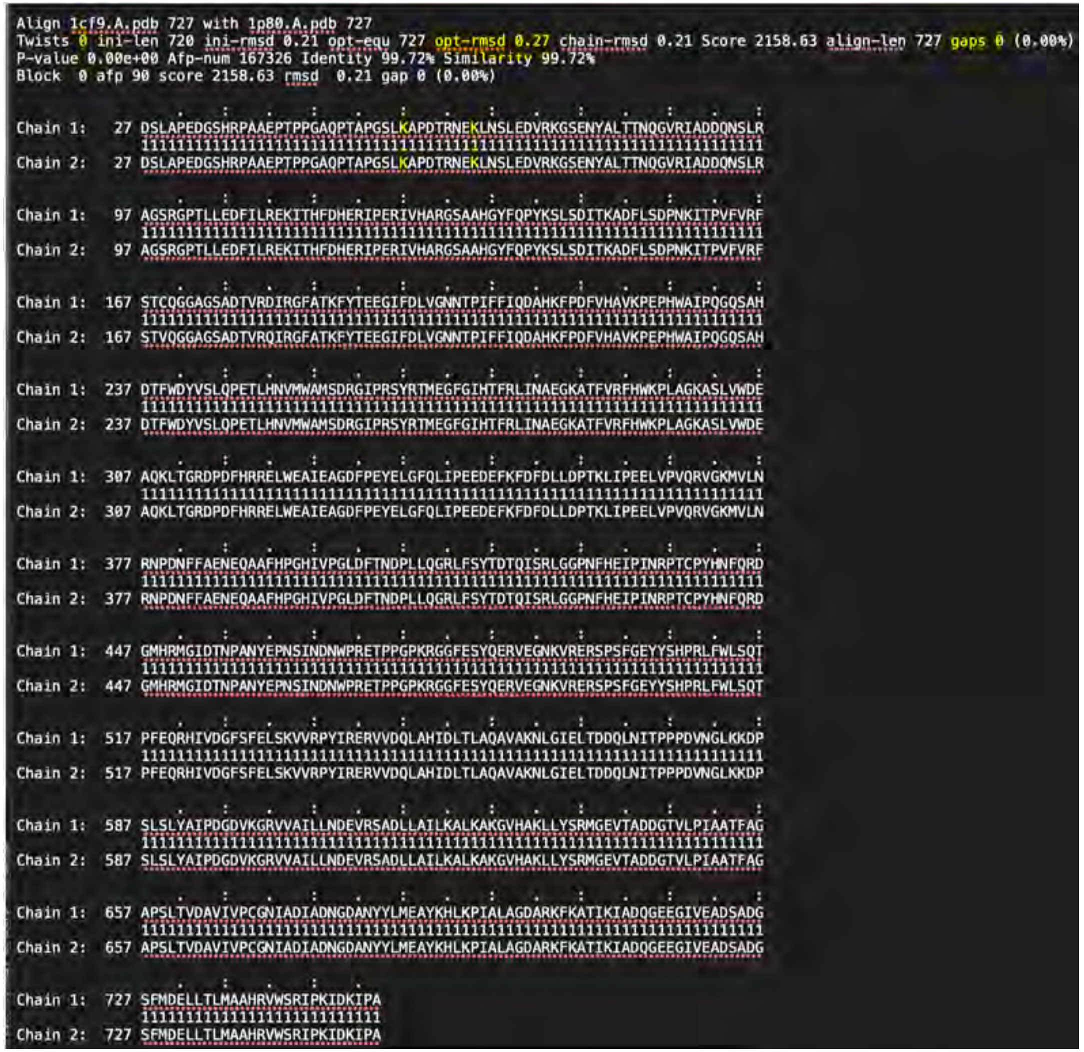

```
Align 1cf9.A.pdb 727 with 1p80.A.pdb 727
Twists 0 ini-len 720 ini-rmsd 0.21 opt-equ 727 opt-rmsd 0.27 chain-rmsd 0.21 Score 2158.63 align-len 727 gaps 0 (0.00%)
P-value 0.00e+00 Afp-num 167326 Identity 99.72% Similarity 99.72%
Block  0 afp 90 score 2158.63 rmsd  0.21 gap 0 (0.00%)

                 .    :    .    :    .    :    .    :    .    :    .    :    .    :
Chain 1:   27 DSLAPEDGSHRPAAEPTPPGAQPTAPGSLKAPDTRNEKLNSLEDVRKGSENYALTTNQGVRIADDQNSLR
              1111111111111111111111111111111111111111111111111111111111111111111111
Chain 2:   27 DSLAPEDGSHRPAAEPTPPGAQPTAPGSLKAPDTRNEKLNSLEDVRKGSENYALTTNQGVRIADDQNSLR

                 .    :    .    :    .    :    .    :    .    :    .    :    .    :
Chain 1:   97 AGSRGPTLLEDFILREKITHFDHERIPERIVHARGSAAHGYFQPYKSLSDITKADFLSDPNKITPVFVRF
              1111111111111111111111111111111111111111111111111111111111111111111111
Chain 2:   97 AGSRGPTLLEDFILREKITHFDHERIPERIVHARGSAAHGYFQPYKSLSDITKADFLSDPNKITPVFVRF

                 .    :    .    :    .    :    .    :    .    :    .    :    .    :
Chain 1:  167 STCQGGAGSADTVRDIRGFATKFYTEEGIFDLVGNNTPIFFIQDAHKFPDFVHAVKPEPHWAIPQGQSAH
              1111111111111111111111111111111111111111111111111111111111111111111111
Chain 2:  167 STVQGGAGSADTVRQIRGFATKFYTEEGIFDLVGNNTPIFFIQDAHKFPDFVHAVKPEPHWAIPQGQSAH

                 .    :    .    :    .    :    .    :    .    :    .    :    .    :
Chain 1:  237 DTFWDYVSLQPETLHNVMWAMSDRGIPRSYRTMEGFGIHTFRLINAEGKATFVRFHWKPLAGKASLVWDE
              1111111111111111111111111111111111111111111111111111111111111111111111
Chain 2:  237 DTFWDYVSLQPETLHNVMWAMSDRGIPRSYRTMEGFGIHTFRLINAEGKATFVRFHWKPLAGKASLVWDE

                 .    :    .    :    .    :    .    :    .    :    .    :    .    :
Chain 1:  307 AQKLTGRDPDFHRRELWEAIEAGDFPEYELGFQLIPEEDEFKFDFDLLDPTKLIPEELVPVQRVGKMVLN
              1111111111111111111111111111111111111111111111111111111111111111111111
Chain 2:  307 AQKLTGRDPDFHRRELWEAIEAGDFPEYELGFQLIPEEDEFKFDFDLLDPTKLIPEELVPVQRVGKMVLN

                 .    :    .    :    .    :    .    :    .    :    .    :    .    :
Chain 1:  377 RNPDNFFAENEQAAFHPGHIVPGLDFTNDPLLQGRLFSYTDTQISRLGGPNFHEIPINRPTCPYHNFQRD
              1111111111111111111111111111111111111111111111111111111111111111111111
Chain 2:  377 RNPDNFFAENEQAAFHPGHIVPGLDFTNDPLLQGRLFSYTDTQISRLGGPNFHEIPINRPTCPYHNFQRD

                 .    :    .    :    .    :    .    :    .    :    .    :    .    :
Chain 1:  447 GMHRMGIDTNPANYEPNSINDNWPRETPPGPKRGGFESYQERVEGNKVRERSPSFGEYYSHPRLFWLSQT
              1111111111111111111111111111111111111111111111111111111111111111111111
Chain 2:  447 GMHRMGIDTNPANYEPNSINDNWPRETPPGPKRGGFESYQERVEGNKVRERSPSFGEYYSHPRLFWLSQT

                 .    :    .    :    .    :    .    :    .    :    .    :    .    :
Chain 1:  517 PFEQRHIVDGFSFELSKVVRPYIRERVVDQLAHIDLTLAQAVAKNLGIELTDDQLNITPPPDVNGLKKDP
              1111111111111111111111111111111111111111111111111111111111111111111111
Chain 2:  517 PFEQRHIVDGFSFELSKVVRPYIRERVVDQLAHIDLTLAQAVAKNLGIELTDDQLNITPPPDVNGLKKDP

                 .    :    .    :    .    :    .    :    .    :    .    :    .    :
Chain 1:  587 SLSLYAIPDGDVKGRVVAILLNDEVRSADLLAILKALKAKGVHAKLLYSRMGEVTADDGTVLPIAATFAG
              1111111111111111111111111111111111111111111111111111111111111111111111
Chain 2:  587 SLSLYAIPDGDVKGRVVAILLNDEVRSADLLAILKALKAKGVHAKLLYSRMGEVTADDGTVLPIAATFAG

                 .    :    .    :    .    :    .    :    .    :    .    :    .    :
Chain 1:  657 APSLTVDAVIVPCGNIADIADNGDANYYLMEAYKHLKPIALAGDARKFKATIKIADQGEEGIVEADSADG
              1111111111111111111111111111111111111111111111111111111111111111111111
Chain 2:  657 APSLTVDAVIVPCGNIADIADNGDANYYLMEAYKHLKPIALAGDARKFKATIKIADQGEEGIVEADSADG

                 .    :    .    :    .
Chain 1:  727 SFMDELLTLMAAHRVWSRIPKIDKIPA
              111111111111111111111111111
Chain 2:  727 SFMDELLTLMAAHRVWSRIPKIDKIPA
```

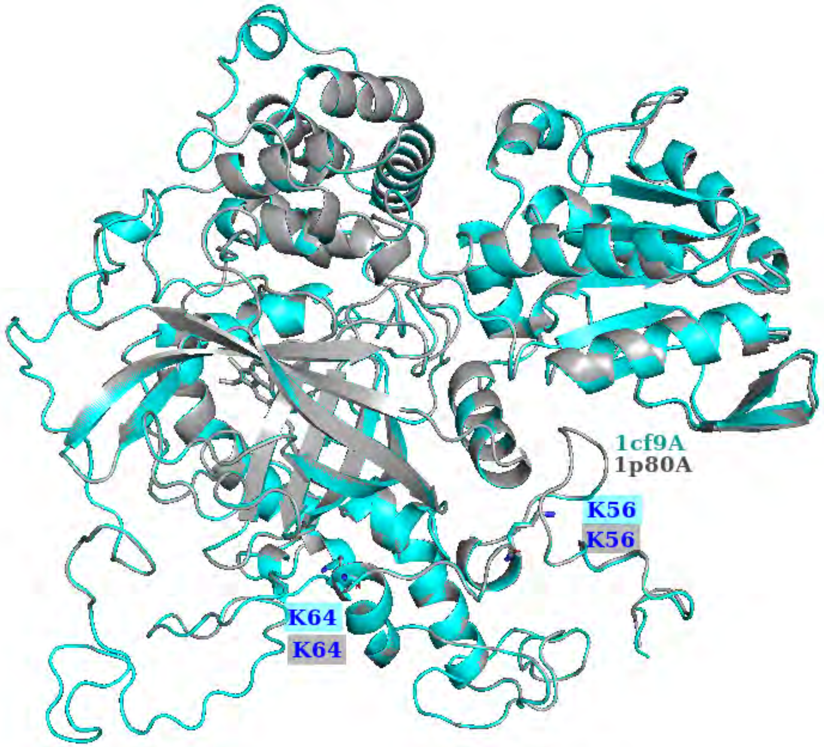

UniProt ID: P21179
PDB ID: 1P81_B

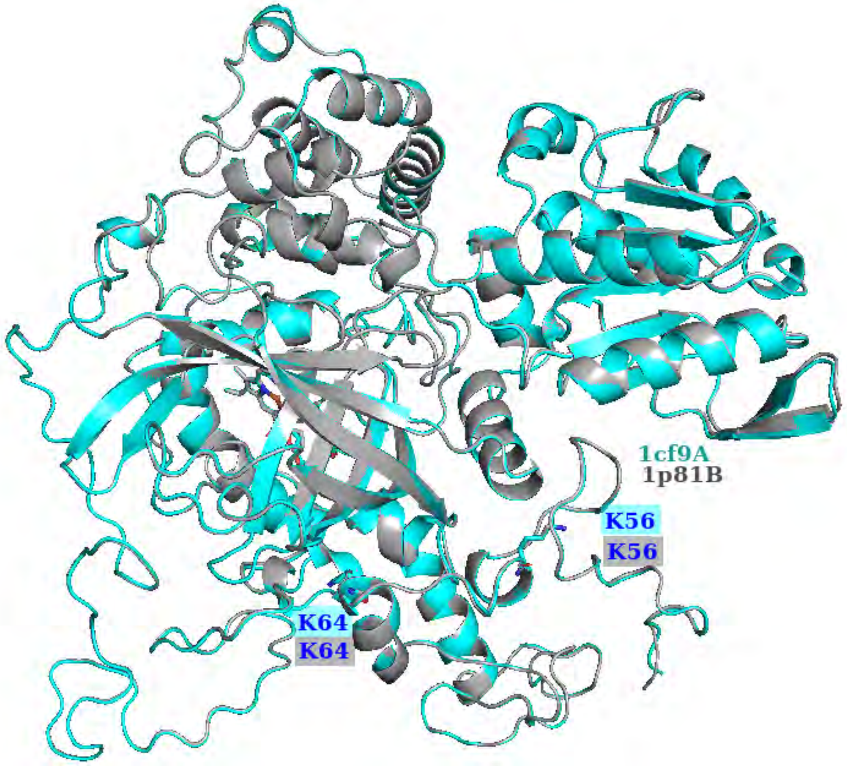

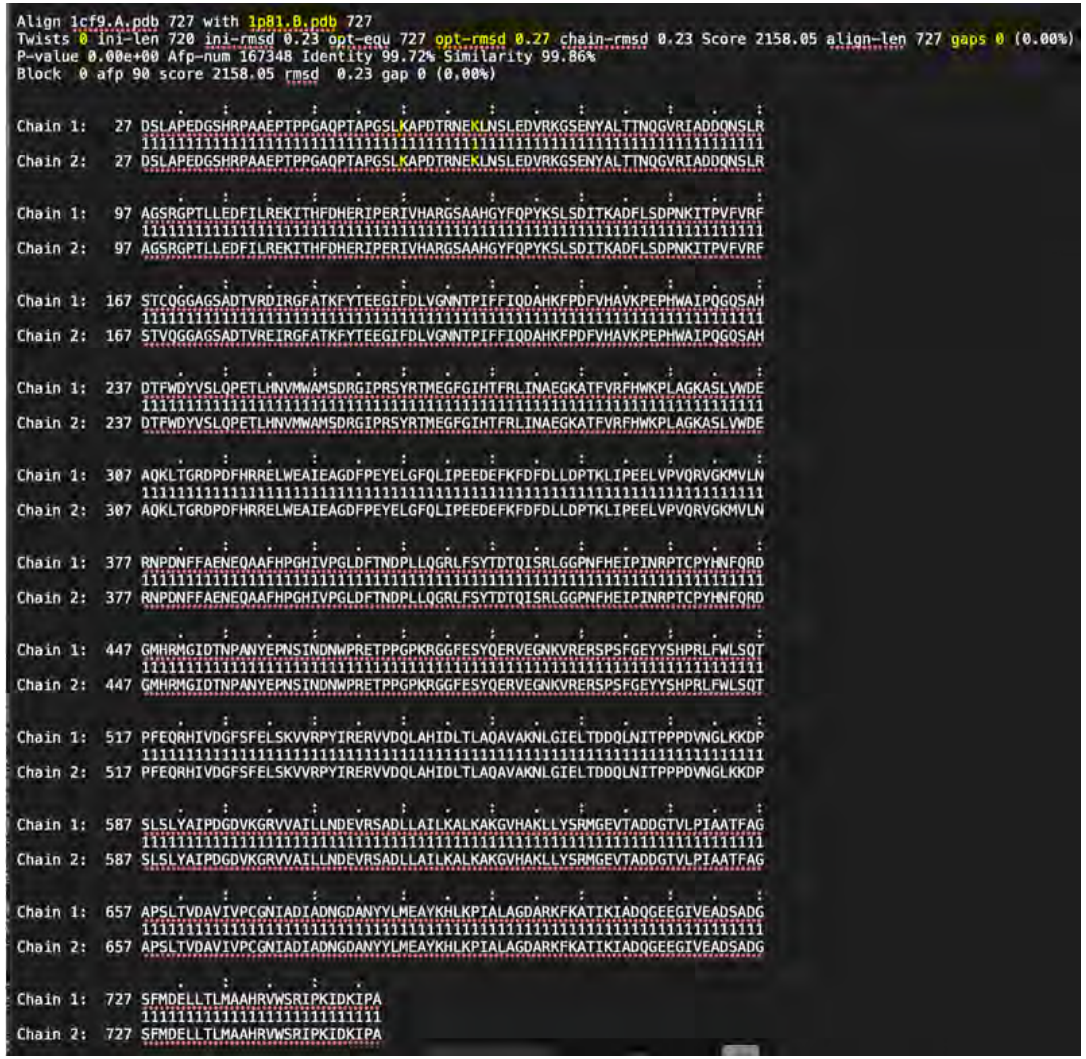

```
Align 1cf9.A.pdb 727 with 1p81.B.pdb 727
Twists 0 ini-len 720 ini-rmsd 0.23 opt-equ 727 opt-rmsd 0.27 chain-rmsd 0.23 Score 2158.05 align-len 727 gaps 0 (0.00%)
P-value 0.00e+00 Afp-num 167348 Identity 99.72% Similarity 99.86%
Block  0 afp 90 score 2158.05 rmsd  0.23 gap 0 (0.00%)

Chain 1:   27 DSLAPEDGSHRPAAEPTPPGAQPTAPGSLKAPDTRNEKLNSLEDVRKGSENYALTTNQGVRIADDQNSLR
              1111111111111111111111111111111111111111111111111111111111111111111111
Chain 2:   27 DSLAPEDGSHRPAAEPTPPGAQPTAPGSLKAPDTRNEKLNSLEDVRKGSENYALTTNQGVRIADDQNSLR

Chain 1:   97 AGSRGPTLLEDFILREKITHFDHERIPERIVHARGSAAHGYFQPYKSLSDITKADFLSDPNKITPVFVRF
              1111111111111111111111111111111111111111111111111111111111111111111111
Chain 2:   97 AGSRGPTLLEDFILREKITHFDHERIPERIVHARGSAAHGYFQPYKSLSDITKADFLSDPNKITPVFVRF

Chain 1:  167 STCQGGAGSADTVRDIRGFATKFYTEEGIFDLVGNNTPIFFIQDAHKFPDFVHAVKPEPHWAIPQGQSAH
              1111111111111111111111111111111111111111111111111111111111111111111111
Chain 2:  167 STVQGGAGSADTVREIRGFATKFYTEEGIFDLVGNNTPIFFIQDAHKFPDFVHAVKPEPHWAIPQGQSAH

Chain 1:  237 DTFWDYVSLQPETLHNVMWAMSDRGIPRSYRTMEGFGIHTFRLINAEGKATFVRFHWKPLAGKASLVWDE
              1111111111111111111111111111111111111111111111111111111111111111111111
Chain 2:  237 DTFWDYVSLQPETLHNVMWAMSDRGIPRSYRTMEGFGIHTFRLINAEGKATFVRFHWKPLAGKASLVWDE

Chain 1:  307 AQKLTGRDPDFHRRELWEAIEAGDFPEYELGFQLIPEEDEFKFDFDLLDPTKLIPEELVPVQRVGKMVLN
              1111111111111111111111111111111111111111111111111111111111111111111111
Chain 2:  307 AQKLTGRDPDFHRRELWEAIEAGDFPEYELGFQLIPEEDEFKFDFDLLDPTKLIPEELVPVQRVGKMVLN

Chain 1:  377 RNPDNFFAENEQAAFHPGHIVPGLDFTNDPLLQGRLFSYTDTQISRLGGPNFHEIPINRPTCPYHNFQRD
              1111111111111111111111111111111111111111111111111111111111111111111111
Chain 2:  377 RNPDNFFAENEQAAFHPGHIVPGLDFTNDPLLQGRLFSYTDTQISRLGGPNFHEIPINRPTCPYHNFQRD

Chain 1:  447 GMHRMGIDTNPANYEPNSINDNWPRETPPGPKRGGFESYQERVEGNKVRERSPSFGEYYSHPRLFWLSQT
              1111111111111111111111111111111111111111111111111111111111111111111111
Chain 2:  447 GMHRMGIDTNPANYEPNSINDNWPRETPPGPKRGGFESYQERVEGNKVRERSPSFGEYYSHPRLFWLSQT

Chain 1:  517 PFEQRHIVDGFSFELSKVVRPYIRERVVDQLAHIDLTLAQAVAKNLGIELTDDQLNITPPPDVNGLKKDP
              1111111111111111111111111111111111111111111111111111111111111111111111
Chain 2:  517 PFEQRHIVDGFSFELSKVVRPYIRERVVDQLAHIDLTLAQAVAKNLGIELTDDQLNITPPPDVNGLKKDP

Chain 1:  587 SLSLYAIPDGDVKGRVVAILLNDEVRSADLLAILKALKAKGVHAKLLYSRMGEVTADDGTVLPIAATFAG
              1111111111111111111111111111111111111111111111111111111111111111111111
Chain 2:  587 SLSLYAIPDGDVKGRVVAILLNDEVRSADLLAILKALKAKGVHAKLLYSRMGEVTADDGTVLPIAATFAG

Chain 1:  657 APSLTVDAVIVPCGNIADIADNGDANYYLMEAYKHLKPIALAGDARKFKATIKIADQGEEGIVEADSADG
              1111111111111111111111111111111111111111111111111111111111111111111111
Chain 2:  657 APSLTVDAVIVPCGNIADIADNGDANYYLMEAYKHLKPIALAGDARKFKATIKIADQGEEGIVEADSADG

Chain 1:  727 SFMDELLTLMAAHRVWSRIPKIDKIPA
              111111111111111111111111111
Chain 2:  727 SFMDELLTLMAAHRVWSRIPKIDKIPA
```

UniProt ID: P21179

PDB ID: 1QF7_A

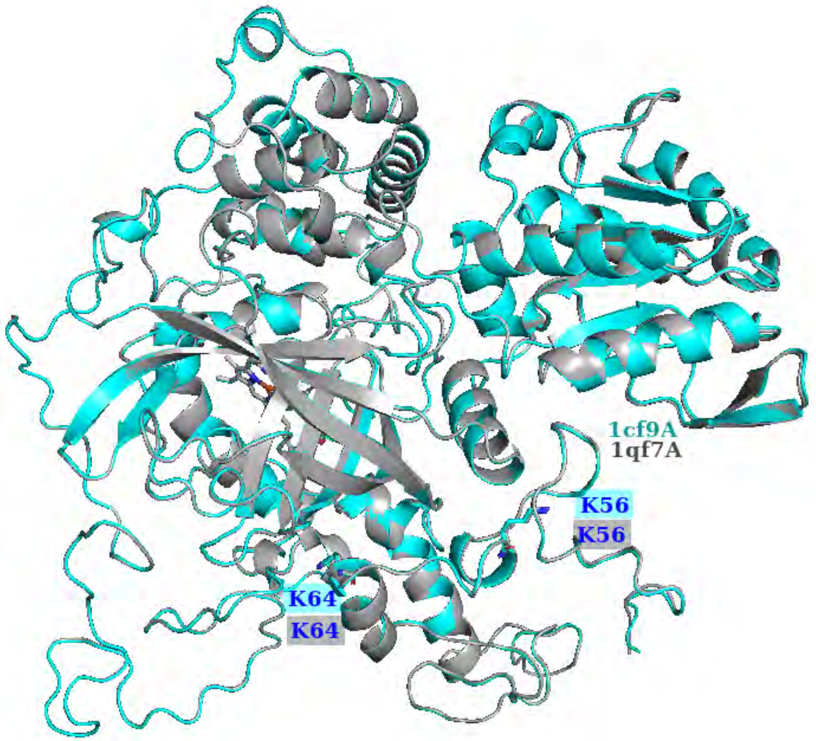

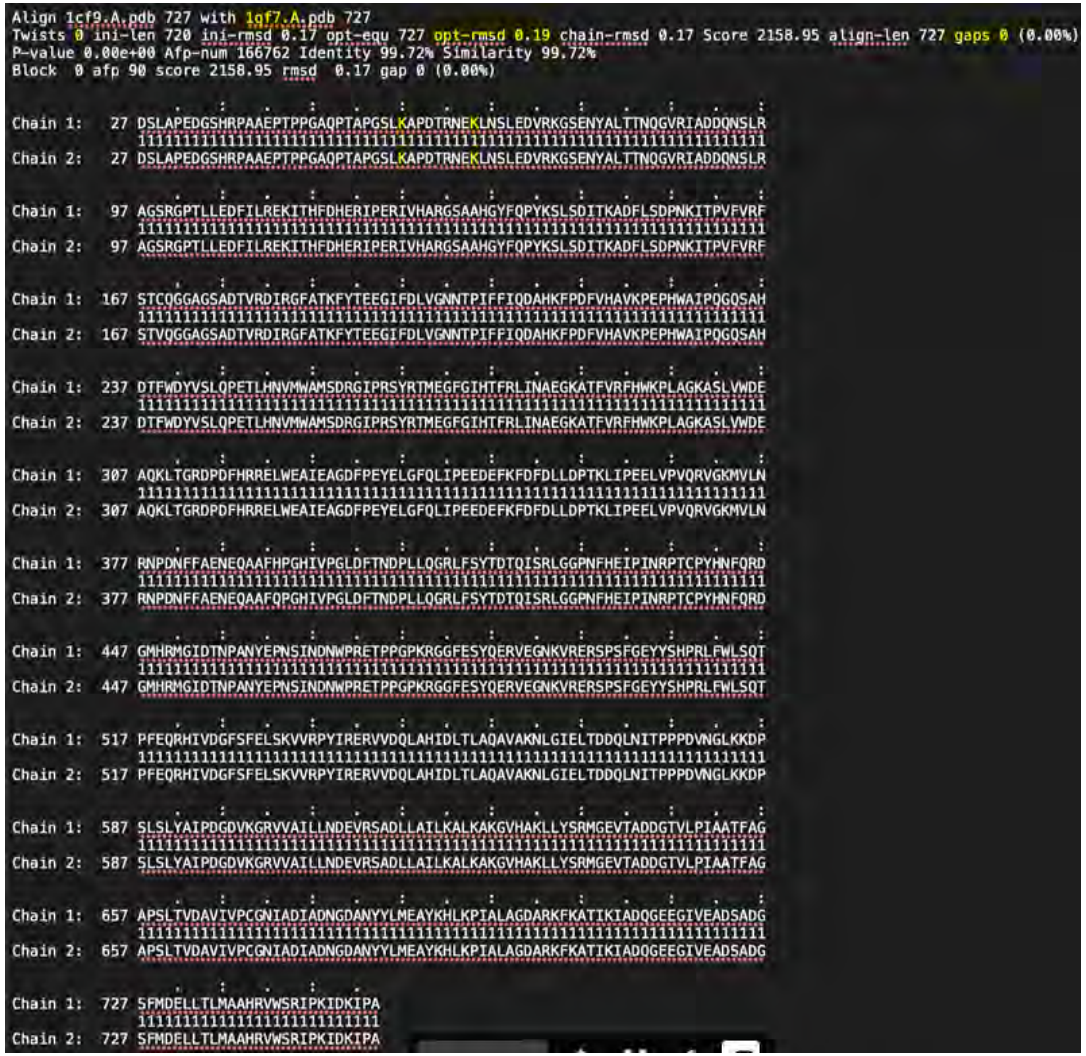

UniProt ID: P21179

PDB ID: 1QWS_A

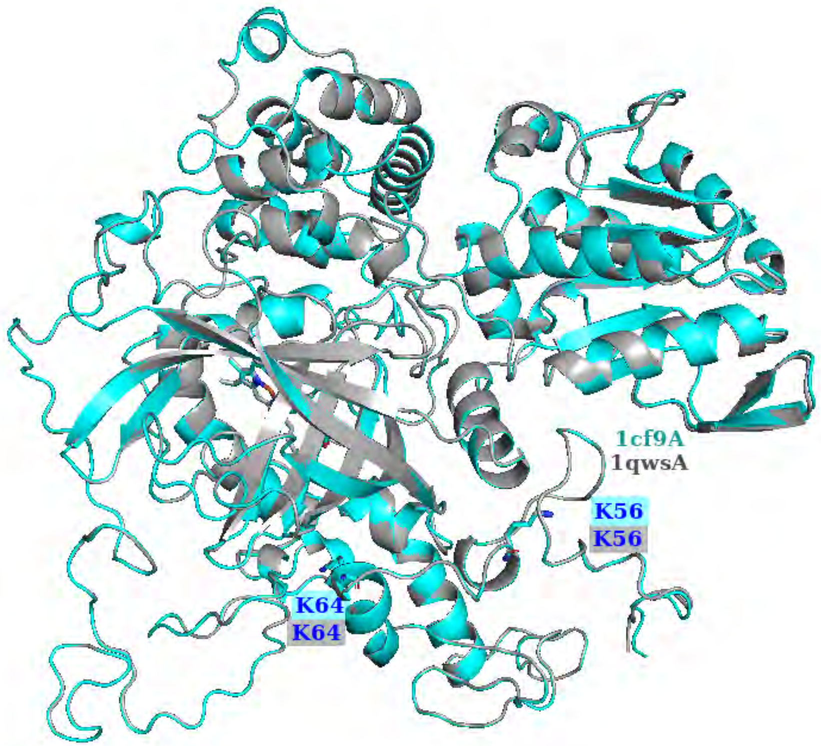

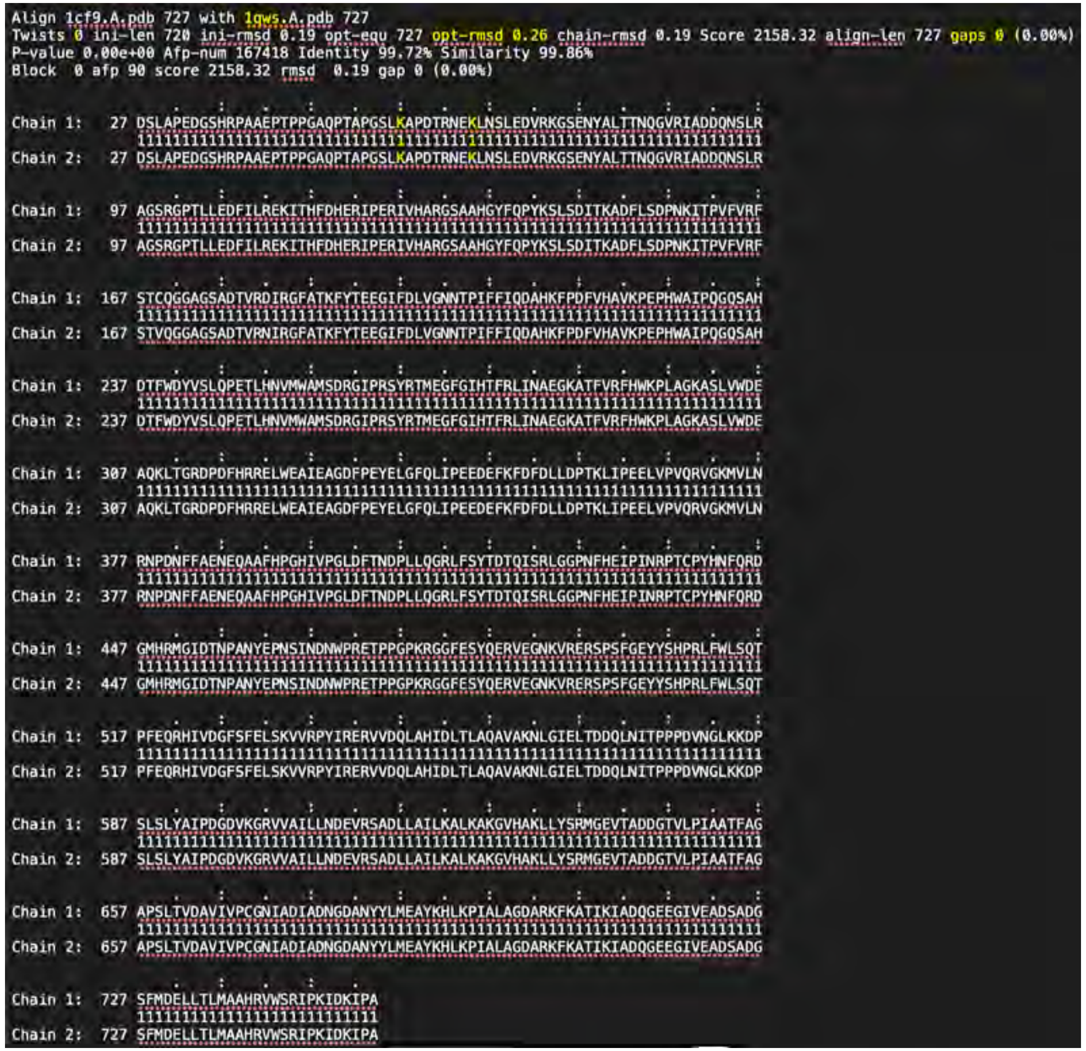

```
Align 1cf9.A.pdb 727 with 1qws.A.pdb 727
Twists 0 ini-len 720 ini-rmsd 0.19 opt-equ 727 opt-rmsd 0.26 chain-rmsd 0.19 Score 2158.32 align-len 727 gaps 0 (0.00%)
P-value 0.00e+00 Afp-num 167418 Identity 99.72% Similarity 99.86%
Block  0 afp 90 score 2158.32 rmsd  0.19 gap 0 (0.00%)

Chain 1:   27 DSLAPEDGSHRPAAEPTPPGAQPTAPGSLKAPDTRNEKLNSLEDVRKGSENYALTTNQGVRIADDQNSLR
              1111111111111111111111111111111111111111111111111111111111111111111111
Chain 2:   27 DSLAPEDGSHRPAAEPTPPGAQPTAPGSLKAPDTRNEKLNSLEDVRKGSENYALTTNQGVRIADDQNSLR

Chain 1:   97 AGSRGPTLLEDFILREKITHFDHERIPERIVHARGSAAHGYFQPYKSLSDITKADFLSDPNKITPVFVRF
              1111111111111111111111111111111111111111111111111111111111111111111111
Chain 2:   97 AGSRGPTLLEDFILREKITHFDHERIPERIVHARGSAAHGYFQPYKSLSDITKADFLSDPNKITPVFVRF

Chain 1:  167 STCQGGAGSADTVRDIRGFATKFYTEEGIFDLVGNNTPIFFIQDAHKFPDFVHAVKPEPHWAIPQGQSAH
              1111111111111111111111111111111111111111111111111111111111111111111111
Chain 2:  167 STVQGGAGSADTVRNIRGFATKFYTEEGIFDLVGNNTPIFFIQDAHKFPDFVHAVKPEPHWAIPQGQSAH

Chain 1:  237 DTFWDYVSLQPETLHNVMWAMSDRGIPRSYRTMEGFGIHTFRLINAEGKATFVRFHWKPLAGKASLVWDE
              1111111111111111111111111111111111111111111111111111111111111111111111
Chain 2:  237 DTFWDYVSLQPETLHNVMWAMSDRGIPRSYRTMEGFGIHTFRLINAEGKATFVRFHWKPLAGKASLVWDE

Chain 1:  307 AQKLTGRDPDFHRRELWEAIEAGDFPEYELGFQLIPEEDEFKFDFDLLDPTKLIPEELVPVQRVGKMVLN
              1111111111111111111111111111111111111111111111111111111111111111111111
Chain 2:  307 AQKLTGRDPDFHRRELWEAIEAGDFPEYELGFQLIPEEDEFKFDFDLLDPTKLIPEELVPVQRVGKMVLN

Chain 1:  377 RNPDNFFAENEQAAFHPGHIVPGLDFTNDPLLQGRLFSYTDTQISRLGGPNFHEIPINRPTCPYHNFQRD
              1111111111111111111111111111111111111111111111111111111111111111111111
Chain 2:  377 RNPDNFFAENEQAAFHPGHIVPGLDFTNDPLLQGRLFSYTDTQISRLGGPNFHEIPINRPTCPYHNFQRD

Chain 1:  447 GMHRMGIDTNPANYEPNSINDNWPRETPPGPKRGGFESYQERVEGNKVRERSPSFGEYYSHPRLFWLSQT
              1111111111111111111111111111111111111111111111111111111111111111111111
Chain 2:  447 GMHRMGIDTNPANYEPNSINDNWPRETPPGPKRGGFESYQERVEGNKVRERSPSFGEYYSHPRLFWLSQT

Chain 1:  517 PFEQRHIVDGFSFELSKVVRPYIRERVVDQLAHIDLTLAQAVAKNLGIELTDDQLNITPPPDVNGLKKDP
              1111111111111111111111111111111111111111111111111111111111111111111111
Chain 2:  517 PFEQRHIVDGFSFELSKVVRPYIRERVVDQLAHIDLTLAQAVAKNLGIELTDDQLNITPPPDVNGLKKDP

Chain 1:  587 SLSLYAIPDGDVKGRVVAILLNDEVRSADLLAILKALKAKGVHAKLLYSRMGEVTADDGTVLPIAATFAG
              1111111111111111111111111111111111111111111111111111111111111111111111
Chain 2:  587 SLSLYAIPDGDVKGRVVAILLNDEVRSADLLAILKALKAKGVHAKLLYSRMGEVTADDGTVLPIAATFAG

Chain 1:  657 APSLTVDAVIVPCGNIADIADNGDANYYLMEAYKHLKPIALAGDARKFKATIKIADQGEEGIVEADSADG
              1111111111111111111111111111111111111111111111111111111111111111111111
Chain 2:  657 APSLTVDAVIVPCGNIADIADNGDANYYLMEAYKHLKPIALAGDARKFKATIKIADQGEEGIVEADSADG

Chain 1:  727 SFMDELLTLMAAHRVWSRIPKIDKIPA
              111111111111111111111111111
Chain 2:  727 SFMDELLTLMAAHRVWSRIPKIDKIPA
```

UniProt ID: P21179

PDB ID: 1YE9_C

```
Align 1cf9.A.pdb 727 with 1ye9.C.pdb 223
Twists 0 ini-len 216 ini-rmsd 0.24 opt-equ 222 opt-rmsd 0.32 chain-rmsd 0.24 Score 646.85 align-len 222 gaps 0 (0.00%)
P-value 0.00e+00 Afp-num 52427 Identity 99.55% Similarity 99.55%
Block  0 afp 27 score 646.85 rmsd  0.24 gap 0 (0.00%)

                  .    :    .    :    .    :    .    :    .    :    .    :    .    :
Chain 1:   76 ENYALTTNQGVRIADDQNSLRAGSRGPTLLEDFILREKITHFDHERIPERIVHARGSAAHGYFQPYKSLS
              1111111111111111111111111111111111111111111111111111111111111111111111
Chain 2:   76 ENYALTTNQGVRIADDQNSLRAGSRGPTLLEDFILREKITHFDHERIPERIVHARGSAAHGYFQPYKSLS

                  .    :    .    :    .    :    .    :    .    :    .    :    .    :
Chain 1:  146 DITKADFLSDPNKITPVFVRFSTCQGGAGSADTVRDIRGFATKFYTEEGIFDLVGNNTPIFFIQDAHKFP
              1111111111111111111111111111111111111111111111111111111111111111111111
Chain 2:  146 DITKADFLSDPNKITPVFVRFSTVQGGAGSADTVRDIRGFATKFYTEEGIFDLVGNNTPIFFIQDAHKFP

                  .    :    .    :    .    :    .    :    .    :    .    :    .    :
Chain 1:  216 DFVHAVKPEPHWAIPQGQSAHDTFWDYVSLQPETLHNVMWAMSDRGIPRSYRTMEGFGIHTFRLINAEGK
              1111111111111111111111111111111111111111111111111111111111111111111111
Chain 2:  216 DFVHAVKPEPHWAIPQGQSAHDTFWDYVSLQPETLHNVMWAMSDRGIPRSYRTMEGFGIHTFRLINAEGK

                  .    :
Chain 1:  286 ATFVRFHWKPLA
              111111111111
Chain 2:  286 ATFVRFHWKPLA
```

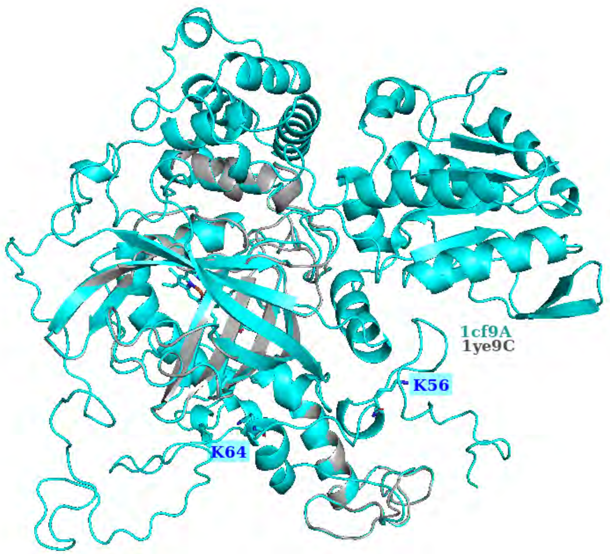

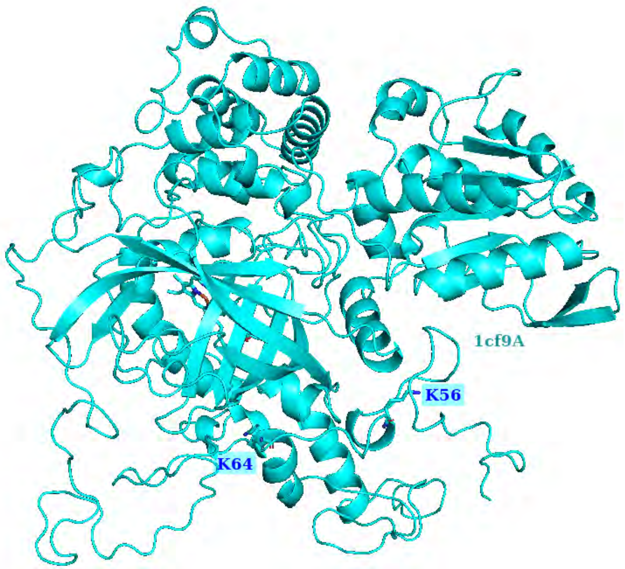

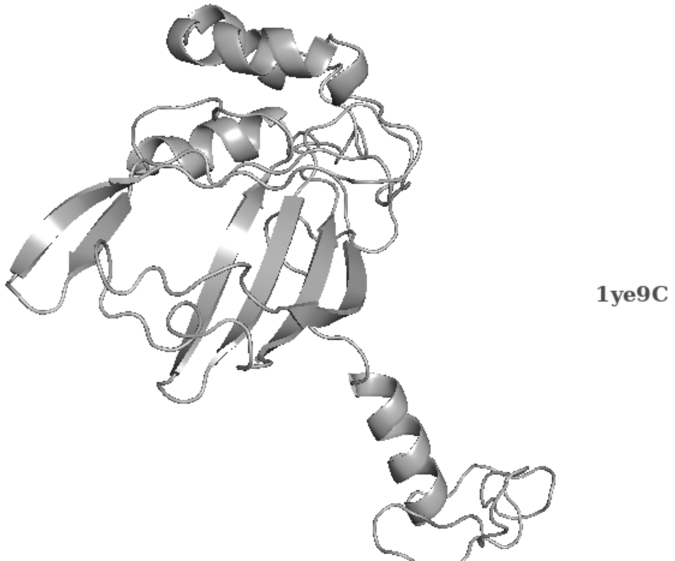

UniProt ID: P21179

PDB ID: 3P9P_A

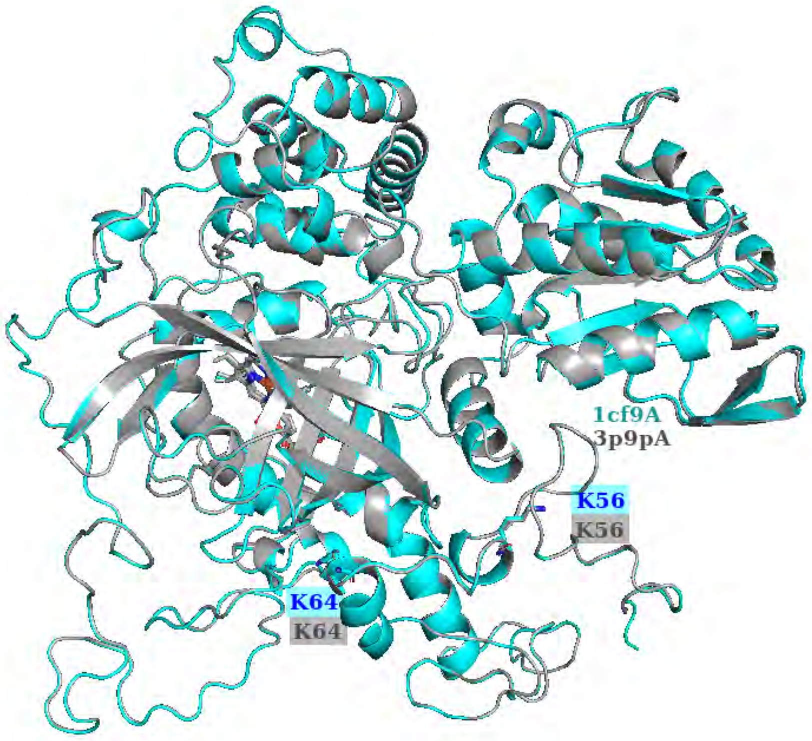

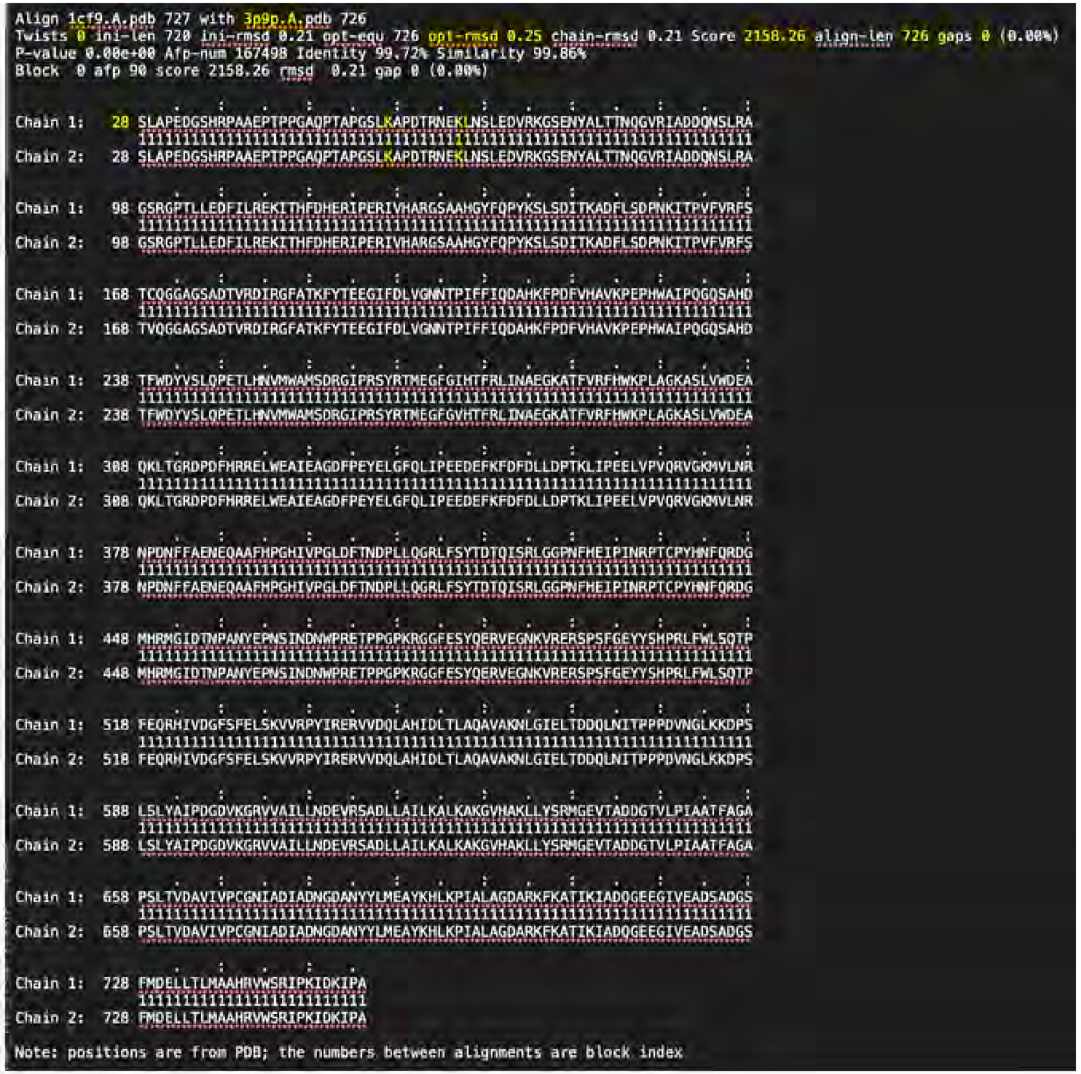

```
Align 1cf9.A.pdb 727 with 3p9p.A.pdb 726
Twists 0 ini-len 720 ini-rmsd 0.21 opt-equ 726 opt-rmsd 0.25 chain-rmsd 0.21 Score 2158.26 align-len 726 gaps 0 (0.00%)
P-value 0.00e+00 Afp-num 167498 Identity 99.72% Similarity 99.86%
Block  0 afp 90 score 2158.26 rmsd  0.21 gap 0 (0.00%)

Chain 1:   28 SLAPEDGSHRPAAEPTPPGAQPTAPGSLKAPDTRNEKLNSLEDVRKGSENYALTTNQGVRIADDQNSLRA
              1111111111111111111111111111111111111111111111111111111111111111111111
Chain 2:   28 SLAPEDGSHRPAAEPTPPGAQPTAPGSLKAPDTRNEKLNSLEDVRKGSENYALTTNQGVRIADDQNSLRA

Chain 1:   98 GSRGPTLLEDFILREKITHFDHERIPERIVHARGSAAHGYFQPYKSLSDITKADFLSDPNKITPVFVRFS
              1111111111111111111111111111111111111111111111111111111111111111111111
Chain 2:   98 GSRGPTLLEDFILREKITHFDHERIPERIVHARGSAAHGYFQPYKSLSDITKADFLSDPNKITPVFVRFS

Chain 1:  168 TCQGGAGSADTVRDIRGFATKFYTEEGIFDLVGNNTPIFFIQDAHKFPDFVHAVKPEPHWAIPQGQSAHD
              1111111111111111111111111111111111111111111111111111111111111111111111
Chain 2:  168 TVQGGAGSADTVRDIRGFATKFYTEEGIFDLVGNNTPIFFIQDAHKFPDFVHAVKPEPHWAIPQGQSAHD

Chain 1:  238 TFWDYVSLQPETLHNVMWAMSDRGIPRSYRTMEGFGIHTFRLINAEGKATFVRFHWKPLAGKASLVWDEA
              1111111111111111111111111111111111111111111111111111111111111111111111
Chain 2:  238 TFWDYVSLQPETLHNVMWAMSDRGIPRSYRTMEGFGVHTFRLINAEGKATFVRFHWKPLAGKASLVWDEA

Chain 1:  308 QKLTGRDPDFHRRELWEAIEAGDFPEYELGFQLIPEEDEFKFDFDLLDPTKLIPEELVPVQRVGKMVLNR
              1111111111111111111111111111111111111111111111111111111111111111111111
Chain 2:  308 QKLTGRDPDFHRRELWEAIEAGDFPEYELGFQLIPEEDEFKFDFDLLDPTKLIPEELVPVQRVGKMVLNR

Chain 1:  378 NPDNFFAENEQAAFHPGHIVPGLDFTNDPLLQGRLFSYTDTQISRLGGPNFHEIPINRPTCPYHNFQRDG
              1111111111111111111111111111111111111111111111111111111111111111111111
Chain 2:  378 NPDNFFAENEQAAFHPGHIVPGLDFTNDPLLQGRLFSYTDTQISRLGGPNFHEIPINRPTCPYHNFQRDG

Chain 1:  448 MHRMGIDTNPANYEPNSINDNWPRETPPGPKRGGFESYQERVEGNKVRERSPSFGEYYSHPRLFWLSQTP
              1111111111111111111111111111111111111111111111111111111111111111111111
Chain 2:  448 MHRMGIDTNPANYEPNSINDNWPRETPPGPKRGGFESYQERVEGNKVRERSPSFGEYYSHPRLFWLSQTP

Chain 1:  518 FEQRHIVDGFSFELSKVVRPYIRERVVDQLAHIDLTLAQAVAKNLGIELTDDQLNITPPPDVNGLKKDPS
              1111111111111111111111111111111111111111111111111111111111111111111111
Chain 2:  518 FEQRHIVDGFSFELSKVVRPYIRERVVDQLAHIDLTLAQAVAKNLGIELTDDQLNITPPPDVNGLKKDPS

Chain 1:  588 LSLYAIPDGDVKGRVVAILLNDEVRSADLLAILKALKAKGVHAKLLYSRMGEVTADDGTVLPIAATFAGA
              1111111111111111111111111111111111111111111111111111111111111111111111
Chain 2:  588 LSLYAIPDGDVKGRVVAILLNDEVRSADLLAILKALKAKGVHAKLLYSRMGEVTADDGTVLPIAATFAGA

Chain 1:  658 PSLTVDAVIVPCGNIADIADNGDANYYLMEAYKHLKPIALAGDARKFKATIKIADQGEEGIVEADSADGS
              1111111111111111111111111111111111111111111111111111111111111111111111
Chain 2:  658 PSLTVDAVIVPCGNIADIADNGDANYYLMEAYKHLKPIALAGDARKFKATIKIADQGEEGIVEADSADGS

Chain 1:  728 FMDELLTLMAAHRVWSRIPKIDKIPA
              11111111111111111111111111
Chain 2:  728 FMDELLTLMAAHRVWSRIPKIDKIPA

Note: positions are from PDB; the numbers between alignments are block index
```

UniProt ID: P21179

PDB ID: 3P9Q_B

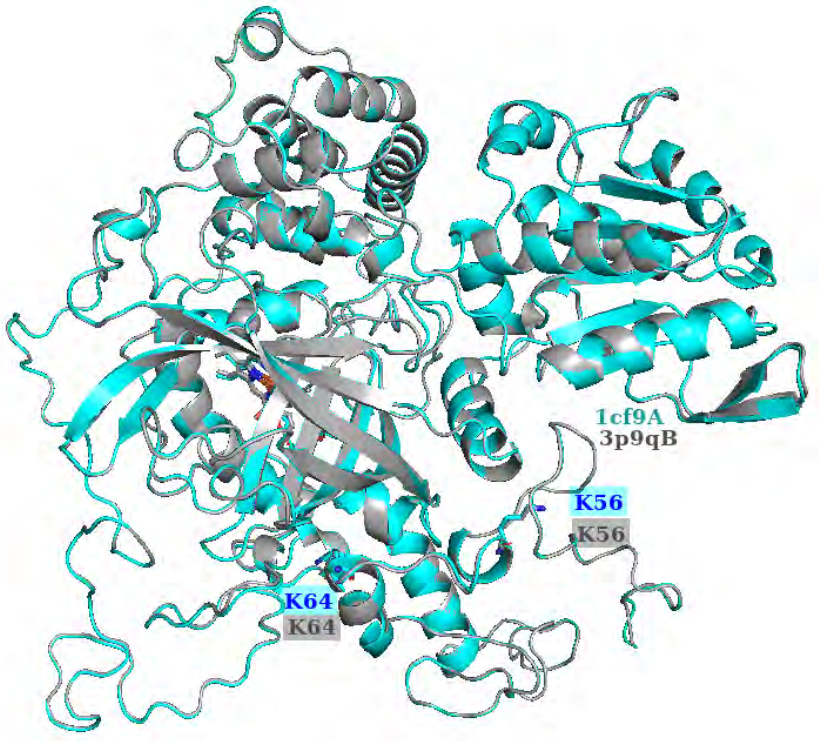

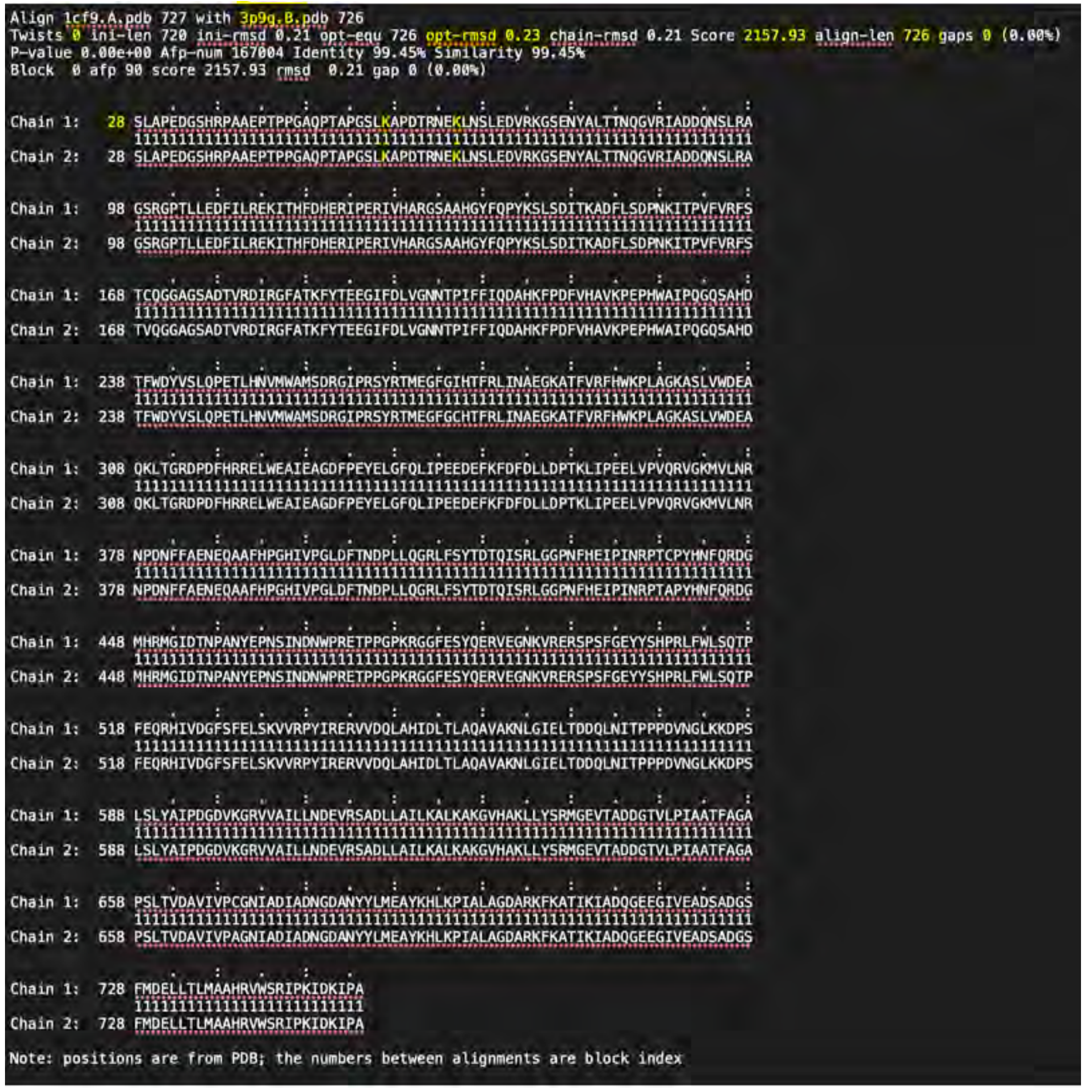

```
Align 1cf9.A.pdb 727 with 3p9q.B.pdb 726
Twists 0 ini-len 720 ini-rmsd 0.21 opt-equ 726 opt-rmsd 0.23 chain-rmsd 0.21 Score 2157.93 align-len 726 gaps 0 (0.00%)
P-value 0.00e+00 Afp-num 167004 Identity 99.45% Similarity 99.45%
Block  0 afp 90 score 2157.93 rmsd  0.21 gap 0 (0.00%)

Chain 1:   28 SLAPEDGSHRPAAEPTPPGAQPTAPGSLKAPDTRNEKLNSLEDVRKGSENYALTTNQGVRIADDQNSLRA
              1111111111111111111111111111111111111111111111111111111111111111111111
Chain 2:   28 SLAPEDGSHRPAAEPTPPGAQPTAPGSLKAPDTRNEKLNSLEDVRKGSENYALTTNQGVRIADDQNSLRA

Chain 1:   98 GSRGPTLLEDFILREKITHFDHERIPERIVHARGSAAHGYFQPYKSLSDITKADFLSDPNKITPVFVRFS
              1111111111111111111111111111111111111111111111111111111111111111111111
Chain 2:   98 GSRGPTLLEDFILREKITHFDHERIPERIVHARGSAAHGYFQPYKSLSDITKADFLSDPNKITPVFVRFS

Chain 1:  168 TCQGGAGSADTVRDIRGFATKFYTEEGIFDLVGNNTPIFFIQDAHKFPDFVHAVKPEPHWAIPQGQSAHD
              1111111111111111111111111111111111111111111111111111111111111111111111
Chain 2:  168 TVQGGAGSADTVRDIRGFATKFYTEEGIFDLVGNNTPIFFIQDAHKFPDFVHAVKPEPHWAIPQGQSAHD

Chain 1:  238 TFWDYVSLQPETLHNVMWAMSDRGIPRSYRTMEGFGIHTFRLINAEGKATFVRFHWKPLAGKASLVWDEA
              1111111111111111111111111111111111111111111111111111111111111111111111
Chain 2:  238 TFWDYVSLQPETLHNVMWAMSDRGIPRSYRTMEGFGCHTFRLINAEGKATFVRFHWKPLAGKASLVWDEA

Chain 1:  308 QKLTGRDPDFHRRELWEAIEAGDFPEYELGFQLIPEEDEFKFDFDLLDPTKLIPEELVPVQRVGKMVLNR
              1111111111111111111111111111111111111111111111111111111111111111111111
Chain 2:  308 QKLTGRDPDFHRRELWEAIEAGDFPEYELGFQLIPEEDEFKFDFDLLDPTKLIPEELVPVQRVGKMVLNR

Chain 1:  378 NPDNFFAENEQAAFHPGHIVPGLDFTNDPLLQGRLFSYTDTQISRLGGPNFHEIPINRPTCPYHNFQRDG
              1111111111111111111111111111111111111111111111111111111111111111111111
Chain 2:  378 NPDNFFAENEQAAFHPGHIVPGLDFTNDPLLQGRLFSYTDTQISRLGGPNFHEIPINRPTAPYHNFQRDG

Chain 1:  448 MHRMGIDTNPANYEPNSINDNWPRETPPGPKRGGFESYQERVEGNKVRERSPSFGEYYSHPRLFWLSQTP
              1111111111111111111111111111111111111111111111111111111111111111111111
Chain 2:  448 MHRMGIDTNPANYEPNSINDNWPRETPPGPKRGGFESYQERVEGNKVRERSPSFGEYYSHPRLFWLSQTP

Chain 1:  518 FEQRHIVDGFSFELSKVVRPYIRERVVDQLAHIDLTLAQAVAKNLGIELTDDQLNITPPPDVNGLKKDPS
              1111111111111111111111111111111111111111111111111111111111111111111111
Chain 2:  518 FEQRHIVDGFSFELSKVVRPYIRERVVDQLAHIDLTLAQAVAKNLGIELTDDQLNITPPPDVNGLKKDPS

Chain 1:  588 LSLYAIPDGDVKGRVVAILLNDEVRSADLLAILKALKAKGVHAKLLYSRMGEVTADDGTVLPIAATFAGA
              1111111111111111111111111111111111111111111111111111111111111111111111
Chain 2:  588 LSLYAIPDGDVKGRVVAILLNDEVRSADLLAILKALKAKGVHAKLLYSRMGEVTADDGTVLPIAATFAGA

Chain 1:  658 PSLTVDAVIVPCGNIADIADNGDANYYLMEAYKHLKPIALAGDARKFKATIKIADQGEEGIVEADSADGS
              1111111111111111111111111111111111111111111111111111111111111111111111
Chain 2:  658 PSLTVDAVIVPAGNIADIADNGDANYYLMEAYKHLKPIALAGDARKFKATIKIADQGEEGIVEADSADGS

Chain 1:  728 FMDELLTLMAAHRVWSRIPKIDKIPA
              11111111111111111111111111
Chain 2:  728 FMDELLTLMAAHRVWSRIPKIDKIPA

Note: positions are from PDB; the numbers between alignments are block index
```

UniProt ID: P21179
PDB ID: 3P9R_B

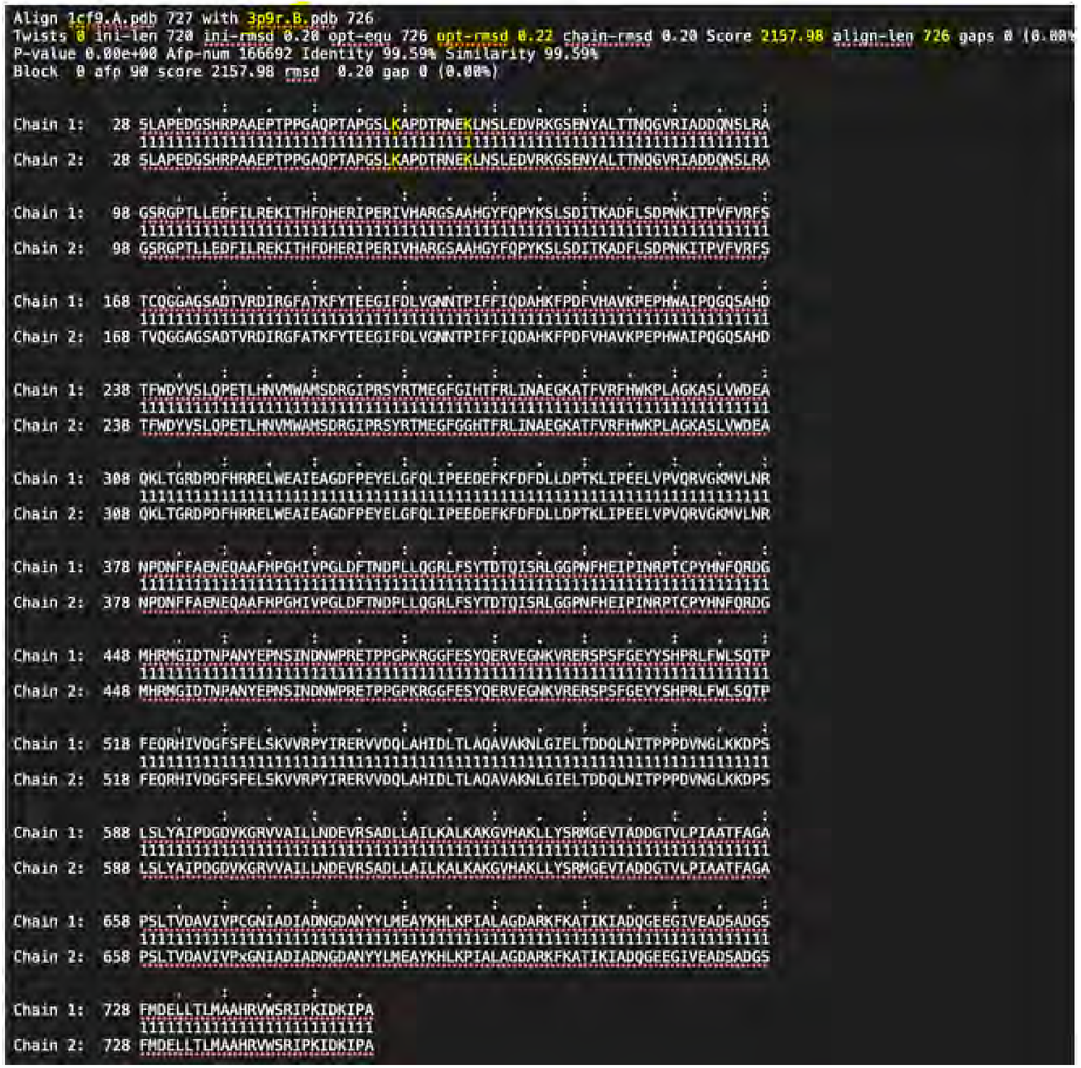

```
Align 1cf9.A.pdb 727 with 3p9r.B.pdb 726
Twists 0 ini-len 720 ini-rmsd 0.20 opt-equ 726 opt-rmsd 0.22 chain-rmsd 0.20 Score 2157.98 align-len 726 gaps 0 (0.00%
P-value 0.00e+00 Afp-num 166692 Identity 99.59% Similarity 99.59%
Block  0 afp 90 score 2157.98 rmsd  0.20 gap 0 (0.00%)

                 .    :    .    :    .    :    .    :    .    :    .    :    .    :
Chain 1:   28 SLAPEDGSHRPAAEPTPPGAQPTAPGSLKAPDTRNEKLNSLEDVRKGSENYALTTNQGVRIADDQNSLRA
              1111111111111111111111111111111111111111111111111111111111111111111111
Chain 2:   28 SLAPEDGSHRPAAEPTPPGAQPTAPGSLKAPDTRNEKLNSLEDVRKGSENYALTTNQGVRIADDQNSLRA

                 .    :    .    :    .    :    .    :    .    :    .    :    .    :
Chain 1:   98 GSRGPTLLEDFILREKITHFDHERIPERIVHARGSAAHGYFQPYKSLSDITKADFLSDPNKITPVFVRFS
              1111111111111111111111111111111111111111111111111111111111111111111111
Chain 2:   98 GSRGPTLLEDFILREKITHFDHERIPERIVHARGSAAHGYFQPYKSLSDITKADFLSDPNKITPVFVRFS

                 .    :    .    :    .    :    .    :    .    :    .    :    .    :
Chain 1:  168 TCQGGAGSADTVRDIRGFATKFYTEEGIFDLVGNNTPIFFIQDAHKFPDFVHAVKPEPHWAIPQGQSAHD
              1111111111111111111111111111111111111111111111111111111111111111111111
Chain 2:  168 TVQGGAGSADTVRDIRGFATKFYTEEGIFDLVGNNTPIFFIQDAHKFPDFVHAVKPEPHWAIPQGQSAHD

                 .    :    .    :    .    :    .    :    .    :    .    :    .    :
Chain 1:  238 TFWDYVSLQPETLHNVMWAMSDRGIPRSYRTMEGFGIHTFRLINAEGKATFVRFHWKPLAGKASLVWDEA
              1111111111111111111111111111111111111111111111111111111111111111111111
Chain 2:  238 TFWDYVSLQPETLHNVMWAMSDRGIPRSYRTMEGFGGHTFRLINAEGKATFVRFHWKPLAGKASLVWDEA

                 .    :    .    :    .    :    .    :    .    :    .    :    .    :
Chain 1:  308 QKLTGRDPDFHRRELWEAIEAGDFPEYELGFQLIPEEDEFKFDFDLLDPTKLIPEELVPVQRVGKMVLNR
              1111111111111111111111111111111111111111111111111111111111111111111111
Chain 2:  308 QKLTGRDPDFHRRELWEAIEAGDFPEYELGFQLIPEEDEFKFDFDLLDPTKLIPEELVPVQRVGKMVLNR

                 .    :    .    :    .    :    .    :    .    :    .    :    .    :
Chain 1:  378 NPDNFFAENEQAAFHPGHIVPGLDFTNDPLLQGRLFSYTDTQISRLGGPNFHEIPINRPTCPYHNFQRDG
              1111111111111111111111111111111111111111111111111111111111111111111111
Chain 2:  378 NPDNFFAENEQAAFHPGHIVPGLDFTNDPLLQGRLFSYTDTQISRLGGPNFHEIPINRPTCPYHNFQRDG

                 .    :    .    :    .    :    .    :    .    :    .    :    .    :
Chain 1:  448 MHRMGIDTNPANYEPNSINDNWPRETPPGPKRGGFESYQERVEGNKVRERSPSFGEYYSHPRLFWLSQTP
              1111111111111111111111111111111111111111111111111111111111111111111111
Chain 2:  448 MHRMGIDTNPANYEPNSINDNWPRETPPGPKRGGFESYQERVEGNKVRERSPSFGEYYSHPRLFWLSQTP

                 .    :    .    :    .    :    .    :    .    :    .    :    .    :
Chain 1:  518 FEQRHIVDGFSFELSKVVRPYIRERVVDQLAHIDLTLAQAVAKNLGIELTDDQLNITPPPDVNGLKKDPS
              1111111111111111111111111111111111111111111111111111111111111111111111
Chain 2:  518 FEQRHIVDGFSFELSKVVRPYIRERVVDQLAHIDLTLAQAVAKNLGIELTDDQLNITPPPDVNGLKKDPS

                 .    :    .    :    .    :    .    :    .    :    .    :    .    :
Chain 1:  588 LSLYAIPDGDVKGRVVAILLNDEVRSADLLAILKALKAKGVHAKLLYSRMGEVTADDGTVLPIAATFAGA
              1111111111111111111111111111111111111111111111111111111111111111111111
Chain 2:  588 LSLYAIPDGDVKGRVVAILLNDEVRSADLLAILKALKAKGVHAKLLYSRMGEVTADDGTVLPIAATFAGA

                 .    :    .    :    .    :    .    :    .    :    .    :    .    :
Chain 1:  658 PSLTVDAVIVPCGNIADIADNGDANYYLMEAYKHLKPIALAGDARKFKATIKIADQGEEGIVEADSADGS
              1111111111111111111111111111111111111111111111111111111111111111111111
Chain 2:  658 PSLTVDAVIVPxGNIADIADNGDANYYLMEAYKHLKPIALAGDARKFKATIKIADQGEEGIVEADSADGS

                 .    :    .    :    .
Chain 1:  728 FMDELLTLMAAHRVWSRIPKIDKIPA
              11111111111111111111111111
Chain 2:  728 FMDELLTLMAAHRVWSRIPKIDKIPA
```

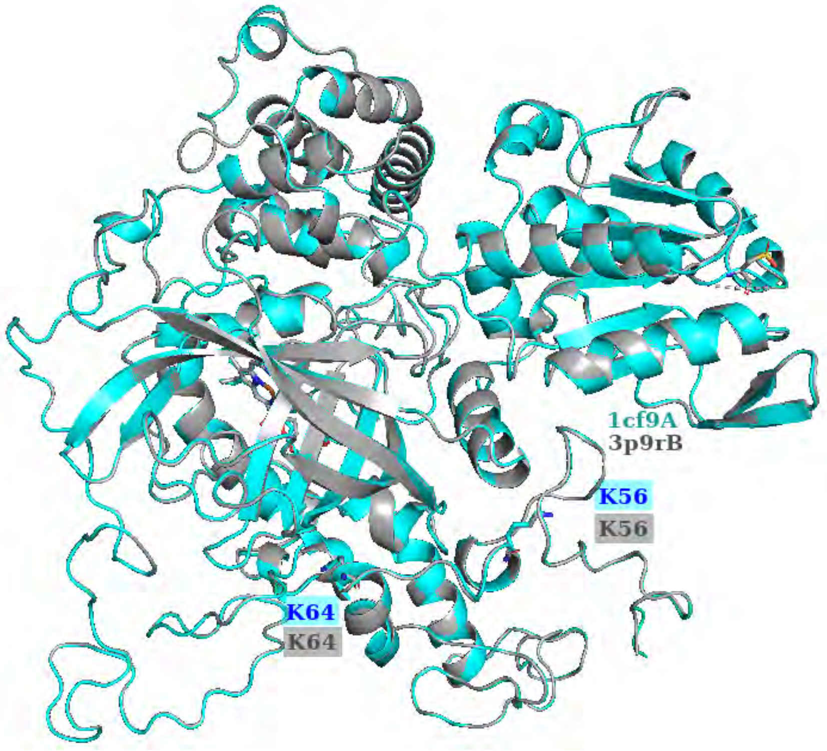

UniProt ID: P21179

PDB ID: 3P9S_B

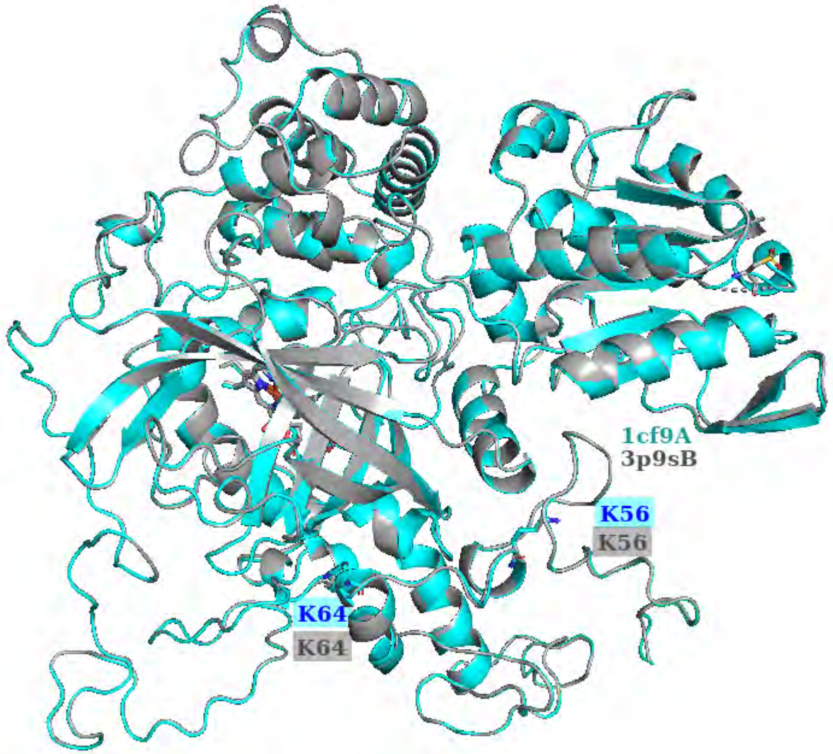


```
Align 1cf9.A.pdb 727 with 3p9s.B.pdb 726
Twists 0 ini-len 720 ini-rmsd 0.20 opt-equ 726 opt-rmsd 0.22 chain-rmsd 0.20 Score 2157.87 align-len 726 gaps 0 (0.00%)
P-value 0.00e+00 Afp-num 166897 Identity 99.59% Similarity 99.59%
Block  0 afp 90 score 2157.87 rmsd  0.20 gap 0 (0.00%)

                    .    :    .    :    .    :    .    :    .    :    .    :    .    :
Chain 1:   28 SLAPEDGSHRPAAEPTPPGAQPTAPGSLKAPDTRNEKLNSLEDVRKGSENYALTTNQGVRIADDQNSLRA
              1111111111111111111111111111111111111111111111111111111111111111111111
Chain 2:   28 SLAPEDGSHRPAAEPTPPGAQPTAPGSLKAPDTRNEKLNSLEDVRKGSENYALTTNQGVRIADDQNSLRA

                    .    :    .    :    .    :    .    :    .    :    .    :    .    :
Chain 1:   98 GSRGPTLLEDFILREKITHFDHERIPERIVHARGSAAHGYFQPYKSLSDITKADFLSDPNKITPVFVRFS
              1111111111111111111111111111111111111111111111111111111111111111111111
Chain 2:   98 GSRGPTLLEDFILREKITHFDHERIPERIVHARGSAAHGYFQPYKSLSDITKADFLSDPNKITPVFVRFS

                    .    :    .    :    .    :    .    :    .    :    .    :    .    :
Chain 1:  168 TCQGGAGSADTVRDIRGFATKFYTEEGIFDLVGNNTPIFFIQDAHKFPDFVHAVKPEPHWAIPQGQSAHD
              1111111111111111111111111111111111111111111111111111111111111111111111
Chain 2:  168 TVQGGAGSADTVRDIRGFATKFYTEEGIFDLVGNNTPIFFIQDAHKFPDFVHAVKPEPHWAIPQGQSAHD

                    .    :    .    :    .    :    .    :    .    :    .    :    .    :
Chain 1:  238 TFWDYVSLQPETLHNVMWAMSDRGIPRSYRTMEGFGIHTFRLINAEGKATFVRFHWKPLAGKASLVWDEA
              1111111111111111111111111111111111111111111111111111111111111111111111
Chain 2:  238 TFWDYVSLQPETLHNVMWAMSDRGIPRSYRTMEGFGAHTFRLINAEGKATFVRFHWKPLAGKASLVWDEA

                    .    :    .    :    .    :    .    :    .    :    .    :    .    :
Chain 1:  308 QKLTGRDPDFHRRELWEAIEAGDFPEYELGFQLIPEEDEFKFDFDLLDPTKLIPEELVPVQRVGKMVLNR
              1111111111111111111111111111111111111111111111111111111111111111111111
Chain 2:  308 QKLTGRDPDFHRRELWEAIEAGDFPEYELGFQLIPEEDEFKFDFDLLDPTKLIPEELVPVQRVGKMVLNR

                    .    :    .    :    .    :    .    :    .    :    .    :    .    :
Chain 1:  378 NPDNFFAENEQAAFHPGHIVPGLDFTNDPLLQGRLFSYTDTQISRLGGPNFHEIPINRPTCPYHNFQRDG
              1111111111111111111111111111111111111111111111111111111111111111111111
Chain 2:  378 NPDNFFAENEQAAFHPGHIVPGLDFTNDPLLQGRLFSYTDTQISRLGGPNFHEIPINRPTCPYHNFQRDG

                    .    :    .    :    .    :    .    :    .    :    .    :    .    :
Chain 1:  448 MHRMGIDTNPANYEPNSINDNWPRETPPGPKRGGFESYQERVEGNKVRERSPSFGEYYSHPRLFWLSQTP
              1111111111111111111111111111111111111111111111111111111111111111111111
Chain 2:  448 MHRMGIDTNPANYEPNSINDNWPRETPPGPKRGGFESYQERVEGNKVRERSPSFGEYYSHPRLFWLSQTP

                    .    :    .    :    .    :    .    :    .    :    .    :    .    :
Chain 1:  518 FEQRHIVDGFSFELSKVVRPYIRERVVDQLAHIDLTLAQAVAKNLGIELTDDQLNITPPPDVNGLKKDPS
              1111111111111111111111111111111111111111111111111111111111111111111111
Chain 2:  518 FEQRHIVDGFSFELSKVVRPYIRERVVDQLAHIDLTLAQAVAKNLGIELTDDQLNITPPPDVNGLKKDPS

                    .    :    .    :    .    :    .    :    .    :    .    :    .    :
Chain 1:  588 LSLYAIPDGDVKGRVVAILLNDEVRSADLLAILKALKAKGVHAKLLYSRMGEVTADDGTVLPIAATFAGA
              1111111111111111111111111111111111111111111111111111111111111111111111
Chain 2:  588 LSLYAIPDGDVKGRVVAILLNDEVRSADLLAILKALKAKGVHAKLLYSRMGEVTADDGTVLPIAATFAGA

                    .    :    .    :    .    :    .    :    .    :    .    :    .    :
Chain 1:  658 PSLTVDAVIVPCGNIADIADNGDANYYLMEAYKHLKPIALAGDARKFKATIKIADQGEEGIVEADSADGS
              1111111111111111111111111111111111111111111111111111111111111111111111
Chain 2:  658 PSLTVDAVIVPxGNIADIADNGDANYYLMEAYKHLKPIALAGDARKFKATIKIADQGEEGIVEADSADGS

                    .    :    .    :    .
Chain 1:  728 FMDELLTLMAAHRVWSRIPKIDKIPA
              11111111111111111111111111
Chain 2:  728 FMDELLTLMAAHRVWSRIPKIDKIPA
```

UniProt ID: P21179

PDB ID: 3PQ2_B

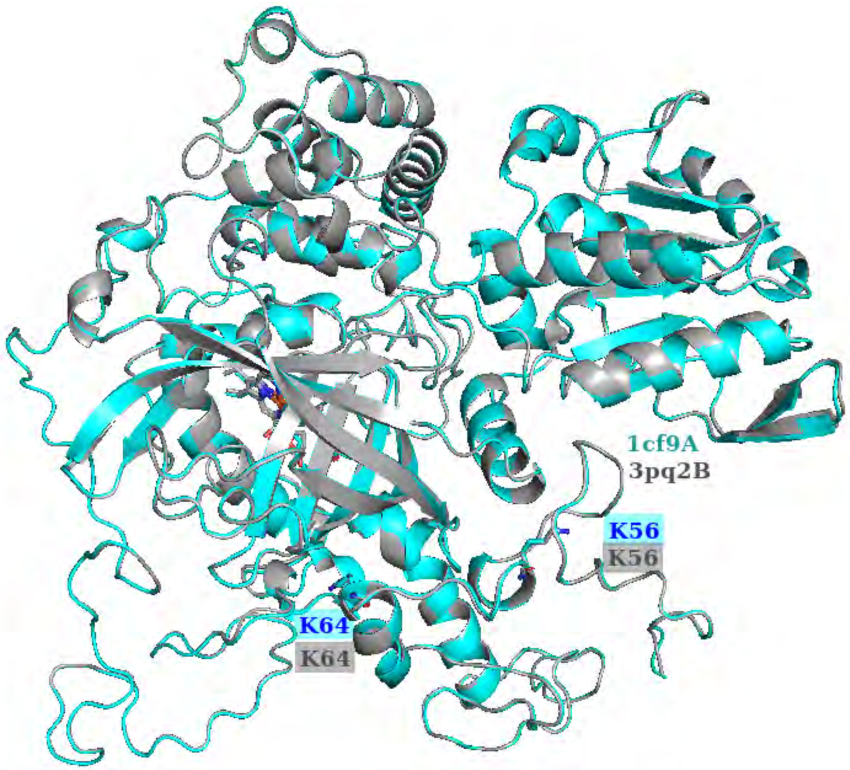


```
Align 1cf9.A.pdb 727 with 3pq2.B.pdb 726
Twists 0 ini-len 720 ini-rmsd 0.21 opt-equ 726 opt-rmsd 0.23 chain-rmsd 0.21 Score 2157.77 align-len 726 gaps 0 (0.00%)
P-value 0.00e+00 Afp-num 166830 Identity 99.45% Similarity 99.45%
Block  0 afp 90 score 2157.77 rmsd  0.21 gap 0 (0.00%)

Chain 1:   28 SLAPEDGSHRPAAEPTPPGAQPTAPGSLKAPDTRNEKLNSLEDVRKGSENYALTTNQGVRIADDQNSLRA
              1111111111111111111111111111111111111111111111111111111111111111111111
Chain 2:   28 SLAPEDGSHRPAAEPTPPGAQPTAPGSLKAPDTRNEKLNSLEDVRKGSENYALTTNQGVRIADDQNSLRA

Chain 1:   98 GSRGPTLLEDFILREKITHFDHERIPERIVHARGSAAHGYFQPYKSLSDITKADFLSDPNKITPVFVRFS
              1111111111111111111111111111111111111111111111111111111111111111111111
Chain 2:   98 GSRGPTLLEDFILREKITHFDHERIPERIVHARGSAAHGYFQPYKSLSDITKADFLSDPNKITPVFVRFS

Chain 1:  168 TCQGGAGSADTVRDIRGFATKFYTEEGIFDLVGNNTPIFFIQDAHKFPDFVHAVKPEPHWAIPQGQSAHD
              1111111111111111111111111111111111111111111111111111111111111111111111
Chain 2:  168 TVQGGAGSADTVRDIRGFATKFYTEEGIFDLVGNNTPIFFIQDAHKFPDFVHAVKPEPHWAIPQGQSAHD

Chain 1:  238 TFWDYVSLQPETLHNVMWAMSDRGIPRSYRTMEGFGIHTFRLINAEGKATFVRFHWKPLAGKASLVWDEA
              1111111111111111111111111111111111111111111111111111111111111111111111
Chain 2:  238 TFWDYVSLQPETLHNVMWAMSDRGIPRSYRTMEGFGCHTFRLINAEGKATFVRFHWKPLAGKASLVWDEA

Chain 1:  308 QKLTGRDPDFHRRELWEAIEAGDFPEYELGFQLIPEEDEFKFDFDLLDPTKLIPEELVPVQRVGKMVLNR
              1111111111111111111111111111111111111111111111111111111111111111111111
Chain 2:  308 QKLTGRDPDFHRRELWEAIEAGDFPEYELGFQLIPEEDEFKFDFDLLDPTKLIPEELVPVQRVGKMVLNR

Chain 1:  378 NPDNFFAENEQAAFHPGHIVPGLDFTNDPLLQGRLFSYTDTQISRLGGPNFHEIPINRPTCPYHNFQRDG
              1111111111111111111111111111111111111111111111111111111111111111111111
Chain 2:  378 NPDNFFAENEQAAFHPGHIVPGLDFTNDPLLQGRLFSYTDTQISRLGGPNFHEIPINRPTAPYHNFQRDG

Chain 1:  448 MHRMGIDTNPANYEPNSINDNWPRETPPGPKRGGFESYQERVEGNKVRERSPSFGEYYSHPRLFWLSQTP
              1111111111111111111111111111111111111111111111111111111111111111111111
Chain 2:  448 MHRMGIDTNPANYEPNSINDNWPRETPPGPKRGGFESYQERVEGNKVRERSPSFGEYYSHPRLFWLSQTP

Chain 1:  518 FEQRHIVDGFSFELSKVVRPYIRERVVDQLAHIDLTLAQAVAKNLGIELTDDQLNITPPPDVNGLKKDPS
              1111111111111111111111111111111111111111111111111111111111111111111111
Chain 2:  518 FEQRHIVDGFSFELSKVVRPYIRERVVDQLAHIDLTLAQAVAKNLGIELTDDQLNITPPPDVNGLKKDPS

Chain 1:  588 LSLYAIPDGDVKGRVVAILLNDEVRSADLLAILKALKAKGVHAKLLYSRMGEVTADDGTVLPIAATFAGA
              1111111111111111111111111111111111111111111111111111111111111111111111
Chain 2:  588 LSLYAIPDGDVKGRVVAILLNDEVRSADLLAILKALKAKGVHAKLLYSRMGEVTADDGTVLPIAATFAGA

Chain 1:  658 PSLTVDAVIVPCGNIADIADNGDANYYLMEAYKHLKPIALAGDARKFKATIKIADQGEEGIVEADSADGS
              1111111111111111111111111111111111111111111111111111111111111111111111
Chain 2:  658 PSLTVDAVIVPAGNIADIADNGDANYYLMEAYKHLKPIALAGDARKFKATIKIADQGEEGIVEADSADGS

Chain 1:  728 FMDELLTLMAAHRVWSRIPKIDKIPA
              11111111111111111111111111
Chain 2:  728 FMDELLTLMAAHRVWSRIPKIDKIPA
```

UniProt ID: P21179
PDB ID: 3PQ3_B

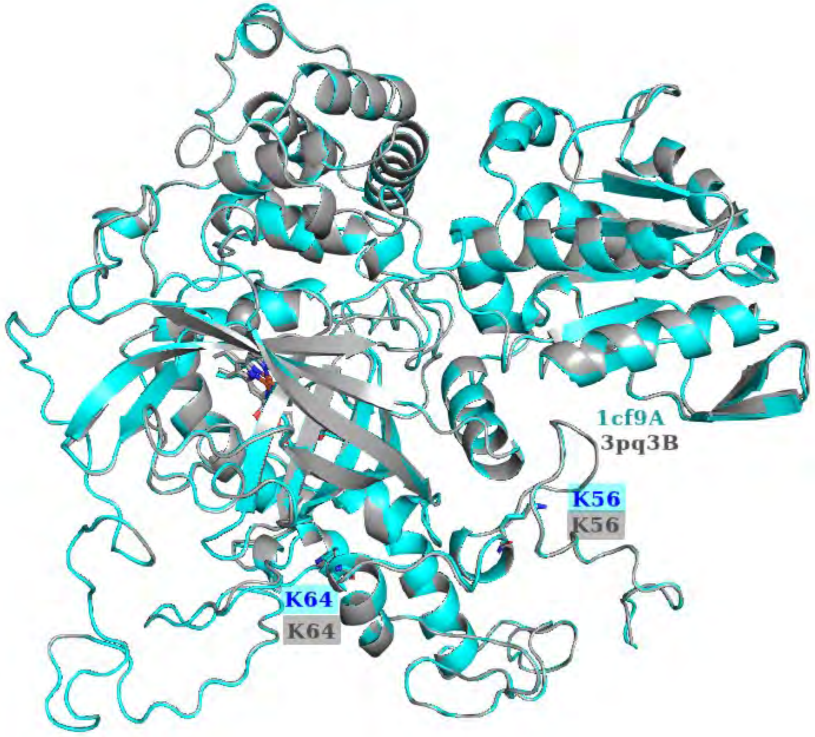

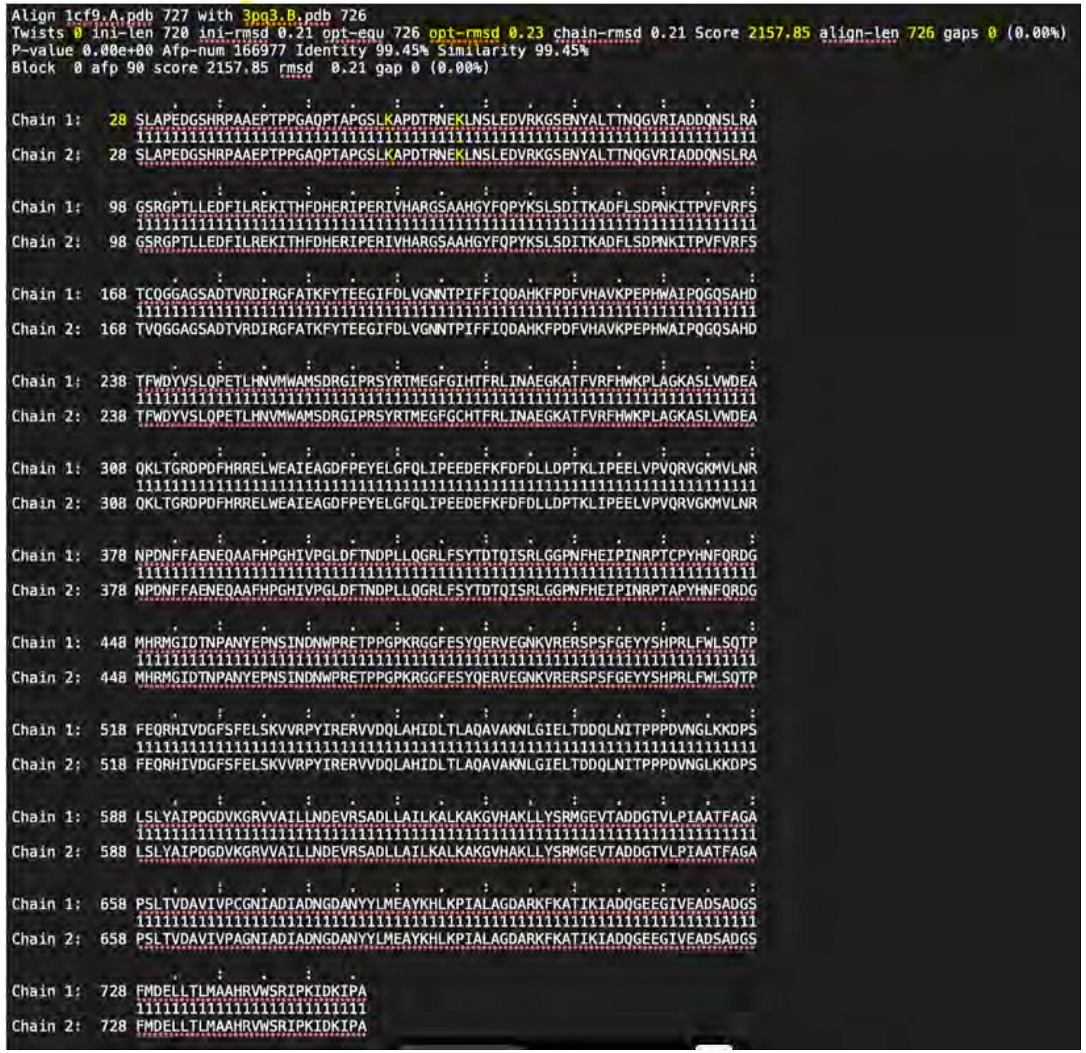

```
Align 1cf9.A.pdb 727 with 3pq3.B.pdb 726
Twists 0 ini-len 720 ini-rmsd 0.21 opt-equ 726 opt-rmsd 0.23 chain-rmsd 0.21 Score 2157.85 align-len 726 gaps 0 (0.00%)
P-value 0.00e+00 Afp-num 166977 Identity 99.45% Similarity 99.45%
Block  0 afp 90 score 2157.85 rmsd  0.21 gap 0 (0.00%)

Chain 1:   28 SLAPEDGSHRPAAEPTPPGAQPTAPGSLKAPDTRNEKLNSLEDVRKGSENYALTTNQGVRIADDQNSLRA
              1111111111111111111111111111111111111111111111111111111111111111111111
Chain 2:   28 SLAPEDGSHRPAAEPTPPGAQPTAPGSLKAPDTRNEKLNSLEDVRKGSENYALTTNQGVRIADDQNSLRA

Chain 1:   98 GSRGPTLLEDFILREKITHFDHERIPERIVHARGSAAHGYFQPYKSLSDITKADFLSDPNKITPVFVRFS
              1111111111111111111111111111111111111111111111111111111111111111111111
Chain 2:   98 GSRGPTLLEDFILREKITHFDHERIPERIVHARGSAAHGYFQPYKSLSDITKADFLSDPNKITPVFVRFS

Chain 1:  168 TCQGGAGSADTVRDIRGFATKFYTEEGIFDLVGNNTPIFFIQDAHKFPDFVHAVKPEPHWAIPQGQSAHD
              1111111111111111111111111111111111111111111111111111111111111111111111
Chain 2:  168 TVQGGAGSADTVRDIRGFATKFYTEEGIFDLVGNNTPIFFIQDAHKFPDFVHAVKPEPHWAIPQGQSAHD

Chain 1:  238 TFWDYVSLQPETLHNVMWAMSDRGIPRSYRTMEGFGIHTFRLINAEGKATFVRFHWKPLAGKASLVWDEA
              1111111111111111111111111111111111111111111111111111111111111111111111
Chain 2:  238 TFWDYVSLQPETLHNVMWAMSDRGIPRSYRTMEGFGCHTFRLINAEGKATFVRFHWKPLAGKASLVWDEA

Chain 1:  308 QKLTGRDPDFHRRELWEAIEAGDFPEYELGFQLIPEEDEFKFDFDLLDPTKLIPEELVPVQRVGKMVLNR
              1111111111111111111111111111111111111111111111111111111111111111111111
Chain 2:  308 QKLTGRDPDFHRRELWEAIEAGDFPEYELGFQLIPEEDEFKFDFDLLDPTKLIPEELVPVQRVGKMVLNR

Chain 1:  378 NPDNFFAENEQAAFHPGHIVPGLDFTNDPLLQGRLFSYTDTQISRLGGPNFHEIPINRPTCPYHNFQRDG
              1111111111111111111111111111111111111111111111111111111111111111111111
Chain 2:  378 NPDNFFAENEQAAFHPGHIVPGLDFTNDPLLQGRLFSYTDTQISRLGGPNFHEIPINRPTAPYHNFQRDG

Chain 1:  448 MHRMGIDTNPANYEPNSINDNWPRETPPGPKRGGFESYQERVEGNKVRERSPSFGEYYSHPRLFWLSQTP
              1111111111111111111111111111111111111111111111111111111111111111111111
Chain 2:  448 MHRMGIDTNPANYEPNSINDNWPRETPPGPKRGGFESYQERVEGNKVRERSPSFGEYYSHPRLFWLSQTP

Chain 1:  518 FEQRHIVDGFSFELSKVVRPYIRERVVDQLAHIDLTLAQAVAKNLGIELTDDQLNITPPPDVNGLKKDPS
              1111111111111111111111111111111111111111111111111111111111111111111111
Chain 2:  518 FEQRHIVDGFSFELSKVVRPYIRERVVDQLAHIDLTLAQAVAKNLGIELTDDQLNITPPPDVNGLKKDPS

Chain 1:  588 LSLYAIPDGDVKGRVVAILLNDEVRSADLLAILKALKAKGVHAKLLYSRMGEVTADDGTVLPIAATFAGA
              1111111111111111111111111111111111111111111111111111111111111111111111
Chain 2:  588 LSLYAIPDGDVKGRVVAILLNDEVRSADLLAILKALKAKGVHAKLLYSRMGEVTADDGTVLPIAATFAGA

Chain 1:  658 PSLTVDAVIVPCGNIADIADNGDANYYLMEAYKHLKPIALAGDARKFKATIKIADQGEEGIVEADSADGS
              1111111111111111111111111111111111111111111111111111111111111111111111
Chain 2:  658 PSLTVDAVIVPAGNIADIADNGDANYYLMEAYKHLKPIALAGDARKFKATIKIADQGEEGIVEADSADGS

Chain 1:  728 FMDELLTLMAAHRVWSRIPKIDKIPA
              11111111111111111111111111
Chain 2:  728 FMDELLTLMAAHRVWSRIPKIDKIPA
```

UniProt ID: P21179

PDB ID: 3PQ4_B

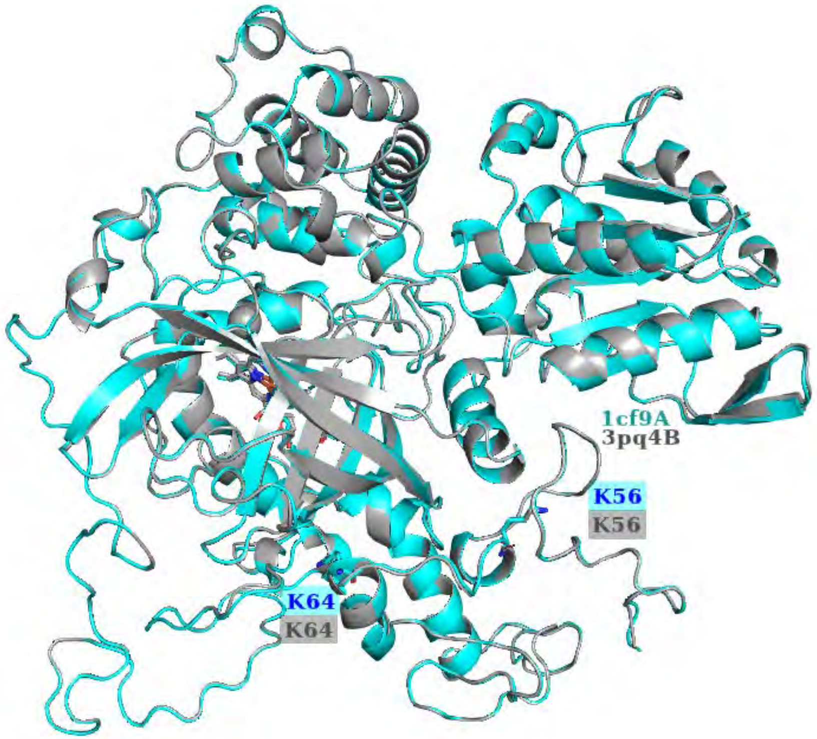


```
Align 1cf9.A.pdb 727 with 3pq4.B.pdb 726
Twists 0 ini-len 720 ini-rmsd 0.21 opt-equ 726 opt-rmsd 0.23 chain-rmsd 0.21 Score 2157.78 align-len 726 gaps 0 (0.00%)
P-value 0.00e+00 Afp-num 166882 Identity 99.45% Similarity 99.45%
Block  0 afp 90 score 2157.78 rmsd  0.21 gap 0 (0.00%)

                    .    :    .    :    .    :    .    :    .    :    .    :    .    :
Chain 1:   28 SLAPEDGSHRPAAEPTPPGAQPTAPGSLKAPDTRNEKLNSLEDVRKGSENYALTTNQGVRIADDQNSLRA
              1111111111111111111111111111111111111111111111111111111111111111111111
Chain 2:   28 SLAPEDGSHRPAAEPTPPGAQPTAPGSLKAPDTRNEKLNSLEDVRKGSENYALTTNQGVRIADDQNSLRA

                    .    :    .    :    .    :    .    :    .    :    .    :    .    :
Chain 1:   98 GSRGPTLLEDFILREKITHFDHERIPERIVHARGSAAHGYFQPYKSLSDITKADFLSDPNKITPVFVRFS
              1111111111111111111111111111111111111111111111111111111111111111111111
Chain 2:   98 GSRGPTLLEDFILREKITHFDHERIPERIVHARGSAAHGYFQPYKSLSDITKADFLSDPNKITPVFVRFS

                    .    :    .    :    .    :    .    :    .    :    .    :    .    :
Chain 1:  168 TCQGGAGSADTVRDIRGFATKFYTEEGIFDLVGNNTPIFFIQDAHKFPDFVHAVKPEPHWAIPQGQSAHD
              1111111111111111111111111111111111111111111111111111111111111111111111
Chain 2:  168 TVQGGAGSADTVRDIRGFATKFYTEEGIFDLVGNNTPIFFIQDAHKFPDFVHAVKPEPHWAIPQGQSAHD

                    .    :    .    :    .    :    .    :    .    :    .    :    .    :
Chain 1:  238 TFWDYVSLQPETLHNVMWAMSDRGIPRSYRTMEGFGIHTFRLINAEGKATFVRFHWKPLAGKASLVWDEA
              1111111111111111111111111111111111111111111111111111111111111111111111
Chain 2:  238 TFWDYVSLQPETLHNVMWAMSDRGIPRSYRTMEGFGCHTFRLINAEGKATFVRFHWKPLAGKASLVWDEA

                    .    :    .    :    .    :    .    :    .    :    .    :    .    :
Chain 1:  308 QKLTGRDPDFHRRELWEAIEAGDFPEYELGFQLIPEEDEFKFDFDLLDPTKLIPEELVPVQRVGKMVLNR
              1111111111111111111111111111111111111111111111111111111111111111111111
Chain 2:  308 QKLTGRDPDFHRRELWEAIEAGDFPEYELGFQLIPEEDEFKFDFDLLDPTKLIPEELVPVQRVGKMVLNR

                    .    :    .    :    .    :    .    :    .    :    .    :    .    :
Chain 1:  378 NPDNFFAENEQAAFHPGHIVPGLDFTNDPLLQGRLFSYTDTQISRLGGPNFHEIPINRPTCPYHNFQRDG
              1111111111111111111111111111111111111111111111111111111111111111111111
Chain 2:  378 NPDNFFAENEQAAFHPGHIVPGLDFTNDPLLQGRLFSYTDTQISRLGGPNFHEIPINRPTAPYHNFQRDG

                    .    :    .    :    .    :    .    :    .    :    .    :    .    :
Chain 1:  448 MHRMGIDTNPANYEPNSINDNWPRETPPGPKRGGFESYQERVEGNKVRERSPSFGEYYSHPRLFWLSQTP
              1111111111111111111111111111111111111111111111111111111111111111111111
Chain 2:  448 MHRMGIDTNPANYEPNSINDNWPRETPPGPKRGGFESYQERVEGNKVRERSPSFGEYYSHPRLFWLSQTP

                    .    :    .    :    .    :    .    :    .    :    .    :    .    :
Chain 1:  518 FEQRHIVDGFSFELSKVVRPYIRERVVDQLAHIDLTLAQAVAKNLGIELTDDQLNITPPPDVNGLKKDPS
              1111111111111111111111111111111111111111111111111111111111111111111111
Chain 2:  518 FEQRHIVDGFSFELSKVVRPYIRERVVDQLAHIDLTLAQAVAKNLGIELTDDQLNITPPPDVNGLKKDPS

                    .    :    .    :    .    :    .    :    .    :    .    :    .    :
Chain 1:  588 LSLYAIPDGDVKGRVVAILLNDEVRSADLLAILKALKAKGVHAKLLYSRMGEVTADDGTVLPIAATFAGA
              1111111111111111111111111111111111111111111111111111111111111111111111
Chain 2:  588 LSLYAIPDGDVKGRVVAILLNDEVRSADLLAILKALKAKGVHAKLLYSRMGEVTADDGTVLPIAATFAGA

                    .    :    .    :    .    :    .    :    .    :    .    :    .    :
Chain 1:  658 PSLTVDAVIVPCGNIADIADNGDANYYLMEAYKHLKPIALAGDARKFKATIKIADQGEEGIVEADSADGS
              1111111111111111111111111111111111111111111111111111111111111111111111
Chain 2:  658 PSLTVDAVIVPAGNIADIADNGDANYYLMEAYKHLKPIALAGDARKFKATIKIADQGEEGIVEADSADGS

                    .    :    .    :    .
Chain 1:  728 FMDELLTLMAAHRVWSRIPKIDKIPA
              11111111111111111111111111
Chain 2:  728 FMDELLTLMAAHRVWSRIPKIDKIPA
```

UniProt ID: P21179
PDB ID: 3PQ5_B

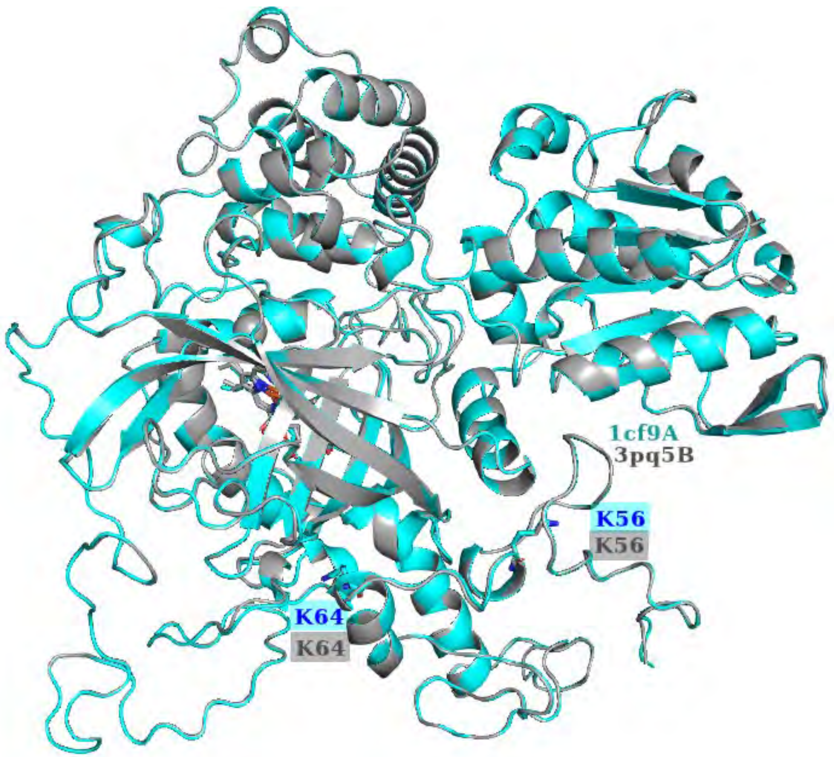


```
Align 1cf9.A.pdb 727 with 3pq5.B.pdb 726
Twists 0 ini-len 720 ini-rmsd 0.21 opt-equ 726 opt-rmsd 0.23 chain-rmsd 0.21 Score 2157.83 align-len 726 gaps 0 (0.00%)
P-value 0.00e+00 Afp-num 166928 Identity 99.45% Similarity 99.45%
Block  0 afp 90 score 2157.83 rmsd  0.21 gap 0 (0.00%)

                   .    :    .    :    .    :    .    :    .    :    .    :    .    :
Chain 1:   28 SLAPEDGSHRPAAEPTPPGAQPTAPGSLKAPDTRNEKLNSLEDVRKGSENYALTTNQGVRIADDQNSLRA
              1111111111111111111111111111111111111111111111111111111111111111111111
Chain 2:   28 SLAPEDGSHRPAAEPTPPGAQPTAPGSLKAPDTRNEKLNSLEDVRKGSENYALTTNQGVRIADDQNSLRA

                   .    :    .    :    .    :    .    :    .    :    .    :    .    :
Chain 1:   98 GSRGPTLLEDFILREKITHFDHERIPERIVHARGSAAHGYFQPYKSLSDITKADFLSDPNKITPVFVRFS
              1111111111111111111111111111111111111111111111111111111111111111111111
Chain 2:   98 GSRGPTLLEDFILREKITHFDHERIPERIVHARGSAAHGYFQPYKSLSDITKADFLSDPNKITPVFVRFS

                   .    :    .    :    .    :    .    :    .    :    .    :    .    :
Chain 1:  168 TCQGGAGSADTVRDIRGFATKFYTEEGIFDLVGNNTPIFFIQDAHKFPDFVHAVKPEPHWAIPQGQSAHD
              1111111111111111111111111111111111111111111111111111111111111111111111
Chain 2:  168 TVQGGAGSADTVRDIRGFATKFYTEEGIFDLVGNNTPIFFIQDAHKFPDFVHAVKPEPHWAIPQGQSAHD

                   .    :    .    :    .    :    .    :    .    :    .    :    .    :
Chain 1:  238 TFWDYVSLQPETLHNVMWAMSDRGIPRSYRTMEGFGIHTFRLINAEGKATFVRFHWKPLAGKASLVWDEA
              1111111111111111111111111111111111111111111111111111111111111111111111
Chain 2:  238 TFWDYVSLQPETLHNVMWAMSDRGIPRSYRTMEGFGCHTFRLINAEGKATFVRFHWKPLAGKASLVWDEA

                   .    :    .    :    .    :    .    :    .    :    .    :    .    :
Chain 1:  308 QKLTGRDPDFHRRELWEAIEAGDFPEYELGFQLIPEEDEFKFDFDLLDPTKLIPEELVPVQRVGKMVLNR
              1111111111111111111111111111111111111111111111111111111111111111111111
Chain 2:  308 QKLTGRDPDFHRRELWEAIEAGDFPEYELGFQLIPEEDEFKFDFDLLDPTKLIPEELVPVQRVGKMVLNR

                   .    :    .    :    .    :    .    :    .    :    .    :    .    :
Chain 1:  378 NPDNFFAENEQAAFHPGHIVPGLDFTNDPLLQGRLFSYTDTQISRLGGPNFHEIPINRPTCPYHNFQRDG
              1111111111111111111111111111111111111111111111111111111111111111111111
Chain 2:  378 NPDNFFAENEQAAFHPGHIVPGLDFTNDPLLQGRLFSYTDTQISRLGGPNFHEIPINRPTAPYHNFQRDG

                   .    :    .    :    .    :    .    :    .    :    .    :    .    :
Chain 1:  448 MHRMGIDTNPANYEPNSINDNWPRETPPGPKRGGFESYQERVEGNKVRERSPSFGEYYSHPRLFWLSQTP
              1111111111111111111111111111111111111111111111111111111111111111111111
Chain 2:  448 MHRMGIDTNPANYEPNSINDNWPRETPPGPKRGGFESYQERVEGNKVRERSPSFGEYYSHPRLFWLSQTP

                   .    :    .    :    .    :    .    :    .    :    .    :    .    :
Chain 1:  518 FEQRHIVDGFSFELSKVVRPYIRERVVDQLAHIDLTLAQAVAKNLGIELTDDQLNITPPPDVNGLKKDPS
              1111111111111111111111111111111111111111111111111111111111111111111111
Chain 2:  518 FEQRHIVDGFSFELSKVVRPYIRERVVDQLAHIDLTLAQAVAKNLGIELTDDQLNITPPPDVNGLKKDPS

                   .    :    .    :    .    :    .    :    .    :    .    :    .    :
Chain 1:  588 LSLYAIPDGDVKGRVVAILLNDEVRSADLLAILKALKAKGVHAKLLYSRMGEVTADDGTVLPIAATFAGA
              1111111111111111111111111111111111111111111111111111111111111111111111
Chain 2:  588 LSLYAIPDGDVKGRVVAILLNDEVRSADLLAILKALKAKGVHAKLLYSRMGEVTADDGTVLPIAATFAGA

                   .    :    .    :    .    :    .    :    .    :    .    :    .    :
Chain 1:  658 PSLTVDAVIVPCGNIADIADNGDANYYLMEAYKHLKPIALAGDARKFKATIKIADQGEEGIVEADSADGS
              1111111111111111111111111111111111111111111111111111111111111111111111
Chain 2:  658 PSLTVDAVIVPAGNIADIADNGDANYYLMEAYKHLKPIALAGDARKFKATIKIADQGEEGIVEADSADGS

                   .    :    .    :    .
Chain 1:  728 FMDELLTLMAAHRVWSRIPKIDKIPA
              11111111111111111111111111
Chain 2:  728 FMDELLTLMAAHRVWSRIPKIDKIPA
```

UniProt ID: P21179

PDB ID: 3PQ6_B

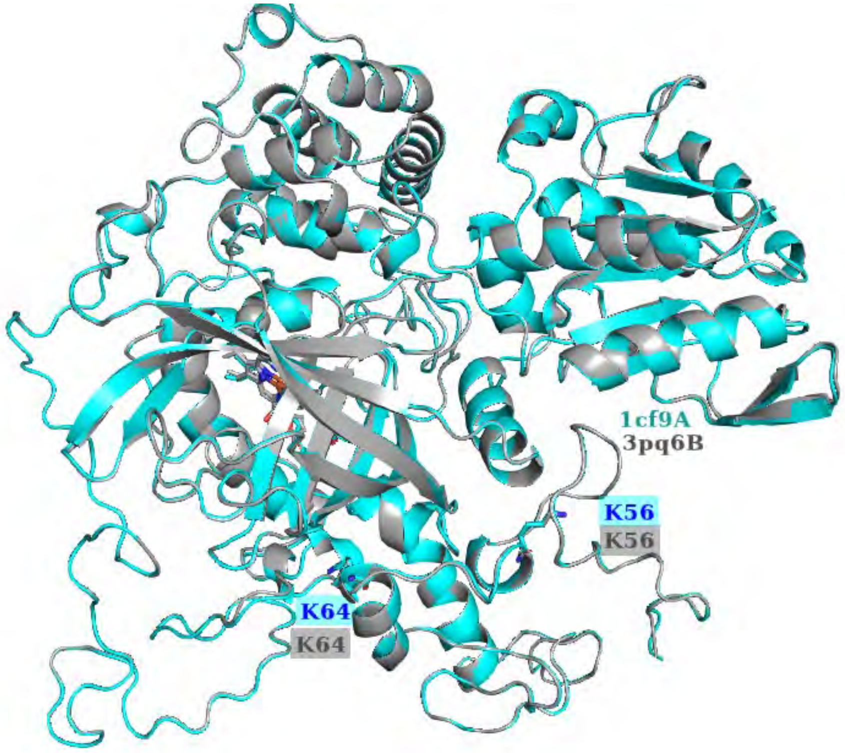

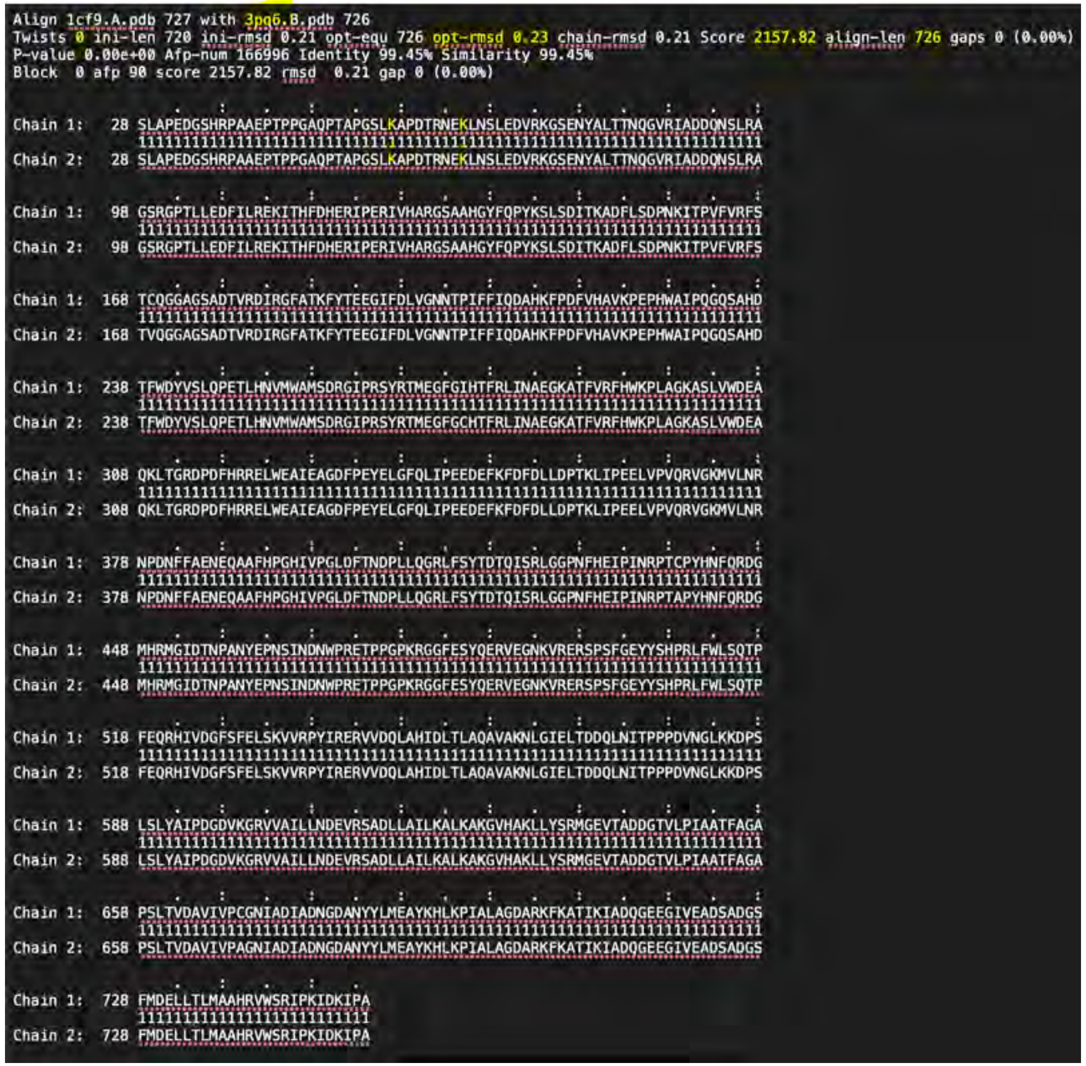

```
Align 1cf9.A.pdb 727 with 3pq6.B.pdb 726
Twists 0 ini-len 720 ini-rmsd 0.21 opt-equ 726 opt-rmsd 0.23 chain-rmsd 0.21 Score 2157.82 align-len 726 gaps 0 (0.00%)
P-value 0.00e+00 Afp-num 166996 Identity 99.45% Similarity 99.45%
Block  0 afp 90 score 2157.82 rmsd  0.21 gap 0 (0.00%)

Chain 1:   28 SLAPEDGSHRPAAEPTPPGAQPTAPGSLKAPDTRNEKLNSLEDVRKGSENYALTTNQGVRIADDQNSLRA
              1111111111111111111111111111111111111111111111111111111111111111111111
Chain 2:   28 SLAPEDGSHRPAAEPTPPGAQPTAPGSLKAPDTRNEKLNSLEDVRKGSENYALTTNQGVRIADDQNSLRA

Chain 1:   98 GSRGPTLLEDFILREKITHFDHERIPERIVHARGSAAHGYFQPYKSLSDITKADFLSDPNKITPVFVRFS
              1111111111111111111111111111111111111111111111111111111111111111111111
Chain 2:   98 GSRGPTLLEDFILREKITHFDHERIPERIVHARGSAAHGYFQPYKSLSDITKADFLSDPNKITPVFVRFS

Chain 1:  168 TCQGGAGSADTVRDIRGFATKFYTEEGIFDLVGNNTPIFFIQDAHKFPDFVHAVKPEPHWAIPQGQSAHD
              1111111111111111111111111111111111111111111111111111111111111111111111
Chain 2:  168 TVQGGAGSADTVRDIRGFATKFYTEEGIFDLVGNNTPIFFIQDAHKFPDFVHAVKPEPHWAIPQGQSAHD

Chain 1:  238 TFWDYVSLQPETLHNVMWAMSDRGIPRSYRTMEGFGIHTFRLINAEGKATFVRFHWKPLAGKASLVWDEA
              1111111111111111111111111111111111111111111111111111111111111111111111
Chain 2:  238 TFWDYVSLQPETLHNVMWAMSDRGIPRSYRTMEGFGCHTFRLINAEGKATFVRFHWKPLAGKASLVWDEA

Chain 1:  308 QKLTGRDPDFHRRELWEAIEAGDFPEYELGFQLIPEEDEFKFDFDLLDPTKLIPEELVPVQRVGKMVLNR
              1111111111111111111111111111111111111111111111111111111111111111111111
Chain 2:  308 QKLTGRDPDFHRRELWEAIEAGDFPEYELGFQLIPEEDEFKFDFDLLDPTKLIPEELVPVQRVGKMVLNR

Chain 1:  378 NPDNFFAENEQAAFHPGHIVPGLDFTNDPLLQGRLFSYTDTQISRLGGPNFHEIPINRPTCPYHNFQRDG
              1111111111111111111111111111111111111111111111111111111111111111111111
Chain 2:  378 NPDNFFAENEQAAFHPGHIVPGLDFTNDPLLQGRLFSYTDTQISRLGGPNFHEIPINRPTAPYHNFQRDG

Chain 1:  448 MHRMGIDTNPANYEPNSINDNWPRETPPGPKRGGFESYQERVEGNKVRERSPSFGEYYSHPRLFWLSQTP
              1111111111111111111111111111111111111111111111111111111111111111111111
Chain 2:  448 MHRMGIDTNPANYEPNSINDNWPRETPPGPKRGGFESYQERVEGNKVRERSPSFGEYYSHPRLFWLSQTP

Chain 1:  518 FEQRHIVDGFSFELSKVVRPYIRERVVDQLAHIDLTLAQAVAKNLGIELTDDQLNITPPPDVNGLKKDPS
              1111111111111111111111111111111111111111111111111111111111111111111111
Chain 2:  518 FEQRHIVDGFSFELSKVVRPYIRERVVDQLAHIDLTLAQAVAKNLGIELTDDQLNITPPPDVNGLKKDPS

Chain 1:  588 LSLYAIPDGDVKGRVVAILLNDEVRSADLLAILKALKAKGVHAKLLYSRMGEVTADDGTVLPIAATFAGA
              1111111111111111111111111111111111111111111111111111111111111111111111
Chain 2:  588 LSLYAIPDGDVKGRVVAILLNDEVRSADLLAILKALKAKGVHAKLLYSRMGEVTADDGTVLPIAATFAGA

Chain 1:  658 PSLTVDAVIVPCGNIADIADNGDANYYLMEAYKHLKPIALAGDARKFKATIKIADQGEEGIVEADSADGS
              1111111111111111111111111111111111111111111111111111111111111111111111
Chain 2:  658 PSLTVDAVIVPAGNIADIADNGDANYYLMEAYKHLKPIALAGDARKFKATIKIADQGEEGIVEADSADGS

Chain 1:  728 FMDELLTLMAAHRVWSRIPKIDKIPA
              11111111111111111111111111
Chain 2:  728 FMDELLTLMAAHRVWSRIPKIDKIPA
```

UniProt ID: P21179

PDB ID: 3PQ7_B

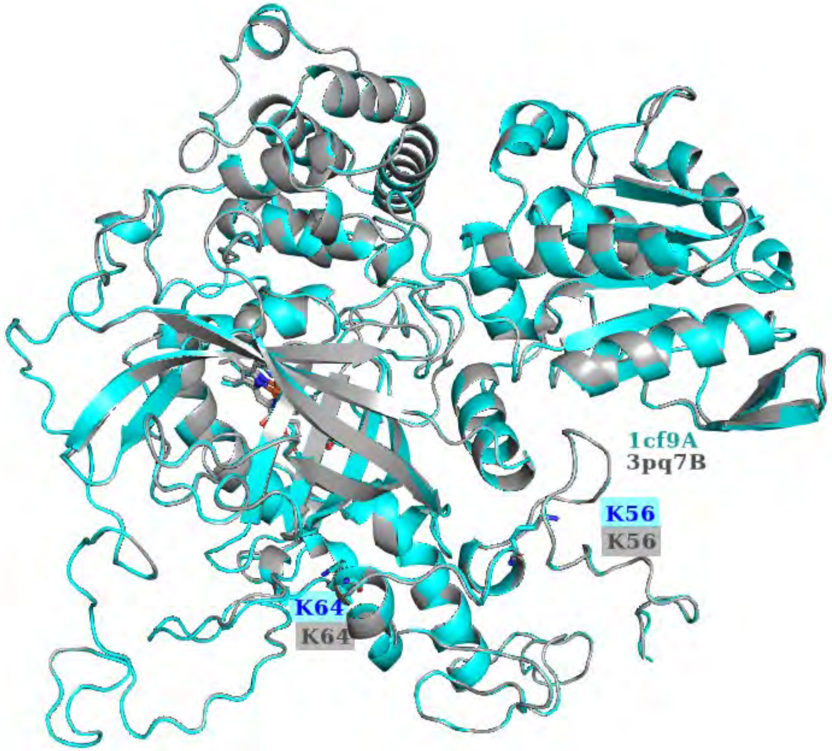


```
Align 1cf9.A.pdb 727 with 3pq7.B.pdb 726
Twists 0 ini-len 720 ini-rmsd 0.21 opt-equ 726 opt-rmsd 0.23 chain-rmsd 0.21 Score 2157.86 align-len 726 gaps 0 (0.00%)
P-value 0.00e+00 Afp-num 167015 Identity 99.45% Similarity 99.45%
Block  0 afp 90 score 2157.86 rmsd  0.21 gap 0 (0.00%)

                   .    :    .    :    .    :    .    :    .    :    .    :    .    :
Chain 1:   28 SLAPEDGSHRPAAEPTPPGAQPTAPGSLKAPDTRNEKLNSLEDVRKGSENYALTTNQGVRIADDQNSLRA
              1111111111111111111111111111111111111111111111111111111111111111111111
Chain 2:   28 SLAPEDGSHRPAAEPTPPGAQPTAPGSLKAPDTRNEKLNSLEDVRKGSENYALTTNQGVRIADDQNSLRA

                   .    :    .    :    .    :    .    :    .    :    .    :    .    :
Chain 1:   98 GSRGPTLLEDFILREKITHFDHERIPERIVHARGSAAHGYFQPYKSLSDITKADFLSDPNKITPVFVRFS
              1111111111111111111111111111111111111111111111111111111111111111111111
Chain 2:   98 GSRGPTLLEDFILREKITHFDHERIPERIVHARGSAAHGYFQPYKSLSDITKADFLSDPNKITPVFVRFS

                   .    :    .    :    .    :    .    :    .    :    .    :    .    :
Chain 1:  168 TCQGGAGSADTVRDIRGFATKFYTEEGIFDLVGNNTPIFFIQDAHKFPDFVHAVKPEPHWAIPQGQSAHD
              1111111111111111111111111111111111111111111111111111111111111111111111
Chain 2:  168 TVQGGAGSADTVRDIRGFATKFYTEEGIFDLVGNNTPIFFIQDAHKFPDFVHAVKPEPHWAIPQGQSAHD

                   .    :    .    :    .    :    .    :    .    :    .    :    .    :
Chain 1:  238 TFWDYVSLQPETLHNVMWAMSDRGIPRSYRTMEGFGIHTFRLINAEGKATFVRFHWKPLAGKASLVWDEA
              1111111111111111111111111111111111111111111111111111111111111111111111
Chain 2:  238 TFWDYVSLQPETLHNVMWAMSDRGIPRSYRTMEGFGCHTFRLINAEGKATFVRFHWKPLAGKASLVWDEA

                   .    :    .    :    .    :    .    :    .    :    .    :    .    :
Chain 1:  308 QKLTGRDPDFHRRELWEAIEAGDFPEYELGFQLIPEEDEFKFDFDLLDPTKLIPEELVPVQRVGKMVLNR
              1111111111111111111111111111111111111111111111111111111111111111111111
Chain 2:  308 QKLTGRDPDFHRRELWEAIEAGDFPEYELGFQLIPEEDEFKFDFDLLDPTKLIPEELVPVQRVGKMVLNR

                   .    :    .    :    .    :    .    :    .    :    .    :    .    :
Chain 1:  378 NPDNFFAENEQAAFHPGHIVPGLDFTNDPLLQGRLFSYTDTQISRLGGPNFHEIPINRPTCPYHNFQRDG
              1111111111111111111111111111111111111111111111111111111111111111111111
Chain 2:  378 NPDNFFAENEQAAFHPGHIVPGLDFTNDPLLQGRLFSYTDTQISRLGGPNFHEIPINRPTAPYHNFQRDG

                   .    :    .    :    .    :    .    :    .    :    .    :    .    :
Chain 1:  448 MHRMGIDTNPANYEPNSINDNWPRETPPGPKRGGFESYQERVEGNKVRERSPSFGEYYSHPRLFWLSQTP
              1111111111111111111111111111111111111111111111111111111111111111111111
Chain 2:  448 MHRMGIDTNPANYEPNSINDNWPRETPPGPKRGGFESYQERVEGNKVRERSPSFGEYYSHPRLFWLSQTP

                   .    :    .    :    .    :    .    :    .    :    .    :    .    :
Chain 1:  518 FEQRHIVDGFSFELSKVVRPYIRERVVDQLAHIDLTLAQAVAKNLGIELTDDQLNITPPPDVNGLKKDPS
              1111111111111111111111111111111111111111111111111111111111111111111111
Chain 2:  518 FEQRHIVDGFSFELSKVVRPYIRERVVDQLAHIDLTLAQAVAKNLGIELTDDQLNITPPPDVNGLKKDPS

                   .    :    .    :    .    :    .    :    .    :    .    :    .    :
Chain 1:  588 LSLYAIPDGDVKGRVVAILLNDEVRSADLLAILKALKAKGVHAKLLYSRMGEVTADDGTVLPIAATFAGA
              1111111111111111111111111111111111111111111111111111111111111111111111
Chain 2:  588 LSLYAIPDGDVKGRVVAILLNDEVRSADLLAILKALKAKGVHAKLLYSRMGEVTADDGTVLPIAATFAGA

                   .    :    .    :    .    :    .    :    .    :    .    :    .    :
Chain 1:  658 PSLTVDAVIVPCGNIADIADNGDANYYLMEAYKHLKPIALAGDARKFKATIKIADQGEEGIVEADSADGS
              1111111111111111111111111111111111111111111111111111111111111111111111
Chain 2:  658 PSLTVDAVIVPAGNIADIADNGDANYYLMEAYKHLKPIALAGDARKFKATIKIADQGEEGIVEADSADGS

                   .    :    .    :    .
Chain 1:  728 FMDELLTLMAAHRVWSRIPKIDKIPA
              11111111111111111111111111
Chain 2:  728 FMDELLTLMAAHRVWSRIPKIDKIPA
```

UniProt ID: P21179

PDB ID: 3PQ8_B

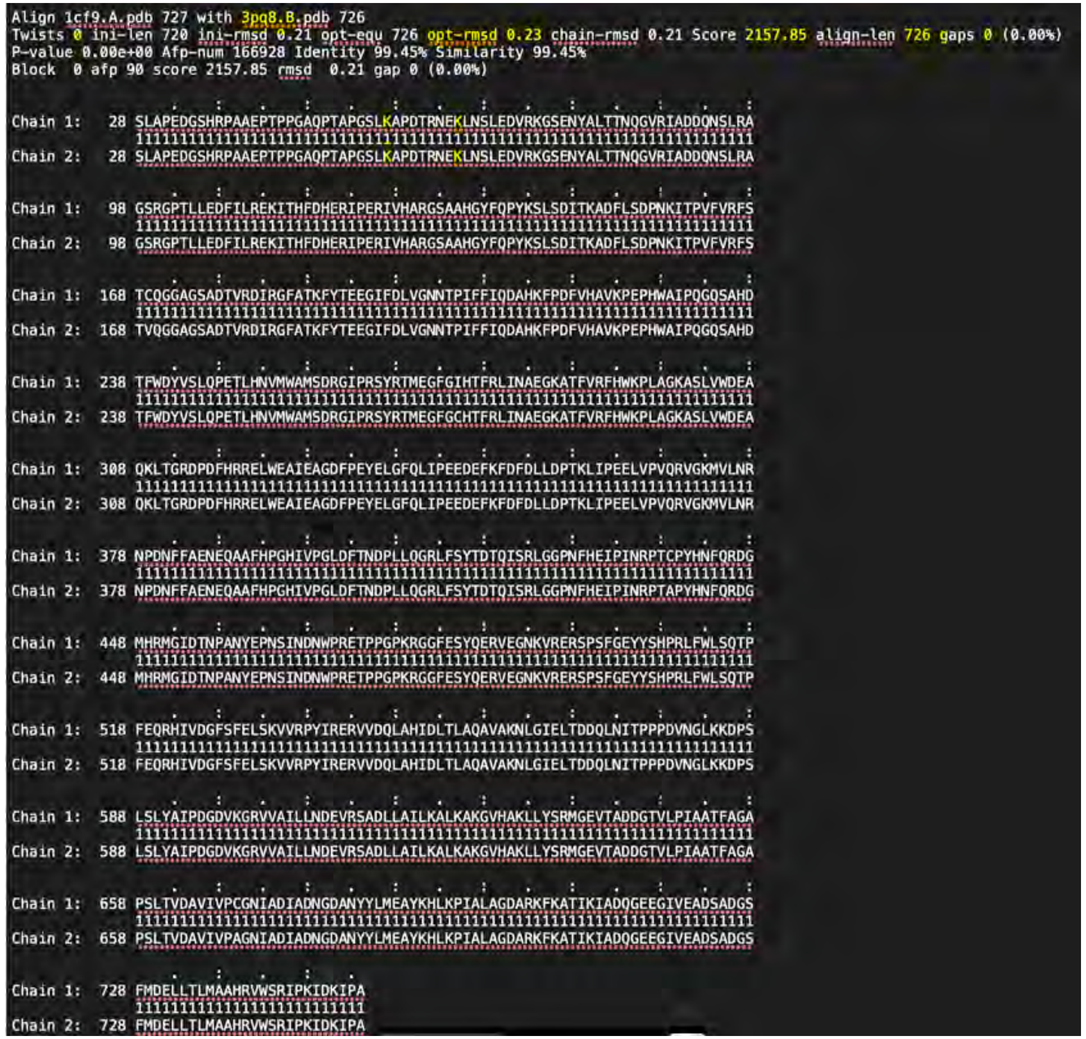

```
Align 1cf9.A.pdb 727 with 3pq8.B.pdb 726
Twists 0 ini-len 720 ini-rmsd 0.21 opt-equ 726 opt-rmsd 0.23 chain-rmsd 0.21 Score 2157.85 align-len 726 gaps 0 (0.00%)
P-value 0.00e+00 Afp-num 166928 Identity 99.45% Similarity 99.45%
Block  0 afp 90 score 2157.85 rmsd  0.21 gap 0 (0.00%)

Chain 1:   28 SLAPEDGSHRPAAEPTPPGAQPTAPGSLKAPDTRNEKLNSLEDVRKGSENYALTTNQGVRIADDQNSLRA
              1111111111111111111111111111111111111111111111111111111111111111111111
Chain 2:   28 SLAPEDGSHRPAAEPTPPGAQPTAPGSLKAPDTRNEKLNSLEDVRKGSENYALTTNQGVRIADDQNSLRA

Chain 1:   98 GSRGPTLLEDFILREKITHFDHERIPERIVHARGSAAHGYFQPYKSLSDITKADFLSDPNKITPVFVRFS
              1111111111111111111111111111111111111111111111111111111111111111111111
Chain 2:   98 GSRGPTLLEDFILREKITHFDHERIPERIVHARGSAAHGYFQPYKSLSDITKADFLSDPNKITPVFVRFS

Chain 1:  168 TCQGGAGSADTVRDIRGFATKFYTEEGIFDLVGNNTPIFFIQDAHKFPDFVHAVKPEPHWAIPQGQSAHD
              1111111111111111111111111111111111111111111111111111111111111111111111
Chain 2:  168 TVQGGAGSADTVRDIRGFATKFYTEEGIFDLVGNNTPIFFIQDAHKFPDFVHAVKPEPHWAIPQGQSAHD

Chain 1:  238 TFWDYVSLQPETLHNVMWAMSDRGIPRSYRTMEGFGIHTFRLINAEGKATFVRFHWKPLAGKASLVWDEA
              1111111111111111111111111111111111111111111111111111111111111111111111
Chain 2:  238 TFWDYVSLQPETLHNVMWAMSDRGIPRSYRTMEGFGCHTFRLINAEGKATFVRFHWKPLAGKASLVWDEA

Chain 1:  308 QKLTGRDPDFHRRELWEAIEAGDFPEYELGFQLIPEEDEFKFDFDLLDPTKLIPEELVPVQRVGKMVLNR
              1111111111111111111111111111111111111111111111111111111111111111111111
Chain 2:  308 QKLTGRDPDFHRRELWEAIEAGDFPEYELGFQLIPEEDEFKFDFDLLDPTKLIPEELVPVQRVGKMVLNR

Chain 1:  378 NPDNFFAENEQAAFHPGHIVPGLDFTNDPLLQGRLFSYTDTQISRLGGPNFHEIPINRPTCPYHNFQRDG
              1111111111111111111111111111111111111111111111111111111111111111111111
Chain 2:  378 NPDNFFAENEQAAFHPGHIVPGLDFTNDPLLQGRLFSYTDTQISRLGGPNFHEIPINRPTAPYHNFQRDG

Chain 1:  448 MHRMGIDTNPANYEPNSINDNWPRETPPGPKRGGFESYQERVEGNKVRERSPSFGEYYSHPRLFWLSQTP
              1111111111111111111111111111111111111111111111111111111111111111111111
Chain 2:  448 MHRMGIDTNPANYEPNSINDNWPRETPPGPKRGGFESYQERVEGNKVRERSPSFGEYYSHPRLFWLSQTP

Chain 1:  518 FEQRHIVDGFSFELSKVVRPYIRERVVDQLAHIDLTLAQAVAKNLGIELTDDQLNITPPPDVNGLKKDPS
              1111111111111111111111111111111111111111111111111111111111111111111111
Chain 2:  518 FEQRHIVDGFSFELSKVVRPYIRERVVDQLAHIDLTLAQAVAKNLGIELTDDQLNITPPPDVNGLKKDPS

Chain 1:  588 LSLYAIPDGDVKGRVVAILLNDEVRSADLLAILKALKAKGVHAKLLYSRMGEVTADDGTVLPIAATFAGA
              1111111111111111111111111111111111111111111111111111111111111111111111
Chain 2:  588 LSLYAIPDGDVKGRVVAILLNDEVRSADLLAILKALKAKGVHAKLLYSRMGEVTADDGTVLPIAATFAGA

Chain 1:  658 PSLTVDAVIVPCGNIADIADNGDANYYLMEAYKHLKPIALAGDARKFKATIKIADQGEEGIVEADSADGS
              1111111111111111111111111111111111111111111111111111111111111111111111
Chain 2:  658 PSLTVDAVIVPAGNIADIADNGDANYYLMEAYKHLKPIALAGDARKFKATIKIADQGEEGIVEADSADGS

Chain 1:  728 FMDELLTLMAAHRVWSRIPKIDKIPA
              11111111111111111111111111
Chain 2:  728 FMDELLTLMAAHRVWSRIPKIDKIPA
```

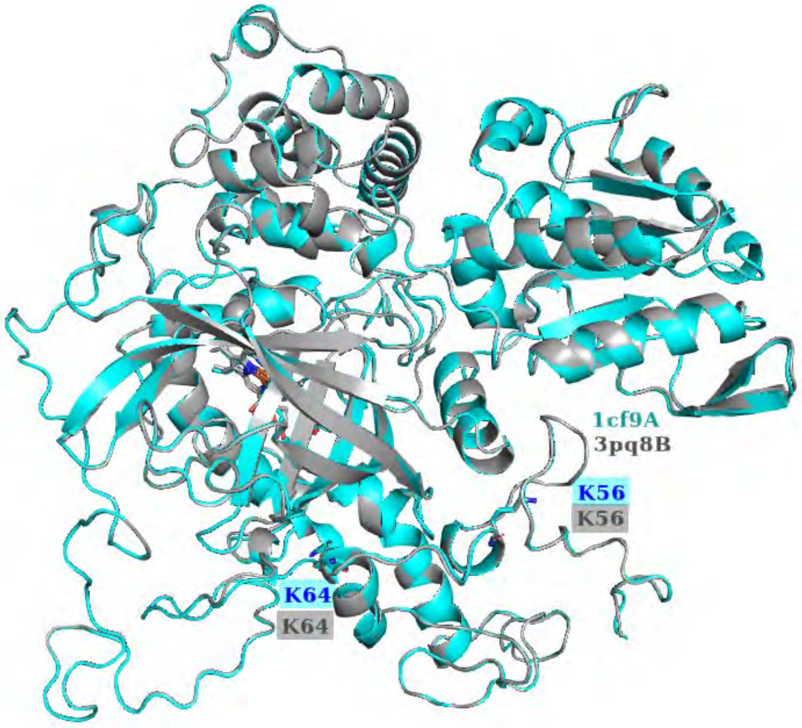

UniProt ID: P21179
PDB ID: 3TTT_B

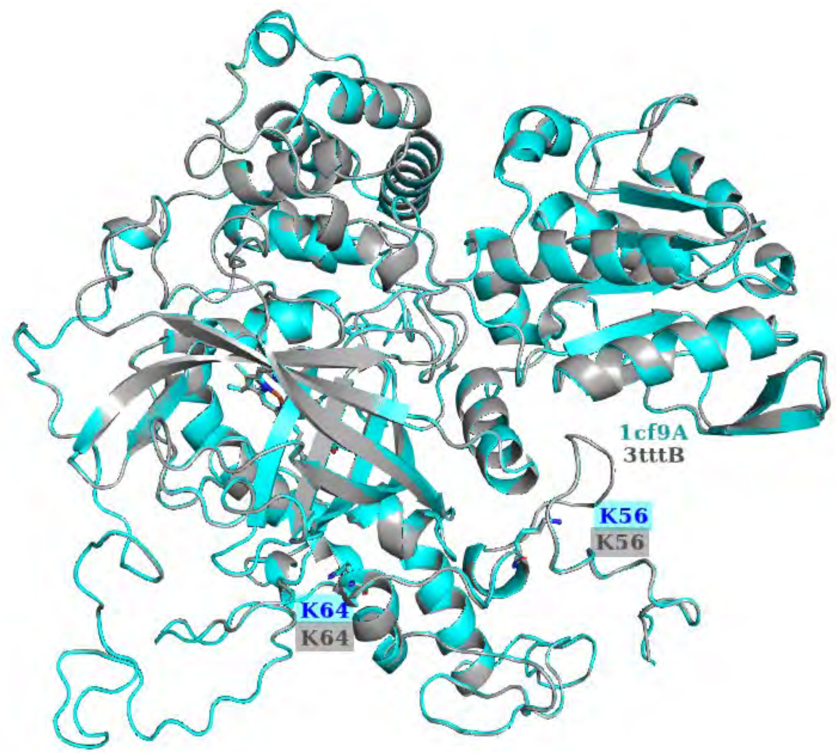


```
Align 1cf9.A.pdb 727 with 3ttt.B.pdb 726
Twists 0 ini-len 720 ini-rmsd 0.21 opt-equ 726 opt-rmsd 0.22 chain-rmsd 0.21 Score 2158.30 align-len 726 gaps 0 (0.00%)
P-value 0.00e+00 Afp-num 166988 Identity 99.72% Similarity 99.86%
Block  0 afp 90 score 2158.30 rmsd  0.21 gap 0 (0.00%)

                   .    :    .    :    .    :    .    :    .    :    .    :    .    :
Chain 1:    28 SLAPEDGSHRPAAEPTPPGAQPTAPGSLKAPDTRNEKLNSLEDVRKGSENYALTTNQGVRIADDQNSLRA
               1111111111111111111111111111111111111111111111111111111111111111111111
Chain 2:    28 SLAPEDGSHRPAAEPTPPGAQPTAPGSLKAPDTRNEKLNSLEDVRKGSENYALTTNQGVRIADDQNSLRA

                   .    :    .    :    .    :    .    :    .    :    .    :    .    :
Chain 1:    98 GSRGPTLLEDFILREKITHFDHERIPERIVHARGSAAHGYFQPYKSLSDITKADFLSDPNKITPVFVRFS
               1111111111111111111111111111111111111111111111111111111111111111111111
Chain 2:    98 GSRGPTLLEDFILREKITHFDHERIPERIVHARGSAAHGYFQPYKSLSDITKADFLSDPNKITPVFVRFS

                   .    :    .    :    .    :    .    :    .    :    .    :    .    :
Chain 1:   168 TCQGGAGSADTVRDIRGFATKFYTEEGIFDLVGNNTPIFFIQDAHKFPDFVHAVKPEPHWAIPQGQSAHD
               1111111111111111111111111111111111111111111111111111111111111111111111
Chain 2:   168 TVQGGAGSADTVRDIRGFATKFYTEEGIFDLVGNNTPIFFIQDAHKFPDFVHAVKPEPHWAIPQGQSAHD

                   .    :    .    :    .    :    .    :    .    :    .    :    .    :
Chain 1:   238 TFWDYVSLQPETLHNVMWAMSDRGIPRSYRTMEGFGIHTFRLINAEGKATFVRFHWKPLAGKASLVWDEA
               1111111111111111111111111111111111111111111111111111111111111111111111
Chain 2:   238 TFWDYVSLQPETLHNVMWAMSDRGIPRSYRTMEGFGIHTFRLINAEGKATFVRFHWKPLAGKASLVWDEA

                   .    :    .    :    .    :    .    :    .    :    .    :    .    :
Chain 1:   308 QKLTGRDPDFHRRELWEAIEAGDFPEYELGFQLIPEEDEFKFDFDLLDPTKLIPEELVPVQRVGKMVLNR
               1111111111111111111111111111111111111111111111111111111111111111111111
Chain 2:   308 QKLTGRDPDFHRRELWEAIEAGDFPEYELGFQLIPEEDEFKFDFDLLDPTKLIPEELVPVQRVGKMVLNR

                   .    :    .    :    .    :    .    :    .    :    .    :    .    :
Chain 1:   378 NPDNFFAENEQAAFHPGHIVPGLDFTNDPLLQGRLFSYTDTQISRLGGPNFHEIPINRPTCPYHNFQRDG
               1111111111111111111111111111111111111111111111111111111111111111111111
Chain 2:   378 NPDNFFAENEQAAFHPGHIVPGLDFTNDPLLQGRLYSYTDTQISRLGGPNFHEIPINRPTCPYHNFQRDG

                   .    :    .    :    .    :    .    :    .    :    .    :    .    :
Chain 1:   448 MHRMGIDTNPANYEPNSINDNWPRETPPGPKRGGFESYQERVEGNKVRERSPSFGEYYSHPRLFWLSQTP
               1111111111111111111111111111111111111111111111111111111111111111111111
Chain 2:   448 MHRMGIDTNPANYEPNSINDNWPRETPPGPKRGGFESYQERVEGNKVRERSPSFGEYYSHPRLFWLSQTP

                   .    :    .    :    .    :    .    :    .    :    .    :    .    :
Chain 1:   518 FEQRHIVDGFSFELSKVVRPYIRERVVDQLAHIDLTLAQAVAKNLGIELTDDQLNITPPPDVNGLKKDPS
               1111111111111111111111111111111111111111111111111111111111111111111111
Chain 2:   518 FEQRHIVDGFSFELSKVVRPYIRERVVDQLAHIDLTLAQAVAKNLGIELTDDQLNITPPPDVNGLKKDPS

                   .    :    .    :    .    :    .    :    .    :    .    :    .    :
Chain 1:   588 LSLYAIPDGDVKGRVVAILLNDEVRSADLLAILKALKAKGVHAKLLYSRMGEVTADDGTVLPIAATFAGA
               1111111111111111111111111111111111111111111111111111111111111111111111
Chain 2:   588 LSLYAIPDGDVKGRVVAILLNDEVRSADLLAILKALKAKGVHAKLLYSRMGEVTADDGTVLPIAATFAGA

                   .    :    .    :    .    :    .    :    .    :    .    :    .    :
Chain 1:   658 PSLTVDAVIVPCGNIADIADNGDANYYLMEAYKHLKPIALAGDARKFKATIKIADQGEEGIVEADSADGS
               1111111111111111111111111111111111111111111111111111111111111111111111
Chain 2:   658 PSLTVDAVIVPCGNIADIADNGDANYYLMEAYKHLKPIALAGDARKFKATIKIADQGEEGIVEADSADGS

                   .    :    .    :    .
Chain 1:   728 FMDELLTLMAAHRVWSRIPKIDKIPA
               1111111111111111111111111
Chain 2:   728 FMDELLTLMAAHRVWSRIPKIDKIPA
```

UniProt ID: P21179
PDB ID: 3TTU_B

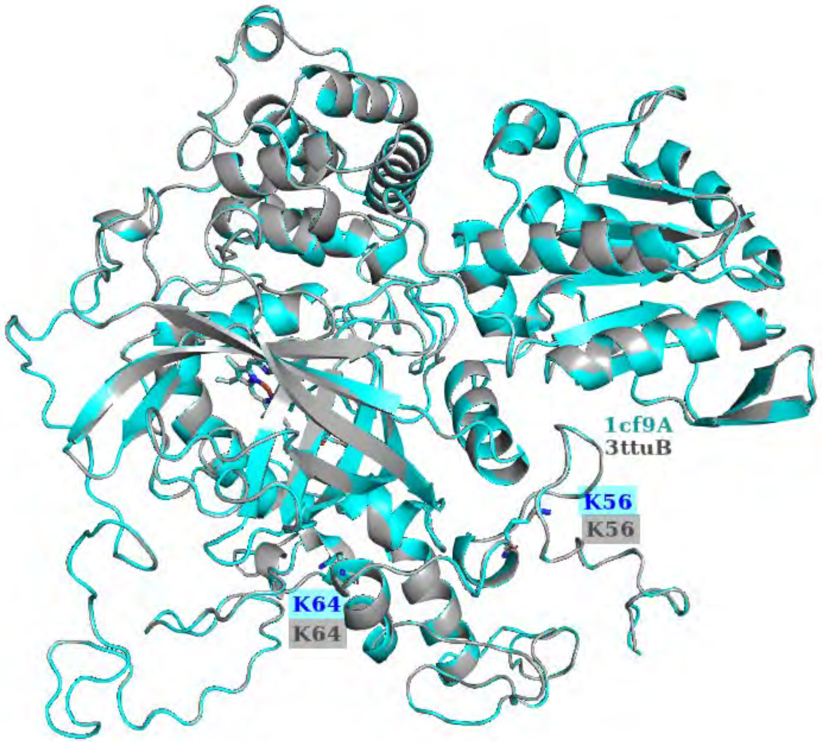


```
Align 1cf9.A.pdb 727 with 3ttu.B.pdb 726
Twists 0 ini-len 720 ini-rmsd 0.17 opt-equ 726 opt-rmsd 0.20 chain-rmsd 0.17 Score 2158.32 align-len 726 gaps 0 (0.00%)
P-value 0.00e+00 Afp-num 166670 Identity 99.59% Similarity 99.86%
Block  0 afp 90 score 2158.32 rmsd  0.17 gap 0 (0.00%)

                  .    :    .    :    .    :    .    :    .    :    .    :    .    :
Chain 1:   28 SLAPEDGSHRPAAEPTPPGAQPTAPGSLKAPDTRNEKLNSLEDVRKGSENYALTTNQGVRIADDQNSLRA
              1111111111111111111111111111111111111111111111111111111111111111111111
Chain 2:   28 SLAPEDGSHRPAAEPTPPGAQPTAPGSLKAPDTRNEKLNSLEDVRKGSENYALTTNQGVRIADDQNSLRA

                  .    :    .    :    .    :    .    :    .    :    .    :    .    :
Chain 1:   98 GSRGPTLLEDFILREKITHFDHERIPERIVHARGSAAHGYFQPYKSLSDITKADFLSDPNKITPVFVRFS
              1111111111111111111111111111111111111111111111111111111111111111111111
Chain 2:   98 GSRGPTLLEDFILREKITHFDHERIPERIVNARGSAAHGYFQPYKSLSDITKADFLSDPNKITPVFVRFS

                  .    :    .    :    .    :    .    :    .    :    .    :    .    :
Chain 1:  168 TCQGGAGSADTVRDIRGFATKFYTEEGIFDLVGNNTPIFFIQDAHKFPDFVHAVKPEPHWAIPQGQSAHD
              1111111111111111111111111111111111111111111111111111111111111111111111
Chain 2:  168 TVQGGAGSADTVRDIRGFATKFYTEEGIFDLVGNNTPIFFIQDAHKFPDFVHAVKPEPHWAIPQGQSAHD

                  .    :    .    :    .    :    .    :    .    :    .    :    .    :
Chain 1:  238 TFWDYVSLQPETLHNVMWAMSDRGIPRSYRTMEGFGIHTFRLINAEGKATFVRFHWKPLAGKASLVWDEA
              1111111111111111111111111111111111111111111111111111111111111111111111
Chain 2:  238 TFWDYVSLQPETLHNVMWAMSDRGIPRSYRTMEGFGIHTFRLINAEGKATFVRFHWKPLAGKASLVWDEA

                  .    :    .    :    .    :    .    :    .    :    .    :    .    :
Chain 1:  308 QKLTGRDPDFHRRELWEAIEAGDFPEYELGFQLIPEEDEFKFDFDLLDPTKLIPEELVPVQRVGKMVLNR
              1111111111111111111111111111111111111111111111111111111111111111111111
Chain 2:  308 QKLTGRDPDFHRRELWEAIEAGDFPEYELGFQLIPEEDEFKFDFDLLDPTKLIPEELVPVQRVGKMVLNR

                  .    :    .    :    .    :    .    :    .    :    .    :    .    :
Chain 1:  378 NPDNFFAENEQAAFHPGHIVPGLDFTNDPLLQGRLFSYTDTQISRLGGPNFHEIPINRPTCPYHNFQRDG
              1111111111111111111111111111111111111111111111111111111111111111111111
Chain 2:  378 NPDNFFAENEQAAFHPGHIVPGLDFTNDPLLQGRLYSYTDTQISRLGGPNFHEIPINRPTCPYHNFQRDG

                  .    :    .    :    .    :    .    :    .    :    .    :    .    :
Chain 1:  448 MHRMGIDTNPANYEPNSINDNWPRETPPGPKRGGFESYQERVEGNKVRERSPSFGEYYSHPRLFWLSQTP
              1111111111111111111111111111111111111111111111111111111111111111111111
Chain 2:  448 MHRMGIDTNPANYEPNSINDNWPRETPPGPKRGGFESYQERVEGNKVRERSPSFGEYYSHPRLFWLSQTP

                  .    :    .    :    .    :    .    :    .    :    .    :    .    :
Chain 1:  518 FEQRHIVDGFSFELSKVVRPYIRERVVDQLAHIDLTLAQAVAKNLGIELTDDQLNITPPPDVNGLKKDPS
              1111111111111111111111111111111111111111111111111111111111111111111111
Chain 2:  518 FEQRHIVDGFSFELSKVVRPYIRERVVDQLAHIDLTLAQAVAKNLGIELTDDQLNITPPPDVNGLKKDPS

                  .    :    .    :    .    :    .    :    .    :    .    :    .    :
Chain 1:  588 LSLYAIPDGDVKGRVVAILLNDEVRSADLLAILKALKAKGVHAKLLYSRMGEVTADDGTVLPIAATFAGA
              1111111111111111111111111111111111111111111111111111111111111111111111
Chain 2:  588 LSLYAIPDGDVKGRVVAILLNDEVRSADLLAILKALKAKGVHAKLLYSRMGEVTADDGTVLPIAATFAGA

                  .    :    .    :    .    :    .    :    .    :    .    :    .    :
Chain 1:  658 PSLTVDAVIVPCGNIADIADNGDANYYLMEAYKHLKPIALAGDARKFKATIKIADQGEEGIVEADSADGS
              1111111111111111111111111111111111111111111111111111111111111111111111
Chain 2:  658 PSLTVDAVIVPCGNIADIADNGDANYYLMEAYKHLKPIALAGDARKFKATIKIADQGEEGIVEADSADGS

                  .    :    .    :    .
Chain 1:  728 FMDELLTLMAAHRVWSRIPKIDKIPA
              11111111111111111111111111
Chain 2:  728 FMDELLTLMAAHRVWSRIPKIDKIPA
```

UniProt ID: P21179

PDB ID: 3TTV_B

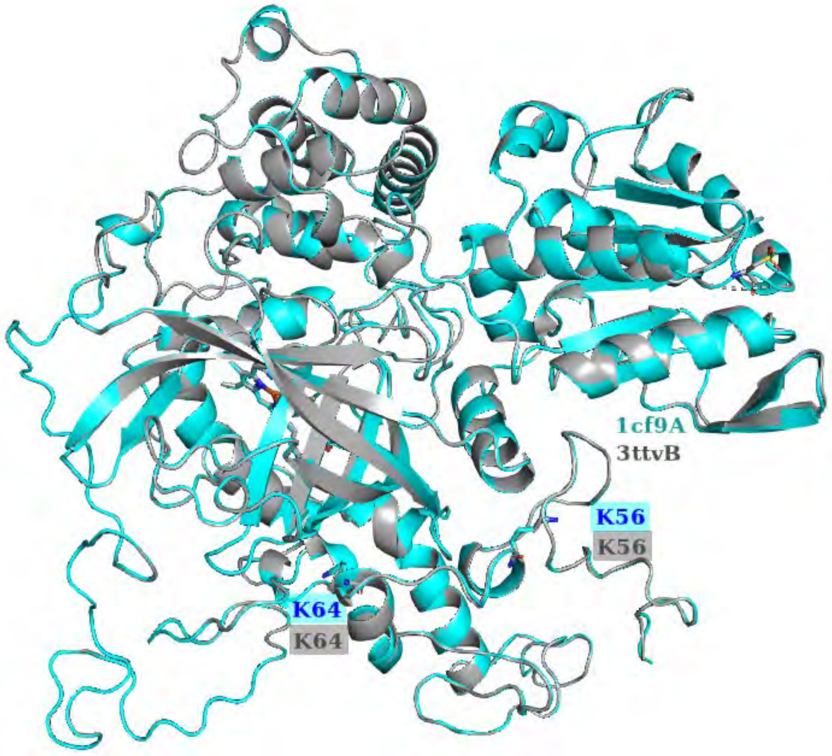


```
Align 1cf9.A.pdb 727 with 3ttv.B.pdb 726
Twists 0 ini-len 720 ini-rmsd 0.19 opt-equ 726 opt-rmsd 0.21 chain-rmsd 0.19 Score 2158.32 align-len 726 gaps 0 (0.00%)
P-value 0.00e+00 Afp-num 167030 Identity 99.45% Similarity 99.59%
Block  0 afp 90 score 2158.32 rmsd  0.19 gap 0 (0.00%)

                  .    :    .    :    .    :    .    :    .    :    .    :    .    :
Chain 1:   28 SLAPEDGSHRPAAEPTPPGAQPTAPGSLKAPDTRNEKLNSLEDVRKGSENYALTTNQGVRIADDQNSLRA
              1111111111111111111111111111111111111111111111111111111111111111111111
Chain 2:   28 SLAPEDGSHRPAAEPTPPGAQPTAPGSLKAPDTRNEKLNSLEDVRKGSENYALTTNQGVRIADDQNSLRA

                  .    :    .    :    .    :    .    :    .    :    .    :    .    :
Chain 1:   98 GSRGPTLLEDFILREKITHFDHERIPERIVHARGSAAHGYFQPYKSLSDITKADFLSDPNKITPVFVRFS
              1111111111111111111111111111111111111111111111111111111111111111111111
Chain 2:   98 GSRGPTLLEDFILREKIAHFDHERIPERIVHARGSAAHGYFQPYKSLSDITKADFLSDPNKITPVFVRFS

                  .    :    .    :    .    :    .    :    .    :    .    :    .    :
Chain 1:  168 TCQGGAGSADTVRDIRGFATKFYTEEGIFDLVGNNTPIFFIQDAHKFPDFVHAVKPEPHWAIPQGQSAHD
              1111111111111111111111111111111111111111111111111111111111111111111111
Chain 2:  168 TVQGGAGSADTVRDIRGFATKFYTEEGIFDLVGNNTPIFFIQDAHKFPDFVHAVKPEPHWAIPQGQSAHD

                  .    :    .    :    .    :    .    :    .    :    .    :    .    :
Chain 1:  238 TFWDYVSLQPETLHNVMWAMSDRGIPRSYRTMEGFGIHTFRLINAEGKATFVRFHWKPLAGKASLVWDEA
              1111111111111111111111111111111111111111111111111111111111111111111111
Chain 2:  238 TFWDYVSLQPETLHNVMWAMSDRGIPRSYRTMEGFGIHTFRLINAEGKATFVRFHWKPLAGKASLVWDEA

                  .    :    .    :    .    :    .    :    .    :    .    :    .    :
Chain 1:  308 QKLTGRDPDFHRRELWEAIEAGDFPEYELGFQLIPEEDEFKFDFDLLDPTKLIPEELVPVQRVGKMVLNR
              1111111111111111111111111111111111111111111111111111111111111111111111
Chain 2:  308 QKLTGRDPDFHRRELWEAIEAGDFPEYELGFQLIPEEDEFKFDFDLLDPTKLIPEELVPVQRVGKMVLNR

                  .    :    .    :    .    :    .    :    .    :    .    :    .    :
Chain 1:  378 NPDNFFAENEQAAFHPGHIVPGLDFTNDPLLQGRLFSYTDTQISRLGGPNFHEIPINRPTCPYHNFQRDG
              1111111111111111111111111111111111111111111111111111111111111111111111
Chain 2:  378 NPDNFFAENEQAAFHPGHIVPGLDFTNDPLLQGRLYSYTDTQISRLGGPNFHEIPINRPTCPYHNFQRDG

                  .    :    .    :    .    :    .    :    .    :    .    :    .    :
Chain 1:  448 MHRMGIDTNPANYEPNSINDNWPRETPPGPKRGGFESYQERVEGNKVRERSPSFGEYYSHPRLFWLSQTP
              1111111111111111111111111111111111111111111111111111111111111111111111
Chain 2:  448 MHRMGIDTNPANYEPNSINDNWPRETPPGPKRGGFESYQERVEGNKVRERSPSFGEYYSHPRLFWLSQTP

                  .    :    .    :    .    :    .    :    .    :    .    :    .    :
Chain 1:  518 FEQRHIVDGFSFELSKVVRPYIRERVVDQLAHIDLTLAQAVAKNLGIELTDDQLNITPPPDVNGLKKDPS
              1111111111111111111111111111111111111111111111111111111111111111111111
Chain 2:  518 FEQRHIVDGFSFELSKVVRPYIRERVVDQLAHIDLTLAQAVAKNLGIELTDDQLNITPPPDVNGLKKDPS

                  .    :    .    :    .    :    .    :    .    :    .    :    .    :
Chain 1:  588 LSLYAIPDGDVKGRVVAILLNDEVRSADLLAILKALKAKGVHAKLLYSRMGEVTADDGTVLPIAATFAGA
              1111111111111111111111111111111111111111111111111111111111111111111111
Chain 2:  588 LSLYAIPDGDVKGRVVAILLNDEVRSADLLAILKALKAKGVHAKLLYSRMGEVTADDGTVLPIAATFAGA

                  .    :    .    :    .    :    .    :    .    :    .    :    .    :
Chain 1:  658 PSLTVDAVIVPCGNIADIADNGDANYYLMEAYKHLKPIALAGDARKFKATIKIADQGEEGIVEADSADGS
              1111111111111111111111111111111111111111111111111111111111111111111111
Chain 2:  658 PSLTVDAVIVPxGNIADIADNGDANYYLMEAYKHLKPIALAGDARKFKATIKIADQGEEGIVEADSADGS

                  .    :    .    :    .
Chain 1:  728 FMDELLTLMAAHRVWSRIPKIDKIPA
              11111111111111111111111111
Chain 2:  728 FMDELLTLMAAHRVWSRIPKIDKIPA
```

UniProt ID: P21179
PDB ID: 3TTW_B

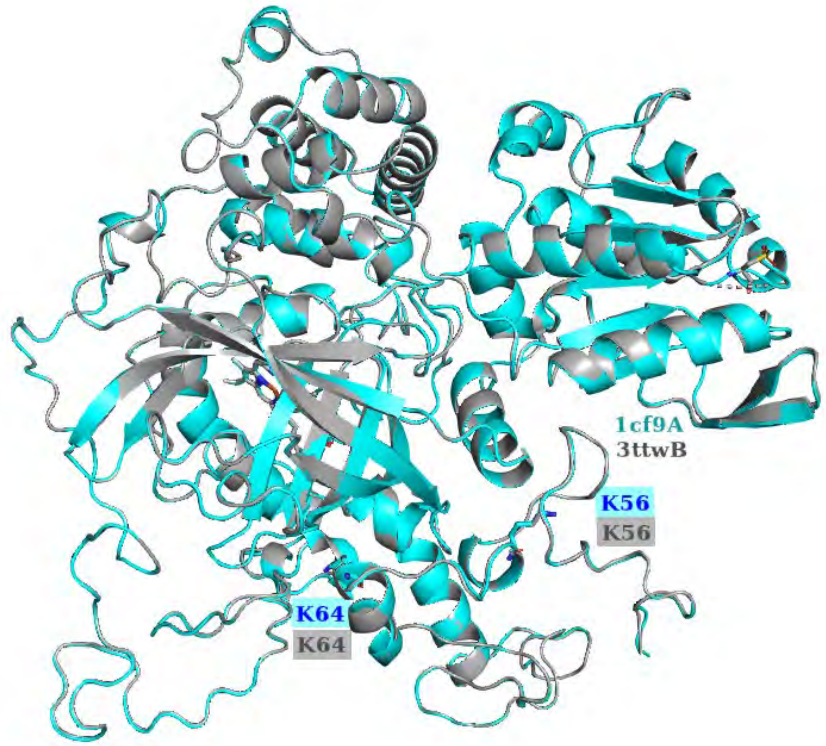


```
Align 1cf9.A.pdb 727 with 3ttw.B.pdb 726
Twists 0 ini-len 720 ini-rmsd 0.20 opt-equ 726 opt-rmsd 0.22 chain-rmsd 0.20 Score 2158.15 align-len 726 gaps 0 (0.00%)
P-value 0.00e+00 Afp-num 167571 Identity 99.59% Similarity 99.59%
Block  0 afp 90 score 2158.15 rmsd  0.20 gap 0 (0.00%)

                  .    :    .    :    .    :    .    :    .    :    .    :    .    :
Chain 1:   28 SLAPEDGSHRPAAEPTPPGAQPTAPGSLKAPDTRNEKLNSLEDVRKGSENYALTTNQGVRIADDQNSLRA
              1111111111111111111111111111111111111111111111111111111111111111111111
Chain 2:   28 SLAPEDGSHRPAAEPTPPGAQPTAPGSLKAPDTRNEKLNSLEDVRKGSENYALTTNQGVRIADDQNSLRA

                  .    :    .    :    .    :    .    :    .    :    .    :    .    :
Chain 1:   98 GSRGPTLLEDFILREKITHFDHERIPERIVHARGSAAHGYFQPYKSLSDITKADFLSDPNKITPVFVRFS
              1111111111111111111111111111111111111111111111111111111111111111111111
Chain 2:   98 GSRGPTLLEDFILREKITHFDHERIPERIVHARGSAAHGYFQPYKSLSDITKADFLSDPNKITPVFVRFS

                  .    :    .    :    .    :    .    :    .    :    .    :    .    :
Chain 1:  168 TCQGGAGSADTVRDIRGFATKFYTEEGIFDLVGNNTPIFFIQDAHKFPDFVHAVKPEPHWAIPQGQSAHD
              1111111111111111111111111111111111111111111111111111111111111111111111
Chain 2:  168 TVQGGAGSADTVRDIRGFATKFYTEEGIFDLVGNNTPIFFIQDAHKFPDFVHAVKPEPHWAIPQGQSAHD

                  .    :    .    :    .    :    .    :    .    :    .    :    .    :
Chain 1:  238 TFWDYVSLQPETLHNVMWAMSDRGIPRSYRTMEGFGIHTFRLINAEGKATFVRFHWKPLAGKASLVWDEA
              1111111111111111111111111111111111111111111111111111111111111111111111
Chain 2:  238 TFWDYVSLQPETLHNVMWAMSDRGIPRSYRTMEGFGIHTFRLINAEGKATFVRFHWKPLAGKASLVWDEA

                  .    :    .    :    .    :    .    :    .    :    .    :    .    :
Chain 1:  308 QKLTGRDPDFHRRELWEAIEAGDFPEYELGFQLIPEEDEFKFDFDLLDPTKLIPEELVPVQRVGKMVLNR
              1111111111111111111111111111111111111111111111111111111111111111111111
Chain 2:  308 QKLTGRDPDFHRRELWEAIEAGDFPEYELGFQLIPEEDEFKFDFDLLDPTKLIPEELVPVQRVGKMVLNR

                  .    :    .    :    .    :    .    :    .    :    .    :    .    :
Chain 1:  378 NPDNFFAENEQAAFHPGHIVPGLDFTNDPLLQGRLFSYTDTQISRLGGPNFHEIPINRPTCPYHNFQRDG
              1111111111111111111111111111111111111111111111111111111111111111111111
Chain 2:  378 NPDNFFAENEQAAFHPGHIVPGLDFTNDPLLQGRLESYTDTQISRLGGPNFHEIPINRPTCPYHNFQRDG

                  .    :    .    :    .    :    .    :    .    :    .    :    .    :
Chain 1:  448 MHRMGIDTNPANYEPNSINDNWPRETPPGPKRGGFESYQERVEGNKVRERSPSFGEYYSHPRLFWLSQTP
              1111111111111111111111111111111111111111111111111111111111111111111111
Chain 2:  448 MHRMGIDTNPANYEPNSINDNWPRETPPGPKRGGFESYQERVEGNKVRERSPSFGEYYSHPRLFWLSQTP

                  .    :    .    :    .    :    .    :    .    :    .    :    .    :
Chain 1:  518 FEQRHIVDGFSFELSKVVRPYIRERVVDQLAHIDLTLAQAVAKNLGIELTDDQLNITPPPDVNGLKKDPS
              1111111111111111111111111111111111111111111111111111111111111111111111
Chain 2:  518 FEQRHIVDGFSFELSKVVRPYIRERVVDQLAHIDLTLAQAVAKNLGIELTDDQLNITPPPDVNGLKKDPS

                  .    :    .    :    .    :    .    :    .    :    .    :    .    :
Chain 1:  588 LSLYAIPDGDVKGRVVAILLNDEVRSADLLAILKALKAKGVHAKLLYSRMGEVTADDGTVLPIAATFAGA
              1111111111111111111111111111111111111111111111111111111111111111111111
Chain 2:  588 LSLYAIPDGDVKGRVVAILLNDEVRSADLLAILKALKAKGVHAKLLYSRMGEVTADDGTVLPIAATFAGA

                  .    :    .    :    .    :    .    :    .    :    .    :    .    :
Chain 1:  658 PSLTVDAVIVPCGNIADIADNGDANYYLMEAYKHLKPIALAGDARKFKATIKIADQGEEGIVEADSADGS
              1111111111111111111111111111111111111111111111111111111111111111111111
Chain 2:  658 PSLTVDAVIVPxGNIADIADNGDANYYLMEAYKHLKPIALAGDARKFKATIKIADQGEEGIVEADSADGS

                  .    :    .    :    .
Chain 1:  728 FMDELLTLMAAHRVWSRIPKIDKIPA
              11111111111111111111111111
Chain 2:  728 FMDELLTLMAAHRVWSRIPKIDKIPA
```

UniProt ID: P21179
PDB ID: 3TTX_B

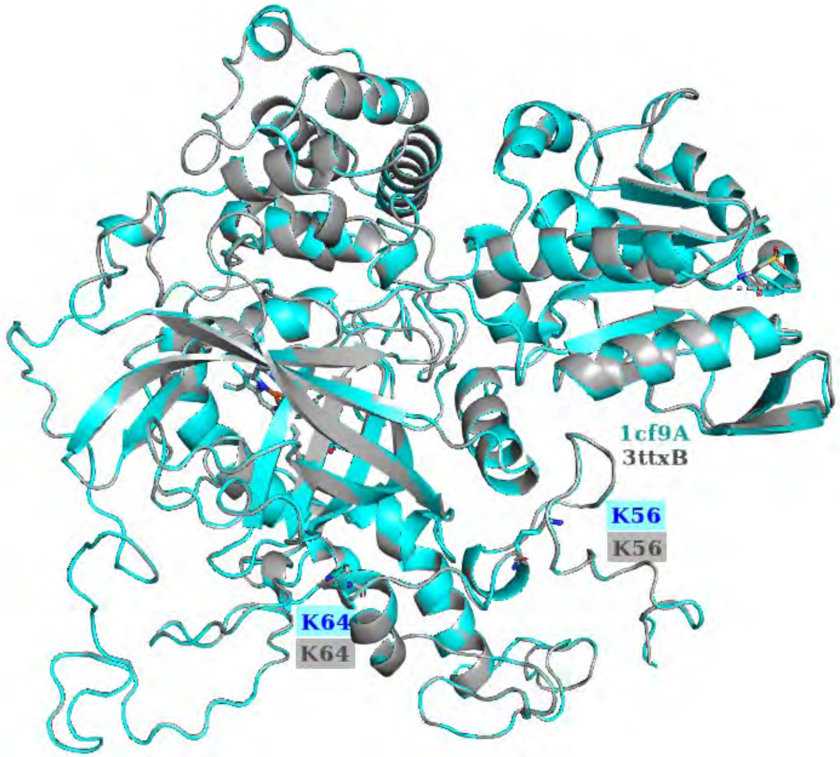


```
Align 1cf9.A.pdb 727 with 3ttx.B.pdb 726
Twists 0 ini-len 720 ini-rmsd 0.20 opt-equ 726 opt-rmsd 0.22 chain-rmsd 0.20 Score 2158.12 align-len 726 gaps 0 (0.00%)
P-value 0.00e+00 Afp-num 166839 Identity 99.59% Similarity 99.59%
Block  0 afp 90 score 2158.12 rmsd  0.20 gap 0 (0.00%)

                    .    :    .    :    .    :    .    :    .    :    .    :    .    :
Chain 1:   28 SLAPEDGSHRPAAEPTPPGAQPTAPGSLKAPDTRNEKLNSLEDVRKGSENYALTTNQGVRIADDQNSLRA
              1111111111111111111111111111111111111111111111111111111111111111111111
Chain 2:   28 SLAPEDGSHRPAAEPTPPGAQPTAPGSLKAPDTRNEKLNSLEDVRKGSENYALTTNQGVRIADDQNSLRA

                    .    :    .    :    .    :    .    :    .    :    .    :    .    :
Chain 1:   98 GSRGPTLLEDFILREKITHFDHERIPERIVHARGSAAHGYFQPYKSLSDITKADFLSDPNKITPVFVRFS
              1111111111111111111111111111111111111111111111111111111111111111111111
Chain 2:   98 GSRGPTLLEDFILREKITHFDHERIPERIVHARGSAAHGYFQPYKSLSDITKADFLSDPNKITPVFVRFS

                    .    :    .    :    .    :    .    :    .    :    .    :    .    :
Chain 1:  168 TCQGGAGSADTVRDIRGFATKFYTEEGIFDLVGNNTPIFFIQDAHKFPDFVHAVKPEPHWAIPQGQSAHD
              1111111111111111111111111111111111111111111111111111111111111111111111
Chain 2:  168 TVQGGAGSADTVRDIRGFATKFYTEEGIFDLVGNNTPIFFIQDAHKFPDFVHAVKPEPHWAIPQGQSAHD

                    .    :    .    :    .    :    .    :    .    :    .    :    .    :
Chain 1:  238 TFWDYVSLQPETLHNVMWAMSDRGIPRSYRTMEGFGIHTFRLINAEGKATFVRFHWKPLAGKASLVWDEA
              1111111111111111111111111111111111111111111111111111111111111111111111
Chain 2:  238 TFWDYVSLQPETLHNVMWAMSDRGIPRSYRTMEGFGIHTFRLINAEGKATFVRFHWKPLAGKASLVWDEA

                    .    :    .    :    .    :    .    :    .    :    .    :    .    :
Chain 1:  308 QKLTGRDPDFHRRELWEAIEAGDFPEYELGFQLIPEEDEFKFDFDLLDPTKLIPEELVPVQRVGKMVLNR
              1111111111111111111111111111111111111111111111111111111111111111111111
Chain 2:  308 QKLTGRDPDFHRRELWEAIEAGDFPEYELGFQLIPEEDEFKFDFDLLDPTKLIPEELVPVQRVGKMVLNR

                    .    :    .    :    .    :    .    :    .    :    .    :    .    :
Chain 1:  378 NPDNFFAENEQAAFHPGHIVPGLDFTNDPLLQGRLFSYTDTQISRLGGPNFHEIPINRPTCPYHNFQRDG
              1111111111111111111111111111111111111111111111111111111111111111111111
Chain 2:  378 NPDNFFAENEQAAFHPGHIVPGLDFTNDPLLQGRLKSYTDTQISRLGGPNFHEIPINRPTCPYHNFQRDG

                    .    :    .    :    .    :    .    :    .    :    .    :    .    :
Chain 1:  448 MHRMGIDTNPANYEPNSINDNWPRETPPGPKRGGFESYQERVEGNKVRERSPSFGEYYSHPRLFWLSQTP
              1111111111111111111111111111111111111111111111111111111111111111111111
Chain 2:  448 MHRMGIDTNPANYEPNSINDNWPRETPPGPKRGGFESYQERVEGNKVRERSPSFGEYYSHPRLFWLSQTP

                    .    :    .    :    .    :    .    :    .    :    .    :    .    :
Chain 1:  518 FEQRHIVDGFSFELSKVVRPYIRERVVDQLAHIDLTLAQAVAKNLGIELTDDQLNITPPPDVNGLKKDPS
              1111111111111111111111111111111111111111111111111111111111111111111111
Chain 2:  518 FEQRHIVDGFSFELSKVVRPYIRERVVDQLAHIDLTLAQAVAKNLGIELTDDQLNITPPPDVNGLKKDPS

                    .    :    .    :    .    :    .    :    .    :    .    :    .    :
Chain 1:  588 LSLYAIPDGDVKGRVVAILLNDEVRSADLLAILKALKAKGVHAKLLYSRMGEVTADDGTVLPIAATFAGA
              1111111111111111111111111111111111111111111111111111111111111111111111
Chain 2:  588 LSLYAIPDGDVKGRVVAILLNDEVRSADLLAILKALKAKGVHAKLLYSRMGEVTADDGTVLPIAATFAGA

                    .    :    .    :    .    :    .    :    .    :    .    :    .    :
Chain 1:  658 PSLTVDAVIVPCGNIADIADNGDANYYLMEAYKHLKPIALAGDARKFKATIKIADQGEEGIVEADSADGS
              1111111111111111111111111111111111111111111111111111111111111111111111
Chain 2:  658 PSLTVDAVIVPxGNIADIADNGDANYYLMEAYKHLKPIALAGDARKFKATIKIADQGEEGIVEADSADGS

                    .    :    .    :    .
Chain 1:  728 FMDELLTLMAAHRVWSRIPKIDKIPA
              11111111111111111111111111
Chain 2:  728 FMDELLTLMAAHRVWSRIPKIDKIPA
```

UniProt ID: P21179

PDB ID: 3VU3_A

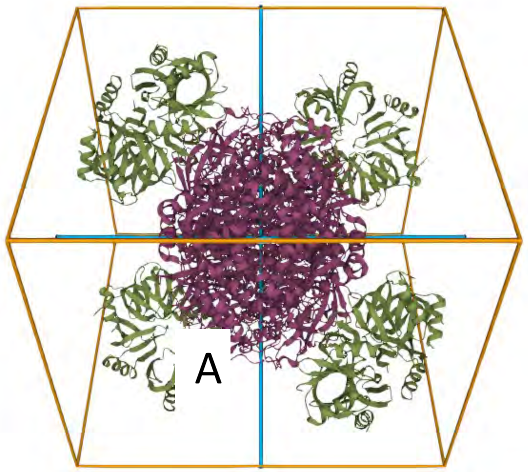

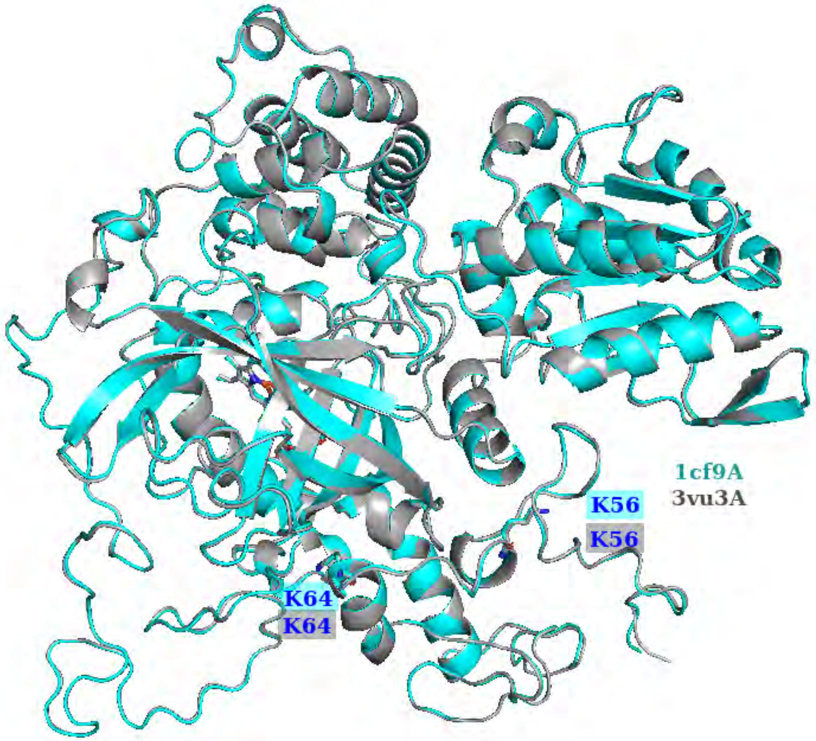

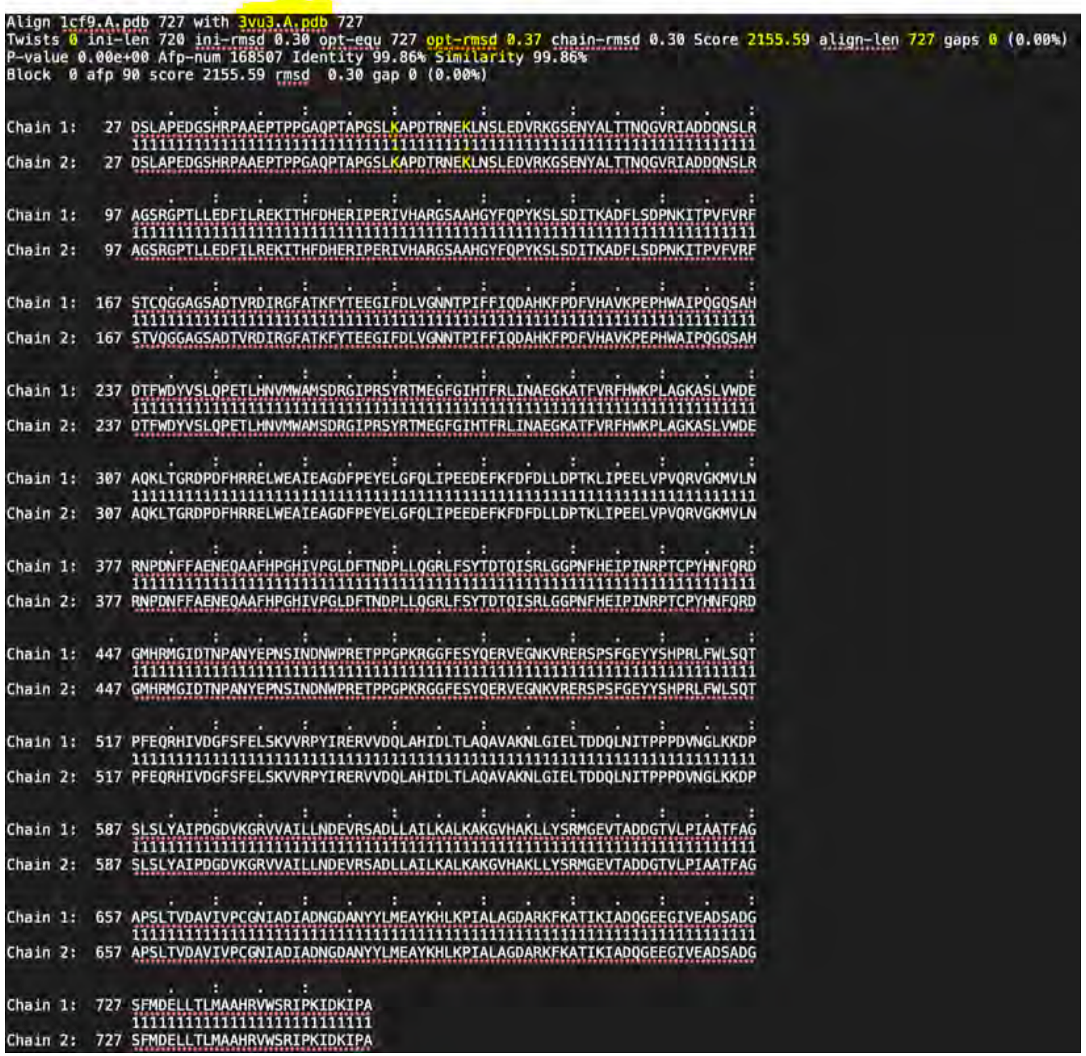

```
Align 1cf9.A.pdb 727 with 3vu3.A.pdb 727
Twists 0 ini-len 720 ini-rmsd 0.30 opt-equ 727 opt-rmsd 0.37 chain-rmsd 0.30 Score 2155.59 align-len 727 gaps 0 (0.00%)
P-value 0.00e+00 Afp-num 168507 Identity 99.86% Similarity 99.86%
Block  0 afp 90 score 2155.59 rmsd  0.30 gap 0 (0.00%)

Chain 1:   27 DSLAPEDGSHRPAAEPTPPGAQPTAPGSLKAPDTRNEKLNSLEDVRKGSENYALTTNQGVRIADDQNSLR
              1111111111111111111111111111111111111111111111111111111111111111111111
Chain 2:   27 DSLAPEDGSHRPAAEPTPPGAQPTAPGSLKAPDTRNEKLNSLEDVRKGSENYALTTNQGVRIADDQNSLR

Chain 1:   97 AGSRGPTLLEDFILREKITHFDHERIPERIVHARGSAAHGYFQPYKSLSDITKADFLSDPNKITPVFVRF
              1111111111111111111111111111111111111111111111111111111111111111111111
Chain 2:   97 AGSRGPTLLEDFILREKITHFDHERIPERIVHARGSAAHGYFQPYKSLSDITKADFLSDPNKITPVFVRF

Chain 1:  167 STCQGGAGSADTVRDIRGFATKFYTEEGIFDLVGNNTPIFFIQDAHKFPDFVHAVKPEPHWAIPQGQSAH
              1111111111111111111111111111111111111111111111111111111111111111111111
Chain 2:  167 STVQGGAGSADTVRDIRGFATKFYTEEGIFDLVGNNTPIFFIQDAHKFPDFVHAVKPEPHWAIPQGQSAH

Chain 1:  237 DTFWDYVSLQPETLHNVMWAMSDRGIPRSYRTMEGFGIHTFRLINAEGKATFVRFHWKPLAGKASLVWDE
              1111111111111111111111111111111111111111111111111111111111111111111111
Chain 2:  237 DTFWDYVSLQPETLHNVMWAMSDRGIPRSYRTMEGFGIHTFRLINAEGKATFVRFHWKPLAGKASLVWDE

Chain 1:  307 AQKLTGRDPDFHRRELWEAIEAGDFPEYELGFQLIPEEDEFKFDFDLLDPTKLIPEELVPVQRVGKMVLN
              1111111111111111111111111111111111111111111111111111111111111111111111
Chain 2:  307 AQKLTGRDPDFHRRELWEAIEAGDFPEYELGFQLIPEEDEFKFDFDLLDPTKLIPEELVPVQRVGKMVLN

Chain 1:  377 RNPDNFFAENEQAAFHPGHIVPGLDFTNDPLLQGRLFSYTDTQISRLGGPNFHEIPINRPTCPYHNFQRD
              1111111111111111111111111111111111111111111111111111111111111111111111
Chain 2:  377 RNPDNFFAENEQAAFHPGHIVPGLDFTNDPLLQGRLFSYTDTQISRLGGPNFHEIPINRPTCPYHNFQRD

Chain 1:  447 GMHRMGIDTNPANYEPNSINDNWPRETPPGPKRGGFESYQERVEGNKVRERSPSFGEYYSHPRLFWLSQT
              1111111111111111111111111111111111111111111111111111111111111111111111
Chain 2:  447 GMHRMGIDTNPANYEPNSINDNWPRETPPGPKRGGFESYQERVEGNKVRERSPSFGEYYSHPRLFWLSQT

Chain 1:  517 PFEQRHIVDGFSFELSKVVRPYIRERVVDQLAHIDLTLAQAVAKNLGIELTDDQLNITPPPDVNGLKKDP
              1111111111111111111111111111111111111111111111111111111111111111111111
Chain 2:  517 PFEQRHIVDGFSFELSKVVRPYIRERVVDQLAHIDLTLAQAVAKNLGIELTDDQLNITPPPDVNGLKKDP

Chain 1:  587 SLSLYAIPDGDVKGRVVAILLNDEVRSADLLAILKALKAKGVHAKLLYSRMGEVTADDGTVLPIAATFAG
              1111111111111111111111111111111111111111111111111111111111111111111111
Chain 2:  587 SLSLYAIPDGDVKGRVVAILLNDEVRSADLLAILKALKAKGVHAKLLYSRMGEVTADDGTVLPIAATFAG

Chain 1:  657 APSLTVDAVIVPCGNIADIADNGDANYYLMEAYKHLKPIALAGDARKFKATIKIADQGEEGIVEADSADG
              1111111111111111111111111111111111111111111111111111111111111111111111
Chain 2:  657 APSLTVDAVIVPCGNIADIADNGDANYYLMEAYKHLKPIALAGDARKFKATIKIADQGEEGIVEADSADG

Chain 1:  727 SFMDELLTLMAAHRVWSRIPKIDKIPA
              111111111111111111111111111
Chain 2:  727 SFMDELLTLMAAHRVWSRIPKIDKIPA
```

UniProt ID: P21179

PDB ID: 4BFL_D

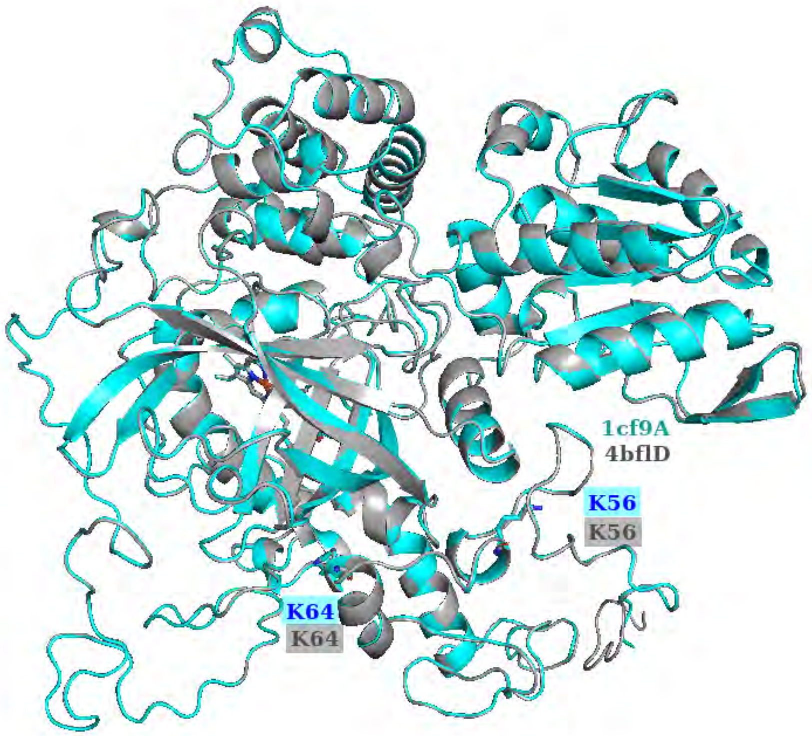


```
Align 1cf9.A.pdb 727 with 4bfl.D.pdb 735
Twists 0 ini-len 720 ini-rmsd 0.22 opt-equ 727 opt-rmsd 0.27 chain-rmsd 0.22 Score 2158.06 align-len 727 gaps 0 (0.00%)
P-value 0.00e+00 Afp-num 167320 Identity 99.86% Similarity 99.86%
Block  0 afp 90 score 2158.06 rmsd  0.22 gap 0 (0.00%)

                   .    :    .    :    .    :    .    :    .    :    .    :    .    :
Chain 1:   27 DSLAPEDGSHRPAAEPTPPGAQPTAPGSLKAPDTRNEKLNSLEDVRKGSENYALTTNQGVRIADDQNSLR
              1111111111111111111111111111111111111111111111111111111111111111111111
Chain 2:   27 DSLAPEDGSHRPAAEPTPPGAQPTAPGSLKAPDTRNEKLNSLEDVRKGSENYALTTNQGVRIADDQNSLR

                   .    :    .    :    .    :    .    :    .    :    .    :    .    :
Chain 1:   97 AGSRGPTLLEDFILREKITHFDHERIPERIVHARGSAAHGYFQPYKSLSDITKADFLSDPNKITPVFVRF
              1111111111111111111111111111111111111111111111111111111111111111111111
Chain 2:   97 AGSRGPTLLEDFILREKITHFDHERIPERIVHARGSAAHGYFQPYKSLSDITKADFLSDPNKITPVFVRF

                   .    :    .    :    .    :    .    :    .    :    .    :    .    :
Chain 1:  167 STCQGGAGSADTVRDIRGFATKFYTEEGIFDLVGNNTPIFFIQDAHKFPDFVHAVKPEPHWAIPQGQSAH
              1111111111111111111111111111111111111111111111111111111111111111111111
Chain 2:  167 STVQGGAGSADTVRDIRGFATKFYTEEGIFDLVGNNTPIFFIQDAHKFPDFVHAVKPEPHWAIPQGQSAH

                   .    :    .    :    .    :    .    :    .    :    .    :    .    :
Chain 1:  237 DTFWDYVSLQPETLHNVMWAMSDRGIPRSYRTMEGFGIHTFRLINAEGKATFVRFHWKPLAGKASLVWDE
              1111111111111111111111111111111111111111111111111111111111111111111111
Chain 2:  237 DTFWDYVSLQPETLHNVMWAMSDRGIPRSYRTMEGFGIHTFRLINAEGKATFVRFHWKPLAGKASLVWDE

                   .    :    .    :    .    :    .    :    .    :    .    :    .    :
Chain 1:  307 AQKLTGRDPDFHRRELWEAIEAGDFPEYELGFQLIPEEDEFKFDFDLLDPTKLIPEELVPVQRVGKMVLN
              1111111111111111111111111111111111111111111111111111111111111111111111
Chain 2:  307 AQKLTGRDPDFHRRELWEAIEAGDFPEYELGFQLIPEEDEFKFDFDLLDPTKLIPEELVPVQRVGKMVLN

                   .    :    .    :    .    :    .    :    .    :    .    :    .    :
Chain 1:  377 RNPDNFFAENEQAAFHPGHIVPGLDFTNDPLLQGRLFSYTDTQISRLGGPNFHEIPINRPTCPYHNFQRD
              1111111111111111111111111111111111111111111111111111111111111111111111
Chain 2:  377 RNPDNFFAENEQAAFHPGHIVPGLDFTNDPLLQGRLFSYTDTQISRLGGPNFHEIPINRPTCPYHNFQRD

                   .    :    .    :    .    :    .    :    .    :    .    :    .    :
Chain 1:  447 GMHRMGIDTNPANYEPNSINDNWPRETPPGPKRGGFESYQERVEGNKVRERSPSFGEYYSHPRLFWLSQT
              1111111111111111111111111111111111111111111111111111111111111111111111
Chain 2:  447 GMHRMGIDTNPANYEPNSINDNWPRETPPGPKRGGFESYQERVEGNKVRERSPSFGEYYSHPRLFWLSQT

                   .    :    .    :    .    :    .    :    .    :    .    :    .    :
Chain 1:  517 PFEQRHIVDGFSFELSKVVRPYIRERVVDQLAHIDLTLAQAVAKNLGIELTDDQLNITPPPDVNGLKKDP
              1111111111111111111111111111111111111111111111111111111111111111111111
Chain 2:  517 PFEQRHIVDGFSFELSKVVRPYIRERVVDQLAHIDLTLAQAVAKNLGIELTDDQLNITPPPDVNGLKKDP

                   .    :    .    :    .    :    .    :    .    :    .    :    .    :
Chain 1:  587 SLSLYAIPDGDVKGRVVAILLNDEVRSADLLAILKALKAKGVHAKLLYSRMGEVTADDGTVLPIAATFAG
              1111111111111111111111111111111111111111111111111111111111111111111111
Chain 2:  587 SLSLYAIPDGDVKGRVVAILLNDEVRSADLLAILKALKAKGVHAKLLYSRMGEVTADDGTVLPIAATFAG

                   .    :    .    :    .    :    .    :    .    :    .    :    .    :
Chain 1:  657 APSLTVDAVIVPCGNIADIADNGDANYYLMEAYKHLKPIALAGDARKFKATIKIADQGEEGIVEADSADG
              1111111111111111111111111111111111111111111111111111111111111111111111
Chain 2:  657 APSLTVDAVIVPCGNIADIADNGDANYYLMEAYKHLKPIALAGDARKFKATIKIADQGEEGIVEADSADG

                   .    :    .    :    .
Chain 1:  727 SFMDELLTLMAAHRVWSRIPKIDKIPA
              111111111111111111111111111
Chain 2:  727 SFMDELLTLMAAHRVWSRIPKIDKIPA
```

UniProt ID: P21179
PDB ID: 4ENP_A

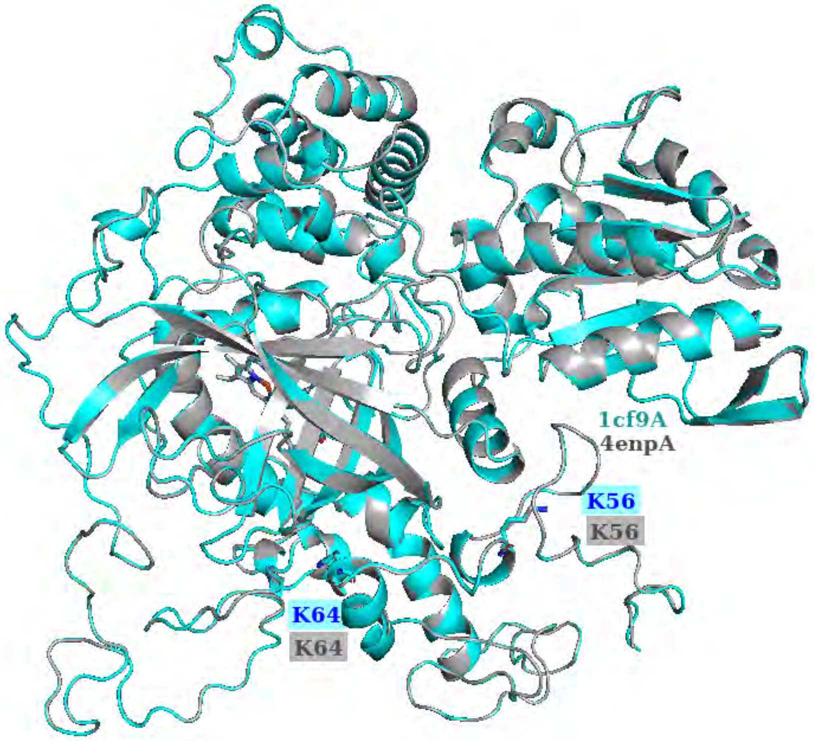

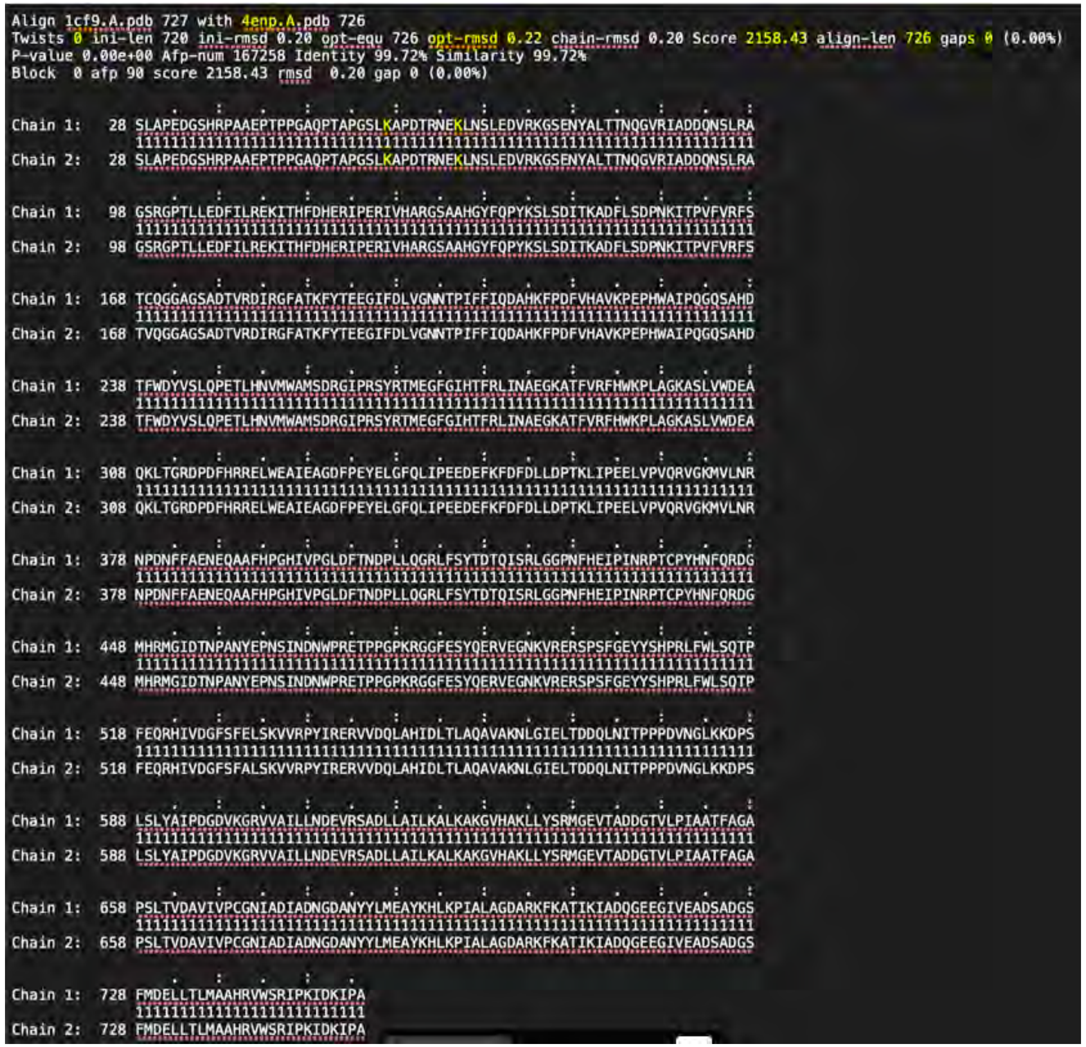

UniProt ID: P21179
PDB ID: 4ENQ_A

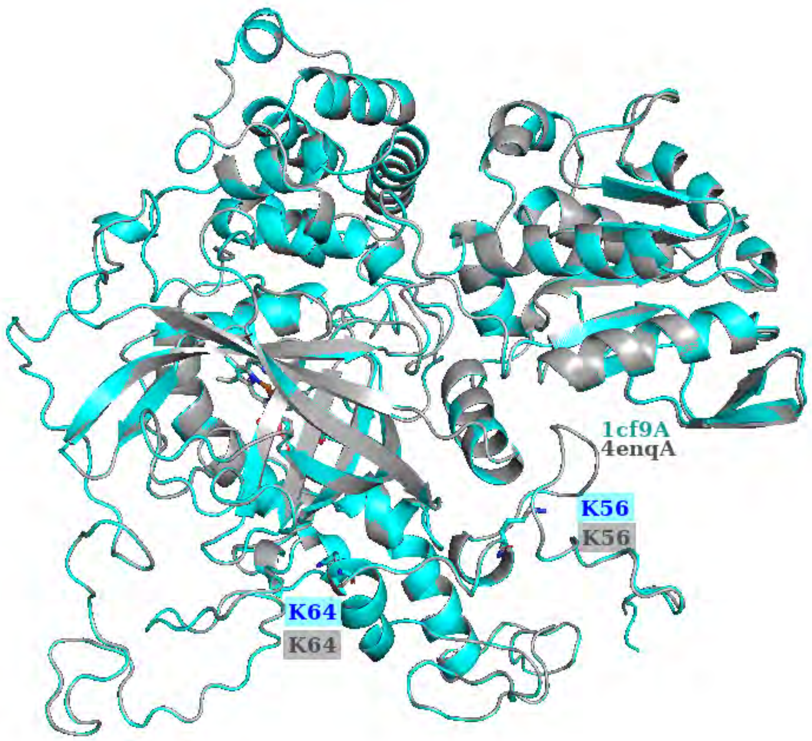

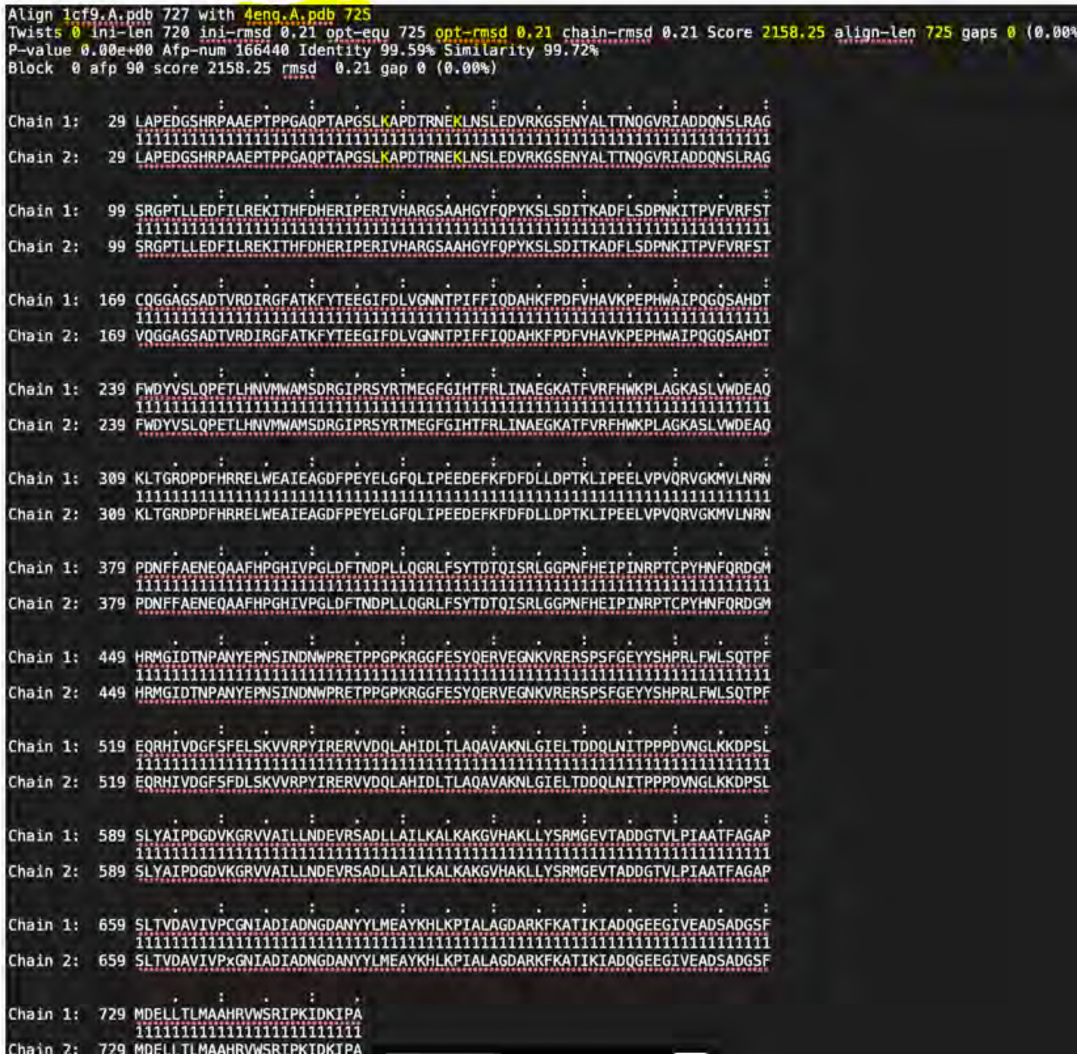

```
Align 1cf9.A.pdb 727 with 4enq.A.pdb 725
Twists 0 ini-len 720 ini-rmsd 0.21 opt-equ 725 opt-rmsd 0.21 chain-rmsd 0.21 Score 2158.25 align-len 725 gaps 0 (0.00%
P-value 0.00e+00 Afp-num 166440 Identity 99.59% Similarity 99.72%
Block  0 afp 90 score 2158.25 rmsd  0.21 gap 0 (0.00%)

                   .    :    .    :    .    :    .    :    .    :    .    :    .    :
Chain 1:   29 LAPEDGSHRPAAEPTPPGAQPTAPGSLKAPDTRNEKLNSLEDVRKGSENYALTTNQGVRIADDQNSLRAG
              1111111111111111111111111111111111111111111111111111111111111111111111
Chain 2:   29 LAPEDGSHRPAAEPTPPGAQPTAPGSLKAPDTRNEKLNSLEDVRKGSENYALTTNQGVRIADDQNSLRAG

                   .    :    .    :    .    :    .    :    .    :    .    :    .    :
Chain 1:   99 SRGPTLLEDFILREKITHFDHERIPERIVHARGSAAHGYFQPYKSLSDITKADFLSDPNKITPVFVRFST
              1111111111111111111111111111111111111111111111111111111111111111111111
Chain 2:   99 SRGPTLLEDFILREKITHFDHERIPERIVHARGSAAHGYFQPYKSLSDITKADFLSDPNKITPVFVRFST

                   .    :    .    :    .    :    .    :    .    :    .    :    .    :
Chain 1:  169 CQGGAGSADTVRDIRGFATKFYTEEGIFDLVGNNTPIFFIQDAHKFPDFVHAVKPEPHWAIPQGQSAHDT
              1111111111111111111111111111111111111111111111111111111111111111111111
Chain 2:  169 VQGGAGSADTVRDIRGFATKFYTEEGIFDLVGNNTPIFFIQDAHKFPDFVHAVKPEPHWAIPQGQSAHDT

                   .    :    .    :    .    :    .    :    .    :    .    :    .    :
Chain 1:  239 FWDYVSLQPETLHNVMWAMSDRGIPRSYRTMEGFGIHTFRLINAEGKATFVRFHWKPLAGKASLVWDEAQ
              1111111111111111111111111111111111111111111111111111111111111111111111
Chain 2:  239 FWDYVSLQPETLHNVMWAMSDRGIPRSYRTMEGFGIHTFRLINAEGKATFVRFHWKPLAGKASLVWDEAQ

                   .    :    .    :    .    :    .    :    .    :    .    :    .    :
Chain 1:  309 KLTGRDPDFHRRELWEAIEAGDFPEYELGFQLIPEEDEFKFDFDLLDPTKLIPEELVPVQRVGKMVLNRN
              1111111111111111111111111111111111111111111111111111111111111111111111
Chain 2:  309 KLTGRDPDFHRRELWEAIEAGDFPEYELGFQLIPEEDEFKFDFDLLDPTKLIPEELVPVQRVGKMVLNRN

                   .    :    .    :    .    :    .    :    .    :    .    :    .    :
Chain 1:  379 PDNFFAENEQAAFHPGHIVPGLDFTNDPLLQGRLFSYTDTQISRLGGPNFHEIPINRPTCPYHNFQRDGM
              1111111111111111111111111111111111111111111111111111111111111111111111
Chain 2:  379 PDNFFAENEQAAFHPGHIVPGLDFTNDPLLQGRLFSYTDTQISRLGGPNFHEIPINRPTCPYHNFQRDGM

                   .    :    .    :    .    :    .    :    .    :    .    :    .    :
Chain 1:  449 HRMGIDTNPANYEPNSINDNWPRETPPGPKRGGFESYQERVEGNKVRERSPSFGEYYSHPRLFWLSQTPF
              1111111111111111111111111111111111111111111111111111111111111111111111
Chain 2:  449 HRMGIDTNPANYEPNSINDNWPRETPPGPKRGGFESYQERVEGNKVRERSPSFGEYYSHPRLFWLSQTPF

                   .    :    .    :    .    :    .    :    .    :    .    :    .    :
Chain 1:  519 EQRHIVDGFSFELSKVVRPYIRERVVDQLAHIDLTLAQAVAKNLGIELTDDQLNITPPPDVNGLKKDPSL
              1111111111111111111111111111111111111111111111111111111111111111111111
Chain 2:  519 EQRHIVDGFSFDLSKVVRPYIRERVVDQLAHIDLTLAQAVAKNLGIELTDDQLNITPPPDVNGLKKDPSL

                   .    :    .    :    .    :    .    :    .    :    .    :    .    :
Chain 1:  589 SLYAIPDGDVKGRVVAILLNDEVRSADLLAILKALKAKGVHAKLLYSRMGEVTADDGTVLPIAATFAGAP
              1111111111111111111111111111111111111111111111111111111111111111111111
Chain 2:  589 SLYAIPDGDVKGRVVAILLNDEVRSADLLAILKALKAKGVHAKLLYSRMGEVTADDGTVLPIAATFAGAP

                   .    :    .    :    .    :    .    :    .    :    .    :    .    :
Chain 1:  659 SLTVDAVIVPCGNIADIADNGDANYYLMEAYKHLKPIALAGDARKFKATIKIADQGEEGIVEADSADGSF
              1111111111111111111111111111111111111111111111111111111111111111111111
Chain 2:  659 SLTVDAVIVPxGNIADIADNGDANYYLMEAYKHLKPIALAGDARKFKATIKIADQGEEGIVEADSADGSF

                   .    :    .    :    .
Chain 1:  729 MDELLTLMAAHRVWSRIPKIDKIPA
              1111111111111111111111111
Chain 2:  729 MDELLTLMAAHRVWSRIPKIDKIPA
```

UniProt ID: P21179

PDB ID: 4ENR_A

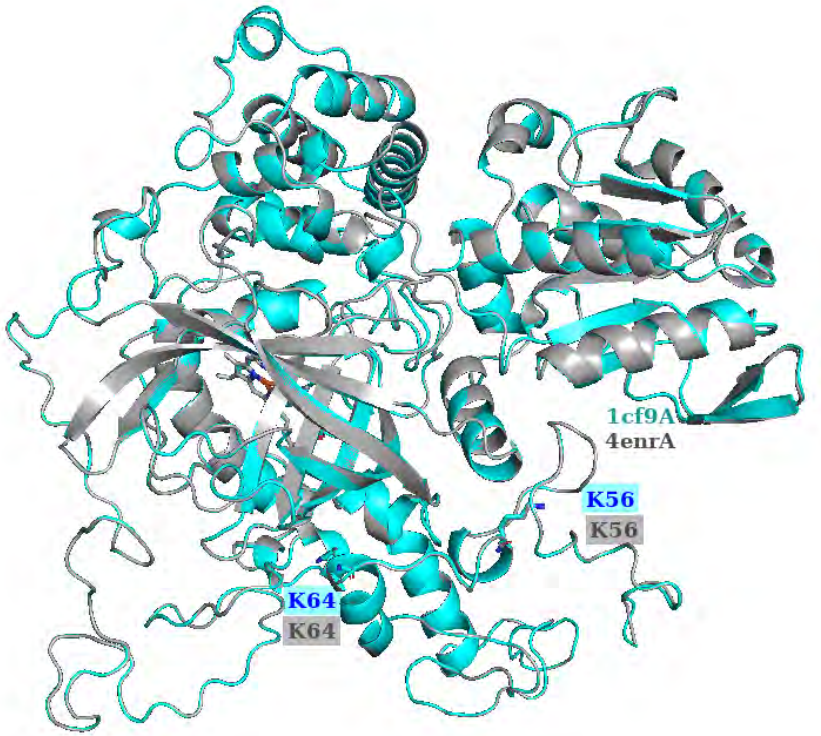

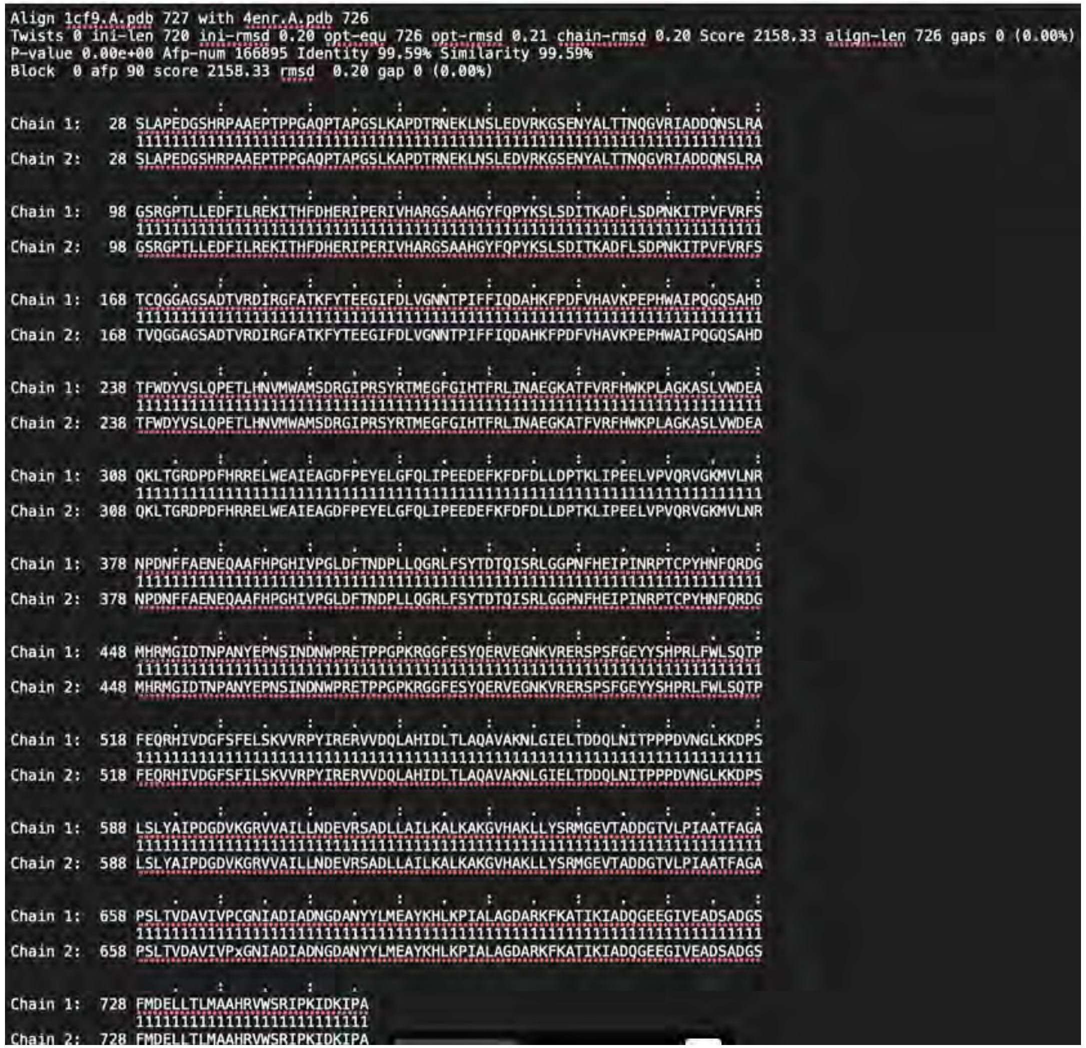

```
Align 1cf9.A.pdb 727 with 4enr.A.pdb 726
Twists 0 ini-len 720 ini-rmsd 0.20 opt-equ 726 opt-rmsd 0.21 chain-rmsd 0.20 Score 2158.33 align-len 726 gaps 0 (0.00%)
P-value 0.00e+00 Afp-num 166895 Identity 99.59% Similarity 99.59%
Block  0 afp 90 score 2158.33 rmsd  0.20 gap 0 (0.00%)

                    .    :    .    :    .    :    .    :    .    :    .    :    .    :
Chain 1:   28 SLAPEDGSHRPAAEPTPPGAQPTAPGSLKAPDTRNEKLNSLEDVRKGSENYALTTNQGVRIADDQNSLRA
              1111111111111111111111111111111111111111111111111111111111111111111111
Chain 2:   28 SLAPEDGSHRPAAEPTPPGAQPTAPGSLKAPDTRNEKLNSLEDVRKGSENYALTTNQGVRIADDQNSLRA

                    .    :    .    :    .    :    .    :    .    :    .    :    .    :
Chain 1:   98 GSRGPTLLEDFILREKITHFDHERIPERIVHARGSAAHGYFQPYKSLSDITKADFLSDPNKITPVFVRFS
              1111111111111111111111111111111111111111111111111111111111111111111111
Chain 2:   98 GSRGPTLLEDFILREKITHFDHERIPERIVHARGSAAHGYFQPYKSLSDITKADFLSDPNKITPVFVRFS

                    .    :    .    :    .    :    .    :    .    :    .    :    .    :
Chain 1:  168 TCQGGAGSADTVRDIRGFATKFYTEEGIFDLVGNNTPIFFIQDAHKFPDFVHAVKPEPHWAIPQGQSAHD
              1111111111111111111111111111111111111111111111111111111111111111111111
Chain 2:  168 TVQGGAGSADTVRDIRGFATKFYTEEGIFDLVGNNTPIFFIQDAHKFPDFVHAVKPEPHWAIPQGQSAHD

                    .    :    .    :    .    :    .    :    .    :    .    :    .    :
Chain 1:  238 TFWDYVSLQPETLHNVMWAMSDRGIPRSYRTMEGFGIHTFRLINAEGKATFVRFHWKPLAGKASLVWDEA
              1111111111111111111111111111111111111111111111111111111111111111111111
Chain 2:  238 TFWDYVSLQPETLHNVMWAMSDRGIPRSYRTMEGFGIHTFRLINAEGKATFVRFHWKPLAGKASLVWDEA

                    .    :    .    :    .    :    .    :    .    :    .    :    .    :
Chain 1:  308 QKLTGRDPDFHRRELWEAIEAGDFPEYELGFQLIPEEDEFKFDFDLLDPTKLIPEELVPVQRVGKMVLNR
              1111111111111111111111111111111111111111111111111111111111111111111111
Chain 2:  308 QKLTGRDPDFHRRELWEAIEAGDFPEYELGFQLIPEEDEFKFDFDLLDPTKLIPEELVPVQRVGKMVLNR

                    .    :    .    :    .    :    .    :    .    :    .    :    .    :
Chain 1:  378 NPDNFFAENEQAAFHPGHIVPGLDFTNDPLLQGRLFSYTDTQISRLGGPNFHEIPINRPTCPYHNFQRDG
              1111111111111111111111111111111111111111111111111111111111111111111111
Chain 2:  378 NPDNFFAENEQAAFHPGHIVPGLDFTNDPLLQGRLFSYTDTQISRLGGPNFHEIPINRPTCPYHNFQRDG

                    .    :    .    :    .    :    .    :    .    :    .    :    .    :
Chain 1:  448 MHRMGIDTNPANYEPNSINDNWPRETPPGPKRGGFESYQERVEGNKVRERSPSFGEYYSHPRLFWLSQTP
              1111111111111111111111111111111111111111111111111111111111111111111111
Chain 2:  448 MHRMGIDTNPANYEPNSINDNWPRETPPGPKRGGFESYQERVEGNKVRERSPSFGEYYSHPRLFWLSQTP

                    .    :    .    :    .    :    .    :    .    :    .    :    .    :
Chain 1:  518 FEQRHIVDGFSFELSKVVRPYIRERVVDQLAHIDLTLAQAVAKNLGIELTDDQLNITPPPDVNGLKKDPS
              1111111111111111111111111111111111111111111111111111111111111111111111
Chain 2:  518 FEQRHIVDGFSFILSKVVRPYIRERVVDQLAHIDLTLAQAVAKNLGIELTDDQLNITPPPDVNGLKKDPS

                    .    :    .    :    .    :    .    :    .    :    .    :    .    :
Chain 1:  588 LSLYAIPDGDVKGRVVAILLNDEVRSADLLAILKALKAKGVHAKLLYSRMGEVTADDGTVLPIAATFAGA
              1111111111111111111111111111111111111111111111111111111111111111111111
Chain 2:  588 LSLYAIPDGDVKGRVVAILLNDEVRSADLLAILKALKAKGVHAKLLYSRMGEVTADDGTVLPIAATFAGA

                    .    :    .    :    .    :    .    :    .    :    .    :    .    :
Chain 1:  658 PSLTVDAVIVPCGNIADIADNGDANYYLMEAYKHLKPIALAGDARKFKATIKIADQGEEGIVEADSADGS
              1111111111111111111111111111111111111111111111111111111111111111111111
Chain 2:  658 PSLTVDAVIVPxGNIADIADNGDANYYLMEAYKHLKPIALAGDARKFKATIKIADQGEEGIVEADSADGS

                    .    :    .    :    .
Chain 1:  728 FMDELLTLMAAHRVWSRIPKIDKIPA
              11111111111111111111111111
Chain 2:  728 FMDELLTLMAAHRVWSRIPKIDKIPA
```

UniProt ID: P21179

PDB ID: 4ENS_A

```
Align 1cf9.A.pdb 727 with 4ens.A.pdb 726
Twists 0 ini-len 720 ini-rmsd 0.20 opt-equ 726 opt-rmsd 0.22 chain-rmsd 0.20 Score 2158.29 align-len 726 gaps 0 (0.00%)
P-value 0.00e+00 Afp-num 166963 Identity 99.72% Similarity 99.86%
Block  0 afp 90 score 2158.29 rmsd  0.20 gap 0 (0.00%)

                      .    :    .    :    .    :    .    :    .    :    .    :    .    :
Chain 1:   28 SLAPEDGSHRPAAEPTPPGAQPTAPGSLKAPDTRNEKLNSLEDVRKGSENYALTTNQGVRIADDQNSLRA
              1111111111111111111111111111111111111111111111111111111111111111111111
Chain 2:   28 SLAPEDGSHRPAAEPTPPGAQPTAPGSLKAPDTRNEKLNSLEDVRKGSENYALTTNQGVRIADDQNSLRA

                      .    :    .    :    .    :    .    :    .    :    .    :    .    :
Chain 1:   98 GSRGPTLLEDFILREKITHFDHERIPERIVHARGSAAHGYFQPYKSLSDITKADFLSDPNKITPVFVRFS
              1111111111111111111111111111111111111111111111111111111111111111111111
Chain 2:   98 GSRGPTLLEDFILREKITHFDHERIPERIVHARGSAAHGYFQPYKSLSDITKADFLSDPNKITPVFVRFS

                      .    :    .    :    .    :    .    :    .    :    .    :    .    :
Chain 1:  168 TCQGGAGSADTVRDIRGFATKFYTEEGIFDLVGNNTPIFFIQDAHKFPDFVHAVKPEPHWAIPQGQSAHD
              1111111111111111111111111111111111111111111111111111111111111111111111
Chain 2:  168 TVQGGAGSADTVRDIRGFATKFYTEEGIFDLVGNNTPIFFIQDAHKFPDFVHAVKPEPHWAIPQGQSAHD

                      .    :    .    :    .    :    .    :    .    :    .    :    .    :
Chain 1:  238 TFWDYVSLQPETLHNVMWAMSDRGIPRSYRTMEGFGIHTFRLINAEGKATFVRFHWKPLAGKASLVWDEA
              1111111111111111111111111111111111111111111111111111111111111111111111
Chain 2:  238 TFWDYVSLQPETLHNVMWAMSDRGIPRSYRTMEGFGIHTFRLINAEGKATFVRFHWKPLAGKASLVWDEA

                      .    :    .    :    .    :    .    :    .    :    .    :    .    :
Chain 1:  308 QKLTGRDPDFHRRELWEAIEAGDFPEYELGFQLIPEEDEFKFDFDLLDPTKLIPEELVPVQRVGKMVLNR
              1111111111111111111111111111111111111111111111111111111111111111111111
Chain 2:  308 QKLTGRDPDFHRRELWEAIEAGDFPEYELGFQLIPEEDEFKFDFDLLDPTKLIPEELVPVQRVGKMVLNR

                      .    :    .    :    .    :    .    :    .    :    .    :    .    :
Chain 1:  378 NPDNFFAENEQAAFHPGHIVPGLDFTNDPLLQGRLFSYTDTQISRLGGPNFHEIPINRPTCPYHNFQRDG
              1111111111111111111111111111111111111111111111111111111111111111111111
Chain 2:  378 NPDNFFAENEQAAFHPGHIVPGLDFTNDPLLQGRLFSYTDTQISRLGGPNFHEIPINRPTCPYHNFQRDG

                      .    :    .    :    .    :    .    :    .    :    .    :    .    :
Chain 1:  448 MHRMGIDTNPANYEPNSINDNWPRETPPGPKRGGFESYQERVEGNKVRERSPSFGEYYSHPRLFWLSQTP
              1111111111111111111111111111111111111111111111111111111111111111111111
Chain 2:  448 MHRMGIDTNPANYEPNSINDNWPRETPPGPKRGGFESYQERVEGNKVRERSPSFGEYYSHPRLFWLSQTP

                      .    :    .    :    .    :    .    :    .    :    .    :    .    :
Chain 1:  518 FEQRHIVDGFSFELSKVVRPYIRERVVDQLAHIDLTLAQAVAKNLGIELTDDQLNITPPPDVNGLKKDPS
              1111111111111111111111111111111111111111111111111111111111111111111111
Chain 2:  518 FEQRHIVDGFSFQLSKVVRPYIRERVVDQLAHIDLTLAQAVAKNLGIELTDDQLNITPPPDVNGLKKDPS

                      .    :    .    :    .    :    .    :    .    :    .    :    .    :
Chain 1:  588 LSLYAIPDGDVKGRVVAILLNDEVRSADLLAILKALKAKGVHAKLLYSRMGEVTADDGTVLPIAATFAGA
              1111111111111111111111111111111111111111111111111111111111111111111111
Chain 2:  588 LSLYAIPDGDVKGRVVAILLNDEVRSADLLAILKALKAKGVHAKLLYSRMGEVTADDGTVLPIAATFAGA

                      .    :    .    :    .    :    .    :    .    :    .    :    .    :
Chain 1:  658 PSLTVDAVIVPCGNIADIADNGDANYYLMEAYKHLKPIALAGDARKFKATIKIADQGEEGIVEADSADGS
              1111111111111111111111111111111111111111111111111111111111111111111111
Chain 2:  658 PSLTVDAVIVPCGNIADIADNGDANYYLMEAYKHLKPIALAGDARKFKATIKIADQGEEGIVEADSADGS

                      .    :    .    :    .
Chain 1:  728 FMDELLTLMAAHRVWSRIPKIDKIPA
              11111111111111111111111111
Chain 2:  728 FMDELLTLMAAHRVWSRIPKIDKIPA
```

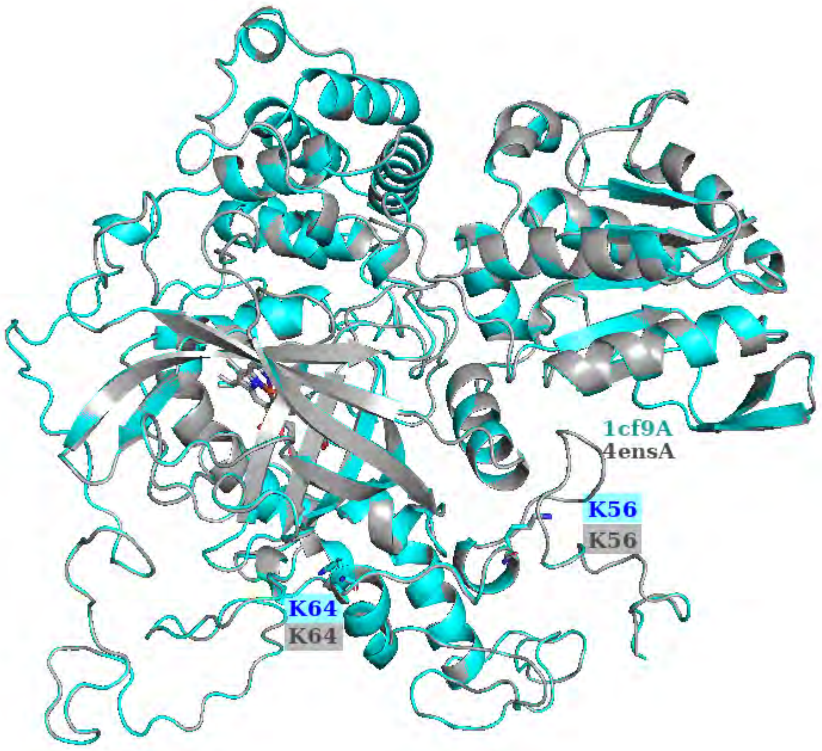

UniProt ID: P21179
PDB ID: 4ENT_B

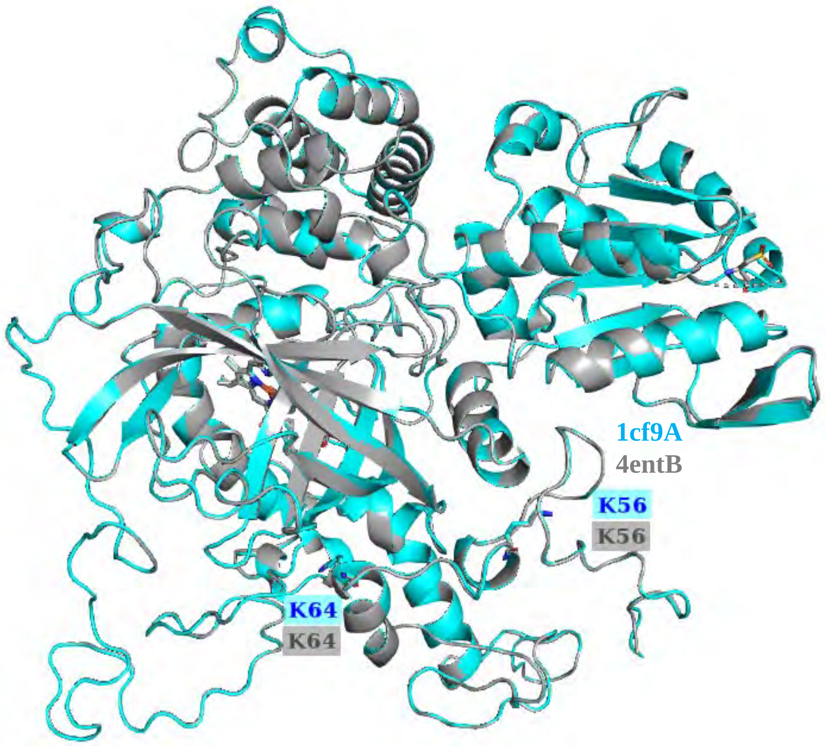

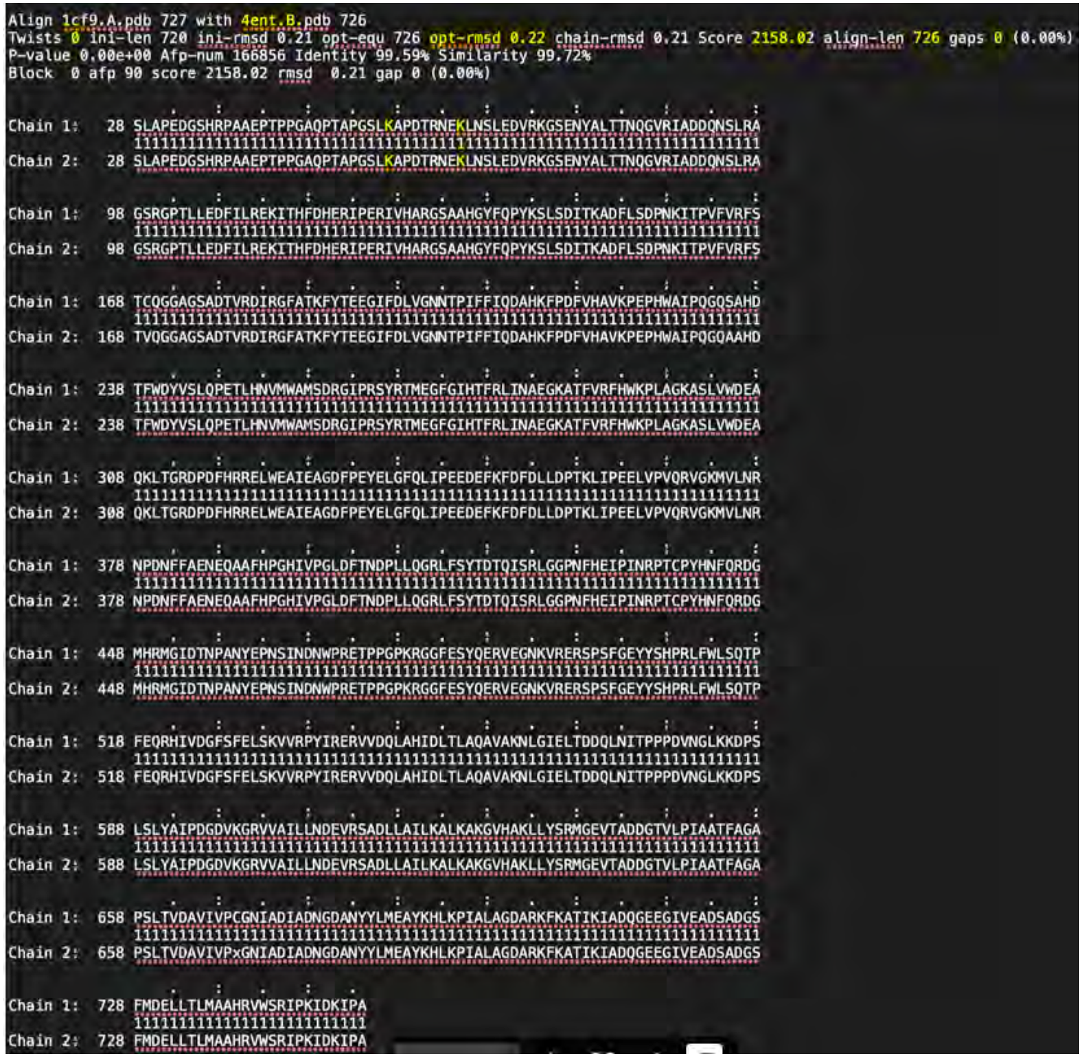

UniProt ID: P21179

PDB ID: 4ENU_A

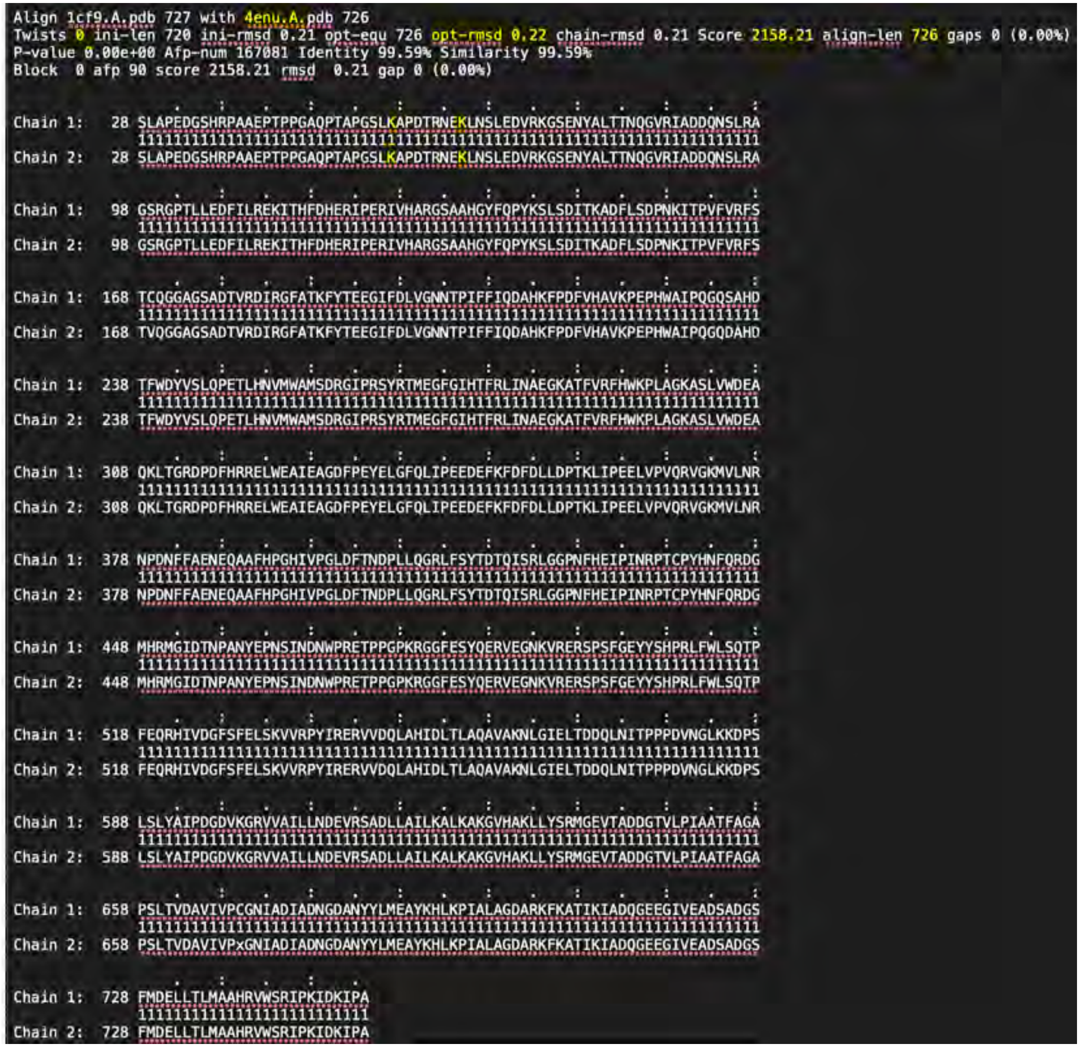

```
Align 1cf9.A.pdb 727 with 4enu.A.pdb 726
Twists 0 ini-len 720 ini-rmsd 0.21 opt-equ 726 opt-rmsd 0.22 chain-rmsd 0.21 Score 2158.21 align-len 726 gaps 0 (0.00%)
P-value 0.00e+00 Afp-num 167081 Identity 99.59% Similarity 99.59%
Block  0 afp 90 score 2158.21 rmsd  0.21 gap 0 (0.00%)

Chain 1:   28 SLAPEDGSHRPAAEPTPPGAQPTAPGSLKAPDTRNEKLNSLEDVRKGSENYALTTNQGVRIADDQNSLRA
              1111111111111111111111111111111111111111111111111111111111111111111111
Chain 2:   28 SLAPEDGSHRPAAEPTPPGAQPTAPGSLKAPDTRNEKLNSLEDVRKGSENYALTTNQGVRIADDQNSLRA

Chain 1:   98 GSRGPTLLEDFILREKITHFDHERIPERIVHARGSAAHGYFQPYKSLSDITKADFLSDPNKITPVFVRFS
              1111111111111111111111111111111111111111111111111111111111111111111111
Chain 2:   98 GSRGPTLLEDFILREKITHFDHERIPERIVHARGSAAHGYFQPYKSLSDITKADFLSDPNKITPVFVRFS

Chain 1:  168 TCQGGAGSADTVRDIRGFATKFYTEEGIFDLVGNNTPIFFIQDAHKFPDFVHAVKPEPHWAIPQGQSAHD
              1111111111111111111111111111111111111111111111111111111111111111111111
Chain 2:  168 TVQGGAGSADTVRDIRGFATKFYTEEGIFDLVGNNTPIFFIQDAHKFPDFVHAVKPEPHWAIPQGQDAHD

Chain 1:  238 TFWDYVSLQPETLHNVMWAMSDRGIPRSYRTMEGFGIHTFRLINAEGKATFVRFHWKPLAGKASLVWDEA
              1111111111111111111111111111111111111111111111111111111111111111111111
Chain 2:  238 TFWDYVSLQPETLHNVMWAMSDRGIPRSYRTMEGFGIHTFRLINAEGKATFVRFHWKPLAGKASLVWDEA

Chain 1:  308 QKLTGRDPDFHRRELWEAIEAGDFPEYELGFQLIPEEDEFKFDFDLLDPTKLIPEELVPVQRVGKMVLNR
              1111111111111111111111111111111111111111111111111111111111111111111111
Chain 2:  308 QKLTGRDPDFHRRELWEAIEAGDFPEYELGFQLIPEEDEFKFDFDLLDPTKLIPEELVPVQRVGKMVLNR

Chain 1:  378 NPDNFFAENEQAAFHPGHIVPGLDFTNDPLLQGRLFSYTDTQISRLGGPNFHEIPINRPTCPYHNFQRDG
              1111111111111111111111111111111111111111111111111111111111111111111111
Chain 2:  378 NPDNFFAENEQAAFHPGHIVPGLDFTNDPLLQGRLFSYTDTQISRLGGPNFHEIPINRPTCPYHNFQRDG

Chain 1:  448 MHRMGIDTNPANYEPNSINDNWPRETPPGPKRGGFESYQERVEGNKVRERSPSFGEYYSHPRLFWLSQTP
              1111111111111111111111111111111111111111111111111111111111111111111111
Chain 2:  448 MHRMGIDTNPANYEPNSINDNWPRETPPGPKRGGFESYQERVEGNKVRERSPSFGEYYSHPRLFWLSQTP

Chain 1:  518 FEQRHIVDGFSFELSKVVRPYIRERVVDQLAHIDLTLAQAVAKNLGIELTDDQLNITPPPDVNGLKKDPS
              1111111111111111111111111111111111111111111111111111111111111111111111
Chain 2:  518 FEQRHIVDGFSFELSKVVRPYIRERVVDQLAHIDLTLAQAVAKNLGIELTDDQLNITPPPDVNGLKKDPS

Chain 1:  588 LSLYAIPDGDVKGRVVAILLNDEVRSADLLAILKALKAKGVHAKLLYSRMGEVTADDGTVLPIAATFAGA
              1111111111111111111111111111111111111111111111111111111111111111111111
Chain 2:  588 LSLYAIPDGDVKGRVVAILLNDEVRSADLLAILKALKAKGVHAKLLYSRMGEVTADDGTVLPIAATFAGA

Chain 1:  658 PSLTVDAVIVPCGNIADIADNGDANYYLMEAYKHLKPIALAGDARKFKATIKIADQGEEGIVEADSADGS
              1111111111111111111111111111111111111111111111111111111111111111111111
Chain 2:  658 PSLTVDAVIVPxGNIADIADNGDANYYLMEAYKHLKPIALAGDARKFKATIKIADQGEEGIVEADSADGS

Chain 1:  728 FMDELLTLMAAHRVWSRIPKIDKIPA
              11111111111111111111111111
Chain 2:  728 FMDELLTLMAAHRVWSRIPKIDKIPA
```

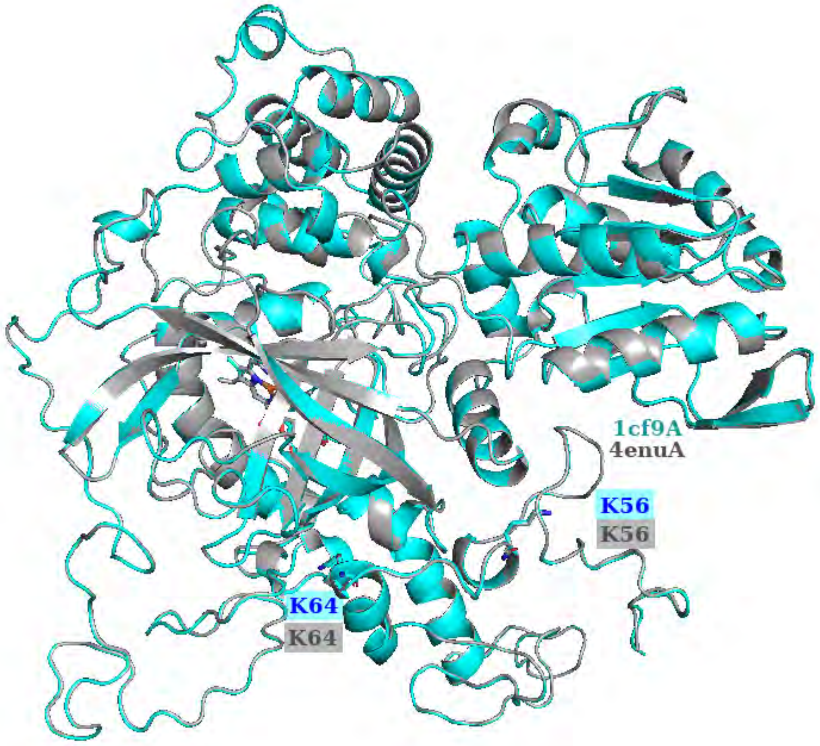

UniProt ID: P21179
PDB ID: 4ENV_B

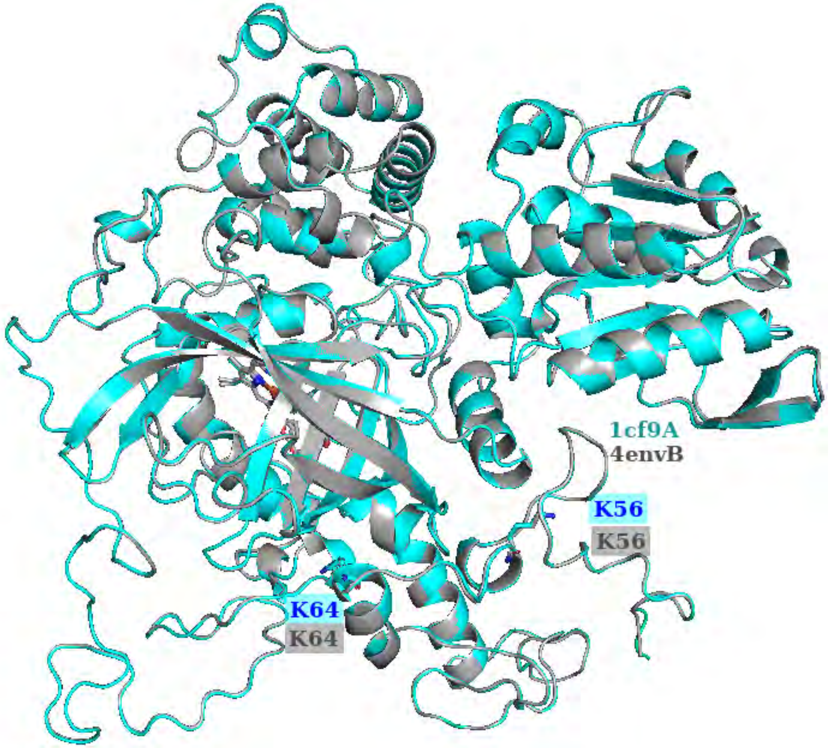

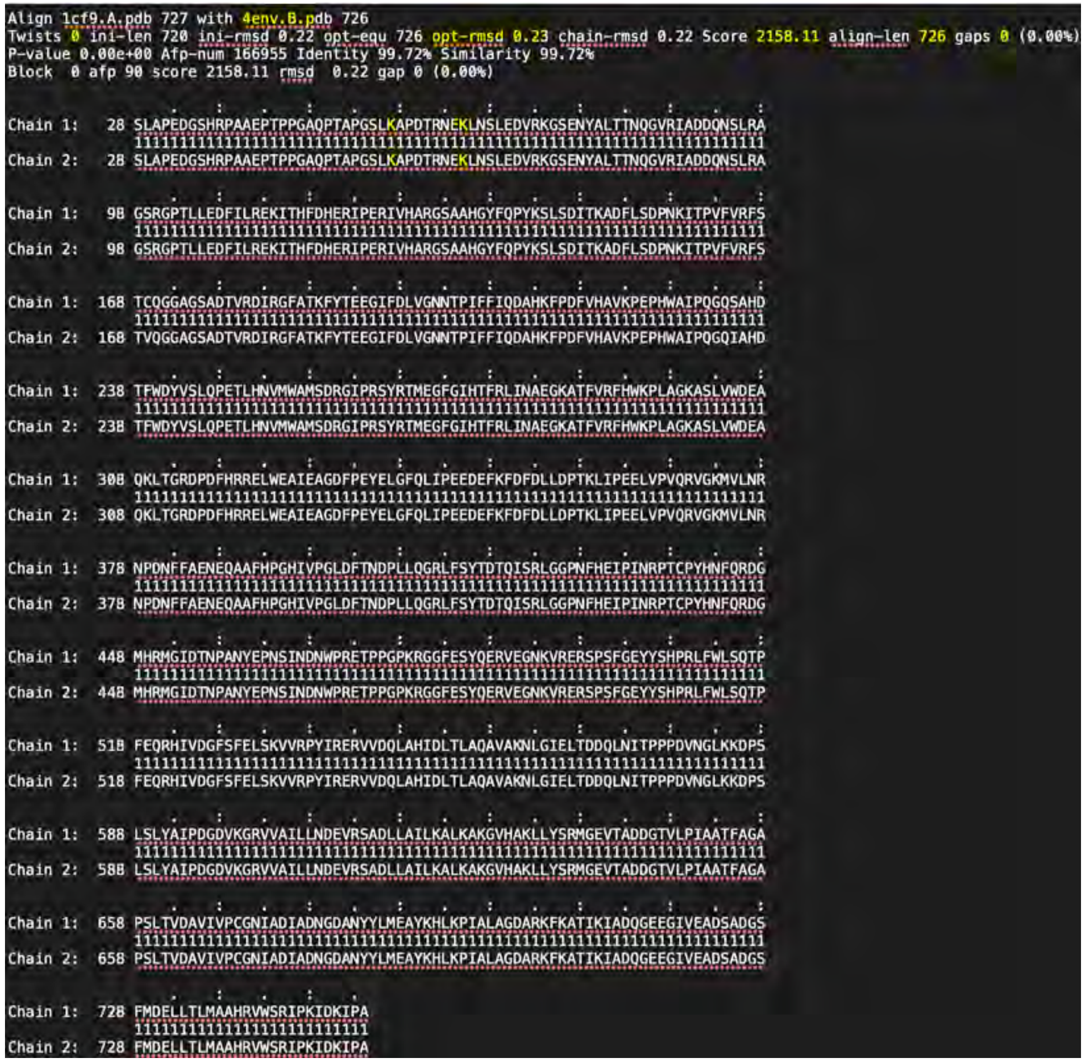

UniProt ID: P21179

PDB ID: 4ENW_B

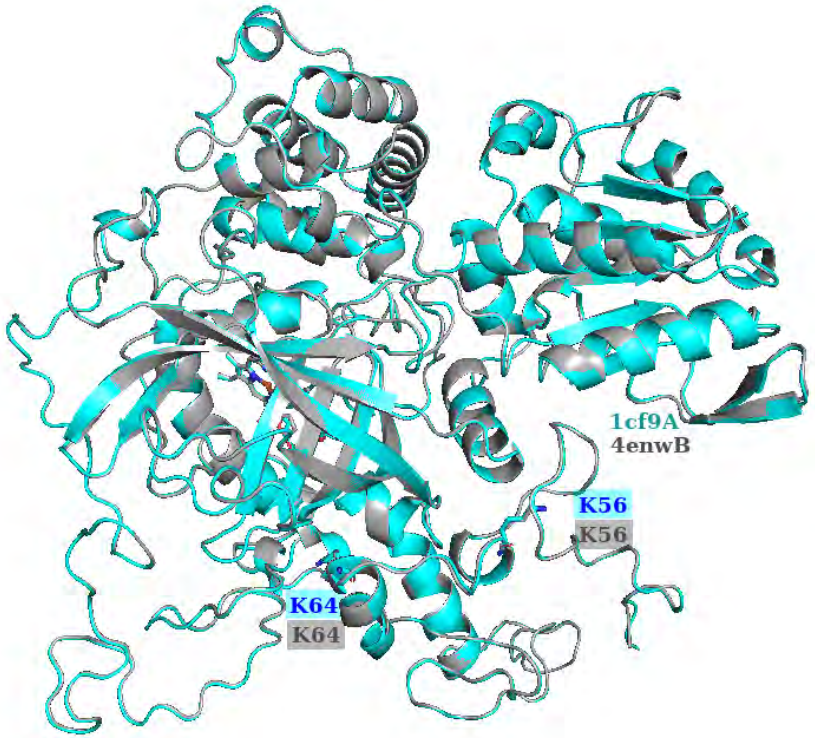

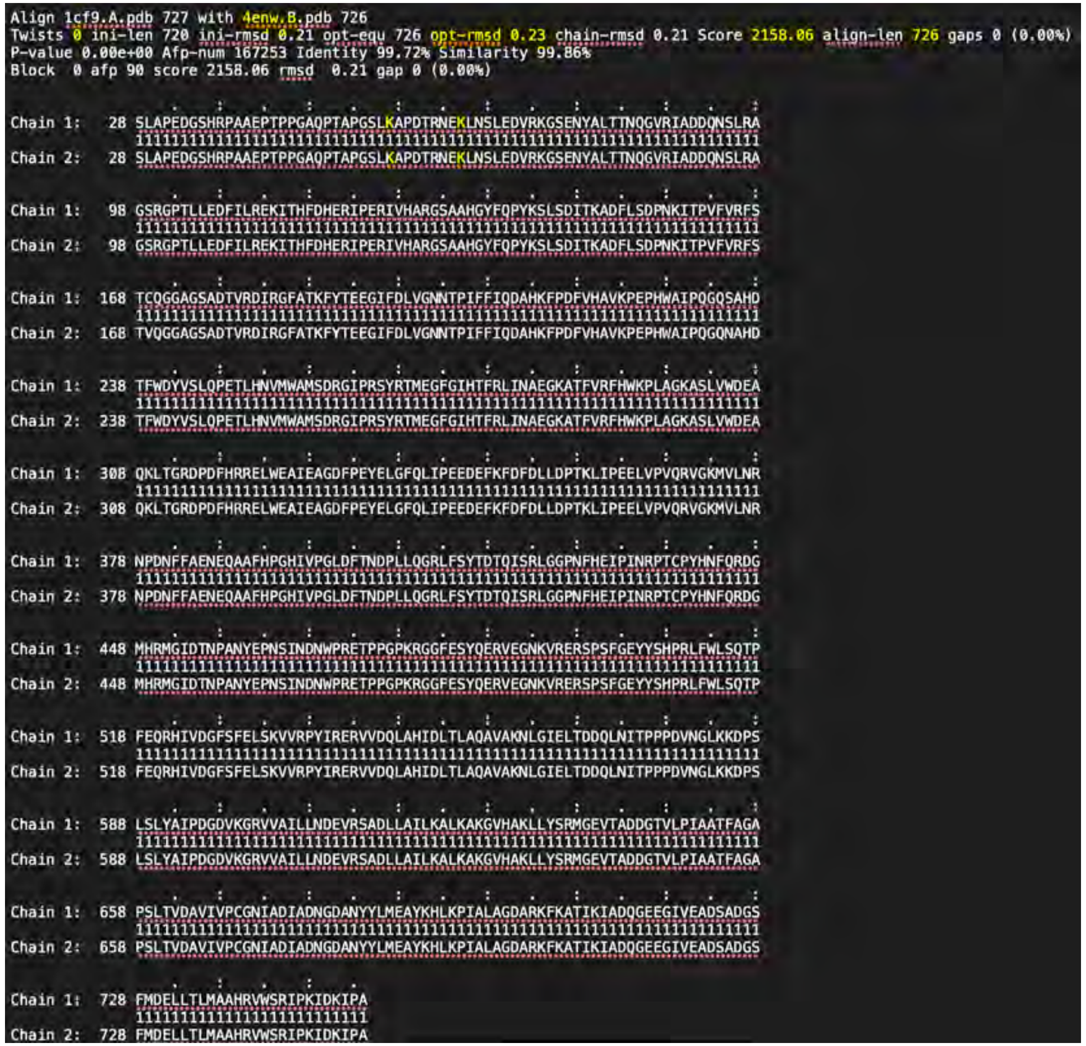

```
Align 1cf9.A.pdb 727 with 4enw.B.pdb 726
Twists 0 ini-len 720 ini-rmsd 0.21 opt-equ 726 opt-rmsd 0.23 chain-rmsd 0.21 Score 2158.06 align-len 726 gaps 0 (0.00%)
P-value 0.00e+00 Afp-num 167253 Identity 99.72% Similarity 99.86%
Block  0 afp 90 score 2158.06 rmsd  0.21 gap 0 (0.00%)

Chain 1:   28 SLAPEDGSHRPAAEPTPPGAQPTAPGSLKAPDTRNEKLNSLEDVRKGSENYALTTNQGVRIADDQNSLRA
              1111111111111111111111111111111111111111111111111111111111111111111111
Chain 2:   28 SLAPEDGSHRPAAEPTPPGAQPTAPGSLKAPDTRNEKLNSLEDVRKGSENYALTTNQGVRIADDQNSLRA

Chain 1:   98 GSRGPTLLEDFILREKITHFDHERIPERIVHARGSAAHGYFQPYKSLSDITKADFLSDPNKITPVFVRFS
              1111111111111111111111111111111111111111111111111111111111111111111111
Chain 2:   98 GSRGPTLLEDFILREKITHFDHERIPERIVHARGSAAHGYFQPYKSLSDITKADFLSDPNKITPVFVRFS

Chain 1:  168 TCQGGAGSADTVRDIRGFATKFYTEEGIFDLVGNNTPIFFIQDAHKFPDFVHAVKPEPHWAIPQGQSAHD
              1111111111111111111111111111111111111111111111111111111111111111111111
Chain 2:  168 TVQGGAGSADTVRDIRGFATKFYTEEGIFDLVGNNTPIFFIQDAHKFPDFVHAVKPEPHWAIPQGQNAHD

Chain 1:  238 TFWDYVSLQPETLHNVMWAMSDRGIPRSYRTMEGFGIHTFRLINAEGKATFVRFHWKPLAGKASLVWDEA
              1111111111111111111111111111111111111111111111111111111111111111111111
Chain 2:  238 TFWDYVSLQPETLHNVMWAMSDRGIPRSYRTMEGFGIHTFRLINAEGKATFVRFHWKPLAGKASLVWDEA

Chain 1:  308 QKLTGRDPDFHRRELWEAIEAGDFPEYELGFQLIPEEDEFKFDFDLLDPTKLIPEELVPVQRVGKMVLNR
              1111111111111111111111111111111111111111111111111111111111111111111111
Chain 2:  308 QKLTGRDPDFHRRELWEAIEAGDFPEYELGFQLIPEEDEFKFDFDLLDPTKLIPEELVPVQRVGKMVLNR

Chain 1:  378 NPDNFFAENEQAAFHPGHIVPGLDFTNDPLLQGRLFSYTDTQISRLGGPNFHEIPINRPTCPYHNFQRDG
              1111111111111111111111111111111111111111111111111111111111111111111111
Chain 2:  378 NPDNFFAENEQAAFHPGHIVPGLDFTNDPLLQGRLFSYTDTQISRLGGPNFHEIPINRPTCPYHNFQRDG

Chain 1:  448 MHRMGIDTNPANYEPNSINDNWPRETPPGPKRGGFESYQERVEGNKVRERSPSFGEYYSHPRLFWLSQTP
              1111111111111111111111111111111111111111111111111111111111111111111111
Chain 2:  448 MHRMGIDTNPANYEPNSINDNWPRETPPGPKRGGFESYQERVEGNKVRERSPSFGEYYSHPRLFWLSQTP

Chain 1:  518 FEQRHIVDGFSFELSKVVRPYIRERVVDQLAHIDLTLAQAVAKNLGIELTDDQLNITPPPDVNGLKKDPS
              1111111111111111111111111111111111111111111111111111111111111111111111
Chain 2:  518 FEQRHIVDGFSFELSKVVRPYIRERVVDQLAHIDLTLAQAVAKNLGIELTDDQLNITPPPDVNGLKKDPS

Chain 1:  588 LSLYAIPDGDVKGRVVAILLNDEVRSADLLAILKALKAKGVHAKLLYSRMGEVTADDGTVLPIAATFAGA
              1111111111111111111111111111111111111111111111111111111111111111111111
Chain 2:  588 LSLYAIPDGDVKGRVVAILLNDEVRSADLLAILKALKAKGVHAKLLYSRMGEVTADDGTVLPIAATFAGA

Chain 1:  658 PSLTVDAVIVPCGNIADIADNGDANYYLMEAYKHLKPIALAGDARKFKATIKIADQGEEGIVEADSADGS
              1111111111111111111111111111111111111111111111111111111111111111111111
Chain 2:  658 PSLTVDAVIVPCGNIADIADNGDANYYLMEAYKHLKPIALAGDARKFKATIKIADQGEEGIVEADSADGS

Chain 1:  728 FMDELLTLMAAHRVWSRIPKIDKIPA
              11111111111111111111111111
Chain 2:  728 FMDELLTLMAAHRVWSRIPKIDKIPA
```

UniProt ID: P21179
PDB ID: 5BV2_P

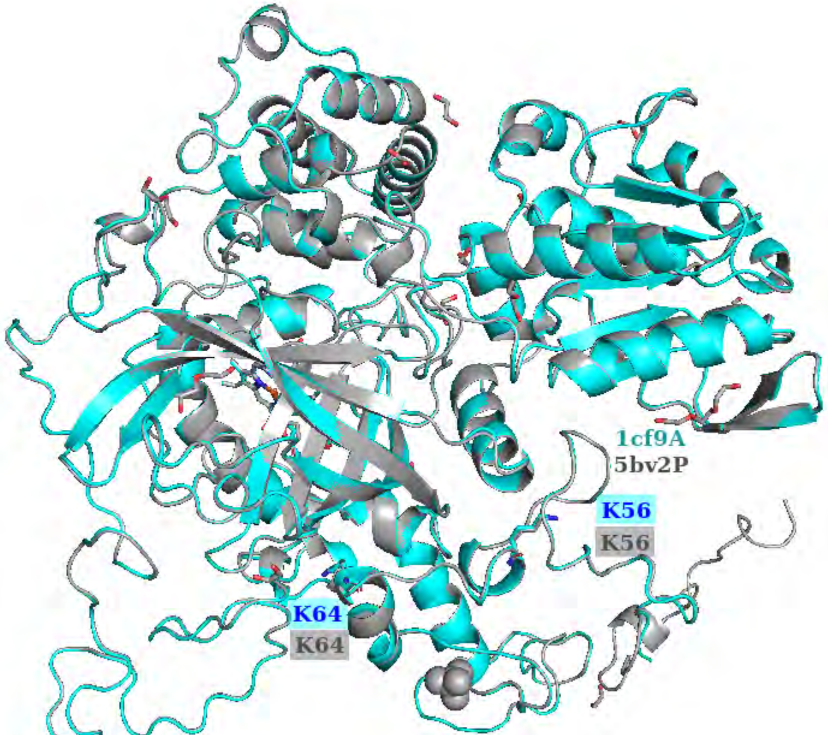


```
Align 1cf9.A.pdb 727 with 5bv2.P.pdb 747
Twists 0 ini-len 720 ini-rmsd 0.24 opt-equ 727 opt-rmsd 0.30 chain-rmsd 0.24 Score 2156.84 align-len 727 gaps 0 (0.00%)
P-value 0.00e+00 Afp-num 173083 Identity 99.04% Similarity 99.17%
Block  0 afp 90 score 2156.84 rmsd  0.24 gap 1 (0.00%)

                  .    :    .    :    .    :    .    :    .    :    .    :    .    :
Chain 1:   27 DSLAPEDGSHRPAAEPTPPGAQPTAPGSLKAPDTRNEKLNSLEDVRKGSENYALTTNQGVRIADDQNSLR
              1111111111111111111111111111111111111111111111111111111111111111111111
Chain 2:   27 DSLAPEDGSHRPAAEPTPPGAQPTAPGSLKAPDTRNEKLNSLEDVRKGSENYALTTNQGVRIADDQNSLR

                  .    :    .    :    .    :    .    :    .    :    .    :    .    :
Chain 1:   97 AGSRGPTLLEDFILREKITHFDHERIPERIVHARGSAAHGYFQPYKSLSDITKADFLSDPNKITPVFVRF
              1111111111111111111111111111111111111111111111111111111111111111111111
Chain 2:   97 AGDRGPTLLEDFILREKITHFDHERIPERIVHARGSAAHGYFQPYKSLSDITKADFLSDPNKITPVFVRF

                  .    :    .    :    .    :    .    :    .    :    .    :    .    :
Chain 1:  167 STCQGGAGSADTVRDIRGFATKFYTEEGIFDLVGNNTPIFFIQDAHKFPDFVHAVKPEPHWAIPQGQSAH
              1111111111111111111111111111111111111111111111111111111111111111111111
Chain 2:  167 STVQGGAGSADTVRDIRGFATKFYTEEGIFDLVGNNTPIFFIQDAHKFPDFVHAVKPEPHWAIPQGQSAH

                  .    :    .    :    .    :    .    :    .    :    .    :    .    :
Chain 1:  237 DTFWDYVSLQPETLHNVMWAMSDRGIPRSYRTMEGFGIHTFRLINAEGKATFVRFHWKPLAGKASLVWDE
              1111111111111111111111111111111111111111111111111111111111111111111111
Chain 2:  237 DTFWDYVSLQPETLHNVMWAMSDRGIPRSYRTMEGFGIHTFRLINASGKATFVRFHWKPLAGKASLVWDE

                  .    :    .    :    .    :    .    :    .    :    .    :    .    :
Chain 1:  307 AQKLTGRDPDFHRRELWEAIEAGDFPEYELGFQLIPEEDEFKFDFDLLDPTKLIPEELVPVQRVGKMVLN
              1111111111111111111111111111111111111111111111111111111111111111111111
Chain 2:  307 AQKLTGRDPDFHRRELWEAIEAGDFPEYELGFQLIPEEDEFKFDFDLLDPTKLIPEELVPVQRVGNMVLN

                  .    :    .    :    .    :    .    :    .    :    .    :    .    :
Chain 1:  377 RNPDNFFAENEQAAFHPGHIVPGLDFTNDPLLQGRLFSYTDTQISRLGGPNFHEIPINRPTCPYHNFQRD
              1111111111111111111111111111111111111111111111111111111111111111111111
Chain 2:  377 RNPDNFFAENEQAAFHPGHIVPGLDFTNDPLLQGRLFSYTDTQISRLGGPNFHEIPINRPTCPYHNFQRD

                  .    :    .    :    .    :    .    :    .    :    .    :    .    :
Chain 1:  447 GMHRMGIDTNPANYEPNSINDNWPRETPPGPKRGGFESYQERVEGNKVRERSPSFGEYYSHPRLFWLSQT
              1111111111111111111111111111111111111111111111111111111111111111111111
Chain 2:  447 GMHRMGIDTNPANYEPNSINDNWPRETPPGPKRGGFESYQERVEGNKVRERSPSFGEYYSHPRLFWLSQT

                  .    :    .    :    .    :    .    :    .    :    .    :    .    :
Chain 1:  517 PFEQRHIVDGFSFELSKVVRPYIRERVVDQLAHIDLTLAQAVAKNLGIELTDDQLNITPPPDVNGLKKDP
              1111111111111111111111111111111111111111111111111111111111111111111111
Chain 2:  517 PFEQRHIVDGFSFELSKVVRPYIRERVVDQLAHIDLTLAQAVAKNLGISLTDDQLNITPPPDVNGLKKDP

                  .    :    .    :    .    :    .    :    .    :    .    :    .    :
Chain 1:  587 SLSLYAIPDGDVKGRVVAILLNDEVRSADLLAILKALKAKGVHAKLLYSRMGEVTADDGTVLPIAATFAG
              1111111111111111111111111111111111111111111111111111111111111111111111
Chain 2:  587 SLSLYAIPDGDVKGRVVAILLNDEVRSADLLAILKALKAKGVHAKLLYSRMGEVTADDGTVLPIAATFAG

                  .    :    .    :    .    :    .    :    .    :    .    :    .    :
Chain 1:  657 APSLTVDAVIVPCGNIADIADNGDANYYLMEAYKHLKPIALAGDARKFKATIKIADQGEEGIVEADSADG
              1111111111111111111111111111111111111111111111111111111111111111111111
Chain 2:  657 APSLTVDAVIVPCGNIADIADNGDANYYLMEAYKHLKPIALAGDARKFKATIKVADQGEEGIVEADSADG

                  .    :    .    :    .
Chain 1:  727 SFMDELLTLMAAHRVWSRIPKIDKIPA
              111111111111111111111111111
Chain 2:  727 SFMDELLTLMAAHRVWSRIPKIDSIPA
```

UniProt ID: P21179
PDB ID: 6BY0_A

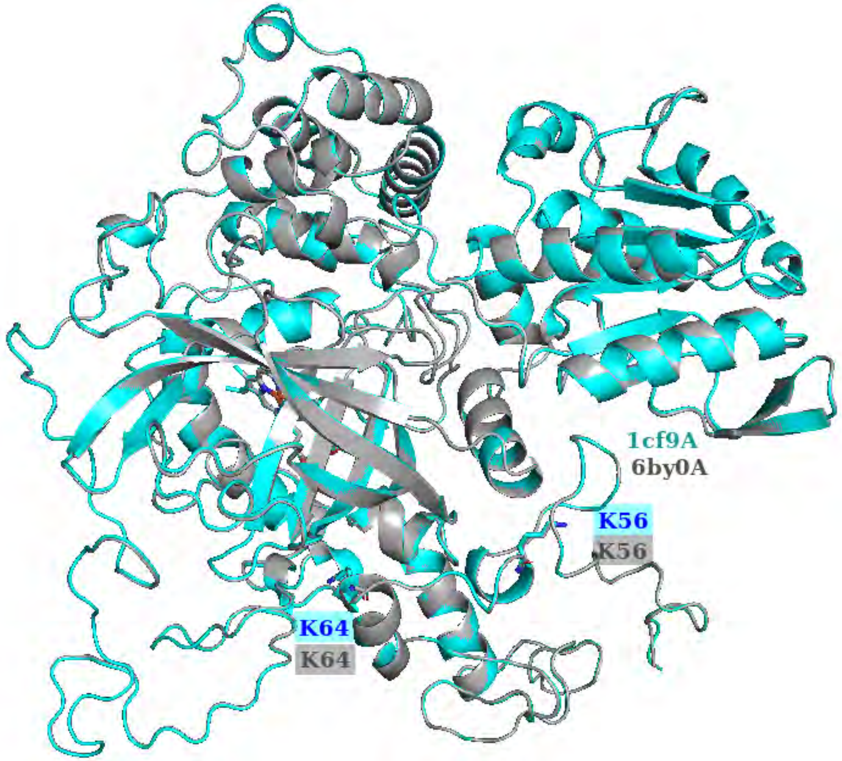

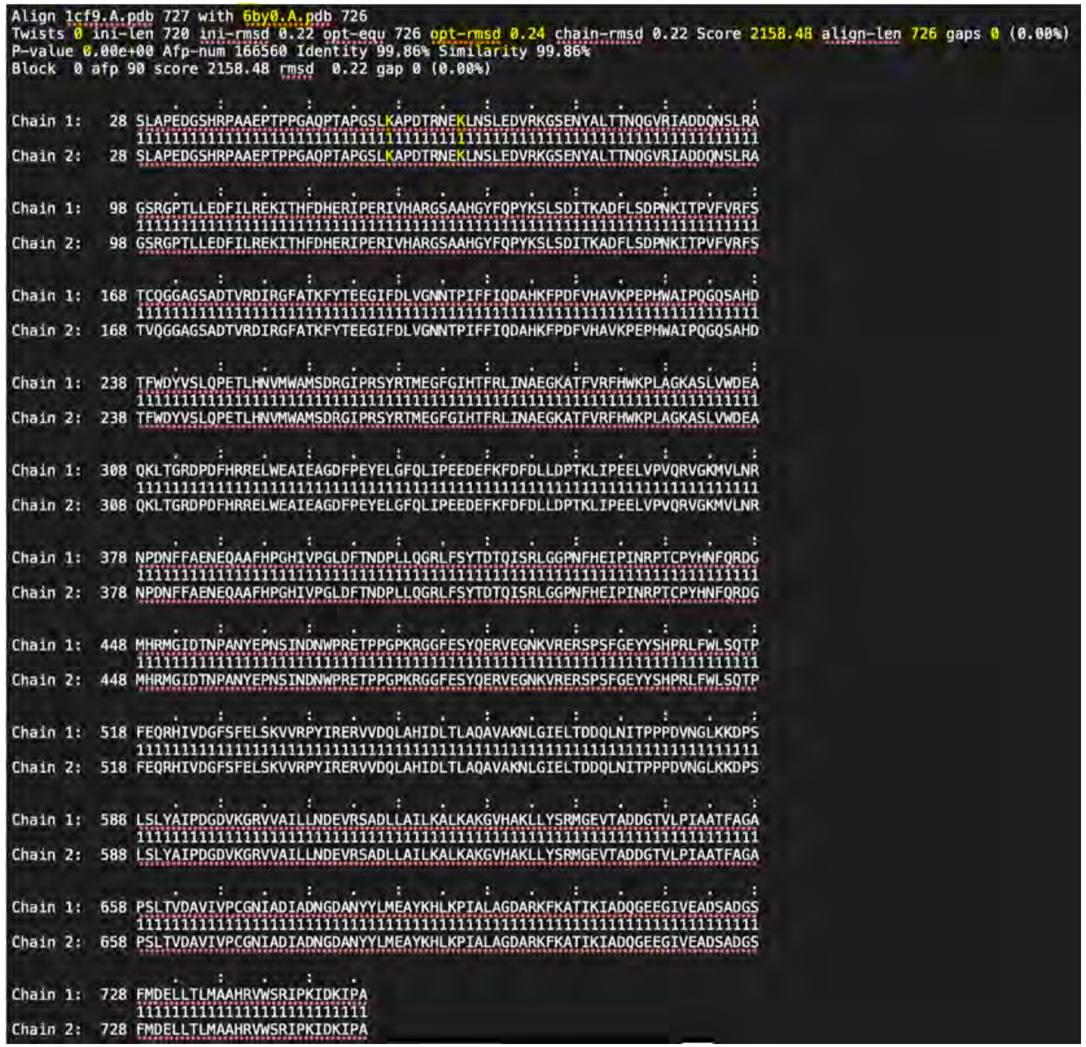

```
Align 1cf9.A.pdb 727 with 6by0.A.pdb 726
Twists 0 ini-len 720 ini-rmsd 0.22 opt-equ 726 opt-rmsd 0.24 chain-rmsd 0.22 Score 2158.48 align-len 726 gaps 0 (0.00%)
P-value 0.00e+00 Afp-num 166560 Identity 99.86% Similarity 99.86%
Block  0 afp 90 score 2158.48 rmsd  0.22 gap 0 (0.00%)

                   .    :    .    :    .    :    .    :    .    :    .    :    .    :
Chain 1:   28 SLAPEDGSHRPAAEPTPPGAQPTAPGSLKAPDTRNEKLNSLEDVRKGSENYALTTNQGVRIADDQNSLRA
              1111111111111111111111111111111111111111111111111111111111111111111111
Chain 2:   28 SLAPEDGSHRPAAEPTPPGAQPTAPGSLKAPDTRNEKLNSLEDVRKGSENYALTTNQGVRIADDQNSLRA

                   .    :    .    :    .    :    .    :    .    :    .    :    .    :
Chain 1:   98 GSRGPTLLEDFILREKITHFDHERIPERIVHARGSAAHGYFQPYKSLSDITKADFLSDPNKITPVFVRFS
              1111111111111111111111111111111111111111111111111111111111111111111111
Chain 2:   98 GSRGPTLLEDFILREKITHFDHERIPERIVHARGSAAHGYFQPYKSLSDITKADFLSDPNKITPVFVRFS

                   .    :    .    :    .    :    .    :    .    :    .    :    .    :
Chain 1:  168 TCQGGAGSADTVRDIRGFATKFYTEEGIFDLVGNNTPIFFIQDAHKFPDFVHAVKPEPHWAIPQGQSAHD
              1111111111111111111111111111111111111111111111111111111111111111111111
Chain 2:  168 TVQGGAGSADTVRDIRGFATKFYTEEGIFDLVGNNTPIFFIQDAHKFPDFVHAVKPEPHWAIPQGQSAHD

                   .    :    .    :    .    :    .    :    .    :    .    :    .    :
Chain 1:  238 TFWDYVSLQPETLHNVMWAMSDRGIPRSYRTMEGFGIHTFRLINAEGKATFVRFHWKPLAGKASLVWDEA
              1111111111111111111111111111111111111111111111111111111111111111111111
Chain 2:  238 TFWDYVSLQPETLHNVMWAMSDRGIPRSYRTMEGFGIHTFRLINAEGKATFVRFHWKPLAGKASLVWDEA

                   .    :    .    :    .    :    .    :    .    :    .    :    .    :
Chain 1:  308 QKLTGRDPDFHRRELWEAIEAGDFPEYELGFQLIPEEDEFKFDFDLLDPTKLIPEELVPVQRVGKMVLNR
              1111111111111111111111111111111111111111111111111111111111111111111111
Chain 2:  308 QKLTGRDPDFHRRELWEAIEAGDFPEYELGFQLIPEEDEFKFDFDLLDPTKLIPEELVPVQRVGKMVLNR

                   .    :    .    :    .    :    .    :    .    :    .    :    .    :
Chain 1:  378 NPDNFFAENEQAAFHPGHIVPGLDFTNDPLLQGRLFSYTDTQISRLGGPNFHEIPINRPTCPYHNFQRDG
              1111111111111111111111111111111111111111111111111111111111111111111111
Chain 2:  378 NPDNFFAENEQAAFHPGHIVPGLDFTNDPLLQGRLFSYTDTQISRLGGPNFHEIPINRPTCPYHNFQRDG

                   .    :    .    :    .    :    .    :    .    :    .    :    .    :
Chain 1:  448 MHRMGIDTNPANYEPNSINDNWPRETPPGPKRGGFESYQERVEGNKVRERSPSFGEYYSHPRLFWLSQTP
              1111111111111111111111111111111111111111111111111111111111111111111111
Chain 2:  448 MHRMGIDTNPANYEPNSINDNWPRETPPGPKRGGFESYQERVEGNKVRERSPSFGEYYSHPRLFWLSQTP

                   .    :    .    :    .    :    .    :    .    :    .    :    .    :
Chain 1:  518 FEQRHIVDGFSFELSKVVRPYIRERVVDQLAHIDLTLAQAVAKNLGIELTDDQLNITPPPDVNGLKKDPS
              1111111111111111111111111111111111111111111111111111111111111111111111
Chain 2:  518 FEQRHIVDGFSFELSKVVRPYIRERVVDQLAHIDLTLAQAVAKNLGIELTDDQLNITPPPDVNGLKKDPS

                   .    :    .    :    .    :    .    :    .    :    .    :    .    :
Chain 1:  588 LSLYAIPDGDVKGRVVAILLNDEVRSADLLAILKALKAKGVHAKLLYSRMGEVTADDGTVLPIAATFAGA
              1111111111111111111111111111111111111111111111111111111111111111111111
Chain 2:  588 LSLYAIPDGDVKGRVVAILLNDEVRSADLLAILKALKAKGVHAKLLYSRMGEVTADDGTVLPIAATFAGA

                   .    :    .    :    .    :    .    :    .    :    .    :    .    :
Chain 1:  658 PSLTVDAVIVPCGNIADIADNGDANYYLMEAYKHLKPIALAGDARKFKATIKIADQGEEGIVEADSADGS
              1111111111111111111111111111111111111111111111111111111111111111111111
Chain 2:  658 PSLTVDAVIVPCGNIADIADNGDANYYLMEAYKHLKPIALAGDARKFKATIKIADQGEEGIVEADSADGS

                   .    :    .    :    .
Chain 1:  728 FMDELLTLMAAHRVWSRIPKIDKIPA
              11111111111111111111111111
Chain 2:  728 FMDELLTLMAAHRVWSRIPKIDKIPA
```

UniProt ID: P21179
PDB ID: 6ZTV_C

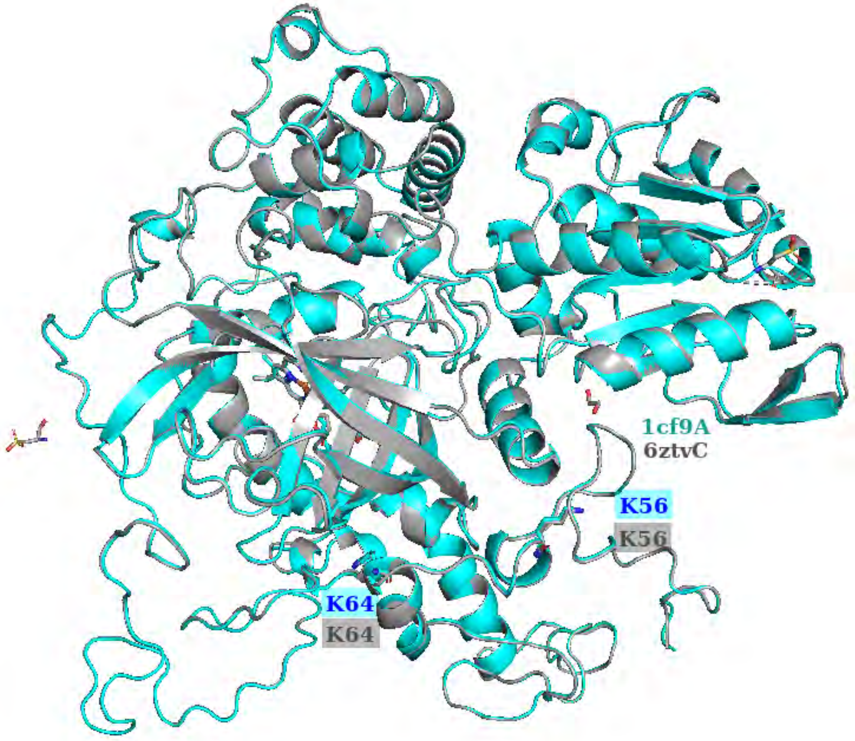

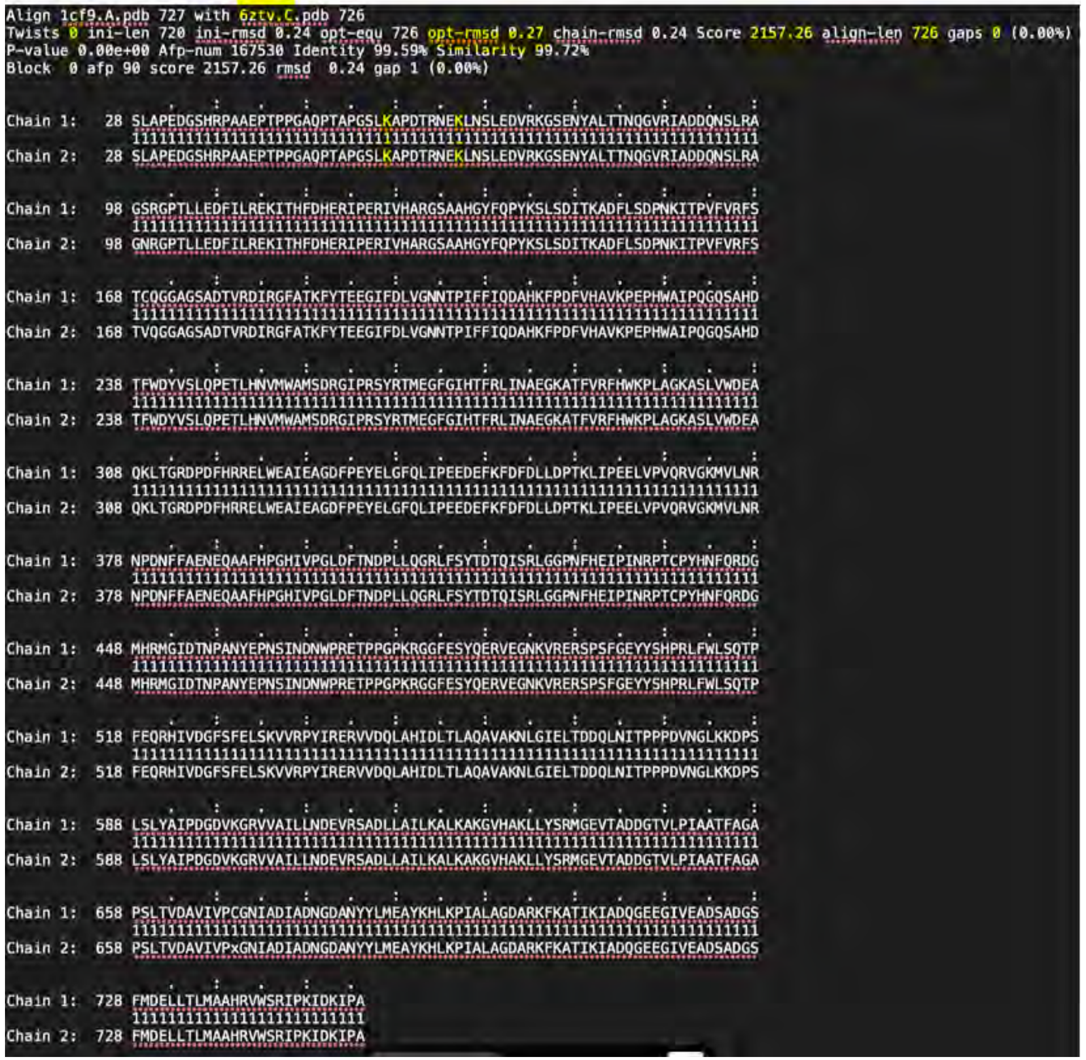

```
Align 1cf9.A.pdb 727 with 6ztv.C.pdb 726
Twists 0 ini-len 720 ini-rmsd 0.24 opt-equ 726 opt-rmsd 0.27 chain-rmsd 0.24 Score 2157.26 align-len 726 gaps 0 (0.00%)
P-value 0.00e+00 Afp-num 167530 Identity 99.59% Similarity 99.72%
Block  0 afp 90 score 2157.26 rmsd  0.24 gap 1 (0.00%)

Chain 1:   28 SLAPEDGSHRPAAEPTPPGAQPTAPGSLKAPDTRNEKLNSLEDVRKGSENYALTTNQGVRIADDQNSLRA
              1111111111111111111111111111111111111111111111111111111111111111111111
Chain 2:   28 SLAPEDGSHRPAAEPTPPGAQPTAPGSLKAPDTRNEKLNSLEDVRKGSENYALTTNQGVRIADDQNSLRA

Chain 1:   98 GSRGPTLLEDFILREKITHFDHERIPERIVHARGSAAHGYFQPYKSLSDITKADFLSDPNKITPVFVRFS
              1111111111111111111111111111111111111111111111111111111111111111111111
Chain 2:   98 GNRGPTLLEDFILREKITHFDHERIPERIVHARGSAAHGYFQPYKSLSDITKADFLSDPNKITPVFVRFS

Chain 1:  168 TCQGGAGSADTVRDIRGFATKFYTEEGIFDLVGNNTPIFFIQDAHKFPDFVHAVKPEPHWAIPQGQSAHD
              1111111111111111111111111111111111111111111111111111111111111111111111
Chain 2:  168 TVQGGAGSADTVRDIRGFATKFYTEEGIFDLVGNNTPIFFIQDAHKFPDFVHAVKPEPHWAIPQGQSAHD

Chain 1:  238 TFWDYVSLQPETLHNVMWAMSDRGIPRSYRTMEGFGIHTFRLINAEGKATFVRFHWKPLAGKASLVWDEA
              1111111111111111111111111111111111111111111111111111111111111111111111
Chain 2:  238 TFWDYVSLQPETLHNVMWAMSDRGIPRSYRTMEGFGIHTFRLINAEGKATFVRFHWKPLAGKASLVWDEA

Chain 1:  308 QKLTGRDPDFHRRELWEAIEAGDFPEYELGFQLIPEEDEFKFDFDLLDPTKLIPEELVPVQRVGKMVLNR
              1111111111111111111111111111111111111111111111111111111111111111111111
Chain 2:  308 QKLTGRDPDFHRRELWEAIEAGDFPEYELGFQLIPEEDEFKFDFDLLDPTKLIPEELVPVQRVGKMVLNR

Chain 1:  378 NPDNFFAENEQAAFHPGHIVPGLDFTNDPLLQGRLFSYTDTQISRLGGPNFHEIPINRPTCPYHNFQRDG
              1111111111111111111111111111111111111111111111111111111111111111111111
Chain 2:  378 NPDNFFAENEQAAFHPGHIVPGLDFTNDPLLQGRLFSYTDTQISRLGGPNFHEIPINRPTCPYHNFQRDG

Chain 1:  448 MHRMGIDTNPANYEPNSINDNWPRETPPGPKRGGFESYQERVEGNKVRERSPSFGEYYSHPRLFWLSQTP
              1111111111111111111111111111111111111111111111111111111111111111111111
Chain 2:  448 MHRMGIDTNPANYEPNSINDNWPRETPPGPKRGGFESYQERVEGNKVRERSPSFGEYYSHPRLFWLSQTP

Chain 1:  518 FEQRHIVDGFSFELSKVVRPYIRERVVDQLAHIDLTLAQAVAKNLGIELTDDQLNITPPPDVNGLKKDPS
              1111111111111111111111111111111111111111111111111111111111111111111111
Chain 2:  518 FEQRHIVDGFSFELSKVVRPYIRERVVDQLAHIDLTLAQAVAKNLGIELTDDQLNITPPPDVNGLKKDPS

Chain 1:  588 LSLYAIPDGDVKGRVVAILLNDEVRSADLLAILKALKAKGVHAKLLYSRMGEVTADDGTVLPIAATFAGA
              1111111111111111111111111111111111111111111111111111111111111111111111
Chain 2:  588 LSLYAIPDGDVKGRVVAILLNDEVRSADLLAILKALKAKGVHAKLLYSRMGEVTADDGTVLPIAATFAGA

Chain 1:  658 PSLTVDAVIVPCGNIADIADNGDANYYLMEAYKHLKPIALAGDARKFKATIKIADQGEEGIVEADSADGS
              1111111111111111111111111111111111111111111111111111111111111111111111
Chain 2:  658 PSLTVDAVIVPxGNIADIADNGDANYYLMEAYKHLKPIALAGDARKFKATIKIADQGEEGIVEADSADGS

Chain 1:  728 FMDELLTLMAAHRVWSRIPKIDKIPA
              11111111111111111111111111
Chain 2:  728 FMDELLTLMAAHRVWSRIPKIDKIPA
```

UniProt ID: P21179

PDB ID: 6ZTW_E

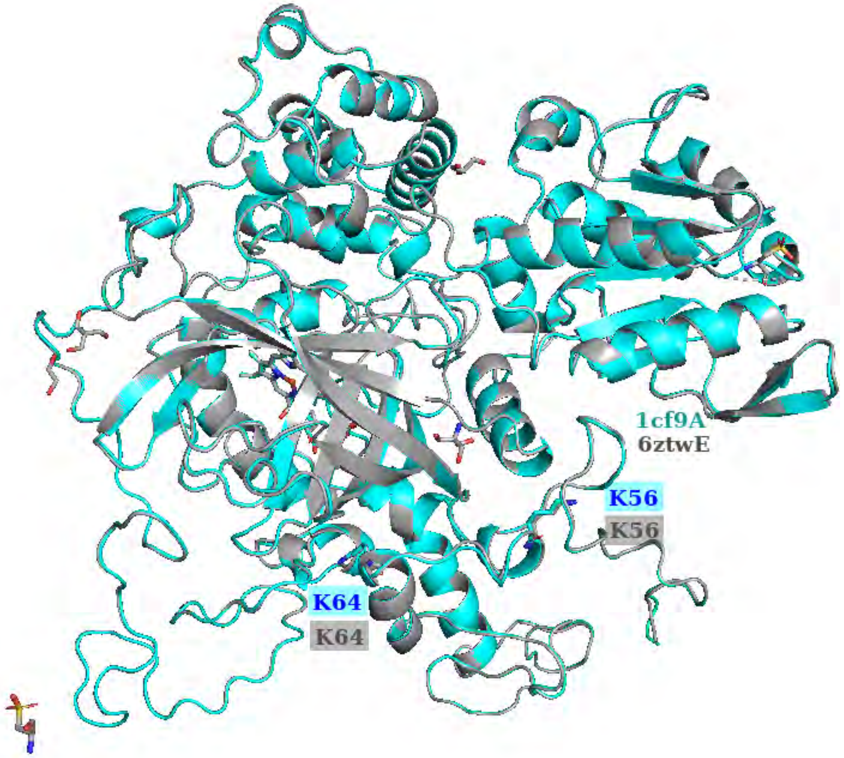

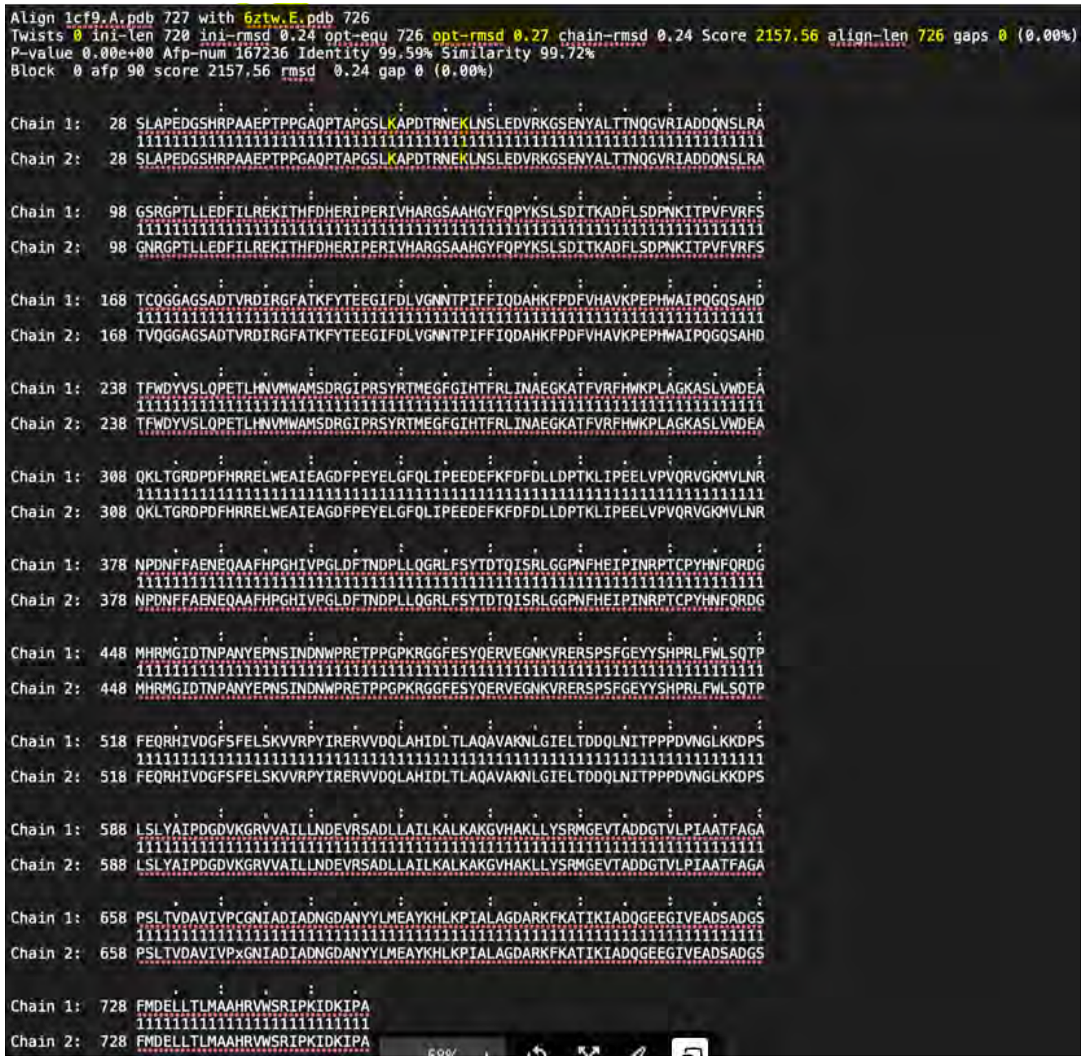

UniProt ID: P21179
PDB ID: 6ZTX_B

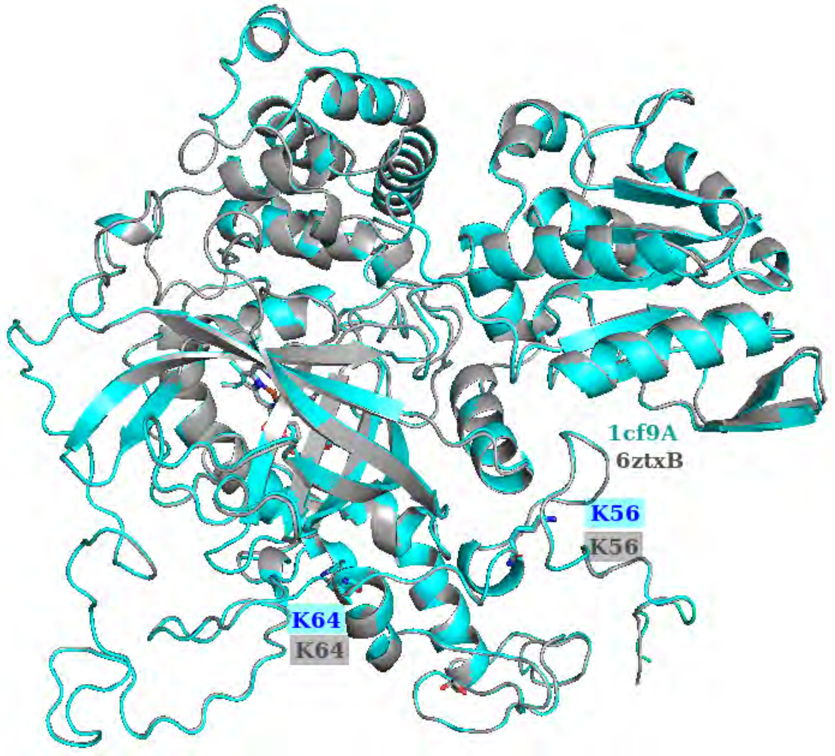

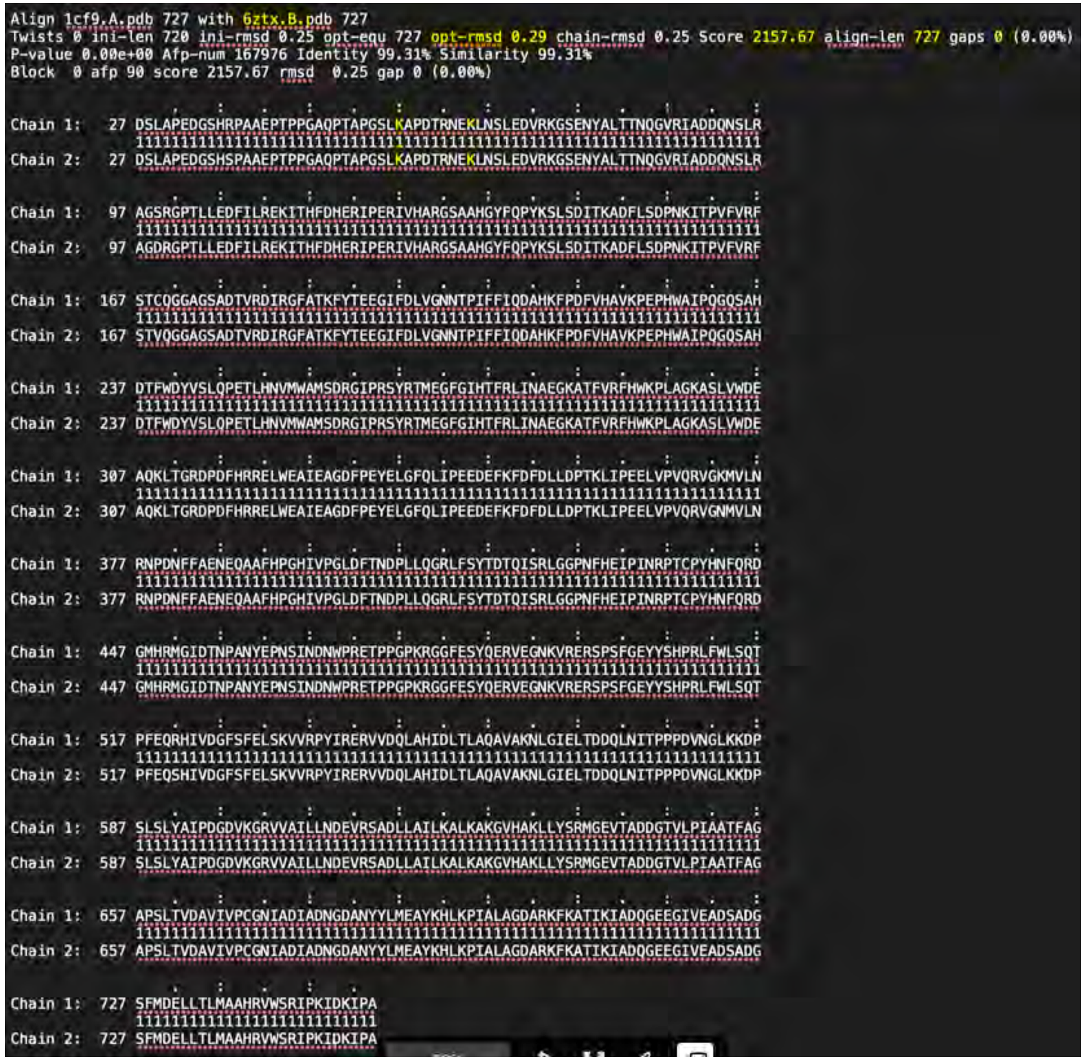

```
Align 1cf9.A.pdb 727 with 6ztx.B.pdb 727
Twists 0 ini-len 720 ini-rmsd 0.25 opt-equ 727 opt-rmsd 0.29 chain-rmsd 0.25 Score 2157.67 align-len 727 gaps 0 (0.00%)
P-value 0.00e+00 Afp-num 167976 Identity 99.31% Similarity 99.31%
Block  0 afp 90 score 2157.67 rmsd  0.25 gap 0 (0.00%)

Chain 1:   27 DSLAPEDGSHRPAAEPTPPGAQPTAPGSLKAPDTRNEKLNSLEDVRKGSENYALTTNQGVRIADDQNSLR
              1111111111111111111111111111111111111111111111111111111111111111111111
Chain 2:   27 DSLAPEDGSHSPAAEPTPPGAQPTAPGSLKAPDTRNEKLNSLEDVRKGSENYALTTNQGVRIADDQNSLR

Chain 1:   97 AGSRGPTLLEDFILREKITHFDHERIPERIVHARGSAAHGYFQPYKSLSDITKADFLSDPNKITPVFVRF
              1111111111111111111111111111111111111111111111111111111111111111111111
Chain 2:   97 AGDRGPTLLEDFILREKITHFDHERIPERIVHARGSAAHGYFQPYKSLSDITKADFLSDPNKITPVFVRF

Chain 1:  167 STCQGGAGSADTVRDIRGFATKFYTEEGIFDLVGNNTPIFFIQDAHKFPDFVHAVKPEPHWAIPQGQSAH
              1111111111111111111111111111111111111111111111111111111111111111111111
Chain 2:  167 STVQGGAGSADTVRDIRGFATKFYTEEGIFDLVGNNTPIFFIQDAHKFPDFVHAVKPEPHWAIPQGQSAH

Chain 1:  237 DTFWDYVSLQPETLHNVMWAMSDRGIPRSYRTMEGFGIHTFRLINAEGKATFVRFHWKPLAGKASLVWDE
              1111111111111111111111111111111111111111111111111111111111111111111111
Chain 2:  237 DTFWDYVSLQPETLHNVMWAMSDRGIPRSYRTMEGFGIHTFRLINAEGKATFVRFHWKPLAGKASLVWDE

Chain 1:  307 AQKLTGRDPDFHRRELWEAIEAGDFPEYELGFQLIPEEDEFKFDFDLLDPTKLIPEELVPVQRVGKMVLN
              1111111111111111111111111111111111111111111111111111111111111111111111
Chain 2:  307 AQKLTGRDPDFHRRELWEAIEAGDFPEYELGFQLIPEEDEFKFDFDLLDPTKLIPEELVPVQRVGNMVLN

Chain 1:  377 RNPDNFFAENEQAAFHPGHIVPGLDFTNDPLLQGRLFSYTDTQISRLGGPNFHEIPINRPTCPYHNFQRD
              1111111111111111111111111111111111111111111111111111111111111111111111
Chain 2:  377 RNPDNFFAENEQAAFHPGHIVPGLDFTNDPLLQGRLFSYTDTQISRLGGPNFHEIPINRPTCPYHNFQRD

Chain 1:  447 GMHRMGIDTNPANYEPNSINDNWPRETPPGPKRGGFESYQERVEGNKVRERSPSFGEYYSHPRLFWLSQT
              1111111111111111111111111111111111111111111111111111111111111111111111
Chain 2:  447 GMHRMGIDTNPANYEPNSINDNWPRETPPGPKRGGFESYQERVEGNKVRERSPSFGEYYSHPRLFWLSQT

Chain 1:  517 PFEQRHIVDGFSFELSKVVRPYIRERVVDQLAHIDLTLAQAVAKNLGIELTDDQLNITPPPDVNGLKKDP
              1111111111111111111111111111111111111111111111111111111111111111111111
Chain 2:  517 PFEQSHIVDGFSFELSKVVRPYIRERVVDQLAHIDLTLAQAVAKNLGIELTDDQLNITPPPDVNGLKKDP

Chain 1:  587 SLSLYAIPDGDVKGRVVAILLNDEVRSADLLAILKALKAKGVHAKLLYSRMGEVTADDGTVLPIAATFAG
              1111111111111111111111111111111111111111111111111111111111111111111111
Chain 2:  587 SLSLYAIPDGDVKGRVVAILLNDEVRSADLLAILKALKAKGVHAKLLYSRMGEVTADDGTVLPIAATFAG

Chain 1:  657 APSLTVDAVIVPCGNIADIADNGDANYYLMEAYKHLKPIALAGDARKFKATIKIADQGEEGIVEADSADG
              1111111111111111111111111111111111111111111111111111111111111111111111
Chain 2:  657 APSLTVDAVIVPCGNIADIADNGDANYYLMEAYKHLKPIALAGDARKFKATIKIADQGEEGIVEADSADG

Chain 1:  727 SFMDELLTLMAAHRVWSRIPKIDKIPA
              111111111111111111111111111
Chain 2:  727 SFMDELLTLMAAHRVWSRIPKIDKIPA
```

UniProt ID: P29422
PDB ID: 1GWE_A

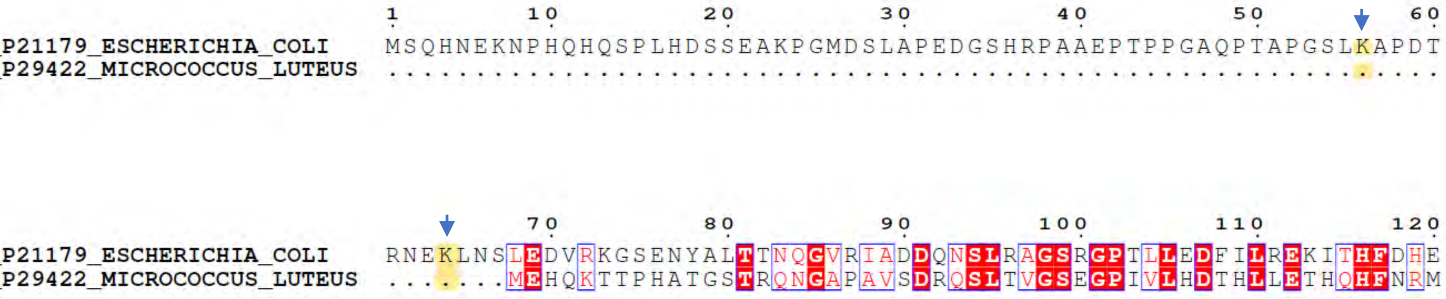

Full sequences in supplemental file.

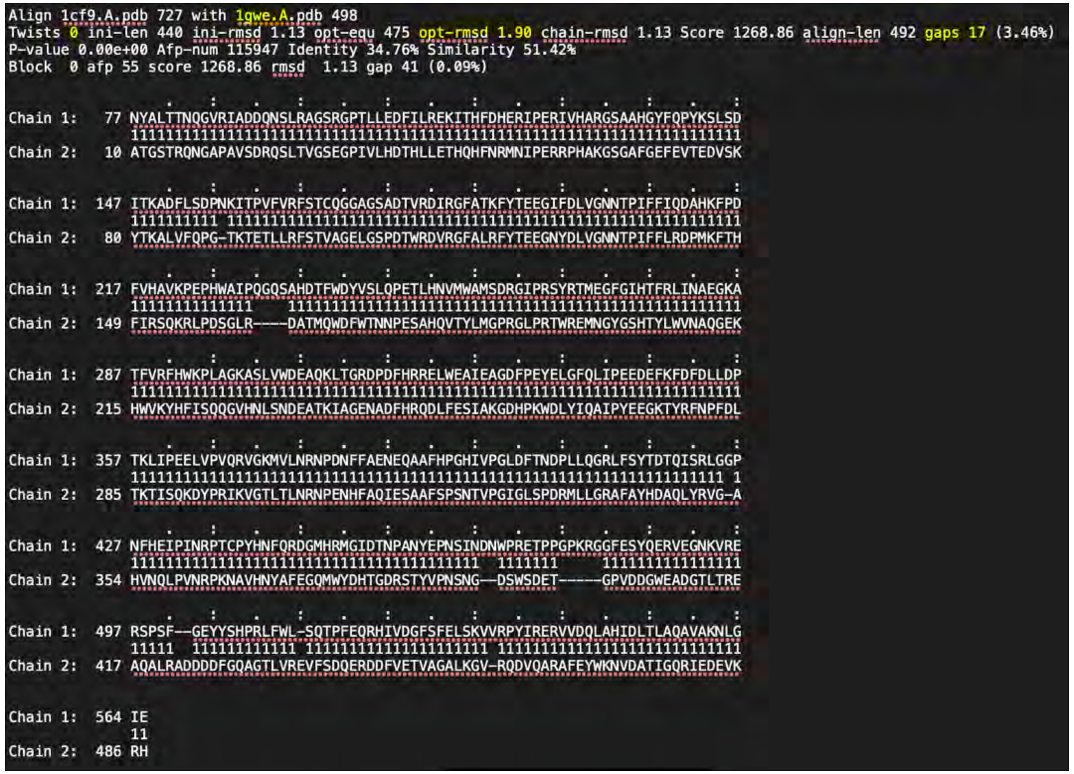

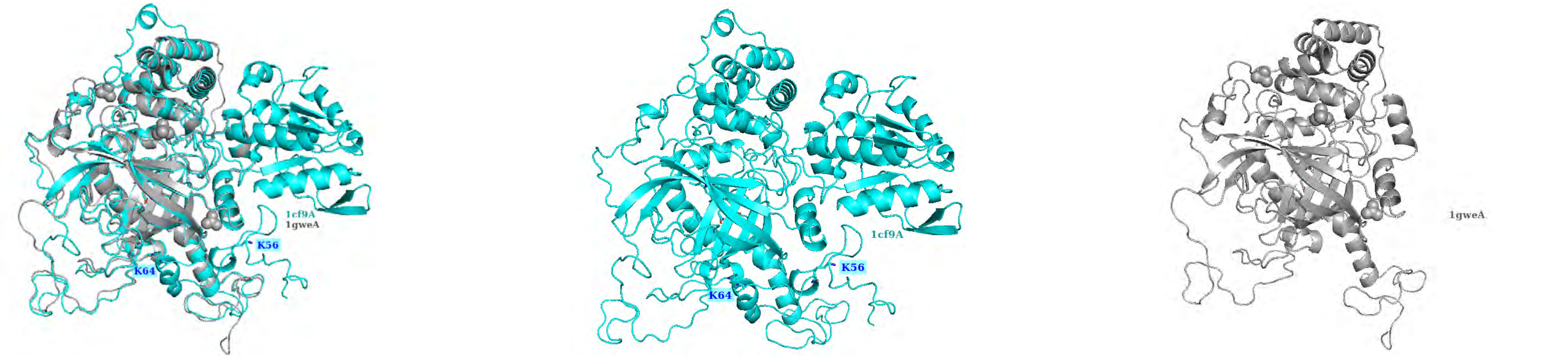

UniProt ID: P29422
PDB ID: 1GWF_A

```
                           1        10        20        30        40        50        60
P21179_ESCHERICHIA_COLI    MSQHNEKNPHQHQSPLHDSSEAKPGMDSLAPEDGSHRPAAEPTPPGAQPTAPGSLKAPDT
P29422_MICROCOCCUS_LUTEUS  ............................................................

                                   70        80        90       100       110       120
P21179_ESCHERICHIA_COLI    RNEKLNSLEDVRKGSENYALTTNQGVRIADDQNSLRAGSRGPTLLEDFILREKITHFDHE
P29422_MICROCOCCUS_LUTEUS  .......MEHQKTTPHATGSTRQNGAPAVSDRQSLTVGSEGPIVLHDTHLLETHQHFNRM
```

Full sequences in supplemental file.

```
Align 1cf9.A.pdb 727 with 1gwf.A.pdb 498
Twists 0 ini-len 440 ini-rmsd 1.11 opt-equ 475 opt-rmsd 1.89 chain-rmsd 1.11 Score 1268.93 align-len 492 gaps 17 (3.46%)
P-value 0.00e+00 Afp-num 116205 Identity 34.55% Similarity 51.22%
Block  0 afp 55 score 1268.93 rmsd  1.11 gap 41 (0.09%)

                   .    :    .    :    .    :    .    :    .    :    .    :    .    :
Chain 1:   77 NYALTTNQGVRIADDQNSLRAGSRGPTLLEDFILREKITHFDHERIPERIVHARGSAAHGYFQPYKSLSD
              1111111111111111111111111111111111111111111111111111111111111111111111
Chain 2:   10 ATGSTRQNGAPAVSDRQSLTVGSEGPIVLHDTHLLETHQHFNRxNIPERRPHAKGSGAFGEFEVTEDVSK

                   .    :    .    :    .    :    .    :    .    :    .    :    .    :
Chain 1:  147 ITKADFLSDPNKITPVFVRFSTCQGGAGSADTVRDIRGFATKFYTEEGIFDLVGNNTPIFFIQDAHKFPD
              1111111111 11111111111111111111111111111111111111111111111111111111111
Chain 2:   80 YTKALVFQPG-TKTETLLRFSTVAGELGSPDTWRDVRGFALRFYTEEGNYDLVGNNTPIFFLRDPxKFTH

                   .    :    .    :    .    :    .    :    .    :    .    :    .    :
Chain 1:  217 FVHAVKPEPHWAIPQGQSAHDTFWDYVSLQPETLHNVMWAMSDRGIPRSYRTMEGFGIHTFRLINAEGKA
              11111111111111    1111111111111111111111111111111111111111111111111111
Chain 2:  149 FIRSQKRLPDSGLR----DATxQWDFWTNNPESAHQVTYLxGPRGLPRTWREMNGYGSHTYLWVNAQGEK

                   .    :    .    :    .    :    .    :    .    :    .    :    .    :
Chain 1:  287 TFVRFHWKPLAGKASLVWDEAQKLTGRDPDFHRRELWEAIEAGDFPEYELGFQLIPEEDEFKFDFDLLDP
              1111111111111111111111111111111111111111111111111111111111111111111111
Chain 2:  215 HWVKYHFISQQGVHNLSNDEATKIAGENADFHRQDLFESIAKGDHPKWDLYIQAIPYEEGKTYRFNPFDL

                   .    :    .    :    .    :    .    :    .    :    .    :    .    :
Chain 1:  357 TKLIPEELVPVQRVGKMVLNRNPDNFFAENEQAAFHPGHIVPGLDFTNDPLLQGRLFSYTDTQISRLGGP
              111111111111111111111111111111111111111111111111111111111111111111111
Chain 2:  285 TKTISQKDYPRIKVGTLTLNRNPENHFAQIESAAFSPSNTVPGIGLSPDRMLLGRAFAYHDAQLYRVGA-

                   .    :    .    :    .    :    .    :    .    :    .    :    .    :
Chain 1:  427 NFHEIPINRPTCPYHNFQRDGMHRMGIDTNPANYEPNSINDNWPRETPPGPKRGGFESYQERVEGNKVRE
              1111111111111111111111111111111111111111  1111111     1111111111111111
Chain 2:  354 HVNQLPVNRPKNAVHNYAFEGQxWYDHTGDRSTYVPNSNG--DSWSDET-----GPVDDGWEADGTLTRE

                   .    :    .    :    .    :    .    :    .    :    .    :    .    :
Chain 1:  497 RSPSF--GEYYSHPRLFWL-SQTPFEQRHIVDGFSFELSKVVRPYIRERVVDQLAHIDLTLAQAVAKNLG
              11111  111111111111 111111111111111111111 1111111111111111111111111111
Chain 2:  417 AQALRADDDDFGQAGTLVREVFSDQERDDFVETVAGALKGV-RQDVQARAFEYWKNVDATIGQRIEDEVK

Chain 1:  564 IE
              11
Chain 2:  486 RH
```

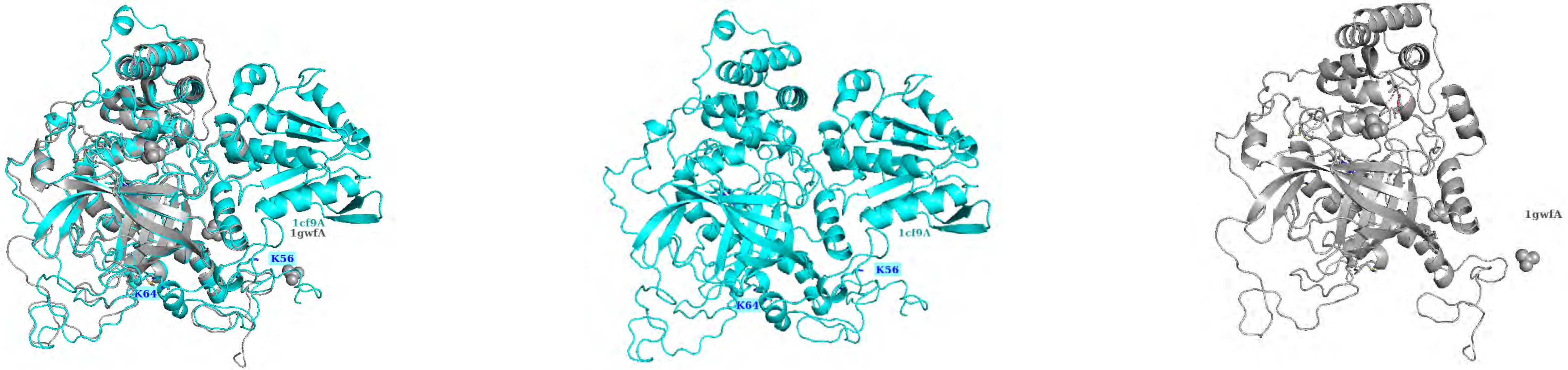

UniProt ID: P29422

PDB ID: 1GWH_A

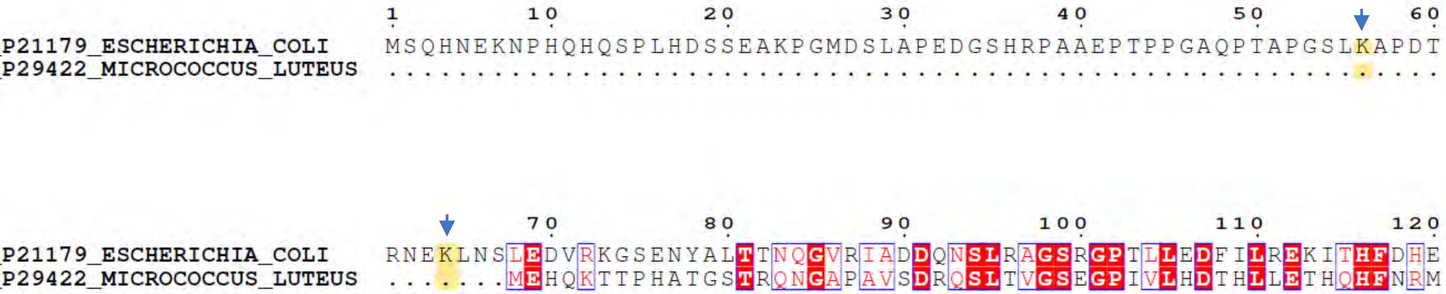

Full sequences in supplemental file.

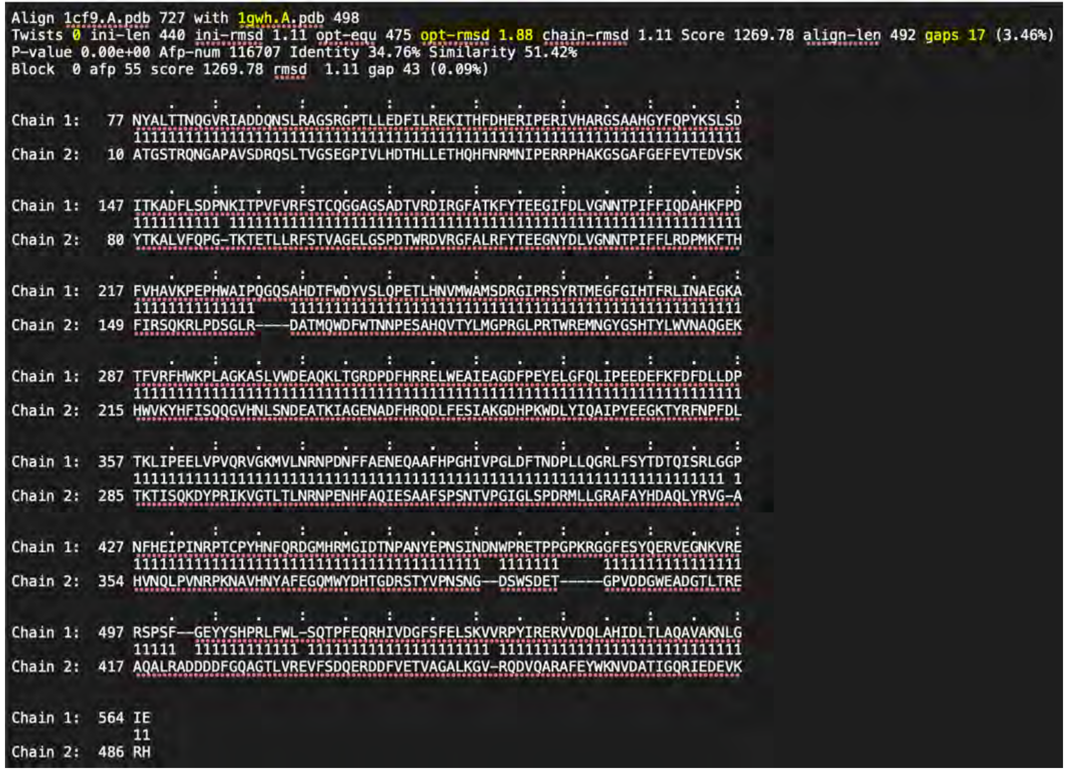

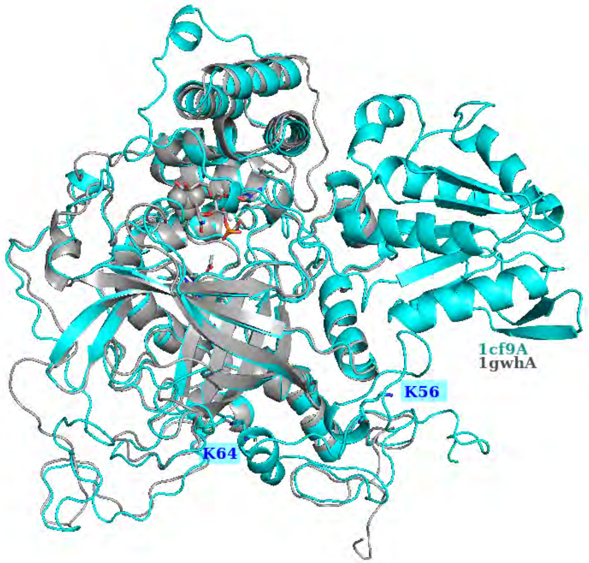

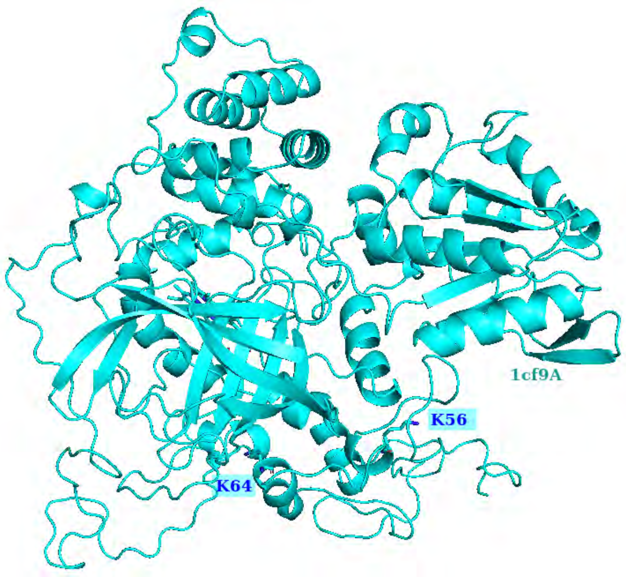

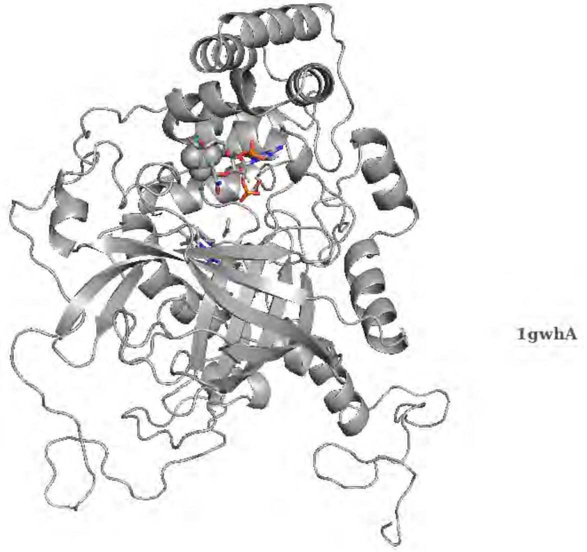

UniProt ID: P29422

PDB ID: 1HBZ_A

```
                            1        10        20        30        40        50        60
P21179_ESCHERICHIA_COLI     MSQHNEKNPHQHQSPLHDSSEAKPGMDSLAPEDGSHRPAAEPTPPGAQPTAPGSLKAPDT
P29422_MICROCOCCUS_LUTEUS   ............................................................

                                    70        80        90       100       110       120
P21179_ESCHERICHIA_COLI     RNEKLNSLEDVRKGSENYALTTNQGVRIADDQNSLRAGSRGPTLLEDFILREKITHFDHE
P29422_MICROCOCCUS_LUTEUS   .......MEHQKTTPHATGSTRQNGAPAVSDRQSLTVGSEGPIVLHDTHLLETHQHFNRM
```

Full sequences in supplemental file.

```
Align 1cf9.A.pdb 727 with 1hbz.A.pdb 498
Twists 0 ini-len 440 ini-rmsd 1.11 opt-equ 475 opt-rmsd 1.89 chain-rmsd 1.11 Score 1269.98 align-len 492 gaps 17 (3.46%)
P-value 0.00e+00 Afp-num 116702 Identity 34.76% Similarity 51.22%
Block  0 afp 55 score 1269.98 rmsd  1.11 gap 43 (0.09%)

Chain 1:   77 NYALTTNQGVRIADDQNSLRAGSRGPTLLEDFILREKITHFDHERIPERIVHARGSAAHGYFQPYKSLSD
              1111111111111111111111111111111111111111111111111111111111111111111111
Chain 2:   10 ATGSTRQNGAPAVSDRQSLTVGSEGPIVLHDTHLLETHQHFNRMNIPERRPHAKGSGAFGEFEVTEDVSK

Chain 1:  147 ITKADFLSDPNKITPVFVRFSTCQGGAGSADTVRDIRGFATKFYTEEGIFDLVGNNTPIFFIQDAHKFPD
              1111111111 11111111111111111111111111111111111111111111111111111111111
Chain 2:   80 YTKALVFQPG-TKTETLLRFSTVAGELGSPDTWRDVRGFALRFYTEEGNYDLVGNNTPIFFLRDPMKFTH

Chain 1:  217 FVHAVKPEPHWAIPQGQSAHDTFWDYVSLQPETLHNVMWAMSDRGIPRSYRTMEGFGIHTFRLINAEGKA
              11111111111111    111111111111111111111111111111111111111111111111111
Chain 2:  149 FIRSQKRLPDSGLR----DATMQWDFWTNNPESAHQVTYLMGPRGLPRTWREMNGYGSHTYLWVNAQGEK

Chain 1:  287 TFVRFHWKPLAGKASLVWDEAQKLTGRDPDFHRRELWEAIEAGDFPEYELGFQLIPEEDEFKFDFDLLDP
              1111111111111111111111111111111111111111111111111111111111111111111111
Chain 2:  215 HWVKYHFISQQGVHNLSNDEATKIAGENADFHRQDLFESIAKGDHPKWDLYIQAIPYEEGKTYRFNPFDL

Chain 1:  357 TKLIPEELVPVQRVGKMVLNRNPDNFFAENEQAAFHPGHIVPGLDFTNDPLLQGRLFSYTDTQISRLGGP
              111111111111111111111111111111111111111111111111111111111111111111 1
Chain 2:  285 TKTISQKDYPRIKVGTLTLNRNPKNHFAQIESAAFSPSNTVPGIGLSPDRMLLGRAFAYHDAQLYRVG-A

Chain 1:  427 NFHEIPINRPTCPYHNFQRDGMHRMGIDTNPANYEPNSINDNWPRETPPGPKRGGFESYQERVEGNKVRE
              111111111111111111111111111111111111111  1111111      111111111111111
Chain 2:  354 HVNQLPVNRPKNAVHNYAFEGQMWYDHTGDRSTYVPNSNG--DSWSDET------GPVDDGWEADGTLTRE

Chain 1:  497 RSPSF--GEYYSHPRLFWL-SQTPFEQRHIVDGFSFELSKVVRPYIRERVVDQLAHIDLTLAQAVAKNLG
              11111  111111111111 1111111111111111111111 1111111111111111111111111111
Chain 2:  417 AQALRADDDDFGQAGTLVREVFSDQERDDFVETVAGALKGV-RQDVQARAFEYWKNVDATIGQRIEDEVK

Chain 1:  564 IE
              11
Chain 2:  486 RH
```

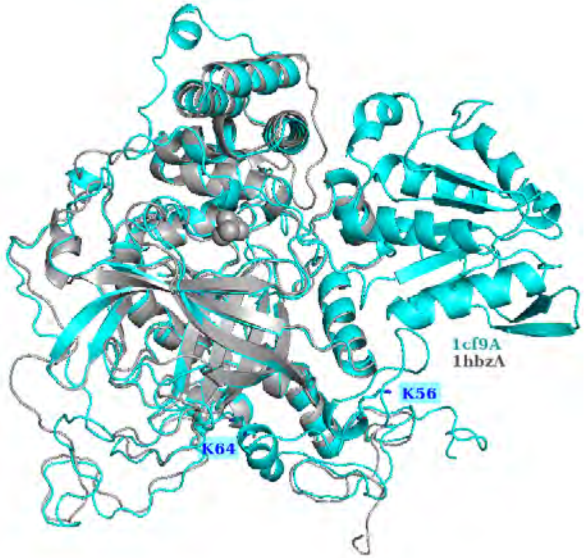

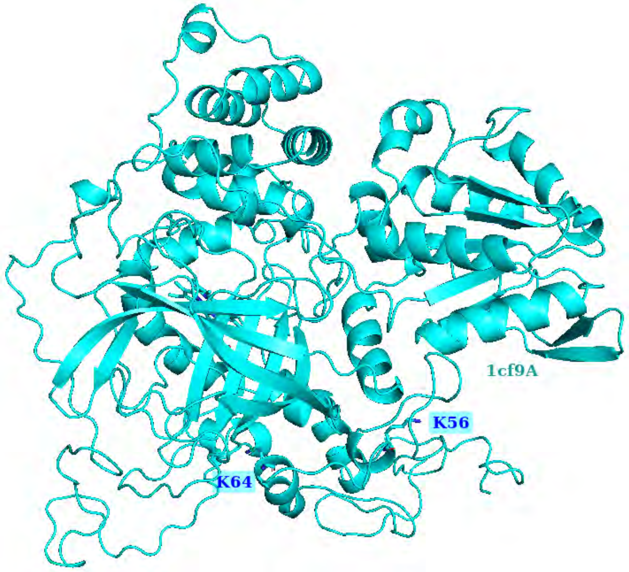

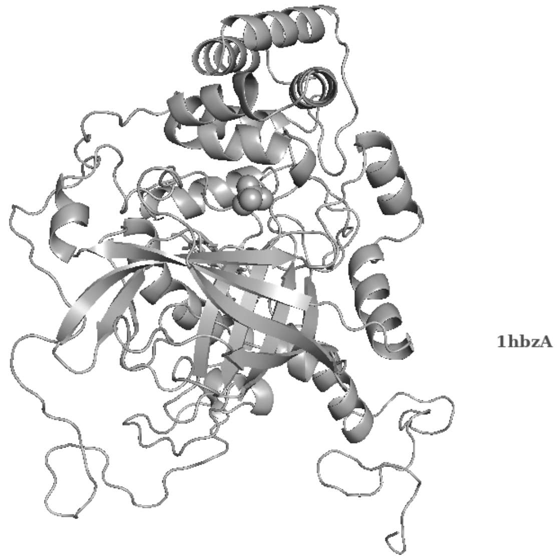

UniProt ID: P30263

PDB ID: 2XQ1_M

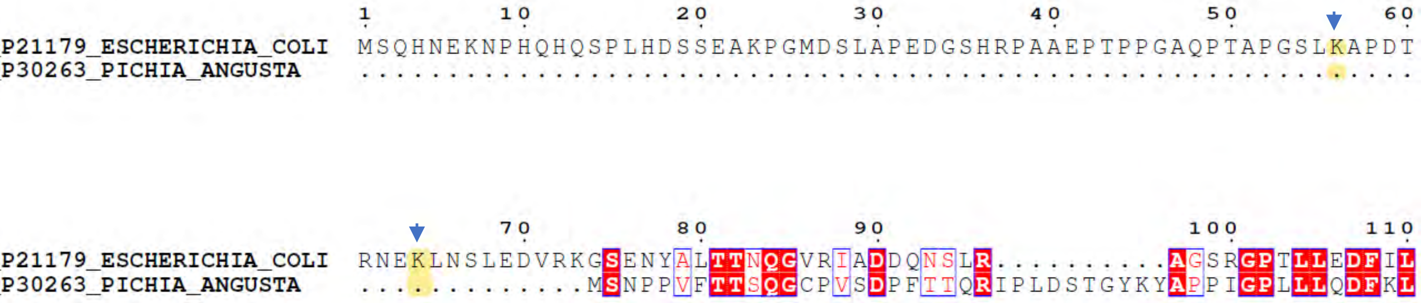

Full sequences in supplemental file.

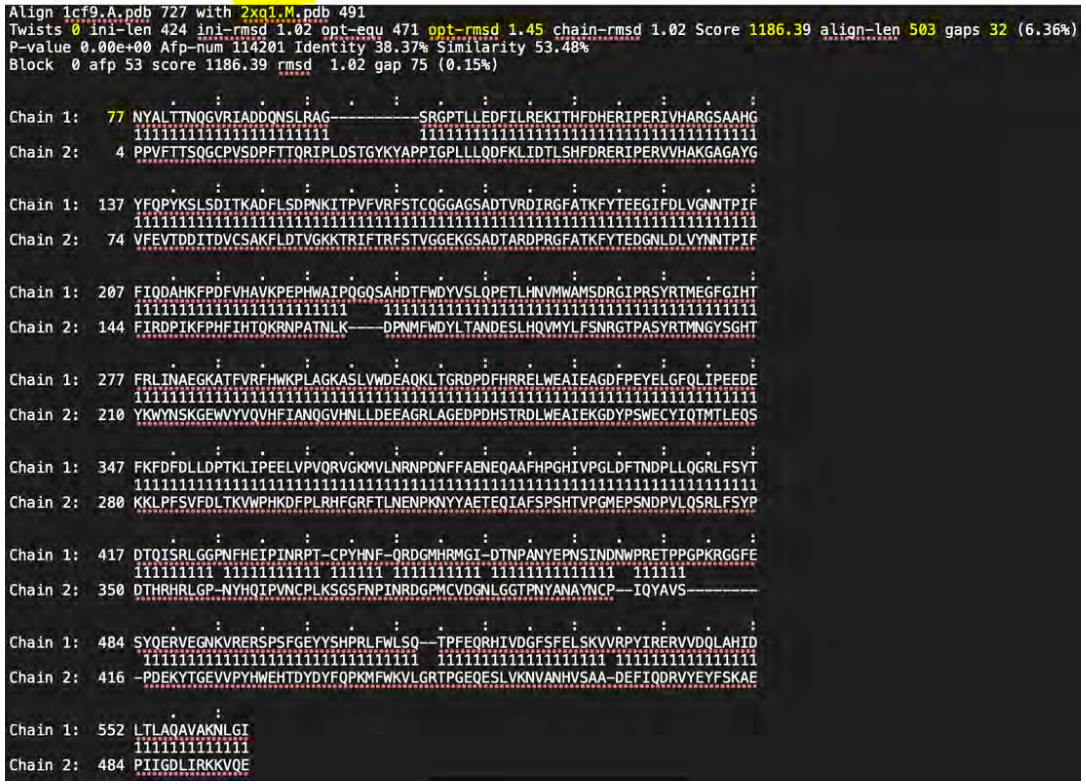

```
Align 1cf9.A.pdb 727 with 2xq1.M.pdb 491
Twists 0 ini-len 424 ini-rmsd 1.02 opt-equ 471 opt-rmsd 1.45 chain-rmsd 1.02 Score 1186.39 align-len 503 gaps 32 (6.36%)
P-value 0.00e+00 Afp-num 114201 Identity 38.37% Similarity 53.48%
Block  0 afp 53 score 1186.39 rmsd  1.02 gap 75 (0.15%)

Chain 1:   77 NYALTTNQGVRIADDQNSLRAG----------SRGPTLLEDFILREKITHFDHERIPERIVHARGSAAHG
Chain 2:    4 PPVFTTSQGCPVSDPFTTQRIPLDSTGYKYAPPIGPLLLQDFKLIDTLSHFDRERIPERVVHAKGAGAYG

Chain 1:  137 YFQPYKSLSDITKADFLSDPNKITPVFVRFSTCQGGAGSADTVRDIRGFATKFYTEEGIFDLVGNNTPIF
Chain 2:   74 VFEVTDDITDVCSAKFLDTVGKKTRIFTRFSTVGGEKGSADTARDPRGFATKFYTEDGNLDLVYNNTPIF

Chain 1:  207 FIQDAHKFPDFVHAVKPEPHWAIPQGQSAHDTFWDYVSLQPETLHNVMWAMSDRGIPRSYRTMEGFGIHT
Chain 2:  144 FIRDPIKFPHFIHTQKRNPATNLK----DPNMFWDYLTANDESLHQVMYLFSNRGTPASYRTMNGYSGHT

Chain 1:  277 FRLINAEGKATFVRFHWKPLAGKASLVWDEAQKLTGRDPDFHRRELWEAIEAGDFPEYELGFQLIPEEDE
Chain 2:  210 YKWYNSKGEWVYVQVHFIANQGVHNLLDEEAGRLAGEDPDHSTRDLWEAIEKGDYPSWECYIQTMTLEQS

Chain 1:  347 FKFDFDLLDPTKLIPEELVPVQRVGKMVLNRNPDNFFAENEQAAFHPGHIVPGLDFTNDPLLQGRLFSYT
Chain 2:  280 KKLPFSVFDLTKVWPHKDFPLRHFGRFTLNENPKNYYAETEQIAFSPSHTVPGMEPSNDPVLQSRLFSYP

Chain 1:  417 DTQISRLGGPNFHEIPINRPT-CPYHNF-QRDGMHRMGI-DTNPANYEPNSINDNWPRETPPGPKRGGFE
Chain 2:  350 DTHRHRLGP-NYHQIPVNCPLKSGSFNPINRDGPMCVDGNLGGTPNYANAYNCP--IQYAVS--------

Chain 1:  484 SYQERVEGNKVRERSPSFGEYYSHPRLFWLSQ--TPFEQRHIVDGFSFELSKVVRPYIRERVVDQLAHID
Chain 2:  416 -PDEKYTGEVVPYHWEHTDYDYFQPKMFWKVLGRTPGEQESLVKNVANHVSAA-DEFIQDRVYEYFSKAE

Chain 1:  552 LTLAQAVAKNLGI
Chain 2:  484 PIIGDLIRKKVQE
```

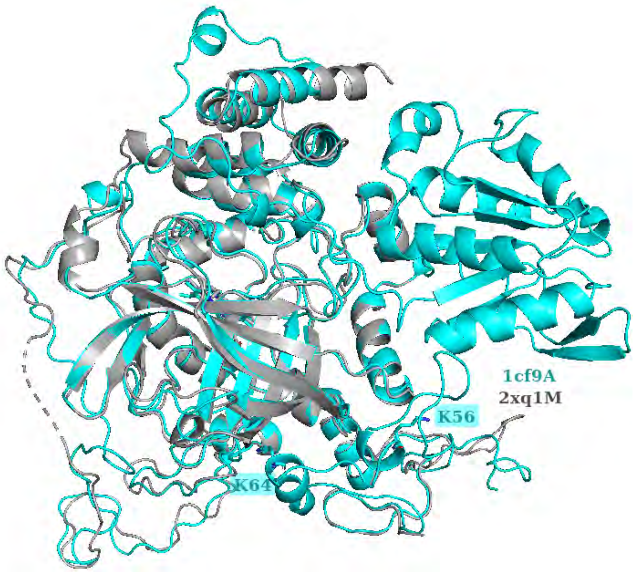

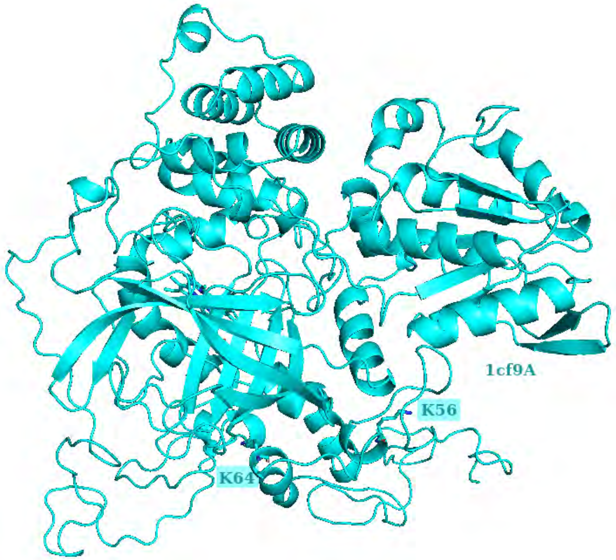

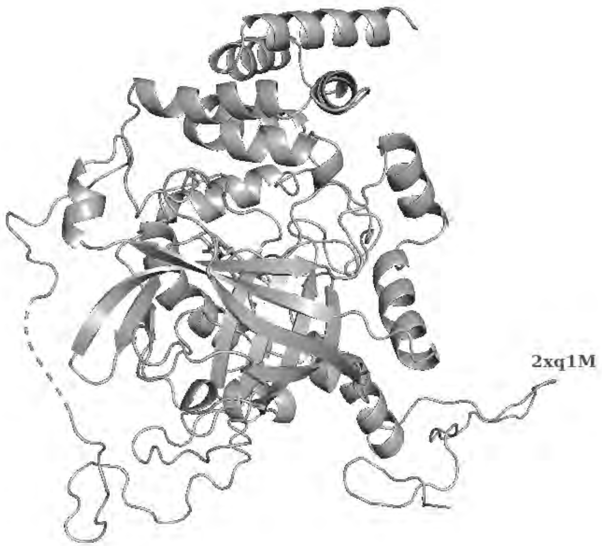

UniProt ID: P42321
PDB ID: 1E93_A

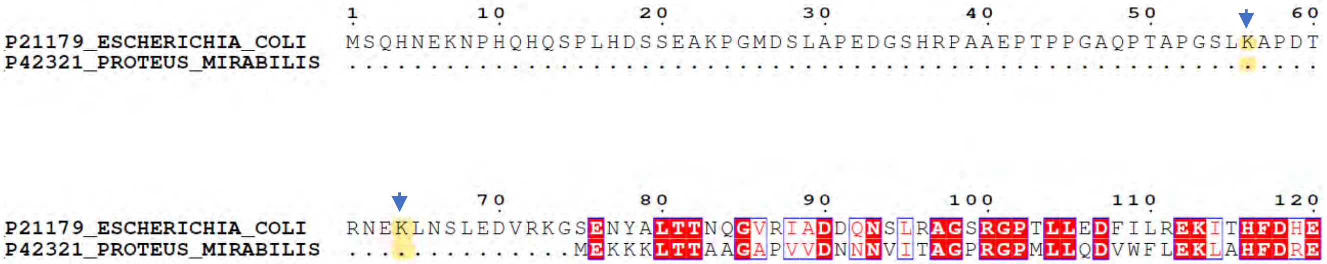

Full sequences in supplemental file.

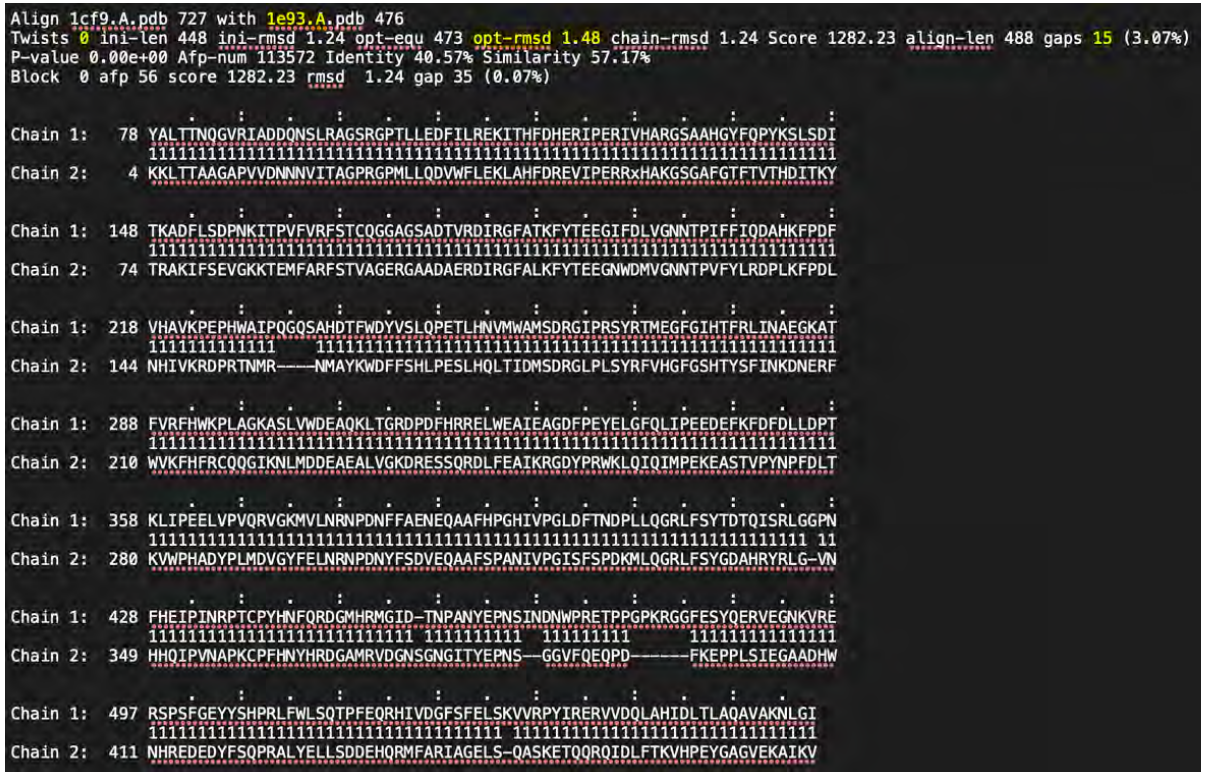

```
Align 1cf9.A.pdb 727 with 1e93.A.pdb 476
Twists 0 ini-len 448 ini-rmsd 1.24 opt-equ 473 opt-rmsd 1.48 chain-rmsd 1.24 Score 1282.23 align-len 488 gaps 15 (3.07%)
P-value 0.00e+00 Afp-num 113572 Identity 40.57% Similarity 57.17%
Block  0 afp 56 score 1282.23 rmsd  1.24 gap 35 (0.07%)

Chain 1:   78 YALTTNQGVRIADDQNSLRAGSRGPTLLEDFILREKITHFDHERIPERIVHARGSAAHGYFQPYKSLSDI
              1111111111111111111111111111111111111111111111111111111111111111111111
Chain 2:    4 KKLTTAAGAPVVDNNNVITAGPRGPMLLQDVWFLEKLAHFDREVIPERRxHAKGSGAFGTFTVTHDITKY

Chain 1:  148 TKADFLSDPNKITPVFVRFSTCQGGAGSADTVRDIRGFATKFYTEEGIFDLVGNNTPIFFIQDAHKFPDF
              1111111111111111111111111111111111111111111111111111111111111111111111
Chain 2:   74 TRAKIFSEVGKKTEMFARFSTVAGERGAADAERDIRGFALKFYTEEGNWDMVGNNTPVFYLRDPLKFPDL

Chain 1:  218 VHAVKPEPHWAIPQGQSAHDTFWDYVSLQPETLHNVMWAMSDRGIPRSYRTMEGFGIHTFRLINAEGKAT
              1111111111111    11111111111111111111111111111111111111111111111111111
Chain 2:  144 NHIVKRDPRTNMR----NMAYKWDFFSHLPESLHQLTIDMSDRGLPLSYRFVHGFGSHTYSFINKDNERF

Chain 1:  288 FVRFHWKPLAGKASLVWDEAQKLTGRDPDFHRRELWEAIEAGDFPEYELGFQLIPEEDEFKFDFDLLDPT
              1111111111111111111111111111111111111111111111111111111111111111111111
Chain 2:  210 WVKFHFRCQQGIKNLMDDEAEALVGKDRESSQRDLFEAIKRGDYPRWKLQIQIMPEKEASTVPYNPFDLT

Chain 1:  358 KLIPEELVPVQRVGKMVLNRNPDNFFAENEQAAFHPGHIVPGLDFTNDPLLQGRLFSYTDTQISRLGGPN
              1111111111111111111111111111111111111111111111111111111111111111111 11
Chain 2:  280 KVWPHADYPLMDVGYFELNRNPDNYFSDVEQAAFSPANIVPGISFSPDKMLQGRLFSYGDAHRYRLG-VN

Chain 1:  428 FHEIPINRPTCPYHNFQRDGMHRMGID-TNPANYEPNSINDNWPRETPPGPKRGGFESYQERVEGNKVRE
              111111111111111111111111111 1111111111  111111111      111111111111111
Chain 2:  349 HHQIPVNAPKCPFHNYHRDGAMRVDGNSGNGITYEPNS--GGVFQEQPD------FKEPPLSIEGAADHW

Chain 1:  497 RSPSFGEYYSHPRLFWLSQTPFEQRHIVDGFSFELSKVVRPYIRERVVDQLAHIDLTLAQAVAKNLGI
              111111111111111111111111111111111111 1111111111111111111111111111111
Chain 2:  411 NHREDEDYFSQPRALYELLSDDEHQRMFARIAGELS-QASKETQQRQIDLFTKVHPEYGAGVEKAIKV
```

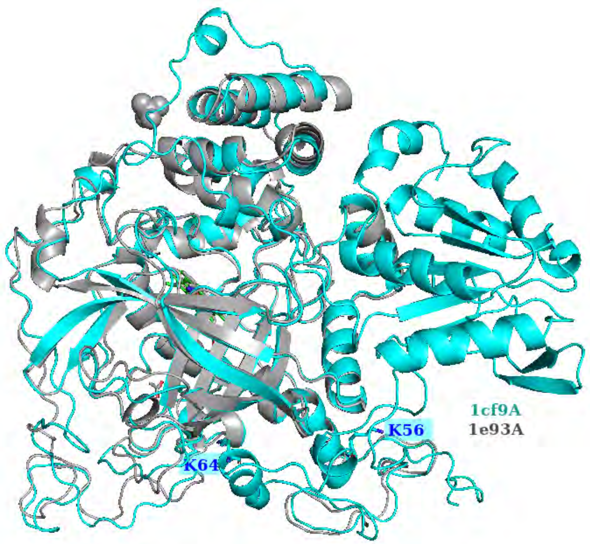

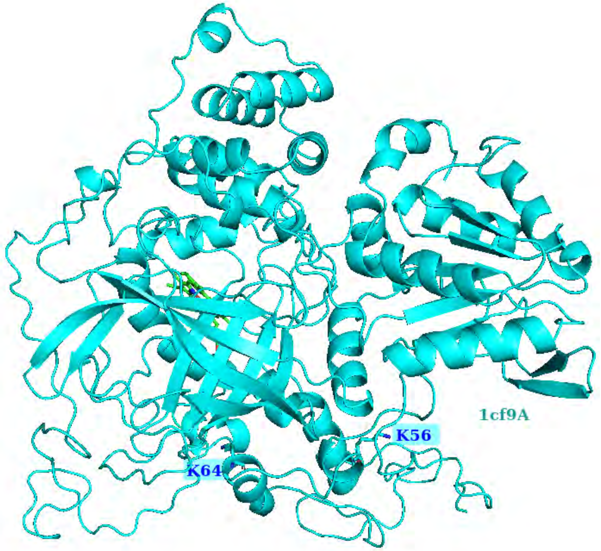

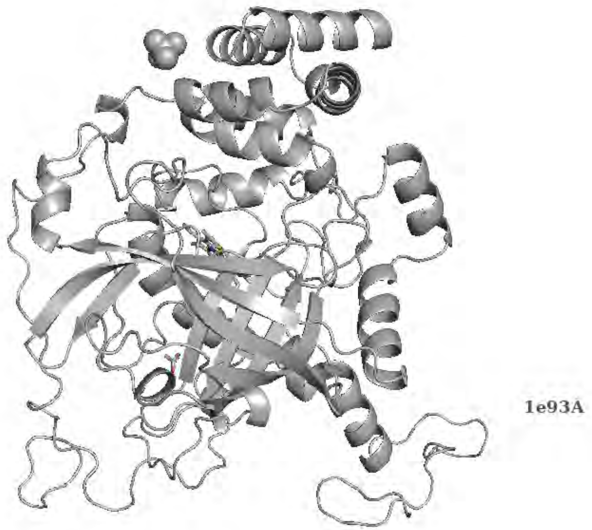

UniProt ID: P42321

PDB ID: 1H6N_A

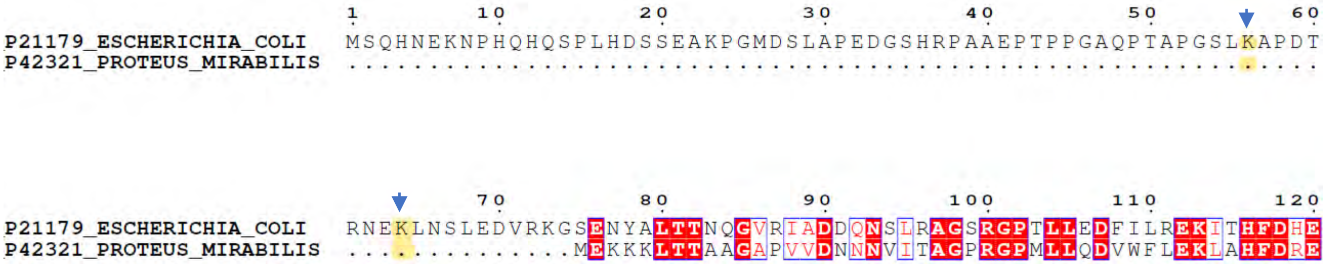

Full sequences in supplemental file.

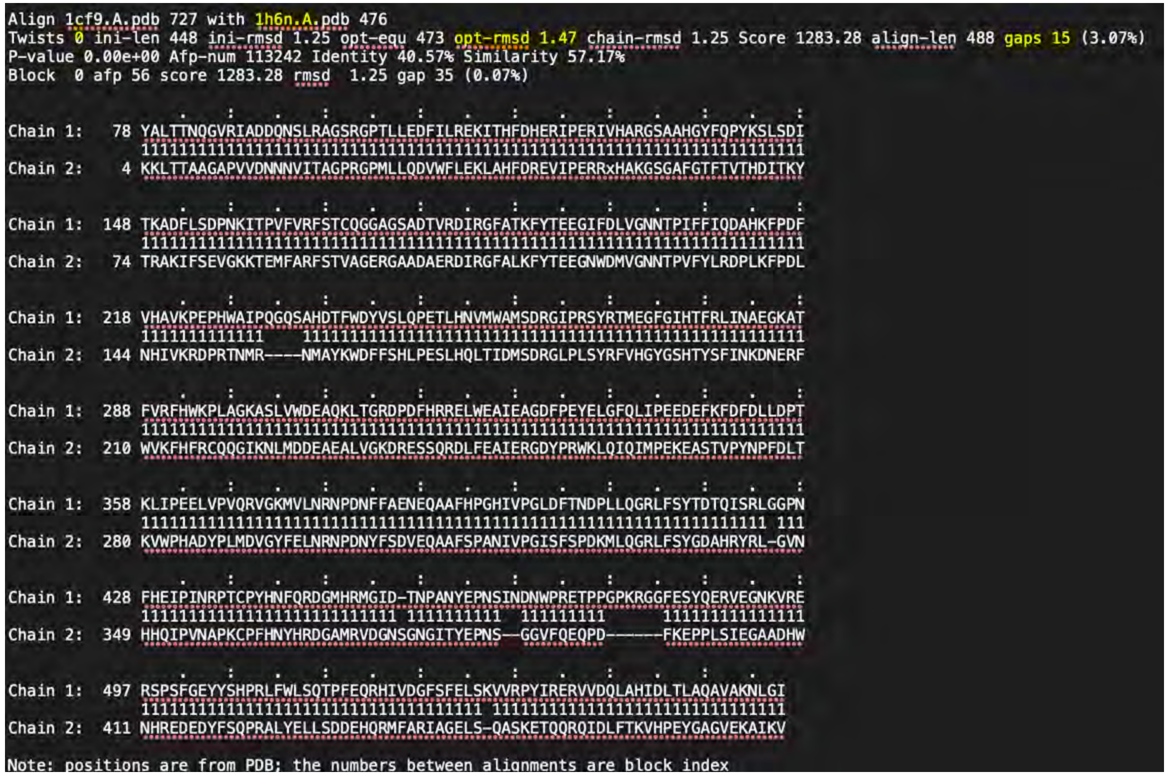

```
Align 1cf9.A.pdb 727 with 1h6n.A.pdb 476
Twists 0 ini-len 448 ini-rmsd 1.25 opt-equ 473 opt-rmsd 1.47 chain-rmsd 1.25 Score 1283.28 align-len 488 gaps 15 (3.07%)
P-value 0.00e+00 Afp-num 113242 Identity 40.57% Similarity 57.17%
Block  0 afp 56 score 1283.28 rmsd  1.25 gap 35 (0.07%)

                   .    :    .    :    .    :    .    :    .    :    .    :    .    :
Chain 1:   78 YALTTNQGVRIADDQNSLRAGSRGPTLLEDFILREKITHFDHERIPERIVHARGSAAHGYFQPYKSLSDI
              1111111111111111111111111111111111111111111111111111111111111111111111
Chain 2:    4 KKLTTAAGAPVVDNNNVITAGPRGPMLLQDVWFLEKLAHFDREVIPERRxHAKGSGAFGTFTVTHDITKY

                   .    :    .    :    .    :    .    :    .    :    .    :    .    :
Chain 1:  148 TKADFLSDPNKITPVFVRFSTCQGGAGSADTVRDIRGFATKFYTEEGIFDLVGNNTPIFFIQDAHKFPDF
              1111111111111111111111111111111111111111111111111111111111111111111111
Chain 2:   74 TRAKIFSEVGKKTEMFARFSTVAGERGAADAERDIRGFALKFYTEEGNWDMVGNNTPVFYLRDPLKFPDL

                   .    :    .    :    .    :    .    :    .    :    .    :    .    :
Chain 1:  218 VHAVKPEPHWAIPQGQSAHDTFWDYVSLQPETLHNVMWAMSDRGIPRSYRTMEGFGIHTFRLINAEGKAT
              1111111111111    11111111111111111111111111111111111111111111111111111
Chain 2:  144 NHIVKRDPRTNMR----NMAYKWDFFSHLPESLHQLTIDMSDRGLPLSYRFVHGYGSHTYSFINKDNERF

                   .    :    .    :    .    :    .    :    .    :    .    :    .    :
Chain 1:  288 FVRFHWKPLAGKASLVWDEAQKLTGRDPDFHRRELWEAIEAGDFPEYELGFQLIPEEDEFKFDFDLLDPT
              1111111111111111111111111111111111111111111111111111111111111111111111
Chain 2:  210 WVKFHFRCQQGIKNLMDDEAEALVGKDRESSQRDLFEAIERGDYPRWKLQIQIMPEKEASTVPYNPFDLT

                   .    :    .    :    .    :    .    :    .    :    .    :    .    :
Chain 1:  358 KLIPEELVPVQRVGKMVLNRNPDNFFAENEQAAFHPGHIVPGLDFTNDPLLQGRLFSYTDTQISRLGGPN
              111111111111111111111111111111111111111111111111111111111111111111 111
Chain 2:  280 KVWPHADYPLMDVGYFELNRNPDNYFSDVEQAAFSPANIVPGISFSPDKMLQGRLFSYGDAHRYRL-GVN

                   .    :    .    :    .    :    .    :    .    :    .    :    .    :
Chain 1:  428 FHEIPINRPTCPYHNFQRDGMHRMGID-TNPANYEPNSINDNWPRETPPGPKRGGFESYQERVEGNKVRE
              111111111111111111111111111 1111111111  111111111       111111111111111
Chain 2:  349 HHQIPVNAPKCPFHNYHRDGAMRVDGNSGNGITYEPNS--GGVFQEQPD-------FKEPPLSIEGAADHW

                   .    :    .    :    .    :    .    :    .    :    .    :    .
Chain 1:  497 RSPSFGEYYSHPRLFWLSQTPFEQRHIVDGFSFELSKVVRPYIRERVVDQLAHIDLTLAQAVAKNLGI
              111111111111111111111111111111111111 1111111111111111111111111111111
Chain 2:  411 NHREDEDYFSQPRALYELLSDDEHQRMFARIAGELS-QASKETQQRQIDLFTKVHPEYGAGVEKAIKV

Note: positions are from PDB; the numbers between alignments are block index
```

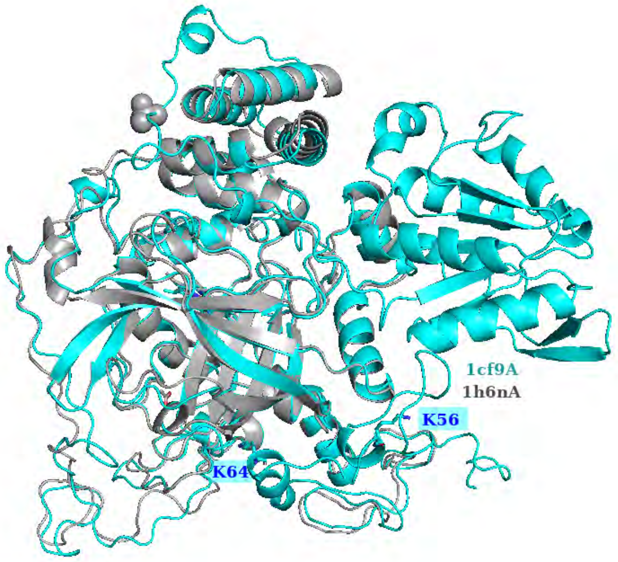

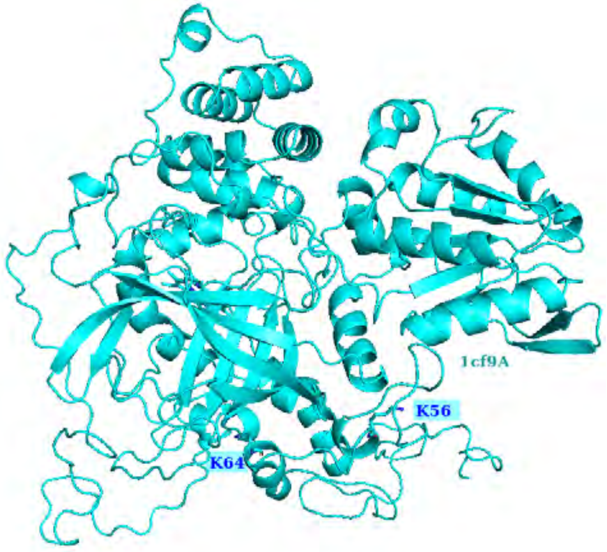

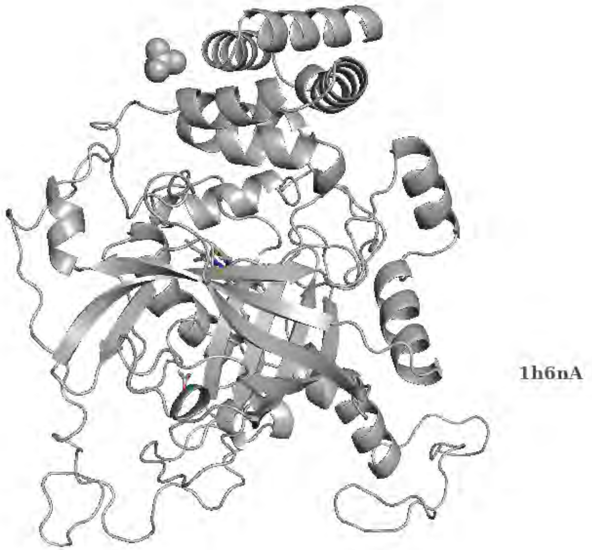

UniProt ID: P42321
PDB ID: 1H7K_A

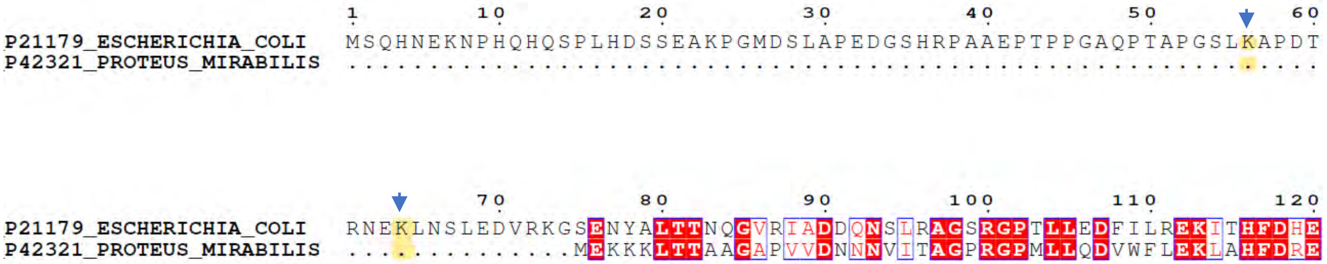

Full sequences in supplemental file.

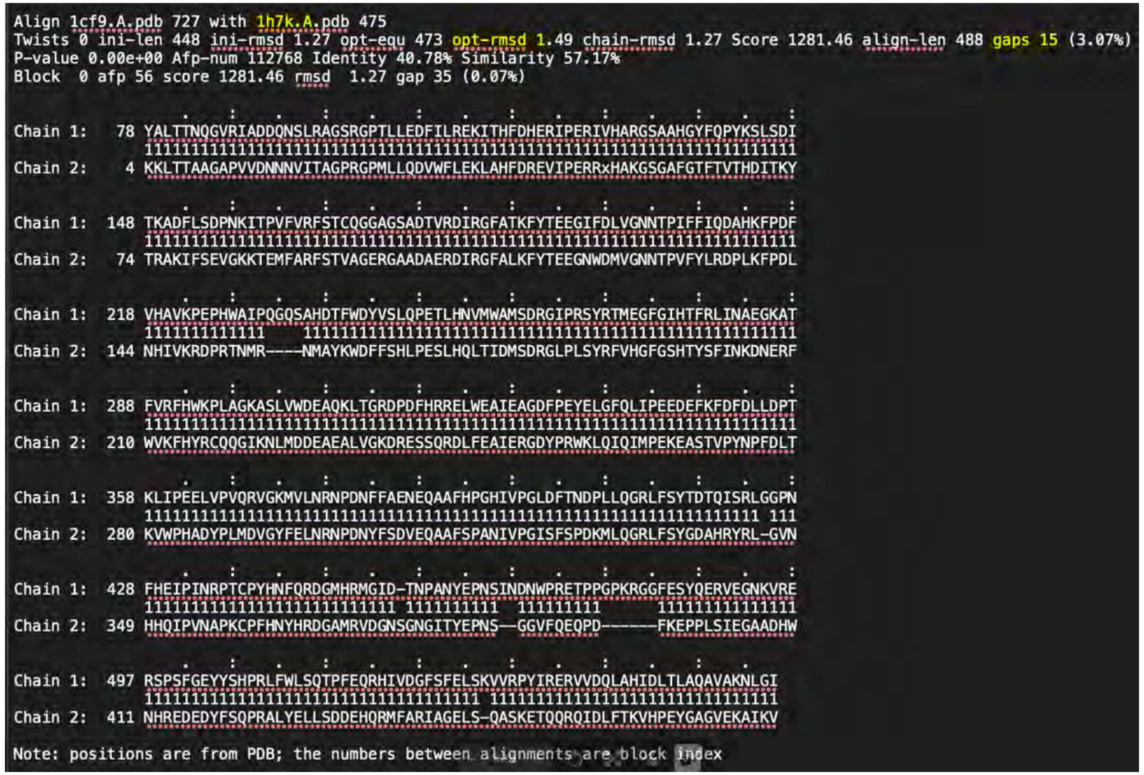

```
Align 1cf9.A.pdb 727 with 1h7k.A.pdb 475
Twists 0 ini-len 448 ini-rmsd 1.27 opt-equ 473 opt-rmsd 1.49 chain-rmsd 1.27 Score 1281.46 align-len 488 gaps 15 (3.07%)
P-value 0.00e+00 Afp-num 112768 Identity 40.78% Similarity 57.17%
Block  0 afp 56 score 1281.46 rmsd  1.27 gap 35 (0.07%)

Chain 1:   78 YALTTNQGVRIADDQNSLRAGSRGPTLLEDFILREKITHFDHERIPERIVHARGSAAHGYFQPYKSLSDI
              1111111111111111111111111111111111111111111111111111111111111111111111
Chain 2:    4 KKLTTAAGAPVVDNNNVITAGPRGPMLLQDVWFLEKLAHFDREVIPERRxHAKGSGAFGTFTVTHDITKY

Chain 1:  148 TKADFLSDPNKITPVFVRFSTCQGGAGSADTVRDIRGFATKFYTEEGIFDLVGNNTPIFFIQDAHKFPDF
              1111111111111111111111111111111111111111111111111111111111111111111111
Chain 2:   74 TRAKIFSEVGKKTEMFARFSTVAGERGAADAERDIRGFALKFYTEEGNWDMVGNNTPVFYLRDPLKFPDL

Chain 1:  218 VHAVKPEPHWAIPQGQSAHDTFWDYVSLQPETLHNVMWAMSDRGIPRSYRTMEGFGIHTFRLINAEGKAT
              1111111111111    11111111111111111111111111111111111111111111111111111
Chain 2:  144 NHIVKRDPRTNMR----NMAYKWDFFSHLPESLHQLTIDMSDRGLPLSYRFVHGFGSHTYSFINKDNERF

Chain 1:  288 FVRFHWKPLAGKASLVWDEAQKLTGRDPDFHRRELWEAIEAGDFPEYELGFQLIPEEDEFKFDFDLLDPT
              1111111111111111111111111111111111111111111111111111111111111111111111
Chain 2:  210 WVKFHYRCQQGIKNLMDDEAEALVGKDRESSQRDLFEAIERGDYPRWKLQIQIMPEKEASTVPYNPFDLT

Chain 1:  358 KLIPEELVPVQRVGKMVLNRNPDNFFAENEQAAFHPGHIVPGLDFTNDPLLQGRLFSYTDTQISRLGGPN
              111111111111111111111111111111111111111111111111111111111111111111 111
Chain 2:  280 KVWPHADYPLMDVGYFELNRNPDNYFSDVEQAAFSPANIVPGISFSPDKMLQGRLFSYGDAHRYRL-GVN

Chain 1:  428 FHEIPINRPTCPYHNFQRDGMHRMGID-TNPANYEPNSINDNWPRETPPGPKRGGFESYQERVEGNKVRE
              111111111111111111111111111 1111111111  111111111      111111111111111
Chain 2:  349 HHQIPVNAPKCPFHNYHRDGAMRVDGNSGNGITYEPNS--GGVFQEQPD------FKEPPLSIEGAADHW

Chain 1:  497 RSPSFGEYYSHPRLFWLSQTPFEQRHIVDGFSFELSKVVRPYIRERVVDQLAHIDLTLAQAVAKNLGI
              111111111111111111111111111111111111 1111111111111111111111111111111
Chain 2:  411 NHREDEDYFSQPRALYELLSDDEHQRMFARIAGELS-QASKETQQRQIDLFTKVHPEYGAGVEKAIKV

Note: positions are from PDB; the numbers between alignments are block index
```

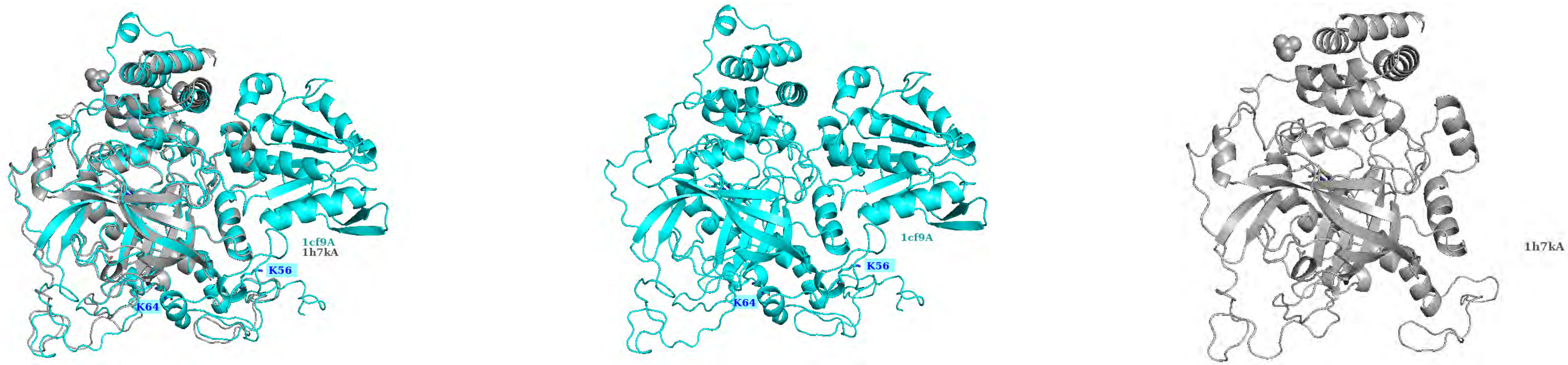

UniProt ID: P42321

PDB ID: 1M85_A

```
                          1       10        20        30        40        50       ▼ 60
P21179_ESCHERICHIA_COLI   MSQHNEKNPHQHQSPLHDSSEAKPGMDSLAPEDGSHRPAAEPTPPGAQPTAPGSLKAPDT
P42321_PROTEUS_MIRABILIS  ............................................................

                          ▼        70        80        90       100       110       120
P21179_ESCHERICHIA_COLI   RNEKLNSLEDVRKGSENYALTTNQGVRIADDQNSLRAGSRGPTLLEDFILREKITHFDHE
P42321_PROTEUS_MIRABILIS  ..............MEKKKLTTAAGAPVVDNNNVITAGPRGPMLLQDVWFLEKLAHFDRE
```

Full sequences in supplemental file.

```
Align 1cf9.A.pdb 727 with 1m85.A.pdb 476
Twists 0 ini-len 448 ini-rmsd 1.28 opt-equ 473 opt-rmsd 1.50 chain-rmsd 1.28 Score 1284.23 align-len 488 gaps 15 (3.07%)
P-value 0.00e+00 Afp-num 112076 Identity 40.78% Similarity 57.17%
Block  0 afp 56 score 1284.23 rmsd  1.28 gap 35 (0.07%)

                   .    :    .    :    .    :    .    :    .    :    .    :    .    :
Chain 1:   78 YALTTNQGVRIADDQNSLRAGSRGPTLLEDFILREKITHFDHERIPERIVHARGSAAHGYFQPYKSLSDI
              1111111111111111111111111111111111111111111111111111111111111111111111
Chain 2:    4 KKLTTAAGAPVVDNNNVITAGPRGPMLLQDVWFLEKLAHFDREVIPERRxHAKGSGAFGTFTVTHDITKY

                   .    :    .    :    .    :    .    :    .    :    .    :    .    :
Chain 1:  148 TKADFLSDPNKITPVFVRFSTCQGGAGSADTVRDIRGFATKFYTEEGIFDLVGNNTPIFFIQDAHKFPDF
              1111111111111111111111111111111111111111111111111111111111111111111111
Chain 2:   74 TRAKIFSEVGKKTEMFARFSTVAGERGAADAERDIRGFALKFYTEEGNWDMVGNNTPVFYLRDPLKFPDL

                   .    :    .    :    .    :    .    :    .    :    .    :    .    :
Chain 1:  218 VHAVKPEPHWAIPQGQSAHDTFWDYVSLQPETLHNVMWAMSDRGIPRSYRTMEGFGIHTFRLINAEGKAT
              1111111111111    11111111111111111111111111111111111111111111111111111
Chain 2:  144 NHIVKRDPRTNMR----NMAYKWDFFSHLPESLHQLTIDMSDRGLPLSYRFVHGFGSHTYSFINKDNERF

                   .    :    .    :    .    :    .    :    .    :    .    :    .    :
Chain 1:  288 FVRFHWKPLAGKASLVWDEAQKLTGRDPDFHRRELWEAIEAGDFPEYELGFQLIPEEDEFKFDFDLLDPT
              1111111111111111111111111111111111111111111111111111111111111111111111
Chain 2:  210 WVKFHFRCQQGIKNLMDDEAEALVGKDRESSQRDLFEAIERGDYPRWKLQIQIMPEKEASTVPYNPFDLT

                   .    :    .    :    .    :    .    :    .    :    .    :    .    :
Chain 1:  358 KLIPEELVPVQRVGKMVLNRNPDNFFAENEQAAFHPGHIVPGLDFTNDPLLQGRLFSYTDTQISRLGGPN
              1111111111111111111111111111111111111111111111111111111111111111111 11
Chain 2:  280 KVWPHADYPLMDVGYFELNRNPDNYFSDVEQAAFSPANIVPGISFSPDKMLQGRLFSYGDAHRYRLG-VN

                   .    :    .    :    .    :    .    :    .    :    .    :    .    :
Chain 1:  428 FHEIPINRPTCPYHNFQRDGMHRMGID-TNPANYEPNSINDNWPRETPPGPKRGGFESYQERVEGNKVRE
              111111111111111111111111111 1111111111  111111111      111111111111111
Chain 2:  349 HHQIPVNAPKCPFHNYHRDGAMRVDGNSGNGITYEPNS--GGVFQEQPD------FKEPPLSIEGAADHW

                   .    :    .    :    .    :    .    :    .    :    .    :    .
Chain 1:  497 RSPSFGEYYSHPRLFWLSQTPFEQRHIVDGFSFELSKVVRPYIERVVDQLAHIDLTLAQAVAKNLGI
              111111111111111111111111111111111111 1111111111111111111111111111111
Chain 2:  411 NHREDEDYFSQPRALYELLSDDEHQRMFARIAGELS-QASKETQQRQIDLFTKVHPEYGAGVEKAIKV
```

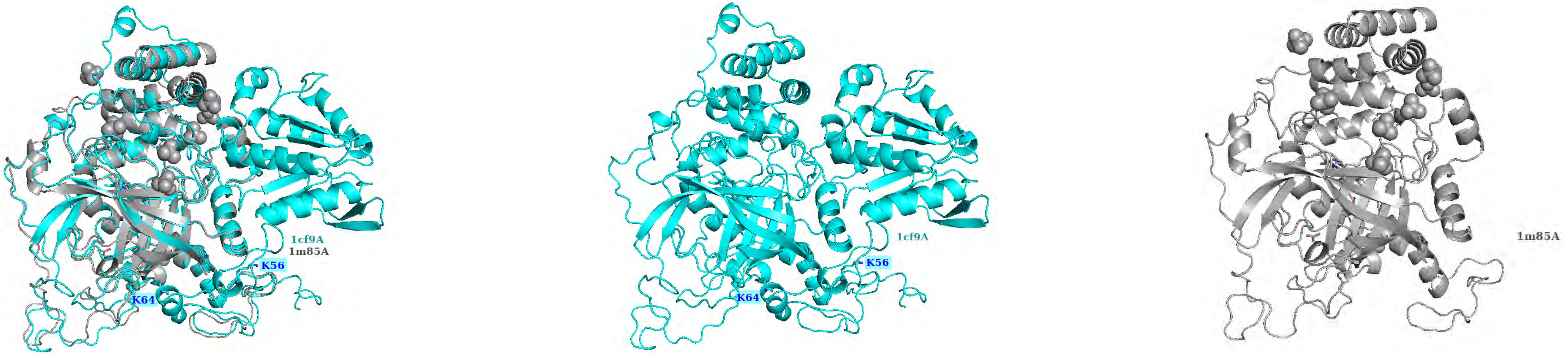

UniProt ID: P42321

PDB ID: 1MQF_A

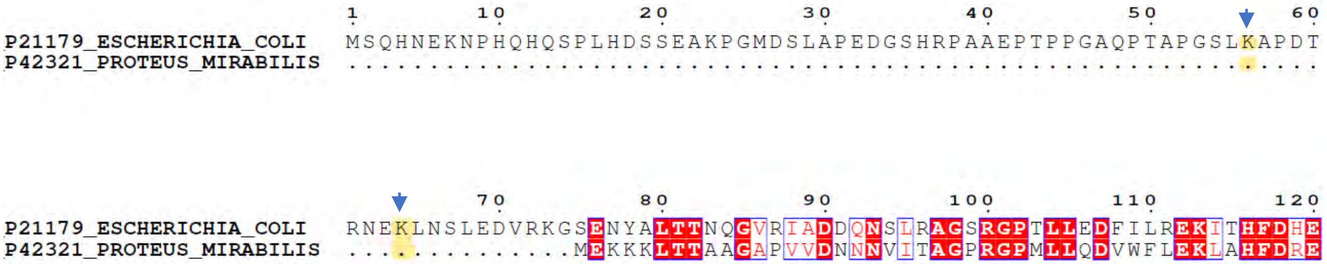

Full sequences in supplemental file.

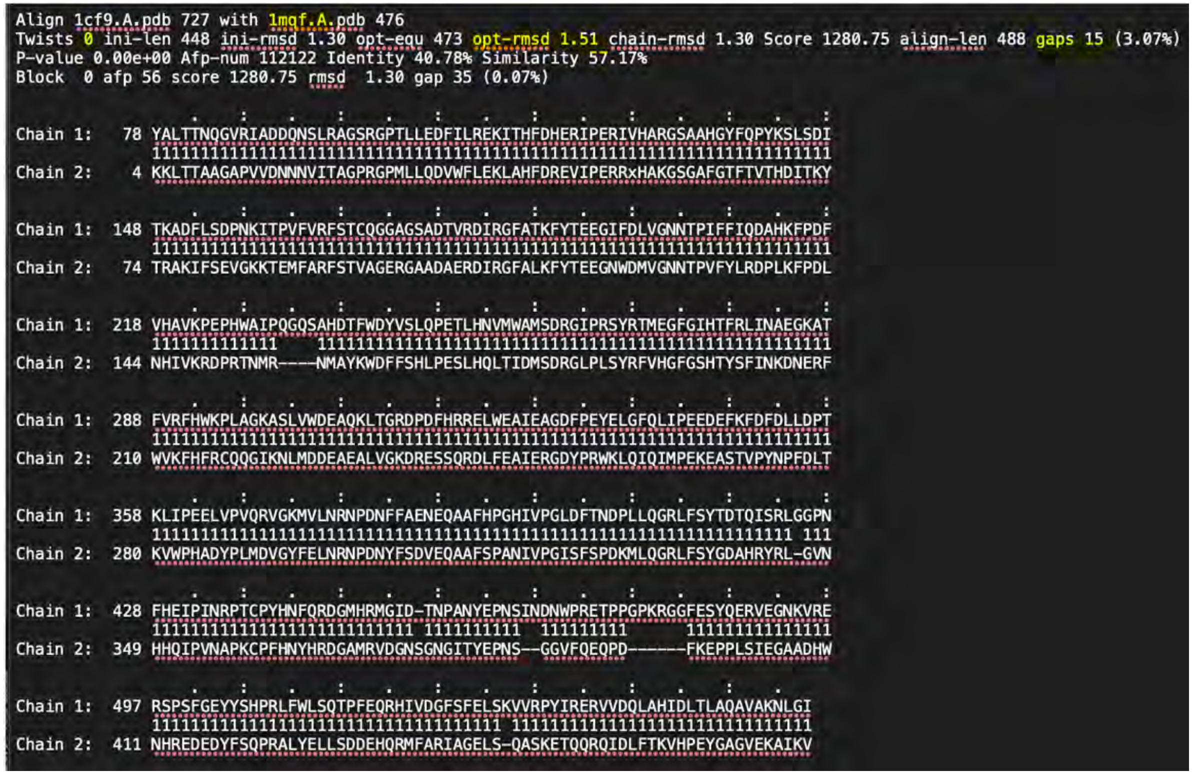

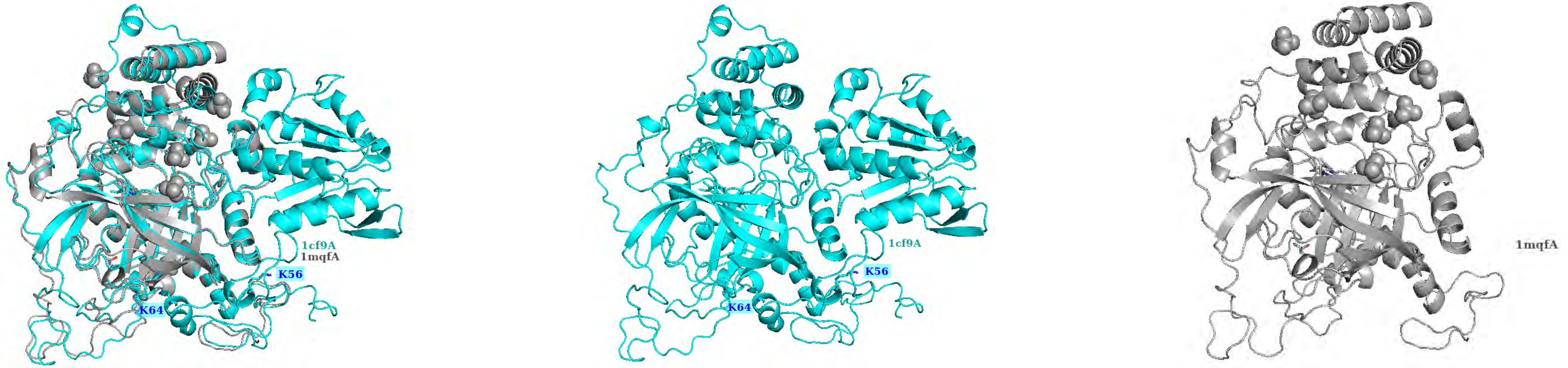

UniProt ID: P42321
PDB ID: 1NM0_A

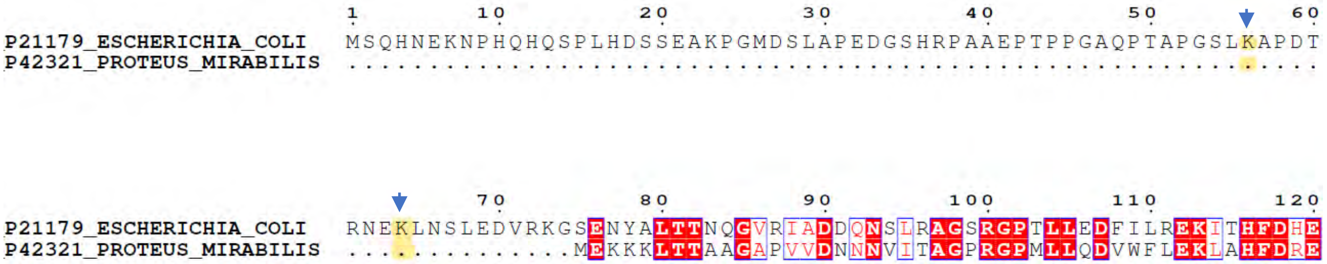

Full sequences in supplemental file.

```
Align 1cf9.A.pdb 727 with 1nm0.A.pdb 476
Twists 0 ini-len 448 ini-rmsd 1.28 opt-equ 473 opt-rmsd 1.50 chain-rmsd 1.28 Score 1285.17 align-len 488 gaps 15 (3.07%)
P-value 0.00e+00 Afp-num 112075 Identity 40.78% Similarity 57.17%
Block  0 afp 56 score 1285.17 rmsd  1.28 gap 35 (0.07%)

                    .    :    .    :    .    :    .    :    .    :    .    :    .    :
Chain 1:   78 YALTTNQGVRIADDQNSLRAGSRGPTLLEDFILREKITHFDHERIPERIVHARGSAAHGYFQPYKSLSDI
              1111111111111111111111111111111111111111111111111111111111111111111111
Chain 2:    4 KKLTTAAGAPVVDNNNVITAGPRGPMLLQDVWFLEKLAHFDREVIPERRxHAKGSGAFGTFTVTHDITKY

                    .    :    .    :    .    :    .    :    .    :    .    :    .    :
Chain 1:  148 TKADFLSDPNKITPVFVRFSTCQGGAGSADTVRDIRGFATKFYTEEGIFDLVGNNTPIFFIQDAHKFPDF
              1111111111111111111111111111111111111111111111111111111111111111111111
Chain 2:   74 TRAKIFSEVGKKTEMFARFSTVAGERGAADAERDIRGFALKFYTEEGNWDMVGNNTPVFYLRDPLKFPDL

                    .    :    .    :    .    :    .    :    .    :    .    :    .    :
Chain 1:  218 VHAVKPEPHWAIPQGQSAHDTFWDYVSLQPETLHNVMWAMSDRGIPRSYRTMEGFGIHTFRLINAEGKAT
              1111111111111    11111111111111111111111111111111111111111111111111111
Chain 2:  144 NHIVKRDPRTNMR----NMAYKWDFFSHLPESLHQLTIDMSDRGLPLSYRFVHGFGSHTYSFINKDNERF

                    .    :    .    :    .    :    .    :    .    :    .    :    .    :
Chain 1:  288 FVRFHWKPLAGKASLVWDEAQKLTGRDPDFHRRELWEAIEAGDFPEYELGFQLIPEEDEFKFDFDLLDPT
              1111111111111111111111111111111111111111111111111111111111111111111111
Chain 2:  210 WVKFHFRCQQGIKNLMDDEAEALVGKDRESSQRDLFEAIERGDYPRWKLQIQIMPEKEASTVPYNPFDLT

                    .    :    .    :    .    :    .    :    .    :    .    :    .    :
Chain 1:  358 KLIPEELVPVQRVGKMVLNRNPDNFFAENEQAAFHPGHIVPGLDFTNDPLLQGRLFSYTDTQISRLGGPN
              111111111111111111111111111111111111111111111111111111111111111111 111
Chain 2:  280 KVWPHADYPLMDVGYFELNRNPDNYFSDVEQAAFSPANIVPGISFSPDKMLQGRLFSYGDAHRYRL-GVN

                    .    :    .    :    .    :    .    :    .    :    .    :    .    :
Chain 1:  428 FHEIPINRPTCPYHNFQRDGMHRMGID-TNPANYEPNSINDNWPRETPPGPKRGGFESYQERVEGNKVRE
              111111111111111111111111111 1111111111  111111111      111111111111111
Chain 2:  349 HHQIPVNAPKCPFHNYHRDGAMRVDGNSGNGITYEPNS--GGVFQEQPD------FKEPPLSIEGAADHW

                    .    :    .    :    .    :    .    :    .    :    .    :    .
Chain 1:  497 RSPSFGEYYSHPRLFWLSQTPFEQRHIVDGFSFELSKVVRPYIRERVVDQLAHIDLTLAQAVAKNLGI
              111111111111111111111111111111111111 1111111111111111111111111111111
Chain 2:  411 NHREDEDYFSQPRALYELLSDDEHQRMFARIAGELS-QASKETQQRQIDLFTKVHPEYGAGVEKAIKV
```

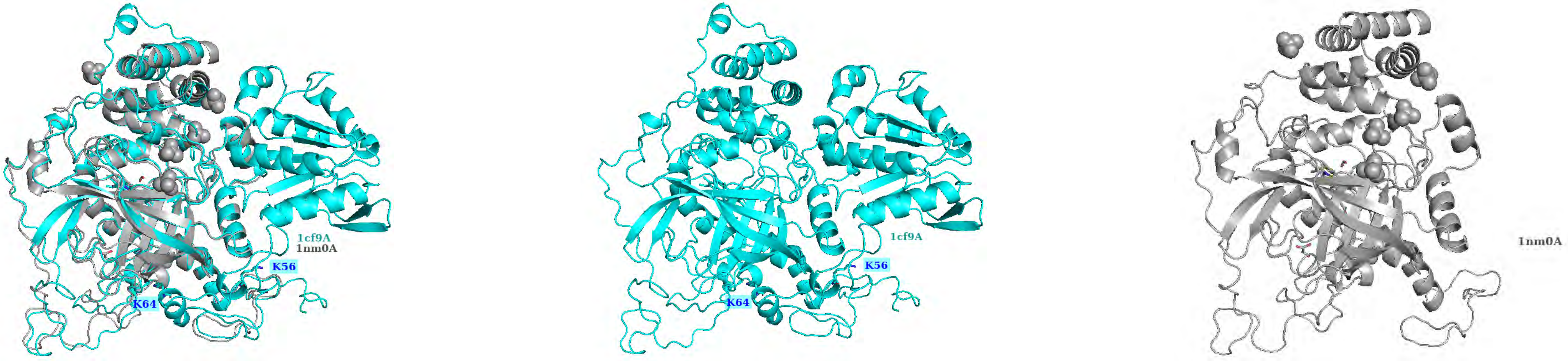

UniProt ID: P42321

PDB ID: 2CAG_A

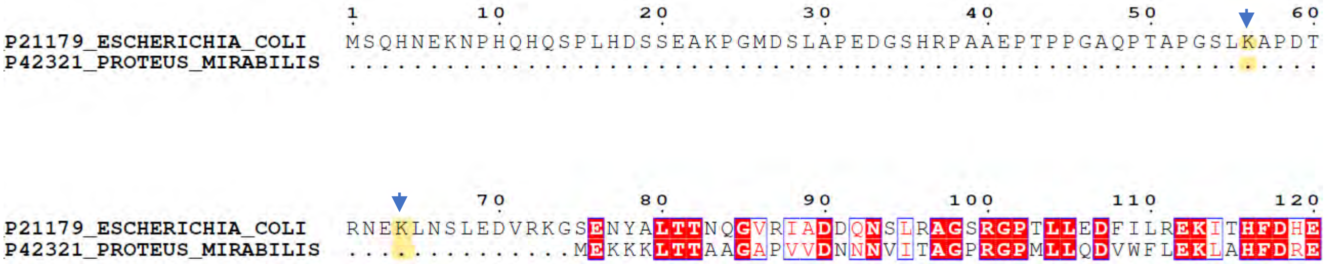

Full sequences in supplemental file.

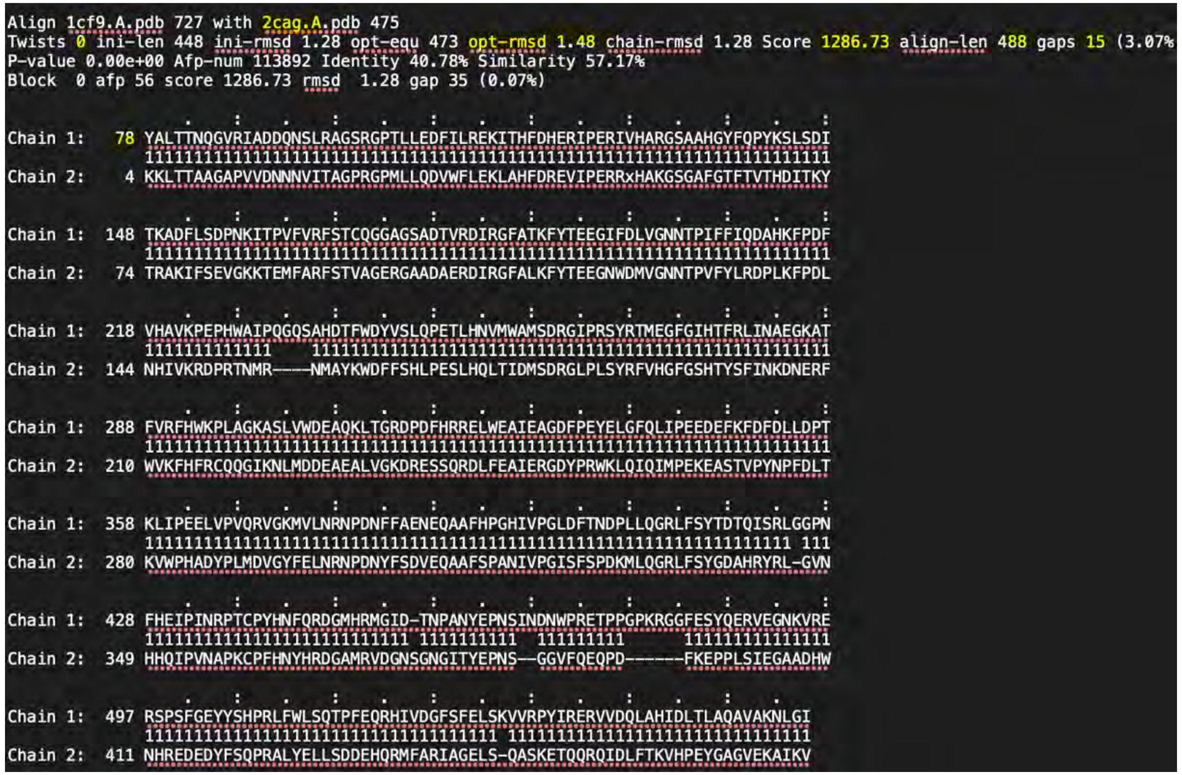

```
Align 1cf9.A.pdb 727 with 2cag.A.pdb 475
Twists 0 ini-len 448 ini-rmsd 1.28 opt-equ 473 opt-rmsd 1.48 chain-rmsd 1.28 Score 1286.73 align-len 488 gaps 15 (3.07%
P-value 0.00e+00 Afp-num 113892 Identity 40.78% Similarity 57.17%
Block  0 afp 56 score 1286.73 rmsd  1.28 gap 35 (0.07%)

              .    :    .    :    .    :    .    :    .    :    .    :    .    :
Chain 1:   78 YALTTNQGVRIADDQNSLRAGSRGPTLLEDFILREKITHFDHERIPERIVHARGSAAHGYFQPYKSLSDI
              1111111111111111111111111111111111111111111111111111111111111111111111
Chain 2:    4 KKLTTAAGAPVVDNNNVITAGPRGPMLLQDVWFLEKLAHFDREVIPERRxHAKGSGAFGTFTVTHDITKY

              .    :    .    :    .    :    .    :    .    :    .    :    .    :
Chain 1:  148 TKADFLSDPNKITPVFVRFSTCQGGAGSADTVRDIRGFATKFYTEEGIFDLVGNNTPIFFIQDAHKFPDF
              1111111111111111111111111111111111111111111111111111111111111111111111
Chain 2:   74 TRAKIFSEVGKKTEMFARFSTVAGERGAADAERDIRGFALKFYTEEGNWDMVGNNTPVFYLRDPLKFPDL

              .    :    .    :    .    :    .    :    .    :    .    :    .    :
Chain 1:  218 VHAVKPEPHWAIPQGQSAHDTFWDYVSLQPETLHNVMWAMSDRGIPRSYRTMEGFGIHTFRLINAEGKAT
              1111111111111    11111111111111111111111111111111111111111111111111111
Chain 2:  144 NHIVKRDPRTNMR----NMAYKWDFFSHLPESLHQLTIDMSDRGLPLSYRFVHGFGSHTYSFINKDNERF

              .    :    .    :    .    :    .    :    .    :    .    :    .    :
Chain 1:  288 FVRFHWKPLAGKASLVWDEAQKLTGRDPDFHRRELWEAIEAGDFPEYELGFQLIPEEDEFKFDFDLLDPT
              1111111111111111111111111111111111111111111111111111111111111111111111
Chain 2:  210 WVKFHFRCQQGIKNLMDDEAEALVGKDRESSQRDLFEAIERGDYPRWKLQIQIMPEKEASTVPYNPFDLT

              .    :    .    :    .    :    .    :    .    :    .    :    .    :
Chain 1:  358 KLIPEELVPVQRVGKMVLNRNPDNFFAENEQAAFHPGHIVPGLDFTNDPLLQGRLFSYTDTQISRLGGPN
              1111111111111111111111111111111111111111111111111111111111111111111 111
Chain 2:  280 KVWPHADYPLMDVGYFELNRNPDNYFSDVEQAAFSPANIVPGISFSPDKMLQGRLFSYGDAHRYRL-GVN

              .    :    .    :    .    :    .    :    .    :    .    :    .    :
Chain 1:  428 FHEIPINRPTCPYHNFQRDGMHRMGID-TNPANYEPNSINDNWPRETPPGPKRGGFESYQERVEGNKVRE
              111111111111111111111111111 1111111111  111111111      111111111111111
Chain 2:  349 HHQIPVNAPKCPFHNYHRDGAMRVDGNSGNGITYEPNS--GGVFQEQPD------FKEPPLSIEGAADHW

              .    :    .    :    .    :    .    :    .    :    .    :    .
Chain 1:  497 RSPSFGEYYSHPRLFWLSQTPFEQRHIVDGFSFELSKVVRPYIRERVVDQLAHIDLTLAQAVAKNLGI
              11111111111111111111111111111111111 11111111111111111111111111111111
Chain 2:  411 NHREDEDYFSQPRALYELLSDDEHQRMFARIAGELS-QASKETQQRQIDLFTKVHPEYGAGVEKAIKV
```

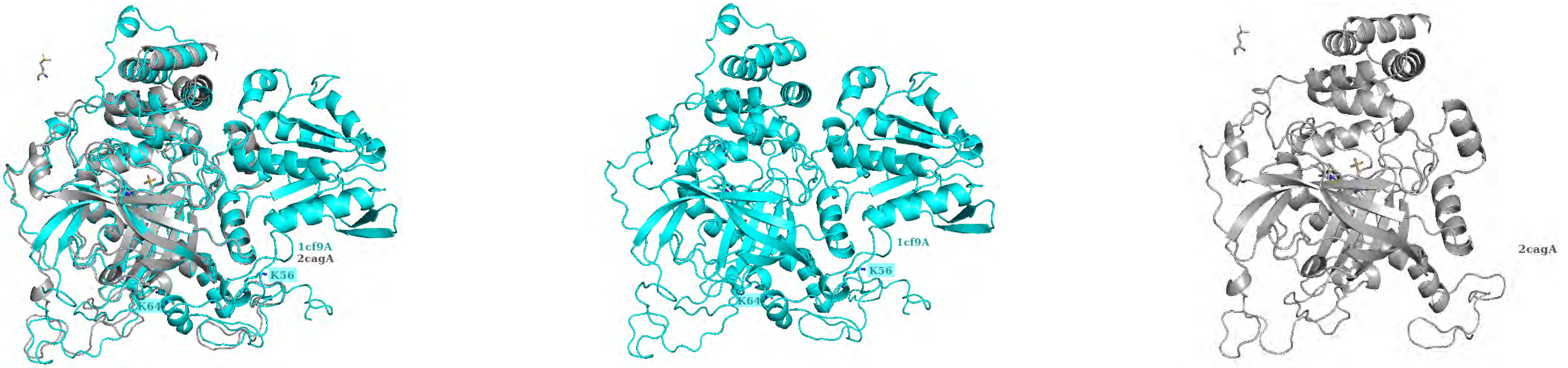

UniProt ID: P42321

PDB ID: 2CAH_A

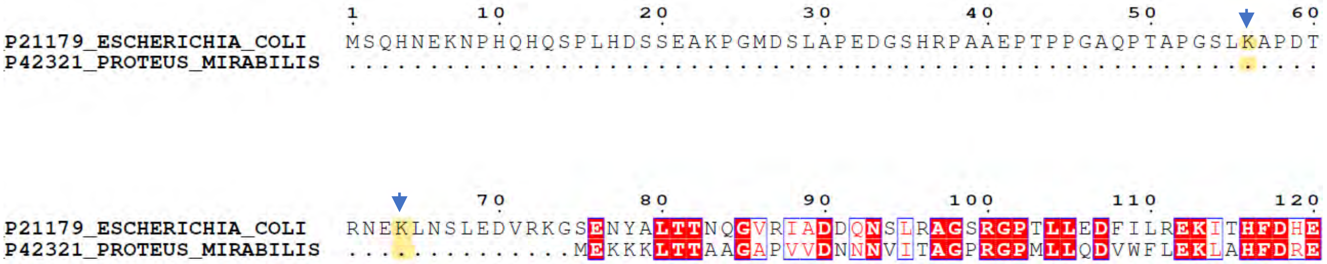

Full sequences in supplemental file.

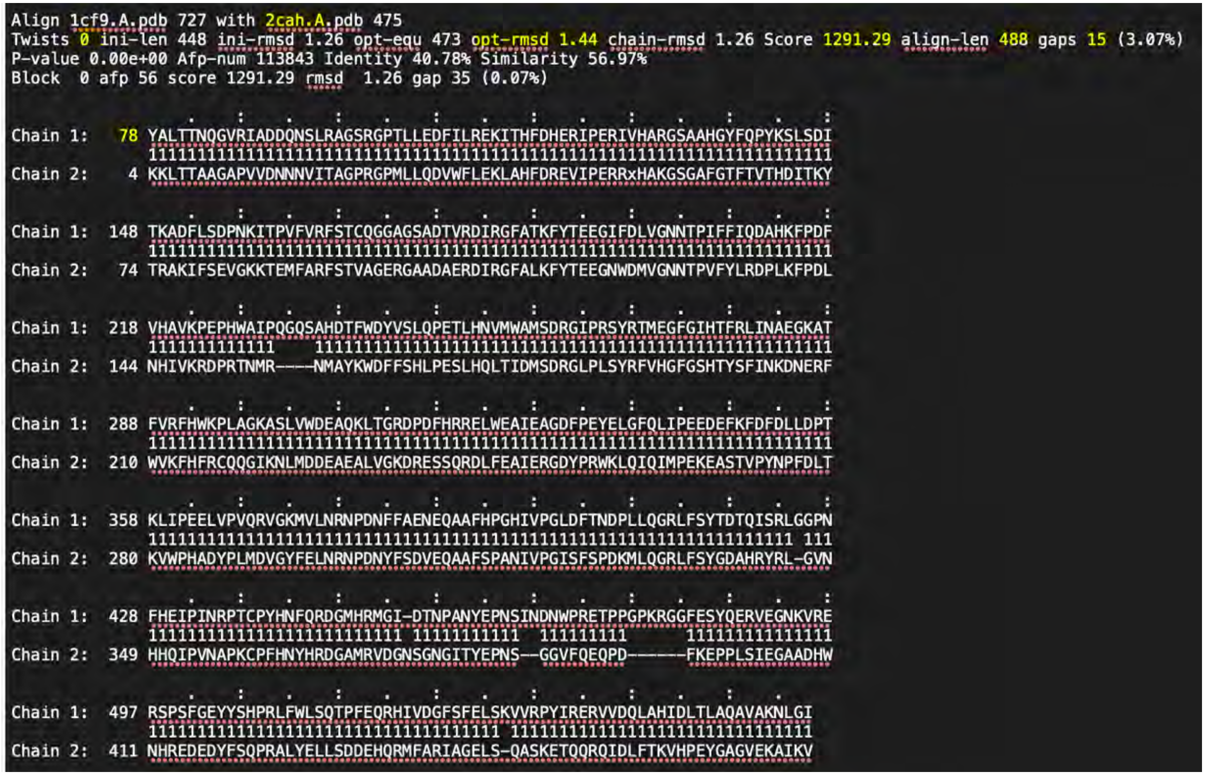

```
Align 1cf9.A.pdb 727 with 2cah.A.pdb 475
Twists 0 ini-len 448 ini-rmsd 1.26 opt-equ 473 opt-rmsd 1.44 chain-rmsd 1.26 Score 1291.29 align-len 488 gaps 15 (3.07%)
P-value 0.00e+00 Afp-num 113843 Identity 40.78% Similarity 56.97%
Block  0 afp 56 score 1291.29 rmsd  1.26 gap 35 (0.07%)

Chain 1:   78 YALTTNQGVRIADDQNSLRAGSRGPTLLEDFILREKITHFDHERIPERIVHARGSAAHGYFQPYKSLSDI
              1111111111111111111111111111111111111111111111111111111111111111111111
Chain 2:    4 KKLTTAAGAPVVDNNNVITAGPRGPMLLQDVWFLEKLAHFDREVIPERRxHAKGSGAFGTFTVTHDITKY

Chain 1:  148 TKADFLSDPNKITPVFVRFSTCQGGAGSADTVRDIRGFATKFYTEEGIFDLVGNNTPIFFIQDAHKFPDF
              1111111111111111111111111111111111111111111111111111111111111111111111
Chain 2:   74 TRAKIFSEVGKKTEMFARFSTVAGERGAADAERDIRGFALKFYTEEGNWDMVGNNTPVFYLRDPLKFPDL

Chain 1:  218 VHAVKPEPHWAIPQGQSAHDTFWDYVSLQPETLHNVMWAMSDRGIPRSYRTMEGFGIHTFRLINAEGKAT
              1111111111111    1111111111111111111111111111111111111111111111111111
Chain 2:  144 NHIVKRDPRTNMR----NMAYKWDFFSHLPESLHQLTIDMSDRGLPLSYRFVHGFGSHTYSFINKDNERF

Chain 1:  288 FVRFHWKPLAGKASLVWDEAQKLTGRDPDFHRRELWEAIEAGDFPEYELGFQLIPEEDEFKFDFDLLDPT
              1111111111111111111111111111111111111111111111111111111111111111111111
Chain 2:  210 WVKFHFRCQQGIKNLMDDEAEALVGKDRESSQRDLFEAIERGDYPRWKLQIQIMPEKEASTVPYNPFDLT

Chain 1:  358 KLIPEELVPVQRVGKMVLNRNPDNFFAENEQAAFHPGHIVPGLDFTNDPLLQGRLFSYTDTQISRLGGPN
              111111111111111111111111111111111111111111111111111111111111111111 111
Chain 2:  280 KVWPHADYPLMDVGYFELNRNPDNYFSDVEQAAFSPANIVPGISFSPDKMLQGRLFSYGDAHRYRL-GVN

Chain 1:  428 FHEIPINRPTCPYHNFQRDGMHRMGI-DTNPANYEPNSINDNWPRETPPGPKRGGFESYQERVEGNKVRE
              11111111111111111111111111 11111111111  111111111      111111111111111
Chain 2:  349 HHQIPVNAPKCPFHNYHRDGAMRVDGNSGNGITYEPNS--GGVFQEQPD------FKEPPLSIEGAADHW

Chain 1:  497 RSPSFGEYYSHPRLFWLSQTPFEQRHIVDGFSFELSKVVRPYIRERVVDQLAHIDLTLAQAVAKNLGI
              111111111111111111111111111111111111 1111111111111111111111111111111
Chain 2:  411 NHREDEDYFSQPRALYELLSDDEHQRMFARIAGELS-QASKETQQRQIDLFTKVHPEYGAGVEKAIKV
```

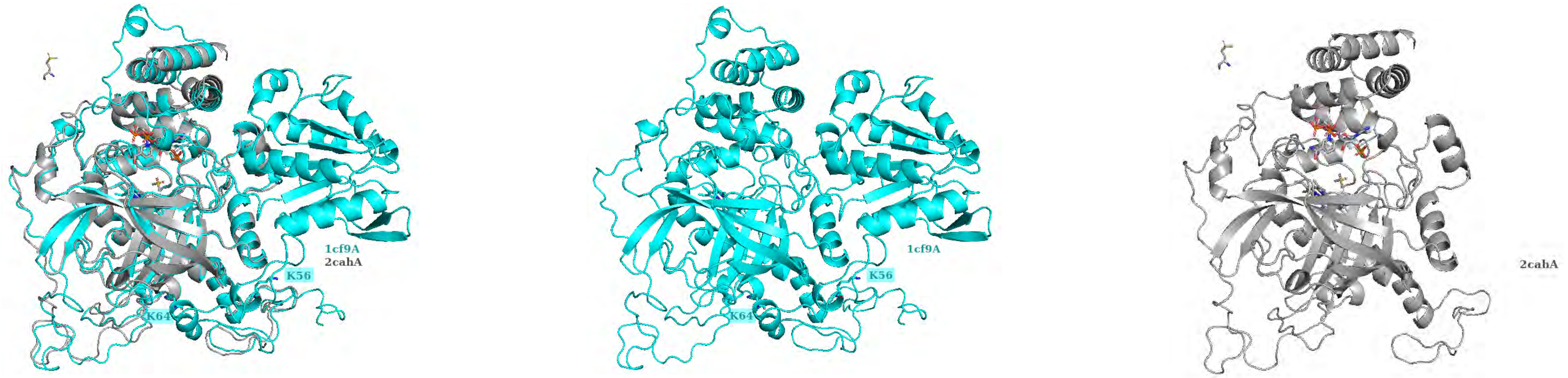

UniProt ID: P42321

PDB ID: 3HB6_A

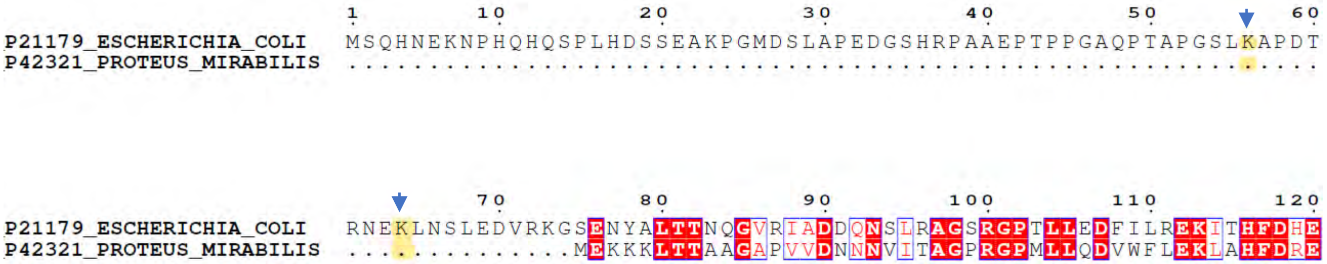

Full sequences in supplemental file.

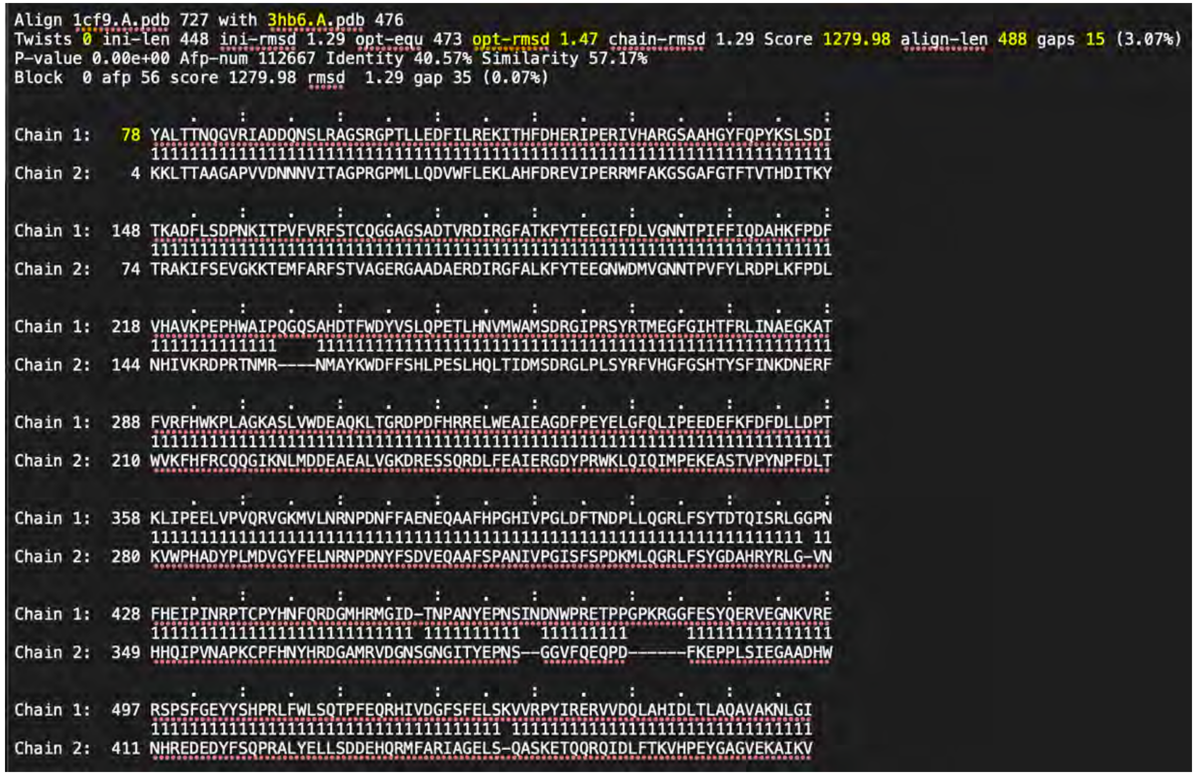

```
Align 1cf9.A.pdb 727 with 3hb6.A.pdb 476
Twists 0 ini-len 448 ini-rmsd 1.29 opt-equ 473 opt-rmsd 1.47 chain-rmsd 1.29 Score 1279.98 align-len 488 gaps 15 (3.07%)
P-value 0.00e+00 Afp-num 112667 Identity 40.57% Similarity 57.17%
Block  0 afp 56 score 1279.98 rmsd  1.29 gap 35 (0.07%)

                .    :    .    :    .    :    .    :    .    :    .    :    .    :
Chain 1:   78 YALTTNQGVRIADDQNSLRAGSRGPTLLEDFILREKITHFDHERIPERIVHARGSAAHGYFQPYKSLSDI
              1111111111111111111111111111111111111111111111111111111111111111111111
Chain 2:    4 KKLTTAAGAPVVDNNNVITAGPRGPMLLQDVWFLEKLAHFDREVIPERRMFAKGSGAFGTFTVTHDITKY

                .    :    .    :    .    :    .    :    .    :    .    :    .    :
Chain 1:  148 TKADFLSDPNKITPVFVRFSTCQGGAGSADTVRDIRGFATKFYTEEGIFDLVGNNTPIFFIQDAHKFPDF
              1111111111111111111111111111111111111111111111111111111111111111111111
Chain 2:   74 TRAKIFSEVGKKTEMFARFSTVAGERGAADAERDIRGFALKFYTEEGNWDMVGNNTPVFYLRDPLKFPDL

                .    :    .    :    .    :    .    :    .    :    .    :    .    :
Chain 1:  218 VHAVKPEPHWAIPQGQSAHDTFWDYVSLQPETLHNVMWAMSDRGIPRSYRTMEGFGIHTFRLINAEGKAT
              1111111111111    11111111111111111111111111111111111111111111111111111
Chain 2:  144 NHIVKRDPRTNMR----NMAYKWDFFSHLPESLHQLTIDMSDRGLPLSYRFVHGFGSHTYSFINKDNERF

                .    :    .    :    .    :    .    :    .    :    .    :    .    :
Chain 1:  288 FVRFHWKPLAGKASLVWDEAQKLTGRDPDFHRRELWEAIEAGDFPEYELGFQLIPEEDEFKFDFDLLDPT
              1111111111111111111111111111111111111111111111111111111111111111111111
Chain 2:  210 WVKFHFRCQQGIKNLMDDEAEALVGKDRESSQRDLFEAIERGDYPRWKLQIQIMPEKEASTVPYNPFDLT

                .    :    .    :    .    :    .    :    .    :    .    :    .    :
Chain 1:  358 KLIPEELVPVQRVGKMVLNRNPDNFFAENEQAAFHPGHIVPGLDFTNDPLLQGRLFSYTDTQISRLGGPN
              111111111111111111111111111111111111111111111111111111111111111111 11
Chain 2:  280 KVWPHADYPLMDVGYFELNRNPDNYFSDVEQAAFSPANIVPGISFSPDKMLQGRLFSYGDAHRYRLG-VN

                .    :    .    :    .    :    .    :    .    :    .    :    .    :
Chain 1:  428 FHEIPINRPTCPYHNFQRDGMHRMGID-TNPANYEPNSINDNWPRETPPGPKRGGFESYQERVEGNKVRE
              111111111111111111111111111 1111111111  111111111      111111111111111
Chain 2:  349 HHQIPVNAPKCPFHNYHRDGAMRVDGNSGNGITYEPNS--GGVFQEQPD------FKEPPLSIEGAADHW

                .    :    .    :    .    :    .    :    .    :    .    :    .
Chain 1:  497 RSPSFGEYYSHPRLFWLSQTPFEQRHIVDGFSFELSKVVRPYIRERVVDQLAHIDLTLAQAVAKNLGI
              111111111111111111111111111111111111 1111111111111111111111111111111
Chain 2:  411 NHREDEDYFSQPRALYELLSDDEHQRMFARIAGELS-QASKETQQRQIDLFTKVHPEYGAGVEKAIKV
```

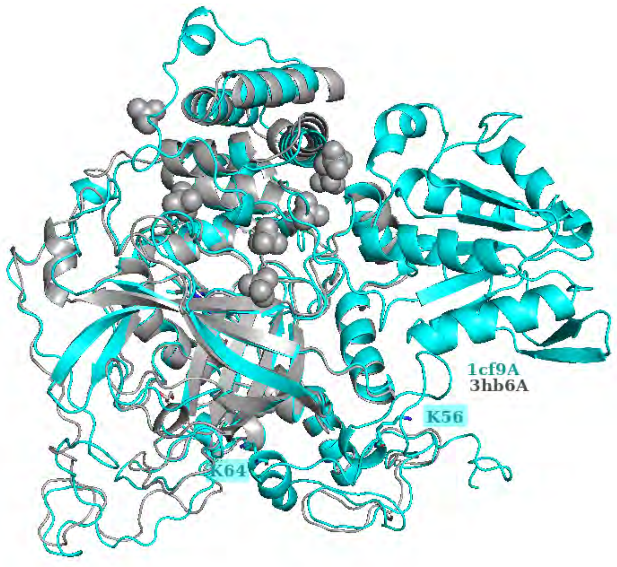

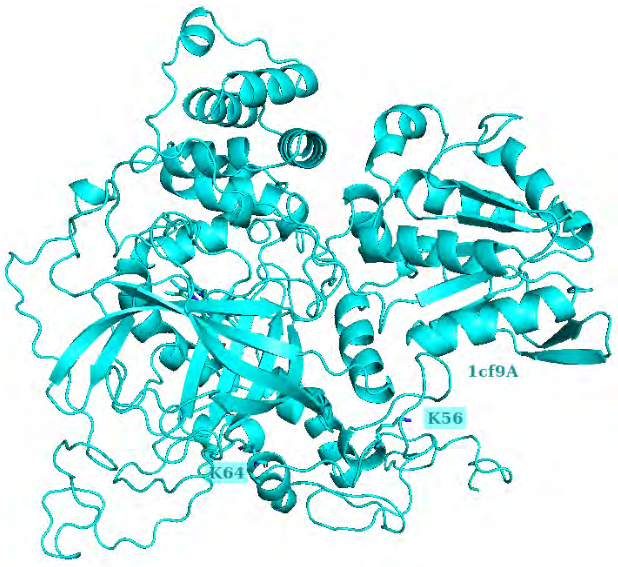

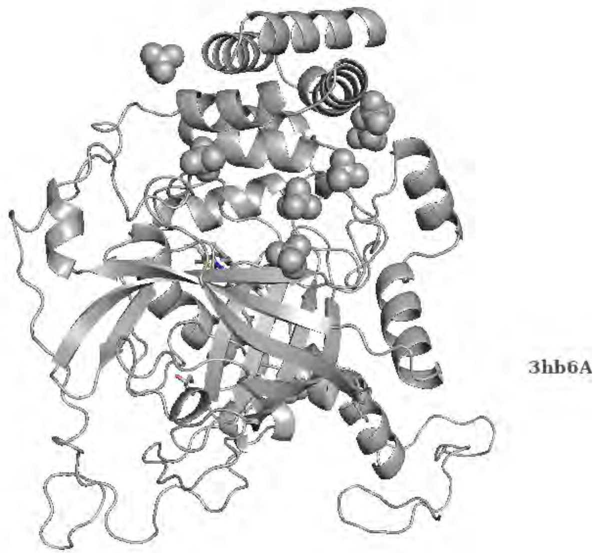

UniProt ID: P46206
PDB ID: 1M7S_B

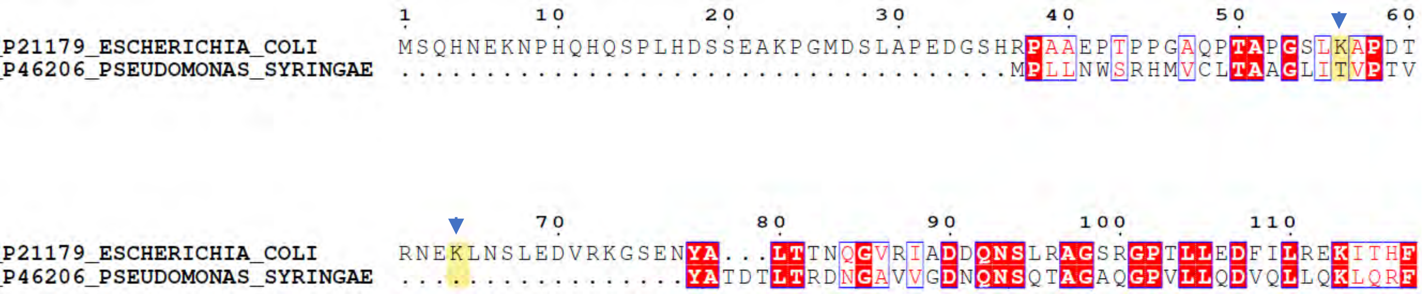

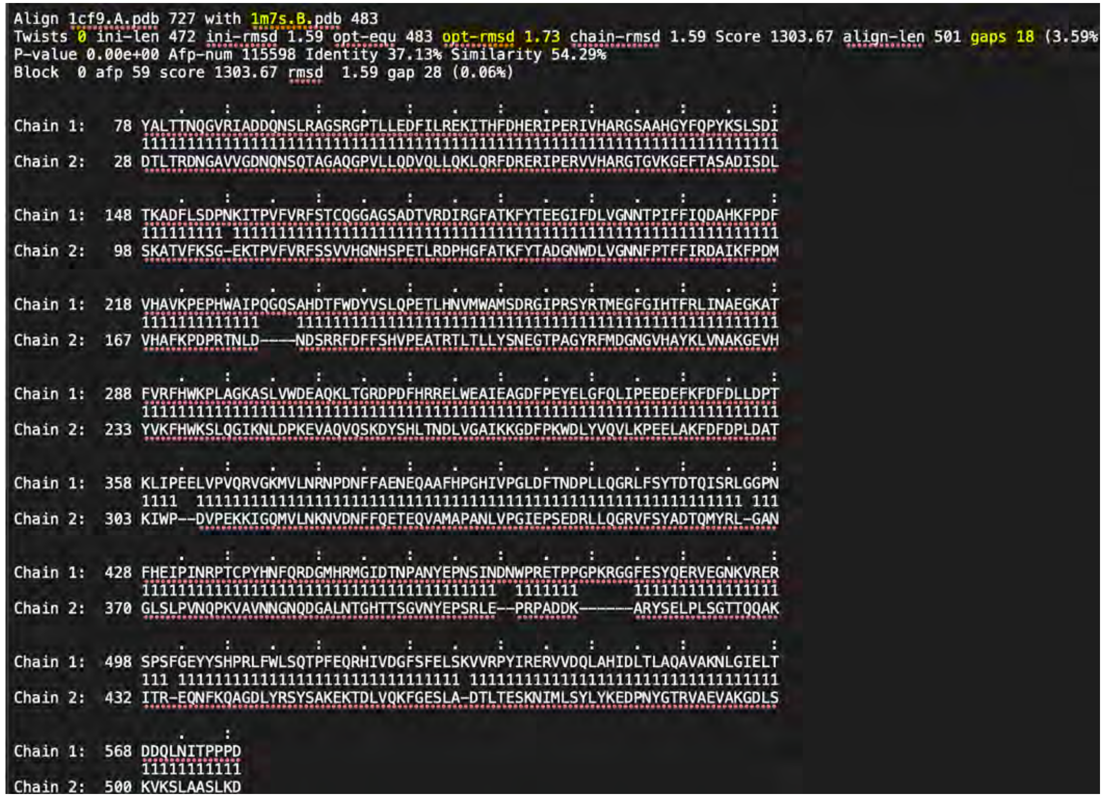

Full sequences in supplemental file.

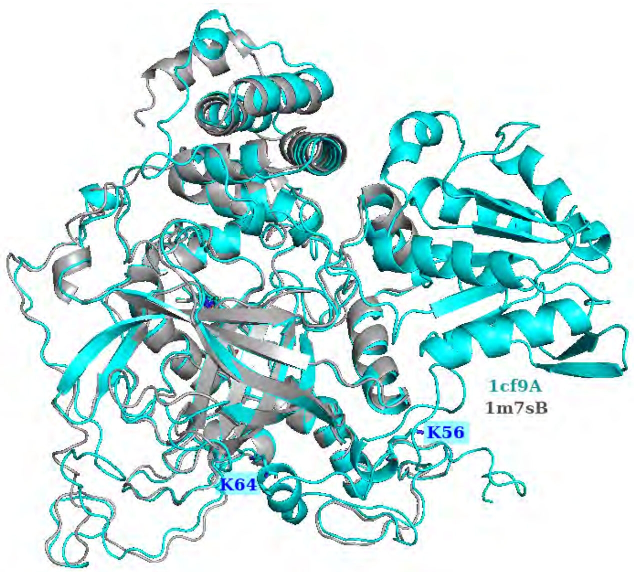

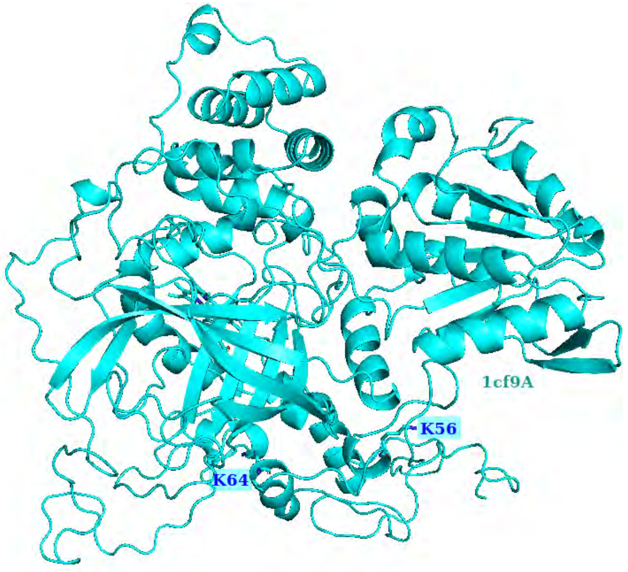

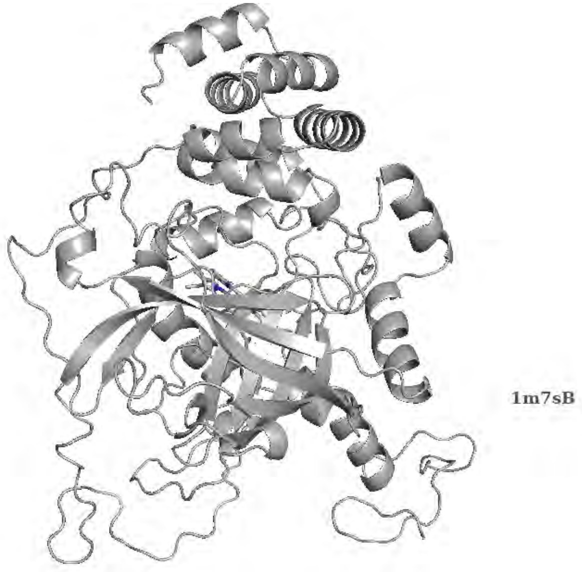

UniProt ID: P77872
PDB ID: 1QWL_B

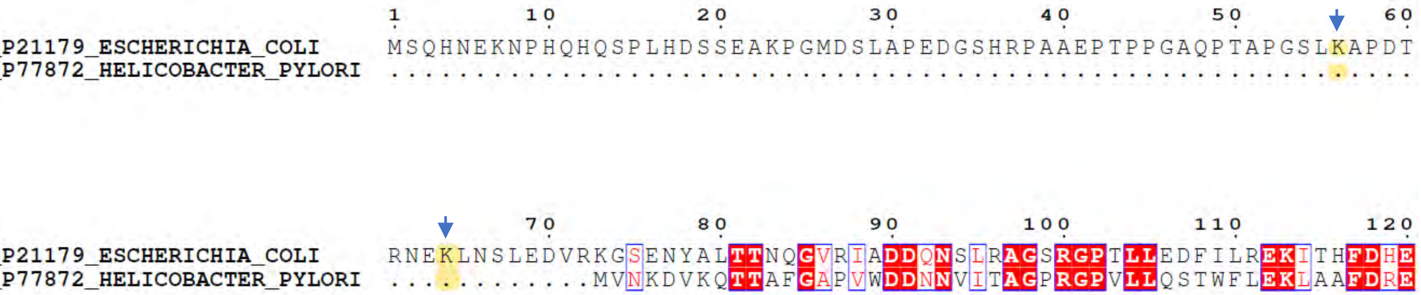

Full sequences in supplemental file.

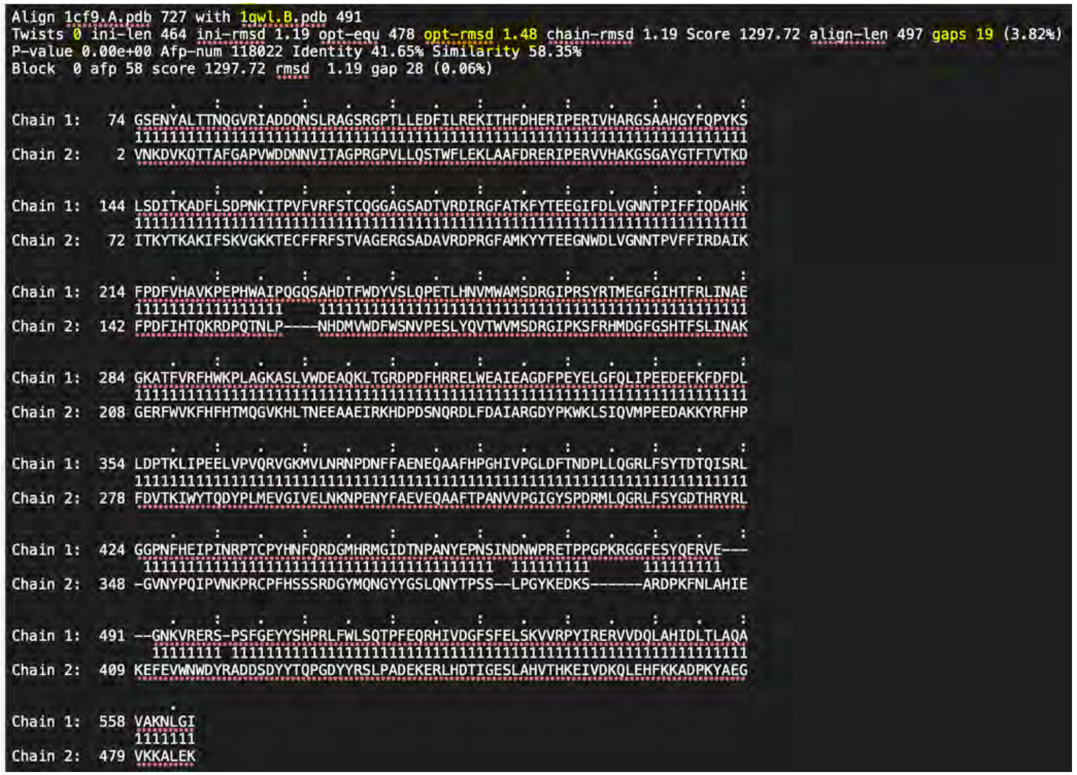

```
Align 1cf9.A.pdb 727 with 1qwl.B.pdb 491
Twists 0 ini-len 464 ini-rmsd 1.19 opt-equ 478 opt-rmsd 1.48 chain-rmsd 1.19 Score 1297.72 align-len 497 gaps 19 (3.82%)
P-value 0.00e+00 Afp-num 118022 Identity 41.65% Similarity 58.35%
Block  0 afp 58 score 1297.72 rmsd  1.19 gap 28 (0.06%)

Chain 1:   74 GSENYALTTNQGVRIADDQNSLRAGSRGPTLLEDFILREKITHFDHERIPERIVHARGSAAHGYFQPYKS
              1111111111111111111111111111111111111111111111111111111111111111111111
Chain 2:    2 VNKDVKQTTAFGAPVWDDNNVITAGPRGPVLLQSTWFLEKLAAFDRERIPERVVHAKGSGAYGTFTVTKD

Chain 1:  144 LSDITKADFLSDPNKITPVFVRFSTCQGGAGSADTVRDIRGFATKFYTEEGIFDLVGNNTPIFFIQDAHK
              1111111111111111111111111111111111111111111111111111111111111111111111
Chain 2:   72 ITKYTKAKIFSKVGKKTECFFRFSTVAGERGSADAVRDPRGFAMKYYTEEGNWDLVGNNTPVFFIRDAIK

Chain 1:  214 FPDFVHAVKPEPHWAIPQGQSAHDTFWDYVSLQPETLHNVMWAMSDRGIPRSYRTMEGFGIHTFRLINAE
              11111111111111111    1111111111111111111111111111111111111111111111111
Chain 2:  142 FPDFIHTQKRDPQTNLP----NHDMVWDFWSNVPESLYQVTWVMSDRGIPKSFRHMDGFGSHTFSLINAK

Chain 1:  284 GKATFVRFHWKPLAGKASLVWDEAQKLTGRDPDFHRRELWEAIEAGDFPEYELGFQLIPEEDEFKFDFDL
              1111111111111111111111111111111111111111111111111111111111111111111111
Chain 2:  208 GERFWVKFHFHTMQGVKHLTNEEAAEIRKHDPDSNQRDLFDAIARGDYPKWKLSIQVMPEEDAKKYRFHP

Chain 1:  354 LDPTKLIPEELVPVQRVGKMVLNRNPDNFFAENEQAAFHPGHIVPGLDFTNDPLLQGRLFSYTDTQISRL
              1111111111111111111111111111111111111111111111111111111111111111111111
Chain 2:  278 FDVTKIWYTQDYPLMEVGIVELNKNPENYFAEVEQAAFTPANVVPGIGYSPDRMLQGRLFSYGDTHRYRL

Chain 1:  424 GGPNFHEIPINRPTCPYHNFQRDGMHRMGIDTNPANYEPNSINDNWPRETPPGPKRGGFESYQERVE---
               1111111111111111111111111111111111111111  111111111      111111111
Chain 2:  348 -GVNYPQIPVNKPRCPFHSSSRDGYMQNGYYGSLQNYTPSS--LPGYKEDKS------ARDPKFNLAHIE

Chain 1:  491 --GNKVRERS-PSFGEYYSHPRLFWLSQTPFEQRHIVDGFSFELSKVVRPYIRERVVDQLAHIDLTLAQA
                11111111 11111111111111111111111111111111111111111111111111111111111
Chain 2:  409 KEFEVWNWDYRADDSDYYTQPGDYYRSLPADEKERLHDTIGESLAHVTHKEIVDKQLEHFKKADPKYAEG

Chain 1:  558 VAKNLGI
              1111111
Chain 2:  479 VKKALEK
```

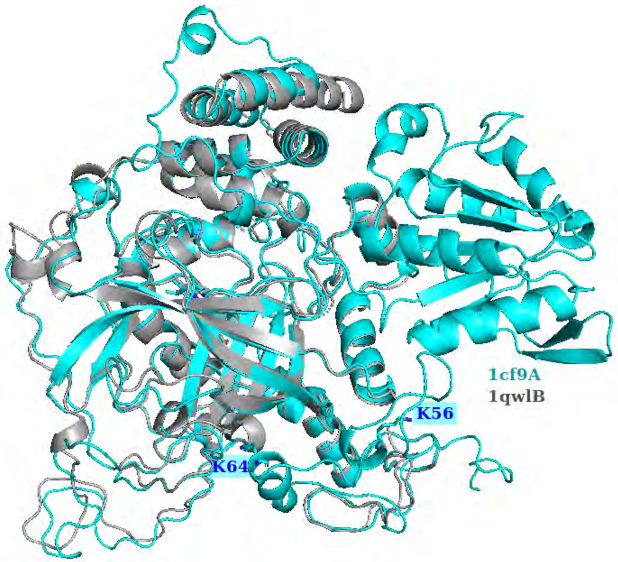

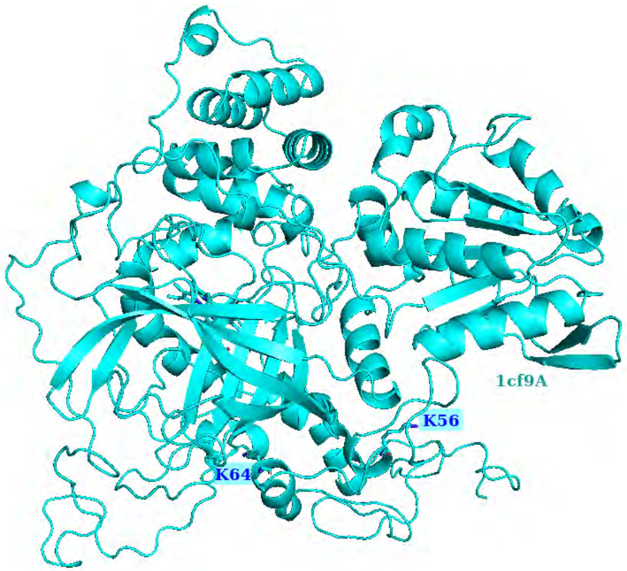

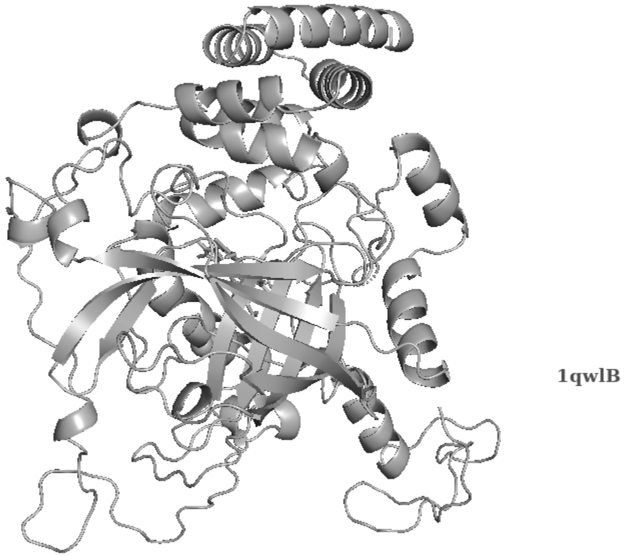

UniProt ID: P77872

PDB ID: 1QWM_B

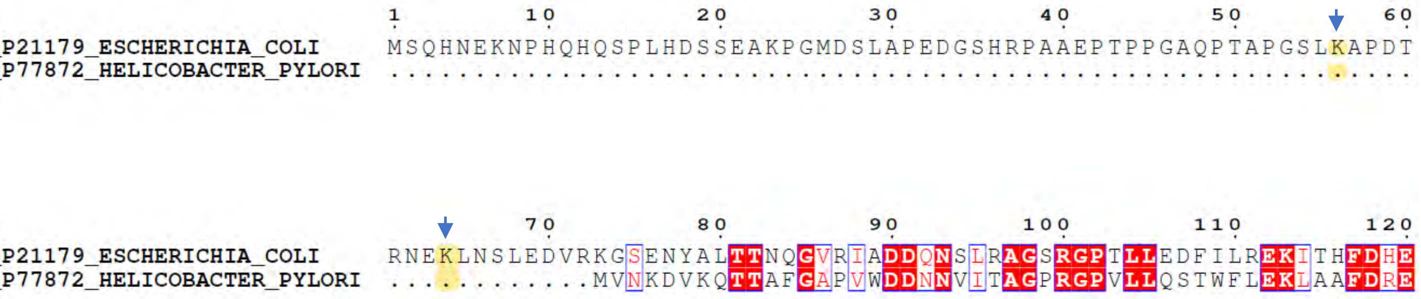

Full sequences in supplemental file.

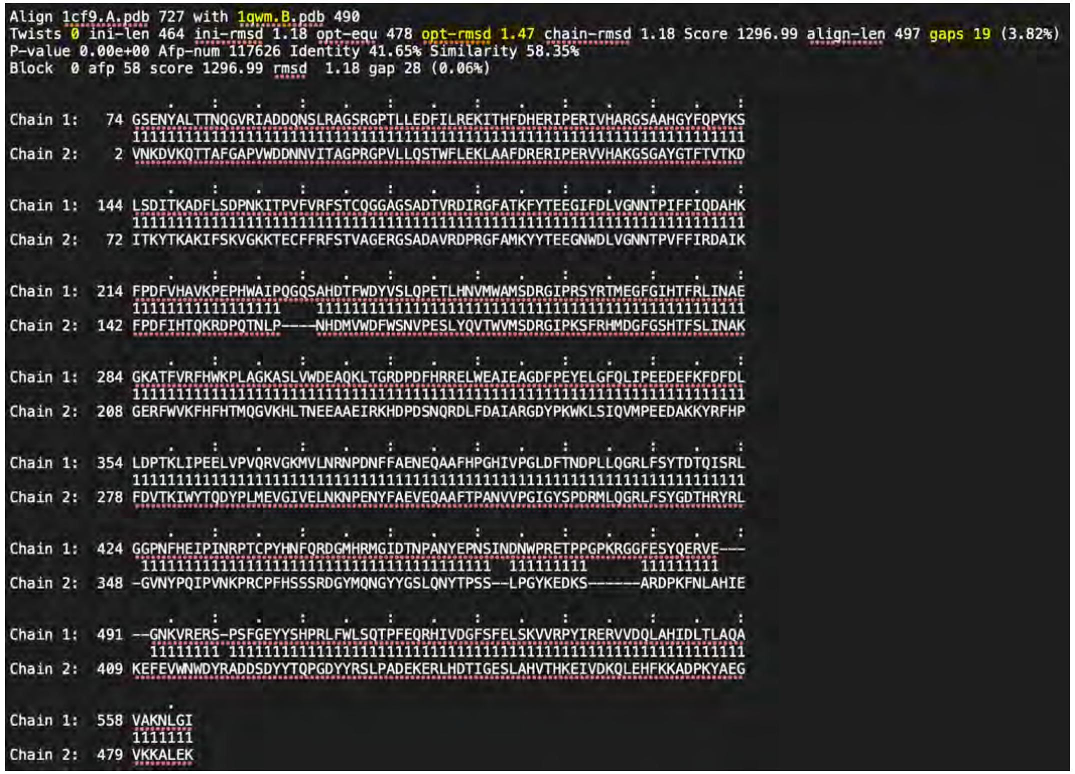
```
Align 1cf9.A.pdb 727 with 1qwm.B.pdb 490
Twists 0 ini-len 464 ini-rmsd 1.18 opt-equ 478 opt-rmsd 1.47 chain-rmsd 1.18 Score 1296.99 align-len 497 gaps 19 (3.82%)
P-value 0.00e+00 Afp-num 117626 Identity 41.65% Similarity 58.35%
Block  0 afp 58 score 1296.99 rmsd  1.18 gap 28 (0.06%)

Chain 1:   74 GSENYALTTNQGVRIADDQNSLRAGSRGPTLLEDFILREKITHFDHERIPERIVHARGSAAHGYFQPYKS
              1111111111111111111111111111111111111111111111111111111111111111111111
Chain 2:    2 VNKDVKQTTAFGAPVWDDNNVITAGPRGPVLLQSTWFLEKLAAFDRERIPERVVHAKGSGAYGTFTVTKD

Chain 1:  144 LSDITKADFLSDPNKITPVFVRFSTCQGGAGSADTVRDIRGFATKFYTEEGIFDLVGNNTPIFFIQDAHK
              1111111111111111111111111111111111111111111111111111111111111111111111
Chain 2:   72 ITKYTKAKIFSKVGKKTECFFRFSTVAGERGSADAVRDPRGFAMKYYTEEGNWDLVGNNTPVFFIRDAIK

Chain 1:  214 FPDFVHAVKPEPHWAIPQGQSAHDTFWDYVSLQPETLHNVMWAMSDRGIPRSYRTMEGFGIHTFRLINAE
              11111111111111111    11111111111111111111111111111111111111111111111111
Chain 2:  142 FPDFIHTQKRDPQTNLP----NHDMVWDFWSNVPESLYQVTWVMSDRGIPKSFRHMDGFGSHTFSLINAK

Chain 1:  284 GKATFVRFHWKPLAGKASLVWDEAQKLTGRDPDFHRRELWEAIEAGDFPEYELGFQLIPEEDEFKFDFDL
              1111111111111111111111111111111111111111111111111111111111111111111111
Chain 2:  208 GERFWVKFHFHTMQGVKHLTNEEAAEIRKHDPDSNQRDLFDAIARGDYPKWKLSIQVMPEEDAKKYRFHP

Chain 1:  354 LDPTKLIPEELVPVQRVGKMVLNRNPDNFFAENEQAAFHPGHIVPGLDFTNDPLLQGRLFSYTDTQISRL
              1111111111111111111111111111111111111111111111111111111111111111111111
Chain 2:  278 FDVTKIWYTQDYPLMEVGIVELNKNPENYFAEVEQAAFTPANVVPGIGYSPDRMLQGRLFSYGDTHRYRL

Chain 1:  424 GGPNFHEIPINRPTCPYHNFQRDGMHRMGIDTNPANYEPNSINDNWPRETPPGPKRGGFESYQERVE---
               111111111111111111111111111111111111111  111111111       111111111
Chain 2:  348 -GVNYPQIPVNKPRCPFHSSSRDGYMQNGYYGSLQNYTPSS--LPGYKEDKS------ARDPKFNLAHIE

Chain 1:  491 --GNKVRERS-PSFGEYYSHPRLFWLSQTPFEQRHIVDGFSFELSKVVRPYIRERVVDQLAHIDLTLAQA
                11111111 111111111111111111111111111111111111111111111111111111111111
Chain 2:  409 KEFEVWNWDYRADDSDYYTQPGDYYRSLPADEKERLHDTIGESLAHVTHKEIVDKQLEHFKKADPKYAEG

Chain 1:  558 VAKNLGI
              1111111
Chain 2:  479 VKKALEK
```

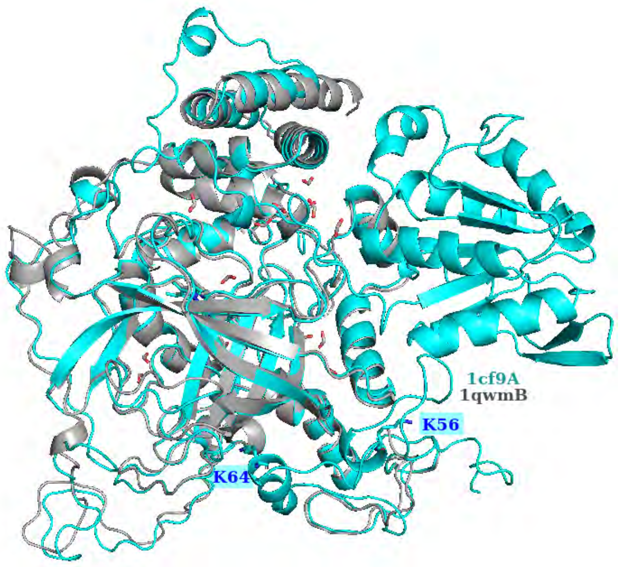

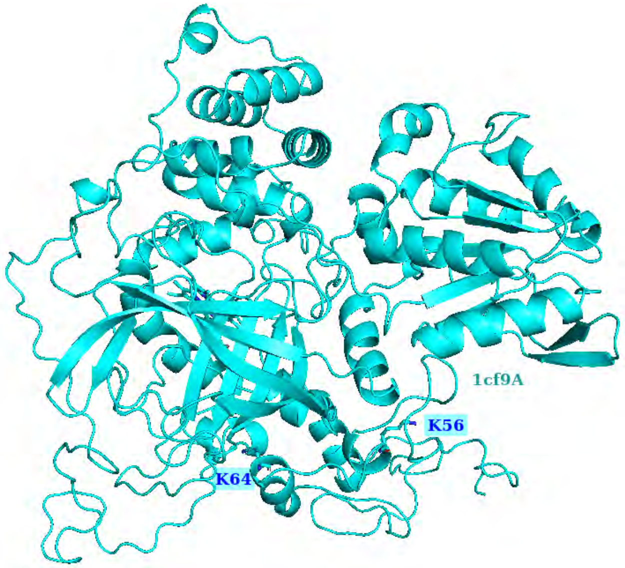

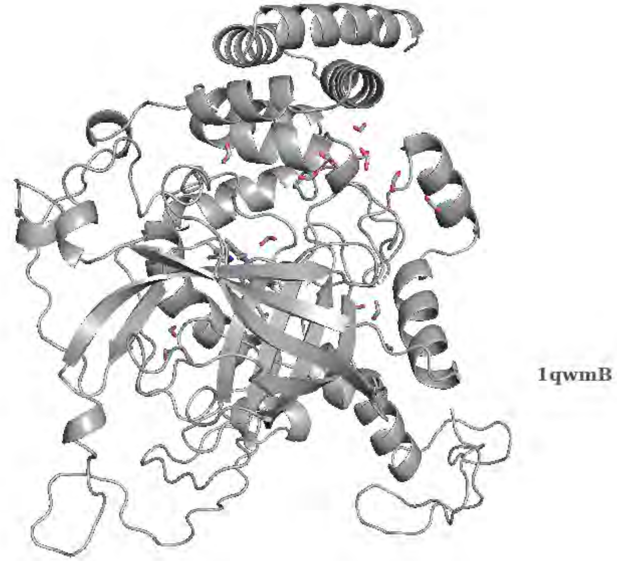

UniProt ID: P77872
PDB ID: 2A9E_B

```
                              1          10         20         30         40         50         60
P21179_ESCHERICHIA_COLI       MSQHNEKNPHQHQSPLHDSSEAKPGMDSLAPEDGSHRPAAEPTPPGAQPTAPGSLKAPDT
P77872_HELICOBACTER_PYLORI    ............................................................

                                        70         80         90        100        110        120
P21179_ESCHERICHIA_COLI       RNEKLNSLEDVRKGSENYALTTNQGVRIADDQNSLRAGSRGPTLLEDFILREKITHFDHE
P77872_HELICOBACTER_PYLORI    ............MVNKDVKQTTAFGAPVWDDNNVITAGPRGPVLLQSTWFLEKLAAFDRE
```

Full sequences in supplemental file.

```
Align 1cf9.A.pdb 727 with 2a9e.B.pdb 491
Twists 0 ini-len 464 ini-rmsd 1.18 opt-equ 478 opt-rmsd 1.47 chain-rmsd 1.18 Score 1298.08 align-len 497 gaps 19 (3.82%)
P-value 0.00e+00 Afp-num 118334 Identity 41.65% Similarity 58.35%
Block  0 afp 58 score 1298.08 rmsd  1.18 gap 27 (0.05%)

               .    :    .    :    .    :    .    :    .    :    .    :    .    :
Chain 1:   74 GSENYALTTNQGVRIADDQNSLRAGSRGPTLLEDFILREKITHFDHERIPERIVHARGSAAHGYFQPYKS
              1111111111111111111111111111111111111111111111111111111111111111111111
Chain 2:    2 VNKDVKQTTAFGAPVWDDNNVITAGPRGPVLLQSTWFLEKLAAFDRERIPERVVHAKGSGAYGTFTVTKD

               .    :    .    :    .    :    .    :    .    :    .    :    .    :
Chain 1:  144 LSDITKADFLSDPNKITPVFVRFSTCQGGAGSADTVRDIRGFATKFYTEEGIFDLVGNNTPIFFIQDAHK
              1111111111111111111111111111111111111111111111111111111111111111111111
Chain 2:   72 ITKYTKAKIFSKVGKKTECFFRFSTVAGERGSADAVRDPRGFAMKYYTEEGNWDLVGNNTPVFFIRDAIK

               .    :    .    :    .    :    .    :    .    :    .    :    .    :
Chain 1:  214 FPDFVHAVKPEPHWAIPQGQSAHDTFWDYVSLQPETLHNVMWAMSDRGIPRSYRTMEGFGIHTFRLINAE
              11111111111111111    1111111111111111111111111111111111111111111111111
Chain 2:  142 FPDFIHTQKRDPQTNLP----NHDxVWDFWSNVPESLYQVTWVxSDRGIPKSFRHMDGFGSHTFSLINAK

               .    :    .    :    .    :    .    :    .    :    .    :    .    :
Chain 1:  284 GKATFVRFHWKPLAGKASLVWDEAQKLTGRDPDFHRRELWEAIEAGDFPEYELGFQLIPEEDEFKFDFDL
              1111111111111111111111111111111111111111111111111111111111111111111111
Chain 2:  208 GERFWVKFHFHTMQGVKHLTNEEAAEIRKHDPDSNQRDLFDAIARGDYPKWKLSIQVMPEEDAKKYRFHP

               .    :    .    :    .    :    .    :    .    :    .    :    .    :
Chain 1:  354 LDPTKLIPEELVPVQRVGKMVLNRNPDNFFAENEQAAFHPGHIVPGLDFTNDPLLQGRLFSYTDTQISRL
              1111111111111111111111111111111111111111111111111111111111111111111111
Chain 2:  278 FDVTKIWYTQDYPLxEVGIVELNKNPENYFAEVEQAAFTPANVVPGIGYSPDRMLQGRLFSYGDTHRYRL

               .    :    .    :    .    :    .    :    .    :    .    :    .    :
Chain 1:  424 GGPNFHEIPINRPTCPYHNFQRDGMHRMGIDTNPANYEPNSINDNWPRETPPGPKRGGFESYQERV----
               1111111111111111111111111111111111111111  111111111      11111111
Chain 2:  348 -GVNYPQIPVNKPRCPFHSSSRDGYxQNGYYGSLQNYTPSS--LPGYKEDKS------ARDPKFNLAHIE

               .    :    .    :    .    :    .    :    .    :    .    :    .    :
Chain 1:  490 -EGNKVRERS-PSFGEYYSHPRLFWLSQTPFEQRHIVDGFSFELSKVVRPYIRERVVDQLAHIDLTLAQA
               111111111 11111111111111111111111111111111111111111111111111111111111
Chain 2:  409 KEFEVWNWDYRADDSDYYTQPGDYYRSLPADEKERLHDTIGESLAHVTHKEIVDKQLEHFKKADPKYAEG

               .
Chain 1:  558 VAKNLGI
              1111111
Chain 2:  479 VKKALEK
```

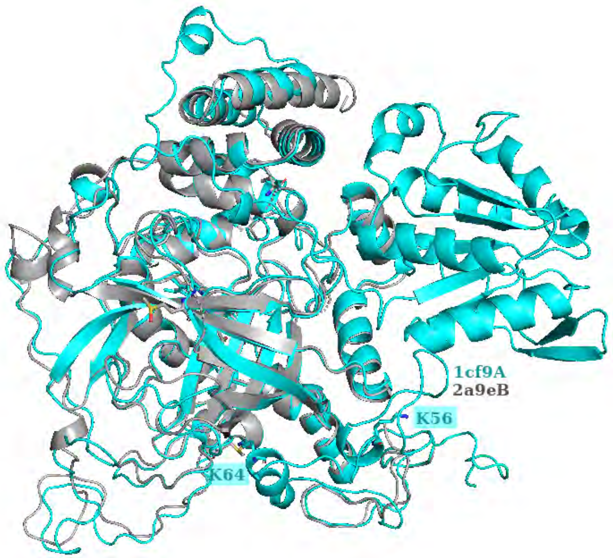

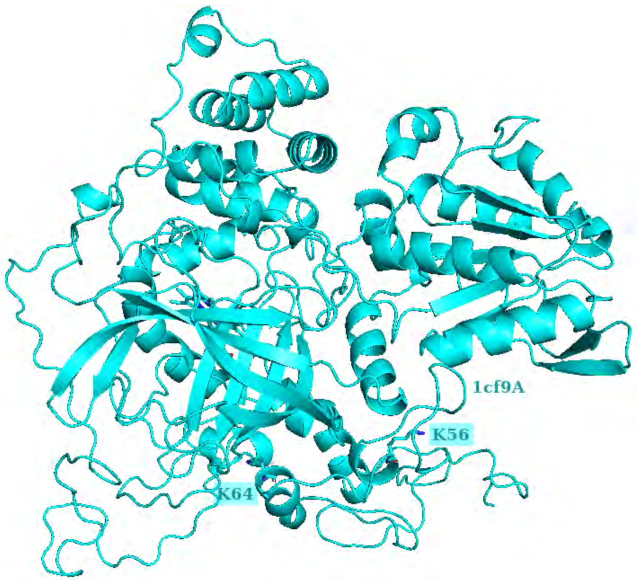

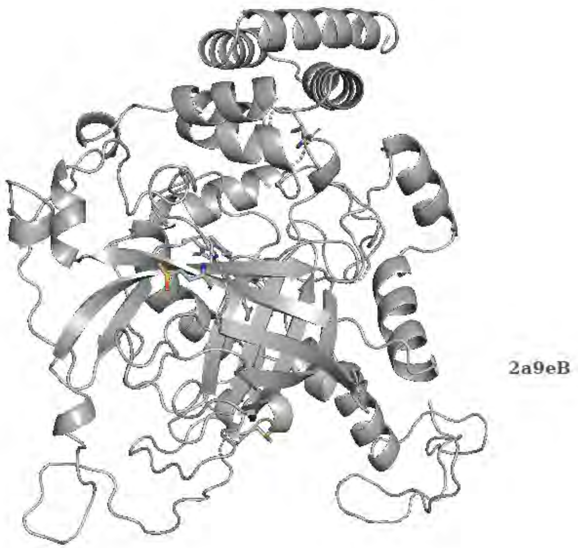

UniProt ID: P77872
PDB ID: 2IQF_B

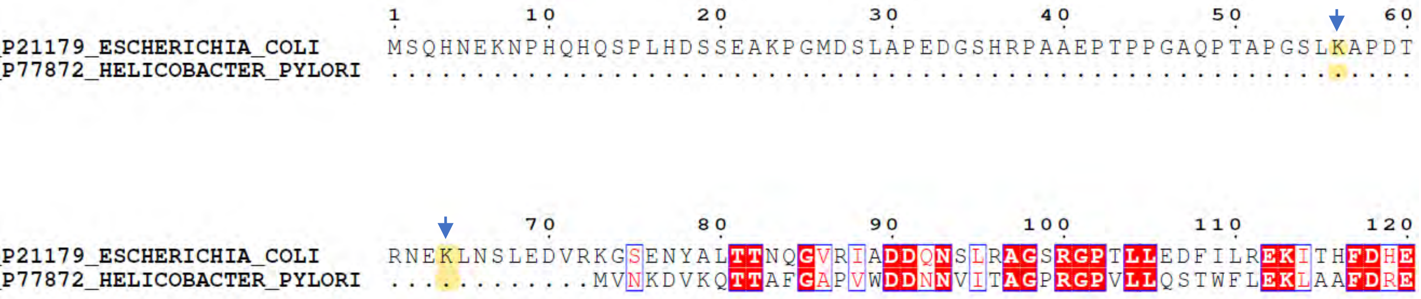

Full sequences in supplemental file.

```
Align 1cf9.A.pdb 727 with 2iqf.B.pdb 491
Twists 0 ini-len 464 ini-rmsd 1.18 opt-equ 478 opt-rmsd 1.47 chain-rmsd 1.18 Score 1297.50 align-len 497 gaps 19 (3.82%)
P-value 0.00e+00 Afp-num 117921 Identity 41.65% Similarity 58.35%
Block  0 afp 58 score 1297.50 rmsd  1.18 gap 28 (0.06%)

                  .    :    .    :    .    :    .    :    .    :    .    :    .    :
Chain 1:   74 GSENYALTTNQGVRIADDQNSLRAGSRGPTLLEDFILREKITHFDHERIPERIVHARGSAAHGYFQPYKS
              1111111111111111111111111111111111111111111111111111111111111111111111
Chain 2:    2 VNKDVKQTTAFGAPVWDDNNVITAGPRGPVLLQSTWFLEKLAAFDRERIPERVVHAKGSGAYGTFTVTKD

                  .    :    .    :    .    :    .    :    .    :    .    :    .    :
Chain 1:  144 LSDITKADFLSDPNKITPVFVRFSTCQGGAGSADTVRDIRGFATKFYTEEGIFDLVGNNTPIFFIQDAHK
              1111111111111111111111111111111111111111111111111111111111111111111111
Chain 2:   72 ITKYTKAKIFSKVGKKTECFFRFSTVAGERGSADAVRDPRGFAMKYYTEEGNWDLVGNNTPVFFIRDAIK

                  .    :    .    :    .    :    .    :    .    :    .    :    .    :
Chain 1:  214 FPDFVHAVKPEPHWAIPQGQSAHDTFWDYVSLQPETLHNVMWAMSDRGIPRSYRTMEGFGIHTFRLINAE
              11111111111111111    11111111111111111111111111111111111111111111111111
Chain 2:  142 FPDFIHTQKRDPQTNLP----NHDxVWDFWSNVPESLYQVTWVxSDRGIPKSFRHMDGFGSHTFSLINAK

                  .    :    .    :    .    :    .    :    .    :    .    :    .    :
Chain 1:  284 GKATFVRFHWKPLAGKASLVWDEAQKLTGRDPDFHRRELWEAIEAGDFPEYELGFQLIPEEDEFKFDFDL
              1111111111111111111111111111111111111111111111111111111111111111111111
Chain 2:  208 GERFWVKFHFHTMQGVKHLTNEEAAEIRKHDPDSNQRDLFDAIARGDYPKWKLSIQVMPEEDAKKYRFHP

                  .    :    .    :    .    :    .    :    .    :    .    :    .    :
Chain 1:  354 LDPTKLIPEELVPVQRVGKMVLNRNPDNFFAENEQAAFHPGHIVPGLDFTNDPLLQGRLFSYTDTQISRL
              1111111111111111111111111111111111111111111111111111111111111111111111
Chain 2:  278 FDVTKIWYTQDYPLxEVGIVELNKNPENYFAEVEQAAFTPANVVPGIGYSPDRMLQGRLFSYGDTHRYRL

                  .    :    .    :    .    :    .    :    .    :    .    :    .    :
Chain 1:  424 GGPNFHEIPINRPTCPYHNFQRDGMHRMGIDTNPANYEPNSINDNWPRETPPGPKRGGFESYQERV----
               111111111111111111111111111111111111111  111111111      11111111
Chain 2:  348 -GVNYPQIPVNKPRCPFHSSSRDGYxQNGYYGSLQNYTPSS--LPGYKEDKS------ARDPKFNLAHIE

                  .    :    .    :    .    :    .    :    .    :    .    :    .    :
Chain 1:  490 -EGNKVRERS-PSFGEYYSHPRLFWLSQTPFEQRHIVDGFSFELSKVVRPYIRERVVDQLAHIDLTLAQA
               111111111 111111111111111111111111111111111111111111111111111111111111
Chain 2:  409 KEFEVWNWDYRADDSDYYTQPGDYYRSLPADEKERLHDTIGESLAHVTHKEIVDKQLEHFKKADPKYAEG

                  .
Chain 1:  558 VAKNLGI
              1111111
Chain 2:  479 VKKALEK
```

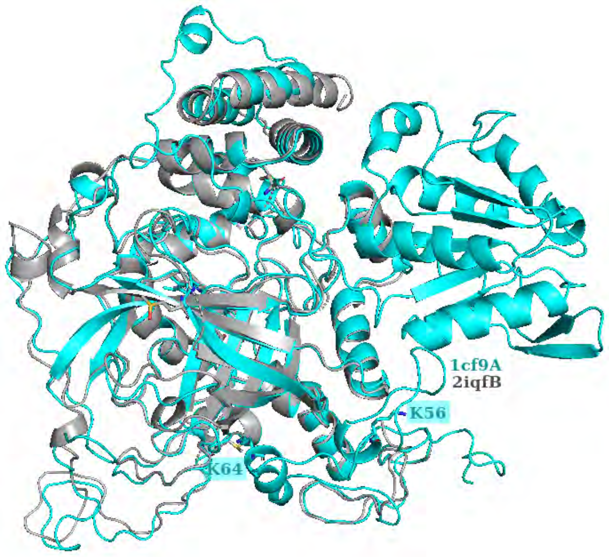

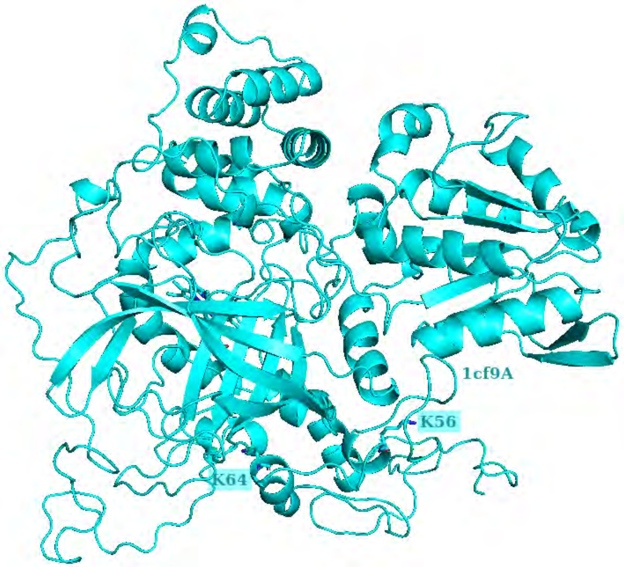

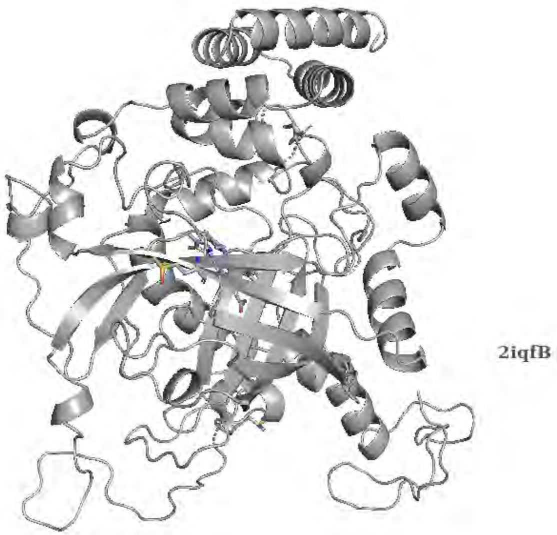

UniProt ID: Q3LSM1

PDB ID: 2ISA_H

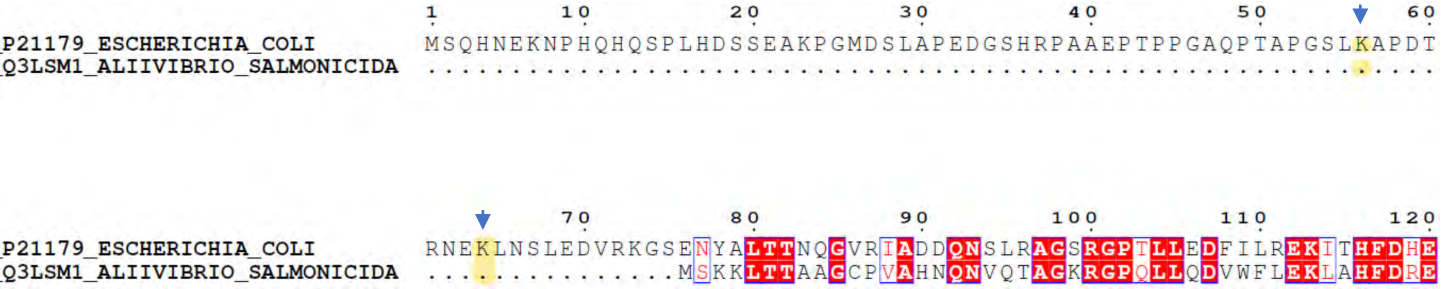

Full sequences in supplemental file.

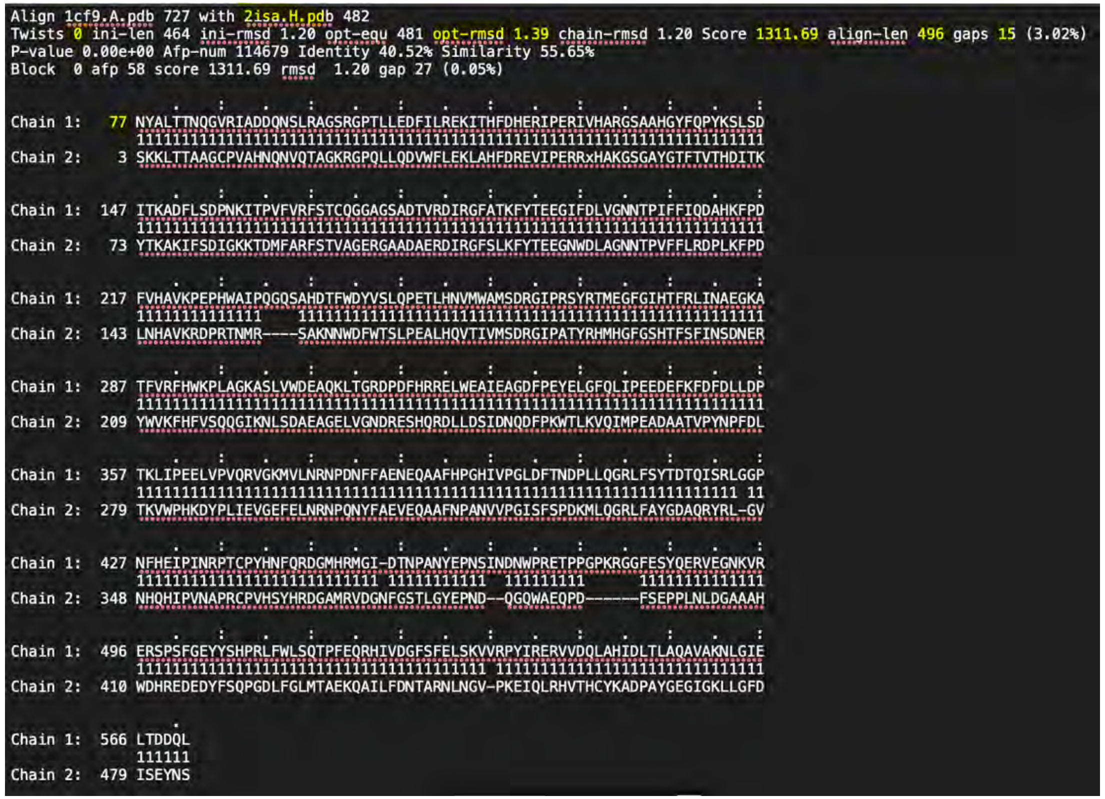

```
Align 1cf9.A.pdb 727 with 2isa.H.pdb 482
Twists 0 ini-len 464 ini-rmsd 1.20 opt-equ 481 opt-rmsd 1.39 chain-rmsd 1.20 Score 1311.69 align-len 496 gaps 15 (3.02%)
P-value 0.00e+00 Afp-num 114679 Identity 40.52% Similarity 55.65%
Block  0 afp 58 score 1311.69 rmsd  1.20 gap 27 (0.05%)

Chain 1:   77 NYALTTNQGVRIADDQNSLRAGSRGPTLLEDFILREKITHFDHERIPERIVHARGSAAHGYFQPYKSLSD
              1111111111111111111111111111111111111111111111111111111111111111111111
Chain 2:    3 SKKLTTAAGCPVAHNQNVQTAGKRGPQLLQDVWFLEKLAHFDREVIPERRxHAKGSGAYGTFTVTHDITK

Chain 1:  147 ITKADFLSDPNKITPVFVRFSTCQGGAGSADTVRDIRGFATKFYTEEGIFDLVGNNTPIFFIQDAHKFPD
              1111111111111111111111111111111111111111111111111111111111111111111111
Chain 2:   73 YTKAKIFSDIGKKTDMFARFSTVAGERGAADAERDIRGFSLKFYTEEGNWDLAGNNTPVFFLRDPLKFPD

Chain 1:  217 FVHAVKPEPHWAIPQGQSAHDTFWDYVSLQPETLHNVMWAMSDRGIPRSYRTMEGFGIHTFRLINAEGKA
              11111111111111    111111111111111111111111111111111111111111111111111
Chain 2:  143 LNHAVKRDPRTNMR----SAKNNWDFWTSLPEALHQVTIVMSDRGIPATYRHMHGFGSHTFSFINSDNER

Chain 1:  287 TFVRFHWKPLAGKASLVWDEAQKLTGRDPDFHRRELWEAIEAGDFPEYELGFQLIPEEDEFKFDFDLLDP
              1111111111111111111111111111111111111111111111111111111111111111111111
Chain 2:  209 YWVKFHFVSQQGIKNLSDAEAGELVGNDRESHQRDLLDSIDNQDFPKWTLKVQIMPEADAATVPYNPFDL

Chain 1:  357 TKLIPEELVPVQRVGKMVLNRNPDNFFAENEQAAFHPGHIVPGLDFTNDPLLQGRLFSYTDTQISRLGGP
              11111111111111111111111111111111111111111111111111111111111111111111 11
Chain 2:  279 TKVWPHKDYPLIEVGEFELNRNPQNYFAEVEQAAFNPANVVPGISFSPDKMLQGRLFAYGDAQRYRL-GV

Chain 1:  427 NFHEIPINRPTCPYHNFQRDGMHRMGI-DTNPANYEPNSINDNWPRETPPGPKRGGFESYQERVEGNKVR
              111111111111111111111111111 11111111111  111111111      11111111111111
Chain 2:  348 NHQHIPVNAPRCPVHSYHRDGAMRVDGNFGSTLGYEPND--QGQWAEQPD------FSEPPLNLDGAAAH

Chain 1:  496 ERSPSFGEYYSHPRLFWLSQTPFEQRHIVDGFSFELSKVVRPYIRERVVDQLAHIDLTLAQAVAKNLGIE
              11111111111111111111111111111111111111111 11111111111111111111111111111
Chain 2:  410 WDHREDEDYFSQPGDLFGLMTAEKQAILFDNTARNLNGV-PKEIQLRHVTHCYKADPAYGEGIGKLLGFD

Chain 1:  566 LTDDQL
              111111
Chain 2:  479 ISEYNS
```

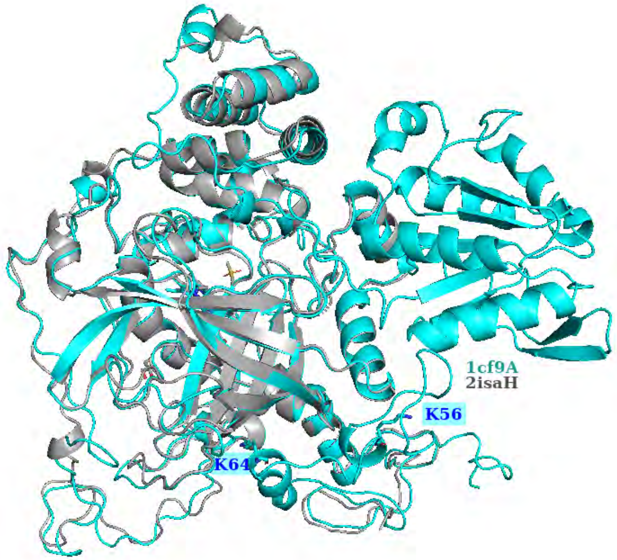

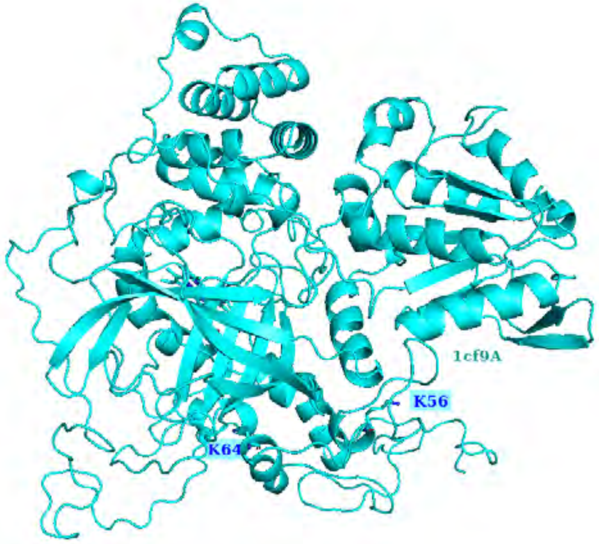

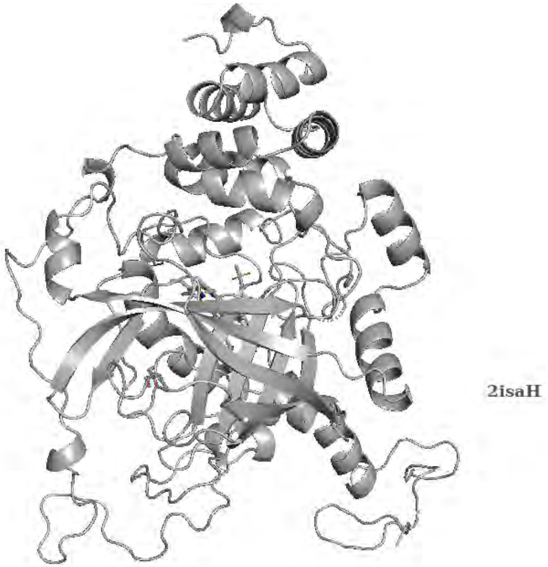

UniProt ID: Q59337

PDB ID: 4CAB_A

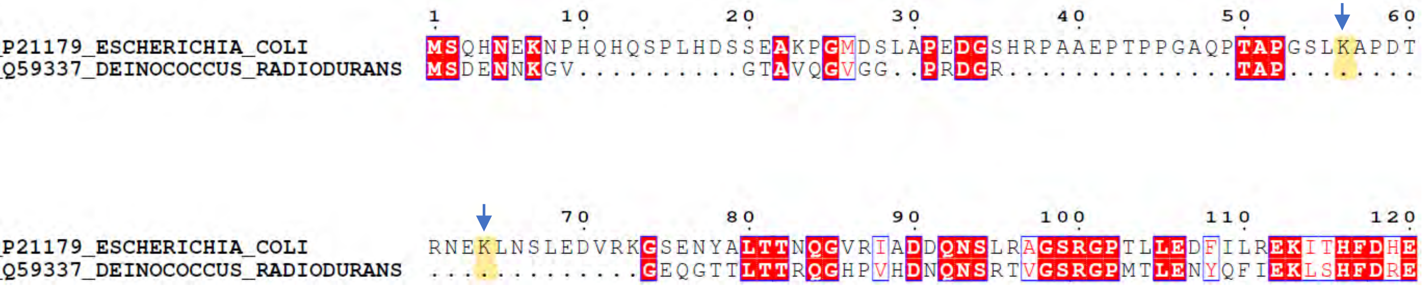

Full sequences in supplemental file.

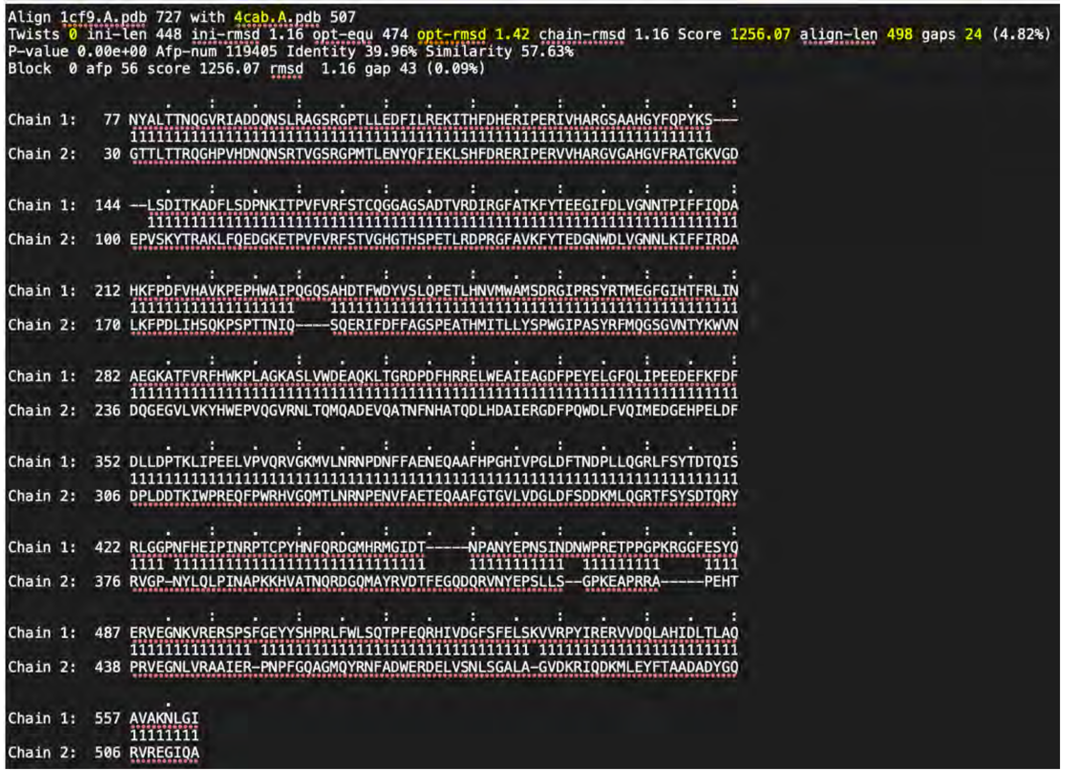

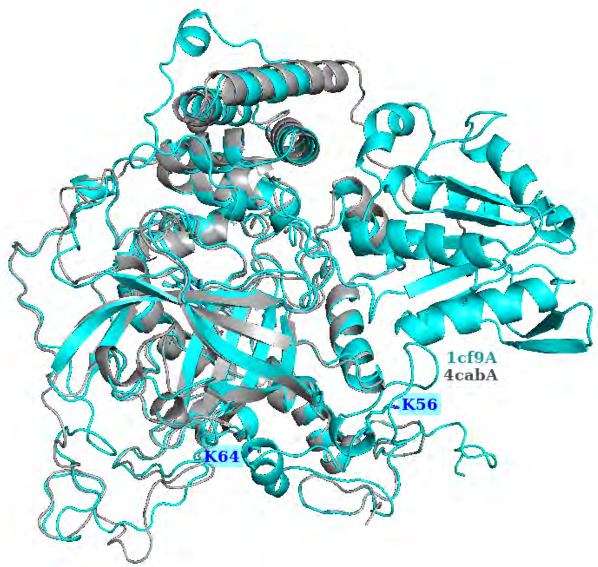

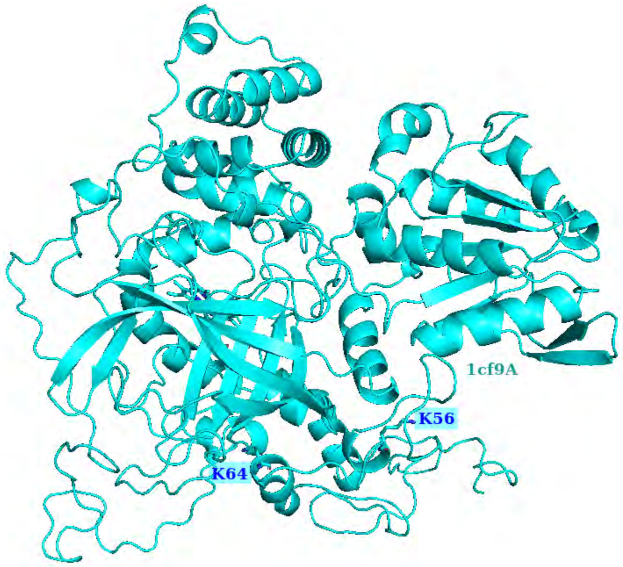

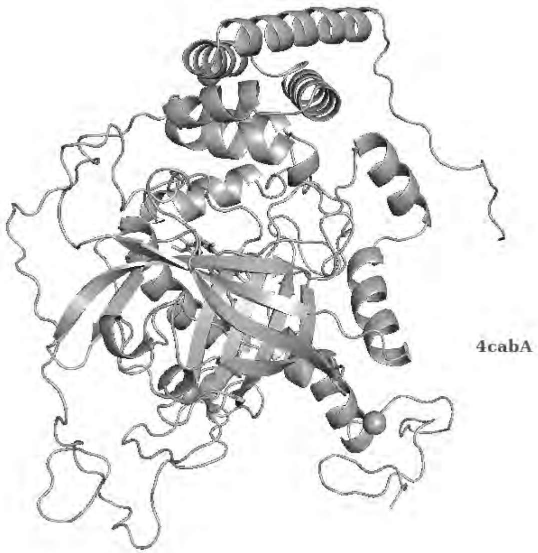

UniProt ID: Q6CR58
PDB ID: 6RJR_A

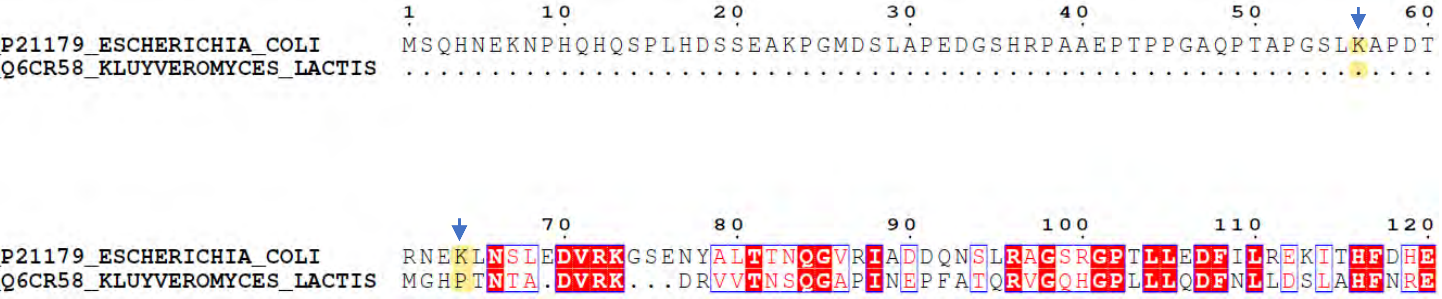

Full sequences in supplemental file.

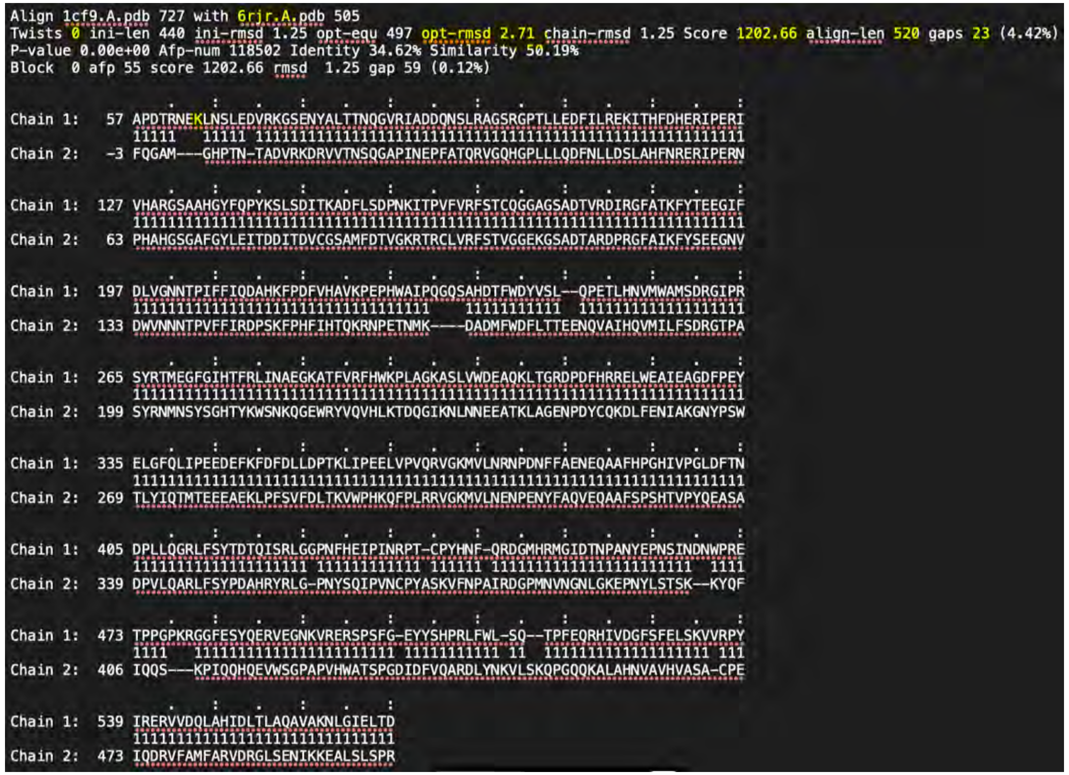

```
Align 1cf9.A.pdb 727 with 6rjr.A.pdb 505
Twists 0 ini-len 440 ini-rmsd 1.25 opt-equ 497 opt-rmsd 2.71 chain-rmsd 1.25 Score 1202.66 align-len 520 gaps 23 (4.42%)
P-value 0.00e+00 Afp-num 118502 Identity 34.62% Similarity 50.19%
Block  0 afp 55 score 1202.66 rmsd  1.25 gap 59 (0.12%)

Chain 1:   57 APDTRNEKLNSLEDVRKGSENYALTTNQGVRIADDQNSLRAGSRGPTLLEDFILREKITHFDHERIPERI
Chain 2:   -3 FQGAM---GHPTN-TADVRKDRVVTNSQGAPINEPFATQRVGQHGPLLLQDFNLLDSLAHFNRERIPERN

Chain 1:  127 VHARGSAAHGYFQPYKSLSDITKADFLSDPNKITPVFVRFSTCQGGAGSADTVRDIRGFATKFYTEEGIF
Chain 2:   63 PHAHGSGAFGYLEITDDITDVCGSAMFDTVGKRTRCLVRFSTVGGEKGSADTARDPRGFAIKFYSEEGNV

Chain 1:  197 DLVGNNTPIFFIQDAHKFPDFVHAVKPEPHWAIPQGQSAHDTFWDYVSL--QPETLHNVMWAMSDRGIPR
Chain 2:  133 DWVNNNTPVFFIRDPSKFPHFIHTQKRNPETNMK----DADMFWDFLTTEENQVAIHQVMILFSDRGTPA

Chain 1:  265 SYRTMEGFGIHTFRLINAEGKATFVRFHWKPLAGKASLVWDEAQKLTGRDPDFHRRELWEAIEAGDFPEY
Chain 2:  199 SYRNMNSYSGHTYKWSNKQGEWRYVQVHLKTDQGIKNLNNEEATKLAGENPDYCQKDLFENIAKGNYPSW

Chain 1:  335 ELGFQLIPEEDEFKFDFDLLDPTKLIPEELVPVQRVGKMVLNRNPDNFFAENEQAAFHPGHIVPGLDFTN
Chain 2:  269 TLYIQTMTEEEAEKLPFSVFDLTKVWPHKQFPLRRVGKMVLNENPENYFAQVEQAAFSPSHTVPYQEASA

Chain 1:  405 DPLLQGRLFSYTDTQISRLGGPNFHEIPINRPT-CPYHNF-QRDGMHRMGIDTNPANYEPNSINDNWPRE
Chain 2:  339 DPVLQARLFSYPDAHRYRLG-PNYSQIPVNCPYASKVFNPAIRDGPMNVNGNLGKEPNYLSTSK--KYQF

Chain 1:  473 TPPGPKRGGFESYQERVEGNKVRERSPSFG-EYYSHPRLFWL-SQ--TPFEQRHIVDGFSFELSKVVRPY
Chain 2:  406 IQQS---KPIQQHQEVWSGPAPVHWATSPGDIDFVQARDLYNKVLSKQPGQQKALAHNVAVHVASA-CPE

Chain 1:  539 IRERVVDQLAHIDLTLAQAVAKNLGIELTD
Chain 2:  473 IQDRVFAMFARVDRGLSENIKKEALSLSPR
```

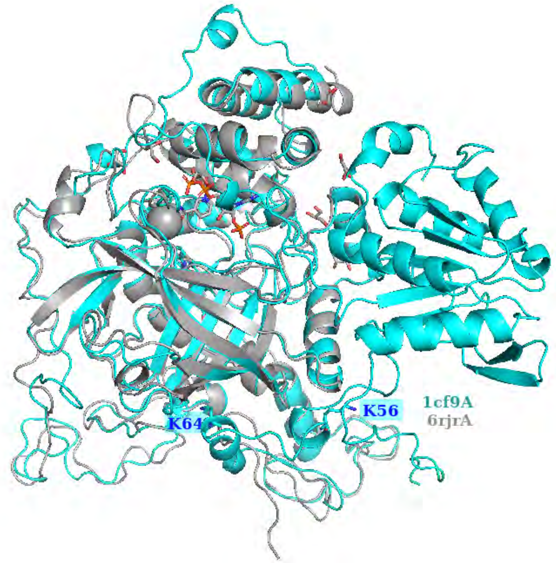

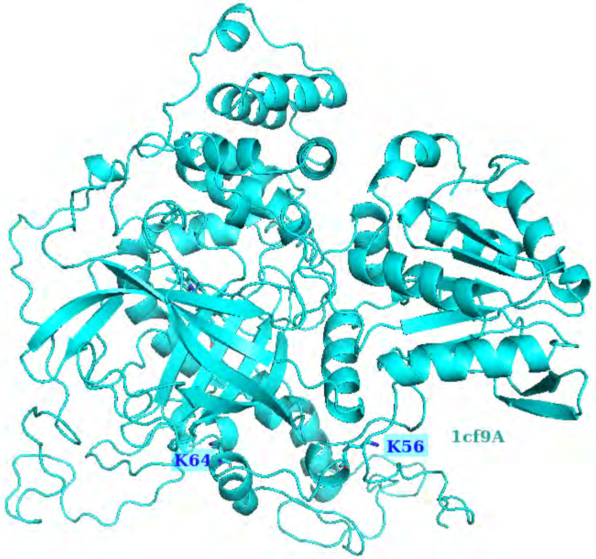

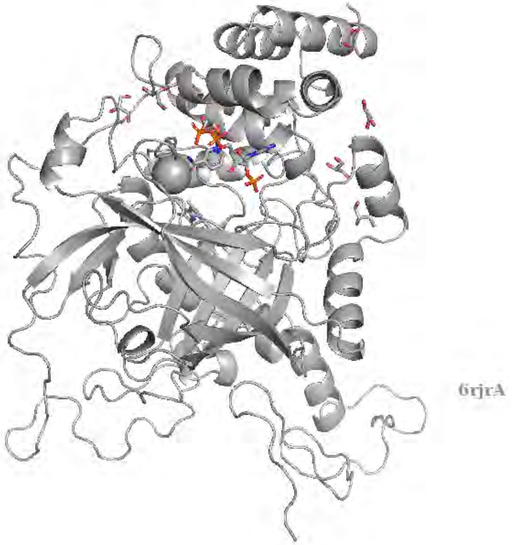

UniProt ID: Q834P5
PDB ID: 1SI8_C

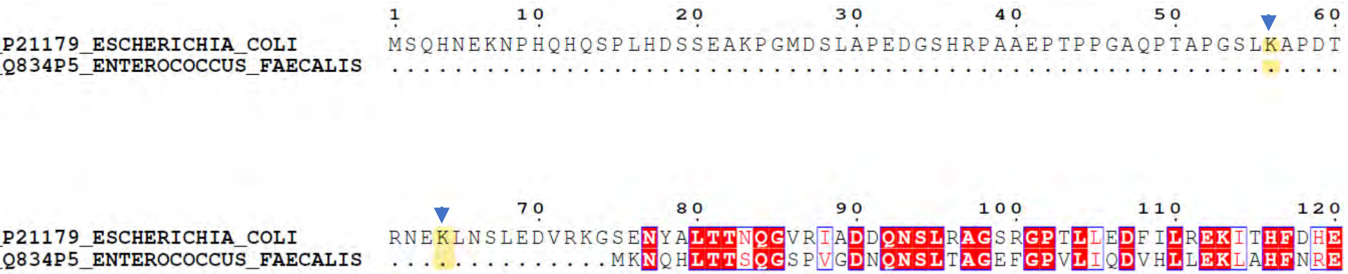

Full sequences in supplemental file.

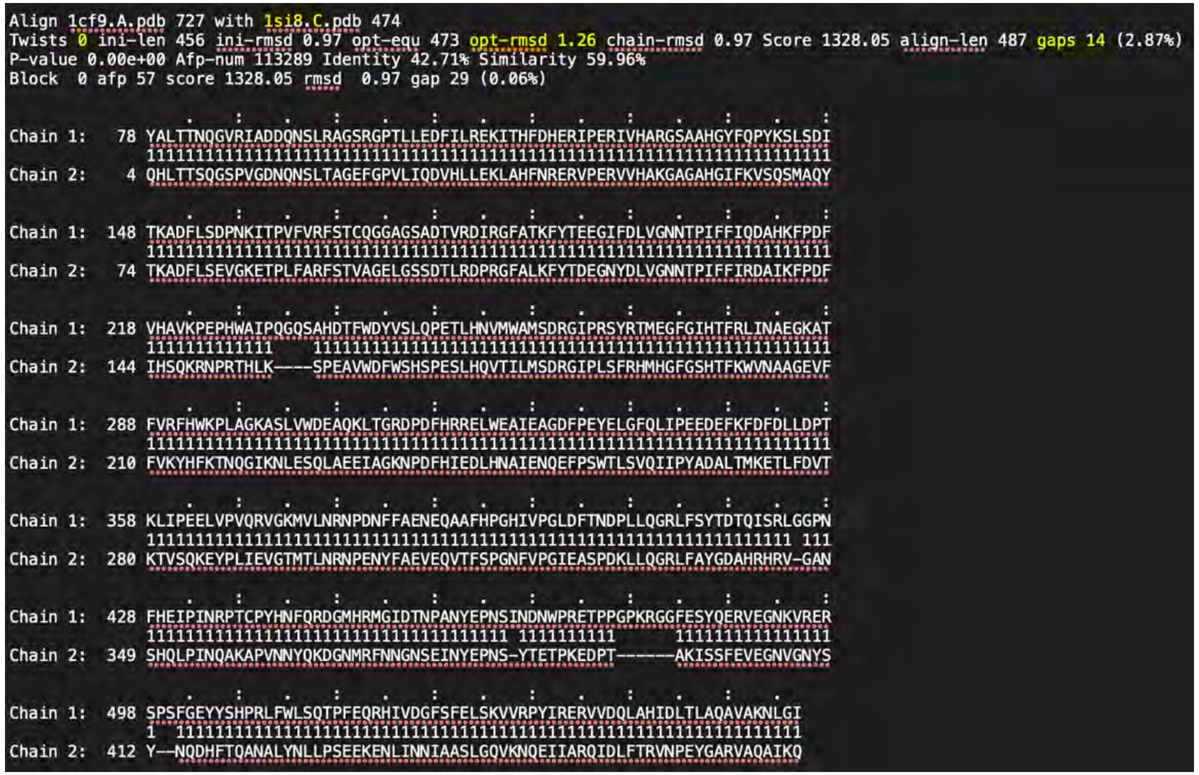

```
Align 1cf9.A.pdb 727 with 1si8.C.pdb 474
Twists 0 ini-len 456 ini-rmsd 0.97 opt-equ 473 opt-rmsd 1.26 chain-rmsd 0.97 Score 1328.05 align-len 487 gaps 14 (2.87%)
P-value 0.00e+00 Afp-num 113289 Identity 42.71% Similarity 59.96%
Block  0 afp 57 score 1328.05 rmsd  0.97 gap 29 (0.06%)

Chain 1:   78 YALTTNQGVRIADDQNSLRAGSRGPTLLEDFILREKITHFDHERIPERIVHARGSAAHGYFQPYKSLSDI
Chain 2:    4 QHLTTSQGSPVGDNQNSLTAGEFGPVLIQDVHLLEKLAHFNRERVPERVVHAKGAGAHGIFKVSQSMAQY

Chain 1:  148 TKADFLSDPNKITPVFVRFSTCQGGAGSADTVRDIRGFATKFYTEEGIFDLVGNNTPIFFIQDAHKFPDF
Chain 2:   74 TKADFLSEVGKETPLFARFSTVAGELGSSDTLRDPRGFALKFYTDEGNYDLVGNNTPIFFIRDAIKFPDF

Chain 1:  218 VHAVKPEPHWAIPQGQSAHDTFWDYVSLQPETLHNVMWAMSDRGIPRSYRTMEGFGIHTFRLINAEGKAT
Chain 2:  144 IHSQKRNPRTHLK----SPEAVWDFWSHSPESLHQVTILMSDRGIPLSFRHMHGFGSHTFKWVNAAGEVF

Chain 1:  288 FVRFHWKPLAGKASLVWDEAQKLTGRDPDFHRRELWEAIEAGDFPEYELGFQLIPEEDEFKFDFDLLDPT
Chain 2:  210 FVKYHFKTNQGIKNLESQLAEEIAGKNPDFHIEDLHNAIENQEFPSWTLSVQIIPYADALTMKETLFDVT

Chain 1:  358 KLIPEELVPVQRVGKMVLNRNPDNFFAENEQAAFHPGHIVPGLDFTNDPLLQGRLFSYTDTQISRLGGPN
Chain 2:  280 KTVSQKEYPLIEVGTMTLNRNPENYFAEVEQVTFSPGNFVPGIEASPDKLLQGRLFAYGDAHRHRV-GAN

Chain 1:  428 FHEIPINRPTCPYHNFQRDGMHRMGIDTNPANYEPNSINDNWPRETPPGPKRGGFESYQERVEGNKVRER
Chain 2:  349 SHQLPINQAKAPVNNYQKDGNMRFNNGNSEINYEPNS-YTETPKEDPT------AKISSFEVEGNVGNYS

Chain 1:  498 SPSFGEYYSHPRLFWLSQTPFEQRHIVDGFSFELSKVVRPYIRERVVDQLAHIDLTLAQAVAKNLGI
Chain 2:  412 Y--NQDHFTQANALYNLLPSEEKENLINNIAASLGQVKNQEIIARQIDLFTRVNPEYGARVAQAIKQ
```

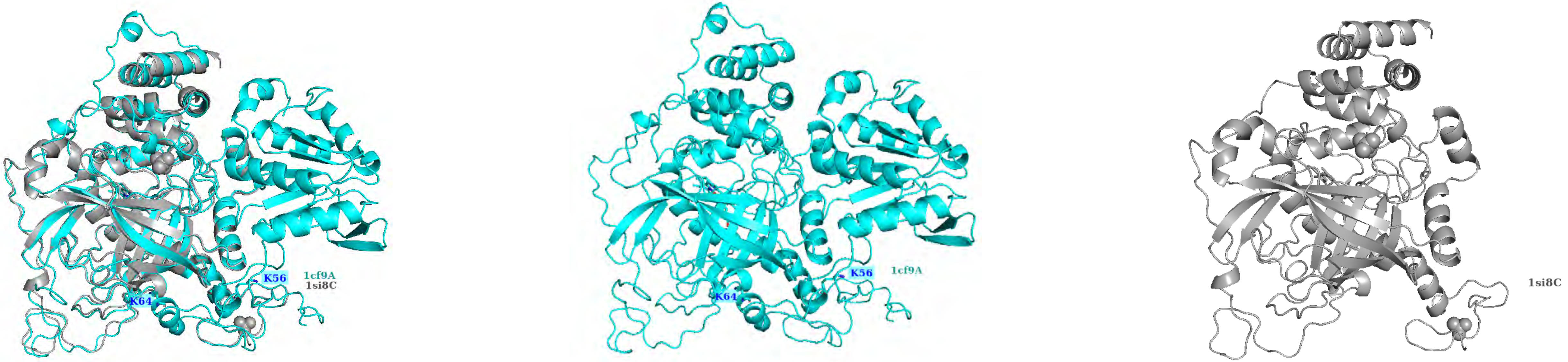

UniProt ID: Q9C168
PDB ID: 1SY7_B

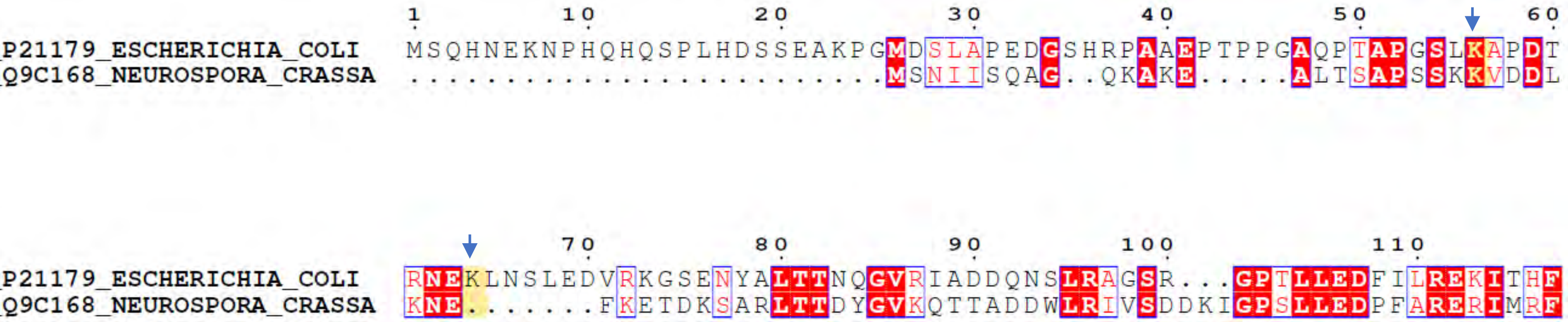

Full sequences in supplemental file.

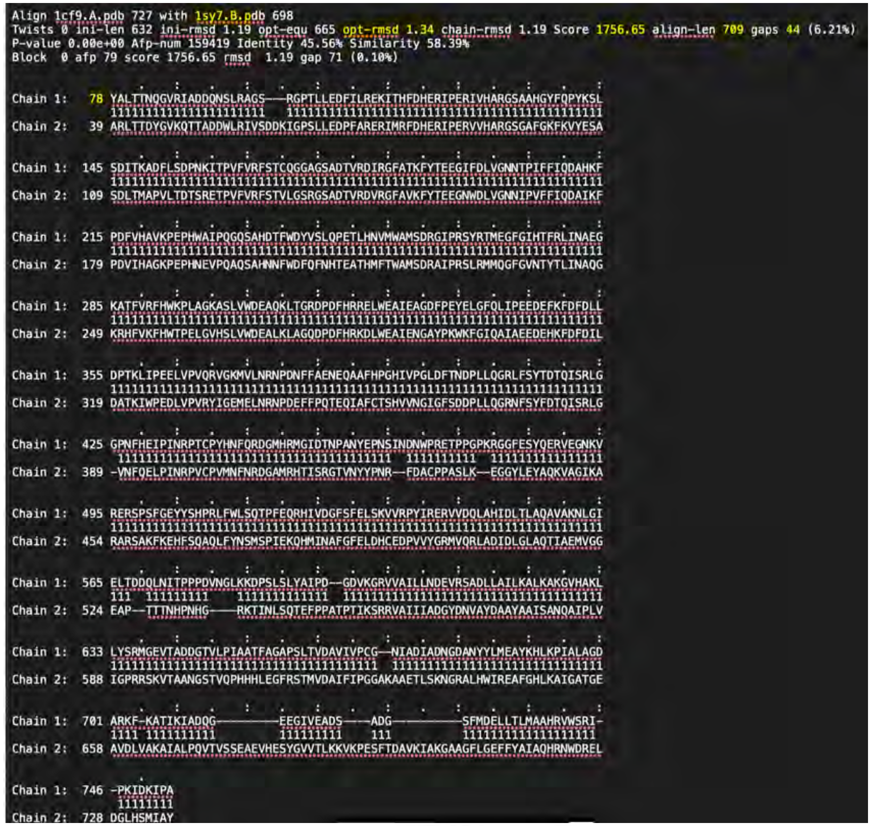

```
Align 1cf9.A.pdb 727 with 1sy7.B.pdb 698
Twists 0 ini-len 632 ini-rmsd 1.19 opt-equ 665 opt-rmsd 1.34 chain-rmsd 1.19 Score 1756.65 align-len 709 gaps 44 (6.21%)
P-value 0.00e+00 Afp-num 159419 Identity 45.56% Similarity 58.39%
Block  0 afp 79 score 1756.65 rmsd  1.19 gap 71 (0.10%)

Chain 1:   78 YALTTNQGVRIADDQNSLRAGS---RGPTLLEDFILREKITHFDHERIPERIVHARGSAAHGYFQPYKSL
              1111111111111111111111   111111111111111111111111111111111111111111111
Chain 2:   39 ARLTTDYGVKQTTADDWLRIVSDDKIGPSLLEDPFARERIMRFDHERIPERVVHARGSGAFGKFKVYESA

Chain 1:  145 SDITKADFLSDPNKITPVFVRFSTCQGGAGSADTVRDIRGFATKFYTEEGIFDLVGNNTPIFFIQDAHKF
              1111111111111111111111111111111111111111111111111111111111111111111111
Chain 2:  109 SDLTMAPVLTDTSRETPVFVRFSTVLGSRGSADTVRDVRGFAVKFYTEEGNWDLVGNNIPVFFIQDAIKF

Chain 1:  215 PDFVHAVKPEPHWAIPQGQSAHDTFWDYVSLQPETLHNVMWAMSDRGIPRSYRTMEGFGIHTFRLINAEG
              1111111111111111111111111111111111111111111111111111111111111111111111
Chain 2:  179 PDVIHAGKPEPHNEVPQAQSAHNNFWDFQFNHTEATHMFTWAMSDRAIPRSLRMMQGFGVNTYTLINAQG

Chain 1:  285 KATFVRFHWKPLAGKASLVWDEAQKLTGRDPDFHRRELWEAIEAGDFPEYELGFQLIPEEDEFKFDFDLL
              1111111111111111111111111111111111111111111111111111111111111111111111
Chain 2:  249 KRHFVKFHWTPELGVHSLVWDEALKLAGQDPDFHRKDLWEAIENGAYPKWKFGIQAIAEEDEHKFDFDIL

Chain 1:  355 DPTKLIPEELVPVQRVGKMVLNRNPDNFFAENEQAAFHPGHIVPGLDFTNDPLLQGRLFSYTDTQISRLG
              1111111111111111111111111111111111111111111111111111111111111111111111
Chain 2:  319 DATKIWPEDLVPVRYIGEMELNRNPDEFFPQTEQIAFCTSHVVNGIGFSDDPLLQGRNFSYFDTQISRLG

Chain 1:  425 GPNFHEIPINRPTCPYHNFQRDGMHRMGIDTNPANYEPNSINDNWPRETPPGPKRGGFESYQERVEGNKV
               11111111111111111111111111111111111111  1111111111  1111111111111111
Chain 2:  389 -VNFQELPINRPVCPVMNFNRDGAMRHTISRGTVNYYPNR--FDACPPASLK--EGGYLEYAQKVAGIKA

Chain 1:  495 RERSPSFGEYYSHPRLFWLSQTPFEQRHIVDGFSFELSKVVRPYIERVVDQLAHIDLTLAQAVAKNLGI
              1111111111111111111111111111111111111111111111111111111111111111111111
Chain 2:  454 RARSAKFKEHFSQAQLFYNSMSPIEKQHMINAFGFELDHCEDPVVYGRMVQRLADIDLGLAQTIAEMVGG

Chain 1:  565 ELTDDQLNITPPPDVNGLKKDPSLSLYAIPD--GDVKGRVVAILLNDEVRSADLLAILKALKAKGVHAKL
              111  111111111    11111111111111  111111111111111111111111111111111111
Chain 2:  524 EAP--TTTNHPNHG----RKTINLSQTEFPPATPTIKSRRVAIIIADGYDNVAYDAAYAAISANQAIPLV

Chain 1:  633 LYSRMGEVTADDGTVLPIAATFAGAPSLTVDAVIVPCG--NIADIADNGDANYYLMEAYKHLKPIALAGD
              11111111111111111111111111111111111111  111111111111111111111111111111
Chain 2:  588 IGPRRSKVTAANGSTVQPHHHLEGFRSTMVDAIFIPGGAKAAETLSKNGRALHWIREAFGHLKAIGATGE

Chain 1:  701 ARKF-KATIKIADQG----------EEGIVEADS----ADG-----------SFMDELLTLMAAHRVWSRI-
              1111 1111111111          111111111     111           1111111111111111111
Chain 2:  658 AVDLVAKAIALPQVTVSSEAEVHESYGVVTLKKVKPESFTDAVKIAKGAAGFLGEFFYAIAQHRNWDREL

Chain 1:  746 -PKIDKIPA
               11111111
Chain 2:  728 DGLHSMIAY
```

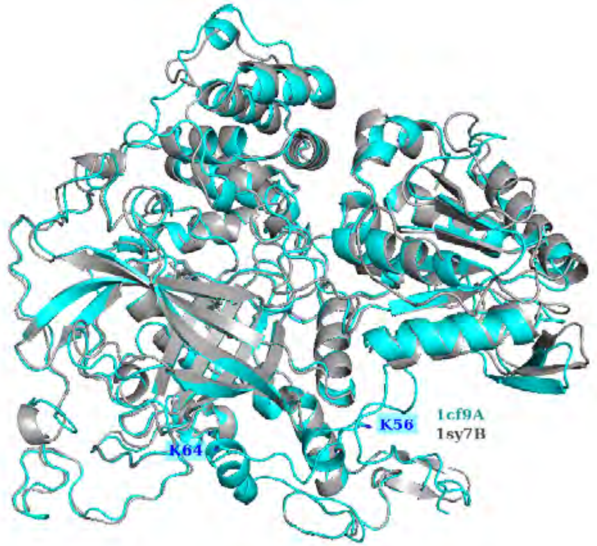

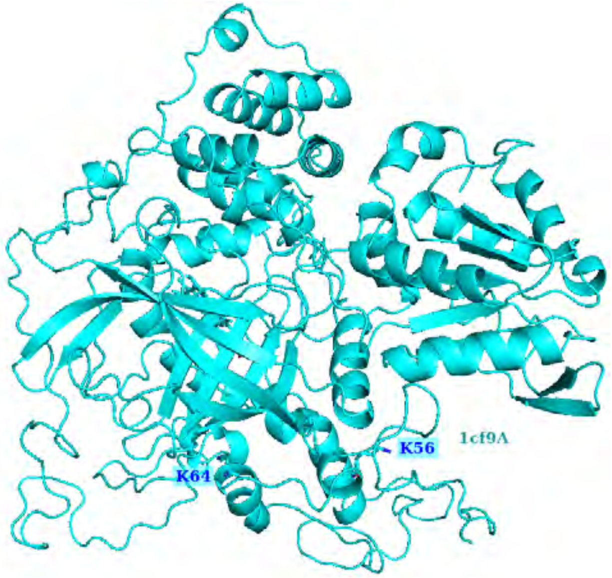

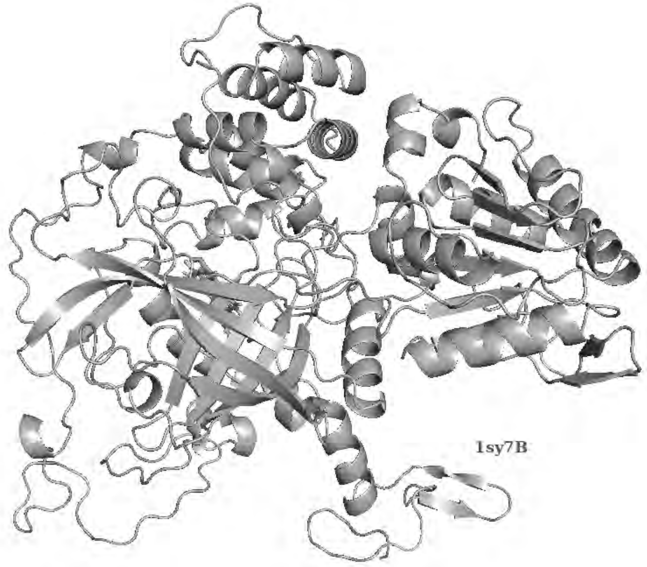

UniProt ID: Q9C169

PDB ID: 3EJ6_A

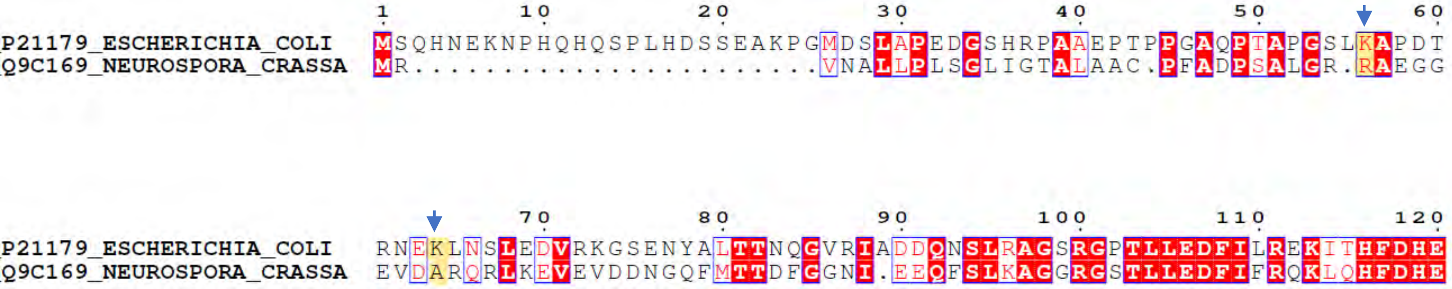

Full sequences in supplemental file.

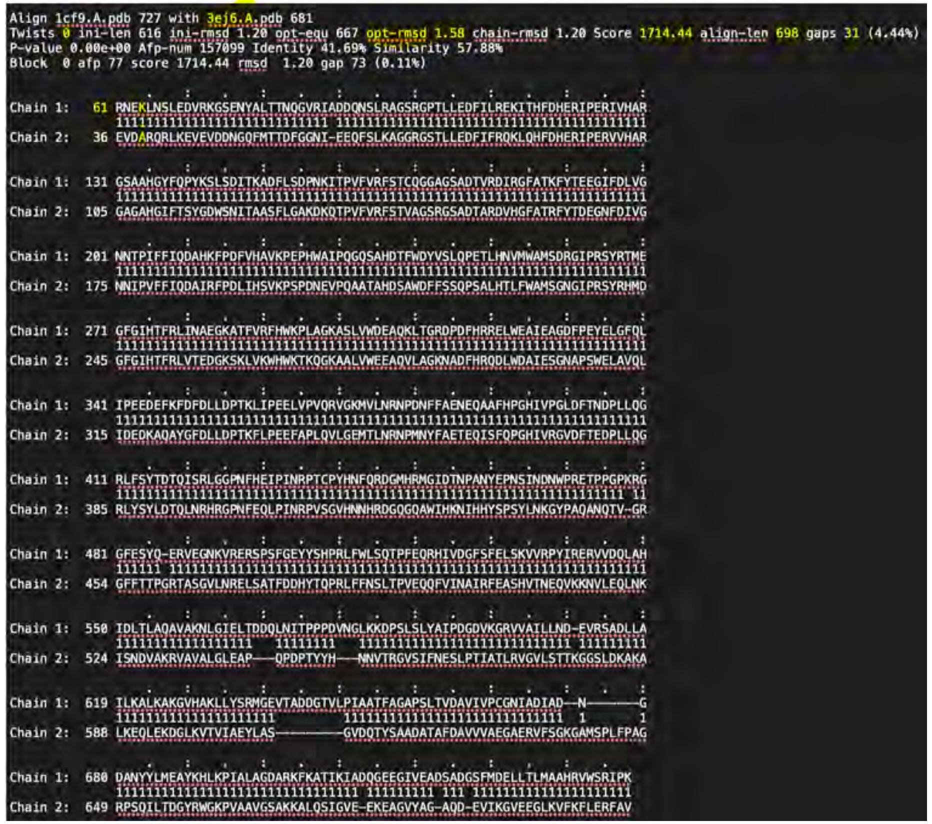

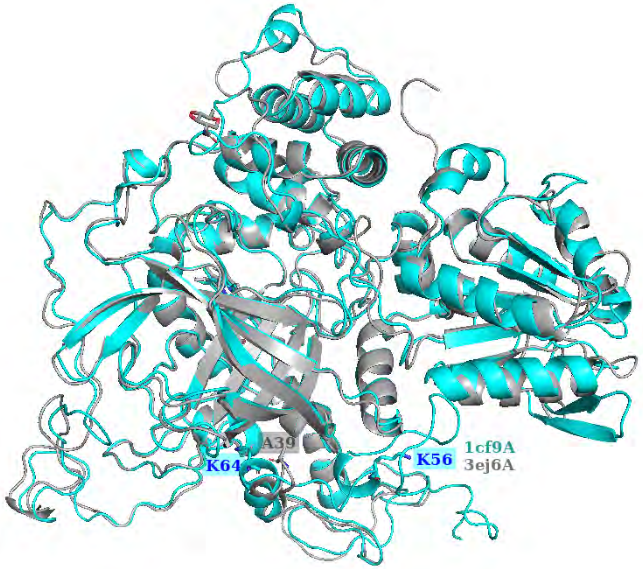

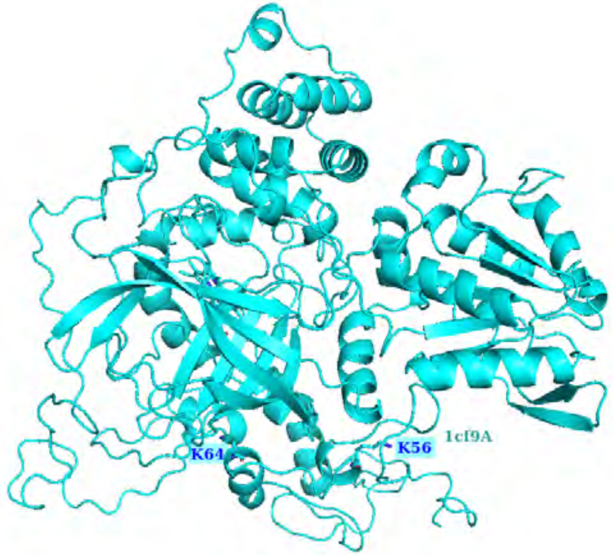

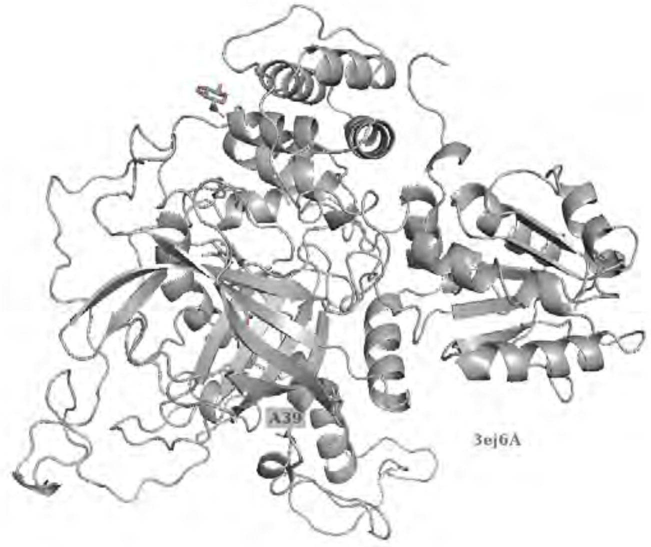

UniProt ID: Q9C169

PDB ID: 3ZJ4_D

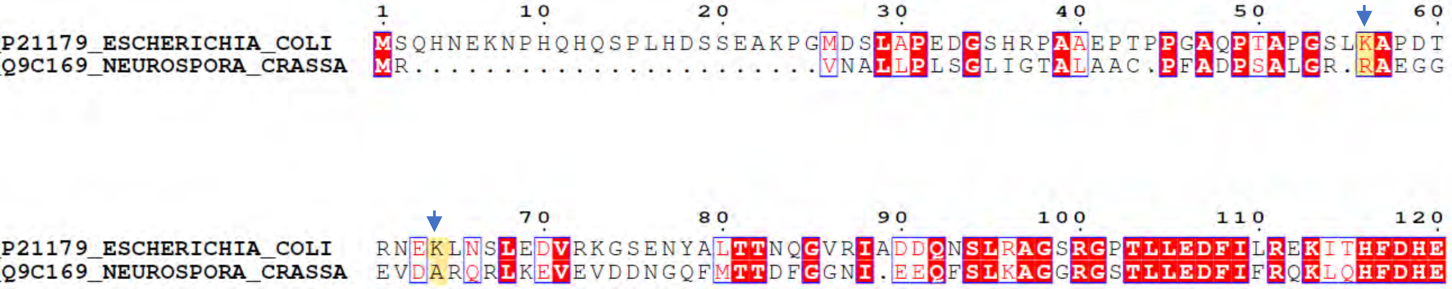

Full sequences in supplemental file.

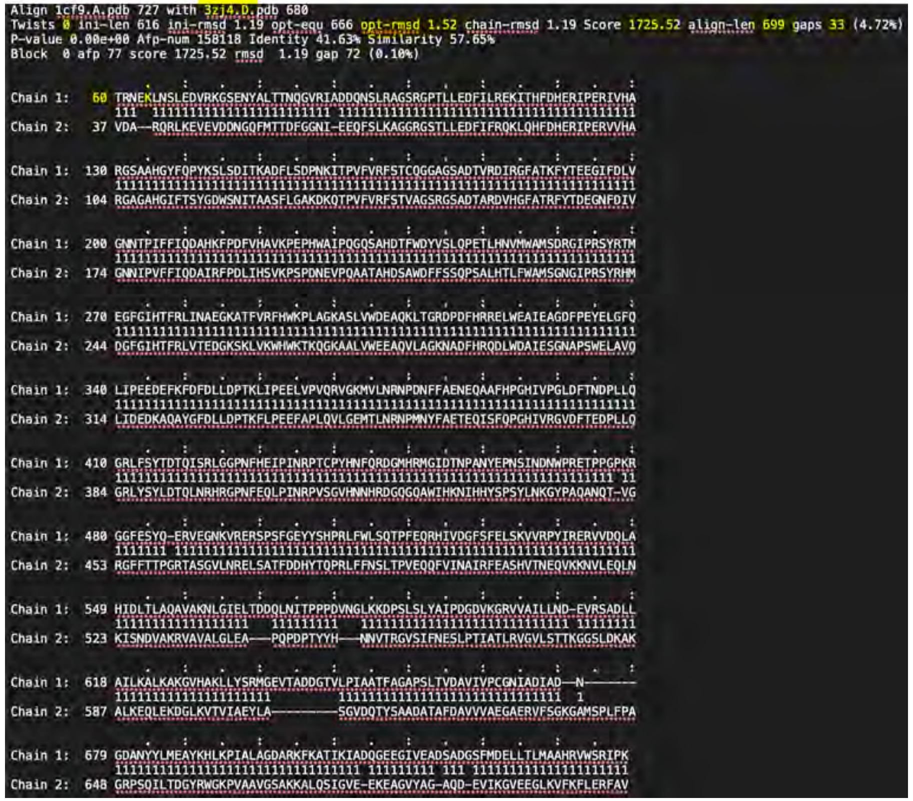

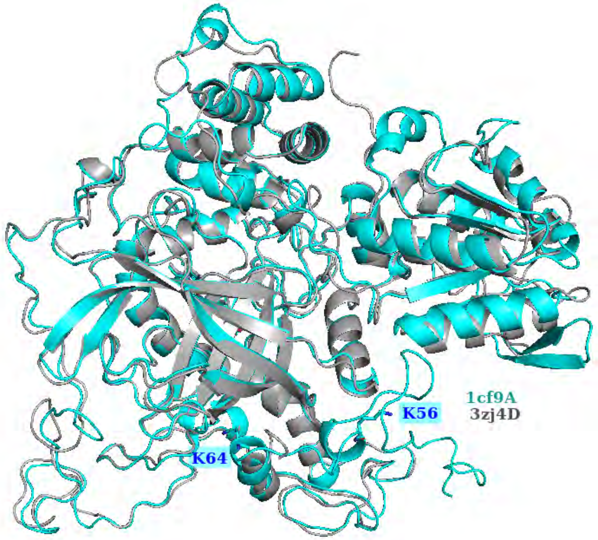

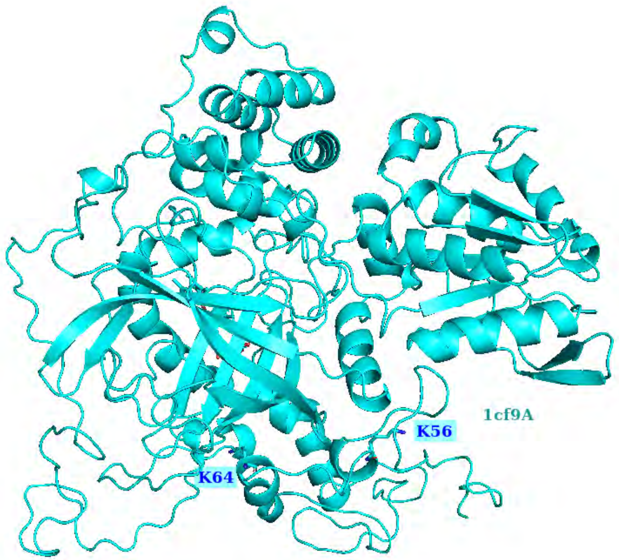

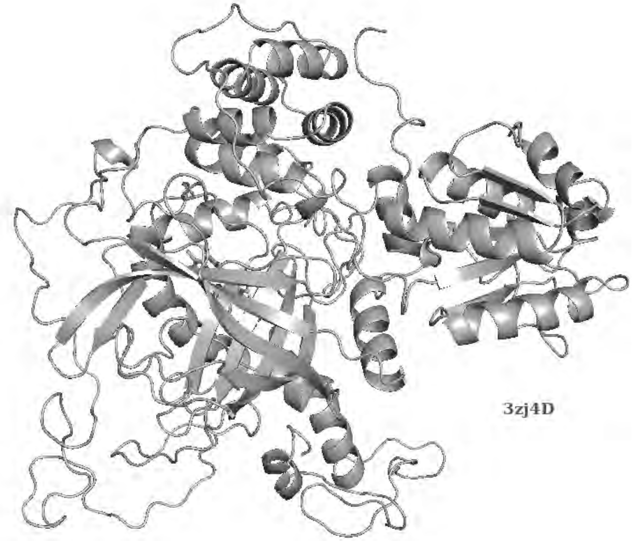

UniProt ID: Q9C169

PDB ID: 3ZJ5_D

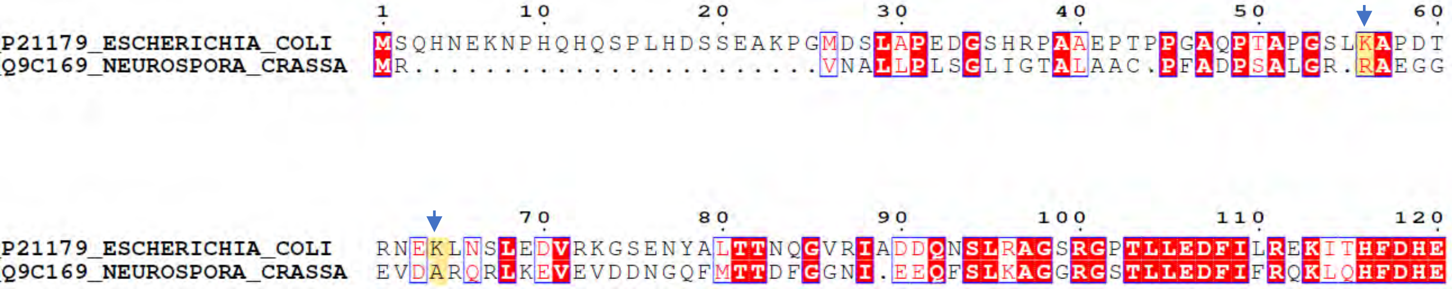

Full sequences in supplemental file.

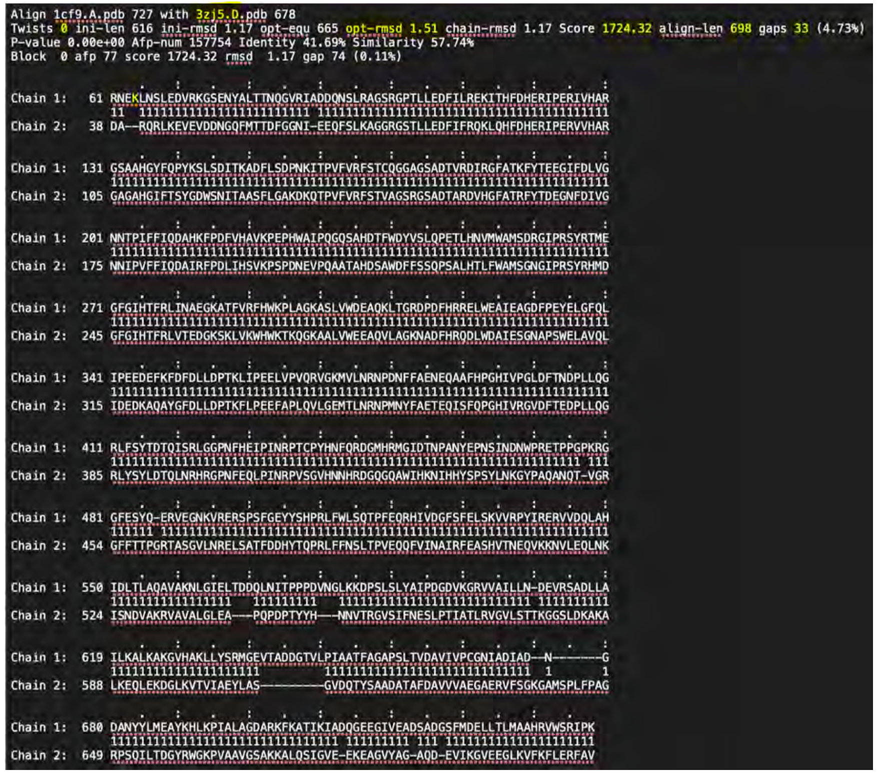
```
Align 1cf9.A.pdb 727 with 3zj5.D.pdb 678
Twists 0 ini-len 616 ini-rmsd 1.17 opt-equ 665 opt-rmsd 1.51 chain-rmsd 1.17 Score 1724.32 align-len 698 gaps 33 (4.73%)
P-value 0.00e+00 Afp-num 157754 Identity 41.69% Similarity 57.74%
Block  0 afp 77 score 1724.32 rmsd  1.17 gap 74 (0.11%)

Chain 1:   61 RNEKLNSLEDVRKGSENYALTTNQGVRIADDQNSLRAGSRGPTLLEDFILREKITHFDHERIPERIVHAR
Chain 2:   38 DA--RQRLKEVEVDDNGQFMTTDFGGNI-EEQFSLKAGGRGSTLLEDFIFRQKLQHFDHERIPERVVHAR

Chain 1:  131 GSAAHGYFQPYKSLSDITKADFLSDPNKITPVFVRFSTCQGGAGSADTVRDIRGFATKFYTEEGIFDLVG
Chain 2:  105 GAGAHGIFTSYGDWSNITAASFLGAKDKQTPVFVRFSTVAGSRGSADTARDVHGFATRFYTDEGNFDIVG

Chain 1:  201 NNTPIFFIQDAHKFPDFVHAVKPEPHWAIPQGQSAHDTFWDYVSLQPETLHNVMWAMSDRGIPRSYRTME
Chain 2:  175 NNIPVFFIQDAIRFPDLIHSVKPSPDNEVPQAATAHDSAWDFFSSQPSALHTLFWAMSGNGIPRSYRHMD

Chain 1:  271 GFGIHTFRLINAEGKATFVRFHWKPLAGKASLVWDEAQKLTGRDPDFHRRELWEAIEAGDFPEYELGFQL
Chain 2:  245 GFGIHTFRLVTEDGKSKLVKWHWKTKQGKAALVWEEAQVLAGKNADFHRQDLWDAIESGNAPSWELAVQL

Chain 1:  341 IPEEDEFKFDFDLLDPTKLIPEELVPVQRVGKMVLNRNPDNFFAENEQAAFHPGHIVPGLDFTNDPLLQG
Chain 2:  315 IDEDKAQAYGFDLLDPTKFLPEEFAPLQVLGEMTLNRNPMNYFAETEQISFQPGHIVRGVDFTEDPLLQG

Chain 1:  411 RLFSYTDTQISRLGGPNFHEIPINRPTCPYHNFQRDGMHRMGIDTNPANYEPNSINDNWPRETPPGPKRG
Chain 2:  385 RLYSYLDTQLNRHRGPNFEQLPINRPVSGVHNNHRDGQGQAWIHKNIHHYSPSYLNKGYPAQANQT-VGR

Chain 1:  481 GFESYQ-ERVEGNKVRERSPSFGEYYSHPRLFWLSQTPFEQRHIVDGFSFELSKVVRPYIRERVVDQLAH
Chain 2:  454 GFFTTPGRTASGVLNRELSATFDDHYTQPRLFFNSLTPVEQQFVINAIRFEASHVTNEQVKKNVLEQLNK

Chain 1:  550 IDLTLAQAVAKNLGIELTDDQLNITPPPDVNGLKKDPSLSLYAIPDGDVKGRVVAILLN-DEVRSADLLA
Chain 2:  524 ISNDVAKRVAVALGLEA---PQPDPTYYH---NNVTRGVSIFNESLPTIATLRVGVLSTTKGGSLDKAKA

Chain 1:  619 ILKALKAKGVHAKLLYSRMGEVTADDGTVLPIAATFAGAPSLTVDAVIVPCGNIADIAD--N-------G
Chain 2:  588 LKEQLEKDGLKVTVIAEYLAS---------GVDQTYSAADATAFDAVVVAEGAERVFSGKGAMSPLFPAG

Chain 1:  680 DANYYLMEAYKHLKPIALAGDARKFKATIKIADQGEEGIVEADSADGSFMDELLTLMAAHRVWSRIPK
Chain 2:  649 RPSQILTDGYRWGKPVAAVGSAKKALQSIGVE-EKEAGVYAG-AQD-EVIKGVEEGLKVFKFLERFAV
```

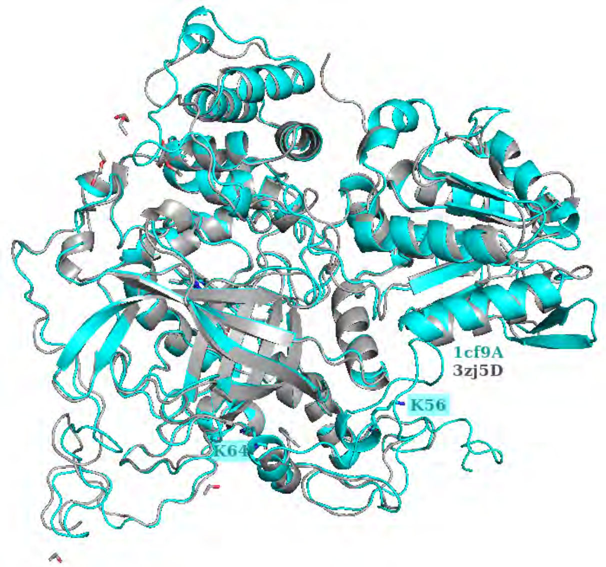

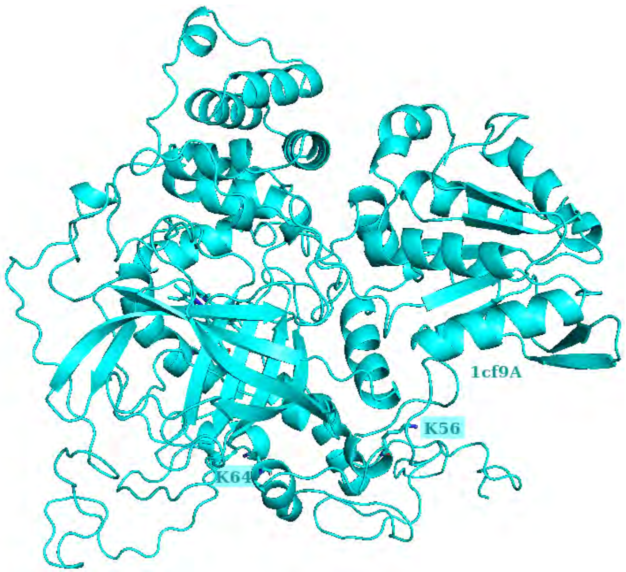

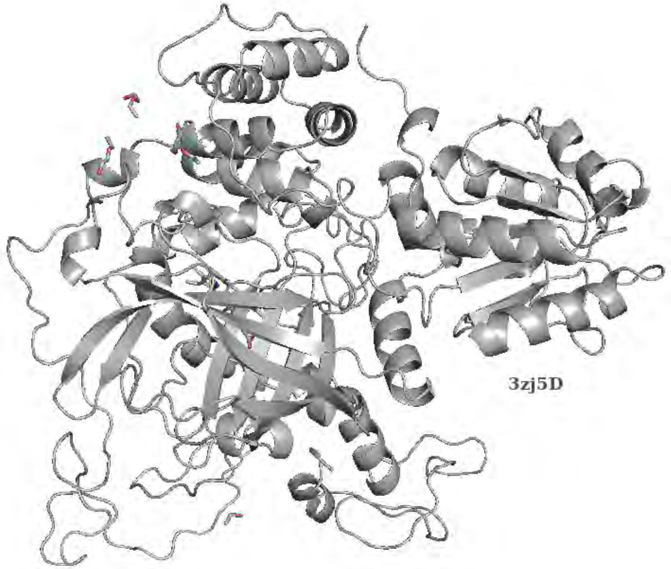

UniProt ID: Q9C169

PDB ID: 4AJ9_B

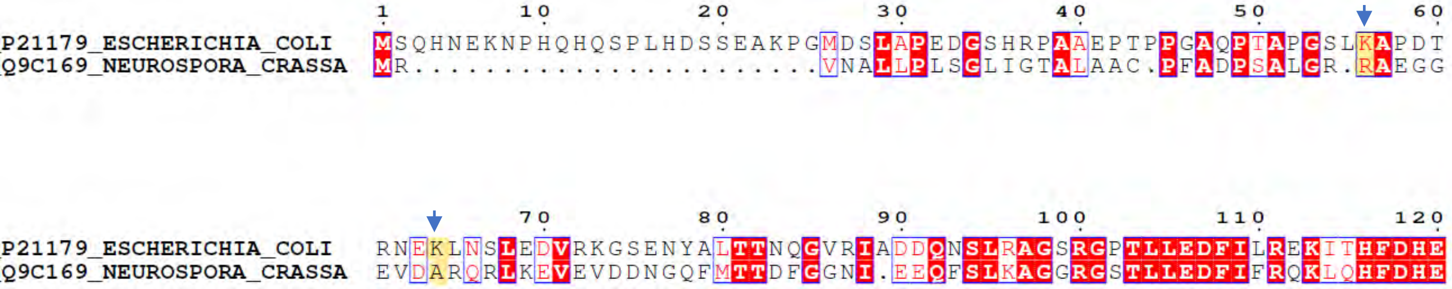

Full sequences in supplemental file.

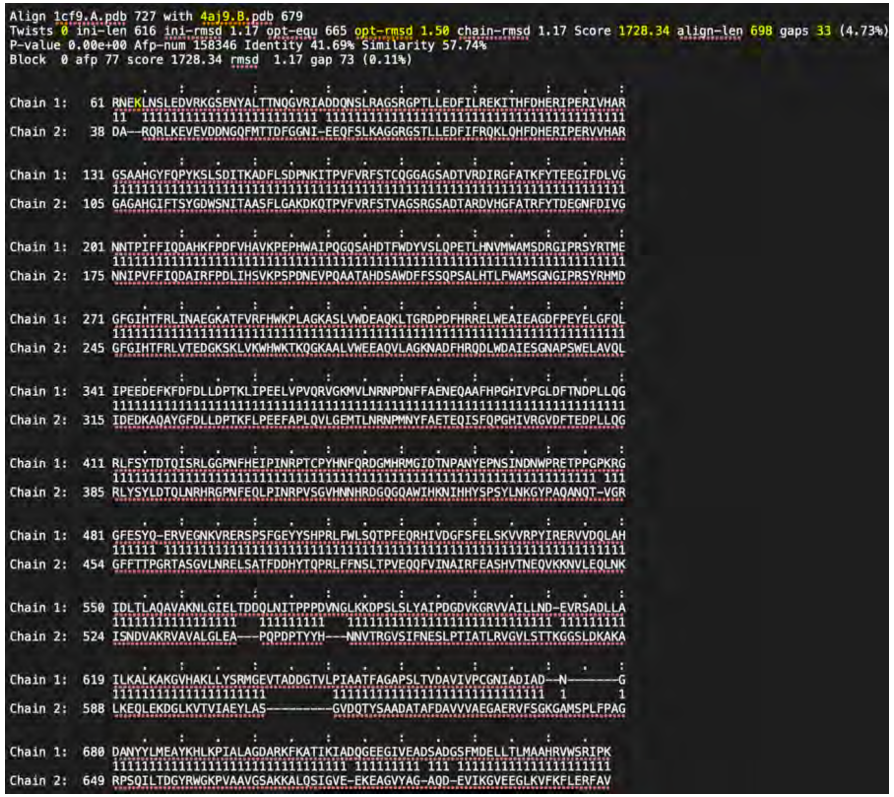

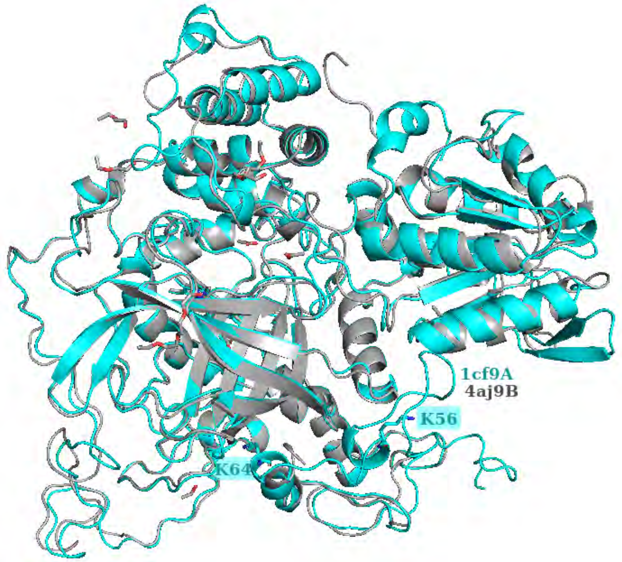

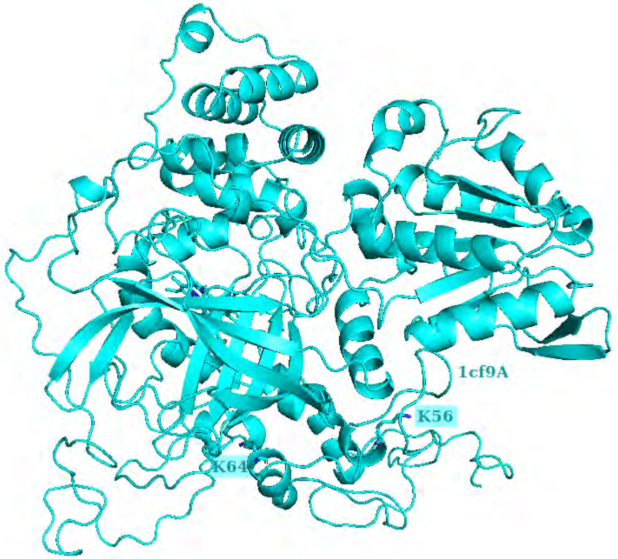

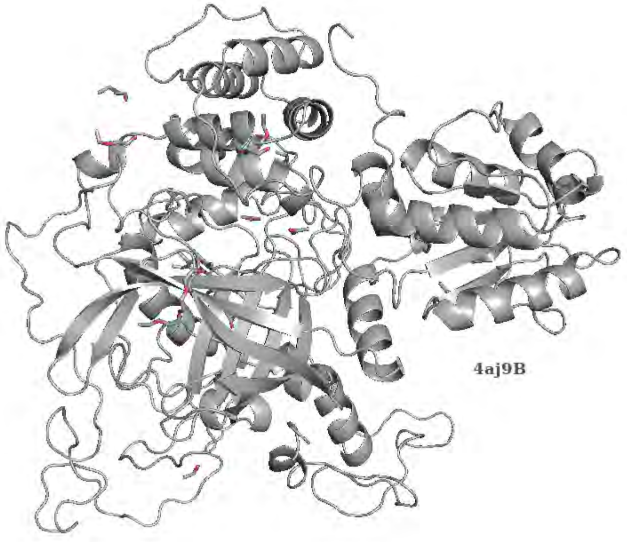

UniProt ID: Q9C169

PDB ID: 4BIM_A

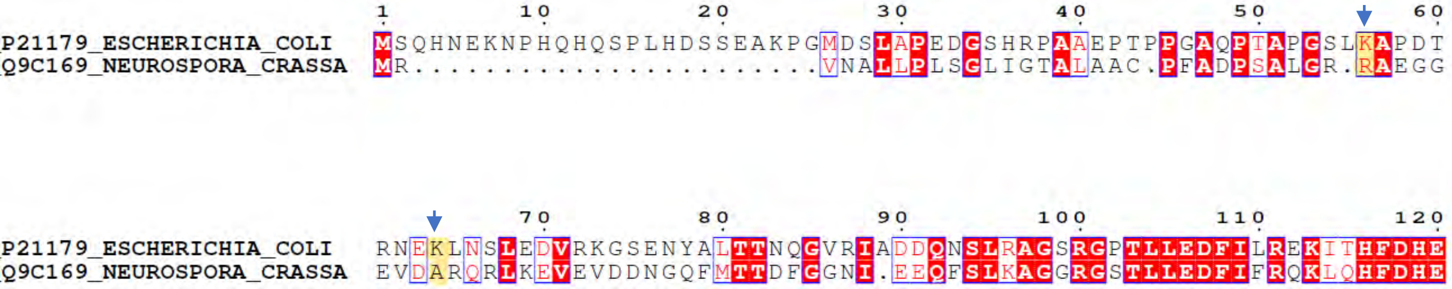

Full sequences in supplemental file.

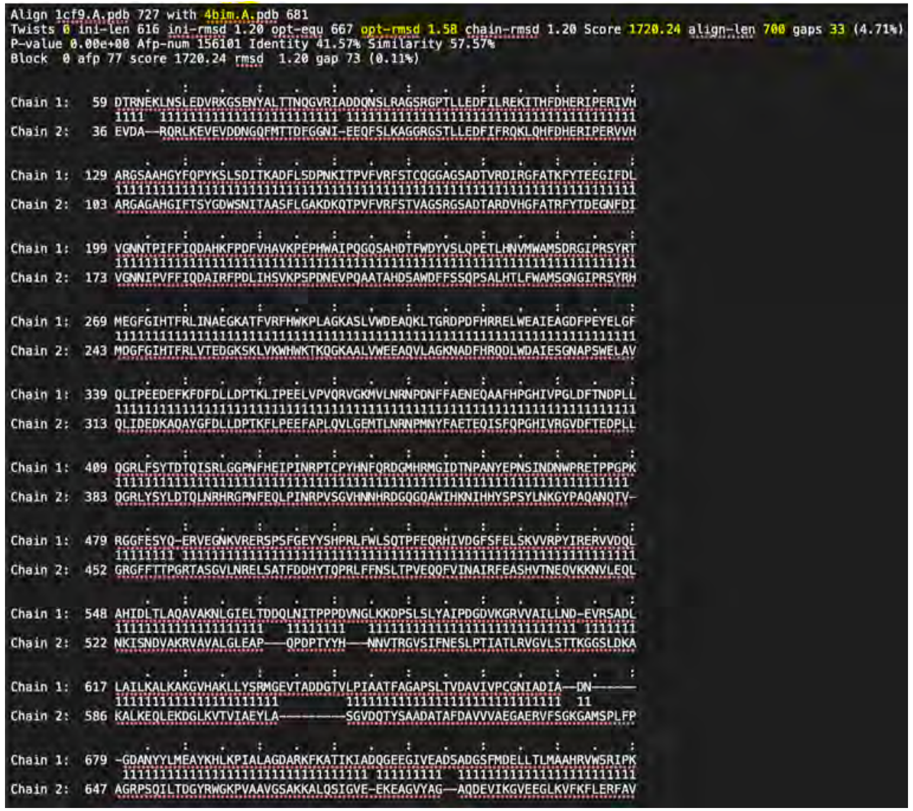

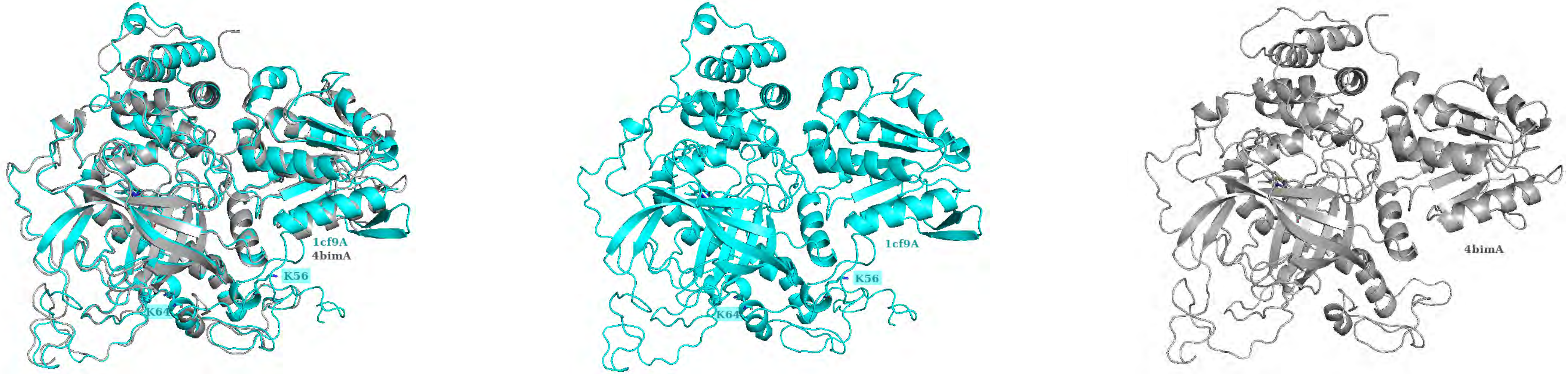

UniProt ID: Q9C169
PDB ID: 6NSW_C

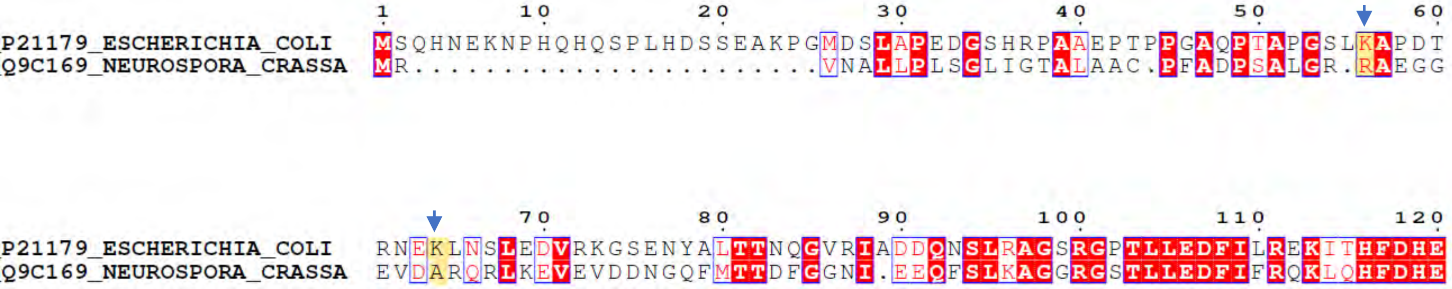

Full sequences in supplemental file.

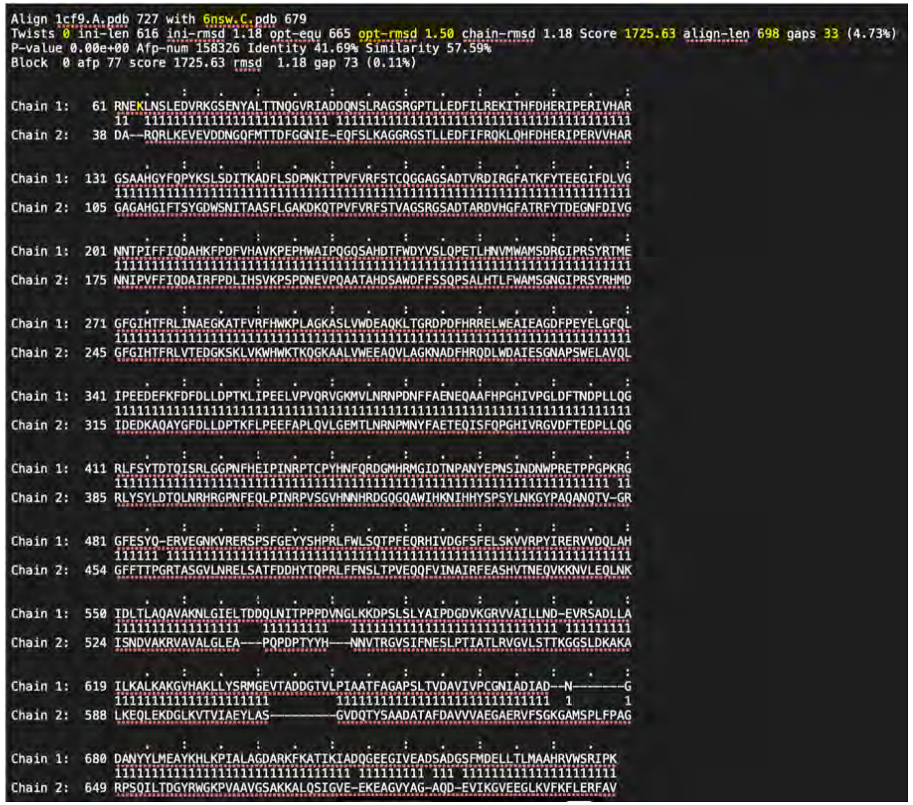

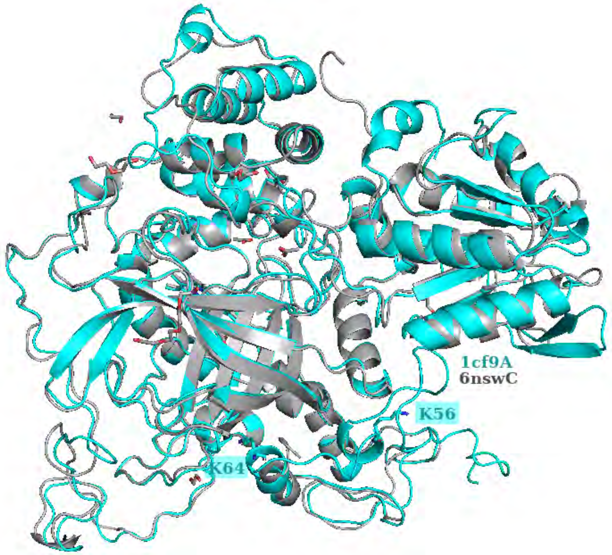

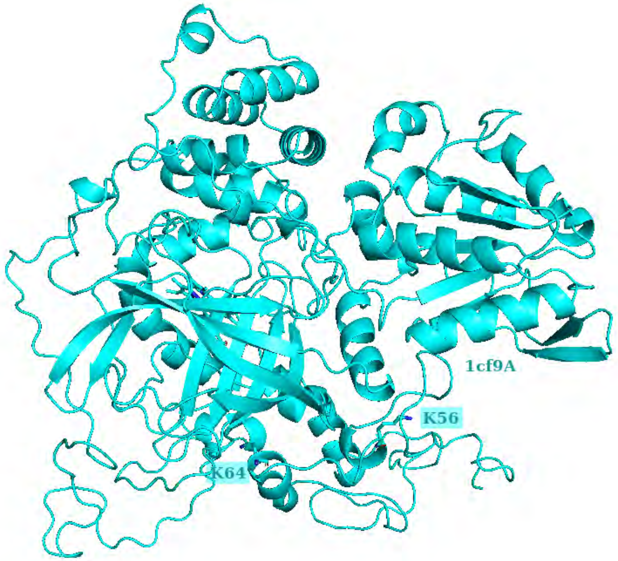

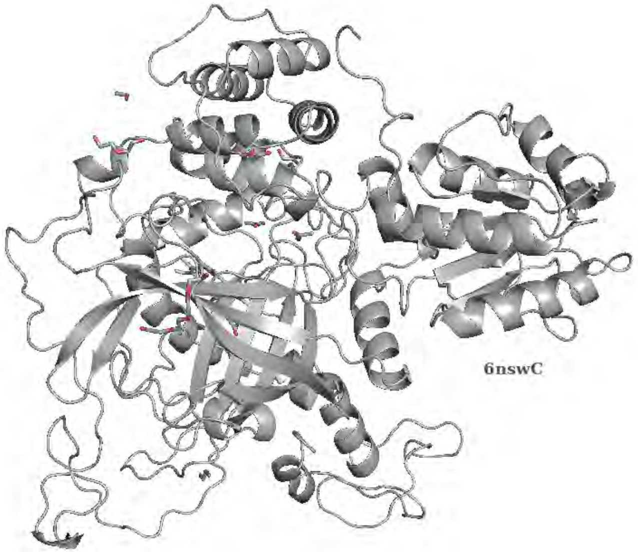

UniProt ID: Q9C169
PDB ID: 6NSY_C

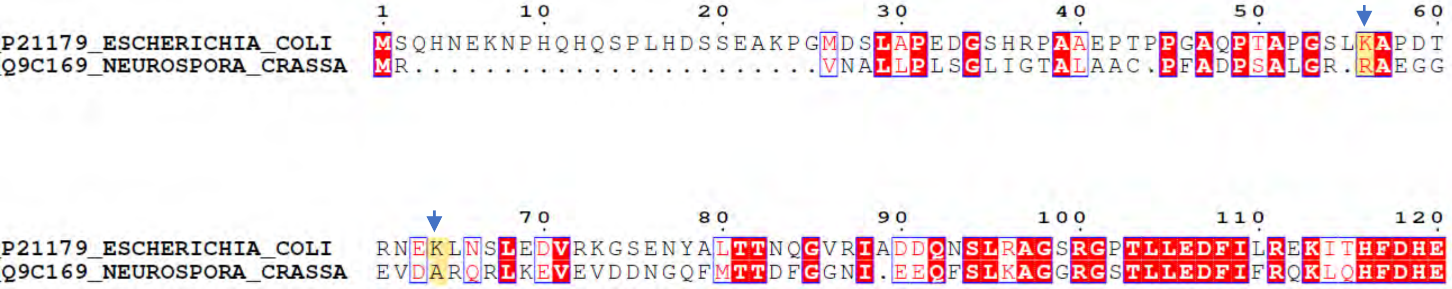

Full sequences in supplemental file.

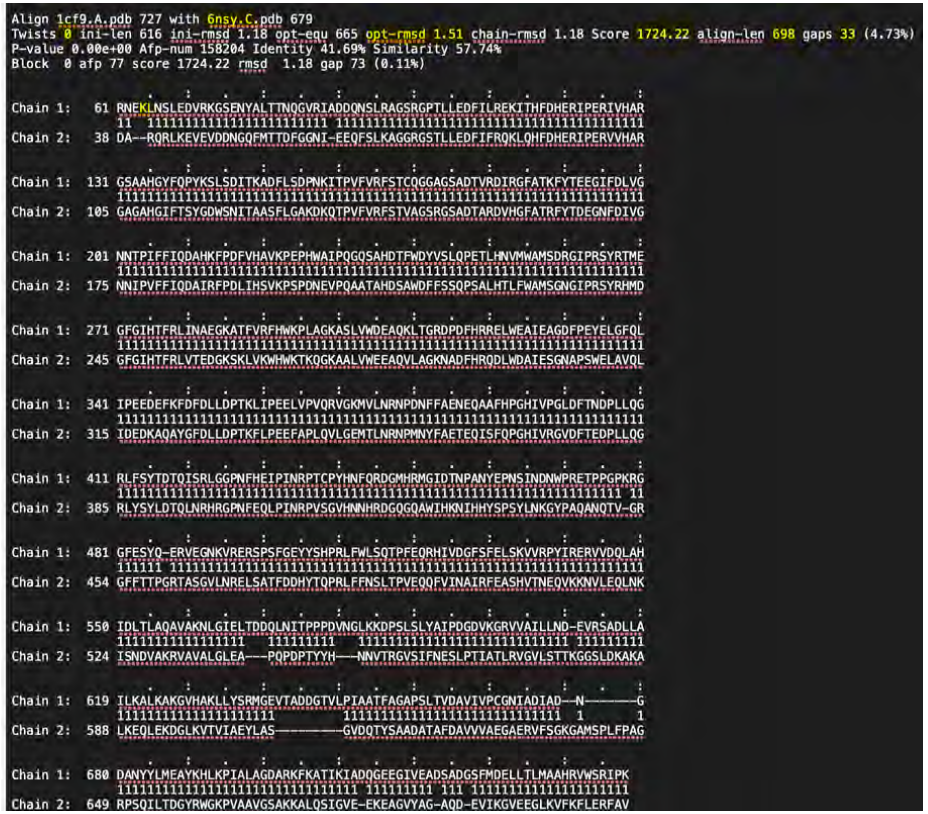

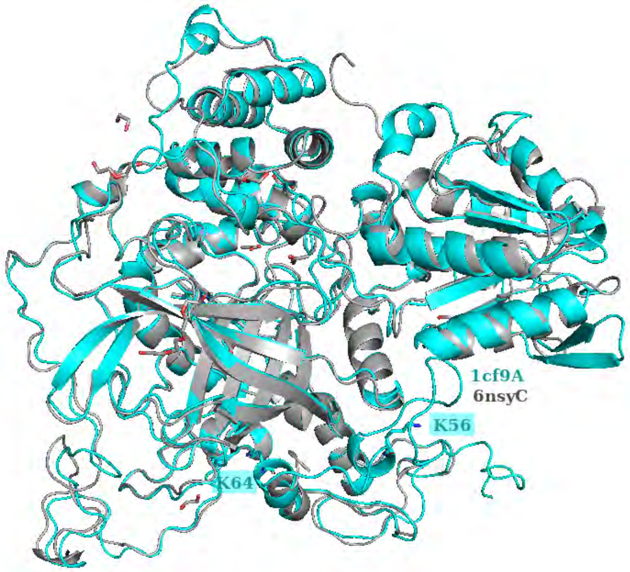

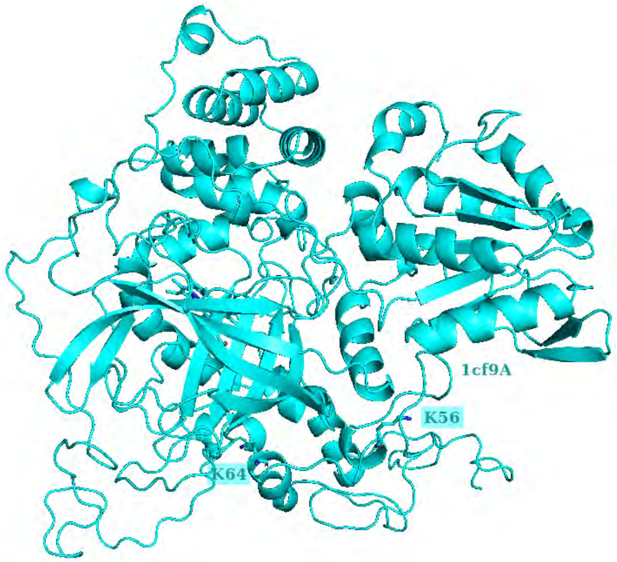

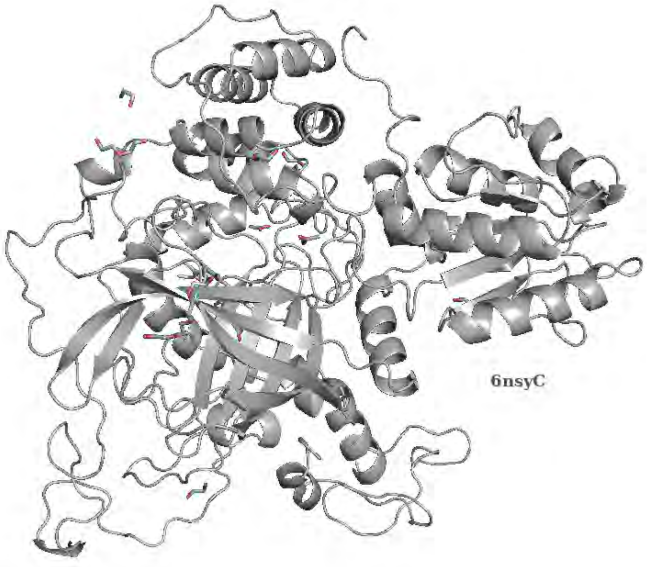

UniProt ID: Q9C169
PDB ID: 6NSZ_C

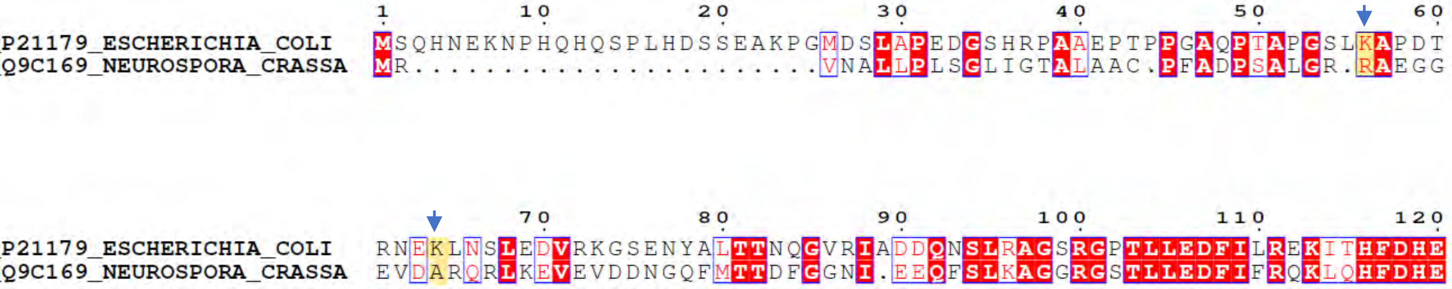

Full sequences in supplemental file.

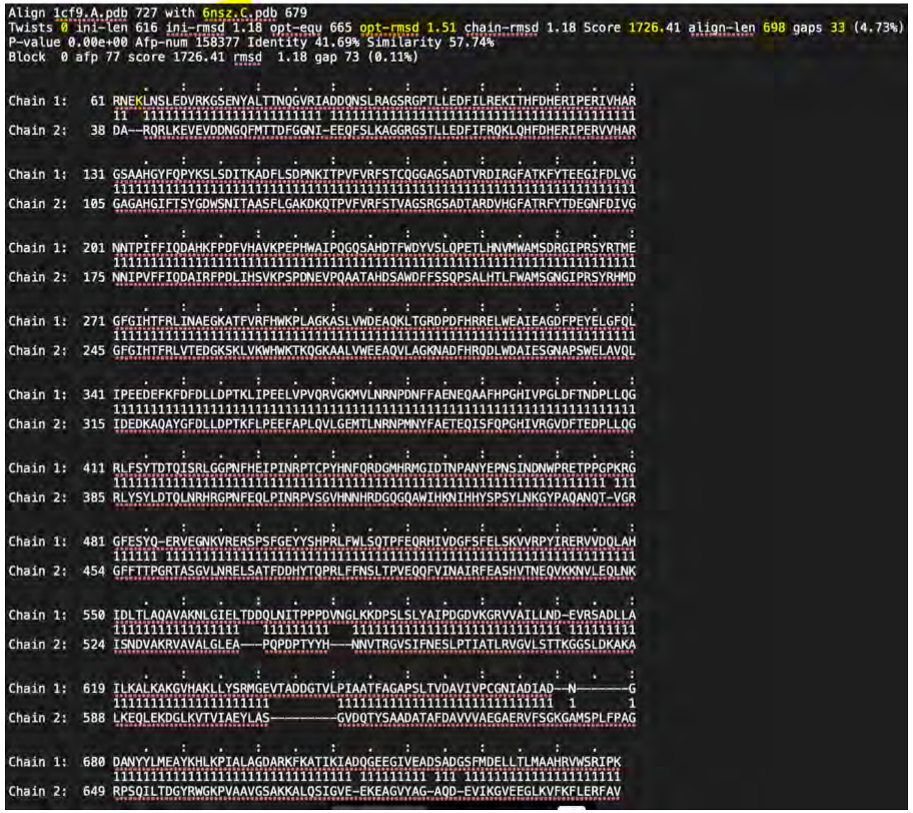

```
Align 1cf9.A.pdb 727 with 6nsz.C.pdb 679
Twists 0 ini-len 616 ini-rmsd 1.18 opt-equ 665 opt-rmsd 1.51 chain-rmsd 1.18 Score 1726.41 align-len 698 gaps 33 (4.73%)
P-value 0.00e+00 Afp-num 158377 Identity 41.69% Similarity 57.74%
Block  0 afp 77 score 1726.41 rmsd  1.18 gap 73 (0.11%)

Chain 1:   61 RNEKLNSLEDVRKGSENYALTTNQGVRIADDQNSLRAGSRGPTLLEDFILREKITHFDHERIPERIVHAR
              11  1111111111111111111111111 111111111111111111111111111111111111111111
Chain 2:   38 DA--RQRLKEVEVDDNGQFMTTDFGGNI-EEQFSLKAGGRGSTLLEDFIFRQKLQHFDHERIPERVVHAR

Chain 1:  131 GSAAHGYFQPYKSLSDITKADFLSDPNKITPVFVRFSTCQGGAGSADTVRDIRGFATKFYTEEGIFDLVG
              1111111111111111111111111111111111111111111111111111111111111111111111
Chain 2:  105 GAGAHGIFTSYGDWSNITAASFLGAKDKQTPVFVRFSTVAGSRGSADTARDVHGFATRFYTDEGNFDIVG

Chain 1:  201 NNTPIFFIQDAHKFPDFVHAVKPEPHWAIPQGQSAHDTFWDYVSLQPETLHNVMWAMSDRGIPRSYRTME
              1111111111111111111111111111111111111111111111111111111111111111111111
Chain 2:  175 NNIPVFFIQDAIRFPDLIHSVKPSPDNEVPQAATAHDSAWDFFSSQPSALHTLFWAMSGNGIPRSYRHMD

Chain 1:  271 GFGIHTFRLINAEGKATFVRFHWKPLAGKASLVWDEAQKLTGRDPDFHRRELWEAIEAGDFPEYELGFQL
              1111111111111111111111111111111111111111111111111111111111111111111111
Chain 2:  245 GFGIHTFRLVTEDGKSKLVKWHWKTKQGKAALVWEEAQVLAGKNADFHRQDLWDAIESGNAPSWELAVQL

Chain 1:  341 IPEEDEFKFDFDLLDPTKLIPEELVPVQRVGKMVLNRNPDNFFAENEQAAFHPGHIVPGLDFTNDPLLQG
              1111111111111111111111111111111111111111111111111111111111111111111111
Chain 2:  315 IDEDKAQAYGFDLLDPTKFLPEEFAPLQVLGEMTLNRNPMNYFAETEQISFQPGHIVRGVDFTEDPLLQG

Chain 1:  411 RLFSYTDTQISRLGGPNFHEIPINRPTCPYHNFQRDGMHRMGIDTNPANYEPNSINDNWPRETPPGPKRG
              111111111111111111111111111111111111111111111111111111111111111111 111
Chain 2:  385 RLYSYLDTQLNRHRGPNFEQLPINRPVSGVHNNHRDGQGQAWIHKNIHHYSPSYLNKGYPAQANQT-VGR

Chain 1:  481 GFESYQ-ERVEGNKVRERSPSFGEYYSHPRLFWLSQTPFEQRHIVDGFSFELSKVVRPYIRERVVDQLAH
              111111 111111111111111111111111111111111111111111111111111111111111111
Chain 2:  454 GFFTTPGRTASGVLNRELSATFDDHYTQPRLFFNSLTPVEQQFVINAIRFEASHVTNEQVKKNVLEQLNK

Chain 1:  550 IDLTLAQAVAKNLGIELTDDQLNITPPPDVNGLKKDPSLSLYAIPDGDVKGRVVAILLND-EVRSADLLA
              11111111111111111   111111111   111111111111111111111111111111 11111111
Chain 2:  524 ISNDVAKRVAVALGLEA---PQPDPTYYH---NNVTRGVSIFNESLPTIATLRVGVLSTTKGGSLDKAKA

Chain 1:  619 ILKALKAKGVHAKLLYSRMGEVTADDGTVLPIAATFAGAPSLTVDAVIVPCGNIADIAD--N-------G
              1111111111111111111111          11111111111111111111111111111  1       1
Chain 2:  588 LKEQLEKDGLKVTVIAEYLAS----------GVDQTYSAADATAFDAVVVAEGAERVFSGKGAMSPLFPAG

Chain 1:  680 DANYYLMEAYKHLKPIALAGDARKFKATIKIADQGEEGIVEADSADGSFMDELLTLMAAHRVWSRIPK
              1111111111111111111111111111111111 111111111 111 11111111111111111111111
Chain 2:  649 RPSQILTDGYRWGKPVAAVGSAKKALQSIGVE-EKEAGVYAG-AQD-EVIKGVEEGLKVFKFLERFAV
```

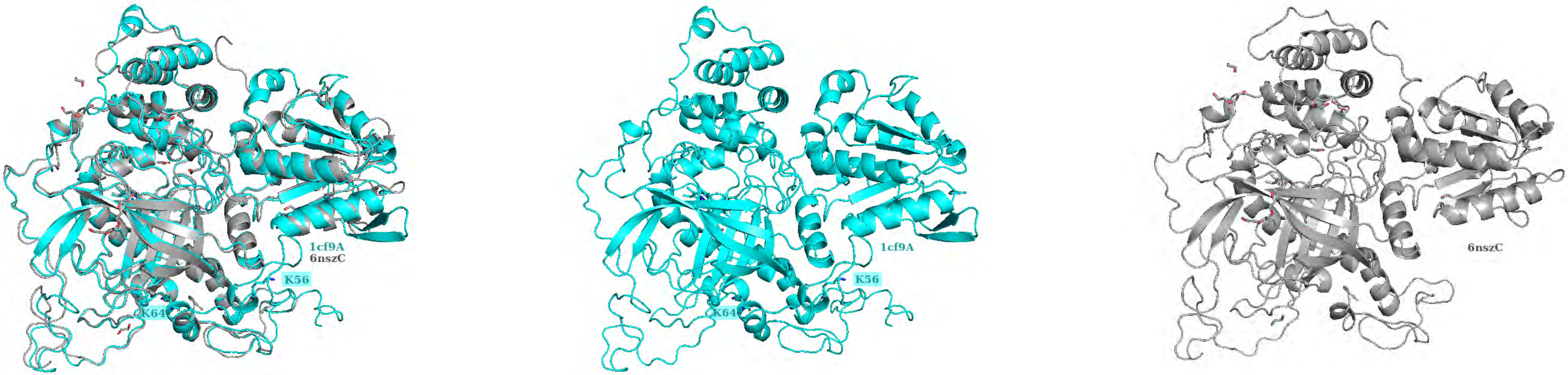

UniProt ID: Q9C169

PDB ID: 6NT0_C

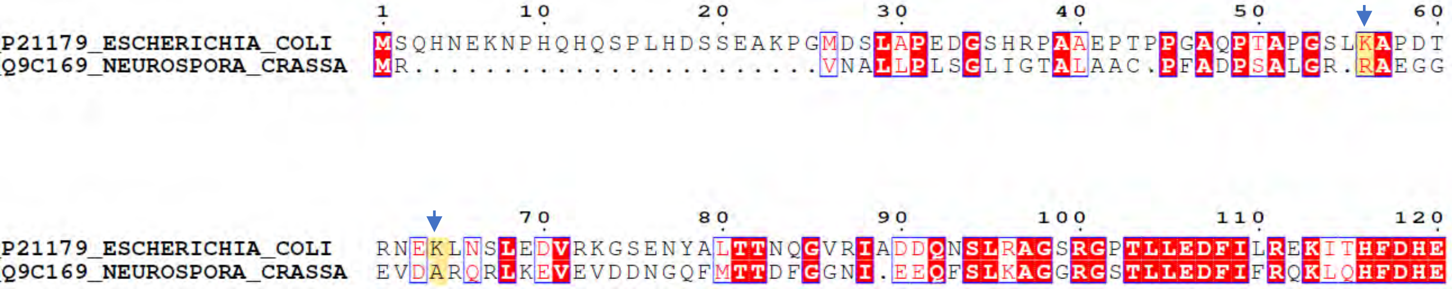

Full sequences in supplemental file.

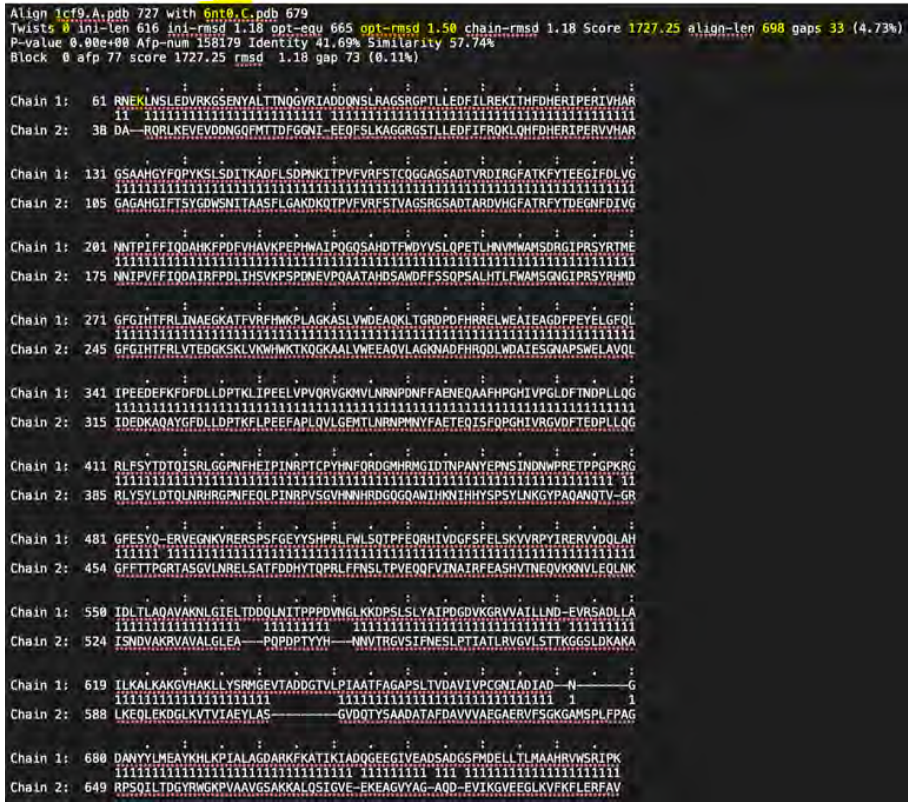

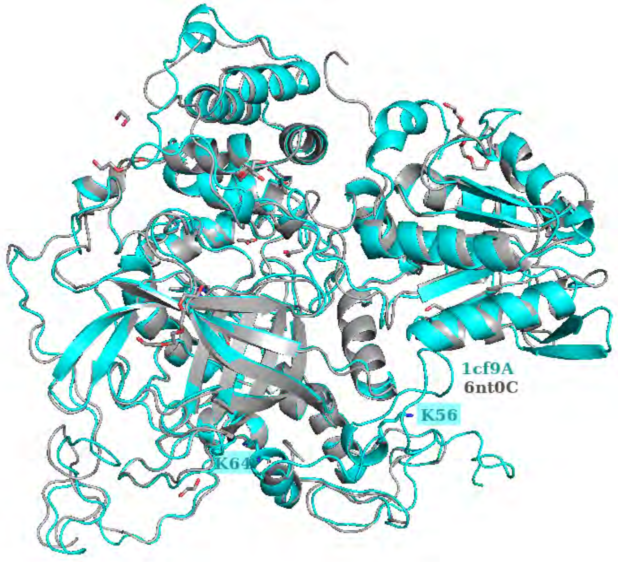

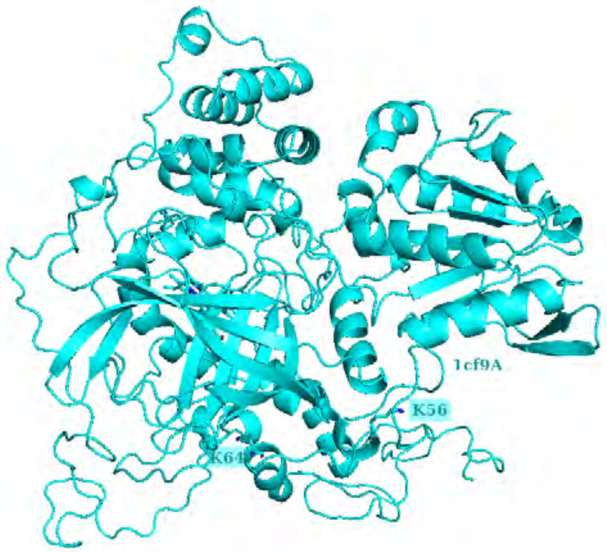

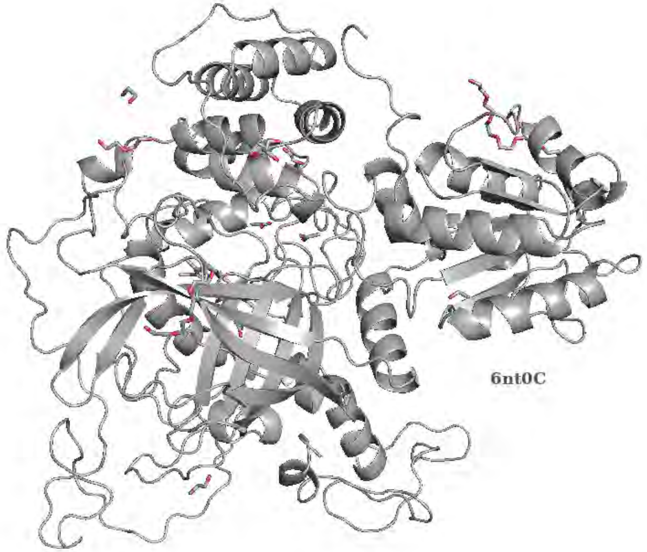

UniProt ID: Q9C169

PDB ID: 6NT1_C

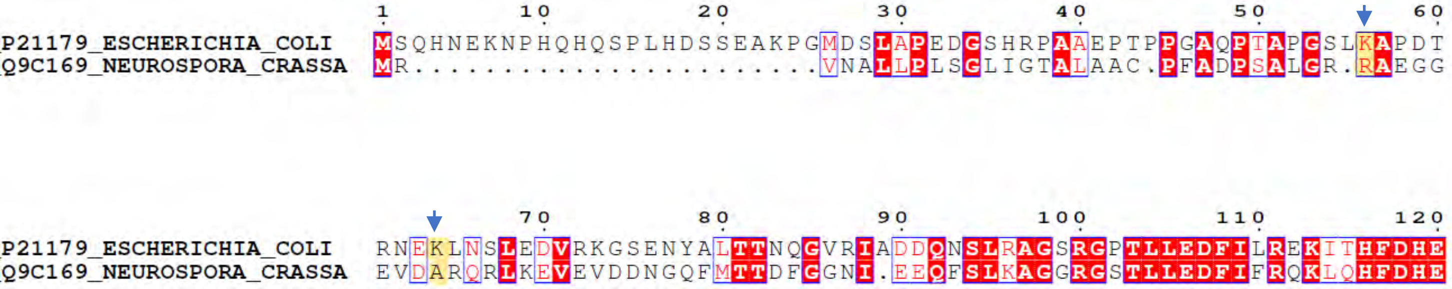

Full sequences in supplemental file.

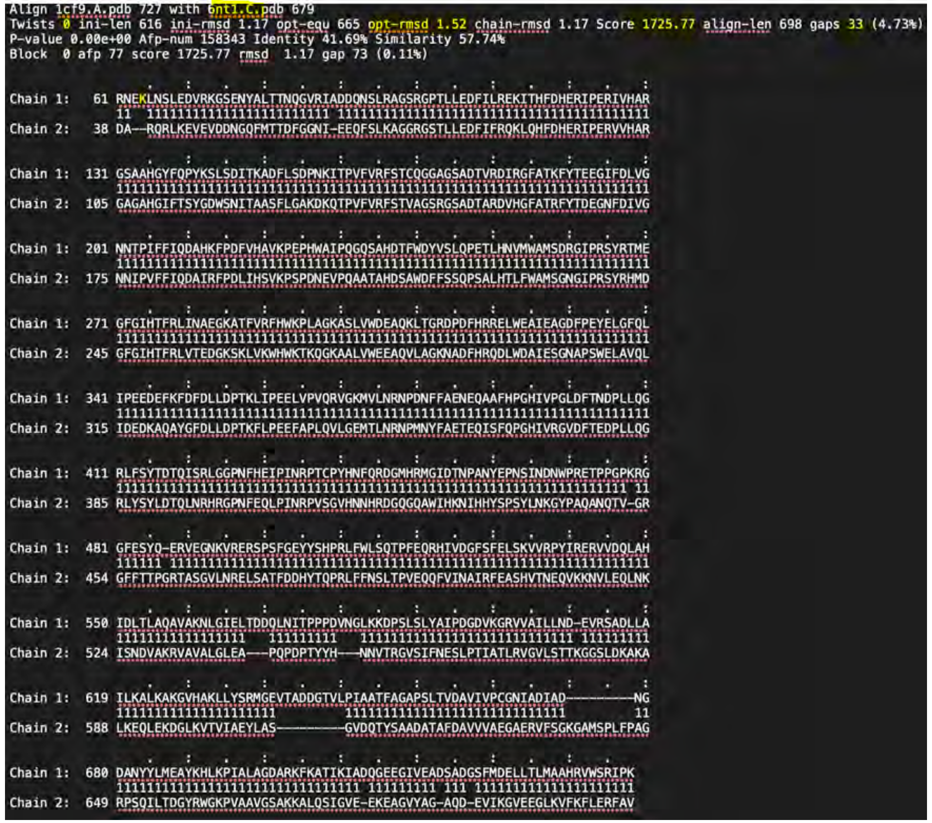

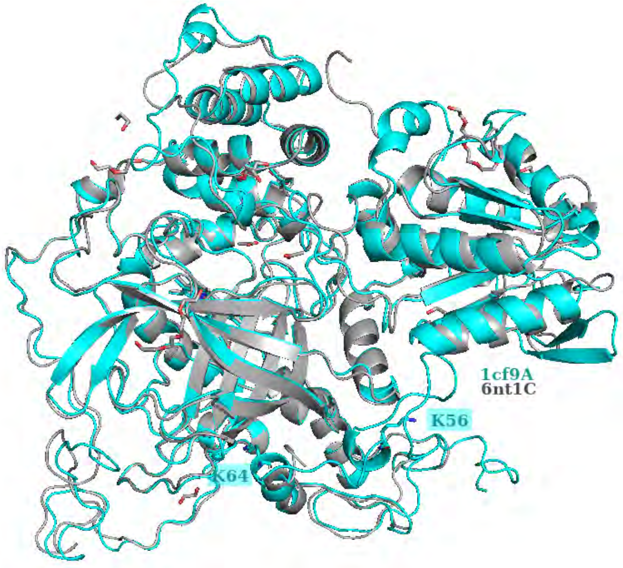

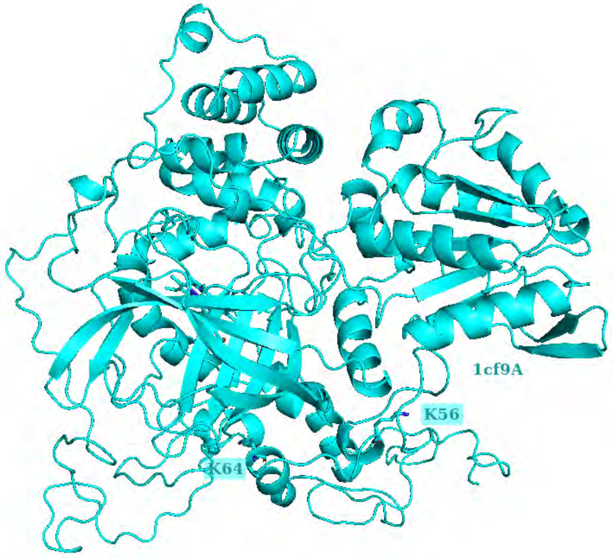

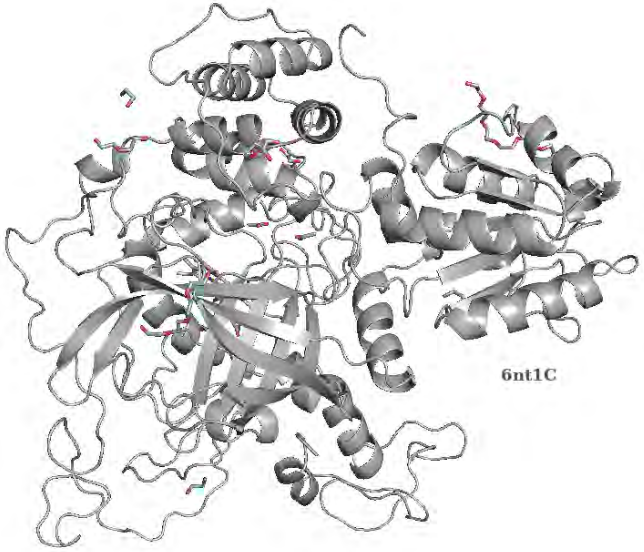

UniProt ID: R4GRT6

PDB ID: 4B2Y_B

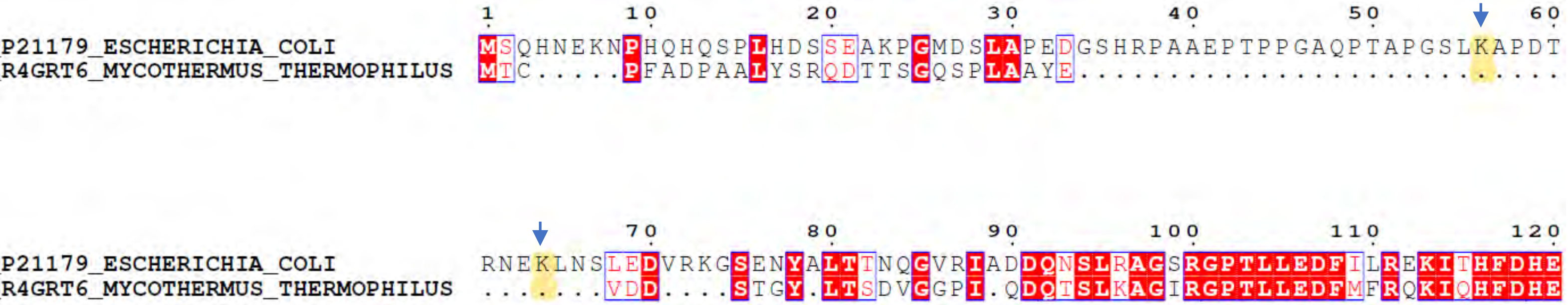

Full sequences in supplemental file.

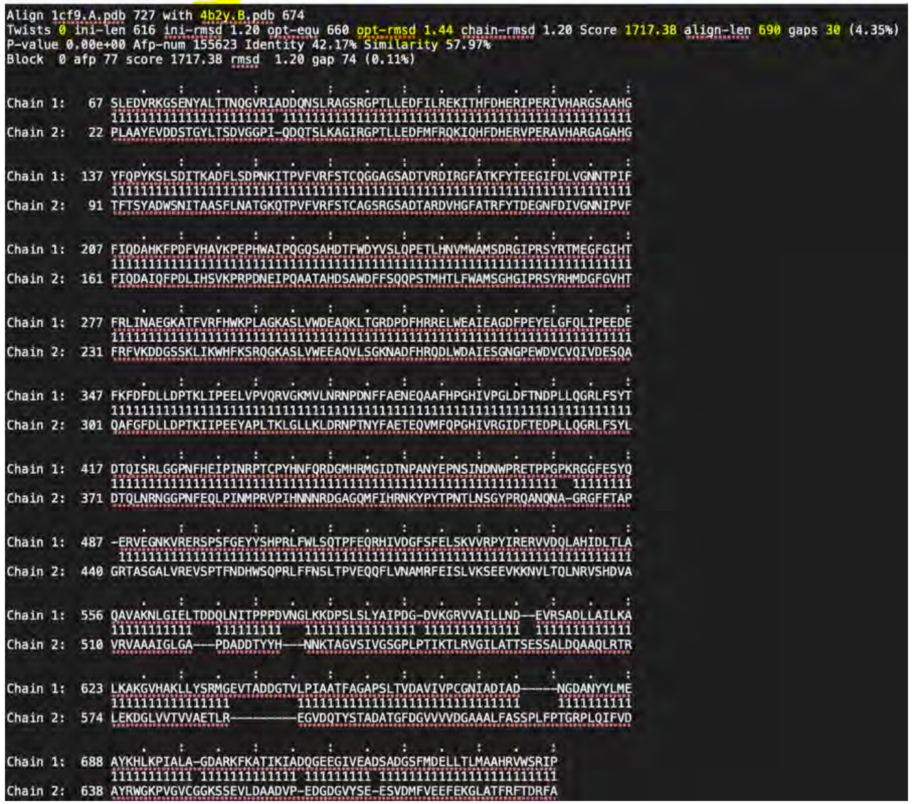

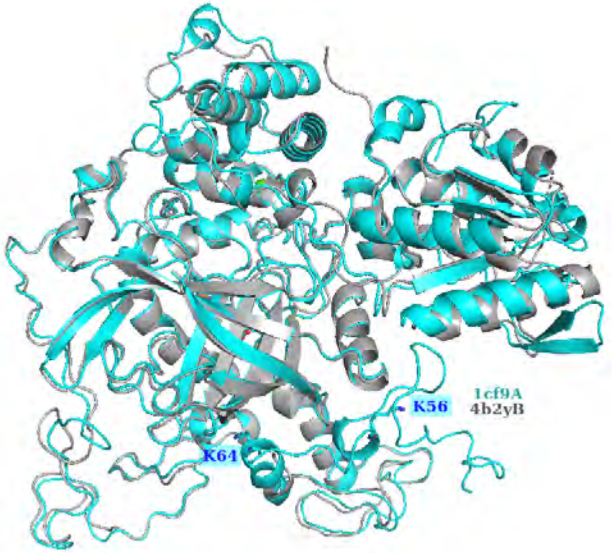

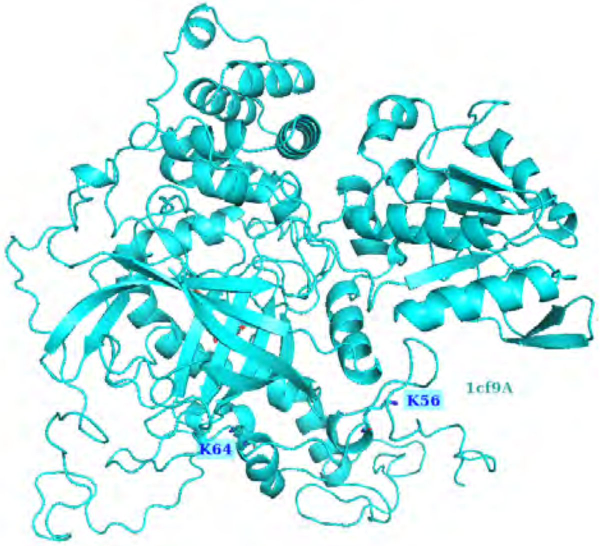

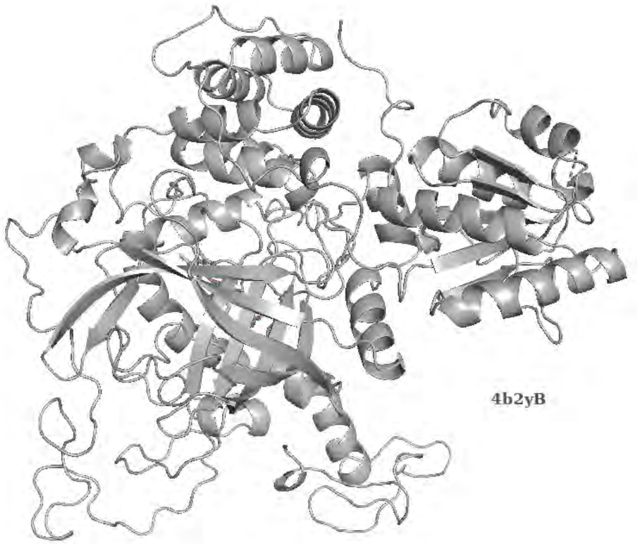

UniProt ID: R4GRT7
PDB ID: 4B31_B

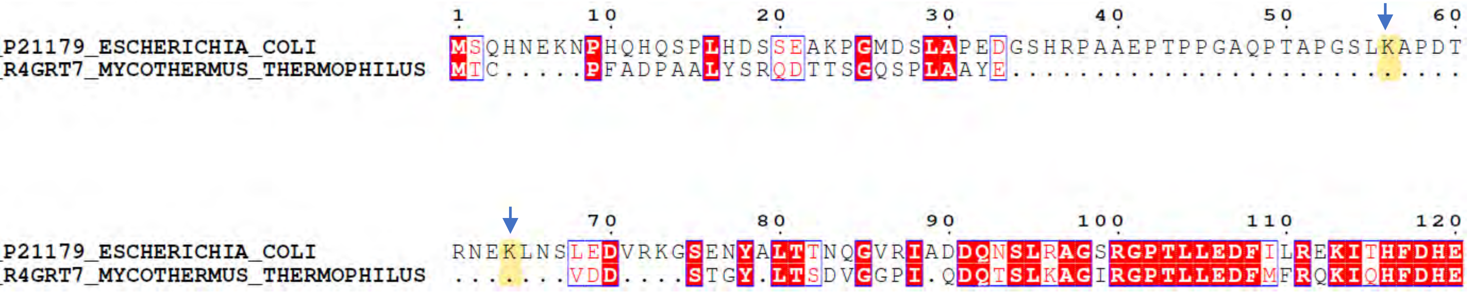

Full sequences in supplemental file.

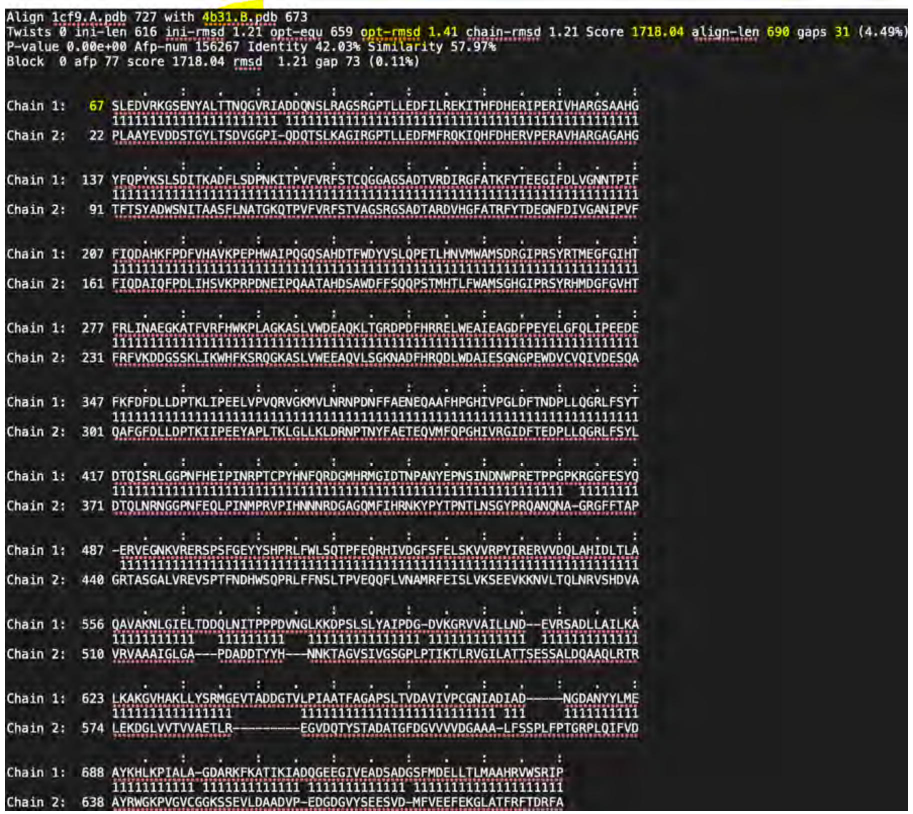

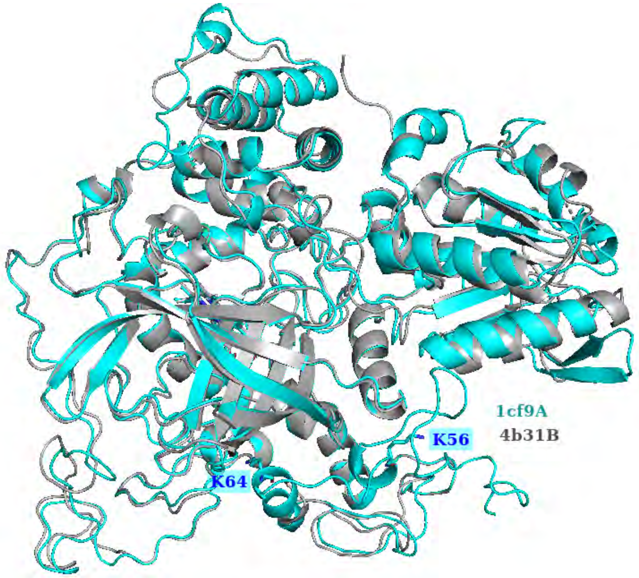

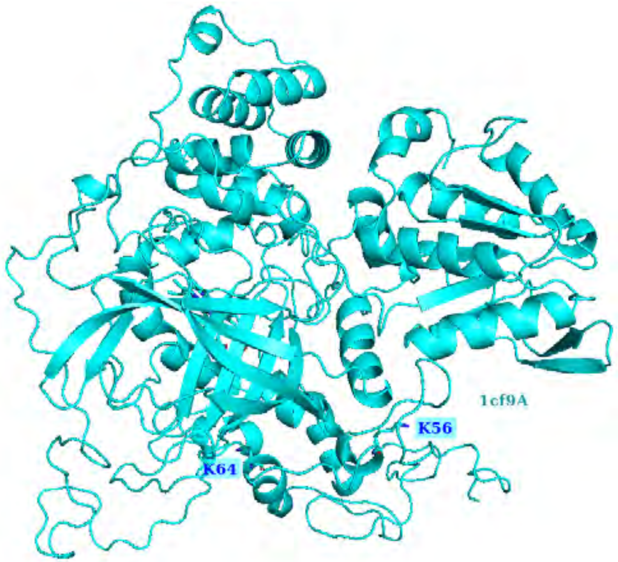

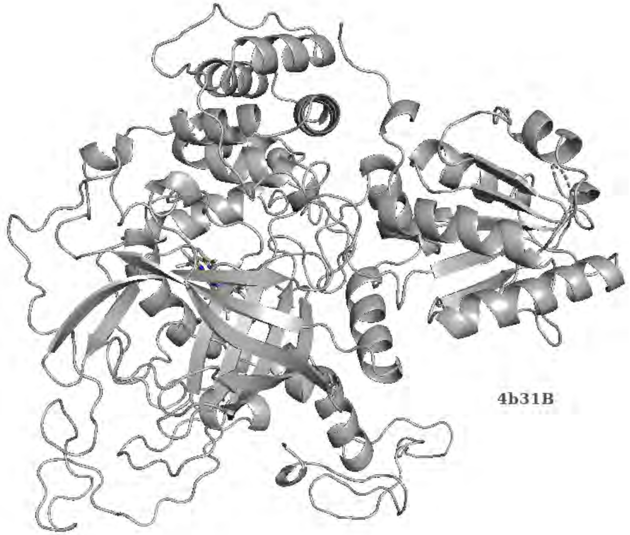

UniProt ID: R4GRT8

PDB ID: 4B40_D

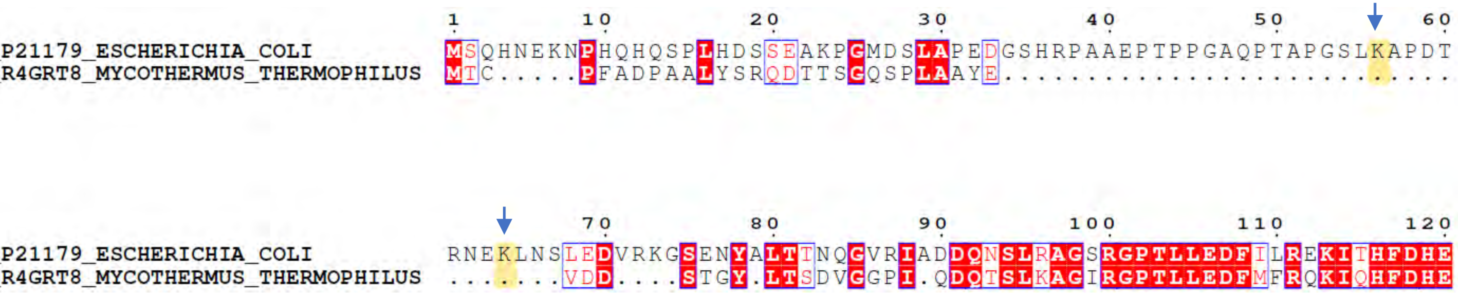

Full sequences in supplemental file.

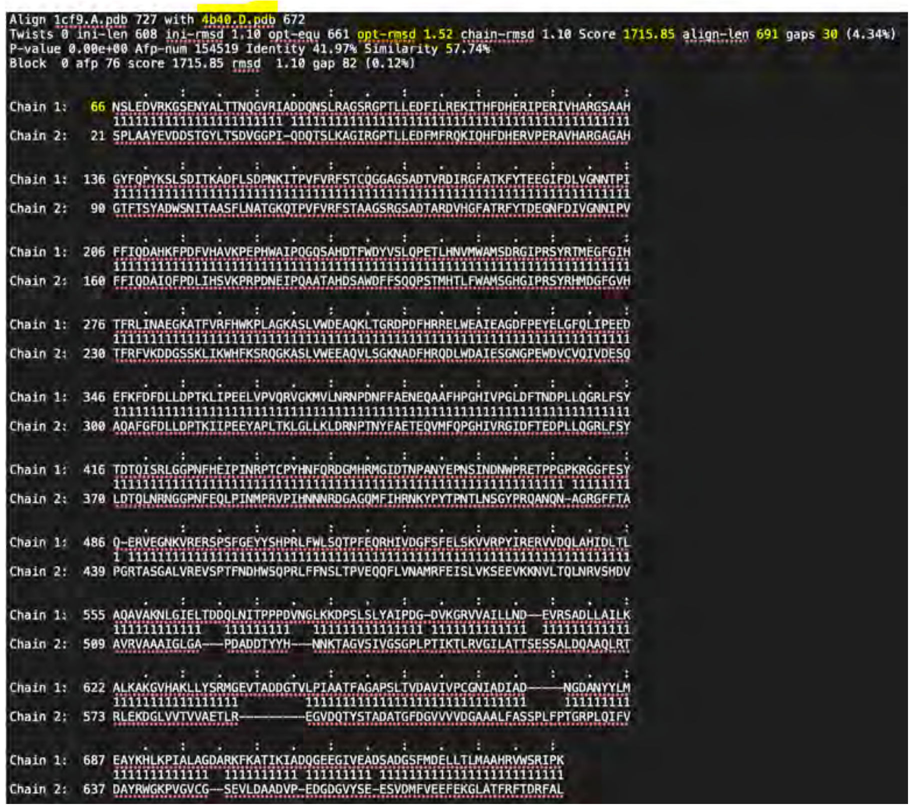

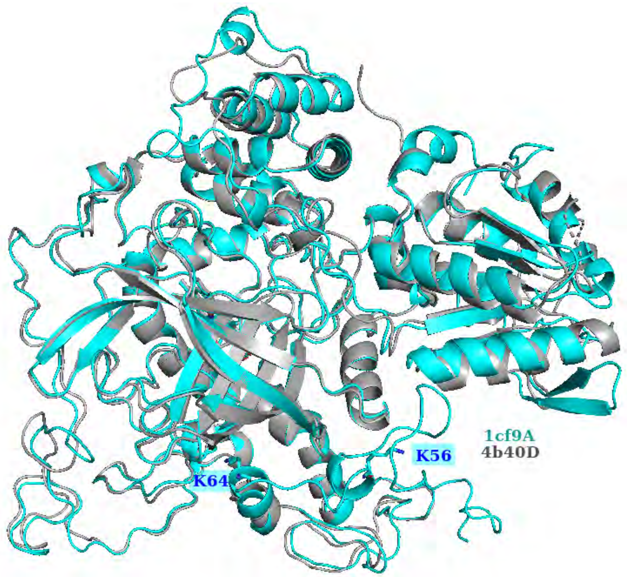

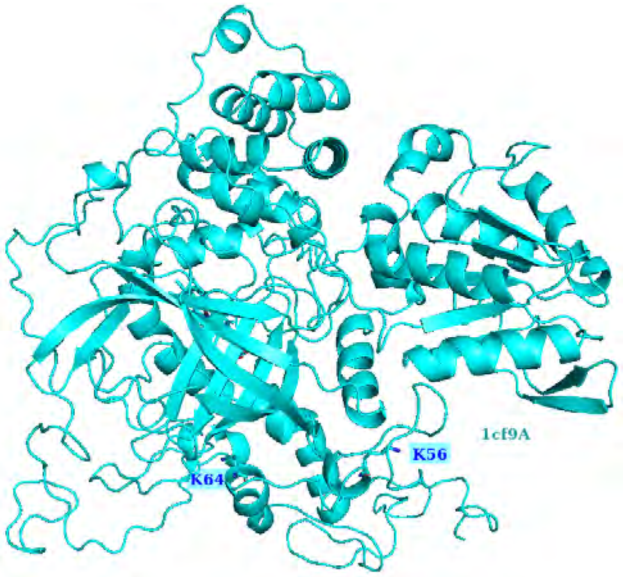

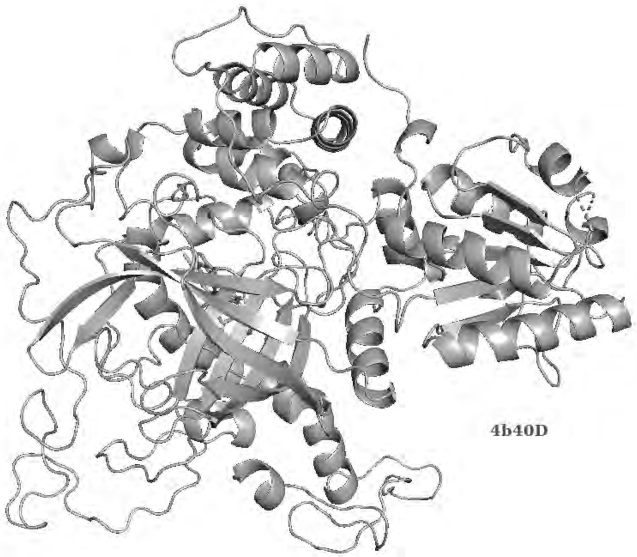

UniProt ID: R4GRT9

PDB ID: 4B5K_D

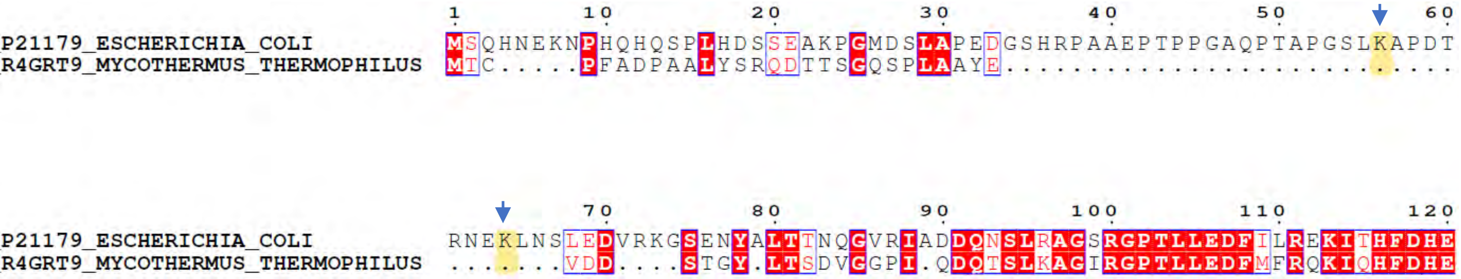

Full sequences in supplemental file.

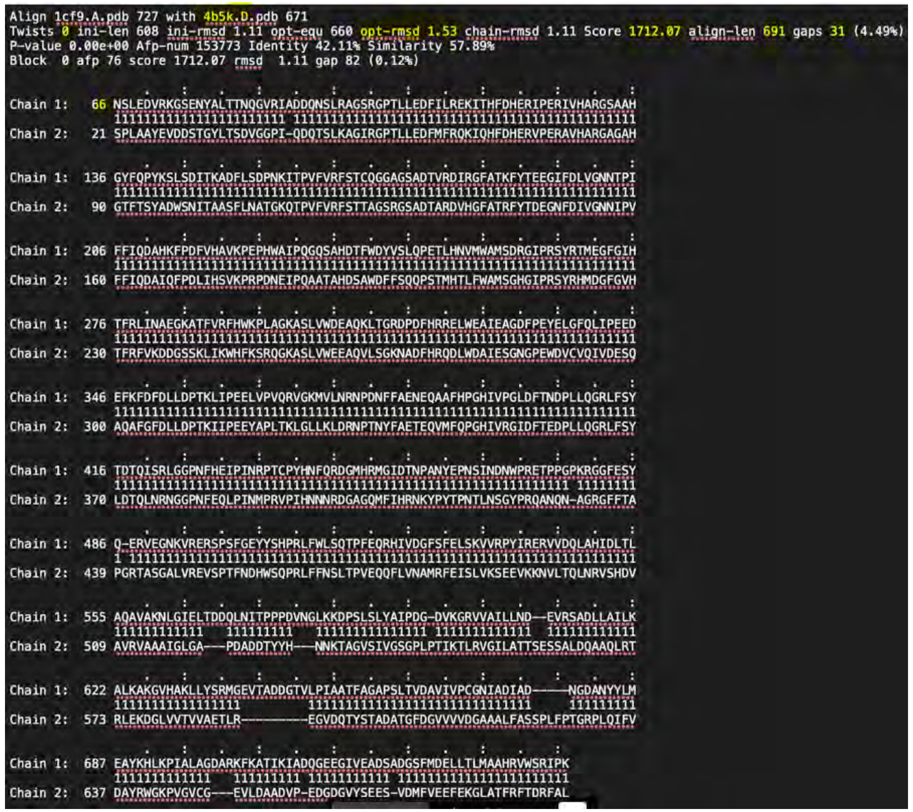

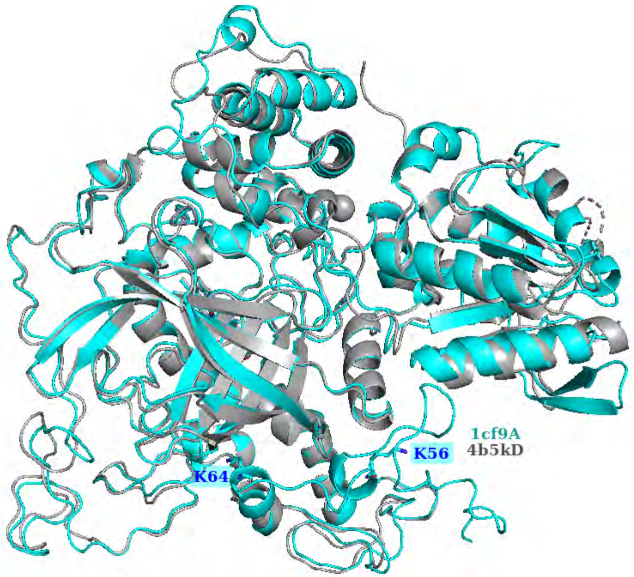

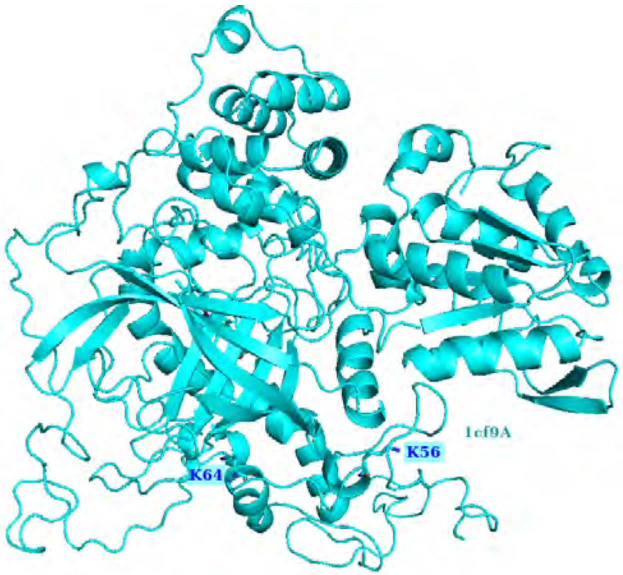

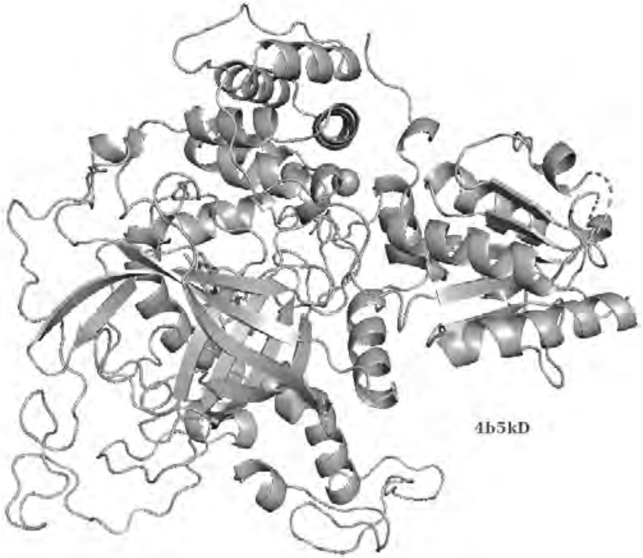

UniProt ID: W1F4G9

PDB ID: 6JQQ_C

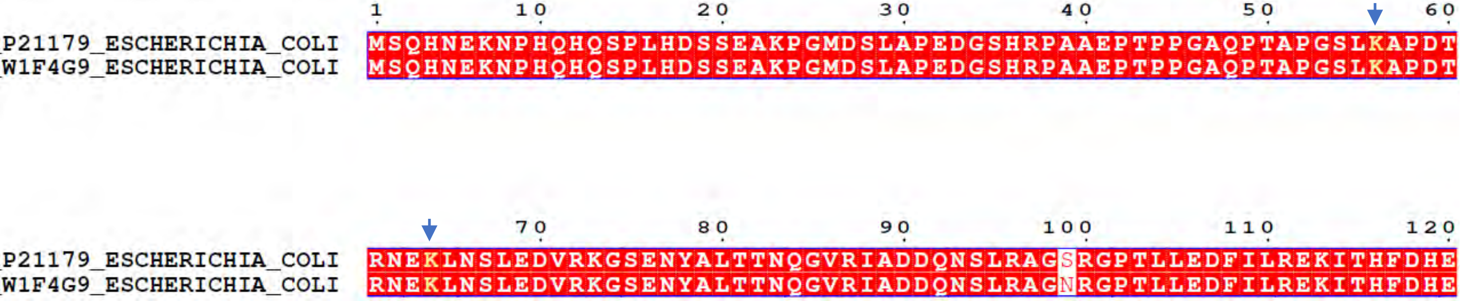

Full sequences in supplemental file.

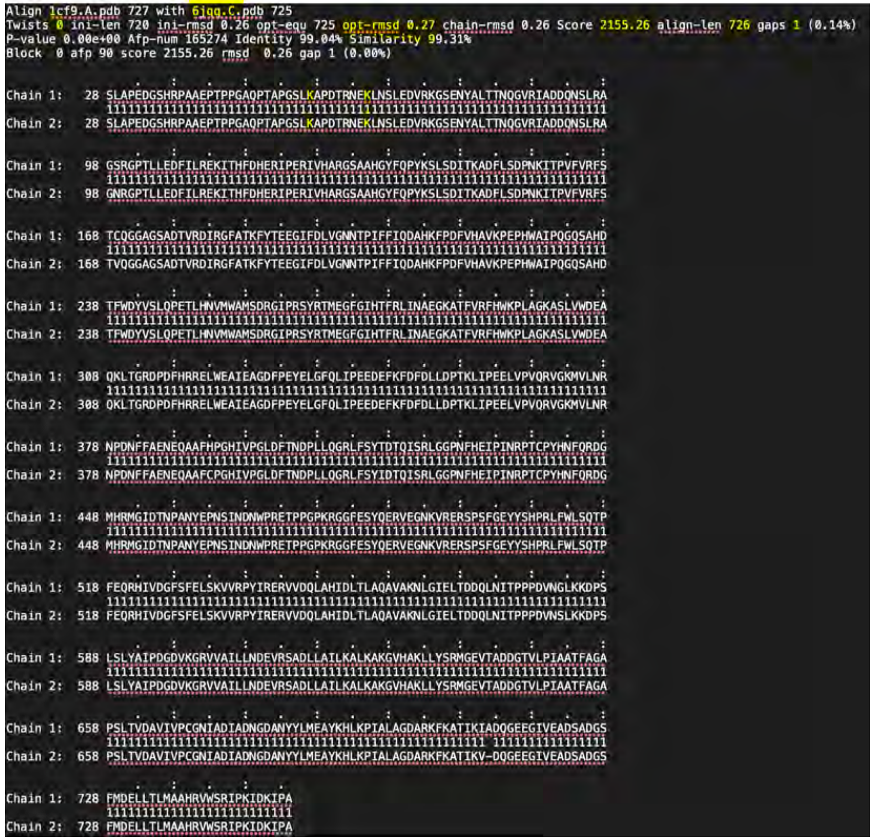

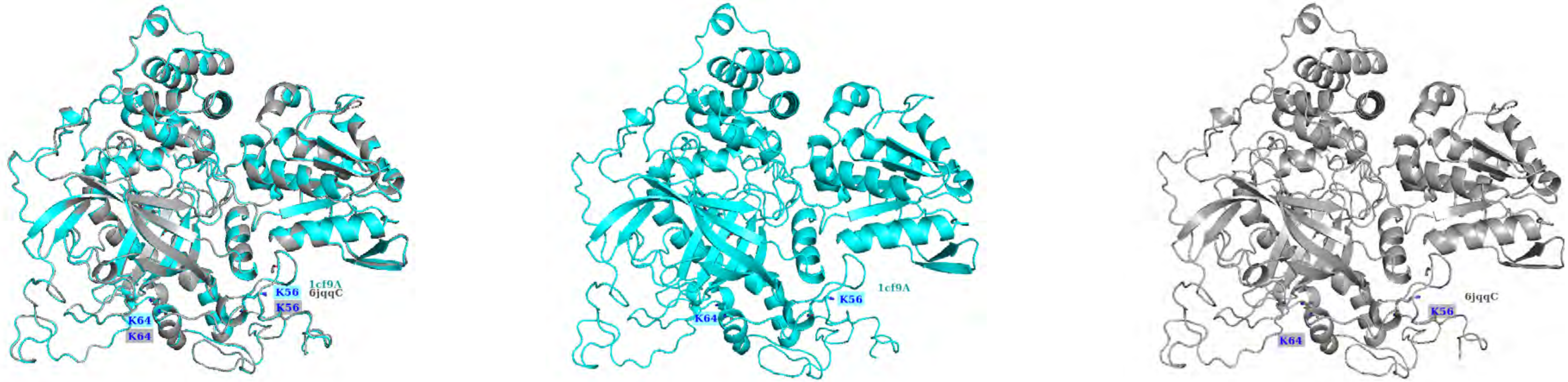

Supplement: Supplementary file 4 [file Data_Sheet_4.PDF]
