# Supplementary material for: Investigation of the Importance of Protein 3D Structure for Assessing Conservation of Lysine Acetylation Sites in Protein Homologs
Source: Front Microbiol. 2022 Jan 31;12:805181. doi: 10.3389/fmicb.2021.805181 (PMC8843374; doi:10.3389/fmicb.2021.805181)

Supplemental Figure SF2D. Compiled pairwise sequence (Cobalt) and structural (FATCAT) alignments between the *E. coli* substrate protein target (Fmt-methionyl-tRNA formyltransferase; PDB ID: 2fmt) and homologs sorted by UniProt ID. Lysine residues previously identified as acetylated in the target protein are highlighted in yellow in the sequence alignments and FATCAT structural alignment xml files to examine conservation. 3D protein structures are shown as ribbon representations with the target protein in cyan and the homolog protein in gray. Blue lysine residues correspond to KAT (lysine acetyltransferase) acetylation sites, red lysine residues correspond to AcP (acetyl phosphate) acetylation sites, and purple lysine residues correspond to sites acetylated by both KAT and AcP. 1D sequence alignments are not shown between *E. coli* proteins because sequences were identical.

UniProt ID: A0A0H2UKZ6

PDB ID: 4S1N_A

```
              1        10        20        30        40        50
P23882_ESCHERICHIA_COLI               MSESLRIIF..AGTPDFAARHLDALLSSGHNVVGVFTQPDRPAGRGKKLMPSPVKVLAEE
A0A0H2UKZ6_STREPTOCOCCUS_PNEUMONIAE   SNAMKKIAVFASGNGSNFQVIAEEF.....PVEFVFSDHRDAYVLERAKQLGVLSYAFEL
```

Full sequences in supplemental file.

```
Align 2fmt.A.pdb 314 with 4s1n.A.pdb 180
Twists 0 ini-len 152 ini-rmsd 1.81 opt-equ 172 opt-rmsd 1.89 chain-rmsd 1.81 Score 401.72 align-len 193 gaps 21 (10.88%)
P-value 1.67e-15 Afp-num 17012 Identity 20.21% Similarity 39.38%
Block  0 afp 19 score 401.72 rmsd  1.81 gap 36 (0.19%)

                  .    :    .    :    .    :    .    :    .    :    .    :    .    :
Chain 1:    3 SLRIIFAGTPDFAARHLDALLSSGHNVVGVFTQPDRPAGRGKKLMPSPVKVLAEEKGLPVFQPVS-----
              11111111111  1111111111 11111111111          11111111111111111111
Chain 2:    1 MKKIAVFASGN--GSNFQVIAEE-FPVEFVFSDHR----------DAYVLERAKQLGVLSYAFELKEFES

                  .    :    .    :    .    :    .    :    .    :    .    :    .    :
Chain 1:   68 LRP--QENQQLVAELQADVMVVVAYGLILPKAVLEMPRLGCINVHGSLLPRWRGAAPIQRSLWAGDAETG
              111  11111111111111111111111111111111 11111111111111111111111111111111
Chain 2:   58 KADYEAALVELLEEHQIDLVCLAGYMKIVGPTLLSAYEGRIVNIHPAYLPEFPGAHGIEDAWNAGVGQSG

                  .    :    .    :    .    :    .    :    .    :
Chain 1:  136 VTIMQMDVGLDTGDMLYKLSCPITAEDTSGTLYDKLAELGPQGLITTLKQLAD
              11111111111111111111111111111111111111111111111111111
Chain 2:  128 VTIHWVDSGVDTGQVIKQVRVPRLADDTIDRFEARIHEAEYRLYPEVVKALFT

Note: positions are from PDB; the numbers between alignments are block index
```

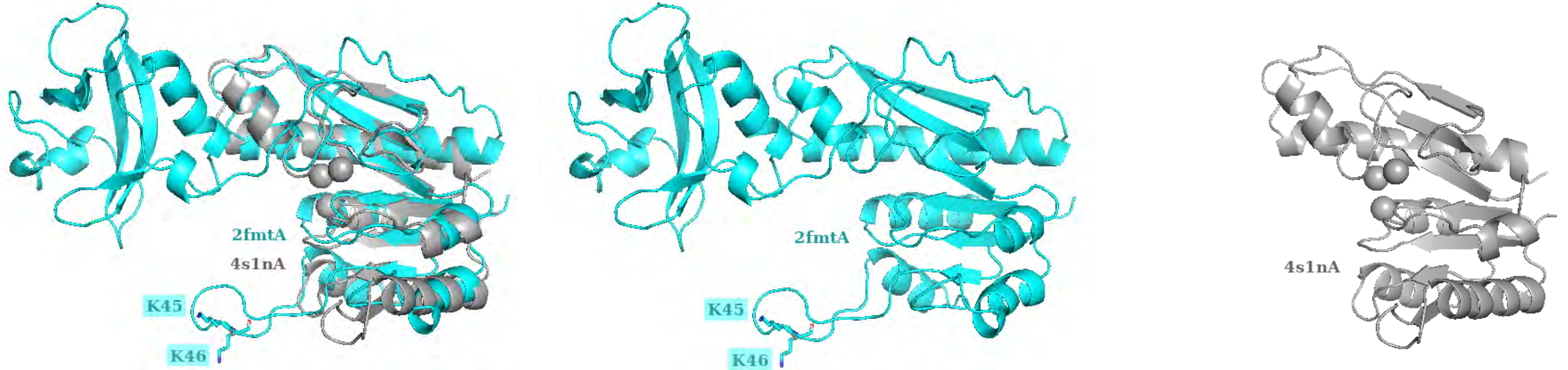

UniProt ID: A3DHJ7

PDB ID: 1ZGH_A

```
                                        1        10        20        30        40        50        60
P23882_ESCHERCHIA_COLI                  MSESLRIIFAGTPDFAARHLDALLSSGHNVVGVFTQPDRPAGRGKKLMPSPVKVLAEEKG
A3DHJ7_HUNGATEICLOSTRIDIUM_THERMOCELLUM ...........................MNIIIATTKSWNIKNAQKFKKENESKYNT....
```

Full sequences in supplemental file.

```
Align 2fmt.A.pdb 314 with 1zgh.A.pdb 227
Twists 0 ini-len 200 ini-rmsd 2.68 opt-equ 221 opt-rmsd 2.96 chain-rmsd 2.68 Score 474.10 align-len 294
gaps 73 (24.83%)
P-value 2.33e-15 Afp-num 21813 Identity 12.93% Similarity 33.67%
Block  0 afp 25 score 474.10 rmsd  2.68 gap 91 (0.31%)

                .    :    .    :    .    :    .    :    .    :    .    :    .    :
Chain 1:    3 SLRIIFAGTPDFAARHLDALLSSG--HNVVGVFTQPDRPAGRGKKLMPSPVKVLAEEKGLPVFQPVSLRP
              111111111111111111111111  1111111111                         111
Chain 2:    0 LMNIIIATTKSWNIKNAQKFKKENESKYNTTIITNK-------------------------DEL-----

                .    :    .    :    .    :    .    :    .    :    .    :    .    :
Chain 1:   71 QENQQLVAELQADVMVVVAYGLILPKAVLEMPRLGCINVHGSLLPRWRGAAPIQRSLWAGDAETGVTIMQ
                11111111111111111111111111111  1111111111111111111111111111111111111
Chain 2:   39 --TFEKVKLINPEYILFPHWSWIIPKEIFENF--TCVVFHMTDLPFGRGGSPLQNLIERGIKKTKISAIK

                .    :    .    :    .    :    .    :    .    :    .    :    .    :
Chain 1:  141 MDVGLDTGDMLYKLSCPITAEDTSGTLYDKLAELGPQ-GLITTLKQLADGTAKPEVQDETLVTYAEKLSK
              1111111111111111111  1111111111111111 111111111    1111111111111111111
Chain 2:  105 VDGGIDTGDIFFKRDLDLY--GTAEEIFMRASKIIFNDMIPELLTKR----PVPQKQEGEATVFQRRKP-

                .    :    .    :    .    :    .    :    .    :    .    :    .    :
Chain 1:  210 EEARIDWSLSAAQLERCIRAFNP--WPMSWLEIEGQPVKVWKASVIDTATNAAPGTILEANKQGIQVATG
              11111111111111111111111  11111111111111111111111
Chain 2:  168 EQSEISPDFDLEKIYDYIRMLDGEGYPRAFIKYGKYRLEFSRASMKN-----------------------

                .    :
Chain 1:  278 DGILNLLSLQPAGK
                 11111111111
Chain 2:  215 ---GKIIADVEIIE

Note: positions are from PDB; the numbers between alignments are block index
```

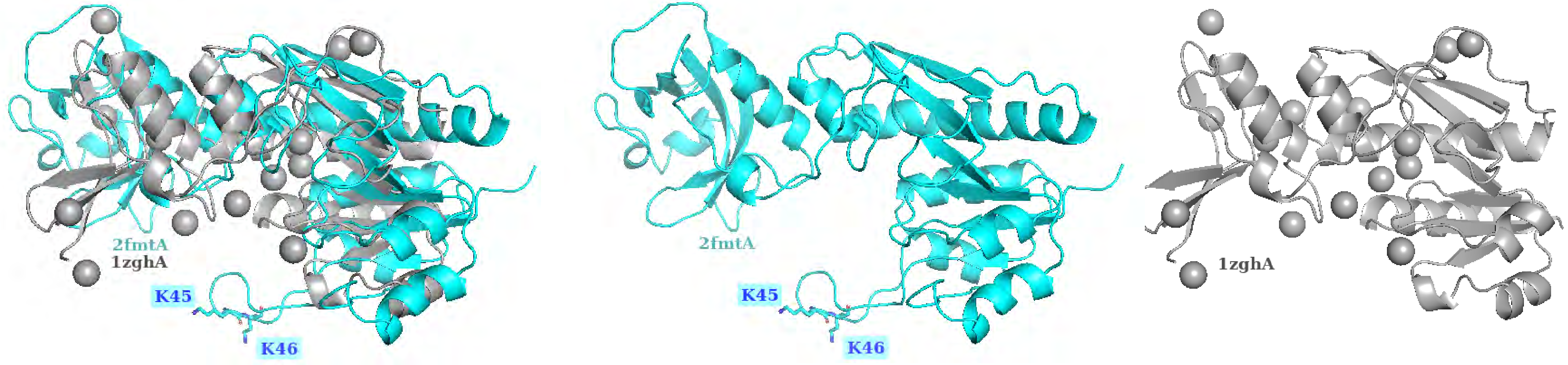

UniProt ID: E3NZ06

PDB ID: 4QPD_B

```
                          1         10        20        30          40        50
P23882_ESCHERICHIA_COLI   MSESLRIIFAGTPDFAARHLDALLSSGHNVVGVFT..QPDRPAGRGKKLMPSPVKVLAEE
E3NZ06_DANIO_RERIO        ....MKIAVIGQSLFGQEVYKELKNEGHMIVGVFTIPDKDGKVDPLAIEAEKDGVPVFKF
```

Full sequences in supplemental file.

```
Align 2fmt.A.pdb 314 with 4qpd.B.pdb 310
Twists 0 ini-len 272 ini-rmsd 1.84 opt-equ 288 opt-rmsd 1.96 chain-rmsd 1.84 Score 745.63 align-len 314 gaps 26 (8.28%)
P-value 0.00e+00 Afp-num 28792 Identity 26.75% Similarity 43.95%
Block  0 afp 34 score 745.63 rmsd  1.84 gap 40 (0.13%)

                  .    :    .    :    .    :    .    :    .    :    .    :    .    :
Chain 1:    4 LRIIFAGTPDFAARHLDALLSSGHNVVGVFTQPDRPAGRGKKLMPSPVKVLAEEKGLPVFQPVSL------
              11111111111111111111111111111111111       11111111111111111111111
Chain 2:    1 MKIAVIGQSLFGQEVYKELKNEGHMIVGVFTIPDK-------DGKVDPLAIEAEKDGVPVFKFPRWRLKGK

                  .    :    .    :    .    :    .    :    .    :    .    :    .    :
Chain 1:   69 RPQENQQLVAELQADVMVVVAYGLILPKAVLEMPRLGCINVHGSLLPRWRGAAPIQRSLWAGDAETGVTI
              1111111111111111111111111111111111111111111111111111111111111111111111
Chain 2:   65 AITEVVDQYKAVGAELNVLPFCSQFIPMEVIDHPKHGSIIYHPSLLPRHRGASAINWTLIHGDKKGGFTV

                  .    :    .    :    .    :    .    :    .    :    .    :    .    :
Chain 1:  139 MQMDVGLDTGDMLYKLSCPITAEDTSGTLYD-KLAELGPQGLITTLKQLADGTAKPEVQDETLVTYAEKL
              1111111111111111111111111111111 11111111111111111111111111111111111111
Chain 2:  135 FWADDGLDTGPILLQRECDVEPNDNVNSIYKRFLFPEGVKGMVEAVRLIATGKAPRIKQPEEGATYECIQ

                  .    :    .    :    .    :    .    :    .    :    .    :    .    :
Chain 1:  208 SKEEARIDWSLSAAQLERCIRAFNPWPMSWLEIEGQPVKVWKASVIDTATNA---------APGTILEAN
              111111111111111111111111111111111111111111111111             111111111
Chain 2:  205 KKENSKIDWNQPAEAIHNWIRGNDRVPGAWAEIDGKSVSFYGSTLLENDHFSSNGQPLEIPGASRAALVT

                  .    :    .    :    .    :
Chain 1:  269 KQGIQVATGD-GILNLLSLQPAGKKAMSAQDLLN
              1111111111 11111111111111111111111
Chain 2:  275 KNGLVLFGNDGKMLLVKNLQFEDGKMIPGSQYFK

Note: positions are from PDB; the numbers between alignments are block index
```

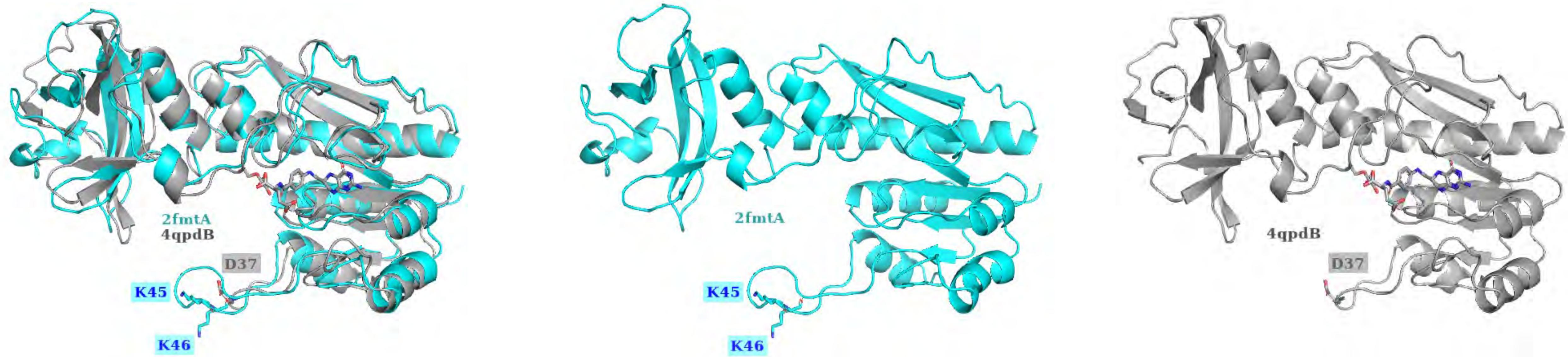

UniProt ID: E3NZ06

PDB ID: 4R8V_A

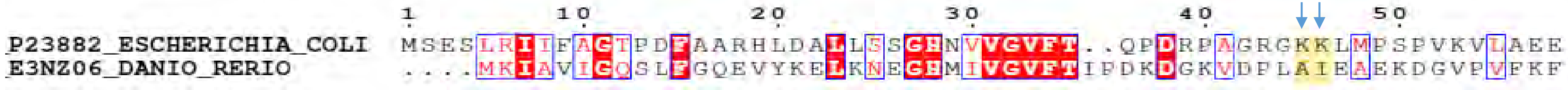

Full sequences in supplemental file.

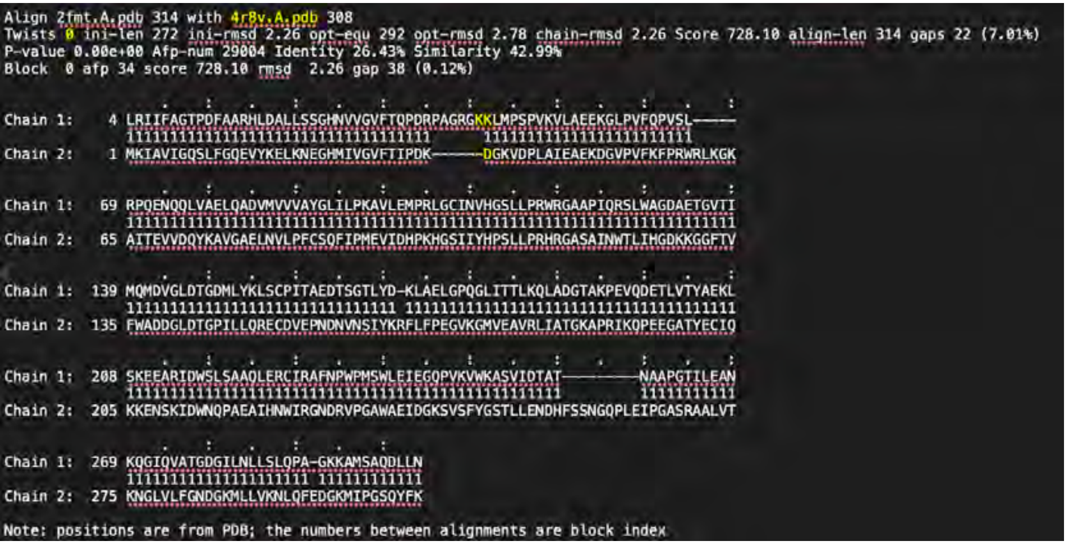

```
Align 2fmt.A.pdb 314 with 4r8v.A.pdb 308
Twists 0 ini-len 272 ini-rmsd 2.26 opt-equ 292 opt-rmsd 2.78 chain-rmsd 2.26 Score 728.10 align-len 314 gaps 22 (7.01%)
P-value 0.00e+00 Afp-num 29004 Identity 26.43% Similarity 42.99%
Block  0 afp 34 score 728.10 rmsd  2.26 gap 38 (0.12%)

                 .    :    .    :    .    :    .    :    .    :    .    :    .    :
Chain 1:    4 LRIIFAGTPDFAARHLDALLSSGHNVVGVFTQPDRPAGRGKKLMPSPVKVLAEEKGLPVFQPVSL-----
              11111111111111111111111111111111111      111111111111111111111111
Chain 2:    1 MKIAVIGQSLFGQEVYKELKNEGHMIVGVFTIPDK------DGKVDPLAIEAEKDGVPVFKFPRWRLKGK

                 .    :    .    :    .    :    .    :    .    :    .    :    .    :
Chain 1:   69 RPQENQQLVAELQADVMVVVAYGLILPKAVLEMPRLGCINVHGSLLPRWRGAAPIQRSLWAGDAETGVTI
              1111111111111111111111111111111111111111111111111111111111111111111111
Chain 2:   65 AITEVVDQYKAVGAELNVLPFCSQFIPMEVIDHPKHGSIIYHPSLLPRHRGASAINWTLIHGDKKGGFTV

                 .    :    .    :    .    :    .    :    .    :    .    :    .    :
Chain 1:  139 MQMDVGLDTGDMLYKLSCPITAEDTSGTLYD-KLAELGPQGLITTLKQLADGTAKPEVQDETLVTYAEKL
              1111111111111111111111111111111 11111111111111111111111111111111111111
Chain 2:  135 FWADDGLDTGPILLQRECDVEPNDNVNSIYKRFLFPEGVKGMVEAVRLIATGKAPRIKQPEEGATYECIQ

                 .    :    .    :    .    :    .    :    .    :    .    :    .    :
Chain 1:  208 SKEEARIDWSLSAAQLERCIRAFNPWPMSWLEIEGQPVKVWKASVIDTAT---------NAAPGTILEAN
              11111111111111111111111111111111111111111111111111         11111111111
Chain 2:  205 KKENSKIDWNQPAEAIHNWIRGNDRVPGAWAEIDGKSVSFYGSTLLENDHFSSNGQPLEIPGASRAALVT

                 .    :    .    :    .    :
Chain 1:  269 KQGIQVATGDGILNLLSLQPA-GKKAMSAQDLLN
              111111111111111111111 111111111111
Chain 2:  275 KNGLVLFGNDGKMLLVKNLQFEDGKMIPGSQYFK

Note: positions are from PDB; the numbers between alignments are block index
```

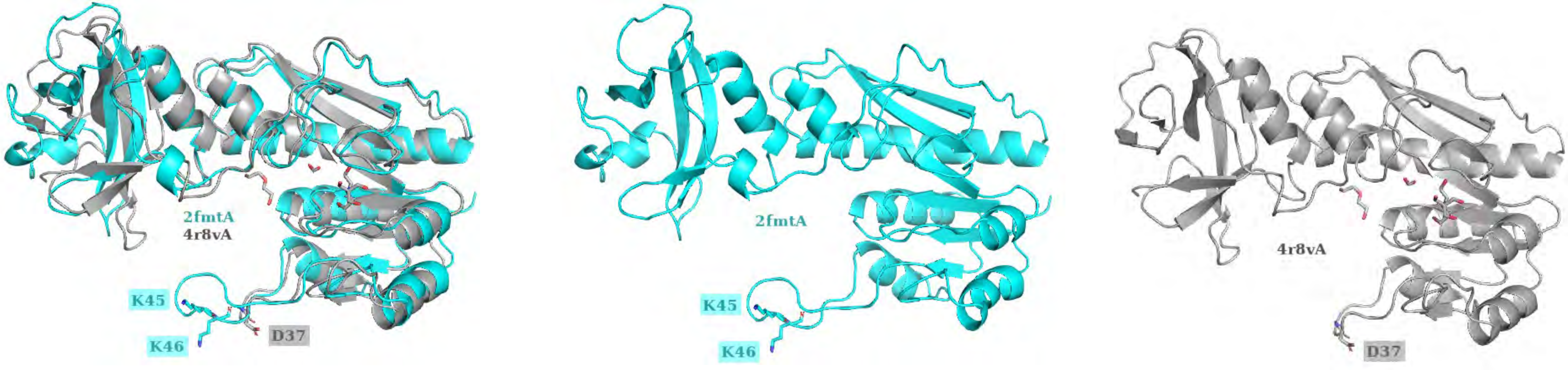

UniProt ID: E3NZ06

PDB ID: 4TS4_A

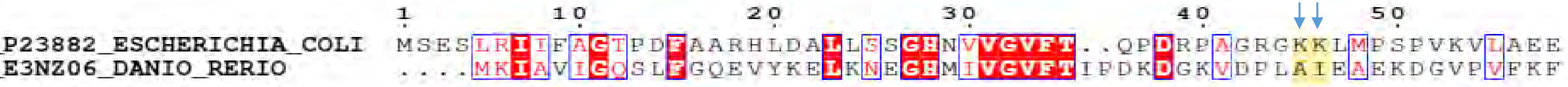

Full sequences in supplemental file.

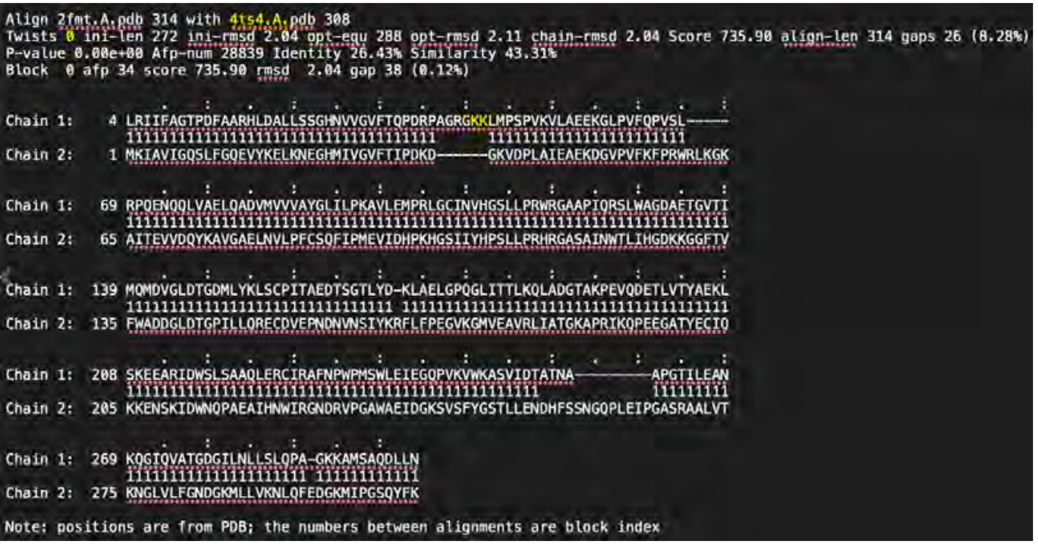

```
Align 2fmt.A.pdb 314 with 4ts4.A.pdb 308
Twists 0 ini-len 272 ini-rmsd 2.04 opt-equ 288 opt-rmsd 2.11 chain-rmsd 2.04 Score 735.90 align-len 314 gaps 26 (8.28%)
P-value 0.00e+00 Afp-num 28839 Identity 26.43% Similarity 43.31%
Block  0 afp 34 score 735.90 rmsd  2.04 gap 38 (0.12%)

                 .    :    .    :    .    :    .    :    .    :    .    :    .    :
Chain 1:    4 LRIIFAGTPDFAARHLDALLSSGHNVVGVFTQPDRPAGRGKKLMPSPVKVLAEEKGLPVFQPVSL------
              111111111111111111111111111111111111      111111111111111111111111
Chain 2:    1 MKIAVIGQSLFGQEVYKELKNEGHMIVGVFTIPDKD------GKVDPLAIEAEKDGVPVFKFPRWRLKGK

                 .    :    .    :    .    :    .    :    .    :    .    :    .    :
Chain 1:   69 RPQENQQLVAELQADVMVVVAYGLILPKAVLEMPRLGCINVHGSLLPRWRGAAPIQRSLWAGDAETGVTI
              1111111111111111111111111111111111111111111111111111111111111111111111
Chain 2:   65 AITEVVDQYKAVGAELNVLPFCSQFIPMEVIDHPKHGSIIYHPSLLPRHRGASAINWTLIHGDKKGGFTV

                 .    :    .    :    .    :    .    :    .    :    .    :    .    :
Chain 1:  139 MQMDVGLDTGDMLYKLSCPITAEDTSGTLYD-KLAELGPQGLITTLKQLADGTAKPEVQDETLVTYAEKL
              1111111111111111111111111111111 11111111111111111111111111111111111111
Chain 2:  135 FWADDGLDTGPILLQRECDVEPNDNVNSIYKRFLFPEGVKGMVEAVRLIATGKAPRIKQPEEGATYECIQ

                 .    :    .    :    .    :    .    :    .    :    .    :    .    :
Chain 1:  208 SKEEARIDWSLSAAQLERCIRAFNPWPMSWLEIEGQPVKVWKASVIDTATNA---------APGTILEAN
              11111111111111111111111111111111111111111111111111             111111111
Chain 2:  205 KKENSKIDWNQPAEAIHNWIRGNDRVPGAWAEIDGKSVSFYGSTLLENDHFSSNGQPLEIPGASRAALVT

                 .    :    .    :    .    :
Chain 1:  269 KQGIQVATGDGILNLLSLQPA-GKKAMSAQDLLN
              111111111111111111111 111111111111
Chain 2:  275 KNGLVLFGNDGKMLLVKNLQFEDGKMIPGSQYFK

Note: positions are from PDB; the numbers between alignments are block index
```

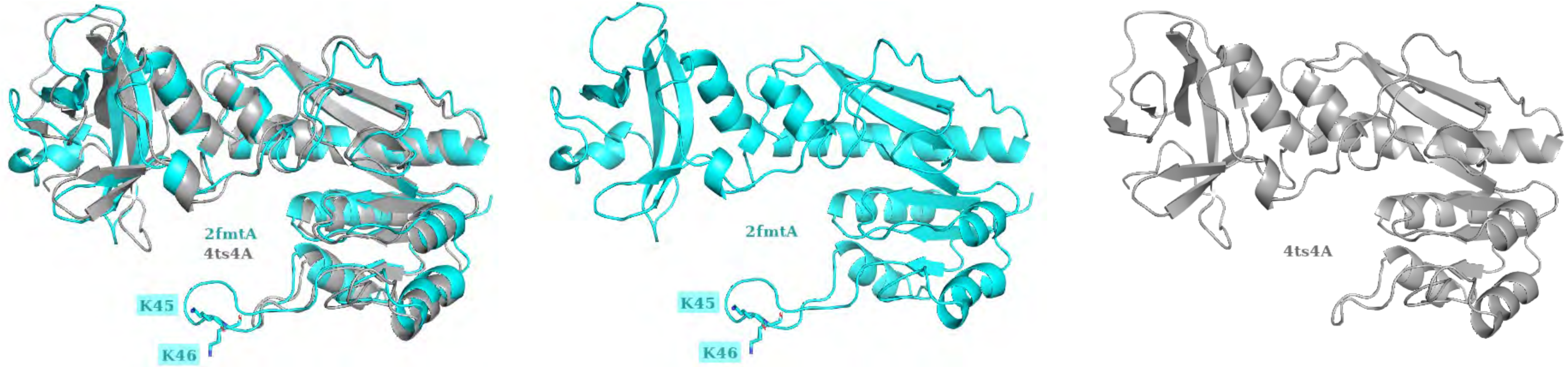

UniProt ID: E3NZ06

PDB ID: 4TT8_A

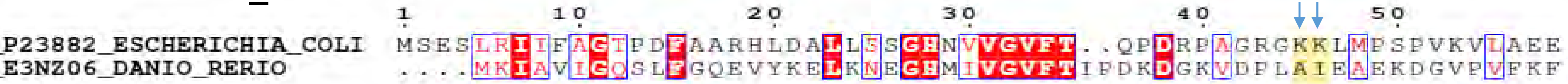

Full sequences in supplemental file.

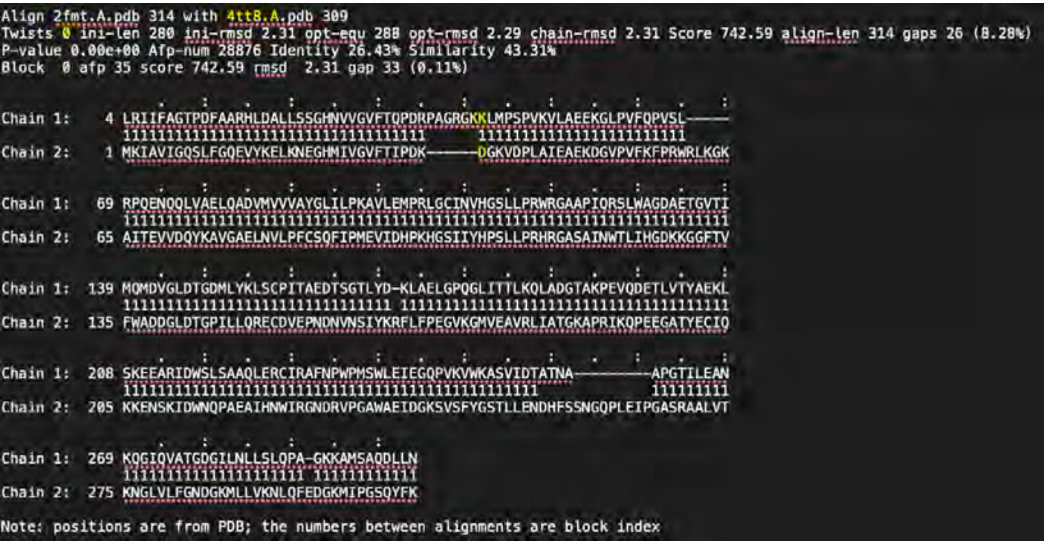

```
Align 2fmt.A.pdb 314 with 4tt8.A.pdb 309
Twists 0 ini-len 280 ini-rmsd 2.31 opt-equ 288 opt-rmsd 2.29 chain-rmsd 2.31 Score 742.59 align-len 314 gaps 26 (8.28%)
P-value 0.00e+00 Afp-num 28876 Identity 26.43% Similarity 43.31%
Block  0 afp 35 score 742.59 rmsd  2.31 gap 33 (0.11%)

Chain 1:    4 LRIIFAGTPDFAARHLDALLSSGHNVVGVFTQPDRPAGRGKKLMPSPVKVLAEEKGLPVFQPVSL------
              1111111111111111111111111111111111        1111111111111111111111111
Chain 2:    1 MKIAVIGQSLFGQEVYKELKNEGHMIVGVFTIPDK--------DGKVDPLAIEAEKDGVPVFKFPRWRLKGK

Chain 1:   69 RPQENQQLVAELQADVMVVVAYGLILPKAVLEMPRLGCINVHGSLLPRWRGAAPIQRSLWAGDAETGVTI
              1111111111111111111111111111111111111111111111111111111111111111111111
Chain 2:   65 AITEVVDQYKAVGAELNVLPFCSQFIPMEVIDHPKHGSIIYHPSLLPRHRGASAINWTLIHGDKKGGFTV

Chain 1:  139 MQMDVGLDTGDMLYKLSCPITAEDTSGTLYD-KLAELGPQGLITTLKQLADGTAKPEVQDETLVTYAEKL
              1111111111111111111111111111111 11111111111111111111111111111111111111
Chain 2:  135 FWADDGLDTGPILLQRECDVEPNDNVNSIYKRFLFPEGVKGMVEAVRLIATGKAPRIKQPEEGATYECIQ

Chain 1:  208 SKEEARIDWSLSAAQLERCIRAFNPWPMSWLEIEGQPVKVWKASVIDTATNA---------APGTILEAN
              111111111111111111111111111111111111111111111111111         111111111
Chain 2:  205 KKENSKIDWNQPAEAIHNWIRGNDRVPGAWAEIDGKSVSFYGSTLLENDHFSSNGQPLEIPGASRAALVT

Chain 1:  269 KQGIQVATGDGILNLLSLQPA-GKKAMSAQDLLN
              111111111111111111111 111111111111
Chain 2:  275 KNGLVLFGNDGKMLLVKNLQFEDGKMIPGSQYFK

Note: positions are from PDB; the numbers between alignments are block index
```

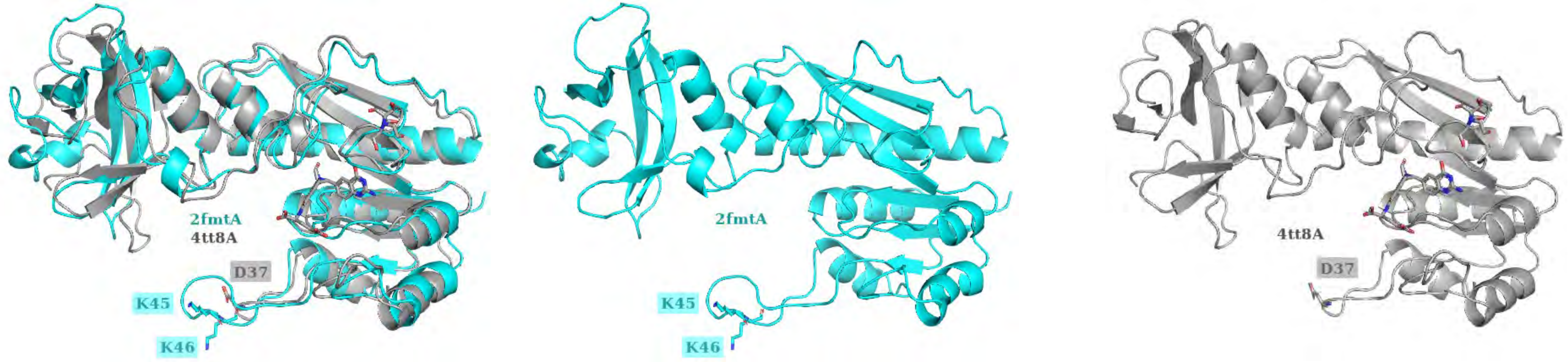

UniProt ID: E3NZ06

PDB ID: 4QPC_A

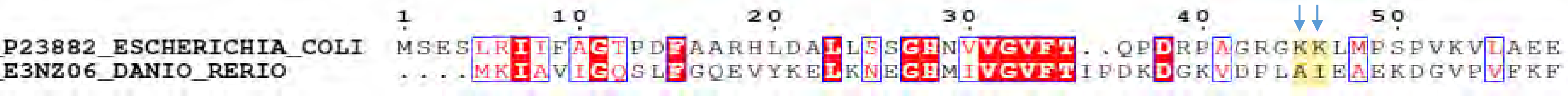

Full sequences in supplemental file.

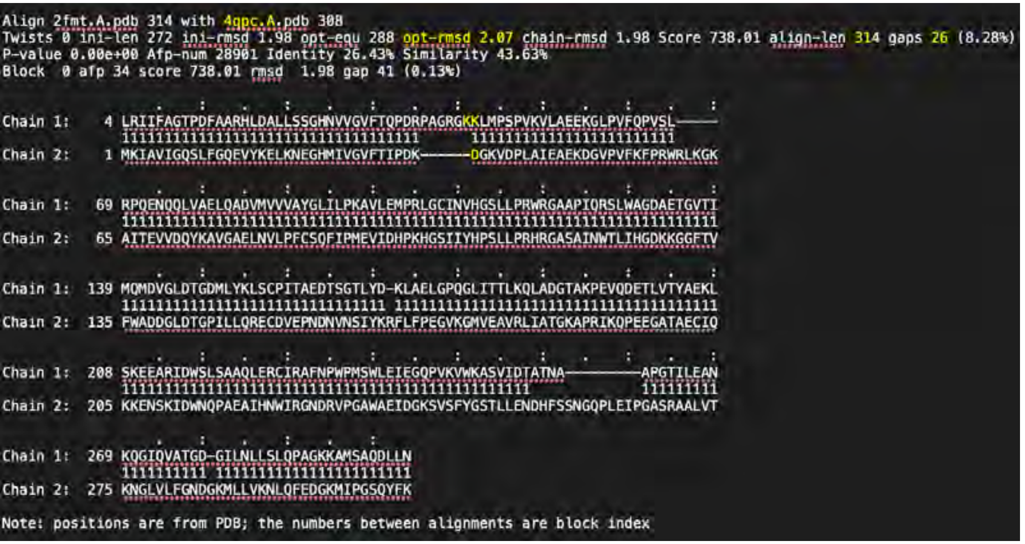

```
Align 2fmt.A.pdb 314 with 4qpc.A.pdb 308
Twists 0 ini-len 272 ini-rmsd 1.98 opt-equ 288 opt-rmsd 2.07 chain-rmsd 1.98 Score 738.01 align-len 314 gaps 26 (8.28%)
P-value 0.00e+00 Afp-num 28901 Identity 26.43% Similarity 43.63%
Block  0 afp 34 score 738.01 rmsd  1.98 gap 41 (0.13%)

Chain 1:    4 LRIIFAGTPDFAARHLDALLSSGHNVVGVFTQPDRPAGRGKKLMPSPVKVLAEEKGLPVFQPVSL------
                11111111111111111111111111111111111      1111111111111111111111111
Chain 2:    1 MKIAVIGQSLFGQEVYKELKNEGHMIVGVFTIPDK------DGKVDPLAIEAEKDGVPVFKFPRWRLKGK

Chain 1:   69 RPQENQQLVAELQADVMVVVAYGLILPKAVLEMPRLGCINVHGSLLPRWRGAAPIQRSLWAGDAETGVTI
                1111111111111111111111111111111111111111111111111111111111111111111111
Chain 2:   65 AITEVVDQYKAVGAELNVLPFCSQFIPMEVIDHPKHGSIIYHPSLLPRHRGASAINWTLIHGDKKGGFTV

Chain 1:  139 MQMDVGLDTGDMLYKLSCPITAEDTSGTLYD-KLAELGPQGLITTLKQLADGTAKPEVQDETLVTYAEKL
                1111111111111111111111111111111 11111111111111111111111111111111111111
Chain 2:  135 FWADDGLDTGPILLQRECDVEPNDNVNSIYKRFLFPEGVKGMVEAVRLIATGKAPRIKQPEEGATAECIQ

Chain 1:  208 SKEEARIDWSLSAAQLERCIRAFNPWPMSWLEIEGQPVKVWKASVIDTATNA---------APGTILEAN
                111111111111111111111111111111111111111111111111          111111111
Chain 2:  205 KKENSKIDWNQPAEAIHNWIRGNDRVPGAWAEIDGKSVSFYGSTLLENDHFSSNGQPLEIPGASRAALVT

Chain 1:  269 KQGIQVATGD-GILNLLSLQPAGKKAMSAQDLLN
                1111111111 11111111111111111111111
Chain 2:  275 KNGLVLFGNDGKMLLVKNLQFEDGKMIPGSQYFK

Note: positions are from PDB; the numbers between alignments are block index
```

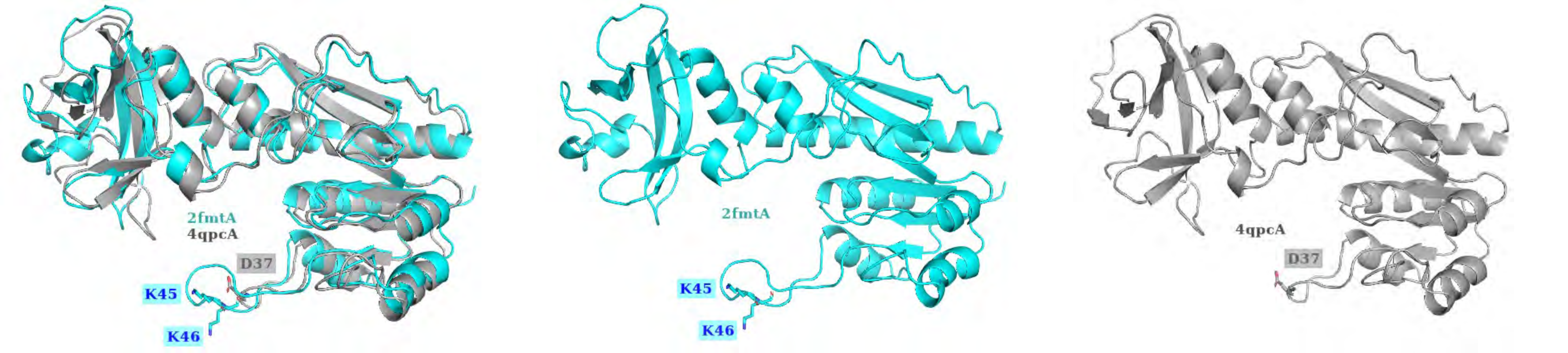

UniProt ID: E3NZ06

PDB ID: 4TTS_A

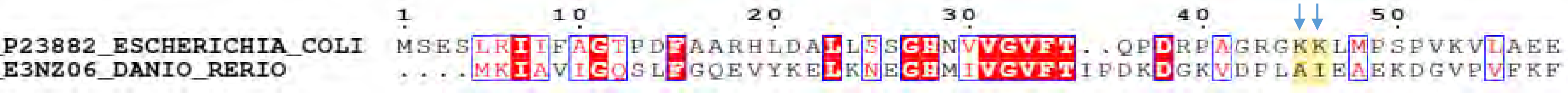

Full sequences in supplemental file.

```
Align 2fmt.A.pdb 314 with 4tts.A.pdb 309
Twists 0 ini-len 272 ini-rmsd 2.06 opt-equ 288 opt-rmsd 2.12 chain-rmsd 2.06 Score 737.12 align-len 314 gaps 26 (8.28%)
P-value 0.00e+00 Afp-num 29079 Identity 26.43% Similarity 43.63%
Block  0 afp 34 score 737.12 rmsd  2.06 gap 39 (0.13%)

                  .    :    .    :    .    :    .    :    .    :    .    :    .    :
Chain 1:    4 LRIIFAGTPDFAARHLDALLSSGHNVVGVFTQPDRPAGRGKKLMPSPVKVLAEEKGLPVFQPVSL------
              111111111111111111111111111111111111      1111111111111111111111
Chain 2:    1 MKIAVIGQSLFGQEVYKELKNEGHMIVGVFTIPDKD------GKVDPLAIEAEKDGVPVFKFPRWRLKGK

                  .    :    .    :    .    :    .    :    .    :    .    :    .    :
Chain 1:   69 RPQENQQLVAELQADVMVVVAYGLILPKAVLEMPRLGCINVHGSLLPRWRGAAPIQRSLWAGDAETGVTI
              1111111111111111111111111111111111111111111111111111111111111111111111
Chain 2:   65 AITEVVDQYKAVGAELNVLPFCSQFIPMEVIDHPKHGSIIYHPSLLPRHRGASAINWTLIHGDKKGGFTV

                  .    :    .    :    .    :    .    :    .    :    .    :    .    :
Chain 1:  139 MQMDVGLDTGDMLYKLSCPITAEDTSGTLYD-KLAELGPQGLITTLKQLADGTAKPEVQDETLVTYAEKL
              1111111111111111111111111111111 11111111111111111111111111111111111111
Chain 2:  135 FWADDGLDTGPILLQRECDVEPNDNVNSIYKRFLFPEGVKGMVEAVRLIATGKAPRIKQPEEGATAECIQ

                  .    :    .    :    .    :    .    :    .    :    .    :    .    :
Chain 1:  208 SKEEARIDWSLSAAQLERCIRAFNPWPMSWLEIEGQPVKVWKASVIDTATNA---------APGTILEAN
              111111111111111111111111111111111111111111111111111         111111111
Chain 2:  205 KKENSKIDWNQPAEAIHNWIRGNDRVPGAWAEIDGKSVSFYGSTLLENDHFSSNGQPLEIPGASRAALVT

                  .    :    .    :    .    :
Chain 1:  269 KQGIQVATGD-GILNLLSLQPAGKKAMSAQDLLN
              1111111111 11111111111111111111111
Chain 2:  275 KNGLVLFGNDGKMLLVKNLQFEDGKMIPGSQYFK

Note: positions are from PDB; the numbers between alignments are block index
```

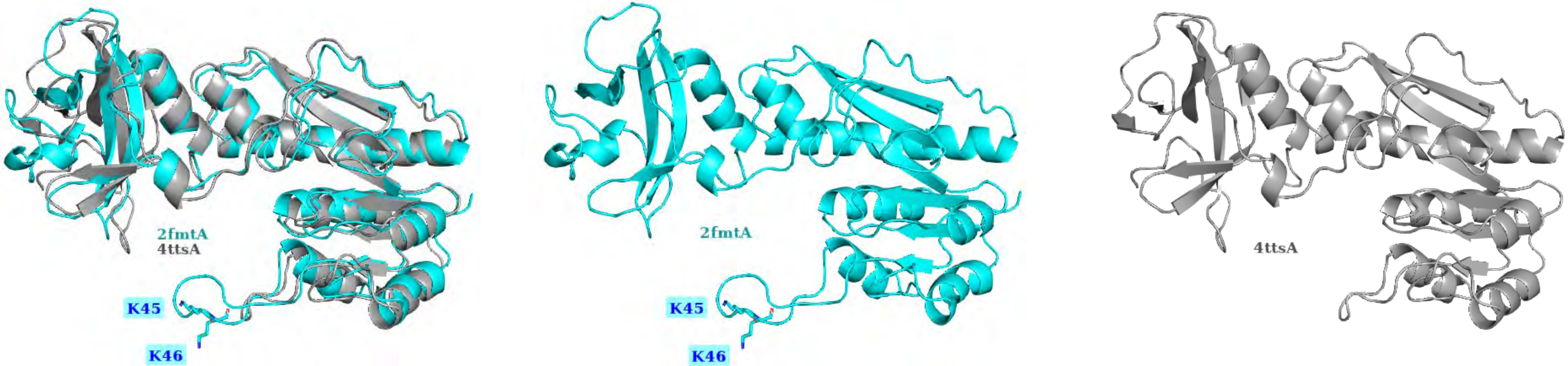

UniProt ID: O75891

PDB ID: 2BW0_A

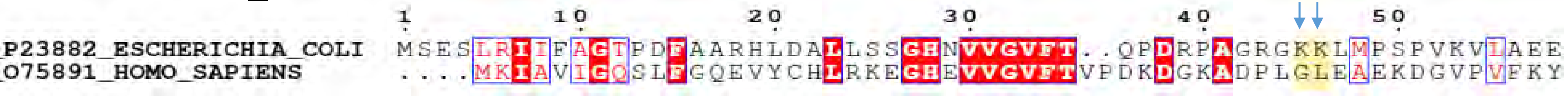

Full sequences in supplemental file.

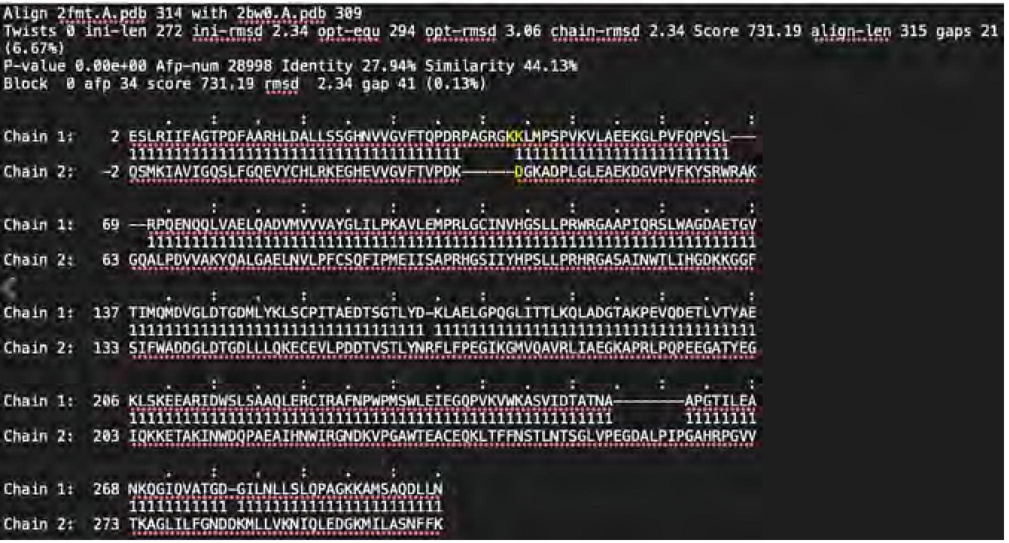

```
Align 2fmt.A.pdb 314 with 2bw0.A.pdb 309
Twists 0 ini-len 272 ini-rmsd 2.34 opt-equ 294 opt-rmsd 3.06 chain-rmsd 2.34 Score 731.19 align-len 315 gaps 21
(6.67%)
P-value 0.00e+00 Afp-num 28998 Identity 27.94% Similarity 44.13%
Block  0 afp 34 score 731.19 rmsd  2.34 gap 41 (0.13%)

                 .    :    .    :    .    :    .    :    .    :    .    :    .    :
Chain 1:    2 ESLRIIFAGTPDFAARHLDALLSSGHNVVGVFTQPDRPAGRGKKLMPSPVKVLAEEKGLPVFQPVSL--
              1111111111111111111111111111111111111      1111111111111111111111111
Chain 2:   -2 QSMKIAVIGQSLFGQEVYCHLRKEGHEVVGVFTVPDK------DGKADPLGLEAEKDGVPVFKYSRWRAK

                 .    :    .    :    .    :    .    :    .    :    .    :    .    :
Chain 1:   69 --RPQENQQLVAELQADVMVVVAYGLILPKAVLEMPRLGCINVHGSLLPRWRGAAPIQRSLWAGDAETGV
                1111111111111111111111111111111111111111111111111111111111111111111
Chain 2:   63 GQALPDVVAKYQALGAELNVLPFCSQFIPMEIISAPRHGSIIYHPSLLPRHRGASAINWTLIHGDKKGGF

                 .    :    .    :    .    :    .    :    .    :    .    :    .    :
Chain 1:  137 TIMQMDVGLDTGDMLYKLSCPITAEDTSGTLYD-KLAELGPQGLITTLKQLADGTAKPEVQDETLVTYAE
              111111111111111111111111111111111 111111111111111111111111111111111111
Chain 2:  133 SIFWADDGLDTGDLLLQKECEVLPDDTVSTLYNRFLFPEGIKGMVQAVRLIAEGKAPRLPQPEEGATYEG

                 .    :    .    :    .    :    .    :    .    :    .    :    .    :
Chain 1:  206 KLSKEEARIDWSLSAAQLERCIRAFNPWPMSWLEIEGQPVKVWKASVIDTATNA--------APGTILEA
              11111111111111111111111111111111111111111111111111111        11111111
Chain 2:  203 IQKKETAKINWDQPAEAIHNWIRGNDKVPGAWTEACEQKLTFFNSTLNTSGLVPEGDALPIPGAHRPGVV

                 .    :    .    :    .    :    .
Chain 1:  268 NKQGIQVATGD-GILNLLSLQPAGKKAMSAQDLLN
              11111111111 11111111111111111111111
Chain 2:  273 TKAGLILFGNDDKMLLVKNIQLEDGKMILASNFFK
```

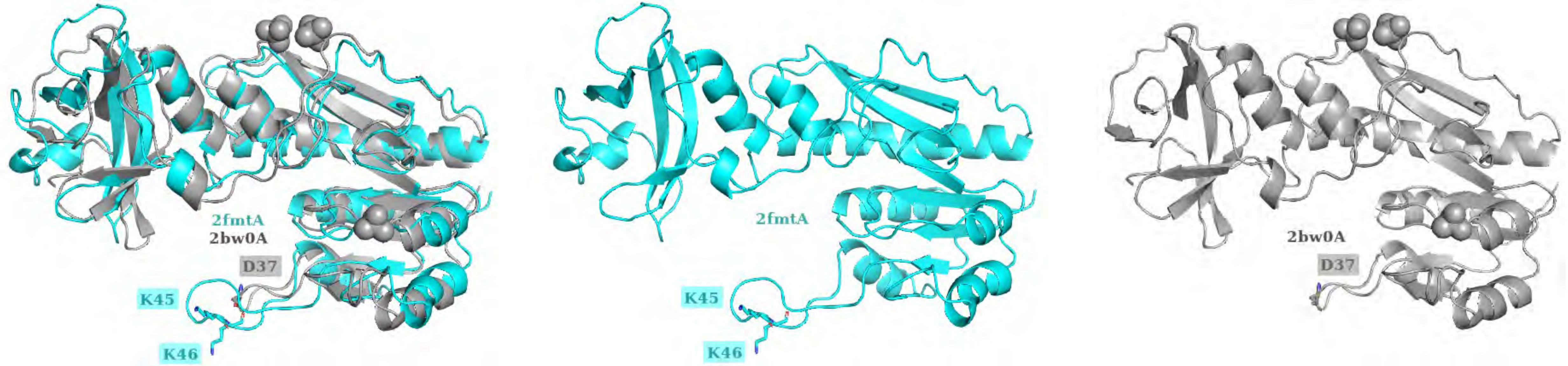

UniProt ID: O75891

PDB ID: 2CFI_A

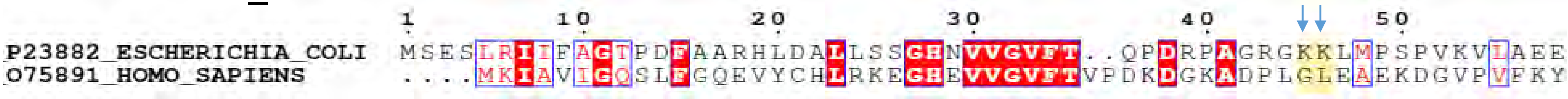

Full sequences in supplemental file.

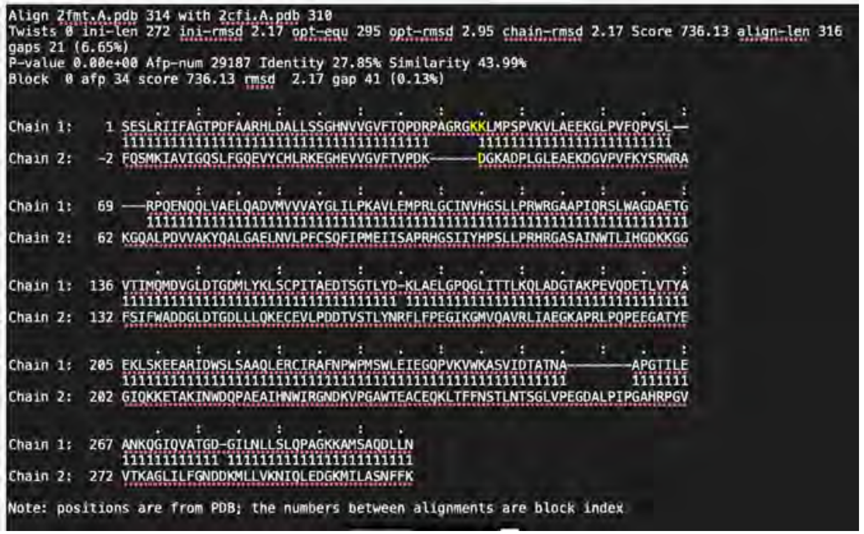

```
Align 2fmt.A.pdb 314 with 2cfi.A.pdb 310
Twists 0 ini-len 272 ini-rmsd 2.17 opt-equ 295 opt-rmsd 2.95 chain-rmsd 2.17 Score 736.13 align-len 316
gaps 21 (6.65%)
P-value 0.00e+00 Afp-num 29187 Identity 27.85% Similarity 43.99%
Block  0 afp 34 score 736.13 rmsd  2.17 gap 41 (0.13%)

Chain 1:    1 SESLRIIFAGTPDFAARHLDALLSSGHNVVGVFTQPDRPAGRGKKLMPSPVKVLAEEKGLPVFQPVSL--
Chain 2:   -2 FQSMKIAVIGQSLFGQEVYCHLRKEGHEVVGVFTVPDK------DGKADPLGLEAEKDGVPVFKYSRWRA

Chain 1:   69 --RPQENQQLVAELQADVMVVVAYGLILPKAVLEMPRLGCINVHGSLLPRWRGAAPIQRSLWAGDAETG
Chain 2:   62 KGQALPDVVAKYQALGAELNVLPFCSQFIPMEIISAPRHGSIIYHPSLLPRHRGASAINWTLIHGDKKGG

Chain 1:  136 VTIMQMDVGLDTGDMLYKLSCPITAEDTSGTLYD-KLAELGPQGLITTLKQLADGTAKPEVQDETLVTYA
Chain 2:  132 FSIFWADDGLDTGDLLLQKECEVLPDDTVSTLYNRFLFPEGIKGMVQAVRLIAEGKAPRLPQPEEGATYE

Chain 1:  205 EKLSKEEARIDWSLSAAQLERCIRAFNPWPMSWLEIEGQPVKVWKASVIDTATNA--------APGTILE
Chain 2:  202 GIQKKETAKINWDQPAEAIHNWIRGNDKVPGAWTEACEQKLTFFNSTLNTSGLVPEGDALPIPGAHRPGV

Chain 1:  267 ANKQGIQVATGD-GILNLLSLQPAGKKAMSAQDLLN
Chain 2:  272 VTKAGLILFGNDDKMLLVKNIQLEDGKMILASNFFK

Note: positions are from PDB; the numbers between alignments are block index
```

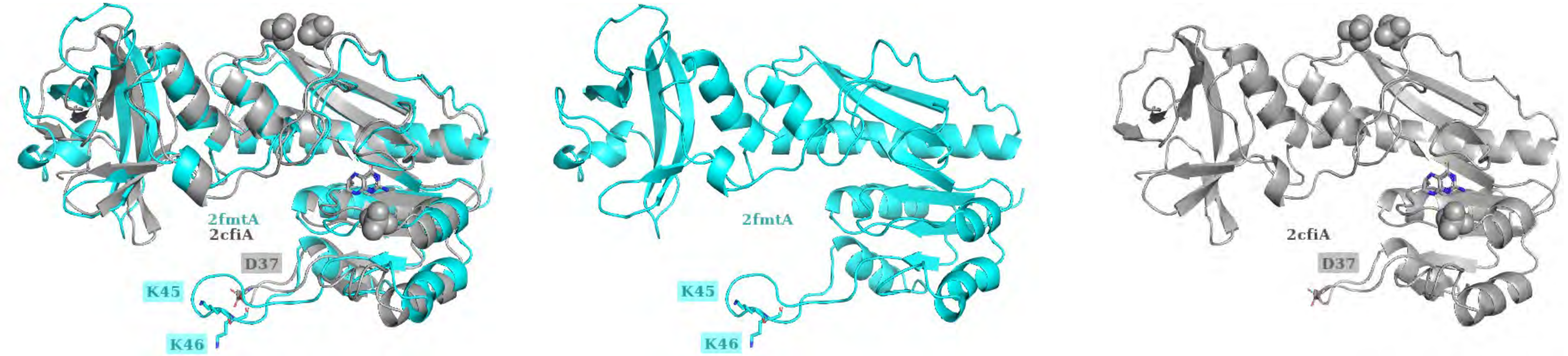

UniProt ID: O85732

PDB ID: 5UAI_C

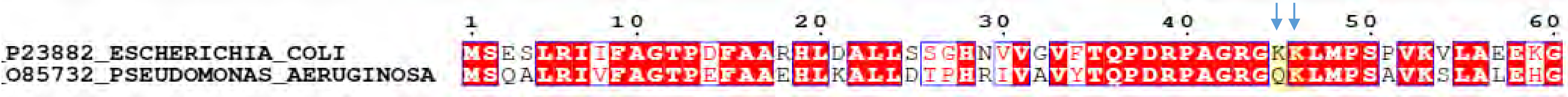

Full sequences in supplemental file.

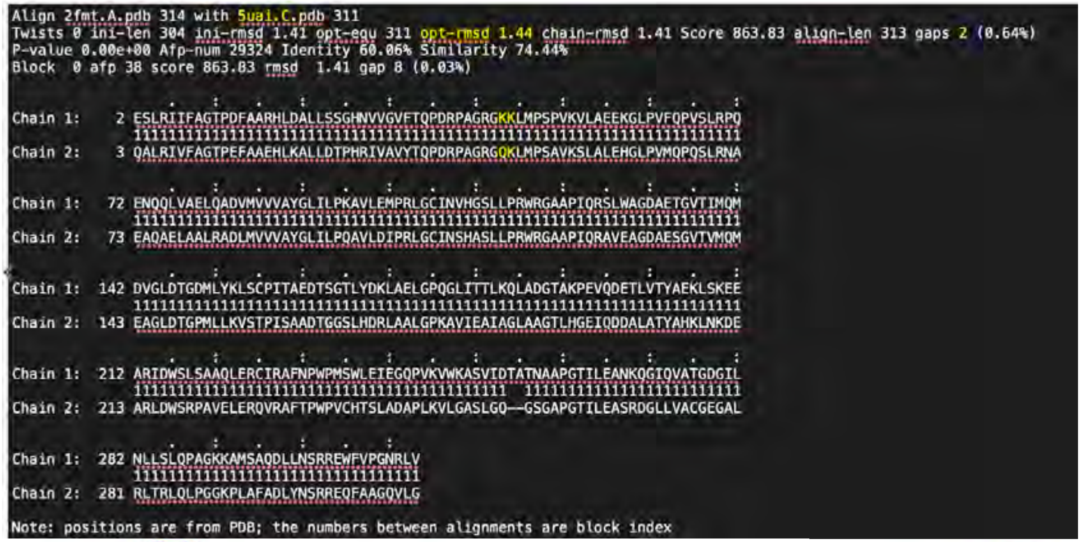

```
Align 2fmt.A.pdb 314 with 5uai.C.pdb 311
Twists 0 ini-len 304 ini-rmsd 1.41 opt-equ 311 opt-rmsd 1.44 chain-rmsd 1.41 Score 863.83 align-len 313 gaps 2 (0.64%)
P-value 0.00e+00 Afp-num 29324 Identity 60.06% Similarity 74.44%
Block  0 afp 38 score 863.83 rmsd  1.41 gap 8 (0.03%)

                .    :    .    :    .    :    .    :    .    :    .    :    .    :
Chain 1:    2 ESLRIIFAGTPDFAARHLDALLSSGHNVVGVFTQPDRPAGRGKKLMPSPVKVLAEEKGLPVFQPVSLRPQ
              111111111111111111111111111111111111111111111111111111111111111111111111
Chain 2:    3 QALRIVFAGTPEFAAEHLKALLDTPHRIVAVYTQPDRPAGRGQKLMPSAVKSLALEHGLPVMQPQSLRNA

                .    :    .    :    .    :    .    :    .    :    .    :    .    :
Chain 1:   72 ENQQLVAELQADVMVVVAYGLILPKAVLEMPRLGCINVHGSLLPRWRGAAPIQRSLWAGDAETGVTIMQM
              111111111111111111111111111111111111111111111111111111111111111111111111
Chain 2:   73 EAQAELAALRADLMVVVAYGLILPQAVLDIPRLGCINSHASLLPRWRGAAPIQRAVEAGDAESGVTVMQM

                .    :    .    :    .    :    .    :    .    :    .    :    .    :
Chain 1:  142 DVGLDTGDMLYKLSCPITAEDTSGTLYDKLAELGPQGLITTLKQLADGTAKPEVQDETLVTYAEKLSKEE
              111111111111111111111111111111111111111111111111111111111111111111111111
Chain 2:  143 EAGLDTGPMLLKVSTPISAADTGGSLHDRLAALGPKAVIEAIAGLAAGTLHGEIQDDALATYAHKLNKDE

                .    :    .    :    .    :    .    :    .    :    .    :    .    :
Chain 1:  212 ARIDWSLSAAQLERCIRAFNPWPMSWLEIEGQPVKVWKASVIDTATNAAPGTILEANKQGIQVATGDGIL
              1111111111111111111111111111111111111111111  111111111111111111111111
Chain 2:  213 ARLDWSRPAVELERQVRAFTPWPVCHTSLADAPLKVLGASLGQ--GSGAPGTILEASRDGLLVACGEGAL

                .    :    .    :    .    :
Chain 1:  282 NLLSLQPAGKKAMSAQDLLNSRREWFVPGNRLV
              111111111111111111111111111111111
Chain 2:  281 RLTRLQLPGGKPLAFADLYNSRREQFAAGQVLG

Note: positions are from PDB; the numbers between alignments are block index
```

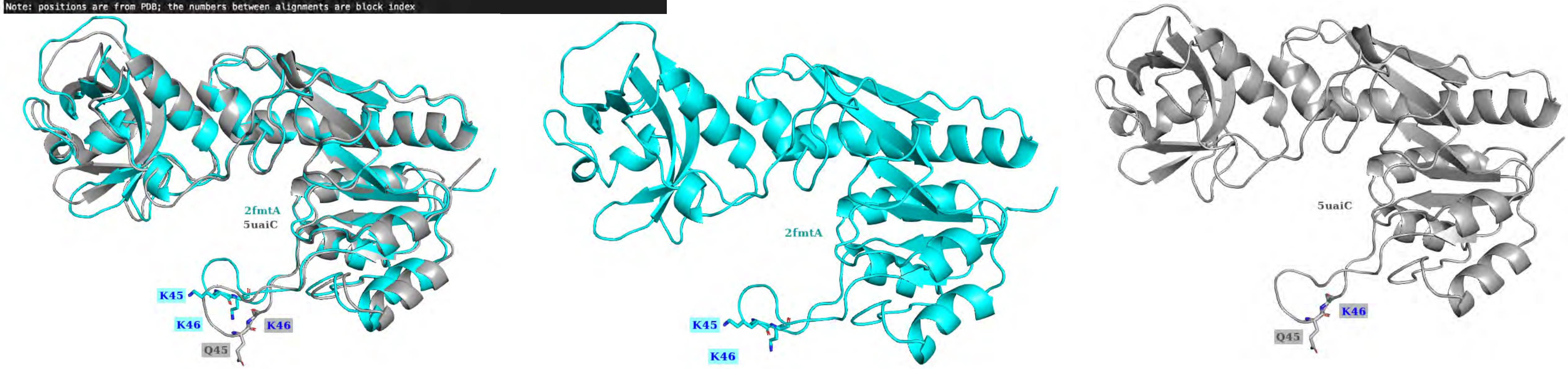

UniProt ID: P28037

PDB ID: 1S3I_A

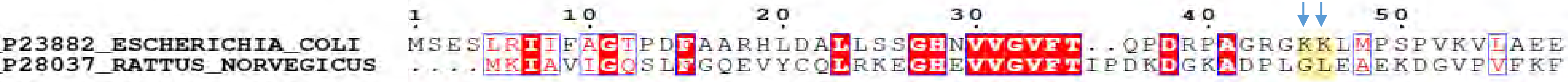

Full sequences in supplemental file.

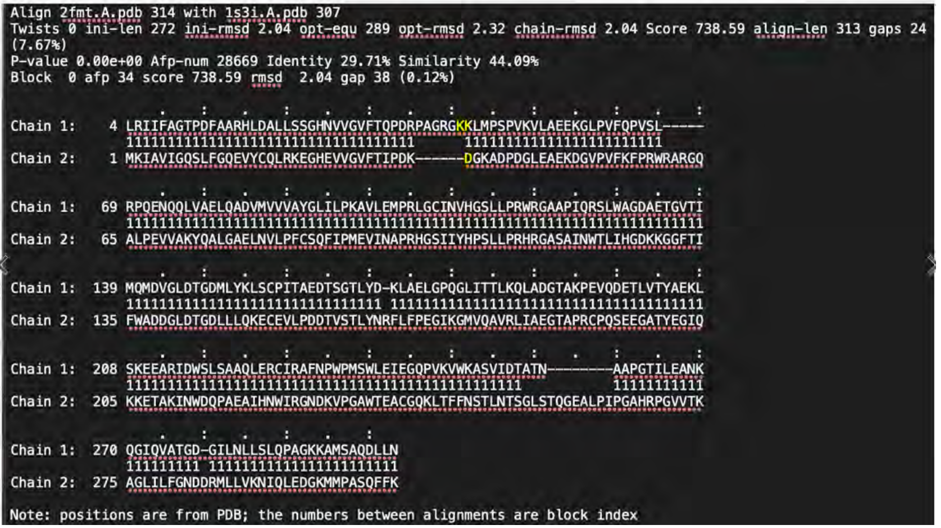

```
Align 2fmt.A.pdb 314 with 1s3i.A.pdb 307
Twists 0 ini-len 272 ini-rmsd 2.04 opt-equ 289 opt-rmsd 2.32 chain-rmsd 2.04 Score 738.59 align-len 313 gaps 24
(7.67%)
P-value 0.00e+00 Afp-num 28669 Identity 29.71% Similarity 44.09%
Block  0 afp 34 score 738.59 rmsd  2.04 gap 38 (0.12%)

                  .    :    .    :    .    :    .    :    .    :    .    :    .    :
Chain 1:    4 LRIIFAGTPDFAARHLDALLSSGHNVVGVFTQPDRPAGRGKKLMPSPVKVLAEEKGLPVFQPVSL-----
              11111111111111111111111111111111111      111111111111111111111111
Chain 2:    1 MKIAVIGQSLFGQEVYCQLRKEGHEVVGVFTIPDK------DGKADPDGLEAEKDGVPVFKFPRWRARGQ

                  .    :    .    :    .    :    .    :    .    :    .    :    .    :
Chain 1:   69 RPQENQQLVAELQADVMVVVAYGLILPKAVLEMPRLGCINVHGSLLPRWRGAAPIQRSLWAGDAETGVTI
              1111111111111111111111111111111111111111111111111111111111111111111111
Chain 2:   65 ALPEVVAKYQALGAELNVLPFCSQFIPMEVINAPRHGSIIYHPSLLPRHRGASAINWTLIHGDKKGGFTI

                  .    :    .    :    .    :    .    :    .    :    .    :    .    :
Chain 1:  139 MQMDVGLDTGDMLYKLSCPITAEDTSGTLYD-KLAELGPQGLITTLKQLADGTAKPEVQDETLVTYAEKL
              1111111111111111111111111111111 11111111111111111111111111111111111111
Chain 2:  135 FWADDGLDTGDLLLQKECEVLPDDTVSTLYNRFLFPEGIKGMVQAVRLIAEGTAPRCPQSEEGATYEGIQ

                  .    :    .    :    .    :    .    :    .    :    .    :    .    :
Chain 1:  208 SKEEARIDWSLSAAQLERCIRAFNPWPMSWLEIEGQPVKVWKASVIDTATN--------AAPGTILEANK
              111111111111111111111111111111111111111111111111111        1111111111
Chain 2:  205 KKETAKINWDQPAEAIHNWIRGNDKVPGAWTEACGQKLTFFNSTLNTSGLSTQGEALPIPGAHRPGVVTK

                  .    :    .    :    .    :
Chain 1:  270 QGIQVATGD-GILNLLSLQPAGKKAMSAQDLLN
              111111111 11111111111111111111111
Chain 2:  275 AGLILFGNDDRMLLVKNIQLEDGKMMPASQFFK

Note: positions are from PDB; the numbers between alignments are block index
```

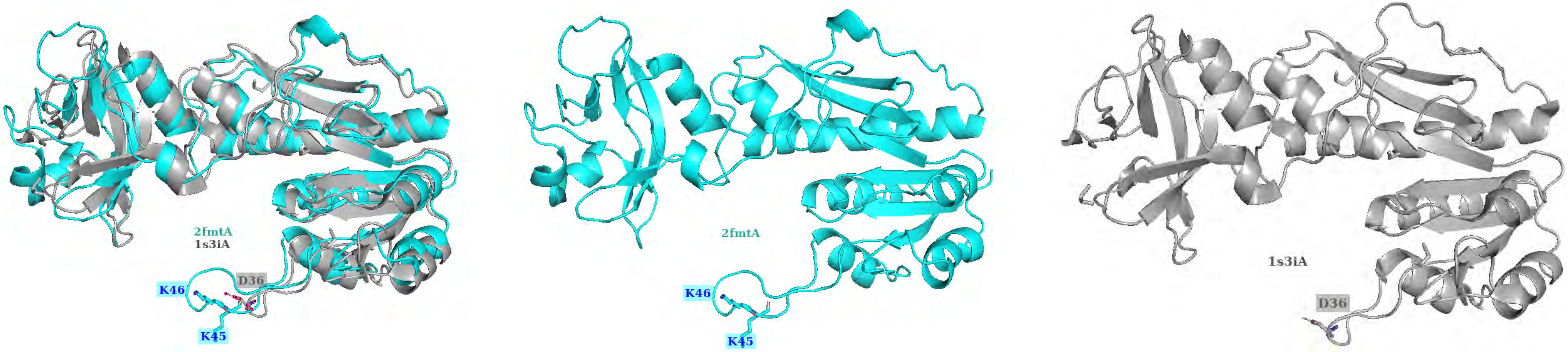

UniProt ID: P9WKZ3

PDB ID: 4PZU_D

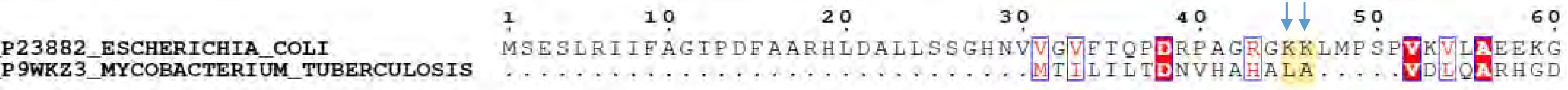

Full sequences in supplemental file.

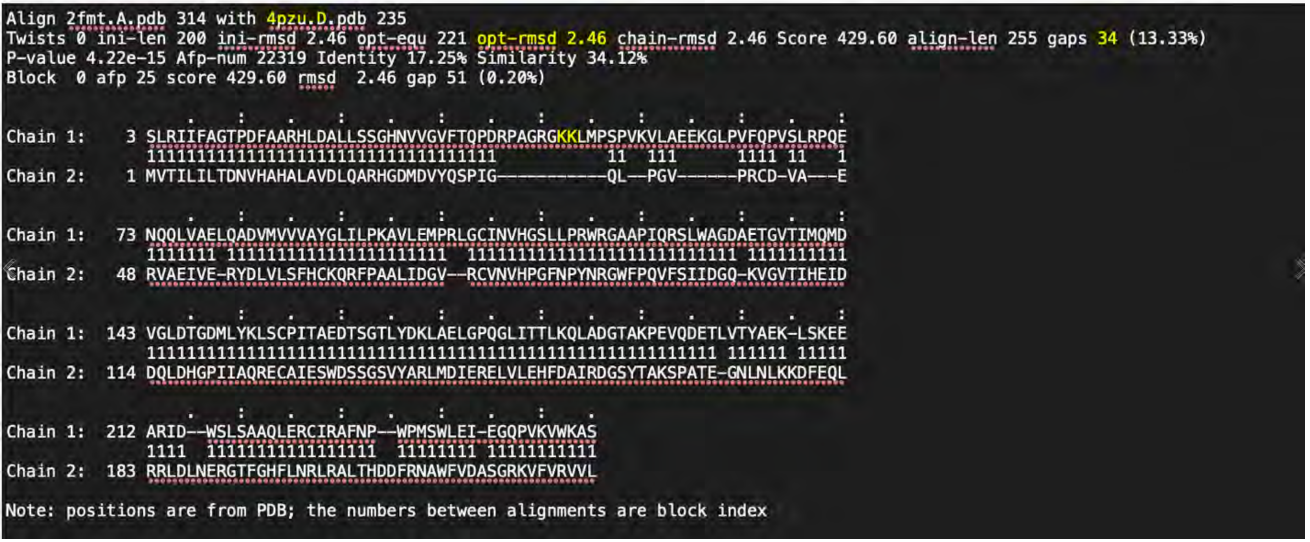

```
Align 2fmt.A.pdb 314 with 4pzu.D.pdb 235
Twists 0 ini-len 200 ini-rmsd 2.46 opt-equ 221 opt-rmsd 2.46 chain-rmsd 2.46 Score 429.60 align-len 255 gaps 34 (13.33%)
P-value 4.22e-15 Afp-num 22319 Identity 17.25% Similarity 34.12%
Block  0 afp 25 score 429.60 rmsd  2.46 gap 51 (0.20%)

                    .    :    .    :    .    :    .    :    .    :    .    :    .    :
Chain 1:    3 SLRIIFAGTPDFAARHLDALLSSGHNVVGVFTQPDRPAGRGKKLMPSPVKVLAEEKGLPVFQPVSLRPQE
              11111111111111111111111111111111111           11  111      1111 11   1
Chain 2:    1 MVTILILTDNVHAHALAVDLQARHGDMDVYQSPIG-----------QL--PGV------PRCD-VA---E

                    .    :    .    :    .    :    .    :    .    :    .    :    .    :
Chain 1:   73 NQQLVAELQADVMVVVAYGLILPKAVLEMPRLGCINVHGSLLPRWRGAAPIQRSLWAGDAETGVTIMQMD
              1111111 1111111111111111111111  11111111111111111111111111 1111111111
Chain 2:   48 RVAEIVE-RYDLVLSFHCKQRFPAALIDGV--RCVNVHPGFNPYNRGWFPQVFSIIDGQ-KVGVTIHEID

                    .    :    .    :    .    :    .    :    .    :    .    :    .    :
Chain 1:  143 VGLDTGDMLYKLSCPITAEDTSGTLYDKLAELGPQGLITTLKQLADGTAKPEVQDETLVTYAEK-LSKEE
              1111111111111111111111111111111111111111111111111111111 111111 11111
Chain 2:  114 DQLDHGPIIAQRECAIESWDSSGSVYARLMDIERELVLEHFDAIRDGSYTAKSPATE-GNLNLKKDFEQL

                    .    :    .    :    .    :    .    :    .
Chain 1:  212 ARID--WSLSAAQLERCIRAFNP--WPMSWLEI-EGQPVKVWKAS
              1111  11111111111111111  11111111 1111111111
Chain 2:  183 RRLDLNERGTFGHFLNRLRALTHDDFRNAWFVDASGRKVFVRVVL

Note: positions are from PDB; the numbers between alignments are block index
```

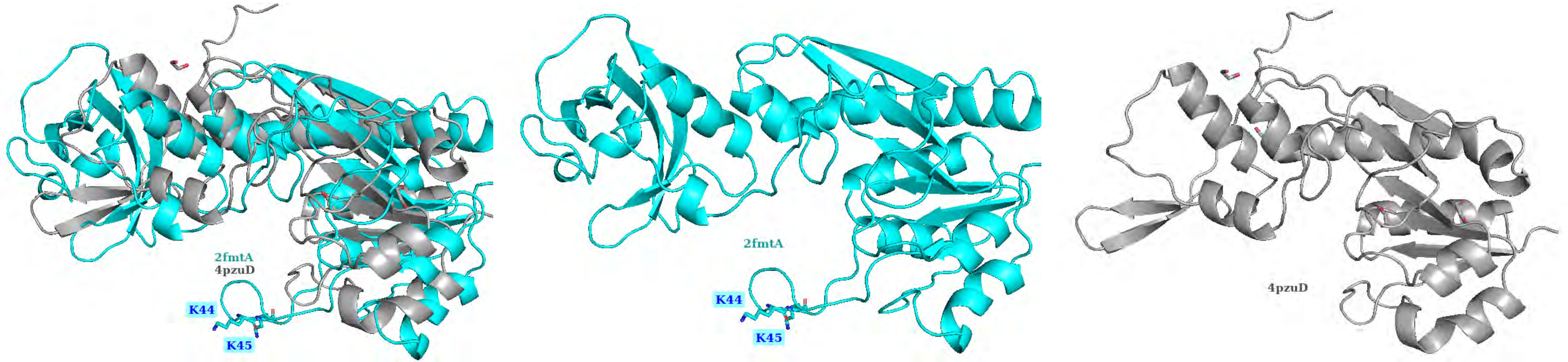

UniProt ID: P9WKZ3

PDB ID: 4Q12_B

```
                                   1       10        20        30        40        50        60
P23882_ESCHERICHIA_COLI            MSESLRIIFAGTPDFAARHLDALLSSGHNVVGVFTQPDRPAGRGKKLMPSPVKVLAEEKG
P9WKZ3_MYCOBACTERIUM_TUBERCULOSIS  ..............................MTILILTDNVHAHALA.....VDLQARHGD
```

Full sequences in supplemental file.

```
Align 2fmt.A.pdb 314 with 4q12.B.pdb 232
Twists 0 ini-len 200 ini-rmsd 2.45 opt-equ 221 opt-rmsd 2.53 chain-rmsd 2.45 Score 432.56 align-len 255 gaps 34 (13.33%)
P-value 5.11e-15 Afp-num 22183 Identity 18.04% Similarity 34.90%
Block  0 afp 25 score 432.56 rmsd  2.45 gap 51 (0.20%)

                    .    :    .    :    .    :    .    :    .    :    .    :    .    :
Chain 1:    3 SLRIIFAGTPDFAARHLDALLSSGHNVVGVFTQPDRPAGRGKKLMPSPVKVLAEEKGLPVFQPVSLRPQE
              11111111111111111111111111111111111          11111        111111    1
Chain 2:    1 MVTILILTDNVHAHALAVDLQARHGDMDVYQSPIG-----------QLPGV--------PRCDVA----E

                    .    :    .    :    .    :    .    :    .    :    .    :    .    :
Chain 1:   73 NQQLVAELQADVMVVVAYGLILPKAVLEMPRLGCINVHGSLLPRWRGAAPIQRSLWAGDAETGVTIMQMD
              11111111 111111111111111111111  111111111111111111111111111 1111111111
Chain 2:   48 RVAEIVER-YDLVLSFHCKQRFPAALIDGV--RCVNVHPGFNPYNRGWFPQVFSIIDGQ-KVGVTIHEID

                    .    :    .    :    .    :    .    :    .    :    .    :    .    :
Chain 1:  143 VGLDTGDMLYKLSCPITAEDTSGTLYDKLAELGPQGLITTLKQLADGTAKPEVQDETLVTYAEK-LSKEE
              111111111111111111111111111111111111111111111111111111111 111111 11111
Chain 2:  114 DQLDHGPIIAQRECAIESWDSSGSVYARLMDIERELVLEHFDAIRDGSYTAKSPATE-GNLNLKKDFEQL

                    .    :    .    :    .    :    .    :    .
Chain 1:  212 ARID--WSLSAAQLERCIRAFNP--WPMSWLEI-EGQPVKVWKAS
              1111  11111111111111111  11111111 11111111111
Chain 2:  183 RRLDLNERGTFGHFLNRLRALTHDDFRNAWFVDASGRKVFVRVVL

Note: positions are from PDB; the numbers between alignments are block index
```

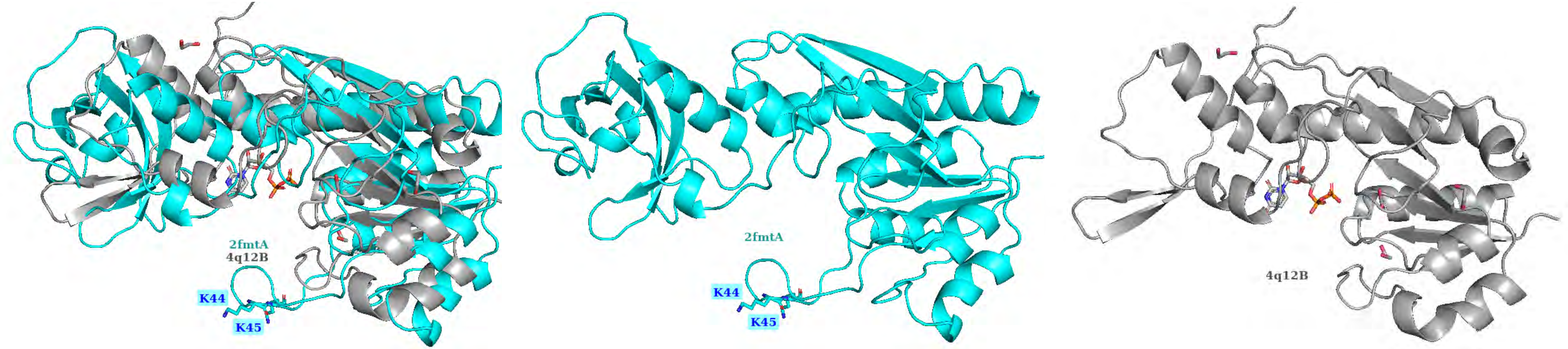

UniProt ID: R4MIX2

PDB ID: 5VYQ_B

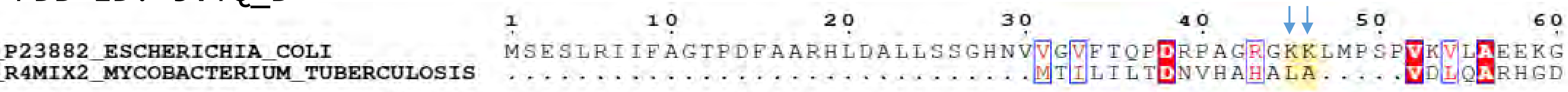

Full sequences in supplemental file.

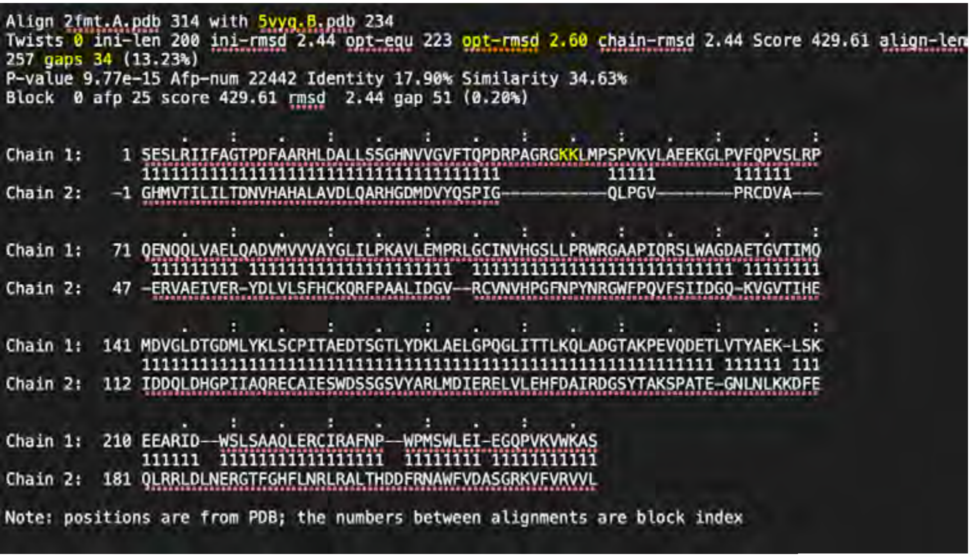

```
Align 2fmt.A.pdb 314 with 5vyq.B.pdb 234
Twists 0 ini-len 200 ini-rmsd 2.44 opt-equ 223 opt-rmsd 2.60 chain-rmsd 2.44 Score 429.61 align-len
257 gaps 34 (13.23%)
P-value 9.77e-15 Afp-num 22442 Identity 17.90% Similarity 34.63%
Block  0 afp 25 score 429.61 rmsd  2.44 gap 51 (0.20%)

                   .    :    .    :    .    :    .    :    .    :    .    :    .    :
Chain 1:     1 SESLRIIFAGTPDFAARHLDALLSSGHNVVGVFTQPDRPAGRGKKLMPSPVKVLAEEKGLPVFQPVSLRP
               1111111111111111111111111111111111111           11111        111111
Chain 2:    -1 GHMVTILILTDNVHAHALAVDLQARHGDMDVYQSPIG-----------QLPGV--------PRCDVA---

                   .    :    .    :    .    :    .    :    .    :    .    :    .    :
Chain 1:    71 QENQQLVAELQADVMVVVAYGLILPKAVLEMPRLGCINVHGSLLPRWRGAAPIQRSLWAGDAETGVTIMQ
                111111111 111111111111111111111  111111111111111111111111111 11111111
Chain 2:    47 -ERVAEIVER-YDLVLSFHCKQRFPAALIDGV--RCVNVHPGFNPYNRGWFPQVFSIIDGQ-KVGVTIHE

                   .    :    .    :    .    :    .    :    .    :    .    :    .    :
Chain 1:   141 MDVGLDTGDMLYKLSCPITAEDTSGTLYDKLAELGPQGLITTLKQLADGTAKPEVQDETLVTYAEK-LSK
               11111111111111111111111111111111111111111111111111111111111 111111 111
Chain 2:   112 IDDQLDHGPIIAQRECAIESWDSSGSVYARLMDIERELVLEHFDAIRDGSYTAKSPATE-GNLNLKKDFE

                   .    :    .    :    .    :    .    :    .
Chain 1:   210 EEARID--WSLSAAQLERCIRAFNP--WPMSWLEI-EGQPVKVWKAS
               111111  11111111111111111  11111111 11111111111
Chain 2:   181 QLRRLDLNERGTFGHFLNRLRALTHDDFRNAWFVDASGRKVFVRVVL

Note: positions are from PDB; the numbers between alignments are block index
```

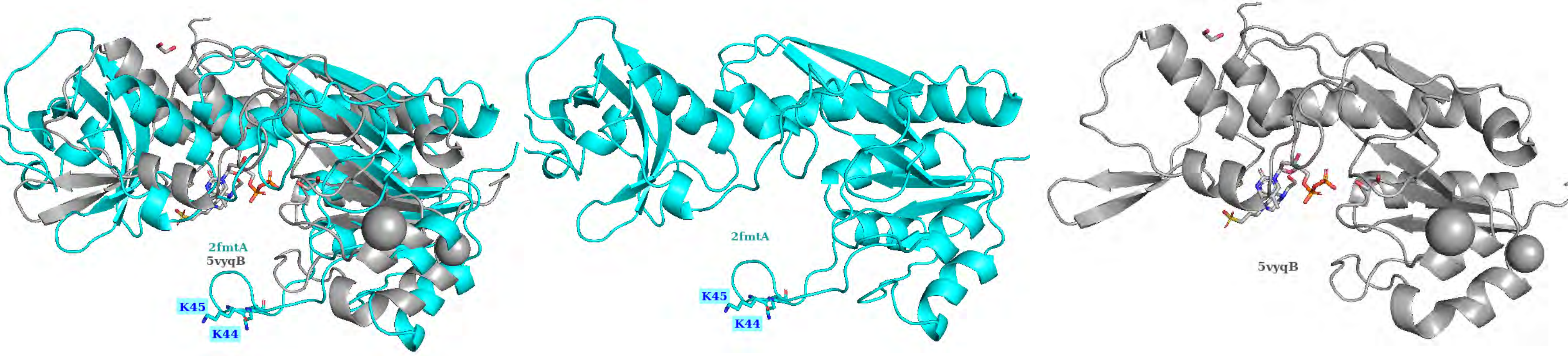

UniProt ID: Q70LM7

PDB ID: 5ES5_B

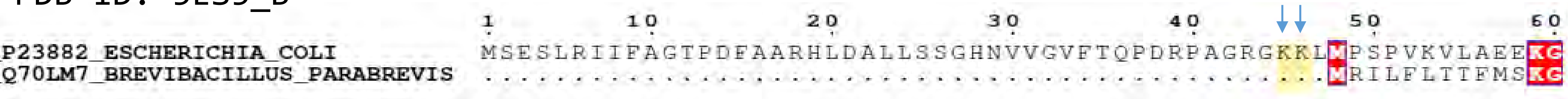

Full sequences in supplemental file.

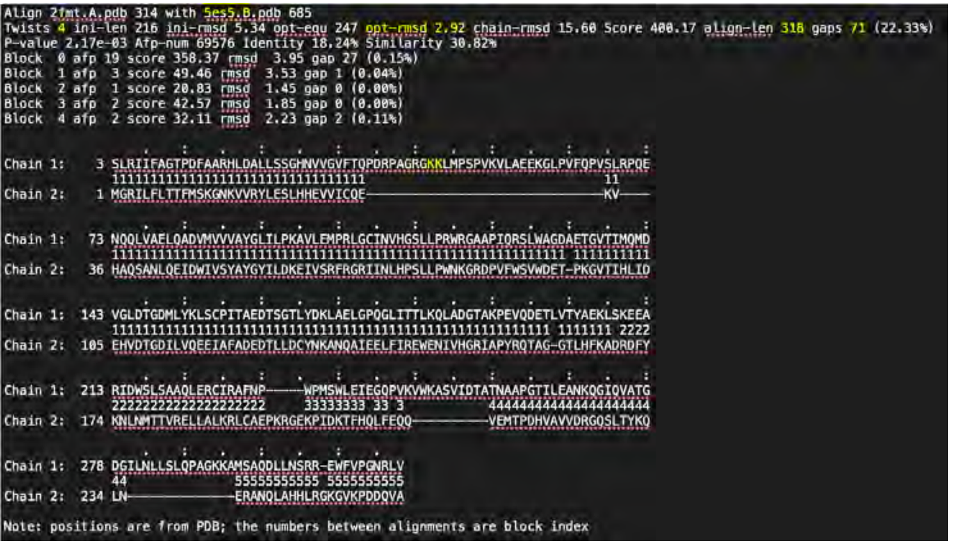

```
Align 2fmt.A.pdb 314 with 5es5.B.pdb 685
Twists 4 ini-len 216 ini-rmsd 5.34 opt-equ 247 opt-rmsd 2.92 chain-rmsd 15.60 Score 400.17 align-len 318 gaps 71 (22.33%)
P-value 2.17e-03 Afp-num 69576 Identity 18.24% Similarity 30.82%
Block  0 afp 19 score 358.37 rmsd  3.95 gap 27 (0.15%)
Block  1 afp  3 score 49.46 rmsd  3.53 gap 1 (0.04%)
Block  2 afp  1 score 20.83 rmsd  1.45 gap 0 (0.00%)
Block  3 afp  2 score 42.57 rmsd  1.85 gap 0 (0.00%)
Block  4 afp  2 score 32.11 rmsd  2.23 gap 2 (0.11%)

                  .    :    .    :    .    :    .    :    .    :    .    :    .    :
Chain 1:    3 SLRIIFAGTPDFAARHLDALLSSGHNVVGVFTQPDRPAGRGKKLMPSPVKVLAEEKGLPVFQPVSLRPQE
              111111111111111111111111111111111                              11
Chain 2:    1 MGRILFLTTFMSKGNKVVRYLESLHHEVVICQE------------------------------KV---

                  .    :    .    :    .    :    .    :    .    :    .    :    .    :
Chain 1:   73 NQQLVAELQADVMVVVAYGLILPKAVLEMPRLGCINVHGSLLPRWRGAAPIQRSLWAGDAETGVTIMQMD
              111111111111111111111111111111111111111111111111111111111111 1111111111
Chain 2:   36 HAQSANLQEIDWIVSYAYGYILDKEIVSRFRGRIINLHPSLLPWNKGRDPVFWSVWDET-PKGVTIHLID

                  .    :    .    :    .    :    .    :    .    :    .    :    .    :
Chain 1:  143 VGLDTGDMLYKLSCPITAEDTSGTLYDKLAELGPQGLITTLKQLADGTAKPEVQDETLVTYAEKLSKEEA
              1111111111111111111111111111111111111111111111111111111 1111111 2222
Chain 2:  105 EHVDTGDILVQEEIAFADEDTLLDCYNKANQAIEELFIREWENIVHGRIAPYRQTAG-GTLHFKADRDFY

                  .    :    .    :    .    :    .    :    .    :    .    :    .    :
Chain 1:  213 RIDWSLSAAQLERCIRAFNP-------WPMSWLEIEGQPVKVWKASVIDTATNAAPGTILEANKQGIQVATG
              222222222222222222       33333333 33 3              444444444444444444444
Chain 2:  174 KNLNMTTVRELLALKRLCAEPKRGEKPIDKTFHQLFEQQ--------------VEMTPDHVAVVDRGQSLTYKQ

                  .    :    .    :    .    :    .
Chain 1:  278 DGILNLLSLQPAGKKAMSAQDLLNSRR-EWFVPGNRLV
              44                       55555555555 5555555555
Chain 2:  234 LN----------------------ERANQLAHHLRGKGVKPDDQVA

Note: positions are from PDB; the numbers between alignments are block index
```

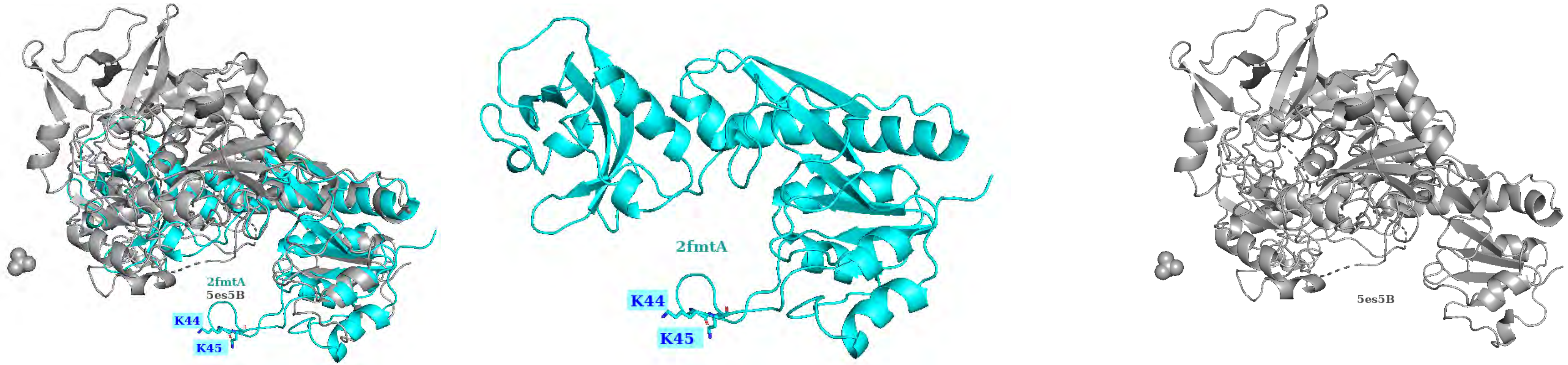

UniProt ID: Q70LM7

PDB ID: 5ES6_A

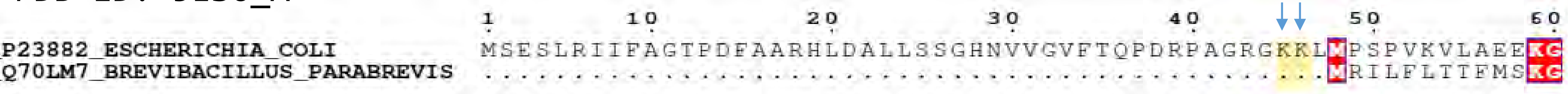

Full sequences in supplemental file.

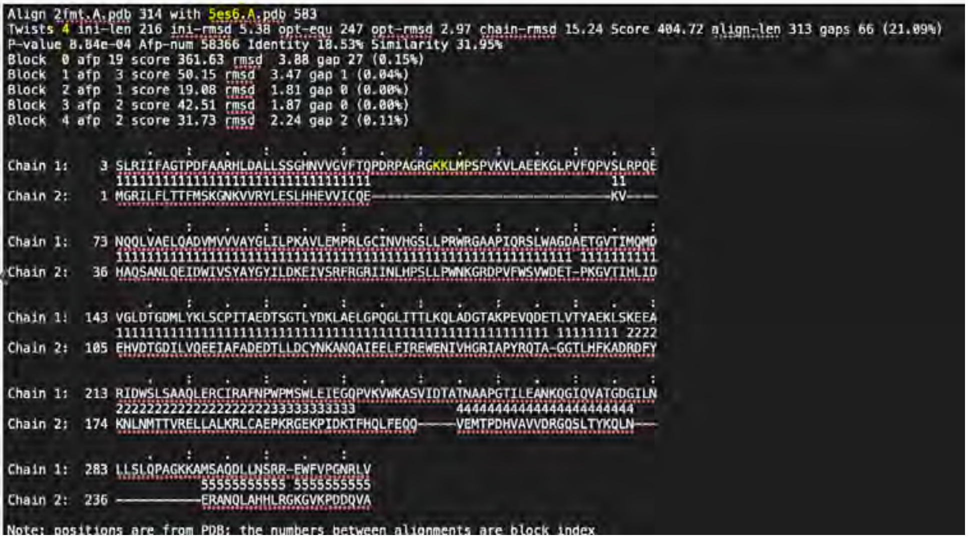

```
Align 2fmt.A.pdb 314 with 5es6.A.pdb 583
Twists 4 ini-len 216 ini-rmsd 5.38 opt-equ 247 opt-rmsd 2.97 chain-rmsd 15.24 Score 404.72 align-len 313 gaps 66 (21.09%)
P-value 8.84e-04 Afp-num 58366 Identity 18.53% Similarity 31.95%
Block  0 afp 19 score 361.63 rmsd  3.88 gap 27 (0.15%)
Block  1 afp  3 score 50.15 rmsd  3.47 gap 1 (0.04%)
Block  2 afp  1 score 19.08 rmsd  1.81 gap 0 (0.00%)
Block  3 afp  2 score 42.51 rmsd  1.87 gap 0 (0.00%)
Block  4 afp  2 score 31.73 rmsd  2.24 gap 2 (0.11%)

                 .    :    .    :    .    :    .    :    .    :    .    :    .    :
Chain 1:    3 SLRIIFAGTPDFAARHLDALLSSGHNVVGVFTQPDRPAGRGKKLMPSPVKVLAEEKGLPVFQPVSLRPQE
              111111111111111111111111111111111                               11
Chain 2:    1 MGRILFLTTFMSKGNKVVRYLESLHHEVVICQE-------------------------------KV---

                 .    :    .    :    .    :    .    :    .    :    .    :    .    :
Chain 1:   73 NQQLVAELQADVMVVVAYGLILPKAVLEMPRLGCINVHGSLLPRWRGAAPIQRSLWAGDAETGVTIMQMD
              1111111111111111111111111111111111111111111111111111111111111 111111111
Chain 2:   36 HAQSANLQEIDWIVSYAYGYILDKEIVSRFRGRIINLHPSLLPWNKGRDPVFWSVWDET-PKGVTIHLID

                 .    :    .    :    .    :    .    :    .    :    .    :    .    :
Chain 1:  143 VGLDTGDMLYKLSCPITAEDTSGTLYDKLAELGPQGLITTLKQLADGTAKPEVQDETLVTYAEKLSKEEA
              111111111111111111111111111111111111111111111111111111111111 11111111 2222
Chain 2:  105 EHVDTGDILVQEEIAFADEDTLLDCYNKANQAIEELFIREWENIVHGRIAPYRQTA-GGTLHFKADRDFY

                 .    :    .    :    .    :    .    :    .    :    .    :    .    :
Chain 1:  213 RIDWSLSAAQLERCIRAFNPWPMSWLEIEGQPVKVWKASVIDTATNAAPGTILEANKQGIQVATGDGILN
              22222222222222222222233333333333          4444444444444444444444
Chain 2:  174 KNLNMTTVRELLALKRLCAEPKRGEKPIDKTFHQLFEQQ-----VEMTPDHVAVVDRGQSLTYKQLN---

                 .    :    .    :    .    :
Chain 1:  283 LLSLQPAGKKAMSAQDLLNSRR-EWFVPGNRLV
                         55555555555 5555555555
Chain 2:  236 -----------ERANQLAHHLRGKGVKPDDQVA

Note: positions are from PDB; the numbers between alignments are block index
```

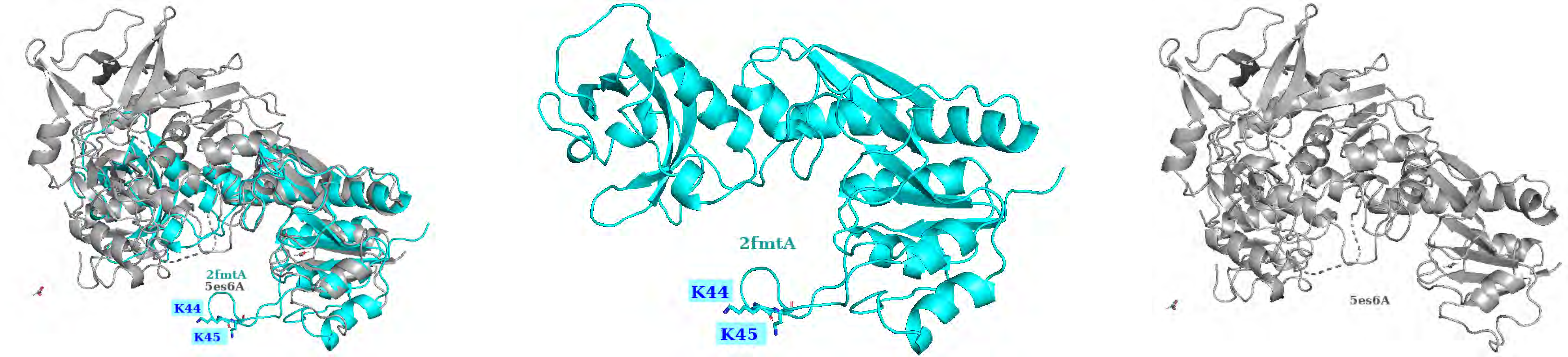

UniProt ID: Q70LM7

PDB ID: 5ES7_A

```
                                 1         10        20        30        40        50        60
P23882_ESCHERICHIA_COLI          MSESLRIIFAGTPDFAARHLDALLSSGHNVVGVFTQPDRPAGRGKKLMPSPVKVLAEEKG
Q70LM7_BREVIBACILLUS_PARABREVIS  ..............................................MRILFLTTFMSKG
```

Full sequences in supplemental file.

```
Align 2fmt.A.pdb 314 with 5es7.A.pdb 582
Twists 4 ini-len 216 ini-rmsd 5.37 opt-equ 247 opt-rmsd 2.98 chain-rmsd 15.25 Score 401.97 align-len 313 gaps 66 (21.09%)
P-value 9.50e-04 Afp-num 58361 Identity 18.53% Similarity 31.95%
Block  0 afp 19 score 359.28 rmsd  3.88 gap 27 (0.15%)
Block  1 afp  3 score 50.02 rmsd  3.47 gap 1 (0.04%)
Block  2 afp  1 score 19.10 rmsd  1.81 gap 0 (0.00%)
Block  3 afp  2 score 42.43 rmsd  1.87 gap 0 (0.00%)
Block  4 afp  2 score 31.80 rmsd  2.19 gap 2 (0.11%)

Chain 1:    3 SLRIIFAGTPDFAARHLDALLSSGHNVVGVFTQPDRPAGRGKKLMPSPVKVLAEEKGLPVFQPVSLRPQE
                111111111111111111111111111111111                               11
Chain 2:    1 MGRILFLTTFMSKGNKVVRYLESLHHEVVICQE-------------------------------KV----

Chain 1:   73 NQQLVAELQADVMVVVAYGLILPKAVLEMPRLGCINVHGSLLPRWRGAAPIQRSLWAGDAETGVTIMQMD
              11111111111111111111111111111111111111111111111111111111111 1111111111
Chain 2:   36 HAQSANLQEIDWIVSYAYGYILDKEIVSRFRGRIINLHPSLLPWNKGRDPVFWSVWDET-PKGVTIHLID

Chain 1:  143 VGLDTGDMLYKLSCPITAEDTSGTLYDKLAELGPQGLITTLKQLADGTAKPEVQDETLVTYAEKLSKEEA
              111111111111111111111111111111111111111111111111111111111 1111111 2222
Chain 2:  105 EHVDTGDILVQEEIAFADEDTLLDCYNKANQAIEELFIREWENIVHGRIAPYRQTAG-GTLHFKADRDFY

Chain 1:  213 RIDWSLSAAQLERCIRAFNPWPMSWLEIEGQPVKVWKASVIDTATNAAPGTILEANKQGIQVATGDGILN
              2222222222222222222233333333333             44444444444444444444444
Chain 2:  174 KNLNMTTVRELLALKRLCAEPKRGEKPIDKTFHQLFEQQ-----VEMTPDHVAVVDRGQSLTYKQLN---

Chain 1:  283 LLSLQPAGKKAMSAQDLLNSRR-EWFVPGNRLV
                         55555555555 5555555555
Chain 2:  236 -----------ERANQLAHHLRGKGVKPDDQVA

Note: positions are from PDB; the numbers between alignments are block index
```

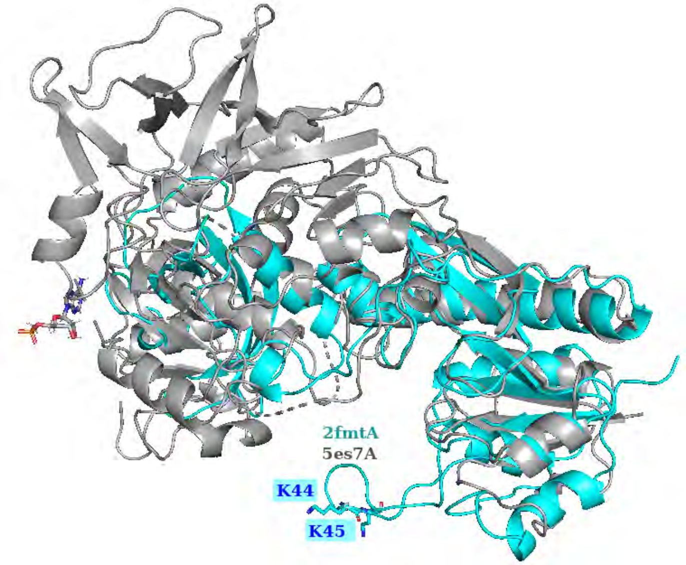

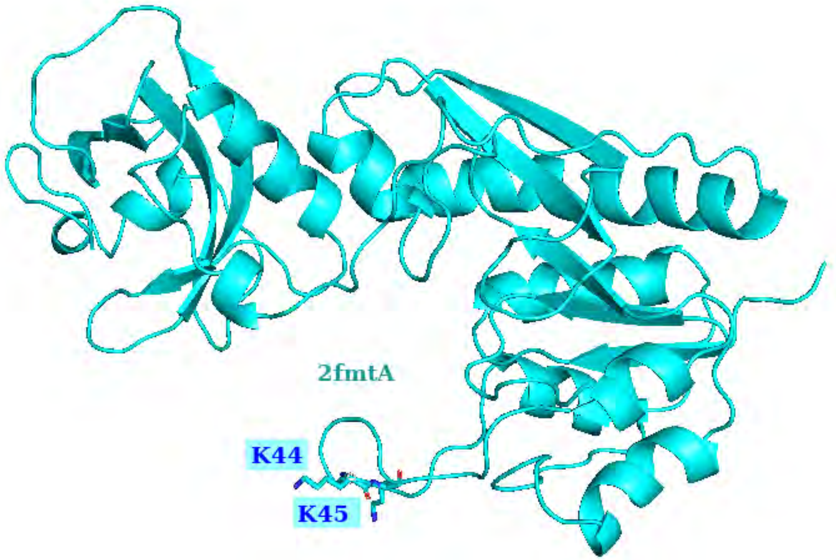

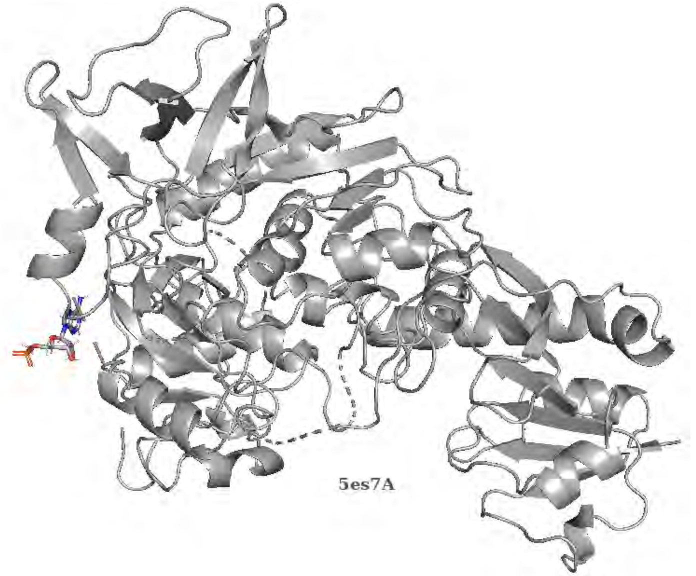

UniProt ID: Q70LM7

PDB ID: 5ES8_B

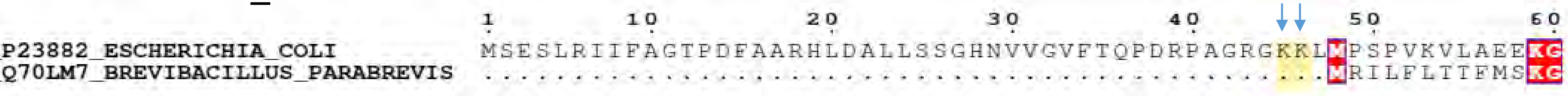

Full sequences in supplemental file.

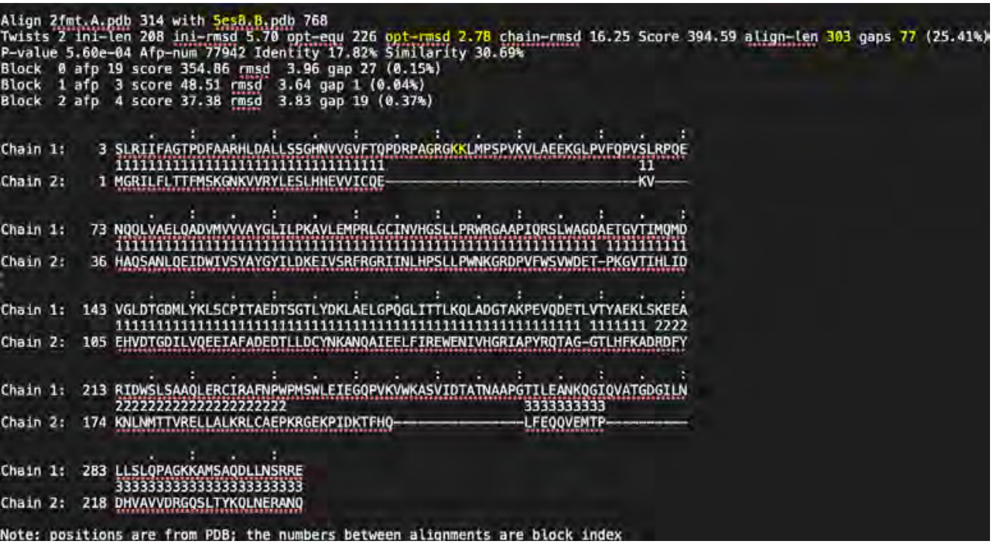

```
Align 2fmt.A.pdb 314 with 5es8.B.pdb 768
Twists 2 ini-len 208 ini-rmsd 5.70 opt-equ 226 opt-rmsd 2.78 chain-rmsd 16.25 Score 394.59 align-len 303 gaps 77 (25.41%)
P-value 5.60e-04 Afp-num 77942 Identity 17.82% Similarity 30.69%
Block  0 afp 19 score 354.86 rmsd  3.96 gap 27 (0.15%)
Block  1 afp  3 score 48.51 rmsd  3.64 gap 1 (0.04%)
Block  2 afp  4 score 37.38 rmsd  3.83 gap 19 (0.37%)

                 .    :    .    :    .    :    .    :    .    :    .    :    .    :
Chain 1:    3 SLRIIFAGTPDFAARHLDALLSSGHNVVGVFTQPDRPAGRGKKLMPSPVKVLAEEKGLPVFQPVSLRPQE
              111111111111111111111111111111111                               11
Chain 2:    1 MGRILFLTTFMSKGNKVVRYLESLHHEVVICQE-------------------------------KV----

                 .    :    .    :    .    :    .    :    .    :    .    :    .    :
Chain 1:   73 NQQLVAELQADVMVVVAYGLILPKAVLEMPRLGCINVHGSLLPRWRGAAPIQRSLWAGDAETGVTIMQMD
              111111111111111111111111111111111111111111111111111111111111 111111111
Chain 2:   36 HAQSANLQEIDWIVSYAYGYILDKEIVSRFRGRIINLHPSLLPWNKGRDPVFWSVWDET-PKGVTIHLID

                 .    :    .    :    .    :    .    :    .    :    .    :    .    :
Chain 1:  143 VGLDTGDMLYKLSCPITAEDTSGTLYDKLAELGPQGLITTLKQLADGTAKPEVQDETLVTYAEKLSKEEA
              1111111111111111111111111111111111111111111111111111111 1111111 2222
Chain 2:  105 EHVDTGDILVQEEIAFADEDTLLDCYNKANQAIEELFIREWENIVHGRIAPYRQTAG-GTLHFKADRDFY

                 .    :    .    :    .    :    .    :    .    :    .    :    .    :
Chain 1:  213 RIDWSLSAAQLERCIRAFNPWPMSWLEIEGQPVKVWKASVIDTATNAAPGTILEANKQGIQVATGDGILN
              22222222222222222222                              3333333333
Chain 2:  174 KNLNMTTVRELLALKRLCAEPKRGEKPIDKTFHQ----------------LFEQQVEMTP----------

                 .    :    .    :
Chain 1:  283 LLSLQPAGKKAMSAQDLLNSRRE
              33333333333333333333333
Chain 2:  218 DHVAVVDRGQSLTYKQLNERANQ

Note: positions are from PDB; the numbers between alignments are block index
```

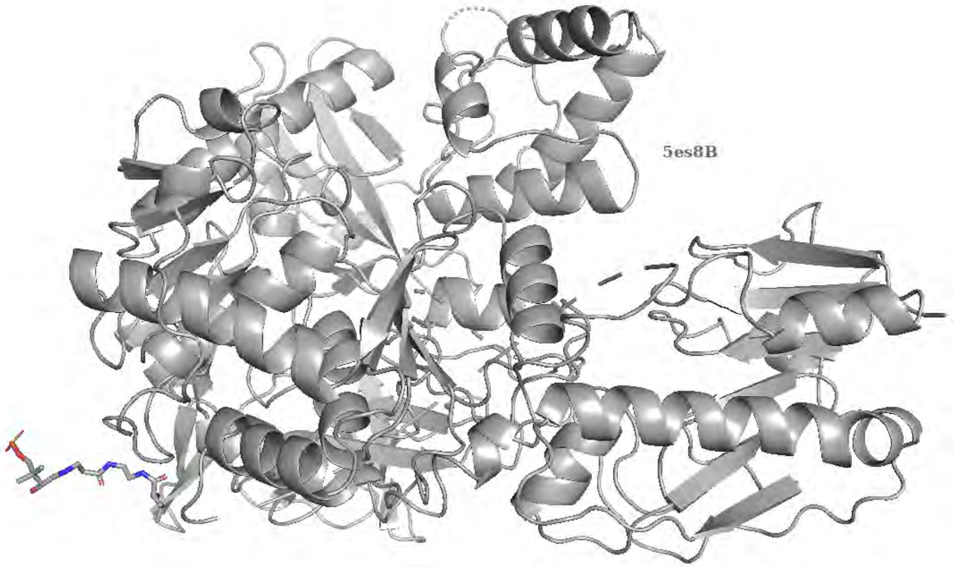

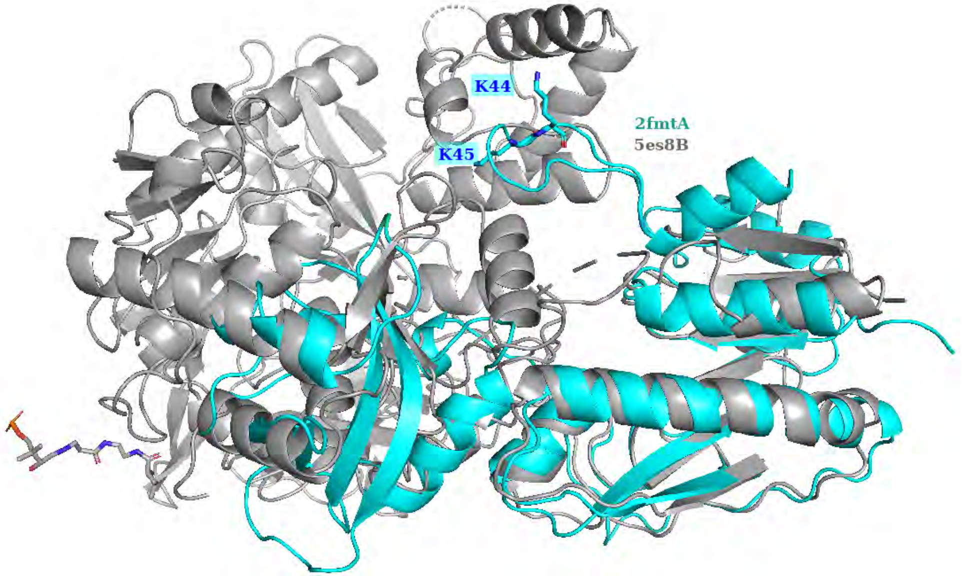

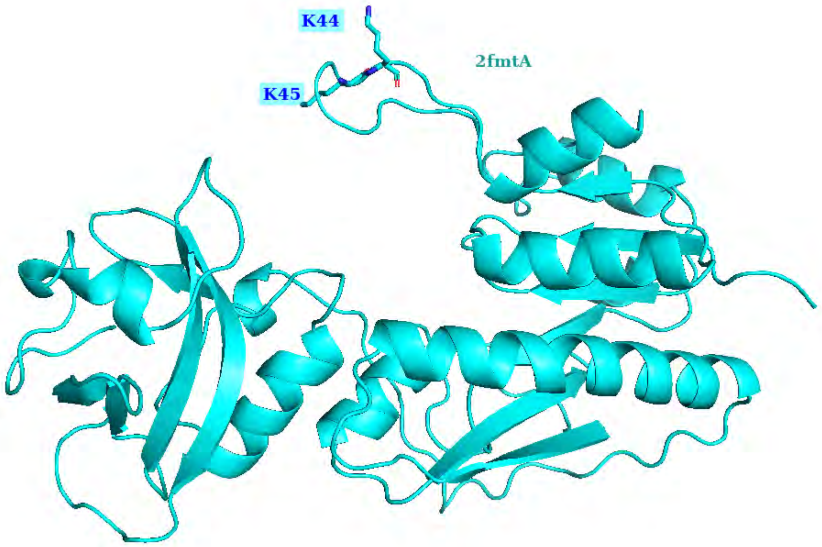

UniProt ID: Q70LM7
PDB ID: 5ES9_A

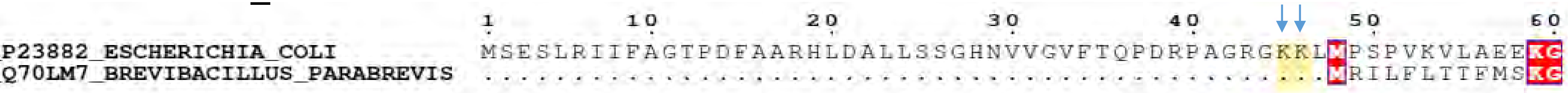

Full sequences in supplemental file.

```
Align 2fmt.A.pdb 314 with 5es9.A.pdb 759
Twists 4 ini-len 216 ini-rmsd 5.38 opt-equ 244 opt-rmsd 2.86 chain-rmsd 15.23 Score 403.80 align-len 313 gaps 69 (22.04%)
P-value 3.32e-03 Afp-num 75307 Identity 18.53% Similarity 31.95%
Block  0 afp 19 score 361.02 rmsd  3.89 gap 27 (0.15%)
Block  1 afp  3 score 50.59 rmsd  3.46 gap 1 (0.04%)
Block  2 afp  1 score 19.11 rmsd  1.81 gap 0 (0.00%)
Block  3 afp  2 score 42.38 rmsd  1.90 gap 0 (0.00%)
Block  4 afp  2 score 31.76 rmsd  2.24 gap 2 (0.11%)

                .    :    .    :    .    :    .    :    .    :    .    :    .    :
Chain 1:    3 SLRIIFAGTPDFAARHLDALLSSGHNVVGVFTQPDRPAGRGKKLMPSPVKVLAEEKGLPVFQPVSLRPQE
              111111111111111111111111111111111                                   11
Chain 2:    1 MGRILFLTTFMSKGNKVVRYLESLHHEVVICQE-----------------------------------KV---

                .    :    .    :    .    :    .    :    .    :    .    :    .    :
Chain 1:   73 NQQLVAELQADVMVVVAYGLILPKAVLEMPRLGCINVHGSLLPRWRGAAPIQRSLWAGDAETGVTIMQMD
              111111111111111111111111111111111111111111111111111111111111 1111111111
Chain 2:   36 HAQSANLQEIDWIVSYAYGYILDKEIVSRFRGRIINLHPSLLPWNKGRDPVFWSVWDET-PKGVTIHLID

                .    :    .    :    .    :    .    :    .    :    .    :    .    :
Chain 1:  143 VGLDTGDMLYKLSCPITAEDTSGTLYDKLAELGPQGLITTLKQLADGTAKPEVQDETLVTYAEKLSKEEA
              1111111111111111111111111111111111111111111111111111111111 11111111 2222
Chain 2:  105 EHVDTGDILVQEEIAFADEDTLLDCYNKANQAIEELFIREWENIVHGRIAPYRQTA-GGTLHFKADRDFY

                .    :    .    :    .    :    .    :    .    :    .    :    .    :
Chain 1:  213 RIDWSLSAAQLERCIRAFNPWPMSWLEIEGQPVKVWKASVIDTATNAAPGTILEANKQGIQVATGDGILN
              2222222222222222222 33333333                    44444444444444444444444
Chain 2:  174 KNLNMTTVRELLALKRLCAEPKRGEKPIDKTFHQLFEQQ-----VEMTPDHVAVVDRGQSLTYKQLN---

                .    :    .    :    .    :
Chain 1:  283 LLSLQPAGKKAMSAQDLLNSRR-EWFVPGNRLV
                           55555555555 5555555555
Chain 2:  236 -----------ERANQLAHHLRGKGVKPDDQVA

Note: positions are from PDB; the numbers between alignments are block index
```

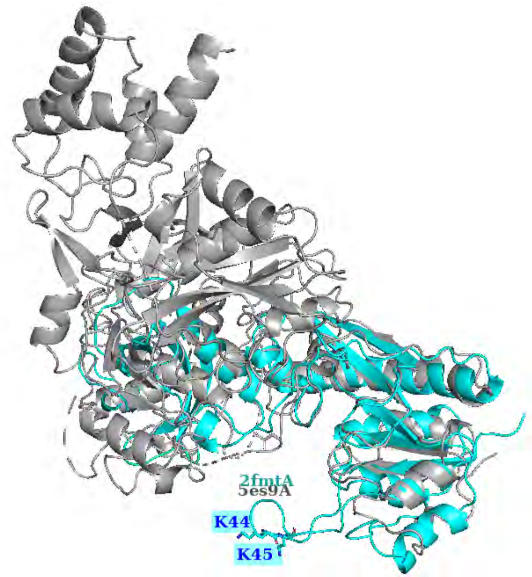

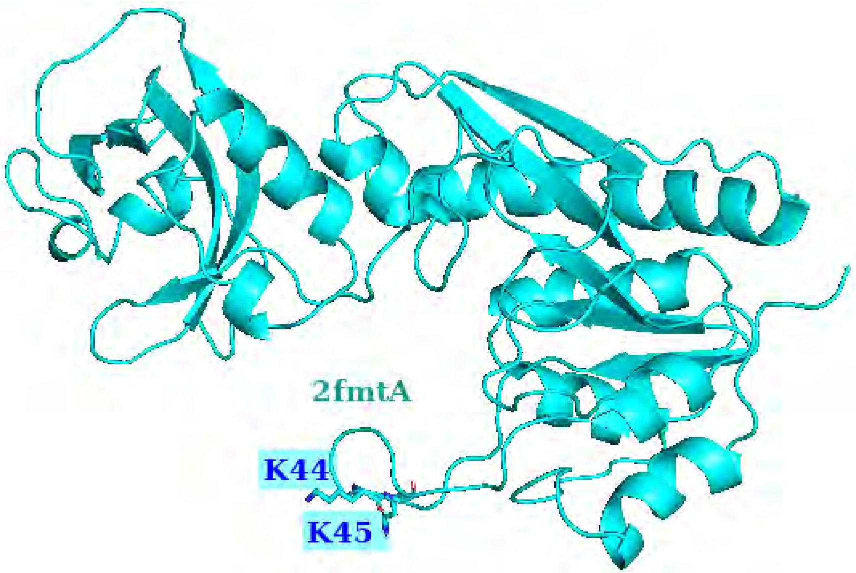

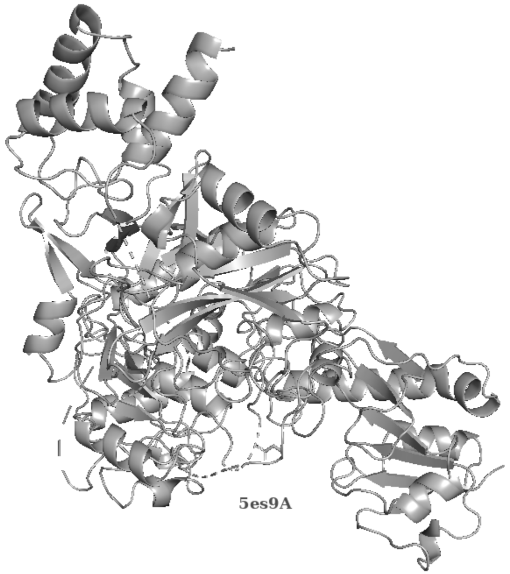

UniProt ID: Q70LM7
PDB ID: 5JNF_A

```
                               1        10        20        30        40      50        60
P23882_ESCHERICHIA_COLI        MSESLRIIFAGTPDFAARHLDALLSSGHNVVGVFTQPDRPAGRGKKLMPSPVKVLAEEKG
Q70LM7_BREVIBACILLUS_PARABREVIS  .............................................MRILFLTTFMSKG
```

Full sequences in supplemental file.

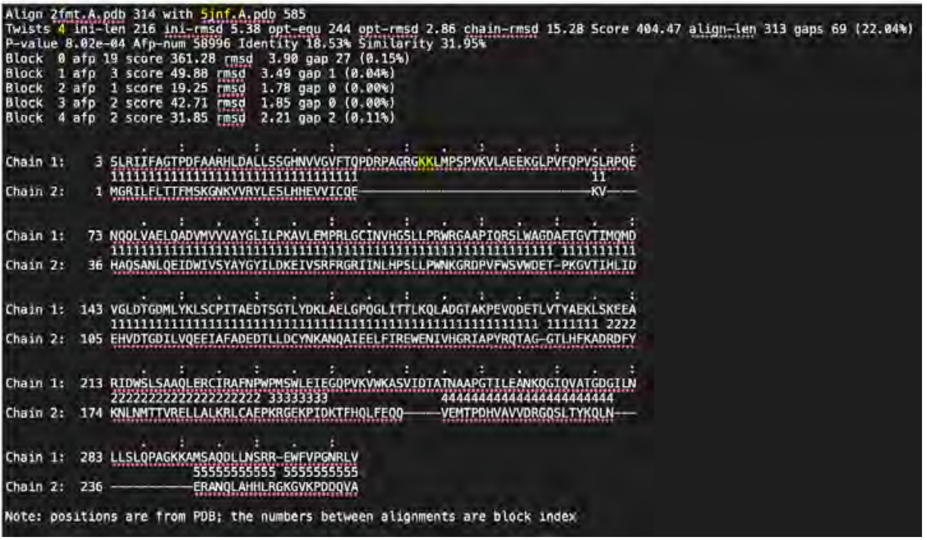

```
Align 2fmt.A.pdb 314 with 5jnf.A.pdb 585
Twists 4 ini-len 216 ini-rmsd 5.38 opt-equ 244 opt-rmsd 2.86 chain-rmsd 15.28 Score 404.47 align-len 313 gaps 69 (22.04%)
P-value 8.02e-04 Afp-num 58996 Identity 18.53% Similarity 31.95%
Block  0 afp 19 score 361.28 rmsd  3.90 gap 27 (0.15%)
Block  1 afp  3 score 49.88 rmsd  3.49 gap 1 (0.04%)
Block  2 afp  1 score 19.25 rmsd  1.78 gap 0 (0.00%)
Block  3 afp  2 score 42.71 rmsd  1.85 gap 0 (0.00%)
Block  4 afp  2 score 31.85 rmsd  2.21 gap 2 (0.11%)

Chain 1:    3 SLRIIFAGTPDFAARHLDALLSSGHNVVGVFTQPDRPAGRGKKLMPSPVKVLAEEKGLPVFQPVSLRPQE
              111111111111111111111111111111111                               11
Chain 2:    1 MGRILFLTTFMSKGNKVVRYLESLHHEVVICQE-------------------------------KV----

Chain 1:   73 NQQLVAELQADVMVVVAYGLILPKAVLEMPRLGCINVHGSLLPRWRGAAPIQRSLWAGDAETGVTIMQMD
              111111111111111111111111111111111111111111111111111111111111 111111111
Chain 2:   36 HAQSANLQEIDWIVSYAYGYILDKEIVSRFRGRIINLHPSLLPWNKGRDPVFWSVWDET-PKGVTIHLID

Chain 1:  143 VGLDTGDMLYKLSCPITAEDTSGTLYDKLAELGPQGLITTLKQLADGTAKPEVQDETLVTYAEKLSKEEA
              1111111111111111111111111111111111111111111111111111111 1111111 2222
Chain 2:  105 EHVDTGDILVQEEIAFADEDTLLDCYNKANQAIEELFIREWENIVHGRIAPYRQTAG-GTLHFKADRDFY

Chain 1:  213 RIDWSLSAAQLERCIRAFNPWPMSWLEIEGQPVKVWKASVIDTATNAAPGTILEANKQGIQVATGDGILN
              2222222222222222222 33333333                4444444444444444444444
Chain 2:  174 KNLNMTTVRELLALKRLCAEPKRGEKPIDKTFHQLFEQQ----VEMTPDHVAVVDRGQSLTYKQLN---

Chain 1:  283 LLSLQPAGKKAMSAQDLLNSRR-EWFVPGNRLV
                         5555555555 5555555555
Chain 2:  236 -----------ERANQLAHHLRGKGVKPDDQVA

Note: positions are from PDB; the numbers between alignments are block index
```

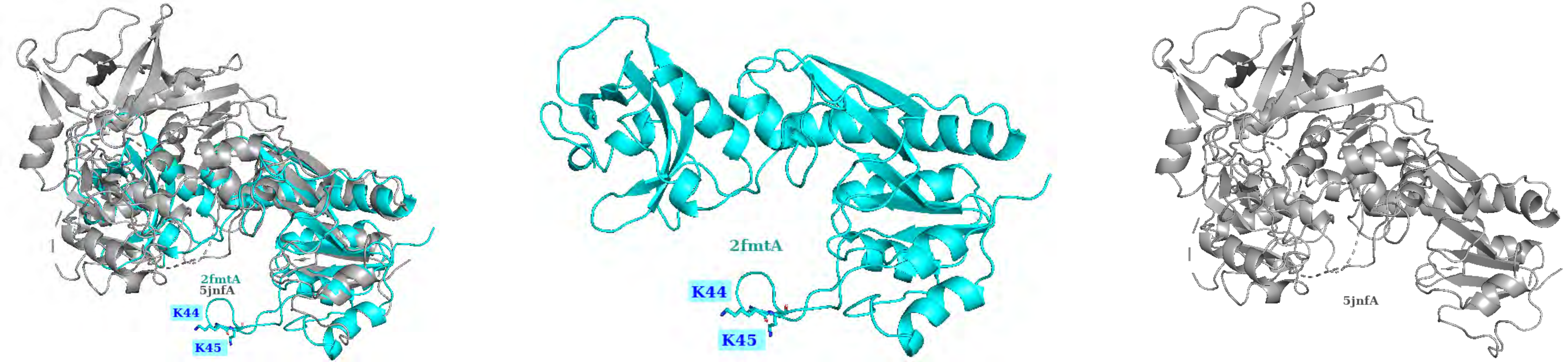

UniProt ID: Q70LM7

PDB ID: 6MFW_A

```
                                 1        10        20        30        40   ↓↓   50        60
P23882_ESCHERICHIA_COLI          MSESLRIIFAGTPDFAARHLDALLSSGHNVVGVFTQPDRPAGRGKKLMPSPVKVLAEEKG
Q70LM7_BREVIBACILLUS_PARABREVIS  ...............................................MRILFLTTFMSKG
```

Full sequences in supplemental file.

```
Align 2fmt.A.pdb 314 with 6mfw.A.pdb 1183
Twists 4 ini-len 208 ini-rmsd 4.83 opt-equ 236 opt-rmsd 3.23 chain-rmsd 15.17 Score 396.90 align-len 315 gaps 79 (25.08%)
P-value 4.70e-02 Afp-num 118390 Identity 17.78% Similarity 29.84%
Block  0 afp  3 score 44.59 rmsd  3.01 gap 1 (0.04%)
Block  1 afp 18 score 350.91 rmsd  5.15 gap 17 (0.11%)
Block  2 afp  1 score 20.80 rmsd  1.46 gap 0 (0.00%)
Block  3 afp  2 score 42.49 rmsd  1.89 gap 0 (0.00%)
Block  4 afp  2 score 32.11 rmsd  2.20 gap 2 (0.11%)

                  .    :    .    :    .    :    .    :    .    :    .    :    .    :
Chain 1:    3 SLRIIFAGTP-DFAARHLDALLSSGHNVVGVFTQPDRPAGRGKKLMPSPVKVLAEEKGLPVFQPVSLRPQ
              1111111111 1111111111111111 111111
Chain 2:    1 MGRILFLTTFMSKGNKVVRYLESLHHEV-VICQEKVH----------------------------------

                  .    :    .    :    .    :    .    :    .    :    .    :    .    :
Chain 1:   72 ENQQLVAELQADVMVVVAYGLILPKAVLEMPRLGCINVHGSLLPRWRGAAPIQRSLWAGDAETGVTIMQM
                222222222222222222222222222222222222222222222222222222222 222222222
Chain 2:   37 --AQSANLQEIDWIVSYAYGYILDKEIVSRFRGRIINLHPSLLPWNKGRDPVFWSVWDET-PKGVTIHLI

                  .    :    .    :    .    :    .    :    .    :    .    :    .    :
Chain 1:  142 DVGLDTGDMLYKLSCPITAEDTSGTLYDKLAELGPQGLITTLKQLADGTAKPEVQDETLVTYAEKLSKEE
              2222222222222222222222222222222222222222222222222222222222222222222222
Chain 2:  104 DEHVDTGDILVQEEIAFADEDTLLDCYNKANQAIEELFIREWENIVHGRIAPYRQTAGGTLHFKADRDFY

                  .    :    .    :    .    :    .    :    .    :    .    :    .    :
Chain 1:  212 ARIDWSLSAAQLERCIRAFNP-WPMSWLEIEGQPVKVWKASVIDTATNAAPGTILEANKQGIQVATGDGI
              222      22222222      33333333 33 3          4444444444444444444444
Chain 2:  174 KNLNMTTVRELLALKRLCAEPKKPIDKTFHQLFEQQ----------VEMTPDHVAVVDRGQSLTYKQLN-

                  .    :    .    :    .    :    .
Chain 1:  281 LNLLSLQPAGKKAMSAQDLLNSRR-EWFVPGNRLV
                                 5555555555 5555555555
Chain 2:  236 -------------ERANQLAHHLRGKGVKPDDQVA
```

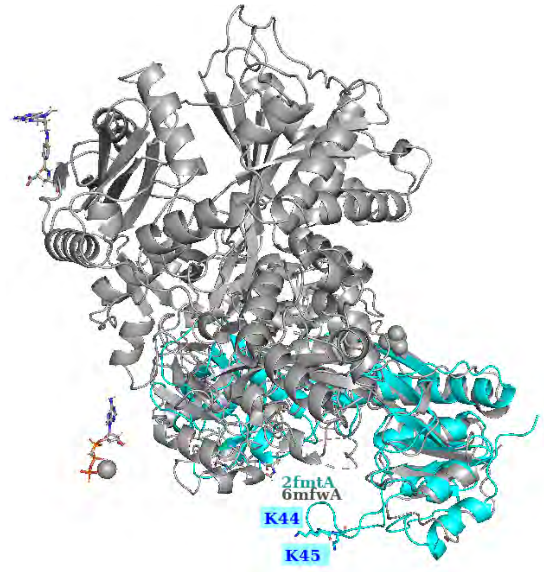

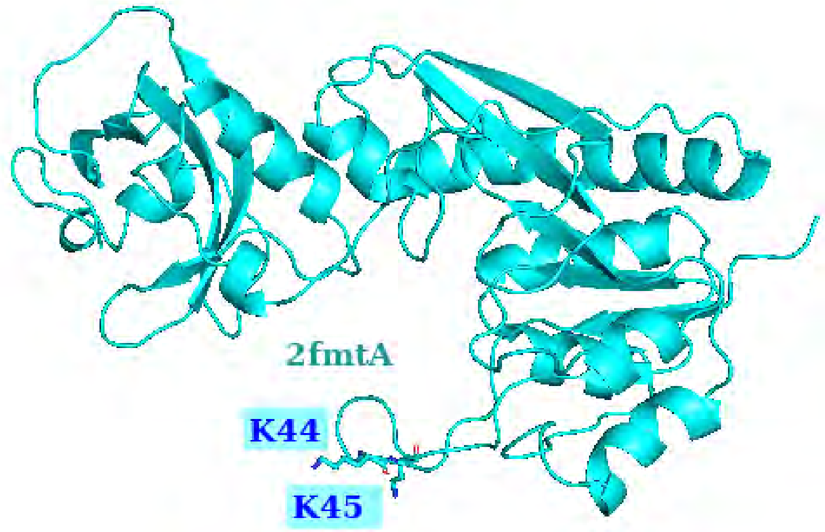

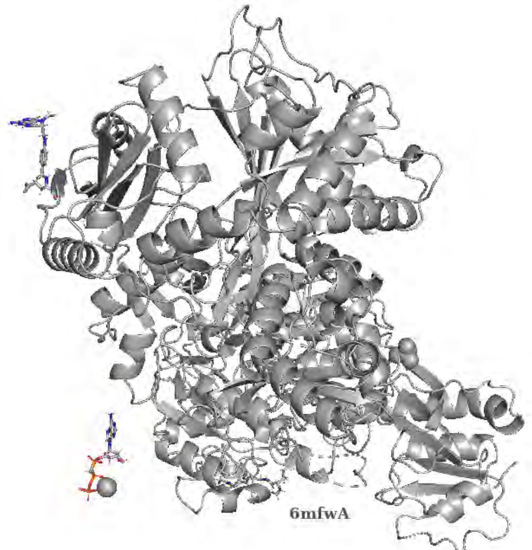

UniProt ID: Q70LM7

PDB ID: 6MFX_A

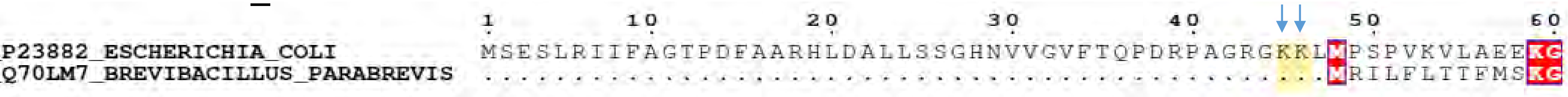

Full sequences in supplemental file.

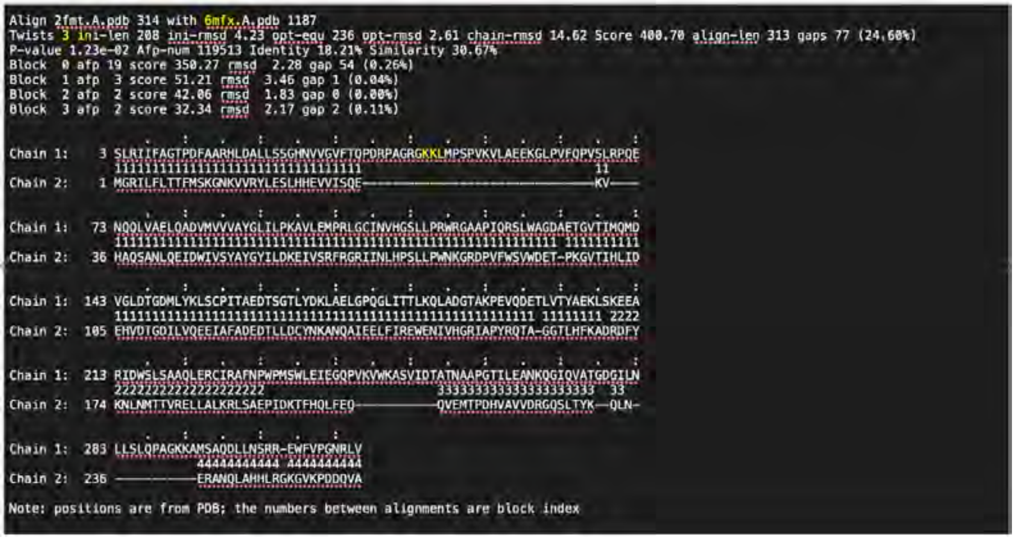

```
Align 2fmt.A.pdb 314 with 6mfx.A.pdb 1187
Twists 3 ini-len 208 ini-rmsd 4.23 opt-equ 236 opt-rmsd 2.61 chain-rmsd 14.62 Score 400.70 align-len 313 gaps 77 (24.60%)
P-value 1.23e-02 Afp-num 119513 Identity 18.21% Similarity 30.67%
Block  0 afp 19 score 350.27 rmsd  2.28 gap 54 (0.26%)
Block  1 afp  3 score 51.21 rmsd  3.46 gap 1 (0.04%)
Block  2 afp  2 score 42.06 rmsd  1.83 gap 0 (0.00%)
Block  3 afp  2 score 32.34 rmsd  2.17 gap 2 (0.11%)

Chain 1:    3 SLRIIFAGTPDFAARHLDALLSSGHNVVGVFTQPDRPAGRGKKLMPSPVKVLAEEKGLPVFQPVSLRPQE
                111111111111111111111111111111111                              11
Chain 2:    1 MGRILFLTTFMSKGNKVVRYLESLHHEVVISQE-------------------------------KV----

Chain 1:   73 NQQLVAELQADVMVVVAYGLILPKAVLEMPRLGCINVHGSLLPRWRGAAPIQRSLWAGDAETGVTIMQMD
                111111111111111111111111111111111111111111111111111111111111 1111111111
Chain 2:   36 HAQSANLQEIDWIVSYAYGYILDKEIVSRFRGRIINLHPSLLPWNKGRDPVFWSVWDET-PKGVTIHLID

Chain 1:  143 VGLDTGDMLYKLSCPITAEDTSGTLYDKLAELGPQGLITTLKQLADGTAKPEVQDETLVTYAEKLSKEEA
                1111111111111111111111111111111111111111111111111111111 11111111 2222
Chain 2:  105 EHVDTGDILVQEEIAFADEDTLLDCYNKANQAIEELFIREWENIVHGRIAPYRQTA-GGTLHFKADRDFY

Chain 1:  213 RIDWSLSAAQLERCIRAFNPWPMSWLEIEGQPVKVWKASVIDTATNAAPGTILEANKQGIQVATGDGILN
                22222222222222222222                      333333333333333333333  33
Chain 2:  174 KNLNMTTVRELLALKRLSAEPIDKTFHQLFEQ-----------QVEMTPDHVAVVDRGQSLTYK--QLN-

Chain 1:  283 LLSLQPAGKKAMSAQDLLNSRR-EWFVPGNRLV
                           44444444444 4444444444
Chain 2:  236 -----------ERANQLAHHLRGKGVKPDDQVA

Note: positions are from PDB; the numbers between alignments are block index
```

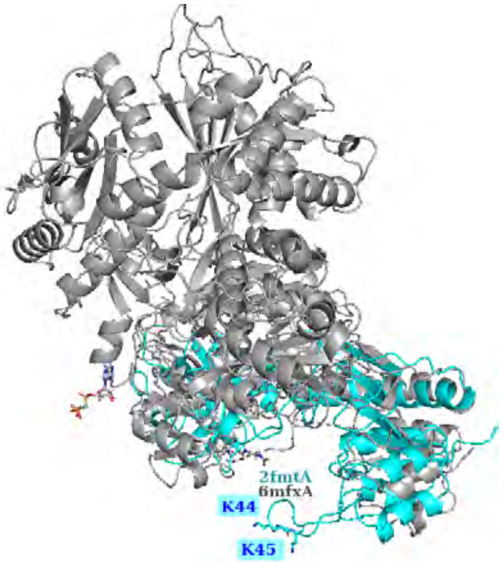

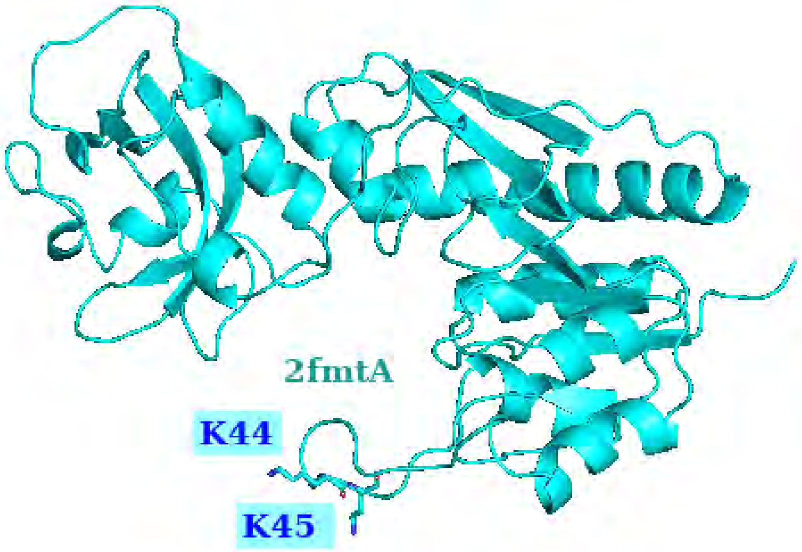

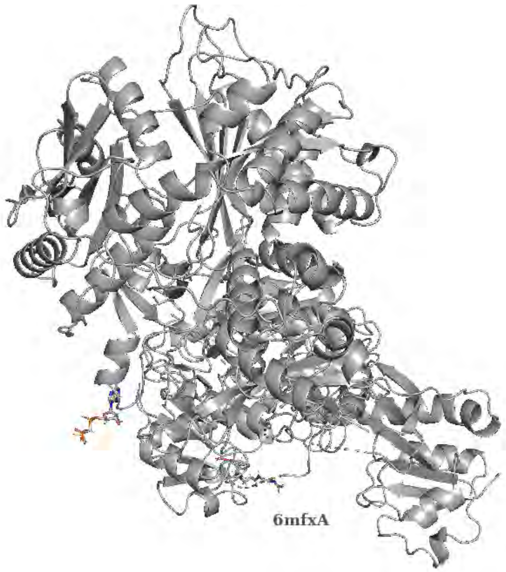

UniProt ID: Q70LM7

PDB ID: 6MFY_A

```
                                 1        10        20        30        40        50        60
P23882_ESCHERICHIA_COLI          MSESLRIIFAGTPDFAARHLDALLSSGHNVVGVFTQPDRPAGRGKKLMPSPVKVLAEEKG
Q70LM7_BREVIBACILLUS_PARABREVIS  ...............................................MRILFLTTFMSKG
```

Full sequences in supplemental file.

```
Align 2fmt.A.pdb 314 with 6mfy.A.pdb 1598
Twists 4 ini-len 216 ini-rmsd 5.41 opt-equ 242 opt-rmsd 3.12 chain-rmsd 15.03 Score 390.45 align-len 313 gaps 71 (22.68%)
P-value 1.30e-01 Afp-num 160954 Identity 18.53% Similarity 30.67%
Block  0 afp 19 score 344.71 rmsd  2.40 gap 54 (0.26%)
Block  1 afp  3 score 52.04 rmsd  3.67 gap 0 (0.00%)
Block  2 afp  1 score 20.52 rmsd  1.52 gap 0 (0.00%)
Block  3 afp  2 score 42.58 rmsd  1.82 gap 0 (0.00%)
Block  4 afp  2 score 30.48 rmsd  2.28 gap 2 (0.11%)

                 .    :    .    :    .    :    .    :    .    :    .    :    .    :
Chain 1:    3 SLRIIFAGTPDFAARHLDALLSSGHNVVGVFTQPDRPAGRGKKLMPSPVKVLAEEKGLPVFQPVSLRPQE
              11111111111111111111111111111111                                111
Chain 2:    1 MGRILFLTTFMSKGNKVVRYLESLHHEVVICQE---------------------------------KVH----

                 .    :    .    :    .    :    .    :    .    :    .    :    .    :
Chain 1:   73 NQQLVAELQADVMVVVAYGLILPKAVLEMPRLGCINVHGSLLPRWRGAAPIQRSLWAGDAETGVTIMQMD
               1111111111111111111111111111111111111111111111111111111 1111111111
Chain 2:   37 -AQSANLQEIDWIVSYAYGYILDKEIVSRFRGRIINLHPSLLPWNKGRDPVFWSVWDET-PKGVTIHLID

                 .    :    .    :    .    :    .    :    .    :    .    :    .    :
Chain 1:  143 VGLDTGDMLYKLSCPITAEDTSGTLYDKLAELGPQGLITTLKQLADGTAKPEVQDETLVTYAEKLSKEEA
              11111111111111111111111111111111111111111111111111111 1111111  2222
Chain 2:  105 EHVDTGDILVQEEIAFADEDTLLDCYNKANQAIEELFIREWENIVHGRIAPYRQTA-GGTLHFKADRDFY

                 .    :    .    :    .    :    .    :    .    :    .    :    .    :
Chain 1:  213 RIDWSLSAAQLERCIRAFNPWPMSWLEIEGQPVKVWKASVIDTATNAAPGTILEANKQGIQVATGDGILN
              222222222222222222 33333333                     4444444444444444444444
Chain 2:  174 KNLNMTTVRELLALKRLCA-KPIDKTFHQLFEQQ----------VEMTPDHVAVVDRGQSLTYKQLN--

                 .    :    .    :    .    :
Chain 1:  283 LLSLQPAGKKAMSAQDLLNSRR-EWFVPGNRLV
                         5555555555 5555555555
Chain 2:  236 -----------ERANQLAHHLRGKGVKPDDQVA
```

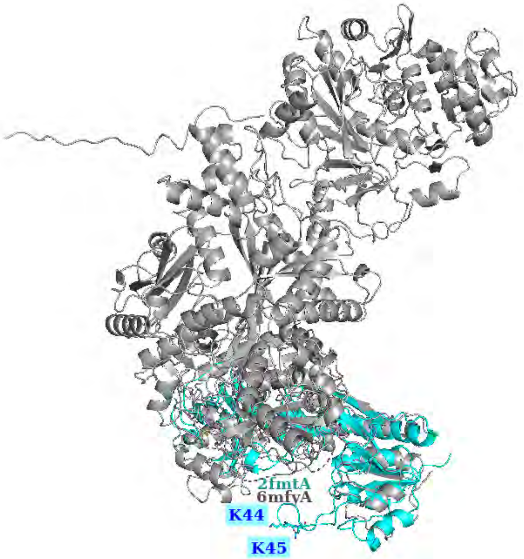

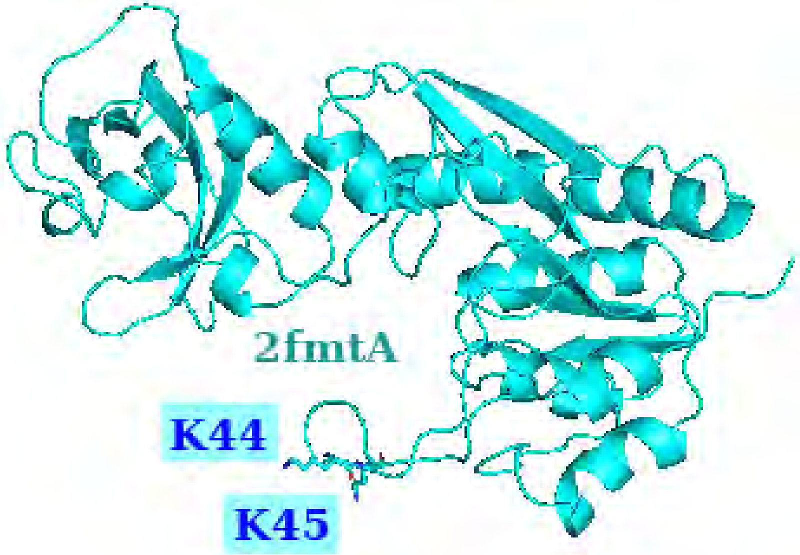

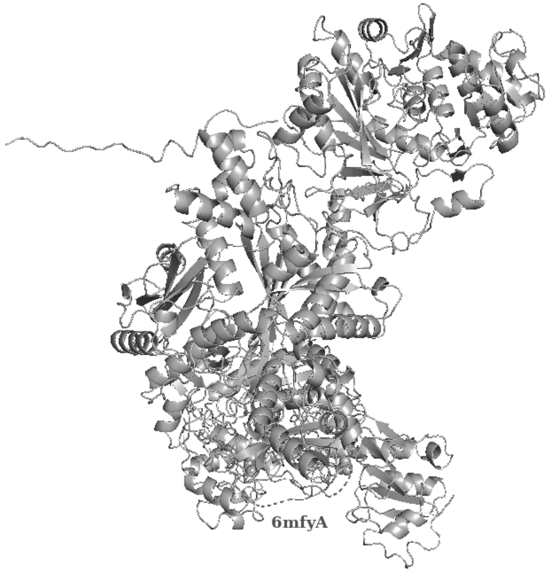

UniProt ID: Q70LM7

PDB ID: 6MFZ_B

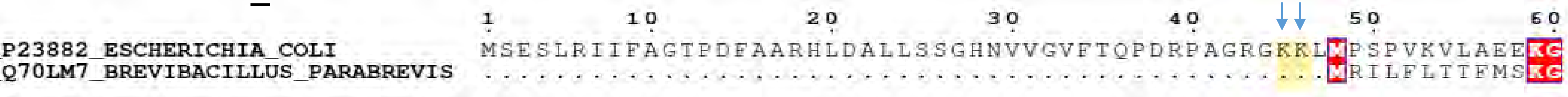

Full sequences in supplemental file.

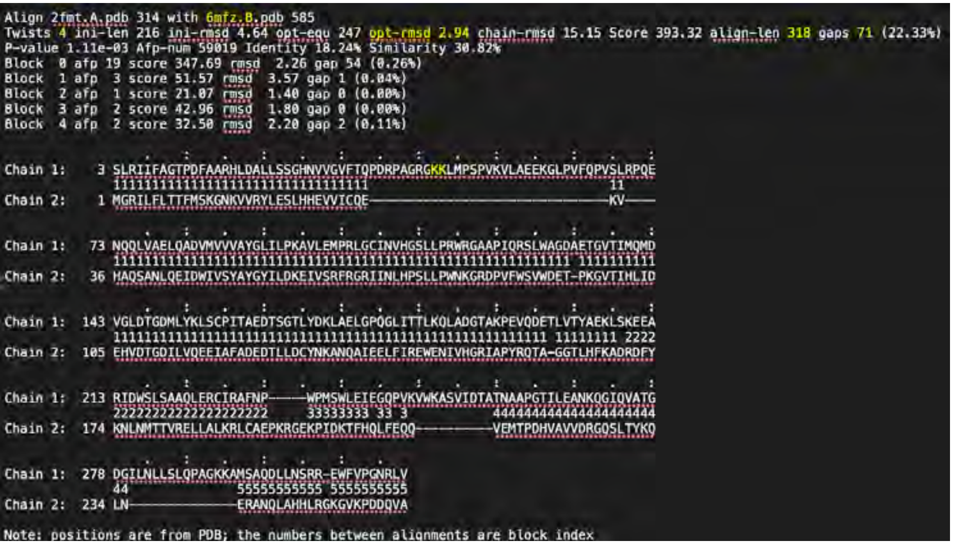

```
Align 2fmt.A.pdb 314 with 6mfz.B.pdb 585
Twists 4 ini-len 216 ini-rmsd 4.64 opt-equ 247 opt-rmsd 2.94 chain-rmsd 15.15 Score 393.32 align-len 318 gaps 71 (22.33%)
P-value 1.11e-03 Afp-num 59019 Identity 18.24% Similarity 30.82%
Block  0 afp 19 score 347.69 rmsd  2.26 gap 54 (0.26%)
Block  1 afp  3 score 51.57 rmsd  3.57 gap 1 (0.04%)
Block  2 afp  1 score 21.07 rmsd  1.40 gap 0 (0.00%)
Block  3 afp  2 score 42.96 rmsd  1.80 gap 0 (0.00%)
Block  4 afp  2 score 32.50 rmsd  2.20 gap 2 (0.11%)

Chain 1:    3 SLRIIFAGTPDFAARHLDALLSSGHNVVGVFTQPDRPAGRGKKLMPSPVKVLAEEKGLPVFQPVSLRPQE
              11111111111111111111111111111111                                  11
Chain 2:    1 MGRILFLTTFMSKGNKVVRYLESLHHEVVICQE---------------------------------KV----

Chain 1:   73 NQQLVAELQADVMVVVAYGLILPKAVLEMPRLGCINVHGSLLPRWRGAAPIQRSLWAGDAETGVTIMQMD
              1111111111111111111111111111111111111111111111111111111111 1111111111
Chain 2:   36 HAQSANLQEIDWIVSYAYGYILDKEIVSRFRGRIINLHPSLLPWNKGRDPVFWSVWDET-PKGVTIHLID

Chain 1:  143 VGLDTGDMLYKLSCPITAEDTSGTLYDKLAELGPQGLITTLKQLADGTAKPEVQDETLVTYAEKLSKEEA
              11111111111111111111111111111111111111111111111111111111 11111111 2222
Chain 2:  105 EHVDTGDILVQEEIAFADEDTLLDCYNKANQAIEELFIREWENIVHGRIAPYRQTA-GGTLHFKADRDFY

Chain 1:  213 RIDWSLSAAQLERCIRAFNP-----WPMSWLEIEGQPVKVWKASVIDTATNAAPGTILEANKQGIQVATG
              22222222222222222222     33333333 33 3          44444444444444444444444
Chain 2:  174 KNLNMTTVRELLALKRLCAEPKRGEKPIDKTFHQLFEQQ----------VEMTPDHVAVVDRGQSLTYKQ

Chain 1:  278 DGILNLLSLQPAGKKAMSAQDLLNSRR-EWFVPGNRLV
              44               55555555555 5555555555
Chain 2:  234 LN---------------ERANQLAHHLRGKGVKPDDQVA

Note: positions are from PDB; the numbers between alignments are block index
```

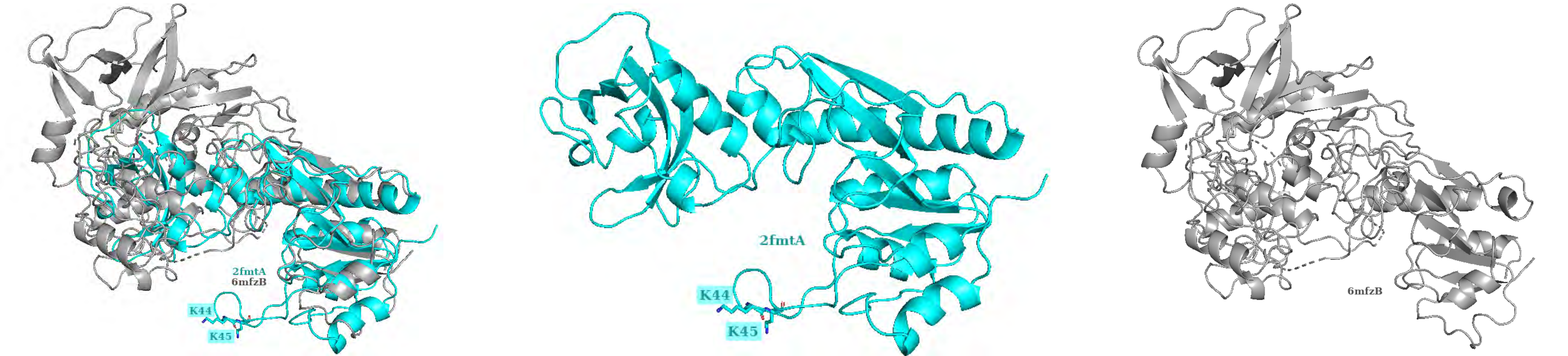

UniProt ID: Q70LM7

PDB ID: 6MG0_A

```
                                 1         10        20        30        40   ↓↓   50        60
P23882_ESCHERICHIA_COLI          MSESLRIIFAGTPDFAARHLDALLSSGHNVVGVFTQPDRPAGRGKKLMPSPVKVLAEEKG
Q70LM7_BREVIBACILLUS_PARABREVIS  ..............................................MRILFLTTFMSKG
```

Full sequences in supplemental file.

```
Align 2fmt.A.pdb 314 with 6mfz.A.pdb 1789
Twists 4 ini-len 216 ini-rmsd 4.69 opt-equ 249 opt-rmsd 3.01 chain-rmsd 15.38 Score 390.56 align-len 318 gaps 69 (21.70%)
P-value 1.63e-01 Afp-num 182776 Identity 18.24% Similarity 30.82%
Block  0 afp 19 score 344.56 rmsd  2.29 gap 54 (0.26%)
Block  1 afp  3 score 51.32 rmsd  3.63 gap 1 (0.04%)
Block  2 afp  1 score 21.83 rmsd  1.20 gap 0 (0.00%)
Block  3 afp  2 score 42.95 rmsd  1.81 gap 0 (0.00%)
Block  4 afp  2 score 32.49 rmsd  2.20 gap 2 (0.11%)

                  .    :    .    :    .    :    .    :    .    :    .    :    .    :
Chain 1:    3 SLRIIFAGTPDFAARHLDALLSSGHNVVGVFTQPDRPAGRGKKLMPSPVKVLAEEKGLPVFQPVSLRPQE
              111111111111111111111111111111111                             11
Chain 2:    1 MGRILFLTTFMSKGNKVVRYLESLHHEVVICQE-----------------------------KV------

                  .    :    .    :    .    :    .    :    .    :    .    :    .    :
Chain 1:   73 NQQLVAELQADVMVVVAYGLILPKAVLEMPRLGCINVHGSLLPRWRGAAPIQRSLWAGDAETGVTIMQMD
              1111111111111111111111111111111111111111111111111111111111 1111111111
Chain 2:   36 HAQSANLQEIDWIVSYAYGYILDKEIVSRFRGRIINLHPSLLPWNKGRDPVFWSVWDET-PKGVTIHLID

                  .    :    .    :    .    :    .    :    .    :    .    :    .    :
Chain 1:  143 VGLDTGDMLYKLSCPITAEDTSGTLYDKLAELGPQGLITTLKQLADGTAKPEVQDETLVTYAEKLSKEEA
              111111111111111111111111111111111111111111111111111111 11111111 2222
Chain 2:  105 EHVDTGDILVQEEIAFADEDTLLDCYNKANQAIEELFIREWENIVHGRIAPYRQTA-GGTLHFKADRDFY

                  .    :    .    :    .    :    .    :    .    :    .    :    .    :
Chain 1:  213 RIDWSLSAAQLERCIRAFNP-----WPMSWLEIEGQPVKVWKASVIDTATNAAPGTILEANKQGIQVATG
              22222222222222222222     3333333333333                    444444444444444444444
Chain 2:  174 KNLNMTTVRELLALKRLCAEPKRGEKPIDKTFHQLFEQQ----------VEMTPDHVAVVDRGQSLTYKQ

                  .    :    .    :    .    :    .
Chain 1:  278 DGILNLLSLQPAGKKAMSAQDLLNSRR-EWFVPGNRLV
              44              55555555555 5555555555
Chain 2:  234 LN--------------ERANQLAHHLRGKGVKPDDQVA

Note: positions are from PDB; the numbers between alignments are block index
```

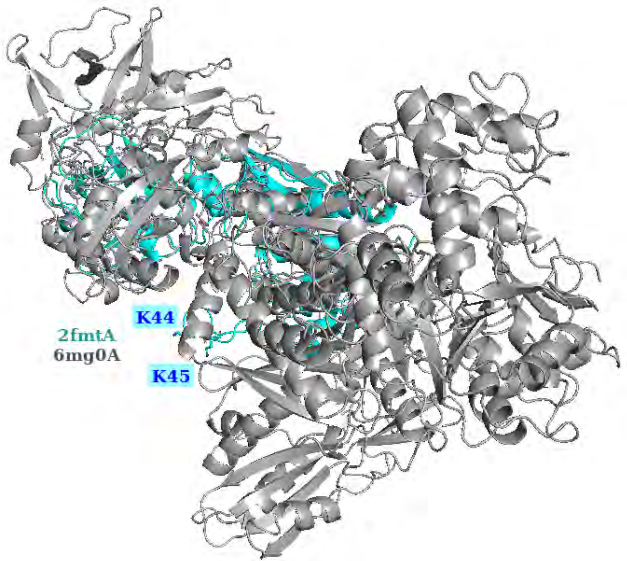

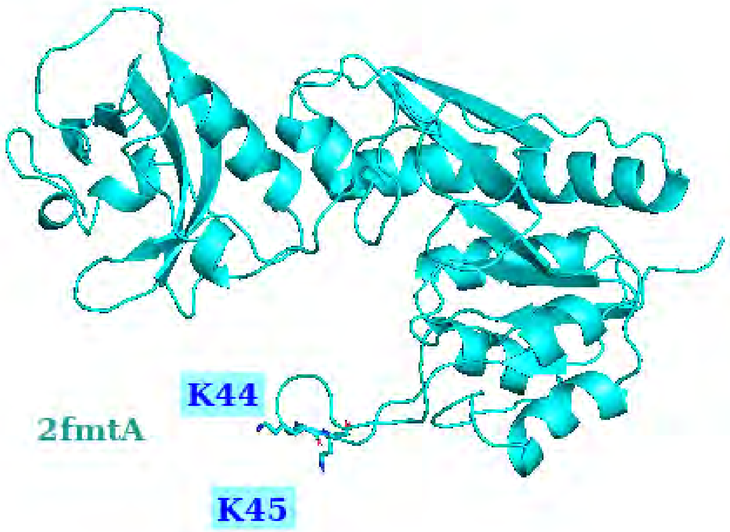

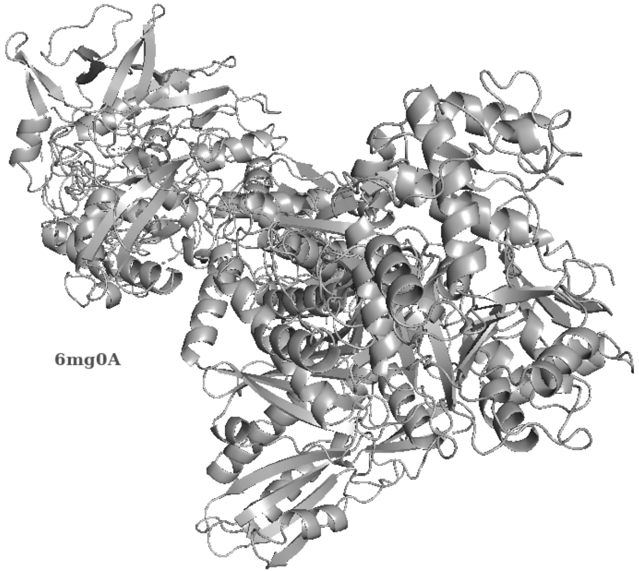

UniProt ID: Q70LM7

PDB ID: 6ULZ_A

```
                              1        10        20        30        40   ↓↓   50        60
P23882_ESCHERICHIA_COLI       MSESLRIIFAGTPDFAARHLDALLSSGHNVVGVFTQPDRPAGRGKKLMPSPVKVLAEEKG
Q70LM7_BREVIBACILLUS_PARABREVIS ...............................................MRILFLTTFMSKG
```

Full sequences in supplemental file.

```
Align 2fmt.A.pdb 314 with 6ulz.A.pdb 585
Twists 4 ini-len 216 ini-rmsd 5.38 opt-equ 247 opt-rmsd 2.99 chain-rmsd 15.27 Score 402.63 align-len 313 gaps 66 (21.09%)
P-value 9.67e-04 Afp-num 59081 Identity 18.53% Similarity 31.95%
Block  0 afp 19 score 359.98 rmsd  3.89 gap 27 (0.15%)
Block  1 afp  3 score 49.79 rmsd  3.45 gap 1 (0.04%)
Block  2 afp  1 score 19.26 rmsd  1.78 gap 0 (0.00%)
Block  3 afp  2 score 42.75 rmsd  1.84 gap 0 (0.00%)
Block  4 afp  2 score 31.66 rmsd  2.23 gap 2 (0.11%)

                      .    :    .    :    .    :    .    :    .    :    .    :    .    :
Chain 1:    3 SLRIIFAGTPDFAARHLDALLSSGHNVVGVFTQPDRPAGRGKKLMPSPVKVLAEEKGLPVFQPVSLRPQE
                111111111111111111111111111111111                                  11
Chain 2:    1 MGRILFLTTFMSKGNKVVRYLESLHHEVVICQE----------------------------------KV--

                      .    :    .    :    .    :    .    :    .    :    .    :    .    :
Chain 1:   73 NQQLVAELQADVMVVVAYGLILPKAVLEMPRLGCINVHGSLLPRWRGAAPIQRSLWAGDAETGVTIMQMD
                11111111111111111111111111111111111111111111111111111111111 1111111111
Chain 2:   36 HAQSANLQEIDWIVSYAYGYILDKEIVSRFRGRIINLHPSLLPWNKGRDPVFWSVWDET-PKGVTIHLID

                      .    :    .    :    .    :    .    :    .    :    .    :    .    :
Chain 1:  143 VGLDTGDMLYKLSCPITAEDTSGTLYDKLAELGPQGLITTLKQLADGTAKPEVQDETLVTYAEKLSKEEA
                11111111111111111111111111111111111111111111111111111111 11111111 2222
Chain 2:  105 EHVDTGDILVQEEIAFADEDTLLDCYNKANQAIEELFIREWENIVHGRIAPYRQTA-GGTLHFKADRDFY

                      .    :    .    :    .    :    .    :    .    :    .    :    .    :
Chain 1:  213 RIDWSLSAAQLERCIRAFNPWPMSWLEIEGQPVKVWKASVIDTATNAAPGTILEANKQGIQVATGDGILN
                2222222222222222222223333333333             44444444444444444444444
Chain 2:  174 KNLNMTTVRELLALKRLCAEPKRGEKPIDKTFHQLFEQQ-----VEMTPDHVAVVDRGQSLTYKQLN---

                      .    :    .    :    .    :
Chain 1:  283 LLSLQPAGKKAMSAQDLLNSRR-EWFVPGNRLV
                           55555555555 5555555555
Chain 2:  236 -----------ERANQLAHHLRGKGVKPDDQVA

Note: positions are from PDB; the numbers between alignments are block index
```

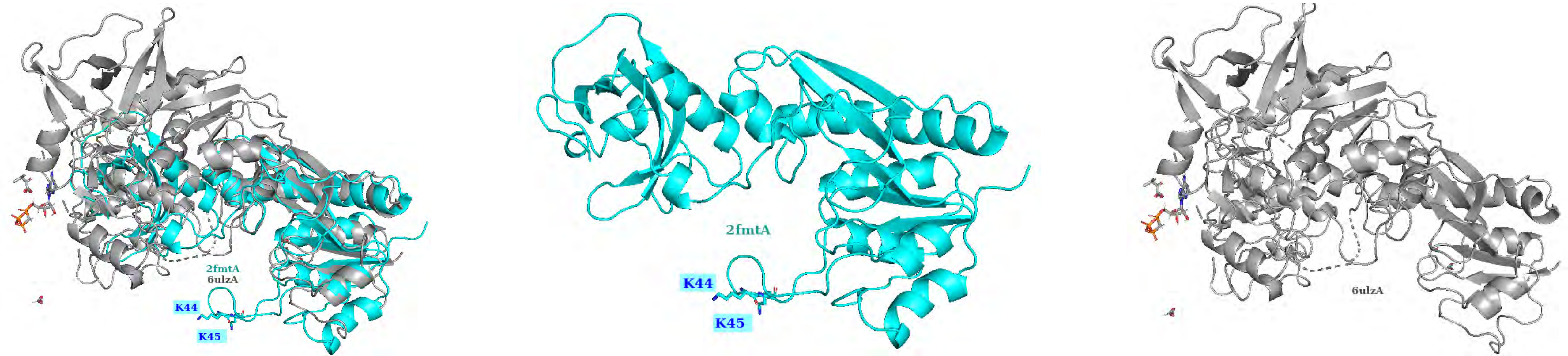

UniProt ID: Q81WH2

PDB ID: 4IQF_B

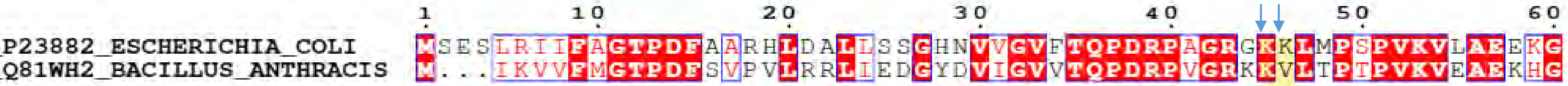

Full sequences in supplemental file.

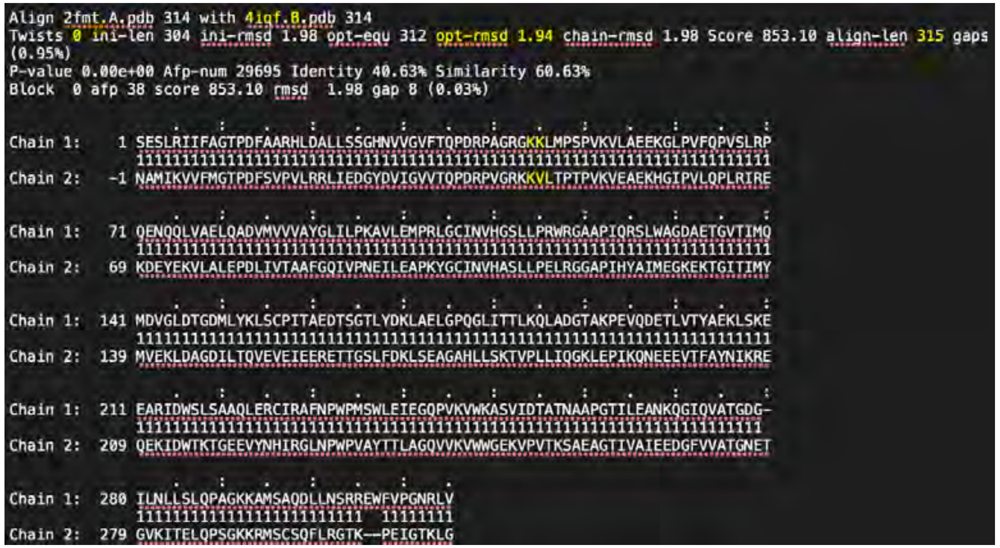

```
Align 2fmt.A.pdb 314 with 4iqf.B.pdb 314
Twists 0 ini-len 304 ini-rmsd 1.98 opt-equ 312 opt-rmsd 1.94 chain-rmsd 1.98 Score 853.10 align-len 315 gaps
(0.95%)
P-value 0.00e+00 Afp-num 29695 Identity 40.63% Similarity 60.63%
Block  0 afp 38 score 853.10 rmsd  1.98 gap 8 (0.03%)

Chain 1:    1 SESLRIIFAGTPDFAARHLDALLSSGHNVVGVFTQPDRPAGRGKKLMPSPVKVLAEEKGLPVFQPVSLRP
              1111111111111111111111111111111111111111111111111111111111111111111111
Chain 2:   -1 NAMIKVVFMGTPDFSVPVLRRLIEDGYDVIGVVTQPDRPVGRKKVLTPTPVKVEAEKHGIPVLQPLRIRE

Chain 1:   71 QENQQLVAELQADVMVVVAYGLILPKAVLEMPRLGCINVHGSLLPRWRGAAPIQRSLWAGDAETGVTIMQ
              1111111111111111111111111111111111111111111111111111111111111111111111
Chain 2:   69 KDEYEKVLALEPDLIVTAAFGQIVPNEILEAPKYGCINVHASLLPELRGGAPIHYAIMEGKEKTGITIMY

Chain 1:  141 MDVGLDTGDMLYKLSCPITAEDTSGTLYDKLAELGPQGLITTLKQLADGTAKPEVQDETLVTYAEKLSKE
              1111111111111111111111111111111111111111111111111111111111111111111111
Chain 2:  139 MVEKLDAGDILTQVEVEIEERETTGSLFDKLSEAGAHLLSKTVPLLIQGKLEPIKQNEEEVTFAYNIKRE

Chain 1:  211 EARIDWSLSAAQLERCIRAFNPWPMSWLEIEGQPVKVWKASVIDTATNAAPGTILEANKQGIQVATGDG-
              111111111111111111111111111111111111111111111111111111111111111111111
Chain 2:  209 QEKIDWTKTGEEVYNHIRGLNPWPVAYTTLAGQVVKVWWGEKVPVTKSAEAGTIVAIEEDGFVVATGNET

Chain 1:  280 ILNLLSLQPAGKKAMSAQDLLNSRREWFVPGNRLV
              11111111111111111111111  11111111
Chain 2:  279 GVKITELQPSGKKRMSCSQFLRGTK--PEIGTKLG
```

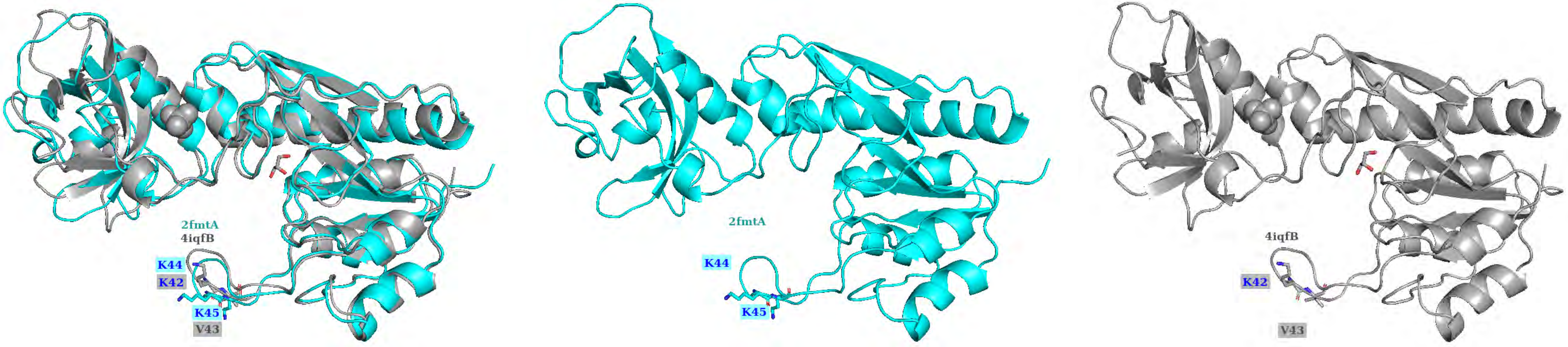

UniProt ID: Q83AA8

PDB ID: 3TQQ_A

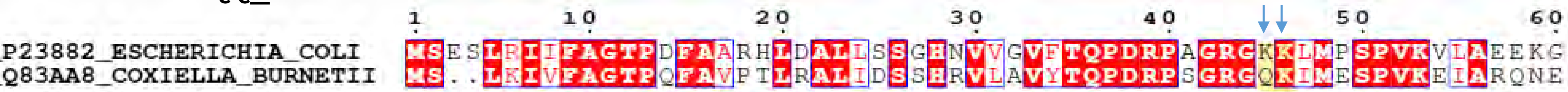

Full sequences in supplemental file.

```
Align 2fmt.A.pdb 314 with 3tqq.A.pdb 304
Twists 0 ini-len 296 ini-rmsd 1.98 opt-equ 303 opt-rmsd 1.98 chain-rmsd 1.98 Score 864.52 align-len 314 gaps 11 (3.50%)
P-value 0.00e+00 Afp-num 28470 Identity 49.36% Similarity 67.52%
Block  0 afp 37 score 864.52 rmsd  1.98 gap 10 (0.03%)

                .    :    .    :    .    :    .    :    .    :    .    :    .    :
Chain 1:    2 ESLRIIFAGTPDFAARHLDALLSSGHNVVGVFTQPDRPAGRGKKLMPSPVKVLAEEKGLPVFQPVSLRPQ
              111111111111111111111111111111111111          111111111111111111111111
Chain 2:    1 MSLKIVFAGTPQFAVPTLRALIDSSHRVLAVYTQPD----------ESPVKEIARQNEIPIIQPFSLRDE

                .    :    .    :    .    :    .    :    .    :    .    :    .    :
Chain 1:   72 ENQQLVAELQADVMVVVAYGLILPKAVLEMPRLGCINVHGSLLPRWRGAAPIQRSLWAGDAETGVTIMQM
              1111111111111111111111111111111111111111111111111111111111111111111111
Chain 2:   71 VEQEKLIAMNADVMVVVAYGLILPKKALNAFRLGCVNVHASLLPRWRGAAPIQRAILAGDRETGISIMQM

                .    :    .    :    .    :    .    :    .    :    .    :    .    :
Chain 1:  142 DVGLDTGDMLYKLSCPITAEDTSGTLYDKLAELGPQGLITTLKQLADGTAKPEVQDETLVTYAEKLSKEE
              1111111111111111111111111111111111111111111111111111111111111111111111
Chain 2:  141 NEGLDTGDVLAKSACVISSEDTAADLHDRLSLIGADLLLESLAKLEKGDIKLEKQDEASATYASKIQKQE

                .    :    .    :    .    :    .    :    .    :    .    :    .    :
Chain 1:  212 ARIDWSLSAAQLERCIRAFNPWPMSWLEIEGQPVKVWKASVIDTATNAAPGTILEANKQGIQVATGDGIL
              1111111111111111111111111111111111111111111111111111111111111111111111
Chain 2:  211 ALIDWRKSAVEIARQVRAFNPTPIAFTYFEGQPMRIWRATVVDEKTDFEPGVLVDADKKGISIAAGSGIL

                .    :    .    :    .    :
Chain 1:  282 NLLSLQPAGKKAMSAQDLLNSRREWFVP-GNRLV
              1111111111111111111111111111 11111
Chain 2:  281 RLHQLQLPGKRVCSAGDFINAHGDKLIPGKTVFG

Note: positions are from PDB; the numbers between alignments are block index
```

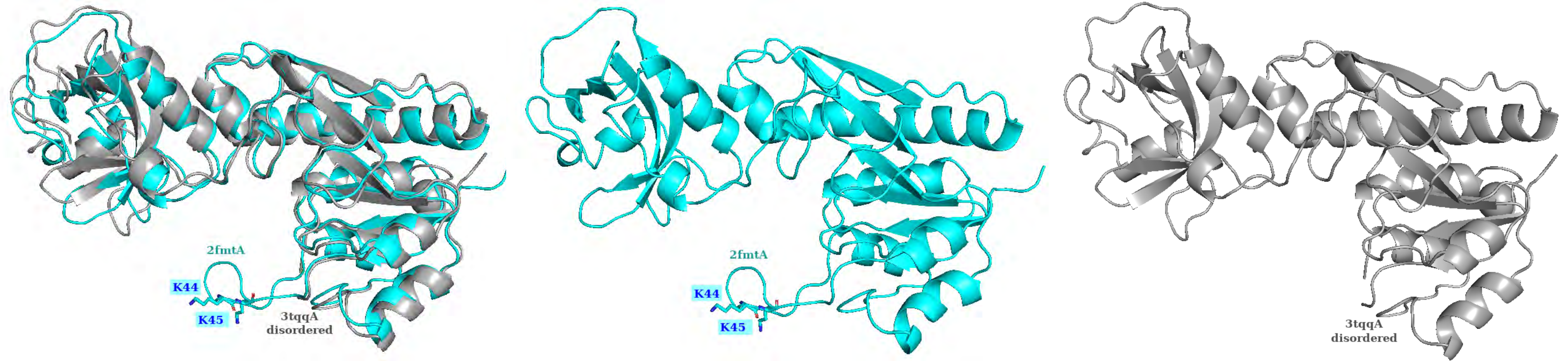

UniProt ID: Q83AY9

PDB ID: 3TQR_A

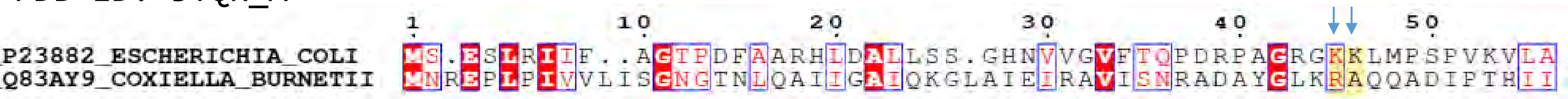

Full sequences in supplemental file.

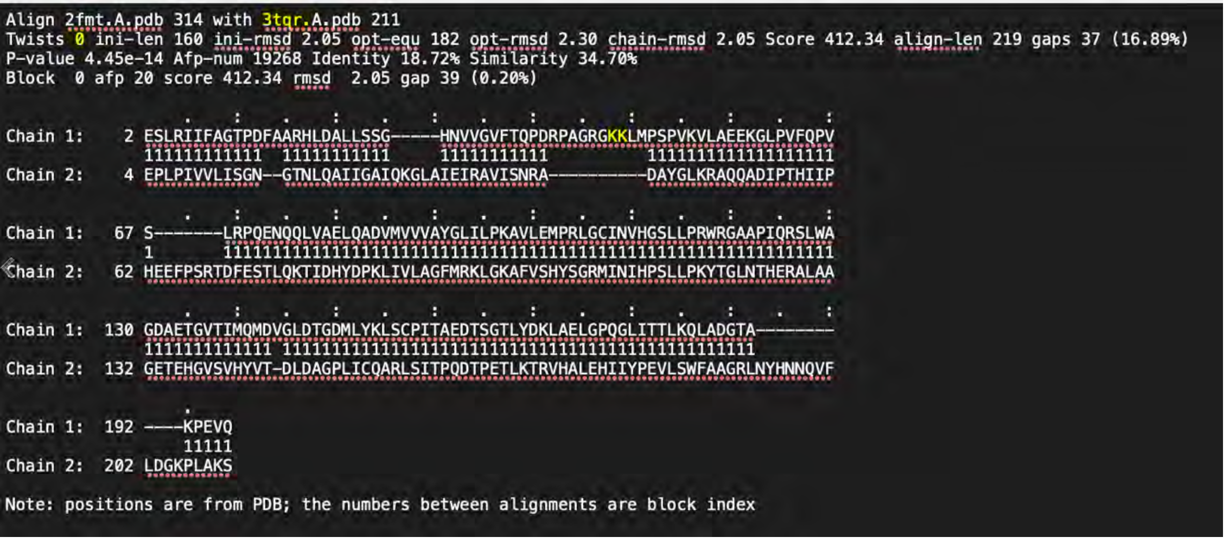

```
Align 2fmt.A.pdb 314 with 3tqr.A.pdb 211
Twists 0 ini-len 160 ini-rmsd 2.05 opt-equ 182 opt-rmsd 2.30 chain-rmsd 2.05 Score 412.34 align-len 219 gaps 37 (16.89%)
P-value 4.45e-14 Afp-num 19268 Identity 18.72% Similarity 34.70%
Block  0 afp 20 score 412.34 rmsd  2.05 gap 39 (0.20%)

                 .    :    .    :    .    :    .    :    .    :    .    :    .    :
Chain 1:    2 ESLRIIFAGTPDFAARHLDALLSSG-----HNVVGVFTQPDRPAGRGKKLMPSPVKVLAEEKGLPVFQPV
              111111111111  11111111111     11111111111          11111111111111111111
Chain 2:    4 EPLPIVVLISGN--GTNLQAIIGAIQKGLAIEIRAVISNRA----------DAYGLKRAQQADIPTHIIP

                 .    :    .    :    .    :    .    :    .    :    .    :    .    :
Chain 1:   67 S-------LRPQENQQLVAELQADVMVVVAYGLILPKAVLEMPRLGCINVHGSLLPRWRGAAPIQRSLWA
              1       111111111111111111111111111111111111111111111111111111111111111
Chain 2:   62 HEEFPSRTDFESTLQKTIDHYDPKLIVLAGFMRKLGKAFVSHYSGRMINIHPSLLPKYTGLNTHERALAA

                 .    :    .    :    .    :    .    :    .    :    .    :    .    :
Chain 1:  130 GDAETGVTIMQMDVGLDTGDMLYKLSCPITAEDTSGTLYDKLAELGPQGLITTLKQLADGTA--------
              11111111111111 111111111111111111111111111111111111111111111111
Chain 2:  132 GETEHGVSVHYVT-DLDAGPLICQARLSITPQDTPETLKTRVHALEHIIYPEVLSWFAAGRLNYHNNQVF

                 .
Chain 1:  192 ----KPEVQ
                  11111
Chain 2:  202 LDGKPLAKS

Note: positions are from PDB; the numbers between alignments are block index
```

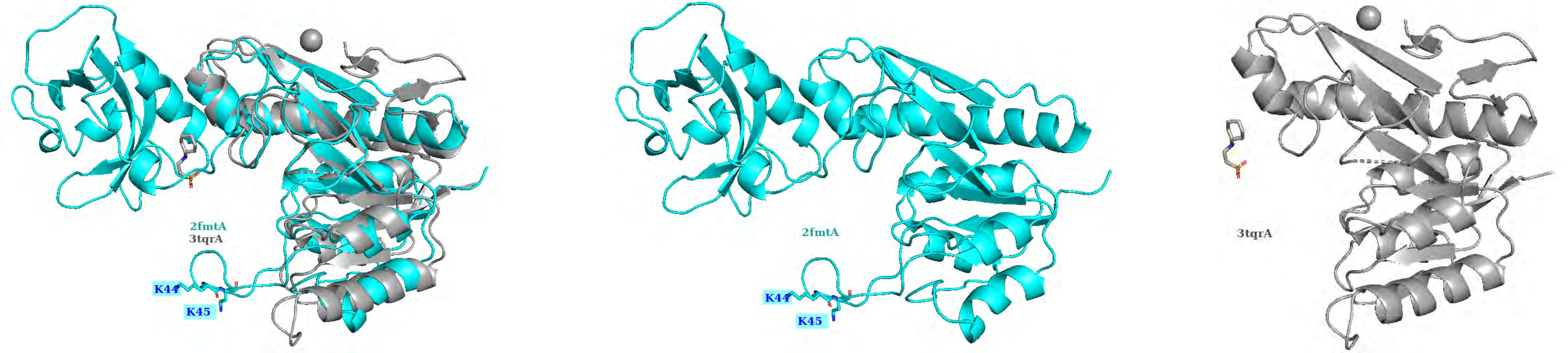

UniProt ID: Q88LI9

PDB ID: 3NRB_A

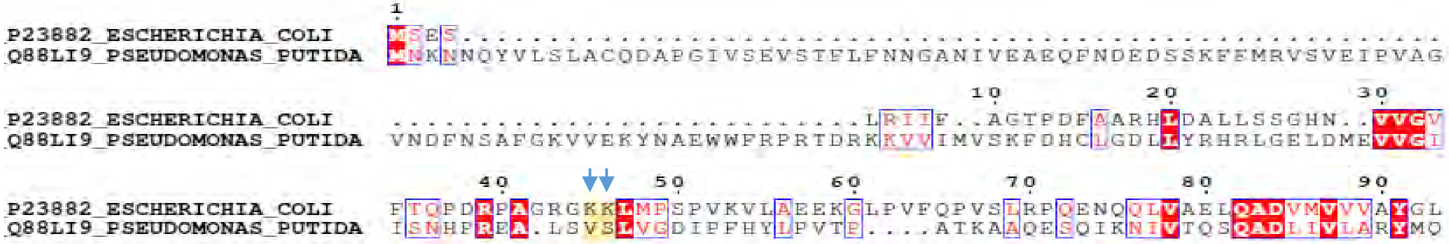

Full sequences in supplemental file.

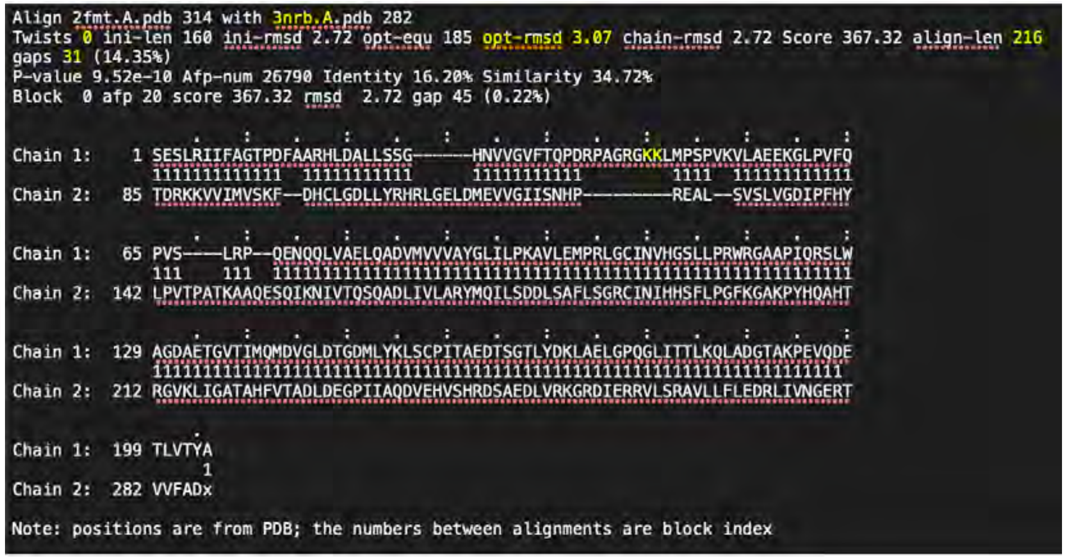

```
Align 2fmt.A.pdb 314 with 3nrb.A.pdb 282
Twists 0 ini-len 160 ini-rmsd 2.72 opt-equ 185 opt-rmsd 3.07 chain-rmsd 2.72 Score 367.32 align-len 216
gaps 31 (14.35%)
P-value 9.52e-10 Afp-num 26790 Identity 16.20% Similarity 34.72%
Block  0 afp 20 score 367.32 rmsd  2.72 gap 45 (0.22%)

Chain 1:    1 SESLRIIFAGTPDFAARHLDALLSSG-------HNVVGVFTQPDRPAGRGKKLMPSPVKVLAEEKGLPVFQ
Chain 2:   85 TDRKKVVIMVSKF--DHCLGDLLYRHRLGELDMEVVGIISNHP---------REAL--SVSLVGDIPFHY

Chain 1:   65 PVS----LRP--QENQQLVAELQADVMVVVAYGLILPKAVLEMPRLGCINVHGSLLPRWRGAAPIQRSLW
Chain 2:  142 LPVTPATKAAQESQIKNIVTQSQADLIVLARYMQILSDDLSAFLSGRCINIHHSFLPGFKGAKPYHQAHT

Chain 1:  129 AGDAETGVTIMQMDVGLDTGDMLYKLSCPITAEDTSGTLYDKLAELGPQGLITTLKQLADGTAKPEVQDE
Chain 2:  212 RGVKLIGATAHFVTADLDEGPIIAQDVEHVSHRDSAEDLVRKGRDIERRVLSRAVLLFLEDRLIVNGERT

Chain 1:  199 TLVTYA
Chain 2:  282 VVFADx

Note: positions are from PDB; the numbers between alignments are block index
```

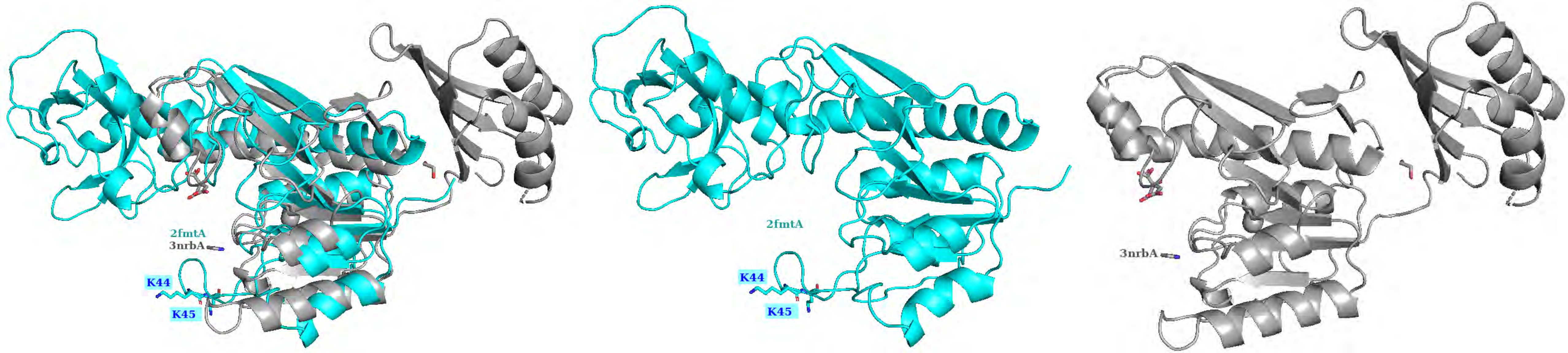

UniProt ID: Q8ZJ80

PDB ID: 3R8X_A

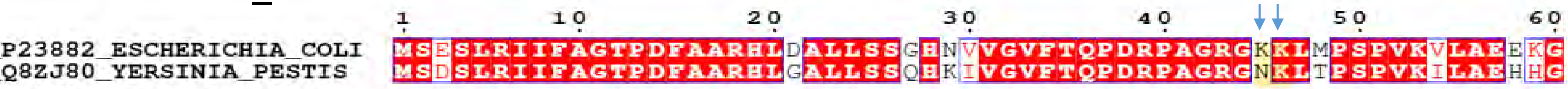

Full sequences in supplemental file.

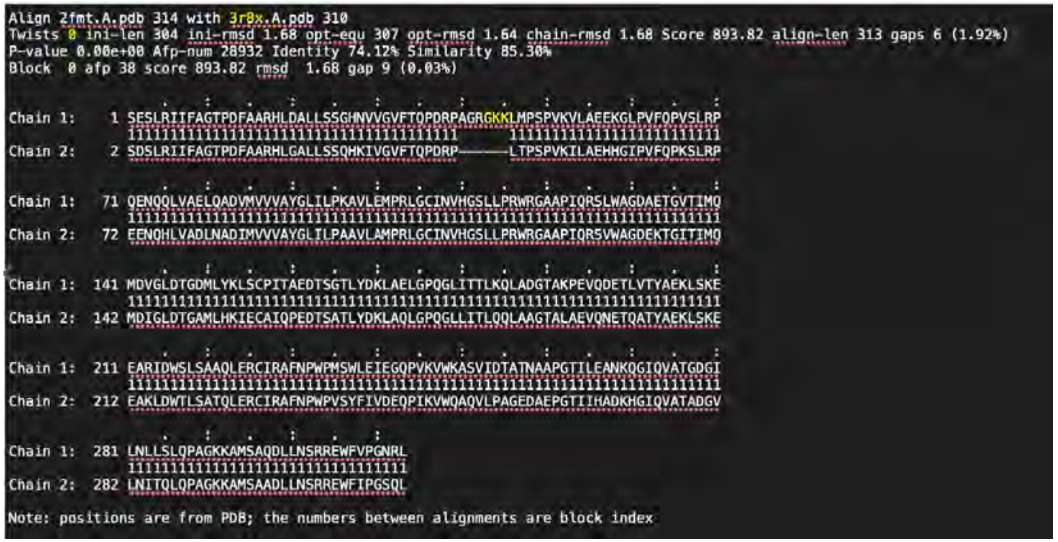

```
Align 2fmt.A.pdb 314 with 3r8x.A.pdb 310
Twists 0 ini-len 304 ini-rmsd 1.68 opt-equ 307 opt-rmsd 1.64 chain-rmsd 1.68 Score 893.82 align-len 313 gaps 6 (1.92%)
P-value 0.00e+00 Afp-num 28932 Identity 74.12% Similarity 85.30%
Block  0 afp 38 score 893.82 rmsd  1.68 gap 9 (0.03%)

                .    :    .    :    .    :    .    :    .    :    .    :    .    :
Chain 1:    1 SESLRIIFAGTPDFAARHLDALLSSGHNVVGVFTQPDRPAGRGKKLMPSPVKVLAEEKGLPVFQPVSLRP
              111111111111111111111111111111111111111      1111111111111111111111111
Chain 2:    2 SDSLRIIFAGTPDFAARHLGALLSSQHKIVGVFTQPDRP------LTPSPVKILAEHHGIPVFQPKSLRP

                .    :    .    :    .    :    .    :    .    :    .    :    .    :
Chain 1:   71 QENQQLVAELQADVMVVVAYGLILPKAVLEMPRLGCINVHGSLLPRWRGAAPIQRSLWAGDAETGVTIMQ
              1111111111111111111111111111111111111111111111111111111111111111111111
Chain 2:   72 EENQHLVADLNADIMVVVAYGLILPAAVLAMPRLGCINVHGSLLPRWRGAAPIQRSVWAGDEKTGITIMQ

                .    :    .    :    .    :    .    :    .    :    .    :    .    :
Chain 1:  141 MDVGLDTGDMLYKLSCPITAEDTSGTLYDKLAELGPQGLITTLKQLADGTAKPEVQDETLVTYAEKLSKE
              1111111111111111111111111111111111111111111111111111111111111111111111
Chain 2:  142 MDIGLDTGAMLHKIECAIQPEDTSATLYDKLAQLGPQGLLITLQQLAAGTALAEVQNETQATYAEKLSKE

                .    :    .    :    .    :    .    :    .    :    .    :    .    :
Chain 1:  211 EARIDWSLSAAQLERCIRAFNPWPMSWLEIEGQPVKVWKASVIDTATNAAPGTILEANKQGIQVATGDGI
              1111111111111111111111111111111111111111111111111111111111111111111111
Chain 2:  212 EAKLDWTLSATQLERCIRAFNPWPVSYFIVDEQPIKVWQAQVLPAGEDAEPGTIIHADKHGIQVATADGV

                .    :    .    :    .    :
Chain 1:  281 LNLLSLQPAGKKAMSAQDLLNSRREWFVPGNRL
              111111111111111111111111111111111
Chain 2:  282 LNITQLQPAGKKAMSAADLLNSRREWFIPGSQL

Note: positions are from PDB; the numbers between alignments are block index
```

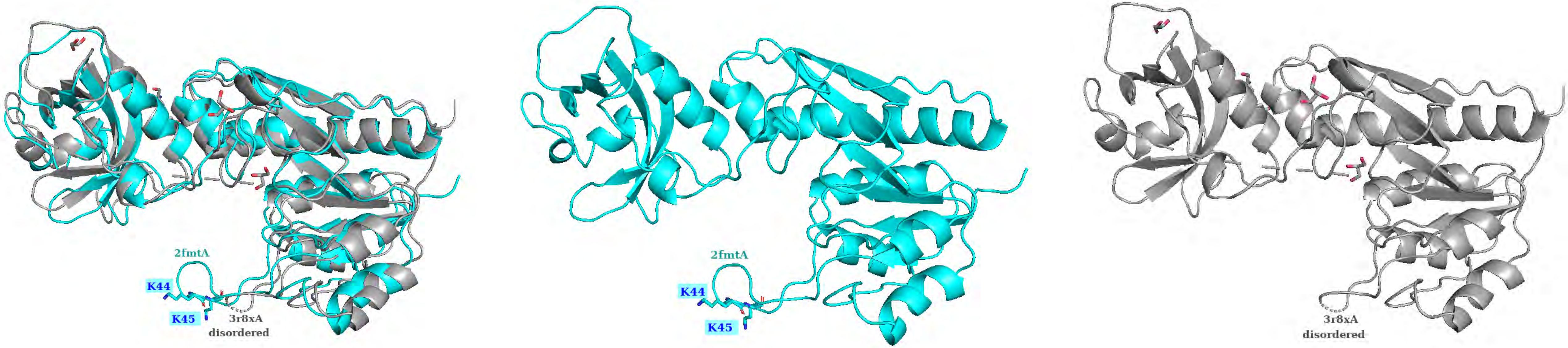

UniProt ID: Q9KF54

PDB ID: 3P9X_B

```
                                1         10        20         30        40         50
P23882_ESCHERICHIA_COLI        MSESLRIIF..AGTPDFAARHLDALLSSGHN..VVGVFTQPDRPAGRGKKLMPSPVKVLA
Q9KF54_BACILLUS_HALODURANS     ...MKRVAIFASGSGTNAEAIIQSQKAGQLPCEVALLITDKPGAKVVERVKVHEIPVCAL
```

Full sequences in supplemental file.

```
Align 2fmt.A.pdb 314 with 3p9x.B.pdb 194
Twists 0 ini-len 152 ini-rmsd 1.58 opt-equ 176 opt-rmsd 2.22 chain-rmsd 1.58 Score 401.51 align-len 201 gaps 25 (12.44%)
P-value 4.44e-14 Afp-num 18118 Identity 22.39% Similarity 38.81%
Block  0 afp 19 score 401.51 rmsd  1.58 gap 44 (0.22%)

Chain 1:    4 LRIIFAGTPDFAARHLDALLSSG-------HNVVGVFTQPDRPAGRGKKLMPSPVKVLAEEKGLPVFQPVS
                111111111  1111111111       11111111111           1111111111111111111
Chain 2:    2 KRVAIFASGS--GTNAEAIIQSQKAGQLPCEVALLITDKP----------GAKVVERVKVHEIPVCALDP

Chain 1:   68 -------LRPQENQQLVAELQADVMVVVAYGLILPKAVLEMPRLGCINVHGSLLPRWRGAAPIQRSLWAG
                     11111111111111111111111111111111111111111111111111111111111111
Chain 2:   60 KTYPSKEAYEIEVVQQLKEKQIDFVVLAGYMRLVGPTLLGAYEGRIVNIHPSLLPAFPGLHAIEQAIRAN

Chain 1:  131 DAETGVTIMQMDVGLDTGDMLYKLSCPITAEDTSGTLYDKLAELGPQGLITTLKQLADGTA
              11111111111111111111111111111111111111111111111111111111111111
Chain 2:  130 VKVTGVTIHYVDEGMDTGPIIAQEAVSIEEEDTLETLTTKIQAVEHRLYPATLHKLLSKAE

Note: positions are from PDB; the numbers between alignments are block index
```

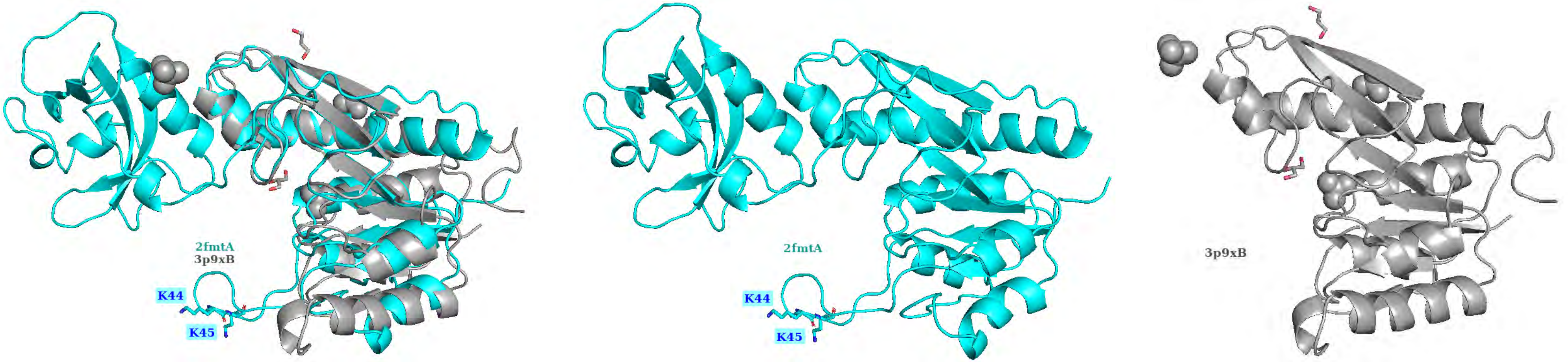

UniProt ID: Q9KVU4

PDB ID: 3Q0I_A

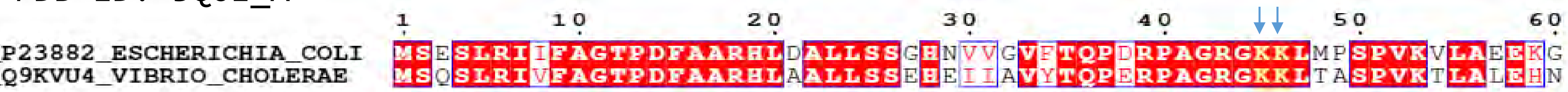

Full sequences in supplemental file.

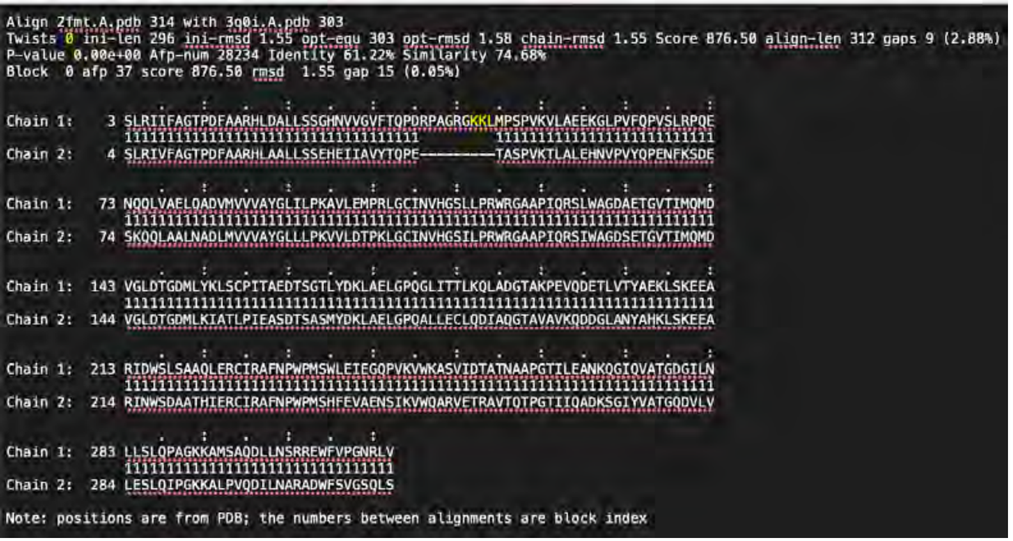

```
Align 2fmt.A.pdb 314 with 3q0i.A.pdb 303
Twists 0 ini-len 296 ini-rmsd 1.55 opt-equ 303 opt-rmsd 1.58 chain-rmsd 1.55 Score 876.50 align-len 312 gaps 9 (2.88%)
P-value 0.00e+00 Afp-num 28234 Identity 61.22% Similarity 74.68%
Block  0 afp 37 score 876.50 rmsd  1.55 gap 15 (0.05%)

                 .    :    .    :    .    :    .    :    .    :    .    :    .    :
Chain 1:    3 SLRIIFAGTPDFAARHLDALLSSGHNVVGVFTQPDRPAGRGKKLMPSPVKVLAEEKGLPVFQPVSLRPQE
              11111111111111111111111111111111111          11111111111111111111111111
Chain 2:    4 SLRIVFAGTPDFAARHLAALLSSEHEIIAVYTQPE----------TASPVKTLALEHNVPVYQPENFKSDE

                 .    :    .    :    .    :    .    :    .    :    .    :    .    :
Chain 1:   73 NQQLVAELQADVMVVVAYGLILPKAVLEMPRLGCINVHGSLLPRWRGAAPIQRSLWAGDAETGVTIMQMD
              1111111111111111111111111111111111111111111111111111111111111111111111
Chain 2:   74 SKQQLAALNADLMVVVAYGLLLPKVVLDTPKLGCINVHGSILPRWRGAAPIQRSIWAGDSETGVTIMQMD

                 .    :    .    :    .    :    .    :    .    :    .    :    .    :
Chain 1:  143 VGLDTGDMLYKLSCPITAEDTSGTLYDKLAELGPQGLITTLKQLADGTAKPEVQDETLVTYAEKLSKEEA
              1111111111111111111111111111111111111111111111111111111111111111111111
Chain 2:  144 VGLDTGDMLKIATLPIEASDTSASMYDKLAELGPQALLECLQDIAQGTAVAVKQDDGLANYAHKLSKEEA

                 .    :    .    :    .    :    .    :    .    :    .    :    .    :
Chain 1:  213 RIDWSLSAAQLERCIRAFNPWPMSWLEIEGQPVKVWKASVIDTATNAAPGTILEANKQGIQVATGDGILN
              1111111111111111111111111111111111111111111111111111111111111111111111
Chain 2:  214 RINWSDAATHIERCIRAFNPWPMSHFEVAENSIKVWQARVETRAVTQTPGTIIQADKSGIYVATGQDVLV

                 .    :    .    :    .    :
Chain 1:  283 LLSLQPAGKKAMSAQDLLNSRREWFVPGNRLV
              11111111111111111111111111111111
Chain 2:  284 LESLQIPGKKALPVQDILNARADWFSVGSQLS

Note: positions are from PDB; the numbers between alignments are block index
```

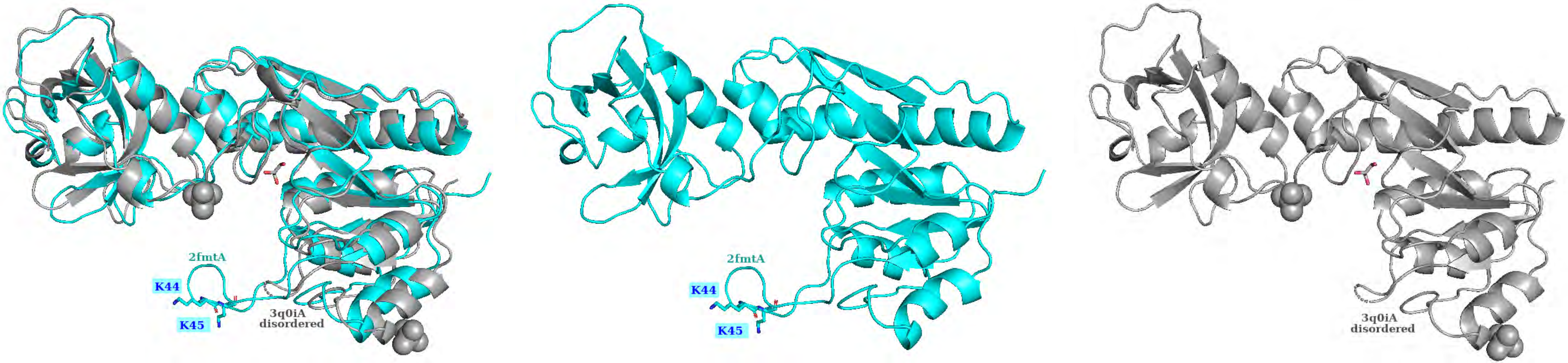

Supplement: Supplementary file 5 [file Data_Sheet_5.PDF]
