# Supplementary material for: Investigation of the Importance of Protein 3D Structure for Assessing Conservation of Lysine Acetylation Sites in Protein Homologs
Source: Front Microbiol. 2022 Jan 31;12:805181. doi: 10.3389/fmicb.2021.805181 (PMC8843374; doi:10.3389/fmicb.2021.805181)
Supplement: Supplementary file 6 [file Data_Sheet_6.PDF]

Supplemental Figure SF3A. Multiple sequence alignment (MSA) of the *E. coli* Adk adenylate kinase target protein (PDB ID: 1ake) and homologs based on 3D structural alignments from FATCAT. Each homolog structure was individually aligned with the Adk target protein in FATCAT and the MSA was generated using a script that compiled individual alignments from the FATCAT xml files into a single alignment based on the target sequence (See Materials and Methods for further details). ESPript was used to visualize the structure-based sequence alignment and lysine residues identified as acetylated on the Adk *E. coli* substrate protein are highlighted in yellow. Note only amino acids that were present in the structural alignment output from FATCAT are shown and do not include residues in disordered regions. Each sequence is identified by the PDB ID and corresponding chain used for the structural alignment. All sequences are organized from highest to lowest sequence similarity based on data within FATCAT xml files.

Adk

```
1ake.A  MRIILLGAPGAGKGTQAQFIMEKYGIPQISTGDMLRAAVKS....G...SE.LGKQAKDIMDAGKLVTDELVIALVKERIA.QED.C....RN.GFL
4jzk.A  MRIILLGAPGAGKGTQAQFIMEKYGIPQISTGDMLRAAVKS....G...SE.LGKQAKDIMDAGKLVTDELVIALVKERIA.QED.C....RN.GFL
3x2s.A  MRIILLGAPGAGKGTQAQFIMEKYGIPQISTGDMLRAAVKS....G...SE.LGKQAKDIMDCGKLVTDELVIALVKERIA.QED.S....RN.GFL
6hap.A  MRIILLGAPGAGKGTQAQFIMEKYGIPQISTGDMLRAAVKS....G...SE.LGKQAKDIMDAGKLVTDELVIALVRERIC.QED.S....RN.GFL
4np6.B  MRIILLGAPGAGKGTQAQFIMEKFGIPQISTGDMLRAAIKA....G...TE.LGKQAKAVIDAGQLVSDDIILGLIKERIA.QAD.C....EK.GFL
4k46.A  MRIILLGAPGAGKGTQAQFIMAKFGIPQISTGDMLRAAIKA....G...TE.LGKQAKSVIDAGQLVSDDIILGLVKERIA.QDD.C....AK.GFL
3gmt.A  MRLILLGAPGAGKGTQANFIKEKFGIPQISTGDMLRAAVKA....G...TP.LGVEAKTYMDEGKLVPDSLIIGLVKERLK.EAD.C....AN.GYL
4pzl.D  MRIILLGAPGAGKGTQAKIIEQKYNIAHISTGDMIRETIKS....GSALGQ.ELKKVLDAG...ELVSDEFIIKIVKDRIS.KND.C....NN.GFL
5g3z.A  MNLVLMGLPGAGKGTQAEKIVEKYGIPHISTGDMFRAAIKE....G...TE.LGLKAKSFMDKGELVPDEVTIGIVRERLS.KDD.C....KK.GFL
5g3y.A  MNLILLGPPGAGKGTQAEKIVEEYGIPHISTGDMFRAAIKE....G...TE.LGLKAKEYMDKGELVPDEVTIGLVKERLS.QPD.C....KK.GFL
5g40.A  MNLVLMGLPGAGKGTQAEKIVEKYGIPHISTGDMFRAAIKE....G...TE.LGLKAKSFMDKGELVPDEVTIGIVRERLS.KDD.C....KK.GFL
3be4.A  HNLILIGAPGSGKGTQCEFIKKEYGLAHLSTGDMLREAIKN....G......IGLEAKSIIESGNFVGDEIVLGLVKEKFD.LGV.C....VN.GFV
1aky.A  IRMVLIGPPGAGKGTQAPNLQERFHAAHLATGDMLRSQIAK....G...TQ.LGLEAKKIMDQGGLVSDDIMVNMIKDELTNNPA.C....KN.GFI
4jl5.A  MILVFLGPPGAGKGTQAKRLAKEKGFVHISTGDILREAVQK....G...TP.LGKKAKEYMERGELVPDDLIIALIEEVFP.....K....HG.NVI
4qbg.B  MNIVLMGLPGAGKGTQAERIVAKYGIPHISTGDMFRAAMKE....E...TP.LGLEAKSYIDKGELVPDEVTIGIVRERLS.KSD.C....ER.GFL
1zin.A  MNLVLMGLPGAGKGTQAEKIVAAYGIPHISTGDMFRAAMKE....G...TP.LGLQAKQYMDRGDLVPDEVTIGIVRERLS.KDD.C....QN.GFL
1s3g.A  MNIVLMGLPGAGKGTQADRIVEKYGTPHISTGDMFRAAIQE....G...TE.LGVKAKSFMDQGALVPDEVTIGIVRERLS.KSD.C....DN.GFL
3fb4.A  MNIVLMGLPGAGKGTQAEQIIEKYEIPHISTGDMFRAAIKN....G...TE.LGLKAKSFMDQGNLVPDEVTIGIVHERLS.KDD.C....QK.GFL
3tlx.A  GRYIFLGAPGSGKGTQSLNLKKSHCYCHLSTGDLLREAAEK....K...TE.LGLKIKNIINEGKLVDDQMVLSLVDEKLK.TPQ.C....KK.GFI
1zak.B  LKVMISGAPASGKGTQCELIKTKYQLAHISAGDLLRAEIAA....G...SE.NGKRAKEFMEKGQLVPDEIVVNMVKERLR.QPD.A....QENGWL
4nu0.B  MNLLIMGLPGAGKGTQAAKIVEQFHVAHISTGDMFRAAMAN....Q...TE.MGVLAKSYIDKGELVPDEVTNGIVKERLS.QDD.I....KETGFL
2ak3.A  LRAAIMGAPGSGKGTVSSRITKHFELKHLSSGDLLRDNMLR....G...TE.IGVLAKTFIDQGKLIPDDVMTRLVLHELK.N.L.T....QY.NWL
1ak2.A  VRAVLLGPPGAGKGTQAPKLAKNFCVCHLATGDMLRAMVAS....G...SE.LGKKLKATMDAGKLVSDEMVLELIEKNLE.TPP.C....KN.GFL
6zje.A  LRAVIMGAPGSGKGTVSSRITTHFELKHLSSGDLLRDNMLR....G...TE.IGVLAKAFIDQGKLIPDDVMTRLALHELK.N.L.T....QY.SWL
2c9y.A  IRAVLLGPPGAGKGTQAPRLAENFCVCHLATGDMLRAMVAS....G...SE.LGKKLKATMDAGKLVSDEMVVELIEKNLE.TPL.C....KN.GFL
2bbw.B  LRAVILGPPGSGKGTVCQRIAQNFGLQHLSSGHFLRENIKA....S...TE.VGEMAKQYIEKSLLVPDHVITRLMMSELE.NR..R....GQ.HWL
2cdn.A  MRVLLLGPPGAGKGTQAVKLAEKLGIPQISTGELFRRNIEE....G...TK.LGVEAKRYLDAGDLVPSDLTNELVDDRLN.NPD.A....AN.GFI
5xz2.A  KIVFVVGGPGSGKGTQCEKIVAKYGYTHLSSGDLLRAEVAS....G...SE.RGKQLQAIMQKGELVPLDTVLDMIKDAMI.AKA.D....VSKGYL
3cm0.A  QAVIFLGPPGAGKGTQASRLAQELGFKKLSTGDILRDHVAR....G...TP.LGERVRPIMERGDLVPDDLILELIREE.......L....AE.RVI
5x6k.A  KIIFVVGGPGSGKGTQCEKVVAKYGYTHLSSGDLLRAEVSS....G...SE.RGKQLQAIMQKGELVPLDTVLDMIKDAMI.AKADV....SK.GYL
5ycd.A  KIIFVVGGPGSGKGTQCEKIVAKYGYTHLSSGDLLRAEVAS....G...SE.RGKQLQAIMQKGELVPLDTVLDMIKDAMI.AKADV....SK.GFL
5ycf.B  KIIFVVGGPGSGKGTQCEKIVAKYGYTHLSSGDLLRAEVAS....G...SE.RGKQLQAIMQKGELVPLDTVLDMIKDAMI.AKADV....SK.GFL
2c95.A  NIIFVVGGPGSGKGTQCEKIVQKYGYTHLSTGDLLRSEVSS....G...SA.RGKKLSEIMEKGQLVPLETVLDMLRDAMV.AKVNT....SK.GFL
3adk.A  KIIFVVGGPGSGKGTQCEKIVQKYGYTHLSTGDLLRAEVSS....G...SA.RGKMLSEIMEKGQLVPLETVLDMLRDAMV.AKV.DT...SK.GFL
3l0s.C  MNILIFGPNGSGKGTQGNLVKDKYSLAHIESGGIFREHIGG....G...TE.LGKKAKEFIDRGDLVPDDITIPMVLETLE.S.K.G....KD.GWL
1tev.A  LVVFVLGGPGAGKGTQCARIVEKYGYTHLSAGELLRDERKN....P...DSQYGELIEKYIKEGKIVPVEITISLLKREMD.QTM.AANAQKN.KFL
3umf.A  KVIFVLGGPGSGKGTQCEKLVQKFHFNHLSSGDLLRAEVQSGSPKG...KE.LKAMMERGE....LVPLEVVLALLKEAMI.KLV.DK...NC.HFL
2bwj.A  KIIFIIGGPGSGKGTQCEKLVEKYGFTHLSTGELLREELAS....ESERSK.LIRDIMERG...DLVPSGIVLELLKEAMV.ASL.GD...TR.GFL
```

```
                                                                                K136     K141 K145          K157
1ake.A  LDGFPRTIPQADAMKEA...G....I....NVD....YVLEFDVPDELIVDRIVGRRVHA.PSGRVYHVKFN.PPKVEGKDDVTGEELTTRKDDQ.E
4jzk.A  LDGFPRTIPQADAMKEA...G....I....NVD....YVLEFDVPDELIVDRIVGRRVHA.PSGRVYHVKFN.PPKVEGKDDVTGEELTTRKDDQ.E
3x2s.A  LDGFPRTIPQADAMKEA...G....I....NVD....YVLEFDVPDELIVDRIVGRRVHA.PSGRVYHVKFN.PPKVEGKDDVTGEELTTRKDDQ.E
6hap.A  LDGFPRTIPQADAMKEA...G....I....NVD....YVLEFDVPDELIVDRIVGRRVHA.PSGRVYHVKFN.PPKVEGKDDVTGEELTTRKDDQ.E
4np6.B  LDGFPRTIPQADGLKEM...G....I....NVD....YVIEFDVADDVIVERMAGRRAHL.PSGRTYHVVYN.PPKVEGKDDVTGEDLVIREDDK.E
4k46.A  LDGFPRTIPQADGLKEV...G....V....VVD....YVIEFDVADSVIVERMAGRRAHL.ASGRTYHNVYN.PPKVEGKDDVTGEDLVIREDDK.E
3gmt.A  FDGFPRTIAQADAMKEA...G....V....AID....YVLEIDVPFSEIIERMSGRRTHP.ASGRTYHVKFN.PPKVEGKDDVTGEPLVQRDDDK.E
4pzl.D  LDGVPRTIPQAQELDKL...G....V....NID....YIVEVDVADNLLIERITGRRIHP.ASGRTYHTKFN.PPKVADKDDVTGEPLITRTDDN.E
5g3z.A  LDGFPRTVAQAEALDNI...LKELGK....KLD....YVINIEVPKEELMERLTGRRICK.TCGATYHLIFN.PPKVEGVCDKCGGELYQRADDN.E
5g3y.A  LDGFPRTVAQAEALDKI...L....KELGIKLD....AVINIEVPREELLERLTGRRVCR.QCGATYHVIFN.PPKVEGVCDKCGGELYQRSDDN.E
5g40.A  LDGFPRTVAQAEALDNI...L....SELGKKLD....YVINIEVPKEELMERLTGRRICK.TCGATYHLIFN.PPKVEGICDKDGGELYQRADDN.P
3be4.A  LDGFPRTIPQAEGLAKI...L....SEIGDSLT....SVIYFEIDDSEIIERISGRCTHP.ASGRIYHVKYN.PPKQPGIDDVTGEPLVWRDDDN.A
1aky.A  LDGFPRTIPQAEKLDQM...L....KEQGTPLE....KAIELKVDDELLVARITGRLIHP.ASGRSYHKIFN.PPKEDMKDDVTGEALVQRSDDN.A
4jl5.A  FDGFPRTVKQAEALDEM...LEKKGL....KVD....HVLLFEVPDEVVIERLSGRRINP.ETGEVYHVKYN.PPPP.......GVKVIQREDDK.P
4qbg.B  LDGFPRTVAQAEALEEI...LEEMGR....KLE....HVIHIEVRQEELMERLTGRRICS.VCGTTYHLVFN.PPKTPGICDKDGGELYQRADDN.E
1zin.A  LDGFPRTVAQAEALETM...LADIGR....KLD....YVIHIDVRQDVLMERLTGRRICR.NCGATYHLIFH.PPAKPGVCDKCGGELYQRADDN.E
1s3g.A  LDGFPRTVPQAEALDQL...L....ADMGRKIE....HVLNIQVEKEELIARLTGRRICK.VCGTSYHLLFN.PPQVEGKCDKDGGELYQRADDN.P
3fb4.A  LDGFPRTVAQADALDSL...L....TDLGKKLD....YVLNIKVEQEELMKRLTGRWICK.TCGATYHTIFN.PPAVEGICDKDGGELYQRIDDK.P
3tlx.A  LDGYPRNVKQAEDLNKL...LQKNQT....KLD....GVFYFNVPDEVLVNRISGRLIHK.PSGRIYHKIFN.PPKVPFRDDVTNEPLIQREDDN.E
1zak.B  LDGYPRSYSQAMALETL...E....I....RPD....TFILLDVPDELLVERVVGRRLDP.VTGKIYHLKYS.PPENEEIA....SRLTQRFDDT.E
4nu0.B  LDGYPRTIEQAHALDKT...LAELGI....ELE....GIINIEVNPDSLLERLSGRIIHR.VTGETFHKVFN.PPVDYKE.....EDYYQREDDK.P
2ak3.A  LDGFPRTLPQAEALDRA.......Y....QID....TVINLNVPFEVIKQRLTARWIHP.GSGRVYNIEFN.PPKTMGIDDLTGEPLVQREDDR.P
1ak2.A  LDGFPRTVRQAEMLDDL...M....EKRKEKLD....SVIEFSIPDSLLIRRITGRLIHP.QSGRSYHEEFN.PPKEPMKDDITGEPLIRRSDD...
6zje.A  LDGFPRTLPQAEALDRA.......Y....QID....TVINLNVPFEVIKQRLTARWIHP.ASGRVYNIEFN.PPKTVGIDDLTGEPLIQREDDK.P
2c9y.A  LDGFPRTVRQAEMLDDL...M....EKRKEKLD....SVIEFSIPDSLLIRRITGRLIHP.KSGRSYHEEFN.PPKEPMKDDITGEPLIRRSDD...
2bbw.B  LDGFPRTLGQAEALDK....I....C....EVD....LVISLNIPFETLKDRLSRRWIHP.PSGRVYNLDFN.PPHVHGIDDVTGEPLVQQEDDK.P
2cdn.A  LDGYPRSVEQAKALHEM...L....ERRGTDID....AVLEFRVSEEVLLERLKGR.................................GRADDT.D
5xz2.A  IDGYPREVKQGEEFEKK...I....G....APA....LLLYIDAKGETMVKRLMKRGE.............................TSGRADDN.E
3cm0.A  FDGFPRTLAQAEALDRL...L....SETGTRLL....GVVLVEVPEEELVRRILRRAE.............................LEGRSDDN.E
5x6k.A  IDGYPREVKQGEEFEKK...I....G....KPC....LLLYVDAKGETMVKRLMKRGE.............................TSGRADDN.E
5ycd.A  IDGYPREVKQGEEFEKK...I....G....KPC....LLLYVDAKAETMVKRLLKRGE.............................TSGRSDDN.E
5ycf.B  IDGYPREVKQGEEFEKK...I....G....KPC....LLLYVDAKAETMVKRLLKR..................................RSDDN.E
2c95.A  IDGYPREVQQGEEFERR...I....G....QPT....LLLYVDAGPETMTQRLLKRGE.............................TSGRVDDN.E
3adk.A  IDGYPREVKQGEEFERK...I....G....QPT....LLLYVDAGPETMTKRLLKRGE.............................TSGRVDDN.E
3l0s.C  LDGFPRNTVQAQKLFEA...L....Q....EKGMKINFVIEILLPREVAKNRIMGRRICKNNPNHPNNIFIEAIKPNGDVCRVCGGALSARADDQDE
1tev.A  IDGFPRNQDNLQGWNKTMDGK....A....DVS....FVLFFDCNNEICIERCLERG..........KS...................SGRSDDN.R
3umf.A  IDGYPRELDQGIKFEKE...V....C....PCL....CVINFDVSEEVMRKRLLKRVDD...................................N.E
2bwj.A  IDGYPREVKQGEEFGRR...I....G....DPQ....LVICMDCSADTMTNRLLQMSRSS.LPVDD............................T.T
```

```
                                                       K192                K211
                                                         .                   .
1ake.A  ETVRKRLVEYHQ...MTAPLIGYYSKEAEA......G..NTKYAKVDGTKPVAEVRADLEKILG
4jzk.A  ETVRKRLVEYHQ...MTAPLIGYYSKEAEA......G..NTKYAKVDGTKPVAEVRADLEKILG
3x2s.A  ETVRKRLCEYHQ...MTAPLIGYYSKEAEA......G..NTKYAKVDGTKPVAEVRADLEKILG
6hap.A  ETVRKRLVEYHQ...MTAPLIGYYSKEAEA......G..NTKYAKVDGTKPVCEVRADLEKILG
4np6.B  ETVRARLNVYHT...QTAPLIEYYGKEAAA......G..KTQYLKFDGTKQVSEVSADIAKALA
4k46.A  ETVLARLGVYHN...QTAPLIAYYGKEAEA......G..NTQYLKFDGTKAVAEVSAELEKALA
3gmt.A  ETVKKRLDVYEA...QTKPLITYYGDWARRGAENGLK..APAYRKISG................
4pzl.D  DTVKQRLSVYHA...QTAKLIDFYRNFSST......NTKIPKYIKINGDQAVEKVSQDIFDQLN
5g3z.A  ETVANRLDVNMK...QTQPLLDFYEEKG.............YLRNIDGQQDINKVFADIDALLG
5g3y.A  ETVSNRLDVYED...QTAPLIDYYEKKG.............LLKNIDGDQDIDAVFADIKAALG
5g40.A  ETVANRLDVNMK...QTQPLLDFYEEKG.............VLRNIDGQQDINKVFADIKALLG
3be4.A  EAVKVRLDVFHK...QTAPLVKFYEDLG.............ILKRVNAKLPPKEVTEQIKKIL.
1aky.A  DALKKRLAAYHA...QTEPIVDFYKKTG.............IWAGVDASQPPATVWADILNKLG
4jl5.A  EVIKKRLEVYRE...QTAPLIEYYKKKG.............ILRIIDASKPVEEVYRQVLEVIG
4qbg.B  ETVAKRLEVNMK...QMAPLLAFYDSKE.............VLRNVNGQQDMEKVFKDLRELLQ
1zin.A  ATVANRLEVNMK...QMKPLVDFYEQKG.............YLRNINGEQDMEKVFADIRELLG
1s3g.A  DTVTNRLEVNMN...QTAPLLAFYDSKE.............VLVNINGQKDIKDVFKDLDVILQ
3fb4.A  ETVKNRLDVNMK...QTQPLLDFYSQKG.............VLKDIDGQQDIKKVFVDINDLLG
3tlx.A  DVLKKRLTVFKS...ETSPLISYYKNKN.............LLINLDATQPANDLEKKISQHID
1zak.B  EKVKLRLETYYQ...NIESLLSTYEN...............IIVKVQGDATVDAVFAKIDELLG
4nu0.B  ETVKRRLDVNIA...QGEPIIAHYRAKG.............LVHDIEGNQDINDVFSDIEKVLT
2ak3.A  ETVVKRLKAYEA...QTEPVLEYYRKKG.............VLETFSGT.ETNKIWPHVYAFLQ
1ak2.A  .NKKALKIRLEA...YHTQTTPLVEYYSKR......G....IHSAIDASQTPDVVFASILAAFS
6zje.A  ETVIKRLKAYED...QTKPVLEYYQKKG.............VLETFSGT.ETNKIWPYVYAFLQ
2c9y.A  .NEKALKIRLQA...YHTQTTPLIEYYRKR......G....IHSAIDASQTPDVVFASILAAFS
2bbw.B  EAVAARLRQYKD...VAKPVIELYKSRG.............VLHQFSGTE.TNKIWPYVYTLFS
2cdn.A  DVILNRMKVYRD...ETAPLLEYYRD...............QLKTVDAVGTMDEVFARALRALG
5xz2.A  ETIKKRLDLYYK...ATEPVIAFYEQRG.............IVRKINSELPVDEVFAIVEKAID
3cm0.A  ETVRRRLEVYRE...KTEPLVGYYEARG.............VLKRVDGLGTPDEVYARIRAALG
5x6k.A  ETIKKRLDLYYK...ATEPVIAFYEGRG.............IVRKVDSELPVDEVFKQVSTAID
5ycd.A  ETIKKRLDLYYK...ATEPVIAFYEGRG.............IVKKVDSELAVDDVFGQVSKAID
5ycf.B  ETIKKRLDLYYK...ATEPVIAFYEGRG.............IVKKVDSELAVDDVFAQVSKAID
2c95.A  ETIKKRLETYYK...ATEPVIAFYEKRG.............IVRKVNAEGSVDSVFSQVCTHLD
3adk.A  ETIKKRLETYYK...ATEPVIAFYEKRG.............IVRKVNAEGSVDDVFSQVCTHLD
3l0s.C  GAINKRHDIYYNTVDGTLAAAYYYKNMAAK......E..GFVYIELDGEGSIDSIKDTLLAQLA
1tev.A  ESLEKRIQTYLQ...STKPIIDLYEEMG.............KVKKIDASKSVDEVFDEVVQIFD
3umf.A  ETIVKRFRTFNE...LTKPVIEHYKQQN.............KVITIDASGTVDAIFDKVNHELQ
2bwj.A  KTIAKRLEAYYR...ASIPVIAYYETKT.............QLHKINAEGTPEDVFLQLCTAID
```

Supplemental Figure SF3B. Multiple sequence alignment (MSA) of the *E. coli* Icd-isocitrate dehydrogenase target protein (PDB ID: 1ai2) and homologs based on 3D structural alignments from FATCAT. Each homolog structure was individually aligned with the Icd target protein in FATCAT and the MSA was generated using a script that compiled individual alignments from the FATCAT xml files into a single alignment based on the target sequence (See Materials and Methods for further details). ESPript was used to visualize the structure-based sequence alignment and lysine residues identified as acetylated on the Icd *E. coli* substrate protein are highlighted in yellow. Note only amino acids that were present in the structural alignment output from FATCAT are shown and do not include residues in disordered regions. Each sequence is identified by the PDB ID and corresponding chain used for the structural alignment. All sequences are organized from highest to lowest sequence similarity based on data within FATCAT xml files.

Icd

```
            K4        K12
            .         .
1ai2.A  SKVVVPAQGKKITLQN.GKLNVP...ENPIIPYIEGDGIGVDVTPAMLKVVDAAVEKAYKGERKISWMEIYTGEKSTQVYGQDVWLPAETLDLIREY
5m2e.D  QKIQVPATGDKITVNADMSLSVP...KNPIIPFIEGDGIGVDISPVMIKVVDAAVEKAYKGERKIAWMEVYAGEKATQVYDQDTWLPQETLDAVRDY
6c0e.B  DKIKVPAQGEAITV....SLHVP...DNPIIPFIEGDGIGVDVTPPMIRVVDAAVQKAYGNKRKISWMEVYAGEKATKVYGGDQWLPKETLDAMKKY
3dms.A  QHIKVPEGGDKITVNKDFSLNVS...DQPIIPYIEGDGTGFDITPVMIKVVDAAVEKAYGGKKKIHWMEIYAGEKATKVYGPDVWLPEETLQVLKEY
1hqs.B  .....MAQGEKITVSN.GVLNVP...NNPIIPFIEGDGTGPDIWNAASKVLEAAVEKAYKGEKKITWKEVYAGEKAYNKTG..EWLPAETLDVIREY
2d4v.C  THIQKPATGSPLTLLN.GVLQVP...DQPIIPFIEGDGIGCDVTPAMRSVVDAAVAKVYGGQRQIAWMELFAGQKAVQLYGEGQYLPDETMAAIREY
2iv0.B  EKVKPPENGEKIRYEN.GKLIVP...DNPIIPYFEGDGIGKDVVPAAIRVLDAAADKIG...KEVVWFQVYAGEDAYKLYG..NYLPDDTLNAIKEF
2e0c.A  MLYKEPEDGEKIKFDK.GKWIVP...NKPVILYIEGDGIGPEITNAAIKVINKAVERAYGSSREIKWLEVYAGEKAEKLVN..DRFPKETQEMLLKY
1tyo.A  EELSPPPGGSLVEYSG.GSLRVP...DNPVVAFIRGDGVGPEVVESALKVVDAAVKKVYGGSRRIVWWELLAGHLAREKCG..ELLPKATLEGIRLA
2d1c.B  .........PLITTET.GKKMHVLEDGRKLITVIPGDGIGPECVEATLKVLEAAKA.......PLAYEVREAGASVFR.RGIASGVPQETIESIRKT
3asj.C  ...........................AYRICLIEGDGIGHEVIPAARRVLEATG.......LPLEFVEAEAGWETFE..RRGTSVPEETVEKILSC
1x0l.A  ...........................AYRICLIEGDGIGHEVIPAARRVLEATG.......LPLEFVEAEAGWETFE..RRGTSVPEETVEKILSC
6l59.A  ...........................VQTVTLIPGDGIGPEISAAVMKIFDAAKA.......PIQWEERNVTA.......IQWMIPSEAKESMDKN
3blx.O  ......................G...GRFTVTLIPGDGVGKEITDSVRTIFEAENI.......PIDWETINIKQTD......HKEGVYEAVESLKRN
5hn3.A  ...........................MYRVAVIPGDGIGPEVIDGAVRVLKAVT.......GRVRFEYYEGGVDVFQ..ECGSPIREEDLEEIRRS
```

```
                                                                                          K174  K177
                                                                                          .  .    .
1ai2.A  RVAIKGPLTTP..VG.GGIRSLNVALRQELDLYICLRPVRYYQGTPSPVKHPELTDMVIFRENSEDIYAGIEWKADSADAEKVIKFLREEMGVKKIR
5m2e.D  VVSIKGPLTTP..VG.GGIRSLNVALRQQLDLYVCQRPVRWFEGVPSPVKKPGDVDMVIFRENSEDIYAGVEWKAGSPEAEKVIKFLTEEMGVKKIR
6c0e.B  VVSIKGPLTTP..VG.GGIRSLNVAIRQDMDLYVCLRPIRYFNGVPSPVREPWKTDMVIFRENSEDIYAGIEWQADTPEAKKVIQFLTKEMGVKKIR
3dms.A  VVSIKGPLTTP..V.....RSLNVALRQELDLYVCLRPIQYFKGVPSPVREPEKTNMVIFRENSEDIYAGIEWAAESEQAKKVIKFLQEEMGVKKIR
1hqs.B  FIAIKGPLTTP..VG.GGIRSLNVALRQELDLFVXLRPVRYFTGVPSPVKRPEDTDMVIFRENTEDIYAGIEYAKGSEEVQKLISFLQNELNVNKIR
2d4v.C  KVAIKGPLETP..VG.GGIRSLNVAMRQDLDLYVCLRPVRYFEGTPSPMRHPEKVDMVIFRENSEDIYAGIEWPAGSPEAEKIIRFLREEMGVTKIR
2iv0.B  RVALKGPLTTP..VG.GGYRSLNVTIRQVLDLYANVRPVYYLKGVPSPIKHPEKVNFVIFRENTEDVYAGIEWPRGSEEALKLIRFLKNEFGVTI..
2e0c.A  RVVLKGPLES...........VNVAIRLMLDLYANIRPVKYIEGLESPLKHPEKVDMIIFRENTDDLYRGIEYPFNSEEAKKIRDFLRKELKVEIED
1tyo.A  RVALKGPLETP..VG.TGYRSLNVAIRQALDLYANIRPVRYYG.QPAPHKYADRVDMVIFRENTEDVYAGIEWPHDSPEAARIRRFLAEEFGISI..
2d1c.B  RVVLKGPLETP..VG.YGEKSANVTLRKLFETYANVRPVREFPNVPT.PYAGRGIDLVVVRENVEDLYAGIEHMQTP....................
3asj.C  HATLFGAATSPTRKV.PGFFGAIRYLRRRLDLYANVRPAKSRPVPGS....RPGVDLVIVRENTEGLYVEQERRYL.....................
1x0l.A  HATLFGAATSPTRKV.PGFFGAIRYLRRRLDLYANVRPAKSRPVPGS....RPGVDLVIVRENTEGLYVEQERR.......................
6l59.A  KMGLKGPLKT.........PSMNLLLRKTFDLYANVRPCVSIEGYKTP...YTDVNIVTIRENTEGEYSGIEH......VI................
3blx.O  KIGLKGLWHTP..ADQTGHGSLNVALRKQLDIYANVALFKSLKGVKTR...IPDIDLIVIRENTEGEFSGLEHESV.....................
5hn3.A  DAVLFGATTTP..FDLPGYRSLILTLRKELGLYANLRIIPDLR.........TGREIVIVRENSEGLYFGIGA........................
```

```
                                              K235                                        K265
                                              .                                            .
1ai2.A  FPEHCGIGIKPCSEEGTKRLVRAAIEYAIANDRDSVTLVHKGNIMKFTEGAFKDWGYQLAREEFG..GELIDGGPW.............LKVKNPNT
5m2e.D  FTENCGIGIKPVSQEGTKRLVRKALQYAVDNDRSSVTLVHKGNIMKFTEGAFKDWGYEVARDEFG..AELLDGGPW.............MQFKNPKT
6c0e.B  FPEHCGIGVKPVSREGTTRLVKAAIQYAIDNDRSTVTLVHKGNIMKFTEGAFKDWGYQVARDSFG..AKEYQGGPW.............MEFKNPKT
3dms.A  FPQTSGIGIKPVSKEGTERLVRKAIQYAIDNDRKSVTLVHKGNIMKFTEGAFRDAGYALAQKEFG..AELIDGGPW.............MKFKNPKT
1hqs.B  FPETSGIGIKPVSEEGTSRLVRAAIDYAIEHGRKSVTLVHKGNIMKFTEGAFKNWGYELAEKEYGDKVFTWAQYDRIAEEQGKDAANKAQSEAE..A
2d4v.C  FPDSSAIGIKPVSTEGSERLIRRTIQYALEHGKPSVSLVHKGNIMKFTEGGFRDWGYALAEREFAGRVFTWRQKAAISKAEGKAAGQKAEQQAI..A
2iv0.B  .REDSGIGIKPISEFATKRLVRMAIRYAIENNRKSVTLVHKGNIMKYTEGAFRDWGYEVAKQEFGEYCITEDELWD.............KYGGKQ.P
2e0c.A  DT...GIGIKVMSKYKTQRITRLAIQYAIEHKRKKVTIMHKGNVMKYTEGAFREWAYEVALKEYRDFIVTEEEINQ............GKPD....
1tyo.A  .REDAGIGVKPISRFATRRLMERALEWALRNGNTVVTIMHKGNIMKYTEGAFMRWAYEVALEKFREHVVTEQEVQE............KYGGVR.P
2d1c.B  ...SVAQTLKLISWKGSEKIVRFAFELARAEGRKKVHCATKSNIMKLAEGTLKRAFEQVAQ..EY..P.............................
3asj.C  ...DVAIADAVISKKASERIGRAALRIAEGRPRKTLHIAHKANVLPLTQGLFLDTVKEVAK.DFP................................
1x0l.A  .YLDVAIADAVISKKASERIGRAALRIAEGRPRKTLHIAHKANVLPLTQGLFLDTVKEVAK.DFP................................
6l59.A  .VDGVVQSIKLITEGASKRIAEFAFEYARNNHRSNVTAVHKANIMRMSDGLFLQKCREVAE.SC...........................KD....
3blx.O  ..PGVVESLKVMTRPKTERIARFAFDFAKKYNRKSVTAVHKANIMKLGDGLFRNIITEIGQKEYP................................
5hn3.A  VVNGRAVDVRLITREGAERIARFAVEQAKARGS.FITFVHKANVLT.GDKFFRRIVREVAGEEG.................................
```

```
1ai2.A  GKEIVIKDVIADAFLQQILLRPAEYDVIACMNLNGDYISDALAAQVGGIGIAPGANIG..D.ECALFEATHGTAPKYAGQDKVNPGSIILSAEMMLR
5m2e.D  GKNVVVKDVIADAMLQQILLRPAEYDVIATLNLNGDYLSDALAAEVGGIGIAPGANLS..D.SVAMFEATHGTAPKYAGQDKVNPGSLILSAEMMLR
6c0e.B  GKQIIINDVIADAFLQQILLRPEDYSVIATLNLNGDYISDALAAQVGGIGIAPGANIS..D.QMAVFEATHGTAPKYAGQNKVNPGSIILSAEMMLR
3dms.A  GNEIVVKDSIADAFLQQILLRPAEYDVIATLNLNGDYISDALAAQVGGIGIAPGANLS..D.SVAMFEATHGTAPKYAGKDYVNPGSEILSAEMMLR
1hqs.B  AGKIIIKDSIADIFLQQILTRPNEFDVVATMNLNGDYISDALAAQVGGIGIAPGANINYET.GHAIFEATHGTAPKYAGLDKVNPSSVILSGVLLLE
2d4v.C  DGKLIIKDVIADNFLQQILLRPEDYSVVATLNLNGDYVSDALAAEVGGIGMAPGANLS..D.THAIFEATHGTAPDIAGQGKANPSSLILSAVMMLE
2iv0.B  EGKIVVKDRIADNMFQQILTRTDEYDVIALPNLNGDYLSDAAAALIGGLGIAPGSNIG..D.GIGVFEPVHGSAPKYAGQNKVNPTAEILTGALMFE
2e0c.A  QGKIILNDRIADNMFQQIIIRPEEYDIILAPNVNGDYISDAAGALIGNIGMLGGANIG..D.EGGMFEAIHGTAPKYAGKNVANPTGIIKAGELMLR
1tyo.A  EGKILVNDRIADNMLQQIITRPWDYQVIVAPNLNGDYISDAASALVGGIGMAAGMNMG..D.GIAVAEPVHGTAPKYAGKDLINPSAEILSASLLIG
2d1c.B  ..DIEAVHIIVDNAAHQLVKRPEQFEVIVTTNMNGDILSDLTSGLIGGLGFAPSANIG..N.EVAIFEAVHGSAPKYAGKNVINPTAVLLSAVMMLR
3asj.C  ..LVNVQDIIVDNCAMQLVMRPERFDVIVTTNLLGDILSDLAAGLVGGLGLAPSGNIG..D.TTAVFEPVHGSAPDIAGKGIANPTAAILSAAMMLD
1x0l.A  ..LVNVQDIIVDNCAMQLVMRPERFDVIVTTNLLGDILSDLAAGLVGGLGLAPSGNIG..D.TTAVFEPVHGSAPDIAGKGIANPTAAILSAAMMLD
6l59.A  ...IKFNEMYLDTVCLNMVQDPSQFDVLVMPNLYGDILSDLCAGLIGGLGVTPSGNIG..ANGVAIFESVHGTAPDIAGKDMANPTALLLSAVMMLR
3blx.O  ..DIDVSSIIVDNASMQAVAKPHQFDVLVTPSMYGTILGNIGAALIGGPGLVAGANFG..R.DYAVFEPGSRHVGKG..QNVANPTAMILSSTLMLN
5hn3.A  ...VEVRDAIIDSFTIKLVRNPWEHGVILSENLFGDILSDLATVHAGSIGIVPSGNYG..D.GIALFEPVHGSAPDIAGKGIANPIGAILSGAMLLD
```

```
                     K378
                     .
1ai2.A  .HMGWTEAADLIVKGMEGAINAK.TVTYDFERLMDGAKLLKCSEFGDAIIENM
5m2e.D  .HMGWTEAADLIIKGTNGAIAAK.TVTYDFERLMDGATLLSCSEFGDAMIAKM
6c0e.B  .HMGWYEAADLIIRGMEGAIEAK.TVTYDFERGMQGATLVSSSGFADAMIKHM
3dms.A  .HLGWTEAADVIISAMEKSIKQK.RVTYDFARLMEGATQVSCSGFGQVLIENM
1hqs.B  .HLGWNEAADLVIKSMEKTIASK.VVTYDFARLMDGATEVKCSEFGEELIKNM
2d4v.C  .HLGWGEAAQAIVAAMNATIAAG.EVTGDLAALRGDVPALSTTEFTAALIRRF
2iv0.B  .YIGWKDASEMIKKAVEMTISSG.IVTYDIHRHMGG.TKVGTREFAEAVVENL
2e0c.A  .WMGWNEAADLIEKAINMAIRDK.KVTQDIAR.FMGVKALGTKEYADELIKIM
1tyo.A  EFMGWREVKSIVEYAIRKAVQSK.KVTQDLARHMPGVQPLRTSEYTETLIAYI
2d1c.B  .YLEEFATADLIENALLYTLEEGRVLTGDVVGYD...RGAKTTEYTEAIIQNL
3asj.C  .YLGEKEAAKRVEKAVDLVLERG.PRTPDLG......GDATTEAFTEAVVEAL
1x0l.A  .YLGEKEAAKRVEKAVDLVLERG.PRTPDLG......GDATTEAFTEAVVEAL
6l59.A  .HMGLFDHAARIEAACFATIKDGKSLTKDLG......GNAKCSDFTEEICRRV
3blx.O  .HLGLNEYATRISKAVHETIAEG.KHTTRDIG.....GSSSTTDFTNEIINKL
5hn3.A  .YLGLD..GSLIRAAVRGYVVNG.ELTPDMG......GRARTEDVVRGIIGEI
```

Supplemental Figure SF3C. Multiple sequence alignment (MSA) of the *E. coli* KatE catalase HPII target protein (PDB ID: 1cf9) and homologs based on 3D structural alignments from FATCAT. Each homolog structure was individually aligned with the KatE target protein in FATCAT and the MSA was generated using a script that compiled individual alignments from the FATCAT xml files into a single alignment based on the target sequence (See Materials and Methods for further details). ESPript was used to visualize the structure-based sequence alignment and lysine residues identified as acetylated on the KatE *E. coli* substrate protein are highlighted in yellow. Note only amino acids that were present in the structural alignment output from FATCAT are shown and do not include residues in disordered regions. Each sequence is identified by the PDB ID and corresponding chain used for the structural alignment. All sequences are organized from highest to lowest sequence similarity based on data within FATCAT xml files.

KatE

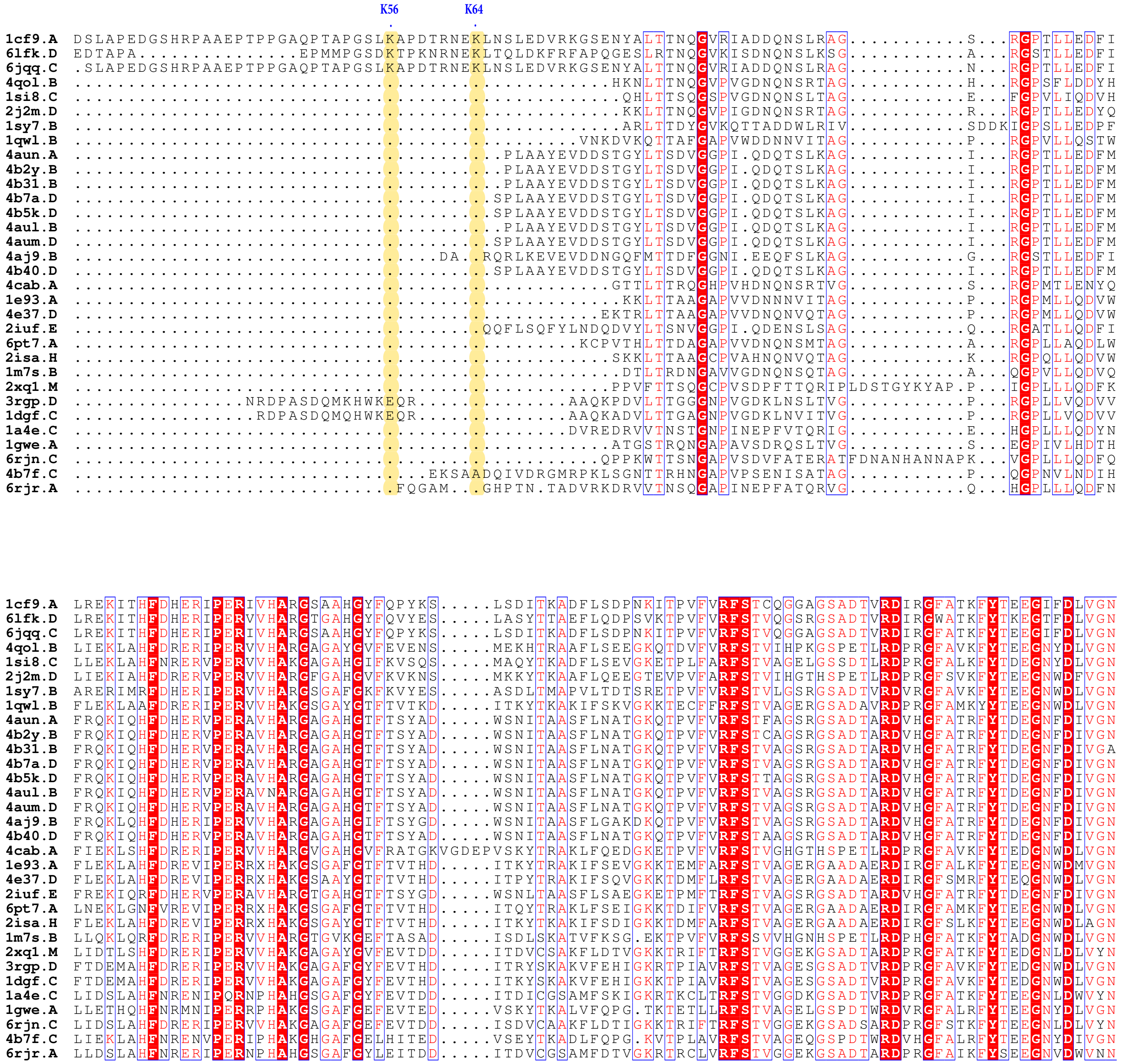
K56
K64
1cf9.A
6lfk.D
6jqq.C
4qol.B
1si8.C
2j2m.D
1sy7.B
1qwl.B
4aun.A
4b2y.B
4b31.B
4b7a.D
4b5k.D
4aul.B
4aum.D
4aj9.B
4b40.D
4cab.A
1e93.A
4e37.D
2iuf.E
6pt7.A
2isa.H
1m7s.B
2xq1.M
3rgp.D
1dgf.C
1a4e.C
1gwe.A
6rjn.C
4b7f.C
6rjr.A

```
1cf9.A   NTPIFFIQDAHKFPDFVHAVKPEPHWAIPQGQSAHDTFWDYVSL..QPETLHNVMWAMSDRGIPRSYRTMEGFGIHTFRLINAEGKATFVRFHWKPL
6lfk.D   NTPVFFIQDAIKFPDFVHAVKPEPHNEIPQGQSAHDTFWDYISL..QPETLHNVMWVMSDRGIPRSYRMMEGFGIHTYKMINAEGQCHFIRFHWKPV
6jqq.C   NTPIFFIQDAHKFPDFVHAVKPEPHWAIPQGQSAHDTFWDYVSL..QPETLHNVMWAMSDRGIPRSYRTMEGFGIHTFRLINAEGKATFVRFHWKPL
4qol.B   NLPIFFIRDALKFPDMVHSLKPDPVTNIQ....DPDRYWDFMTL..TPESTHMLTWLFSDEGIPANYAEMRGSGVHTFRWVNKYGETKYVKYHWRPS
1si8.C   NTPIFFIRDAIKFPDFIHSQKRNPRTHLK....SPEAVWDFWSH..SPESLHQVTILMSDRGIPLSFRHMHGFGSHTFKWVNAAGEVFFVKYHFKTN
2j2m.D   NLPVFFIRDAMKFPDMVHSLKPDPRTNIQ....DPDRYWDFMTL..RPESTNMLMHIFTDEGIPASYRKMRGSSVHSFKWVNAHGNTVYIKLRWVPK
1sy7.B   NIPVFFIQDAIKFPDVIHAGKPEPHNEVPQAQSAHNNFWDFQFN..HTEATHMFTWAMSDRAIPRSLRMMQGFGVNTYTLINAQGKRHFVKFHWTPE
1qwl.B   NTPVFFIRDAIKFPDFIHTQKRDPQTNLP....NHDMVWDFWSN..VPESLYQVTWVMSDRGIPKSFRHMDGFGSHTFSLINAKGERFWVKFHFHTM
4aun.A   NIPVFFIQDAIQFPDLIHSVKPRPDNEIPQAATAHDSAWDFFSQ..QPSTMHTLFWAMSGHGIPRSYRHMDGFGVHTFRFVKDDGSSKLIKWHFKSR
4b2y.B   NIPVFFIQDAIQFPDLIHSVKPRPDNEIPQAATAHDSAWDFFSQ..QPSTMHTLFWAMSGHGIPRSYRHMDGFGVHTFRFVKDDGSSKLIKWHFKSR
4b31.B   NIPVFFIQDAIQFPDLIHSVKPRPDNEIPQAATAHDSAWDFFSQ..QPSTMHTLFWAMSGHGIPRSYRHMDGFGVHTFRFVKDDGSSKLIKWHFKSR
4b7a.D   NIPVFFIQDAIQFPDLIHSVKPRPDNEIPQAATAHDSAWDFFSQ..QPSTMHTLFWAMSGHGIPRSYRHMDGFGVHTFRFVKDDGSSKLIKWHFKSR
4b5k.D   NIPVFFIQDAIQFPDLIHSVKPRPDNEIPQAATAHDSAWDFFSQ..QPSTMHTLFWAMSGHGIPRSYRHMDGFGVHTFRFVKDDGSSKLIKWHFKSR
4aul.B   NIPVFFIQDAIQFPDLIHSVKPRPDNEIPQAATAHDSAWDFFSQ..QPSTMHTLFWAMSGHGIPRSYRHMDGFGVHTFRFVKDDGSSKLIKWHFKSR
4aum.D   NIPVFFIQDAIQFPDLIHSVKPRPDNEIPQAATAHDSAWDFFSQ..QPSTMHTLFWAMSGHGIPRSYRHMDGFGVHTFRFVKDDGSSKLIKWHFKSR
4aj9.B   NIPVFFIQDAIRFPDLIHSVKPSPDNEVPQAATAHDSAWDFFSS..QPSALHTLFWAMSGNGIPRSYRHMDGFGIHTFRLVTEDGKSKLVKWHWKTK
4b40.D   NIPVFFIQDAIQFPDLIHSVKPRPDNEIPQAATAHDSAWDFFSQ..QPSTMHTLFWAMSGHGIPRSYRHMDGFGVHTFRFVKDDGSSKLIKWHFKSR
4cab.A   NLKIFFIRDALKFPDLIHSQKPSPTTNIQ....SQERIFDFFAG..SPEATHMITLLYSPWGIPASYRFMQGSGVNTYKWVNDQGEGVLVKYHWEPV
1e93.A   NTPVFYLRDPLKFPDLNHIVKRDPRTNMR....NMAYKWDFFSH..LPESLHQLTIDMSDRGLPLSYRFVHGFGSHTYSFINKDNERFWVKFHFRCQ
4e37.D   NTPVFYLRDPLKFPDLNHVVKRDPRTNLR....NATFKWDFFSH..LPESLHQLTIDFSDRGLPKSYRHIHGFGSHTFSFINANNERFWVKFHFKTQ
2iuf.E   NIPVFFIQDAILFPDLIHAVKPRGDNQIPQAATAHDSAWDFFSQ..QPSVLHTLLWAXAGHGIPRSFRHVNGFGVHTFRLVTDDGKTKLVKFHWKGL
6pt7.A   NTPVFFLRDARKFPDLNKAVKRDPKTNKR....SATNNWDFWTL..LPEALHQVTIVMSDRGIPDGYRHMHGFGSHTFSFINANNERFWVKFHMRTQ
2isa.H   NTPVFFLRDPLKFPDLNHAVKRDPRTNMR....SAKNNWDFWTS..LPEALHQVTIVMSDRGIPATYRHMHGFGSHTFSFINSDNERYWVKFHFVSQ
1m7s.B   NFPTFFIRDAIKFPDMVHAFKPDPRTNLD....NDSRRFDFFSH..VPEATRTLTLLYSNEGTPAGYRFMDGNGVHAYKLVNAKGEVHYVKFHWKSL
2xq1.M   NTPIFFIRDPIKFPHFIHTQKRNPATNLK....DPNMFWDYLTA..NDESLHQVMYLFSNRGTPASYRTMNGYSGHTYKWYNSKGEWVYVQVHFIAN
3rgp.D   NTPIFFIRDALLFPSFIHSQKRNPQTHLK....DPDMVWDFWSL..RPESLHQVSFLFSDRGIPDGHRHMNGYGSHTFKLVNANGEAVYCKFHYKTD
1dgf.C   NTPIFFIRDPILFPSFIHSQKRNPQTHLK....DPDMVWDFWSL..RPESLHQVSFLFSDRGIPDGHRHMNGYGSHTFKLVNANGEAVYCKFHYKTD
1a4e.C   NTPVFFIRDPSKFPHFIHTQKRNPQTNLR....DADMFWDFLTTPENQVAIHQVMILFSDRGTPANYRSMHGYSGHTYKWSNKNGDWHYVQVHIKTD
1gwe.A   NTPIFFLRDPMKFTHFIRSQKRLPDSGLR....DATMQWDFWTN..NPESAHQVTYLMGPRGLPRTWREMNGYGSHTYLWVNAQGEKHWVKYHFISQ
6rjn.C   NTPIFFIRDPSKFPHFIHTQKRNPATNLK....DANMFWDYLVN..NQESIHQVMYLFSDRGTPASLRKMNGYSGHTYKWYNKKGEWVYVQVHFKSD
4b7f.C   NTPTFFLRDGMKFPDFIHSQKRLNKNGLR....DADMQWDFWTR..APESAHQVTYLMGDRGTPKTSRHQDGFGSHTFQWINAEGKPVWVKYHFKTR
6rjr.A   NTPVFFIRDPSKFPHFIHTQKRNPETNMK....DADMFWDFLTTEENQVAIHQVMILFSDRGTPASYRNMNSYSGHTYKWSNKQGEWRYVQVHLKTD
```

```
1cf9.A   AGKASLVWDEAQKLTGRDPDFHRRELWEAIEAGDFPEYELGFQLIPEEDEFKFDFDLLDPTKLIPEELVPVQRVGKMVLNRNPDNFFAENEQAAFHP
6lfk.D   YGVSSLIWDEAQLLTGCDPDFHRRELWESIEAGDYPEYELGLQIIPEEDEHKFDFDILDPTKLIPESLVPVHLVGKMVLNRNPDNYFSETEQVAFCP
6jqq.C   AGKASLVWDEAQKLTGRDPDFHRRELWEAIEAGDFPEYELGFQLIPEEDEFKFDFDLLDPTKLIPEELVPVQRVGKMVLNRNPDNFFAENEQAAFCP
4qol.B   EGIRNLSMEEAAEIQANDFQHATRDLYDRIEKGNYPAWDLYVQLMPLSDYDELDYDPCDPTKTWSEEDYPLQKVGRMTLNRNPENFFAETEQAAFTP
1si8.C   QGIKNLESQLAEEIAGKNPDFHIEDLHNAIENQEFPSWTLSVQIIPYADALTMKETLFDVTKTVSQKEYPLIEVGTMTLNRNPENYFAEVEQVTFSP
2j2m.D   EGVHNLSADEATEVQGKDFNHASNDTFQAIENGDFPEWDLFVQVLDPADVENFDFDPLDATKDWFEDVIPFQHVGTMTLNKNVDNYFAETESVGFNP
1sy7.B   LGVHSLVWDEALKLAGQDPDFHRKDLWEAIENGAYPKWKFGIQAIAEEDEHKFDFDILDATKIWPEDLVPVRYIGEMELNRNPDEFFPQTEQIAFCT
1qwl.B   QGVKHLTNEEAAEIRKHDPDSNQRDLFDAIARGDYPKWKLSIQVMPEEDAKKYRFHPFDVTKIWYTQDYPLMEVGIVELNKNPENYFAEVEQAAFTP
4aun.A   QGKASLVWEEAQVLSGKNADFHRQDLWDAIESGNGPEWDVCVQIVDESQAQAFGFDLLDPTKIIPEEYAPLTKLGLLKLDRNPTNYFAETEQVMFQP
4b2y.B   QGKASLVWEEAQVLSGKNADFHRQDLWDAIESGNGPEWDVCVQIVDESQAQAFGFDLLDPTKIIPEEYAPLTKLGLLKLDRNPTNYFAETEQVMFQP
4b31.B   QGKASLVWEEAQVLSGKNADFHRQDLWDAIESGNGPEWDVCVQIVDESQAQAFGFDLLDPTKIIPEEYAPLTKLGLLKLDRNPTNYFAETEQVMFQP
4b7a.D   QGKASLVWEEAQVLSGKNADFHRQDLWDAIESGNGPEWDVCVQIVDESQAQAFGFDLLDPTKIIPEEYAPLTKLGLLKLDRNPTNYFAETEQVMFQP
4b5k.D   QGKASLVWEEAQVLSGKNADFHRQDLWDAIESGNGPEWDVCVQIVDESQAQAFGFDLLDPTKIIPEEYAPLTKLGLLKLDRNPTNYFAETEQVMFQP
4aul.B   QGKASLVWEEAQVLSGKNADFHRQDLWDAIESGNGPEWDVCVQIVDESQAQAFGFDLLDPTKIIPEEYAPLTKLGLLKLDRNPTNYFAETEQVMFQP
4aum.D   QGKASLVWEEAQVLSGKNADFHRQDLWDAIESGNGPEWDVCVQIVDESQAQAFGFDLLDPTKIIPEEYAPLTKLGLLKLDRNPTNYFAETEQVMFQP
4aj9.B   QGKAALVWEEAQVLAGKNADFHRQDLWDAIESGNAPSWELAVQLIDEDKAQAYGFDLLDPTKFLPEEFAPLQVLGEMTLNRNPMNYFAETEQISFQP
4b40.D   QGKASLVWEEAQVLSGKNADFHRQDLWDAIESGNGPEWDVCVQIVDESQAQAFGFDLLDPTKIIPEEYAPLTKLGLLKLDRNPTNYFAETEQVMFQP
4cab.A   QGVRNLTQMQADEVQATNFNHATQDLHDAIERGDFPQWDLFVQIMEDGEHPELDFDPLDDTKIWPREQFPWRHVGQMTLNRNPENVFAETEQAAFGT
1e93.A   QGIKNLMDDEAEALVGKDRESSQRDLFEAIKRGDYPRWKLQIQIMPEKEASTVPYNPFDLTKVWPHADYPLMDVGYFELNRNPDNYFSDVEQAAFSP
4e37.D   QGIENLTNAEAAEVIAQDRESSQRDLYESIEKGDFPRWKMYVQIMPEKEAATYRYNPFDLTKVWPHGDYPLIEVGFFELNRNPDNYFAEVEQAAFTP
2iuf.E   QGKASFVWEEAQQTAGKNADFXRQDLFQSIQAGRFPEWELGVQIMQEQDQLKFGFDLLDPTKIVPEELVPVTILGKMQLNRNPXNYFAETEQVMFQP
6pt7.A   QGIKNLTDAEAEAIIAKDRESSQTDLFDAIERGDFPKWKMYVQIMPELDAEKVPYHPFDLTKVWPKGDYPLIEVGEFELNRNPENYFQDVEQAAFAP
2isa.H   QGIKNLSDAEAGELVGNDRESHQRDLLDSIDNQDFPKWTLKVQIMPEADAATVPYNPFDLTKVWPHKDYPLIEVGEFELNRNPQNYFAEVEQAAFNP
1m7s.B   QGIKNLDPKEVAQVQSKDYSHLTNDLVGAIKKGDFPKWDLYVQVLKPEELAKFDFDPLDATKIWP..DVPEKKIGQMVLNKNVDNFFQETEQVAMAP
2xq1.M   QGVHNLLDEEAGRLAGEDPDHSTRDLWEAIEKGDYPSWECYIQTMTLEQSKKLPFSVFDLTKVWPHKDFPLRHFGRFTLNENPKNYYAETEQIAFSP
3rgp.D   QGIKNLSVEDAARLAHEDPDYGLRDLFNAIATGNYPSWTLYIQVMTFSEAEIFPFNPFDLTKVWPHGDYPLIPVGKLVLNRNPVNYFAEVEQLAFDP
1dgf.C   QGIKNLSVEDAARLSQEDPDYGIRDLFNAIATGKYPSWTFYIQVMTFNQAETFPFNPFDLTKVWPHKDYPLIPVGKLVLNRNPVNYFAEVEQIAFDP
1a4e.C   QGIKNLTIEEATKIAGSNPDYCQQDLFEAIQNGNYPSWTVYIQTMTERDAKKLPFSVFDLTKVWPQGQFPLRRVGKIVLNENPLNFFAQVEQAAFAP
1gwe.A   QGVHNLSNDEATKIAGENADFHRQDLFESIAKGDHPKWDLYIQAIPYEEGKTYRFNPFDLTKTISQKDYPRIKVGTLTLNRNPENHFAQIESAAFSP
6rjn.C   LGVVNFNNEEAGKLAGEDPDYHTGDLFNAIERGEYPSWTCYIQTMTQEQAAKQPFSVFDLTKVWPHKDFPLRRFGKFTLNENPKNYFAEVEQAAFSP
4b7f.C   QGWDCFTDAEAAKVAGENADYQREDLYNAIENGDFPIWDVKVQIMPFEDAENYRWNPFDLTKTWSQKDYPLIPVGYFILNRNPRNFFAQIEQIALDP
6rjr.A   QGIKNLNNEEATKLAGENPDYCQKDLFENIAKGNYPSWTLYIQTMTEEEAEKLPFSVFDLTKVWPHKQFPLRRVGKMVLNENPENYFAQVEQAAFSP
```

```
1cf9.A  GHIVPGLDFTNDPLLQGRLFSYTDTQISRLGGPNFHEIPINRPT.CPYHNF.QRDGMHRMGI..D.T.....NPANYEPNSINDNWPRETPPGPKRG
6lfk.D  GNIVPGIDFSDDPLLQGRLFSYIDTQISRLGGVNFHEIPINKPI.CPFHNH.QRDGMHRMSI..S.G.....T.ANYEPNSINNNWPREAPPT..EG
6jqq.C  GHIVPGLDFTNDPLLQGRLFSYIDTQISRLGGPNFHEIPINRPT.CPYHNF.QRDGMHRMGI..D.T.....NPANYEPNSINDNWPRETPPGPKRG
4qol.B  SALVPGIEASEDKLLQGRLFSYPDTQRHRL.GANYMRIPVNCPY.APVHNN.QQDGFMTTTR..P.S.....GHINYEPNR.YDDQPKENPH.....
1si8.C  GNFVPGIEASPDKLLQGRLFAYGDAHRHRV.GANSHQLPINQAK.APVNNY.QKDGNMRFNN..G.N.....SEINYEPNS.YTETPKEDPT.....
2j2m.D  GVLVPGMLPSEDKLLQGRLFSYSDTQRHRIGP.NYQQLPINCPF.AQVNNY.QRDGAMPFKQ..Q.T.....SSVNYEPNR.YQDEPKQTPE.....
1sy7.B  SHVVNGIGFSDDPLLQGRNFSYFDTQISRLG.VNFQELPINRPV.CPVMNF.NRDGAMRHTI..S.R.....GTVNYYPNR..FDACPPASLK..EG
1qwl.B  ANVVPGIGYSPDRMLQGRLFSYGDTHRYRL.GVNYPQIPVNKPR.CPFHSS.SRDGYMQNGY..Y.G.....SLQNYTPSS..LPGYKEDKS.....
4aun.A  GHIVRGIDFTEDPLLQGRLFSYLDTQLNRNGGPNFEQLPINMPR.VPIHNN.NRDGAGQMFI..H.R.....NKYPYTPNTLNSGYPRQANQN.AGR
4b2y.B  GHIVRGIDFTEDPLLQGRLFSYLDTQLNRNGGPNFEQLPINMPR.VPIHNN.NRDGAGQMFI..H.R.....NKYPYTPNTLNSGYPRQANQNA.GR
4b31.B  GHIVRGIDFTEDPLLQGRLFSYLDTQLNRNGGPNFEQLPINMPR.VPIHNN.NRDGAGQMFI..H.R.....NKYPYTPNTLNSGYPRQANQNA.GR
4b7a.D  GHIVRGIDFTEDPLLQGRLFSYLDTQLNRNGGPNFEQLPINMPR.VPIHNN.NRDGAGQMFI..H.R.....NKYPYTPNTLNSGYPRQANQN.AGR
4b5k.D  GHIVRGIDFTEDPLLQGRLFSYLDTQLNRNGGPNFEQLPINMPR.VPIHNN.NRDGAGQMFI..H.R.....NKYPYTPNTLNSGYPRQANQN.AGR
4aul.B  GHIVRGIDFTEDPLLQGRLFSYLDTQLNRNGGPNFEQLPINMPR.VPIHNN.NRDGAGQMFI..H.R.....NKYPYTPNTLNSGYPRQANQN.AGR
4aum.D  GHIVRGIDFTEDPLLQGRLFSYLDTQLNRNGGPNFEQLPINMPR.VPIHNN.NRDGAGQMFI..H.R.....NKYPYTPNTLNSGYPRQANQN.AGR
4aj9.B  GHIVRGVDFTEDPLLQGRLYSYLDTQLNRHRGPNFEQLPINRPV.SGVHNN.HRDGQGQAWI..H.K.....NIHHYSPSYLNKGYPAQANQT.VGR
4b40.D  GHIVRGIDFTEDPLLQGRLFSYLDTQLNRNGGPNFEQLPINMPR.VPIHNN.NRDGAGQMFI..H.R.....NKYPYTPNTLNSGYPRQANQN.AGR
4cab.A  GVLVDGLDFSDDKMLQGRTFSYSDTQRYRVGP.NYLQLPINAPK.KHVATN.QRDGQMAYRV..D.TFEGQDQRVNYEPSLLS..GPKEAPRRA...
1e93.A  ANIVPGISFSPDKMLQGRLFSYGDAHRYRLG.VNHHQIPVNAPK.CPFHNY.HRDGAMRVDG..NSG.....NGITYEPNS..GGVFQEQPD.....
4e37.D  ANVVPGIGFSPDKMLQGRLFSYGDAHRYRL.GVNHHQIPVNAAR.CPHQVY.HRDGGMRVDGNNA.H.....QRVTYEPNS..FNQWQEQPD.....
2iuf.E  GHIVRGVDFTEDPLLQGRLFSYLDTQLNRHGGPNFEQLPINRPR.APIHNN.NRDGAGQMFI..P.L.....DPNAYSPNTENKGSPKQANET.VGK
6pt7.A  SNLVPGISFSPDRMLQARLFNYADAARYRV.GVNHYQIPVNAPR.CPVHSN.RRDGQGRTDGN.Y.G.....ALPHYEPNS..FSQWQEQPQ.....
2isa.H  ANVVPGISFSPDKMLQGRLFAYGDAQRYRL.GVNHQHIPVNAPR.CPVHSY.HRDGAMRVDGN.F.G.....STLGYEPND..QGQWAEQPD.....
1m7s.B  ANLVPGIEPSEDRLLQGRVFSYADTQMYRL.GANGLSLPVNQPK.VAVNNG.NQDGALNTGH..T.T.....SGVNYEPSRLE..PRPADDK.....
2xq1.M  SHTVPGMEPSNDPVLQSRLFSYPDTHRHRLGP.NYHQIPVNCPLKSGSFNPINRDGPMCVDGN.L.G.....GTPNYANAYNCP..IQYAVS.....
3rgp.D  SNMPPGIEPSPDKMLQGRLFAYPDTHRHRLG.PNYLQIPVNCPYRARVANY.QRDGPMCMMDN.Q.G.....GAPNYYPNS..FSAPEHQPS.....
1dgf.C  SNMPPGIEASPDKMLQGRLFAYPDTHRHRL.GPNYLHIPVNCPYRARVANY.QRDGPMCMQDN.Q.G.....GAPNYYPNS..FGAPEQQPS.....
1a4e.C  STTVPYQEASADPVLQARLFSYADAHRYRLG.PNFHQIPVNCPYASKFFNPAIRDGPMNVNG..N.F.....GSEPTYLANDK..SYTYIQQD...R
1gwe.A  SNTVPGIGLSPDRMLLGRAFAYHDAQLYRVG.AHVNQLPVNRPK.NAVHNY.AFEGQMWYDH..T.G.....DRSTYVPNSNG..DSWSDET.....
6rjn.C  SHTIPSMQPSADPVLQSRLFSYPDTHRHRL.GVNYQQIPVNCPV.APVFTPQMRDGSMTVNG..N.L.....GSTPNYKSSFC..PFSTEAQIQ..T
4b7f.C  GNIVPGVGLSPDRMLQARIFAYADQQRYRIG.ANYRDLPVNRPI.NEVNTY.SREGSMQYIF..DAE.....GEPSYSPNR..YDKGAGYLDNGTDS
6rjr.A  SHTVPYQEASADPVLQARLFSYPDAHRYRLG.PNYSQIPVNCPYASKVFNPAIRDGPMNVNG..N.L.....GKEPNYLSTSK..KYQFIQQS...K
```

```
1cf9.A  .........GFESYQ.ERV..E.....GNKVRERS.PSF..G....EYYSHPRLFWL.SQ..TPFEQRHIVDGFSFELSKVVRPYIRERVVDQLAHI
6lfk.D  .........GFTTYP.QPV..N.....GYKSRKRS.STF..I....DFYSQPRLFWL.SQ..TKVEQNHIVGGFSFELGKVVRPWIRERVVNQLTYI
6jqq.C  .........GFESYQ.ERV..E.....GNKVRERS.PSF..G....EYYSHPRLFWL.SQ..TPFEQRHIVDGFSFELSKVVRPYIRERVVDQLAHI
4qol.B  ..........YKESE.PVLH.G.....DRMVRQKI.EKP.......NDFKQAGEKYR.SY..SEEEKQALIKNLTADLKGV.NEKTKLLAICNFYRA
1si8.C  ..........AKISS.FEV..E.....GNVGNYSY...N..Q....DHFTQANALYN.LL..PSEEKENLINNIAASLGQVKNQEIIARQIDLFTRV
2j2m.D  .........YTEDTQ.PLH..D.....DIHGRLEI.EK...T....NNFGQAGEVYR.RM..TEEEQMALLNNLVNDLQQVRHENTVLLAICNFYRA
1sy7.B  .........GYLEYA.QKV..A.....GIKARARS.AKF..K....EHFSQAQLFYN.SM..SPIEKQHMINAFGFELDHCEDPVVYGRMVQRLADI
1qwl.B  ..........ARDPK.FNL..AHIEKEFEVWNWDYRADD..S....DYYTQPGDYYR.SL..PADEKERLHDTIGESLAHVTHKEIVDKQLEHFKKA
4aun.A  .........GFFTAPGRTA..S.....GALVREVS.PTF..N....DHWSQPRLFFN.SL..TPVEQQFLVNAMRFEISLVKSEEVKKNVLTQLNRV
4b2y.B  .........GFFTAPGRTA..S.....GALVREVS.PTF..N....DHWSQPRLFFN.SL..TPVEQQFLVNAMRFEISLVKSEEVKKNVLTQLNRV
4b31.B  .........GFFTAPGRTA..S.....GALVREVS.PTF..N....DHWSQPRLFFN.SL..TPVEQQFLVNAMRFEISLVKSEEVKKNVLTQLNRV
4b7a.D  .........GFFTAPGRTA..S.....GALVREVS.PTF..N....DHWSQPRLFFN.SL..TPVEQQFLVNAMRFEISLVKSEEVKKNVLTQLNRV
4b5k.D  .........GFFTAPGRTA..S.....GALVREVS.PTF..N....DHWSQPRLFFN.SL..TPVEQQFLVNAMRFEISLVKSEEVKKNVLTQLNRV
4aul.B  .........GFFTAPGRTA..S.....GALVREVS.PTF..N....DHWSQPRLFFN.SL..TPVEQQFLVNAMRFEISLVKSEEVKKNVLTQLNRV
4aum.D  .........GFFTAPGRTA..S.....GALVREVS.PTF..N....DHWSQPRLFFN.SL..TPVEQQFLVNAMRFEISLVKSEEVKKNVLTQLNRV
4aj9.B  .........GFFTTPGRTA..S.....GVLNRELS.ATF..D....DHYTQPRLFFN.SL..TPVEQQFVINAIRFEASHVTNEQVKKNVLEQLNKI
4b40.D  .........GFFTAPGRTA..S.....GALVREVS.PTF..N....DHWSQPRLFFN.SL..TPVEQQFLVNAMRFEISLVKSEEVKKNVLTQLNRV
4cab.A  ...........PEHT.PRV..E.....GNLVRAAI.ER...P....NPFGQAGMQYR.NF..ADWERDELVSNLSGALA.GVDKRIQDKMLEYFTAA
1e93.A  ..........FKEPP.LSI..E.....GAADHWNH.RED..E....DYFSQPRALYE.LL..SDDEHQRMFARIAGELS.QASKETQQRQIDLFTKV
4e37.D  ..........FSEPP.LSL..E.....GAADHWNH.RVD..D....DYYSQPAALFH.LF..TDEQKQRLFANIAEDIRDV.PEQIQRRQIGLFLKV
2iuf.E  .........GFFTAPERTA..S.....GKLQRTLS.TTF..E....NNWSQPRLFWN.SL..VNAQKEFIVDAXRFETSNVSSSVVRDDVIIQLNRI
6pt7.A  ..........YKEPP.LKI..S.....GAADFWDYREDD..N....DYFSQPRALFN.LM..NDQQKQALFDNTAAAMGDA.LDFIKYRHIRNCYAC
2isa.H  ..........FSEPP.LNL..D.....GAAAHWDH.RED..E....DYFSQPGDLFG.LM..TAEKQAILFDNTARNLNGV.PKEIQLRHVTHCYKA
1m7s.B  ..........ARYSE.LPL..S.....GTTQQAKI.TR...E....QNFKQAGDLYR.SY..SAKEKTDLVQKFGESLA.DTLTESKNIMLSYLYKE
2xq1.M  .............PD.EKY..T.....GEVVPYHW.EHT..D....YDYFQPKMFWK.VLGRTPGEQESLVKNVANHVSAA.DEFIQDRVYEYFSKA
3rgp.D  ..........ALEHR.THF..S.....GDVQRFNS.A.N..D....DNVTQVRTFYLKVL..NEEQRKRLCENIAGHLKDA.QLFIQKKAVKNFSDV
1dgf.C  ..........ALEHS.IQY..S.....GEVRRFNT.A.N..D....DNVTQVRAFYVNVL..NEEQRKRLCENIAGHLKDA.QIFIQKKAVKNFTEV
1a4e.C  .........PIQQHQ.EVW..N.....GPAIPYHW.ATS..PGD..VDFVQARNLYR.VLGKQPGQQKNLAYNIGIHVEGA.CPQIQQRVYDMFARV
1gwe.A  .........GPVDDG.WEA..D.....GTLTREAQ.ALRADD....DDFGQAGTLVREVF..SDQERDDFVETVAGALKGV.RQDVQARAFEYWKNV
6rjn.C  .........NSHTPE.EVL..A.....AHTEKFHW.GGI..LDSKSYDFEQPRALWK.VFGKTPGQQRNFCHNVAVHVAAA.NHEIQDRVFEYFSKV
4b7f.C  SSNHTSYGQADDIYV.NPDPHG.....TDLVRAAY.VKHQDD....DDFIQPGILYREVL..DEGEKERLADNISNAMQGI.SEATEPRVYDYWNNV
6rjr.A  .........PIQQHQ.EVW..S.....GPAPVHWA.TSP..GD...IDFVQARDLYNKVLSKQPGQQKALAHNVAVHVASA.CPEIQDRVFAMFARV
```

```
1cf9.A DLTLAQAVAKNLGIELTDDQLNITPPPDVNGLKKDPSLSLYAIPD..G.DVKGRVVAILLND..EVRSADLLAILKALKAKGVHAKLLYSRMGEVTA
6lfk.D DHQLAQSVADNLGIKLSQEQLKHPLPGPINGLSKDRSLSMYDGHH..Q.ILKSRQVAILAAD..GVCGDAIDNIMKTLKKYGVHGKIFAPHVGRITS
6jqq.C DLTLAQAVAKNLGIELTDDQLNITPPPDVNSLKKDPSLSLYAIPD..G.DVKGRVVAILLND..EVRSADLLAILKALKAKGVHAKLLYSRMGEVTA
4qol.B DEDYGQRLADSLGVDIRSY..............................................................................
1si8.C NPEYGARVAQAIKQ...................................................................................
2j2m.D DASLGEKLSEALNVDIKPF..............................................................................
1sy7.B DLGLAQTIAEMVGGEAP..TTTNHPNHG....RKTINLSQTEFPPATP.TIKSRRVAIIIAD..GYDNVAYDAAYAAISANQAIPLVIGPRRSKVTA
1qwl.B DPKYAEGVKKALEK...................................................................................
4aun.A SHDVAVRVAAAIGLGA...PDADDTYYH...NNKTAGVSIVGSGP..LPTIKTLRVGILATTSESSALDQAAQLRTRLEKDGLVVTVVAETLR....
4b2y.B SHDVAVRVAAAIGLGA...PDADDTYYH...NNKTAGVSIVGSGP..LPTIKTLRVGILATTSESSALDQAAQLRTRLEKDGLVVTVVAETLR....
4b31.B SHDVAVRVAAAIGLGA...PDADDTYYH...NNKTAGVSIVGSGP..LPTIKTLRVGILATTSESSALDQAAQLRTRLEKDGLVVTVVAETLR....
4b7a.D SHDVAVRVAAAIGLGA...PDADDTYYH...NNKTAGVSIVGSGP..LPTIKTLRVGILATTSESSALDQAAQLRTRLEKDGLVVTVVAETLR....
4b5k.D SHDVAVRVAAAIGLGA...PDADDTYYH...NNKTAGVSIVGSGP..LPTIKTLRVGILATTSESSALDQAAQLRTRLEKDGLVVTVVAETLR....
4aul.B SHDVAVRVAAAIGLGA...PDADDTYYH...NNKTAGVSIVGSGP..LPTIKTLRVGILATTSESSALDQAAQLRTRLEKDGLVVTVVAETLR....
4aum.D SHDVAVRVAAAIGLGA...PDADDTYYH...NNKTAGVSIVGSGP..LPTIKTLRVGILATTSESSALDQAAQLRTRLEKDGLVVTVVAETLR....
4aj9.B SNDVAKRVAVALGLEA...PQPDPTYYH...NNVTRGVSIFNESL..P.TIATLRVGVLSTTK.GGSLDKAKALKEQLEKDGLKVTVIAEYLAS...
4b40.D SHDVAVRVAAAIGLGA...PDADDTYYH...NNKTAGVSIVGSGP..LPTIKTLRVGILATTSESSALDQAAQLRTRLEKDGLVVTVVAETLR....
4cab.A DADYGQRVREGIQA...................................................................................
1e93.A HPEYGAGVEKAIKV...................................................................................
4e37.D DPAYGKGVADALGLKLD................................................................................
2iuf.E SDNLATRVASAIGVEA...PKPNSSFYH...DNTTAHIGAFGEKL..A.KLDGLKVGLLASVNKPASIAQGAKLQVALSSVGVDVVVVAERXAN...
6pt7.A DPAYGEGVAKALGMTVADAQAAR..........................................................................
2isa.H DPAYGEGIGKLLGFDISEYNS............................................................................
1m7s.B DPNYGTRVAEVAKGDLSKVKSLAASLKD.....................................................................
2xq1.M EPIIGDLIRKKVQE...................................................................................
3rgp.D HPEYGSRIQALLDKYNE................................................................................
1dgf.C HPDYGSHIQALLDKYN.................................................................................
1a4e.C DKGLSEAIKKVAE....................................................................................
1gwe.A DATIGQRIEDEVKRH..................................................................................
6rjn.C YPEIGDQIRKEVLQLSPR...............................................................................
4b7f.C DENLGARVKELYLQ...................................................................................
6rjr.A DRGLSENIKKEALSLSPR...............................................................................
```

```
1cf9.A DDGTVLPIAATFAGAPSLTVDAVIVPCG..N..IADIAD..................N.......GDANYYLMEAYKHLKPIALA.GDARKF.KA.T
6lfk.D LQGNEIEVNGTIEGNPSVMVDAVIIPDG..EDSIDSLMK..................N.......GNAKHYVIQAFKHLKAIGLQ.GKAFKL.YDAL
6jqq.C DDGTVLPIAATFAGAPSLTVDAVIVPCG..N..IADIAD..................N.......GDANYYLMEAYKHLKPIALA.GDARKF.KA.T
4qol.B .................................................................................................
1si8.C .................................................................................................
2j2m.D .................................................................................................
1sy7.B ANGSTVQPHHHLEGFRSTMVDAIFIPGGAKA..AETLSK..................N.......GRALHWIREAFGHLKAIGAT.GEAVDLVAK.A
1qwl.B .................................................................................................
4aun.A .....EGVDQTYSTADATGFDGVVVVDG..A..AALFASSPLFP.............T.......GRPLQIFVDAYRWGKPVGVC.G..SEV.LD.A
4b2y.B .....EGVDQTYSTADATGFDGVVVVDG..A..AALFASSPLFP.............T.......GRPLQIFVDAYRWGKPVGVCGGKSSEV.LD.A
4b31.B .....EGVDQTYSTADATGFDGVVVVDG..A..AA.LFSSPLFP.............T.......GRPLQIFVDAYRWGKPVGVCGGKSSEV.LD.A
4b7a.D .....EGVDQTYSTADATGFDGVVVVDG..A..AALFASSPLFP.............T.......GRPLQIFVDAYRWGKPVGVC.G...EV.LD.A
4b5k.D .....EGVDQTYSTADATGFDGVVVVDG..A..AALFASSPLFP.............T.......GRPLQIFVDAYRWGKPVGVC.G...EV.LD.A
4aul.B .....EGVDQTYSTADATGFDGVVVVDG..A..AALFASSPLFP.............T.......GRPLQIFVDAYRWGKPVGVCGGKSSEV.LD.A
4aum.D .....EGVDQTYSTADATGFDGVVVVDG..A..AALFASSPLFP.............T.......GRPLQIFVDAYRWGKPVGVC.G..SEV.LD.A
4aj9.B ......GVDQTYSAADATAFDAVVVAEG..A..ERVFSGKG................AMSPLFPAGRPSQILTDGYRWGKPVAAV.GSAKKA.LQ.S
4b40.D .....EGVDQTYSTADATGFDGVVVVDG..A..AALFASSPLFP.............T.......GRPLQIFVDAYRWGKPVGVC.G..SEV.LD.A
4cab.A .................................................................................................
1e93.A .................................................................................................
4e37.D .................................................................................................
2iuf.E ......NVDETYSASDAVQFDAVVVADG..A..EGLFGADSFTVEPSAGSGASTLYPA.......GRPLNILLDAFRFGKTVGAL.GSGSDA.LE.S
6pt7.A .................................................................................................
2isa.H .................................................................................................
1m7s.B .................................................................................................
2xq1.M .................................................................................................
3rgp.D .................................................................................................
1dgf.C .................................................................................................
1a4e.C .................................................................................................
1gwe.A .................................................................................................
6rjn.C .................................................................................................
4b7f.C .................................................................................................
6rjr.A .................................................................................................
```

```
1cf9.A IKIADQG.........EEGIVEADS....ADG..........SFMDELLTLMAAHRVWSRI..PKIDKIPA
6lfk.D PLPK..P.........DEGIVVGDK....AAD...........LAEAFCNVMRGHRIWSRE..SVAQEIAG
6jqq.C IKV.DQG.........EEGIVEADS....ADG..........SFMDELLTLMAAHRVWSRI..PKIDKIPA
4qol.B .......................................................................
1si8.C .......................................................................
2j2m.D .......................................................................
1sy7.B IALPQVTVSSEAEVHESYGVVTLKKVKPESFTDAVKIAKGAAGFLGEFFYAIAQHRNWDRELDGLHSMIAY
1qwl.B .......................................................................
4aun.A ADVP.ED.........GDGVYSEES....VD...........MFVEEFEKGLATFRFTDRF..AL......
4b2y.B ADVP.ED.........GDGVYSE.E....SVD..........MFVEEFEKGLATFRFTDRF..A.......
4b31.B ADVP.ED.........GDGVYSEES....VD...........MFVEEFEKGLATFRFTDRF..A.......
4b7a.D ADVP.ED.........GDGVYSEES....V.D..........MFVEEFEKGLATFRFTDRF..AL......
4b5k.D ADVP.ED.........GDGVYSEES.....VD..........MFVEEFEKGLATFRFTDRF..AL......
4aul.B ADVP.ED.........GDGVYSE.E....SVD..........MFVEEFEKGLATFRFTDRF..AL......
4aum.D ADVP.ED.........GDGVYSE.E....SVD..........MFVEEFEKGLATFRFTDRF..AL......
4aj9.B IGVE.EK.........EAGVYAG.A....QD...........EVIKGVEEGLKVFKFLERF..AV......
4b40.D ADVP.ED.........GDGVYSE.E....SVD..........MFVEEFEKGLATFRFTDRF..AL......
4cab.A .......................................................................
1e93.A .......................................................................
4e37.D .......................................................................
2iuf.E GQIS.SE.........RQGVYTGKN....AGD..........AFAKDIKSGLSTFKFLDRF..AV......
6pt7.A .......................................................................
2isa.H .......................................................................
1m7s.B .......................................................................
2xq1.M .......................................................................
3rgp.D .......................................................................
1dgf.C .......................................................................
1a4e.C .......................................................................
1gwe.A .......................................................................
6rjn.C .......................................................................
4b7f.C .......................................................................
6rjr.A .......................................................................
```

Supplemental Figure SF3D. Multiple sequence alignment (MSA) of the *E. coli* Fmt-methionyl-tRNA formyltransferase target protein (PDB ID: 2fmt) and homologs based on 3D structural alignments from FATCAT. Each homolog structure was individually aligned with the Fmt target protein in FATCAT and the MSA was generated using a script that compiled individual alignments from the FATCAT xml files into a single alignment based on the target sequence (See Materials and Methods for further details). ESPript was used to visualize the structure-based sequence alignment and lysine residues identified as acetylated on the Fmt *E. coli* substrate protein are highlighted in yellow. Note only amino acids that were present in the structural alignment output from FATCAT are shown and do not include residues in disordered regions. Each sequence is identified by the PDB ID and corresponding chain used for the structural alignment. All sequences are organized from highest to lowest sequence similarity based on data within FATCAT xml files.

Fmt

```
                                                          K45 K46
                                                           .  .
2fmt.A  SESLRIIFAGTPDFAARHLDALLSSG.....HNVVGVFTQPDRPAGRGKKLMPSPVKVLAEEKGLPVFQPVS.......L.....RP..QENQQLV
3r8x.A  SDSLRIIFAGTPDFAARHLGALLSSQ.....HKIVGVFTQPDRP......LTPSPVKILAEHHGIPVFQPKS.......L.....RP..EENQHLV
3q0i.A  ..SLRIVFAGTPDFAARHLAALLSSE.....HEIIAVYTQPE.........TASPVKTLALEHNVPVYQPEN.......F.....KS..DESKQQL
5uai.C  .QALRIVFAGTPEFAAEHLKALLDTP.....HRIVAVYTQPDRPAGRGQKLMPSAVKSLALEHGLPVMQPQS.......L.....RN..AEAQAEL
3tqq.A  .MSLKIVFAGTPQFAVPTLRALIDSS.....HRVLAVYTQPD..........ESPVKEIARQNEIPIIQPFS.......L.....RD..EVEQEKL
4iqf.B  NAMIKVVFMGTPDFSVPVLRRLIEDG.....YDVIGVVTQPDRPVGRKKVLTPTPVKVEAEKHGIPVLQPLR.......I.....RE..KDEYEKV
2bw0.A  .QSMKIAVIGQSLFGQEVYCHLRKEG.....HEVVGVFTVPDK......DGKADPLGLEAEKDGVPVFKYSR.......WRAKGQAL..PDVVAKY
1s3i.A  ...MKIAVIGQSLFGQEVYCQLRKEG.....HEVVGVFTIPDK......DGKADPDGLEAEKDGVPVFKFPR.......WRARGQAL..PEVVAKY
4ts4.A  ...MKIAVIGQSLFGQEVYKELKNEG.....HMIVGVFTIPDKD......GKVDPLAIEAEKDGVPVFKFPR.......WRLKGKAI..TEVVDQY
4s1n.A  ..MKKIAVFASGN..GSNFQVIAEE.......FPVEFVFSDHR..........DAYVLERAKQLGVLSYAFELKEFES..K.....ADYEAALVELL
3p9x.B  ...KRVAIFASGS..GTNAEAIIQSQKAGQLPCEVALLITDKP..........GAKVVERVKVHEIPVCALDPKTYPSKEA.....YE..IEVVQQL
3nrb.A  TDRKKVVIMVSKF..DHCLGDLLYRHRLGELDMEVVGIISNHP..........REAL..SVSLVGDIPFHYLPVTPAT...K.....AAQESQIKNIV
3tqr.A  .EPLPIVVLISGN..GTNLQAIIGAIQKGLA.IEIRAVISNRA..........DAYGLKRAQQADIPTHIIPHEEFPSRTD.....FE..STLQKTI
4pzu.D  ..MVTILILTDNVHAHALAVDLQARH.....GDMDVYQSPIG...........QL..PGV.....PRCD.V.......A.........ERVAEI
1zgh.A  ..LMNIIIATTKSWNIKNAQKFKKENES....KYNTTIITNK..........................DEL...................TFEKV
6mfx.A  ..MGRILFLTTFMSKGNKVVRYLESL.....HHEVVISQE.........................................K.......V...........HAQSA


2fmt.A  AELQADVMVVVAYGLILPKAVLEMPRLGCINVHGSLLPRWRGAAPIQRSLWAGDAETGVTIMQMDVGLDTGDMLYKLSCPITAEDTSGTLYD.KLAE
3r8x.A  ADLNADIMVVVAYGLILPAAVLAMPRLGCINVHGSLLPRWRGAAPIQRSVWAGDEKTGITIMQMDIGLDTGAMLHKIECAIQPEDTSATLYD.KLAQ
3q0i.A  AALNADLMVVVAYGLLLPKVVLDTPKLGCINVHGSILPRWRGAAPIQRSIWAGDSETGVTIMQMDVGLDTGDMLKIATLPIEASDTSASMYD.KLAE
5uai.C  AALRADLMVVVAYGLILPQAVLDIPRLGCINSHASLLPRWRGAAPIQRAVEAGDAESGVTVMQMEAGLDTGPMLLKVSTPISAADTGGSLHD.RLAA
3tqq.A  IAMNADVMVVVAYGLILPKKALNAFRLGCVNVHASLLPRWRGAAPIQRAILAGDRETGISIMQMNEGLDTGDVLAKSACVISSEDTAADLHD.RLSL
4iqf.B  LALEPDLIVTAAFGQIVPNEILEAPKYGCINVHASLLPELRGGAPIHYAIMEGKEKTGITIMYMVEKLDAGDILTQVEVEIEERETTGSLFD.KLSE
2bw0.A  QALGAELNVLPFCSQFIPMEIISAPRHGSIIYHPSLLPRHRGASAINWTLIHGDKKGGFSIFWADDGLDTGDLLLQKECEVLPDDTVSTLYNRFLFP
1s3i.A  QALGAELNVLPFCSQFIPMEVINAPRHGSIIYHPSLLPRHRGASAINWTLIHGDKKGGFTIFWADDGLDTGDLLLQKECEVLPDDTVSTLYNRFLFP
4ts4.A  KAVGAELNVLPFCSQFIPMEVIDHPKHGSIIYHPSLLPRHRGASAINWTLIHGDKKGGFTVFWADDGLDTGPILLQRECDVEPNDNVNSIYKRFLFP
4s1n.A  EEHQIDLVCLAGYMKIVGPTLLSAYEGRIVNIHPAYLPEFPGAHGIEDAWNAGVGQSGVTIHWVDSGVDTGQVIKQVRVPRLADDTIDRFEA.RIHE
3p9x.B  KEKQIDFVVLAGYMRLVGPTLLGAYEGRIVNIHPSLLPAFPGLHAIEQAIRANVKVTGVTIHYVDEGMDTGPIIAQEAVSIEEEDTLETLTT.KIQA
3nrb.A  TQSQADLIVLARYMQILSDDLSAFLSGRCINIHHSFLPGFKGAKPYHQAHTRGVKLIGATAHFVTADLDEGPIIAQDVEHVSHRDSAEDLVR.KGRD
3tqr.A  DHYDPKLIVLAGFMRKLGKAFVSHYSGRMINIHPSLLPKYTGLNTHERALAAGETEHGVSVHYVT.DLDAGPLICQARLSITPQDTPETLKT.RVHA
4pzu.D  VE.RYDLVLSFHCKQRFPAALIDGV..RCVNVHPGFNPYNRGWFPQVFSIIDGQ.KVGVTIHEIDDQLDHGPIIAQRECAIESWDSSGSVYA.RLMD
1zgh.A  KLINPEYILFPHWSWIIPKEIFENF..TCVVFHMTDLPFGRGGSPLQNLIERGIKKTKISAIKVDGGIDTGDIFFKRDLDLY..GTAEEIFM.RASK
6mfx.A  NLQEIDWIVSYAYGYILDKEIVSRFRGRIINLHPSLLPWNKGRDPVFWSVWDET.PKGVTIHLIDEHVDTGDILVQEEIAFADEDTLLDCYN.KANQ


2fmt.A  LGPQ.GLITTLKQLADGTA...........KPEVQDETLVTYAEK.LSKEEARID..WSLSAAQLERCIRAFNP..WPMSWLEI.EGQPVKVWKAS
3r8x.A  LGPQ.GLLITLQQLAAGTA...........LAEVQNETQATYAEK.LSKEEAKLD..WTLSATQLERCIRAFNP..WPVSYFIV.DEQPIKVWQAQ
3q0i.A  LGPQ.ALLECLQDIAQGTA...........VAVKQDDGLANYAHK.LSKEEARIN..WSDAATHIERCIRAFNP..WPMSHFEV.AENSIKVWQAR
5uai.C  LGPK.AVIEAIAGLAAGTL...........HGEIQDDALATYAHK.LNKDEARLD..WSRPAVELERQVRAFTP..WPVCHTSL.ADAPLKVLGAS
3tqq.A  IGAD.LLLESLAKLEKGDI...........KLEKQDEASATYASK.IQKQEALID..WRKSAVEIARQVRAFNP..TPIAFTYF.EGQPMRIWRAT
4iqf.B  AGAH.LLSKTVPLLIQGKL...........EPIKQNEEEVTFAYN.IKREQEKID..WTKTGEEVYNHIRGLNP..WPVAYTTL.AGQVVKVWWGE
2bw0.A  EGIK.GMVQAVRLIAEGKA...........PRLPQPEEGATYEGI.QKKETAKIN..WDQPAEAIHNWIRGNDK..VPGAWTEA.CEQKLTFFNST
1s3i.A  EGIK.GMVQAVRLIAEGTA...........PRCPQSEEGATYEGI.QKKETAKIN..WDQPAEAIHNWIRGNDK..VPGAWTEA.CGQKLTFFNST
4ts4.A  EGVK.GMVEAVRLIATGKA...........PRIKQPEEGATYECI.QKKENSKID..WNQPAEAIHNWIRGNDR..VPGAWAEI.DGKSVSFYGST
4s1n.A  AEYR.LYPEVVKALFT.......................................................................................
3p9x.B  VEHR.LYPATLHKLLSKAE....................................................................................
3nrb.A  IERR.VLSRAVLLFLEDRL...........IVNGERTVVFAD...........................................................
3tqr.A  LEHI.IYPEVLSWFAAGRLNYHNNQVFLDGKPLAKS...................................................................
4pzu.D  IERE.LVLEHFDAIRDGSY...........TAKSPATE.GNLNLKKDFEQLRRLDLNERGTFGHFLNRLRALTHDDFRNAWFVDASGRKVFVRVVL
1zgh.A  IIFNDMIPELLTKR...P...........VPQKQEGEATVFQRR.KP.EQSEIS..PDFDLEKIYDYIRMLDGEGYPRAFIKY.GKYRLEFSRAS
6mfx.A  AIEE.LFIREWENIVHGRI...........APYRQTA.GGTLHFK.ADRDFYKNL..NMTTVRELLALKRLSAE..PIDKTFHQ.LFEQ.......


2fmt.A  VIDTATN........A.........APGTILEANKQGIQVATGD.G.ILNLLSLQPA.GKKAMSAQDLLNSRR.EWFVP.GNRL.
3r8x.A  VLPAGED........A.........EPGTIIHADKHGIQVATAD.G.VLNITQLQPA.GKKAMSAADLLNSRR.EWFIP.GSQL.
3q0i.A  VETRAVT........Q.........TPGTIIQADKSGIYVATGQ.D.VLVLESLQIP.GKKALPVQDILNARA.DWFSV.GSQLS
5uai.C  LGQ..GS........G.........APGTILEASRDGLLVACGE.G.ALRLTRLQLP.GGKPLAFADLYNSRR.EQFAA.GQVLG
3tqq.A  VVDEKTD........F.........EPGVLVDADKKGISIAAGS.G.ILRLHQLQLP.GKRVCSAGDFINAHG.DKLIPGKTVFG
4iqf.B  KVPVTKS........A.........EAGTIVAIEEDGFVVATGN.ETGVKITELQPS.GKKRMSCSQFLRGTK...PEI.GTKLG
2bw0.A  LNTSGLV........PEGDALPIP.GAHRPGVVTKAGLILFGNDDK.MLLVKNIQLE.DGKMILASNFFK..............
1s3i.A  LNTSGLSTQGEALPIP.........GAHRPGVVTKAGLILFGNDDR.MLLVKNIQLE.DGKMMPASQFFK..............
4ts4.A  LLENDHF........SSNGQPLEIPGASRAALVTKNGLVLFGND.G.KMLLVKNLQFEDGKMIPGSQYFK..............
4s1n.A  ......................................................................................
3p9x.B  ......................................................................................
3nrb.A  ......................................................................................
3tqr.A  ......................................................................................
4pzu.D  ......................................................................................
1zgh.A  MKN.................................GKIIADVEI.IE.......................
6mfx.A  ....QVE........M.........TPDHVAVVDRGQSLTYK...Q.LN.............ERANQLAHHLRGKGVKP.DDQVA
```
