# Supplementary material for: Investigation of the Importance of Protein 3D Structure for Assessing Conservation of Lysine Acetylation Sites in Protein Homologs
Source: Front Microbiol. 2022 Jan 31;12:805181. doi: 10.3389/fmicb.2021.805181 (PMC8843374; doi:10.3389/fmicb.2021.805181)
Supplement: Supplementary file 7 [file Data_Sheet_7.PDF]

Supplemental Figure SF4. **Multiple sequence alignments (MSAs) of homologs of KAT substrate proteins Adk, Icd, KatE, Fmt, and YaaA identified from the UniProt database (https://www.uniprot.org/); not restricted to 3D structures in the PDB.** See Materials and Methods for more detailed information. The *E. coli* substrate protein sequence and secondary structure of the target structure used in our comparative analysis is shown on top (PDB ID 1ake for Adk, PDB ID 1ai2 for Icd, PDB ID 1cf9 for KatE, and PDB ID 2fmt for Fmt). Uniprot IDs for each protein are listed adjacent to each sequence and further information about the proteins are found in the tables after the MSAs.

Adk-adenylate kinase

```
sp|P69441|KAD_ECOLI   β1 TT α1 β2 α2 η1 α3 α4 η2 β3 α5 β4 η3 α6
                      1 10 20 30 40 50 60 70 80 90 100 110 120

sp|P69441|KAD_ECOLI                   MRIILLGAPGAGKGTQAQFIMEKYGIPQISTGDMLRAAVKSGSELGKQAKDIMDAGKLVTDELVIALVKERIAQEDCRNGFLLDGFPRTIPQADAMKEAGINVDYVLEFDVPDELIVDRI
sp|Q83M40|KAD_SHIFL                   MRIILLGAPGAGKGTQAQFIMEKYGIPQISTGDMLRAAVKSGSELGKQAKDIMDAGKLVTDELVIALVKERIAQEDCRNGFLLDGFPRTIPQADAMKEAGINVDYVLEFDVPDELIVDRI
tr|A0A653BDL8|A0A653BDL8_CALMS        MRIILLGAPGAGKGTQAQFIMEKYGIPQISTGDMLRAAVKSGSELGKQAKDIMDAGKLVTDELVIALVKERIAQEDCRNGFLLDGFPRTIPQADAMKEAGINVDYVLEFDVPDELIVDRI
tr|E3G6A2|E3G6A2_ENTLS                MRIILLGAPGAGKGTQAQFIMEKYGIPQISTGDMLRAAVKSGSELGKQAKDIMDAGKLVTDELVIALVKERIAQEDCRNGFLLDGFPRTIPQADAMKEAGINVDYVLEFDVPDELIVDRI
sp|B5Y0N3|KAD_KLEP3                   MRIILLGAPGAGKGTQAQFIMEKYGIPQISTGDMLRAAVKSGSELGKQAKDIMDAGKLVTDELVIALVKERIAQEDCRNGFLLDGFPRTIPQADAMKEAGITVDYVLEFDVPDELIVDRI
sp|A8AJW9|KAD_CITK8                   MRIILLGAPGAGKGTQAQFIMEKYGIVQISTGDMLRAAVKSGSELGKQAKDIMDAGKLVTDELVIALVKERIAQDDCRNGFLLDGFPRTIPQADAMKEAGIAVDYVLEFDVPDELIVDRI
tr|U4UP47|U4UP47_DENPD                MRIILLGAPGAGKGTQAQFIMENYGIPQISTGDMLRAAVKAGSDLGKKAKEIMDAGKLVTDELVIALVKERITQEDCRNGFLLDGFPRTIPQADAMKEAGISVDFVLEFDVPDELIVDRI
sp|B1JHN1|KAD_YERPY                   MRIILLGAPGAGKGTQAQFIMEKYGIPQISTGDMLRAAVKAGSELGLKAKEIMDAGKLVTDELVIALVKERITQEDCRDGFLLDGFPRTIPQADAMKEAGIKVDYVLEFDVPDELIVERI
sp|C6DB87|KAD_PECCP                   MRIILLGAPGAGKGTQAQFIMEKYGIPQISTGDMLRAAVKAGTELGKQAKEIMDAGKLVTDELVIALVKERIAQDDCRNGFLLDGFPRTIPQADAMKDVGINVDYVIEFAVPDELIIDRI
sp|P43412|KAD_YEREN                   MRIILLGAPGAGKGTQAQFIMEKYGIPQMCTGDMLRAAVKAGSELGLKAKEIMDAGKLVTHELVIALVKERITQDDCRDGFLLHGFPRTIPQADAMKEAGIKVDYVLEFDVPDELIVDRI
sp|B4F1Q2|KAD_PROMH                   MRIILLGAPGAGKGTQAQFIKENYGIPQISTGDMLRAAVSAGTELGLKAKALMDNGQLVTDELVIALVKERIKQDDCRNGFLLDGFPRTIPQADAMKEAGINVDFVLEFAVPDEIIVERI
sp|A7MT47|KAD_VIBCB                   MRIILLGAPGAGKGTQANFIMDKYGIPQISTGDMLRAAIKAGTELGKQAKAVIDAGQLVSDEIILGLIKERIAQDDCEKGFLLDGFPRTIPQADGLKEMGVDVDYVIEFDVADDVIVERM
sp|Q8DFM1|KAD_VIBVU                   MRIILLGAPGAGKGTQAQFIMEKYGIPQISTGDMLRAAIKAGTELGKQAKAVIDAGQLVSDEIILGLIKERIAQEDCAKGFLLDGFPRTIPQADGLKEMGVAVDYVIEFDVADDVIVERM
sp|C4K7W8|KAD_HAMD5                   MRIILLGAPGAGKGTQAQFIMKKYGIVQISTGDMLRAAVKAGTKLGQQVQGIMAAGKLVTDDLVIALVKERIRQEDCKEGFLLDGFPRTIPQADAMAEADIKIDYVLELVVPDEFIVERI
tr|A0A640Q9T2|A0A640Q9T2_9FLAO        MRIILLGAPGAGKGTQAQFLMGKYGIPQISTGDMLRAAIKAGTELGLEAKRVMDEGKLVSDDIIIGLVKERIAQEDCKDGFLLDGFPRTIPQADAMKEAGVSVDHCIEFDVPDDVIVERM
sp|P24323|KAD_HAEIN                   MKIILLGAPGAGKGTQAQFIMNKFGIPQISTGDMFRAAIKAGTELGKQAKALMDEGKLVPDELTVALVKDRIAQADCTNGFLLDGFPRTIPQADALKDSGVKIDFVLEFDVPDEVIVERM
sp|Q7NSS7|KAD_CHRVO                   MRLILLGAPGAGKGTQANYIREKFGIPQISTGDMLRAAVKAGTPLGLEAKAIMDAGGLVRDDIIIGLVKERIAQDDCANGFLFDGFPRTIPQAEAMIAAGVDIDYVVEIDVPDAAIVERM
tr|A0A6A5LFQ2|A0A6A5LFQ2_LUPAL        MRLILLGAPGAGKGTQANFIKEKYNIPQISTGDMLRAAIKAGTELGLAAKKVMDAGQLVSDDIIIGLVKERLKEADCANGYLFDGFPRTVAQADAMKDSGVQIDYVLEIDVPDELILERM
sp|A4G7X8|KAD_HERAR                   MRLILLGAPGAGKGTQAGFIKDKYNIPQISTGDMLRAAVKAGTPLGIAAKKIMDEGGLVSDDIIIGLVKDRLKDGDCANGYLFDGFPRTIPQADAMKDAGVAIDYVLEIDVPDEAIVERM
tr|J2M519|J2M519_9BURK                MRLILLGAPGAGKGTQATFIKEKYNIPQISTGDMLRAAVAAGTELGIAAKKVMDAGGLVSDDIIIGLVKDRLKQADCANGYLFDGFPRTVPQADAMKDAGVKIDYVLEIDVPDSAIVERM

sp|P69441|KAD_ECOLI   β5 β6 TTT TT TTT TT α7 α8 β7 TT α9
                      130 140 150 160 ★170 180 190 200 210

sp|P69441|KAD_ECOLI                   VGRRVHAPSGRVYHVKFNPPKVEGKDDVTGEELTTRKDDQEETVRKRLVEYHQMTAPLIGYYSKEAEAGNT...KYAKVDGTKPVAEVRADLEKILG..
sp|Q83M40|KAD_SHIFL                   VGRRVHAPSGRVYHVKFNPPKVEGKDDVTGEELTTRKDDQEETVRKRLVEYHQMTAPLIGYYSKEAEAGNT...KYAKVDGTKQVAEVRADLEKILG..
tr|A0A653BDL8|A0A653BDL8_CALMS        VGRRVHAASGRVYHIKFNPPKVEGKDDVTGEELTTRKDDQEETVRKRLVEYHQMTAPLIGYYSKEAQAGNT...KYAKVDGTKAVADVRAELEKILG..
tr|E3G6A2|E3G6A2_ENTLS                VGRRVHAASGRVYHIKFNPPKVEGKDDVTGEELTTRKDDQEETVRKRLVEYHQMTAPLIGYYSKEAQAGNT...RYAKVDGTKAVADVRAELEKILG..
sp|B5Y0N3|KAD_KLEP3                   VGRRVHAASGRVYHIKFNPPKVEGKDDVTGEELTTRKDDQEETVRKRLVEYHQMTAPLIGYYTKEAQAGNT...KYAKVDGTKAVADVRAELEKILG..
sp|A8AJW9|KAD_CITK8                   VGRRVHAASGRVYHIKFNPPKVEGKDDVTGEELTTRKDDQEETVRKRLVEYHQMTAPLIGYYQKEAEAGNT...KYAKVDGTQAVADVRADLEKILG..
tr|U4UP47|U4UP47_DENPD                VGRRVHAPSGRVYHIKFNPPKVEGKDDATGEELTTRKDDQEETVRKRLVEYHELTEPLVSYYGKEAEAGNT...KYVKIDGTKKVSEVREELAAILG..
sp|B1JHN1|KAD_YERPY                   VGRRVHAASGRVYHVKFNPPKVEDKDDVTGEELTIRKDDQEATVRKRLIEYHQQTAPLVSYYHKEADAGNT...QYFKLDGTRNVAEVSAELATILG..
sp|C6DB87|KAD_PECCP                   VGRRVHAASGRVYHVKFNPPKVEGKDDVTGEDLTIRKDDHEDTVRKRLVEYHQQTAPLVSYYQKEADAGNT...RYFKVEGTRKVEEVRAELETILG..
sp|P43412|KAD_YEREN                   VGRRVHAASGRVYHIKFNPPKVEDKDDVTGEELTIRKDDQEATVRKRLVEYHQQTAPLVSYYRKEADAGNT...QYFKLDGTRKVAEVSAELATILG..
sp|B4F1Q2|KAD_PROMH                   VGRRVHAPSGRVYHIKFNPPKVENRDDVTGEELTTRKDDQEETVRKRLIEYHSQTAPLVSYYQNEAKAGNA...QYHKIDGTQKVSEINAELKNILG..
sp|A7MT47|KAD_VIBCB                   AGRRAHLPSGRTYHVVYNPPKVEGKDDVTGEDLVVRDDDKEETVRARLGVYHEQTAPLINYYGKEAEAGNT...KYLKFDGTKQVAEVSADIEKALA..
sp|Q8DFM1|KAD_VIBVU                   AGRRAHLPSGRTYHVVYNPPKVEGKDDVTGEDLVVRDDDKEETVRARLGVYHSQTAPLIEYYGKEAAEGNT...KYLKFDGTKQVAQVSADIEKALA..
sp|C4K7W8|KAD_HAMD5                   SGRRVHMPSGRIYHLKFNPPKITDKDDMTGESLTLRKDDQETTVRERLLEYHQQTMALVDYYRQRAKRGDT...KYFKLDGTRTVNEVSQQLSSILGES
tr|A0A640Q9T2|A0A640Q9T2_9FLAO        GGRRVHPASGRVYHVVYNPPKVEGKDNETGDDLIVRDDDKEETVRKRLAIYHEQTKPLVNYYSAEADAGNC...EYHKLDGTRPVEEVSAELAERLG..
sp|P24323|KAD_HAEIN                   SGRRVHQASGRSYHIVYNPPKVEGKDDVTGEDLIIRADDKPETVLDRLAVYHKQTSPLIDYYQAEAKAGNT...QYFRLDGTQKVEEVSQELDKILG..
sp|Q7NSS7|KAD_CHRVO                   AGRRVHLASGRTYHVTFNPPKAAGKDDVTGEDLVQRDDDKEETVKKRLAVYHEQTAVLVGFYGKLAESGSAKAPKYVKIDGTRAVETVRDDVLKALGA.
tr|A0A6A5LFQ2|A0A6A5LFQ2_LUPAL        TGRRSHPGSGRVYHVKFNPPKVEGKDDVTGEPLVQRDDDTEETVKKRLAVYHNQTEVLLGYYNKWAESGLPGAPKYRKISGVGPVEQVRDAAFAALAG.
sp|A4G7X8|KAD_HERAR                   SGRRVHPASGRTYHVKFNPPKVAGRDDVTGEELIQRDDDKEETVKKRLSVYHDQTEVLVGYYNKWAESGQPGAPKYRKIAGVGPVDQIRDNAFKALEG.
tr|J2M519|J2M519_9BURK                SGRRVHQPSGRTYHVKFNPPKVEGKDDVTGEALIQRDDDKEETVKKRLSVYHEQTEVLVGYYGKWAESGQPGAPQYRKIAGVGPVEQIRESAFAALSS.
```

Sequences used in Adk alignment:

Order	% Sequence	UniProt ID	Organism	Length	Entry name	Protein names	Gene names
1	100	P69441	Escherichia coli (strain K12)	214	KAD_ECOLI	Adenylate kinase (AK) (EC 2.7.4.3) (ATP-AMP transphosphorylase) (ATP:AMP phosphotransferase) (Adenylate monophosphate kinase)	adk dnaW plsA b0474 JW0463
2	99.53	Q83M40	Shigella flexneri	214	KAD_SHIFL	Adenylate kinase (AK) (EC 2.7.4.3) (ATP-AMP transphosphorylase) (ATP:AMP phosphotransferase) (Adenylate monophosphate kinase)	adk SF0419 S0426
3	97.2	A0A653BDL8	Callosobruchus maculatus (Southern cowpea weevil) (Pulse bruchid)	214	A0A653BDL8_CALMS	ADK_lid domain-containing protein	CALMAC_LOCUS138
4	96.73	E3G6A2	Enterobacter lignolyticus (strain SCF1)	214	E3G6A2_ENTLS	Adenylate kinase (AK) (EC 2.7.4.3) (ATP-AMP transphosphorylase) (ATP:AMP phosphotransferase) (Adenylate monophosphate kinase)	adk Entcl_3316
5	96.26	B5Y0N3	Klebsiella pneumoniae (strain 342)	214	KAD_KLEP3	Adenylate kinase (AK) (EC 2.7.4.3) (ATP-AMP transphosphorylase) (ATP:AMP phosphotransferase) (Adenylate monophosphate kinase)	adk KPK_4224
6	95.79	A8AJW9	Citrobacter koseri (strain ATCC BAA-895 / CDC 4225-83 / SGSC4696)	214	KAD_CITK8	Adenylate kinase (AK) (EC 2.7.4.3) (ATP-AMP transphosphorylase) (ATP:AMP phosphotransferase) (Adenylate monophosphate kinase)	adk CKO_02675
7	88.79	U4UP47	Dendroctonus ponderosae (Mountain pine beetle)	214	U4UP47_DENPD	ADK_lid domain-containing protein	D910_00396
8	87.38	B1JHN1	Yersinia pseudotuberculosis serotype O:3 (strain YPIII)	214	KAD_YERPY	Adenylate kinase (AK) (EC 2.7.4.3) (ATP-AMP transphosphorylase) (ATP:AMP phosphotransferase) (Adenylate monophosphate kinase)	adk YPK_3194
9	87.38	C6DB87	Pectobacterium carotovorum subsp. carotovorum (strain PC1)	214	KAD_PECCP	Adenylate kinase (AK) (EC 2.7.4.3) (ATP-AMP transphosphorylase) (ATP:AMP phosphotransferase) (Adenylate monophosphate kinase)	adk PC1_1080
10	85.51	P43412	Yersinia enterocolitica	214	KAD_YEREN	Adenylate kinase (AK) (EC 2.7.4.3) (ATP-AMP transphosphorylase) (ATP:AMP phosphotransferase) (Adenylate monophosphate kinase)	adk
11	81.31	B4F1Q2	Proteus mirabilis (strain HI4320)	214	KAD_PROMH	Adenylate kinase (AK) (EC 2.7.4.3) (ATP-AMP transphosphorylase) (ATP:AMP phosphotransferase) (Adenylate monophosphate kinase)	adk PMI2184
12	74.77	A7MT47	Vibrio campbellii (strain ATCC BAA-1116 / BB120)	214	KAD_VIBCB	Adenylate kinase (AK) (EC 2.7.4.3) (ATP-AMP transphosphorylase) (ATP:AMP phosphotransferase) (Adenylate monophosphate kinase)	adk VIBHAR_01328
13	74.77	Q8DFM1	Vibrio vulnificus (strain CMCP6)	214	KAD_VIBVU	Adenylate kinase (AK) (EC 2.7.4.3) (ATP-AMP transphosphorylase) (ATP:AMP phosphotransferase) (Adenylate monophosphate kinase)	adk VV1_0188
14	73.83	C4K7W8	Hamiltonella defensa subsp. Acyrthosiphon pisum (strain 5AT)	216	KAD_HAMD5	Adenylate kinase (AK) (EC 2.7.4.3) (ATP-AMP transphosphorylase) (ATP:AMP phosphotransferase) (Adenylate monophosphate kinase)	adk HDEF_2095
15	72.43	A0A640Q9T2	Tenacibaculum sp. KUL118	214	A0A640Q9T2_9FLAO	Adenylate kinase (AK) (EC 2.7.4.3) (ATP-AMP transphosphorylase) (ATP:AMP phosphotransferase) (Adenylate monophosphate kinase)	adk KUL118_53660
16	71.5	P24323	Haemophilus influenzae (strain ATCC 51907 / DSM 11121 / KW20 / Rd)	214	KAD_HAEIN	Adenylate kinase (AK) (EC 2.7.4.3) (ATP-AMP transphosphorylase) (ATP:AMP phosphotransferase) (Adenylate monophosphate kinase)	adk HI_0349
17	68.69	Q7NSS7	Chromobacterium violaceum (strain ATCC 12472 / DSM 30191 / JCM 1249 / NBRC 12614 / NCIMB 9131 / NCTC 9757)	218	KAD_CHRVO	Adenylate kinase (AK) (EC 2.7.4.3) (ATP-AMP transphosphorylase) (ATP:AMP phosphotransferase) (Adenylate monophosphate kinase)	adk CV_3343
18	66.82	A0A6A5LFQ2	Lupinus albus (White lupine) (Lupinus termis)	218	A0A6A5LFQ2_LUPAL	ATP:AMP phosphotransferase (EC 2.7.4.3)	Lal_00015020
19	66.82	A4G7X8	Herminiimonas arsenicoxydans	218	KAD_HERAR	Adenylate kinase (AK) (EC 2.7.4.3) (ATP-AMP transphosphorylase) (ATP:AMP phosphotransferase) (Adenylate monophosphate kinase)	adk HEAR2487
20	66.82	J2M519	Herbaspirillum sp. CF444	218	J2M519_9BURK	Adenylate kinase (AK) (EC 2.7.4.3) (ATP-AMP transphosphorylase) (ATP:AMP phosphotransferase) (Adenylate monophosphate kinase)	adk PMI16_01020

Icd-isocitrate dehydrogenase

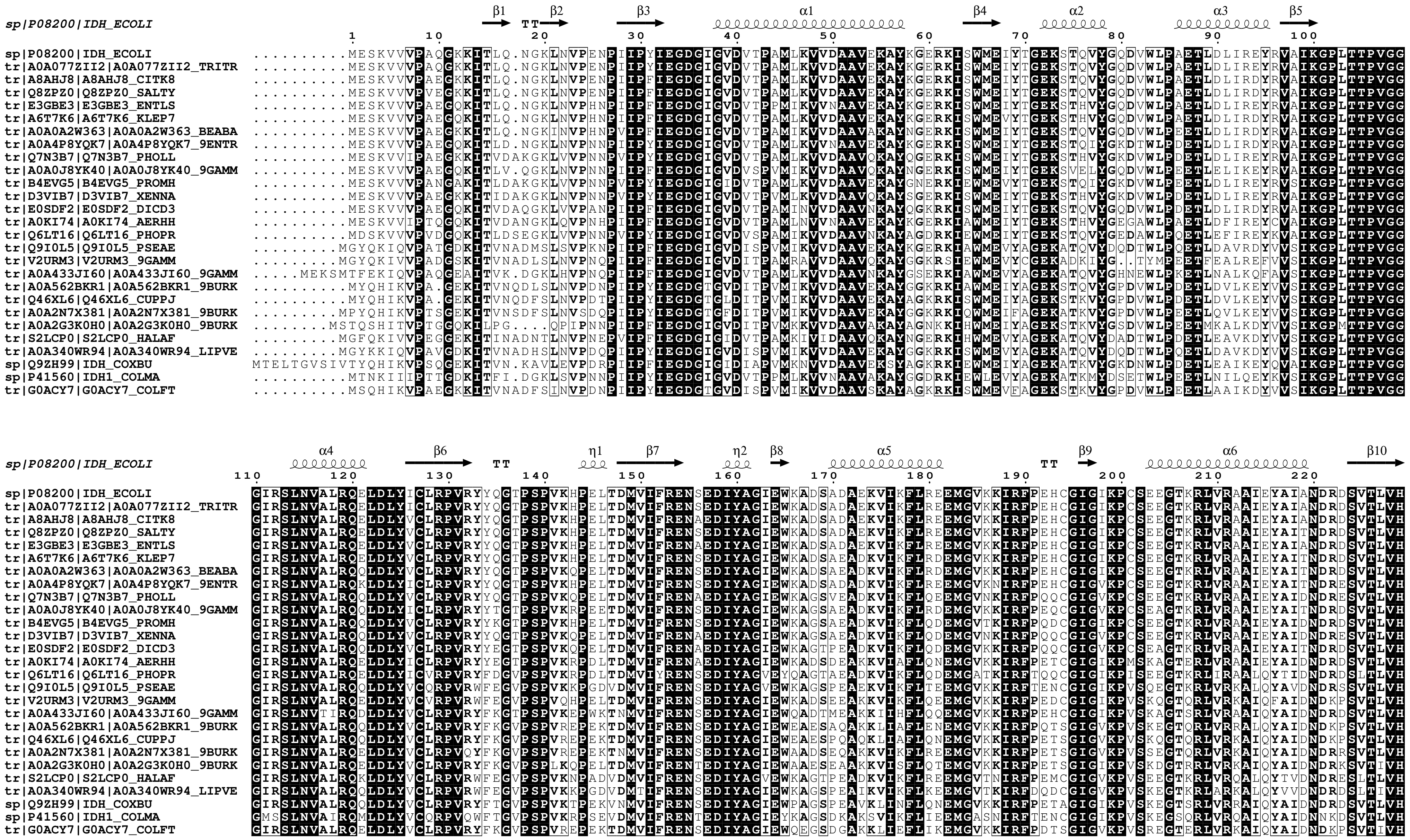

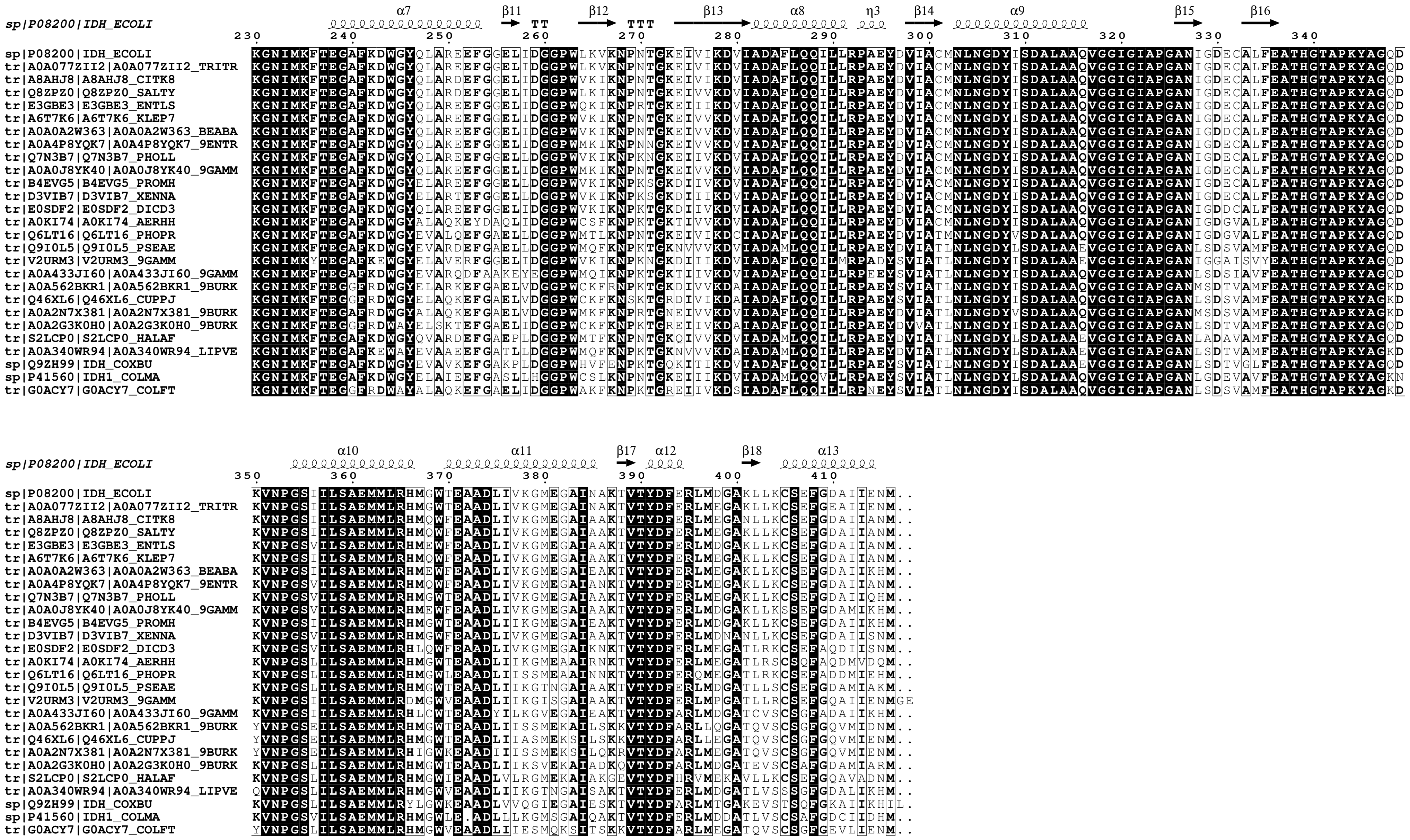
α7
β11
TT
β12
TTT
β13
α8
η3
β14
α9
β15
β16
sp|P08200|IDH_ECOLI
230
240
250
260
270
280
290
300
310
320
330
340
sp|P08200|IDH_ECOLI KGNIMKFTEGAFKDWGYQLAREEFGGELIDGGPWLKVKNPNTGKEIVIKDVIADAFLQQILLRPAEYDVIACMNLNGDYISDALAAQVGGIGIAPGANIGDECALFEATHGTAPKYAGQD
tr|A0A077ZII2|A0A077ZII2_TRITR KGNIMKFTEGAFKDWGYQLAREEFGGELIDGGPWLKVKNPNTGKEIVIKDVIADAFLQQILLRPAEYDVIACMNLNGDYISDALAAQVGGIGIAPGANIGDECALFEATHGTAPKYAGQD
tr|A8AHJ8|A8AHJ8_CITK8 KGNIMKFTEGAFKDWGYQLARDEFGGELIDGGPWVKIKNPNTGKEIVIKDVIADAFLQQILLRPAEYDVIACMNLNGDYISDALAAQVGGIGIAPGANIGDECALFEATHGTAPKYAGQD
tr|Q8ZPZ0|Q8ZPZ0_SALTY KGNIMKFTEGAFKDWGYQLARDEFGGELIDGGPWLKIKNPNTGKEIVVKDVIADAFLQQILLRPAEYDVIACMNLNGDYISDALAAQVGGIGIAPGANIGDECALFEATHGTAPKYAGQD
tr|E3GBE3|E3GBE3_ENTLS KGNIMKFTEGAFKDWGYQLAREEFGGELIDGGPWQKIKNPRTGKEIIIKDVIADAFLQQILLRPAEYDVIACMNLNGDYISDALAAQVGGIGIAPGANIGDECALFEATHGTAPKYAGQD
tr|A6T7K6|A6T7K6_KLEP7 KGNIMKFTEGAFKDWGYQLAREEFGGELIDGGPWVKIKNPNTGKEIVVKDVIADAFLQQILLRPAEYDVIACMNLNGDYISDALAAQVGGIGIAPGANIGDECALFEATHGTAPKYAGQD
tr|A0A0A2W363|A0A0A2W363_BEABA KGNIMKFTEGAFKDWGYQLAREEFGGELIDGGPWVKIKNPNTGKEIVVKDVIADAFLQQILLRPAEYDVIACMNLNGDYISDALAAQVGGIGIAPGANIGDDCALFEATHGTAPKYAGQD
tr|A0A4P8YQK7|A0A4P8YQK7_9ENTR KGNIMKFTEGAFKDWGYQLAKEEFGGELIDGGPWMKIKNPNNGKEIVVKDVIADAFLQQILLRPAEYDVIACMNLNGDYISDALAAQVGGIGIAPGANIGDECALFEATHGTAPKYAGQD
tr|Q7N3B7|Q7N3B7_PHOLL KGNIMKFTEGAFKDWGYQLAREEFGGELLDGGPWVKIKNPKNGKEIIVKDVIADAFLQQILLRPAEYDVIACMNLNGDYISDALAAQVGGIGIAPGANIGDECALFEATHGTAPKYAGQD
tr|A0A0J8YK40|A0A0J8YK40_9GAMM KGNIMKFTEGAFKDWGYQLAKEEFGGELIDGGPWMKIKNPNTGKEIVVKDVIADAFLQQILLRPAEYDVIACMNLNGDYISDALAAQVGGIGIAPGANIGDECALFEATHGTAPKYAGQD
tr|B4EVG5|B4EVG5_PROMH KGNIMKFTEGAFKDWGYELAREEFGGELLDGGPWVKIKNPKSGKDIIVKDVIADAFLQQILLRPAEYDVIACMNLNGDYISDALAAQVGGIGIAPGANIGDECALFEATHGTAPKYAGQD
tr|D3VIB7|D3VIB7_XENNA KGNIMKFTEGAFKDWGYELARTEFGGELLDGGPWVKIKNPKSGKDIIIKDVIADAFLQQILLRPAEYDVIACMNLNGDYISDALAAQVGGIGIAPGANIGDECALFEATHGTAPKYAGQD
tr|E0SDF2|E0SDF2_DICD3 KGNIMKFTEGAFKDWGYQLAREEFGGELIDGGPWVKIKNPKTGKDIIVKDVIADAFLQQILLRPAEYDVIACMNLNGDYISDALAAQVGGIGIAPGANIGDDCALFEATHGTAPKYAGQD
tr|A0KI74|A0KI74_AERHH KGNIMKFTEGAFKDWGYALAQKEYDAQLIDGGPWCSFKNPKTGKTIVVKDVIADAFLQQILLRPAEYDVIACMNLNGDYISDALAAQVGGIGIAPGANIGDGVALFEATHGTAPKYAGQD
tr|Q6LT16|Q6LT16_PHOPR KGNIMKFTEGAFKDWGYEVALQEFGAELLDGGPWMTLKNPNTGKEIVIKDCIADAFLQQILLRPAEYDVIATMNLNGDYVSDALAAQVGGIGIAPGANIGDGVALFEATHGTAPKYAGQD
tr|Q9I0L5|Q9I0L5_PSEAE KGNIMKFTEGAFKDWGYEVARDEFGAELLDGGPWMQFKNPKTGKNVVVKDVIADAMLQQILLRPAEYDVIATLNLNGDYLSDALAAEVGGIGIAPGANLSDSVAMFEATHGTAPKYAGQD
tr|V2URM3|V2URM3_9GAMM KGNIMKYTEGAFKEWGYELAVERFGGELIDGGPWVKIKNPKNGKDIIIKDVIADAFLQQILMRPADYSVIATLNLNGDYISDALAAEVGGIGIAPGANIGGAISVYEATHGTAPKYAGQD
tr|A0A433JI60|A0A433JI60_9GAMM KGNIMKFTEGAFKDWGYEVARQDFAAKEYEGGPWMQIKNPKTGKTIIVKDVIADAFLQQILLRPEEYSVIATLNLNGDYISDALAAQVGGIGIAPGANLSDSIAVFEATHGTAPKYAGQD
tr|A0A562BKR1|A0A562BKR1_9BURK KGNIMKFTEGGFRDWGYELARKEFGAELVDGGPWCKFRNPKTGKDIVVKDAIADAFLQQILLRPAEYSVIATLNLNGDYISDALAAQVGGIGIAPGANMSDTVAMFEATHGTAPKYAGKD
tr|Q46XL6|Q46XL6_CUPPJ KGNIMKFTEGGFRDWGYELAQKEFGAELVDGGPWCKFKNSKTGRDIVIKDAIADAFLQQILLRPAEYSVIATLNLNGDYISDALAAQVGGIGIAPGANLSDSVAMFEATHGTAPKYAGKD
tr|A0A2N7X381|A0A2N7X381_9BURK KGNIMKFTEGAFRDWGYALAQKEFGAELVDGGPWMKFKNPRTGNEIVVKDVIADAFLQQILLRPAEYDVIATLNLNGDYVSDALAAQVGGIGIAPGANMSDSVAMFEATHGTAPKYAGKD
tr|A0A2G3K0H0|A0A2G3K0H0_9BURK KGNIMKFTEGGFRDWAYELSKTEFGAELIDGGPWCKFKNPKTGREIIVKDAIADAFLQQILLRPAEYDVVATLNLNGDYISDALAAQVGGIGIAPGANLSDTVAMFEATHGTAPKYAGQD
tr|S2LCP0|S2LCP0_HALAF KGNIMKFTEGAFKDWGYQVARDEFGAEPLDGGPWMTFKNPNTGKQIVVKDVIADAMLQQILLRPAEYDVIATLNLNGDYLSDALAAEVGGIGIAPGANLADAVAMFEATHGTAPKYAGQD
tr|A0A340WR94|A0A340WR94_LIPVE KGNIMKFTEGAFKEWAYEVAAEEFGATLLDGGPWMQFKNPKTGKNVVVKDAIADAMLQQILLRPAEYDVIATLNLNGDYLSDALAAEVGGIGIAPGANLSDTVAMFEATHGTAPKYAGKD
sp|Q9ZH99|IDH_COXBU KGNIMKFTEGAFKDWGYEVAVKEFGAKPLDGGPWHVFENPKTGQKITIKDVIADAFLQQILLRPAEYSVIATLNLNGDYISDALAAEVGGIGIAPGANLSDTVGLFEATHGTAPKYAGQD
sp|P41560|IDH1_COLMA KGNIMKFTEGAFKDWGYELAIEEFGASLLHGGPWCSLKNPNTGKEIIIKDVIADAMLQQVLLRPAEYSVIATLNLNGDYLSDALAAQVGGIGIAPGANLGDEVAVFEATHGTAPKYAGKN
tr|G0ACY7|G0ACY7_COLFT KGNIMKFTEGGFRDWAYALAQKEFGAELIDGGPWAKFKNPKTGREIIVKDSIADAFLQQILLRPNEYSVIATLNLNGDYISDALAAQVGGIGIAPGANLSDSVAMFEATHGTAPKYAGKD
α10
α11
β17
α12
β18
α13
sp|P08200|IDH_ECOLI
350
360
370
380
390
400
410
sp|P08200|IDH_ECOLI KVNPGSIILSAEMMLRHMGWTEAADLIVKGMEGAINAKTVTYDFERLMDGAKLLKCSEFGDAIIENM..
tr|A0A077ZII2|A0A077ZII2_TRITR KVNPGSIILSAEMMLRHMGWTEAADLIVKGMEGAINAKTVTYDFERLMEGAKLLKCSEFGEAIIENM..
tr|A8AHJ8|A8AHJ8_CITK8 KVNPGSIILSAEMMLRHMQWFEAADLIVKGMEGAIAAKTVTYDFERLMEGANLLKCSEFGDAIIANM..
tr|Q8ZPZ0|Q8ZPZ0_SALTY KVNPGSIILSAEMMLRHMQWFEAADLIVKGMEGAIAAKTVTYDFERLMEGAKLLKCSEFGDAIIANM..
tr|E3GBE3|E3GBE3_ENTLS KVNPGSVILSAEMMLRHMEWFEAADLIVKGMEGAIAAKTVTYDFERLMDGAKLLKCSEFGDAIIANM..
tr|A6T7K6|A6T7K6_KLEP7 KVNPGSIILSAEMMLRHMQWFEAADLIVKGMEGAIAAKTVTYDFERLMEGAKLLKCSEFGDAIIANM..
tr|A0A0A2W363|A0A0A2W363_BEABA KVNPGSIILSAEMMLRHMEWFEAADLIVKGMEGAIEAKTVTYDFERLMEGAKLLKCSEFGDAIIKHM..
tr|A0A4P8YQK7|A0A4P8YQK7_9ENTR KVNPGSVILSAEMMLRHMQWFEAADLIVKGMEGAIANKTVTYDFERLMEGAKLLKCSEFGDAIIANM..
tr|Q7N3B7|Q7N3B7_PHOLL KVNPGSVILSAEMMLRHMGWTEAADLIVKGMEGAIAAKTVTYDFERLMEGAKLLKCSEFGDAIIQHM..
tr|A0A0J8YK40|A0A0J8YK40_9GAMM KVNPGSVILSAEMMLRHMEWFEAADLIVKGMEGAIAAKTVTYDFERLMDGAKLLKSSEFGDAMIKHM..
tr|B4EVG5|B4EVG5_PROMH KVNPGSIILSAEMMLRHMGWTEAADLIIKGMEGAIEAKTVTYDFERLMDGAKLLKCSEFGDAIIKHM..
tr|D3VIB7|D3VIB7_XENNA KVNPGSVILSAEMMLRHMGWFEAADLIVKGMEGAIAAKTVTYDFERLMDNANLLKCSEFGDAIISNM..
tr|E0SDF2|E0SDF2_DICD3 KVNPGSVILSAEMMLRHLQWFEAADLIVKGMEGAIKNKTVTYDFERLMEGATLRKCSEFAQDIISNM..
tr|A0KI74|A0KI74_AERHH KVNPGSLILSAEMMLRHMGWTEAADLIIKGMEAAIRNKTVTYDFERLMEGATLRSCSQFAQDMVDQM..
tr|Q6LT16|Q6LT16_PHOPR KVNPGSLILSAEMMLRHMGWLEAADLIISSMEAAINNKTVTYDFERQMEGATLRKCSEFADDMIEHM..
tr|Q9I0L5|Q9I0L5_PSEAE KVNPGSLILSAEMMLRHMGWTEAADLIIKGTNGAIAAKTVTYDFERLMDGATLLSCSEFGDAMIAKM..
tr|V2URM3|V2URM3_9GAMM KVNPGSIILSAEMMLRDMGWVEAADLIIKGISGAIAAKTVTYDFERLMPGATLLRCSEFGQAIIENMGE
tr|A0A433JI60|A0A433JI60_9GAMM KVNPGSIILSAEMMLRHLCWTEAADYILKGVEGAIEAKTVTYDFARLMDGATCVSCSGFADAIIKHM..
tr|A0A562BKR1|A0A562BKR1_9BURK YVNPGSEILSAEMMLRHMGWTEAADLIISSMEKAILSKKVTYDFARLLQGATQVSCSGFGQVMIDNM..
tr|Q46XL6|Q46XL6_CUPPJ YVNPGSEILSAEMMLRHMGWTEAADLIIASMEKSILSKKVTYDFARLLEGATQVSCSGFGQVMIENM..
tr|A0A2N7X381|A0A2N7X381_9BURK YVNPGSEILSAEMMLRHIGWKEAADIIISSMEKSILQKRVTYDFARLMEGATQVSCSGFGQVMIENM..
tr|A0A2G3K0H0|A0A2G3K0H0_9BURK KVNPGSLILSAEMMLRHMGWTEAADLIIKSVEKAIADKQVTYDFARLMDGATEVSCSAFGDAMIARM..
tr|S2LCP0|S2LCP0_HALAF KVNPGSLILSAEMMLRHMGWIEAADLVLRGMEKAIAKGEVTYDFHRVMEKAVLLKCSEFGQAVADNM..
tr|A0A340WR94|A0A340WR94_LIPVE QVNPGSLILSAEMMLRHMGWVEAADLIIKGTNGAISAKTVTYDFERLMDGATLVSSSGFGDALIKHM..
sp|Q9ZH99|IDH_COXBU KVNPGSLILSAEMMLRYLGWKEAADLVVQGIEGAIESKTVTYDFARLMTGAKEVSTSQFGKAIIKHIL.
sp|P41560|IDH1_COLMA KVNPGSVILSAEMMLRHMGWLE.ADLLLKGMSGAIQAKTVTYDFERLMDDATLVSCSAFGDCIIDHM..
tr|G0ACY7|G0ACY7_COLFT YVNPGSLILSAEMMLRHMGWVEAADLIIESMQKSITSKKVTYDFARLMEGATQVSCSGFGEVLIENM..

Sequences used in Icd alignment:

Order	% Sequence identity	UniProt ID	Organism	Length	Entry name	Protein names	Gene names
1	100	P08200	Escherichia coli (strain K12)	416	IDH_ECOLI	Isocitrate dehydrogenase [NADP] (IDH) (EC 1.1.1.42) (IDP) (NADP(+)-specific ICDH) (Oxalosuccinate decarboxylase)	icd icdA icdE b1136 JW1122
2	99.52	A0A077ZII2	Trichuris trichiura (Whipworm) (Trichocephalus trichiurus)	416	A0A077ZII2_TRITR	Isocitrate dehydrogenase [NADP] (EC 1.1.1.42)	TTRE_0000855501
3	96.63	A8AHJ8	Citrobacter koseri (strain ATCC BAA-895 / CDC 4225-83 / SGSC4696)	416	A8AHJ8_CITK8	Isocitrate dehydrogenase [NADP] (EC 1.1.1.42)	CKO_01835
4	96.63	Q8ZPZ0	Salmonella typhimurium (strain LT2 / SGSC1412 / ATCC 700720)	416	Q8ZPZ0_SALTY	Isocitrate dehydrogenase [NADP] (EC 1.1.1.42)	icdA STM1238
5	95.43	E3GBE3	Enterobacter lignolyticus (strain SCF1)	416	E3GBE3_ENTLS	Isocitrate dehydrogenase [NADP] (EC 1.1.1.42)	Entcl_2658
6	95.19	A6T7K6	Klebsiella pneumoniae subsp. pneumoniae (strain ATCC 700721 / MGH 78578)	416	A6T7K6_KLEP7	Isocitrate dehydrogenase [NADP] (EC 1.1.1.42)	icdA KPN_01144
7	93.51	A0A0A2W363	Beauveria bassiana D1-5	416	A0A0A2W363_BEABA	Isocitrate dehydrogenase [NADP] (EC 1.1.1.42)	BBAD15_g1181
8	93.27	A0A4P8YQK7	Jejubacter calystegiae	416	A0A4P8YQK7_9ENTR	Isocitrate dehydrogenase [NADP] (EC 1.1.1.42)	FEM41_15735
9	90.38	Q7N3B7	Photorhabdus laumondii subsp. laumondii (strain DSM 15139 / CIP 105565 / TT01)	417	Q7N3B7_PHOLL	Isocitrate dehydrogenase [NADP] (EC 1.1.1.42)	icd plu2801
10	90.14	A0A0J8YK40	bacteria symbiont BFo1 of Frankliniella occidentalis	416	A0A0J8YK40_9GAMM	Isocitrate dehydrogenase [NADP] (EC 1.1.1.42)	AI28_17055
11	90.14	B4EVG5	Proteus mirabilis (strain HI4320)	417	B4EVG5_PROMH	Isocitrate dehydrogenase [NADP] (EC 1.1.1.42)	icd PMI0891
12	89.9	D3VIB7	Xenorhabdus nematophila (strain ATCC 19061 / DSM 3370 / CCUG 14189 / LMG 1036 / NCIMB 9965 / AN6)	417	D3VIB7_XENNA	Isocitrate dehydrogenase [NADP] (EC 1.1.1.42)	icd XNC1_2703
13	87.5	E0SDF2	Dickeya dadantii (strain 3937) (Erwinia chrysanthemi (strain 3937))	417	E0SDF2_DICD3	Isocitrate dehydrogenase [NADP] (EC 1.1.1.42)	icd Dda3937_02082
14	84.13	A0KI74	Aeromonas hydrophila subsp. hydrophila (strain ATCC 7966 / DSM 30187 / BCRC 13018 / CCUG 14551 / JCM 1027 / KCTC 2358 / NCIMB 9240 / NCTC 8049)	417	A0KI74_AERHH	Isocitrate dehydrogenase [NADP] (EC 1.1.1.42)	icd AHA_1436
15	81.25	Q6LT16	Photobacterium profundum (strain SS9)	417	Q6LT16_PHOPR	Isocitrate dehydrogenase [NADP] (EC 1.1.1.42)	T1682 PBPRA1149
16	78.61	Q9I0L5	Pseudomonas aeruginosa (strain ATCC 15692 / DSM 22644 / CIP 104116 / JCM 14847 / LMG 12228 / 1C / PRS 101 / PAO1)	418	Q9I0L5_PSEAE	Isocitrate dehydrogenase [NADP] (EC 1.1.1.42)	icd PA2623
17	77.29	V2URM3	Acinetobacter brisouii CIP 110357	418	V2URM3_9GAMM	Isocitrate dehydrogenase [NADP] (EC 1.1.1.42)	P255_01775
18	76.2	A0A433JI60	Legionella septentrionalis	421	A0A433JI60_9GAMM	Isocitrate dehydrogenase [NADP] (EC 1.1.1.42)	EKM59_08945
19	76.14	A0A562BKR1	Cupriavidus gilardii J11	416	A0A562BKR1_9BURK	Isocitrate dehydrogenase [NADP] (EC 1.1.1.42)	L602_002300000540
20	75.42	Q46XL6	Cupriavidus pinatubonensis (strain JMP 134 / LMG 1197) (Cupriavidus necator (strain JMP 134))	416	Q46XL6_CUPPJ	Isocitrate dehydrogenase [NADP] (EC 1.1.1.42)	Reut_A2756
21	75.24	A0A2N7X381	Trinickia symbiotica	418	A0A2N7X381_9BURK	Isocitrate dehydrogenase [NADP] (EC 1.1.1.42)	C0Z20_15365
22	75.06	A0A2G3K0H0	Chitinimonas sp. BJB300	415	A0A2G3K0H0_9BURK	Isocitrate dehydrogenase [NADP] (EC 1.1.1.42)	CSQ89_16965
23	74.76	S2LCP0	Halomonas anticariensis (strain DSM 16096 / CECT 5854 / LMG 22089 / FP35)	418	S2LCP0_HALAF	Isocitrate dehydrogenase [NADP] (EC 1.1.1.42)	L861_09285
24	74.52	A0A340WR94	Lipotes vexillifer (Yangtze river dolphin)	418	A0A340WR94_LIPVE	Isocitrate dehydrogenase [NADP] (EC 1.1.1.42)	LOC103084848
25	74.04	Q9ZH99	Coxiella burnetii (strain RSA 493 / Nine Mile phase I)	427	IDH_COXBU	Isocitrate dehydrogenase [NADP] (IDH) (EC 1.1.1.42) (IDP) (NADP(+)-specific ICDH) (Oxalosuccinate decarboxylase)	icd CBU_1200
26	73.98	P41560	Colwellia maris	415	IDH1_COLMA	Isocitrate dehydrogenase [NADP] 1 (EC 1.1.1.42) (IDH-I) (IDP-1) (NADP(+)-specific ICDH 1) (Oxalosuccinate decarboxylase 1)	icdI
27	73.8	G0ACY7	Collimonas fungivorans (strain Ter331)	417	G0ACY7_COLFT	Isocitrate dehydrogenase [NADP] (EC 1.1.1.42)	icdA CFU_3268

KatE-catalase HPII

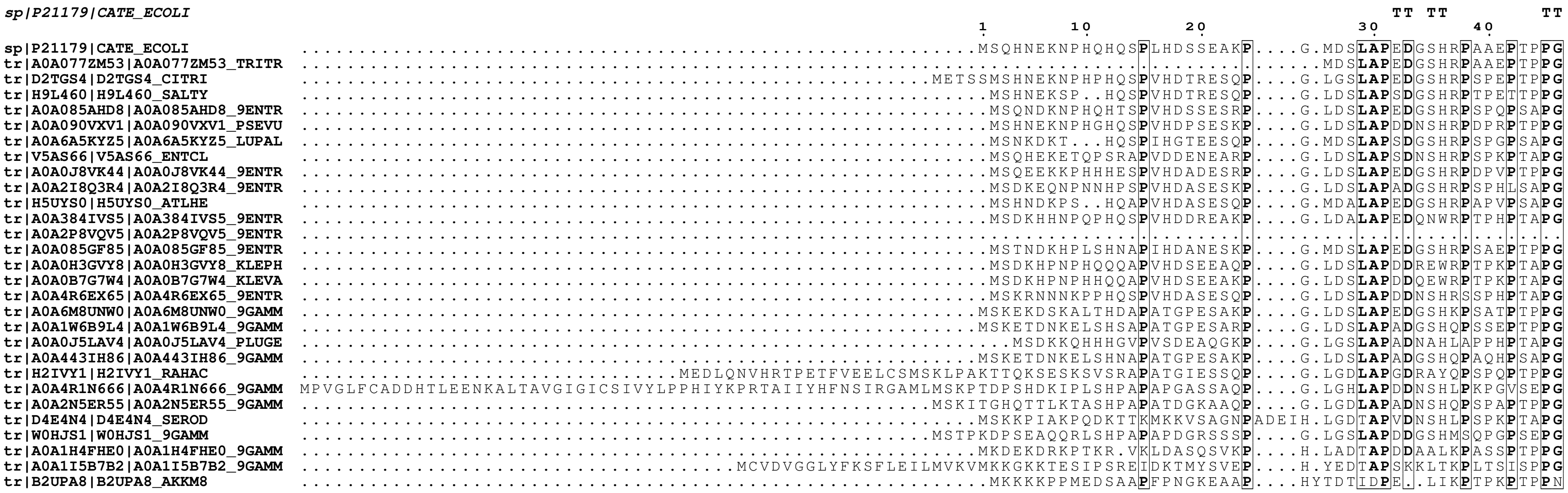

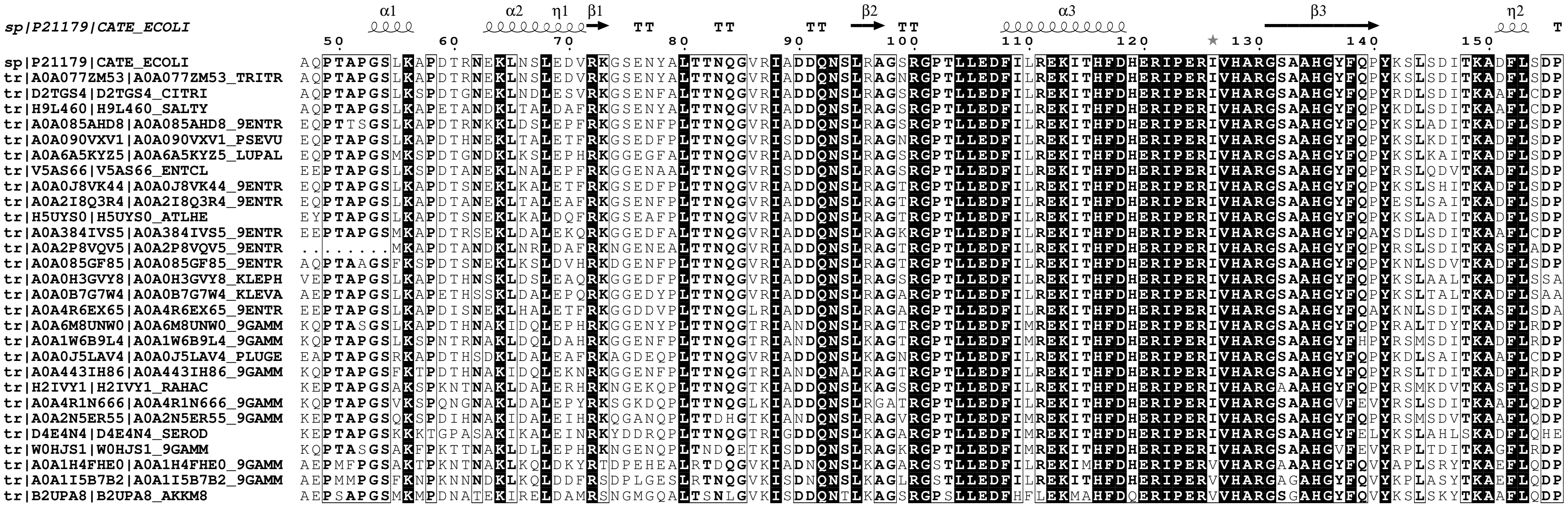

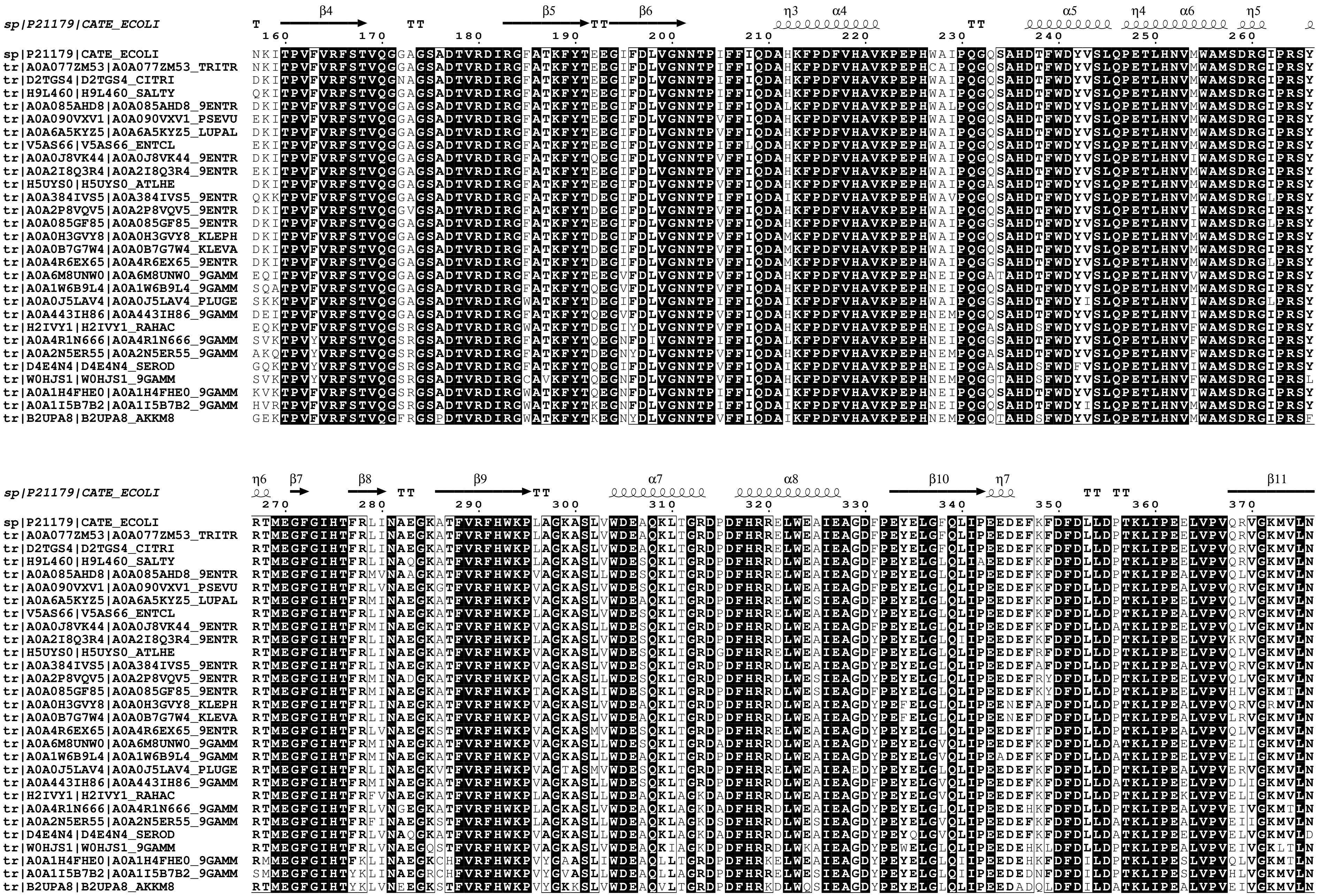
sp|P21179|CATE_ECOLI
β4 β5 β6 η3 α4 α5 η4 α6 η5
T TT TT TT
160 170 180 190 200 210 220 230 240 250 260
sp|P21179|CATE_ECOLI
tr|A0A077ZM53|A0A077ZM53_TRITR
tr|D2TGS4|D2TGS4_CITRI
tr|H9L460|H9L460_SALTY
tr|A0A085AHD8|A0A085AHD8_9ENTR
tr|A0A090VXV1|A0A090VXV1_PSEVU
tr|A0A6A5KYZ5|A0A6A5KYZ5_LUPAL
tr|V5AS66|V5AS66_ENTCL
tr|A0A0J8VK44|A0A0J8VK44_9ENTR
tr|A0A2I8Q3R4|A0A2I8Q3R4_9ENTR
tr|H5UYS0|H5UYS0_ATLHE
tr|A0A384IVS5|A0A384IVS5_9ENTR
tr|A0A2P8VQV5|A0A2P8VQV5_9ENTR
tr|A0A085GF85|A0A085GF85_9ENTR
tr|A0A0H3GVY8|A0A0H3GVY8_KLEPH
tr|A0A0B7G7W4|A0A0B7G7W4_KLEVA
tr|A0A4R6EX65|A0A4R6EX65_9ENTR
tr|A0A6M8UNW0|A0A6M8UNW0_9GAMM
tr|A0A1W6B9L4|A0A1W6B9L4_9GAMM
tr|A0A0J5LAV4|A0A0J5LAV4_PLUGE
tr|A0A443IH86|A0A443IH86_9GAMM
tr|H2IVY1|H2IVY1_RAHAC
tr|A0A4R1N666|A0A4R1N666_9GAMM
tr|A0A2N5ER55|A0A2N5ER55_9GAMM
tr|D4E4N4|D4E4N4_SEROD
tr|W0HJS1|W0HJS1_9GAMM
tr|A0A1H4FHE0|A0A1H4FHE0_9GAMM
tr|A0A1I5B7B2|A0A1I5B7B2_9GAMM
tr|B2UPA8|B2UPA8_AKKM8
sp|P21179|CATE_ECOLI
η6 β7 β8 β9 α7 α8 β10 η7 β11
TT TT TT TT
270 280 290 300 310 320 330 340 350 360 370

```
sp|P21179|CATE_ECOLI
α9  TT  TT β12  α10  η8  η9  TT  β13
380  390  400  410  420  430  440  450  460  470  480

sp|P21179|CATE_ECOLI                 RNPDNFFAENEQAAFHPGHIVPGLDFTNDPLLQGRLFSYTDTQISRLGGPNFHEIPINRPTCPYHNFQRDGMHRMGIDTNPANYEPNSINDNWPRETPPGPKRGGFESYQ
tr|A0A077ZM53|A0A077ZM53_TRITR       RNPDNFFAENEQAAFHPGHIVPGLDFTNDPLLQGRLFSYTDTQISRLGGPNFHEIPINRPTCPYHNFQRDGMHRMGIDTNPANYEPNSINDNWPRETPPGPKRGGFESYQ
tr|D2TGS4|D2TGS4_CITRI               RNPDNFFAENEQAAFHPGHIVPGIDFTNDPLLQGRLFSYTDTQISRLGGPNFHEIPINRPTCPYHNFQRDGMHRMDIDTNPANYEPNSINDNWPRETPPAPKRGGFETYQ
tr|H9L460|H9L460_SALTY               RNPDNFFAENEQAAFHPGHIVPGIDFTNDPLLQGRLFSYTDTQISRLGGPNFHEIPINRPTCPYHNFQRDGMHRMDIDTNPANYEPNSINDNWPRETPPAPKRGGFESYQ
tr|A0A085AHD8|A0A085AHD8_9ENTR       RNPDNFFSENEQAAFHPGHIVPGLDFTNDPLLQGRLFSYTDTQISRLGGPNFHEIPINRPTCPYHNYQRDGMHRMDIETNPANYEPNSINDNWPRETPPGPKRGGFETYQ
tr|A0A090VXV1|A0A090VXV1_PSEVU       RNPDNFFAENEQAAFHPGHIVPGLDFTNDPLLQGRLFSYTDTQISRLGGPNFHEIPINRPTCPYHNFQRDGMHRMDIDTNPANYEPNSINDNWPRETPPGPKRGGFESYQ
tr|A0A6A5KYZ5|A0A6A5KYZ5_LUPAL       RNPDNFFAENEQAAFHPGHIVPGLDFTNDPLLQGRLFSYTDTQISRLGGPNFHEIPINRPTCPYHNFQRDGMHRQDIDTNPANYEPNSINDNWPRETPPGPKRGGFESYQ
tr|V5AS66|V5AS66_ENTCL               RNPDNFFAENEQVAFHPGHIVPGLDFTNDPLLQGRLFSYTDTQISRLGGPNFHEIPINRPTCPYHNFQRDGMHRMDIDTNPANYEPNSINDNWPRETPPGPKRGGFESYQ
tr|A0A0J8VK44|A0A0J8VK44_9ENTR       RNPDNFFAENEQAAFHPGHIVPGLDFTNDPLLQGRLFSYTDTQISRLGGPNFHEIPINRPTCPYHNFQRDGMHRMDIDTNPANYEPNSINDNWPRETPPGPKRGGFESYQ
tr|A0A2I8Q3R4|A0A2I8Q3R4_9ENTR       RNPDNFFAENEQAAFHPGHIVPGLDFSNDPLLQGRLFSYTDTQISRLGGPNFHEIPINRPGCPYHNFQRDGMHRMDIDTNPANYEPNSINDNWPRETPPGPKRGGFESYQ
tr|H5UYS0|H5UYS0_ATLHE               RNPDNFFAENEQAAFHPGHIVPGLDFTNDPLLQGRLFSYTDTQISRLGGPNFHEIPINRPVCPYHNFQRDGMHRMDIDTNPANYEPNSINDNWPRETPPGPKRGGFESYQ
tr|A0A384IVS5|A0A384IVS5_9ENTR       RNPDNFFAENEQAAFHPGHIVPGIDFSNDPLLQGRLFSYTDTQISRLGGPNFHEIPINKPTCPYHNFQRDGMHRMDIDTNPANYEPNSINDNWPRETPPAAKRGGFESYA
tr|A0A2P8VQV5|A0A2P8VQV5_9ENTR       RNPDNFFAENEQAAFHPGHIVPGLDFSNDPLLQGRLFSYTDTQISRLGGPNFHEIPINRPTCPYHNFQRDGMHRMDIDTNPANYEPNSINDNWPRETPPAPQRGGFESYQ
tr|A0A085GF85|A0A085GF85_9ENTR       RNPDNFFAENEQAAFHPGHIVPGLDFSNDPLLQGRLFSYTDTQISRLGGPNFHEIPINRPTCPYHNFQRDGMHRMDIDSNPANYEPNSINDNWPRETPPGPKRGGFESYQ
tr|A0A0H3GVY8|A0A0H3GVY8_KLEPH       RNPDNFFAENEQAAFHPGHIVPGIDFSNDPLLQGRLFSYTDTQISRLGGPNFHEIPINRPTCPYHNFQRDGMHRMDIDTNPANYEPNSINDNWPRETPPAAKRGGFESLA
tr|A0A0B7G7W4|A0A0B7G7W4_KLEVA       RNPDNFFAENEQAAFHPGHIVPGIDFSNDPLLQGRLFSYTDTQISRLGGPNFHEIPINRPTCPYHNFQRDGMHRMDIDTNPANYEPNSINDNWPRETPPAAKRGGFESLA
tr|A0A4R6EX65|A0A4R6EX65_9ENTR       RNPDNFFAENEQVAFHPGHIVPGIDFSNDPLLQGRLFSYTDTQISRLGGPNFHEIPINRPACPYHNFQRDGMHRMDIDTNPANYEPNSINDNWPRETPPGPHRGGFESHH
tr|A0A6M8UNW0|A0A6M8UNW0_9GAMM       RNPDNFFAETEQVAFHPGHIVPGLDFSNDPLLQGRLFSYTDTQISRLGGPNFHEIPINRPVCPYHNFQRQGMHRMDIDTNPANYEPNSINDNWPRETPAGPHRGGFESYQ
tr|A0A1W6B9L4|A0A1W6B9L4_9GAMM       RNPDNFFAETEQVAFHPGHIVPGLDFTNDPLLQGRLFSYTDTQISRLGGPNFHEIPINRPVCPYHNFQRQGMHRMDIDTNPANYEPNSINDNWPRETPPQPRRGGFESYQ
tr|A0A0J5LAV4|A0A0J5LAV4_PLUGE       RNPDNFFAENEQAAFHPGHIVPGIDFSDDPLLQGRLFSYTDTQISRLGGPNFHEIPINRPTCPYHNLQRDGMHRMDIDTNPANYEPNSINDNWPRETPPAAKGGGFESRR
tr|A0A443IH86|A0A443IH86_9GAMM       RNPDNFFAETEQVAFHPGHIVPGLDFSNDPLLQGRLFSYTDTQISRLGGPNFHEIPINRPVCPYHNFQRGGMHRQDIDTNPANYEPNSINDNWPRETPPGPHRGGFESYQ
tr|H2IVY1|H2IVY1_RAHAC               RNPDNFFAETEQAAFHPGHIVPGIDFTNDPLLQGRLFSYTDTQISRLGGPNFHEIPINRPTCPFHNFQRDGMHRQDIDTNPANYEPNSLNNNWPRETPPAASGGGFESYP
tr|A0A4R1N666|A0A4R1N666_9GAMM       RNPDNFFAETEQVAFHPGHIVPGIDFTNDPLLQGRLFSYTDTQISRLGGPNFHEIPINRPVCPYHNFQRDGMHRQDIDTNPAAHEPNSINDNWPRETAPAAQNGGFETYQ
tr|A0A2N5ER55|A0A2N5ER55_9GAMM       RNPDNFHAETEQVAFHPGHVVPGIDFTNDPLLQGRLFSYTDTQISRLGGPNFHEIPINRPVCPYHNFQRDGMHRMDIDTNPASYEPNSINNNWPRETPPAASGGGFESHY
tr|D4E4N4|D4E4N4_SEROD               RNPDNFFAETEQVAFHPGHIVPGIDFTNDPLLQGRLFSYTDTQISRLGGPNFHEIPINRPTCPYHNLQRDGMHRMDIDTNPANYSPNSINNNWPRETPPSASGGGFASYA
tr|W0HJS1|W0HJS1_9GAMM               RNPDNFFAETEQVAFHSGHIVPCIDFSNDPLLQGRLFSYTDTQISRLGGPNFHEIPINRPVCPYHNFQRDGMHRQDINTNPAAYEPNSLNDNWPRETAPAAENGGFENYH
tr|A0A1H4FHE0|A0A1H4FHE0_9GAMM       RNPDNYFSETEQVAFCPGNIVPGIDFSDDPLLQGRLFSYLDTQISRLGGPNFHELPINKPVCPFHNHQRDGMHRMEI.SGAANYEPNSINNNWPRETAPAQANGGFSTYP
tr|A0A1I5B7B2|A0A1I5B7B2_9GAMM       RNPDSYFNETEQVAFCPGNIVSGIDFSDDPLLQGRLFSYIDTQISRLGGANFHEIPINRPVCPFHNHQRDGMHRISI.SGAANYEPNSINNNWPRETPPA..GGVFSSYP
tr|B2UPA8|B2UPA8_AKKM8               RNPDNFFAETEQVAFCPANIVPGIDFSDDPLLQGRIFSYSDTQRHRLGGANFTEIPINRPICPFHNNQRDGFHRMQIDASPANYDPNSIGNNWPRETPP..EKGGFTTSP

sp|P21179|CATE_ECOLI
β14  η10  α11  α12  α13  α14  α15  TT  η11
490  500  510  520  530  540  550  560  570  580  590

sp|P21179|CATE_ECOLI                 ERVEGNKVRERSPSFGEYYSHPRLFWLSQTPFEQRHIVDGFSFELSKVVRPYIRERVVDQLAHIDLTLAQAVAKNLGIELTDDQLNITPPPDVNGLKKDPSLSLYAIP.D
tr|A0A077ZM53|A0A077ZM53_TRITR       ERVEGNKVRERSPSFGEYYSHPRLFWLSQTPFEQRHIVDGFSFELSKVVRPYIRERVVDQLAHIDLTLAQAVAKNLGIELTDDQLNITPPPDVNGLKKDPSLSLYAIP.D
tr|D2TGS4|D2TGS4_CITRI               ERVDGNKIRERSPSFGEYYAHPRLFWLSQTPVEQLHIVDAFSFELSKVVRTYIRERVVDQLAHIDVNLAQAVGKNLGIELTPEQTQIAPPPDVNGLKKDPSLSLYAIP.D
tr|H9L460|H9L460_SALTY               ERVDGNKIRERSPSFGEYYSHPRLFWLSQTPIEQQHIIDAFSFELGKVARAYIRERVVDQLAHIDVTLAQGVAHNLGFALTHEQTQIAPPPDVNGLKKDPALSLYAVP.D
tr|A0A085AHD8|A0A085AHD8_9ENTR       ERVDGHKIRERSPSFGEYYSHPRLFWNSQTPQEQEHIVGGFSFELSKVGRPYIRERVVDQLAHIDLTLAQAVADNLGITLTDEQQRIMPPPDVSGVNKDPTLSLYAIP.G
tr|A0A090VXV1|A0A090VXV1_PSEVU       ERIEGNKIRERSPSFGEYYAHPRLFWQSQTPHEQQHIIDAFSFELSKVVREYIRERVVDQLACVDTMLALSVAKNLGISLTDEQLNVTPPKEVNGIASDPSLSLYAQP.D
tr|A0A6A5KYZ5|A0A6A5KYZ5_LUPAL       ERIDGNKIRERSPSFGEYYAHPRLFWNSQTPIEQQHIIGGFSFELSKVVRTYIRERVVDQLAHIDVQLAQSVADNLGITLTDEQRNLAPPKEVNGVKKDPSLSLYAVP.G
tr|V5AS66|V5AS66_ENTCL               ERVEGHKVRERSPSFGEYYAHPRLFWLSQTPFEQRHIVDAFSFELSKVARPYIRERVVDLLAHIDVNLAQSVAENLGITLTEEQCNITPPPAVNGVEKDPSLSLYAIP.D
tr|A0A0J8VK44|A0A0J8VK44_9ENTR       ERIEGYKVRERSPSFGEYYAHPRLFWQSQTPVEQRHIIDAFSFELSKVVRPYIRERVVDQLCRIDISLAQPVAENLGIELTDEQMHIAPPKDVNGLKKDASLSLYAVP.G
tr|A0A2I8Q3R4|A0A2I8Q3R4_9ENTR       ERVEGGKIRERSPSFAEYYAHPRLFWLSQTPFEQQHIIDAFSFELSKVVREYIRERVVDQLAHIDVHLANAVAGNLGITLSEAQRNVAPPKDVNGLKKDPSLSLYAIP.E
tr|H5UYS0|H5UYS0_ATLHE               ERIEGHKIRERSPSFGEYYSHPRLFWLSQTPVEQQHIIDAFSFELGKVARAYIRERVVDQLCHIDVSLASAVAENLGITLTDEQVHTAPPKDVNGLKKDASLSLYAVP.G
tr|A0A384IVS5|A0A384IVS5_9ENTR       ERVDGEKIRQRSPSFGEYYSQPLLFWRSQTPIEQQHIIDGFSFELSKVVREWIRERVVDQLAHIDLQLAQAVGKNLGIELTDEQRSITPPPDVNGLKKDPTLSLYAIP.S
tr|A0A2P8VQV5|A0A2P8VQV5_9ENTR       TRLEGHKIRERSPSFGEYYSHPRLFWLSQTPVEQQHIIDAFSFELSKVVRSYIRERVVDHLARIDISLAHPVATNLGITLTDEQMHLAPPKDVNGLKKDASLSLYAVP.G
tr|A0A085GF85|A0A085GF85_9ENTR       TRVEGHKIRERSPSFGEYYSHPRLFWQSQTPVEQQHIISAFSFELSKVARPYIRERVLDHLTRIDISLAQPVAENLGIELSHEQMHVAPPKDVNGLKKDPSLSLYAVP.S
tr|A0A0H3GVY8|A0A0H3GVY8_KLEPH       ERVDGEKIRQRSPSFGEYYAQPRLFWLSQTPIEQQHIIDGFSFELSKVVRTWIRERVVDHLAHIDTKLAEAVGANLGIELSDDQRNITLPAPVNGVEKDPSLSLYADA.E
tr|A0A0B7G7W4|A0A0B7G7W4_KLEVA       ERVDGEKIRQRSPSFGEYYAQPRLFWLSQTPIEQQHIIDGFSFELSKVVRTWIRERVVDHLAHIDTKLAEAVGANLGIELSDDQCNITLPSPVNGVEKDPSLSLYADA.E
tr|A0A4R6EX65|A0A4R6EX65_9ENTR       ARVDGDKIRERSPSFGEYYAHPRLFWNSQTAVEQQHIVSAFSFELSKVARAYIRERVVDQLVHIDHQLASAVADNLGITLTEEQRNIAPPAAINGISKDPTLSLYAIP.S
tr|A0A6M8UNW0|A0A6M8UNW0_9GAMM       ERIEGHKVRERSPSFGEYYSQPRLFWLSQTPIEQQHIVDAFSFELSKVSRPYIRERVLDHLLHIDVSLAHGVAENLGIELSDEQLHTAPPKDVNGLKKDASLSLYAVP.S
tr|A0A1W6B9L4|A0A1W6B9L4_9GAMM       ERIEGHKVRERSPSFGEYYSQPRLFWLSQTPIEQQHIIEAFSFELGKVARAYIRERVVDHLLHIDVSLAHGVAQNLGIALSDEKLHTAPPKDVNGLKKDASLSLYAIS.N
tr|A0A0J5LAV4|A0A0J5LAV4_PLUGE       ERVDAETIRQRSPSFGEYYAHPRLFWLSQTPVEQQHIIDAFSFELSKVARAYIRERVVDRLTHIDLQLAQGVADNLGITLSDAQRNVAPPPPVNGLSKDPTLSLYALP.G
tr|A0A443IH86|A0A443IH86_9GAMM       ERIEGHKVRDRSPSFGEYYSQPRLFWHSQTPIEQQHIIDAFSFELGKLSRPYIRERVVDHLVRIDISLAHAVAENLGIALSDEKLHTAPPKDVNGLKKDASLSLYAVP.S
tr|H2IVY1|H2IVY1_RAHAC               ERVDAEKIRQRSPSFAEYYAHPRLFWLSQTPAEQDHIVGAFSFELSKVARPYIRERVVDLLAHVDQTLAQKVAKNLGITLSAEILKIKPPKPVNGLKNDPSLSLYASG.K
tr|A0A4R1N666|A0A4R1N666_9GAMM       ERIDGHKIRERSDSFLEYYSHPRLFWLSQTPAEQQHIVDAFSFELGKVARPYIRERVVDLLTRVDVQLAKRVAAKLGLALSADALKTAPPKPVNGISQDPTLSLYASG.S
tr|A0A2N5ER55|A0A2N5ER55_9GAMM       ERIDGHKVRERSESFADYYSQPRLFWMSQTPVEQEHIIGAFSFELSKVGRTYIRERVVDLLARVDINLAEEVGKNLGFRLPDDAKKYPLPHSVNGLQKDETLSLYATG.D
tr|D4E4N4|D4E4N4_SEROD               ERIDAHKIRERSESFQEYYAQPRLFWLSQTPVEQQHIIDAFSFELGKVARAYIRQRVVEKLAHIDTTLAKKVAFNLGITLSASVLKTPAPKPVNGLQKDPALSLYAMP.G
tr|W0HJS1|W0HJS1_9GAMM               ERIDGEKIRQRRDSFNEYFAHPRLFWLNQTPAEQQHIIDAFSFELGKVARPYIRERVIDLLTRVDVDLASQVAARLGYRLSDEAKNIAPPPAVNGITRDPTLSLYATG.S
tr|A0A1H4FHE0|A0A1H4FHE0_9GAMM       QAPDGEKIRQRSESFADYYSQPRLFWLSQTDAEKKHIVGAYSFELGKVARPWIRERVVDQLTYIDHDLAEKVAENLGMKLTAEQRQHPLPGAINGITTDATLSLYAADKK
tr|A0A1I5B7B2|A0A1I5B7B2_9GAMM       QLTTGEKIRKRSGTFIDYYSHPRLFWLSQTKVEQNHIIGGFSFELGKVVRPWIRERVVDQLTYIDHNLAQAVAENLGIELSHEQLRHPLPGPVNGISKDRSLSIYDGH.S
tr|B2UPA8|B2UPA8_AKKM8               QTVSGVKERLRNPSFAEYYSHPRLFWMSQTPVEQEHIINAFSFELGKVTRPYIRERVVDLLTRIDPDLASGVARNLGIQLTREQLSRELPRPVCGLEQDPSLSLYAHT.D
```

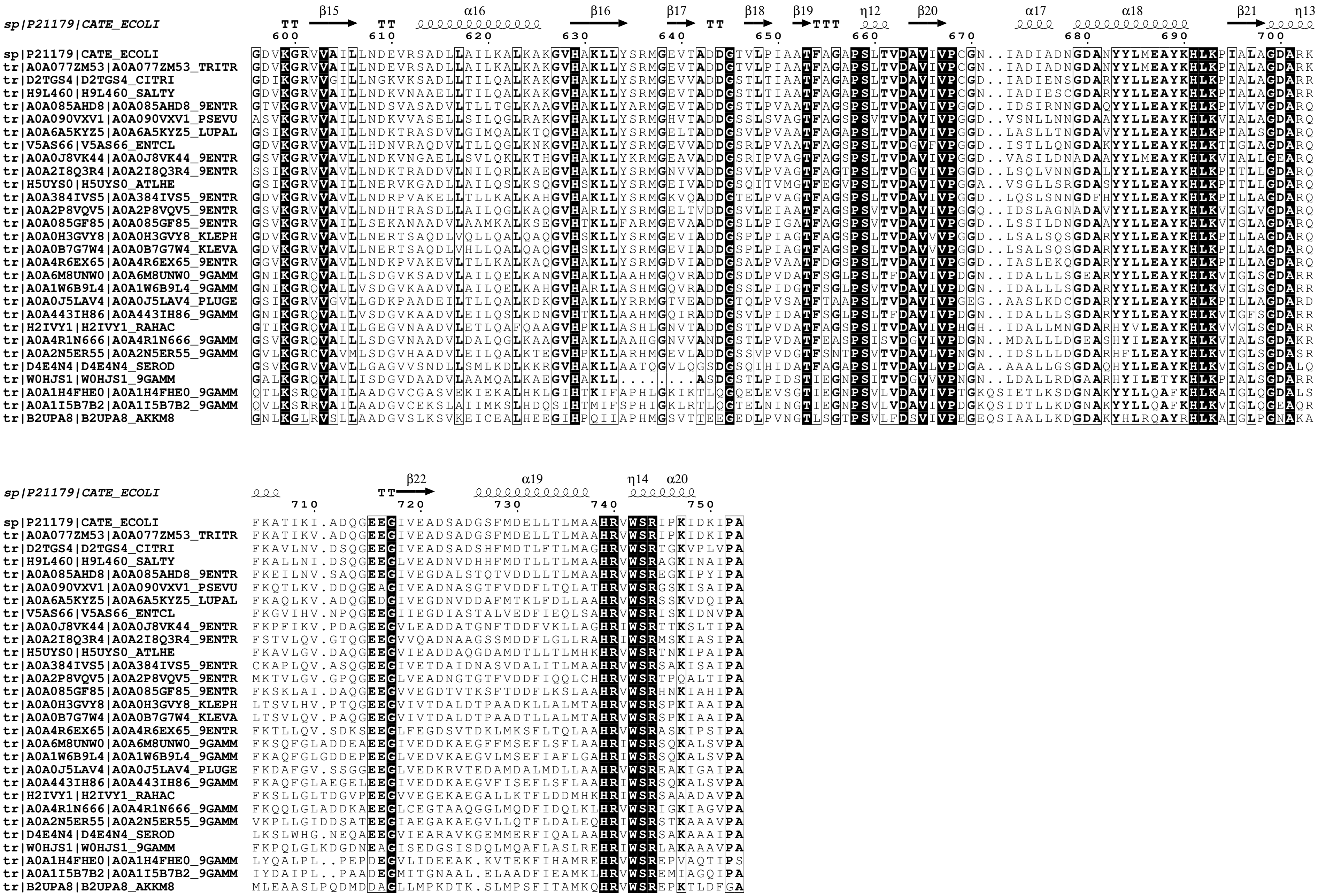
sp|P21179|CATE_ECOLI
β15 α16 β16 β17 β18 β19 η12 β20 α17 α18 β21 η13
600 610 620 630 640 650 660 670 680 690 700
sp|P21179|CATE_ECOLI
tr|A0A077ZM53|A0A077ZM53_TRITR
tr|D2TGS4|D2TGS4_CITRI
tr|H9L460|H9L460_SALTY
tr|A0A085AHD8|A0A085AHD8_9ENTR
tr|A0A090VXV1|A0A090VXV1_PSEVU
tr|A0A6A5KYZ5|A0A6A5KYZ5_LUPAL
tr|V5AS66|V5AS66_ENTCL
tr|A0A0J8VK44|A0A0J8VK44_9ENTR
tr|A0A2I8Q3R4|A0A2I8Q3R4_9ENTR
tr|H5UYS0|H5UYS0_ATLHE
tr|A0A384IVS5|A0A384IVS5_9ENTR
tr|A0A2P8VQV5|A0A2P8VQV5_9ENTR
tr|A0A085GF85|A0A085GF85_9ENTR
tr|A0A0H3GVY8|A0A0H3GVY8_KLEPH
tr|A0A0B7G7W4|A0A0B7G7W4_KLEVA
tr|A0A4R6EX65|A0A4R6EX65_9ENTR
tr|A0A6M8UNW0|A0A6M8UNW0_9GAMM
tr|A0A1W6B9L4|A0A1W6B9L4_9GAMM
tr|A0A0J5LAV4|A0A0J5LAV4_PLUGE
tr|A0A443IH86|A0A443IH86_9GAMM
tr|H2IVY1|H2IVY1_RAHAC
tr|A0A4R1N666|A0A4R1N666_9GAMM
tr|A0A2N5ER55|A0A2N5ER55_9GAMM
tr|D4E4N4|D4E4N4_SEROD
tr|W0HJS1|W0HJS1_9GAMM
tr|A0A1H4FHE0|A0A1H4FHE0_9GAMM
tr|A0A1I5B7B2|A0A1I5B7B2_9GAMM
tr|B2UPA8|B2UPA8_AKKM8
sp|P21179|CATE_ECOLI
β22 α19 η14 α20
710 720 730 740 750

Sequences used in KatE alignment:

Order	% Sequence Identity	UniProt ID	Organism	Length	Entry name	Protein names	Gene names
1	100	P21179	Escherichia coli (strain K12)	753	CATE_ECOLI	Catalase HPII (EC 1.11.1.6) (Hydroxyperoxidase II)	katE b1732 JW1721
2	99.59	A0A077ZM53	Trichuris trichiura (Whipworm) (Trichocephalus trichiurus)	728	A0A077ZM53_TRITR	Catalase (EC 1.11.1.6)	TTRE_0000922701
3	89.64	D2TGS4	Citrobacter rodentium (strain ICC168) (Citrobacter freundii biotype 4280)	757	D2TGS4_CITRI	Catalase (EC 1.11.1.6)	katE ROD_13241
4	88.8	H9L460	Salmonella typhimurium (strain LT2 / SGSC1412 / ATCC 700720)	750	H9L460_SALTY	Catalase (EC 1.11.1.6)	katE STM1318
5	85.77	A0A085AHD8	Trabulsiella guamensis ATCC 49490	752	A0A085AHD8_9ENTR	Catalase (EC 1.11.1.6)	katE GTGU_00780
6	85.77	A0A090VXV1	Pseudescherichia vulneris NBRC 102420	752	A0A090VXV1_PSEVU	Catalase (EC 1.11.1.6)	katE EV102420_26_00720
7	85.58	A0A6A5KYZ5	Lupinus albus (White lupine) (Lupinus termis)	749	A0A6A5KYZ5_LUPAL	Catalase (EC 1.11.1.6)	Lal_00009202
8	84.71	V5AS66	Enterobacter cloacae S611	752	V5AS66_ENTCL	Catalase (EC 1.11.1.6)	katE EDP2_456
9	84.57	A0A0J8VK44	Franconibacter pulveris	752	A0A0J8VK44_9ENTR	Catalase (EC 1.11.1.6)	katE ACH50_14065
10	84.31	A0A2I8Q3R4	Enterobacteriaceae bacterium ENNIH1	752	A0A2I8Q3R4_9ENTR	Catalase (EC 1.11.1.6)	C2U51_07780
11	84	H5UYS0	Atlantibacter hermannii NBRC 105704	750	H5UYS0_ATLHE	Catalase (EC 1.11.1.6)	katE EH105704_01_00790
12	83.51	A0A384IVS5	Klebsiella michiganensis	752	A0A384IVS5_9ENTR	Catalase (EC 1.11.1.6)	katE SAMEA2273883_03549
13	83.26	A0A2P8VQV5	Siccibacter turicensis	699	A0A2P8VQV5_9ENTR	Catalase (EC 1.11.1.6)	C7G83_03930
14	82.71	A0A085GF85	Buttiauxella agrestis ATCC 33320	752	A0A085GF85_9ENTR	Catalase (EC 1.11.1.6)	katE GBAG_1566
15	81.25	A0A0H3GVY8	Klebsiella pneumoniae subsp. pneumoniae (strain HS11286)	752	A0A0H3GVY8_KLEPH	Catalase (EC 1.11.1.6)	KPHS_21360
16	80.98	A0A0B7G7W4	Klebsiella variicola	752	A0A0B7G7W4_KLEVA	Catalase (EC 1.11.1.6)	katE KVR801_220087
17	80.19	A0A4R6EX65	Scandinavium goeteborgense	752	A0A4R6EX65_9ENTR	Catalase (EC 1.11.1.6)	EC847_101320
18	79.28	A0A6M8UNW0	Erwiniaceae bacterium PD-1	754	A0A6M8UNW0_9GAMM	Catalase (EC 1.11.1.6)	PMPD1_2045
19	78.09	A0A1W6B9L4	Pantoea alhagi	754	A0A1W6B9L4_9GAMM	Catalase (EC 1.11.1.6)	B1H58_18360
20	77.87	A0A0J5LAV4	Pluralibacter gergoviae (Enterobacter gergoviae)	750	A0A0J5LAV4_PLUGE	Catalase (EC 1.11.1.6)	katE ABW06_03560
21	77.82	A0A443IH86	[Pantoea] beijingensis	754	A0A443IH86_9GAMM	Catalase (EC 1.11.1.6)	katE ED28_00425
22	73.44	H2IVY1	Rahnella aquatilis (strain ATCC 33071 / DSM 4594 / JCM 1683 / NBRC 105701 / NCIMB 13365 / CIP 78.65)	780	H2IVY1_RAHAC	Catalase (EC 1.11.1.6)	Rahaq2_4283
23	71.85	A0A4R1N666	Sodalis sp. 159R	813	A0A4R1N666_9GAMM	Catalase (EC 1.11.1.6)	EZJ58_0735
24	71.05	A0A2N5ER55	Chimaeribacter arupi	758	A0A2N5ER55_9GAMM	Catalase (EC 1.11.1.6)	CYR34_04990
25	70.78	D4E4N4	Serratia odorifera DSM 4582	757	D4E4N4_SEROD	Catalase (EC 1.11.1.6)	katE HMPREF0758_3134
26	68.46	W0HJS1	Candidatus Sodalis pierantonius str. SOPE	750	W0HJS1_9GAMM	Catalase (EC 1.11.1.6)	katE SOPEG_2280
27	64.35	A0A1H4FHE0	Lonsdalea quercina	752	A0A1H4FHE0_9GAMM	Catalase (EC 1.11.1.6)	SAMN02982996_03062
28	63.37	A0A1I5B7B2	Izhakiella capsodis	771	A0A1I5B7B2_9GAMM	Catalase (EC 1.11.1.6)	SAMN05216516_1153
29	62.92	B2UPA8	Akkermansia muciniphila (strain ATCC BAA-835 / DSM 22959 / JCM 33894 / BCRC 81048 / CCUG 64013 / CIP 107961 / Muc)	751	B2UPA8_AKKM8	Catalase (EC 1.11.1.6)	Amuc_2070

Fmt-methionyl-tRNA formyltransferase

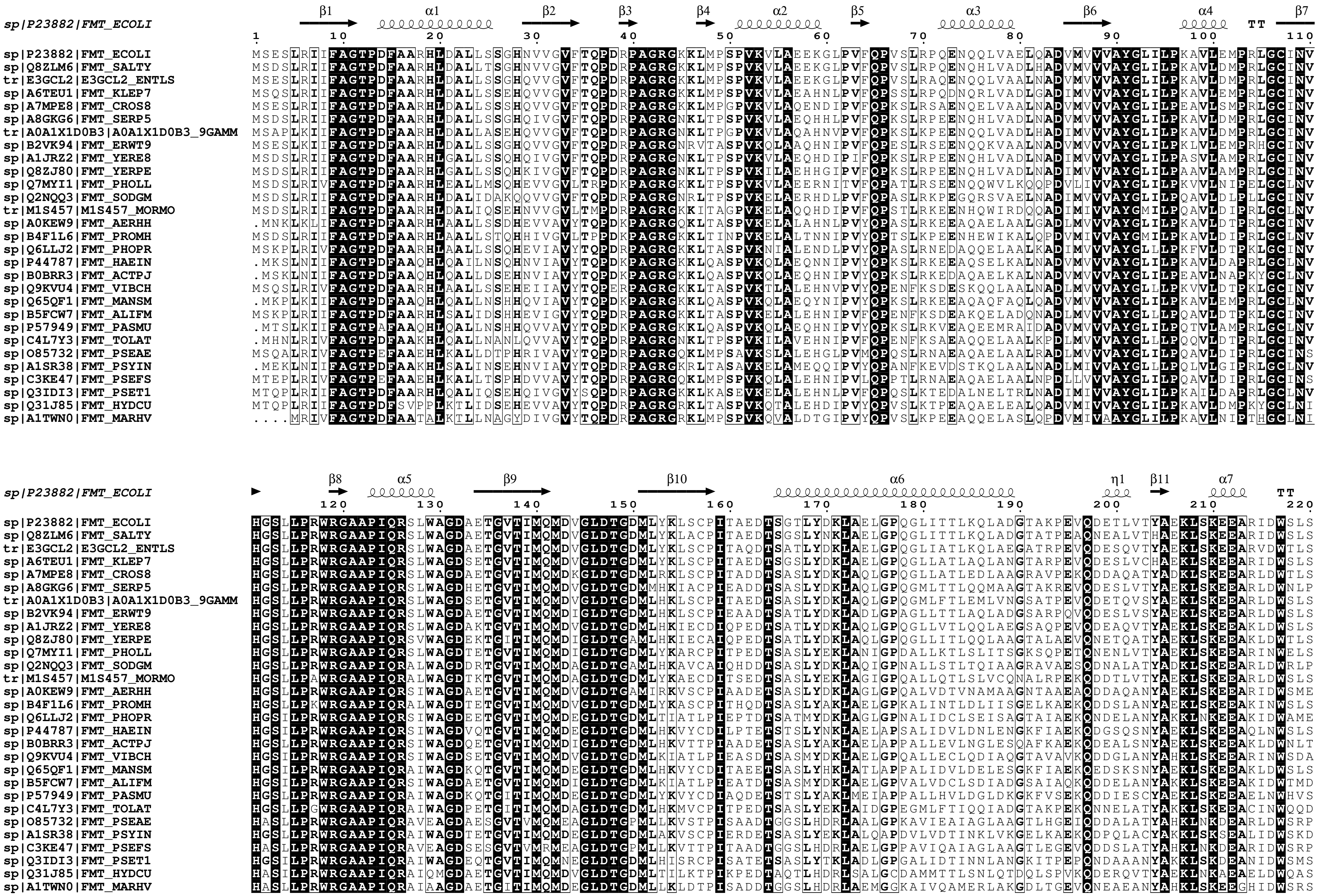

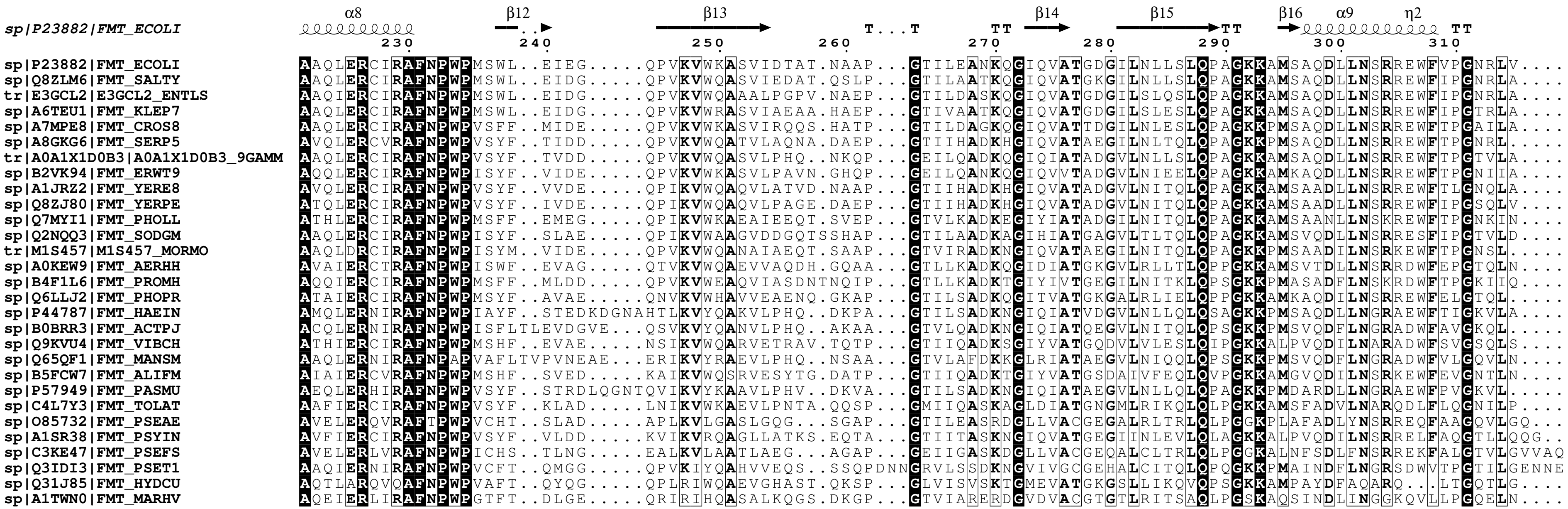
sp|P23882|FMT_ECOLI
α8
β12
β13
T...T
TT
β14
β15
TT
β16
α9
η2
TT
230
240
250
260
270
280
290
300
310
sp|P23882|FMT_ECOLI AAQLERCIRAFNPWPMSWL..EIEG.....QPVKVWKASVIDTAT.NAAP...GTILEANKQGIQVATGDGILNLLSLQPAGKKAMSAQDLLNSRREWFVPGNRLV....
sp|Q8ZLM6|FMT_SALTY AAQLERCIRAFNPWPMSWL..EIDG.....QPVKVWQASVIEDAT.QSLP...GTILAATKQGIQVATGKGILNLLSLQPAGKKAMSAQDLLNSRREWFIPGNRLA....
tr|E3GCL2|E3GCL2_ENTLS AAQIERCIRAFNPWPMSWL..EIDG.....QPVKVWQAAALPGPV.NAEP...GTILDASKQGIQVATGDGILSLQSLQPAGKKAMSAQDLLNSRREWFIPGNRLA....
sp|A6TEU1|FMT_KLEP7 AAQLERCIRAFNPWPMSWL..EIDG.....QPVKVWRASVIAEAA.HAEP...GTIVAATKQGIQVATGDGILSLESLQPAGKKAMSAQDLLNSRREWFIPGTRLA....
sp|A7MPE8|FMT_CROS8 AAQLERCIRAFNPWPVSFF..MIDE.....QPVKVWKASVIRQQS.HATP...GTILDAGKQGIQVATTDGILNLESLQPAGKKPMSAQDLLNSRREWFTPGAILA....
sp|A8GKG6|FMT_SERP5 AVQLERCVRAFNPWPVSYF..TIDD.....QPVKVWQATVLAQNA.DAEP...GTIIHADKHGIQVATAEGILNLTQLQPAGKKPMSAQDLLNSRREWFTPGNRL.....
tr|A0A1X1D0B3|A0A1X1D0B3_9GAMM AAQLERCIRAFNPWPVSYF..TVDD.....QPVKVWQASVLPHQ..NKQP...GEILQADKQGIQIATADGVLNLLSLQPAGKKAMSAQDLLNSRREWFTPGTVLA....
sp|B2VK94|FMT_ERWT9 AQQLERCIRAFNPWPISYF..VIDE.....QPVKVWKASVLPAVN.GHQP...GEILQANKQGIQVVTADGVLNIEELQPAGKKAMKAQDLLNSRREWFTPGNIIA....
sp|A1JRZ2|FMT_YERE8 AVQLERCIRAFNPWPVSYF..VVDE.....QPIKVWQAQVLATVD.NAAP...GTIIHADKHGIQVATADGVLNITQLQPAGKKAMSAADLLNSRREWFTLGNQLA....
sp|Q8ZJ80|FMT_YERPE ATQLERCIRAFNPWPVSYF..IVDE.....QPIKVWQAQVLPAGE.DAEP...GTIIHADKHGIQVATADGVLNITQLQPAGKKAMSAADLLNSRREWFIPGSQLV....
sp|Q7MYI1|FMT_PHOLL ATHLERCIRAFNPWPMSFF..EMEG.....QPIKVWKAEAIEEQT.SVEP...GTVLKADKEGIYIATADGILNITQLQPAGKKAMSAANLLNSKREWFTPGNKIN....
sp|Q2NQQ3|FMT_SODGM AAQLERCIRAFNPWPISYF..SLAE.....QPIKVWAAGVDDGQTSSHAP...GTILAADKAGIHIATGAGVLTLTQLQPAGKKAMSVQDLLNSRRESFIPGTVLD....
tr|M1S457|M1S457_MORMO AAQLDRCIRAFNPWPISYM..VIDE.....QPVKVWQANAIAEQT.SAAP...GTVIRADKNGIQVATAEGILNITQLQPAGKKPMSAADILNSRKEWFTPGNSL.....
sp|A0KEW9|FMT_AERHH AVAIERCTRAFNPWPISWF..EVAG.....QTVKVWQAEVVAQDH.GQAA...GTLLKADKQGIDIATGKGVLRLLTLQPPGKKAMSVTDLLNSRRDWFEPGTQLN....
sp|B4F1L6|FMT_PROMH AQQIERCIRAFNPWPMSFF..MLDD.....QPVKVWEAQVIASDNTNQIP...GTLLKADKTGIYIVTGEGILNITKLQPSGKKPMASADFLNSKRDWFTPGKIIQ....
sp|Q6LLJ2|FMT_PHOPR ATAIERCIRAFNPWPMSYF..AVAE.....QNVKVWHAVVEAENQ.GKAP...GTILSADKQGITVATGKGALRLIELQPPGKKAMKAQDILNSRREWFELGTQL.....
sp|P44787|FMT_HAEIN AMQLERNIRAFNPWPIAYF..STEDKDGNAHTLKVYQAKVLPHQ..DKPA...GTILSADKNGIQIATVDGVLNLLQLQSAGKKPMSAQDLLNGRAEWFTIGKVLA....
sp|B0BRR3|FMT_ACTPJ ACQLERNIRAFNPWPISFLTLEVDGVE...QSVKVYQANVLPHQ..AKAA...GTVLQADKNGIQIATQEGVLNITQLQPSGKKPMSVQDFLNGRADWFAVGKQL.....
sp|Q9KVU4|FMT_VIBCH ATHIERCIRAFNPWPMSHF..EVAE.....NSIKVWQARVETRAV.TQTP...GTIIQADKSGIYVATGQDVLVLESLQIPGKKALPVQDILNARADWFSVGSQLS....
sp|Q65QF1|FMT_MANSM AAQLERNIRAFNPAPVAFLTVPVNEAE...ERIKVYRAEVLPHQ..NSAA...GTVLAFDKKGLRIATAEGVLNIQQLQPSGKKPMSVQDFLNGRADWFVLGQVLN....
sp|B5FCW7|FMT_ALIFM AIAIERCVRAFNPWPMSHF..SVED.....KAIKVWQSRVESYTG.DATP...GTIIQADKTGIYVATGSDAIVFEQLQVPGKKAMGVQDILNSRKEWFEVGNTLN....
sp|P57949|FMT_PASMU AEQLERHIRAFNPWPVSYF..STRDLQGNTQVIKVYKAAVLPHV..DKVA...GTILSADKNGIQIATAEGVLNLLQLQPAGKKPMDARDLLNGRAEWFPVGKVL.....
sp|C4L7Y3|FMT_TOLAT AAFIERCIRAFNPWPVSYF..KLAD.....LNIKVWKAEVLPNTA.QQSP...GMIIQASKAGLDIATGNGMLRIKQLQLPGKKAMSFADVLNARQDLFLQGNILP....
sp|O85732|FMT_PSEAE AVELERQVRAFTPWPVCHT..SLAD.....APLKVLGASLGQG...SGAP...GTILEASRDGLLVACGEGALRLTRLQLPGGKPLAFADLYNSRREQFAAGQVLGQ...
sp|A1SR38|FMT_PSYIN AVFIERCIRAFNPWPVSYF..VLDD.....KVIKVRQAGLLATKS.EQTA...GTIITASKNGIQVATGEGIINLEVLQLAGKKALPVQDILNSRRELFAQGTLLQQG..
sp|C3KE47|FMT_PSEFS AVELERLVRAFNPWPICHS..TLNG.....EALKVLAATLAEG...AGAP...GEIIGASKDGLLVACGEQALCLTRLQLPGGKALNFSDLFNSRREKFALGTVLGVVAQ
sp|Q3IDI3|FMT_PSET1 AAQIERNIRAFNPWPVCFT..QMGG.....QPVKIYQAHVVEQS..SSQPDNNGRVLSSDKNGVIVGCGEHALCITQLQPQGKKPMAINDFLNGRSDWVTPGTILGENNE
sp|Q31J85|FMT_HYDCU AQTLARQVQAFNPWPVAFT..QYQG.....QPLRIWQAEVGHAST.QKSP...GLVISVSKTGMEVATGKGSLLIKQVQPSGKKAMPAYDFAQARQ...LTGQTLG....
sp|A1TWN0|FMT_MARHV AQEIERLIRAFNPWPGTFT..DLGE.....QRIRIHQASALKQGS.DKGP...GTVIARERDGVDVACGTGTLRITSAQLPGSKAQSINDLINGGKQVLLPGQELN....

Sequences used in Fmt alignment:

Order	% Sequence Identity	UniProt ID	Organism	Length	Entry name	Protein names	Gene names
1	100	P23882	Escherichia coli (strain K12)	315	FMT_ECOLI	Methionyl-tRNA formyltransferase (EC 2.1.2.9) (Met-tRNA(fMet) formyltransferase)	fmt yhdD b3288 JW3249
2	91.75	Q8ZLM6	Salmonella typhimurium (strain LT2 / SGSC1412 / ATCC 700720)	315	FMT_SALTY	Methionyl-tRNA formyltransferase (EC 2.1.2.9)	fmt STM3407
3	89.52	E3GCL2	Enterobacter lignolyticus (strain SCF1)	315	E3GCL2_ENTLS	Methionyl-tRNA formyltransferase (EC 2.1.2.9)	fmt Entcl_0427
4	88.25	A6TEU1	Klebsiella pneumoniae subsp. pneumoniae (strain ATCC 700721 / MGH 78578)	315	FMT_KLEP7	Methionyl-tRNA formyltransferase (EC 2.1.2.9)	fmt KPN78578_36510 KPN_03688
5	81.9	A7MPE8	Cronobacter sakazakii (strain ATCC BAA-894) (Enterobacter sakazakii)	315	FMT_CROS8	Methionyl-tRNA formyltransferase (EC 2.1.2.9)	fmt ESA_00038
6	80.57	A8GKG6	Serratia proteamaculans (strain 568)	314	FMT_SERP5	Methionyl-tRNA formyltransferase (EC 2.1.2.9)	fmt Spro_4512
7	80.25	A0A1X1D0B3	Pantoea wallisii	314	A0A1X1D0B3_9GAMM	Methionyl-tRNA formyltransferase (EC 2.1.2.9)	fmt HA48_18360
8	79.68	B2VK94	Erwinia tasmaniensis (strain DSM 17950 / CFBP 7177 / CIP 109463 / NCPPB 4357 / Et1/99)	315	FMT_ERWT9	Methionyl-tRNA formyltransferase (EC 2.1.2.9)	fmt ETA_31290
9	78.1	A1JRZ2	Yersinia enterocolitica serotype O:8 / biotype 1B (strain NCTC 13174 / 8081)	315	FMT_YERE8	Methionyl-tRNA formyltransferase (EC 2.1.2.9)	fmt YE3890
10	75.87	Q8ZJ80	Yersinia pestis	315	FMT_YERPE	Methionyl-tRNA formyltransferase (EC 2.1.2.9)	fmt YPO0241 y4022 YP_0239
11	72.06	Q7MYI1	Photorhabdus laumondii subsp. laumondii (strain DSM 15139 / CIP 105565 / TT01)	315	FMT_PHOLL	Methionyl-tRNA formyltransferase (EC 2.1.2.9)	fmt plu4696
12	71.11	Q2NQQ3	Sodalis glossinidius (strain morsitans)	316	FMT_SODGM	Methionyl-tRNA formyltransferase (EC 2.1.2.9)	fmt SG2247
13	70.7	M1S457	Morganella morganii subsp. morganii KT	314	M1S457_MORMO	Methionyl-tRNA formyltransferase (EC 2.1.2.9)	fmt MU9_278
14	68.47	A0KEW9	Aeromonas hydrophila subsp. hydrophila (strain ATCC 7966 / DSM 30187 / BCRC 13018 / CCUG 14551 / JCM 1027 / KCTC 2358 / NCIMB 9240 / NCTC 8049)	314	FMT_AERHH	Methionyl-tRNA formyltransferase (EC 2.1.2.9)	fmt AHA_0257
15	67.94	B4F1L6	Proteus mirabilis (strain HI4320)	316	FMT_PROMH	Methionyl-tRNA formyltransferase (EC 2.1.2.9)	fmt PMI3287
16	66.88	Q6LLJ2	Photobacterium profundum (strain SS9)	314	FMT_PHOPR	Methionyl-tRNA formyltransferase (EC 2.1.2.9)	fmt PBPRA3580
17	64.86	P44787	Haemophilus influenzae (strain ATCC 51907 / DSM 11121 / KW20 / Rd)	318	FMT_HAEIN	Methionyl-tRNA formyltransferase (EC 2.1.2.9)	fmt HI_0623
18	64.42	B0BRR3	Actinobacillus pleuropneumoniae serotype 3 (strain JL03)	316	FMT_ACTPJ	Methionyl-tRNA formyltransferase (EC 2.1.2.9)	fmt APJL_1620
19	64.13	Q9KVU4	Vibrio cholerae serotype O1 (strain ATCC 39315 / El Tor Inaba N16961)	315	FMT_VIBCH	Methionyl-tRNA formyltransferase (EC 2.1.2.9)	fmt VC_0045
20	63.9	Q65QF1	Mannheimia succiniciproducens (strain MBEL55E)	317	FMT_MANSM	Methionyl-tRNA formyltransferase (EC 2.1.2.9)	fmt MS2202
21	63.49	B5FCW7	Aliivibrio fischeri (strain MJ11) (Vibrio fischeri)	315	FMT_ALIFM	Methionyl-tRNA formyltransferase (EC 2.1.2.9)	fmt VFMJ11_2675
22	62.82	P57949	Pasteurella multocida (strain Pm70)	317	FMT_PASMU	Methionyl-tRNA formyltransferase (EC 2.1.2.9)	fmt PM1560
23	62.1	C4L7Y3	Tolumonas auensis (strain DSM 9187 / TA4)	314	FMT_TOLAT	Methionyl-tRNA formyltransferase (EC 2.1.2.9)	fmt Tola_0152
24	60.7	O85732	Pseudomonas aeruginosa (strain ATCC 15692 / DSM 22644 / CIP 104116 / JCM 14847 / LMG 12228 / 1C / PRS 101 / PAO1)	314	FMT_PSEAE	Methionyl-tRNA formyltransferase (EC 2.1.2.9)	fmt PA0018
25	60.19	A1SR38	Psychromonas ingrahamii (strain 37)	316	FMT_PSYIN	Methionyl-tRNA formyltransferase (EC 2.1.2.9)	fmt Ping_0079
26	59.74	C3KE47	Pseudomonas fluorescens (strain SBW25)	317	FMT_PSEFS	Methionyl-tRNA formyltransferase (EC 2.1.2.9)	fmt PFLU_0017
27	59.55	Q3IDI3	Pseudoalteromonas translucida (strain TAC 125)	321	FMT_PSET1	Methionyl-tRNA formyltransferase (EC 2.1.2.9)	fmt PSHAa0022
28	56.73	Q31J85	Hydrogenovibrio crunogenus (strain DSM 25203 / XCL-2) (Thiomicrospira crunogena)	312	FMT_HYDCU	Methionyl-tRNA formyltransferase (EC 2.1.2.9)	fmt Tcr_0192
29	56.59	A1TWN0	Marinobacter hydrocarbonoclasticus (strain ATCC 700491 / DSM 11845 / VT8)	311	FMT_MARHV	Methionyl-tRNA formyltransferase (EC 2.1.2.9)	fmt Maqu_0042

YaaA-peroxide stress resistance protein

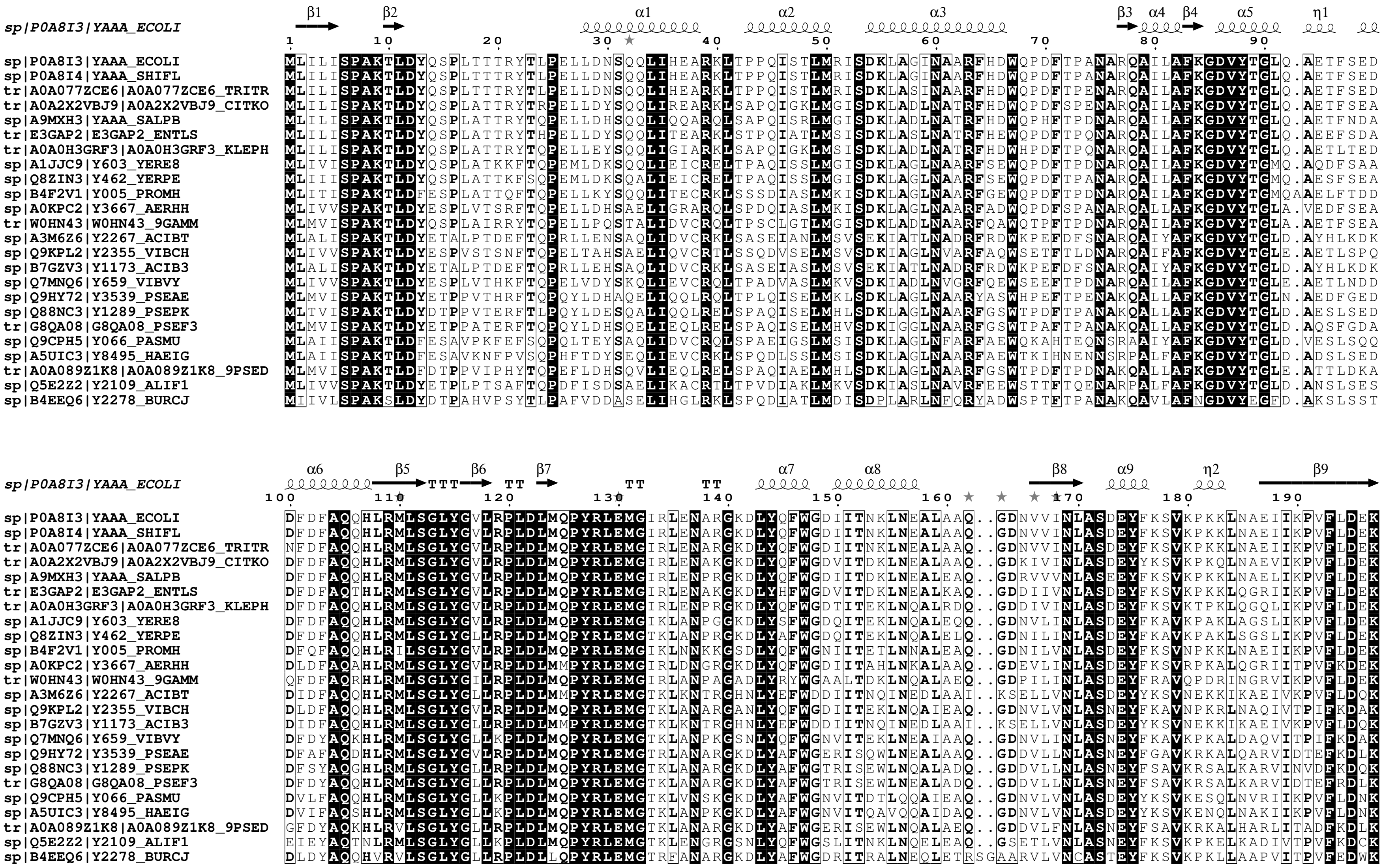

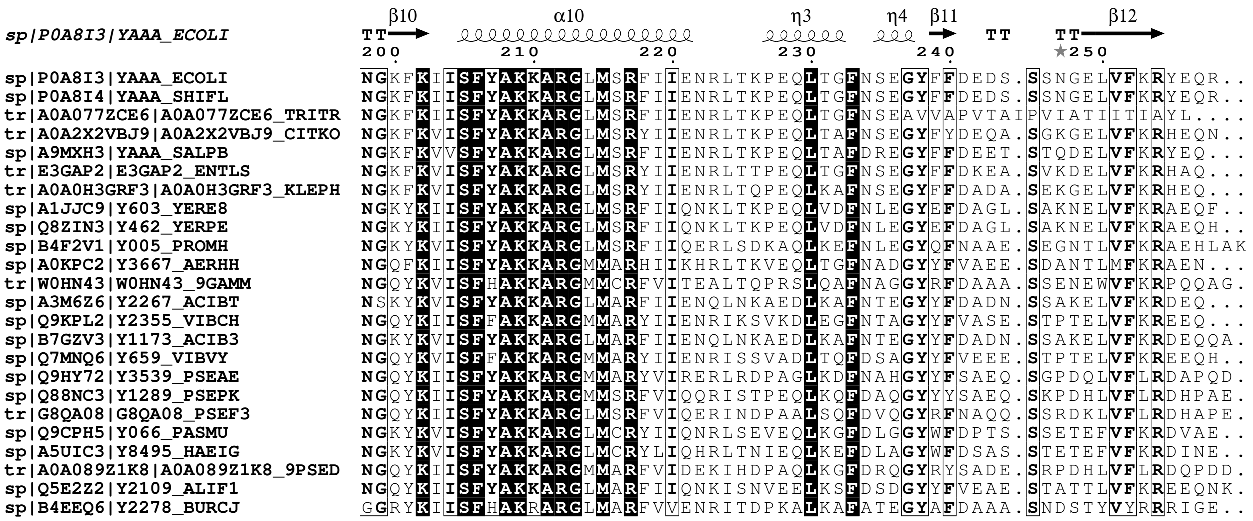

sp|P0A8I3|YAAA_ECOLI
β10
α10
η3
η4
β11
β12
TT
TT
TT
200
210
220
230
240
250
sp|P0A8I3|YAAA_ECOLI NGKFKIISFYAKKARGLMSRFIIENRLTKPEQLTGFNSEGYFFDEDS.SSNGELVFKRYEQR..
sp|P0A8I4|YAAA_SHIFL NGKFKIISFYAKKARGLMSRFIIENRLTKPEQLTGFNSEGYFFDEDS.SSNGELVFKRYEQR..
tr|A0A077ZCE6|A0A077ZCE6_TRITR NGKFKIISFYAKKARGLMSRFIIENRLTKPEQLTGFNSEAVVAPVTAIPVIATIITIAYL....
tr|A0A2X2VBJ9|A0A2X2VBJ9_CITKO NGKFKVISFYAKKARGLMSRYIIENRLTKPEQLTAFNSEGYFYDEQA.SGKGELVFKRHEQN..
sp|A9MXH3|YAAA_SALPB NGKFKVVSFYAKKARGLMSRFIIENRLTKPEQLTAFDREGYFFDEET.STQDELVFKRYEQ...
tr|E3GAP2|E3GAP2_ENTLS NGKFKVISFYAKKARGLMSRYIIENRLTTPEQLTGFNSEGYFFDKEA.SVKDELVFKRHAQ...
tr|A0A0H3GRF3|A0A0H3GRF3_KLEPH NGKFKVISFYAKKARGLMSRYIIENRLTQPEQLKAFNSEGYFFDADA.SEKGELVFKRHEQ...
sp|A1JJC9|Y603_YERE8 NGKYKIISFYAKKARGLMSRFIIQNKLTKPEQLVDFNLEGYEFDAGL.SAKNELVFKRAEQF..
sp|Q8ZIN3|Y462_YERPE NGKYKIISFYAKKARGLMSRFIIQNKLTKPEQLVDFNLEGYEFDAGL.SAKNELVFKRAEQH..
sp|B4F2V1|Y005_PROMH NGKYKVISFYAKKARGLMSRFIIQERLSDKAQLKEFNLEGYQFNAAE.SEGNTLVFKRAEHLAK
sp|A0KPC2|Y3667_AERHH NGQFKIISFYAKKARGMMARHIIKHRLTKVEQLTGFNADGYYFVAEE.SDANTLMFKRAEN...
tr|W0HN43|W0HN43_9GAMM NGQYKVISFHAKKARGMMCRFVITEALTQPRSLQAFNAGGYRFDAAA.SSENEWVFKRPQQAG.
sp|A3M6Z6|Y2267_ACIBT NSKYKVISFYAKKARGLMARFIIENQLNKAEDLKAFNTEGYYFDADN.SSAKELVFKRDEQ...
sp|Q9KPL2|Y2355_VIBCH NGQYKIISFFAKKARGMMARYIIENRIKSVKDLEGFNTAGYYFVASE.STPTELVFKREEQ...
sp|B7GZV3|Y1173_ACIB3 NGKYKVISFYAKKARGLMARFIIENQLNKAEDLKAFNTEGYYFDADN.SSAKELVFKRDEQQA.
sp|Q7MNQ6|Y659_VIBVY NGQYKVISFFAKKARGMMARYIIENRISSVADLTQFDSAGYYFVEEE.STPTELVFKREEQH..
sp|Q9HY72|Y3539_PSEAE NGQYKIISFYAKKARGMMARYVIRERLRDPAGLKDFNAHGYYFSAEQ.SGPDQLVFLRDAPQD.
sp|Q88NC3|Y1289_PSEPK NGQYKIISFYAKKARGMMSRFVIQQRISTPEQLKQFDAQGYYYSAEQ.SKPDHLVFLRDHPAE.
tr|G8QA08|G8QA08_PSEF3 NGQYKIISFYAKKARGLMSRFVIQERINDPAALSQFDVQGYRFNAQQ.SSRDKLVFLRDHAPE.
sp|Q9CPH5|Y066_PASMU NGKYKVISFYAKKARGLMCRYIIQNRLSEVEQLKGFDLGGYWFDPTS.SSETEFVFKRDVAE..
sp|A5UIC3|Y8495_HAEIG NGKYKVISFYAKKARGLMCRYLIQHRLTNIEQLKEFDLAGYWFDSAS.STETEFVFKRDINE..
tr|A0A089Z1K8|A0A089Z1K8_9PSED NGQYKIISFYAKKARGMMARFVIDEKIHDPAQLKGFDRQGYRYSADE.SRPDHLVFLRDQPDD.
sp|Q5E2Z2|Y2109_ALIF1 NGQYKIISFYAKKARGLMARFIIQNKISNVEELKSFDSDGYYFVEAE.STATTLVFKREEQNK.
sp|B4EEQ6|Y2278_BURCJ GGRYKIISFHAKRARGLMARFVVENRITDPKALKAFATEGYAFDAAA.SNDSTYVYRRRIGE..

Sequences used in YaaA alignment:

Order	% Sequence Identity	UniProt ID	Organism	Length	Entry name	Protein names	Gene names
1	100	P0A8I3	Escherichia coli (strain K12)	258	YAAA_ECOLI	Peroxide stress resistance protein YaaA (UPF0246 protein YaaA)	yaaA b0006 JW0005
2	100	P0A8I4	Shigella flexneri	258	YAAA_SHIFL	UPF0246 protein YaaA	yaaA SF0006 S0006
3	91.41	A0A077ZCE6	Trichuris trichiura (Whipworm) (Trichocephalus trichiurus)	257	A0A077ZCE6_TRITR	DUF328 domain containing protein	TTRE_0000636301
4	87.21	A0A2X2VBJ9	Citrobacter koseri (Citrobacter diversus)	258	A0A2X2VBJ9_CITKO	UPF0246 protein GTJ50_09415	yaaA E4T98_12495 GTJ50_09415 NCTC10786_01761 NCTC10810_04751 NCTC5055_03920
5	86.38	A9MXH3	Salmonella paratyphi B (strain ATCC BAA-1250 / SPB7)	257	YAAA_SALPB	UPF0246 protein YaaA	yaaA SPAB_00005
6	84.44	E3GAP2	Enterobacter lignolyticus (strain SCF1)	257	E3GAP2_ENTLS	UPF0246 protein Entcl_3712	Entcl_3712
7	83.66	A0A0H3GRF3	Klebsiella pneumoniae subsp. pneumoniae (strain HS11286)	257	A0A0H3GRF3_KLEPH	UPF0246 protein KPHS_07160	KPHS_07160
8	78.29	A1JJC9	Yersinia enterocolitica serotype O:8 / biotype 1B (strain NCTC 13174 / 8081)	258	Y603_YERE8	UPF0246 protein YE0603	YE0603
9	78.29	Q8ZIN3	Yersinia pestis	258	Y462_YERPE	UPF0246 protein YPO0462/y3714/YP_3720	YPO0462 y3714 YP_3720
10	75.81	B4F2V1	Proteus mirabilis (strain HI4320)	261	Y005_PROMH	UPF0246 protein PMI0005	PMI0005
11	75.1	A0KPC2	Aeromonas hydrophila subsp. hydrophila (strain ATCC 7966 / DSM 30187 / BCRC 13018 / CCUG 14551 / JCM 1027 / KCTC 2358 / NCIMB 9240 / NCTC 8049)	257	Y3667_AERHH	UPF0246 protein AHA_3667	AHA_3667
12	68.22	W0HN43	Candidatus Sodalis pierantonius str. SOPE	259	W0HN43_9GAMM	UPF0246 protein SOPEG_1358	yaaA SOPEG_1358
13	66.15	A3M6Z6	Acinetobacter baumannii (strain ATCC 17978 / CIP 53.77 / LMG 1025 / NCDC KC755 / 5377)	257	Y2267_ACIBT	UPF0246 protein A1S_2267	A1S_2267
14	66.15	Q9KPL2	Vibrio cholerae serotype O1 (strain ATCC 39315 / El Tor Inaba N16961)	257	Y2355_VIBCH	UPF0246 protein VC_2355	VC_2355
15	65.89	B7GZV3	Acinetobacter baumannii (strain AB307-0294)	259	Y1173_ACIB3	UPF0246 protein ABBFA_001173	ABBFA_001173
16	65.89	Q7MNQ6	Vibrio vulnificus (strain YJ016)	258	Y659_VIBVY	UPF0246 protein VV0659	VV0659
17	65.12	Q9HY72	Pseudomonas aeruginosa (strain ATCC 15692 / DSM 22644 / CIP 104116 / JCM 14847 / LMG 12228 / 1C / PRS 101 / PAO1)	259	Y3539_PSEAE	UPF0246 protein PA3539	PA3539
18	64.73	Q88NC3	Pseudomonas putida (strain ATCC 47054 / DSM 6125 / NCIMB 11950 / KT2440)	259	Y1289_PSEPK	UPF0246 protein PP_1289	PP_1289
19	63.57	G8QA08	Pseudomonas fluorescens (strain F113)	259	G8QA08_PSEF3	UPF0246 protein PSF113_4751	yaaA PSF113_4751
20	63.18	Q9CPH5	Pasteurella multocida (strain Pm70)	258	Y066_PASMU	UPF0246 protein PM0066	PM0066
21	62.02	A5UIC3	Haemophilus influenzae (strain PittGG)	258	Y8495_HAEIG	UPF0246 protein CGSHiGG_08495	CGSHiGG_08495
22	61.63	A0A089Z1K8	Pseudomonas rhizosphaerae	259	A0A089Z1K8_9PSED	UPF0246 protein LT40_20520	LT40_20520
23	61.24	Q5E2Z2	Aliivibrio fischeri (strain ATCC 700601 / ES114) (Vibrio fischeri)	259	Y2109_ALIF1	UPF0246 protein VF_2109	VF_2109
24	58.53	B4EEQ6	Burkholderia cenocepacia (strain ATCC BAA-245 / DSM 16553 / LMG 16656 / NCTC 13227 / J2315 / CF5610) (Burkholderia cepacia (strain J2315))	260	Y2278_BURCJ	UPF0246 protein BceJ2315_22780	BceJ2315_22780 BCAL2318
