# Supplementary material for: Investigation of the Importance of Protein 3D Structure for Assessing Conservation of Lysine Acetylation Sites in Protein Homologs
Source: Front Microbiol. 2022 Jan 31;12:805181. doi: 10.3389/fmicb.2021.805181 (PMC8843374; doi:10.3389/fmicb.2021.805181)
Supplement: Supplementary file 8 [file Data_Sheet_8.PDF]

Supplemental Figure SF5A. Pairwise sequence alignments between the *E. coli* substrate protein target (Adk-adenylate kinase) and homologs sorted by UniProt ID. Labels adjacent to each sequence include PDB ID, UniProt ID, and organism name. Lysine residues previously identified as acetylated in the target protein are highlighted in yellow to examine conservation in 1D.

```
                              1        10        20        30        40        50        60
P69441_ESCHERICHIA_COLI       MRIILLGAPGAGKGTQAQFIMEKYGIPQISTGDMLRAAVKSGSELGKQAKDIMDAGKLVT
A0A0J9X1X4_ESCHERICHIA_COLI   MRIILLGAPGAGKGTQAQFIMEKYGIPQISTGDMLRAAVKSGSELGKQAKDIMDCGKLVT

                                       70        80        90       100       110       120
P69441_ESCHERICHIA_COLI       DELVIALVKERIAQEDCRNGFLLDGFPRTIPQADAMKEAGINVDYVLEFDVPDELIVDRI
A0A0J9X1X4_ESCHERICHIA_COLI   DELVIALVKERIAQEDSRNGFLLDGFPRTIPQADAMKEAGINVDYVLEFDVPDELIVDRI

                                      130       140       150       160       170       180
P69441_ESCHERICHIA_COLI       VGRRVHAPSGRVYHVKFNPPKVEGKDDVTGEELTTRKDDQEETVRKRLVEYHQMTAPLIG
A0A0J9X1X4_ESCHERICHIA_COLI   VGRRVHAPSGRVYHVKFNPPKVEGKDDVTGEELTTRKDDQEETVRKRLCEYHQMTAPLIG

                                      190       200       210
P69441_ESCHERICHIA_COLI       YYSKEAEAGNTKYAKVDGTKPVAEVRADLEKILG
A0A0J9X1X4_ESCHERICHIA_COLI   YYSKEAEAGNTKYAKVDGTKPVAEVRADLEKILG
```

```
                                            1         10         20         30         40         50
P69441_ESCHERICHIA_COLI             ..........MRIILLGAPGAGKGTQAQFIMEKYGIPQISTGDMLRAAVKSGSELGKQAK
A0A2R2JFU5_NOTOTHENIA_CORIICEPS     GHMADKIKDAKIIFVVGGPGSGKGTQCEKVVAKYGYTHLSSGDLLRAEVSSGSERGKQLQ

                                          60         70         80         90        100
P69441_ESCHERICHIA_COLI             DIMDAGKLVTDELVIALVKER.IAQEDCRNGFLLDGFPRTIPQADAMKEAGINVDYVLEF
A0A2R2JFU5_NOTOTHENIA_CORIICEPS     AIMQKGELVPLDTVLDMIKDAMIAKADVSKGYLIDGYPREVKQGEEFEKKIGKPCLLLYV

                                    110        120        130        140        150        160
P69441_ESCHERICHIA_COLI             DVPDELIVDRIVGRRVHAPSGRVYHVKFNPPKVEGKDDVTGEELTTRKDDQEETVRKRLV
A0A2R2JFU5_NOTOTHENIA_CORIICEPS     DAKGETMVKRLMKRG...........................ETSGRADDNEETIKKRLD

                                    170        180        190        200        210
P69441_ESCHERICHIA_COLI             EYHQMTAPLIGYYSKEAEAGNTKYAKVDGTKPVAEVRADLEKILG..
A0A2R2JFU5_NOTOTHENIA_CORIICEPS     LYYKATEPVIAFYE.....GRGIVRKVDSELPVDEVFKQVSTAIDAL
```

```
                                                1         10        20        30        40        50
P69441_ESCHERICHIA_COLI               ..........MRIILLGAPGAGKGTQAQFIMEKYGIPQISTGDMLRAAVKSGSELGKQAK
A0A2R2JFU6_NOTOTHENIA_CORIICEPS       GHMADKIKDAKIIFVVGGPGSGKGTQCEKIVAKYGYTHLSSGDLLRAEVSSGSERGKQLQ

                                              60        70        80        90       100
P69441_ESCHERICHIA_COLI               DIMDAGKLVTDELVIALVKER.IAQEDCRNGFLLDGFPRTIPQADAMKEAGINVDYVLEF
A0A2R2JFU6_NOTOTHENIA_CORIICEPS       AIMQKGELVPLDTVLDMIKDAMIAKADVSKGYLIDGYPREVKQGEEFEKKIGKPCLLLYV

                                    110       120       130       140       150       160
P69441_ESCHERICHIA_COLI               DVPDELIVDRIVGRRVHAPSGRVYHVKFNPPKVEGKDDVTGEELTTRKDDQEETVRKRLV
A0A2R2JFU6_NOTOTHENIA_CORIICEPS       DAKGETMVKRLMKRG...........................ETSGRADDNEETIKKRLD

                                    170       180       190       200       210
P69441_ESCHERICHIA_COLI               EYHQMTAPLIGYYSKEAEAGNTKYAKVDGTKPVAEVRADLEKILG..
A0A2R2JFU6_NOTOTHENIA_CORIICEPS       LYYKATEPVIAFYE.....GRGIVRKVDSELPVDEVFKQVSTAIDAL
```

```
                                1        10        20        30        40        50
P69441_ESCHERICHIA_COLI         MR........IILLGAPGAGKGTQAQFIMEKYGIPQISTGDMLRAAVKSGSELGKQAKDI
A0A452CSM2_POECILIA_RETICULATA  MADKIKDAKIIFVVGGPGSGKGTQCEKIVAKYGYTHLSSGDLLRAEVASGSERGKQLQAI

                                      60        70        80        90       100       110
P69441_ESCHERICHIA_COLI         MDAGKLVTDELVIALVKER.IAQEDCRNGFLLDGFPRTIPQADAMKEAGINVDYVLEFDV
A0A452CSM2_POECILIA_RETICULATA  MQKGELVPLDTVLDMIKDAMIAKADVSKGFLIDGYPREVKQGEEFEKKIGKPCLLLYVDA

                                      120       130       140       150       160       170
P69441_ESCHERICHIA_COLI         PDELIVDRIVGRRVHAPSGRVYHVKFNPPKVEGKDDVTGEELTTRKDDQEETVRKRLVEY
A0A452CSM2_POECILIA_RETICULATA  KAETMVKRLLKRG.........................ETSGRSDDNEETIKKRLDLY

                                      180       190       200       210
P69441_ESCHERICHIA_COLI         HQMTAPLIGYYSKEAEAGNTKYAKVDGTKPVAEVRADLEKILG..
A0A452CSM2_POECILIA_RETICULATA  YKATEPVIAFYE.....GRGIVKKVDSELAVDDVFGQVSKAIDAL
```

```
                            1        10        20        30        40        50        60
P69441_ESCHERICHIA_COLI     MRIILLGAPGAGKGTQAQFIMEKYGIPQISTGDMLRAAVKSGSELGKQAKDIMDAGKLVT
A7ZIN4_ESCHERICHIA_COLI     MRIILLGAPGAGKGTQAQFIMEKYGIPQISTGDMLRAAVKSGSELGKQAKDIMDAGKLVT

                                     70        80        90       100       110       120
P69441_ESCHERICHIA_COLI     DELVIALVKERIAQEDCRNGFLLDGFPRTIPQADAMKEAGINVDYVLEFDVPDELIVDRI
A7ZIN4_ESCHERICHIA_COLI     DELVIALVKERIAQEDCRNGFLLDGFPRTIPQADAMKEAGINVDYVLEFDVPDELIVDRI

                                    130       140       150       160       170       180
P69441_ESCHERICHIA_COLI     VGRRVHAPSGRVYHVKFNPPKVEGKDDVTGEELTTRKDDQEETVRKRLVEYHQMTAPLIG
A7ZIN4_ESCHERICHIA_COLI     VGRRVHAPSGRVYHVKFNPPKVEGKDDVTGEELTTRKDDQEETVRKRLVEYHQMTAPLIG

                                    190       200       210
P69441_ESCHERICHIA_COLI     YYSKEAEAGNTKYAKVDGTKPVAEVRADLEKILG
A7ZIN4_ESCHERICHIA_COLI     YYSKEAEAGNTKYAKVDGTKPVAEVRADLEKILG
```

```
                             1        10        20        30        40        50        60
P69441_ESCHERICHIA_COLI      MRIILLGAPGAGKGTQAQFIMEKYGIPQISTGDMLRAAVKSGSELGKQAKDIMDAGKLVT
C7U112_DESULFOVIBRIO_GIGAS   MNILIFGPNGSGKGTQGNLVKDKYSLAHIESGGIFREHIGGGTELGKKAKEFIDRGDLVP

                                      70        80        90         100       110
P69441_ESCHERICHIA_COLI      DELVIALVKERIAQEDCRNGFLLDGFPRTIPQA....DAMKEAGINVDYVLEFDVPDELI
C7U112_DESULFOVIBRIO_GIGAS   DDITIPMVLETLESKG.KDGWLLDGFPRNTVQAQKLFEALQEKGMKINFVIEILLPREVA

                               120            130       140       150       160
P69441_ESCHERICHIA_COLI      VDRIVGRRV......HAPSGRVYHVKFNPPKVEGKDDVTGEELTTRKDDQEE.TVRKRLV
C7U112_DESULFOVIBRIO_GIGAS   KNRIMGRRICKNNPNHPNNIFIEAIKPNGDVCR....VCGGALSARADDQDEGAINKRHD

                          170         180       190       200       210
P69441_ESCHERICHIA_COLI      EYHQM...TAPLIGYYSKEAEAGNTKYAKVDGTKPVAEVRADLEKILG
C7U112_DESULFOVIBRIO_GIGAS   IYYNTVDGTLAAAYYYKNMAAKEGFVYIELDGEGSIDSIKDTLLAQLA
```

```
                               1        10        20        30        40        50
P69441_ESCHERICHIA_COLI        MR.........IILLGAPGAGKGTQAQFIMEKYGIPQISTGDMLRAAVKSGSELGKQAKD
G4V9S0_SCHISTOSOMA_MANSONI     MTDQKLAKAKVIFVLGGPGSGKGTQCEKLVQKFHFNHLSSGDLLRAEVQSGSPKGKELKA

                                        60        70        80        90       100
P69441_ESCHERICHIA_COLI        IMDAGKLVTDELVIALVKE...RIAQEDCRNGFLLDGFPRTIPQADAMKEAGINVDYVLE
G4V9S0_SCHISTOSOMA_MANSONI     MMERGELVPLEVVLALLKEAMIKLVDKNCH..FLIDGYPRELDQGIKFEKEVCPCLCVIN

                              110       120       130       140       150       160
P69441_ESCHERICHIA_COLI        FDVPDELIVDRIVGRRVHAPSGRVYHVKFNPPKVEGKDDVTGEELTTRKDDQEETVRKRL
G4V9S0_SCHISTOSOMA_MANSONI     FDVSEEVMRKRLLKRA...........................ETSNRVDDNEETIVKRF

                              170       180       190       200       210
P69441_ESCHERICHIA_COLI        VEYHQMTAPLIGYYSKEAEAGNTKYAKVDGTKPVA....EVRADLEKILG..
G4V9S0_SCHISTOSOMA_MANSONI     RTFNELTKPVIEHYKQQ.....NKVITIDASGTVDAIFDKVNHELQKF.GVK
```

```
                                    1        10        20        30        40        50
P69441_ESCHERICHIA_COLI             MR........IILLGAPGAGKGTQAQFIMEKYGIPQISTGDMLRAAVKSGSELGKQAKDI
M4AA20_XIPHOPHORUS_MACULATUS        MADKIKDAKIIFVVGGPGSGKGTQCEKIVAKYGYTHLSSGDLLRAEVASGSERGKQLQAI

                                         60        70        80        90       100       110
P69441_ESCHERICHIA_COLI             MDAGKLVTDELVIALVKER.IAQEDCRNGFLLDGFPRTIPQADAMKEAGINVDYVLEFDV
M4AA20_XIPHOPHORUS_MACULATUS        MQKGELVPLDTVLDMIKDAMIAKADVSKGFLIDGYPREVKQGEEFEKKIGKPCLLLYVDA

                                         120       130       140       150       160       170
P69441_ESCHERICHIA_COLI             PDELIVDRIVGRRVHAPSGRVYHVKFNPPKVEGKDDVTGEELTTRKDDQEETVRKRLVEY
M4AA20_XIPHOPHORUS_MACULATUS        KAETMVKRLLKRG..........................ETSGRSDDNEETIKKRLDLY

                                         180       190       200       210
P69441_ESCHERICHIA_COLI             HQMTAPLIGYYSKEAEAGNTKYAKVDGTKPVAEVRADLEKILG..
M4AA20_XIPHOPHORUS_MACULATUS        YKATEPVIAFYE.....GRGIVKKVDSELAVDDVFAQVSKAIDAL
```

```
                            1        10        20        30        40        50        60
P69441_ESCHERICHIA_COLI     MRIILLGAPGAGKGTQAQFIMEKYGIPQISTGDMLRAAVKSGSELGKQAKDIMDAGKLVT
O66490_AQUIFEX_AEOLICUS     MILVFLGPPGAGKGTQAKRLAKEKGFVHISTGDILREAVQKGTPLGKKAKEYMERGELVP

                                     70        80        90       100       110
P69441_ESCHERICHIA_COLI     DELVIALVKERIAQEDCRNGFLLDGFPRTIPQADAMKEA....GINVDYVLEFDVPDELI
O66490_AQUIFEX_AEOLICUS     DDLIIALIEEVFPKH...GNVIFDGFPRTVKQAEALDEMLEKKGLKVDHVLLFEVPDEVV

                             120       130       140       150       160       170
P69441_ESCHERICHIA_COLI     VDRIVGRRVHAPSGRVYHVKFNPPKVEGKDDVTGEELTTRKDDQEETVRKRLVEYHQMTA
O66490_AQUIFEX_AEOLICUS     IERLSGRRINPETGEVYHVKYNPPP.......PGVKVIQREDDKPEVIKKRLEVYREQTA

                             180       190       200       210
P69441_ESCHERICHIA_COLI     PLIGYYSKEAEAGNTKYAKVDGTKPVAEVRADLEKILG...
O66490_AQUIFEX_AEOLICUS     PLIEYYKKK...GILRI..IDASKPVEEVYRQVLEVIGDGN
```

```
                             1        10        20        30        40        50
P69441_ESCHERICHIA_COLI  MR........IILLGAPGAGKGTQAQFIMEKYGIPQISTGDMLRAAVKSGSELGKQAKDI
P00568_HOMO_SAPIENS      MEEKLKKTKIIFVVGGPGSGKGTQCEKIVQKYGYTHLSTGDLLRSEVSSGSARGKKLSEI

                                60        70         80        90       100       110
P69441_ESCHERICHIA_COLI  MDAGKLVTDELVIALVKER.IAQEDCRNGFLLDGFPRTIPQADAMKEAGINVDYVLEFDV
P00568_HOMO_SAPIENS      MEKGQLVPLETVLDMLRDAMVAKVNTSKGFLIDGYPREVQQGEEFERRIGQPTLLLYVDA

                               120       130       140       150       160       170
P69441_ESCHERICHIA_COLI  PDELIVDRIVGRRVHAPSGRVYHVKFNPPKVEGKDDVTGEELTTRKDDQEETVRKRLVEY
P00568_HOMO_SAPIENS      GPETMTQRLLKRG..ETSGRV.........................DDNEETIKKRLETY

                               180       190         200       210
P69441_ESCHERICHIA_COLI  HQMTAPLIGYYSKEAEAGNTKYAKVDGT..KPVAEVRADLEKILG
P00568_HOMO_SAPIENS      YKATEPVIAFYEKR...GIVRKVNAEGSVDSVFSQVCTHLDALK.
```

```
                          1        10        20        30        40        50
P69441_ESCHERICHIA_COLI   MR........IILLGAPGAGKGTQAQFIMEKYGIPQISTGDMLRAAVKSGSELGKQAKDI
P00571_SUS_SCROFA         MEEKLKKSKIIFVVGGPGSGKGTQCEKIVQKYGYTHLSTGDLLRAEVSSGSARGKMLSEI

                                 60        70        80        90       100       110
P69441_ESCHERICHIA_COLI   MDAGKLVTDELVIALVKER.IAQEDCRNGFLLDGFPRTIPQADAMKEAGINVDYVLEFDV
P00571_SUS_SCROFA         MEKGQLVPLETVLDMLRDAMVAKVDTSKGFLIDGYPREVKQGEEFERKIGQPTLLLYVDA

                                120       130       140       150       160       170
P69441_ESCHERICHIA_COLI   PDELIVDRIVGRRVHAPSGRVYHVKFNPPKVEGKDDVTGEELTTRKDDQEETVRKRLVEY
P00571_SUS_SCROFA         GPETMTKRLLKRG..ETSGRV.........................DDNEETIKKRLETY

                                180       190       200         210
P69441_ESCHERICHIA_COLI   HQMTAPLIGYYSKEAEAGNTKYAKVDGTKP..VAEVRADLEKILG
P00571_SUS_SCROFA         YKATEPVIAFYEKR...GIVRKVNAEGSVDDVFSQVCTHLDTLK.
```

```
                                           1       10        20        30        40        50
P69441_ESCHERICHIA_COLI              ......MRIILLGAPGAGKGTQAQFIMEKYGIPQISTGDMLRAAVKSGSELGKQAKDIMD
P07170_SACCHAROMYCES_CEREVISIAE      MSSSESIRMVLIGPPGAGKGTQAPNLQERFHAAHLATGDMLRSQIAKGTQLGLEAKKIMD

                                         60        70         80        90            100
P69441_ESCHERICHIA_COLI              AGKLVTDELVIALVKERIAQED.CRNGFLLDGFPRTIPQADAM....KEAGINVDYVLEF
P07170_SACCHAROMYCES_CEREVISIAE      QGGLVSDDIMVNMIKDELTNNPACKNGFILDGFPRTIPQAEKLDQMLKEQGTPLEKAIEL

                                   110       120       130       140       150       160
P69441_ESCHERICHIA_COLI              DVPDELIVDRIVGRRVHAPSGRVYHVKFNPPKVEGKDDVTGEELTTRKDDQEETVRKRLV
P07170_SACCHAROMYCES_CEREVISIAE      KVDDELLVARITGRLIHPASGRSYHKIFNPPKEDMKDDVTGEALVQRSDDNADALKKRLA

                                   170       180       190       200       210
P69441_ESCHERICHIA_COLI              EYHQMTAPLIGYYSKEAEAGNTKYAKVDGTKPVAEVRADLEKILG..
P07170_SACCHAROMYCES_CEREVISIAE      AYHAQTEPIVDFYKKTGI.....WAGVDASQPPATVWADILNKLGKD
```

```
                                           1         10        20        30        40
P69441_ESCHERICHIA_COLI  .................MRIILLGAPGAGKGTQAQFIMEKYGIPQISTGDMLRAAVKSGS
P08166_BOS_TAURUS        MAPNVPAAEPVPESPKGVRAVLLGPPGAGKGTQAPKLAKNFCVCHLATGDMLRAMVASGS

                                 50        60        70        80        90
P69441_ESCHERICHIA_COLI  ELGKQAKDIMDAGKLVTDELVIALVKERIAQEDCRNGFLLDGFPRTIPQADAMKEA....
P08166_BOS_TAURUS        ELGKKLKATMDAGKLVSDEMVLELIEKNLETPPCKNGFLLDGFPRTVRQAEMLDDLMEKR

                         100       110       120       130       140       150
P69441_ESCHERICHIA_COLI  GINVDYVLEFDVPDELIVDRIVGRRVHAPSGRVYHVKFNPPKVEGKDDVTGEELTTRKDD
P08166_BOS_TAURUS        KEKLDSVIEFSIPDSLLIRRITGRLIHPQSGRSYHEEFNPPKEPMKDDITGEPLIRRSDD

                         160       170       180       190       200       210
P69441_ESCHERICHIA_COLI  QEETVRKRLVEYHQMTAPLIGYYSKEAEAGNTKYAKVDGTKPVAEVRADLEKILG.....
P08166_BOS_TAURUS        NKKALKIRLEAYHTQTTPLVEYYSKRG.....IHSAIDASQTPDVVFASILAAFSKATCK

P69441_ESCHERICHIA_COLI  ......
P08166_BOS_TAURUS        DLVMFI
```

```
                                 1       10        20        30        40        50
P69441_ESCHERICHIA_COLI   .......MRIILLGAPGAGKGTQAQFIMEKYGIPQISTGDMLRAAVKSGSELGKQAKDIM
P08760_BOS_TAURUS         MGASARLLRAAIMGAPGSGKGTVSSRITKHFELKHLSSGDLLRDNMLRGTEIGVLAKTFI

                               60        70        80        90       100       110
P69441_ESCHERICHIA_COLI   DAGKLVTDELVIALVKERIAQEDCRNGFLLDGFPRTIPQADAMKEAGINVDYVLEFDVPD
P08760_BOS_TAURUS         DQGKLIPDDVMTRLVLHELKNLTQYN.WLLDGFPRTLPQAEALDRA.YQIDTVINLNVPF

                              120       130       140       150       160       170
P69441_ESCHERICHIA_COLI   ELIVDRIVGRRVHAPSGRVYHVKFNPPKVEGKDDVTGEELTTRKDDQEETVRKRLVEYHQ
P08760_BOS_TAURUS         EVIKQRLTARWIHPGSGRVYNIEFNPPKTMGIDDLTGEPLVQREDDRPETVVKRLKAYEA

                              180       190       200       210
P69441_ESCHERICHIA_COLI   MTAPLIGYYSKEAEAGNTKYAKVDGTKPVAEVRADLEKILG..........
P08760_BOS_TAURUS         QTEPVLEYYRKKGVLET..FSGTETNKIWPHVYAFLQTKLPQRSQETSVTP
```

```
                           1        10        20        30        40        50        60
P69441_ESCHERICHIA_COLI    MRIILLGAPGAGKGTQAQFIMEKYGIPQISTGDMLRAAVKSGSELGKQAKDIMDAGKLVT
P16304_BACILLUS_SUBTILIS   MNLVLMGLPGAGKGTQGERIVEDYGIPHISTGDMFRAAMKEETPLGLEAKSYIDKGELVP

                                    70        80        90       100       110
P69441_ESCHERICHIA_COLI    DELVIALVKERIAQEDCRNGFLLDGFPRTIPQADAMKEA....GINVDYVLEFDVPDELI
P16304_BACILLUS_SUBTILIS   DEVTIGIVKERLGKDDCERGFLLDGFPRTVAQAEALEEILEEYGKPIDYVINIEVDKDVL

                             120       130       140       150       160       170
P69441_ESCHERICHIA_COLI    VDRIVGRRVHAPSGRVYHVKFNPPKVEGKDDVTGEELTTRKDDQEETVRKRLVEYHQMTA
P16304_BACILLUS_SUBTILIS   MERLTGRRICSVCGTTYHLVFNPPKTPGICDKDGGELYQRADDNEETVSKRLEVNMKQTQ

                             180       190       200       210
P69441_ESCHERICHIA_COLI    PLIGYYSKEAEAGNTKYAKVDGTKPVAEVRADLEKILG....
P16304_BACILLUS_SUBTILIS   PLLDFYSEKGYLAN.....VNGQQDIQDVYADVKDLLGLKK
```

```
                                    1        10        20        30        40        50        60
P69441_ESCHERICHIA_COLI             MRIILLGAPGAGKGTQAQFIMEKYGIPQISTGDMLRAAVKSGSELGKQAKDIMDAGKLVT
P27142_GEOBACILLUS_STEAROTHERMOPHILUS MNLVLMGLPGAGKGTQAEKIVAAYGIPHISTGDMFRAAMKEGTPLGLQAKQYMDRGDLVP

                                             70        80        90       100       110
P69441_ESCHERICHIA_COLI             DELVIALVKERIAQEDCRNGFLLDGFPRTIPQADAMK....EAGINVDYVLEFDVPDELI
P27142_GEOBACILLUS_STEAROTHERMOPHILUS DEVTIGIVRERLSKDDCQNGFLLDGFPRTVAQAEALETMLADIGRKLDYVIHIDVRQDVL

                                      120       130       140       150       160       170
P69441_ESCHERICHIA_COLI             VDRIVGRRVHAPSGRVYHVKFNPPKVEGKDDVTGEELTTRKDDQEETVRKRLVEYHQMTA
P27142_GEOBACILLUS_STEAROTHERMOPHILUS MERLTGRRICRNCGATYHLIFHPPAKPGVCDKCGGELYQRADDNEATVANRLEVNMKQMK

                                      180       190       200       210
P69441_ESCHERICHIA_COLI             PLIGYYSKEAEAGNTKYAKVDGTKPVAEVRADLEKILG....
P27142_GEOBACILLUS_STEAROTHERMOPHILUS PLVDFYEQKGYLRN.....INGEQDMEKVFADIRELLGGLAR
```

```
                            1        10        20        30        40        50
P69441_ESCHERICHIA_COLI  .....MRIILLGAPGAGKGTQAQFIMEKYGIPQISTGDMLRAAVKSGSELGKQAKDIMDA
P27144_HOMO_SAPIENS      MASKLLRAVILGPPGSGKGTVCQRIAQNFGLQHLSSGHFLRENIKASTEVGEMAKQYIEK

                           60        70        80        90       100       110
P69441_ESCHERICHIA_COLI  GKLVTDELVIALVKERIAQEDCRNGFLLDGFPRTIPQADAMKEAGINVDYVLEFDVPDEL
P27144_HOMO_SAPIENS      SLLVPDHVITRLMMSELENRRGQH.WLLDGFPRTLGQAEALDKI.CEVDLVISLNIPFET

                          120       130       140       150       160       170
P69441_ESCHERICHIA_COLI  IVDRIVGRRVHAPSGRVYHVKFNPPKVEGKDDVTGEELTTRKDDQEETVRKRLVEYHQMT
P27144_HOMO_SAPIENS      LKDRLSRRWIHPPSGRVYNLDFNPPHVHGIDDVTGEPLVQQEDDKPEAVAARLRQYKDVA

                          180          190                 200       210
P69441_ESCHERICHIA_COLI  APLIGYYSKEA...EAGNTKYAKV.........DGTKPVAEVRADLEKILG
P27144_HOMO_SAPIENS      KPVIELYKSRGVLHQFSGTETNKIWPYVYTLFSNKITPIQSKEAY......
```

```
                              1         10        20        30        40        50
P69441_ESCHERICHIA_COLI       MR...IILLGAPGAGKGTQAQFIMEKYGIPQISTGDMLRAAVKS.GSELGKQAKDIMDAG
P30085_HOMO_SAPIENS           MKPLVVFVLGGPGAGKGTQCARIVEKYGYTHLSAGELLRDERKNPDSQYGELIEKYIKEG

                                60        70        80        90        100
P69441_ESCHERICHIA_COLI       KLVTDELVIALVKERIAQEDC....RNGFLLDGFPRTIPQADAMK...EAGINVDYVLEF
P30085_HOMO_SAPIENS           KIVPVEITISLLKREMDQTMAANAQKNKFLIDGFPRNQDNLQGWNKTMDGKADVSFVLFF

                            110       120       130       140       150       160
P69441_ESCHERICHIA_COLI       DVPDELIVDRIVGRRVHAPSGRVYHVKFNPPKVEGKDDVTGEELTTRKDDQEETVRKRLV
P30085_HOMO_SAPIENS           DCNNEICIERCLER...................GKS.......SGRSDDNRESLEKRIQ

                            170       180       190       200       210
P69441_ESCHERICHIA_COLI       EYHQMTAPLIGYYSKEAEAGNTKYAKVDGTKPVAEVRADLEKIL...G
P30085_HOMO_SAPIENS           TYLQSTKPIIDLYE...EMGKVK..KIDASKSVDEVFDEVVQIFDKEG
```

```
                              1        10        20        30        40        50
P69441_ESCHERICHIA_COLI  .....MRIILLGAPGAGKGTQAQFIMEKYGIPQISTGDMLRAAVKSGSELGKQAKDIMDA
P43188_ZEA_MAYS          ALADPLKVMISGAPASGKGTQCELIKTKYQLAHISAGDLLRAEIAAGSENGKRAKEFMEK

                            60        70        80        90       100       110
P69441_ESCHERICHIA_COLI  GKLVTDELVIALVKERIAQEDCR.NGFLLDGFPRTIPQADAMKEAGINVDYVLEFDVPDE
P43188_ZEA_MAYS          GQLVPDEIVVNMVKERLRQPDAQENGWLLDGYPRSYSQAMALETLEIRPDTFILLDVPDE

                           120       130       140       150       160       170
P69441_ESCHERICHIA_COLI  LIVDRIVGRRVHAPSGRVYHVKFNPPKVEGKDDVTGEELTTRKDDQEETVRKRLVEYHQM
P43188_ZEA_MAYS          LLVERVVGRRLDPVTGKIYHLKYSPPE....NEEIASRLTQRFDDTEEKVKLRLETYYQN

                           180       190       200       210
P69441_ESCHERICHIA_COLI  TAPLIGYYSKEAEAGNTKYAKVDGTKPVAEVRADLEKILG.............
P43188_ZEA_MAYS          IESLLSTYENII.......VKVQGDATVDAVFAKIDELLGSILEKKNEMVSST
```

```
                                          1        10        20        30        40
P69441_ESCHERICHIA_COLI    ...............MRIILLGAPGAGKGTQAQFIMEKYGIPQISTGDMLRAAVKSGSEL
P54819_HOMO_SAPIENS        MAPSVPAAEPEYPKGIRAVLLGPPGAGKGTQAPRLAENFCVCHLATGDMLRAMVASGSEL

                                50        60        70        80        90         100
P69441_ESCHERICHIA_COLI    GKQAKDIMDAGKLVTDELVIALVKERIAQEDCRNGFLLDGFPRTIPQA....DAMKEAGI
P54819_HOMO_SAPIENS        GKKLKATMDAGKLVSDEMVVELIEKNLETPLCKNGFLLDGFPRTVRQAEMLDDLMEKRKE

                                     110       120       130       140       150       160
P69441_ESCHERICHIA_COLI    NVDYVLEFDVPDELIVDRIVGRRVHAPSGRVYHVKFNPPKVEGKDDVTGEELTTRKDDQE
P54819_HOMO_SAPIENS        KLDSVIEFSIPDSLLIRRITGRLIHPKSGRSYHEEFNPPKEPMKDDITGEPLIRRSDDNE

                                     170       180                 190       200       210
P69441_ESCHERICHIA_COLI    ETVRKRLVEYHQMTAPLIGYYSKE........AEAGNTKYAKVDGTKPVAEVRADLEKIL
P54819_HOMO_SAPIENS        KALKIRLQAYHTQTTPLIEYYRKRGIHSAIDASQTPDVVFASILAAFSKATCK.DLVMFI
```

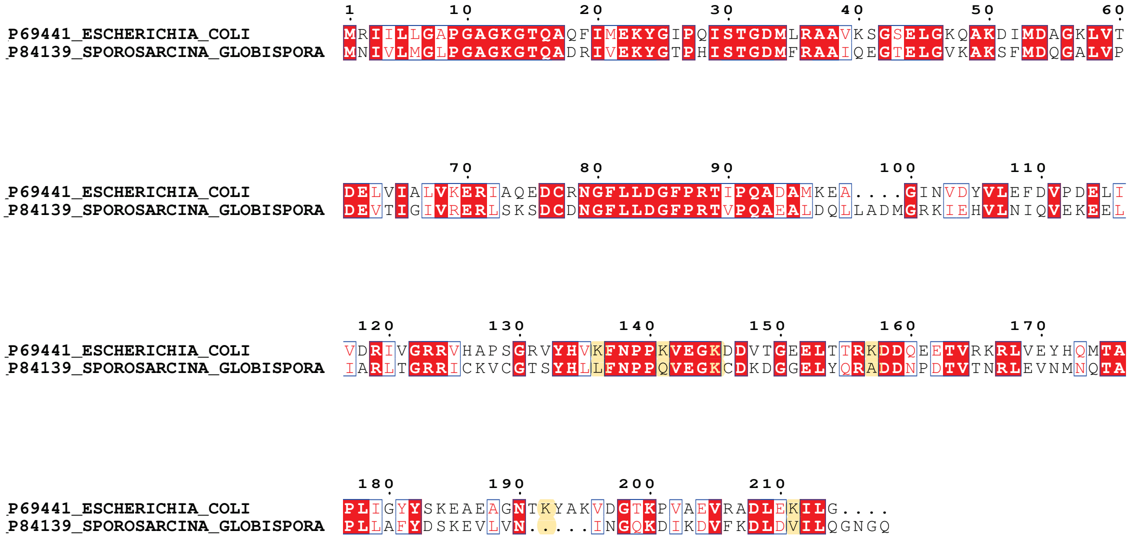
1 10 20 30 40 50 60
P69441_ESCHERICHIA_COLI MRIILLGAPGAGKGTQAQFIMEKYGIPQISTGDMLRAAVKSGSELGKQAKDIMDAGKLVT
P84139_SPOROSARCINA_GLOBISPORA MNIVLMGLPGAGKGTQADRIVEKYGTPHISTGDMFRAAIQEGTELGVKAKSFMDQGALVP
70 80 90 100 110
P69441_ESCHERICHIA_COLI DELVIALVKERIAQEDCRNGFLLDGFPRTIPQAADAMKEA....GINVDYVLEFDVPDELI
P84139_SPOROSARCINA_GLOBISPORA DEVTIGIVRERLSKSDCDNGFLLDGFPRTVPQAEALDQLLADMGRKIEHVLNIQVEKEEL
120 130 140 150 160 170
P69441_ESCHERICHIA_COLI VDRIVGRRVHAPSGRVYHVKFNPPKVEGKDDVTGEELTTRKDDQEETVRKRLVEYHQMTA
P84139_SPOROSARCINA_GLOBISPORA IARLTGRRICKVCGTSYHLLFNPPQVEGKCDKDGGELYQRADDNPDTVTNRLEVNMNQTA
180 190 200 210
P69441_ESCHERICHIA_COLI PLIGYYSKEAEAGNTKYAKVDGTKPVAEVRADLEKILG....
P84139_SPOROSARCINA_GLOBISPORA PLLAFYDSKEVLVN.....INGQKDIKDVFKDLDVILQGNGQ

```
                                     1        10        20        30        40        50        60
P69441_ESCHERICHIA_COLI              MRIILLGAPGAGKGTQAQFIMEKYGIPQISTGDMLRAAVKSGSELGKQAKDIMDAGKLVT
P9WKF5_MYCOBACTERIUM_TUBERCULOSIS    MRVLLLGPPGAGKGTQAVKLAEKLGIPQISTGELFRRNIEEGTKLGVEAKRYLDAGDLVP

                                               70        80        90       100       110
P69441_ESCHERICHIA_COLI              DELVIALVKERIAQEDCRNGFLLDGFPRTIPQADAMKEA....GINVDYVLEFDVPDELI
P9WKF5_MYCOBACTERIUM_TUBERCULOSIS    SDLTNELVDDRLNNPDAANGFILDGYPRSVEQAKALHEMLERRGTDIDAVLEFRVSEEVL

                                       120       130       140       150       160       170
P69441_ESCHERICHIA_COLI              VDRIVGRRVHAPSGRVYHVKFNPPKVEGKDDVTGEELTTRKDDQEETVRKRLVEYHQMTA
P9WKF5_MYCOBACTERIUM_TUBERCULOSIS    LERLKGRG................................RADDTDDVILNRMKVYRDETA

                                       180       190       200       210
P69441_ESCHERICHIA_COLI              PLIGYYSKEAEAGNTKYAKVDGTKPVAEVRADLEKILG.
P9WKF5_MYCOBACTERIUM_TUBERCULOSIS    PLLEYYRDQLKT.......VDAVGTMDEVFARALRALGK
```

```
                                    1         10        20        30        40        50        60
P69441_ESCHERICHIA_COLI             MRIILLGAPGAGKGTQAQFIMEKYGIPQISTGDMLRAAVKSGSELGKQAKDIMDAGKLVT
Q04ML5_STREPTOCOCCUS_PNEUMONIAE     MNLLIMGLPGAGKGTQAAKIVEQFHVAHISTGDMFRAAMANQTEMGVLAKSYIDKGELVP

                                              70        80        90        100       110
P69441_ESCHERICHIA_COLI             DELVIALVKERIAQEDCRN.GFLLDGFPRTIPQADAMK....EAGINVDYVLEFDVPDEL
Q04ML5_STREPTOCOCCUS_PNEUMONIAE     DEVTNGIVKERLSQDDIKETGFLLDGYPRTIEQAHALDKTLAELGIELEGIINIEVNPDS

                                      120       130       140       150       160       170
P69441_ESCHERICHIA_COLI             IVDRIVGRRVHAPSGRVYHVKFNPPKVEGKDDVTGEELTTRKDDQEETVRKRLVEYHQMT
Q04ML5_STREPTOCOCCUS_PNEUMONIAE     LLERLSGRIIHRVTGETFHKVFNPPV.....DYKEEDYYQREDDKPETVKRRLDVNIAQG

                                      180       190       200       210
P69441_ESCHERICHIA_COLI             APLIGYYSKEAEAGNTKYAKVDGTKPVAEVRADLEKILG...
Q04ML5_STREPTOCOCCUS_PNEUMONIAE     EPIIAHYRAKGLVHD.....IEGNQDINDVFSDIEKVLTNLK
```

```
                                     1        10        20        30        40        50        60
P69441_ESCHERICHIA_COLI              MRIILLGAPGAGKGTQAQFIMEKYGIPQISTGDMLRAAVKSGSELGKQAKDIMDAGKLVT
Q3JVB1_BURKHOLDERIA_PSEUDOMALLEI     MRLILLGAPGAGKGTQANFIKEKFGIPQISTGDMLRAAVKAGTPLGVEAKTYMDEGKLVP

                                              70        80        90       100       110       120
P69441_ESCHERICHIA_COLI              DELVIALVKERIAQEDCRNGFLLDGFPRTIPQADAMKEAGINVDYVLEFDVPDELIVDRI
Q3JVB1_BURKHOLDERIA_PSEUDOMALLEI     DSLIIGLVKERLKEADCANGYLFDGFPRTIAQADAMKEAGVAIDYVLEIDVPFSEIIERM

                                             130       140       150       160       170       180
P69441_ESCHERICHIA_COLI              VGRRVHAPSGRVYHVKFNPPKVEGKDDVTGEELTTRKDDQEETVRKRLVEYHQMTAPLIG
Q3JVB1_BURKHOLDERIA_PSEUDOMALLEI     SGRRTHPASGRTYHVKFNPPKVEGKDDVTGEPLVQRDDDKEETVKKRLDVYEAQTKPLIT

                                             190                 200       210
P69441_ESCHERICHIA_COLI              YYSKEAEAGNTK......YAKVDGTKPVAEVRADLEKILG
Q3JVB1_BURKHOLDERIA_PSEUDOMALLEI     YYGDWARRGAENGLKAPAYRKISGLGAVEEIRARVFDALK
```

```
                                      1        10        20        30        40        50
P69441_ESCHERICHIA_COLI        .......MRIILLGAPGAGKGTQAQFIMEKYGIPQISTGDMLRAAVKSGSELGKQAKDIM
Q5CRC5_CRYPTOSPORIDIUM_PARVUM  KMSNSKKHNLILIGAPGSGKGTQCEFIKKEYGLAHLSTGDMLREAIKNGTKIGLEAKSII

                                    60        70        80        90          100
P69441_ESCHERICHIA_COLI        DAGKLVTDELVIALVKERIAQEDCRNGFLLDGFPRTIPQADAM....KEAGINVDYVLEF
Q5CRC5_CRYPTOSPORIDIUM_PARVUM  ESGNFVGDEIVLGLVKEKFDLGVCVNGFVLDGFPRTIPQAEGLAKILSEIGDSLTSVIYF

                                110       120       130       140       150       160
P69441_ESCHERICHIA_COLI        DVPDELIVDRIVGRRVHAPSGRVYHVKFNPPKVEGKDDVTGEELTTRKDDQEETVRKRLV
Q5CRC5_CRYPTOSPORIDIUM_PARVUM  EIDDSEIIERISGRCTHPASGRIYHVKYNPPKQPGIDDVTGEPLVWRDDDNAEAVKVRLD

                                170       180       190       200       210
P69441_ESCHERICHIA_COLI        EYHQMTAPLIGYYSKEAEAGNTKYAKVDGTKPVAEVRADLEKILG.
Q5CRC5_CRYPTOSPORIDIUM_PARVUM  VFHKQTAPLVKFYE...DLGILK..RVNAKLPPKEVTEQIKKILEN
```

```
                                1        10        20        30        40        50        60
P69441_ESCHERICHIA_COLI         MRIILLGAPGAGKGTQAQFIMEKYGIPQISTGDMLRAAVKSGSELGKQAKDIMDAGKLVT
Q5NFR4_FRANCISELLA_TULARENSIS   MRIILLGAPGAGKGTQAKIIEQKYNIAHISTGDMIRETIKSGSALGQELKKVLDAGELVS

                                         70        80        90       100       110       120
P69441_ESCHERICHIA_COLI         DELVIALVKERIAQEDCRNGFLLDGFPRTIPQADAMKEAGINVDYVLEFDVPDELIVDRI
Q5NFR4_FRANCISELLA_TULARENSIS   DEFIIKIVKDRISKNDCNNGFLLDGVPRTIPQAQELDKLGVNIDYIVEVDVADNLLIERI

                                        130       140       150       160       170       180
P69441_ESCHERICHIA_COLI         VGRRVHAPSGRVYHVKFNPPKVEGKDDVTGEELTTRKDDQEETVRKRLVEYHQMTAPLIG
Q5NFR4_FRANCISELLA_TULARENSIS   TGRRIHPASGRTYHTKFNPPKVADKDDVTGEPLITRTDDNEDTVKQRLSVYHAQTAKLID

                                        190          200       210
P69441_ESCHERICHIA_COLI         YYSKEAEAGNTK...YAKVDGTKPVAEVRADLEKILG..
Q5NFR4_FRANCISELLA_TULARENSIS   FY.RNFSSTNTKIPKYIKINGDQAVEKVSQDIFDQLNKR
```

```
                                     1        10        20        30        40        50
P69441_ESCHERICHIA_COLI         ....MRIILLGAPGAGKGTQAQFIMEKYGIPQISTGDMLRAAVKSGSELGKQAKDIMDAG
Q5SHQ9_THERMUS_THERMOPHILUS     MDVGQAVIFLGPPGAGKGTQASRLAQELGFKKLSTGDILRDHVARGTPLGERVRPIMERG

                                  60        70        80        90           100       110
P69441_ESCHERICHIA_COLI         KLVTDELVIALVKERIAQEDCRNGFLLDGFPRTIPQADAM....KEAGINVDYVLEFDVP
Q5SHQ9_THERMUS_THERMOPHILUS     DLVPDDLILELIREELAER.....VIFDGFPRTLAQAEALDRLLSETGTRLLGVVLVEVP

                                     120       130       140       150       160       170
P69441_ESCHERICHIA_COLI         DELIVDRIVGRRVHAPSGRVYHVKFNPPKVEGKDDVTGEELTTRKDDQEETVRKRLVEYH
Q5SHQ9_THERMUS_THERMOPHILUS     EEELVRRILRRA.........................ELEGRSDDNEETVRRRLEVYR

                                     180       190       200       210
P69441_ESCHERICHIA_COLI         QMTAPLIGYYSKEAEAGNTKYAKVDGTKPVAEVRADLEKILG.
Q5SHQ9_THERMUS_THERMOPHILUS     EKTEPLVGYYEAR...GVLK..RVDGLGTPDEVYARIRAALGI
```

```
                              1        10        20        30        40        50
P69441_ESCHERICHIA_COLI       MR........IILLGAPGAGKGTQAQFIMEKYGIPQISTGDMLRAAVKSGSELGKQAKDI
Q68EH2_DANIO_RERIO            MADKIKNAKIVFVVGGPGSGKGTQCEKIVAKYGYTHLSSGDLLRAEVASGSERGKQLQAI

                                      60        70        80        90       100       110
P69441_ESCHERICHIA_COLI       MDAGKLVTDELVIALVKER.IAQEDCRNGFLLDGFPRTIPQADAMKEAGINVDYVLEFDV
Q68EH2_DANIO_RERIO            MQKGELVPLDTVLDMIKDAMIAKADVSKGYLIDGYPREVKQGEEFEKKIGAPALLLYIDA

                                     120       130       140       150       160       170
P69441_ESCHERICHIA_COLI       PDELIVDRIVGRRVHAPSGRVYHVKFNPPKVEGKDDVTGEELTTRKDDQEETVRKRLVEY
Q68EH2_DANIO_RERIO            KGETMVKRLMKRG.............................ETSGRADDNEETIKKRLDLY

                                     180       190       200       210
P69441_ESCHERICHIA_COLI       HQMTAPLIGYYSKEAEAGNTKYAKVDGTKPVAEVRADLEKILG...
Q68EH2_DANIO_RERIO            YKATEPVIAFYEQRGIV.....RKINSELPVDEVFAIVEKAIDELK
```

```
                                  1        10        20        30        40        50        60
P69441_ESCHERICHIA_COLI           MRIILLGAPGAGKGTQAQFIMEKYGIPQISTGDMLRAAVKSGSELGKQAKDIMDAGKLVT
Q6B341_JEOTGALIBACILLUS_MARINUS   MNIVLMGLPGAGKGTQAEQIIEKYEIPHISTGDMFRAAIKNGTELGLKAKSFMDQGNLVP

                                            70        80        90           100       110
P69441_ESCHERICHIA_COLI           DELVIALVKERIAQEDCRNGFLLDGFPRTIPQADAMK....EAGINVDYVLEFDVPDELI
Q6B341_JEOTGALIBACILLUS_MARINUS   DEVTIGIVHERLSKDDCQKGFLLDGFPRTVAQADALDSLLTDLGKKLDYVLNIKVEQEEL

                                   120       130       140       150       160       170
P69441_ESCHERICHIA_COLI           VDRIVGRRVHAPSGRVYHVKFNPPKVEGKDDVTGEELTTRKDDQEETVRKRLVEYHQMTA
Q6B341_JEOTGALIBACILLUS_MARINUS   MKRLTGRWICKTCGATYHTIFNPPAVEGICDKDGGELYQRIDDKPETVKNRLDVNMKQTQ

                                   180       190       200       210
P69441_ESCHERICHIA_COLI           PLIGYYSKEAEAGNTKYAKVDGTKPVAEVRADLEKILG...
Q6B341_JEOTGALIBACILLUS_MARINUS   PLLDFYSQKGVLKD.....IDGQQDIKKVFVDINDLLGGLR
```

```
                                  1        10        20        30        40        50        60
P69441_ESCHERICHIA_COLI           MRIILLGAPGAGKGTQAQFIMEKYGIPQISTGDMLRAAVKSGSELGKQAKDIMDAGKLVT
Q6LTE1_PHOTOBACTERIUM_PROFUNDUM   MRIILLGAPGAGKGTQAQFIMAKFGIPQISTGDMLRAAIKAGTELGKQAKSVIDAGQLVS

                                          70        80        90       100       110       120
P69441_ESCHERICHIA_COLI           DELVIALVKERIAQEDCRNGFLLDGFPRTIPQADAMKEAGINVDYVLEFDVPDELIVDRI
Q6LTE1_PHOTOBACTERIUM_PROFUNDUM   DDIILGLVKERIAQDDCAKGFLLDGFPRTIPQADGLKEVGVVVDYVIEFDVADSVIVERM

                                         130       140       150       160       170       180
P69441_ESCHERICHIA_COLI           VGRRVHAPSGRVYHVKFNPPKVEGKDDVTGEELTTRKDDQEETVRKRLVEYHQMTAPLIG
Q6LTE1_PHOTOBACTERIUM_PROFUNDUM   AGRRAHLASGRTYHNVYNPPKVEGKDDVTGEDLVIREDDKEETVLARLGVYHNQTAPLIA

                                         190       200       210
P69441_ESCHERICHIA_COLI           YYSKEAEAGNTKYAKVDGTKPVAEVRADLEKILG
Q6LTE1_PHOTOBACTERIUM_PROFUNDUM   YYGKEAEAGNTQYLKFDGTKAVAEVSAELEKALA
```

```
                                 1                                       10        20        30
P69441_ESCHERICHIA_COLI          M............................RIILLGAPGAGKGTQAQFIMEKYGIPQISTG
Q7Z0H0_PLASMODIUM_FALCIPARUM     MNENLENFSTIDLLNELKRRYACLSKPDGRYIFLGAPGSGKGTQSLNLKKSHCYCHLSTG

                                      40        50        60        70        80        90
P69441_ESCHERICHIA_COLI          DMLRAAVKSGSELGKQAKDIMDAGKLVTDELVIALVKERIAQEDCRNGFLLDGFPRTIPQ
Q7Z0H0_PLASMODIUM_FALCIPARUM     DLLREAAEKKTELGLKIKNIINEGKLVDDQMVLSLVDEKLKTPQCKKGFILDGYPRNVKQ

                                         100       110       120       130       140
P69441_ESCHERICHIA_COLI          AD....AMKEAGINVDYVLEFDVPDELIVDRIVGRRVHAPSGRVYHVKFNPPKVEGKDDV
Q7Z0H0_PLASMODIUM_FALCIPARUM     AEDLNKLLQKNQTKLDGVFYFNVPDEVLVNRISGRLIHKPSGRIYHKIFNPPKVPFRDDV

                                150       160       170       180       190       200
P69441_ESCHERICHIA_COLI          TGEELTTRKDDQEETVRKRLVEYHQMTAPLIGYYSKEAEAGNTKYAKVDGTKPVAEVRAD
Q7Z0H0_PLASMODIUM_FALCIPARUM     TNEPLIQREDDNEDVLKKRLTVFKSETSPLISYYKNKNLLIN.....LDATQPAN....D

                                210
P69441_ESCHERICHIA_COLI          LEK.....ILG
Q7Z0H0_PLASMODIUM_FALCIPARUM     LEKKISQHIDG
```

```
                         1       10        20        30        40        50        60
P69441_ESCHERICHIA_COLI  MRIILLGAPGAGKGTQAQFIMEKYGIPQISTGDMLRAAVKSGSELGKQAKDIMDAGKLVT
Q9KTB7_VIBRIO_CHOLERAE   MRIILLGAPGAGKGTQAQFIMEKFGIPQISTGDMLRAAIKAGTELGKQAKAVIDAGQLVS

                                 70        80        90       100       110       120
P69441_ESCHERICHIA_COLI  DELVIALVKERIAQEDCRNGFLLDGFPRTIPQADAMKEAGINVDYVLEFDVPDELIVDRI
Q9KTB7_VIBRIO_CHOLERAE   DDIILGLIKERIAQADCEKGFLLDGFPRTIPQADGLKEMGINVDYVIEFDVADDVIVERM

                                130       140       150       160       170       180
P69441_ESCHERICHIA_COLI  VGRRVHAPSGRVYHVKFNPPKVEGKDDVTGEELTTRKDDQEETVRKRLVEYHQMTAPLIG
Q9KTB7_VIBRIO_CHOLERAE   AGRRAHLPSGRTYHVVYNPPKVEGKDDVTGEDLVIREDDKEETVRARLNVYHTQTAPLIE

                                190       200       210
P69441_ESCHERICHIA_COLI  YYSKEAEAGNTKYAKVDGTKPVAEVRADLEKILG
Q9KTB7_VIBRIO_CHOLERAE   YYGKEAAAGKTQYLKFDGTKQVSEVSADIAKALA
```

```
                          1        10        20        30        40        50
P69441_ESCHERICHIA_COLI   .......MRIILLGAPGAGKGTQAQFIMEKYGIPQISTGDMLRAAVKSGSELGKQAKDIM
Q9UIJ7_HOMO_SAPIENS       MGASARLLRAVIMGAPGSGKGTVSSRITTHFELKHLSSGDLLRDNMLRGTEIGVLAKAFI

                        60        70            80        90       100
P69441_ESCHERICHIA_COLI   DAGKLVTDELVIALVKERIAQEDCRN....GFLLDGFPRTIPQADAMKEAGINVDYVLEF
Q9UIJ7_HOMO_SAPIENS       DQGKLIPDD.....VMTRLALHELKNLTQYSWLLDGFPRTLPQAEALDRA.YQIDTVINL

                       110       120       130       140       150       160
P69441_ESCHERICHIA_COLI   DVPDELIVDRIVGRRVHAPSGRVYHVKFNPPKVEGKDDVTGEELTTRKDDQEETVRKRLV
Q9UIJ7_HOMO_SAPIENS       NVPFEVIKQRLTARWIHPASGRVYNIEFNPPKTVGIDDLTGEPLIQREDDKPETVIKRLK

                       170       180       190                 200       210
P69441_ESCHERICHIA_COLI   EYHQMTAPLIGYYSKEAEAGNTK............YAKVDGTKPVAEVRADLEKILG
Q9UIJ7_HOMO_SAPIENS       AYEDQTKPVLEYYQKKGVLETFSGTETNKIWPYVYAFLQTKVPQRSQKASVTP...
```

```
P69441_ESCHERICHIA_COLI    ............................................................
Q9Y6K8_HOMO_SAPIENS        MNTNDAKEYLARREIPQLFESLLNGLMCSKPEDPVEYLESCLQKVKELGGCDKVKWDTFV

P69441_ESCHERICHIA_COLI    ............................................................
Q9Y6K8_HOMO_SAPIENS        SQEKKTLPPLNGGQSRRSFLRNVMPENSNFPYRRYDRLPPIHQFSIESDTDLSETAELIE

P69441_ESCHERICHIA_COLI    ............................................................
Q9Y6K8_HOMO_SAPIENS        EYEVFDPTRPRPKIILVIGGPGSGKGTQSLKIAERYGFQYISVGELLRKKIHSTSSNRKW

P69441_ESCHERICHIA_COLI    ............................................................
Q9Y6K8_HOMO_SAPIENS        SLIAKIITTGELAPQETTITEIKQKLMQIPDEEGIVIDGFPRDVAQALSFEDQICTPDLV

P69441_ESCHERICHIA_COLI    ............................................................
Q9Y6K8_HOMO_SAPIENS        VFLACANQRLKERLLKRAEQQGRPDDNVKATQRRLMNFKQNAAPLVKYFQEKGLIMTFDA

P69441_ESCHERICHIA_COLI    ............................................................
Q9Y6K8_HOMO_SAPIENS        DRDEDEVFYDISMAVDNKLFPNKEAAAGSSDLDPSMILDTGEIIDTGSDYEDQGDDQLNV

                                           1         10        20        30        40
P69441_ESCHERICHIA_COLI    ................MRIILLGAPGAGKGTQAQFIMEKYGIPQISTGDMLRAAVKSGSE
Q9Y6K8_HOMO_SAPIENS        FGEDTMGGFMEDLRKCKIIFIIGGPGSGKGTQCEKLVEKYGFTHLSTGELLREELASESE

                                 50        60        70        80        90        100
P69441_ESCHERICHIA_COLI    LGKQAKDIMDAGKLVTDELVIALVKER.IAQEDCRNGFLLDGFPRTIPQADAMKEAGINV
Q9Y6K8_HOMO_SAPIENS        RSKLIRDIMERGDLVPSGIVLELLKEAMVASLGDTRGFLIDGYPREVKQGEEFGRRIGDP

                                 110       120       130       140       150       160
P69441_ESCHERICHIA_COLI    DYVLEFDVPDELIVDRIVGRRVHAPSGRVYHVKFNPPKVEGKDDVTGEELTTRKDDQEET
Q9Y6K8_HOMO_SAPIENS        QLVICMDCSADTMTNRLLQR...........................SRSSLPVDDTTKT

                                 170       180       190       200       210
P69441_ESCHERICHIA_COLI    VRKRLVEYHQMTAPLIGYYSKEAEAGNTKYAKVDGTKP..VAEVRADLEKILG
Q9Y6K8_HOMO_SAPIENS        IAKRLEAYYRASIPVIAYYETKTQLHKIN...AEGTPEDVFLQLCTAIDSIF.
```

```
                           1         10        20        30        40        50        60
P69441_ESCHERICHIA_COLI    MRIILLGAPGAGKGTQAQFIMEKYGIPQISTGDMLRAAVKSGSELGKQAKDIMDAGKLVT
ESCHERICHIA_COLI           MRIILLGAPGAGKGTQAQFIMEKYGIPQISTGDMLRAAVKSGSELGKQAKDIMDAGKLVT

                                     70        80        90       100       110       120
P69441_ESCHERICHIA_COLI    DELVIALVKERIAQEDCRNGFLLDGFPRTIPQADAMKEAGINVDYVLEFDVPDELIVDRI
ESCHERICHIA_COLI           DELVIALVKERIAQEDCRNGFLLDGFPRTIPQADAMKEAGINVDYVLEFDVPDELIVDRI

                                    130       140       150       160       170       180
P69441_ESCHERICHIA_COLI    VGRRVHAPSGRVYHVKFNPPKVEGKDDVTGEELTTRKDDQEETVRKRLVEYHQMTAPLIG
ESCHERICHIA_COLI           VGRRVHAPSGRVYHVKFNPPKVEGKDDVTGEELTTRKDDQEETVRKRLVEYHQMTAPLIG

                                    190       200       210
P69441_ESCHERICHIA_COLI    YYSKEAEAGNTKYAKVDGTKPVAEVRADLEKILG
ESCHERICHIA_COLI           YYSKEAEAGNTKYAKVDGTKPVAEVRADLEKILG
```

```
                           1        10        20        30        40        50        60
P69441_ESCHERICHIA_COLI    MRIILLGAPGAGKGTQAQFIMEKYGIPQISTGDMLRAAVKSGSELGKQAKDIMDAGKLVT
SYNTHETIC_CONSTRUCT        MNLILLGPPGAGKGTQAEKIVEEYGIPHISTGDMFRAAIKEGTELGLKAKEYMDKGELVP

                                    70        80        90        100       110
P69441_ESCHERICHIA_COLI    DELVIALVKERIAQEDCRNGFLLDGFPRTIPQADAM....KEAGINVDYVLEFDVPDELI
SYNTHETIC_CONSTRUCT        DEVTIGLVKERLSQPDCKKGFLLDGFPRTVAQAEALDKILKELGIKLDAVINIEVPREEL

                            120       130       140       150       160       170
P69441_ESCHERICHIA_COLI    VDRIVGRRVHAPSGRVYHVKFNPPKVEGKDDVTGEELTTRKDDQEETVRKRLVEYHQMTA
SYNTHETIC_CONSTRUCT        LERLTGRRVCRQCGATYHVIFNPPKVEGVCDKCGGELYQRSDDNEETVSNRLDVYEDQTA

                            180       190       200       210
P69441_ESCHERICHIA_COLI    PLIGYYSKEAEAGNTKYAKVDGTKPVAEVRADLEKILG.............
SYNTHETIC_CONSTRUCT        PLIDYYEKKGLLKN.....IDGDQDIDAVFADIKAALGRDKQGGGENLYFQ
```

```
                          1        10        20        30        40        50        60
P69441_ESCHERICHIA_COLI   MRIILLGAPGAGKGTQAQFIMEKYGIPQISTGDMLRAAVKSGSELGKQAKDIMDAGKLVT
SYNTHETIC_CONSTRUCT       MNLVLMGLPGAGKGTQAEKIVEKYGIPHISTGDMFRAAIKEGTELGLKAKSFMDKGELVP

                                    70        80        90          100       110
P69441_ESCHERICHIA_COLI   DELVIALVKERIAQEDCRNGFLLDGFPRTIPQADAM....KEAGINVDYVLEFDVPDELI
SYNTHETIC_CONSTRUCT       DEVTIGIVRERLSKDDCKKGFLLDGFPRTVAQAEALDNILKELGKKLDYVINIEVPKEEL

                           120       130       140       150       160       170
P69441_ESCHERICHIA_COLI   VDRIVGRRVHAPSGRVYHVKFNPPKVEGKDDVTGEELTTRKDDQEETVRKRLVEYHQMTA
SYNTHETIC_CONSTRUCT       MERLTGRRICKTCGATYHLIFNPPKVEGVCDKCGGELYQRADDNEETVANRLDVNMKQTQ

                           180       190       200       210
P69441_ESCHERICHIA_COLI   PLIGYYSKEAEAGNTKYAKVDGTKPVAEVRADLEKILG....
SYNTHETIC_CONSTRUCT       PLLDFYEEKGYLRN.....IDGQQDINKVFADIDALLGGLKQ
```

```
                         1        10        20        30        40        50        60
P69441_ESCHERICHIA_COLI  MRIILLGAPGAGKGTQAQFIMEKYGIPQISTGDMLRAAVKSGSELGKQAKDIMDAGKLVT
SYNTHETIC_CONSTRUCT      MNLVLMGLPGAGKGTQAEKIVEKYGIPHISTGDMFRAAIKEGTELGLKAKSFMDKGELVP

                                  70        80        90         100       110
P69441_ESCHERICHIA_COLI  DELVIALVKERIAQEDCRNGFLLDGFPRTIPQADAM....KEAGINVDYVLEFDVPDELI
SYNTHETIC_CONSTRUCT      DEVTIGIVRERLSKDDCKKGFLLDGFPRTVAQAEALDNILSELGKKLDYVINIEVPKEEL

                           120       130       140       150       160       170
P69441_ESCHERICHIA_COLI  VDRIVGRRVHAPSGRVYHVKFNPPKVEGKDDVTGEELTTRKDDQEETVRKRLVEYHQMTA
SYNTHETIC_CONSTRUCT      MERLTGRRICKTCGATYHLIFNPPKVEGICDKDGGELYQRADDNPETVANRLDVNMKQTQ

                           180       190       200       210
P69441_ESCHERICHIA_COLI  PLIGYYSKEAEAGNTKYAKVDGTKPVAEVRADLEKILG..........
SYNTHETIC_CONSTRUCT      PLLDFYEEKGVLRN.....IDGQQDINKVFADIKALLGGLKQENLYFQ
```

```
                              1       10        20        30        40        50        60
P69441_ESCHERICHIA_COLI       MRIILLGAPGAGKGTQAQFIMEKYGIPQISTGDMLRAAVKSGSELGKQAKDIMDAGKLVT
SYNTHETIC_CONSTRUCT           MNLVLMGLPGAGKGTQAEKIVEKYGIPHISTGDMFRAAIKEGTELGLKAKSFMDKGELVP

                                      70        80        90       100       110
P69441_ESCHERICHIA_COLI       DELVIALVKERIAQEDCRNGFLLDGFPRTIPQADAM....KEAGINVDYVLEFDVPDELI
SYNTHETIC_CONSTRUCT           DEVTIGIVRERLSKDDCKKGFLLDGFPRTVAQAEALDNILSELGKKLDYVINIEVPKEEL

                               120       130       140       150       160       170
P69441_ESCHERICHIA_COLI       VDRIVGRRVHAPSGRVYHVKFNPPKVEGKDDVTGEELTTRKDDQEETVRKRLVEYHQMTA
SYNTHETIC_CONSTRUCT           MERLTGRRICKTCGATYHLIFNPPKVEGICDKDGGELYQRADDNPETVANRLDVNMKQTQ

                               180       190       200       210
P69441_ESCHERICHIA_COLI       PLIGYYSKEAEAGNTKYAKVDGTKPVAEVRADLEKILG..........
SYNTHETIC_CONSTRUCT           PLLDFYEEKGVLRN.....IDGQQDINKVFADIKALLGGLKQENLYFQ
```

Supplemental Figure SF5B. Pairwise sequence alignments between the *E. coli* substrate protein target (Icd-isocitrate dehydrogenase) and homologs sorted by UniProt ID. Labels adjacent to each sequence include PDB ID, UniProt ID, and organism name. Lysine residues previously identified as acetylated in the target protein are highlighted in yellow to examine conservation in 1D.

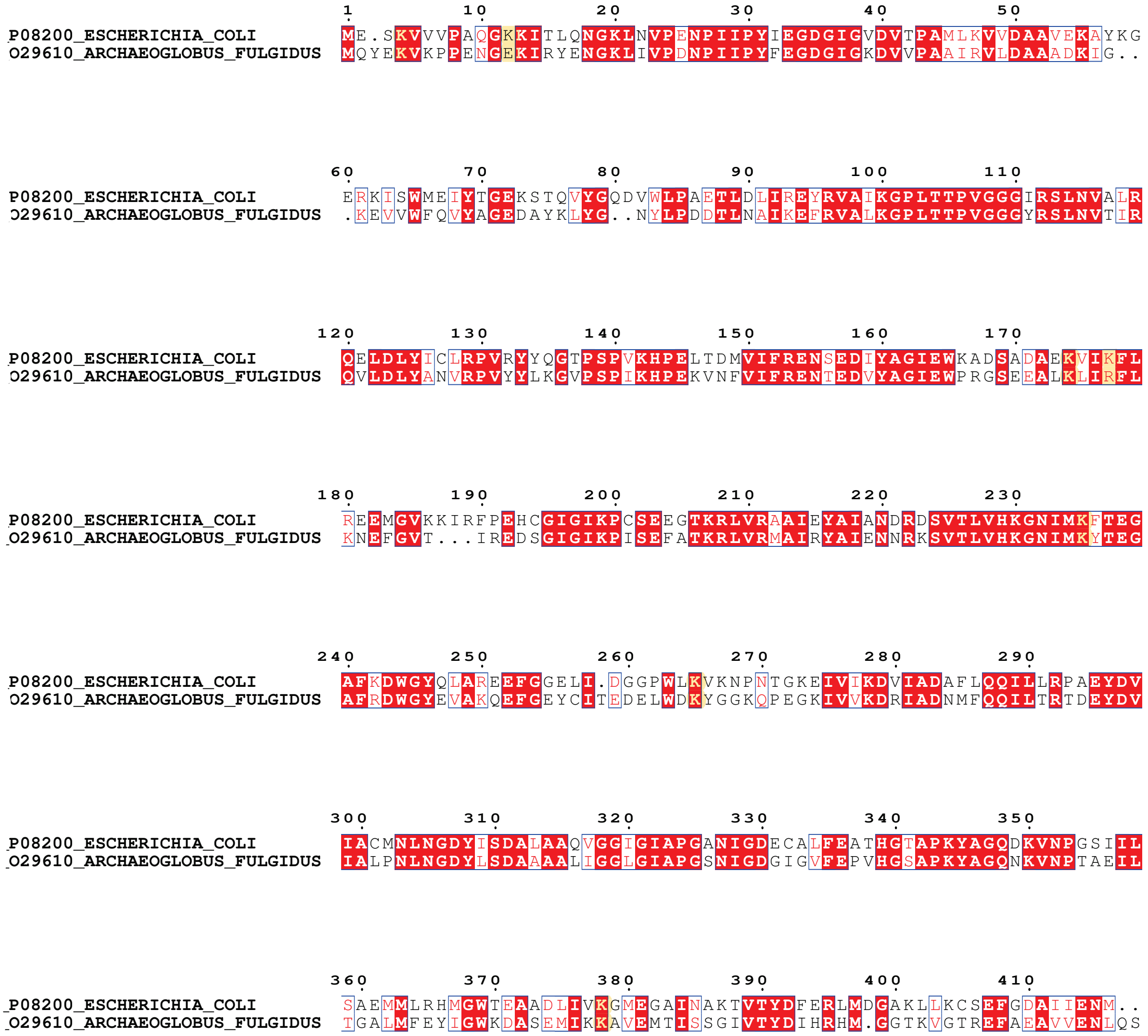
1 10 20 30 40 50
P08200_ESCHERICHIA_COLI ME.SKVVVPAQGKKITLQNGKLNVPENPIIPYIEGDGIGVDVTPAMLKVVDAAVEKAYKG
O29610_ARCHAEOGLOBUS_FULGIDUS MQYEKVKPPENGEKIRYENGKLIVPDNPIIPYFEGDGIGKDVVPAAIRVLDAAADKIG..
60 70 80 90 100 110
P08200_ESCHERICHIA_COLI ERKISWMEIYTGEKSTQVYGQDVWLPAETLDLIREYRVAIKGPLTTPVGGGIRSLNVALR
O29610_ARCHAEOGLOBUS_FULGIDUS .KEVVWFQVYAGEDAYKLYG..NYLPDDTLNAIKEFRVALKGPLTTPVGGGYRSLNVTIR
120 130 140 150 160 170
P08200_ESCHERICHIA_COLI QELDLYICLRPVRYYQGTPSPVKHPELTDMVIFRENSEDIYAGIEWKADSADAEKVIKFL
O29610_ARCHAEOGLOBUS_FULGIDUS QVLDLYANVRPVYYLKGVPSPIKHPEKVNFVIFRENTEDVYAGIEWPRGSEEALKLIRFL
180 190 200 210 220 230
P08200_ESCHERICHIA_COLI REEMGVKKIRFPEHCGIGIKPCSEEGTKRLVRAAIEYAIANDRDSVTLVHKGNIMKFTEG
O29610_ARCHAEOGLOBUS_FULGIDUS KNEFGVT...IREDSGIGIKPISEFATKRLVRMAIRYAIENNRKSVTLVHKGNIMKYTEG
240 250 260 270 280 290
P08200_ESCHERICHIA_COLI AFKDWGYQLAREEFGGELI.DGGPWLKVKNPNTGKEIVIKDVIADAFLQQILLRPAEYDV
O29610_ARCHAEOGLOBUS_FULGIDUS AFRDWGYEVAKQEFGEYCITEDELWDKYGGKQPEGKIVVKDRIADNMFQQILTRTDEYDV
300 310 320 330 340 350
P08200_ESCHERICHIA_COLI IACMNLNGDYISDALAAQVGGIGIAPGANIGDECALFEATHGTAPKYAGQDKVNPGSIIL
O29610_ARCHAEOGLOBUS_FULGIDUS IALPNLNGDYLSDAAAALIGGLGIAPGSNIGDGIGVFEPVHGSAPKYAGQNKVNPTAEIL
360 370 380 390 400 410
P08200_ESCHERICHIA_COLI SAEMMLRHMGWTEAADLIVKGMEGAINAKTVTYDFERLMDGAKLLKCSEFGDAIIENM..
O29610_ARCHAEOGLOBUS_FULGIDUS TGALMFEYIGWKDASEMIKKAVEMTISSGIVTYDIHRHM.GGTKVGTREFAEAVVENLQS

```
                        1        10                  20        30
P08200_ESCHERICHIA_COLI MESKVVV............PAQGKKITL..........QNGKLNVPENPIIPYIEGDGIG
O43837_HOMO_SAPIENS     MAALSGVRWLTRALVSAGNPGAWRGLSTSAAAHAASRSQAEDVRVEGSFPVTMLPGDGVG

                         40        50        60        70        80        90
P08200_ESCHERICHIA_COLI VDVTPAMLKVVDAAVEKAYKGERKISWMEIYTGEKSTQVYGQDVWLPAETLDLIREYRVA
O43837_HOMO_SAPIENS     PELMHAVKEVFKAAAVPV.......EFQEHHLSEVQNMASEEKL...EQVLSSMKENKVA

                        100         110       120       130       140       150
P08200_ESCHERICHIA_COLI IKGPLTTPVG..GGIRSLNVALRQELDLYICLRPVRYYQGTPSPVKHPELTDMVIFRENS
O43837_HOMO_SAPIENS     IIGKIHTPMEYKGELASYDMRLRRKLDLFANVVHVKSLPGYMT..RHNNL.DLVIIREQT

                          160       170       180       190       200       210
P08200_ESCHERICHIA_COLI EDIYAGIEWKADSADAEKVIKFLREEMGVKKIRFPEHCGIGIKPCSEEGTKRLVRAAIEY
O43837_HOMO_SAPIENS     EGEYSSLE....HESARGVIECL...................KIVTRAKSQRIAKFAFDY

                          220       230       240       250       260       270
P08200_ESCHERICHIA_COLI AIANDRDSVTLVHKGNIMKFTEGAFKDWGYQLAREEFGGELIDGGPWLKVKNPNTGKEIV
O43837_HOMO_SAPIENS     ATKKGRGKVTAVHKANIMKLGD.GLFLQCCEEVAELY...................PKIK

                          280       290       300       310       320       330
P08200_ESCHERICHIA_COLI IKDVIADAFLQQILLRPAEYDVIACMNLNGDYISDALAAQVGGIGIAPGANIGDECALFE
O43837_HOMO_SAPIENS     FETMIIDNCCMQLVQNPYQFDVLVMPNLYGNIIDNLAAGLVGGAGVVPGESYSAEYAVFE

                            340       350       360       370       380       390
P08200_ESCHERICHIA_COLI ..ATHGTAPKYAGQDKVNPGSIILSAEMMLRHMGWTEAADLIVKGMEGAINAKTVTYDFE
O43837_HOMO_SAPIENS     TGARHPFAQAV.GRNIANPTAMLLSASNMLRHLNLEYHSSMIADAVKKVIKVGKV.....

                            400       410
P08200_ESCHERICHIA_COLI RLMDGAKLLKCSEFGDAIIENM.....
O43837_HOMO_SAPIENS     RTRDMGGYSTTTDFIKSVIGHLQTKGS
```

```
                                    1        10        20        30        40        50
P08200_ESCHERICHIA_COLI             M.........ESKVVVPAQGKKITLQNGKLNVPENP.IIPYIEGDGIGVDVTPAMLKVVD
P28241_SACCHAROMYCES_CEREVISIAE     MLRNTFFRNTSRRFLATVKQPSIGRYTGKPNPSTGKYTVSFIEGDGIGPEISKSVKKIFS

                                            60        70        80        90       100       110
P08200_ESCHERICHIA_COLI             AAVEKAYKGERKISWMEIYTGEKSTQVYGQDVWLPAETLDLIREYRVAIKGPLTTPVGGG
P28241_SACCHAROMYCES_CEREVISIAE     AAN.......VPIEWESCDVSPIFVNGL...TTIPDPAVQSITKNLVALKGPLATPIGKG

                                           120       130       140       150       160       170
P08200_ESCHERICHIA_COLI             IRSLNVALRQELDLYICLRPVRYYQGTPSPVKHPELTDMVIFRENSEDIYAGIEWKADSA
P28241_SACCHAROMYCES_CEREVISIAE     HRSLNLTLRKTFGLFANVRPAKSIEGFKTTYEN...VDLVLIRENTEGEYSGIEHIVCPG

                                           180       190       200       210       220       230
P08200_ESCHERICHIA_COLI             DAEKVIKFLREEMGVKKIRFPEHCGIGIKPCSEEGTKRLVRAAIEYAIANDRDSVTLVHK
P28241_SACCHAROMYCES_CEREVISIAE     .......................VVQSIKLITRDASERVIRYAFEYARAIGRPRVIVVHK

                                           240       250       260       270       280       290
P08200_ESCHERICHIA_COLI             GNIMKFTEGAFKDWGYQLAREEFGGELIDGGPWLKVKNPNTGKEIVIKDVIADAFLQQIL
P28241_SACCHAROMYCES_CEREVISIAE     STIQRLAD.GLFVNVAKELSKEY...................PDLTLETELIDNSVLKVV

                                               300       310        320       330       340
P08200_ESCHERICHIA_COLI             LRPAEYD..VIACMNLNGDYISDALAAQV.GGIGIAPGANIGDECALFEATHGTAPKYAG
P28241_SACCHAROMYCES_CEREVISIAE     TNPSAYTDAVSVCPNLYGDILSDLNSGLSAGSLGLTPSANIGHKISIFEAVHGSAPDIAG

                                    350       360       370       380         390       400
P08200_ESCHERICHIA_COLI             QDKVNPGSIILSAEMMLRHMGWTEAADLIVKGMEGAINAK..TVTYDFERLMDGAKLLKC
P28241_SACCHAROMYCES_CEREVISIAE     QDKANPTALLLSSVMMLNHMGLTNHADQIQNAVLSTIASGPENRTGDLAGTA......TT

                                       410
P08200_ESCHERICHIA_COLI             SEFGDAIIENM
P28241_SACCHAROMYCES_CEREVISIAE     SSFTEAVIKRL
```

```
                                 1        10        20            30        40        50
P08200_ESCHERICHIA_COLI          MESKVVVPAQGKKITLQNGKLNVPENP....IIPYIEGDGIGVDVTPAMLKVVDAAVEKA
P28834_SACCHAROMYCES_CEREVISIAE  MLNRTI..AKRTLATAAQAERTLPKKYGGRFTVTLIPGDGVGKEITDSVRTIFEAENI..

                                   60        70        80        90       100       110
P08200_ESCHERICHIA_COLI          YKGERKISWMEIYTGEKSTQVYGQDVWLPAETLDLIREYRVAIKGPLTTPVG.GGIRSLN
P28834_SACCHAROMYCES_CEREVISIAE  .....PIDWETINIKQTDHKEG......VYEAVESLKRNKIGLKGLWHTPADQTGHGSLN

                                   120       130       140       150       160       170
P08200_ESCHERICHIA_COLI          VALRQELDLYICLRPVRYYQGTPSPVKHPELTDMVIFRENSEDIYAGIEWKADSADAEKV
P28834_SACCHAROMYCES_CEREVISIAE  VALRKQLDIYANVALFKSLKGVKTRIPD...IDLIVIRENTEGEFSGLEHESVPG.....

                                   180       190       200       210       220       230
P08200_ESCHERICHIA_COLI          IKFLREEMGVKKIRFPEHCGIGIKPCSEEGTKRLVRAAIEYAIANDRDSVTLVHKGNIMK
P28834_SACCHAROMYCES_CEREVISIAE  ..................VVESLKVMTRPKTERIARFAFDFAKKYNRKSVTAVHKANIMK

                                   240       250       260       270       280       290
P08200_ESCHERICHIA_COLI          FTEGAFKDWGYQLAREEFGGELIDGGPWLKVKNPNTGKEIVIKDVIADAFLQQILLRPAE
P28834_SACCHAROMYCES_CEREVISIAE  LGDGLFRNIITEIGQKEY....................PDIDVSSIIVDNASMQAVAKPHQ

                                   300       310       320       330        340       350
P08200_ESCHERICHIA_COLI          YDVIACMNLNGDYISDALAAQVGGIGIAPGANIGDECALFEA.THGTAPKYAGQDKVNPG
P28834_SACCHAROMYCES_CEREVISIAE  FDVLVTPSMYGTILGNIGAALIGGPGLVAGANFGRDYAVFEPGSRHVGLDIKGQNVANPT

                                     360       370       380        390       400       410
P08200_ESCHERICHIA_COLI          SIILSAEMMLRHMGWTEAADLIVKGMEGAINA.KTVTYDFERLMDGAKLLKCSEFGDAII
P28834_SACCHAROMYCES_CEREVISIAE  AMILSSTLMLNHLGLNEYATRISKAVHETIAEGKHTTRDIGGSS......STTDFTNEII

P08200_ESCHERICHIA_COLI          ENM...
P28834_SACCHAROMYCES_CEREVISIAE  NKLSTM
```

```
                              1        10        20        30        40        50
P08200_ESCHERICHIA_COLI       MESKVVVPAQGKKI.TLQNGKLNVPENPIIPYIEGDGIGVDVTPAMLKVVDAAVEKAYKG
P33197_THERMUS_THERMOPHILUS   MP..LITTETGKKMHVLEDGR......KLITVIPGDGIGPECVEATLKVLEAAKAPLA..

                             60        70        80        90       100       110
P08200_ESCHERICHIA_COLI       ERKISWMEIYTGEKSTQVYGQDVWLPAETLDLIREYRVAIKGPLTTPVGGGIRSLNVALR
P33197_THERMUS_THERMOPHILUS   ......YEVREAGASVFRRGIASGVPQETIESIRKTRVVLKGPLETPVGYGEKSANVTLR

                            120       130       140       150       160       170
P08200_ESCHERICHIA_COLI       QELDLYICLRPVRYYQGTPSPVKHPELTDMVIFRENSEDIYAGIEWKADSADAEKVIKFL
P33197_THERMUS_THERMOPHILUS   KLFETYANVRPVREFPNVPTPYAGRG.IDLVVVRENVEDLYAGIEHMQTPS.........

                            180       190       200       210       220       230
P08200_ESCHERICHIA_COLI       REEMGVKKIRFPEHCGIGIKPCSEEGTKRLVRAAIEYAIANDRDSVTLVHKGNIMKFTEG
P33197_THERMUS_THERMOPHILUS   ..............VAQTLKLISWKGSEKIVRFAFELARAEGRKKVHCATKSNIMKLAEG

                            240       250       260       270       280       290
P08200_ESCHERICHIA_COLI       AFKDWGYQLAREEFGGELIDGGPWLKVKNPNTGKEIVIKDVIADAFLQQILLRPAEYDVI
P33197_THERMUS_THERMOPHILUS   TLKRAFEQVAQEY....................PDIEAVHIIVDNAAHQLVKRPEQFEVI

                            300       310       320       330       340       350
P08200_ESCHERICHIA_COLI       ACMNLNGDYISDALAAQVGGIGIAPGANIGDECALFEATHGTAPKYAGQDKVNPGSIILS
P33197_THERMUS_THERMOPHILUS   VTTNMNGDILSDLTSGLIGGLGFAPSANIGNEVAIFEAVHGSAPKYAGKNVINPTAVLLS

                            360       370       380        390       400       410
P08200_ESCHERICHIA_COLI       AEMMLRHMGWTEAADLIVKGMEGAINAK.TVTYDFERLMDGAKLLKCSEFGDAIIENM..
P33197_THERMUS_THERMOPHILUS   AVMMLRYLEEFATADLIENALLYTLEEGRVLTGDVVGYD...RGAKTTEYTEAIIQNLGK

P08200_ESCHERICHIA_COLI       ............................................................
P33197_THERMUS_THERMOPHILUS   RKTQVRGYKPFRLPQVDGAIAPIVPRSRRVVGVDVFVETNLLPEALGKALEDLAAGTPLK

P08200_ESCHERICHIA_COLI       ............................................................
P33197_THERMUS_THERMOPHILUS   MISNRGTQVYPPTGGLTDLVDHYRCRFLYTGEGEAKDPEILDLVSRVASRFRWMHLLQEF

P08200_ESCHERICHIA_COLI       .............
P33197_THERMUS_THERMOPHILUS   DGEPGFTKAQGED
```

```
                            1        10        20        30        40        50        60
P08200_ESCHERICHIA_COLI     MESKVVVPAQGKKITLQNGKLNVPENPIIPYIEGDGIGVDVTPAMLKVVDAAVEKAYKGE
P39126_BACILLUS_SUBTILIS    M.......AQGEKITVSNGVLNVPNNPIIPFIEGDGTGPDIWNAASKVLEAAVEKAYKGE
```

```
                                     70        80        90       100       110       120
P08200_ESCHERICHIA_COLI     RKISWMEIYTGEKSTQVYGQDVWLPAETLDLIREYRVAIKGPLTTPVGGGIRSLNVALRQ
P39126_BACILLUS_SUBTILIS    KKITWKEVYAGEKAYNKTGE..WLPAETLDVIREYFIAIKGPLTTPVGGGIRSLNVALRQ
```

```
                                    130       140       150       160       170       180
P08200_ESCHERICHIA_COLI     ELDLYICLRPVRYYQGTPSPVKHPELTDMVIFRENSEDIYAGIEWKADSADAEKVIKFLR
P39126_BACILLUS_SUBTILIS    ELDLFVCLRPVRYFTGVPSPVKRPEDTDMVIFRENTEDIYAGIEYAKGSEEVQKLISFLQ
```

```
                                    190       200       210       220       230       240
P08200_ESCHERICHIA_COLI     EEMGVKKIRFPEHCGIGIKPCSEEGTKRLVRAAIEYAIANDRDSVTLVHKGNIMKFTEGA
P39126_BACILLUS_SUBTILIS    NELNVNKIRFPETSGIGIKPVSEEGTSRLVRAAIDYAIEHGRKSVTLVHKGNIMKFTEGA
```

```
                                    250       260                      270       280
P08200_ESCHERICHIA_COLI     FKDWGYQLAREEFGGELIDGGPWLKV.............KNPNTGKEIVIKDVIADAFLQ
P39126_BACILLUS_SUBTILIS    FKNWGYELAEKEYGDKVFTWAQYDRIAEEQGKDAANKAQSEAEAAGKIIIKDSIADIFLQ
```

```
                            290       300       310       320       330         340
P08200_ESCHERICHIA_COLI     QILLRPAEYDVIACMNLNGDYISDALAAQVGGIGIAPGANIGDEC..ALFEATHGTAPKY
P39126_BACILLUS_SUBTILIS    QILTRPNEFDVVATMNLNGDYISDALAAQVGGIGIAPGANINYETGHAIFEATHGTAPKY
```

```
                              350       360       370       380       390       400
P08200_ESCHERICHIA_COLI     AGQDKVNPGSIILSAEMMLRHMGWTEAADLIVKGMEGAINAKTVTYDFERLMDGAKLLKC
P39126_BACILLUS_SUBTILIS    AGLDKVNPSSVILSGVLLLEHLGWNEAADLVIKSMEKTIASKVVTYDFARLMDGATEVKC
```

```
                              410
P08200_ESCHERICHIA_COLI     SEFGDAIIENM.
P39126_BACILLUS_SUBTILIS    SEFGEELIKNMD
```

```
                         1        10        20                  30       40        50
P08200_ESCHERICHIA_COLI  MESKVVVPAQGKKITLQNGKLNVPEN.........PIIPYIEGDGIGVDVTPAMLKVVDA
P50213_HOMO_SAPIENS      MAG....PAWISKVSRLLGAFHNPKQVTRGFTGGVQTVTLIPGDGIGPEISAAVMKIFDA

                                 60        70        80        90       100       110
P08200_ESCHERICHIA_COLI  AVEKAYKGERKISWMEIYTGEKSTQVYGQDVWLPAETLDLIREYRVAIKGPLTTPVGGGI
P50213_HOMO_SAPIENS      AK.......APIQWEERNVTAIQGPGGK..WMIPSEAKESMDKNKMGLKGPLKTPIAAGH

                                120       130       140       150       160       170
P08200_ESCHERICHIA_COLI  RSLNVALRQELDLYICLRPVRYYQGTPSPVKHPELTDMVIFRENSEDIYAGIEWKADSAD
P50213_HOMO_SAPIENS      PSMNLLLRKTFDLYANVRPCVSIEGYKTPYTD...VNIVTIRENTEGEYSGIEHVIVDG.

                                180       190       200       210       220       230
P08200_ESCHERICHIA_COLI  AEKVIKFLREEMGVKKIRFPEHCGIGIKPCSEEGTKRLVRAAIEYAIANDRDSVTLVHKG
P50213_HOMO_SAPIENS      ......................VVQSIKLITEGASKRIAEFAFEYARNNHRSNVTAVHKA

                                240       250       260       270       280       290
P08200_ESCHERICHIA_COLI  NIMKFTEGAFKDWGYQLAREEFGGELIDGGPWLKVKNPNTGKEIVIKDVIADAFLQQILL
P50213_HOMO_SAPIENS      NIMRMSD.GLFLQKCREVAESC...................KDIKFNEMYLDTVCLNMVQ

                                300       310       320       330       340       350
P08200_ESCHERICHIA_COLI  RPAEYDVIACMNLNGDYISDALAAQVGGIGIAPGANIGDE.CALFEATHGTAPKYAGQDK
P50213_HOMO_SAPIENS      DPSQFDVLVMPNLYGDILSDLCAGLIGGLGVTPSGNIGANGVAIFESVHGTAPDIAGKDM

                                  360       370       380        390       400
P08200_ESCHERICHIA_COLI  VNPGSIILSAEMMLRHMGWTEAADLIVKGMEGAINAKT.VTYDFERLMDGAKLLKCSEFG
P50213_HOMO_SAPIENS      ANPTALLLSAVMMLRHMGLFDHAARIEAACFATIKDGKSLTKDLGGNA......KCSDFT

                        410
P08200_ESCHERICHIA_COLI  DAIIENM....
P50213_HOMO_SAPIENS      EEICRRVKDLD
```

```
                        1        10        20                                   30
P08200_ESCHERICHIA_COLI MESKVV.VPAQGKKITLQNGKLNVP.......ENP....................IIPYI
P51553_HOMO_SAPIENS     MALKVATVAGSAAKAVLGPALLCRPWEVLGAHEVPSRNIFSEQTIPPSAKYGGRHTVTMI

                                40        50        60        70        80        90
P08200_ESCHERICHIA_COLI EGDGIGVDVTPAMLKVVDAAVEKAYKGERKISWMEIYTGEKSTQVYGQDVWLPAETLDLI
P51553_HOMO_SAPIENS     PGDGIGPELMLHVKSVFRHACVPV.......DFEEVHVSSNA......DEEDIRNAIMAI

                             100         110       120       130       140       150
P08200_ESCHERICHIA_COLI REYRVAIKGPLTTPVG..GGIRSLNVALRQELDLYICLRPVRYYQGTPSPVKHPELTDMV
P51553_HOMO_SAPIENS     RRNRVALKGNIETNHNLPPSHKSRNNILRTSLDLYANVIHCKSLPGVVT..RHKD.IDIL

                                160       170       180       190       200       210
P08200_ESCHERICHIA_COLI IFRENSEDIYAGIEWKADSADAEKVIKFLREEMGVKKIRFPEHCGIGIKPCSEEGTKRLV
P51553_HOMO_SAPIENS     IVRENTEGEYSSLEHESVAG.......................VVESLKIITKAKSLRIA

                                220       230       240       250       260       270
P08200_ESCHERICHIA_COLI RAAIEYAIANDRDSVTLVHKGNIMKFTEGAFKDWGYQLAREEFGGELIDGGPWLKVKNPN
P51553_HOMO_SAPIENS     EYAFKLAQESGRKKVTAVHKANIMKLGD.GLFLQCCREVAARY.................

                                280       290       300       310       320       330
P08200_ESCHERICHIA_COLI TGKEIVIKDVIADAFLQQILLRPAEYDVIACMNLNGDYISDALAAQVGGIGIAPGANIGD
P51553_HOMO_SAPIENS     ..PQITFENMIVDNTTMQLVSRPQQFDVMVMPNLYGNIVNNVCAGLVGGPGLVAGANYGH

                                 340       350       360       370       380
P08200_ESCHERICHIA_COLI ECALFE.ATHGTAPKYAGQDKVNPGSIILSAEMMLRHMGWTEAADLIVKGMEGAINAKTV
P51553_HOMO_SAPIENS     VYAVFETATRNTGKSIANKNIANPTATLLASCMMLDHLKLHSYATSIRKAVLASMDNENM

                        390       400       410
P08200_ESCHERICHIA_COLI .TYDFERLMDGAKLLKCSEFGDAIIENM..........
P51553_HOMO_SAPIENS     HTPDIGGQG......TTSEAIQDVIRHIRVINGRAVEA
```

```
                                 1        10        20        30        40        50
_P08200_ESCHERICHIA_COLI         MES.KVVVPAQGKKITLQ.NGKLNVPENPIIPYIEGDGIGVDVTPAMLKVVDAAVEKAYK
_Q02NB5_PSEUDOMONAS_AERUGINOSA   MGYQKIQVPATGDKITVNADMSLSVPKNPIIPFIEGDGIGVDISPVMIKVVDAAVEKAYK

                                 60        70        80        90        100       110
_P08200_ESCHERICHIA_COLI         GERKISWMEIYTGEKSTQVYGQDVWLPAETLDLIREYRVAIKGPLTTPVGGGIRSLNVAL
_Q02NB5_PSEUDOMONAS_AERUGINOSA   GERKIAWMEVYAGEKATQVYDQDTWLPQETLDAVRDYVVSIKGPLTTPVGGGIRSLNVAL

                                120       130       140       150       160       170
_P08200_ESCHERICHIA_COLI         RQELDLYICLRPVRYYQGTPSPVKHPELTDMVIFRENSEDIYAGIEWKADSADAEKVIKF
_Q02NB5_PSEUDOMONAS_AERUGINOSA   RQQLDLYVCQRPVRWFEGVPSPVKKPGDVDMVIFRENSEDIYAGVEWKAGSPEAEKVIKF

                                180       190       200       210       220       230
_P08200_ESCHERICHIA_COLI         LREEMGVKKIRFPEHCGIGIKPCSEEGTKRLVRAAIEYAIANDRDSVTLVHKGNIMKFTE
_Q02NB5_PSEUDOMONAS_AERUGINOSA   LTEEMGVKKIRFTENCGIGIKPVSQEGTKRLVRKALQYAVDNDRSSVTLVHKGNIMKFTE

                                240       250       260       270       280       290
_P08200_ESCHERICHIA_COLI         GAFKDWGYQLAREEFGGELIDGGPWLKVKNPNTGKEIVIKDVIADAFLQQILLRPAEYDV
_Q02NB5_PSEUDOMONAS_AERUGINOSA   GAFKDWGYEVARDEFGAELLDGGPWMQFKNPKTGKNVVVKDVIADAMLQQILLRPAEYDV

                                300       310       320       330       340       350
P08200_ESCHERICHIA_COLI          IACMNLNGDYISDALAAQVGGIGIAPGANIGDECALFEATHGTAPKYAGQDKVNPGSIIL
Q02NB5_PSEUDOMONAS_AERUGINOSA    IATLNLNGDYLSDALAAEVGGIGIAPGANLSDSVAMFEATHGTAPKYAGQDKVNPGSLIL

                                360       370       380       390       400       410
P08200_ESCHERICHIA_COLI          SAEMMLRHMGWTEAADLIVKGMEGAINAKTVTYDFERLMDGAKLLKCSEFGDAIIENM
Q02NB5_PSEUDOMONAS_AERUGINOSA    SAEMMLRHMGWTEAADLIIKGTNGAIAAKTVTYDFERLMDGATLLSCSEFGDAMIAKM
```

```
                                  1        10        20        30        40        50
P08200_ESCHERICHIA_COLI           MESK.VVVPAQGKKITLQNG.KLNVPENPIIPYIEGDGIGVDVTPAMLKVVDAAVEKAYK
Q3JV82_BURKHOLDERIA_PSEUDOMALLEI  MPYQHIKVPEGGDKITVNKDFSLNVSDQPIIPYIEGDGTGFDITPVMIKVVDAAVEKAYG

                                  60        70        80        90        100       110
P08200_ESCHERICHIA_COLI           GERKISWMEIYTGEKSTQVYGQDVWLPAETLDLIREYRVAIKGPLTTPVGGGIRSLNVAL
Q3JV82_BURKHOLDERIA_PSEUDOMALLEI  GKKKIHWMEIYAGEKATKVYGPDVWLPEETLQVLKEYVVSIKGPLTTPVGGGIRSLNVAL

                                 120       130       140       150       160       170
P08200_ESCHERICHIA_COLI           RQELDLYICLRPVRYYQGTPSPVKHPELTDMVIFRENSEDIYAGIEWKADSADAEKVIKF
Q3JV82_BURKHOLDERIA_PSEUDOMALLEI  RQELDLYVCLRPIQYFKGVPSPVREPEKTNMVIFRENSEDIYAGIEWAAESEQAKKVIKF

                                 180       190       200       210       220       230
P08200_ESCHERICHIA_COLI           LREEMGVKKIRFPEHCGIGIKPCSEEGTKRLVRAAIEYAIANDRDSVTLVHKGNIMKFTE
Q3JV82_BURKHOLDERIA_PSEUDOMALLEI  LQEEMGVKKIRFPQTSGIGIKPVSKEGTERLVRKAIQYAIDNDRKSVTLVHKGNIMKFTE

                                 240       250       260       270       280       290
P08200_ESCHERICHIA_COLI           GAFKDWGYQLAREEFGGELIDGGPWLKVKNPNTGKEIVIKDVIADAFLQQILLRPAEYDV
Q3JV82_BURKHOLDERIA_PSEUDOMALLEI  GAFRDAGYALAQKEFGAELIDGGPWMKFKNPKTGNEIVVKDSIADAFLQQILLRPAEYDV

                                 300       310       320       330       340       350
P08200_ESCHERICHIA_COLI           IACMNLNGDYISDALAAQVGGIGIAPGANIGDECALFEATHGTAPKYAGQDKVNPGSIIL
Q3JV82_BURKHOLDERIA_PSEUDOMALLEI  IATLNLNGDYISDALAAQVGGIGIAPGANLSDSVAMFEATHGTAPKYAGKDYVNPGSEIL

                                 360       370       380       390       400       410
P08200_ESCHERICHIA_COLI           SAEMMLRHMGWTEAADLIVKGMEGAINAKTVTYDFERLMDGAKLLKCSEFGDAIIENM.
Q3JV82_BURKHOLDERIA_PSEUDOMALLEI  SAEMMLRHLGWTEAADVIISAMEKSIKQKRVTYDFARLMEGATQVSCSGFGQVLIENME
```

```
                                1         10        20        30        40        50        60
P08200_ESCHERICHIA_COLI         MESKVVVPAQGKKITLQNGKLNVPENPIIPYIEGDGIGVDVTPAMLKVVDAAVEKAYKGE
Q5JFV8_THERMOCOCCUS_KODAKARENSIS MY.........................RVAVIPGDGIGPEVIDGAVRVLKAVTGRVR...

                                          70        80        90        100       110
P08200_ESCHERICHIA_COLI         RKISWMEIYTGEKSTQVYGQDVWLPAETLDLIREYRVAIKGPLTTPVG.GGIRSLNVALR
Q5JFV8_THERMOCOCCUS_KODAKARENSIS ....FEYYEGGVDVFQECG..SPIREEDLEEIRRSDAVLFGATTTPFDLPGYRSLILTLR

                               120       130       140       150       160       170
P08200_ESCHERICHIA_COLI         QELDLYICLRPVRYYQGTPSPVKHPEL...TDMVIFRENSEDIYAGIEWKADSADAEKVI
Q5JFV8_THERMOCOCCUS_KODAKARENSIS KELGLYANLRII............PDLRTGREIVIVRENSEGLYFGIGAVVNGR......

                                  180       190       200       210       220       230
P08200_ESCHERICHIA_COLI         KFLREEMGVKKIRFPEHCGIGIKPCSEEGTKRLVRAAIEYAIANDRDSVTLVHKGNIMKF
Q5JFV8_THERMOCOCCUS_KODAKARENSIS ..................AVDVRLITREGAERIARFAVEQAKAR.GSFITFVHKANVLTG

                                  240       250       260       270       280       290
P08200_ESCHERICHIA_COLI         TEGAFKDWGYQLAREEFGGELIDGGPWLKVKNPNTGKEIVIKDVIADAFLQQILLRPAEY
Q5JFV8_THERMOCOCCUS_KODAKARENSIS DK.FFRIVREVAGE......................EGVEVRDAIIDSFTIKLVRNPWEH

                                  300       310       320       330       340       350
P08200_ESCHERICHIA_COLI         DVIACMNLNGDYISDALAAQVGGIGIAPGANIGDECALFEATHGTAPKYAGQDKVNPGSI
Q5JFV8_THERMOCOCCUS_KODAKARENSIS GVILSENLFGDILSDLATVHAGSIGIVPSGNYGDGIALFEPVHGSAPDIAGKGIANPIGA

                                  360       370       380       390       400       410
P08200_ESCHERICHIA_COLI         ILSAEMMLRHMGWTEAADLIVKGMEGAINAKTVTYDFERLMDGAKLLKCSEFGDAIIENM
Q5JFV8_THERMOCOCCUS_KODAKARENSIS ILSGAMLLDYLGLD..GSLIRAAVRGYVVNGELTPDMGGRA......RTEDVVRGIIGEI

P08200_ESCHERICHIA_COLI         ............................
Q5JFV8_THERMOCOCCUS_KODAKARENSIS EDLLSMDEVWRDEIRLSRLESDISRMAG
```

```
                              1        10        20        30        40        50        60
P08200_ESCHERICHIA_COLI       MESKVVVPAQGKKITLQNGKLNVPENPIIPYIEGDGIGVDVTPAMLKVVDAAVEKAYKGE
Q5SIJ1_THERMUS_THERMOPHILUS   ........................MAYRICLIEGDGIGHEVIPAARRVLEATG.......

                                       70        80        90       100       110
P08200_ESCHERICHIA_COLI       RKISWMEIYTGEKSTQVYGQDVWLPAETLDLIREYRVAIKGPLTTPVGG..GIRSLNVAL
Q5SIJ1_THERMUS_THERMOPHILUS   LPLEFVEAEAGWETFERRG..TSVPEETVEKILSCHATLFGAATSPTRKVPGFFGAIRYL

                             120       130       140       150       160       170
P08200_ESCHERICHIA_COLI       RQELDLYICLRPVRYYQGTPSPVKHPELTDMVIFRENSEDIYAGIEWKADSADAEKVIKF
Q5SIJ1_THERMUS_THERMOPHILUS   RRRLDLYANVRPAKSR...PVPGSRPG.VDLVIVRENTEGLYVEQE..............

                             180       190       200       210       220       230
P08200_ESCHERICHIA_COLI       LREEMGVKKIRFPEHCGIGIKPCSEEGTKRLVRAAIEYAIANDRDSVTLVHKGNIMKFTE
Q5SIJ1_THERMUS_THERMOPHILUS   ..........RRYLDVAIADAVISKKASERIGRAALRIAEGRPRKTLHIAHKANVLPLTQ

                             240       250       260       270       280       290
P08200_ESCHERICHIA_COLI       GAFKDWGYQLAREEFGGELIDGGPWLKVKNPNTGKEIVIKDVIADAFLQQILLRPAEYDV
Q5SIJ1_THERMUS_THERMOPHILUS   GLFLDTVKEVAKDF....................PLVNVQDIIVDNCAMQLVMRPERFDV

                             300       310       320       330       340       350
P08200_ESCHERICHIA_COLI       IACMNLNGDYISDALAAQVGGIGIAPGANIGDECALFEATHGTAPKYAGQDKVNPGSIIL
Q5SIJ1_THERMUS_THERMOPHILUS   IVTTNLLGDILSDLAAGLVGGLGLAPSGNIGDTTAVFEPVHGSAPDIAGKGIANPTAAIL

                             360       370       380       390       400       410
P08200_ESCHERICHIA_COLI       SAEMMLRHMGWTEAADLIVKGMEGAINAKTVTYDFERLMDGAKLLKCSEFGDAIIENM..
Q5SIJ1_THERMUS_THERMOPHILUS   SAAMMLDYLGEKEAAKRVEKAVDLVLERGPRTPDLGGDA......TTEAFTEAVVEALKS
```

```
                                 1        10        20        30        40        50
P08200_ESCHERICHIA_COLI          MES.....KVVVPAQGKKITLQ.NGKLNVPENPIIPYIEGDGIGVDVTPAMLKVVDAAVE
Q5ZXB6_LEGIONELLA_PNEUMOPHILA    MESSMTYDKIKVPAQGEAITVAADHSLHVPDNPIIPFIEGDGIGVDVTPPMIRVVDAAVQ

                                      60        70        80        90       100       110
P08200_ESCHERICHIA_COLI          KAYKGERKISWMEIYTGEKSTQVYGQDVWLPAETLDLIREYRVAIKGPLTTPVGGGIRSL
Q5ZXB6_LEGIONELLA_PNEUMOPHILA    KAYGNKRKISWMEVYAGEKATKVYGGDQWLPKETLDAMKKYVVSIKGPLTTPVGGGIRSL

                                      120       130       140       150       160       170
P08200_ESCHERICHIA_COLI          NVALRQELDLYICLRPVRYYQGTPSPVKHPELTDMVIFRENSEDIYAGIEWKADSADAEK
Q5ZXB6_LEGIONELLA_PNEUMOPHILA    NVAIRQDMDLYVCLRPIRYFNGVPSPVREPWKTDMVIFRENSEDIYAGIEWQADTPEAKK

                                      180       190       200       210       220       230
P08200_ESCHERICHIA_COLI          VIKFLREEMGVKKIRFPEHCGIGIKPCSEEGTKRLVRAAIEYAIANDRDSVTLVHKGNIM
Q5ZXB6_LEGIONELLA_PNEUMOPHILA    VIQFLTKEMGVKKIRFPEHCGIGVKPVSREGTTRLVKAAIQYAIDNDRSTVTLVHKGNIM

                                      240       250       260       270       280       290
P08200_ESCHERICHIA_COLI          KFTEGAFKDWGYQLAREEFGGELIDGGPWLKVKNPNTGKEIVIKDVIADAFLQQILLRPA
Q5ZXB6_LEGIONELLA_PNEUMOPHILA    KFTEGAFKDWGYQVARDSFGAKEYQGGPWMEFKNPKTGKQIIINDVIADAFLQQILLRPE

                                      300       310       320       330       340       350
P08200_ESCHERICHIA_COLI          EYDVIACMNLNGDYISDALAAQVGGIGIAPGANIGDECALFEATHGTAPKYAGQDKVNPG
Q5ZXB6_LEGIONELLA_PNEUMOPHILA    DYSVIATLNLNGDYISDALAAQVGGIGIAPGANISDQMAVFEATHGTAPKYAGQNKVNPG

                                      360       370       380       390       400       410
P08200_ESCHERICHIA_COLI          SIILSAEMMLRHMGWTEAADLIVKGMEGAINAKTVTYDFERLMDGAKLLKCSEFGDAIIE
Q5ZXB6_LEGIONELLA_PNEUMOPHILA    SIILSAEMMLRHMGWYEAADLIIRGMEGAIEAKTVTYDFERGMQGATLVSSSGFADAMIK

P08200_ESCHERICHIA_COLI          NM
Q5ZXB6_LEGIONELLA_PNEUMOPHILA    HM
```

```
                              1        10        20        30        40        50        60
P08200_ESCHERICHIA_COLI       MESKVVVPAQGKKITLQNGKLNVPENPIIPYIEGDGIGVDVTPAMLKVVDAAVEKAYKGE
Q72IW9_THERMUS_THERMOPHILUS   ........................MAYRICLIEGDGIGHEVIPAARRVLEATG.......

                                       70        80        90       100       110
P08200_ESCHERICHIA_COLI       RKISWMEIYTGEKSTQVYGQDVWLPAETLDLIREYRVAIKGPLTTPVGG..GIRSLNVAL
Q72IW9_THERMUS_THERMOPHILUS   LPLEFVEAEAGWETFERRG..TSVPEETVEKILSCHATLFGAATSPTRKVPGFFGAIRYL

                             120       130       140       150       160       170
P08200_ESCHERICHIA_COLI       RQELDLYICLRPVRYYQGTPSPVKHPELTDMVIFRENSEDIYAGIEWKADSADAEKVIKF
Q72IW9_THERMUS_THERMOPHILUS   RRRLDLYANVRPAKSR...PVPGSRPG.VDLVIVRENTEGLYVEQE..............

                             180       190       200       210       220       230
P08200_ESCHERICHIA_COLI       LREEMGVKKIRFPEHCGIGIKPCSEEGTKRLVRAAIEYAIANDRDSVTLVHKGNIMKFTE
Q72IW9_THERMUS_THERMOPHILUS   ..........RRYLDVAIADAVISKKASERIGRAALRIAEGRPRKTLHIAHKANVLPLTQ

                             240       250       260       270       280       290
P08200_ESCHERICHIA_COLI       GAFKDWGYQLAREEFGGELIDGGPWLKVKNPNTGKEIVIKDVIADAFLQQILLRPAEYDV
Q72IW9_THERMUS_THERMOPHILUS   GLFLDTVKEVAKDF....................PLVNVQDIIVDNCAMQLVMRPERFDV

                             300       310       320       330       340       350
P08200_ESCHERICHIA_COLI       IACMNLNGDYISDALAAQVGGIGIAPGANIGDECALFEATHGTAPKYAGQDKVNPGSIIL
Q72IW9_THERMUS_THERMOPHILUS   IVTTNLLGDILSDLAAGLVGGLGLAPSGNIGDTTAVFEPVHGSAPDIAGKGIANPTAAIL

                             360       370       380       390       400       410
P08200_ESCHERICHIA_COLI       SAEMMLRHMGWTEAADLIVKGMEGAINAKTVTYDFERLMDGAKLLKCSEFGDAIIENM..
Q72IW9_THERMUS_THERMOPHILUS   SAAMMLDYLGEKEAAKRVEKAVDLVLERGPRTPDLGGDA......TTEAFTEAVVEALKS
```

```
                                          1        10        20        30        40        50        60
P08200_ESCHERICHIA_COLI                   MESKVVVPAQGKKITLQNGKLNVPENPIIPYIEGDGIGVDVTPAMLKVVDAAVEKAYKGE
Q8GAX0_ACIDTHIOBACILLUS_THIOOXIDANS       MTTHIQKPATGSPLTLLNGVLQVPDQPIIPFIEGDGIGCDVTPAMRSVVDAAVAKVYGGQ

                                                   70        80        90       100       110       120
P08200_ESCHERICHIA_COLI                   RKISWMEIYTGEKSTQVYGQDVWLPAETLDLIREYRVAIKGPLTTPVGGGIRSLNVALRQ
Q8GAX0_ACIDTHIOBACILLUS_THIOOXIDANS       RQIAWMELFAGQKAVQLYGEGQYLPDETMAAIREYKVAIKGPLETPVGGGIRSLNVAMRQ

                                                  130       140       150       160       170       180
P08200_ESCHERICHIA_COLI                   ELDLYICLRPVRYYQGTPSPVKHPELTDMVIFRENSEDIYAGIEWKADSADAEKVIKFLR
Q8GAX0_ACIDTHIOBACILLUS_THIOOXIDANS       DLDLYVCLRPVRYFEGTPSPMRHPEKVDMVIFRENSEDIYAGIEWPAGSPEAEKIIRFLR

                                                  190       200       210       220       230       240
P08200_ESCHERICHIA_COLI                   EEMGVKKIRFPEHCGIGIKPCSEEGTKRLVRAAIEYAIANDRDSVTLVHKGNIMKFTEGA
Q8GAX0_ACIDTHIOBACILLUS_THIOOXIDANS       EEMGVTKIRFPDSSAIGIKPVSTEGSERLIRRTIQYALEHGKPSVSLVHKGNIMKFTEGG

                                                  250          260       270                280
P08200_ESCHERICHIA_COLI                   FKDWGYQLAREEFGGELI...DGGPWLKVKNPNTGK..........EIVIKDVIADAFLQ
Q8GAX0_ACIDTHIOBACILLUS_THIOOXIDANS       FRDWGYALAERERFAGRVFTWRQKAAISKAEGKAAGQKAEQQAIADGKLIIKDVIADNFLQ

                                          290       300       310       320       330       340
P08200_ESCHERICHIA_COLI                   QILLRPAEYDVIACMNLNGDYISDALAAQVGGIGIAPGANIGDECALFEATHGTAPKYAG
Q8GAX0_ACIDTHIOBACILLUS_THIOOXIDANS       QILLRPEDYSVVATLNLNGDYVSDALAAEVGGIGMAPGANLSDTHAIFEATHGTAPDIAG

                                          350       360       370       380       390       400
P08200_ESCHERICHIA_COLI                   QDKVNPGSIILSAEMMLRHMGWTEAADLIVKGMEGAINAKTVTYDFERLMDGAKLLKCSE
Q8GAX0_ACIDTHIOBACILLUS_THIOOXIDANS       QGKANPSSLILSAVMMLEHLGWGEAAQAIVAAMNATIAAGEVTGDLAALRGDVPALSTTE

                                          410
P08200_ESCHERICHIA_COLI                   FGDAIIENM
Q8GAX0_ACIDTHIOBACILLUS_THIOOXIDANS       FTAALIRRF
```

```
                                 1        10        20        30        40        50        60
P08200_ESCHERICHIA_COLI          MESKVVVPAQGKKITLQNGKLNVPENPIIPYIEGDGIGVDVTPAMLKVVDAAVEKAYKGE
Q96YK6_SULFURISPHAERA_TOKODAII   MLYKE..PEDGEKIKFDKGKWIVPNKPVILYIEGDGIGPEITNAAIKVINKAVERAYGSS

                                          70        80        90       100       110       120
P08200_ESCHERICHIA_COLI          RKISWMEIYTGEKSTQVYGQDVWLPAETLDLIREYRVAIKGPLTTPVGGGIRSLNVALRQ
Q96YK6_SULFURISPHAERA_TOKODAII   REIKWLEVYAGEKAEKLVN..DRFPKETQEMLLKYRVVLKGPLETPIGKGWKSVNVAIRL

                                         130       140       150       160       170       180
P08200_ESCHERICHIA_COLI          ELDLYICLRPVRYYQGTPSPVKHPELTDMVIFRENSEDIYAGIEWKADSADAEKVIKFLR
Q96YK6_SULFURISPHAERA_TOKODAII   MLDLYANIRPVKYIEGLESPLKHPEKVDMIIFRENTDDLYRGIEYPFNSEEAKKIRDFLR

                                         190       200       210       220       230       240
P08200_ESCHERICHIA_COLI          EEMGVKKIRFPEHCGIGIKPCSEEGTKRLVRAAIEYAIANDRDSVTLVHKGNIMKFTEGA
Q96YK6_SULFURISPHAERA_TOKODAII   KEL...KVEIEDDTGIGIKVMSKYKTQRITRLAIQYAIEHKRKKVTIMHKGNVMKYTEGA

                                         250       260       270       280       290       300
P08200_ESCHERICHIA_COLI          FKDWGYQLAREEFGGELIDGGPWLKVKNPNTGKEIVIKDVIADAFLQQILLRPAEYDVIA
Q96YK6_SULFURISPHAERA_TOKODAII   FREWAYEVALKEYRDFIVTEEE.INQGKPDQGK.IILNDRIADNMFQQIIIRPEEYDIIL

                                         310       320       330       340       350       360
P08200_ESCHERICHIA_COLI          CMNLNGDYISDALAAQVGGIGIAPGANIGDECALFEATHGTAPKYAGQDKVNPGSIILSA
Q96YK6_SULFURISPHAERA_TOKODAII   APNVNGDYISDAAGALIGNIGMLGGANIGDEGGMFEAIHGTAPKYAGKNVANPTGIIKAG

                                         370       380       390       400       410
P08200_ESCHERICHIA_COLI          EMMLRHMGWTEAADLIVKGMEGAINAKTVTYDFERLMDGAKLLKCSEFGDAIIENM...
Q96YK6_SULFURISPHAERA_TOKODAII   ELMLRWMGWNEAADLIEKAINMAIRDKKVTQDIARFM.GVKALGTKEYADELIKIMDTI
```

```
                              1        10        20        30        40        50
P08200_ESCHERICHIA_COLI       MES......KVVVPAQGKKITLQNGKLNVPENPIIPYIEGDGIGVDVTPAMLKVVDAAVE
Q9YE81_AEROPYRUM_PERNIX       MASPPCTTEELSPPPGGSLVEYSGGSLRVPDNPVVAFIRGDGVGPEVVESALKVVDAAVK

                                  60        70        80        90       100       110
P08200_ESCHERICHIA_COLI       KAYKGERKISWMEIYTGEKSTQVYGQDVWLPAETLDLIREYRVAIKGPLTTPVGGGIRSL
Q9YE81_AEROPYRUM_PERNIX       KVYGGSRRIVWWELLAGHLAREKCG..ELLPKATLEGIRLARVALKGPLETPVGTGYRSL

                                 120       130       140       150       160       170
P08200_ESCHERICHIA_COLI       NVALRQELDLYICLRPVRYYQGTPSPVKHPELTDMVIFRENSEDIYAGIEWKADSADAEK
Q9YE81_AEROPYRUM_PERNIX       NVAIRQALDLYANIRPVRYY.GQPAPHKYADRVDMVIFRENTEDVYAGIEWPHDSPEAAR

                                 180       190       200       210       220       230
P08200_ESCHERICHIA_COLI       VIKFLREEMGVKKIRFPEHCGIGIKPCSEEGTKRLVRAAIEYAIANDRDSVTLVHKGNIM
Q9YE81_AEROPYRUM_PERNIX       IRRFLAEEFGIS...IREDAGIGVKPISRFATRRLMERALEWALRNGNTVVTIMHKGNIM

                                 240       250        260       270       280       290
P08200_ESCHERICHIA_COLI       KFTEGAFKDWGYQLAREEFGGELI.DGGPWLKVKNPNTGKEIVIKDVIADAFLQQILLRP
Q9YE81_AEROPYRUM_PERNIX       KYTEGAFMRWAYEVALEKFREHVVTEQEVQEKYGGVRPEGKILVNDRIADNMLQQIITRP

                                  300       310       320       330       340       350
P08200_ESCHERICHIA_COLI       AEYDVIACMNLNGDYISDALAAQVGGIGIAPGANIGDECALFEATHGTAPKYAGQDKVNP
Q9YE81_AEROPYRUM_PERNIX       WDYQVIVAPNLNGDYISDAASALVGGIGMAAGMNMGDGIAVAEPVHGTAPKYAGKDLINP

                                  360        370       380       390       400       410
P08200_ESCHERICHIA_COLI       GSIILSAEMMLRH.MGWTEAADLIVKGMEGAINAKTVTYDFERLMDGAKLLKCSEFGDAI
Q9YE81_AEROPYRUM_PERNIX       SAEILSASLLIGEFMGWREVKSIVEYAIRKAVQSKKVTQDLARHMPGVQPLRTSEYTETL

P08200_ESCHERICHIA_COLI       IENM...............
Q9YE81_AEROPYRUM_PERNIX       IAYIDEADLNEVLAGKRG
```

Supplemental Figure SF5C. Pairwise sequence alignments between the *E. coli* substrate protein target (KatE-catalase HPII) and homologs sorted by UniProt ID. Labels adjacent to each sequence include PDB ID, UniProt ID, and organism name. Lysine residues previously identified as acetylated in the target protein are highlighted in yellow to examine conservation in 1D.

```
                            1        10        20        30        40        50        60
P21179_ESCHERICHIA_COLI     MSQHNEKNPHQHQSPLHDSSEAKPGMDSLAPEDGSHRPAAEPTPPGAQPTAPGSLKAPDT
A0A031LXI5_ACINETOBACTER    ............................................................

                                    70        80        90       100       110       120
P21179_ESCHERICHIA_COLI     RNEKLNSLEDVRKGSENYALTTNQGVRIADDQNSLRAGSRGPTLLEDFILREKITHFDHE
A0A031LXI5_ACINETOBACTER    .......MSDDSKKCPVTHLTTDAGAPVVDNQNSMTAGARGPLLAQDLWLNEKLGNFVRE

                                   130       140       150       160       170       180
P21179_ESCHERICHIA_COLI     RIPERIVHARGSAAHGYFQPYKSLSDITKADFLSDPNKITPVFVRFSTVQGGAGSADTVR
A0A031LXI5_ACINETOBACTER    VIPERRMHAKGSGAFGTFTVTHDITQYTRAKLFSEIGKKTDIFVRFSTVAGERGAADAER

                                   190       200       210       220       230       240
P21179_ESCHERICHIA_COLI     DIRGFATKFYTEEGIFDLVGNNTPIFFIQDAHKFPDFVHAVKPEPHWAIPQGQSAHDTFW
A0A031LXI5_ACINETOBACTER    DIRGFAMKFYTEEGNWDLVGNNTPVFFLRDARKFPDLNKAVKRDPK...TNKRSATNN.W

                                   250       260       270       280       290       300
P21179_ESCHERICHIA_COLI     DYVSLQPETLHNVMWAMSDRGIPRSYRTMEGFGIHTFRLINAEGKATFVRFHWKPLAGKA
A0A031LXI5_ACINETOBACTER    DFWTLLPEALHQVTIVMSDRGIPDGYRHMHGFGSHTFSFINANNERFWVKFHMRTQQGIK

                                   310       320       330       340       350       360
P21179_ESCHERICHIA_COLI     SLVWDEAQKLTGRDPDFHRRELWEAIEAGDFPEYELGFQLIPEEDEFKFDFDLLDPTKLI
A0A031LXI5_ACINETOBACTER    NLTDAEAEAIIAKDRESSQTDLFDAIERGDFPKWKMYVQIMPELDAEKVPYHPFDLTKVW

                                   370       380       390       400       410       420
P21179_ESCHERICHIA_COLI     PEELVPVQRVGKMVLNRNPDNFFAENEQAAFHPGHIVPGLDFTNDPLLQGRLFSYTDTQI
A0A031LXI5_ACINETOBACTER    PKGDYPLIEVGEFELNRNPENYFQDVEQAAFAPSNLVPGISFSPDRMLQARLFNYADAAR

                                   430       440       450          460       470
P21179_ESCHERICHIA_COLI     SRLGGPNFHEIPINRPTCPYHNFQRDGMHRMGIDTNPA...NYEPNSINDNWPRET....
A0A031LXI5_ACINETOBACTER    YRVG.VNHYQIPVNAPRCPVHSNRRDGQGRT..DGNYGALPHYEPNSFSQ.WQEQPQYKE

                                 480       490       500       510       520       530
P21179_ESCHERICHIA_COLI     PPGPKRGGFESYQERVEGNKVRERSPSFGEYYSHPRLFWLSQTPFEQRHIVDGFSFELSK
A0A031LXI5_ACINETOBACTER    PPLKISGAADFWDYREDDN..........DYFSQPRALFNLMNDQQKQALFDNTAAAMGD

                                 540       550       560       570        580        590
P21179_ESCHERICHIA_COLI     VVRPYIRERVVDQLAHIDLTLAQAVAKNLGIELTDDQL.NITPPPDVN.GLKKDPSLSLY
A0A031LXI5_ACINETOBACTER    AL.DFIKYRHIRNCYACDPAYGEGVAKALGMTVADAQAARATDPAQGNPGLL........

                                  600       610       620       630       640       650
P21179_ESCHERICHIA_COLI     AIPDGDVKGRVVAILLNDEVRSADLLAILKALKAKGVHAKLLYSRMGEVTADDGTVLPIA
A0A031LXI5_ACINETOBACTER    ............................................................

                                  660       670       680       690       700       710
P21179_ESCHERICHIA_COLI     ATFAGAPSLTVDAVIVPCGNIADIADNGDANYYLMEAYKHLKPIALAGDARKFKATIKIA
A0A031LXI5_ACINETOBACTER    ............................................................
```

```
                             720       730       740       750
                               .         .         .         .
P21179_ESCHERICHIA_COLI   DQGEEGIVEADSADGSFMDELLTLMAAHRVWSRIPKIDKIPA
A0A031LXI5_ACINETOBACTER  ..........................................
```

```
                                        1        10        20        30        40        50        60
P21179_ESCHERICHIA_COLI                 MSQHNEKNPHQHQSPLHDSSEAKPGMDSLAPEDGSHRPAAEPTPPGAQPTAPGSLKAPDT
AOAOU4WRC5_CORYNEBACTERIUM_GLUTAMICUM   ............................................................

                                                 70            80        90       100       110
P21179_ESCHERICHIA_COLI                 RNEKLNSLEDVRKGSE...NYALTTNQGVRIADDQNSLRAGSRGPTLLEDFILREKITHF
AOAOU4WRC5_CORYNEBACTERIUM_GLUTAMICUM   MSEKSAADQIVDRGMRPKLSGNTTRHNGAPVPSENISATAGPQGPNVLNDIHLIEKLAHF

                                        120       130       140       150       160       170
P21179_ESCHERICHIA_COLI                 DHERIPERIVHARGSAAHGYFQPYKSLSDITKADFLSDPNKITPVFVRFSTVQGGAGSAD
AOAOU4WRC5_CORYNEBACTERIUM_GLUTAMICUM   NRENVPERIPHAKGHGAFGELHITEDVSEYTKAD.LFQPGKVTPLAVRFSTVAGEQGSPD

                                        180       190       200       210       220       230
P21179_ESCHERICHIA_COLI                 TVRDIRGFATKFYTEEGIFDLVGNNTPIFFIQDAHKFPDFVHAVKPEPHWAIPQGQSAHD
AOAOU4WRC5_CORYNEBACTERIUM_GLUTAMICUM   TWRDVHGFALRFYTEEGNYDIVGNNTPTFFLRDGMKFPDFIHSQKR....LNKNGLRDAD

                                        240       250       260       270       280       290
P21179_ESCHERICHIA_COLI                 TFWDYVSLQPETLHNVMWAMSDRGIPRSYRTMEGFGIHTFRLINAEGKATFVRFHWKPLA
AOAOU4WRC5_CORYNEBACTERIUM_GLUTAMICUM   MQWDFWTRAPESAHQVTYLMGDRGTPKTSRHQDGFGSHTFQWINAEGKPVWVKYHFKTRQ

                                        300       310       320       330       340       350
P21179_ESCHERICHIA_COLI                 GKASLVWDEAQKLTGRDPDFHRRELWEAIEAGDFPEYELGFQLIPEEDEFKFDFDLLDPT
AOAOU4WRC5_CORYNEBACTERIUM_GLUTAMICUM   GWDCFTDAEAAKVAGENADYQREDLYNAIENGDFPIWDVKVQIMPFEDAENYRWNPFDLT

                                        360       370       380       390       400       410
P21179_ESCHERICHIA_COLI                 KLIPEELVPVQRVGKMVLNRNPDNFFAENEQAAFHPGHIVPGLDFTNDPLLQGRLFSYTD
AOAOU4WRC5_CORYNEBACTERIUM_GLUTAMICUM   KTWSQKDYPLIPVGYFILNRNPRNFFAQIEQIALDPGNIVPGVGLSPDRMLQARVFAYAD

                                        420       430       440       450        460       470
P21179_ESCHERICHIA_COLI                 TQISRLGGPNFHEIPINRPTCPYHNFQRDGMHRMGIDTN.PANYEPNSINDNWPRETPPG
AOAOU4WRC5_CORYNEBACTERIUM_GLUTAMICUM   QQRYRIGA.NYRDLPVNRPINEVNTYSREGSMQYIFDAEGEPSYSPNRYD..........

                                          480       490       500          510       520       530
P21179_ESCHERICHIA_COLI                 PKRGGFESYQERVEGNKVRERSPSFGE...YYSHPRLFWLSQTPFEQRHIVDGFSFELSK
AOAOU4WRC5_CORYNEBACTERIUM_GLUTAMICUM   .KGAGYLD.....NGTDSSSNHTSYGQADDIYVNP...............DPHGTDLVR

                                            540       550       560       570       580       590
P21179_ESCHERICHIA_COLI                 VVRPYIRERVVDQLAHIDLTLAQAVAKNLGIELTDDQLNITPPPDVNGLKKDPSLSLYAI
AOAOU4WRC5_CORYNEBACTERIUM_GLUTAMICUM   AA..YVKHQDDDDFIQPGILYREVLDEGEKERLADNISNA.....MQGISEATEPRVYDY

                                                 600       610       620       630       640       650
P21179_ESCHERICHIA_COLI                 ...PDGDVKGRVVAILLNDEVRSADLLAILKALKAKGVHAKLLYSRMGEVTADDGTVLPI
AOAOU4WRC5_CORYNEBACTERIUM_GLUTAMICUM   WNKVDENLGARVKELYLQKKA.......................................

                                                 660       670       680       690       700       710
P21179_ESCHERICHIA_COLI                 AATFAGAPSLTVDAVIVPCGNIADIADNGDANYYLMEAYKHLKPIALAGDARKFKATIKI
AOAOU4WRC5_CORYNEBACTERIUM_GLUTAMICUM   ............................................................
```

```
                                      720       730       740       750
P21179_ESCHERICHIA_COLI               ADQGEEGIVEADSADGSFMDELLTLMAAHRVWSRIPKIDKIPA
A0A0U4WRC5_CORYNEBACTERIUM_GLUTAMICUM ...........................................
```

```
                                 1        10        20        30        40        50        60
P21179_ESCHERICHIA_COLI          MSQHNEKNPHQHQSPLHDSSEAKPGMDSLAPEDGSHRPAAEPTPPGAQPTAPGSLKAPDT
A0A6I8WFM0_ESCHERICHIA_COLI      MIKKNKADFTSNGSNNKAISTVEPHYEDTAPAEKVIKSLTSISPPGVEPMMPGSDKTPKN

                                          70        80        90       100       110       120
P21179_ESCHERICHIA_COLI          RNEKLNSLEDVRKGSENYALTTNQGVRIADDQNSLRAGSRGPTLLEDFILREKITHFDHE
A0A6I8WFM0_ESCHERICHIA_COLI      RNEKLTQLDKFRFAPQGESLRTNQGVKISDNQNSLKSGARGSTLLEDFILREKITHFDHE

                                         130       140       150       160       170       180
P21179_ESCHERICHIA_COLI          RIPERIVHARGSAAHGYFQPYKSLSDITKADFLSDPNKITPVFVRFSTVQGGAGSADTVR
A0A6I8WFM0_ESCHERICHIA_COLI      RIPERVVHARGTGAHGYFQVYESLASYTTAEFLQDPSVKTPVFVRFSTVQGSRGSADTVR

                                         190       200       210       220       230       240
P21179_ESCHERICHIA_COLI          DIRGFATKFYTEEGIFDLVGNNTPIFFIQDAHKFPDFVHAVKPEPHWAIPQGQSAHDTFW
A0A6I8WFM0_ESCHERICHIA_COLI      DIRGWATKFYTKEGTFDLVGNNTPVFFIQDAIKFPDFVHAVKPEPHNEIPQGQSAHDTFW

                                         250       260       270       280       290       300
P21179_ESCHERICHIA_COLI          DYVSLQPETLHNVMWAMSDRGIPRSYRTMEGFGIHTFRLINAEGKATFVRFHWKPLAGKA
A0A6I8WFM0_ESCHERICHIA_COLI      DYISLQPETLHNVMWVMSDRGIPRSYRMMEGFGIHTYKMINAEGQCHFIRFHWKPVYGVS

                                         310       320       330       340       350       360
P21179_ESCHERICHIA_COLI          SLVWDEAQKLTGRDPDFHRRELWEAIEAGDFPEYELGFQLIPEEDEFKFDFDLLDPTKLI
A0A6I8WFM0_ESCHERICHIA_COLI      SLIWDEAQLLTGCDPDFHRRELWESIEAGDYPEYELGLQIIPEEDEHKFDFDILDPTKLI

                                         370       380       390       400       410       420
P21179_ESCHERICHIA_COLI          PEELVPVQRVGKMVLNRNPDNFFAENEQAAFHPGHIVPGLDFTNDPLLQGRLFSYTDTQI
A0A6I8WFM0_ESCHERICHIA_COLI      PESLVPVHLVGKMVLNRNPDNYFSETEQVAFCPGNIVPGIDFSDDPLLQGRLFSYIDTQI

                                         430       440       450       460       470       480
P21179_ESCHERICHIA_COLI          SRLGGPNFHEIPINRPTCPYHNFQRDGMHRMGIDTNPANYEPNSINDNWPRETPPGPKRG
A0A6I8WFM0_ESCHERICHIA_COLI      SRLGGVNFHEIPINKPICPFHNHQRDGMHRMSI.SGTANYEPNSINNNWPREAPP..TEG

                                         490       500       510       520       530       540
P21179_ESCHERICHIA_COLI          GFESYQERVEGNKVRERSPSFGEYYSHPRLFWLSQTPFEQRHIVDGFSFELSKVVRPYIR
A0A6I8WFM0_ESCHERICHIA_COLI      GFTTYPQPVNGYKSRKRSSTFIDFYSQPRLFWLSQTKVEQNHIVGGFSFELGKVVRPWIR

                                         550       560       570       580       590       600
P21179_ESCHERICHIA_COLI          ERVVDQLAHIDLTLAQAVAKNLGIELTDDQLNITPPPDVNGLKKDPSLSLYAIPDGDVKG
A0A6I8WFM0_ESCHERICHIA_COLI      ERVVNQLTYIDHQLAQSVADNLGIKLSQEQLKHPLPGPINGLSKDRSLSMYDGHHQILKS

                                         610       620       630       640       650       660
P21179_ESCHERICHIA_COLI          RVVAILLNDEVRSADLLAILKALKAKGVHAKLLYSRMGEVTADDGTVLPIAATFAGAPSL
A0A6I8WFM0_ESCHERICHIA_COLI      RQVAILAADGVCGDAIDNIMKTLKKYGVHGKIFAPHVGRITSLQGNEIEVNGTIEGNPSV

                                         670         680       690       700       710
P21179_ESCHERICHIA_COLI          TVDAVIVPCG..NIADIADNGDANYYLMEAYKHLKPIALAGDARKFKATIKIADQGEEGI
A0A6I8WFM0_ESCHERICHIA_COLI      MVDAVIIPDGEDSIDSLMKNGNAKHYVIQAFKHLKAIGLQGKAFKLYDALPLP.KPDEGI
```

```
                                 720       730       740       750
P21179_ESCHERICHIA_COLI          VEADSADGSFMDELLTLMAAHRVWSRIPKIDKIPA
A0A6I8WFM0_ESCHERICHIA_COLI      VVGDKA.ADLAEAFCNVMRGHRIWSRESVAQEIAG
```

```
                                    1        10        20        30        40        50        60
P21179_ESCHERICHIA_COLI             MSQHNEKNPHQHQSPLHDSSEAKPGMDSLAPEDGSHRPAAEPTPPGAQPTAPGSLKAPDT
A2A136_EXIGUOBACTERIUM_OXIDOTOLERANS ............................................................

                                             70        80        90       100       110       120
P21179_ESCHERICHIA_COLI             RNEKLNSLEDVRKGSENYALTTNQGVRIADDQNSLRAGSRGPTLLEDFILREKITHFDHE
A2A136_EXIGUOBACTERIUM_OXIDOTOLERANS ............MNENEKKLTTNQGVPIGDNQNSRTAGRRGPTLLEDYQLIEKIAHFDRE

                                            130       140       150       160       170       180
P21179_ESCHERICHIA_COLI             RIPERIVHARGSAAHGYFQPYKSLSDITKADFLSDPNKITPVFVRFSTVQGGAGSADTVR
A2A136_EXIGUOBACTERIUM_OXIDOTOLERANS RVPERVVHARGFGAHGVFKVKNSMKKYTKAAFLQEEGTEVPVFARFSTVIHGTHSPETLR

                                            190       200       210       220       230       240
P21179_ESCHERICHIA_COLI             DIRGFATKFYTEEGIFDLVGNNTPIFFIQDAHKFPDFVHAVKPEPHWAIPQGQSAHDTFW
A2A136_EXIGUOBACTERIUM_OXIDOTOLERANS DPRGFSVKFYTEEGNWDFVGNNLPVFFIRDAMKFPDMVHSLKPDPRTNIQDP....DRYW

                                            250       260       270       280       290       300
P21179_ESCHERICHIA_COLI             DYVSLQPETLHNVMWAMSDRGIPRSYRTMEGFGIHTFRLINAEGKATFVRFHWKPLAGKA
A2A136_EXIGUOBACTERIUM_OXIDOTOLERANS DFMTLRPESTNMLMHIFTDEGIPASYRKMRGSSVHSFKWVNAHGNTVYIKLRWVPKEGVH

                                            310       320       330       340       350       360
P21179_ESCHERICHIA_COLI             SLVWDEAQKLTGRDPDFHRRELWEAIEAGDFPEYELGFQLIPEEDEFKFDFDLLDPTKLI
A2A136_EXIGUOBACTERIUM_OXIDOTOLERANS NLSADEATEVQGKDFNHASNDTFQAIENGDFPEWDLFVQVLDPADVENFDFDPLDATKDW

                                            370       380       390       400       410       420
P21179_ESCHERICHIA_COLI             PEELVPVQRVGKMVLNRNPDNFFAENEQAAFHPGHIVPGLDFTNDPLLQGRLFSYTDTQI
A2A136_EXIGUOBACTERIUM_OXIDOTOLERANS FEDVIPFQHVGTMTLNKNVDNYFAETESVGFNPGVLVPGMLPSEDKLLQGRLFSYSDTQR

                                            430       440       450       460       470       480
P21179_ESCHERICHIA_COLI             SRLGGPNFHEIPINRPTCPYHNFQRDGMHRMGIDTNPANYEPNSINDNWPRETPPGPKRG
A2A136_EXIGUOBACTERIUM_OXIDOTOLERANS HRIG.PNYQQLPINCPFAQVNNYQRDGAMPFKQQTSSVNYEPNRYQDE.PKQTPEYTE..

                                            490       500       510       520       530       540
P21179_ESCHERICHIA_COLI             GFESYQERVEGNKVRERSPSFGEYYSHPRLFWLSQTPFEQRHIVDGFSFELSKVVRPYIR
A2A136_EXIGUOBACTERIUM_OXIDOTOLERANS DTQPLHDDIHGRLEIEKTNNFGQAGEVYR....RMTEEEQMALLNNLVNDLQQVRHENTV

                                            550       560       570       580       590       600
P21179_ESCHERICHIA_COLI             ERVVDQLAHIDLTLAQAVAKNLGIELTDDQLNITPPPDVNGLKKDPSLSLYAIPDGDVKG
A2A136_EXIGUOBACTERIUM_OXIDOTOLERANS LLAICNFYRADASLGEKLSEALNVDIKPFLQQMQK.........................

                                            610       620       630       640       650       660
P21179_ESCHERICHIA_COLI             RVVAILLNDEVRSADLLAILKALKAKGVHAKLLYSRMGEVTADDGTVLPIAATFAGAPSL
A2A136_EXIGUOBACTERIUM_OXIDOTOLERANS ............................................................

                                            670       680       690       700       710       720
P21179_ESCHERICHIA_COLI             TVDAVIVPCGNIADIADNGDANYYLMEAYKHLKPIALAGDARKFKATIKIADQGEEGIVE
A2A136_EXIGUOBACTERIUM_OXIDOTOLERANS ............................................................
```

```
                                           730       740       750
P21179_ESCHERICHIA_COLI                 ADSADGSFMDELLTLMAAHRVWSRIPKIDKIPA
A2A136_EXIGUOBACTERIUM_OXIDOTOLERANS    .................................
```

```
                              1        10        20        30        40        50        60
P21179_ESCHERICHIA_COLI       MSQHNEKNPHQHQSPLHDSSEAKPGMDSLAPEDGSHRPAAEPTPPGAQPTAPGSLKAPDT
C1PHG1_KOMAGATAELLA_PASTORIS  ............................................................

                                       70        80        90                100
P21179_ESCHERICHIA_COLI       RNEKLNSLEDVRKGSENYALTTNQGVRIAD...........DQNSLRAGSRGPTLIEDFI
C1PHG1_KOMAGATAELLA_PASTORIS  .............MSQPPKWTTSNGAPVSDVFATERATFDNANHANNAPKVGPLLLQDFQ

                             110       120       130       140       150       160
P21179_ESCHERICHIA_COLI       LREKITHFDHERIPERIVHARGSAAHGYFQPYKSLSDITKADFLSDPNKITPVFVRFSTV
C1PHG1_KOMAGATAELLA_PASTORIS  LIDSLAHFDRERIPERVVHAKGAGAFGEFEVTDDISDVCAAKFLDTIGKKTRIFTRFSTV

                             170       180       190       200       210       220
P21179_ESCHERICHIA_COLI       QGGAGSADTVRDIRGFATKFYTEEGIFDLVGNNTPIFFIQDAHKFPDFVHAVKPEPHWAI
C1PHG1_KOMAGATAELLA_PASTORIS  GGEKGSADSARDPRGFSTKFYTEEGNLDLVYNNTPIFFIRDPSKFPHFIHTQKRNPATNL

                             230       240       250       260       270       280
P21179_ESCHERICHIA_COLI       PQGQSAHDTFWDYVSLQPETLHNVMWAMSDRGIPRSYRTMEGFGIHTFRLINAEGKATFV
C1PHG1_KOMAGATAELLA_PASTORIS  KDA....NMFWDYLVNNQESIHQVMYLFSDRGTPASLRKMNGYSGHTYKWYNKKGEWVYV

                             290       300       310       320       330       340
P21179_ESCHERICHIA_COLI       RFHWKPLAGKASLVWDEAQKLTGRDPDFHRRELWEAIEAGDFPEYELGFQLIPEEDEFKF
C1PHG1_KOMAGATAELLA_PASTORIS  QVHFKSDLGVVNFNNEEAGKLAGEDPDYHTGDLFNAIERGEYPSWTCYIQTMTQEQAAKQ

                             350       360       370       380       390       400
P21179_ESCHERICHIA_COLI       DFDLLDPTKLIPEELVPVQRVGKMVLNRNPDNFFAENEQAAFHPGHIVPGLDFTNDPLLQ
C1PHG1_KOMAGATAELLA_PASTORIS  PFSVFDLTKVWPHKDFPLRRFGKFTLNENPKNYFAEVEQAAFSPSHTIPSMQPSADPVLQ

                             410       420       430       440        450       460
P21179_ESCHERICHIA_COLI       GRLFSYTDTQISRLGGPNFHEIPINRPTCPYHNFQ.RDG.MHRMGIDTNPANYEPN....
C1PHG1_KOMAGATAELLA_PASTORIS  SRLFSYPDTHRHRLG.VNYQQIPVNCPVAPVFTPQMRDGSMTVNGNLGSTPNYKSSFCPF

                                        470       480       490       500       510
P21179_ESCHERICHIA_COLI       ......SINDNWPRETPPGPKRGGFESYQERVEGNKVRERSPSFGEYYSHPRLFW..LSQ
C1PHG1_KOMAGATAELLA_PASTORIS  STEAQIQTNSHTPEEV........LAAHTEKFHWGGILD.SKSYD..FEQPRALWKVFGK

                                520       530       540       550       560       570
P21179_ESCHERICHIA_COLI       TPFEQRHIVDGFSFELSKVVRPYIRERVVDQLAHIDLTLAQAVAKNLGIELTDDQLNITP
C1PHG1_KOMAGATAELLA_PASTORIS  TPGQQRNFCHNVAVHVA.AANHEIQDRVFEYFSKVYPEIGDQIRKEV.............

                                580       590       600       610       620       630
P21179_ESCHERICHIA_COLI       PPDVNGLKKDPSLSLYAIPDGDVKGRVVAILLNDEVRSADLLAILKALKAKGVHAKLLYS
C1PHG1_KOMAGATAELLA_PASTORIS  ..............LQLSPRGDSAARL.................................

                                640       650       660       670       680       690
P21179_ESCHERICHIA_COLI       RMGEVTADDGTVLPIAATFAGAPSLTVDAVIVPCGNIADIADNGDANYYLMEAYKHLKPI
C1PHG1_KOMAGATAELLA_PASTORIS  ............................................................
```

```
                                  700       710       720       730       740       750
P21179_ESCHERICHIA_COLI      ALAGDARKFKATIKIADQGEEGIVEADSADGSFMDELLTLMAAHRVWSRIPKIDKIPA
C1PHG1_KOMAGATAELLA_PASTORIS ..........................................................
```

```
                                 1       10        20        30        40        50        60
P21179_ESCHERICHIA_COLI          MSQHNEKNPHQHQSPLHDSSEAKPGMDSLAPEDGSHRPAAEPTPPGAQPTAPGSLKAPDT
D9N167_PENICILLIUM_JANTHINELLUM  .......QQFLSQFYLND..........................................

                                         70        80        90       100       110       120
P21179_ESCHERICHIA_COLI          RNEKLNSLEDVRKGSENYALTTNQGVRIADDQNSLRAGSRGPTLLEDFILREKITHFDHE
D9N167_PENICILLIUM_JANTHINELLUM  ...............QDVYLTSNVGGPI.QDENSLSAGQRGATLLQDFIFREKIQRFDHE

                                        130       140       150       160       170       180
P21179_ESCHERICHIA_COLI          RIPERIVHARGSAAHGYFQPYKSLSDITKADFLSDPNKITPVFVRFSTVQGGAGSADTVR
D9N167_PENICILLIUM_JANTHINELLUM  RVPERAVHARGTGAHGTFTSYGDWSNLTAASFLSAEGKETPMFTRFSTVAGSRGSADTAR

                                        190       200       210       220       230       240
P21179_ESCHERICHIA_COLI          DIRGFATKFYTEEGIFDLVGNNTPIFFIQDAHKFPDFVHAVKPEPHWAIPQGQSAHDTFW
D9N167_PENICILLIUM_JANTHINELLUM  DVHGFATRFYTDEGNFDIVGNNIPVFFIQDAILFPDLIHAVKPRGDNQIPQAATAHDSAW

                                        250       260       270       280       290       300
P21179_ESCHERICHIA_COLI          DYVSLQPETLHNVMWAMSDRGIPRSYRTMEGFGIHTFRLINAEGKATFVRFHWKPLAGKA
D9N167_PENICILLIUM_JANTHINELLUM  DFFSQQPSVLHTLLWAMAGHGIPRSFRHVNGFGVHTFRLVTDDGKTKLVKFHWKGLQGKA

                                        310       320       330       340       350       360
P21179_ESCHERICHIA_COLI          SLVWDEAQKLTGRDPDFHRRELWEAIEAGDFPEYELGFQLIPEEDEFKFDFDLLDPTKLI
D9N167_PENICILLIUM_JANTHINELLUM  SFVWEEAQQTAGKNADFMRQDLFQSIQAGRFPEWELGVQIMQEQDQLKFGFDLLDPTKIV

                                        370       380       390       400       410       420
P21179_ESCHERICHIA_COLI          PEELVPVQRVGKMVLNRNPDNFFAENEQAAFHPGHIVPGLDFTNDPLLQGRLFSYTDTQI
D9N167_PENICILLIUM_JANTHINELLUM  PEELVPVTILGKMQLNRNPMNYFAETEQVMFQPGHIVRGVDFTEDPLLQGRLFSYLDTQL

                                        430       440       450       460       470       480
P21179_ESCHERICHIA_COLI          SRLGGPNFHEIPINRPTCPYHNFQRDGMHRMGIDTNPANYEPNSINDNWPRETPPGPKRG
D9N167_PENICILLIUM_JANTHINELLUM  NRHGGPNFEQLPINRPRAPIHNNNRDGAGQMFIPLDPNAYSPNTENKGSPKQANETVGKG

                                        490       500       510       520       530       540
P21179_ESCHERICHIA_COLI          GFESYQERVEGNKVRERSPSFGEYYSHPRLFWLSQTPFEQRHIVDGFSFELSKVVRPYIR
D9N167_PENICILLIUM_JANTHINELLUM  FFTAPERTASGKLQRTLSTTFENNWSQPRLFWNSLVNAQKEFIVDAMRFETSNVSSSVVR

                                        550       560       570       580       590
P21179_ESCHERICHIA_COLI          ERVVDQLAHIDLTLAQAVAKNLGIELTDDQLNITPPPDVNGLKKDPSLSLYAIPDGDVK.
D9N167_PENICILLIUM_JANTHINELLUM  DDVIIQLNRISDNLATRVASAIGVE........APKPNSSFYHDNTTAHIGAFGEKLAKL

                                 600        610       620       630       640       650
P21179_ESCHERICHIA_COLI          .GRVVAIL..LNDEVRSADLLAILKALKAKGVHAKLLYSRMGEVTADDGTVLPIAATFAG
D9N167_PENICILLIUM_JANTHINELLUM  DGLKVGLLASVNKPASIAQGAKLQVALSSVGVDVVVVAERMAN.........NVDETYSA

                                  660       670                 680               690
P21179_ESCHERICHIA_COLI          APSLTVDAVIVPCG..........NIADIADNGDANYY........LMEAYKHLKPIALA
D9N167_PENICILLIUM_JANTHINELLUM  SDAVQFDAVVVADGAEGLFGADSFTVEPSAGSGASTLYPAGRPLNILLDAFRFGKTVGAL
```

```
                                  700       710       720       730       740       750
P21179_ESCHERICHIA_COLI           GDARKFKATIKIADQGEEGIVEADSADGSFMDELLTLMAAHRVWSRIPKIDKIPA
D9N167_PENICILLIUM_JANTHINELLUM   GSGSDALESGQISSE.RQGVYTGKNAGDAFAKDIKSGLSTFKFLDRFA.VDE...
```

```
                                  1        10        20        30        40        50        60
P21179_ESCHERICHIA_COLI           MSQHNEKNPHQHQSPLHDSSEAKPGMDSLAPEDGSHRPAAEPTPPGAQPTAPGSLKAPDT
M4GGR5_MYCOTHERMUS_THERMOPHILUS   MNRVT.............NLLAWAGAIGL..........AQATCPFADPAALYSRQDTTS

                                           70        80        90       100       110       120
P21179_ESCHERICHIA_COLI           RNEKLNSLEDVRKGSENYALTTNQGVRIADDQNSLRAGSRGPTLLEDFILREKITHFDHE
M4GGR5_MYCOTHERMUS_THERMOPHILUS   GQSPLAAYEV..DDSTGY.LTSDVGGPI.QDQTSLKAGIRGPTLLEDFMFRQKIQHFDHE

                                          130       140       150       160       170       180
P21179_ESCHERICHIA_COLI           RIPERIVHARGSAAHGYFQPYKSLSDITKADFLSDPNKITPVFVRFSTVQGGAGSADTVR
M4GGR5_MYCOTHERMUS_THERMOPHILUS   RVPERAVHARGAGAHGTFTSYADWSNITAASFLNATGKQTPVFVRFSTVAGSRGSADTAR

                                          190       200       210       220       230       240
P21179_ESCHERICHIA_COLI           DIRGFATKFYTEEGIFDLVGNNTPIFFIQDAHKFPDFVHAVKPEPHWAIPQGQSAHDTFW
M4GGR5_MYCOTHERMUS_THERMOPHILUS   DVHGFATRFYTDEGNFDIVGNNIPVFFIQDAIQFPDLIHSVKPRPDNEIPQAATAHDSAW

                                          250       260       270       280       290       300
P21179_ESCHERICHIA_COLI           DYVSLQPETLHNVMWAMSDRGIPRSYRTMEGFGIHTFRLINAEGKATFVRFHWKPLAGKA
M4GGR5_MYCOTHERMUS_THERMOPHILUS   DFFSQQPSTMHTLFWAMSGHGIPRSYRHMDGFGVHTFRFVKDDGSSKLIKWHFKSRQGKA

                                          310       320       330       340       350       360
P21179_ESCHERICHIA_COLI           SLVWDEAQKLTGRDPDFHRRELWEAIEAGDFPEYELGFQLIPEEDEFKFDFDLLDPTKLI
M4GGR5_MYCOTHERMUS_THERMOPHILUS   SLVWEEAQVLSGKNADFHRQDLWDAIESGNGPEWDVCVQIVDESQAQAFGFDLLDPTKII

                                          370       380       390       400       410       420
P21179_ESCHERICHIA_COLI           PEELVPVQRVGKMVLNRNPDNFFAENEQAAFHPGHIVPGLDFTNDPLLQGRLFSYTDTQI
M4GGR5_MYCOTHERMUS_THERMOPHILUS   PEEYAPLTKLGLLKLDRNPTNYFAETEQVMFQPGHIVRGIDFTEDPLLQGRLFSYLDTQL

                                          430       440       450       460       470       480
P21179_ESCHERICHIA_COLI           SRLGGPNFHEIPINRPTCPYHNFQRDGMHRMGIDTNPANYEPNSINDNWPRETPPGPKRG
M4GGR5_MYCOTHERMUS_THERMOPHILUS   NRNGGPNFEQLPINMPRVPIHNNNRDGAGQMFIHRNKYPYTPNTLNSGYPRQANQNAGRG

                                          490       500       510       520       530       540
P21179_ESCHERICHIA_COLI           GFESYQERVEGNKVRERSPSFGEYYSHPRLFWLSQTPFEQRHIVDGFSFELSKVVRPYIR
M4GGR5_MYCOTHERMUS_THERMOPHILUS   FFTAPGRTASGALVREVSPTFNDHWSQPRLFFNSLTPVEQQFLVNAMRFEISLVKSEEVK

                                          550         560       570         580       590
P21179_ESCHERICHIA_COLI           ERVVDQLAHI..DLTLAQAVAKNLGIELTDDQL...NITPPPDVNGLKKDPSLSLYAIPD
M4GGR5_MYCOTHERMUS_THERMOPHILUS   KNVLTQLNRVSHDVAVRVAAAIGLGAPDADDTYYHNNKTAGVSIVGSGPLPTIKTL....

                                     600       610       620         630       640       650
P21179_ESCHERICHIA_COLI           GDVKGRVVAILLNDEVRSADLLAILKA.LKAKGVHAKLLYSRMGEVTADDGTVLPIAATF
M4GGR5_MYCOTHERMUS_THERMOPHILUS   .....RVGILATTSESSALDQAAQLRTRLEKDGLVVTVVAETLRE.........GVDQTY

                                     660       670       680                690       700
P21179_ESCHERICHIA_COLI           AGAPSLTVDAVIVPCGNIADIADNGDANYY........LMEAYKHLKPIALAGDARKFKA
M4GGR5_MYCOTHERMUS_THERMOPHILUS   STADATGFDGVVVVDGAAALFASTASSPLFPTGRPLQIFVDAYRWGKPVGVCGG..KSSE
```

```
                                    710       720       730       740       750
P21179_ESCHERICHIA_COLI             TIKIADQGEEG..IVEADSADGSFMDELLTLMAAHRVWSRIPKIDKIPA
M4GGR5_MYCOTHERMUS_THERMOPHILUS     VLDAADVPEDGDGVYSEESVD.MFVEEFEKGLATFRFTDRFA.LDS...
```

```
                                  1        10        20        30        40        50        60
P21179_ESCHERICHIA_COLI           MSQHNEKNPHQHQSPLHDSSEAKPGMDSLAPEDGSHRPAAEPTPPGAQPTAPGSLKAPDT
M4GGR6_MYCOTHERMUS_THERMOPHILUS   MTC.....PFADPAALYSRQDTTSGQSPLAAYE...........................

                                           70        80        90       100       110       120
P21179_ESCHERICHIA_COLI           RNEKLNSLEDVRKGSENYALTTNQGVRIADDQNSLRAGSRGPTLLEDFILREKITHFDHE
M4GGR6_MYCOTHERMUS_THERMOPHILUS   .......VDD....STGY.LTSDVGGPI.QDQTSLKAGIRGPTLLEDFMFRQKIQHFDHE

                                          130       140       150       160       170       180
P21179_ESCHERICHIA_COLI           RIPERIVHARGSAAHGYFQPYKSLSDITKADFLSDPNKITPVFVRFSTVQGGAGSADTVR
M4GGR6_MYCOTHERMUS_THERMOPHILUS   RVPERAVNARGAGAHGTFTSYADWSNITAASFLNATGKQTPVFVRFSTVAGSRGSADTAR

                                          190       200       210       220       230       240
P21179_ESCHERICHIA_COLI           DIRGFATKFYTEEGIFDLVGNNTPIFFIQDAHKFPDFVHAVKPEPHWAIPQGQSAHDTFW
M4GGR6_MYCOTHERMUS_THERMOPHILUS   DVHGFATRFYTDEGNFDIVGNNIPVFFIQDAIQFPDLIHSVKPRPDNEIPQAATAHDSAW

                                          250       260       270       280       290       300
P21179_ESCHERICHIA_COLI           DYVSLQPETLHNVMWAMSDRGIPRSYRTMEGFGIHTFRLINAEGKATFVRFHWKPLAGKA
M4GGR6_MYCOTHERMUS_THERMOPHILUS   DFFSQQPSTMHTLFWAMSGHGIPRSYRHMDGFGVHTFRFVKDDGSSKLIKWHFKSRQGKA

                                          310       320       330       340       350       360
P21179_ESCHERICHIA_COLI           SLVWDEAQKLTGRDPDFHRRELWEAIEAGDFPEYELGFQLIPEEDEFKFDFDLLDPTKLI
M4GGR6_MYCOTHERMUS_THERMOPHILUS   SLVWEEAQVLSGKNADFHRQDLWDAIESGNGPEWDVCVQIVDESQAQAFGFDLLDPTKII

                                          370       380       390       400       410       420
P21179_ESCHERICHIA_COLI           PEELVPVQRVGKMVLNRNPDNFFAENEQAAFHPGHIVPGLDFTNDPLLQGRLFSYTDTQI
M4GGR6_MYCOTHERMUS_THERMOPHILUS   PEEYAPLTKLGLLKLDRNPTNYFAETEQVMFQPGHIVRGIDFTEDPLLQGRLFSYLDTQL

                                          430       440       450       460       470       480
P21179_ESCHERICHIA_COLI           SRLGGPNFHEIPINRPTCPYHNFQRDGMHRMGIDTNPANYEPNSINDNWPRETPPGPKRG
M4GGR6_MYCOTHERMUS_THERMOPHILUS   NRNGGPNFEQLPINMPRVPIHNNNRDGAGQMFIHRNKYPYTPNTLNSGYPRQANQNAGRG

                                          490       500       510       520       530       540
P21179_ESCHERICHIA_COLI           GFESYQERVEGNKVRERSPSFGEYYSHPRLFWLSQTPFEQRHIVDGFSFELSKVVRPYIR
M4GGR6_MYCOTHERMUS_THERMOPHILUS   FFTAPGRTASGALVREVSPTFNDHWSQPRLFFNSLTPVEQQFLVNAMRFEISLVKSEEVK

                                          550         560       570          580       590
P21179_ESCHERICHIA_COLI           ERVVDQLAHI..DLTLAQAVAKNLGIELTDDQL...NITPPPDVNGLKKDPSLSLYAIPD
M4GGR6_MYCOTHERMUS_THERMOPHILUS   KNVLTQLNRVSHDVAVRVAAAIGLGAPDADDTYYHNNKTAGVSIVGSGPLPTIKTL....

                                       600       610       620        630       640       650
P21179_ESCHERICHIA_COLI           GDVKGRVVAILLNDEVRSADLLAILKA.LKAKGVHAKLLYSRMGEVTADDGTVLPIAATF
M4GGR6_MYCOTHERMUS_THERMOPHILUS   .....RVGILATTSESSALDQAAQLRTRLEKDGLVVTVVAETLRE.........GVDQTY

                                        660       670       680                690       700
P21179_ESCHERICHIA_COLI           AGAPSLTVDAVIVPCGNIADIADNGDANYY........LMEAYKHLKPIALAGDARKFKA
M4GGR6_MYCOTHERMUS_THERMOPHILUS   STADATGFDGVVVVDGAAALFASTASSPLFPTGRPLQIFVDAYRWGKPVGVCGG..KSSE
```

```
                                   710       720       730       740       750
P21179_ESCHERICHIA_COLI            TIKIADQGEEG..IVEADSADGSFMDELLTLMAAHRVWSRIPKIDKIPA
M4GGR6_MYCOTHERMUS_THERMOPHILUS    VLDAADVPEDGDGVYSEESVD.MFVEEFEKGLATFRFTDRFA.LDS...
```

```
                                 1        10        20        30        40        50        60
P21179_ESCHERICHIA_COLI          MSQHNEKNPHQHQSPLHDSSEAKPGMDSLAPEDGSHRPAAEPTPPGAQPTAPGSLKAPDT
M4GGR7_MYCOTHERMUS_THERMOPHILUS  MTC.....PFADPAALYSRQDTTSGQSPLAAYE...........................

                                          70        80        90       100       110       120
P21179_ESCHERICHIA_COLI          RNEKLNSLEDVRKGSENYALTTNQGVRIADDQNSLRAGSRGPTLLEDFILREKITHFDHE
M4GGR7_MYCOTHERMUS_THERMOPHILUS  .......VDD....STGY.LTSDVGGPI.QDQTSLKAGIRGPTLLEDFMFRQKIQHFDHE

                                         130       140       150       160       170       180
P21179_ESCHERICHIA_COLI          RIPERIVHARGSAAHGYFQPYKSLSDITKADFLSDPNKITPVFVRFSTVQGGAGSADTVR
M4GGR7_MYCOTHERMUS_THERMOPHILUS  RVPERAVHARGAGAHGTFTSYADWSNITAASFLNATGKQTPVFVRFSTVAGSRGSADTAR

                                         190       200       210       220       230       240
P21179_ESCHERICHIA_COLI          DIRGFATKFYTEEGIFDLVGNNTPIFFIQDAHKFPDFVHAVKPEPHWAIPQGQSAHDTFW
M4GGR7_MYCOTHERMUS_THERMOPHILUS  DVHGFATRFYTDEGNFDIVGNNIPVFFIQDAIQFPDLIHSVKPRPDNEIPQAATAHDSAW

                                         250       260       270       280       290       300
P21179_ESCHERICHIA_COLI          DYVSLQPETLHNVMWAMSDRGIPRSYRTMEGFGIHTFRLINAEGKATFVRFHWKPLAGKA
M4GGR7_MYCOTHERMUS_THERMOPHILUS  DFFSQQPSTMHTLFWAMSGHGIPRSYRHMDGFGVHTFRFVKDDGSSKLIKWHFKSRQGKA

                                         310       320       330       340       350       360
P21179_ESCHERICHIA_COLI          SLVWDEAQKLTGRDPDFHRRELWEAIEAGDFPEYELGFQLIPEEDEFKFDFDLLDPTKLI
M4GGR7_MYCOTHERMUS_THERMOPHILUS  SLVWEEAQVLSGKNADFHRQDLWDAIESGNGPEWDVCVQIVDESQAQAFGFDLLDPTKII

                                         370       380       390       400       410       420
P21179_ESCHERICHIA_COLI          PEELVPVQRVGKMVLNRNPDNFFAENEQAAFHPGHIVPGLDFTNDPLLQGRLFSYTDTQI
M4GGR7_MYCOTHERMUS_THERMOPHILUS  PEEYAPLTKLGLLKLDRNPTNYFAETEQVMFQPGHIVRGIDFTEDPLLQGRLFSYLDTQL

                                         430       440       450       460       470       480
P21179_ESCHERICHIA_COLI          SRLGGPNFHEIPINRPTCPYHNFQRDGMHRMGIDTNPANYEPNSINDNWPRETPPGPKRG
M4GGR7_MYCOTHERMUS_THERMOPHILUS  NRNGGPNFEQLPINMPRVPIHNNNRDGAGQMFIHRNKYPYTPNTLNSGYPRQANQNAGRG

                                         490       500       510       520       530       540
P21179_ESCHERICHIA_COLI          GFESYQERVEGNKVRERSPSFGEYYSHPRLFWLSQTPFEQRHIVDGFSFELSKVVRPYIR
M4GGR7_MYCOTHERMUS_THERMOPHILUS  FFTAPGRTASGALVREVSPTFNDHWSQPRLFFNSLTPVEQQFLVNAMRFEISLVKSEEVK

                                         550         560       570          580       590
P21179_ESCHERICHIA_COLI          ERVVDQLAHI..DLTLAQAVAKNLGIELTDDQL...NITPPPDVNGLKKDPSLSLYAIPD
M4GGR7_MYCOTHERMUS_THERMOPHILUS  KNVLTQLNRVSHDVAVRVAAAIGLGAPDADDTYYHNNKTAGVSIVGSGPLPTIKTL....

                                      600       610       620        630       640       650
P21179_ESCHERICHIA_COLI          GDVKGRVVAILLNDEVRSADLLAILKA.LKAKGVHAKLLYSRMGEVTADDGTVLPIAATF
M4GGR7_MYCOTHERMUS_THERMOPHILUS  .....RVGILATTSESSALDQAAQLRTRLEKDGLVVTVVAETLRE.........GVDQTY

                                       660       670       680               690       700
P21179_ESCHERICHIA_COLI          AGAPSLTVDAVIVPCGNIADIADNGDANYY........LMEAYKHLKPIALAGDARKFKA
M4GGR7_MYCOTHERMUS_THERMOPHILUS  STADATGFDGVVVVDGAAALFASTASSPLFPTGRPLQIFVDAYRWGKPVGVCGG..KSSE
```

```
                                     710        720        730        740        750
P21179_ESCHERICHIA_COLI              TIKIADQGEEG..IVEADSADGSFMDELLTLMAAHRVWSRIPKIDKIPA
M4GGR7_MYCOTHERMUS_THERMOPHILUS      VLDAADVPEDGDGVYSEESVD.MFVEEFEKGLATFRFTDRFA.LDS...
```

```
                                   1        10        20        30        40        50        60
P21179_ESCHERICHIA_COLI            MSQHNEKNPHQHQSPLHDSSEAKPGMDSLAPEDGSHRPAAEPTPPGAQPTAPGSLKAPDT
M4GGR8_MYCOTHERMUS_THERMOPHILUS    MTC.....PFADPAALYSRQDTTSGQSPLAAYE...........................

                                            70        80        90       100       110       120
P21179_ESCHERICHIA_COLI            RNEKLNSLEDVRKGSENYALTTNQGVRIADDQNSLRAGSRGPTLLEDFILREKITHFDHE
M4GGR8_MYCOTHERMUS_THERMOPHILUS    ......VDD....STGY.LTSDVGGPI.QDQTSLKAGIRGPTLLEDFMFRQKIQHFDHE

                                           130       140       150       160       170       180
P21179_ESCHERICHIA_COLI            RIPERIVHARGSAAHGYFQPYKSLSDITKADFLSDPNKITPVFVRFSTVQGGAGSADTVR
M4GGR8_MYCOTHERMUS_THERMOPHILUS    RVPERAVHARGAGAHGTFTSYADWSNITAASFLNATGKQTPVFVRFSTFAGSRGSADTAR

                                           190       200       210       220       230       240
P21179_ESCHERICHIA_COLI            DIRGFATKFYTEEGIFDLVGNNTPIFFIQDAHKFPDFVHAVKPEPHWAIPQGQSAHDTFW
M4GGR8_MYCOTHERMUS_THERMOPHILUS    DVHGFATRFYTDEGNFDIVGNNIPVFFIQDAIQFPDLIHSVKPRPDNEIPQAATAHDSAW

                                           250       260       270       280       290       300
P21179_ESCHERICHIA_COLI            DYVSLQPETLHNVMWAMSDRGIPRSYRTMEGFGIHTFRLINAEGKATFVRFHWKPLAGKA
M4GGR8_MYCOTHERMUS_THERMOPHILUS    DFFSQQPSTMHTLFWAMSGHGIPRSYRHMDGFGVHTFRFVKDDGSSKLIKWHFKSRQGKA

                                           310       320       330       340       350       360
P21179_ESCHERICHIA_COLI            SLVWDEAQKLTGRDPDFHRRELWEAIEAGDFPEYELGFQLIPEEDEFKFDFDLLDPTKLI
M4GGR8_MYCOTHERMUS_THERMOPHILUS    SLVWEEAQVLSGKNADFHRQDLWDAIESGNGPEWDVCVQIVDESQAQAFGFDLLDPTKII

                                           370       380       390       400       410       420
P21179_ESCHERICHIA_COLI            PEELVPVQRVGKMVLNRNPDNFFAENEQAAFHPGHIVPGLDFTNDPLLQGRLFSYTDTQI
M4GGR8_MYCOTHERMUS_THERMOPHILUS    PEEYAPLTKLGLLKLDRNPTNYFAETEQVMFQPGHIVRGIDFTEDPLLQGRLFSYLDTQL

                                           430       440       450       460       470       480
P21179_ESCHERICHIA_COLI            SRLGGPNFHEIPINRPTCPYHNFQRDGMHRMGIDTNPANYEPNSINDNWPRETPPGPKRG
M4GGR8_MYCOTHERMUS_THERMOPHILUS    NRNGGPNFEQLPINMPRVPIHNNNRDGAGQMFIHRNKYPYTPNTLNSGYPRQANQNAGRG

                                           490       500       510       520       530       540
P21179_ESCHERICHIA_COLI            GFESYQERVEGNKVRERSPSFGEYYSHPRLFWLSQTPFEQRHIVDGFSFELSKVVRPYIR
M4GGR8_MYCOTHERMUS_THERMOPHILUS    FFTAPGRTASGALVREVSPTFNDHWSQPRLFFNSLTPVEQQFLVNAMRFEISLVKSEEVK

                                           550          560       570           580       590
P21179_ESCHERICHIA_COLI            ERVVDQLAHI..DLTLAQAVAKNLGIELTDDQL...NITPPPDVNGLKKDPSLSLYAIPD
M4GGR8_MYCOTHERMUS_THERMOPHILUS    KNVLTQLNRVSHDVAVRVAAAIGLGAPDADDTYYHNNKTAGVSIVGSGPLPTIKTL....

                                       600       610       620         630       640       650
P21179_ESCHERICHIA_COLI            GDVKGRVVAILLNDEVRSADLLAILKA.LKAKGVHAKLLYSRMGEVTADDGTVLPIAATF
M4GGR8_MYCOTHERMUS_THERMOPHILUS    .....RVGILATTSESSALDQAAQLRTRLEKDGLVVTVVAETLRE.........GVDQTY

                                     660       670       680                690       700
P21179_ESCHERICHIA_COLI            AGAPSLTVDAVIVPCGNIADIADNGDANYY........LMEAYKHLKPIALAGDARKFKA
M4GGR8_MYCOTHERMUS_THERMOPHILUS    STADATGFDGVVVVDGAAALFASTASSPLFPTGRPLQIFVDAYRWGKPVGVCGG..KSSE
```

```
                                     710        720        730        740        750
P21179_ESCHERICHIA_COLI              TIKIADQGEEG..IVEADSADGSFMDELLTLMAAHRVWSRIPKIDKIPA
M4GGR8_MYCOTHERMUS_THERMOPHILUS      VLDAADVPEDGDGVYSEESVD.MFVEEFEKGLATFRFTDRFA.LDS...
```

```
                                  1        10        20        30        40        50        60
P21179_ESCHERICHIA_COLI           MSQHNEKNPHQHQSPLHDSSEAKPGMDSLAPEDGSHRPAAEPTPPGAQPTAPGSLKAPDT
O52762_PSEUDOMONAS_AERUGINOSA     ............................................................

                                            70        80        90       100       110       120
P21179_ESCHERICHIA_COLI           RNEKLNSLEDVRKGSENYALTTNQGVRIADDQNSLRAGSRGPTLLEDFILREKITHFDHE
O52762_PSEUDOMONAS_AERUGINOSA     .............MEEKTRLTTAAGAPVVDNQNVQTAGPRGPMLLQDVWFLEKLAHFDRE

                                           130       140       150       160       170       180
P21179_ESCHERICHIA_COLI           RIPERIVHARGSAAHGYFQPYKSLSDITKADFLSDPNKITPVFVRFSTVQGGAGSADTVR
O52762_PSEUDOMONAS_AERUGINOSA     VIPERRMHAKGSAAYGTFTVTHDITPYTRAKIFSQVGKKTDMFLRFSTVAGERGAADAER

                                           190       200       210       220       230
P21179_ESCHERICHIA_COLI           DIRGFATKFYTEEGIFDLVGNNTPIFFIQDAHKFPDFVHAVKPEPHWAIPQGQSAHDTF.
O52762_PSEUDOMONAS_AERUGINOSA     DIRGFSMRFYTEQGNWDLVGNNTPVFYLRDPLKFPDLNHVVKRDPRTNLRNA.....TFK

                               240       250       260       270       280       290
P21179_ESCHERICHIA_COLI           WDYVSLQPETLHNVMWAMSDRGIPRSYRTMEGFGIHTFRLINAEGKATFVRFHWKPLAGK
O52762_PSEUDOMONAS_AERUGINOSA     WDFFSHLPESLHQLTIDFSDRGLPKSYRHIHGFGSHTFSFINANNERFWVKFHFKTQQGI

                               300       310       320       330       340       350
P21179_ESCHERICHIA_COLI           ASLVWDEAQKLTGRDPDFHRRELWEAIEAGDFPEYELGFQLIPEEDEFKFDFDLLDPTKL
O52762_PSEUDOMONAS_AERUGINOSA     ENLTNAEAAEVIAQDRESSQRDLYESIEKGDFPRWKMYVQIMPEKEAATYRYNPFDLTKV

                               360       370       380       390       400       410
P21179_ESCHERICHIA_COLI           IPEELVPVQRVGKMVLNRNPDNFFAENEQAAFHPGHIVPGLDFTNDPLLQGRLFSYTDTQ
O52762_PSEUDOMONAS_AERUGINOSA     WPHGDYPLIEVGFFELNRNPDNYFAEVEQAAFTPANVVPGIGFSPDKMLQGRLFSYGDAH

                               420       430       440       450           460       470
P21179_ESCHERICHIA_COLI           ISRLGGPNFHEIPINRPTCPYHNFQRDGMHRMGIDTNPAN....YEPNSINDNWPRET..
O52762_PSEUDOMONAS_AERUGINOSA     RYRLG.VNHHQIPVNAARCPHQVYHRDG..GMRVDGNNAHQRVTYEPNSFNQ.WQEQPDF

                                         480       490       500       510       520       530
P21179_ESCHERICHIA_COLI           ..PPGPKRGGFESYQERVEGNKVRERSPSFGEYYSHPRLFWLSQTPFEQRHIVDGFSFEL
O52762_PSEUDOMONAS_AERUGINOSA     SEPPLSLEGAADHWNHRVDD...........DYYSQPAALFHLFTDEQKQRLFANIAEDI

                                         540       550       560       570       580       590
P21179_ESCHERICHIA_COLI           SKVVRPYIRERVVDQLAHIDLTLAQAVAKNLGIELTDDQLNITPPPDVNGLKKDPSLSLY
O52762_PSEUDOMONAS_AERUGINOSA     RDVPEQ.IQRRQIGLFLKVDPAYGKGVADALGLKLD........................

                                         600       610       620       630       640       650
P21179_ESCHERICHIA_COLI           AIPDGDVKGRVVAILLNDEVRSADLLAILKALKAKGVHAKLLYSRMGEVTADDGTVLPIA
O52762_PSEUDOMONAS_AERUGINOSA     ............................................................

                                         660       670       680       690       700       710
P21179_ESCHERICHIA_COLI           ATFAGAPSLTVDAVIVPCGNIADIADNGDANYYLMEAYKHLKPIALAGDARKFKATIKIA
O52762_PSEUDOMONAS_AERUGINOSA     ............................................................
```

```
                                       720       730       740       750
                                         .         .         .         .
P21179_ESCHERICHIA_COLI         DQGEEGIVEADSADGSFMDELLTLMAAHRVWSRIPKIDKIPA
O52762_PSEUDOMONAS_AERUGINOSA   ..........................................
```

```
                          1        10        20        30        40        50        60
P21179_ESCHERICHIA_COLI   MSQHNEKNPHQHQSPLHDSSEAKPGMDSLAPEDGSHRPAAEPTPPGAQPTAPGSLKAPDT
P00432_BOS_TAURUS         .....................................................MADNRDP

                                   70        80        90       100       110       120
P21179_ESCHERICHIA_COLI   RNEKLNSLEDVRKGSENYALTTNQGVRIADDQNSLRAGSRGPTLLEDFILREKITHFDHE
P00432_BOS_TAURUS         ASDQMKHWKEQRAAQKPDVLTTGGGNPVGDKLNSLTVGPRGPLLVQDVVFTDEMAHFDRE

                                  130       140       150       160       170       180
P21179_ESCHERICHIA_COLI   RIPERIVHARGSAAHGYFQPYKSLSDITKADFLSDPNKITPVFVRFSTVQGGAGSADTVR
P00432_BOS_TAURUS         RIPERVVHAKGAGAFGYFEVTHDITRYSKAKVFEHIGKRTPIAVRFSTVAGESGSADTVR

                                  190       200       210       220         230
P21179_ESCHERICHIA_COLI   DIRGFATKFYTEEGIFDLVGNNTPIFFIQDAHKFPDFVHAVK..PEPHWAIPQGQSAHDT
P00432_BOS_TAURUS         DPRGFAVKFYTEDGNWDLVGNNTPIFFIRDALLFPSFIHSQKRNPQTHLKDP......DM

                         240       250       260       270       280       290
P21179_ESCHERICHIA_COLI   FWDYVSLQPETLHNVMWAMSDRGIPRSYRTMEGFGIHTFRLINAEGKATFVRFHWKPLAG
P00432_BOS_TAURUS         VWDFWSLRPESLHQVSFLFSDRGIPDGHRHMNGYGSHTFKLVNANGEAVYCKFHYKTDQG

                         300       310       320       330       340       350
P21179_ESCHERICHIA_COLI   KASLVWDEAQKLTGRDPDFHRRELWEAIEAGDFPEYELGFQLIPEEDEFKFDFDLLDPTK
P00432_BOS_TAURUS         IKNLSVEDAARLAHEDPDYGLRDLFNAIATGNYPSWTLYIQVMTFSEAEIFPFNPFDLTK

                         360       370       380       390       400       410
P21179_ESCHERICHIA_COLI   LIPEELVPVQRVGKMVLNRNPDNFFAENEQAAFHPGHIVPGLDFTNDPLLQGRLFSYTDT
P00432_BOS_TAURUS         VWPHGDYPLIPVGKLVLNRNPVNYFAEVEQLAFDPSNMPPGIEPSPDKMLQGRLFAYPDT

                         420       430       440          450       460       470
P21179_ESCHERICHIA_COLI   QISRLGGPNFHEIPINRPTCPYH....NFQRDG.MHRMGIDTNPANYEPNSINDNWPRET
P00432_BOS_TAURUS         HRHRLG.PNYLQIPVN...CPYRARVANYQRDGPMCMMDNQGGAPNYYPNSFSA..PEHQ

                                480       490       500       510       520       530
P21179_ESCHERICHIA_COLI   PPGPKRGGFESYQERVEGNKVRERSPSFGEYYSHPRLFWLSQTPFEQR.HIVDGFSFELS
P00432_BOS_TAURUS         PSALE......HRTHFSGDVQRFNSAN.DDNVTQVRTFYLKVLNEEQRKRLCENIAGHL.

                                  540       550       560       570       580       590
P21179_ESCHERICHIA_COLI   KVVRPYIRERVVDQLAHIDLTLAQAVAKNLGIELTDDQLNITPPPDVNGLKKDPSLSLYA
P00432_BOS_TAURUS         KDAQLFIQKKAVKNFSDVHPEYGSRIQALL......DKYNEEKPKN........AVHTYV

                                  600       610       620       630       640       650
P21179_ESCHERICHIA_COLI   IPDGDVKGRVVAILLNDEVRSADLLAILKALKAKGVHAKLLYSRMGEVTADDGTVLPIAA
P00432_BOS_TAURUS         QHGSHLSAREKANL..............................................

                                  660       670       680       690       700       710
P21179_ESCHERICHIA_COLI   TFAGAPSLTVDAVIVPCGNIADIADNGDANYYLMEAYKHLKPIALAGDARKFKATIKIAD
P00432_BOS_TAURUS         ............................................................
```

```
                                 720          730          740          750
P21179_ESCHERICHIA_COLI   QGEEGIVEADSADGSFMDELLTLMAAHRVWSRIPKIDKIPA
P00432_BOS_TAURUS         .........................................
```

```
                         1        10        20        30        40        50         60
P21179_ESCHERICHIA_COLI  MSQHNEKNPHQHQSPLHDSSEAKPGMDSLAPEDGSHRPAAEPTPPGAQPTAPGSLKAPDT
P04040_HOMO_SAPIENS      .....................................................MADSRDP

                                  70        80        90       100       110       120
P21179_ESCHERICHIA_COLI  RNEKLNSLEDVRKGSENYALTTNQGVRIADDQNSLRAGSRGPTLLEDFILREKITHFDHE
P04040_HOMO_SAPIENS      ASDQMQHWKEQRAAQKADVLTTGAGNPVGDKLNVITVGPRGPLLVQDVVFTDEMAHFDRE

                                 130       140       150       160       170       180
P21179_ESCHERICHIA_COLI  RIPERIVHARGSAAHGYFQPYKSLSDITKADFLSDPNKITPVFVRFSTVQGGAGSADTVR
P04040_HOMO_SAPIENS      RIPERVVHAKGAGAFGYFEVTHDITKYSKAKVFEHIGKKTPIAVRFSTVAGESGSADTVR

                                 190       200       210       220          230
P21179_ESCHERICHIA_COLI  DIRGFATKFYTEEGIFDLVGNNTPIFFIQDAHKFPDFVHAVK..PEPHWAIPQGQSAHDT
P04040_HOMO_SAPIENS      DPRGFAVKFYTEDGNWDLVGNNTPIFFIRDPILFPSFIHSQKRNPQTHLKDP......DM

                        240       250       260       270       280       290
P21179_ESCHERICHIA_COLI  FWDYVSLQPETLHNVMWAMSDRGIPRSYRTMEGFGIHTFRLINAEGKATFVRFHWKPLAG
P04040_HOMO_SAPIENS      VWDFWSLRPESLHQVSFLFSDRGIPDGHRHMNGYGSHTFKLVNANGEAVYCKFHYKTDQG

                        300       310       320       330       340       350
P21179_ESCHERICHIA_COLI  KASLVWDEAQKLTGRDPDFHRRELWEAIEAGDFPEYELGFQLIPEEDEFKFDFDLLDPTK
P04040_HOMO_SAPIENS      IKNLSVEDAARLSQEDPDYGIRDLFNAIATGKYPSWTFYIQVMTFNQAETFPFNPFDLTK

                        360       370       380       390       400       410
P21179_ESCHERICHIA_COLI  LIPEELVPVQRVGKMVLNRNPDNFFAENEQAAFHPGHIVPGLDFTNDPLLQGRLFSYTDT
P04040_HOMO_SAPIENS      VWPHKDYPLIPVGKLVLNRNPVNYFAEVEQIAFDPSNMPPGIEASPDKMLQGRLFAYPDT

                        420       430       440           450       460       470
P21179_ESCHERICHIA_COLI  QISRLGGPNFHEIPINRPTCPYH....NFQRDGMHRMGIDTNPA.NYEPNSINDNWPRET
P04040_HOMO_SAPIENS      HRHRLG.PNYLHIPVN...CPYRARVANYQRDGPMCMQDNQGGAPNYYPNSFGA..PEQQ

                             480       490        500       510       520
P21179_ESCHERICHIA_COLI  PPGPKRGGFESYQERVE.GNKVRERSPSFGEYYSHPRLFWLSQTPFEQR.........HI
P04040_HOMO_SAPIENS      P........SALEHSIQYSGEVRRFNTANDDNVTQVRAFYVNVLNEEQRKRLCENIAGHL

                             530       540       550       560       570       580
P21179_ESCHERICHIA_COLI  VDGFSFELSKVVRPYIRERVVDQLAHIDLTLAQAVAKNLGIELTDDQLNITPPPDVNGLK
P04040_HOMO_SAPIENS      KDAQIFIQKKAVKNFT.EVHPDYGSHIQALL.............DKYNAEKPKN.....

                             590       600       610       620       630       640
P21179_ESCHERICHIA_COLI  KDPSLSLYAIPDGDVKGRVVAILLNDEVRSADLLAILKALKAKGVHAKLLYSRMGEVTAD
P04040_HOMO_SAPIENS      ...AIHTFVQSGSHLAARREKANL....................................

                             650       660       670       680       690       700
P21179_ESCHERICHIA_COLI  DGTVLPIAATFAGAPSLTVDAVIVPCGNIADIADNGDANYYLMEAYKHLKPIALAGDARK
P04040_HOMO_SAPIENS      ............................................................
```

```
                           710       720       730       740       750
                             .         .         .         .         .
P21179_ESCHERICHIA_COLI  FKATIKIADQGEEGIVEADSADGSFMDELLTLMAAHRVWSRIPKIDKIPA
P04040_HOMO_SAPIENS      ..................................................
```

```
                                    1         10        20        30        40        50        60
P21179_ESCHERICHIA_COLI             MSQHNEKNPHQHQSPLHDSSEAKPGMDSLAPEDGSHRPAAEPTPPGAQPTAPGSLKAPDT
P15202_SACCHAROMYCES_CEREVISIAE     .....................................................MSKLGQE

                                              70        80        90       100       110       120
P21179_ESCHERICHIA_COLI             RNEKLNSLEDVRKGSENYALTTNQGVRIADDQNSLRAGSRGPTLLEDFILREKITHFDHE
P15202_SACCHAROMYCES_CEREVISIAE     KNEV..NYSDVR...EDRVVTNSTGNPINEPFVTQRIGEHGPLLLQDYNLIDSLAHFNRE

                                              130       140       150       160       170       180
P21179_ESCHERICHIA_COLI             RIPERIVHARGSAAHGYFQPYKSLSDITKADFLSDPNKITPVFVRFSTVQGGAGSADTVR
P15202_SACCHAROMYCES_CEREVISIAE     NIPQRNPHAHGSGAFGYFEVTDDITDICGSAMFSKIGKRTKCLTRFSTVGGDKGSADTVR

                                              190       200       210       220       230       240
P21179_ESCHERICHIA_COLI             DIRGFATKFYTEEGIFDLVGNNTPIFFIQDAHKFPDFVHAVKPEPHWAIPQGQSAHDTFW
P15202_SACCHAROMYCES_CEREVISIAE     DPRGFATKFYTEEGNLDWVYNNTPVFFIRDPSKFPHFIHTQKRNPQTNLRDA....DMFW

                                                 250       260       270       280       290
P21179_ESCHERICHIA_COLI             DYVSLQPET...LHNVMWAMSDRGIPRSYRTMEGFGIHTFRLINAEGKATFVRFHWKPLA
P15202_SACCHAROMYCES_CEREVISIAE     DFLTT.PENQVAIHQVMILFSDRGTPANYRSMHGYSGHTYKWSNKNGDWHYVQVHIKTDQ

                                    300       310       320       330       340       350
P21179_ESCHERICHIA_COLI             GKASLVWDEAQKLTGRDPDFHRRELWEAIEAGDFPEYELGFQLIPEEDEFKFDFDLLDPT
P15202_SACCHAROMYCES_CEREVISIAE     GIKNLTIEEATKIAGSNPDYCQQDLFEAIQNGNYPSWTVYIQTMTERDAKKLPFSVFDLT

                                    360       370       380       390       400       410
P21179_ESCHERICHIA_COLI             KLIPEELVPVQRVGKMVLNRNPDNFFAENEQAAFHPGHIVPGLDFTNDPLLQGRLFSYTD
P15202_SACCHAROMYCES_CEREVISIAE     KVWPQGQFPLRRVGKIVLNENPLNFFAQVEQAAFAPSTTVPYQEASADPVLQARLFSYAD

                                    420       430       440        450       460       470
P21179_ESCHERICHIA_COLI             TQISRLGGPNFHEIPINRPTCPYHN..FQ...RDG.MHRMGIDTNPANYEPNSINDNWPR
P15202_SACCHAROMYCES_CEREVISIAE     AHRYRLG.PNFHQIPVN...CPYASKFFNPAIRDGPMNVNGNFGSEPTYLANDKSYTYIQ

                                          480       490          500       510       520
P21179_ESCHERICHIA_COLI             ETPPGPKRGGFESYQERVEGNKVRER...SPSFGEYYSHPRLFW.LSQTPFEQRHIVDGF
P15202_SACCHAROMYCES_CEREVISIAE     QDRP......IQQHQEVWNGPAIPYHWATSPGDVDFVQARNLYRVLGKQPGQQKNLAYNI

                                    530       540       550       560       570       580
P21179_ESCHERICHIA_COLI             SFELSKVVRPYIRERVVDQLAHIDLTLAQAVAKNLGIELTDDQLNITPPPDVNGLKKDPS
P15202_SACCHAROMYCES_CEREVISIAE     GIHVEGAC.PQIQQRVYDMFARVDKGLSEAIKK............VAEAKHASELSSNSK

                                    590       600       610       620       630       640
P21179_ESCHERICHIA_COLI             LSLYAIPDGDVKGRVVAILLNDEVRSADLLAILKALKAKGVHAKLLYSRMGEVTADDGTV
P15202_SACCHAROMYCES_CEREVISIAE     F...........................................................

                                    650       660       670       680       690       700
P21179_ESCHERICHIA_COLI             LPIAATFAGAPSLTVDAVIVPCGNIADIADNGDANYYLMEAYKHLKPIALAGDARKFKAT
P15202_SACCHAROMYCES_CEREVISIAE     ............................................................
```

```
                                   710         720         730         740         750
P21179_ESCHERICHIA_COLI            IKIADQGEEGIVEADSADGSFMDELLTLMAAHRVWSRIPKIDKIPA
P15202_SACCHAROMYCES_CEREVISIAE    ..............................................
```

```
                           1        10        20        30        40        50        60
P21179_ESCHERICHIA_COLI    MSQHNEKNPHQHQSPLHDSSEAKPGMDSLAPEDGSHRPAAEPTPPGAQPTAPGSLKAPDT
P29422_MICROCOCCUS_LUTEUS  ............................................................

                                  70        80        90       100       110       120
P21179_ESCHERICHIA_COLI    RNEKLNSLEDVRKGSENYALTTNQGVRIADDQNSLRAGSRGPTLLEDFILREKITHFDHE
P29422_MICROCOCCUS_LUTEUS  .......MEHQKTTPHATGSTRQNGAPAVSDRQSLTVGSEGPIVLHDTHLLETHQHFNRM

                                 130       140       150       160       170       180
P21179_ESCHERICHIA_COLI    RIPERIVHARGSAAHGYFQPYKSLSDITKADFLSDPNKITPVFVRFSTVQGGAGSADTVR
P29422_MICROCOCCUS_LUTEUS  NIPERRPHAKGSGAFGEFEVTEDVSKYTKA.LVFQPGTKTETLLRFSTVAGELGSPDTWR

                                 190       200       210       220       230       240
P21179_ESCHERICHIA_COLI    DIRGFATKFYTEEGIFDLVGNNTPIFFIQDAHKFPDFVHAVKPEPHWAIPQGQSAHDTFW
P29422_MICROCOCCUS_LUTEUS  DVRGFALRFYTEEGNYDLVGNNTPIFFLRDPMKFTHFIRSQKRLPDSGLRDATMQ....W

                                 250       260       270       280       290       300
P21179_ESCHERICHIA_COLI    DYVSLQPETLHNVMWAMSDRGIPRSYRTMEGFGIHTFRLINAEGKATFVRFHWKPLAGKA
P29422_MICROCOCCUS_LUTEUS  DFWTNNPESAHQVTYLMGPRGLPRTWREMNGYGSHTYLWVNAQGEKHWVKYHFISQQGVH

                                 310       320       330       340       350       360
P21179_ESCHERICHIA_COLI    SLVWDEAQKLTGRDPDFHRRELWEAIEAGDFPEYELGFQLIPEEDEFKFDFDLLDPTKLI
P29422_MICROCOCCUS_LUTEUS  NLSNDEATKIAGENADFHRQDLFESIAKGDHPKWDLYIQAIPYEEGKTYRFNPFDLTKTI

                                 370       380       390       400       410       420
P21179_ESCHERICHIA_COLI    PEELVPVQRVGKMVLNRNPDNFFAENEQAAFHPGHIVPGLDFTNDPLLQGRLFSYTDTQI
P29422_MICROCOCCUS_LUTEUS  SQKDYPRIKVGTLTLNRNPENHFAQIESAAFSPSNTVPGIGLSPDRMLLGRAFAYHDAQL

                                 430       440       450       460       470       480
P21179_ESCHERICHIA_COLI    SRLGGPNFHEIPINRPTCPYHNFQRDGMHRMGIDTNPANYEPNSINDNWPRETPPGPKRG
P29422_MICROCOCCUS_LUTEUS  YRVGA.HVNQLPVNRPKNAVHNYAFEGQMWYDHTGDRSTYVPNSNGDSWSDET..GPVDD

                                  490       500       510       520       530
P21179_ESCHERICHIA_COLI    GFESYQERV.EGNKVRERSPSFGEYYSHPRLFWLSQTPFEQRHIVDGFSFELSKVVRPYI
P29422_MICROCOCCUS_LUTEUS  GWEADGTLTREAQALRADDDDFGQAGTLVREVFSDQ...ERDDFVETVAGAL.KGVRQDV

                           540       550       560       570       580       590
P21179_ESCHERICHIA_COLI    RERVVDQLAHIDLTLAQAVAKNLGIELTDDQLNITPPPDVNGLKKDPSLSLYAIPDGDVK
P29422_MICROCOCCUS_LUTEUS  QARAFEYWKNVDATIGQRI.........EDEVKRHEGDGIPGVEAGGEARM.........

                           600       610       620       630       640       650
P21179_ESCHERICHIA_COLI    GRVVAILLNDEVRSADLLAILKALKAKGVHAKLLYSRMGEVTADDGTVLPIAATFAGAPS
P29422_MICROCOCCUS_LUTEUS  ............................................................

                           660       670       680       690       700       710
P21179_ESCHERICHIA_COLI    LTVDAVIVPCGNIADIADNGDANYYLMEAYKHLKPIALAGDARKFKATIKIADQGEEGIV
P29422_MICROCOCCUS_LUTEUS  ............................................................
```

```
                          720       730       740       750
                            .         .         .         .
P21179_ESCHERICHIA_COLI   EADSADGSFMDELLTLMAAHRVWSRIPKIDKIPA
P29422_MICROCOCCUS_LUTEUS ..................................
```

```
                        1         10        20        30        40        50        60
P21179_ESCHERICHIA_COLI MSQHNEKNPHQHQSPLHDSSEAKPGMDSLAPEDGSHRPAAEPTPPGAQPTAPGSLKAPDT
P30263_PICHIA_ANGUSTA   ............................................................

                                  70        80        90                  100       110
P21179_ESCHERICHIA_COLI RNEKLNSLEDVRKGSENYALTTNQGVRIADDQNSLR..........AGSRGPTLLEDFIL
P30263_PICHIA_ANGUSTA   .............MSNPPVFTTSQGCPVSDPFTTQRIPLDSTGYKYAPPIGPLLLQDFKL

                                 120       130       140       150       160       170
P21179_ESCHERICHIA_COLI REKITHFDHERIPERIVHARGSAAHGYFQPYKSLSDITKADFLSDPNKITPVFVRFSTVQ
P30263_PICHIA_ANGUSTA   IDTLSHFDRERIPERVVHAKGAGAYGVFEVTDDITDVCSAKFLDTVGKKTRIFTRFSTVG

                                 180       190       200       210       220       230
P21179_ESCHERICHIA_COLI GGAGSADTVRDIRGFATKFYTEEGIFDLVGNNTPIFFIQDAHKFPDFVHAVKPEPHWAIP
P30263_PICHIA_ANGUSTA   GEKGSADTARDPRGFATKFYTEDGNLDLVYNNTPIFFIRDPIKFPHFIHTQKRNPATNLK

                                 240       250       260       270       280       290
P21179_ESCHERICHIA_COLI QGQSAHDTFWDYVSLQPETLHNVMWAMSDRGIPRSYRTMEGFGIHTFRLINAEGKATFVR
P30263_PICHIA_ANGUSTA   DP....NMFWDYLTANDESLHQVMYLFSNRGTPASYRTMNGYSGHTYKWYNSKGEWVYVQ

                                 300       310       320       330       340       350
P21179_ESCHERICHIA_COLI FHWKPLAGKASLVWDEAQKLTGRDPDFHRRELWEAIEAGDFPEYELGFQLIPEEDEFKFD
P30263_PICHIA_ANGUSTA   VHFIANQGVHNLLDEEAGRLAGEDPDHSTRDLWEAIEKGDYPSWECYIQTMTLEQSKKLP

                                 360       370       380       390       400       410
P21179_ESCHERICHIA_COLI FDLLDPTKLIPEELVPVQRVGKMVLNRNPDNFFAENEQAAFHPGHIVPGLDFTNDPLLQG
P30263_PICHIA_ANGUSTA   FSVFDLTKVWPHKDFPLRHFGRFTLNENPKNYYAETEQIAFSPSHTVPGMEPSNDPVLQS

                                 420       430         440       450       460
P21179_ESCHERICHIA_COLI RLFSYTDTQISRLGGPNFHEIPINRP..TCPYHNFQRDGMHRMGIDTNPANYEPNSIND.
P30263_PICHIA_ANGUSTA   RLFSYPDTHRHRLG.PNYHQIPVNCPLKSGSFNPINRDGP..MCVDGNLGG.TPNYANAY

                        470       480       490       500       510       520
P21179_ESCHERICHIA_COLI NWPRETPPGPKRGGFESYQERVEGNKVRERSPSFGEYYSHPRLFW..LSQTPFEQRHIVD
P30263_PICHIA_ANGUSTA   NCPIQYAVSPKASGNKP.DEKYTGEVVPYHWEHTDYDYFQPKMFWKVLGRTPGEQESLVK

                            530       540       550       560       570       580
P21179_ESCHERICHIA_COLI GFSFELSKVVRPYIRERVVDQLAHIDLTLAQAVAKNLGIELTDDQLNITPPPDVNGLKKD
P30263_PICHIA_ANGUSTA   NVANHVS.AADEFIQDRVYEYFSKAEPIIGDLIRKKV....QELKRKASSPSKI......

                            590       600       610       620       630       640
P21179_ESCHERICHIA_COLI PSLSLYAIPDGDVKGRVVAILLNDEVRSADLLAILKALKAKGVHAKLLYSRMGEVTADDG
P30263_PICHIA_ANGUSTA   ............................................................

                            650       660       670       680       690       700
P21179_ESCHERICHIA_COLI TVLPIAATFAGAPSLTVDAVIVPCGNIADIADNGDANYYLMEAYKHLKPIALAGDARKFK
P30263_PICHIA_ANGUSTA   ............................................................
```

```
                                710       720       730       740       750
P21179_ESCHERICHIA_COLI  ATIKIADQGEEGIVEADSADGSFMDELLTLMAAHRVWSRIPKIDKIPA
P30263_PICHIA_ANGUSTA    ................................................
```

```
                             1        10        20        30        40        50        60
P21179_ESCHERICHIA_COLI      MSQHNEKNPHQHQSPLHDSSEAKPGMDSLAPEDGSHRPAAEPTPPGAQPTAPGSLKAPDT
P42321_PROTEUS_MIRABILIS     ............................................................

                                      70        80        90       100       110       120
P21179_ESCHERICHIA_COLI      RNEKLNSLEDVRKGSENYALTTNQGVRIADDQNSLRAGSRGPTLLEDFILREKITHFDHE
P42321_PROTEUS_MIRABILIS     ..............MEKKKLTTAAGAPVVDNNNVITAGPRGPMLLQDVWFLEKLAHFDRE

                                     130       140       150       160       170       180
P21179_ESCHERICHIA_COLI      RIPERIVHARGSAAHGYFQPYKSLSDITKADFLSDPNKITPVFVRFSTVQGGAGSADTVR
P42321_PROTEUS_MIRABILIS     VIPERRMHAKGSGAFGTFTVTHDITKYTRAKIFSEVGKKTEMFARFSTVAGERGAADAER

                                     190       200       210       220       230       240
P21179_ESCHERICHIA_COLI      DIRGFATKFYTEEGIFDLVGNNTPIFFIQDAHKFPDFVHAVKPEPHWAIPQGQSAHDTFW
P42321_PROTEUS_MIRABILIS     DIRGFALKFYTEEGNWDMVGNNTPVFYLRDPLKFPDLNHIVKRDPRTNMRNMAYK....W

                                     250       260       270       280       290       300
P21179_ESCHERICHIA_COLI      DYVSLQPETLHNVMWAMSDRGIPRSYRTMEGFGIHTFRLINAEGKATFVRFHWKPLAGKA
P42321_PROTEUS_MIRABILIS     DFFSHLPESLHQLTIDMSDRGLPLSYRFVHGFGSHTYSFINKDNERFWVKFHFRCQQGIK

                                     310       320       330       340       350       360
P21179_ESCHERICHIA_COLI      SLVWDEAQKLTGRDPDFHRRELWEAIEAGDFPEYELGFQLIPEEDEFKFDFDLLDPTKLI
P42321_PROTEUS_MIRABILIS     NLMDDEAEALVGKDRESSQRDLFEAIERGDYPRWKLQIQIMPEKEASTVPYNPFDLTKVW

                                     370       380       390       400       410       420
P21179_ESCHERICHIA_COLI      PEELVPVQRVGKMVLNRNPDNFFAENEQAAFHPGHIVPGLDFTNDPLLQGRLFSYTDTQI
P42321_PROTEUS_MIRABILIS     PHADYPLMDVGYFELNRNPDNYFSDVEQAAFSPANIVPGISFSPDKMLQGRLFSYGDAHR

                                     430       440       450       460       470
P21179_ESCHERICHIA_COLI      SRLGGPNFHEIPINRPTCPYHNFQRDGMHRM.GIDTNPANYEPNS...INDNWPRETPPG
P42321_PROTEUS_MIRABILIS     YRLG.VNHHQIPVNAPKCPFHNYHRDGAMRVDGNSGNGITYEPNSGGVFQEQPDFKEPPL

                                480       490       500       510       520       530
P21179_ESCHERICHIA_COLI      PKRGGFESYQERVEGNKVRERSPSFGEYYSHPRLFWLSQTPFEQRHIVDGFSFELSKVVR
P42321_PROTEUS_MIRABILIS     SIEGAADHWNHREDE...........DYFSQPRALYELLSDDEHQRMFARIAGELSQASK

                                540       550       560       570       580       590
P21179_ESCHERICHIA_COLI      PYIRERVVDQLAHIDLTLAQAVAKNLGIELTDDQLNITPPPDVNGLKKDPSLSLYAIPDG
P42321_PROTEUS_MIRABILIS     E.TQQRQIDLFTKVHPEYGAGVEKAIKVLEGKDAK.........................

                                600       610       620       630       640       650
P21179_ESCHERICHIA_COLI      DVKGRVVAILLNDEVRSADLLAILKALKAKGVHAKLLYSRMGEVTADDGTVLPIAATFAG
P42321_PROTEUS_MIRABILIS     ............................................................

                                660       670       680       690       700       710
P21179_ESCHERICHIA_COLI      APSLTVDAVIVPCGNIADIADNGDANYYLMEAYKHLKPIALAGDARKFKATIKIADQGEE
P42321_PROTEUS_MIRABILIS     ............................................................
```

```
                          720       730       740       750
                            .         .         .         .
P21179_ESCHERICHIA_COLI   GIVEADSADGSFMDELLTLMAAHRVWSRIPKIDKIPA
P42321_PROTEUS_MIRABILIS  .....................................
```

```
                             1        10        20        30        40        50        60
P21179_ESCHERICHIA_COLI      MSQHNEKNPHQHQSPLHDSSEAKPGMDSLAPEDGSHRPAAEPTPPGAQPTAPGSLKAPDT
P46206_PSEUDOMONAS_SYRINGAE  ....................................MPLLNWSRHMVCLTAAGLITVPTV

                                      70        80        90       100       110
P21179_ESCHERICHIA_COLI      RNEKLNSLEDVRKGSENYA...LTTNQGVRIADDQNSLRAGSRGPTLLEDFILREKITHF
P46206_PSEUDOMONAS_SYRINGAE  .................YATDTLTRDNGAVVGDNQNSQTAGAQGPVLLQDVQLLQKLQRF

                             120       130       140       150       160       170
P21179_ESCHERICHIA_COLI      DHERIPERIVHARGSAAHGYFQPYKSLSDITKADFLSDPNKITPVFVRFSTVQGGAGSAD
P46206_PSEUDOMONAS_SYRINGAE  DRERIPERVVHARGTGVKGEFTASADISDLSKATVFKSGEK.TPVFVRFSSVVHGNHSPE

                             180       190       200       210       220       230
P21179_ESCHERICHIA_COLI      TVRDIRGFATKFYTEEGIFDLVGNNTPIFFIQDAHKFPDFVHAVKPEPHWAIPQGQSAHD
P46206_PSEUDOMONAS_SYRINGAE  TLRDPHGFATKFYTADGNWDLVGNNFPTFFIRDAIKFPDMVHAFKPDPRTNLDNDSRRFD

                             240       250       260       270       280       290
P21179_ESCHERICHIA_COLI      TFWDYVSLQPETLHNVMWAMSDRGIPRSYRTMEGFGIHTFRLINAEGKATFVRFHWKPLA
P46206_PSEUDOMONAS_SYRINGAE  TF....SHVPEATRTLTLLYSNEGTPAGYRFMDGNGVHAYKLVNAKGEVHYVKFHWKSLQ

                             300       310       320       330       340       350
P21179_ESCHERICHIA_COLI      GKASLVWDEAQKLTGRDPDFHRRELWEAIEAGDFPEYELGFQLIPEEDEFKFDFDLLDPT
P46206_PSEUDOMONAS_SYRINGAE  GIKNLDPKEVAQVQSKDYSHLTNDLVGAIKKGDFPKWDLYVQVLKPEELAKFDFDPLDAT

                             360       370       380       390       400       410
P21179_ESCHERICHIA_COLI      KLIPEELVPVQRVGKMVLNRNPDNFFAENEQAAFHPGHIVPGLDFTNDPLLQGRLFSYTD
P46206_PSEUDOMONAS_SYRINGAE  KIWPD..VPEKKIGQMVLNKNVDNFFQETEQVAMAPANLVPGIEPSEDRLLQGRVFSYAD

                             420       430       440       450       460       470
P21179_ESCHERICHIA_COLI      TQISRLGGPNFHEIPINRPTCPYHNFQRDGMHRMGIDTNPANYEPNSINDNWPRETPPGP
P46206_PSEUDOMONAS_SYRINGAE  TQMYRLGAIGL.SLPVNQPKVAVNNGNQDGALNTGHTTSGVNYEPSRL............

                             480       490       500       510       520       530
P21179_ESCHERICHIA_COLI      KRGGFESYQERVEGNKVRERSPSFGEYYSHPRLFWLSQTPFEQRHIVDGFSF.ELSKVVR
P46206_PSEUDOMONAS_SYRINGAE  ........EPRPADDKAR.........YSELPLSGTTQ....QAKITREQNFKQAGDLFR

                               540       550       560       570       580       590
P21179_ESCHERICHIA_COLI      PYIRERVVDQLAHIDLTLAQAVAKNLGIELTDDQLNITPPPDVNGLKKDPSLSLYAIPDG
P46206_PSEUDOMONAS_SYRINGAE  SYSAKEKTDLVQRFGESLA......................DTHTESKNIMLSVLYKEDR

                               600       610       620       630       640       650
P21179_ESCHERICHIA_COLI      DVKGRVVAILLNDEVRSADLLAILKALKAKGVHAKLLYSRMGEVTADDGTVLPIAATFAG
P46206_PSEUDOMONAS_SYRINGAE  HYGTRVAEVAKGDLSKVKSLAASLKD..................................

                               660       670       680       690       700       710
P21179_ESCHERICHIA_COLI      APSLTVDAVIVPCGNIADIADNGDANYYLMEAYKHLKPIALAGDARKFKATIKIADQGEE
P46206_PSEUDOMONAS_SYRINGAE  ............................................................
```

```
                                 720       730       740       750
                                   .         .         .         .
P21179_ESCHERICHIA_COLI       GIVEADSADGSFMDELLTLMAAHRVWSRIPKIDKIPA
P46206_PSEUDOMONAS_SYRINGAE   .....................................
```

```
                             1        10        20        30        40        50        60
P21179_ESCHERICHIA_COLI      MSQHNEKNPHQHQSPLHDSSEAKPGMDSLAPEDGSHRPAAEPTPPGAQPTAPGSLKAPDT
P77872_HELICOBACTER_PYLORI   ............................................................

                                      70        80        90       100       110       120
P21179_ESCHERICHIA_COLI      RNEKLNSLEDVRKGSENYALTTNQGVRIADDQNSLRAGSRGPTLLEDFILREKITHFDHE
P77872_HELICOBACTER_PYLORI   ............MVNKDVKQTTAFGAPVWDDNNVITAGPRGPVLLQSTWFLEKLAAFDRE

                                     130       140       150       160       170       180
P21179_ESCHERICHIA_COLI      RIPERIVHARGSAAHGYFQPYKSLSDITKADFLSDPNKITPVFVRFSTVQGGAGSADTVR
P77872_HELICOBACTER_PYLORI   RIPERVVHAKGSGAYGTFTVTKDITKYTKAKIFSKVGKKTECFFRFSTVAGERGSADAVR

                                     190       200       210       220       230       240
P21179_ESCHERICHIA_COLI      DIRGFATKFYTEEGIFDLVGNNTPIFFIQDAHKFPDFVHAVKPEPHWAIPQGQSAHDTFW
P77872_HELICOBACTER_PYLORI   DPRGFAMKYYTEEGNWDLVGNNTPVFFIRDAIKFPDFIHTQKRDPQTNLPN....HDMVW

                                     250       260       270       280       290       300
P21179_ESCHERICHIA_COLI      DYVSLQPETLHNVMWAMSDRGIPRSYRTMEGFGIHTFRLINAEGKATFVRFHWKPLAGKA
P77872_HELICOBACTER_PYLORI   DFWSNVPESLYQVTWVMSDRGIPKSFRHMDGFGSHTFSLINAKGERFWVKFHFHTMQGVK

                                     310       320       330       340       350       360
P21179_ESCHERICHIA_COLI      SLVWDEAQKLTGRDPDFHRRELWEAIEAGDFPEYELGFQLIPEEDEFKFDFDLLDPTKLI
P77872_HELICOBACTER_PYLORI   HLTNEEAAEVRKYDPDSNQRDLFNAIARGDFPKWKLSIQVMPEEDAKKYRFHPFDVTKIW

                                     370       380       390       400       410       420
P21179_ESCHERICHIA_COLI      PEELVPVQRVGKMVLNRNPDNFFAENEQAAFHPGHIVPGLDFTNDPLLQGRLFSYTDTQI
P77872_HELICOBACTER_PYLORI   YLQDYPLMEVGIVELNKNPENYFAEVEQAAFSPANVVPGIGYSPDRMLQGRLFSYGDTHR

                                     430       440       450       460       470       480
P21179_ESCHERICHIA_COLI      SRLGGPNFHEIPINRPTCPYHNFQRDGMHRMGIDTNPANYEPNSINDNWPRETPPGPKRG
P77872_HELICOBACTER_PYLORI   YRLG.VNYPQIPVNKPRCPFHSSSRDGYMQNGYYGSLQNYTPSSLPGYKEDKSARDPKFN

                                     490       500       510       520       530       540
P21179_ESCHERICHIA_COLI      GFESYQERVEGNKVRERSPSFGEYYSHPRLFWLSQTPFEQRHIVDGFSFELSKVVRPYIR
P77872_HELICOBACTER_PYLORI   LAHIEKEFEVWNWDYRADDS..DYYTQPGDYYRSLPADEKERLHDTIGESLAHVTHKEIV

                                     550       560       570       580       590       600
P21179_ESCHERICHIA_COLI      ERVVDQLAHIDLTLAQAVAKNLGIELTDDQLNITPPPDVNGLKKDPSLSLYAIPDGDVKG
P77872_HELICOBACTER_PYLORI   DKQLEHFKKADPKYAEGVKKAL..EKHQKMMKDMHGKDMHHTKKKK..............

                                     610       620       630       640       650       660
P21179_ESCHERICHIA_COLI      RVVAILLNDEVRSADLLAILKALKAKGVHAKLLYSRMGEVTADDGTVLPIAATFAGAPSL
P77872_HELICOBACTER_PYLORI   ............................................................

                                     670       680       690       700       710       720
P21179_ESCHERICHIA_COLI      TVDAVIVPCGNIADIADNGDANYYLMEAYKHLKPIALAGDARKFKATIKIADQGEEGIVE
P77872_HELICOBACTER_PYLORI   ............................................................
```

```
                                  730           740           750
P21179_ESCHERICHIA_COLI      ADSADGSFMDELLTLMAAHRVWSRIPKIDKIPA
P77872_HELICOBACTER_PYLORI   .................................
```

```
                              1        10        20        30        40        50        60
P21179_ESCHERICHIA_COLI       MSQHNEKNPHQHQSPLHDSSEAKPGMDSLAPEDGSHRPAAEPTPPGAQPTAPGSLKAPDT
Q3LSM1_ALIIVIBRIO_SALMONICIDA ............................................................

                                       70        80        90       100       110       120
P21179_ESCHERICHIA_COLI       RNEKLNSLEDVRKGSENYALTTNQGVRIADDQNSLRAGSRGPTLLEDFILREKITHFDHE
Q3LSM1_ALIIVIBRIO_SALMONICIDA ...............MSKKLTTAAGCPVAHNQNVQTAGKRGPQLLQDVWFLEKLAHFDRE

                                      130       140       150       160       170       180
P21179_ESCHERICHIA_COLI       RIPERIVHARGSAAHGYFQPYKSLSDITKADFLSDPNKITPVFVRFSTVQGGAGSADTVR
Q3LSM1_ALIIVIBRIO_SALMONICIDA VIPERRMHAKGSGAYGTFTVTHDITKYTKAKIFSDIGKKTDMFARFSTVAGERGAADAER

                                      190       200       210       220       230       240
P21179_ESCHERICHIA_COLI       DIRGFATKFYTEEGIFDLVGNNTPIFFIQDAHKFPDFVHAVKPEPHWAIPQGQSAHDTFW
Q3LSM1_ALIIVIBRIO_SALMONICIDA DIRGFSLKFYTEEGNWDLAGNNTPVFFLRDPLKFPDLNHAVKRDPRTNMRSAKNN....W

                                      250       260       270       280       290       300
P21179_ESCHERICHIA_COLI       DYVSLQPETLHNVMWAMSDRGIPRSYRTMEGFGIHTFRLINAEGKATFVRFHWKPLAGKA
Q3LSM1_ALIIVIBRIO_SALMONICIDA DFWTSLPEALHQVTIVMSDRGIPATYRHMHGFGSHTFSFINSDNERYWVKFHFVSQQGIK

                                      310       320       330       340       350       360
P21179_ESCHERICHIA_COLI       SLVWDEAQKLTGRDPDFHRRELWEAIEAGDFPEYELGFQLIPEEDEFKFDFDLLDPTKLI
Q3LSM1_ALIIVIBRIO_SALMONICIDA NLSDAEAGELVGNDRESHQRDLLDSIDNQDFPKWTLKVQIMPEADAATVPYNPFDLTKVW

                                      370       380       390       400       410       420
P21179_ESCHERICHIA_COLI       PEELVPVQRVGKMVLNRNPDNFFAENEQAAFHPGHIVPGLDFTNDPLLQGRLFSYTDTQI
Q3LSM1_ALIIVIBRIO_SALMONICIDA PHKDYPLIEVGEFELNRNPQNYFAEVEQAAFNPANVVPGISFSPDKMLQGRLFAYGDAQR

                                      430       440       450       460       470
P21179_ESCHERICHIA_COLI       SRLGGPNFHEIPINRPTCPYHNFQRDGMHRM.GIDTNPANYEPNSINDNWPRET....PP
Q3LSM1_ALIIVIBRIO_SALMONICIDA YRLG.VNHQHIPVNAPRCPVHSYHRDGAMRVDGNFGSTLGYEPND.QGQWAEQPDFSEPP

                                 480       490       500       510       520       530
P21179_ESCHERICHIA_COLI       GPKRGGFESYQERVEGNKVRERSPSFGEYYSHPRLFWLSQTPFEQRHIVDGFSFELSKVV
Q3LSM1_ALIIVIBRIO_SALMONICIDA LNLDGAAAHWDHREDE..........DYFSQPGDLFGLMTAEKQAILFDNTARNLNGVP

                                 540       550       560       570       580       590
P21179_ESCHERICHIA_COLI       RPYIRERVVDQLAHIDLTLAQAVAKNLGIELTDDQLNITPPPDVNGLKKDPSLSLYAIPD
Q3LSM1_ALIIVIBRIO_SALMONICIDA KE.IQLRHVTHCYKADPAYGEGIGKLLGFDISEYNS........................

                                 600       610       620       630       640       650
P21179_ESCHERICHIA_COLI       GDVKGRVVAILLNDEVRSADLLAILKALKAKGVHAKLLYSRMGEVTADDGTVLPIAATFA
Q3LSM1_ALIIVIBRIO_SALMONICIDA ............................................................

                                 660       670       680       690       700       710
P21179_ESCHERICHIA_COLI       GAPSLTVDAVIVPCGNIADIADNGDANYYLMEAYKHLKPIALAGDARKFKATIKIADQGE
Q3LSM1_ALIIVIBRIO_SALMONICIDA ............................................................
```

```
                                  720       730       740       750
P21179_ESCHERICHIA_COLI           EGIVEADSADGSFMDELLTLMAAHRVWSRIPKIDKIPA
Q3LSM1_ALIIVIBRIO_SALMONICIDA     ......................................
```

```
                                1       10        20        30        40        50        60
P21179_ESCHERICHIA_COLI         MSQHNEKNPHQHQSPLHDSSEAKPGMDSLAPEDGSHRPAAEPTPPGAQPTAPGSLKAPDT
Q59337_DEINOCOCCUS_RADIODURANS  MSDENNKGV..........GTAVQGVGG..PRDGR..............TAP........

                                        70        80        90       100       110       120
P21179_ESCHERICHIA_COLI         RNEKLNSLEDVRKGSENYALTTNQGVRIADDQNSLRAGSRGPTLLEDFILREKITHFDHE
Q59337_DEINOCOCCUS_RADIODURANS  .............GEQGTTLTTRQGHPVHDNQNSRTVGSRGPMTLENYQFIEKLSHFDRE

                                       130       140            150       160       170
P21179_ESCHERICHIA_COLI         RIPERIVHARGSAAHGYFQPYKSLSD.....ITKADFLSDPNKITPVFVRFSTVQGGAGS
Q59337_DEINOCOCCUS_RADIODURANS  RIPERVVHARGVGAHGVFRATGKVGDEPVSKYTRAKLFQEDGKETPVFVRFSTVGHGTHS

                                  180       190       200       210       220       230
P21179_ESCHERICHIA_COLI         ADTVRDIRGFATKFYTEEGIFDLVGNNTPIFFIQDAHKFPDFVHAVKPEPHWAIPQGQSA
Q59337_DEINOCOCCUS_RADIODURANS  PETLRDPRGFAVKFYTEDGNWDLVGNNLKIFFIRDALKFPDLIHSQKPSPTTNI....QS

                                  240       250       260       270       280       290
P21179_ESCHERICHIA_COLI         HDTFWDYVSLQPETLHNVMWAMSDRGIPRSYRTMEGFGIHTFRLINAEGKATFVRFHWKP
Q59337_DEINOCOCCUS_RADIODURANS  QERIFDFFAGSPEATHMITLLYSPWGIPASYRFMQGSGVNTYKWVNDQGEGVLVKYHWEP

                                  300       310       320       330       340       350
P21179_ESCHERICHIA_COLI         LAGKASLVWDEAQKLTGRDPDFHRRELWEAIEAGDFPEYELGFQLIPEEDEFKFDFDLLD
Q59337_DEINOCOCCUS_RADIODURANS  VQGVRNLTQMQADEVQATNFNHATQDLHDAIERGDFPQWDLFVQIMEDGEHPELDFDPLD

                                  360       370       380       390       400       410
P21179_ESCHERICHIA_COLI         PTKLIPEELVPVQRVGKMVLNRNPDNFFAENEQAAFHPGHIVPGLDFTNDPLLQGRLFSY
Q59337_DEINOCOCCUS_RADIODURANS  DTKIWPREQFPWRHVGQMTLNRNPENVFAETEQAAFGTGVLVDGLDFSDDKMLQGRTFSY

                                  420       430       440       450            460       470
P21179_ESCHERICHIA_COLI         TDTQISRLGGPNFHEIPINRPTCPYHNFQRDGMHRMGIDT.....NPANYEPNSINDNWP
Q59337_DEINOCOCCUS_RADIODURANS  SDTQRYRVG.PNYLQLPINAPKKHVATNQRDGQMAYRVDTFEGQDQRVNYEPSLLSG..P

                                       480       490          500       510       520
P21179_ESCHERICHIA_COLI         RETPPGPKRGGFESYQERVEGNKVR...ERSPSFGEYYSHPRLFWLSQTPFEQRHIVDGF
Q59337_DEINOCOCCUS_RADIODURANS  KEAP...RRA..PEHTPRVEGNLVRAAIERPNPFGQAGMQYRNF....ADWERDELVSNL

                                530       540       550       560       570       580
P21179_ESCHERICHIA_COLI         SFELSKVVRPYIRERVVDQLAHIDLTLAQAVAKNLGIELTDDQLNITPPPDVNGLKKDPS
Q59337_DEINOCOCCUS_RADIODURANS  SGALAGVDKR.IQDKMLEYFTAADADYGQRVREGIQAKEAEMKGQKQEAP.VYGTEAS..

                                590       600       610       620       630       640
P21179_ESCHERICHIA_COLI         LSLYAIPDGDVKGRVVAILLNDEVRSADLLAILKALKAKGVHAKLLYSRMGEVTADDGTV
Q59337_DEINOCOCCUS_RADIODURANS  .SLY........................................................

                                650       660       670       680       690       700
P21179_ESCHERICHIA_COLI         LPIAATFAGAPSLTVDAVIVPCGNIADIADNGDANYYLMEAYKHLKPIALAGDARKFKAT
Q59337_DEINOCOCCUS_RADIODURANS  ............................................................
```

```
                                    710       720       730       740       750
                                      .         .         .         .         .
P21179_ESCHERICHIA_COLI             IKIADQGEEGIVEADSADGSFMDELLTLMAAHRVWSRIPKIDKIPA
Q59337_DEINOCOCCUS_RADIODURANS      ..............................................
```

```
                              1        10        20        30        40        50        60
P21179_ESCHERICHIA_COLI       MSQHNEKNPHQHQSPLHDSSEAKPGMDSLAPEDGSHRPAAEPTPPGAQPTAPGSLKAPDT
Q6CR58_KLUYVEROMYCES_LACTIS   ............................................................

                                       70        80        90       100       110       120
P21179_ESCHERICHIA_COLI       RNEKLNSLEDVRKGSENYALTTNQGVRIADDQNSLRAGSRGPTLLEDFILREKITHFDHE
Q6CR58_KLUYVEROMYCES_LACTIS   MGHPTNTA.DVRK...DRVVTNSQGAPINEPFATQRVGQHGPLLLQDFNLLDSLAHFNRE

                                      130       140       150       160       170       180
P21179_ESCHERICHIA_COLI       RIPERIVHARGSAAHGYFQPYKSLSDITKADFLSDPNKITPVFVRFSTVQGGAGSADTVR
Q6CR58_KLUYVEROMYCES_LACTIS   RIPERNPHAHGSGAFGYLEITDDITDVCGSAMFDTVGKRTRCLVRFSTVGGEKGSADTAR

                                      190       200       210       220       230       240
P21179_ESCHERICHIA_COLI       DIRGFATKFYTEEGIFDLVGNNTPIFFIQDAHKFPDFVHAVKPEPHWAIPQGQSAHDTFW
Q6CR58_KLUYVEROMYCES_LACTIS   DPRGFAIKFYSEEGNVDWVNNNTPVFFIRDPSKFPHFIHTQKRNPETNMKDA....DMFW

                                        250       260       270       280       290
P21179_ESCHERICHIA_COLI       DYVSLQPE..TLHNVMWAMSDRGIPRSYRTMEGFGIHTFRLINAEGKATFVRFHWKPLAG
Q6CR58_KLUYVEROMYCES_LACTIS   DFLTTEENQVAIHQVMILFSDRGTPASYRNMNSYSGHTYKWSNKQGEWRYVQVHLKTDQG

                              300       310       320       330       340       350
P21179_ESCHERICHIA_COLI       KASLVWDEAQKLTGRDPDFHRRELWEAIEAGDFPEYELGFQLIPEEDEFKFDFDLLDPTK
Q6CR58_KLUYVEROMYCES_LACTIS   IKNLNNEEATKLAGENPDYCQKDLFENIAKGNYPSWTLYIQTMTEEEAEKLPFSVFDLTK

                              360       370       380       390       400       410
P21179_ESCHERICHIA_COLI       LIPEELVPVQRVGKMVLNRNPDNFFAENEQAAFHPGHIVPGLDFTNDPLLQGRLFSYTDT
Q6CR58_KLUYVEROMYCES_LACTIS   VWPHKQFPLRRVGKMVLNENPENYFAQVEQAAFSPSHTVPYQEASADPVLQARLFSYPDA

                              420       430       440            450       460       470
P21179_ESCHERICHIA_COLI       QISRLGGPNFHEIPINRPTCPYHNFQ.....RDG.MHRMGIDTNPANYEPNSINDNWPRE
Q6CR58_KLUYVEROMYCES_LACTIS   HRYRLG.PNYSQIPVN...CPYASKVFNPAIRDGPMNVNGNLGKEPNYLSTSKKYQFIQQ

                                   480       490          500       510          520
P21179_ESCHERICHIA_COLI       TPPGPKRGGFESYQERVEGNKVRER...SPSFGEYYSHPRLF..WLSQTPFEQRHIVDGF
Q6CR58_KLUYVEROMYCES_LACTIS   SKP......IQQHQEVWSGPAMPVHWATSPGDIDFVQARDLYNKVLSKQPGQQKALAHNV

                                530       540       550       560       570       580
P21179_ESCHERICHIA_COLI       SFELSKVVRPYIRERVVDQLAHIDLTLAQAVAKNLGIELTDDQLNITPPPDVNGLKKDPS
Q6CR58_KLUYVEROMYCES_LACTIS   AVHVASAC.PEIQDRVFAMFARVDRGLSENIKK........EALSLSP.......RKAAA

                                590       600       610       620       630       640
P21179_ESCHERICHIA_COLI       LSLYAIPDGDVKGRVVAILLNDEVRSADLLAILKALKAKGVHAKLLYSRMGEVTADDGTV
Q6CR58_KLUYVEROMYCES_LACTIS   LNAKL.......................................................

                                650       660       670       680       690       700
P21179_ESCHERICHIA_COLI       LPIAATFAGAPSLTVDAVIVPCGNIADIADNGDANYYLMEAYKHLKPIALAGDARKFKAT
Q6CR58_KLUYVEROMYCES_LACTIS   ............................................................
```

```
                              710       720       730       740       750
                                .         .         .         .         .
P21179_ESCHERICHIA_COLI       IKIADQGEEGIVEADSADGSFMDELLTLMAAHRVWSRIPKIDKIPA
Q6CR58_KLUYVEROMYCES_LACTIS   ..............................................
```

```
                             1        10        20        30        40        50        60
P21179_ESCHERICHIA_COLI      MSQHNEKNPHQHQSPLHDSSEAKPGMDSLAPEDGSHRPAAEPTPPGAQPTAPGSLKAPDT
Q834P5_ENTEROCOCCUS_FAECALIS ............................................................

                                      70        80        90       100       110       120
P21179_ESCHERICHIA_COLI      RNEKLNSLEDVRKGSENYALTTNQGVRIADDQNSLRAGSRGPTLLEDFILREKITHFDHE
Q834P5_ENTEROCOCCUS_FAECALIS ..............MKNQHLTTSQGSPVGDNQNSLTAGEFGPVLIQDVHLLEKLAHFNRE

                                     130       140       150       160       170       180
P21179_ESCHERICHIA_COLI      RIPERIVHARGSAAHGYFQPYKSLSDITKADFLSDPNKITPVFVRFSTVQGGAGSADTVR
Q834P5_ENTEROCOCCUS_FAECALIS RVPERVVHAKGAGAHGIFKVSQSMAQYTKADFLSEVGKETPLFARFSTVAGELGSSDTLR

                                     190       200       210       220       230       240
P21179_ESCHERICHIA_COLI      DIRGFATKFYTEEGIFDLVGNNTPIFFIQDAHKFPDFVHAVKPEPHWAIPQGQSAHDTFW
Q834P5_ENTEROCOCCUS_FAECALIS DPRGFALKFYTDEGNYDLVGNNTPIFFIRDAIKFPDFIHSQKRNPRTHLKSPEAV....W

                                     250       260       270       280       290       300
P21179_ESCHERICHIA_COLI      DYVSLQPETLHNVMWAMSDRGIPRSYRTMEGFGIHTFRLINAEGKATFVRFHWKPLAGKA
Q834P5_ENTEROCOCCUS_FAECALIS DFWSHSPESLHQVTILMSDRGIPLSFRHMHGFGSHTFKWVNAAGEVFFVKYHFKTNQGIK

                                     310       320       330       340       350       360
P21179_ESCHERICHIA_COLI      SLVWDEAQKLTGRDPDFHRRELWEAIEAGDFPEYELGFQLIPEEDEFKFDFDLLDPTKLI
Q834P5_ENTEROCOCCUS_FAECALIS NLESQLAEEIAGKNPDFHIEDLHNAIENQEFPSWTLSVQIIPYADALTMKETLFDVTKTV

                                     370       380       390       400       410       420
P21179_ESCHERICHIA_COLI      PEELVPVQRVGKMVLNRNPDNFFAENEQAAFHPGHIVPGLDFTNDPLLQGRLFSYTDTQI
Q834P5_ENTEROCOCCUS_FAECALIS SQKEYPLIEVGTMTLNRNPENYFAEVEQVTFSPGNFVPGIEASPDKLLQGRLFAYGDAHR

                                     430       440       450       460       470       480
P21179_ESCHERICHIA_COLI      SRLGGPNFHEIPINRPTCPYHNFQRDGMHRMGIDTNPANYEPNSINDNWPRETPPGPKRG
Q834P5_ENTEROCOCCUS_FAECALIS HRVGA.NSHQLPINQAKAPVNNYQKDGNMRFNNGNSEINYEPNSYTET.PKEDPTA.KIS

                                     490       500       510       520       530       540
P21179_ESCHERICHIA_COLI      GFESYQERVEGNKVRERSPSFGEYYSHPRLFWLSQTPFEQRHIVDGFSFELSKVVRPYIR
Q834P5_ENTEROCOCCUS_FAECALIS SFE.....VEGN.VGNYSYNQDHFTQANALYNLLPSE.EKENLINNIAASLGQVKNQEII

                                     550       560       570       580       590       600
P21179_ESCHERICHIA_COLI      ERVVDQLAHIDLTLAQAVAKNLGIELTDDQLNITPPPDVNGLKKDPSLSLYAIPDGDVKG
Q834P5_ENTEROCOCCUS_FAECALIS ARQIDLFTRVNPEYGARVAQAIKQQA..................................

                                     610       620       630       640       650       660
P21179_ESCHERICHIA_COLI      RVVAILLNDEVRSADLLAILKALKAKGVHAKLLYSRMGEVTADDGTVLPIAATFAGAPSL
Q834P5_ENTEROCOCCUS_FAECALIS ............................................................

                                     670       680       690       700       710       720
P21179_ESCHERICHIA_COLI      TVDAVIVPCGNIADIADNGDANYYLMEAYKHLKPIALAGDARKFKATIKIADQGEEGIVE
Q834P5_ENTEROCOCCUS_FAECALIS ............................................................
```

```
                                  730           740           750
P21179_ESCHERICHIA_COLI        ADSADGSFMDELLTLMAAHRVWSRIPKIDKIPA
Q834P5_ENTEROCOCCUS_FAECALIS   .................................
```

```
                            1        10        20        30        40        50        60
P21179_ESCHERICHIA_COLI     MSQHNEKNPHQHQSPLHDSSEAKPGMDSLAPEDGSHRPAAEPTPPGAQPTAPGSLKAPDT
Q9C168_NEUROSPORA_CRASSA    .........................MSNIISQAG..QKAKE.....ALTSAPSSKKVDDL

                                    70        80        90        100           110
P21179_ESCHERICHIA_COLI     RNEKLNSLEDVRKGSENYALTTNQGVRIADDQNSLRAGSR...GPTLLEDFILREKITHF
Q9C168_NEUROSPORA_CRASSA    KNE.......FKETDKSARLTTDYGVKQTTADDWLRIVSDDKIGPSLLEDPFARERIMRF

                            120       130       140       150       160       170
P21179_ESCHERICHIA_COLI     DHERIPERIVHARGSAAHGYFQPYKSLSDITKADFLSDPNKITPVFVRFSTVQGGAGSAD
Q9C168_NEUROSPORA_CRASSA    DHERIPERVVHARGSGAFGKFKVYESASDLTMAPVLTDTSRETPVFVRFSTVLGSRGSAD

                            180       190       200       210       220       230
P21179_ESCHERICHIA_COLI     TVRDIRGFATKFYTEEGIFDLVGNNTPIFFIQDAHKFPDFVHAVKPEPHWAIPQGQSAHD
Q9C168_NEUROSPORA_CRASSA    TVRDVRGFAVKFYTEEGNWDLVGNNIPVFFIQDAIKFPDVIHAGKPEPHNEVPQAQSAHN

                            240       250       260       270       280       290
P21179_ESCHERICHIA_COLI     TFWDYVSLQPETLHNVMWAMSDRGIPRSYRTMEGFGIHTFRLINAEGKATFVRFHWKPLA
Q9C168_NEUROSPORA_CRASSA    NFWDFQFNHTEATHMFTWAMSDRAIPRSLRMMQGFGVNTYTLINAQGKRHFVKFHWTPEL

                            300       310       320       330       340       350
P21179_ESCHERICHIA_COLI     GKASLVWDEAQKLTGRDPDFHRRELWEAIEAGDFPEYELGFQLIPEEDEFKFDFDLLDPT
Q9C168_NEUROSPORA_CRASSA    GVHSLVWDEALKLAGQDPDFHRKDLWEAIENGAYPKWKFGIQAIAEEDEHKFDFDILDAT

                            360       370       380       390       400       410
P21179_ESCHERICHIA_COLI     KLIPEELVPVQRVGKMVLNRNPDNFFAENEQAAFHPGHIVPGLDFTNDPLLQGRLFSYTD
Q9C168_NEUROSPORA_CRASSA    KIWPEDLVPVRYIGEMELNRNPDEFFPQTEQIAFCTSHVVNGIGFSDDPLLQGRNFSYFD

                            420       430       440       450       460       470
P21179_ESCHERICHIA_COLI     TQISRLGGPNFHEIPINRPTCPYHNFQRDGMHRMGIDTNPANYEPNSINDNWPRETPPGP
Q9C168_NEUROSPORA_CRASSA    TQISRLG.VNFQELPINRPVCPVMNFNRDGAMRHTISRGTVNYYPNRF.DACP...PASL

                            480       490       500       510       520       530
P21179_ESCHERICHIA_COLI     KRGGFESYQERVEGNKVRERSPSFGEYYSHPRLFWLSQTPFEQRHIVDGFSFELSKVVRP
Q9C168_NEUROSPORA_CRASSA    KEGGYLEYAQKVAGIKARARSAKFKEHFSQAQLFYNSMSPIEKQHMINAFGFELDHCEDP

                            540       550       560       570       580       590
P21179_ESCHERICHIA_COLI     YIRERVVDQLAHIDLTLAQAVAKNLGIELTDDQLNITPPPDVNGLKKDPSLSLYAIPDGD
Q9C168_NEUROSPORA_CRASSA    VVYGRMVQRLADIDLGLAQTIAEMVGGEAP......TTTNHPNHGRKTINLSQTEFPPAT

                              600       610       620       630       640       650
P21179_ESCHERICHIA_COLI     ..VKGRVVAILLNDEVRSADLLAILKALKAKGVHAKLLYSRMGEVTADDGTVLPIAATFA
Q9C168_NEUROSPORA_CRASSA    PTIKSRRVAIIIADGYDNVAYDAAYAAISANQAIPLVIGPRRSKVTAANGSTVQPHHHLE

                              660       670         680       690       700
P21179_ESCHERICHIA_COLI     GAPSLTVDAVIVPCGNIA..DIADNGDANYYLMEAYKHLKPIALAGDARKFKATI.....
Q9C168_NEUROSPORA_CRASSA    GFRSTMVDAIFIPGGAKAAETLSKNGRALHWIREAFGHLKAIGATGEAVDLVAKAIALPQ
```

```
                                                    710       720       730       740
P21179_ESCHERICHIA_COLI      ..................KIADQGEEGIVEADSADGSFMDELLTLMAAHRVWSRIPKID
Q9C168_NEUROSPORA_CRASSA     VTVSSEAEVHESYGVVTLKKVKPESFTDAVKIAKGAAGFLGEFFYAIAQHRNWDR..ELD
```

```
                              750
P21179_ESCHERICHIA_COLI      KIPA....
Q9C168_NEUROSPORA_CRASSA     GLHSMIAY
```

```
                           1        10        20        30        40        50        60
P21179_ESCHERICHIA_COLI    MSQHNEKNPHQHQSPLHDSSEAKPGMDSLAPEDGSHRPAAEPTPPGAQPTAPGSLKAPDT
Q9C169_NEUROSPORA_CRASSA   MR.......................VNALLPLSGLIGTALAAC.PFADPSALGR.RAEGG

                                    70        80        90       100       110       120
P21179_ESCHERICHIA_COLI    RNEKLNSLEDVRKGSENYALTTNQGVRIADDQNSLRAGSRGPTLLEDFILREKITHFDHE
Q9C169_NEUROSPORA_CRASSA   EVDARQRLKEVEVDDNGQFMTTDFGGNI.EEQFSLKAGGRGSTLLEDFIFRQKLQHFDHE

                                   130       140       150       160       170       180
P21179_ESCHERICHIA_COLI    RIPERIVHARGSAAHGYFQPYKSLSDITKADFLSDPNKITPVFVRFSTVQGGAGSADTVR
Q9C169_NEUROSPORA_CRASSA   RIPERVVHARGAGAHGIFTSYGDWSNITAASFLGAKDKQTPVFVRFSTVAGSRGSADTAR

                                   190       200       210       220       230       240
P21179_ESCHERICHIA_COLI    DIRGFATKFYTEEGIFDLVGNNTPIFFIQDAHKFPDFVHAVKPEPHWAIPQGQSAHDTFW
Q9C169_NEUROSPORA_CRASSA   DVHGFATRFYTDEGNFDIVGNNIPVFFIQDAIRFPDLIHSVKPSPDNEVPQAATAHDSAW

                                   250       260       270       280       290       300
P21179_ESCHERICHIA_COLI    DYVSLQPETLHNVMWAMSDRGIPRSYRTMEGFGIHTFRLINAEGKATFVRFHWKPLAGKA
Q9C169_NEUROSPORA_CRASSA   DFFSSQPSALHTLFWAMSGNGIPRSYRHMDGFGIHTFRLVTEDGKSKLVKWHWKTKQGKA

                                   310       320       330       340       350       360
P21179_ESCHERICHIA_COLI    SLVWDEAQKLTGRDPDFHRRELWEAIEAGDFPEYELGFQLIPEEDEFKFDFDLLDPTKLI
Q9C169_NEUROSPORA_CRASSA   ALVWEEAQVLAGKNADFHRQDLWDAIESGNAPSWELAVQLIDEDKAQAYGFDLLDPTKFL

                                   370       380       390       400       410       420
P21179_ESCHERICHIA_COLI    PEELVPVQRVGKMVLNRNPDNFFAENEQAAFHPGHIVPGLDFTNDPLLQGRLFSYTDTQI
Q9C169_NEUROSPORA_CRASSA   PEEFAPLQVLGEMTLNRNPMNYFAETEQISFQPGHIVRGVDFTEDPLLQGRLYSYLDTQL

                                   430       440       450       460       470       480
P21179_ESCHERICHIA_COLI    SRLGGPNFHEIPINRPTCPYHNFQRDGMHRMGIDTNPANYEPNSINDNWPRETPPGPKRG
Q9C169_NEUROSPORA_CRASSA   NRHRGPNFEQLPINRPVSGVHNNHRDGQGQAWIHKNIHHYSPSYLNKGYPAQANQTVGRG

                                   490       500       510       520       530       540
P21179_ESCHERICHIA_COLI    GFESYQERVEGNKVRERSPSFGEYYSHPRLFWLSQTPFEQRHIVDGFSFELSKVVRPYIR
Q9C169_NEUROSPORA_CRASSA   FFTTPGRTASGVLNRELSATFDDHYTQPRLFFNSLTPVEQQFVINAIRFEASHVTNEQVK

                                   550       560       570           580       590
P21179_ESCHERICHIA_COLI    ERVVDQLAHIDLTLAQAVAKNLGIELTDDQLNITPPPDV....NGLKKDPSLSLYAIPDG
Q9C169_NEUROSPORA_CRASSA   KNVLEQLNKISNDVAKRVAVALGLE........APQPDPTYYHNNVTRGVSIFNESLPT.

                             600       610       620       630       640       650
P21179_ESCHERICHIA_COLI    DVKGRVVAILLNDEVRSADLLAILKALKAKGVHAKLLYSRMGEVTADDGTVLPIAATFAG
Q9C169_NEUROSPORA_CRASSA   .IATLRVGVLSTTKGGSLD........KAKALKEQLEKDGLKVTVIAEYLASGVDQTYSA

                             660                 670       680       690       700
P21179_ESCHERICHIA_COLI    APSLTVDAVIVP.........CGNIADIADNGDANYYLMEAYKHLKPIALAGDARKFKAT
Q9C169_NEUROSPORA_CRASSA   ADATAFDAVVVAEGAERVFSGKGAMSPLFPAGRPSQILTDGYRWGKPVAAVGSAKKALQS
```

```
                             710       720       730       740       750
P21179_ESCHERICHIA_COLI      IKIADQGEEGIVEADSADGSFMDELLTLMAAHRVWSRIPKIDKIPA
Q9C169_NEUROSPORA_CRASSA     IGVEEK.EAGVYAG..AQDEVIKGVEEGLKVFKFLERFAVDGDDEE
```

```
                                  1        10        20        30        40        50        60
P21179_ESCHERICHIA_COLI           MSQHNEKNPHQHQSPLHDSSEAKPGMDSLAPEDGSHRPAAEPTPPGAQPTAPGSLKAPDT
R4GRT6_MYCOTHERMUS_THERMOPHILUS   MTC.....PFADPAALYSRQDTTSGQSPLAAYE...........................

                                           70        80        90       100       110       120
P21179_ESCHERICHIA_COLI           RNEKLNSLEDVRKGSENYALTTNQGVRIADDQNSLRAGSRGPTLLEDFILREKITHFDHE
R4GRT6_MYCOTHERMUS_THERMOPHILUS   .......VDD....STGY.LTSDVGGPI.QDQTSLKAGIRGPTLLEDFMFRQKIQHFDHE

                                          130       140       150       160       170       180
P21179_ESCHERICHIA_COLI           RIPERIVHARGSAAHGYFQPYKSLSDITKADFLSDPNKITPVFVRFSTVQGGAGSADTVR
R4GRT6_MYCOTHERMUS_THERMOPHILUS   RVPERAVHARGAGAHGTFTSYADWSNITAASFLNATGKQTPVFVRFSTCAGSRGSADTAR

                                          190       200       210       220       230       240
P21179_ESCHERICHIA_COLI           DIRGFATKFYTEEGIFDLVGNNTPIFFIQDAHKFPDFVHAVKPEPHWAIPQGQSAHDTFW
R4GRT6_MYCOTHERMUS_THERMOPHILUS   DVHGFATRFYTDEGNFDIVGNNIPVFFIQDAIQFPDLIHSVKPRPDNEIPQAATAHDSAW

                                          250       260       270       280       290       300
P21179_ESCHERICHIA_COLI           DYVSLQPETLHNVMWAMSDRGIPRSYRTMEGFGIHTFRLINAEGKATFVRFHWKPLAGKA
R4GRT6_MYCOTHERMUS_THERMOPHILUS   DFFSQQPSTMHTLFWAMSGHGIPRSYRHMDGFGVHTFRFVKDDGSSKLIKWHFKSRQGKA

                                          310       320       330       340       350       360
P21179_ESCHERICHIA_COLI           SLVWDEAQKLTGRDPDFHRRELWEAIEAGDFPEYELGFQLIPEEDEFKFDFDLLDPTKLI
R4GRT6_MYCOTHERMUS_THERMOPHILUS   SLVWEEAQVLSGKNADFHRQDLWDAIESGNGPEWDVCVQIVDESQAQAFGFDLLDPTKII

                                          370       380       390       400       410       420
P21179_ESCHERICHIA_COLI           PEELVPVQRVGKMVLNRNPDNFFAENEQAAFHPGHIVPGLDFTNDPLLQGRLFSYTDTQI
R4GRT6_MYCOTHERMUS_THERMOPHILUS   PEEYAPLTKLGLLKLDRNPTNYFAETEQVMFQPGHIVRGIDFTEDPLLQGRLFSYLDTQL

                                          430       440       450       460       470       480
P21179_ESCHERICHIA_COLI           SRLGGPNFHEIPINRPTCPYHNFQRDGMHRMGIDTNPANYEPNSINDNWPRETPPGPKRG
R4GRT6_MYCOTHERMUS_THERMOPHILUS   NRNGGPNFEQLPINMPRVPIHNNNRDGAGQMFIHRNKYPYTPNTLNSGYPRQANQNAGRG

                                          490       500       510       520       530       540
P21179_ESCHERICHIA_COLI           GFESYQERVEGNKVRERSPSFGEYYSHPRLFWLSQTPFEQRHIVDGFSFELSKVVRPYIR
R4GRT6_MYCOTHERMUS_THERMOPHILUS   FFTAPGRTASGALVREVSPTFNDHWSQPRLFFNSLTPVEQQFLVNAMRFEISLVKSEEVK

                                          550         560       570          580       590
P21179_ESCHERICHIA_COLI           ERVVDQLAHI..DLTLAQAVAKNLGIELTDDQL...NITPPPDVNGLKKDPSLSLYAIPD
R4GRT6_MYCOTHERMUS_THERMOPHILUS   KNVLTQLNRVSHDVAVRVAAAIGLGAPDADDTYYHNNKTAGVSIVGSGPLPTIKTL....

                                       600       610       620        630       640       650
P21179_ESCHERICHIA_COLI           GDVKGRVVAILLNDEVRSADLLAILKA.LKAKGVHAKLLYSRMGEVTADDGTVLPIAATF
R4GRT6_MYCOTHERMUS_THERMOPHILUS   .....RVGILATTSESSALDQAAQLRTRLEKDGLVVTVVAETLRE.........GVDQTY

                                        660       670       680               690       700
P21179_ESCHERICHIA_COLI           AGAPSLTVDAVIVPCGNIADIADNGDANYY........LMEAYKHLKPIALAGDARKFKA
R4GRT6_MYCOTHERMUS_THERMOPHILUS   STADATGFDGVVVVDGAAALFASTASSPLFPTGRPLQIFVDAYRWGKPVGVCGG..KSSE
```

```
                                     710       720       730       740       750
P21179_ESCHERICHIA_COLI              TIKIADQGEEG..IVEADSADGSFMDELLTLMAAHRVWSRIPKIDKIPA
_R4GRT6_MYCOTHERMUS_THERMOPHILUS     VLDAADVPEDGDGVYSEESVD.MFVEEFEKGLATFRFTDRFA.LDS...
```

```
                                  1        10        20        30        40        50        60
P21179_ESCHERICHIA_COLI           MSQHNEKNPHQHQSPLHDSSEAKPGMDSLAPEDGSHRPAAEPTPPGAQPTAPGSLKAPDT
R4GRT7_MYCOTHERMUS_THERMOPHILUS   MTC.....PFADPAALYSRQDTTSGQSPLAAYE...........................

                                           70        80        90       100       110       120
P21179_ESCHERICHIA_COLI           RNEKLNSLEDVRKGSENYALTTNQGVRIADDQNSLRAGSRGPTLLEDFILREKITHFDHE
R4GRT7_MYCOTHERMUS_THERMOPHILUS   .......VDD....STGY.LTSDVGGPI.QDQTSLKAGIRGPTLLEDFMFRQKIQHFDHE

                                           130       140       150       160       170       180
P21179_ESCHERICHIA_COLI           RIPERIVHARGSAAHGYFQPYKSLSDITKADFLSDPNKITPVFVRFSTVQGGAGSADTVR
R4GRT7_MYCOTHERMUS_THERMOPHILUS   RVPERAVHARGAGAHGTFTSYADWSNITAASFLNATGKQTPVFVRFSTVAGSRGSADTAR

                                           190       200       210       220       230       240
P21179_ESCHERICHIA_COLI           DIRGFATKFYTEEGIFDLVGNNTPIFFIQDAHKFPDFVHAVKPEPHWAIPQGQSAHDTFW
R4GRT7_MYCOTHERMUS_THERMOPHILUS   DVHGFATRFYTDEGNFDIVGANIPVFFIQDAIQFPDLIHSVKPRPDNEIPQAATAHDSAW

                                           250       260       270       280       290       300
P21179_ESCHERICHIA_COLI           DYVSLQPETLHNVMWAMSDRGIPRSYRTMEGFGIHTFRLINAEGKATFVRFHWKPLAGKA
R4GRT7_MYCOTHERMUS_THERMOPHILUS   DFFSQQPSTMHTLFWAMSGHGIPRSYRHMDGFGVHTFRFVKDDGSSKLIKWHFKSRQGKA

                                           310       320       330       340       350       360
P21179_ESCHERICHIA_COLI           SLVWDEAQKLTGRDPDFHRRELWEAIEAGDFPEYELGFQLIPEEDEFKFDFDLLDPTKLI
R4GRT7_MYCOTHERMUS_THERMOPHILUS   SLVWEEAQVLSGKNADFHRQDLWDAIESGNGPEWDVCVQIVDESQAQAFGFDLLDPTKII

                                           370       380       390       400       410       420
P21179_ESCHERICHIA_COLI           PEELVPVQRVGKMVLNRNPDNFFAENEQAAFHPGHIVPGLDFTNDPLLQGRLFSYTDTQI
R4GRT7_MYCOTHERMUS_THERMOPHILUS   PEEYAPLTKLGLLKLDRNPTNYFAETEQVMFQPGHIVRGIDFTEDPLLQGRLFSYLDTQL

                                           430       440       450       460       470       480
P21179_ESCHERICHIA_COLI           SRLGGPNFHEIPINRPTCPYHNFQRDGMHRMGIDTNPANYEPNSINDNWPRETPPGPKRG
R4GRT7_MYCOTHERMUS_THERMOPHILUS   NRNGGPNFEQLPINMPRVPIHNNNRDGAGQMFIHRNKYPYTPNTLNSGYPRQANQNAGRG

                                           490       500       510       520       530       540
P21179_ESCHERICHIA_COLI           GFESYQERVEGNKVRERSPSFGEYYSHPRLFWLSQTPFEQRHIVDGFSFELSKVVRPYIR
R4GRT7_MYCOTHERMUS_THERMOPHILUS   FFTAPGRTASGALVREVSPTFNDHWSQPRLFFNSLTPVEQQFLVNAMRFEISLVKSEEVK

                                           550         560       570          580       590
P21179_ESCHERICHIA_COLI           ERVVDQLAHI..DLTLAQAVAKNLGIELTDDQL...NITPPPDVNGLKKDPSLSLYAIPD
R4GRT7_MYCOTHERMUS_THERMOPHILUS   KNVLTQLNRVSHDVAVRVAAAIGLGAPDADDTYYHNNKTAGVSIVGSGPLPTIKTL....

                                      600       610       620        630       640       650
P21179_ESCHERICHIA_COLI           GDVKGRVVAILLNDEVRSADLLAILKA.LKAKGVHAKLLYSRMGEVTADDGTVLPIAATF
R4GRT7_MYCOTHERMUS_THERMOPHILUS   .....RVGILATTSESSALDQAAQLRTRLEKDGLVVTVVAETLRE.........GVDQTY

                                        660       670       680               690       700
P21179_ESCHERICHIA_COLI           AGAPSLTVDAVIVPCGNIADIADNGDANYY........LMEAYKHLKPIALAGDARKFKA
R4GRT7_MYCOTHERMUS_THERMOPHILUS   STADATGFDGVVVVDGAAALFASTASSPLFPTGRPLQIFVDAYRWGKPVGVCGG..KSSE
```

```
                                  710       720       730       740       750
P21179_ESCHERICHIA_COLI           TIKIADQGEEG..IVEADSADGSFMDELLTLMAAHRVWSRIPKIDKIPA
R4GRT7_MYCOTHERMUS_THERMOPHILUS   VLDAADVPEDGDGVYSEESVD.MFVEEFEKGLATFRFTDRFA.LDS...
```

```
                                  1        10        20        30        40        50        60
P21179_ESCHERICHIA_COLI           MSQHNEKNPHQHQSPLHDSSEAKPGMDSLAPEDGSHRPAAEPTPPGAQPTAPGSLKAPDT
R4GRT8_MYCOTHERMUS_THERMOPHILUS   MTC.....PFADPAALYSRQDTTSGQSPLAAYE...........................

                                           70        80        90       100       110       120
P21179_ESCHERICHIA_COLI           RNEKLNSLEDVRKGSENYALTTNQGVRIADDQNSLRAGSRGPTLLEDFILREKITHFDHE
R4GRT8_MYCOTHERMUS_THERMOPHILUS   .......VDD....STGY.LTSDVGGPI.QDQTSLKAGIRGPTLLEDFMFRQKIQHFDHE

                                          130       140       150       160       170       180
P21179_ESCHERICHIA_COLI           RIPERIVHARGSAAHGYFQPYKSLSDITKADFLSDPNKITPVFVRFSTVQGGAGSADTVR
R4GRT8_MYCOTHERMUS_THERMOPHILUS   RVPERAVHARGAGAHGTFTSYADWSNITAASFLNATGKQTPVFVRFSTAAGSRGSADTAR

                                          190       200       210       220       230       240
P21179_ESCHERICHIA_COLI           DIRGFATKFYTEEGIFDLVGNNTPIFFIQDAHKFPDFVHAVKPEPHWAIPQGQSAHDTFW
R4GRT8_MYCOTHERMUS_THERMOPHILUS   DVHGFATRFYTDEGNFDIVGNNIPVFFIQDAIQFPDLIHSVKPRPDNEIPQAATAHDSAW

                                          250       260       270       280       290       300
P21179_ESCHERICHIA_COLI           DYVSLQPETLHNVMWAMSDRGIPRSYRTMEGFGIHTFRLINAEGKATFVRFHWKPLAGKA
R4GRT8_MYCOTHERMUS_THERMOPHILUS   DFFSQQPSTMHTLFWAMSGHGIPRSYRHMDGFGVHTFRFVKDDGSSKLIKWHFKSRQGKA

                                          310       320       330       340       350       360
P21179_ESCHERICHIA_COLI           SLVWDEAQKLTGRDPDFHRRELWEAIEAGDFPEYELGFQLIPEEDEFKFDFDLLDPTKLI
R4GRT8_MYCOTHERMUS_THERMOPHILUS   SLVWEEAQVLSGKNADFHRQDLWDAIESGNGPEWDVCVQIVDESQAQAFGFDLLDPTKII

                                          370       380       390       400       410       420
P21179_ESCHERICHIA_COLI           PEELVPVQRVGKMVLNRNPDNFFAENEQAAFHPGHIVPGLDFTNDPLLQGRLFSYTDTQI
R4GRT8_MYCOTHERMUS_THERMOPHILUS   PEEYAPLTKLGLLKLDRNPTNYFAETEQVMFQPGHIVRGIDFTEDPLLQGRLFSYLDTQL

                                          430       440       450       460       470       480
P21179_ESCHERICHIA_COLI           SRLGGPNFHEIPINRPTCPYHNFQRDGMHRMGIDTNPANYEPNSINDNWPRETPPGPKRG
R4GRT8_MYCOTHERMUS_THERMOPHILUS   NRNGGPNFEQLPINMPRVPIHNNNRDGAGQMFIHRNKYPYTPNTLNSGYPRQANQNAGRG

                                          490       500       510       520       530       540
P21179_ESCHERICHIA_COLI           GFESYQERVEGNKVRERSPSFGEYYSHPRLFWLSQTPFEQRHIVDGFSFELSKVVRPYIR
R4GRT8_MYCOTHERMUS_THERMOPHILUS   FFTAPGRTASGALVREVSPTFNDHWSQPRLFFNSLTPVEQQFLVNAMRFEISLVKSEEVK

                                          550         560       570          580       590
P21179_ESCHERICHIA_COLI           ERVVDQLAHI..DLTLAQAVAKNLGIELTDDQL...NITPPPDVNGLKKDPSLSLYAIPD
R4GRT8_MYCOTHERMUS_THERMOPHILUS   KNVLTQLNRVSHDVAVRVAAAIGLGAPDADDTYYHNNKTAGVSIVGSGPLPTIKTL....

                                       600       610       620        630       640       650
P21179_ESCHERICHIA_COLI           GDVKGRVVAILLNDEVRSADLLAILKA.LKAKGVHAKLLYSRMGEVTADDGTVLPIAATF
R4GRT8_MYCOTHERMUS_THERMOPHILUS   .....RVGILATTSESSALDQAAQLRTRLEKDGLVVTVVAETLRE.........GVDQTY

                                        660       670       680               690       700
P21179_ESCHERICHIA_COLI           AGAPSLTVDAVIVPCGNIADIADNGDANYY........LMEAYKHLKPIALAGDARKFKA
R4GRT8_MYCOTHERMUS_THERMOPHILUS   STADATGFDGVVVVDGAAALFASTASSPLFPTGRPLQIFVDAYRWGKPVGVCGG..KSSE
```

```
                                 710       720       730       740       750
P21179_ESCHERICHIA_COLI          TIKIADQGEEG..IVEADSADGSFMDELLTLMAAHRVWSRIPKIDKIPA
R4GRT8_MYCOTHERMUS_THERMOPHILUS  VLDAADVPEDGDGVYSEESVD.MFVEEFEKGLATFRFTDRFA.LDS...
```

```
                                  1        10        20        30        40        50        60
P21179_ESCHERICHIA_COLI           MSQHNEKNPHQHQSPLHDSSEAKPGMDSLAPEDGSHRPAAEPTPPGAQPTAPGSLKAPDT
R4GRT9_MYCOTHERMUS_THERMOPHILUS   MTC.....PFADPAALYSRQDTTSGQSPLAAYE...........................

                                           70        80        90       100       110       120
P21179_ESCHERICHIA_COLI           RNEKLNSLEDVRKGSENYALTTNQGVRIADDQNSLRAGSRGPTLLEDFILREKITHFDHE
R4GRT9_MYCOTHERMUS_THERMOPHILUS   .......VDD....STGY.LTSDVGGPI.QDQTSLKAGIRGPTLLEDFMFRQKIQHFDHE

                                          130       140       150       160       170       180
P21179_ESCHERICHIA_COLI           RIPERIVHARGSAAHGYFQPYKSLSDITKADFLSDPNKITPVFVRFSTVQGGAGSADTVR
R4GRT9_MYCOTHERMUS_THERMOPHILUS   RVPERAVHARGAGAHGTFTSYADWSNITAASFLNATGKQTPVFVRFSTTAGSRGSADTAR

                                          190       200       210       220       230       240
P21179_ESCHERICHIA_COLI           DIRGFATKFYTEEGIFDLVGNNTPIFFIQDAHKFPDFVHAVKPEPHWAIPQGQSAHDTFW
R4GRT9_MYCOTHERMUS_THERMOPHILUS   DVHGFATRFYTDEGNFDIVGNNIPVFFIQDAIQFPDLIHSVKPRPDNEIPQAATAHDSAW

                                          250       260       270       280       290       300
P21179_ESCHERICHIA_COLI           DYVSLQPETLHNVMWAMSDRGIPRSYRTMEGFGIHTFRLINAEGKATFVRFHWKPLAGKA
R4GRT9_MYCOTHERMUS_THERMOPHILUS   DFFSQQPSTMHTLFWAMSGHGIPRSYRHMDGFGVHTFRFVKDDGSSKLIKWHFKSRQGKA

                                          310       320       330       340       350       360
P21179_ESCHERICHIA_COLI           SLVWDEAQKLTGRDPDFHRRELWEAIEAGDFPEYELGFQLIPEEDEFKFDFDLLDPTKLI
R4GRT9_MYCOTHERMUS_THERMOPHILUS   SLVWEEAQVLSGKNADFHRQDLWDAIESGNGPEWDVCVQIVDESQAQAFGFDLLDPTKII

                                          370       380       390       400       410       420
P21179_ESCHERICHIA_COLI           PEELVPVQRVGKMVLNRNPDNFFAENEQAAFHPGHIVPGLDFTNDPLLQGRLFSYTDTQI
R4GRT9_MYCOTHERMUS_THERMOPHILUS   PEEYAPLTKLGLLKLDRNPTNYFAETEQVMFQPGHIVRGIDFTEDPLLQGRLFSYLDTQL

                                          430       440       450       460       470       480
P21179_ESCHERICHIA_COLI           SRLGGPNFHEIPINRPTCPYHNFQRDGMHRMGIDTNPANYEPNSINDNWPRETPPGPKRG
R4GRT9_MYCOTHERMUS_THERMOPHILUS   NRNGGPNFEQLPINMPRVPIHNNNRDGAGQMFIHRNKYPYTPNTLNSGYPRQANQNAGRG

                                          490       500       510       520       530       540
P21179_ESCHERICHIA_COLI           GFESYQERVEGNKVRERSPSFGEYYSHPRLFWLSQTPFEQRHIVDGFSFELSKVVRPYIR
R4GRT9_MYCOTHERMUS_THERMOPHILUS   FFTAPGRTASGALVREVSPTFNDHWSQPRLFFNSLTPVEQQFLVNAMRFEISLVKSEEVK

                                          550         560       570          580       590
P21179_ESCHERICHIA_COLI           ERVVDQLAHI..DLTLAQAVAKNLGIELTDDQL...NITPPPDVNGLKKDPSLSLYAIPD
R4GRT9_MYCOTHERMUS_THERMOPHILUS   KNVLTQLNRVSHDVAVRVAAAIGLGAPDADDTYYHNNKTAGVSIVGSGPLPTIKTL....

                                       600       610       620        630       640       650
P21179_ESCHERICHIA_COLI           GDVKGRVVAILLNDEVRSADLLAILKA.LKAKGVHAKLLYSRMGEVTADDGTVLPIAATF
R4GRT9_MYCOTHERMUS_THERMOPHILUS   .....RVGILATTSESSALDQAAQLRTRLEKDGLVVTVVAETLRE.........GVDQTY

                                        660       670       680                690       700
P21179_ESCHERICHIA_COLI           AGAPSLTVDAVIVPCGNIADIADNGDANYY........LMEAYKHLKPIALAGDARKFKA
R4GRT9_MYCOTHERMUS_THERMOPHILUS   STADATGFDGVVVVDGAAALFASTASSPLFPTGRPLQIFVDAYRWGKPVGVCGG..KSSE
```

```
                                     710       720       730       740       750
P21179_ESCHERICHIA_COLI              TIKIADQGEEG..IVEADSADGSFMDELLTLMAAHRVWSRIPKIDKIPA
R4GRT9_MYCOTHERMUS_THERMOPHILUS      VLDAADVPEDGDGVYSEESVD.MFVEEFEKGLATFRFTDRFA.LDS...
```

```
                        1        10        20        30        40        50        60
P21179_ESCHERICHIA_COLI MSQHNEKNPHQHQSPLHDSSEAKPGMDSLAPEDGSHRPAAEPTPPGAQPTAPGSLKAPDT
W1F4G9_ESCHERICHIA_COLI MSQHNEKNPHQHQSPLHDSSEAKPGMDSLAPEDGSHRPAAEPTPPGAQPTAPGSLKAPDT

                                 70        80        90       100       110       120
P21179_ESCHERICHIA_COLI RNEKLNSLEDVRKGSENYALTTNQGVRIADDQNSLRAGSRGPTLLEDFILREKITHFDHE
W1F4G9_ESCHERICHIA_COLI RNEKLNSLEDVRKGSENYALTTNQGVRIADDQNSLRAGNRGPTLLEDFILREKITHFDHE

                               130       140       150       160       170       180
P21179_ESCHERICHIA_COLI RIPERIVHARGSAAHGYFQPYKSLSDITKADFLSDPNKITPVFVRFSTVQGGAGSADTVR
W1F4G9_ESCHERICHIA_COLI RIPERIVHARGSAAHGYFQPYKSLSDITKADFLSDPNKITPVFVRFSTVQGGAGSADTVR

                               190       200       210       220       230       240
P21179_ESCHERICHIA_COLI DIRGFATKFYTEEGIFDLVGNNTPIFFIQDAHKFPDFVHAVKPEPHWAIPQGQSAHDTFW
W1F4G9_ESCHERICHIA_COLI DIRGFATKFYTEEGIFDLVGNNTPIFFIQDAHKFPDFVHAVKPEPHWAIPQGQSAHDTFW

                               250       260       270       280       290       300
P21179_ESCHERICHIA_COLI DYVSLQPETLHNVMWAMSDRGIPRSYRTMEGFGIHTFRLINAEGKATFVRFHWKPLAGKA
W1F4G9_ESCHERICHIA_COLI DYVSLQPETLHNVMWAMSDRGIPRSYRTMEGFGIHTFRLINAEGKATFVRFHWKPLAGKA

                               310       320       330       340       350       360
P21179_ESCHERICHIA_COLI SLVWDEAQKLTGRDPDFHRRELWEAIEAGDFPEYELGFQLIPEEDEFKFDFDLLDPTKLI
W1F4G9_ESCHERICHIA_COLI SLVWDEAQKLTGRDPDFHRRELWEAIEAGDFPEYELGFQLIPEEDEFKFDFDLLDPTKLI

                               370       380       390       400       410       420
P21179_ESCHERICHIA_COLI PEELVPVQRVGKMVLNRNPDNFFAENEQAAFHPGHIVPGLDFTNDPLLQGRLFSYTDTQI
W1F4G9_ESCHERICHIA_COLI PEELVPVQRVGKMVLNRNPDNFFAENEQAAFHPGHIVPGLDFTNDPLLQGRLFSYIDTQI

                               430       440       450       460       470       480
P21179_ESCHERICHIA_COLI SRLGGPNFHEIPINRPTCPYHNFQRDGMHRMGIDTNPANYEPNSINDNWPRETPPGPKRG
W1F4G9_ESCHERICHIA_COLI SRLGGPNFHEIPINRPTCPYHNFQRDGMHRMGIDTNPANYEPNSINDNWPRETPPGPKRG

                               490       500       510       520       530       540
P21179_ESCHERICHIA_COLI GFESYQERVEGNKVRERSPSFGEYYSHPRLFWLSQTPFEQRHIVDGFSFELSKVVRPYIR
W1F4G9_ESCHERICHIA_COLI GFESYQERVEGNKVRERSPSFGEYYSHPRLFWLSQTPFEQRHIVDGFSFELSKVVRPYIR

                               550       560       570       580       590       600
P21179_ESCHERICHIA_COLI ERVVDQLAHIDLTLAQAVAKNLGIELTDDQLNITPPPDVNGLKKDPSLSLYAIPDGDVKG
W1F4G9_ESCHERICHIA_COLI ERVVDQLAHIDLTLAQAVAKNLGIELTDDQLNITPPPDVNSLKKDPSLSLYAIPDGDVKG

                               610       620       630       640       650       660
P21179_ESCHERICHIA_COLI RVVAILLNDEVRSADLLAILKALKAKGVHAKLLYSRMGEVTADDGTVLPIAATFAGAPSL
W1F4G9_ESCHERICHIA_COLI RVVAILLNDEVRSADLLAILKALKAKGVHAKLLYSRMGEVTADDGTVLPIAATFAGAPSL

                               670       680       690       700       710       720
P21179_ESCHERICHIA_COLI TVDAVIVPCGNIADIADNGDANYYLMEAYKHLKPIALAGDARKFKATIKIADQGEEGIVE
W1F4G9_ESCHERICHIA_COLI TVDAVIVPCGNIADIADNGDANYYLMEAYKHLKPIALAGDARKFKATIKVADQGEEGIVE
```

```
                                 730          740          750
P21179_ESCHERICHIA_COLI  ADSADGSFMDELLTLMAAHRVWSRIPKIDKIPA
W1F4G9_ESCHERICHIA_COLI  ADSADGSFMDELLTLMAAHRVWSRIPKIDKIPA
```

```
                       1        10        20        30        40        50        60
P21179_ESCHERICHIA_COLI  MSQHNEKNPHQHQSPLHDSSEAKPGMDSLAPEDGSHRPAAEPTPPGAQPTAPGSLKAPDT
BACILLUS_PUMILUS         ............................................................

                                  70        80        90       100       110       120
P21179_ESCHERICHIA_COLI  RNEKLNSLEDVRKGSENYALTTNQGVRIADDQNSLRAGSRGPTLLEDFILREKITHFDHE
BACILLUS_PUMILUS         ...........MTNSNHKNLTTNQGVPVGDNQNSRTAGHRGPSFLDDYHLIEKLAHFDRE

                                 130       140       150       160       170       180
P21179_ESCHERICHIA_COLI  RIPERIVHARGSAAHGYFQPYKSLSDITKADFLSDPNKITPVFVRFSTVQGGAGSADTVR
BACILLUS_PUMILUS         RIPERVVHARGAGAYGVFEVENSMEKHTRAAFLSEEGKQTDVFVRFSTVIHPKGSPETLR

                                 190       200       210       220       230       240
P21179_ESCHERICHIA_COLI  DIRGFATKFYTEEGIFDLVGNNTPIFFIQDAHKFPDFVHAVKPEPHWAIPQGQSAHDTFW
BACILLUS_PUMILUS         DPRGFAVKFYTEEGNYDLVGNNLPIFFIRDALKFPDMVHSLKPDPVTNIQDP....DRYW

                                 250       260       270       280       290       300
P21179_ESCHERICHIA_COLI  DYVSLQPETLHNVMWAMSDRGIPRSYRTMEGFGIHTFRLINAEGKATFVRFHWKPLAGKA
BACILLUS_PUMILUS         DFMTLTPESTHMLTWLFSDEGIPANYAEMRGSGVHTFRWVNKYGETKYVKYHWRPSEGIR

                                 310       320       330       340       350       360
P21179_ESCHERICHIA_COLI  SLVWDEAQKLTGRDPDFHRRELWEAIEAGDFPEYELGFQLIPEEDEFKFDFDLLDPTKLI
BACILLUS_PUMILUS         NLSMEEAAEIQANDFQHATRDLYDRIEKGNYPAWDLYVQLMPLSDYDELDYDPCDPTKTW

                                 370       380       390       400       410       420
P21179_ESCHERICHIA_COLI  PEELVPVQRVGKMVLNRNPDNFFAENEQAAFHPGHIVPGLDFTNDPLLQGRLFSYTDTQI
BACILLUS_PUMILUS         SEEDYPLQKVGRMTLNRNPENFFAETEQAAFTPSALVPGIEASEDKLLQGRLFSYPDTQR

                                 430       440       450       460       470
P21179_ESCHERICHIA_COLI  SRLGGPNFHEIPINRPTCPYHNFQRDGMHRMGIDTNPANYEPNSINDNWPRETP....PG
BACILLUS_PUMILUS         HRLGA.NYMRIPVNCPYAPVHNNQQDGFMTTTRPSGHINYEPNRYDDQ.PKENPHYKESE

                            480       490       500       510       520       530
P21179_ESCHERICHIA_COLI  PKRGGFESYQERVEG.NKVRERSPSFGEYYSHPRLFWLSQTPFEQRHIVDGFSFELSKVV
BACILLUS_PUMILUS         PVLHGDRMVRQKIEKPNDFKQAGEKYRSYSEE...........EKQALIKNLTADL.KGV

                             540       550       560       570       580       590
P21179_ESCHERICHIA_COLI  RPYIRERVVDQLAHIDLTLAQAVAKNLGIELTDDQLNITPPPDVNGLKKDPSLSLYAIPD
BACILLUS_PUMILUS         NEKTKLLAICNFYRADEDYGQRLADSLGVDIRSYLQGSMK....................

                             600       610       620       630       640       650
P21179_ESCHERICHIA_COLI  GDVKGRVVAILLNDEVRSADLLAILKALKAKGVHAKLLYSRMGEVTADDGTVLPIAATFA
BACILLUS_PUMILUS         ............................................................

                             660       670       680       690       700       710
P21179_ESCHERICHIA_COLI  GAPSLTVDAVIVPCGNIADIADNGDANYYLMEAYKHLKPIALAGDARKFKATIKIADQGE
BACILLUS_PUMILUS         ............................................................
```

```
                          720       730       740       750
P21179_ESCHERICHIA_COLI  EGIVEADSADGSFMDELLTLMAAHRVWSRIPKIDKIPA
BACILLUS_PUMILUS         ......................................
```

Supplemental Figure SF5D. Pairwise sequence alignments between the *E. coli* substrate protein target (Fmt-methionyl-tRNA formyltransferase) and homologs sorted by UniProt ID. Labels adjacent to each sequence include PDB ID, UniProt ID, and organism name. Lysine residues previously identified as acetylated in the target protein are highlighted in yellow to examine conservation in 1D.

```
                                           1        10        20        30        40        50
P23882_ESCHERICHIA_COLI                    MSESLRIIF..AGTPDFAARHLDALLSSGHNVVGVFTQPDRPAGRGKKLMPSPVKVLAEE
A0A0H2UKZ6_STREPTOCOCCUS_PNEUMONIAE        SNAMKKIAVFASGNGSNFQVIAEEF.....PVEFVFSDHRDAYVLERAKQLGVLSYAFEL

                                           60        70        80        90       100       110
P23882_ESCHERICHIA_COLI                    KGLPVFQPVSLRPQENQQLVAELQADVMVVVAYGLILPKAVLEMPRLGCINVHGSLLPRW
A0A0H2UKZ6_STREPTOCOCCUS_PNEUMONIAE        KE...FESKADYEAALVELLEEHQIDLVCLAGYMKIVGPTLLSAYEGRIVNIHPAYLPEF

                                         120       130       140       150       160       170
P23882_ESCHERICHIA_COLI                    RGAAPIQRSLWAGDAETGVTIMQMDVGLDTGDMLYKLSCPITAEDTSGTLYDKLAELGPQ
A0A0H2UKZ6_STREPTOCOCCUS_PNEUMONIAE        PGAHGIEDAWNAGVGQSGVTIHWVDSGVDTGQVIKQVRVPRLADDTIDRFEARIHEAEYR

                                         180       190       200       210       220       230
P23882_ESCHERICHIA_COLI                    GLITTLKQLADGTAKPEVQDETLVTYAEKLSKEEARIDWSLSAAQLERCIRAFNPWPMSW
A0A0H2UKZ6_STREPTOCOCCUS_PNEUMONIAE        LYPEVVKALFTD................................................

                                         240       250       260       270       280       290
P23882_ESCHERICHIA_COLI                    LEIEGQPVKVWKASVIDTATNAAPGTILEANKQGIQVATGDGILNLLSLQPAGKKAMSAQ
A0A0H2UKZ6_STREPTOCOCCUS_PNEUMONIAE        ............................................................

                                         300       310
P23882_ESCHERICHIA_COLI                    DLLNSRREWFVPGNRLV
A0A0H2UKZ6_STREPTOCOCCUS_PNEUMONIAE        .................
```

```
                                             1        10        20        30        40        50        60
P23882_ESCHERCHIA_COLI                       MSESLRIIFAGTPDFAARHLDALLSSGHNVVGVFTQPDRPAGRGKKLMPSPVKVLAEEKG
A3DHJ7_HUNGATEICLOSTRIDIUM_THERMOCELLUM      ...........................MNIIIATTKSWNIKNAQKFKKENESKYNT....

                                                      70        80        90       100       110       120
P23882_ESCHERCHIA_COLI                       LPVFQPVSLRPQENQQLVAELQADVMVVVAYGLILPKAVLEMPRLGCINVHGSLLPRWRG
A3DHJ7_HUNGATEICLOSTRIDIUM_THERMOCELLUM      ....TIITNKDELTFEKVKLINPEYILFPHWSWIIPKEIFE..NFTCVVFHMTDLPFGRG

                                                     130       140       150       160       170
P23882_ESCHERCHIA_COLI                       AAPIQRSLWAGDAETGVTIMQMDVGLDTGDMLYKLSCPI..TAEDTSGTLYDKLAELGPQ
A3DHJ7_HUNGATEICLOSTRIDIUM_THERMOCELLUM      GSPLQNLIERGIKKTKISAIKVDGGIDTGDIFFKRDLDLYGTAEEI.......FMRASKI

                                           180       190       200       210       220       230
P23882_ESCHERCHIA_COLI                       GLITTLKQLADGTAKPEVQDETLVTYAEKLSKEEARIDWSLSAAQLERCIRAFNP..WPM
A3DHJ7_HUNGATEICLOSTRIDIUM_THERMOCELLUM      IFNDMIPELLTKRPVPQKQ.EGEATVFQRRKPEQSEISPDFDLEKIYDYIRMLDGEGYPR

                                               240       250       260       270       280       290
P23882_ESCHERCHIA_COLI                       SWLEIEGQPVKVWKASVIDTATNAAPGTILEANKQGIQVATGDGILNLLSLQPAGKKAMS
A3DHJ7_HUNGATEICLOSTRIDIUM_THERMOCELLUM      AFIKYGKYRLEFSRASMKNGKIIADVEIIEGDENE.........................

                                               300       310
P23882_ESCHERCHIA_COLI                       AQDLLNSRREWFVPGNRLV
A3DHJ7_HUNGATEICLOSTRIDIUM_THERMOCELLUM      ...................
```

```
                     1        10        20        30        40        50
P23882_ESCHERICHIA_COLI  MSESLRIIFAGTPDFAARHLDALLSSGHNVVGVFT..QPDRPAGRGKKLMPSPVKVLAEE
E3NZ06_DANIO_RERIO       ....MKIAVIGQSLFGQEVYKELKNEGHMIVGVFTIPDKDGKVDPLAIEAEKDGVPVFKF
```

```
                         60        70        80        90        100       110
P23882_ESCHERICHIA_COLI  KGLPVFQPVSLRPQENQQLVAELQADVMVVVAYGLILPKAVLEMPRLGCINVHGSLLPRW
E3NZ06_DANIO_RERIO       PR...WRLKGKAITEVVDQYKAVGAELNVLPFCSQFIPMEVIDHPKHGSIIYHPSLLPRH
```

```
                        120       130       140       150       160       170
P23882_ESCHERICHIA_COLI  RGAAPIQRSLWAGDAETGVTIMQMDVGLDTGDMLYKLSCPITAEDTSGTLYDKLAELGP.
E3NZ06_DANIO_RERIO       RGASAINWTLIHGDKKGGFTVFWADDGLDTGPILLQRECDVEPNDNVNSIYKRFLFPEGV
```

```
                         180       190       200       210       220       230
P23882_ESCHERICHIA_COLI  QGLITTLKQLADGTAKPEVQDETLVTYAEKLSKEEARIDWSLSAAQLERCIRAFNPWPMS
E3NZ06_DANIO_RERIO       KGMVEAVRLIATGKAPRIKQPEEGATYECIQKKENSKIDWNQPAEAIHNWIRGNDRVPGA
```

```
                         240       250           260             270       280
P23882_ESCHERICHIA_COLI  WLEIEGQPVKVWKASVIDTA...TNAAPGTILEAN......KQGIQVATGDG.ILNLLSL
E3NZ06_DANIO_RERIO       WAEIDGKSVSFYGSTLLENDHFSSNGQPLEIPGASRAALVTKNGLVLFGNDGKMLLVKNL
```

```
                         290       300           310
P23882_ESCHERICHIA_COLI  QPAGKKAMSAQDLLN....SRREWFVPGNRLV............................
E3NZ06_DANIO_RERIO       QFEDGKMIPGSQYFKAGVTSTVELSEDENRFAEQMRVVWKSILTNVEKIEDTTDFFKSGA
```

```
P23882_ESCHERICHIA_COLI  ............................................................
E3NZ06_DANIO_RERIO       ASMDVVRLVEEVKLRAAQLQLQNEDVYMATSFQEFIQMCVRKLRGEDEEEEFNVDYVEKK
```

```
P23882_ESCHERICHIA_COLI  ............................................................
E3NZ06_DANIO_RERIO       LNNMTIHIPHQLFINGEFVDAEGGKTYKSINPTDGQAICDVSLAQISDVEKAVAAAKEAF
```

```
P23882_ESCHERICHIA_COLI  ............................................................
E3NZ06_DANIO_RERIO       EEGEWGKMNPRDRGKLLYKLADLMEQHQEELATIESIDSGAVYTLALKTHIGMSIQTFRY
```

```
P23882_ESCHERICHIA_COLI  ............................................................
E3NZ06_DANIO_RERIO       FAGWCDKIQGCTIPINQARPNRNLTFTKKEPIGVCGIVIPWNYPLMMLAWKTAACLAAGN
```

```
P23882_ESCHERICHIA_COLI  ............................................................
E3NZ06_DANIO_RERIO       TVVLKPAQVTPLTALKFAELTALAKFPKGVVNILPGSGSLVGQRLSDHPDVRKLGFTGST
```

```
P23882_ESCHERICHIA_COLI  ............................................................
E3NZ06_DANIO_RERIO       EIGKHIMKSCAVSNVKKVSLELGGKSPLIIFNDCDLDKAVRMGMSSVFFNKGENCIAAGR
```

```
P23882_ESCHERICHIA_COLI  ............................................................
E3NZ06_DANIO_RERIO       LFIEESIHDIFLERVVSEIRKMKIGDPLDRSTDHGPQNHKAHLDKLVEYCEKGVKEGAKL


P23882_ESCHERICHIA_COLI  ............................................................
E3NZ06_DANIO_RERIO       VCGGKQVERPGFFFEPTVFTDVQDHMYIAVEESFGPVMIISKFSNGEVDKVLQRANATEF


P23882_ESCHERICHIA_COLI  ............................................................
E3NZ06_DANIO_RERIO       GLASGVFTRDISKALYVSEKLQAGTVFINTYNKTDVAAPFGGFKQSGFGKDLGQEALNEY


P23882_ESCHERICHIA_COLI  ..........
E3NZ06_DANIO_RERIO       LKTKCVTVEY
```

```
                         1        10        20        30        40        50
P23882_ESCHERICHIA_COLI  MSESLRIIFAGTPDFAARHLDALLSSGHNVVGVFT..QPDRPAGRGKKLMPSPVKVLAEE
O75891_HOMO_SAPIENS      ....MKIAVIGQSLFGQEVYCHLRKEGHEVVGVFTVPDKDGKADPLGLEAEKDGVPVFKY

                         60        70        80        90       100       110
P23882_ESCHERICHIA_COLI  KGLPVFQPVSLRPQENQQLVAELQADVMVVVAYGLILPKAVLEMPRLGCINVHGSLLPRW
O75891_HOMO_SAPIENS      SR...WRAKGQALPDVVAKYQALGAELNVLPFCSQFIPMEIISAPRHGSIIYHPSLLPRH

                        120       130       140       150       160       170
P23882_ESCHERICHIA_COLI  RGAAPIQRSLWAGDAETGVTIMQMDVGLDTGDMLYKLSCPITAEDTSGTLYDKLAELGP.
O75891_HOMO_SAPIENS      RGASAINWTLIHGDKKGGFSIFWADDGLDTGDLLLQKECEVLPDDTVSTLYNRFLFPEGI

                         180       190       200       210       220       230
P23882_ESCHERICHIA_COLI  QGLITTLKQLADGTAKPEVQDETLVTYAEKLSKEEARIDWSLSAAQLERCIRAFNPWPMS
O75891_HOMO_SAPIENS      KGMVQAVRLIAEGKAPRLPQPEEGATYEGIQKKETAKINWDQPAEAIHNWIRGNDKVPGA

                         240       250                 260       270       280
P23882_ESCHERICHIA_COLI  WLEIEGQPVKVWKASVIDTA...........TNAAPGTILEANKQGIQVATG.DGILNLL
O75891_HOMO_SAPIENS      WTEACEQKLTFFNSTLNTSGLVPEGDALPIPGAHRPGVVTKA...GLILFGNDDKMLLVK

                           290       300       310
P23882_ESCHERICHIA_COLI  SLQPAGKKAMSAQDLLNSRREWFVPGNRLV..............................
O75891_HOMO_SAPIENS      NIQLEDGKMILASNFFKGAASSVLELTEAELVTAEAVRSVWQRILPKVLEVEDSTDFFKS

P23882_ESCHERICHIA_COLI  ............................................................
O75891_HOMO_SAPIENS      GAASVDVVRLVEEVKELCDGLELENEDVYMASTFGDFIQLLVRKLRGDDEEGECSIDYVE

P23882_ESCHERICHIA_COLI  ............................................................
O75891_HOMO_SAPIENS      MAVNKRTVRMPHQLFIGGEFVDAEGAKTSETINPTDGSVICQVSLAQVTDVDKAVAAAKD

P23882_ESCHERICHIA_COLI  ............................................................
O75891_HOMO_SAPIENS      AFENGRWGKISARDRGRLMYRLADLMEQHQEELATIEALDAGAVYTLALKTHVGMSIQTF

P23882_ESCHERICHIA_COLI  ............................................................
O75891_HOMO_SAPIENS      RYFAGWCDKIQGSTIPINQARPNRNLTLTRKEPVGVCGIIIPWNYPLMMLSWKTAACLAA

P23882_ESCHERICHIA_COLI  ............................................................
O75891_HOMO_SAPIENS      GNTVVIKPAQVTPLTALKFAELTLKAGIPKGVVNVLPGSGSLVGQRLSDHPDVRKIGFTG

P23882_ESCHERICHIA_COLI  ............................................................
O75891_HOMO_SAPIENS      STEVGKHIMKSCAISNVKKVSLELGGKSPLIIFADCDLNKAVQMGMSSVFFNKGENCIAA
```

```
P23882_ESCHERICHIA_COLI   ............................................................
O75891_HOMO_SAPIENS       GRLFVEDSIHDEFVRRVVEEVRKMKVGNPLDRDTDHGPQNHHAHLVKLMEYCQHGVKEGA

P23882_ESCHERICHIA_COLI   ............................................................
O75891_HOMO_SAPIENS       TLVCGGNQVPRPGFFFEPTVFTDVEDHMFIAKEESFGPVMIISRFADGDLDAVLSRANAT

P23882_ESCHERICHIA_COLI   ............................................................
O75891_HOMO_SAPIENS       EFGLASGVFTRDINKALYVSDKLQAGTVFVNTYNKTDVAAPFGGFKQSGFGKDLGEAALN

P23882_ESCHERICHIA_COLI   ............
O75891_HOMO_SAPIENS       EYLRVKTVTFEY
```

```
                                 1       10        20        30        40        50        60
P23882_ESCHERICHIA_COLI          MSESLRIIFAGTPDFAARHLDALLSSGHNVVGVFTQPDRPAGRGKKLMPSPVKVLAEEKG
O85732_PSEUDOMONAS_AERUGINOSA    MSQALRIVFAGTPEFAAEHLKALLDTPHRIVAVYTQPDRPAGRGQKLMPSAVKSLALEHG

                                        70        80        90       100       110       120
P23882_ESCHERICHIA_COLI          LPVFQPVSLRPQENQQLVAELQADVMVVVAYGLILPKAVLEMPRLGCINVHGSLLPRWRG
O85732_PSEUDOMONAS_AERUGINOSA    LPVMQPQSLRNAEAQAELAALRADLMVVVAYGLILPQAVLDIPRLGCINSHASLLPRWRG

                                       130       140       150       160       170       180
P23882_ESCHERICHIA_COLI          AAPIQRSLWAGDAETGVTIMQMDVGLDTGDMLYKLSCPITAEDTSGTLYDKLAELGPQGL
O85732_PSEUDOMONAS_AERUGINOSA    AAPIQRAVEAGDAESGVTVMQMEAGLDTGPMLLKVSTPISAADTGGSLHDRLAALGPKAV

                                       190       200       210       220       230       240
P23882_ESCHERICHIA_COLI          ITTLKQLADGTAKPEVQDETLVTYAEKLSKEEARIDWSLSAAQLERCIRAFNPWPMSWLE
O85732_PSEUDOMONAS_AERUGINOSA    IEAIAGLAAGTLHGEIQDDALATYAHKLNKDEARLDWSRPAVELERQVRAFTPWPVCHTS

                                       250       260       270       280       290       300
P23882_ESCHERICHIA_COLI          IEGQPVKVWKASVIDTATNAAPGTILEANKQGIQVATGDGILNLLSLQPAGKKAMSAQDL
O85732_PSEUDOMONAS_AERUGINOSA    LADAPLKVLGASL..GQGSGAPGTILEASRDGLLVACGEGALRLTRLQLPGGKPLAFADL

                                       310
P23882_ESCHERICHIA_COLI          LNSRREWFVPGNRLV.
O85732_PSEUDOMONAS_AERUGINOSA    YNSRREQFAAGQVLGQ
```

```
                             1        10        20        30         40        50
P23882_ESCHERICHIA_COLI      MSESLRIIFAGTPDFAARHLDALLSSGHNVVGVFT..QPDRPAGRGKKLMPSPVKVLAEE
P28037_RATTUS_NORVEGICUS     ....MKIAVIGQSLFGQEVYCQLRKEGHEVVGVFTIPDKDGKADPLGLEAEKDGVPVFKF

                             60        70        80        90        100       110
P23882_ESCHERICHIA_COLI      KGLPVFQPVSLRPQENQQLVAELQADVMVVVAYGLILPKAVLEMPRLGCINVHGSLLPRW
P28037_RATTUS_NORVEGICUS     PR...WRARGQALPEVVAKYQALGAELNVLPFCSQFIPMEVINAPRHGSIIYHPSLLPRH

                            120       130       140       150       160       170
P23882_ESCHERICHIA_COLI      RGAAPIQRSLWAGDAETGVTIMQMDVGLDTGDMLYKLSCPITAEDTSGTLYDKLAELGP.
P28037_RATTUS_NORVEGICUS     RGASAINWTLIHGDKKGGFTIFWADDGLDTGDLLLQKECEVLPDDTVSTLYNRFLFPEGI

                             180       190       200       210       220       230
P23882_ESCHERICHIA_COLI      QGLITTLKQLADGTAKPEVQDETLVTYAEKLSKEEARIDWSLSAAQLERCIRAFNPWPMS
P28037_RATTUS_NORVEGICUS     KGMVQAVRLIAEGTAPRCPQSEEGATYEGIQKKETAKINWDQPAEAIHNWIRGNDKVPGA

                             240       250                 260       270        280
P23882_ESCHERICHIA_COLI      WLEIEGQPVKVWKASVIDTA...........TNAAPGTILEANKQGIQVATG.DGILNLL
P28037_RATTUS_NORVEGICUS     WTEACGQKLTFFNSTLNTSGLSTQGEALPIPGAHRPGVVTKA...GLILFGNDDRMLLVK

                               290       300       310
P23882_ESCHERICHIA_COLI      SLQPAGKKAMSAQDLLNSRREWFVPGNRLV..............................
P28037_RATTUS_NORVEGICUS     NIQLEDGKMMPASQFFKGSASSDLELTEAELATAEAVRSSWMRILPNVPEVEDSTDFFKS

P23882_ESCHERICHIA_COLI      ............................................................
P28037_RATTUS_NORVEGICUS     GAASVDVVRLVEEVKELCDGLELENEDVYMATTFRGFIQLLVRKLRGEDDESECVINYVE

P23882_ESCHERICHIA_COLI      ............................................................
P28037_RATTUS_NORVEGICUS     KAVNKLTLQMPYQLFIGGEFVDAEGSKTYNTINPTDGSVICQVSLAQVSDVDKAVAAAKE

P23882_ESCHERICHIA_COLI      ............................................................
P28037_RATTUS_NORVEGICUS     AFENGLWGKINARDRGRLLYRLADVMEQHQEELATIEALDAGAVYTLALKTHVGMSIQTF

P23882_ESCHERICHIA_COLI      ............................................................
P28037_RATTUS_NORVEGICUS     RYFAGWCDKIQGATIPINQARPNRNLTLTKKEPVGVCGIVIPWNYPLMMLSWKTAACLAA

P23882_ESCHERICHIA_COLI      ............................................................
P28037_RATTUS_NORVEGICUS     GNTVVIKPAQVTPLTALKFAELTLKAGIPKGVVNILPGSGSLVGQRLSDHPDVRKIGFTG

P23882_ESCHERICHIA_COLI      ............................................................
P28037_RATTUS_NORVEGICUS     STEVGKHIMKSCALSNVKKVSLELGGKSPLIIFADCDLNKAVQMGMSSVFFNKGENCIAA
```

```
P23882_ESCHERICHIA_COLI    ............................................................
P28037_RATTUS_NORVEGICUS   GRLFVEESIHNQFVQKVVEEVEKMKIGNPLERDTNHGPQNHEAHLRKLVEYCQRGVKEGA

P23882_ESCHERICHIA_COLI    ............................................................
P28037_RATTUS_NORVEGICUS   TLVCGGNQVPRPGFFFQPTVFTDVEDHMYIAKEESFGPIMIISRFADGDVDAVLSRANAT

P23882_ESCHERICHIA_COLI    ............................................................
P28037_RATTUS_NORVEGICUS   EFGLASGVFTRDINKALYVSDKLQAGTVFINTYNKTDVAAPFGGFKQSGFGKDLGEAALN

P23882_ESCHERICHIA_COLI    ............
P28037_RATTUS_NORVEGICUS   EYLRIKTVTFEY
```

```
                                    1        10        20        30        40        50        60
P23882_ESCHERICHIA_COLI             MSESLRIIFAGTPDFAARHLDALLSSGHNVVGVFTQPDRPAGRGKKLMPSPVKVLAEEKG
P9WKZ3_MYCOBACTERIUM_TUBERCULOSIS   ..............................MTILILTDNVHAHALA.....VDLQARHGD

                                             70        80        90       100       110
P23882_ESCHERICHIA_COLI             LPVFQ.PVSLRPQENQQLVAELQADVMVVVAYGLIL........PKAVLEMPRLGCINVH
P9WKZ3_MYCOBACTERIUM_TUBERCULOSIS   MDVYQSPIGQLPGVPRCDVAERVAE..IVERYDLVLSFHCKQRFPAALIDGVR..CVNVH

                                          120       130       140       150       160       170
P23882_ESCHERICHIA_COLI             GSLLPRWRGAAPIQRSLWAGDAETGVTIMQMDVGLDTGDMLYKLSCPITAEDTSGTLYDK
P9WKZ3_MYCOBACTERIUM_TUBERCULOSIS   PGFNPYNRGWFPQVFSIIDGQ.KVGVTIHEIDDQLDHGPIIAQRECAIESWDSSGSVYAR

                                          180       190        200       210       220
P23882_ESCHERICHIA_COLI             LAELGPQGLITTLKQLADG..TAKPEVQDETLVTYAEKLSKEEARIDWSLSAAQLERCIR
P9WKZ3_MYCOBACTERIUM_TUBERCULOSIS   LMDIERELVLEHFDAIRDGSYTAKSPATEGNLNLKKDFEQLRRLDLNERGTFGHFLNRLR

                                  230        240       250       260       270       280
P23882_ESCHERICHIA_COLI             AF..NPWPMSWLEIEGQPVKVWKASVIDTATNAAPGTILEANKQGIQVATGDGILNLLSL
P9WKZ3_MYCOBACTERIUM_TUBERCULOSIS   ALTHDDFRNAWF.VDASGRKVFVRVVLEPEKPAEA.........................

                                    290       300       310
P23882_ESCHERICHIA_COLI             QPAGKKAMSAQDLLNSRREWFVPGNRLV
P9WKZ3_MYCOBACTERIUM_TUBERCULOSIS   ............................
```

```
                                1        10        20        30        40        50        60
P23882_ESCHERICHIA_COLI         MSESLRIIFAGTPDFAARHLDALLSSGHNVVGVFTQPDRPAGRGKKLMPSPVKVLAEEKG
Q70LM7_BREVIBACILLUS_PARABREVIS ...............................................MRILFLTTFMSKG

                                         70        80        90       100       110
P23882_ESCHERICHIA_COLI         LPVFQPVSLRPQE........NQQLVAELQADVMVVVAYGLILPKAVLEMPRLGCINVHG
Q70LM7_BREVIBACILLUS_PARABREVIS NKVVRYLESLHHEVVICQEKVHAQSANLQEIDWIVSYAYGYILDKEIVSRFRGRIINLHP

                                      120       130       140       150       160       170
P23882_ESCHERICHIA_COLI         SLLPRWRGAAPIQRSLWAGDAETGVTIMQMDVGLDTGDMLYKLSCPITAEDTSGTLYDKL
Q70LM7_BREVIBACILLUS_PARABREVIS SLLPWNKGRDPVFWSVW.DETPKGVTIHLIDEHVDTGDILVQEEIAFADEDTLLDCYNKA

                                      180       190        200       210       220
P23882_ESCHERICHIA_COLI         AELGPQGLITTLKQLADGTAKPEVQDE..TLVTYAEKLSKEEARIDWSLSAAQLERCI..
Q70LM7_BREVIBACILLUS_PARABREVIS NQAIEELFIREWENIVHGRIAPYRQTAGGTLHFKADRDFYKNLNMTTVRELLALKRLCAE

                                  230       240       250                 260       270
P23882_ESCHERICHIA_COLI         ..RAFNPWPMSWLEIEGQPVKVWK..............ASVIDTATNAAPGTILEANKQG
Q70LM7_BREVIBACILLUS_PARABREVIS PKRGEKPIDKTFHQLFEQQVEMTPDHVAVVDRGQSLTYKQLNERANQLAHHLRGKGVKPD

                                             280       290       300       310
P23882_ESCHERICHIA_COLI         IQVA.....TGDGILNLLSLQPAGKKAMSAQDLLNSRREWFVPGNRLV............
Q70LM7_BREVIBACILLUS_PARABREVIS DQVAIMLDKSLDMIVSILAVMKAGGAYVPIDPDYPGERIAYMLADSSAAILLTNALHEEK

P23882_ESCHERICHIA_COLI         ............................................................
Q70LM7_BREVIBACILLUS_PARABREVIS ANGACDIIDVHDPDSYSENTNNLPHVNRPDDLVYVMYTSGSTGLAKGVMIEHHNLVNFCE

P23882_ESCHERICHIA_COLI         ............................................................
Q70LM7_BREVIBACILLUS_PARABREVIS WYRPYFGVTPADKALVYSSFSFDGSALDIFTHLLAGAALHIVPSERKYDLDALNDYCNQE

P23882_ESCHERICHIA_COLI         ............................................................
Q70LM7_BREVIBACILLUS_PARABREVIS GITISYLPTGAAEQFMQMDNQSFRVVITGGDVLKKIERNGTYKLYNGYGPTECTIMVTMF

P23882_ESCHERICHIA_COLI         ............................................................
Q70LM7_BREVIBACILLUS_PARABREVIS EVDKPYANIPIGKPIDRTRILILDEALALQPIGVAGELFIVGEGLGRGYLNRPELTAEKF

P23882_ESCHERICHIA_COLI         ............................................................
Q70LM7_BREVIBACILLUS_PARABREVIS IVHPQTGERMYRTGDRARFLPDGNIEFLGRLDNLVKIRGYRIEPGEIEPFLMNHPLIELT

P23882_ESCHERICHIA_COLI         ............................................................
Q70LM7_BREVIBACILLUS_PARABREVIS TVLAKEQADGRKYLVGYYVAPEEIPHGELREWLGNDLPDYMIPTYFVHMKAFPLTANGKV
```

```
P23882_ESCHERICHIA_COLI            ............................................................
Q70LM7_BREVIBACILLUS_PARABREVIS    DRRALPDVQADAELLGEDYVAPTDELEQQLAQVWSHVLGIPQMGIDDHFLERGGDSIKVM

P23882_ESCHERICHIA_COLI            ............................................................
Q70LM7_BREVIBACILLUS_PARABREVIS    QLIHQLKNIGLSLRYDQLFTHPTIRQLKRLLTEQKQVSLEPLRELDEQAEYETSAVEKRM

P23882_ESCHERICHIA_COLI            ............................................................
Q70LM7_BREVIBACILLUS_PARABREVIS    YIIQQQDVESIAYNVVYTINFPLTVDTEQIRVALEQLVLRHEGLRSTYHMRGDEIVKRIV

P23882_ESCHERICHIA_COLI            ............................................................
Q70LM7_BREVIBACILLUS_PARABREVIS    PRAELSFVRQTGEEESVQSLLAEQIKPFDLAKAPLLRAGVIETADKKVLWFDSHHILLDG

P23882_ESCHERICHIA_COLI            ............................................................
Q70LM7_BREVIBACILLUS_PARABREVIS    LSKSILARELQALLGQQVLSPVEKTYKSFARWQNEWFASDEYEQQIAYWKTLLQGELPAV

P23882_ESCHERICHIA_COLI            ............................................................
Q70LM7_BREVIBACILLUS_PARABREVIS    QLPTKKRPPQLTFDGAIQMYRVNPEITRKLKATAAKHDLTLYMLMLTIVSIWLSKMNSDS

P23882_ESCHERICHIA_COLI            ............................................................
Q70LM7_BREVIBACILLUS_PARABREVIS    NQVILGTVTDGRQHPDTRELLGMFVNTLPLLLSIDHEESFLHNLQQVKAKLLPALQNQYV

P23882_ESCHERICHIA_COLI            ............................................................
Q70LM7_BREVIBACILLUS_PARABREVIS    PFDKILEAARVKREGNRHPLFDVMFMMQGAPETELESNMHHINAGISKFDLTLEVLEREN

P23882_ESCHERICHIA_COLI            ............................................................
Q70LM7_BREVIBACILLUS_PARABREVIS    GLNIVFEYNTHLFDEGMILRMVAQFEHLLLQAVHGLDQQVKRFELVTEDEKRDLFLRVND

P23882_ESCHERICHIA_COLI            ............................................................
Q70LM7_BREVIBACILLUS_PARABREVIS    TAKAYPNKLIMSMLEDWAAATPDKTALVFREQRVTYRELNERVNQLAHTLREKGVQPDDL

P23882_ESCHERICHIA_COLI            ............................................................
Q70LM7_BREVIBACILLUS_PARABREVIS    VMLMAERSVEMMVAIFAVLKAGGAYLPIDPHSPAERIAYIFADSGAKLVLAQSPFVEKAS

P23882_ESCHERICHIA_COLI            ............................................................
Q70LM7_BREVIBACILLUS_PARABREVIS    MAEVVLDLNSASSYAADTSNPPLVNQPGDLVYVMYTSGSTGKPKGVMIEHGALLNVLHGM
```

```
P23882_ESCHERICHIA_COLI              ............................................................
Q70LM7_BREVIBACILLUS_PARABREVIS      QDEYPLLQDDAFLLKTTYIFDISVAEIFGWVPGRGKLVILEPEAEKNPKAIWQAVVGAGI

P23882_ESCHERICHIA_COLI              ............................................................
Q70LM7_BREVIBACILLUS_PARABREVIS      THINFVPSMLIPFVEYLEGRTEANRLRYILACGEAMPDELVPKVYEVLPEVKLENIYGPT

P23882_ESCHERICHIA_COLI              ............................................................
Q70LM7_BREVIBACILLUS_PARABREVIS      EATIYASRYSLAKGSQESPVPIGKPLPNYRMYIINRHGQLQPIGVPGELCIAGASLARGY

P23882_ESCHERICHIA_COLI              ............................................................
Q70LM7_BREVIBACILLUS_PARABREVIS      LNNPALTEEKFTPHPLEKGERIYRTGDLARYREDGNIEYLGRMDHQVKIRGYRIELDEIR

P23882_ESCHERICHIA_COLI              ............................................................
Q70LM7_BREVIBACILLUS_PARABREVIS      SKLIQEETIQDAVVVARNDQNGQAYLCAYLLSEQEWTVGQLRELLRRELPEYMIPAHFVL

P23882_ESCHERICHIA_COLI              ............................................................
Q70LM7_BREVIBACILLUS_PARABREVIS      LKQFPLTANGKLDRKALPEPDGSVKAEAEYAAPRTELEATLAHIWGEVLGIERIGIRDNF

P23882_ESCHERICHIA_COLI              ............................................................
Q70LM7_BREVIBACILLUS_PARABREVIS      FELGGDSIKGLQIASRLQRINWTMVINHLFLYPTIEQIAPFVTSEQVVIEQGLVEGLVKL

P23882_ESCHERICHIA_COLI              ............................................................
Q70LM7_BREVIBACILLUS_PARABREVIS      TPIQRDFFERITADRHHWNQARMLFCRDGLEREWVVETLNALVLQHDALRMRFRETEQGI

P23882_ESCHERICHIA_COLI              ............................................................
Q70LM7_BREVIBACILLUS_PARABREVIS      VQFHQGNEGKLFGFHVFDCTEELDIAKKVEEQANVLQSGMNLQEGPLVQAALFMTRTGDH

P23882_ESCHERICHIA_COLI              ............................................................
Q70LM7_BREVIBACILLUS_PARABREVIS      LLLAIHQLVVDEASWRIILEDFQTAYKQKAAGEPIALPNKTHSYQSWAEELHNAANSKKL

P23882_ESCHERICHIA_COLI              ............................................................
Q70LM7_BREVIBACILLUS_PARABREVIS      TSELGYWRKIASSPTRPLPQDQEPLSRTEQSTATAAIRFAKAETANLLHEANHAYQTEAQ

P23882_ESCHERICHIA_COLI              ............................................................
Q70LM7_BREVIBACILLUS_PARABREVIS      ELLLAALGMALRDWTRADDVTVFLEKDGRESAAKGLDVSRTVGWFHSLFPVVLSAARSGD
```

```
P23882_ESCHERICHIA_COLI            ............................................................
Q70LM7_BREVIBACILLUS_PARABREVIS    PGEQIKQVKEMLRAIPHQGSGYSILKQLTDLRHKHPDDFTLQPKIVVHAWEQLDAGLETD


P23882_ESCHERICHIA_COLI            ............................................................
Q70LM7_BREVIBACILLUS_PARABREVIS    WLTLSHLPQGSVRGANAERMQQLDVFSKISNGELTIHIQYHRDEYRKATIDKLLELYQAH


P23882_ESCHERICHIA_COLI            .........................................
Q70LM7_BREVIBACILLUS_PARABREVIS    LNALLAHCLQKTETELTPSDFVDKNLSRSELDDIMDLISDL
```

```
                              1        10        20        30        40        50        60
P23882_ESCHERICHIA_COLI       MSESLRIIFAGTPDFAARHLDALLSSGHNVVGVFTQPDRPAGRGKKLMPSPVKVLAEEKG
Q81WH2_BACILLUS_ANTHRACIS     M...IKVVFMGTPDFSVPVLRRLIEDGYDVIGVVTQPDRPVGRKKVLTPTPVKVEAEKHG

                                       70        80        90       100       110       120
P23882_ESCHERICHIA_COLI       LPVFQPVSLRPQENQQLVAELQADVMVVVAYGLILPKAVLEMPRLGCINVHGSLLPRWRG
Q81WH2_BACILLUS_ANTHRACIS     IPVLQPLRIREKDEYEKVLALEPDLIVTAAFGQIVPNEILEAPKYGCINVHASLLPELRG

                                      130       140       150       160       170       180
P23882_ESCHERICHIA_COLI       AAPIQRSLWAGDAETGVTIMQMDVGLDTGDMLYKLSCPITAEDTSGTLYDKLAELGPQGL
Q81WH2_BACILLUS_ANTHRACIS     GAPIHYAIMEGKEKTGITIMYMVEKLDAGDILTQVEVEIEERETTGSLFDKLSEAGAHLL

                                      190       200       210       220       230       240
P23882_ESCHERICHIA_COLI       ITTLKQLADGTAKPEVQDETLVTYAEKLSKEEARIDWSLSAAQLERCIRAFNPWPMSWLE
Q81WH2_BACILLUS_ANTHRACIS     SKTVPLLIQGKLEPIKQNEEEVTFAYNIKREQEKIDWTKTGEEVYNHIRGLNPWPVAYTT

                                      250       260       270       280       290
P23882_ESCHERICHIA_COLI       IEGQPVKVWKASVIDTATNAAPGTILEANKQGIQVATG.DGILNLLSLQPAGKKAMSAQD
Q81WH2_BACILLUS_ANTHRACIS     LAGQVVKVWWGEKVPVTKSAEAGTIVAIEEDGFVVATGNETGVKITELQPSGKKRMSCSQ

                           300       310
P23882_ESCHERICHIA_COLI       LLNSRREWFVPGNRLV...
Q81WH2_BACILLUS_ANTHRACIS     FLRGTKPEI..GTKLGENA
```

```
                           1        10        20        30        40        50        60
P23882_ESCHERICHIA_COLI    MSESLRIIFAGTPDFAARHLDALLSSGHNVVGVFTQPDRPAGRGKKLMPSPVKVLAEEKG
Q83AA8_COXIELLA_BURNETII   MS..LKIVFAGTPQFAVPTLRALIDSSHRVLAVYTQPDRPSGRGQKIMESPVKEIARQNE

                                    70        80        90       100       110       120
P23882_ESCHERICHIA_COLI    LPVFQPVSLRPQENQQLVAELQADVMVVVAYGLILPKAVLEMPRLGCINVHGSLLPRWRG
Q83AA8_COXIELLA_BURNETII   IPIIQPFSLRDEVEQEKLIAMNADVMVVVAYGLILPKKALNAFRLGCVNVHASLLPRWRG

                                   130       140       150       160       170       180
P23882_ESCHERICHIA_COLI    AAPIQRSLWAGDAETGVTIMQMDVGLDTGDMLYKLSCPITAEDTSGTLYDKLAELGPQGL
Q83AA8_COXIELLA_BURNETII   AAPIQRAILAGDRETGISIMQMNEGLDTGDVLAKSACVISSEDTAADLHDRLSLIGADLL

                                   190       200       210       220       230       240
P23882_ESCHERICHIA_COLI    ITTLKQLADGTAKPEVQDETLVTYAEKLSKEEARIDWSLSAAQLERCIRAFNPWPMSWLE
Q83AA8_COXIELLA_BURNETII   LESLAKLEKGDIKLEKQDEASATYASKIQKQEALIDWRKSAVEIARQVRAFNPTPIAFTY

                                   250       260       270       280       290       300
P23882_ESCHERICHIA_COLI    IEGQPVKVWKASVIDTATNAAPGTILEANKQGIQVATGDGILNLLSLQPAGKKAMSAQDL
Q83AA8_COXIELLA_BURNETII   FEGQPMRIWRATVVDEKTDFEPGVLVDADKKGISIAAGSGILRLHQLQLPGKRVCSAGDF

                                   310
P23882_ESCHERICHIA_COLI    LNSRREWFVPGNRLV.
Q83AA8_COXIELLA_BURNETII   INAHGDKLIPGKTVFG
```

```
                            1        10        20        30        40        50
P23882_ESCHERICHIA_COLI     MS.ESLRIIF..AGTPDFAARHLDALLSS.GHNVVGVFTQPDRPAGRGKKLMPSPVKVLA
Q83AY9_COXIELLA_BURNETII    MNREPLPIVVLISGNGTNLQAIIGAIQKGLAIEIRAVISNRADAYGLKRAQQADIPTHII

                               60        70        80        90       100       110
P23882_ESCHERICHIA_COLI     EEKGLPVFQPVSLRPQENQQLVAELQADVMVVVAYGLILPKAVLEMPRLGCINVHGSLLP
Q83AY9_COXIELLA_BURNETII    PHEE...FPSRTDFESTLQKTIDHYDPKLIVLAGFMRKLGKAFVSHYSGRMINIHPSLLP

                              120       130       140       150       160       170
P23882_ESCHERICHIA_COLI     RWRGAAPIQRSLWAGDAETGVTIMQMDVGLDTGDMLYKLSCPITAEDTSGTLYDKLAELG
Q83AY9_COXIELLA_BURNETII    KYTGLNTHERALAAGETEHGVSVHYVTEDLDAGPLICQARLSITPQDTPETLKTRVHALE

                              180       190       200       210       220       230
P23882_ESCHERICHIA_COLI     PQGLITTLKQLADGTAKPEVQDETLVTYAEKLSKEEARIDWSLSAAQLERCIRAFNPWPM
Q83AY9_COXIELLA_BURNETII    HIIYPEVLSWFAAG..RLNYHNNQVFLDGKPLAKSGHAFP....................

                              240       250       260       270       280       290
P23882_ESCHERICHIA_COLI     SWLEIEGQPVKVWKASVIDTATNAAPGTILEANKQGIQVATGDGILNLLSLQPAGKKAMS
Q83AY9_COXIELLA_BURNETII    ............................................................

                              300       310
P23882_ESCHERICHIA_COLI     AQDLLNSRREWFVPGNRLV
Q83AY9_COXIELLA_BURNETII    ...................
```

```
                           1
P23882_ESCHERICHIA_COLI    MSES........................................................
Q88LI9_PSEUDOMONAS_PUTIDA  MNKNNQYVLSLACQDAPGIVSEVSTFLFNNGANIVEAEQFNDEDSSKFFMRVSVEIPVAG

                                                            10        20          30
P23882_ESCHERICHIA_COLI    ...........................LRIIF..AGTPDFAARHLDALLSSGHN..VVGV
Q88LI9_PSEUDOMONAS_PUTIDA  VNDFNSAFGKVVEKYNAEWWFRPRTDRKKVVIMVSKFDHCLGDLLYRHRLGELDMEVVGI

                                40        50        60        70        80        90
P23882_ESCHERICHIA_COLI    FTQPDRPAGRGKKLMPSPVKVLAEEKGLPVFQPVSLRPQENQQLVAELQADVMVVVAYGL
Q88LI9_PSEUDOMONAS_PUTIDA  ISNHPREA.LSVSLVGDIPFHYLPVTP....ATKAAQESQIKNIVTQSQADLIVLARYMQ

                               100       110       120       130       140       150
P23882_ESCHERICHIA_COLI    ILPKAVLEMPRLGCINVHGSLLPRWRGAAPIQRSLWAGDAETGVTIMQMDVGLDTGDMLY
Q88LI9_PSEUDOMONAS_PUTIDA  ILSDDLSAFLSGRCINIHHSFLPGFKGAKPYHQAHTRGVKLIGATAHFVTADLDEGPIIA

                               160       170       180       190       200       210
P23882_ESCHERICHIA_COLI    KLSCPITAEDTSGTLYDKLAELGPQGLITTLKQLADGTAKPEVQDETLVTYAEKLSKEEA
Q88LI9_PSEUDOMONAS_PUTIDA  QDVEHVSHRDSAEDLVRKGRDIERRVLSRAVLLFLED..RLIVNGERTVVFAD.......

                               220       230       240       250       260       270
P23882_ESCHERICHIA_COLI    RIDWSLSAAQLERCIRAFNPWPMSWLEIEGQPVKVWKASVIDTATNAAPGTILEANKQGI
Q88LI9_PSEUDOMONAS_PUTIDA  ............................................................

                               280       290       300       310
P23882_ESCHERICHIA_COLI    QVATGDGILNLLSLQPAGKKAMSAQDLLNSRREWFVPGNRLV
Q88LI9_PSEUDOMONAS_PUTIDA  ..........................................
```

```
                               1        10        20        30        40        50        60
P23882_ESCHERICHIA_COLI        MSESLRIIFAGTPDFAARHLDALLSSGHNVVGVFTQPDRPAGRGKKLMPSPVKVLAEEKG
Q8ZJ80_YERSINIA_PESTIS         MSDSLRIIFAGTPDFAARHLGALLSSQHKIVGVFTQPDRPAGRGNKLTPSPVKILAEHHG

                                        70        80        90       100       110       120
P23882_ESCHERICHIA_COLI        LPVFQPVSLRPQENQQLVAELQADVMVVVAYGLILPKAVLEMPRLGCINVHGSLLPRWRG
Q8ZJ80_YERSINIA_PESTIS         IPVFQPKSLRPEENQHLVADLNADIMVVVAYGLILPAAVLAMPRLGCINVHGSLLPRWRG

                                       130       140       150       160       170       180
P23882_ESCHERICHIA_COLI        AAPIQRSLWAGDAETGVTIMQMDVGLDTGDMLYKLSCPITAEDTSGTLYDKLAELGPQGL
Q8ZJ80_YERSINIA_PESTIS         AAPIQRSVWAGDEKTGITIMQMDIGLDTGAMLHKIECAIQPEDTSATLYDKLAQLGPQGL

                                       190       200       210       220       230       240
P23882_ESCHERICHIA_COLI        ITTLKQLADGTAKPEVQDETLVTYAEKLSKEEARIDWSLSAAQLERCIRAFNPWPMSWLE
Q8ZJ80_YERSINIA_PESTIS         LITLQQLAAGTALAEVQNETQATYAEKLSKEEAKLDWTLSATQLERCIRAFNPWPVSYFI

                                       250       260       270       280       290       300
P23882_ESCHERICHIA_COLI        IEGQPVKVWKASVIDTATNAAPGTILEANKQGIQVATGDGILNLLSLQPAGKKAMSAQDL
Q8ZJ80_YERSINIA_PESTIS         VDEQPIKVWQAQVLPAGEDAEPGTIIHADKHGIQVATADGVLNITQLQPAGKKAMSAADL

                                       310
P23882_ESCHERICHIA_COLI        LNSRREWFVPGNRLV
Q8ZJ80_YERSINIA_PESTIS         LNSRREWFIPGSQLV
```

```
                            1         10        20        30        40        50
P23882_ESCHERICHIA_COLI     MSESLRIIF..AGTPDFAARHLDALLSSGHN..VVGVFTQPDRPAGRGKKLMPSPVKVLA
Q9KF54_BACILLUS_HALODURANS  ...MKRVAIFASGSGTNAEAIIQSQKAGQLPCEVALLITDKPGAKVVERVKVHEIPVCAL

                              60        70        80        90        100       110
P23882_ESCHERICHIA_COLI     EEKGLPVFQPVSLRPQENQQLVAELQADVMVVVAYGLILPKAVLEMPRLGCINVHGSLLP
Q9KF54_BACILLUS_HALODURANS  DPKT...YPSKEAYEIEVVQQLKEKQIDFVVLAGYMRLVGPTLLGAYEGRIVNIHPSLLP

                              120       130       140       150       160       170
P23882_ESCHERICHIA_COLI     RWRGAAPIQRSLWAGDAETGVTIMQMDVGLDTGDMLYKLSCPITAEDTSGTLYDKLAELG
Q9KF54_BACILLUS_HALODURANS  AFPGLHAIEQAIRANVKVTGVTIHYVDEGMDTGPIIAQEAVSIEEEDTLETLTTKIQAVE

                              180       190       200       210       220       230
P23882_ESCHERICHIA_COLI     PQGLITTLKQLADGTAKPEVQDETLVTYAEKLSKEEARIDWSLSAAQLERCIRAFNPWPM
Q9KF54_BACILLUS_HALODURANS  HRLYPATLHKLLSK..............................................

                              240       250       260       270       280       290
P23882_ESCHERICHIA_COLI     SWLEIEGQPVKVWKASVIDTATNAAPGTILEANKQGIQVATGDGILNLLSLQPAGKKAMS
Q9KF54_BACILLUS_HALODURANS  ............................................................

                              300       310
P23882_ESCHERICHIA_COLI     AQDLLNSRREWFVPGNRLV
Q9KF54_BACILLUS_HALODURANS  ...................
```

```
                         1        10        20        30        40        50        60
P23882_ESCHERICHIA_COLI  MSESLRIIFAGTPDFAARHLDALLSSGHNVVGVFTQPDRPAGRGKKLMPSPVKVLAEEKG
Q9KVU4_VIBRIO_CHOLERAE   MSQSLRIVFAGTPDFAARHLAALLSSEHEIIAVYTQPERPAGRGKKLTASPVKTLALEHN

                                  70        80        90       100       110       120
P23882_ESCHERICHIA_COLI  LPVFQPVSLRPQENQQLVAELQADVMVVVAYGLILPKAVLEMPRLGCINVHGSLLPRWRG
Q9KVU4_VIBRIO_CHOLERAE   VPVYQPENFKSDESKQQLAALNADLMVVVAYGLLLPKVVLDTPKLGCINVHGSILPRWRG

                                 130       140       150       160       170       180
P23882_ESCHERICHIA_COLI  AAPIQRSLWAGDAETGVTIMQMDVGLDTGDMLYKLSCPITAEDTSGTLYDKLAELGPQGL
Q9KVU4_VIBRIO_CHOLERAE   AAPIQRSIWAGDSETGVTIMQMDVGLDTGDMLKIATLPIEASDTSASMYDKLAELGPQAL

                                 190       200       210       220       230       240
P23882_ESCHERICHIA_COLI  ITTLKQLADGTAKPEVQDETLVTYAEKLSKEEARIDWSLSAAQLERCIRAFNPWPMSWLE
Q9KVU4_VIBRIO_CHOLERAE   LECLQDIAQGTAVAVKQDDGLANYAHKLSKEEARINWSDAATHIERCIRAFNPWPMSHFE

                                 250       260       270       280       290       300
P23882_ESCHERICHIA_COLI  IEGQPVKVWKASVIDTATNAAPGTILEANKQGIQVATGDGILNLLSLQPAGKKAMSAQDL
Q9KVU4_VIBRIO_CHOLERAE   VAENSIKVWQARVETRAVTQTPGTIIQADKSGIYVATGQDVLVLESLQIPGKKALPVQDI

                                 310
P23882_ESCHERICHIA_COLI  LNSRREWFVPGNRLV
Q9KVU4_VIBRIO_CHOLERAE   LNARADWFSVGSQLS
```
