# Supplementary material for: Investigation of the Importance of Protein 3D Structure for Assessing Conservation of Lysine Acetylation Sites in Protein Homologs
Source: Front Microbiol. 2022 Jan 31;12:805181. doi: 10.3389/fmicb.2021.805181 (PMC8843374; doi:10.3389/fmicb.2021.805181)

Supplemental Figure SF6. Conserved and non-conserved domains of each protein homolog structure compared to the *E. coli* target substrate protein. Each group of target protein (Adk, Icd, KatE, Fmt) homologs is divided into sub-groups of conserved and non-conserved structural domains compared to the target protein. The target proteins are colored based on domains defined in **Figure 1**. All homolog structures are in gray and the corresponding AcP and KAT lysine acetylation sites are colored in red and blue, respectively. Lysine sites acetylated by both AcP and KAT proteins are in purple on the Icd homologs.

Adk

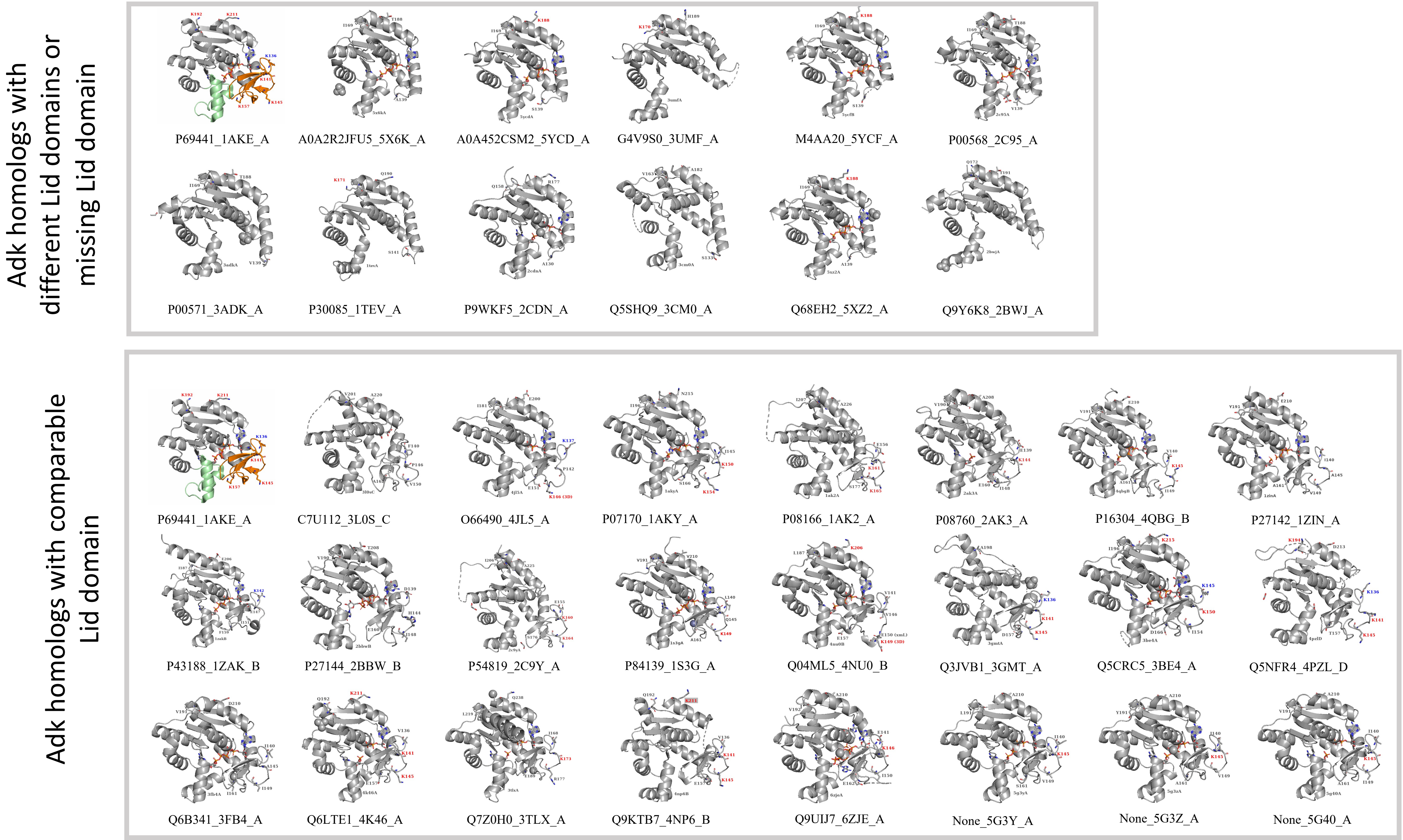
Adk homologs with different Lid domains or missing Lid domain
P69441_1AKE_A
A0A2R2JFU5_5X6K_A
A0A452CSM2_5YCD_A
G4V9S0_3UMF_A
M4AA20_5YCF_A
P00568_2C95_A
P00571_3ADK_A
P30085_1TEV_A
P9WKF5_2CDN_A
Q5SHQ9_3CM0_A
Q68EH2_5XZ2_A
Q9Y6K8_2BWJ_A
Adk homologs with comparable Lid domain
P69441_1AKE_A
C7U112_3L0S_C
O66490_4JL5_A
P07170_1AKY_A
P08166_1AK2_A
P08760_2AK3_A
P16304_4QBG_B
P27142_1ZIN_A
P43188_1ZAK_B
P27144_2BBW_B
P54819_2C9Y_A
P84139_1S3G_A
Q04ML5_4NU0_B
Q3JVB1_3GMT_A
Q5CRC5_3BE4_A
Q5NFR4_4PZL_D
Q6B341_3FB4_A
Q6LTE1_4K46_A
Q7Z0H0_3TLX_A
Q9KTB7_4NP6_B
Q9UIJ7_6ZJE_A
None_5G3Y_A
None_5G3Z_A
None_5G40_A

Icd

Icd homolog with different clasp domain and loop of small domain

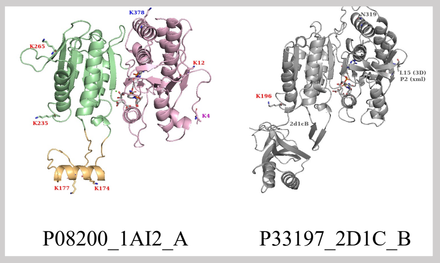

Icd homologs with extra helix on small domain

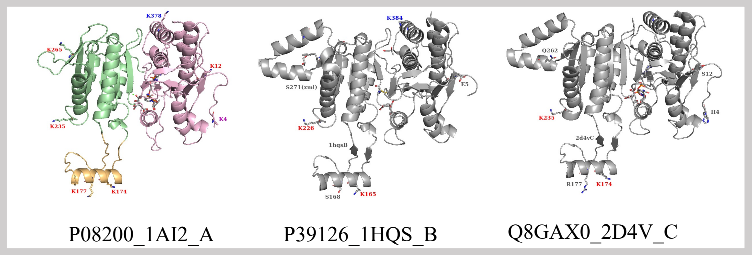

Icd homologs with short helix instead of loop in small domain

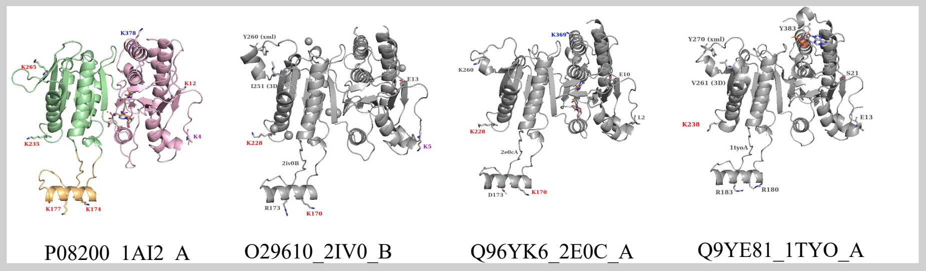

Icd homologs with different type of clasp domain and loop of small domain missing

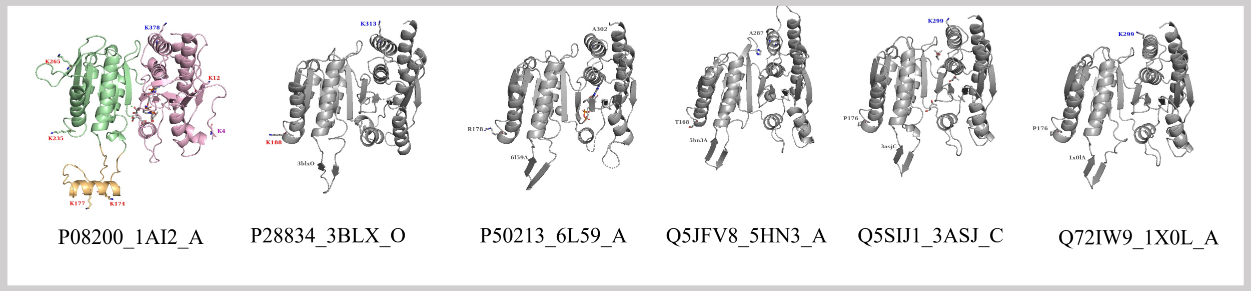

Icd homologs with all domains conserved

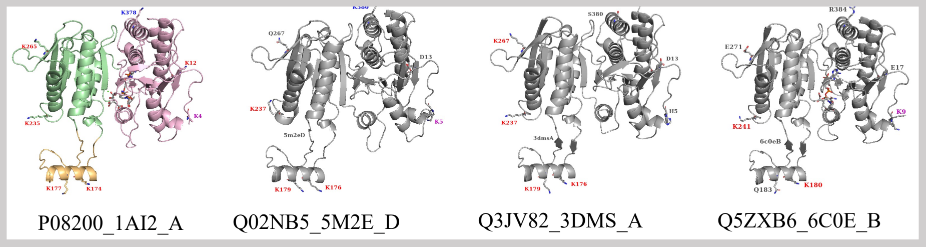

KatE

KatE homologs (Clade I) N-terminal domain

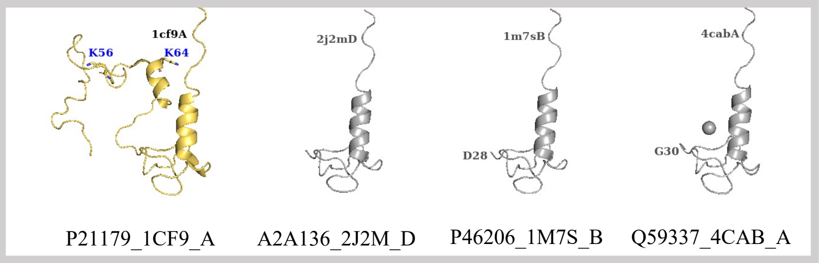

KatE homologs (Clade II)
N-terminal domain

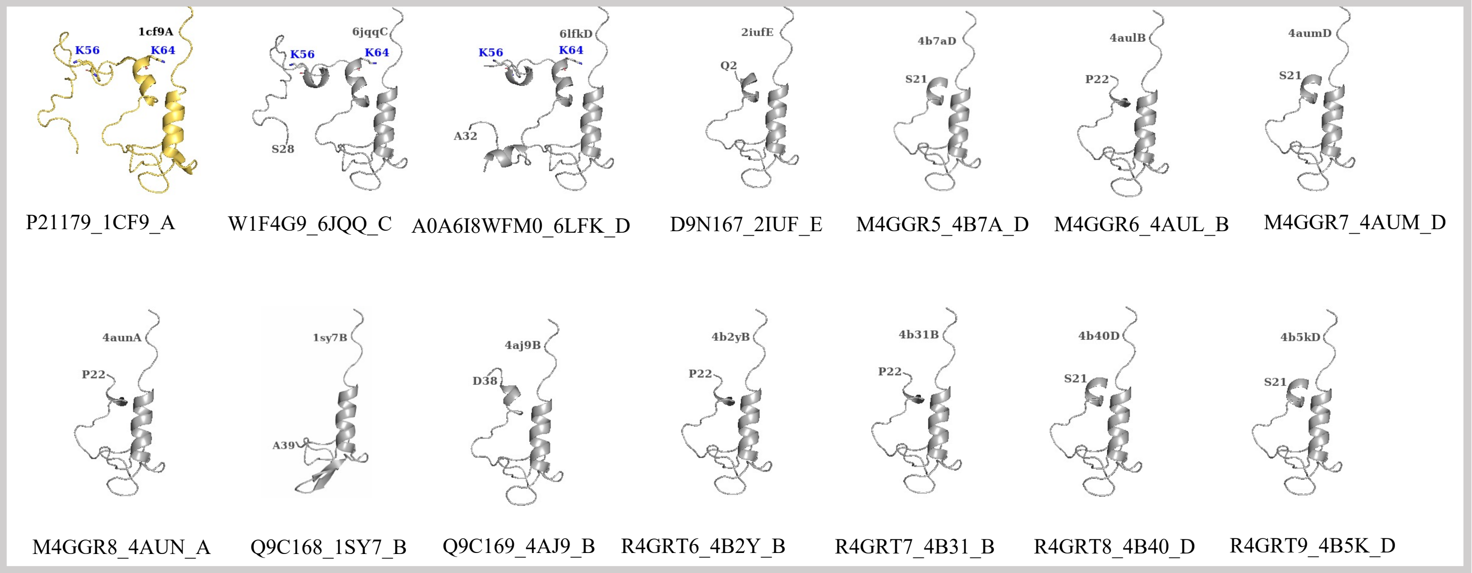

KatE homologs (Clade III)
N-terminal domain

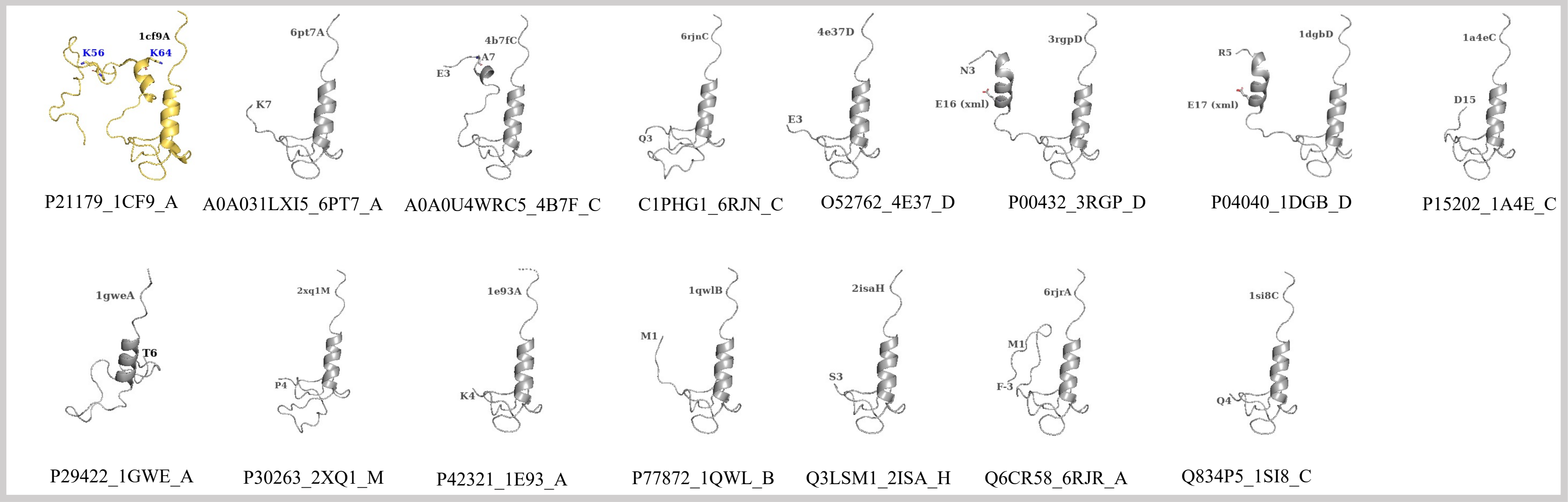

Fmt

Fmt homolog with short loop and missing β-barrel domain

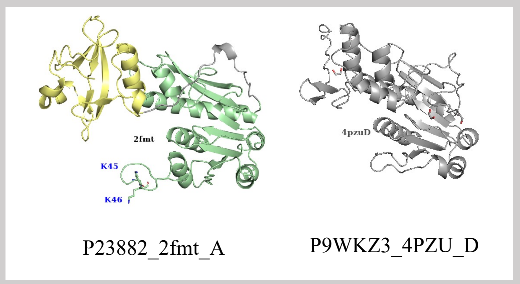

Fmt homologs with loop and β-barrel domain missing

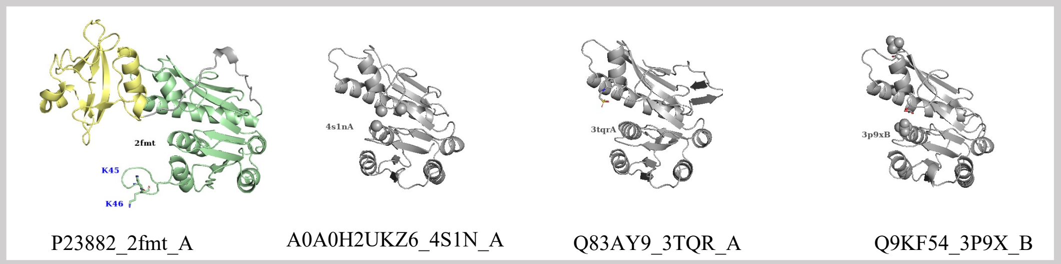

Fmt homologs with loop and β-barrel domain missing, and extra domain

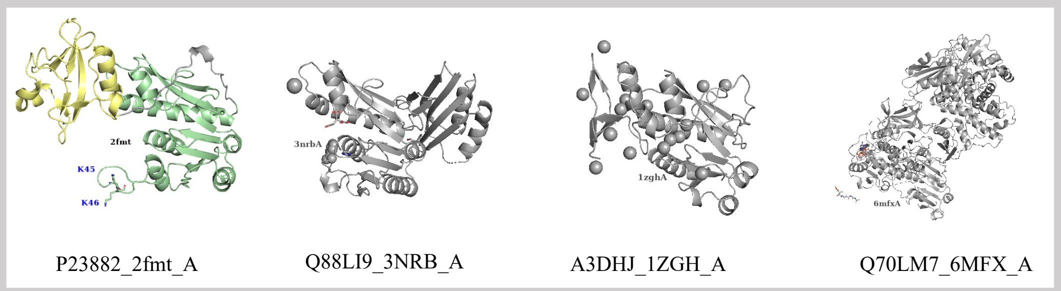

Fmt homologs with a shortened loop

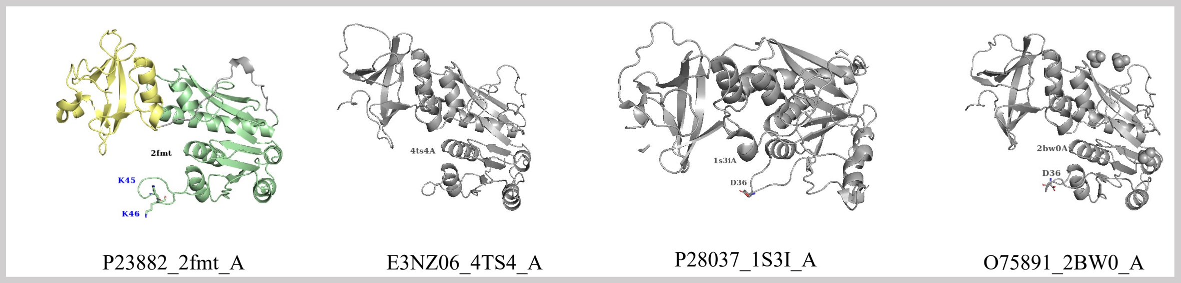

Fmt homologs with conserved domains or a disordered loop

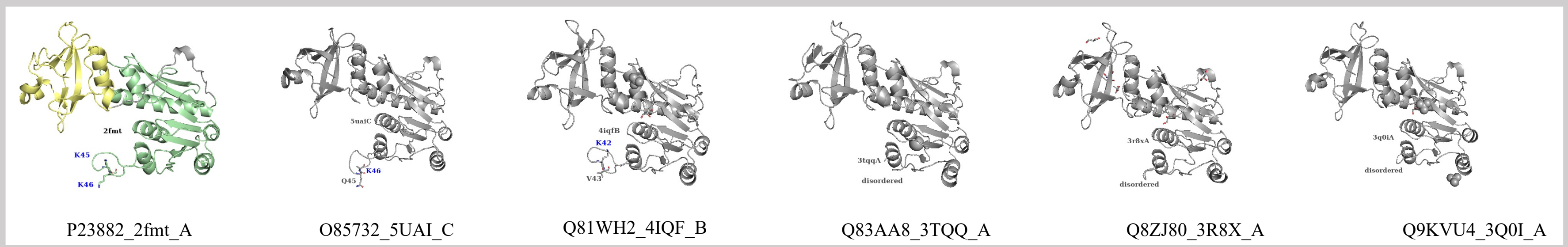

Supplement: Supplementary file 9 [file Data_Sheet_9.PDF]
