# Supplementary material for: Investigation of the Importance of Protein 3D Structure for Assessing Conservation of Lysine Acetylation Sites in Protein Homologs
Source: Front Microbiol. 2022 Jan 31;12:805181. doi: 10.3389/fmicb.2021.805181 (PMC8843374; doi:10.3389/fmicb.2021.805181)
Supplement: Supplementary file 10 [file Data_Sheet_10.PDF]

Supplemental Table ST3. FATCAT alignment parameters from aligning homologs to the Adk, Icd, KatE, and Fmt target structures (PDB ID: 1ake, 1ai2, 1cf9, and 2fmt, respectively). AA means amino acid. Data are sorted by UniProt ID (alphabetical order) and represent all homologs with structures deposited into the PDB.

Uniprot	PDB	Structure Length	P-value	twists	opt-len	opt-rmsd	align-len	gap	seq-identity	score
**Adk**										
A0A0J9X1X4	3x2s.A	214	0.00e+00	0	214	0.61	214	0	98.60%	620.62
A0A2R2JFU5	5x6k.A	185	0.00e+00	0	181	1.72	215	34	31.16%	440.76
A0A2R2JFU5	5ycb.B	186	0.00e+00	0	181	1.69	215	34	30.70%	435.61
A0A2R2JFU5	5ycc.B	186	0.00e+00	0	182	1.72	215	33	31.16%	437.01
A0A2R2JFU6	5x6l.A	186	0.00e+00	0	181	1.72	215	34	31.63%	440.48
A0A2R2JFU6	5xru.A	186	0.00e+00	0	181	1.70	215	34	31.16%	440.54
A0A452CSM2	5ycd.A	191	0.00e+00	0	181	1.72	215	34	31.63%	440.29
A7ZIN4	6hap.A	214	0.00e+00	0	214	1.34	214	0	98.13%	612.53
C7U112	2xb4.A	223	6.59e-11	2	206	2.88	224	18	30.80%	502.09
C7U112	3l0p.A	222	1.78e-11	1	197	3.23	230	33	28.26%	466.88
C7U112	3l0s.C	223	8.93e-11	2	209	2.93	224	15	31.25%	497.51
G4V9S0	3umf.A	188	4.77e-13	0	167	3.37	219	52	26.03%	389.13
M4AA20	5ycf.B	188	0.00e+00	0	177	1.54	215	38	32.09%	420.97
None	4jzk.A	214	0.00e+00	0	214	0.33	214	0	100.00%	622.95
O66490	2rgx.A	203	0.00e+00	0	199	1.62	218	19	42.66%	504.20
O66490	2rh5.B	202	8.98e-10	2	193	2.93	217	24	42.40%	452.71
O66490	3sr0.A	203	0.00e+00	0	199	1.46	218	19	43.12%	516.67
O66490	4cf7.B	203	0.00e+00	0	199	1.57	218	19	43.12%	508.15
O66490	4ike.A	206	0.00e+00	0	199	1.61	218	19	42.66%	507.23
O66490	4jky.A	203	0.00e+00	0	199	1.59	218	19	42.66%	504.76
O66490	4jl5.A	203	0.00e+00	0	199	1.46	218	19	43.12%	516.49
O66490	4jl6.A	203	0.00e+00	0	199	1.50	218	19	43.12%	512.76
O66490	4jl8.B	203	0.00e+00	0	199	1.45	218	19	43.12%	508.74
O66490	4jla.B	203	0.00e+00	0	199	1.45	218	19	43.12%	508.86
O66490	4jlb.B	203	0.00e+00	0	199	1.47	218	19	43.12%	510.80
O66490	4jld.A	203	0.00e+00	0	199	1.49	218	19	43.12%	508.34
O66490	4jlo.A	203	0.00e+00	0	199	1.48	218	19	42.66%	512.29
O66490	4jlp.A	203	0.00e+00	0	199	1.46	218	19	42.66%	511.76
P00568	1z83.C	195	0.00e+00	0	181	1.78	215	34	30.23%	440.40
P00568	2c95.A	195	0.00e+00	0	181	1.78	215	34	30.23%	434.96
P00571	3adk.A	194	1.27e-14	0	179	3.06	215	36	30.70%	405.42
P07170	1aky.A	218	0.00e+00	0	209	1.41	219	10	44.75%	552.61
P07170	1dvr.B	220	9.99e-16	1	202	2.44	219	17	43.84%	544.27
P07170	2aky.A	218	0.00e+00	0	209	1.26	219	10	44.75%	554.83

P07170	3aky.A	218	0.00e+00	0	209	1.35	219	10	44.75%	552.43
P08166	1ak2.A	220	4.25e-14	1	209	2.93	218	9	41.28%	526.59
P08166	2ak2.A	220	3.13e-14	1	209	2.94	218	9	41.28%	531.97
P08760	2ak3.A	226	2.23e-14	2	202	1.54	214	12	39.25%	489.70
P16304	1p3j.A	212	0.00e+00	0	208	1.21	217	9	46.54%	543.11
P16304	2eu8.B	216	0.00e+00	0	209	1.25	218	9	46.79%	551.23
P16304	2oo7.B	216	0.00e+00	0	209	1.22	218	9	46.33%	552.20
P16304	2ori.B	216	0.00e+00	0	209	1.27	218	9	46.33%	550.64
P16304	2osb.B	216	0.00e+00	0	209	1.30	218	9	46.33%	550.74
P16304	2p3s.A	216	0.00e+00	0	209	1.14	218	9	46.79%	550.90
P16304	2qaj.B	216	0.00e+00	0	209	1.29	218	9	46.33%	553.02
P16304	3dkv.A	217	0.00e+00	0	209	1.30	218	9	48.17%	553.04
P16304	3dl0.A	216	0.00e+00	0	209	1.70	218	9	47.25%	552.22
P16304	4mkf.A	217	0.00e+00	0	209	1.30	218	9	46.33%	552.02
P16304	4mkg.A	217	0.00e+00	0	209	1.25	218	9	48.17%	553.68
P16304	4mkh.A	218	0.00e+00	0	209	2.40	218	9	46.79%	549.69
P16304	4qbf.A	216	0.00e+00	0	209	2.30	218	9	48.17%	552.19
P16304	4qbg.B	217	0.00e+00	0	209	2.69	218	9	45.41%	547.72
P16304	4typ.C	190	0.00e+00	0	186	1.41	217	31	41.94%	448.99
P16304	4tyq.A	215	0.00e+00	0	209	2.13	218	9	46.33%	553.73
P16304	5x6i.A	212	0.00e+00	0	208	1.15	217	9	46.08%	546.32
P27142	1zin.A	217	0.00e+00	0	209	2.10	218	9	44.50%	553.77
P27142	1zio.A	217	0.00e+00	0	209	2.14	218	9	44.50%	553.22
P27142	1zip.A	217	0.00e+00	0	209	2.11	218	9	44.50%	553.38
P27142	4qbh.A	217	0.00e+00	0	209	1.19	218	9	45.87%	556.88
P27142	4qbi.A	217	0.00e+00	0	209	1.22	218	9	46.33%	557.31
P27144	2ar7.B	221	3.17e-12	2	206	2.19	214	8	35.51%	487.79
P27144	2bbw.B	220	0.00e+00	0	206	2.95	214	8	35.51%	518.20
P27144	3ndp.B	206	1.83e-09	2	181	2.12	214	33	31.31%	388.37
P30085	1tev.A	194	4.49e-14	0	174	3.02	222	48	29.28%	393.16
P43188	1zak.B	220	0.00e+00	0	203	1.86	215	12	42.33%	493.72
P54819	2c9y.A	218	8.85e-14	1	208	3.07	218	10	41.74%	526.06
P69441	1ank.A	214	0.00e+00	0	214	0.45	214	0	100.00%	621.63
P69441	1e4v.A	214	0.00e+00	0	214	0.25	214	0	99.53%	623.32
P69441	1e4y.A	214	0.00e+00	0	214	0.93	214	0	99.07%	613.00
P69441	2eck.A	214	0.00e+00	0	214	0.28	214	0	100.00%	622.90
P69441	3hpq.B	214	0.00e+00	0	214	0.35	214	0	100.00%	623.00
P69441	3hpr.A	214	0.00e+00	0	214	0.31	214	0	99.53%	623.16
P69441	4ake.B	214	1.11e-14	2	210	2.35	217	7	92.63%	592.04
P69441	4x8h.A	214	2.23e-14	2	210	2.37	217	7	92.63%	583.05
P69441	4x8l.B	214	0.00e+00	0	214	0.42	214	0	99.53%	622.75

P69441	4x8m.A	214	7.75e-14	2	214	2.62	214	0	99.53%	584.38
P69441	4x8o.B	214	0.00e+00	0	214	0.40	214	0	99.53%	622.87
P69441	5eje.B	214	0.00e+00	0	214	0.40	214	0	99.07%	623.18
P69441	6f7u.A	214	2.48e-14	2	211	2.37	217	6	93.09%	580.35
P69441	6ham.A	214	0.00e+00	0	214	0.83	214	0	97.20%	611.19
P69441	6rze.A	214	3.73e-14	2	211	2.39	217	6	92.63%	575.03
P69441	6s36.A	214	1.99e-14	2	210	2.34	217	7	92.17%	581.91
P84139	1s3g.A	217	0.00e+00	0	209	1.29	218	9	47.71%	550.78
P84139	5x6j.A	213	0.00e+00	0	209	1.28	218	9	48.17%	551.38
P9WKF5	1p4s.A	181	2.16e-11	0	174	3.00	218	44	36.24%	309.98
P9WKF5	2cdn.A	186	0.00e+00	0	176	1.31	218	42	36.70%	423.25
Q04ML5	4ntz.A	213	1.71e-10	2	201	2.68	222	21	35.14%	463.64
Q04ML5	4nu0.B	212	0.00e+00	0	204	1.45	219	15	36.99%	513.71
Q04ML5	4w5h.A	208	9.73e-09	3	188	2.41	219	31	35.62%	435.52
Q04ML5	4w5j.B	211	0.00e+00	0	203	1.44	219	16	36.99%	507.43
Q3JVB1	3gmt.A	204	1.00e-10	3	197	2.42	204	7	65.69%	516.72
Q5CRC5	3be4.A	215	0.00e+00	0	206	1.20	217	11	46.54%	536.04
Q5NFR4	4pzl.D	218	1.72e-12	2	198	2.69	219	21	53.42%	561.75
Q5SHQ9	3cm0.A	184	7.85e-10	1	173	3.06	218	45	34.40%	387.74
Q68EH2	5xz2.A	192	0.00e+00	0	181	1.82	215	34	31.63%	437.60
Q6B341	3fb4.A	215	0.00e+00	0	209	1.37	218	9	47.71%	559.62
Q6LTE1	4k46.A	214	0.00e+00	0	214	1.00	214	0	72.90%	613.03
Q7Z0H0	3tlx.A	235	0.00e+00	0	209	1.60	218	9	41.28%	544.11
Q9KTB7	4np6.B	215	1.38e-14	2	211	2.36	214	3	73.36%	588.44
Q9UIJ7	1zd8.A	212	1.99e-14	2	206	1.64	214	8	38.79%	489.46
Q9UIJ7	6zjb.A	217	0.00e+00	0	206	1.50	214	8	38.79%	534.04
Q9UIJ7	6zjd.A	214	5.91e-14	2	206	1.74	214	8	38.79%	490.37
Q9UIJ7	6zje.A	214	2.62e-13	2	204	1.92	214	10	38.79%	493.23
Q9Y6K8	2bwj.A	196	6.11e-15	0	171	2.93	218	47	22.94%	416.16
synthetic construct	5g3y.A	213	0.00e+00	0	209	1.19	218	9	53.21%	563.86
synthetic construct	5g3z.A	215	0.00e+00	0	209	1.49	218	9	48.62%	553.79
synthetic construct	5g40.A	215	0.00e+00	0	209	1.09	218	9	48.17%	555.45
synthetic construct	5g41.A	214	0.00e+00	0	209	1.96	218	9	48.17%	555.70
**Icd**										
O29610	2iv0.B	412	0.00e+00	0	404	2.62	416	12	55.29%	1023.68
P08200	1ai3.A	414	0.00e+00	0	414	0.11	414	0	100.00%	1223.58
P08200	1bl5.A	414	0.00e+00	0	414	0.56	414	0	100.00%	1218.52
P08200	1cw1.A	415	0.00e+00	0	414	0.26	414	0	99.76%	1222.85
P08200	1cw4.A	415	0.00e+00	0	414	0.26	414	0	99.76%	1222.95

P08200	1cw7.A	415	0.00e+00	0	414	0.29	414	0	99.76%	1222.46
P08200	1gro.A	414	0.00e+00	0	414	0.48	414	0	99.28%	1220.75
P08200	1grp.A	414	0.00e+00	0	414	0.48	414	0	99.52%	1220.69
P08200	1hj6.A	414	0.00e+00	0	414	0.88	414	0	99.76%	1218.53
P08200	1idc.A	414	0.00e+00	0	414	0.53	414	0	99.76%	1218.00
P08200	1idd.A	414	0.00e+00	0	414	0.54	414	0	99.76%	1218.56
P08200	1ide.A	414	0.00e+00	0	414	0.57	414	0	99.76%	1217.20
P08200	1idf.A	414	0.00e+00	0	414	0.57	414	0	99.76%	1217.65
P08200	1ika.A	414	0.00e+00	0	414	0.91	414	0	100.00%	1190.73
P08200	1iso.A	414	0.00e+00	0	414	0.09	414	0	98.31%	1223.67
P08200	1p8f.A	416	0.00e+00	0	414	0.35	414	0	100.00%	1222.73
P08200	1pb1.A	416	0.00e+00	0	414	0.36	414	0	100.00%	1222.77
P08200	1pb3.A	416	0.00e+00	0	414	0.32	414	0	100.00%	1222.80
P08200	1sjs.A	415	0.00e+00	0	414	2.38	414	0	100.00%	1218.28
P08200	3icd.A	414	0.00e+00	0	414	0.46	414	0	100.00%	1220.74
P08200	3lcb.D	415	0.00e+00	0	414	2.51	415	1	98.31%	1214.46
P08200	4p69.D	415	0.00e+00	0	414	2.57	415	1	98.31%	1207.46
P08200	4aj3.A	416	0.00e+00	0	414	1.19	414	0	100.00%	1217.74
P08200	4aja.A	415	0.00e+00	0	414	0.40	414	0	100.00%	1222.16
P08200	4ajb.A	415	0.00e+00	0	414	0.42	414	0	99.76%	1221.86
P08200	4ajc.A	415	0.00e+00	0	414	0.35	414	0	99.76%	1222.49
P08200	4ajr.A	415	0.00e+00	0	414	1.20	414	0	99.76%	1214.83
P08200	4ajs.A	415	0.00e+00	0	414	0.38	414	0	99.76%	1222.38
P08200	4bnp.A	415	0.00e+00	0	414	0.31	414	0	99.76%	1222.57
P08200	4icd.A	414	0.00e+00	0	414	0.47	414	0	99.76%	1220.74
P08200	5icd.A	414	0.00e+00	0	414	0.50	414	0	100.00%	1220.19
P08200	6icd.A	414	0.00e+00	0	414	0.47	414	0	99.76%	1220.22
P08200	7icd.A	414	0.00e+00	0	414	0.46	414	0	99.76%	1220.53
P08200	8icd.A	414	0.00e+00	0	414	0.47	414	0	99.76%	1220.35
P08200	9icd.A	414	0.00e+00	0	414	0.45	414	0	100.00%	1220.91
P28834	3blv.G	332	0.00e+00	0	324	1.87	396	72	23.99%	791.57
P28834	3blw.A	330	0.00e+00	0	323	1.83	395	72	24.05%	792.60
P28834	3blx.O	332	0.00e+00	0	328	2.88	394	66	23.86%	697.45
P33197	2d1c.B	495	0.00e+00	0	350	2.27	409	59	33.25%	841.44
P39126	1hqs.B	423	0.00e+00	0	405	1.79	426	21	65.73%	1096.34
P50213	5gre.A	325	0.00e+00	0	320	2.00	393	73	29.26%	792.53
P50213	5grf.A	324	0.00e+00	0	319	2.14	393	74	28.24%	751.78
P50213	5grh.A	327	0.00e+00	0	324	2.27	393	69	28.24%	765.27
P50213	5gri.A	323	0.00e+00	0	318	2.08	393	75	29.26%	774.96
P50213	5grl.A	323	0.00e+00	0	318	2.09	393	75	29.26%	777.30
P50213	5yvt.A	332	0.00e+00	0	322	1.97	393	71	28.75%	758.10

P50213	6kde.C	336	0.00e+00	0	326	1.85	394	68	30.20%	808.54
P50213	6kdf.I	333	0.00e+00	0	329	2.12	395	66	29.37%	806.10
P50213	6kdy.E	327	0.00e+00	0	323	1.99	393	70	29.52%	765.41
P50213	6ke3.E	318	0.00e+00	0	314	1.89	391	77	29.16%	737.90
P50213	6l57.A	327	0.00e+00	0	321	2.14	393	72	29.26%	778.22
P50213	6l59.A	325	0.00e+00	0	319	2.02	393	74	29.26%	784.22
Q02NB5	5m2e.D	418	0.00e+00	0	414	2.08	415	1	78.80%	1205.29
Q3JV82	3dms.A	413	0.00e+00	0	410	0.75	415	5	73.98%	1215.35
Q5JFV8	5hn3.A	332	0.00e+00	0	318	2.64	392	74	26.79%	682.92
Q5JFV8	5hn4.A	329	0.00e+00	0	318	1.93	392	74	26.53%	729.78
Q5JFV8	5hn5.A	327	0.00e+00	0	318	1.88	392	74	26.53%	726.39
Q5JFV8	5hn6.A	329	0.00e+00	0	318	1.87	392	74	26.53%	725.27
Q5SIJ1	3asj.C	333	0.00e+00	0	328	1.61	393	65	28.50%	808.86
Q5ZXB6	6c0e.B	414	0.00e+00	0	411	1.35	414	3	75.36%	1177.31
Q72IW9	1x0l.A	333	0.00e+00	0	325	2.04	393	68	28.50%	769.86
Q72IW9	3ah3.C	333	0.00e+00	0	328	2.37	393	65	28.75%	731.81
Q72IW9	4yb4.A	333	0.00e+00	0	328	1.65	393	65	28.50%	811.66
Q8GAX0	2d4v.C	428	0.00e+00	0	412	2.07	429	17	59.67%	1132.62
Q96YK6	2dht.A	403	0.00e+00	0	398	2.79	416	18	47.60%	1054.82
Q96YK6	2e0c.A	401	0.00e+00	0	396	2.71	416	20	46.88%	1049.07
Q96YK6	2e5m.A	403	0.00e+00	0	398	2.79	416	18	47.60%	1062.93
Q9YE81	1tyo.A	427	0.00e+00	0	407	2.65	417	10	47.00%	1068.26
Q9YE81	1v94.B	423	0.00e+00	0	407	2.58	417	10	47.00%	1063.38
Q9YE81	1xgv.A	430	0.00e+00	0	405	2.62	416	11	47.36%	1056.61
Q9YE81	1xkd.B	419	0.00e+00	0	402	2.11	416	14	47.12%	1051.55
**KatE**										
A0A031LXI5	6pt7.A	500	0.00e+00	0	486	1.81	502	16	39.24%	1310.53
A0A0U4WRC5	4b7f.C	514	0.00e+00	0	491	1.70	520	29	33.65%	1269.34
A0A0U4WRC5	4b7g.C	513	0.00e+00	0	500	2.86	521	21	33.21%	1268.92
A0A0U4WRC5	4b7h.C	514	0.00e+00	0	501	2.86	522	21	33.14%	1269.26
A0A6I8WFM0	6lfk.D	720	0.00e+00	0	706	0.79	730	24	62.88%	2013.58
A2A136	2j2m.D	480	0.00e+00	0	480	1.28	492	12	42.07%	1321.53
C1PHG1	6rjn.C	502	0.00e+00	0	483	2.83	511	28	35.62%	1182.69
D9N167	2iuf.E	688	0.00e+00	0	665	1.57	704	39	41.62%	1750.26
D9N167	2xf2.A	688	0.00e+00	0	665	1.55	704	39	41.90%	1754.11
M4GGR5	4aue.A	669	0.00e+00	0	655	1.45	686	31	42.13%	1702.19
M4GGR5	4b7a.D	670	0.00e+00	0	660	1.52	691	31	42.11%	1716.36
M4GGR5	5yem.B	678	0.00e+00	0	663	1.54	695	32	41.73%	1710.51
M4GGR6	4aul.B	674	0.00e+00	0	662	1.54	691	29	41.82%	1720.25
M4GGR7	4aum.D	671	0.00e+00	0	661	1.53	691	30	41.97%	1712.65
M4GGR7	5xvz.B	675	0.00e+00	0	663	1.55	692	29	42.05%	1724.88

M4GGR7	5xy4.B	678	0.00e+00	0	663	1.55	695	32	42.01%	1723.26
M4GGR7	5xzm.B	678	0.00e+00	0	661	1.45	694	33	41.93%	1720.34
M4GGR7	5xzn.B	678	0.00e+00	0	663	1.55	695	32	42.01%	1722.10
M4GGR7	5y17.D	675	0.00e+00	0	663	1.55	692	29	41.76%	1720.29
M4GGR7	5zz1.B	678	0.00e+00	0	663	1.53	695	32	42.01%	1724.05
M4GGR8	4aun.A	671	0.00e+00	0	660	1.53	690	30	42.32%	1706.80
none	4qol.B	480	0.00e+00	0	479	1.23	494	15	43.32%	1307.51
none	4qom.D	480	0.00e+00	0	479	1.21	494	15	43.32%	1307.58
none	4qon.D	480	0.00e+00	0	479	1.22	494	15	43.32%	1308.72
none	4qoo.D	480	0.00e+00	0	479	1.20	494	15	43.32%	1308.74
none	4qop.D	480	0.00e+00	0	479	1.22	494	15	43.32%	1308.77
none	4qoq.D	481	0.00e+00	0	480	1.22	495	15	43.23%	1308.83
none	4qor.B	480	0.00e+00	0	479	1.24	494	15	43.32%	1308.49
O52762	4e37.D	480	0.00e+00	0	478	1.33	494	16	40.08%	1294.35
P00432	1tgu.B	499	0.00e+00	0	495	2.35	527	32	37.19%	1284.15
P00432	1th2.A	499	0.00e+00	0	495	2.35	527	32	37.19%	1282.91
P00432	1th3.B	499	0.00e+00	0	495	2.36	527	32	37.19%	1279.89
P00432	1th4.A	499	0.00e+00	0	495	2.36	527	32	37.19%	1280.54
P00432	3j7b.C	499	0.00e+00	0	495	2.38	527	32	37.19%	1286.74
P00432	3nwl.C	499	0.00e+00	0	496	2.53	528	32	37.12%	1288.09
P00432	3re8.C	499	0.00e+00	0	495	2.37	527	32	37.19%	1283.88
P00432	3rgp.D	499	0.00e+00	0	496	2.45	528	32	37.12%	1287.11
P00432	3rgs.C	499	0.00e+00	0	496	2.49	528	32	37.12%	1283.46
P00432	4blc.A	499	0.00e+00	0	495	2.36	527	32	37.19%	1282.26
P00432	5gkn.C	499	0.00e+00	0	496	2.53	528	32	37.12%	1290.74
P00432	6jnt.A	499	0.00e+00	0	496	2.52	528	32	37.12%	1288.74
P00432	6jnu.C	499	0.00e+00	0	496	2.52	528	32	37.12%	1288.70
P00432	6pm7.B	498	0.00e+00	0	495	2.37	527	32	37.19%	1282.49
P00432	6po0.A	499	0.00e+00	0	496	2.51	528	32	37.12%	1282.83
P00432	7cat.A	498	0.00e+00	0	495	2.37	527	32	37.19%	1288.03
P00432	7di8.D	499	0.00e+00	0	496	2.52	528	32	37.12%	1285.17
P00432	8cat.A	498	0.00e+00	0	495	2.37	527	32	37.19%	1289.06
P04040	1dgb.D	497	0.00e+00	0	494	2.36	526	32	36.12%	1292.53
P04040	1dgf.C	497	0.00e+00	0	494	2.34	526	32	36.12%	1284.91
P04040	1dgg.A	497	0.00e+00	0	494	2.35	526	32	36.12%	1283.94
P04040	1dgh.B	498	0.00e+00	0	494	2.28	527	33	35.86%	1286.71
P04040	1f4j.B	479	0.00e+00	0	472	1.22	490	18	38.57%	1288.71
P04040	1qqw.D	499	0.00e+00	0	496	2.52	528	32	35.98%	1284.37
P15202	1a4e.C	488	0.00e+00	0	480	1.68	499	19	36.47%	1202.61
P21179	1gg9.B	727	0.00e+00	0	727	0.29	727	0	99.72%	2158.43
P21179	1gge.B	727	0.00e+00	0	727	0.29	727	0	99.86%	2158.07

P21179	1ggf.D	727	0.00e+00	0	727	0.32	727	0	99.72%	2156.11
P21179	1ggh.B	727	0.00e+00	0	727	0.29	727	0	99.72%	2158.07
P21179	1ggj.B	727	0.00e+00	0	727	0.29	727	0	99.72%	2157.88
P21179	1ggk.A	727	0.00e+00	0	727	0.43	727	0	99.72%	2157.17
P21179	1iph.A	727	0.00e+00	0	727	0.48	727	0	99.86%	2155.37
P21179	1p7y.A	727	0.00e+00	0	727	0.31	727	0	99.72%	2157.89
P21179	1p7z.A	727	0.00e+00	0	727	0.29	727	0	99.72%	2158.39
P21179	1p80.A	727	0.00e+00	0	727	0.27	727	0	99.72%	2158.63
P21179	1p81.B	727	0.00e+00	0	727	0.27	727	0	99.72%	2158.05
P21179	1qf7.A	727	0.00e+00	0	727	0.19	727	0	99.72%	2158.95
P21179	1qws.A	727	0.00e+00	0	727	0.26	727	0	99.72%	2158.32
P21179	1ye9.C	223	0.00e+00	0	222	0.32	222	0	99.55%	646.85
P21179	3p9p.A	726	0.00e+00	0	726	0.25	726	0	99.72%	2158.26
P21179	3p9q.B	726	0.00e+00	0	726	0.23	726	0	99.45%	2157.93
P21179	3p9r.B	726	0.00e+00	0	726	0.22	726	0	99.59%	2157.98
P21179	3p9s.B	726	0.00e+00	0	726	0.22	726	0	99.59%	2157.87
P21179	3pq2.B	726	0.00e+00	0	726	0.23	726	0	99.45%	2157.77
P21179	3pq3.B	726	0.00e+00	0	726	0.23	726	0	99.45%	2157.85
P21179	3pq4.B	726	0.00e+00	0	726	0.23	726	0	99.45%	2157.78
P21179	3pq5.B	726	0.00e+00	0	726	0.23	726	0	99.45%	2157.83
P21179	3pq6.B	726	0.00e+00	0	726	0.23	726	0	99.45%	2157.82
P21179	3pq7.B	726	0.00e+00	0	726	0.23	726	0	99.45%	2157.86
P21179	3pq8.B	726	0.00e+00	0	726	0.23	726	0	99.45%	2157.85
P21179	3ttt.B	726	0.00e+00	0	726	0.22	726	0	99.72%	2158.30
P21179	3ttu.B	726	0.00e+00	0	726	0.20	726	0	99.59%	2158.32
P21179	3ttv.B	726	0.00e+00	0	726	0.21	726	0	99.45%	2158.32
P21179	3ttw.B	726	0.00e+00	0	726	0.22	726	0	99.59%	2158.15
P21179	3ttx.B	726	0.00e+00	0	726	0.22	726	0	99.59%	2158.12
P21179	3vu3.A	727	0.00e+00	0	727	0.37	727	0	99.86%	2155.59
P21179	4bfl.D	735	0.00e+00	0	727	0.27	727	0	99.86%	2158.06
P21179	4enp.A	726	0.00e+00	0	726	0.22	726	0	99.72%	2158.43
P21179	4enq.A	725	0.00e+00	0	725	0.21	725	0	99.59%	2158.25
P21179	4enr.A	726	0.00e+00	0	726	0.21	726	0	99.59%	2158.33
P21179	4ens.A	726	0.00e+00	0	726	0.22	726	0	99.72%	2158.29
P21179	4ent.B	726	0.00e+00	0	726	0.22	726	0	99.59%	2158.02
P21179	4enu.A	726	0.00e+00	0	726	0.22	726	0	99.59%	2158.21
P21179	4env.B	726	0.00e+00	0	726	0.23	726	0	99.72%	2158.11
P21179	4enw.B	726	0.00e+00	0	726	0.23	726	0	99.72%	2158.06
P21179	5bv2.P	747	0.00e+00	0	727	0.30	727	0	99.04%	2156.84
P21179	6by0.A	726	0.00e+00	0	726	0.24	726	0	99.86%	2158.48
P21179	6ztv.C	726	0.00e+00	0	726	0.27	726	0	99.59%	2157.26

P21179	6ztw.E	726	0.00e+00	0	726	0.27	726	0	99.59%	2157.56
P21179	6ztx.B	727	0.00e+00	0	727	0.29	727	0	99.31%	2157.67
P29422	1gwe.A	498	0.00e+00	0	475	1.90	492	17	34.76%	1268.86
P29422	1gwf.A	498	0.00e+00	0	475	1.89	492	17	34.55%	1268.93
P29422	1gwh.A	498	0.00e+00	0	475	1.88	492	17	34.76%	1269.78
P29422	1hbz.A	498	0.00e+00	0	475	1.89	492	17	34.76%	1269.98
P30263	2xq1.M	491	0.00e+00	0	471	1.45	503	32	38.37%	1186.39
P42321	1e93.A	476	0.00e+00	0	473	1.48	488	15	40.57%	1282.23
P42321	1h6n.A	476	0.00e+00	0	473	1.47	488	15	40.57%	1283.28
P42321	1h7k.A	475	0.00e+00	0	473	1.49	488	15	40.78%	1281.46
P42321	1m85.A	476	0.00e+00	0	473	1.50	488	15	40.78%	1284.23
P42321	1mqf.A	476	0.00e+00	0	473	1.51	488	15	40.78%	1280.75
P42321	1nm0.A	476	0.00e+00	0	473	1.50	488	15	40.78%	1285.17
P42321	2cag.A	475	0.00e+00	0	473	1.48	488	15	40.78%	1286.73
P42321	2cah.A	475	0.00e+00	0	473	1.44	488	15	40.78%	1291.29
P42321	3hb6.A	476	0.00e+00	0	473	1.47	488	15	40.57%	1279.98
P46206	1m7s.B	483	0.00e+00	0	483	1.73	501	18	37.13%	1303.67
P77872	1qwl.B	491	0.00e+00	0	478	1.48	497	19	41.65%	1297.72
P77872	1qwm.B	490	0.00e+00	0	478	1.47	497	19	41.65%	1296.99
P77872	2a9e.B	491	0.00e+00	0	478	1.47	497	19	41.65%	1298.08
P77872	2iqf.B	491	0.00e+00	0	478	1.47	497	19	41.65%	1297.50
Q3LSM1	2isa.H	482	0.00e+00	0	481	1.39	496	15	40.52%	1311.69
Q59337	4cab.A	507	0.00e+00	0	474	1.42	498	24	39.96%	1256.07
Q6CR58	6rjr.A	505	0.00e+00	0	497	2.71	520	23	34.62%	1202.66
Q834P5	1si8.C	474	0.00e+00	0	473	1.26	487	14	42.71%	1328.05
Q9C168	1sy7.B	698	0.00e+00	0	665	1.34	709	44	45.56%	1756.65
Q9C169	3ej6.A	681	0.00e+00	0	667	1.58	698	31	41.69%	1714.44
Q9C169	3zj4.D	680	0.00e+00	0	666	1.52	699	33	41.63%	1725.52
Q9C169	3zj5.D	678	0.00e+00	0	665	1.51	698	33	41.69%	1724.32
Q9C169	4aj9.B	679	0.00e+00	0	665	1.50	698	33	41.69%	1728.34
Q9C169	4bim.A	681	0.00e+00	0	667	1.58	700	33	41.57%	1720.24
Q9C169	6nsw.C	679	0.00e+00	0	665	1.50	698	33	41.69%	1725.63
Q9C169	6nsy.C	679	0.00e+00	0	665	1.51	698	33	41.69%	1724.22
Q9C169	6nsz.C	679	0.00e+00	0	665	1.51	698	33	41.69%	1726.41
Q9C169	6nt0.C	679	0.00e+00	0	665	1.50	698	33	41.69%	1727.25
Q9C169	6nt1.C	679	0.00e+00	0	665	1.52	698	33	41.69%	1725.77
R4GRT6	4b2y.B	674	0.00e+00	0	660	1.44	690	30	42.17%	1717.38
R4GRT7	4b31.B	673	0.00e+00	0	659	1.41	690	31	42.03%	1718.04
R4GRT8	4b40.D	672	0.00e+00	0	661	1.52	691	30	41.97%	1715.85
R4GRT9	4b5k.D	671	0.00e+00	0	660	1.53	691	31	42.11%	1712.07
W1F4G9	6jqq.C	725	0.00e+00	0	725	0.27	726	1	99.04%	2155.26

**Fmt**										
A0A0H2UKZ6	4s1n.A	180	1.67e-15	0	172	1.89	193	21	20.21%	401.72
A3DHJ7	1zgh.A	227	2.33e-15	0	221	2.96	294	73	12.93%	474.10
E3NZ06	4qpd.B	310	0.00e+00	0	288	1.96	314	26	26.75%	745.63
E3NZ06	4r8v.A	308	0.00e+00	0	292	2.78	314	22	26.43%	728.10
E3NZ06	4ts4.A	308	0.00e+00	0	288	2.11	314	26	26.43%	735.90
E3NZ06	4tt8.A	309	0.00e+00	0	288	2.29	314	26	26.43%	742.59
E3NZ06	4qpc.A	308	0.00e+00	0	288	2.07	314	26	26.43%	738.01
E3NZ06	4tts.A	309	0.00e+00	0	288	2.12	314	26	26.43%	737.12
O75891	2bw0.A	309	0.00e+00	0	294	3.06	315	21	27.94%	731.19
O75891	2cfi.A	310	0.00e+00	0	295	2.95	316	21	27.85%	736.13
O85732	5uai.C	311	0.00e+00	0	311	1.44	313	2	60.06%	863.83
P23882	1fmt.A	308	0.00e+00	0	308	1.17	314	6	98.09%	899.44
P28037	1s3i.A	307	0.00e+00	0	289	2.32	313	24	29.71%	738.59
P9WKZ3	4pzu.D	235	4.22e-15	0	221	2.46	255	34	17.25%	429.60
P9WKZ3	4q12.B	232	5.11e-15	0	221	2.53	255	34	18.04%	432.56
P9WKZ3	5vyq.B	234	9.77e-15	0	223	2.60	257	34	17.90%	429.61
Q70LM7	5es5.B	685	2.17e-03	4	247	2.92	318	71	18.24%	400.17
Q70LM7	5es6.A	583	8.84e-04	4	247	2.97	313	66	18.53%	404.72
Q70LM7	5es7.A	582	9.50e-04	4	247	2.98	313	66	18.53%	401.97
Q70LM7	5es8.B	768	5.60e-04	2	226	2.78	303	77	17.82%	394.59
Q70LM7	5es9.A	759	3.32e-03	4	244	2.86	313	69	18.53%	403.80
Q70LM7	5jnf.A	585	8.02e-04	4	244	2.86	313	69	18.53%	404.47
Q70LM7	6mfw.A	1183	4.70e-02	4	236	3.23	315	79	17.78%	396.90
Q70LM7	6mfx.A	1187	1.23e-02	3	236	2.61	313	77	18.21%	400.70
Q70LM7	6mfy.A	1598	1.30e-01	4	242	3.12	313	71	18.53%	390.45
Q70LM7	6mfz.B	585	1.11e-03	4	247	2.94	318	71	18.24%	393.32
Q70LM7	6mg0.A	1693	1.19e-01	4	235	2.70	315	80	18.10%	394.49
Q70LM7	6ulz.A	585	9.67e-04	4	247	2.99	313	66	18.53%	402.63
Q81WH2	4iqf.B	314	0.00e+00	0	312	1.94	315	3	40.63%	853.10
Q83AA8	3tqq.A	304	0.00e+00	0	303	1.98	314	11	49.36%	864.52
Q83AY9	3tqr.A	211	4.45e-14	0	182	2.30	219	37	18.72%	412.34
Q88LI9	3nrb.A	282	9.52e-10	0	185	3.07	216	31	16.20%	367.32
Q8ZJ80	3r8x.A	310	0.00e+00	0	307	1.64	313	6	74.12%	893.82
Q9KF54	3p9x.B	194	4.44e-14	0	176	2.22	201	25	22.39%	401.51
Q9KVU4	3q0i.A	303	0.00e+00	0	303	1.58	312	9	61.22%	876.50
