# Supplementary material for: Investigation of the Importance of Protein 3D Structure for Assessing Conservation of Lysine Acetylation Sites in Protein Homologs
Source: Front Microbiol. 2022 Jan 31;12:805181. doi: 10.3389/fmicb.2021.805181 (PMC8843374; doi:10.3389/fmicb.2021.805181)
Supplement: Supplementary file 11 [file Data_Sheet_11.PDF]

Supplemental Table ST5. FATCAT alignment parameters from aligning homologs to the Adk, Icd, KatE, and Fmt target structures (PDB IDs: 1ake, 1ai2, 1cf9, and 2fmt respectively). AA means amino acid. Data are sorted by UniProt ID (alphabetical order) and are from single representative structures from each UniProt ID of Adk, Icd, KatE, and Fmt homologs with structures deposited into the PDB.

Uniprot	PDB	Structure Length	P-value	twists	opt-len	opt-rmsd	align-len	gap	seq-identity	score
**Adk**										
A0A0J9X1X4	3x2s.A	214	0.00e+00	0	214	0.61	214	0	98.60%	620.62
A0A2R2JFU5	5x6k.A	185	0.00e+00	0	181	1.72	215	34	31.16%	440.76
A0A452CSM2	5ycd.A	191	0.00e+00	0	181	1.72	215	34	31.63%	440.29
A7ZIN4	6hap.A	214	0.00e+00	0	214	1.34	214	0	98.13%	612.53
C7U112	3l0s.C	223	8.93e-11	2	209	2.93	224	15	31.25%	497.51
G4V9S0	3umf.A	188	4.77e-13	0	167	3.37	219	52	26.03%	389.13
M4AA20	5ycf.B	188	0.00e+00	0	177	1.54	215	38	32.09%	420.97
None	4jzk.A	214	0.00e+00	0	214	0.33	214	0	100.00%	622.95
O66490	4jl5.A	203	0.00e+00	0	199	1.46	218	19	43.12%	516.49
P00568	2c95.A	195	0.00e+00	0	181	1.78	215	34	30.23%	434.96
P00571	3adk.A	194	1.27e-14	0	179	3.06	215	36	30.70%	405.42
P07170	1aky.A	218	0.00e+00	0	209	1.41	219	10	44.75%	552.61
P08166	1ak2.A	220	4.25e-14	1	209	2.93	218	9	41.28%	526.59
P08760	2ak3.A	226	2.23e-14	2	202	1.54	214	12	39.25%	489.70
P16304	4qbg.B	217	0.00e+00	0	209	2.69	218	9	45.41%	547.72
P27142	1zin.A	217	0.00e+00	0	209	2.10	218	9	44.50%	553.77
P27144	2bbw.B	220	0.00e+00	0	206	2.95	214	8	35.51%	518.20
P30085	1tev.A	194	4.49e-14	0	174	3.02	222	48	29.28%	393.16
P43188	1zak.B	220	0.00e+00	0	203	1.86	215	12	42.33%	493.72
P54819	2c9y.A	218	8.85e-14	1	208	3.07	218	10	41.74%	526.06
P84139	1s3g.A	217	0.00e+00	0	209	1.29	218	9	47.71%	550.78
P9WKF5	2cdn.A	186	0.00e+00	0	176	1.31	218	42	36.70%	423.25
Q04ML5	4nu0.B	212	0.00e+00	0	204	1.45	219	15	36.99%	513.71
Q3JVB1	3gmt.A	204	1.00e-10	3	197	2.42	204	7	65.69%	516.72
Q5CRC5	3be4.A	215	0.00e+00	0	206	1.20	217	11	46.54%	536.04
Q5NFR4	4pzl.D	218	1.72e-12	2	198	2.69	219	21	53.42%	561.75
Q5SHQ9	3cm0.A	184	7.85e-10	1	173	3.06	218	45	34.40%	387.74
Q68EH2	5xz2.A	192	0.00e+00	0	181	1.82	215	34	31.63%	437.60
Q6B341	3fb4.A	215	0.00e+00	0	209	1.37	218	9	47.71%	559.62
Q6LTE1	4k46.A	214	0.00e+00	0	214	1.00	214	0	72.90%	613.03
Q7Z0H0	3tlx.A	235	0.00e+00	0	209	1.60	218	9	41.28%	544.11
Q9KTB7	4np6.B	215	1.38e-14	2	211	2.36	214	3	73.36%	588.44
Q9UIJ7	6zje.A	214	2.62e-13	2	204	1.92	214	10	38.79%	493.23

Q9Y6K8	2bwj.A	196	6.11e-15	0	171	2.93	218	47	22.94%	416.16
synthetic construct	5g3y.A	213	0.00e+00	0	209	1.19	218	9	53.21%	563.86
synthetic construct	5g3z.A	215	0.00e+00	0	209	1.49	218	9	48.62%	553.79
synthetic construct	5g40.A	215	0.00e+00	0	209	1.09	218	9	48.17%	555.45
**Icd**										
O29610	2iv0.B	412	0.00e+00	0	404	2.62	416	12	55.29%	1023.68
P28834	3blx.O	332	0.00e+00	0	328	2.88	394	66	23.86%	697.45
P33197	2d1c.B	495	0.00e+00	0	350	2.27	409	59	33.25%	841.44
P39126	1hqs.B	423	0.00e+00	0	405	1.79	426	21	65.73%	1096.34
P50213	6l59.A	325	0.00e+00	0	319	2.02	393	74	29.26%	784.22
Q02NB5	5m2e.D	418	0.00e+00	0	414	2.08	415	1	78.80%	1205.29
Q3JV82	3dms.A	413	0.00e+00	0	410	0.75	415	5	73.98%	1215.35
Q5JFV8	5hn3.A	332	0.00e+00	0	318	2.64	392	74	26.79%	682.92
Q5SIJ1	3asj.C	333	0.00e+00	0	328	1.61	393	65	28.50%	808.86
Q5ZXB6	6c0e.B	414	0.00e+00	0	411	1.35	414	3	75.36%	1177.31
Q72IW9	1x0l.A	333	0.00e+00	0	325	2.04	393	68	28.50%	769.86
Q8GAX0	2d4v.C	428	0.00e+00	0	412	2.07	429	17	59.67%	1132.62
Q96YK6	2e0c.A	401	0.00e+00	0	396	2.71	416	20	46.88%	1049.07
Q9YE81	1tyo.A	427	0.00e+00	0	407	2.65	417	10	47.00%	1068.26
**KatE**										
A0A031LXI5	6pt7.A	500	0.00e+00	0	486	1.81	502	16	39.24%	1310.53
A0A0U4WRC5	4b7f.C	514	0.00e+00	0	491	1.70	520	29	33.65%	1269.34
A0A6I8WFM0	6lfk.D	720	0.00e+00	0	706	0.79	730	24	62.88%	2013.58
A2A136	2j2m.D	480	0.00e+00	0	480	1.28	492	12	42.07%	1321.53
C1PHG1	6rjn.C	502	0.00e+00	0	483	2.83	511	28	35.62%	1182.69
D9N167	2iuf.E	688	0.00e+00	0	665	1.57	704	39	41.62%	1750.26
M4GGR5	4b7a.D	670	0.00e+00	0	660	1.52	691	31	42.11%	1716.36
M4GGR6	4aul.B	674	0.00e+00	0	662	1.54	691	29	41.82%	1720.25
M4GGR7	4aum.D	671	0.00e+00	0	661	1.53	691	30	41.97%	1712.65
M4GGR8	4aun.A	671	0.00e+00	0	660	1.53	690	30	42.32%	1706.80
none	4qol.B	480	0.00e+00	0	479	1.23	494	15	43.32%	1307.51
O52762	4e37.D	480	0.00e+00	0	478	1.33	494	16	40.08%	1294.35
P00432	3rgp.D	499	0.00e+00	0	496	2.45	528	32	37.12%	1287.11
P15202	1a4e.C	488	0.00e+00	0	480	1.68	499	19	36.47%	1202.61
P29422	1gwe.A	498	0.00e+00	0	475	1.90	492	17	34.76%	1268.86
P30263	2xq1.M	491	0.00e+00	0	471	1.45	503	32	38.37%	1186.39
P42321	1e93.A	476	0.00e+00	0	473	1.48	488	15	40.57%	1282.23
P46206	1m7s.B	483	0.00e+00	0	483	1.73	501	18	37.13%	1303.67
P77872	1qwl.B	491	0.00e+00	0	478	1.48	497	19	41.65%	1297.72
Q3LSM1	2isa.H	482	0.00e+00	0	481	1.39	496	15	40.52%	1311.69

Q59337	4cab.A	507	0.00e+00	0	474	1.42	498	24	39.96%	1256.07
Q6CR58	6rjr.A	505	0.00e+00	0	497	2.71	520	23	34.62%	1202.66
Q834P5	1si8.C	474	0.00e+00	0	473	1.26	487	14	42.71%	1328.05
Q9C168	1sy7.B	698	0.00e+00	0	665	1.34	709	44	45.56%	1756.65
Q9C169	4aj9.B	679	0.00e+00	0	665	1.50	698	33	41.69%	1728.34
R4GRT6	4b2y.B	674	0.00e+00	0	660	1.44	690	30	42.17%	1717.38
R4GRT7	4b31.B	673	0.00e+00	0	659	1.41	690	31	42.03%	1718.04
R4GRT8	4b40.D	672	0.00e+00	0	661	1.52	691	30	41.97%	1715.85
R4GRT9	4b5k.D	671	0.00e+00	0	660	1.53	691	31	42.11%	1712.07
W1F4G9	6jqq.C	725	0.00e+00	0	725	0.27	726	1	99.04%	2155.26
**Fmt**										
A0A0H2UKZ6	4s1n.A	180	1.67e-15	0	172	1.89	193	21	20.21%	401.72
A3DHJ7	1zgh.A	227	2.33e-15	0	221	2.96	294	73	12.93%	474.10
E3NZ06	4ts4.A	308	0.00e+00	0	288	2.11	314	26	26.43%	735.90
O75891	2bw0.A	309	0.00e+00	0	294	3.06	315	21	27.94%	731.19
O85732	5uai.C	311	0.00e+00	0	311	1.44	313	2	60.06%	863.83
P23882	1fmt.A	308	0.00e+00	0	308	1.17	314	6	98.09%	899.44
P28037	1s3i.A	307	0.00e+00	0	289	2.32	313	24	29.71%	738.59
P9WKZ3	4pzu.D	235	4.22e-15	0	221	2.46	255	34	17.25%	429.60
Q70LM7	6mfx.A	1187	1.23e-02	3	236	2.61	313	77	18.21%	400.70
Q81WH2	4iqf.B	314	0.00e+00	0	312	1.94	315	3	40.63%	853.10
Q83AA8	3tqq.A	304	0.00e+00	0	303	1.98	314	11	49.36%	864.52
Q83AY9	3tqr.A	211	4.45e-14	0	182	2.30	219	37	18.72%	412.34
Q88LI9	3nrb.A	282	9.52e-10	0	185	3.07	216	31	16.20%	367.32
Q8ZJ80	3r8x.A	310	0.00e+00	0	307	1.64	313	6	74.12%	893.82
Q9KF54	3p9x.B	194	4.44e-14	0	176	2.22	201	25	22.39%	401.51
Q9KVU4	3q0i.A	303	0.00e+00	0	303	1.58	312	9	61.22%	876.50
