# Supplementary material for: Investigation of the Importance of Protein 3D Structure for Assessing Conservation of Lysine Acetylation Sites in Protein Homologs
Source: Front Microbiol. 2022 Jan 31;12:805181. doi: 10.3389/fmicb.2021.805181 (PMC8843374; doi:10.3389/fmicb.2021.805181)
Supplement: Supplementary file 12 [file Data_Sheet_12.PDF]

Supplemental Table ST2. Identity of unique organisms for each KAT substrate protein and number of 3D structures of each organism analyzed.

**Substrate protein**		**Organism**	**Number of structures**
**Adk homologs**			
Bacteria	Gram negative	*Aquifex aeolicus*	14
		Burkholderia pseudomallei	1
		Desulfovibrio gigas	3
		Escherichia coli	20
		Francisella tularensis subsp. tularensis	1
		Photobacterium profundum	1
		Thermus thermophilus	1
		Vibrio cholerae	1
	Gram positive	*Bacillus subtilis*	17
		Geobacillus stearothermophilus	5
		Jeotgalibacillus marinus	1
		Sporosarcina globispora	2
		Streptococcus pneumoniae	4
	Gram variable	*Mycobacterium tuberculosis*	2
Eukaryote		*Bos taurus*	3
		Cryptosporidium parvum	1
		Danio rerio	1
		Homo sapiens	12
		Notothenia coriiceps	5
		Plasmodium falciparum	1
		Poecilia reticulata	1
		Saccharomyces cerevisiae	4
		Schistosoma mansoni	1
		Sus scrofa	1
		Xiphophorus maculatu	1
		Zea mays	1
Archaea			0
Synthetic constructs			4
**Icd homologs**			
Bacteria	Gram negative	*Acidithiobacillus thiooxidans*	1
		Burkholderia pseudomallei	1
		Escherichia coli	35
		Legionella pneumophila subsp. pneumophila	1

		Pseudomonas aeruginosa	1
		Thermus thermophilus	5
	Gram positive	*Bacillus subtilis*	1
Eukaryote		*Homo sapiens*	12
		Saccharomyces cerevisiae	3
Archaea		*Aeropyrum pernix*	4
		Archaeoglobus fulgidus	1
		Sulfurisphaera tokodaii	3
		Thermococcus kodakarensis	4

**KatE homologs**			
Bacteria	Gram negative	*Acinetobacter sp*	1
		Aliivibrio salmonicida	1
		Escherichia coli	48
		Helicobacter pylori	4
		Proteus mirabilis	9
		Pseudomonas aeruginosa	1
		Pseudomonas syringae	1
	Gram positive	*Bacillus pumilus*	7
		Corynebacterium glutamicum	3
		Deinococcus radiodurans	1
		Enterococcus faecalis	1
		Exiguobacterium oxidotolerans	1
		Micrococcus luteus	4
Eukaryote		*Bos taurus*	18
		Homo sapiens	6
		Kluyveromyces lactis	1
		Komagataella pastoris	1
		Mycothermus thermophilus	16
		Neurospora crassa	11
		Penicillium janthinellum	2
		Pichia angusta	1
		Saccharomyces cerevisiae	1
Archaea			0

**Fmt homologs**			
Bacteria	Gram negative	*Coxiella burnetii*	2
		Escherichia coli	2
		Pseudomonas aeruginosa	1
		Pseudomonas putida	1
		Vibrio cholerae	1
		Yersinia pestis	1
	Gram positive	*Bacillus anthracis*	1

		Bacillus halodurans	1
		Brevibacillus parabrevis	12
		Hungateiclostridium thermocellum	1
		Streptococcus pneumoniae	1
	Gram variable	*Mycobacterium tuberculosis*	3
Eukaryote		*Danio rerio*	6
		Homo sapiens	2
		Rattus norvegicus	1
Archaea			0
